# Systematic and functional identification of small non-coding RNAs associated with exogenous biofuel stress in cyanobacterium *Synechocystis* sp. PCC 6803

**DOI:** 10.1186/s13068-017-0743-y

**Published:** 2017-03-07

**Authors:** Guangsheng Pei, Tao Sun, Shuo Chen, Lei Chen, Weiwen Zhang

**Affiliations:** 1 Laboratory of Synthetic Microbiology, School of Chemical Engineering & Technology, Tianjin University, Tianjin, 300072 People’s Republic of China; 2 Key Laboratory of Systems Bioengineering, Ministry of Education of China, Tianjin, 300072 People’s Republic of China; 3 Collaborative Innovation Center of Chemical Science and Engineering, Tianjin, People’s Republic of China; 4 Center for Biosafety Research and Strategy, Tianjin University, Tianjin, People’s Republic of China

**Keywords:** sRNA, *Synechocystis*, Biofuels, Tolerance

## Abstract

**Background:**

The unicellular model cyanobacterium *Synechocystis* sp. PCC 6803 is considered a promising microbial chassis for biofuel production. However, its low tolerance to biofuel toxicity limits its potential application. Although recent studies showed that bacterial small RNAs (sRNAs) play important roles in regulating cellular processes in response to various stresses, the role of sRNAs in resisting exogenous biofuels is yet to be determined.

**Results:**

Based on genome-wide sRNA sequencing combined with systematic analysis of previous transcriptomic and proteomic data under the same biofuel or environmental perturbations, we report the identification of 133 *trans*-encoded sRNA transcripts with high-resolution mapping of sRNAs in *Synechocystis*, including 23 novel sRNAs identified for the first time. In addition, according to quantitative expression analysis and sRNA regulatory network prediction, sRNAs potentially involved in biofuel tolerance were identified and functionally confirmed by constructing sRNA overexpression or suppression strains of *Synechocystis*. Notably, overexpression of sRNA Nc117 revealed an improved tolerance to ethanol and butanol, while suppression of Nc117 led to increased sensitivity.

**Conclusions:**

The study provided the first comprehensive responses to exogenous biofuels at the sRNA level in *Synechocystis* and opens an avenue to engineering sRNA regulatory elements for improved biofuel tolerance in the cyanobacterium *Synechocysti*s.

**Electronic supplementary material:**

The online version of this article (doi:10.1186/s13068-017-0743-y) contains supplementary material, which is available to authorized users.

## Background

The production of biofuels using solar energy and CO_2_ in metabolically engineered photosynthetic cyanobacteria holds promise for replacing fossil fuel and generating sustainable energy [1, 2]. However, current biofuel productivity in the cyanobacterial system is still several orders of magnitude lower than that of native producing microbes [3, 4]. This may be due to multiple reasons, such as low expression of foreign metabolic pathways and efficiency to direct metabolic flux to end-products in the cyanobacterial chassis, as well as high end-product toxicity to cyanobacterial hosts [5, 6]. Therefore, in addition to further optimizing expression and functionality of foreign pathways, there is urgency to systematically understand the tolerance mechanism of the cyanobacterial chassis to biofuels, as well as various resistance mechanisms for surviving adverse environmental perturbations during fermentation.

Recent studies showed that small RNAs (sRNAs) between 50 and 300 nucleotides play key regulatory roles in prokaryotic cells at the post-transcriptional level [7]. These RNAs interfere with ribosome binding sites and block translation initiation by base-pairing or affecting mRNA secondary structure and consequently altering mRNA stability, or they interact with proteins directly to modulate their activity [8]. RNA sequencing (RNA-seq) is a powerful analytical tool that provides insight into changes in gene expression and leads to the discovery of novel small and regulatory RNAs. RNA-seq has recently been applied in research of sRNAs in the model cyanobacterium *Synechocystis* sp. PCC 6803 (hereafter *Synechocystis*) [9–11], as well as in various groups of cyanobacteria, such as *Prochlorococcus* and *Anabaena* [12, 13]. Many identified sRNAs were shown to be involved in cellular responses to a variety of environmental stresses and stimuli [9, 12, 13]. For example, both *cis*-acting antisense sRNA (e.g., IsrR, Asl_flv4) and *trans*-encoded sRNAs (e.g., PsrR1, NsiR4) in *Synechocystis* participate in iron depletion, inorganic carbon supply, or photosynthetic and nitrogen assimilation control metabolism [14–17]. However, sRNAs involved in regulating or improving biofuel tolerance have not yet been described in cyanobacteria.

Engineering of cyanobacteria for improved biofuel tolerance would require a level of understanding of the mode of action of sRNAs. As experimental investigation of multiple potential targets is often slow, numerous bioinformatics tools have been developed to predict gene targets of sRNAs at a genomic scale [18, 19]. These tools are based on the phylogenetic conservation of either sRNA or target sequences. After an initial interaction energy-dependent target prediction within the individual whole genome, the CopraRNA algorithm utilizes functional enrichment to achieve a list of potential target genes and performs further regulatory network analysis with the aim of uncovering a function for sRNA [18]. However, sRNA regulatory mechanisms in bacteria are not limited to base-pairing regulation [7, 20]. In particular, some sRNAs interact with proteins that have regulatory roles in a pathway or biological process [8]. Therefore, identification and engineering of master regulatory sRNAs, particularly highly abundant and stable sRNAs that have predictable secondary structures, will lead to a novel strategy for *Synechocystis* to be adapted to a growth condition with higher biofuel concentrations.

With an ultimate goal to construct a robust and product-tolerant photosynthetic chassis for synthesizing various renewable biofuels, we have previously applied various omics analytical tools to determine cellular responses of *Synechocystis* cells under exogenous biofuel stress. The results showed that the cells tended to employ a combination of multiple resistance mechanisms in dealing with various stresses [21–27], which has created challenges to improve tolerance by conventional sequential multi-gene modification approaches [3, 28]. To address the issue, approaches have been proposed to analyze regulatory systems for tolerance improvement [29]. For example, Song et al. [30] and Chen et al. [28] used quantitative iTRAQ LC–MS/MS proteomics to discover the two regulators Sll0794 and Slr1037, which participate in *Synechocystis* biofuel tolerance [28, 30]. Kaczmarzyk et al. [31] overexpressed *sigB* to increase both temperature and butanol tolerance in *Synechocystis*. Therefore, “transcriptional engineering” for tolerance improvement [29, 31], which includes systematic analysis and engineering of master regulatory sRNAs could be an applicable approach [32]. The sRNA engineering approach could have many advantages, such as rapid response, flexible and precise control, ready restoration, and low metabolic burden [32]. When the study was initiated, it was shown that increased sRNA expression in *E. coli* resulted in superior tolerance to acid and provided protection against oxidative stress [33]. In addition, a comprehensive RNA-seq study of all mRNAs and sRNAs under ten different growth or environmental stress conditions was also recently reported for *Synechocystis* [10]. Here, we conducted a deep-sequencing analysis of sRNAs in *Synechocystis* under various exogenous biofuel stresses including ethanol, butanol, and hexane, and proposed a multi-step approach for the identification of sRNAs in *Synechocystis.* Because most current sRNA target prediction algorithms may have overlooked structured sRNAs that function with no short seed base-pairing sequence, the potential secondary structures of sRNAs with top abundance in our list of sRNAs were also selected for analysis. To identify sRNAs specifically related to biofuel tolerance from a large number of candidates, we further applied multivariate statistical analysis and sRNA regulatory network construction approaches via extensive target prediction [18], correlation analysis between sRNA and paired transcriptomic data [34], and functional enrichment analysis [35]. These efforts led to the identification of several sRNAs related to biofuel tolerance, among which a *trans*-encoded sRNA Nc117 was shown to improve cell tolerance against both ethanol and butanol when overexpressed in *Synechocystis*. In contrast, overexpression of three other sRNAs, whose possible targets were enriched in porphyrin and chlorophyll metabolism or photosynthesis, rendered the *Synechocystis* cells more sensitive to ethanol and butanol.

## Results

### sRNA deep-sequencing and identification

**Fig. 1:**
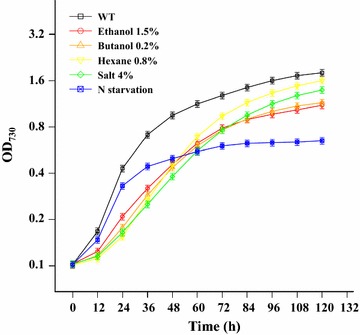
Effects of ethanol, butanol, hexane, salt, and nitrogen starvation on growth of *Synechocystis*. Growth curves of wild-type *Synechocystis* in BG11 medium control (WT), medium with biofuel at the indicated concentration (*v/v*), medium with 4% NaCl (*w/v*), or BG11 medium without nitrogen sources (N starvation). *Error bars* represent the calculated standard deviation of the measurements of three biological replicates

To ensure that the sRNA sequence data obtained in this study were compatible with our previous transcriptomic and proteomic data, *Synechocystis* was grown under the same concentration treatments as in several previous studies [i.e., ethanol 1.5% (*v*/*v*), butanol 0.2% (*v*/*v*), hexane 0.8% (*v*/*v*), salt 4% (*w*/*v*), and nitrogen starvation] [21–27], which led to an approximate 50% growth decrease at 48 h (Fig. 1). To identify the sRNAs of *Synechocystis* that potentially participated in responses to various stresses, sRNA isolation was performed with cultures prepared at 24, 48, and 72 h for further sRNA sequencing. This resulted in a total of 294.6-million raw sequencing reads from 18 samples, with an average of 16.37 million reads per sample. After trimming the 3′ and 5′ adapter sequences and low-quality bases, all samples showed a mapping ratio greater than 50%, except for hexane-treated samples (Additional file 1: Table S1). Based on the read mapping and coverage statistics (Additional file 2: Figure S1), a total of 3378 and 131 small-size RNA genes were identified in the chromosomal DNA and four plasmids of *Synechocystis*, respectively, excluding 43 tRNA genes. Based on their location, these candidates were further classified into three categories: (1) 133 *trans*-encoded sRNAs located in intergenic regions, which are the subjects of this study and referred to as sRNAs below, (2) 1824 *cis*-antisense sRNAs (asRNAs) inversely oriented within an annotated gene and (3) 1421 sRNAs located in mRNA untranslated regions (UTRs) (100 nt upstream or 50 nt downstream of annotated genes) on the chromosome of *Synechocystis* (Additional file 3: Table S2).

**Fig. 2:**
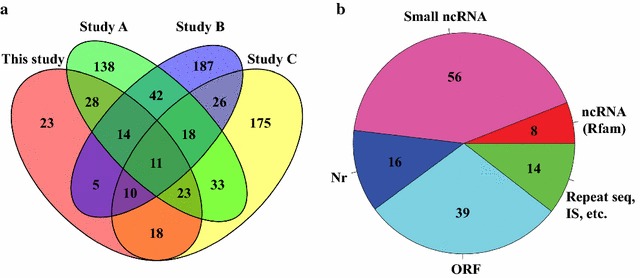
The *trans*-encoded sRNA gene distribution in *Synechocystis*. **a** Venn diagram of *trans*-encoded sRNA inventories identified in this study and previous studies—Study A [9], Study B [10], and Study C [11]—using different technical platforms. **b** Pie chart showing the number of *trans*-encoded sRNAs identified in this study belonging to different categories: Nr, potential sRNAs identified by BLAST search in non-redundant protein sequence database with *E* value <1*e* ^−10^; ORF, potential sRNAs with an open reading frame; Repeat, IS: potential sRNAs located in genome-interspersed repeat region or identified as an insert sequence; ncRNA (Rfam), sRNAs identified in conserved non-coding RNA family in Rfam database

Various strategies have been utilized for systematic genome-wide searching for sRNAs in *Synechocystis* in the past. However, the results obtained by various experimental (i.e., sequencing platform, library construction) and bioinformatics approaches vary widely [9–11]. Therefore, a comparison was conducted for regulatory sRNAs identified in this study with those identified previously in *Synechocystis* [9–11] (Additional file 4: Table S3). The Venn diagram plots showed that only 11 sRNAs were identified by all four independent studies of *Synechocystis* sRNAs, while a majority of *trans*-encoded sRNAs were only identified by one or two approaches (Fig. 2a) [9–11]. In addition, the 11 sRNAs identified in all studies were defined with slightly different boundaries, indicating that sRNAs in *Synechocystis* have not been exhaustively investigated and well characterized. However, a few experimentally validated sRNAs indeed appeared on our list, such as *cis*-antisense sRNAs such as IsrR [14] (As717) and As1_flv4 [15] (As59), and the *trans*-encoded sRNAs PsrR1 [16] (Nc57) and NsiR4 [17] (Nc42). Moreover, the expression level of PsrR1 and NsiR4 was up-regulated nine and threefold under nitrogen starvation conditions, as revealed by the deep-sequencing data and consistent with the previous experimental report [10]. Furthermore, the 4.5S RNA (Nc64), 6S RNA (Nc70) [36], and tmRNA (Nc121) were also identified in this study in high abundance [37] (Additional file 5: Figure S2). Notably, the comparative analysis showed that 23 new *trans*-encoded sRNAs were identified by this study (Fig. 2a), some of which were found with relatively high read coverage depth, such as Nc7, Nc123, Nc66, Nc56, and Nc33. It is likely that expression of some sRNAs was only activated under specific stress-treated samples, consistent with previous reports that any single experimental condition could not accommodate identification of all sRNAs in a species [9–11].

Previous transcriptomic sequencing showed that reads that can map to multiple locations due to close paralogs can lead to inaccurate estimation of expression level in genes that reside in repetitive regions [38]. Although we did not discard reads that could map to multiple locations, it was necessary to classify sRNAs located in repetitive regions as potential false positives [39]. For example, nine *trans*-encoded sRNAs were identified from the pSYSA plasmid of *Synechocystis*, among which six were located in three CRISPR sequence regions of the plasmid [8, 40]. Although no CRISPR sequences were found in the *Synechocystis* genome, a number of interspersed repeat sequences (IRSs) were widely distributed in the *Synechocystis* chromosome. One type of IRS, the retrotransposon, is a major class of transposable elements that can duplicate through RNA intermediates and would bring interference to sRNA-library construction [41]. In addition, some *Synechocystis* sRNAs may contain uORFs that are in fact short mRNAs or dual-function RNAs [42]. Therefore, all identified sRNA sequences were subjected to BLAST searches against a non-redundant protein sequence database (Nr) and potential open reading frame (ORF) and ribosomal binding site (RBS) prediction, respectively, for further confirmation (Fig. 2b). The results showed that 14 *trans*-encoded sRNAs were located in repetitive regions of the genome, and four of these were identified as insert sequence (IS) elements. Approximately 16 *trans*-encoded sRNAs matched to sequences encoding hypothetical proteins in the Nr database with an *E* value less than 1*e* ^*−*10^, and 39 *trans*-encoded sRNAs potentially encoded ORFs. Interestingly, beyond *trans*-encoded sRNA genes, eight sRNAs identified here had records in Rfam database [43], such as 6S and tmRNA. Finally, 56 of the remaining *trans*-encoded sRNAs identified in this study could potentially be authentic small non-coding RNAs (small ncRNAs) in *Synechocystis*. Notably, 16 *trans*-encoded sRNAs located near *Rho*-independent transcription terminators have been reported for numerous bacterial sRNAs [44]. Detailed classification and annotation results for *trans*-encoded sRNA are provided in Additional file 4: Table S3.

### Stress response analysis for top abundant sRNAs in *Synechocystis*

**Fig. 3:**
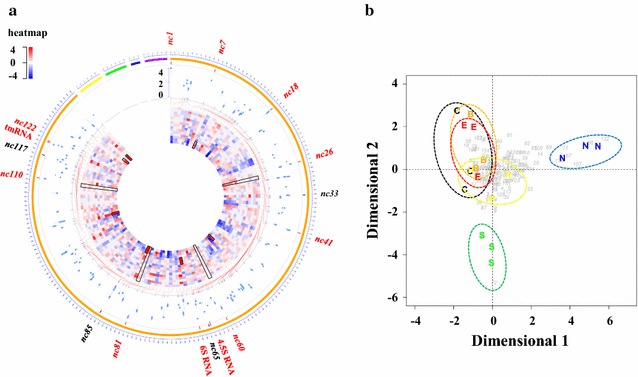
Circular representation and correspondence analysis of expression of *trans*-encoded sRNAs in *Synechocystis* under control and five stress conditions. **a** From outside to inside: (1) Whole chromosome and four plasmids (pSYSM, pSYSA, pSYSG, and pSYSX) of *Synechocystis* with color order *orange*, *yellow*, *green*, *blue*, and *purple*, respectively. *Numbers in blue* labeled outside the circle reflect the scale, and each unit corresponds to 0.01 M of the genome; (2) Location of sRNAs in the *Synechocystis* genome. Several key sRNAs were labeled in the *outer circle*: biofuel-responsive sRNAs are in *black*; names of the top abundant sRNAs are marked in *red*; (3) Circular boxplot presentation for the range of sRNA absolute expression levels under various conditions (recorded as the log_10_-transformed maximum, upper quartile, median, lower quartile, and minimum expression values of sRNA in 18 different samples). Higher sRNA expression is correlated with a further outside boxplot position; (4) Circular heatmap presentation for sRNA log_2_-transformed expression changes under five stress conditions at 24, 48, and 72 h. From outside to inside, the order is E24, E48, E72, B24, B48, B72, H24, H48, H72, S24, S48, S72, N24, N48, and N72. The color scale is in the *top-left corner* of the figure. Several biofuel-responsive sRNAs or stress-specific responsive sRNAs are marked by *black framed lines*; **b** Correspondence analysis of sRNA expression under 18 experimental conditions. Samples in control, ethanol, butanol, hexane, salt, or nitrogen starvation conditions are labeled *black*, *red*, *orange*, *yellow*, *green*, and *blue*, respectively. Each *gray number* represents one of the 133 identified *trans*-encoded sRNAs. The *x-axis* and *y-axis* represent the first dimension and the second dimension, respectively, for correspondence analysis

Great advances have been made in the computational prediction of sRNA targets, and current target prediction algorithms typically start with single short seed base-pairing sequences, which assume a regulatory mechanism involving sRNA–mRNA interaction. However, this sequence-based prediction can be problematic, especially for sRNAs that have complex secondary and tertiary structures that confer potential to interact with other biomolecules such as proteins (e.g., CsrB/RsmZ) [7]. Due to the structures and potential binding to proteins, sRNAs must be relatively stable through the course of cultivation and become a constitutive component of cell physiology when *Synechocystis* is adapted to biofuels. One way to identify these sRNAs is to examine the abundance of sRNA candidates [42]. The abundance of 133 sRNAs listed in Additional file 3: Table S2 was thus determined. Although the sRNAs with top abundance in the small RNA sequencing (sRNA-seq) data for biofuel-treated cells were somewhat different from the highly abundant sRNAs identified in other studies [42], commonly known sRNAs such as 4.5S RNA (Nc64), tmRNA (Nc121) [37], and 6S RNA (Nc70) [36] were highly ranked in the list (1st, 4th, and 9th, respectively) (Fig. 3a, details in legend), suggesting the consistency of sRNA sequencing compared to conventional RNA blotting methods.

Two-step RT-PCR analysis was also performed to estimate the abundance and determine the transcriptional orientation of the 12 most abundant sRNAs selected from the sRNA-seq data list (Additional file 6: Figure S3). The result showed that the expression of almost all sRNAs could be confirmed, and the abundance of several sRNAs, such as Nc18, Nc41, Nc110 and Nc122, was nearly as high as the three positive control sRNAs. The orientation of sRNA except *nc81*, located in the genome repeat region with a 60% high GC content was also determined in relation to the adjacent genes for all sRNAs, and the results were in good agreement with either Rfam annotation or previous reports [9–11]. Many of these sRNAs are located in genetically less characterized regions of the genome. Interestingly, the proximal genes of *nc7* and *nc122* (*ncr1650*) seem to be related to photosynthesis. Furthermore, analysis using the RNAfold program [45] showed that these highly abundant sRNAs could fold into complex secondary structures, implying their stable nature and possibly important physiological roles (Additional file 7: Figure S4). These sRNAs were found to be *Synechocystis*-specific and most of the sRNA abundance appeared to not be affected by the biofuels (except Nc81), as revealed by the sRNA-seq data. However, more data are still needed to define possible functions of the abundant *Synechocystis* sRNAs with stable structure.

### Quantification analysis for stress responsive *trans*-encoded sRNAs

To investigate sRNAs potentially involved in stress responses, a systematic presentation of the related information for the identified *trans*-encoded sRNAs is shown in Fig. 3a, including location, absolute expression abundance, and expression response change to different stresses in the sRNAs. In addition, a multivariate statistics approach called correspondence analysis (CA) focuses on the relationships between samples and sRNA expression. This method was applied to determine possible biofuel-specific sRNAs in *Synechocystis*. The score plot of CA in Fig. 3b shows that: (i) samples under the same treatment condition were clearly clustered together on the CA plot, suggesting that the stress condition is a significant factor determining the expression of sRNAs; (ii) significantly different responses between the wild type (WT) and samples treated with environmental perturbations (i.e., salt and nitrogen starvation) were observed, while the samples stressed by several biofuels tended to be similar to the WT, suggesting a relatively high degree of similarity between all biofuel-stressed samples. The responses resulting from biofuel stress are less significant than the high salt and nitrogen starvation at the sRNA level; (iii) notably, the sRNA Nc72 was found to be specifically expressed under high salt conditions, and was nearest to the sample treated with high salt on the CA score plot. Similarly, several sRNAs including Nc11, Nc57 (PsrR1), Nc86, Nc107, Nc130 and Nc132 were identified as possible sRNAs whose expression specifically responded to nitrogen starvation conditions. In contrast, there was no significant difference in CA score plot between the WT and biofuel-treated samples, suggesting that no sRNA was uniquely regulated by any specific type of the exogenous biofuel.

**Fig. 4:**
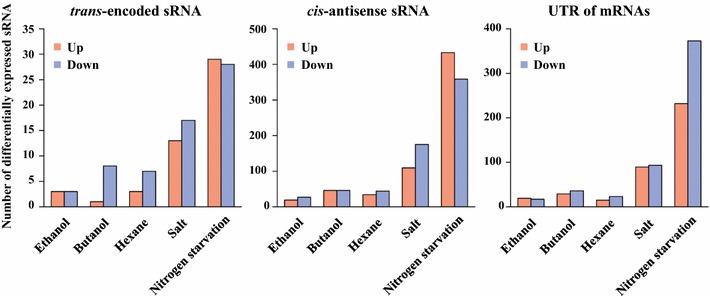
Number of differentially regulated sRNAs in the genome of *Synechocystis* under five conditions

To further investigate biofuel-responsive sRNA, differential expression profiling analysis between the WT and stress-treated conditions was performed using DESeq software [46] (Additional file 3: Table S2). Using criteria of fold change greater than 1.5 and adjusted *p* values less than 0.05, only six, nine, and ten sRNAs were found differentially expressed under the ethanol, butanol, or hexane stress conditions, respectively (Fig. 4). This number is far less than the high salt (30) and nitrogen starvation conditions (57), consistent with overall trends revealed by the CA analysis. To capture the early responses at the transcriptional level, we also performed a differential expression profiling analysis for sRNAs at early stages (i.e., 24 and 48 h) (details in Additional file 3: Table S2). Similar results were obtained to those from the CA analysis.

### Identification of biofuel-related sRNAs by construction of an sRNA regulatory network and experimental confirmation

Recently, a network approach combined with global omics datasets has been proposed as a useful tool to identify responses under multiple growth conditions [47]. A functional sRNA regulatory network in *Synechocystis* was constructed with the aid of the CopraRNA tool [18] integrated with paired transcriptomic analysis (Additional file 8: Figure S5). The results showed that the potential targets of Nc57 (PsrR1) were enriched in photosynthesis targets, consistent with the results of recently published verification experiments [16]. This example demonstrated that the network approach implemented in this study could provide reliable identification of sRNAs involved in regulating key biological processes that might be associated with biofuel tolerance of *Synechocystis*.

**Fig. 5:**
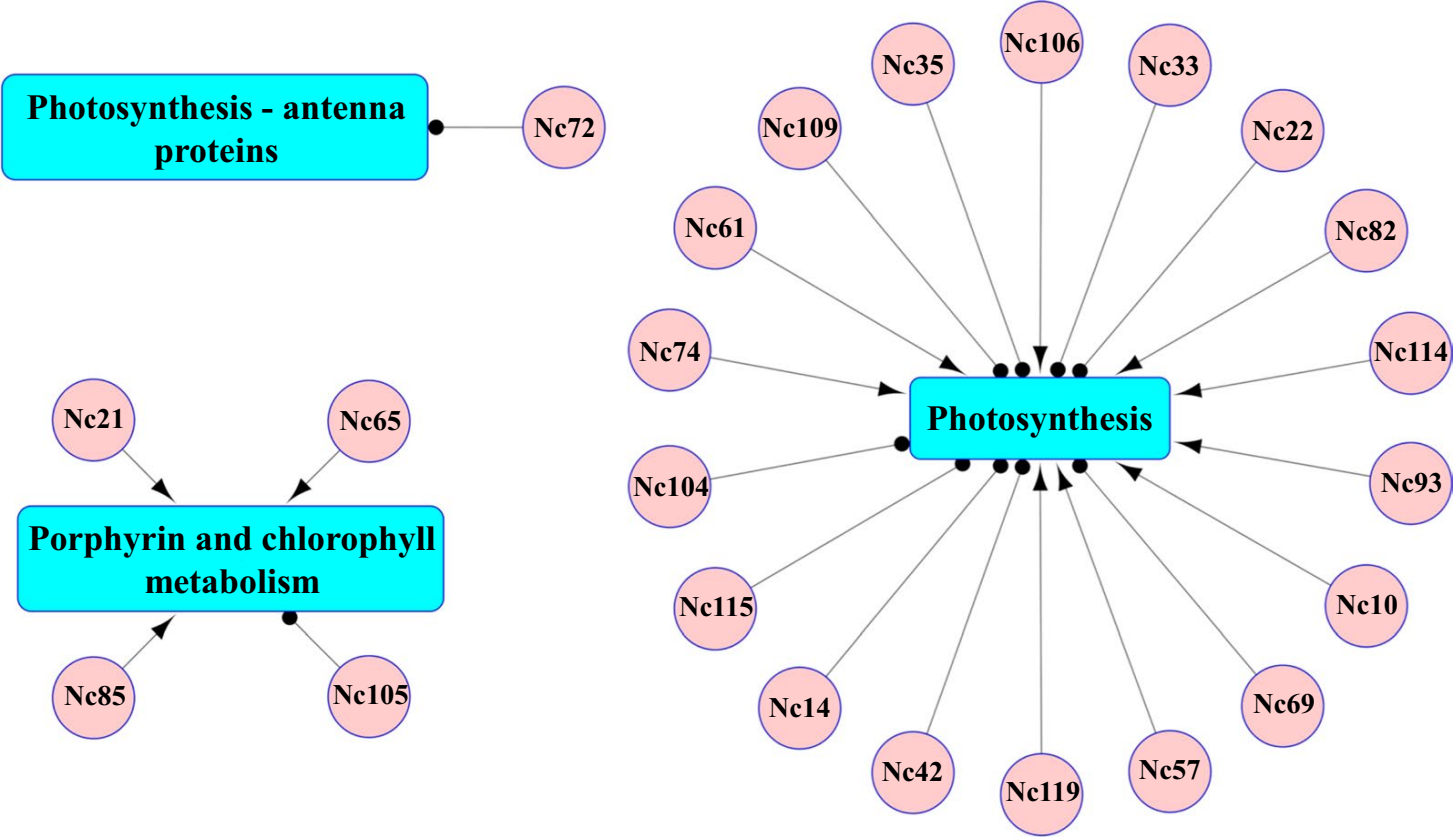
Representation of the top three biofuel-related pathways in the *trans*-encoded sRNA regulatory network of *Synechocystis.* A *pink* node represents an sRNA and a *turquoise rectangle* represents a metabolic pathway. An *arrow* between an sRNA and a metabolic pathway represents the predicted target gene for an sRNA on the positive strand, while a *dotted line* between an sRNA and a metabolic pathway represents the predicted target gene for an sRNA on the negative strand

According to our previously weighted gene co-expression network analysis (WGCNA) with the *Synechocystis* proteomic data [48], photosynthesis antenna proteins, porphyrin and chlorophyll metabolism and photosynthesis were identified as the top three significant biofuel-specific responsive pathways after cells were treated with exogenous biofuels [48]. Therefore, considering the association of responsive sRNA with these three pathways (Fig. 5) and the fold change of responsive sRNAs, a total of 20 sRNAs were chosen for quantitative real time polymerase chain reaction (qRT-PCR) validation. To quantitatively confirm results from the sRNA analysis, all samples were collected in the same manner as previous studies [21–27] and were used for qRT-PCR analysis (sRNA and primer sequences in Additional file 9: Table S4). The qRT-PCR results confirmed that a majority of sRNAs were significantly down- or up-regulated under specific stress conditions. Overall, comparative qRT-PCR and deep-sequencing analysis of sRNAs suggested a positive correlation with *Pearson* correlation coefficients of 0.57–0.81 (Additional file 10: Figure S6), demonstrating the high reliability of the sRNA-seq analysis.

### Validation of putative sRNAs involved in biofuels tolerance

**Fig. 6:**
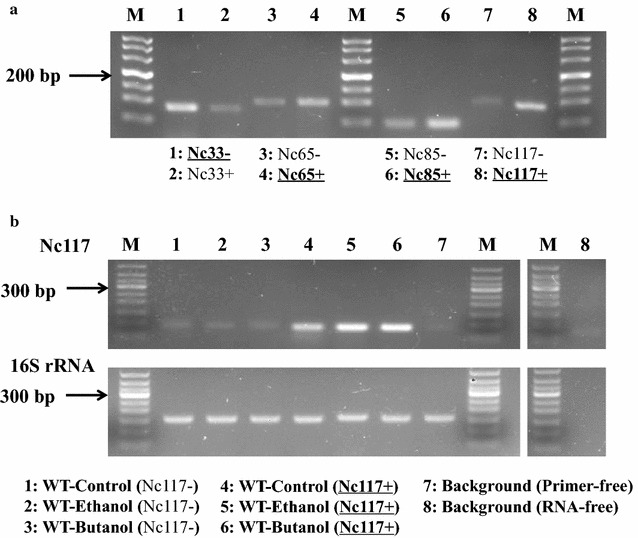
Experimental determination of transcriptional orientation by two-step RT-PCR and verification of biofuel-responsive sRNA. **a** Four biofuel-response sRNA orientations by two-step RT-PCR. “+” denotes the orientation of sRNA on the positive genome strand, “−” denotes the orientation of sRNA on the negative genome strand. The *bold and underlined names* indicate the determined orientation of an sRNA. **b** Ethanol- and butanol-responsive expression of Nc117 validation using two-step RT-PCR. The *upper part* indicates Nc117 validation, the control background primer-free or RNA-free for Nc117 were conducted with no primers or reverse transcriptase added during first step reverse transcription. The *lower part* shows 16S rRNA indicates an internal control, all samples with random primers during reverse transcription, except for lane 8 RNA-free without reverse transcriptase. All samples were collected in the logarithmic growth phase

Based on the bioinformatics analysis combined with qRT-PCR validation, a total of 18 sRNAs were selected for constructing overexpression and suppression strains for further functional and phenotypic confirmation. As the sRNA-seq in this study provided no information on the orientation of sRNA gene candidates, the constructed strains with a selected sRNA expressed in either the positive (named as WT/pJA2-*sRNA*+) or negative (named as WT/pJA2-*sRNA*−) strand direction were constructed, corresponding to an overexpression or suppression strain, respectively (sRNA and primer sequences provided in Additional file 11: Table S5). All mutants and the wild-type *Synechocystis* were monitored for growth under the biofuel stress conditions in shake flasks. The results showed that only four of the 18 constructs had visible differential growth phenotypes under biofuel conditions: pJA2-*nc33*−, pJA2-*nc65*+, pJA2-*nc85*+, and pJA2-*nc117*+. In addition, the constructed strains carrying the four sRNAs caused no change in growth phenotype under the control growth condition. To validate that these sRNAs were indeed stably overexpressed, RT-PCR verification was conducted between wild-type and pJA2-*ncRNA* mutants (Additional file 12: Figure S7). Moreover, a two-step RT-PCR procedure that can differentially amplify the target sRNA transcript from one direction in comparison to potential transcription from the opposite direction was applied to determine the transcriptional direction of the four sRNAs (Fig. 6a). Reproducible results showed that Nc33 was on the negative strand, while the other three sRNAs were on the positive strand.

### Characterization of the overexpression strain of Nc117 sRNA

**Fig. 7:**
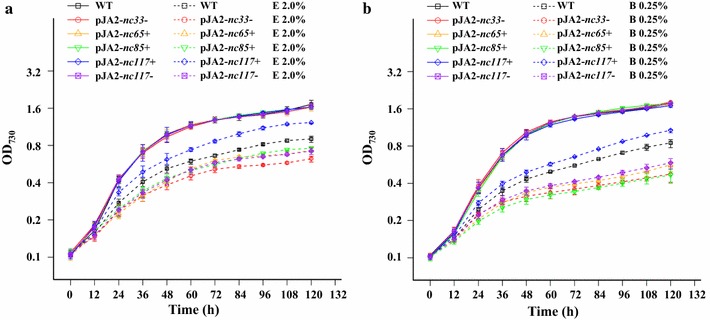
Growth curves of WT and overexpression strains under control and biofuel stress conditions. **a** WT, pJA2-*nc33*−, pJA2-*nc65*+, pJA2-*nc85*+, pJA2-*nc117*+, and pJA2-*nc117*− in normal BG11 medium with or without 2.0% (*v/v*) ethanol (E 2.0%). **b** WT, pJA2-*nc33*−, pJA2-*nc65*+, pJA2-*nc85*+, pJA2-*nc117*+, and pJA2-*nc117*− in normal BG11 medium with or without 0.25% (*v/v*) butanol (B 0.25%). The *error bar* represents the calculated standard deviation of the measurements of three biological replicates

The WT/pJA2-*nc117*+ strain overexpressing *nc117*, located between *slr0550* and *slr0551*, grew faster than the WT under 1.5–2.0% (*v/v*) ethanol and 0.20–0.25% (*v/v*) butanol conditions. No difference was observed when they both grew in normal BG11 medium (Fig. 7a, b). In addition, the cell aggregation commonly seen in WT under biofuel stress was significantly alleviated in the pJA2-*nc117*+ strain (data not shown). However, no visible growth difference was observed when the strain grew under hexane (0.6–0.8%), high salt (3–4%), or nitrogen starvation conditions (data not shown). These results were consistent with the up-regulation of Nc117 only under ethanol and butanol conditions revealed by sRNA deep-sequencing and qRT-PCR analysis (Additional file 9: Table S4) and suggested that *nc117* overexpression could confer *Synechocystis*-specific resistance to both ethanol and butanol. Two-step RT-PCR analysis for Nc117 under ethanol and butanol conditions further confirmed these results (Fig. 6b). To further confirm these, the WT/pJA2-*nc117*− (Nc117 suppression) strain was constructed and validated by two-step RT-PCR (Additional file 13: Figure S8). In accordance with our expectation, the pJA2-*nc117*− showed a reverse phenotype (Fig. 7a, b) that is more sensitive to ethanol or butanol stress, demonstrating exclusively that Nc117 played an important role in biofuel tolerance in *Synechocystis*.

The sRNA homologs from six strains of *Synechocystis* were subject to CopraRNA for prediction of potential target genes, as no homolog of *nc117* was found in other species of cyanobacteria. Based on the integrated analysis with transcriptomic datasets, the potential target genes of Nc117 were predicted and listed in Additional file 14: Table S6. The results showed that although most potential targets were annotated as hypothetical proteins, some targets were involved in pathways related to biofuel response, including transporter proteins and cell wall/membrane modifications [6]. In addition, functional categorization of the predicted target genes for Nc117 showed that the peptidoglycan biosynthesis pathway was significantly enriched. As previous analyses reported, changes in membrane composition or membrane-associated proteins could improve the viability of *E. coli* and cyanobacteria under fatty acid and alcohol stress conditions [49, 50]. The increase in the degree of cross-linking between constituents of the cell wall and modifications of the cell wall hydrophobicity protected cells against the toxic effects of lipophilic compounds [6, 51, 52]. Peptidoglycan as an important component of cell wall in *Synechocystis* cells could be modified at the cell surface as a mechanism for improving tolerance to biofuels [6]. Moreover, Nc117 was also found significantly up-regulated under cold stress condition (15 °C for 30 min) (“Ncr1600” in the study) [10]. It is widely accepted that low temperature can affect the fluidity and function of cellular membranes, suggesting possible roles for Nc117 targets in fatty acid or membrane modification and metabolism. Further experimental validation of Nc117 sRNA target genes, including the generation of a deletion mutant, is still needed.

### Characterization of overexpression strains of photosynthesis-related sRNAs

Growth analysis showed that three sRNA overexpression strains, including pJA2-*nc33*−, pJA2-*nc65*+, and pJA2-*nc85*+, grew poorly in BG11 medium supplemented with ethanol or butanol, suggesting that overexpression of sRNAs led to increased sensitivity to biofuels, which could be negatively involved in biofuel tolerance (Fig. 7a, b). The genetic locations of *nc33*, *nc65*, and *nc85* sRNA genes are provided in Table 1. Interestingly, target enrichment analysis showed that Nc65 and Nc85 were enriched in porphyrin and chlorophyll metabolism and Nc33 in photosynthesis; both metabolic pathways were identified as biofuel-specific responsive pathways in our previous study [48]. This was consistent with a previous study that showed that proteins related to photosynthesis and chlorophyll concentration were up-regulated upon ethanol exposure in *Synechocystis* cells [23], indicating that the responses of sRNAs and protein to biofuels could point to similar mechanisms. However, suppression of the three sRNAs (i.e., pJA2-*nc33*+, pJA2-*nc65*− and pJA2-*nc85*−) did not improve *Synechocystis* biofuel tolerance (data not shown), suggesting that indirect mechanisms may be involved.

**Table 1:** Selected *Synechocystis* sRNAs for two-step RT-PCR analysis and relative abundance (ranked by sRNA abundance)

sRNA	Location	Dir*	∆*G* (kcal/mol)	5′ gene	Annotation	Distance (bp)	3′ gene	Annotation	Distance (bp)	Relative abundance	E24	E48	
*nc64*	1,832,231–1,832,345	<>>	−40.6	*sll1382*	Ferredoxin	302	*slr1474*	Hypothetical protein	15	Nc64 (4.5S rRNA)	1.01	0.51	
*nc18*	552,943–553,017	<<<	−16.8	*sll1635*	FAD-dependent thymidylate synthase	76	*sml0005*	Photosystem II reaction center protein K	48	Nc18 (Ncl0200)	0.965	0.837	
*nc1*	2948–3061	<><	−14.1	*sll0558*	Hypothetical protein	75	*sll1214*	Magnesium-protoporphyrin IX monomethyl ester cyclase	131	Nc1	0.881	1.274	
*nc121*	3,316,752–3,317,151	><<	−108.3	*slr1464*	Hypothetical protein	17	*ssl2667*	NifU protein	212	Nc121 (tmRNA)	1.315	0.646	
*nc81*	2,250,490–2,250,580	><>	−34.9	*ssr3307*	Preprotein translocase subunit SecG	16	*slr1946*	Hypothetical protein	131	Nc81	1.133	3.577	
*nc110*	3,138,648–3,138,738	<<<	−18.2	*sll0737*	Hypothetical protein	44	*sll0048*	Hypothetical protein	179	Nc110 (SyR5)	1.19	2.171	
*nc26*	842,648–842,758	<<>	−40.5	*sll1799*	50S ribosomal protein L3	35	*slr1894*	Hypothetical protein	347	Nc26	1.201	0.635	
*nc41*	1,190,736–1,190,822	<>>	−22.2	*sll1783*	Hypothetical protein	353	*slr1852*	Hypothetical protein	45	Nc41 (Ncr0480)	0.678	1.053	
*nc70*	1,886,895–1,887,070	>><	−47.8	*slr1288*	Hypothetical protein	158	*sll1166*	Hypothetical protein	89	Nc70 (6S rRNA)	0.743	0.701	
*nc7*	219,563–219,783	<<<	−66.9	*sll1029*	Carbon dioxide concentrating mechanism protein CcmK	46	*sll1027*	NADH-glutamate synthase small subunit	196	Nc7	1.303	0.632	
*nc122*	3,323,090–3,323,230	<>>	−25.8	*sll0629*	Photosystem I subunit X	327	*slr0653*	RNA polymerase sigma factor RpoD	45	Nc122 (Ncr1650)	1.042	0.848	
*nc60*	1,748,923–1,749,070	<<>	−44.8	*sll1247*	Hypothetical protein	30	*slr1351*	UDP-*N*-acetylmuramoylalanyl-d-glutamyl-2,6-diamino-pimelate-d-alanyl-d-alanine ligase	76	Nc60 (SyR7)	0.912	1.229	
*nc117*	3,250,549–3,250,647	>>>	−10.1	*slr0550*	Dihydrodipicolinate synthase	262	*slr0551*	Hypothetical protein	353	Nc117 (Ncr1600)	1.519	1.67	
*nc33*	1,004,885–1,005,121	<<<	−40.2	*sll1454*	Nitrate reductase	108	*sll1453*	Nitrate transport protein NrtD	60	Nc33	1.607	0.815	
*nc85*	2,383,750–2,383,875	>><	−42.3	*slr0381*	Hypothetical protein	114	*sll0330*	3-Ketoacyl-ACP reductase	74	Nc85	1.13	2.111	
*nc65*	1,840,822–1,840,980	<><	−25.4	*sll1376*	Bifunctional sterol desaturase/short chain dehydrogenase	331	*sll1374*	Melibiose carrier protein	76	Nc65	1.068	1.089	

sRNA	E72	B24	B48	B72	H24	H48	H72	S24	S48	S72	N24	N48	N72
*nc64*	1.519	1.254	0.709	2.69	1.387	0.609	1.572	2.061	1.09	2.143	0.674	0.387	1.131
*nc18*	0.834	1.289	1.001	0.596	0.972	0.634	0.221	0.366	0.298	0.278	0.111	0.048	0.05
*nc1*	0.858	0.927	1.584	0.804	0.72	0.844	0.548	0.642	0.993	0.748	0.189	0.188	0.115
*nc121*	0.853	1.452	0.36	1.469	2.311	0.544	1.534	1.107	0.853	1.626	0.888	0.768	3.146
*nc81*	0.882	1.641	7.166	1.058	1.326	1.784	0.381	0.691	1.021	0.261	2.293	4.651	1.237
*nc110*	1.04	1.335	2.358	1.799	3.616	1.22	0.647	1.097	1.398	0.538	1.276	3.595	3.599
*nc26*	0.64	0.852	0.76	0.653	0.75	0.813	0.489	2.159	1.544	1.709	0.587	0.225	0.163
*nc41*	0.973	0.959	0.955	0.665	0.969	1.249	0.619	0.207	0.314	0.296	0.319	0.209	0.149
*nc70*	1.635	0.896	0.465	1.346	1.511	0.577	1.472	1.005	0.865	1.809	1.086	0.876	2.212
*nc7*	1.178	1.08	0.454	0.981	1.001	0.572	1.259	1.193	1.054	2.444	0.398	0.311	1.158
*nc122*	1.031	0.864	0.968	0.823	0.723	0.875	1.059	0.58	0.768	0.677	1.531	1.263	1.962
*nc60*	1.078	0.504	0.73	0.506	0.979	0.512	0.365	0.179	0.201	0.26	1.438	0.965	0.573
*nc117*	1.293	1.743	1.623	1.035	1.101	0.851	0.866	2.083	1.12	1.12	0.875	1.061	1.404
*nc33*	1.282	2.254	0.773	1.172	1.239	0.905	0.788	4.606	2.041	3.843	3.152	3.674	4.558
*nc85*	1.39	1.305	2.13	1.212	0.898	0.964	0.472	1.077	1.402	0.769	4.358	4.151	3.34
*nc65*	0.927	1.165	1.148	0.987	1.223	0.99	0.857	1.044	1.129	0.94	1.392	1.526	1.364

* Direction of sRNA and adjacent genes

To evaluate whether biofuel tolerance of *Synechocystis* related to photosynthesis and chlorophyll contents, cells of WT, pJA2-*nc33*−, pJA2-*nc65*+, pJA2-*nc85*+, and pJA2-*nc117*+ grown under normal BG11, 2.0% (*v/v*) ethanol, and 0.25% (*v/v*) butanol at the exponential growth phase were also collected for flow cytometric analysis. The result showed that the cell morphology and chlorophyll content of five tested strains were similar under normal conditions (Additional file 15: Figure S9A, B). In addition, the main peak of FL3-H that indicates chlorophyll intensity per active cell did not change in the WT and the mutants under ethanol or butanol stress conditions (Additional file 15: Figure S9C, D).

## Conclusions

Recent progress in metabolic engineering and synthetic biology has demonstrated great potential in the use of photosynthetic cyanobacteria for biofuel production. However, the highest production of biofuels in renewable cyanobacterial systems still largely lags behind yeast or other native (heterotrophic) systems [53]. Previous studies suggested that low tolerance to biofuel toxicity could be one reason for the low productivity in the cyanobacterial chassis [3], which prompted us to initiate the rational construction of a highly tolerant chassis. Among various promising approaches, sRNAs, especially those with global regulatory effects, have been proposed as powerful tools for chassis engineering [29]. However, very limited information is currently available for sRNAs related to biofuel tolerance in cyanobacteria.

To identify the sRNAs involved in the adaptation of model cyanobacterial *Synechocystis* sp. PCC 6803 to biofuel growth conditions, samples from five growth conditions were collected for sRNA sequencing. Bioinformatics analysis and experimental validation led to the identification of the first sRNA (Nc117) that could improve the tolerance of *Synechocystis* to both exogenous ethanol and butanol. In contrast, overexpression of three other sRNAs with predicted functions related to photosynthesis made cells more sensitive to ethanol and butanol. A few highly abundant and structure-stable sRNAs of *Synechocystis*, which can function by interacting with other biomolecules to enable cell fitness, were studied. Although the individual function of sRNAs at the molecular level must be elucidated in the future, our results provide important knowledge of potential sRNA targets and demonstrate a new strategy of engineering adaptive sRNAs to improve tolerance to biofuels in *Synechocystis*.

Finally, the potential limitations of developing a tolerant chassis alone should be acknowledged. For example, some studies have shown that simply increasing tolerance does not necessarily correlate with increased yield [6]. Tolerance mechanisms identified under exogenous biofuel stress may not be identical to those caused by biofuels synthesized internally [54]. In the future, further advances may target other important aspects, such as improving metabolic flux and enhancing reductive forces for the cyanobacterial chassis. This research will eventually lead economically feasible cyanobacterial cell factories in the future.

## Methods

### Strains, culture, and stress conditions for sRNA samples

*Synechocystis* sp. PCC 6803 was grown in BG11 medium (pH 7.5) under a light intensity of approximately 50 μmol photons/m^2^ s^1^ in an illuminating incubator (HNY-211B Illuminating Shaker, Honour, China) at 130 rpm and 30 °C with a starting cell density of OD_730_ = 0.1 [21, 23–27]. Cell density was measured with a UV-1750 spectrophotometer (Shimadzu, Japan). For growth and stress treatment, 10 mL fresh cells at OD_730_ approximately 0.5 was collected by centrifugation and inoculated into 50 mL BG11 liquid medium in a 250-mL flask. Ethanol 1.5% (*v*/*v*), butanol 0.2% (*v*/*v*), hexane 0.8% (*v*/*v*), and salt 4% (*w*/*v*) were added at the beginning of cultivation. The nitrogen starvation condition was established by excluding NaNO_3_ from the BG11 medium. Two milliliter of culture was sampled and measured at OD_730_ every 12 h. Finally, a total of 18 culture samples including six conditions at three time points (i.e., 24, 48, and 72 h) were collected for RNA preparation.

### RNA preparation and cDNA synthesis

Approximately 10 mg of cell pellets were frozen in liquid nitrogen immediately after centrifugation at 8000×*g* for 10 min at 4 °C, and cell walls were broken by liquid nitrogen mortar grinding. Cell pellets were re-suspended in TRIzol reagent (Ambion, Austin, TX) and mixed well by vortexing. Total RNA extraction was achieved using a miRNeasy Mini Kit (Qiagen, Valencia, CA). Contaminating DNA in RNA samples was removed with DNase I according to the instructions for the miRNeasy Mini Kit (Qiagen, Valencia, CA). The RNA quality and quantity were determined using an Agilent 2100 Bioanalyzer (Agilent, Santa Clara, CA) and subjected to complementary DNA (cDNA) synthesis. The RNA integrity number (RIN) of every RNA sample used for sequencing was more than 7.0. To enrich small RNA for the sRNA-seq analysis, the pool of total RNAs was size-selected, and transcripts smaller than 250 nucleotides (nt) was used to prepare cDNA libraries. For each sample, 500 ng size-fractionated sRNAs were subjected to cDNA synthesis using a NuGEN OvationW Prokaryotic sRNA-Seq System according to the manufacturer’s protocol (NuGEN, San Carlos, CA). The resulting double-stranded cDNA was purified using the MinElute Reaction Cleanup Kit (Qiagen, Valencia, CA).

### Library preparation for sRNA and sequencing

The double-stranded cDNA was subjected to library preparation using the Illumina TruSeq™ RNA Sample Preparation Kit (Illumina, San Diego, CA), through a four-step protocol including end repairing, addition of adenylate 3′ ends, adapter ligation, and cDNA template enrichment. The amplification program were: 98 °C 30 s; 98 °C 10 s, 60 °C 30 s, 72 °C 30 s for 15 cycles; 72 °C for 5 min, and hold at 4 °C. To determine the quality of the libraries, a QubitW 2.0 Fluorometer and Qubit™ dsDNA HS (Invitrogen, Grand Island, NY) were first used to determine the DNA concentration of the libraries, and a FlashGel DNA Cassette (Lonza, USA) or Agilent Technologies 2100 Bioanalyzer (Agilent, Santa Clara, CA) was used to determine the product size of the libraries, with good libraries typically 250–300 bp. The product was used directly for cluster generation using an Illumina Solexa Sequencer according to the manufacturer’s instructions.

RNA 2 × 100 bp paired-end sequencing was performed using the standard protocol for the Illumina Genome Analyzer IIx. The cDNA library for each sample was loaded onto a single lane of an Illumina flow cell. The image deconvolution and calculation of quality values were performed using a Goat module (Firecrest *v*.1.4.0 and Bustard *v*.1.4.0 programs) with Illumina pipeline *v*.1.4. Sequenced reads were generated by base calling using the Illumina standard pipeline.

### sRNA data analysis

Genome sequence and annotation information for *Synechocystis* were downloaded from NCBI (ftp://ftp.ncbi.nlm.nih.gov/genomes). The sRNA sequence reads were pre-processed using an NGS QC Toolkit (*v.* 2.3) to remove low-quality bases and adapter sequences [55]. Reads after QC were aligned to the *Synechocystis* genome using the Burrows-Wheeler Alignment tool software (*v.* 0.7.10) [56] with perfect match parameters. As the length of some small RNAs might be shorter than 100 nucleotides, we applied a strategy of 50 cycles of read trimming and re-mapping to detect bacterial sRNAs between 50 and 100 bp [7]. Briefly, we re-extracted the reads that did not match the *Synechocystis* genome from the aligned SAM files and trimmed one low-quality base from 3′ or 5′ end of these reads (observed by FastQC software). We then re-mapped these trimmed reads to the *Synechocystis* genome. If reads still did not match, the entire process was repeated until the reads matched the *Synechocystis* genome. Reads shorter than 50 bp that could align were discarded. Finally, we use Samtools [57] software (*v.* 0.1.19) to merge all original SAM files from the same sample.

After sRNA pair-end reads were mapped to the *Synechocystis* genome, Bedtools (*v.* 2.20.1) [58] was used to calculate read mapping statistics for each sample from BAM files generated with Samtools. The coverage of each nucleotide was calculated by counting the number of reads mapped at corresponding nucleotide positions in the genome. To normalize the sRNA expression level in different samples, we removed nucleotide coverage deep in the rRNA operon and tRNA gene regions, summed all the nucleotide coverage in the remaining genome regions as total mapped bases, and normalized them to 100,000,000 bases. This created a reads base for all samples, which corresponded to an approximately 25× sequence depth for the whole *Synechocystis* genome.

The sRNAs were identified using a multi-tiered approach. We first searched for enriched regions of sRNA expression and then estimated their 3′ and 5′ positions through a manual correction. For highly transcribed sRNA, we defined s_*i*_ as the coverage depth at nucleotide *i* in the *Synechocystis* genome and then set a real expression level for a given sRNA at each nucleotide of at least 50× sequencing depth. We looked for the first location in s_*i*_ > 50 representing the start site of an sRNA and then determined whether s_*i*+1_ > 50, until s_*i*+*j*_ < 50 is the end site of a sRNA. For low-transcribed sRNAs, we used previously reported sRNA gene candidates as references and only retained sRNA with an obvious reads coverage reduction 50 bp upstream or downstream of the adjacent region. To obtain a robust sRNA mapping with a low false positive rate, especially for the induced sRNAs, we retained the sRNAs that were repeatedly observed in at least in three samples across all 18 samples. Finally, manual correction of sRNA boundaries was conducted to identify a point of max rapid coverage decline, considered as the end of the sRNA. We used an *R* script based on the core code from Kopf [10] to produce a PDF file to display distribution of sRNA reads in the *Synechocystis* genome (Additional file 2: Figure S1) and four plasmids including pSYSM, pSYSA, pSYSG, and pSYSMX (Additional file 16: Figure S10, Additional file 17: Figure S11, Additional file 18: Figure S12, Additional file 19: Figure S13, respectively). Two adjacent sRNAs located on the same strand shorter than 50 bp with the same expression trend were merged as a single sRNA. The sRNAs shorter than 50 bp were discarded. By determining the 5′ and 3′ ends and inspecting the locations, these potential sRNA genes were classified into *trans*-encoded sRNAs, *cis*-antisense sRNAs and UTRs of mRNAs based on their location and were annotated as ncRNA (nc), asRNA (as) and UTR (U), respectively.

To overcome challenge that potential identified sRNAs due to reads matched to multiple locations [59], repetitive regions in the *Synechocystis* genome were searching by BLAST software. Fragments with identity >80%, *E* value <1*e* ^−5^ and length >50 bp were considered a repeat region. The IS was predict using Isfinder software [60]. The sRNA-seq data was compared with the Nr database and Rfam database with a 1*e* ^−10^ cut-off. Candidate ORFs and RBSs were predicted by Glimmer [61] and RBSfinder [62], respectively. *Rho*-independent terminators in *Synechocystis* were searched for using TransTermHP [63] with standard settings. RNA secondary structure and **∆** *G* analyses of sRNAs were performed using RNAfold software [45].

### Quantification and statistical analysis of sRNAs

For all comparisons, the aligned total base counts were normalized to obtain relative levels of expression. We defined s_*i*_ as the coverage depth at nucleotide *i* in an sRNA, summed coverage depth at each nucleotide for an sRNA and divided by its length. The sRNA expression level was calculated as: $\mathop \sum \nolimits_{i}^{i + j} {{{\text{s}}_{i} } \mathord{\left/ {\vphantom {{{\text{s}}_{i} } j}} \right. \kern-0pt} j}$. Differentially expressed sRNAs were identified using the *R* package from DESeq software [46] with identified sRNAs as input. For comparison, the resulting *p* values were adjusted using Benjamini and Hochberg’s approach for controlling the false discovery rate. The sRNAs with fold change >1.5 and adjusted *p* values <0.05 were assigned as significantly differentially expressed. Correspondence analysis was conducted using *R* software. The annular heatmap representation was conducted using the OmicCircos package [64] in *R* software.

### Construction of sRNA regulatory networks

For construction of sRNA regulatory networks, each *trans*-encoded sRNA target prediction was first performed with CopraRNA software [65] using sRNA homologs from six strains of *Synechocystis* 6803 genomes (NC_000911, NC_017038, NC_017039, NC_017052, NC_017277, and NC_020286) as inputs. The top 100 predictions obtained from CopraRNA with a free-energy cut-off of −10 kcal/mol were retained to remove potential false positive targets [34]. Later, a *Pearson* correlation analysis with a set of paired RNA samples in previous studies was used to further improve the accuracy of target prediction. Only correlation <−0.4 or >0.4 and *p* values <0.05 by Fisher’s exact test in two-sided analysis were kept to reduce false positive target prediction. The approach is a modification of previous reports where only anti-correlated relationships were retained for sRNA regulatory network construction [66] as positive regulation of sRNA also found in *Synechocystis* [67]. To assess sRNAs-regulated metabolic pathways, we performed functional enrichment analyses for each *trans*-encoded sRNA (see “Functional enrichment analysis” section). Only pathways containing at least two target genes with hypergeometric test *p* values <0.05 were considered enriched metabolic pathways potentially regulated by sRNAs (Additional file 20: Table S7). Finally, we generated an sRNA regulatory network after assembling all significantly enriched target results. A display of the sRNA regulatory network was conducted with Cytoscape software [68].

### Functional enrichment analysis

The metabolic pathway enrichment analysis of genes was conducted according to the KEGG (Kyoto Encyclopedia of Genes and Genomes) pathway database using the following hypergeometric test formula:

$$P = 1 - \mathop \sum \limits_{i = 0}^{m - 1} \frac{{\left( {\begin{array}{*{20}c} M \\ i \\ \end{array} } \right)\left( {\begin{array}{*{20}c} {N - M} \\ {n - i} \\ \end{array} } \right)}}{{\left( {\begin{array}{*{20}c} N \\ n \\ \end{array} } \right)}}$$

*N* is the total number of genes with KEGG pathway annotation information, *M* is the number of genes with a given KEGG pathway annotation, *n* is the number of sRNA target genes with all KEGG pathway annotation information and *m* is the number of the sRNA target genes with a given KEGG pathway annotation. KEGG pathways with *p* values less than 0.05 were considered enriched response pathways.

### qRT-PCR analysis and two-step RT-PCR analysis

The RNA samples used in sRNA sequencing and qRT-PCR were prepared from identical cultures, and qRT-PCR analysis was performed as previously described [21]. Quantification of sRNA expression was determined according to a standard process of qRT-PCR that used serial dilutions of known concentrations of chromosomal DNA as a template to construct a standard curve. A total of 20 sRNAs were selected for verification and 16S rRNA was used as an internal control. Three technical replicates and three biological replicates were analyzed for each sRNA. The data analysis was carried out using the StepOnePlus analytical software (Applied Biosystems, Foster City, CA). Briefly, the amount of relative gene transcript was normalized by that of 16S rRNA in each sample, and the data presented were ratios of the amount of normalized transcripts in the treatment between the stress-treated and normal conditions.

In two-step RT-PCR analysis, a specific set of primer pairs located in the inner boundary of sRNA was designed for each sRNA (Additional file 9: Table S4). As Additional file 21: Figure S14 shows, two opposite RT primers that sit inside the sRNA region were separately added to approximately 200 ng of total RNA from first-strand cDNA synthesis at 40 °C for 1 h using the RevertAid™ First Strand Synthesis Kit (Fermentas, USA). Both nested primers of the sRNAs were added to 1 μL of the two separate first-strand cDNA products for amplification using Dream Taq Green PCR Master Mix (Thermo Scientific). The PCR cycling was as follows: 95 °C 2 min, then 95 °C 30 s, 55 °C 30 s or 57 °C 30 s, 72 °C 15 s for 35 cycles, followed by a 5-min final extension at 72 °C. The PCR products were separated on 3.0% agarose gels.

### Construction and analysis of sRNA mutant strains

Gene expression vector pJA2, kindly provided by Prof. Paul Hudson (KTH Royal Institute of Technology of Sweden), was used to overexpress the sRNAs. All sRNAs were cloned under the control of the *psbA2* promoter. Briefly, the pJA2 backbone was amplified by PCR, treated with *Dpn*I and digested with *Bam*HI and *Xba*I to create cohesive ends. The sRNA sequence was PCR-amplified using primers pJA2-*sRNA*-F and pJA2-*sRNA*-R and cloned into the *Bam*HI/*Xba*I sites of pJA2, resulting in the recombinant plasmid pJA2-*sRNA* (details in Additional file 22: Figure S15). The plasmid was introduced into the WT by electro-transformation as previously described [69]. Positive clones grew on BG-11 agar plates with 10 μg/mL kanamycin and were confirmed by colony PCR analysis. The sRNA overexpression strains were designated WT/pJA2-*sRNA*.

### Phenotypic analysis

*Synechocystis* and sRNA mutant strains were grown under the same culture condition with sRNA sampling starting at a cell density of OD_730_ = 0.1. For biofuel treatment, 2.0–2.5% (*v/v*) ethanol, 0.20–0.25% (*v/v*) butanol, or 0.8–1.0% (*v/v*) hexane was added at the beginning of the cultivation. For salt treatment, BG11 with 5% NaCl (*w/v*) was prepared and sterilized. Next, 3–4% (*w/v*) NaCl of BG11 was prepared by adjusting the ratio between normal and 5% NaCl (*w/v*) BG11 at the beginning of cultivation. For nitrogen starvation treatment, fresh cells at the same (logarithmic) phase were collected by centrifugation at 1500×*g* at 4 °C and washed twice with nitrogen depletion BG11 medium. Cells were inoculated into nitrogen-depleted BG11 liquid medium in flasks. Cell density was measured on a UV-1750 spectrophotometer (Shimadzu, Japan) at OD_730_. Growth experiments were repeated at least five times to confirm growth patterns.

### Flow cytometric analysis

Flow cytometric analysis was performed using a fluorescence-activated cell sorting (FACS) Calibur cytometer (Becton–Dickinson) equipped with a 488-nm blue laser with the following settings: forward scatter (FSC), E00 log; side scatter, 400 V. Control and stress-treated cells were harvested at 48 h, washed twice with phosphate buffer (pH 7.2), and re-suspended in the same buffer to a final OD_580_ of 0.3 (approximately 1.5 × 10^7^ cells/mL^1^). A total of 5 × 10^4^ cells were used for each analysis according to the published method [70]. Chlorophyll fluorescence was detected by the FL3 channel with a 670/LP filter. The data analysis was conducted using CellQuest software, version 3.1 (Becton–Dickinson).

## Additional files

**Additional file 1: Table S1.** sRNA mapping statistics of different samples.

**Additional file 2: Figure S1.** Genome-wide visualization of all sRNA mapping data in the chromosome genome of *Synechocystis* sp. PCC 6803. The figure can be divided into two regions, and the upper region can be further divided into three parts corresponding to samples collected at 24, 48 and 72 h. Each part provides detailed visualization of sRNA mapping depth for each nucleotide. Each sample is distinguished using a different color. The lower part can be further divided into ten parts, and each part shows detailed sRNA and gene information. From up to down: (1) all identified sRNAs in this study, (2, 3) all identified sRNAs in the positive and negative chains of Study A—Mitschkea et al. [9], (4, 5) All identified sRNAs in the positive and negative chains of Study B—Kopf et al. [10], (6, 7) all identified sRNAs in the positive and negative chains of Study C—Xu et al. [11], (8) Gene name and annotation in the NCBI database of *Synechocystis* PCC 6803, (9) Open reading frame prediction of *Synechocystis* sp. PCC 6803, (10) Characteristic sequences identified in *Synechocystis* sp. PCC 6803 genome, including genome repeat regions, insert sequences, ribosomal binding sites and *Rho*-independent transcription terminators.

**Additional file 3: Table S2.** Identification, quantitative and differential expression analysis of sRNAs in *Synechocystis.*

**Additional file 4: Table S3.** Detailed comparison and classification of *trans*-encoded sRNAs in *Synechocystis.*

**Additional file 5: Figure S2.** Detailed visualization of sRNA mapping data of 133 *trans*-encoded sRNAs in *Synechocystis*. Detailed description is the same as Additional file 2: Figure S1.

**Additional file 6: Figure S3.** Experimental verification of the top abundant sRNAs and determination of transcriptional orientation by two-step RT-PCR. “+” denotes the orientation of the sRNA on the positive genome strand, “−” denotes the orientation of the sRNA on the negative genome strand. The bold and underlined names indicate the determined orientation of an sRNA, and “rc” denotes the reverse complement direction of the known sRNAs.

**Additional file 7: Figure S4.** Secondary structures predicted for abundant sRNAs. Color bar represents base-pair probabilities within sRNAs.

**Additional file 8: Figure S5.** Visualization of *trans*-encoded sRNAs regulatory network in *Synechocystis*. Detailed description is the same as Fig. 5.

**Additional file 9: Table S4.** sRNA-related primers for qRT-PCR analysis and two-step RT-PCR combined with qRT-PCR results.

**Additional file 10: Figure S6.** Correlation between qRT-PCR and sRNA-seq analyses for selected genes. For sRNA-seq (horizontal coordinate), values represent log_2_ fold change of sRNA under stress conditions compared to WT. For qRT-PCR (vertical coordinate), values represent the mean log_2_ fold changes in sRNA of three technical and three biological replicates under stress conditions compared to WT. The error bar represents the standard deviation of three replicates. Sample names and *Pearson* correlation coefficients are indicated at the right lower corner of each plot.

**Additional file 11: Table S5.** sRNA-related primers used for mutant construction.

**Additional file 12: Figure S7.** RT-PCR verification for biofuel responsive sRNA mutant construction. The upper portion is the validation for the target sRNAs, while the lower part is an internal control of 16S rRNA.

**Additional file 13: Figure S8.** Two-step RT-PCR validation for Nc117 overexpression and suppression strains. The upper portion is the target sRNA validation; the lower portion is 16S rRNA used as an internal control.

**Additional file 14: Table S6.** Target information for Nc117 based on CopraRNA and integrated transcriptomic analysis.

**Additional file 15: Figure S9.** Flow-cytometric analysis of WT, pJA2-*nc33*−, pJA2-*nc65*+, pJA2-*nc85*+ and pJA2-*nc117*+ strains. Forward scatter (FSC) is related to cell size and the FL3 channel with the 670/LP filter, which is related to chlorophyll fluorescence. The *y*-axis was normalized according to pixel count. (A) FSC histogram of cells under BG11; (B) FL3 histogram of cells under BG11; (C) FL3 histogram of cells under BG11 with 2.0% (*v/v*) ethanol; (D) FL3 histogram of cells under BG11 with 0.25% (*v/v*) butanol.

**Additional file 16: Figure S10.** Genome-wide visualization of all sRNA mapping data in pSYSM of *Synechocystis*. Detailed description is the same as Additional file 2: Figure S1.

**Additional file 17: Figure S11.** Genome-wide visualization of all sRNA mapping data in pSYSA of *Synechocystis*. Detailed description is the same as Additional file 2: Figure S1.

**Additional file 18: Figure S12.** Genome-wide visualization of all sRNA mapping data in pSYSG of *Synechocystis*. Detailed description is the same as Additional file 2: Figure S1.

**Additional file 19: Figure S13.** Genome-wide visualization of all sRNA mapping data in pSYSX of *Synechocystis*. Detailed description is the same as Additional file 2: Figure S1.

**Additional file 20: Table S7.** Functional enrichment analysis of the predicted gene targets of *trans*-encoded sRNAs.

**Additional file 21: Figure S14.** Schematic diagram of two-step RT-PCR for sRNA orienting.

**Additional file 22: Figure S15.** Schematic diagram for construction of sRNA overexpression and suppression strains.

## Abbreviations

BLAST: basic local alignment search tool
CA: correspondence analysis
cDNA: complementary DNA
FACS: fluorescence-activated cell sorting
FSC: forward scatter
IRS: interspersed repeat sequence
IS: insert sequence
KEGG: Kyoto Encyclopedia of Genes and Genomes
Nr: non-redundant protein sequence database
ORF: open reading frame
qRT-PCR: quantitative real time polymerase chain reaction
RBS: ribosome binding site
RIN: RNA integrity number
RNA-seq: RNA sequencing
small ncRNA: small non-coding RNA
sRNA: small RNA
sRNA-seq: small RNA sequencing
UTR: untranslated region
WGCNA: weighted gene co-expression network analysis
WT: wild type
