# Supplementary material for: Systematic and functional identification of small non-coding RNAs associated with exogenous biofuel stress in cyanobacterium Synechocystis sp. PCC 6803
Source: Biotechnol Biofuels. 2017 Mar 7;10:57. doi: 10.1186/s13068-017-0743-y (PMC5341163; doi:10.1186/s13068-017-0743-y)
Supplement: Supplementary file 2 — Additional file 2: Figure S1. Genome-wide visualization of all sRNA mapping data in the chromosome genome of Synechocystis sp. PCC 6803. The figure can be divided into two regions, and the upper region can be further divided into three parts corresponding to samples collected at 24, 48 and 72 h. Each part provides detailed visualization of sRNA mapping depth for each nucleotide. Each sample is distinguished using a different color. The lower part can be further divided into ten parts, and each part shows detailed sRNA and gene information. From up to down: (1) all identified sRNAs in this study, (2, 3) all identified sRNAs in the positive and negative chains of Study A—Mitschkea et al. [9], (4, 5) All identified sRNAs in the positive and negative chains of Study B—Kopf et al. [10], (6, 7) all identified sRNAs in the positive and negative chains of Study C—Xu et al. [11], (8) Gene name and annotation in the NCBI database of Synechocystis PCC 6803, (9) Open reading frame prediction of Synechocystis sp. PCC 6803, (10) Characteristic sequences identified in Synechocystis sp. PCC 6803 genome, including genome repeat regions, insert sequences, ribosomal binding sites and Rho-independent transcription terminators. [file 13068_2017_743_MOESM2_ESM.pdf]

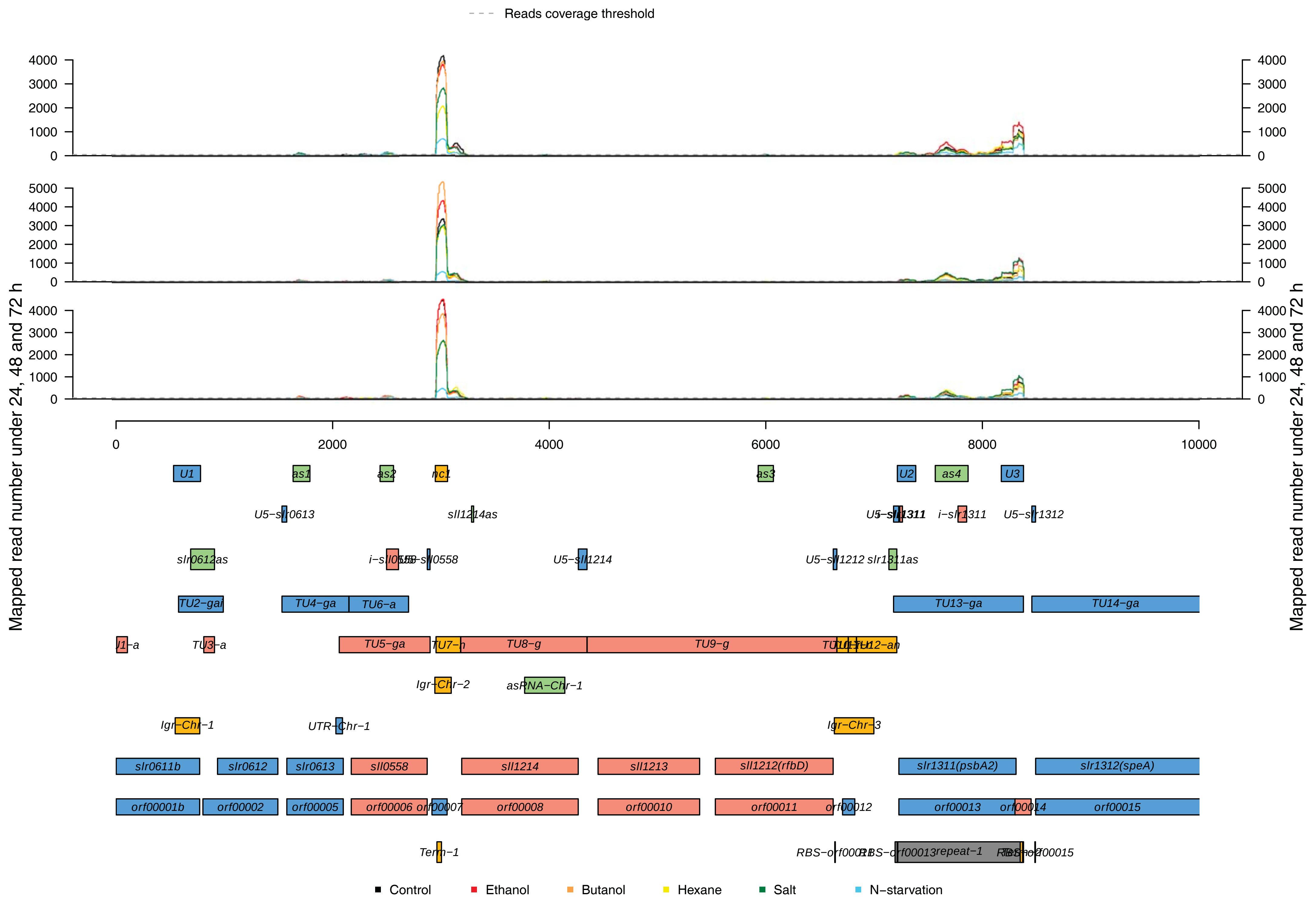

Reads coverage threshold
Mapped read number under 24, 48 and 72 h
U1
as1
as2
nc1
as3
U2
as4
U3
U5−slr0613
sll1214as
i−slr1311
U5−slr1312
slr0612as
U5−sll1214
U5−sll1212
slr1311as
TU2−gai
TU4−ga
TU6−a
TU13−ga
TU14−ga
TU3−a
TU5−ga
TU8−g
TU9−g
Igr−Chr−2
asRNA−Chr−1
Igr−Chr−1
UTR−Chr−1
Igr−Chr−3
slr0611b
slr0612
slr0613
sll0558
sll1214
sll1213
sll1212(rfbD)
slr1311(psbA2)
slr1312(speA)
orf00001b
orf00002
orf00005
orf00006
orf00008
orf00010
orf00011
orf00013
orf00015
Term−1
repeat−1
Control
Ethanol
Butanol
Hexane
Salt
N−starvation

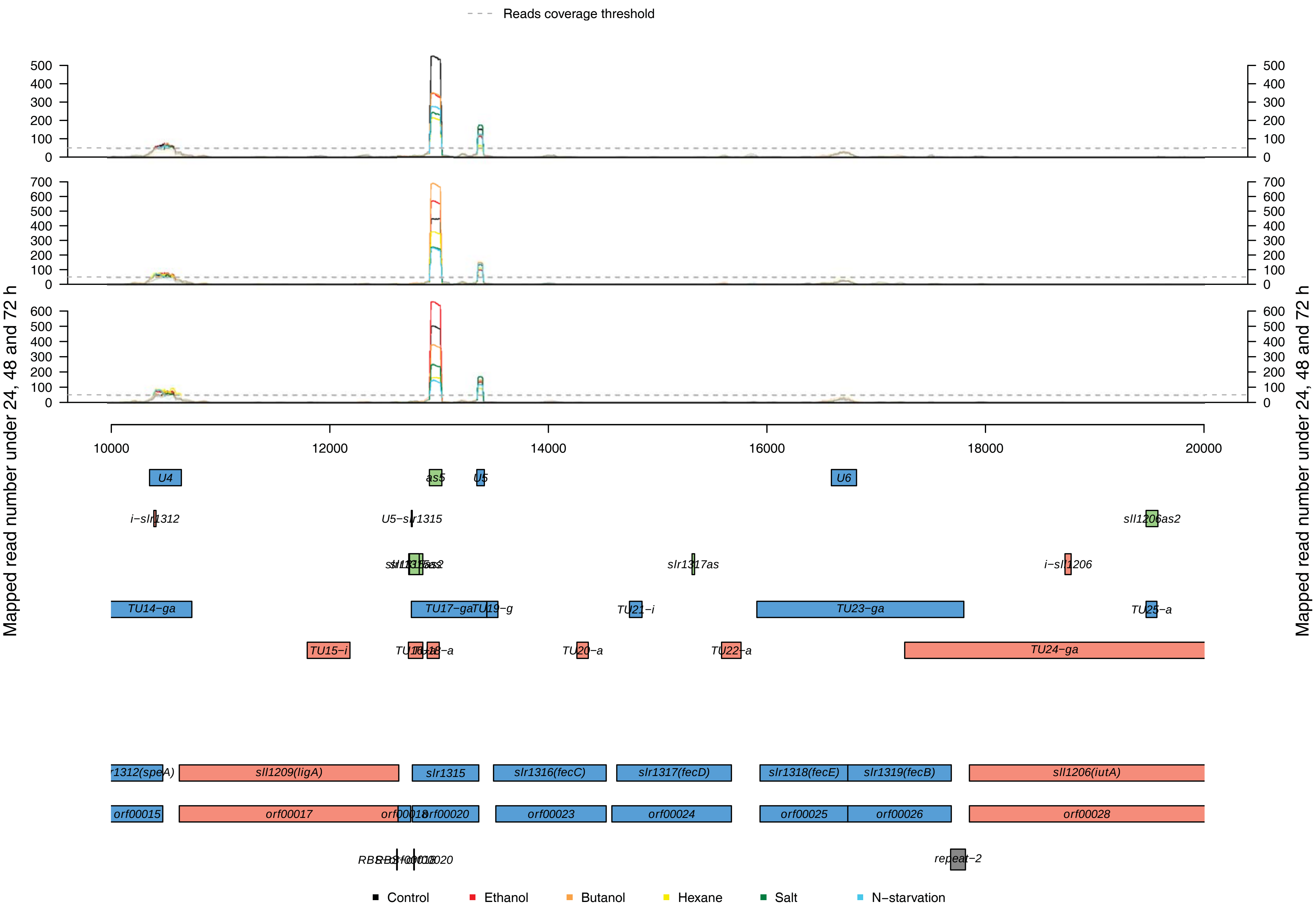

Reads coverage threshold
Mapped read number under 24, 48 and 72 h
500
400
300
200
100
0
700
600
500
400
300
200
100
0
600
500
400
300
200
100
0
10000
12000
14000
16000
18000
20000
U4
as5
U5
U6
i−slr1312
U5−slr1315
sll1206as2
slr1317as
i−sll1206
TU14−ga
TU17−ga
TU19−g
TU21−i
TU23−ga
TU25−a
TU15−i
TU20−a
TU22−a
TU24−ga
sll1209(ligA)
slr1315
slr1316(fecC)
slr1317(fecD)
slr1318(fecE)
slr1319(fecB)
sll1206(iutA)
orf00015
orf00017
orf00020
orf00023
orf00024
orf00025
orf00026
orf00028
repeat−2
Control
Ethanol
Butanol
Hexane
Salt
N−starvation

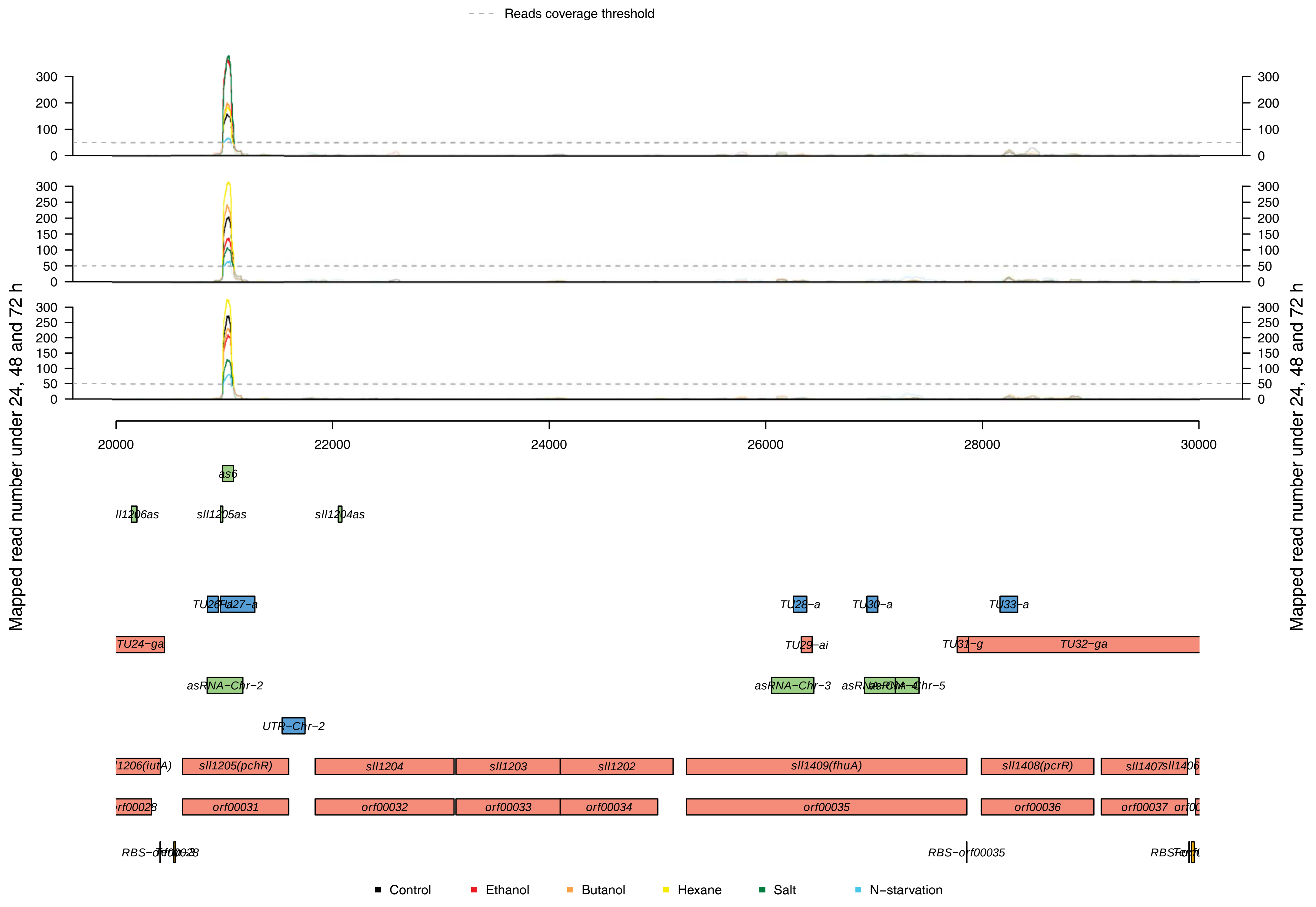

Reads coverage threshold
Mapped read number under 24, 48 and 72 h
20000
22000
24000
26000
28000
30000
as6
sll1205as
sll1204as
TU28−a
TU30−a
TU33−a
TU24−ga
TU29−ai
TU32−ga
asRNA−Chr−2
asRNA−Chr−3
UTR−Chr−2
sll1205(pchR)
sll1204
sll1203
sll1202
sll1409(fhuA)
sll1408(pcrR)
orf00031
orf00032
orf00033
orf00034
orf00035
orf00036
RBS−orf00035
Control
Ethanol
Butanol
Hexane
Salt
N−starvation

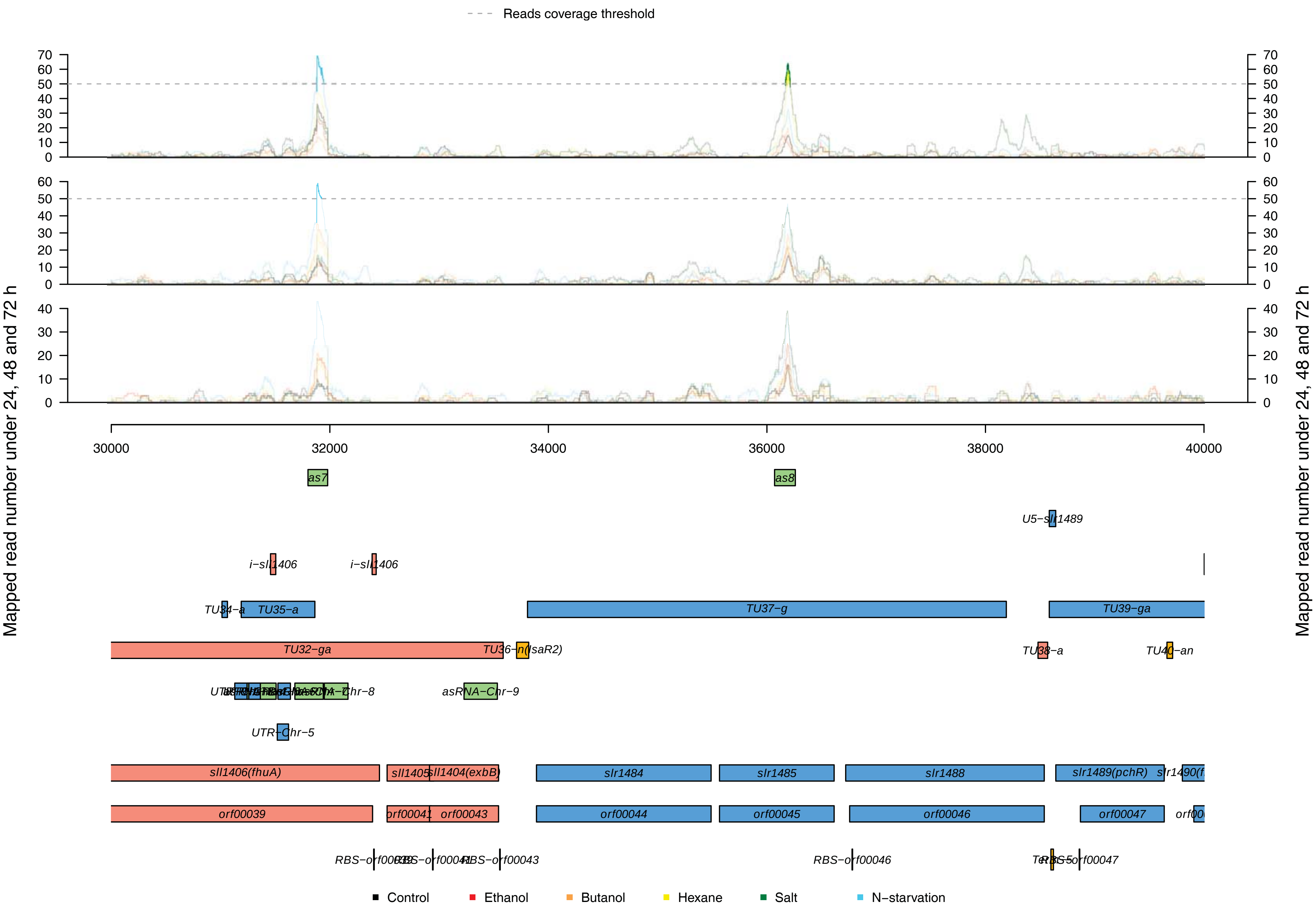

Reads coverage threshold
Mapped read number under 24, 48 and 72 h
30000
32000
34000
36000
38000
40000
as7
as8
U5−slr1489
i−sll1406
i−sll1406
TU34−a
TU35−a
TU37−g
TU39−ga
TU32−ga
TU36−n(IsaR2)
TU38−a
TU40−an
asRNA−Chr−9
UTR−Chr−5
sll1406(fhuA)
sll1405
sll1404(exbB)
slr1484
slr1485
slr1488
slr1489(pchR)
orf00039
orf00041
orf00043
orf00044
orf00045
orf00046
orf00047
RBS−orf00046
Control
Ethanol
Butanol
Hexane
Salt
N−starvation

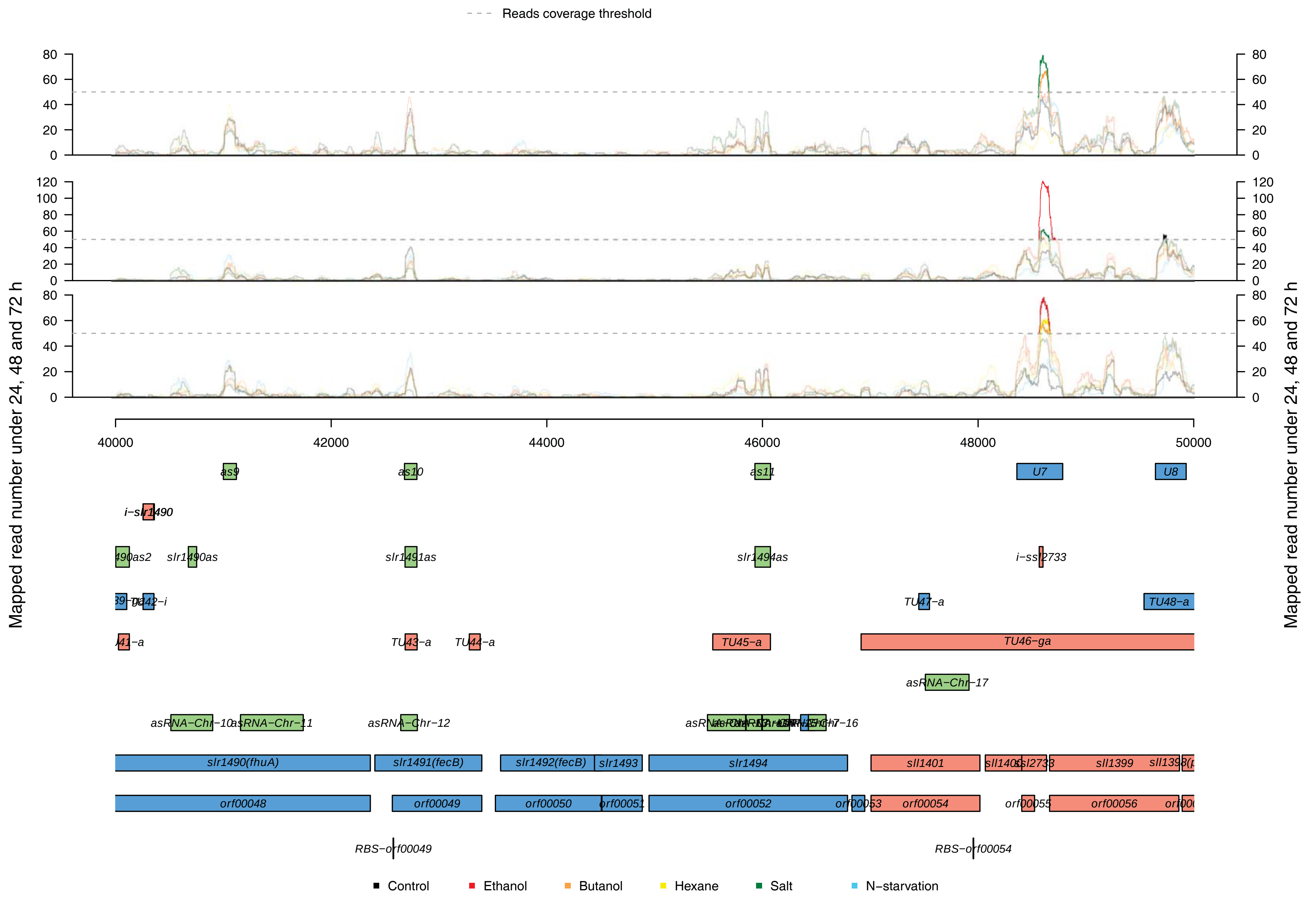

Reads coverage threshold
Mapped read number under 24, 48 and 72 h
40000
42000
44000
46000
48000
50000
as9
as10
as11
U7
U8
i−slr1490
slr1490as
slr1491as
slr1494as
i−ssl2733
TU47−a
TU48−a
TU43−a
TU44−a
TU45−a
TU46−ga
asRNA−Chr−17
asRNA−Chr−10
asRNA−Chr−11
asRNA−Chr−12
slr1490(fhuA)
slr1491(fecB)
slr1492(fecB)
slr1493
slr1494
sll1401
sll1399
orf00048
orf00049
orf00050
orf00052
orf00054
orf00056
RBS−orf00049
RBS−orf00054
Control
Ethanol
Butanol
Hexane
Salt
N−starvation

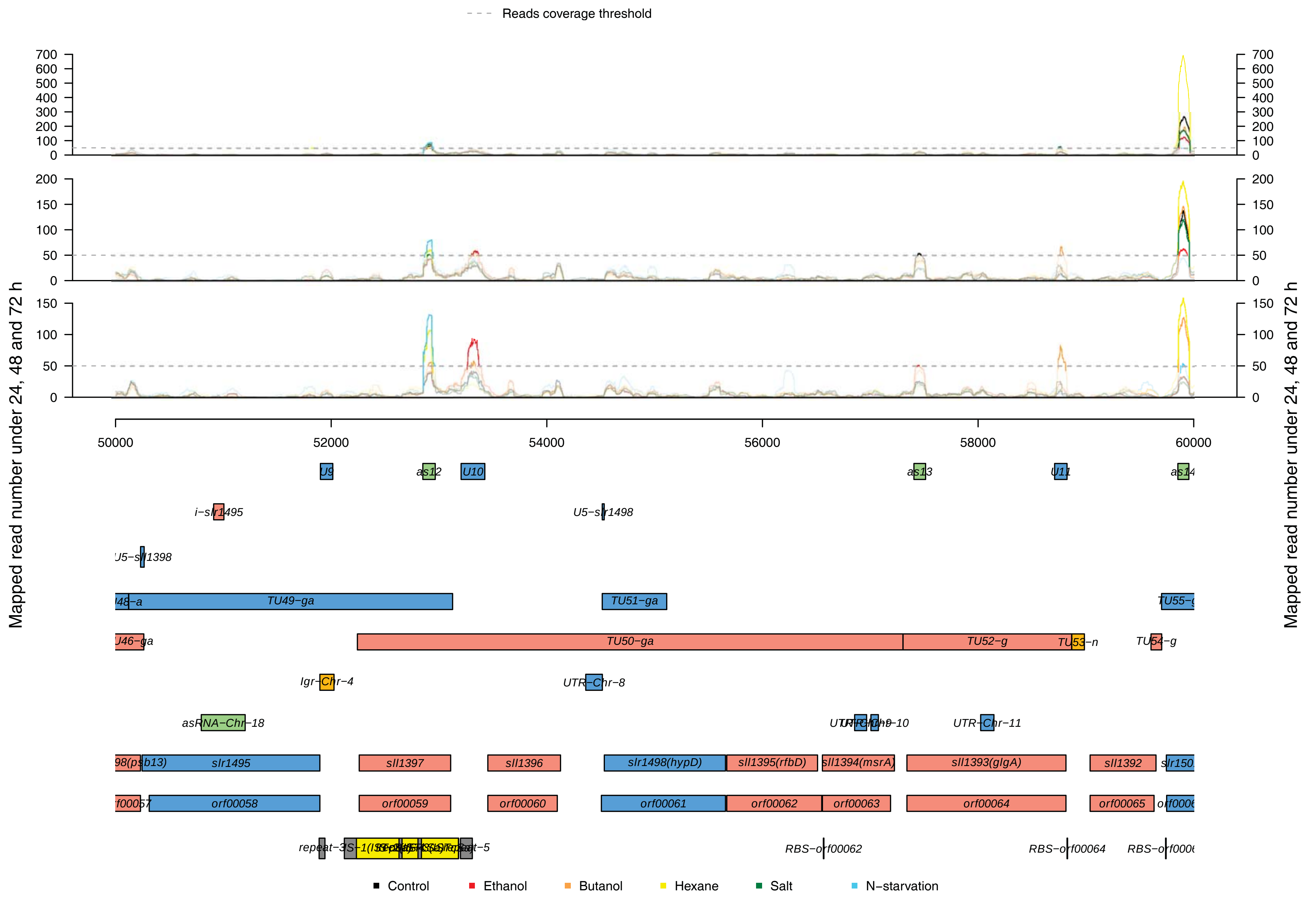

Reads coverage threshold
Mapped read number under 24, 48 and 72 h
50000
52000
54000
56000
58000
60000
TU49–ga
TU50–ga
TU51–ga
TU52–g
UTR–Chr–8
UTR–Chr–11
asRNA–Chr–18
Igr–Chr–4
slr1495
sll1397
sll1396
slr1498(hypD)
sll1395(rfbD)
sll1394(msrA)
sll1393(glgA)
sll1392
orf00058
orf00059
orf00060
orf00061
orf00062
orf00063
orf00064
orf00065
RBS–orf00062
RBS–orf00064
U5–slr1498
Control
Ethanol
Butanol
Hexane
Salt
N–starvation

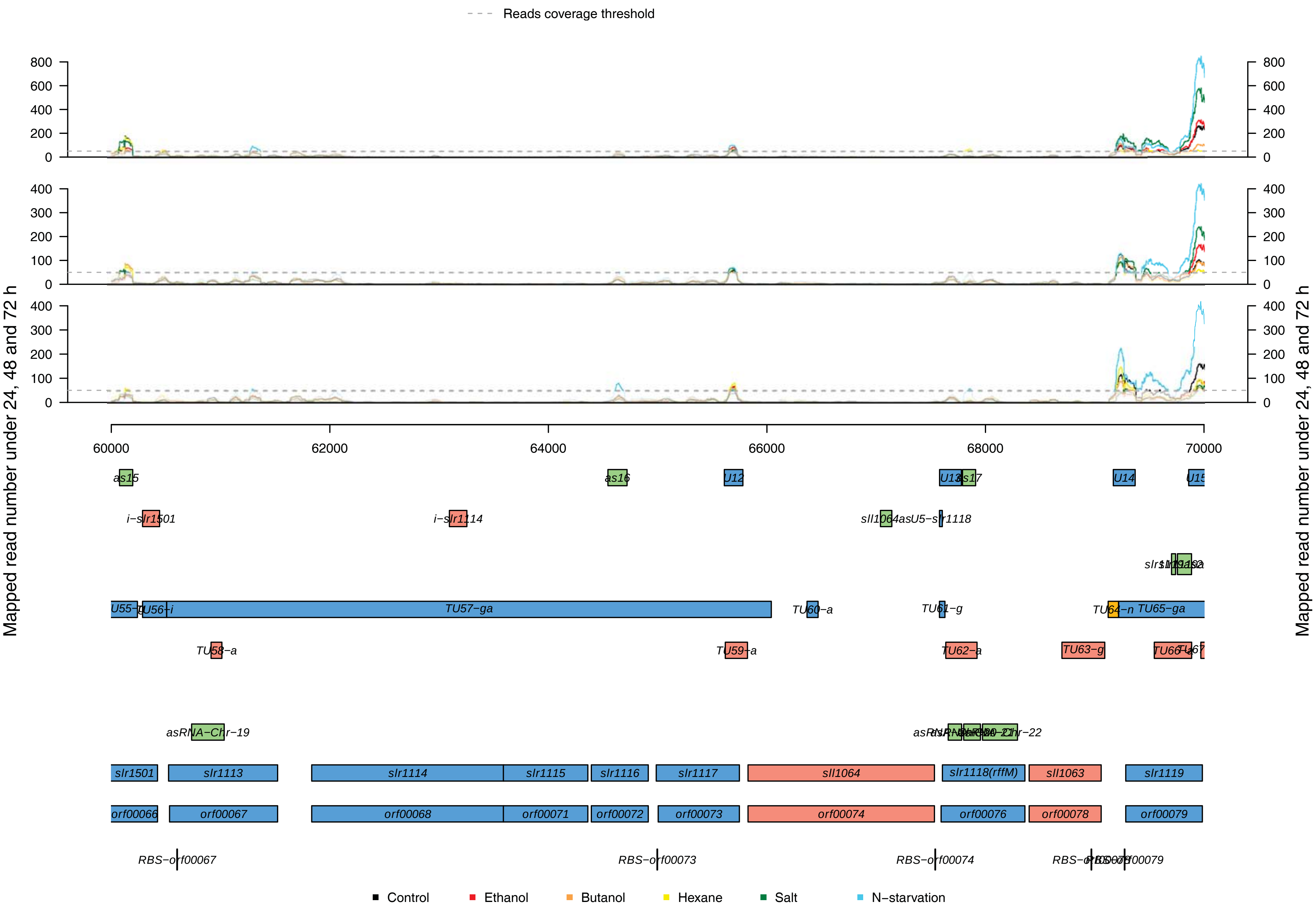

Reads coverage threshold
Mapped read number under 24, 48 and 72 h
60000
62000
64000
66000
68000
70000
as15
as16
U12
as17
U14
i−slr1501
i−slr1114
TU57−ga
TU60−a
TU61−g
TU65−ga
TU58−a
TU59−a
TU62−a
TU63−g
asRNA−Chr−19
slr1501
slr1113
slr1114
slr1115
slr1116
slr1117
sll1064
slr1118(rffM)
sll1063
slr1119
orf00066
orf00067
orf00068
orf00071
orf00072
orf00073
orf00074
orf00076
orf00078
orf00079
RBS−orf00067
RBS−orf00073
RBS−orf00074
Control
Ethanol
Butanol
Hexane
Salt
N−starvation

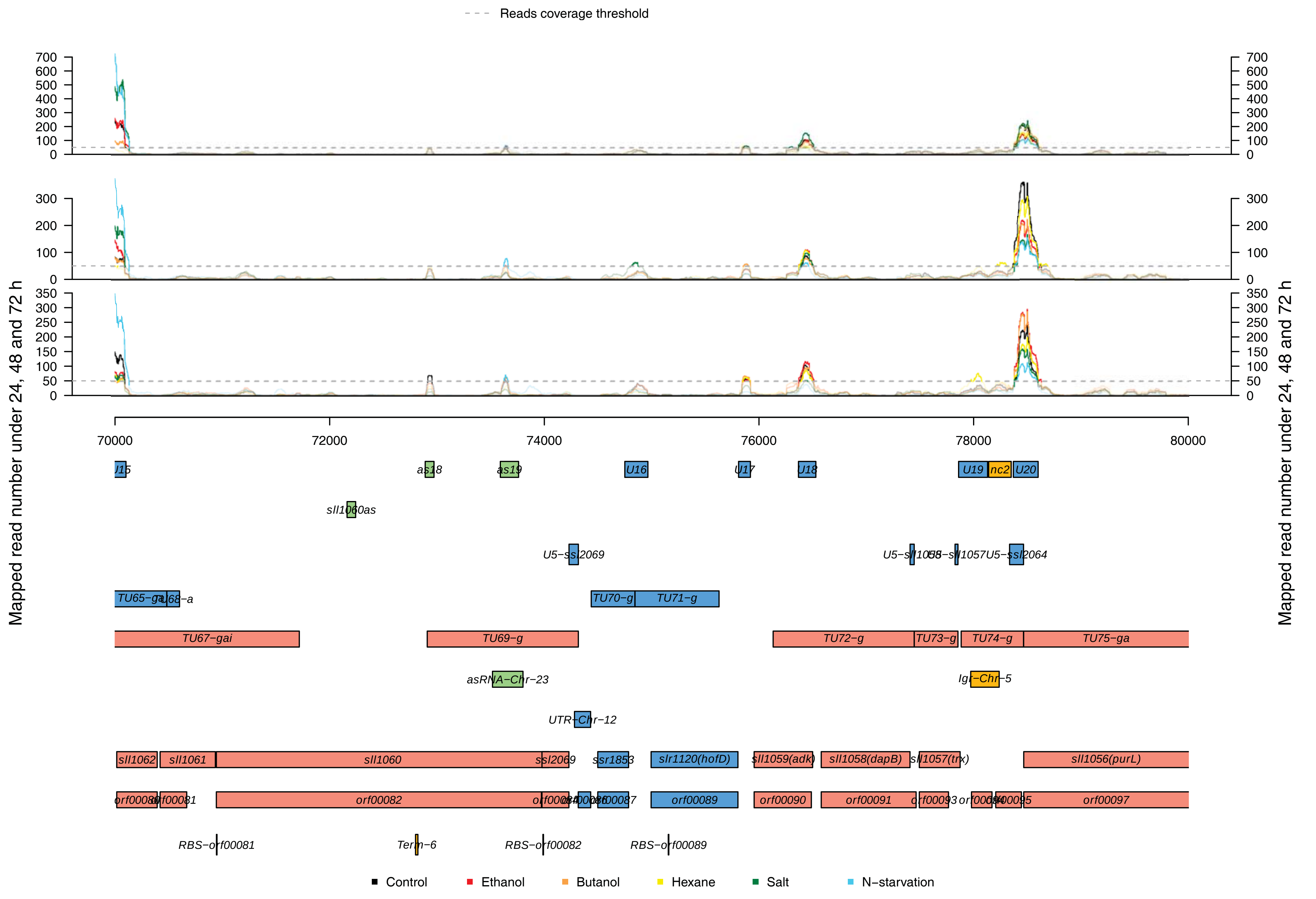

Reads coverage threshold
Mapped read number under 24, 48 and 72 h
70000
72000
74000
76000
78000
80000
U15
as18
as19
U16
U17
U18
U19
nc2
U20
sll1060as
U5−ssl2069
U5−sll1058
U5−sll1057
U5−ssl2064
TU65−ga
TU68−a
TU70−g
TU71−g
TU67−gai
TU69−g
TU72−g
TU73−g
TU74−g
TU75−ga
asRNA−Chr−23
Igr−Chr−5
UTR−Chr−12
sll1062
sll1061
sll1060
ssl2069
ssr1853
slr1120(hofD)
sll1059(adk)
sll1058(dapB)
sll1057(trx)
sll1056(purL)
orf00082
orf00089
orf00090
orf00091
orf00093
orf00095
orf00097
RBS−orf00081
Term−6
RBS−orf00082
RBS−orf00089
Control
Ethanol
Butanol
Hexane
Salt
N−starvation

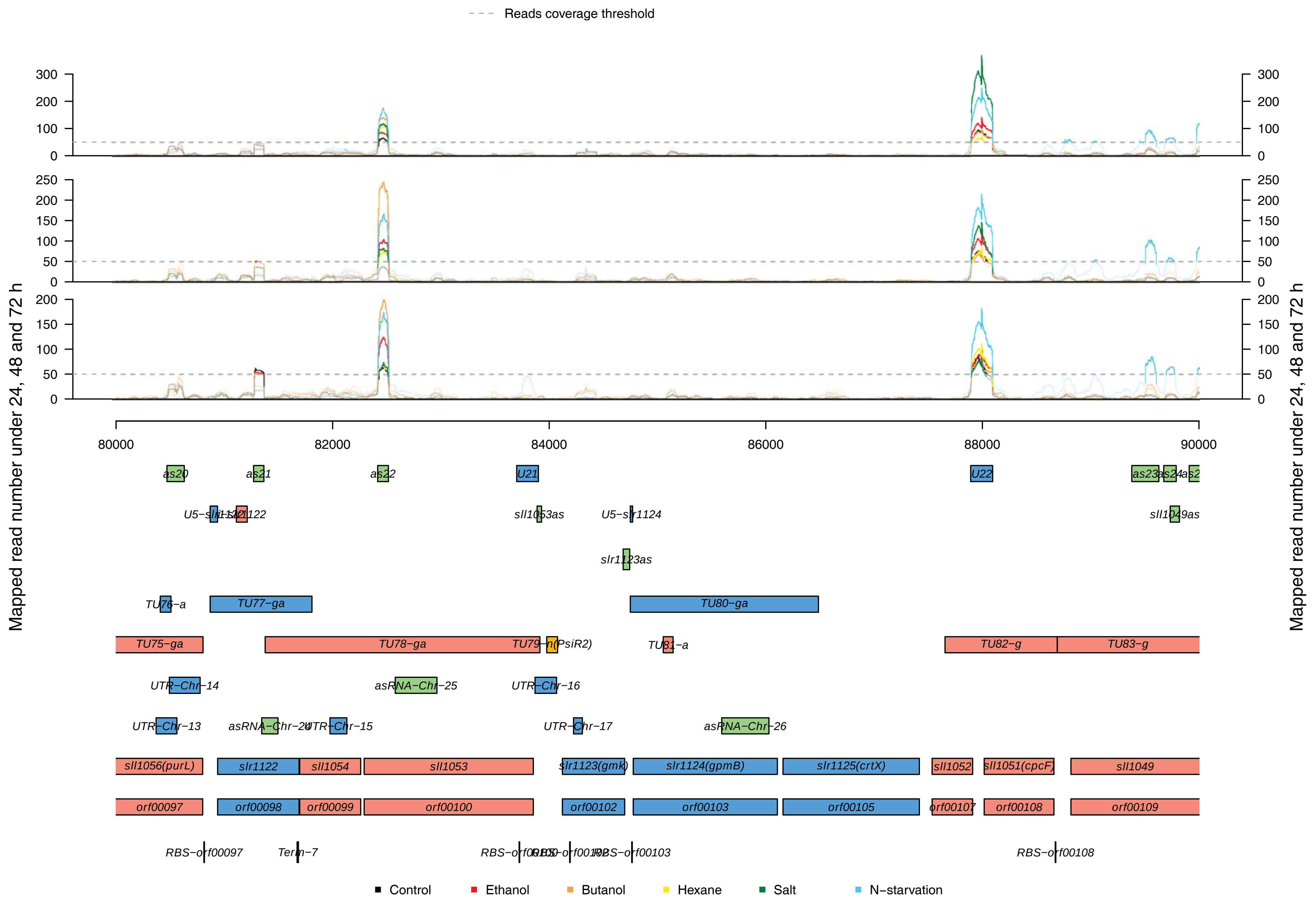

Reads coverage threshold
Mapped read number under 24, 48 and 72 h
80000
82000
84000
86000
88000
90000
as20
as21
as22
U21
U22
sll1053as
slr1123as
sll1049as
TU76-a
TU77-ga
TU80-ga
TU75-ga
TU78-ga
TU82-g
TU83-g
UTR-Chr-14
asRNA-Chr-25
UTR-Chr-16
UTR-Chr-13
UTR-Chr-17
asRNA-Chr-26
sll1056(purL)
slr1122
sll1054
sll1053
slr1123(gmk)
slr1124(gpmB)
slr1125(crtX)
sll1052
sll1051(cpcF)
sll1049
orf00097
orf00098
orf00099
orf00100
orf00102
orf00103
orf00105
orf00107
orf00108
orf00109
RBS-orf00097
Term-7
RBS-orf00108
Control
Ethanol
Butanol
Hexane
Salt
N-starvation

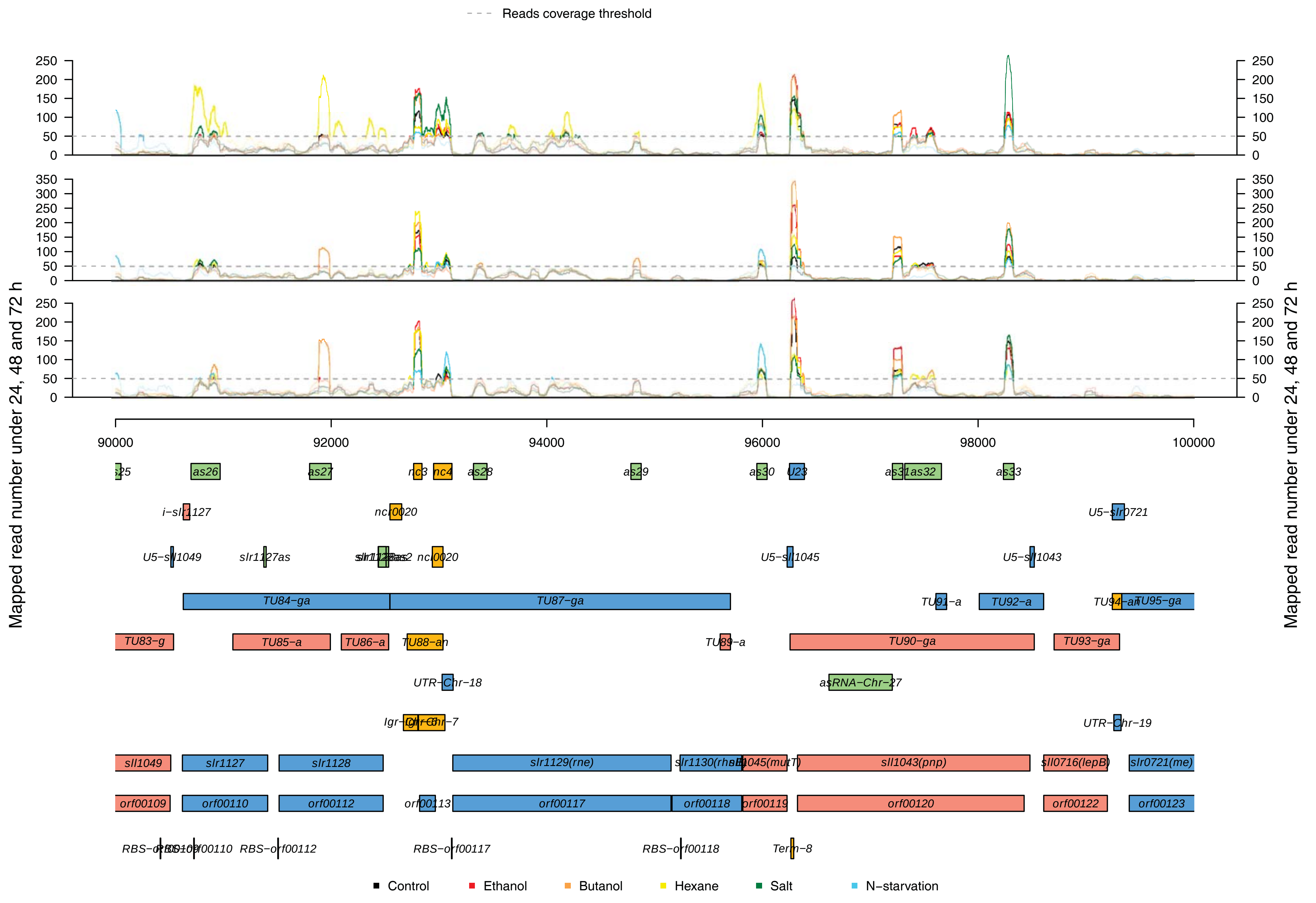

Reads coverage threshold
Mapped read number under 24, 48 and 72 h
90000
92000
94000
96000
98000
100000
as26
as27
nc3
nc4
as28
as29
as30
U23
as31
as32
as33
i−slr1127
ncl0020
U5−slr0721
U5−sll1049
slr1127as
U5−sll1045
U5−sll1043
TU84−ga
TU87−ga
TU91−a
TU92−a
TU95−ga
TU83−g
TU85−a
TU86−a
TU88−an
TU89−a
TU90−ga
TU93−ga
UTR−Chr−18
asRNA−Chr−27
UTR−Chr−19
sll1049
slr1127
slr1128
slr1129(rne)
sll1043(pnp)
sll0716(lepB)
slr0721(me)
orf00109
orf00110
orf00112
orf00113
orf00117
orf00118
orf00120
orf00122
orf00123
RBS−orf00110
RBS−orf00112
RBS−orf00117
RBS−orf00118
Term−8
Control
Ethanol
Butanol
Hexane
Salt
N−starvation

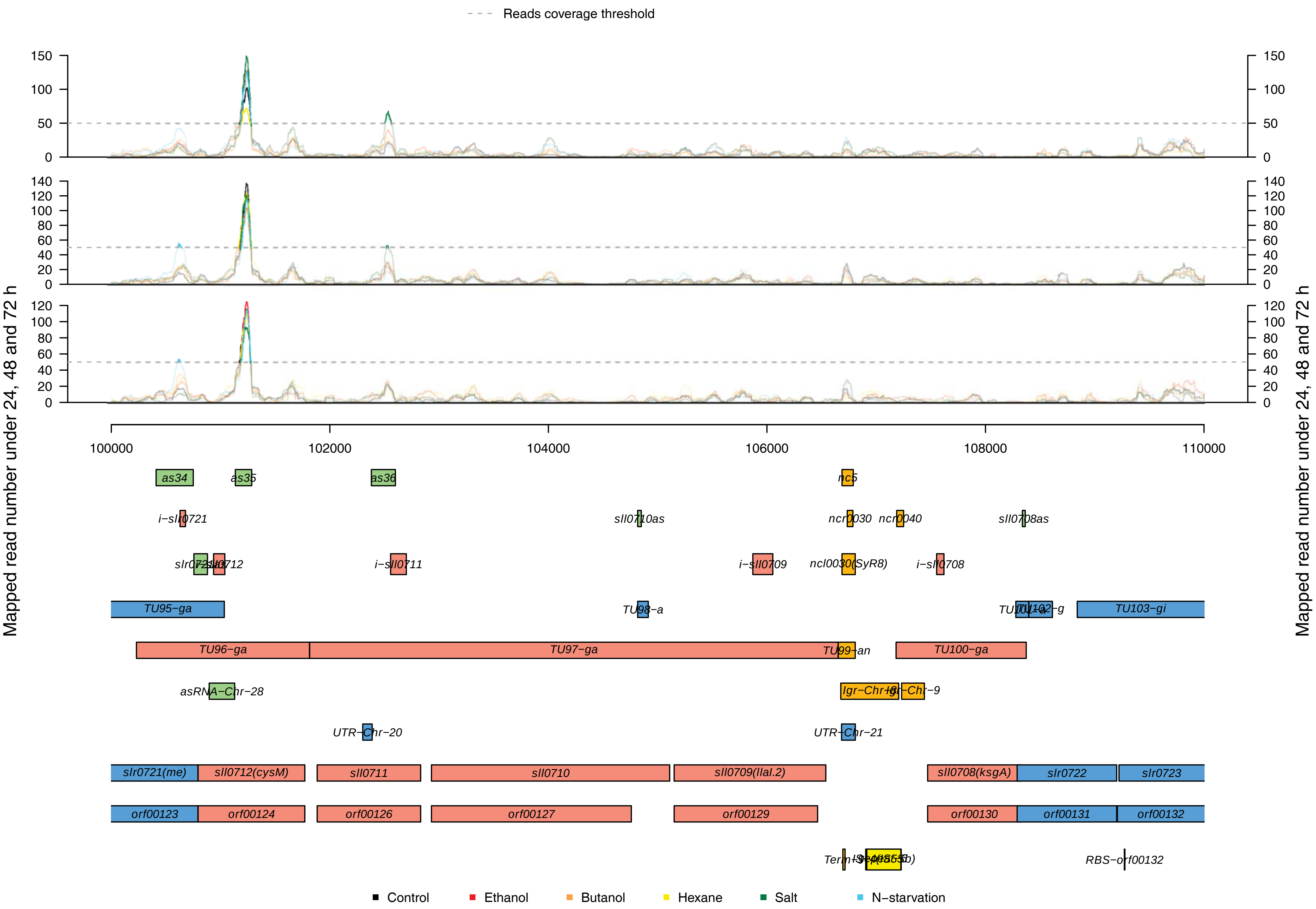

Reads coverage threshold
Mapped read number under 24, 48 and 72 h
100000
102000
104000
106000
108000
110000
as34
as35
as36
nc5
i−slr0721
sll0710as
ncr0030
ncr0040
sll0708as
i−sll0711
i−sll0709
ncl0030(SyR8)
i−sll0708
TU95−ga
TU98−a
TU103−gi
TU96−ga
TU97−ga
TU100−ga
asRNA−Chr−28
UTR−Chr−20
UTR−Chr−21
slr0721(me)
sll0712(cysM)
sll0711
sll0710
sll0709(llal.2)
sll0708(ksgA)
slr0722
slr0723
orf00123
orf00124
orf00126
orf00127
orf00129
orf00130
orf00131
orf00132
RBS−orf00132
Control
Ethanol
Butanol
Hexane
Salt
N−starvation

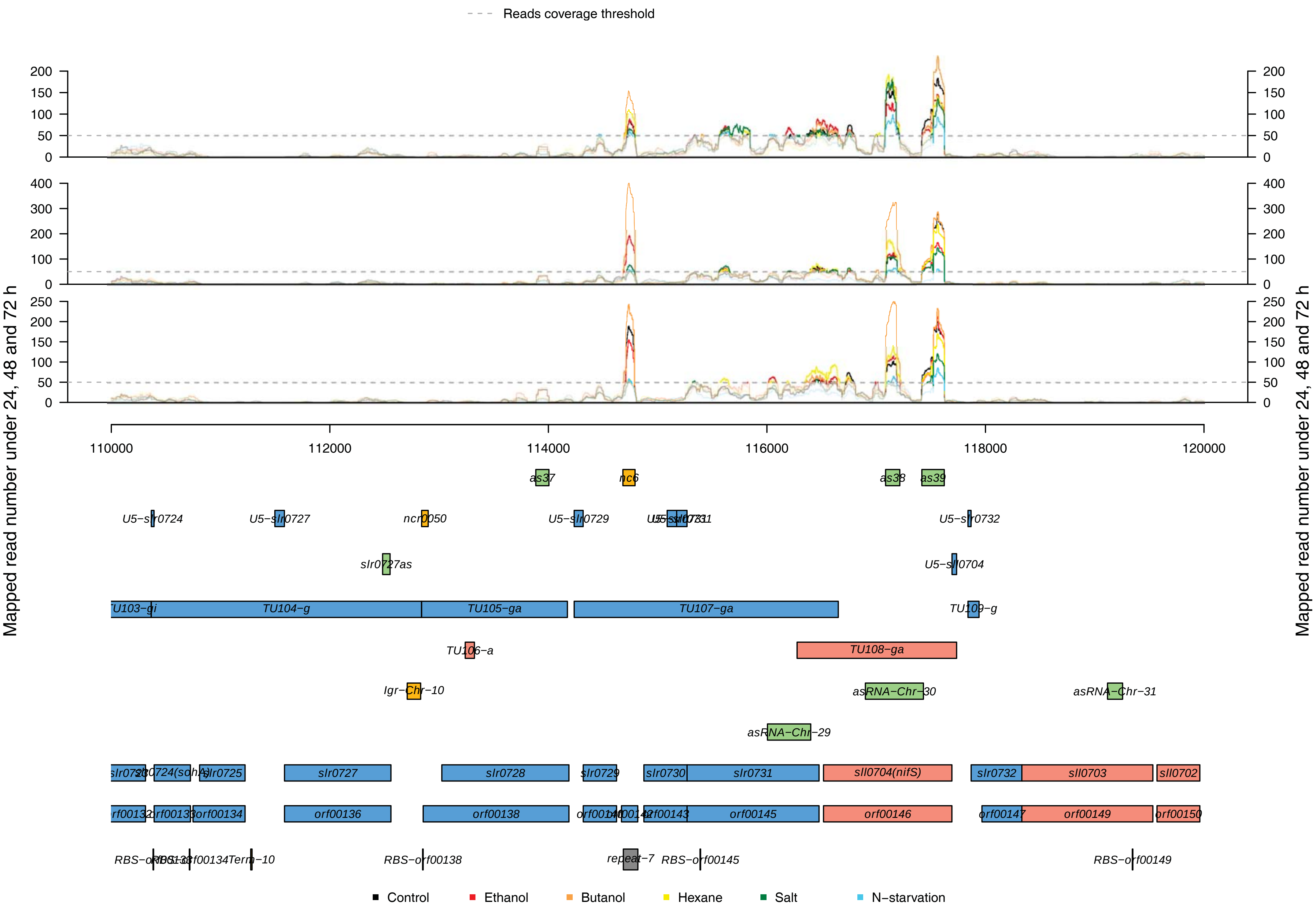
Reads coverage threshold
Mapped read number under 24, 48 and 72 h
110000
112000
114000
116000
118000
120000
as37
nc6
as38
as39
U5−slr0724
U5−slr0727
ncr0050
U5−slr0729
U5−slr0732
slr0727as
U5−sll0704
TU104−g
TU105−ga
TU107−ga
TU109−g
TU106−a
TU108−ga
Igr−Chr−10
asRNA−Chr−30
asRNA−Chr−31
asRNA−Chr−29
slr0725
slr0727
slr0728
slr0729
slr0730
slr0731
sll0704(nifS)
slr0732
sll0703
sll0702
orf00134
orf00136
orf00138
orf00145
orf00146
orf00147
orf00149
orf00150
RBS−orf00138
repeat−7
RBS−orf00145
RBS−orf00149
Control
Ethanol
Butanol
Hexane
Salt
N−starvation

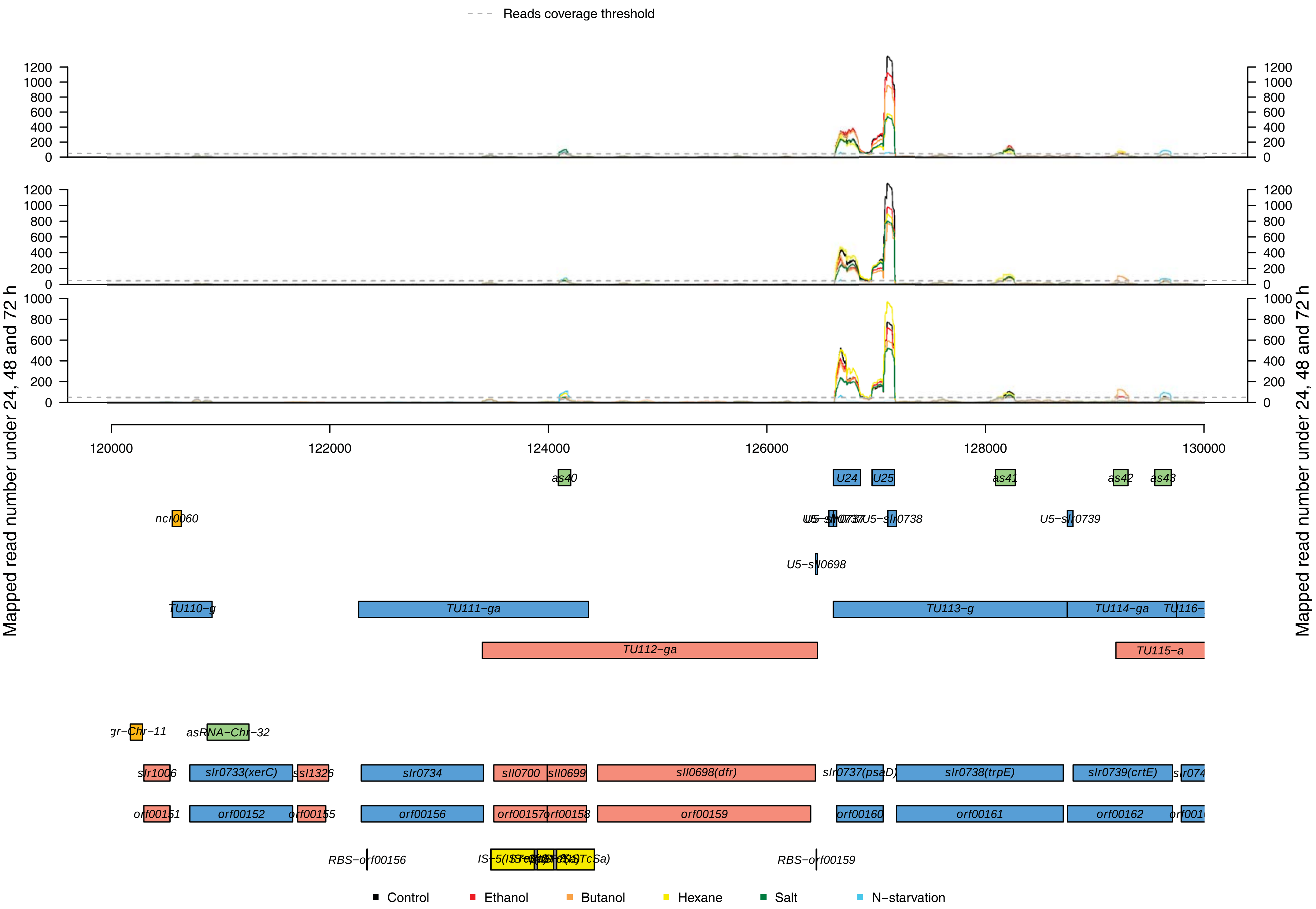

Reads coverage threshold
Mapped read number under 24, 48 and 72 h
120000
122000
124000
126000
128000
130000
as40
U24
U25
as41
as42
as43
ncr0060
U5−sll0738
U5−sll0739
U5−sll0698
TU110−g
TU111−ga
TU113−g
TU114−ga
TU112−ga
TU115−a
asRNA−Chr−32
slr1006
slr0733(xerC)
ssl1326
slr0734
sll0700
sll0699
sll0698(dfr)
slr0737(psaD)
slr0738(trpE)
slr0739(crtE)
orf00151
orf00152
orf00155
orf00156
orf00157
orf00158
orf00159
orf00160
orf00161
orf00162
RBS−orf00156
RBS−orf00159
Control
Ethanol
Butanol
Hexane
Salt
N−starvation

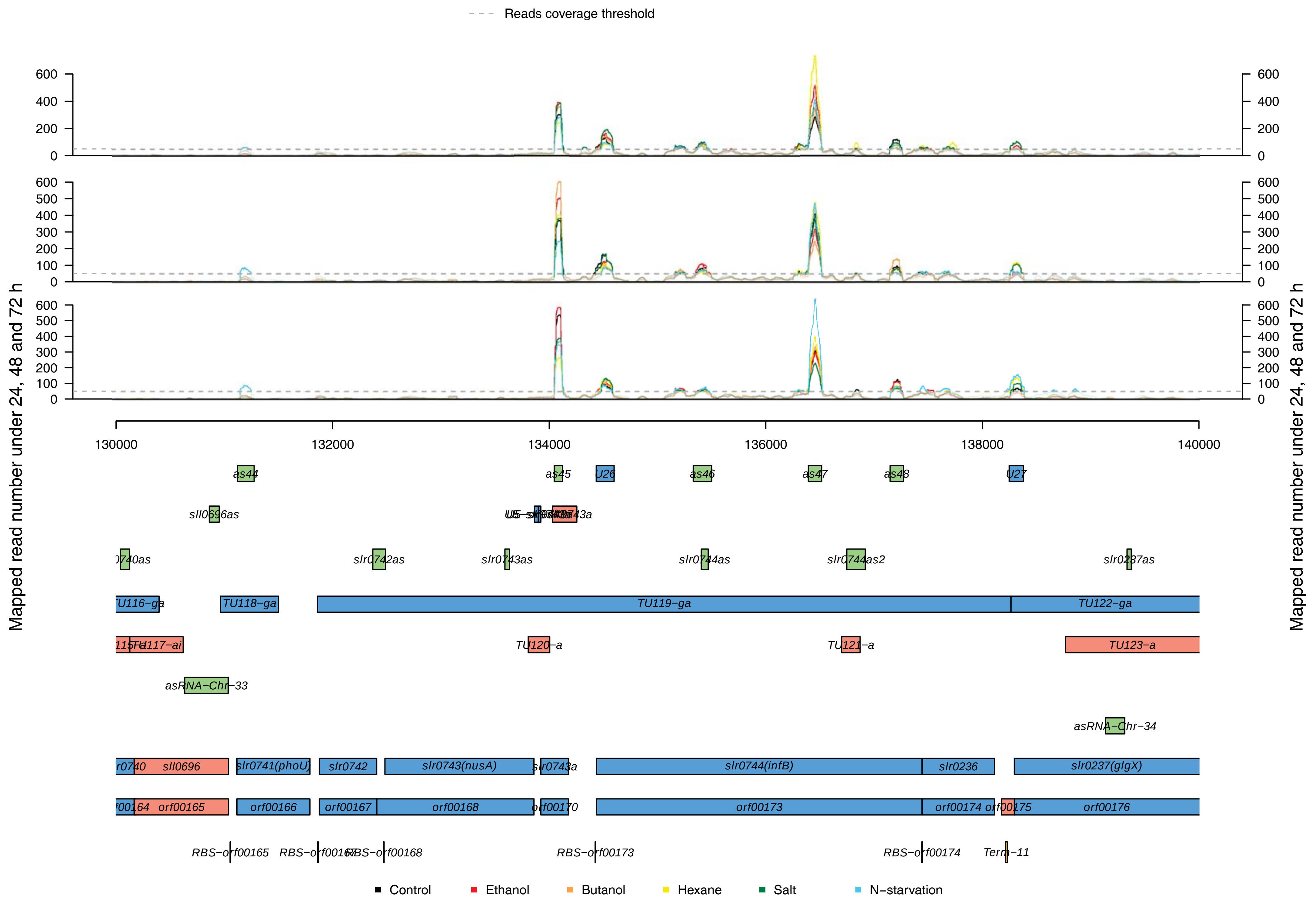

Reads coverage threshold
Mapped read number under 24, 48 and 72 h
130000
132000
134000
136000
138000
140000
as44
as45
U26
as46
as47
as48
U27
sll0696as
slr0742as
slr0743as
slr0744as
slr0744as2
slr0237as
TU118−ga
TU119−ga
TU122−ga
TU120−a
TU121−a
TU123−a
asRNA−Chr−33
asRNA−Chr−34
sll0696
slr0741(phoU)
slr0742
slr0743(nusA)
slr0744(infB)
slr0236
slr0237(glgX)
orf00165
orf00166
orf00167
orf00168
orf00173
orf00176
RBS−orf00165
RBS−orf00173
RBS−orf00174
Control
Ethanol
Butanol
Hexane
Salt
N−starvation

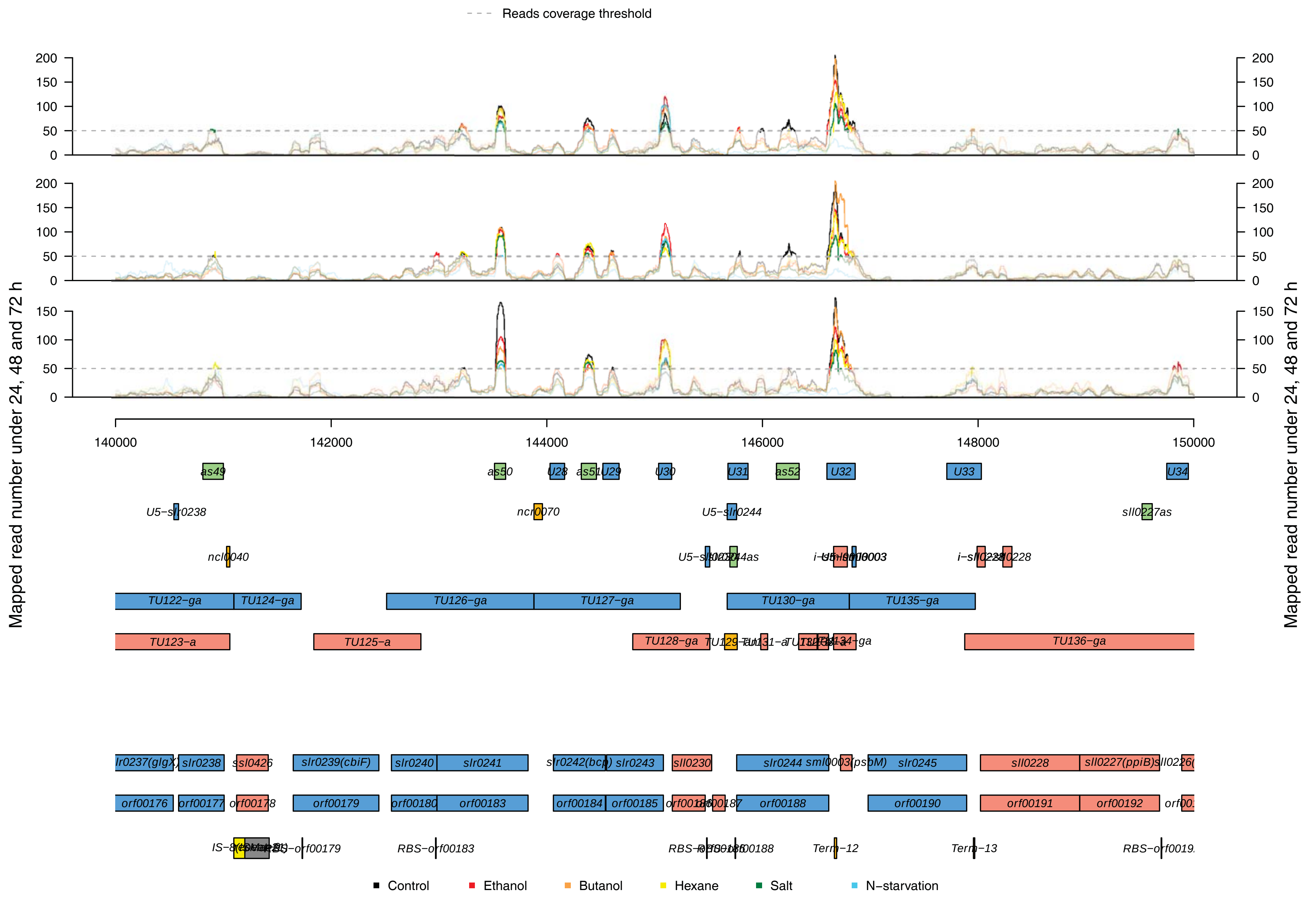

Reads coverage threshold
Mapped read number under 24, 48 and 72 h
140000
142000
144000
146000
148000
150000
Control
Ethanol
Butanol
Hexane
Salt
N−starvation

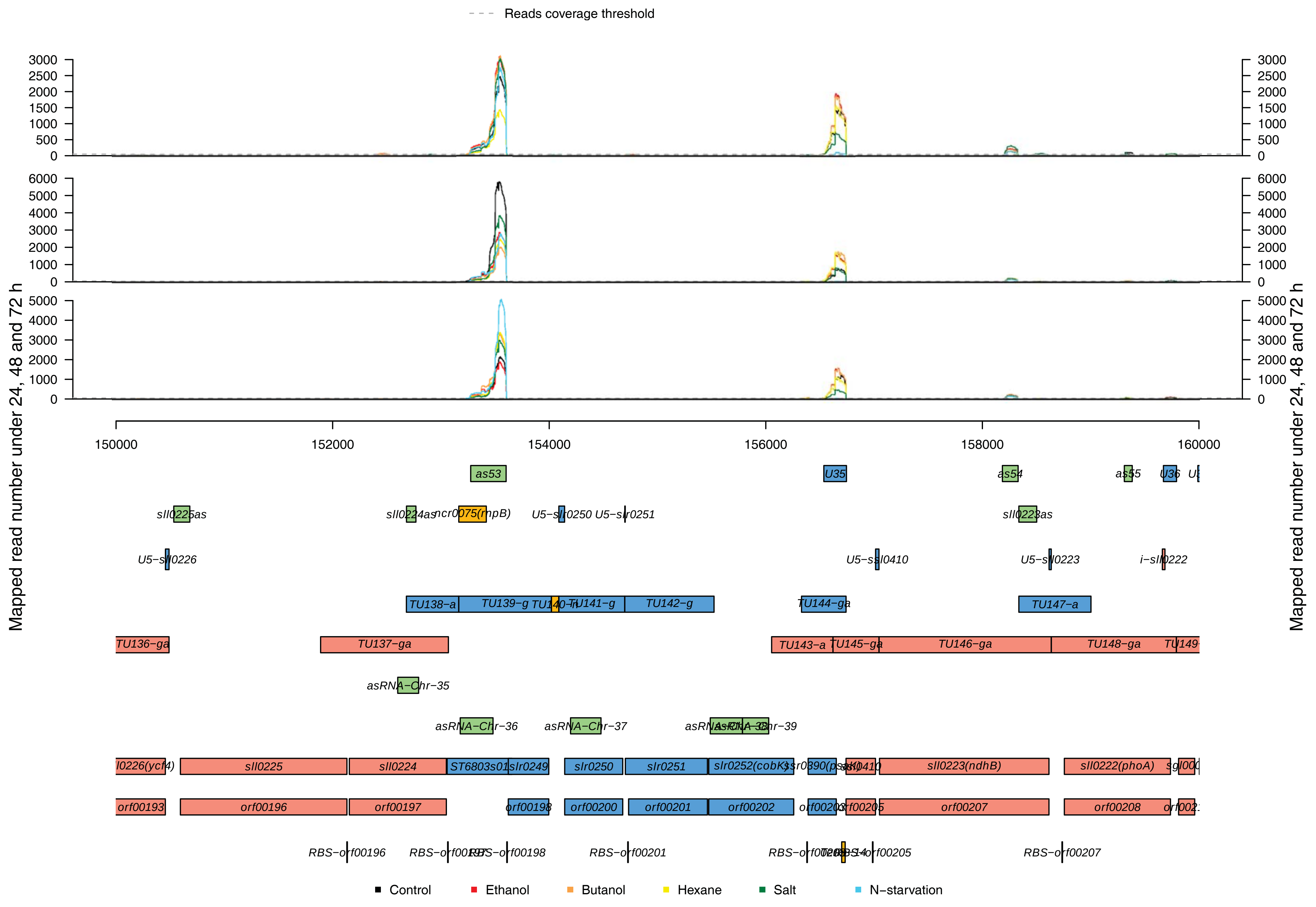

Reads coverage threshold
Mapped read number under 24, 48 and 72 h
150000
152000
154000
156000
158000
160000
as53
U35
as54
as55
U36
sll0225as
sll0224as
ncr0075(rnpB)
sll0223as
U5−sll0226
U5−ssl0410
U5−sll0223
i−sll0222
TU138−a
TU139−g
TU141−g
TU142−g
TU144−ga
TU147−a
TU136−ga
TU137−ga
TU143−a
TU145−ga
TU146−ga
TU148−ga
asRNA−Chr−35
asRNA−Chr−36
asRNA−Chr−37
sll0225
sll0224
slr0249
slr0250
slr0251
sll0223(ndhB)
sll0222(phoA)
orf00193
orf00196
orf00197
orf00198
orf00200
orf00201
orf00202
orf00207
orf00208
RBS−orf00196
RBS−orf00198
RBS−orf00201
RBS−orf00205
RBS−orf00207
Control
Ethanol
Butanol
Hexane
Salt
N−starvation

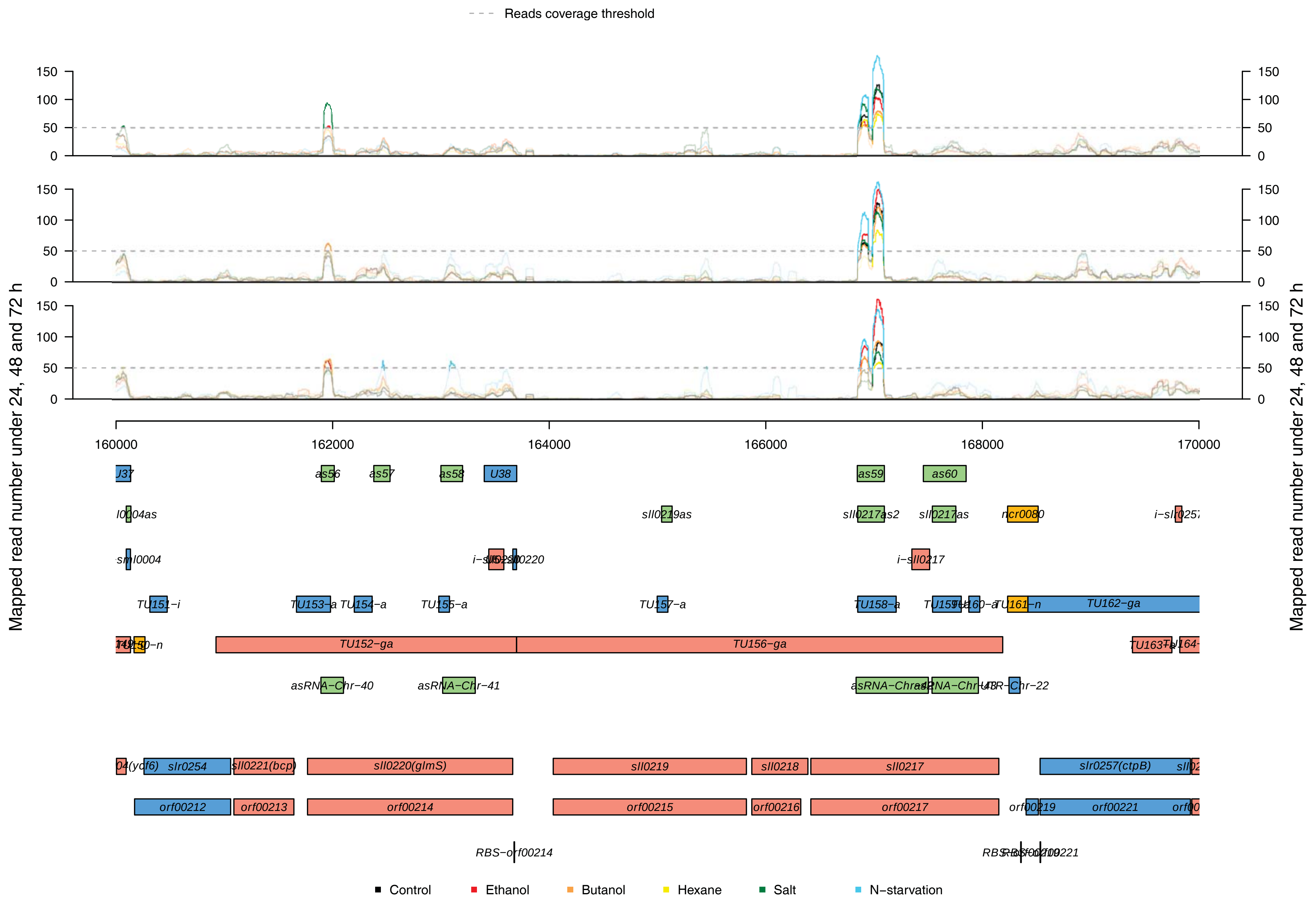

Reads coverage threshold
Mapped read number under 24, 48 and 72 h
160000
162000
164000
166000
168000
170000
as56
as57
as58
U38
as59
as60
sll0219as
sll0217as2
sll0217as
ncr0080
TU151−i
TU153−a
TU154−a
TU155−a
TU157−a
TU158−a
TU161−n
TU162−ga
TU152−ga
TU156−ga
asRNA−Chr−40
asRNA−Chr−41
slr0254
sll0221(bcp)
sll0220(glmS)
sll0219
sll0218
sll0217
slr0257(ctpB)
orf00212
orf00213
orf00214
orf00215
orf00216
orf00217
orf00221
RBS−orf00214
Control
Ethanol
Butanol
Hexane
Salt
N−starvation

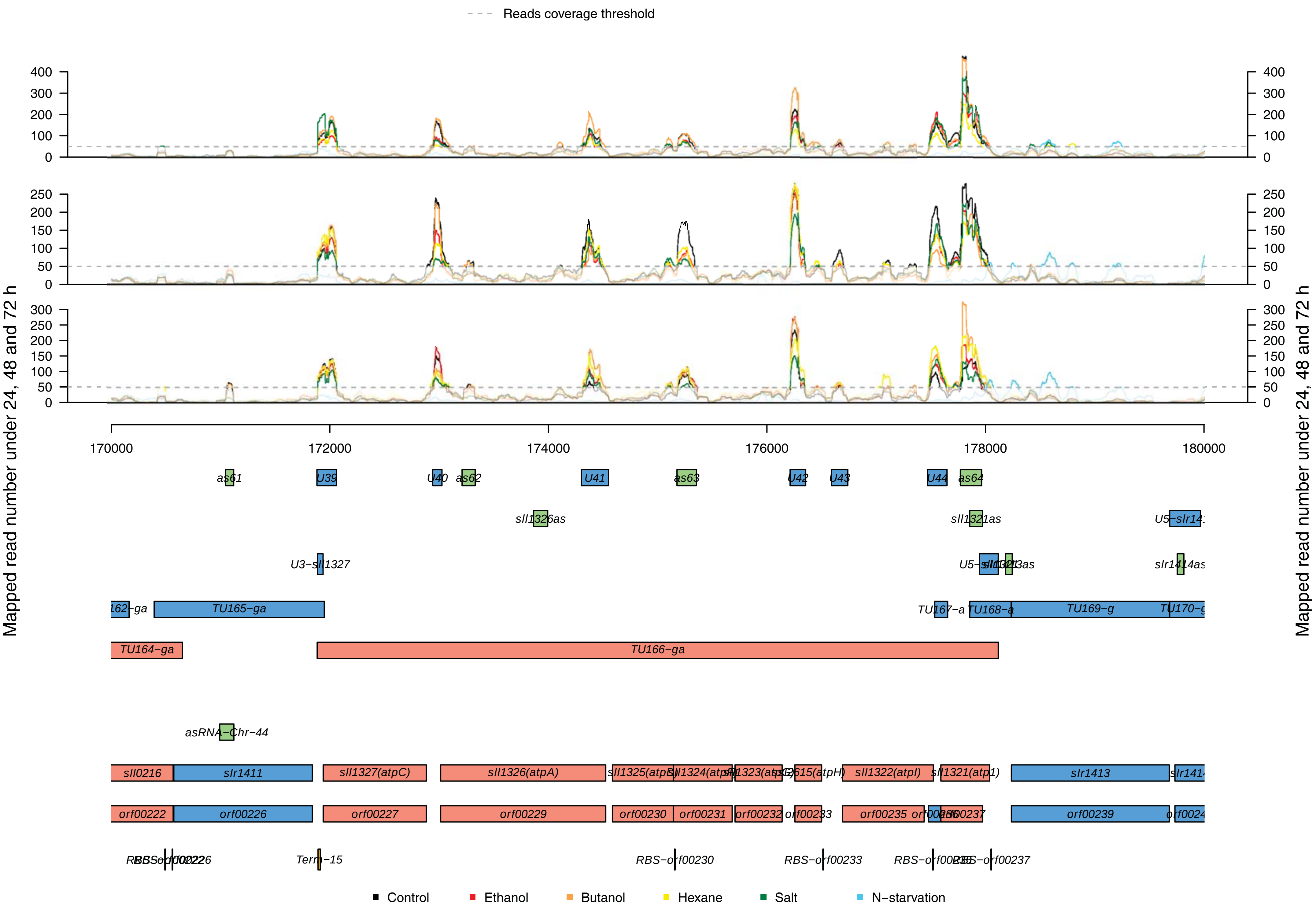

Reads coverage threshold
Mapped read number under 24, 48 and 72 h
170000
172000
174000
176000
178000
180000
as61
U39
U40
as62
U41
as63
U42
U43
U44
as64
sll1326as
sll1321as
U3−sll1327
TU165−ga
TU164−ga
TU166−ga
TU167−a
TU168−a
TU169−g
asRNA−Chr−44
sll0216
slr1411
sll1327(atpC)
sll1326(atpA)
sll1322(atpI)
slr1413
orf00222
orf00226
orf00227
orf00229
orf00230
orf00231
orf00232
orf00235
orf00239
Term−15
RBS−orf00230
RBS−orf00233
RBS−orf00237
Control
Ethanol
Butanol
Hexane
Salt
N−starvation

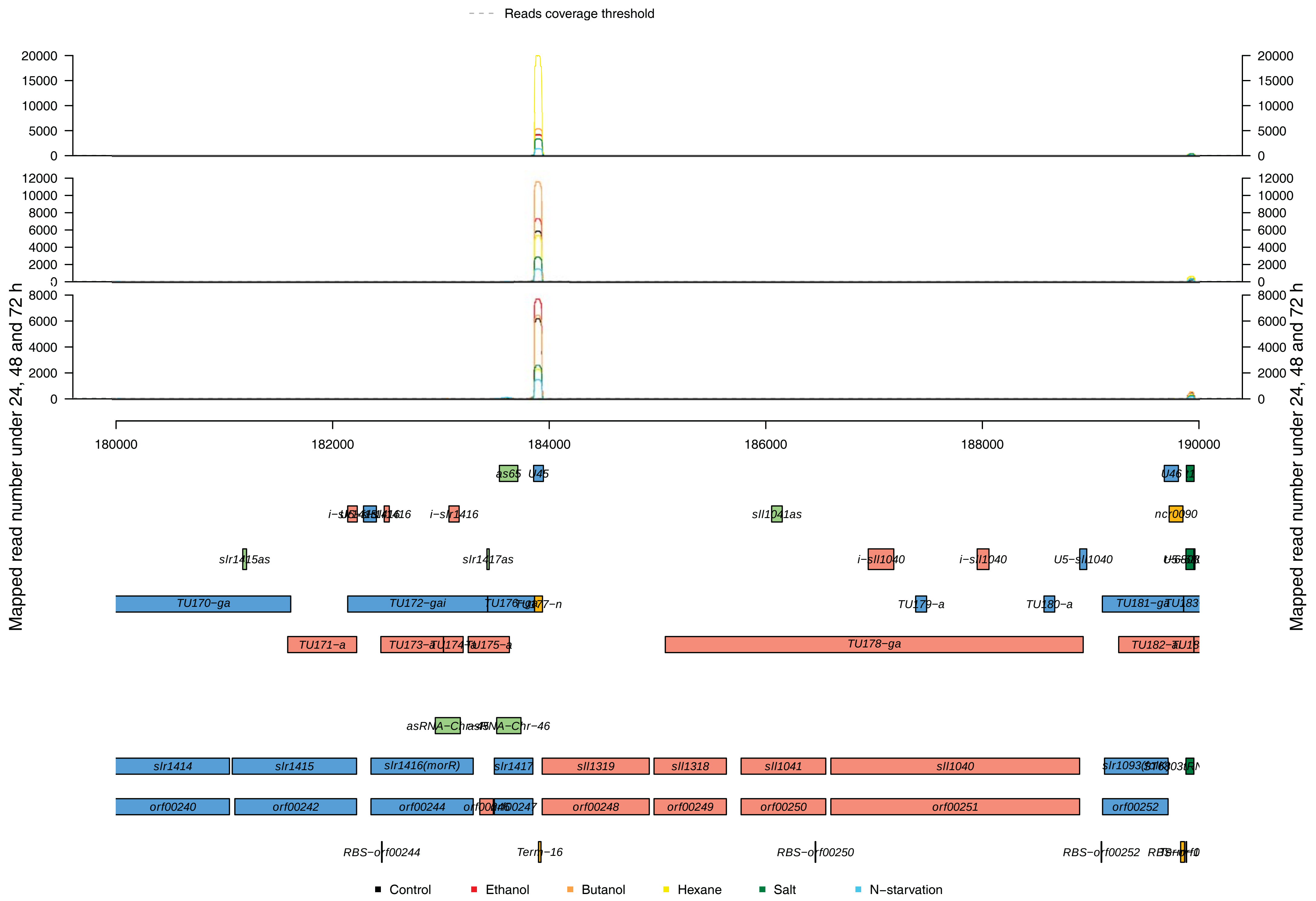

Reads coverage threshold
Mapped read number under 24, 48 and 72 h
180000
182000
184000
186000
188000
190000
as65
sll1041as
slr1415as
slr1417as
i−sll1040
TU170−ga
TU172−gai
TU179−a
TU180−a
TU171−a
TU178−ga
slr1414
slr1415
slr1416(morR)
slr1417
sll1319
sll1318
sll1041
sll1040
orf00240
orf00242
orf00244
orf00248
orf00249
orf00250
orf00251
orf00252
RBS−orf00244
Term−16
RBS−orf00250
RBS−orf00252
Control
Ethanol
Butanol
Hexane
Salt
N−starvation

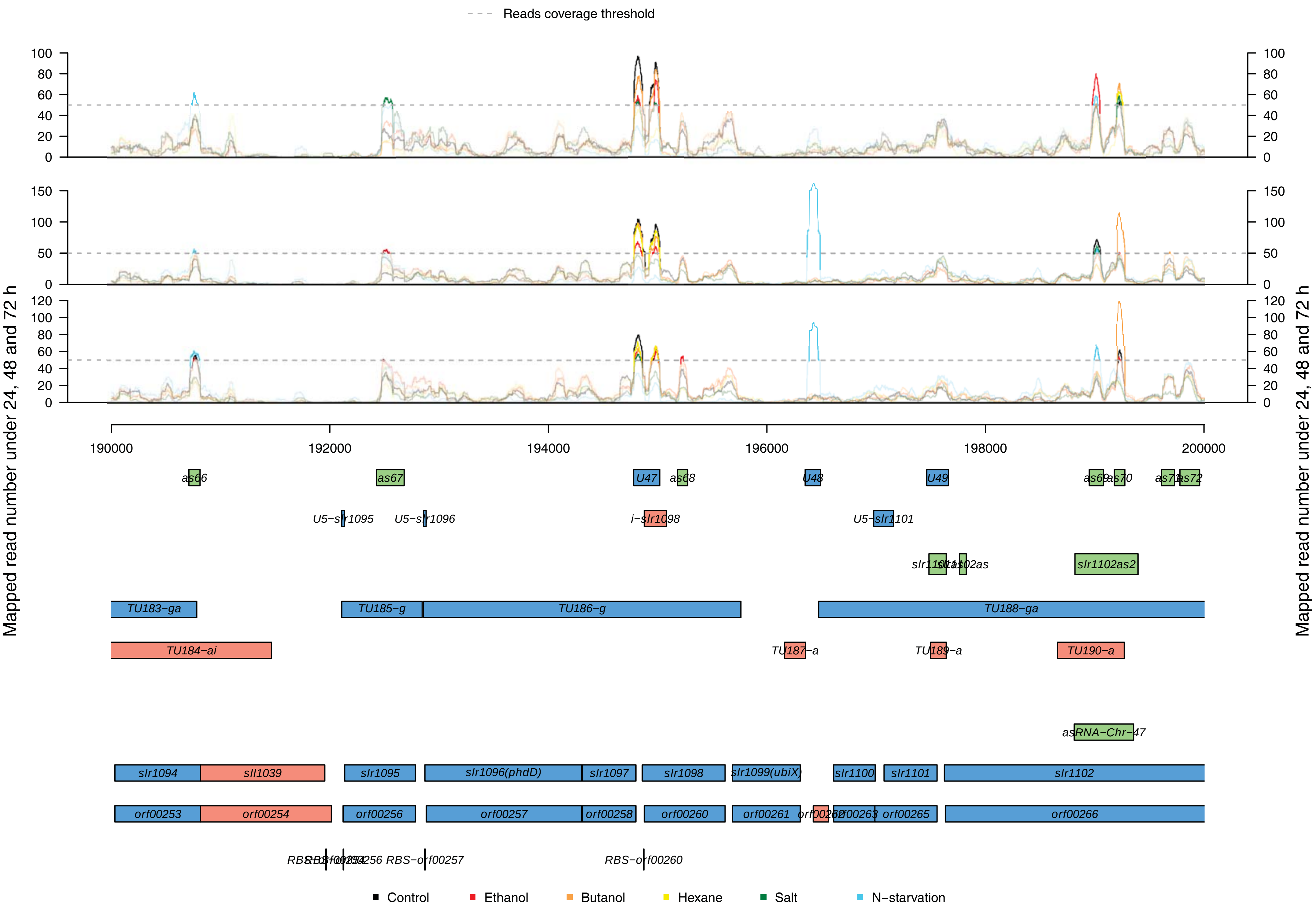

Reads coverage threshold
Mapped read number under 24, 48 and 72 h
190000
192000
194000
196000
198000
200000
as66
as67
U47
as68
U48
U49
U5−slr1095
U5−slr1096
i−slr1098
U5−slr1101
slr1102as2
TU183−ga
TU185−g
TU186−g
TU188−ga
TU184−ai
TU187−a
TU189−a
TU190−a
asRNA−Chr−47
slr1094
sll1039
slr1095
slr1096(phdD)
slr1097
slr1098
slr1099(ubiX)
slr1100
slr1101
slr1102
orf00253
orf00254
orf00256
orf00257
orf00258
orf00260
orf00261
orf00265
orf00266
RBS−orf00257
RBS−orf00260
Control
Ethanol
Butanol
Hexane
Salt
N−starvation

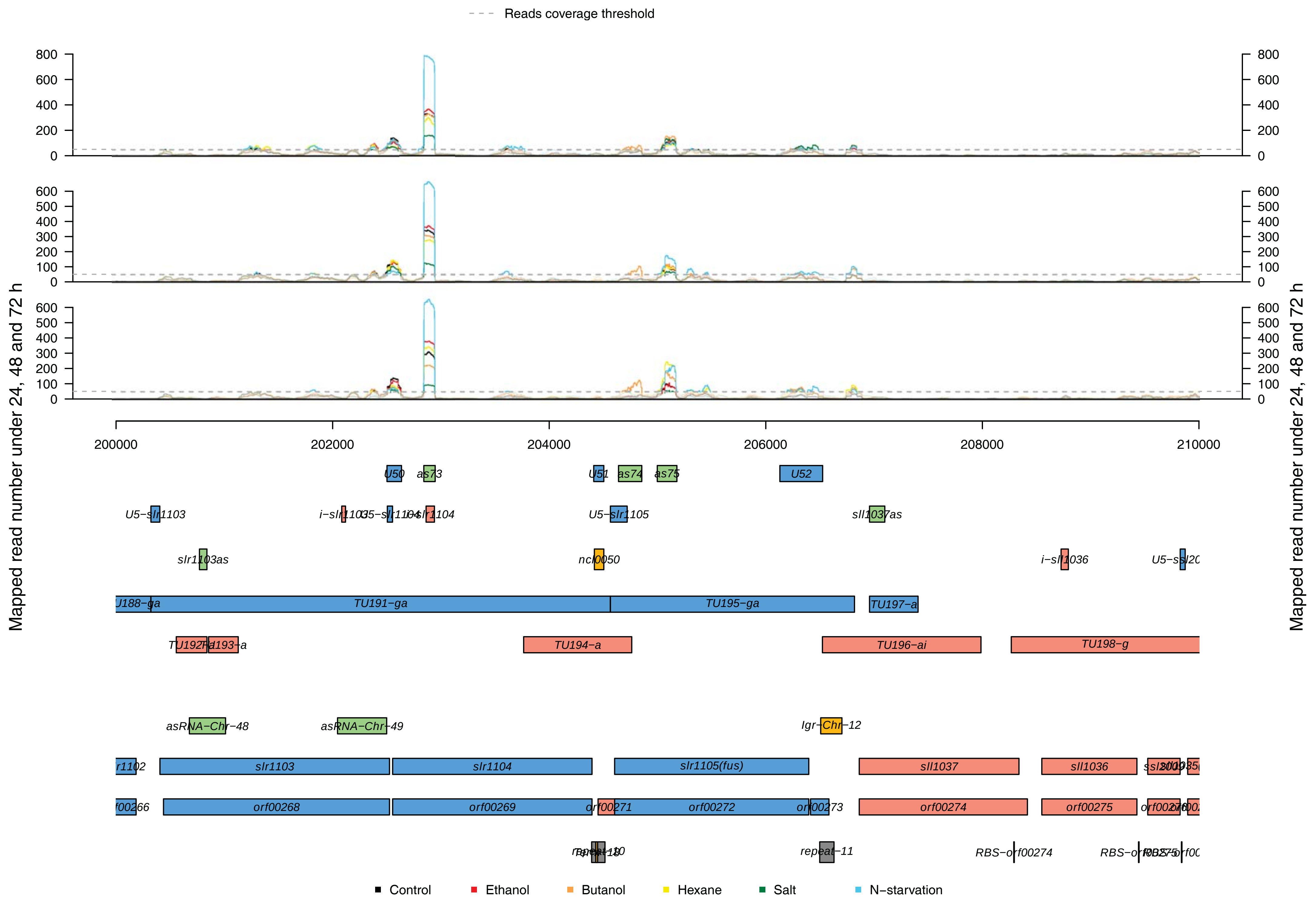
Reads coverage threshold
Mapped read number under 24, 48 and 72 h
0
200
400
600
800
100
300
500
200000
202000
204000
206000
208000
210000
U50
as73
U51
as74
as75
U52
U5−slr1103
i−slr1103
U5−slr1104
i−slr1104
U5−slr1105
sll1037as
slr1103as
ncl0050
i−sll1036
TU191−ga
TU195−ga
TU197−a
TU194−a
TU196−ai
TU198−g
asRNA−Chr−48
asRNA−Chr−49
Igr−Chr−12
slr1103
slr1104
slr1105(fus)
sll1037
sll1036
orf00268
orf00269
orf00271
orf00272
orf00273
orf00274
orf00275
repeat−11
RBS−orf00274
Control
Ethanol
Butanol
Hexane
Salt
N−starvation

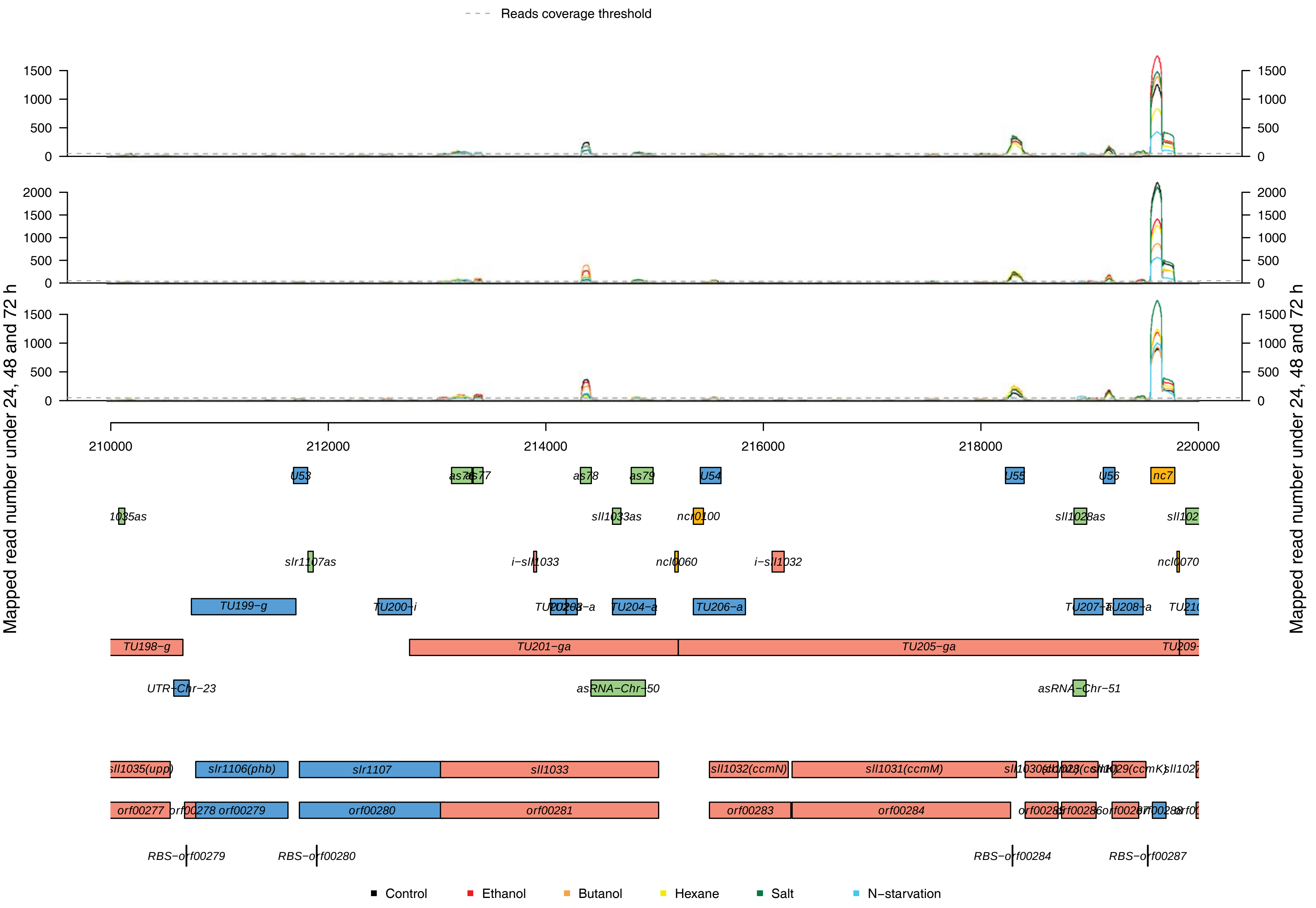

Reads coverage threshold
Mapped read number under 24, 48 and 72 h
210000
212000
214000
216000
218000
220000
TU199−g
TU201−ga
TU205−ga
TU198−g
asRNA−Chr−50
asRNA−Chr−51
UTR−Chr−23
slr1106(phb)
slr1107
sll1033
sll1032(ccmN)
sll1031(ccmM)
orf00277
orf00280
orf00281
orf00283
orf00284
RBS−orf00279
RBS−orf00280
RBS−orf00284
RBS−orf00287
Control
Ethanol
Butanol
Hexane
Salt
N−starvation

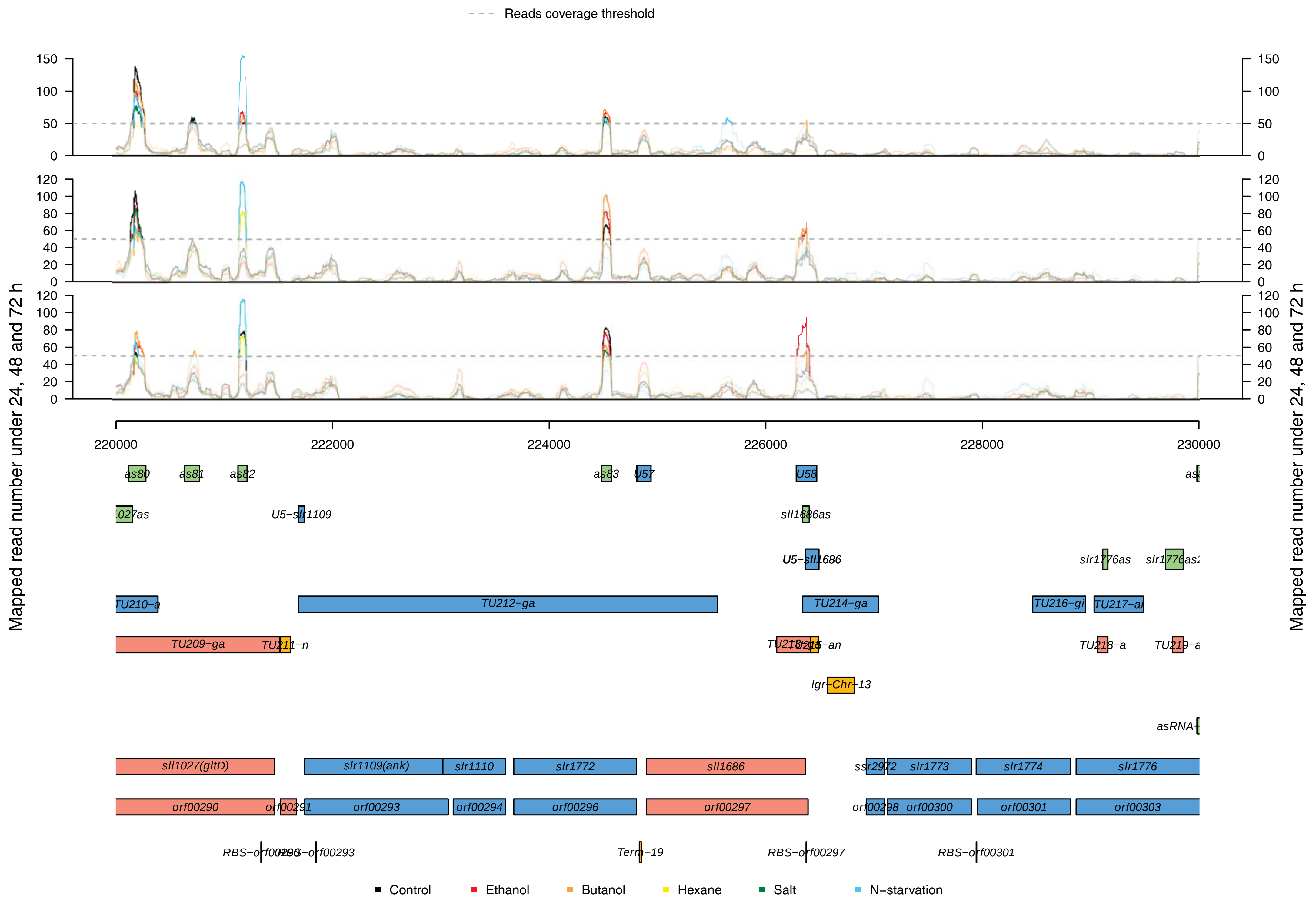

Reads coverage threshold
Mapped read number under 24, 48 and 72 h
220000
222000
224000
226000
228000
230000
as80
as81
as82
as83
U57
U58
U5−slr1109
sll1686as
U5−sll1686
slr1776as
TU212−ga
TU214−ga
TU209−ga
Igr−Chr−13
sll1027(gltD)
slr1109(ank)
slr1110
slr1772
sll1686
slr1773
slr1774
slr1776
orf00290
orf00293
orf00294
orf00296
orf00297
orf00300
orf00301
orf00303
Term−19
RBS−orf00297
RBS−orf00301
Control
Ethanol
Butanol
Hexane
Salt
N−starvation

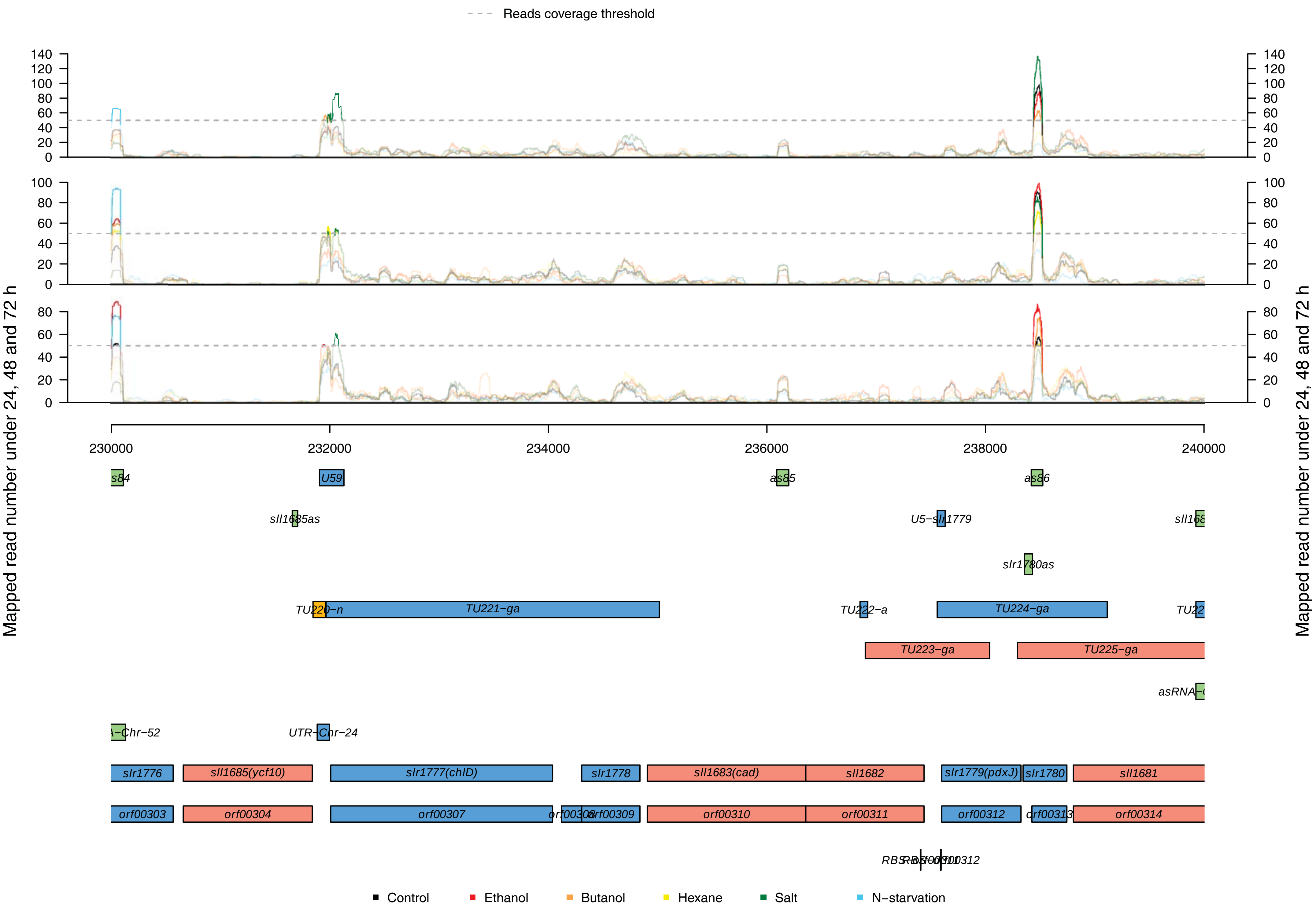

Reads coverage threshold
Mapped read number under 24, 48 and 72 h
230000
232000
234000
236000
238000
240000
s84
U59
as85
as86
sll1685as
U5−slr1779
slr1780as
TU220−n
TU221−ga
TU222−a
TU224−ga
TU223−ga
TU225−ga
asRNA
UTR−Chr−24
slr1776
sll1685(ycf10)
slr1777(chlD)
slr1778
sll1683(cad)
sll1682
slr1779(pdxJ)
slr1780
sll1681
orf00303
orf00304
orf00307
orf00309
orf00310
orf00311
orf00312
orf00314
Control
Ethanol
Butanol
Hexane
Salt
N−starvation

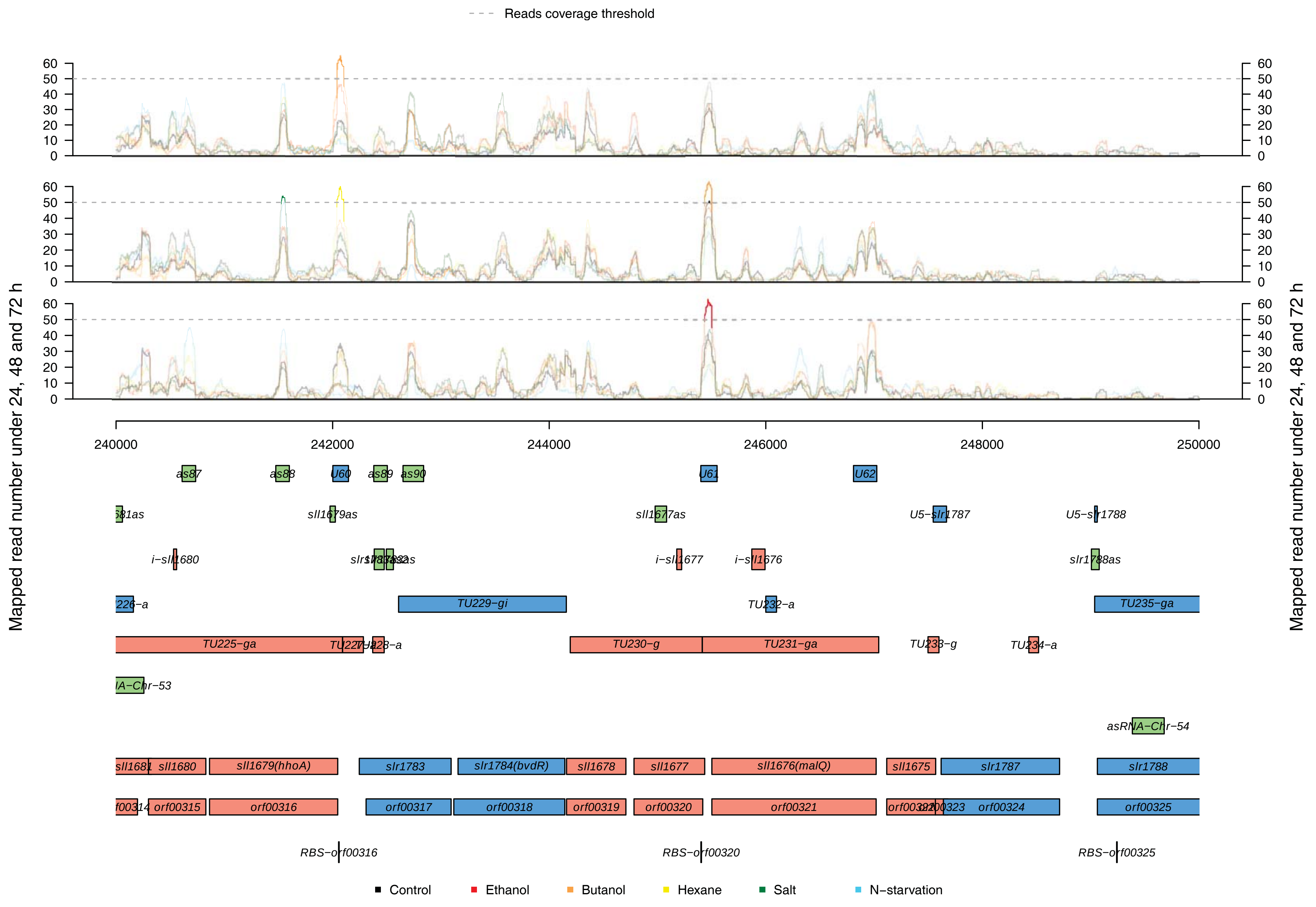

Reads coverage threshold
Mapped read number under 24, 48 and 72 h
240000
242000
244000
246000
248000
250000
as87
as88
U60
as89
as90
U61
U62
sll1679as
sll1677as
U5−slr1787
U5−slr1788
i−sll1680
i−sll1677
i−sll1676
slr1788as
TU229−gi
TU232−a
TU235−ga
TU225−ga
TU230−g
TU231−ga
TU234−a
asRNA−Chr−54
sll1680
sll1679(hhoA)
slr1783
slr1784(bvdR)
sll1678
sll1677
sll1676(malQ)
sll1675
slr1787
slr1788
orf00315
orf00316
orf00317
orf00318
orf00319
orf00320
orf00321
orf00324
orf00325
RBS−orf00316
RBS−orf00320
RBS−orf00325
Control
Ethanol
Butanol
Hexane
Salt
N−starvation

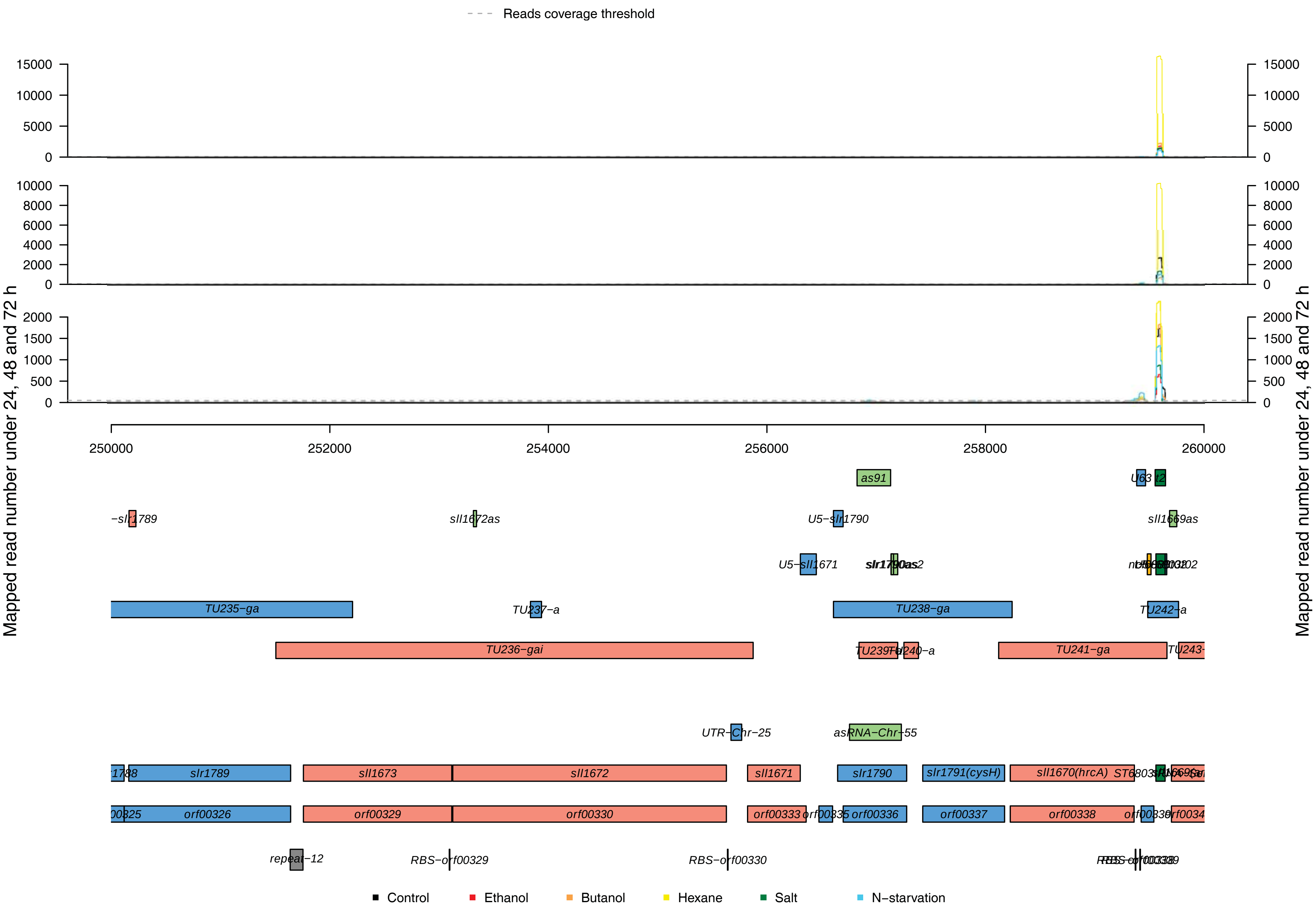

Reads coverage threshold
Mapped read number under 24, 48 and 72 h
250000
252000
254000
256000
258000
260000
as91
sll1672as
U5−slr1790
sll1669as
U5−sll1671
TU235−ga
TU238−ga
TU236−gai
TU241−ga
UTR−Chr−25
asRNA−Chr−55
slr1789
sll1673
sll1672
sll1671
slr1790
slr1791(cysH)
sll1670(hrcA)
orf00326
orf00329
orf00330
orf00333
orf00336
orf00337
orf00338
repeat−12
RBS−orf00329
RBS−orf00330
Control
Ethanol
Butanol
Hexane
Salt
N−starvation

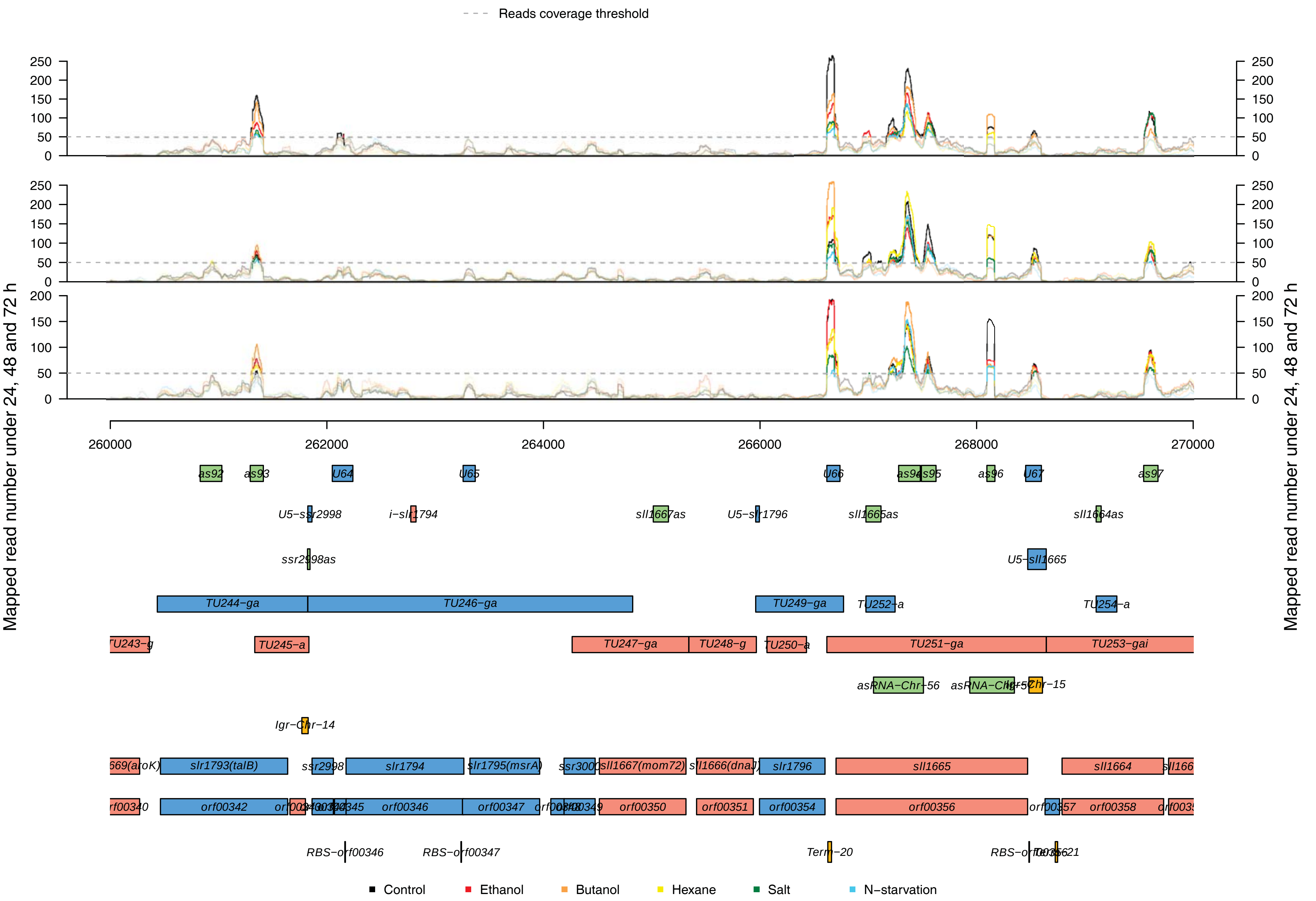

Reads coverage threshold
Mapped read number under 24, 48 and 72 h
260000
262000
264000
266000
268000
270000
as92
as93
U64
U65
U66
as94
as95
as96
U67
as97
U5−ssr2998
i−slr1794
sll1667as
U5−slr1796
sll1665as
sll1664as
ssr2998as
U5−sll1665
TU244−ga
TU246−ga
TU249−ga
TU252−a
TU254−a
TU243−g
TU245−a
TU247−ga
TU248−g
TU250−a
TU251−ga
TU253−gai
asRNA−Chr−56
Igr−Chr−15
Igr−Chr−14
slr1793(talB)
ssr2998
slr1794
slr1795(msrA)
sll1667(mom72)
slr1796
sll1665
sll1664
orf00340
orf00342
orf00345
orf00346
orf00347
orf00349
orf00350
orf00351
orf00354
orf00356
orf00358
RBS−orf00346
RBS−orf00347
Term−20
Control
Ethanol
Butanol
Hexane
Salt
N−starvation

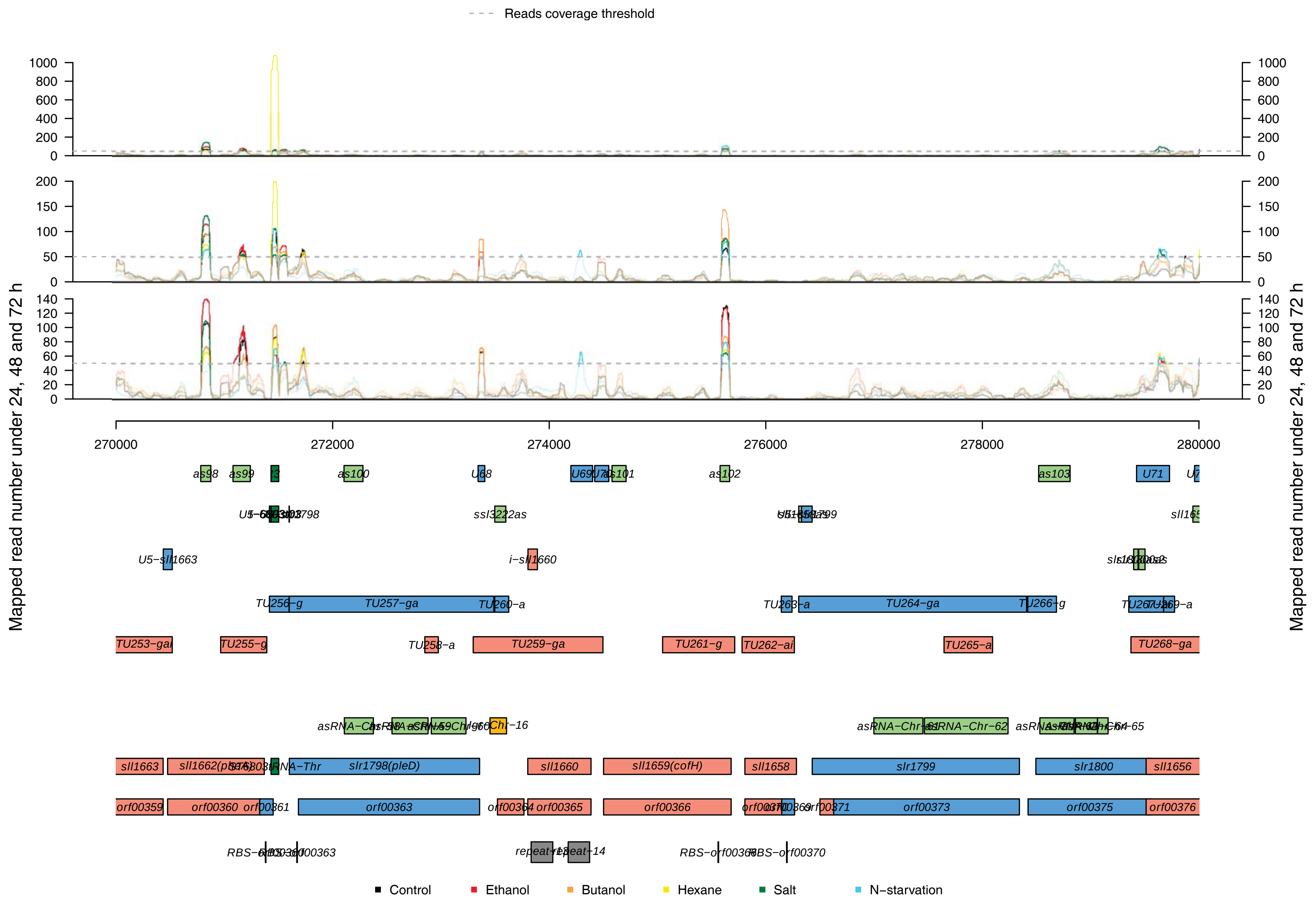

Reads coverage threshold
Mapped read number under 24, 48 and 72 h
270000
272000
274000
276000
278000
280000
Control
Ethanol
Butanol
Hexane
Salt
N−starvation

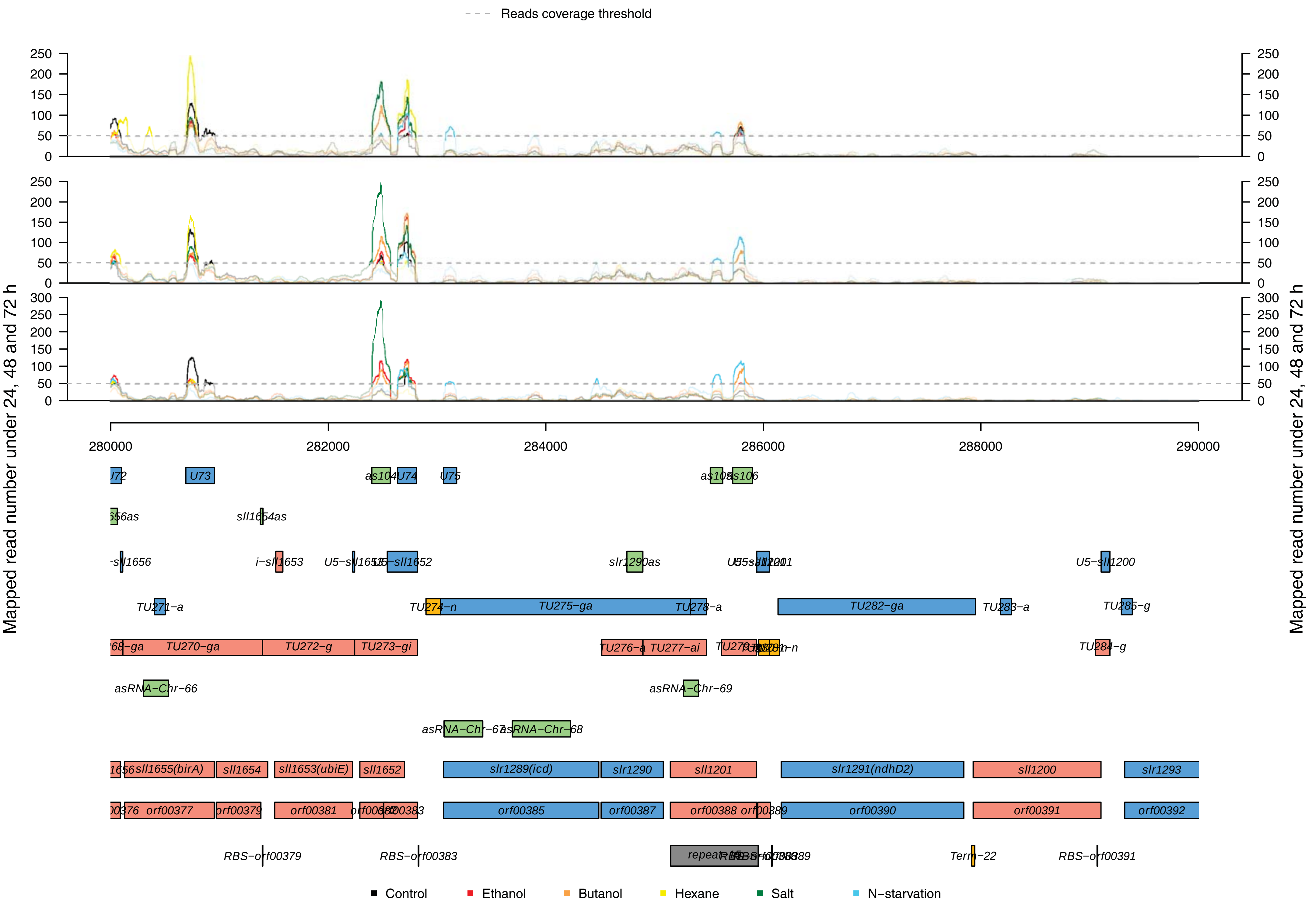

Reads coverage threshold
Mapped read number under 24, 48 and 72 h
280000
282000
284000
286000
288000
290000
TU275–ga
TU282–ga
TU270–ga
TU272–g
TU273–gi
TU277–ai
asRNA–Chr–66
asRNA–Chr–69
slr1289(icd)
slr1290
sll1201
slr1291(ndhD2)
sll1200
slr1293
sll1655(birA)
sll1654
sll1653(ubiE)
sll1652
orf00377
orf00379
orf00381
orf00385
orf00387
orf00390
orf00391
orf00392
RBS–orf00379
RBS–orf00383
RBS–orf00391
Control
Ethanol
Butanol
Hexane
Salt
N–starvation

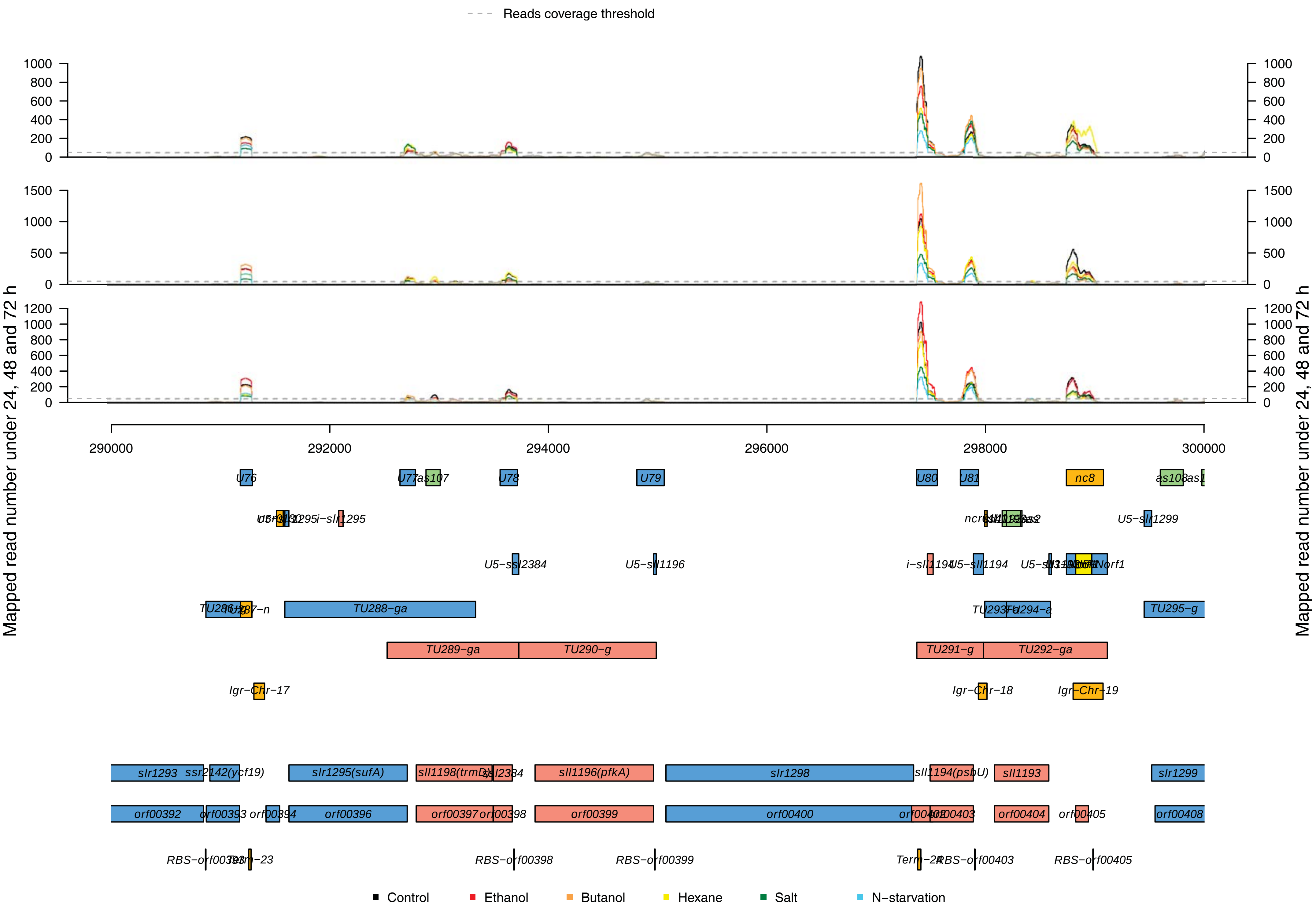

Reads coverage threshold
Mapped read number under 24, 48 and 72 h
1000
800
600
400
200
0
1500
1000
500
0
1200
1000
800
600
400
200
0
290000
292000
294000
296000
298000
300000
U76
as107
U78
U79
U80
U81
nc8
i−slr1295
U5−ssl2384
U5−sll1196
U5−sll1194
U5−slr1299
TU288−ga
TU295−g
TU289−ga
TU290−g
TU291−g
TU292−ga
Igr−Chr−17
Igr−Chr−18
Igr−Chr−19
slr1293
slr1295(sufA)
sll1196(pfkA)
slr1298
sll1193
slr1299
orf00392
orf00396
orf00399
orf00400
orf00404
orf00408
RBS−orf00398
RBS−orf00399
RBS−orf00403
RBS−orf00405
Control
Ethanol
Butanol
Hexane
Salt
N−starvation

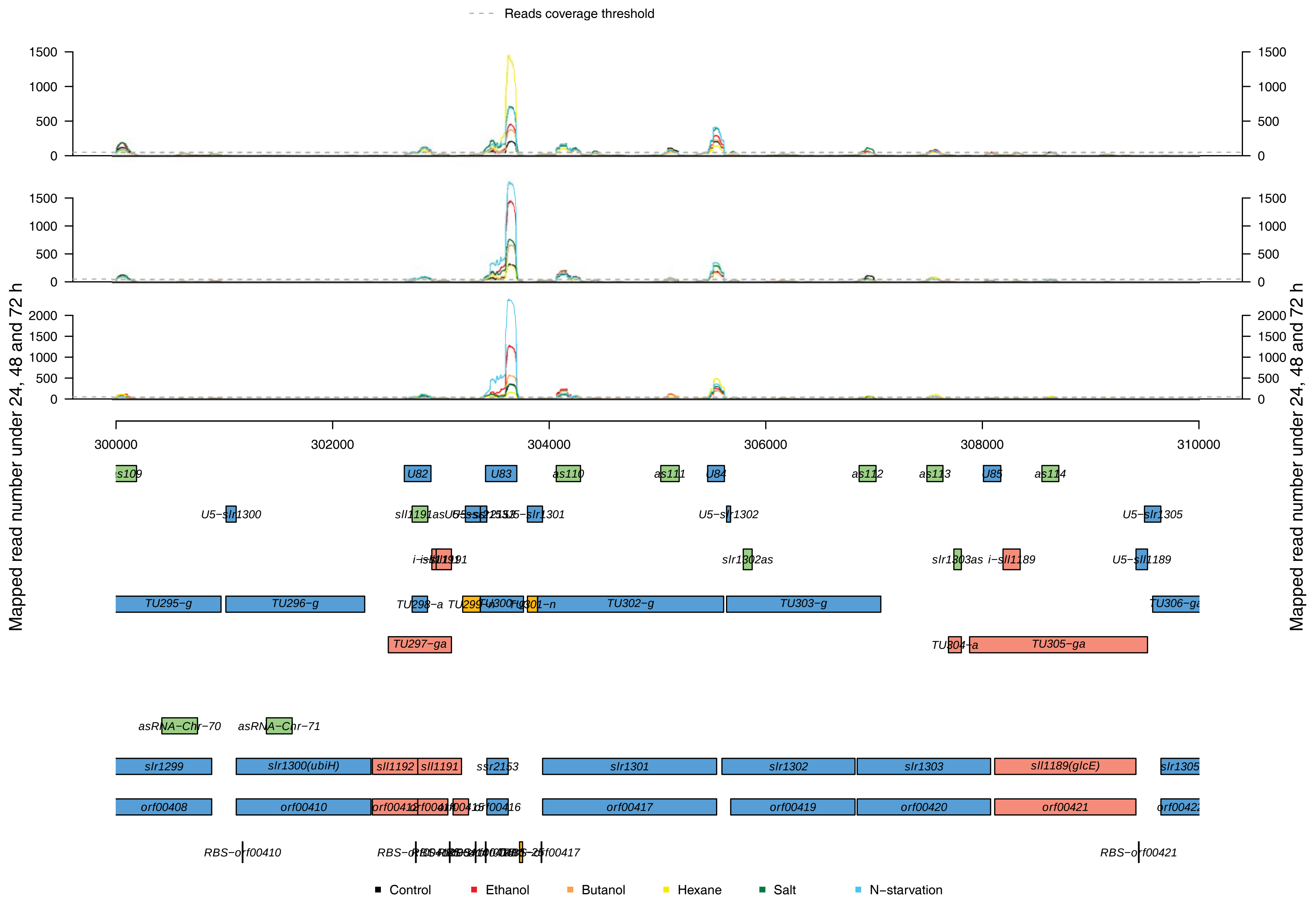

Reads coverage threshold
Mapped read number under 24, 48 and 72 h
300000
302000
304000
306000
308000
310000
TU295−g
TU296−g
TU297−ga
TU302−g
TU303−g
TU305−ga
asRNA−Chr−70
asRNA−Chr−71
slr1299
slr1300(ubiH)
sll1192
sll1191
ssr2153
slr1301
slr1302
slr1303
sll1189(glcE)
slr1305
orf00408
orf00410
orf00416
orf00417
orf00419
orf00420
orf00421
RBS−orf00410
RBS−orf00421
Control
Ethanol
Butanol
Hexane
Salt
N−starvation

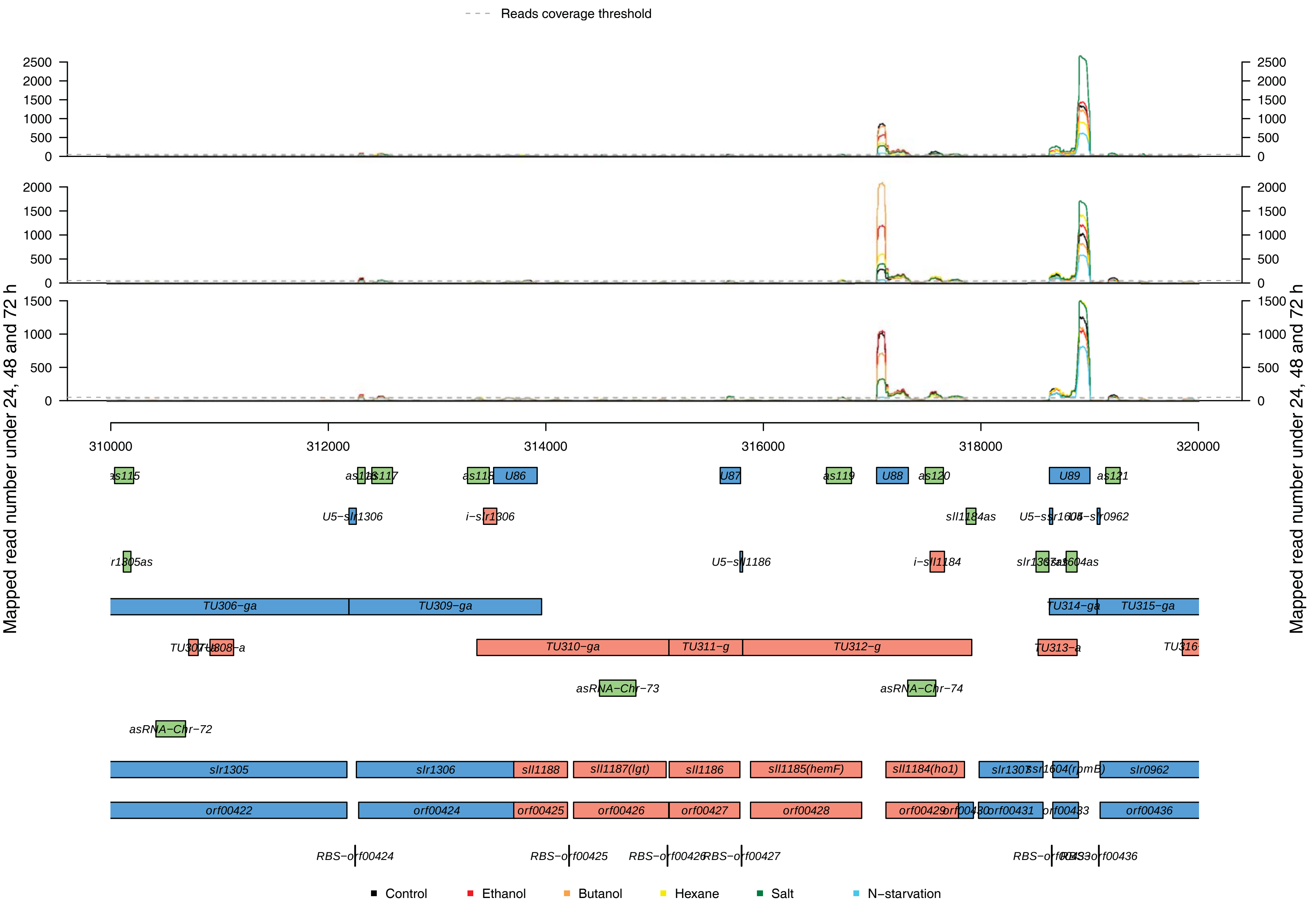
Reads coverage threshold
Mapped read number under 24, 48 and 72 h
310000
312000
314000
316000
318000
320000
TU306−ga
TU309−ga
TU310−ga
TU311−g
TU312−g
TU314−ga
TU315−ga
asRNA−Chr−73
asRNA−Chr−74
asRNA−Chr−72
slr1305
slr1306
sll1188
sll1187(lgt)
sll1186
sll1185(hemF)
sll1184(ho1)
slr0962
orf00422
orf00424
orf00425
orf00426
orf00427
orf00428
orf00431
orf00436
RBS−orf00424
RBS−orf00425
RBS−orf00427
U86
U87
U88
U89
as117
as119
as120
as121
U5−slr1306
i−slr1306
sll1184as
U5−sll1186
i−sll1184
Control
Ethanol
Butanol
Hexane
Salt
N−starvation

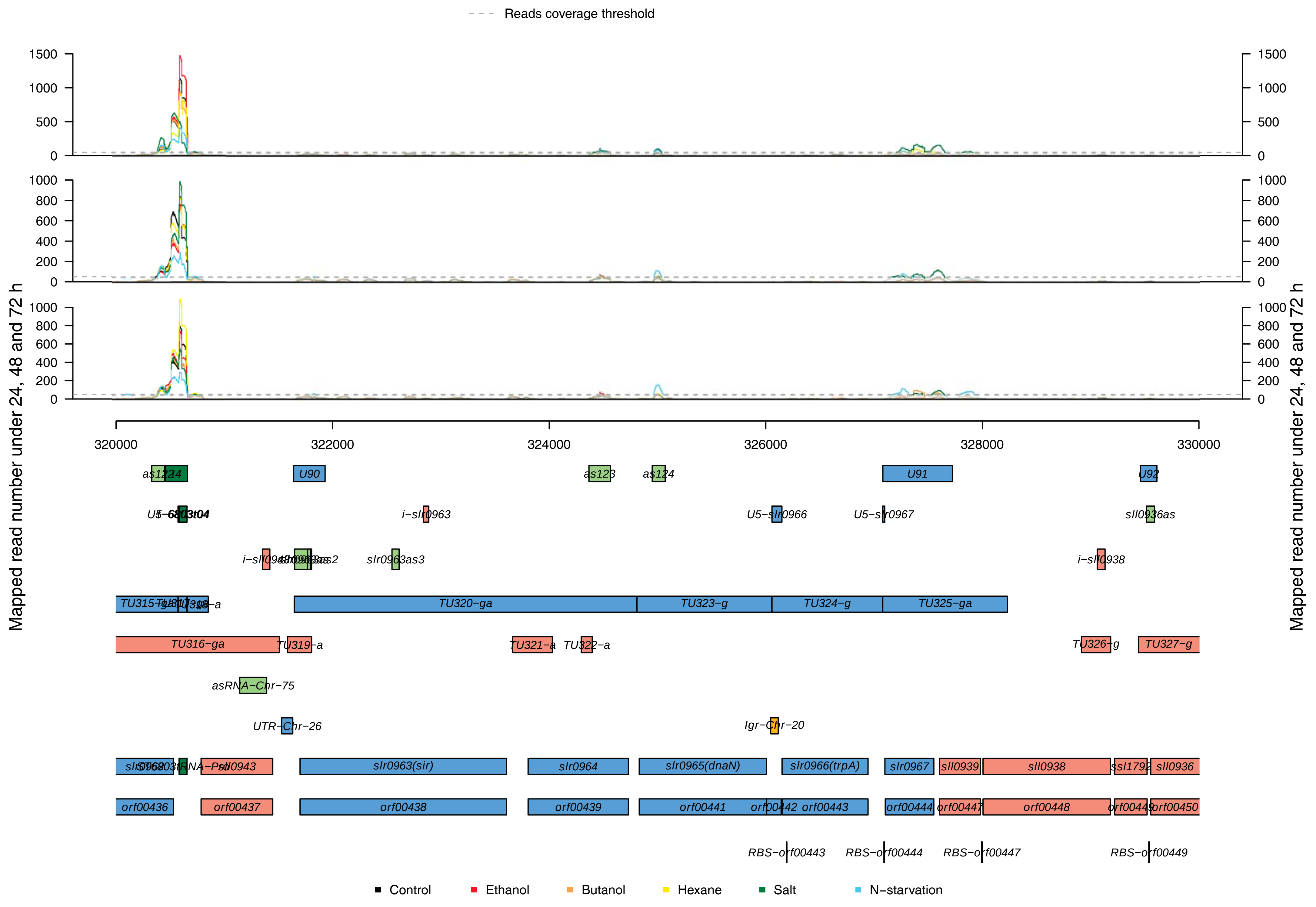

Reads coverage threshold
Mapped read number under 24, 48 and 72 h
1500
1000
500
0
800
600
400
200
320000
322000
324000
326000
328000
330000
U90
as123
as124
U91
U92
i-sll0963
U5-slr0966
U5-slr0967
sll0936as
slr0963as3
i-sll0938
TU320-ga
TU323-g
TU324-g
TU325-ga
TU316-ga
TU319-a
TU321-a
TU322-a
TU326-g
TU327-g
asRNA-Chr-75
UTR-Chr-26
Igr-Chr-20
slr0963(sir)
slr0964
slr0965(dnaN)
slr0966(trpA)
slr0967
sll0939
sll0938
ssl1792
sll0936
sll0943
orf00436
orf00437
orf00438
orf00439
orf00441
orf00442
orf00443
orf00444
orf00447
orf00448
orf00449
orf00450
RBS-orf00443
RBS-orf00444
RBS-orf00447
RBS-orf00449
Control
Ethanol
Butanol
Hexane
Salt
N-starvation

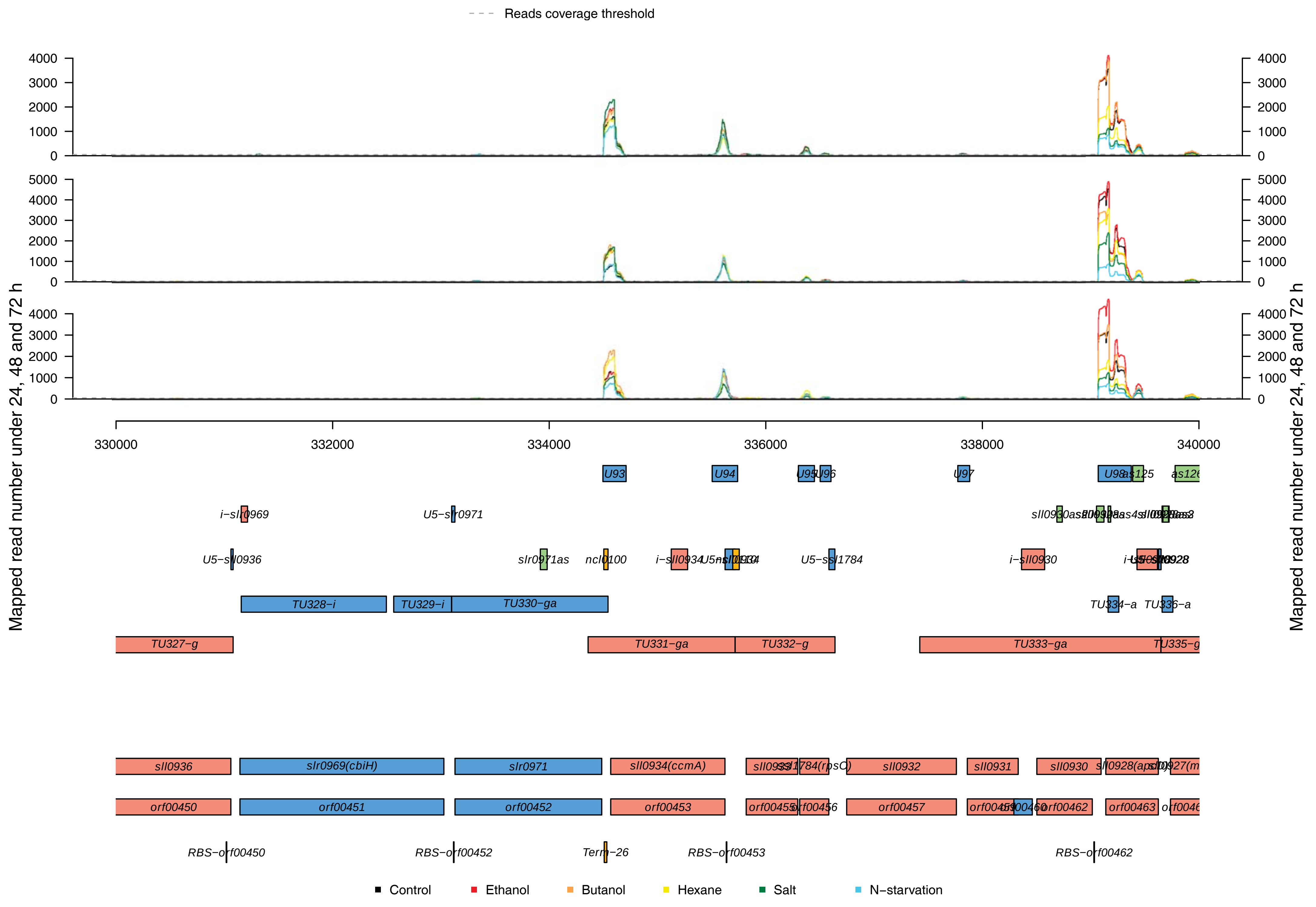

Reads coverage threshold
Mapped read number under 24, 48 and 72 h
330000
332000
334000
336000
338000
340000
U93
U94
U97
TU328−i
TU329−i
TU330−ga
TU331−ga
TU332−g
TU333−ga
TU327−g
sll0936
slr0969(cbiH)
slr0971
sll0934(ccmA)
sll0932
sll0931
sll0930
orf00450
orf00451
orf00452
orf00453
orf00457
orf00462
orf00463
RBS−orf00450
RBS−orf00452
Term−26
RBS−orf00453
RBS−orf00462
Control
Ethanol
Butanol
Hexane
Salt
N−starvation

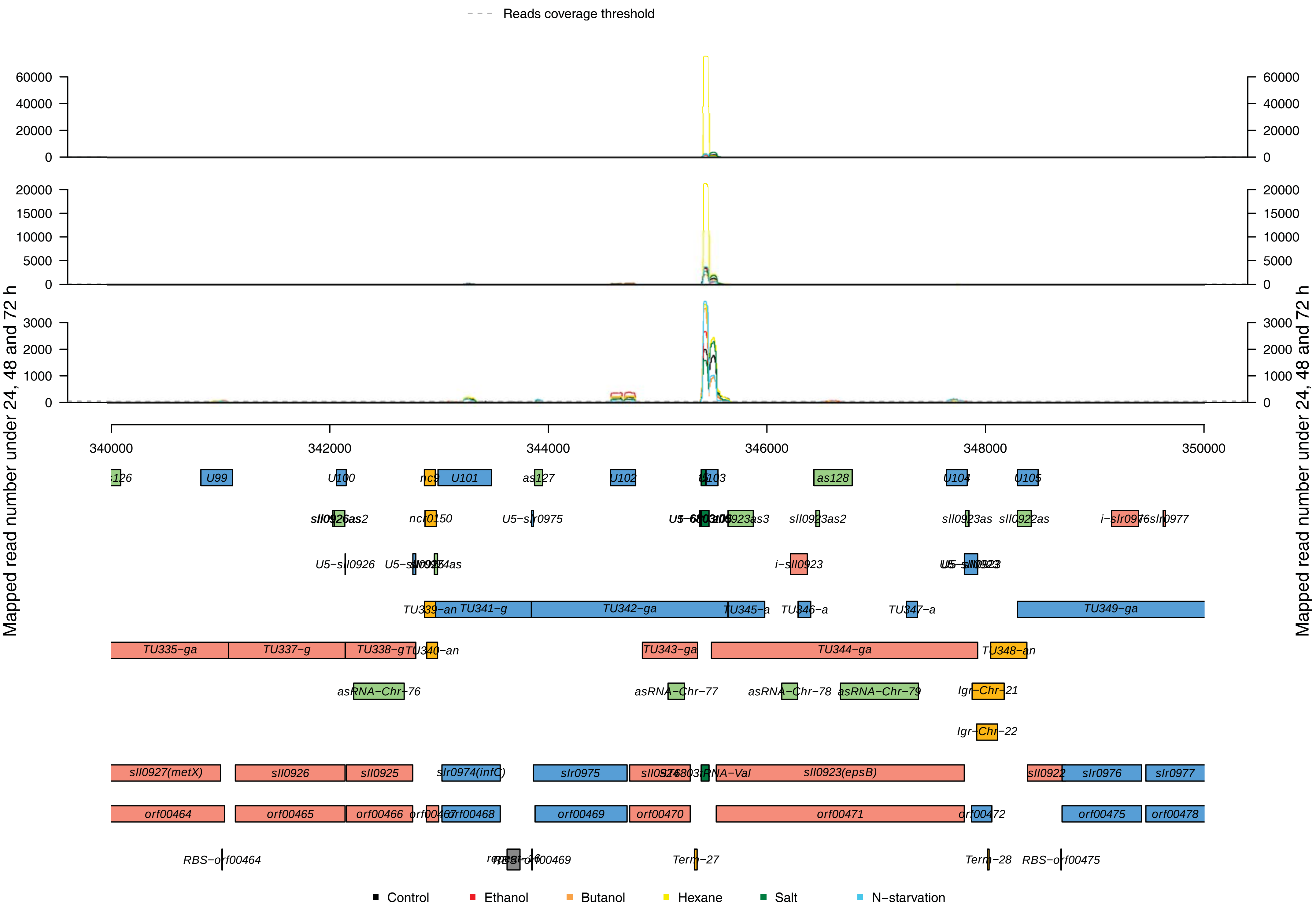

Reads coverage threshold
Mapped read number under 24, 48 and 72 h
60000
40000
20000
0
20000
15000
10000
5000
0
3000
2000
1000
0
340000
342000
344000
346000
348000
350000
U99
U100
U101
as127
U102
as128
U104
U105
ncl0150
U5−slr0975
sll0923as2
sll0923as
sll0922as
U5−sll0926
i−sll0923
TU341−g
TU342−ga
TU346−a
TU347−a
TU349−ga
TU335−ga
TU337−g
TU338−g
TU343−ga
TU344−ga
asRNA−Chr−76
asRNA−Chr−77
asRNA−Chr−78
asRNA−Chr−79
Igr−Chr−21
Igr−Chr−22
sll0927(metX)
sll0926
sll0925
slr0974(infC)
slr0975
sll0923(epsB)
sll0922
slr0976
slr0977
orf00464
orf00465
orf00466
orf00468
orf00469
orf00470
orf00471
orf00472
orf00475
orf00478
RBS−orf00464
Term−27
Term−28
RBS−orf00475
Control
Ethanol
Butanol
Hexane
Salt
N−starvation

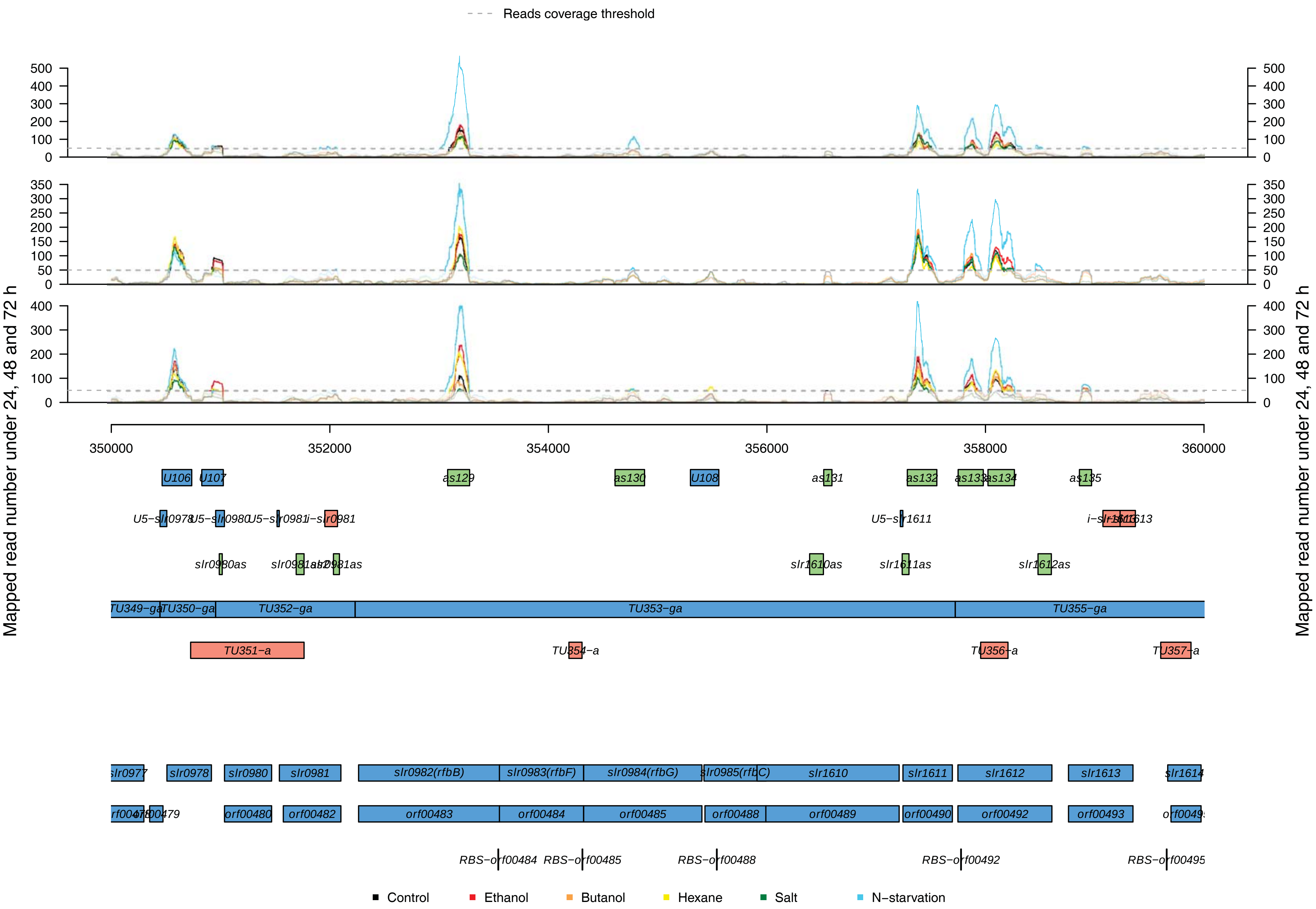

Reads coverage threshold
Mapped read number under 24, 48 and 72 h
350000
352000
354000
356000
358000
360000
U106
U107
as129
as130
U108
as131
as132
as133
as134
as135
U5−slr0978
U5−slr0980
U5−slr0981
i−slr0981
U5−slr1611
slr0980as
slr0981as
slr1610as
slr1611as
slr1612as
TU353−ga
TU355−ga
TU351−a
TU354−a
TU356−a
TU357−a
slr0977
slr0978
slr0980
slr0981
slr0982(rfbB)
slr0983(rfbF)
slr0984(rfbG)
slr1610
slr1611
slr1612
slr1613
slr1614
orf00480
orf00482
orf00483
orf00484
orf00485
orf00488
orf00489
orf00490
orf00492
orf00493
RBS−orf00484
RBS−orf00485
RBS−orf00488
RBS−orf00492
RBS−orf00495
Control
Ethanol
Butanol
Hexane
Salt
N−starvation

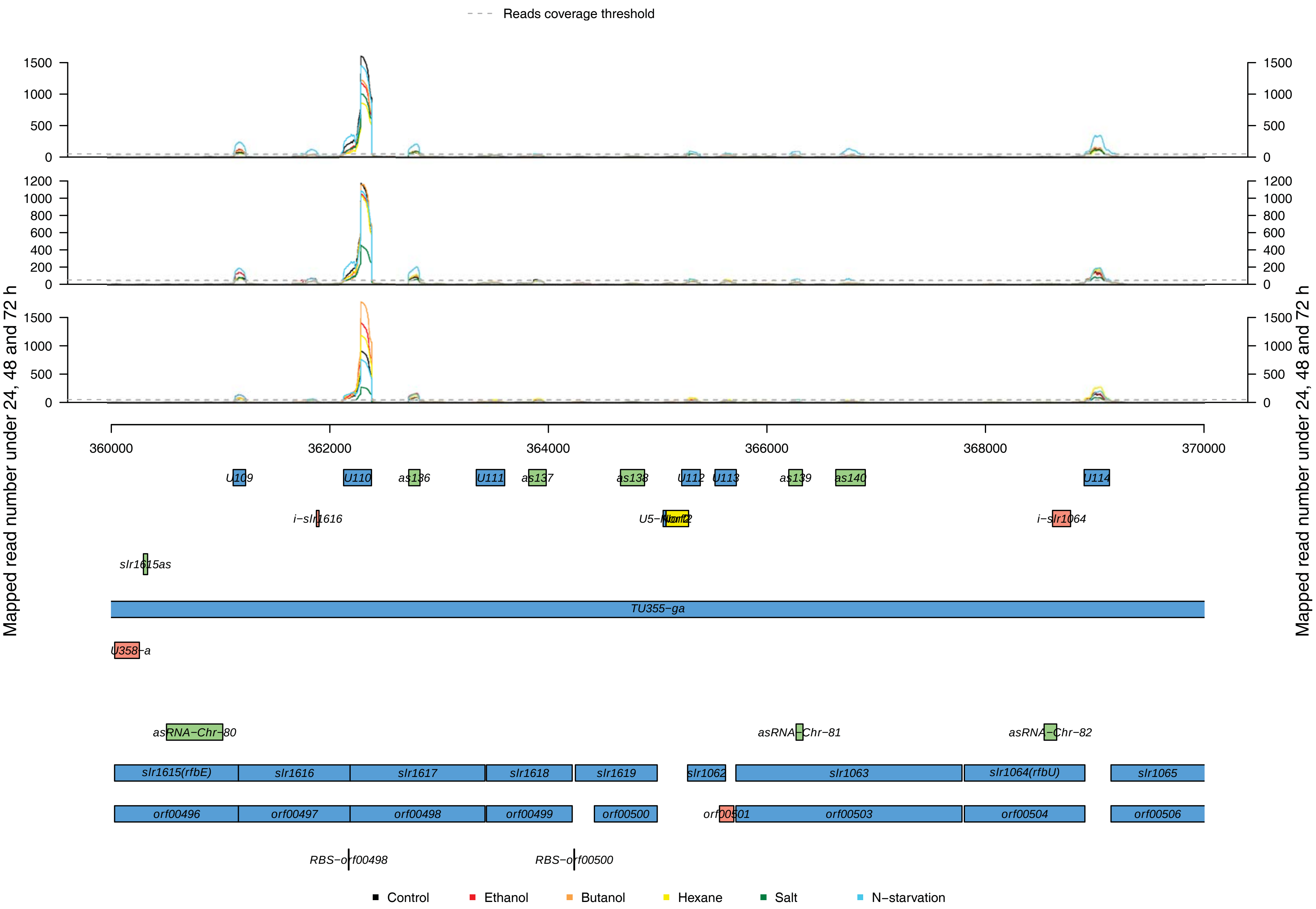

Reads coverage threshold
Mapped read number under 24, 48 and 72 h
360000
362000
364000
366000
368000
370000
U109
U110
as136
U111
as137
as138
U112
U113
as139
as140
U114
i−slr1616
i−slr1064
slr1615as
TU355−ga
U358−a
asRNA−Chr−80
asRNA−Chr−81
asRNA−Chr−82
slr1615(rfbE)
slr1616
slr1617
slr1618
slr1619
slr1062
slr1063
slr1064(rfbU)
slr1065
orf00496
orf00497
orf00498
orf00499
orf00500
orf00501
orf00503
orf00504
orf00506
RBS−orf00498
RBS−orf00500
Control
Ethanol
Butanol
Hexane
Salt
N−starvation

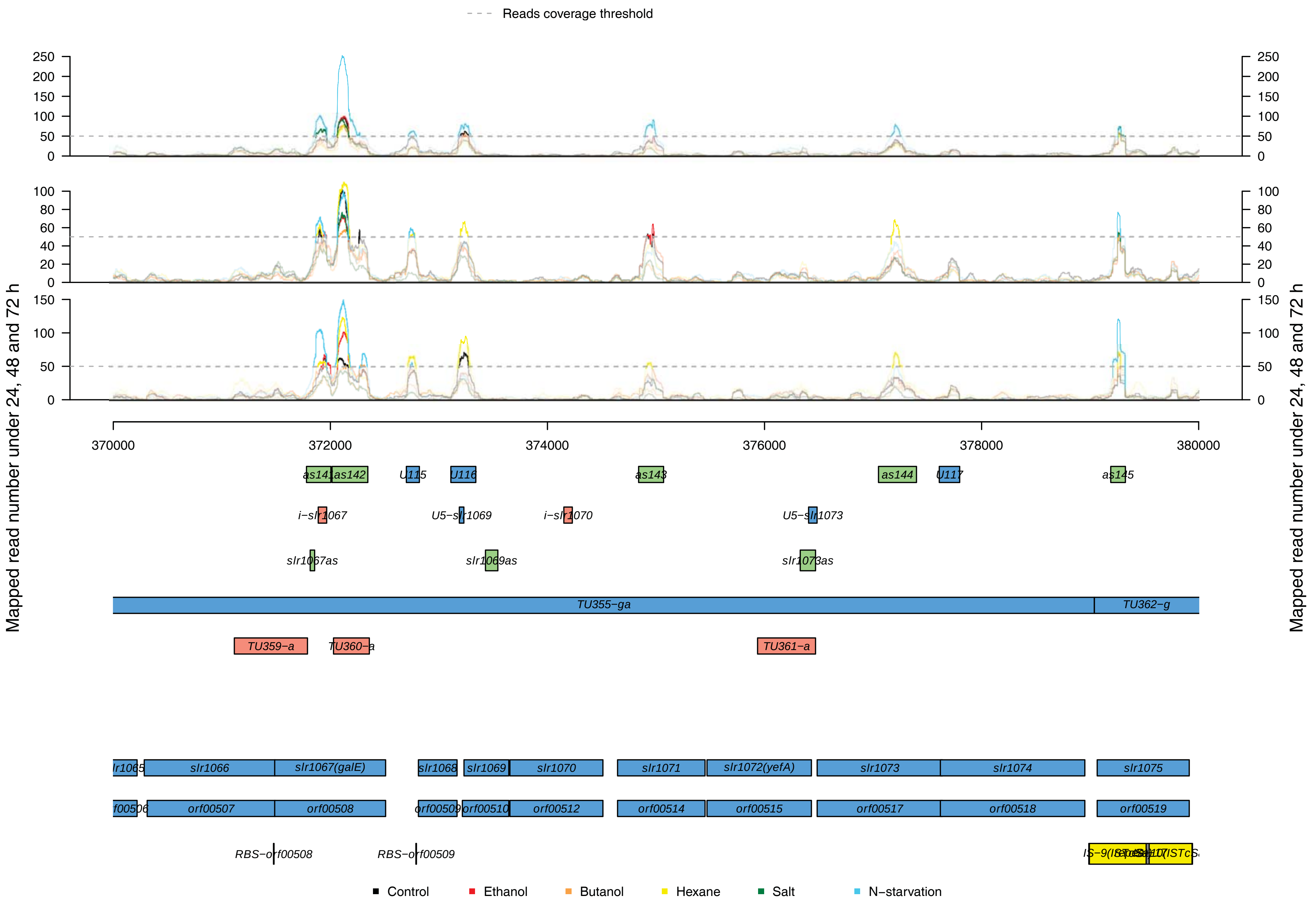

Reads coverage threshold
Mapped read number under 24, 48 and 72 h
370000
372000
374000
376000
378000
380000
as141
as142
U115
U116
as143
as144
U117
as145
i−slr1067
U5−slr1069
i−slr1070
U5−sll1073
slr1067as
slr1069as
slr1073as
TU355−ga
TU362−g
TU359−a
TU360−a
TU361−a
slr1066
slr1067(galE)
slr1068
slr1069
slr1070
slr1071
slr1072(yefA)
slr1073
slr1074
slr1075
orf00507
orf00508
orf00509
orf00510
orf00512
orf00514
orf00515
orf00517
orf00518
orf00519
RBS−orf00508
RBS−orf00509
Control
Ethanol
Butanol
Hexane
Salt
N−starvation

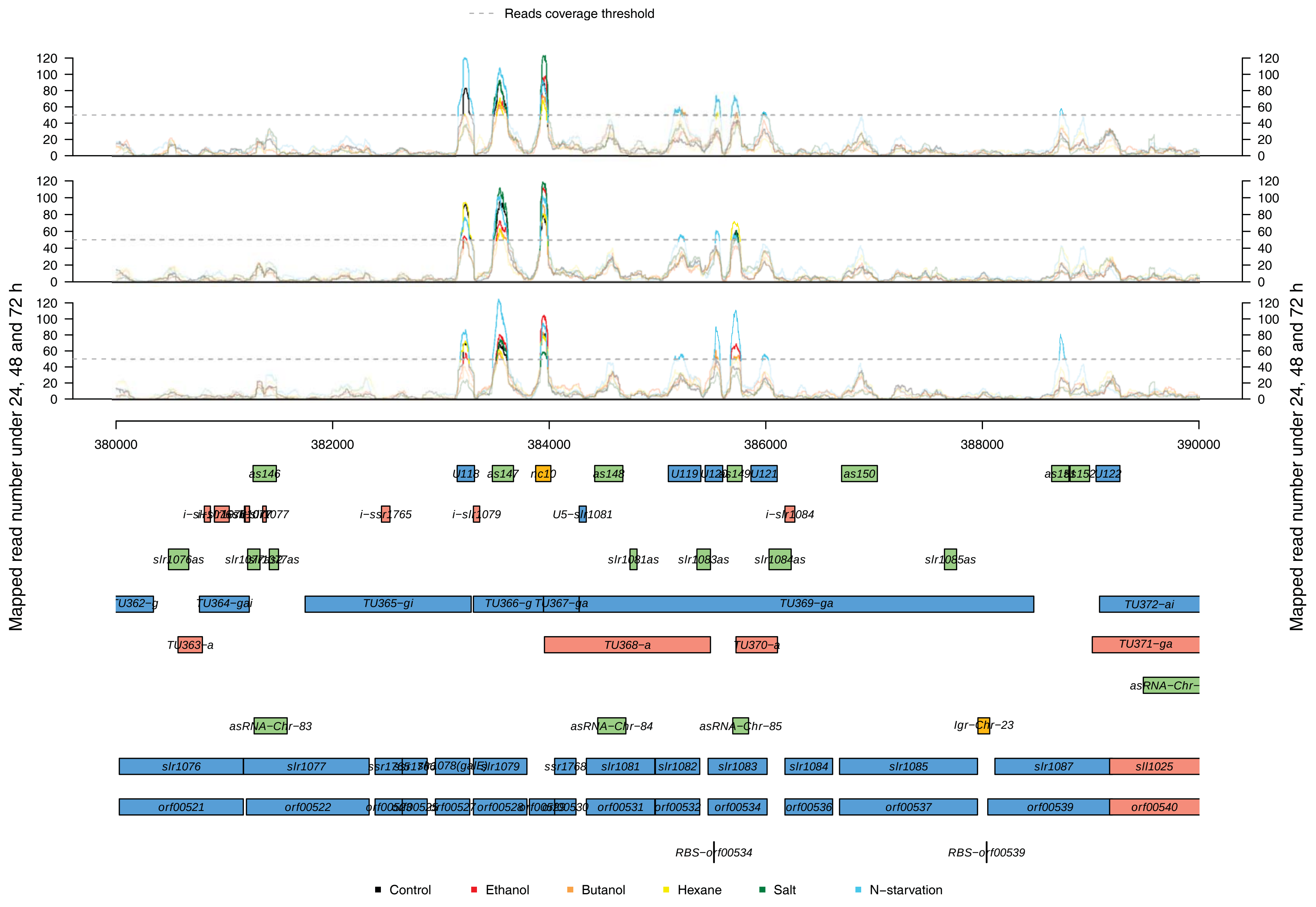

Reads coverage threshold
Mapped read number under 24, 48 and 72 h
380000
382000
384000
386000
388000
390000
as146
U118
as147
ncl10
as148
U119
U120
as149
U121
as150
as152
U122
i−ssr1765
i−slr1079
U5−slr1081
i−slr1084
slr1076as
slr1081as
slr1083as
slr1084as
slr1085as
TU362−g
TU364−gai
TU365−gi
TU366−g
TU367−ga
TU369−ga
TU372−ai
TU363−a
TU368−a
TU370−a
TU371−ga
asRNA−Chr−83
asRNA−Chr−84
asRNA−Chr−85
Igr−Chr−23
slr1076
slr1077
slr1079
ssr1768
slr1081
slr1082
slr1083
slr1084
slr1085
slr1087
sll1025
orf00521
orf00522
orf00527
orf00528
orf00531
orf00532
orf00534
orf00536
orf00537
orf00539
orf00540
RBS−orf00534
RBS−orf00539
Control
Ethanol
Butanol
Hexane
Salt
N−starvation

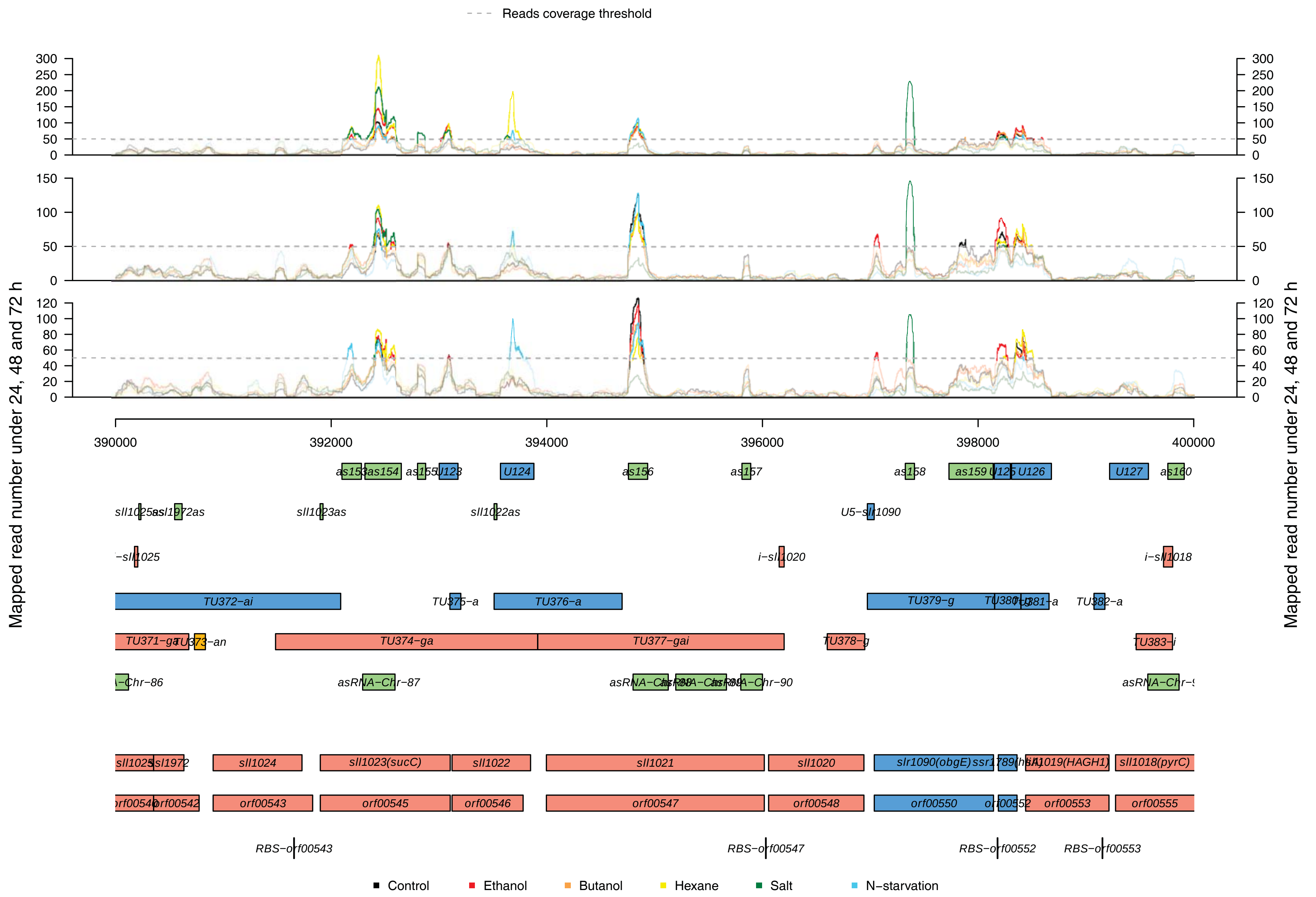

Reads coverage threshold
Mapped read number under 24, 48 and 72 h
390000
392000
394000
396000
398000
400000
as153
as154
as155
U123
U124
as156
as157
as158
as159
U125
U126
U127
as160
sll1025as
sll1972as
sll1023as
sll1022as
U5−slr1090
i−sll1020
i−sll1018
TU372−ai
TU375−a
TU376−a
TU379−g
TU381−a
TU382−a
TU374−ga
TU377−gai
TU378−g
TU383−i
asRNA−Chr−87
asRNA−Chr−90
sll1025
sll1972
sll1024
sll1023(sucC)
sll1022
sll1021
sll1020
slr1090(obgE)
sll1019(HAGH1)
sll1018(pyrC)
orf00542
orf00543
orf00545
orf00546
orf00547
orf00548
orf00550
orf00552
orf00553
orf00555
RBS−orf00543
RBS−orf00547
RBS−orf00552
RBS−orf00553
Control
Ethanol
Butanol
Hexane
Salt
N−starvation

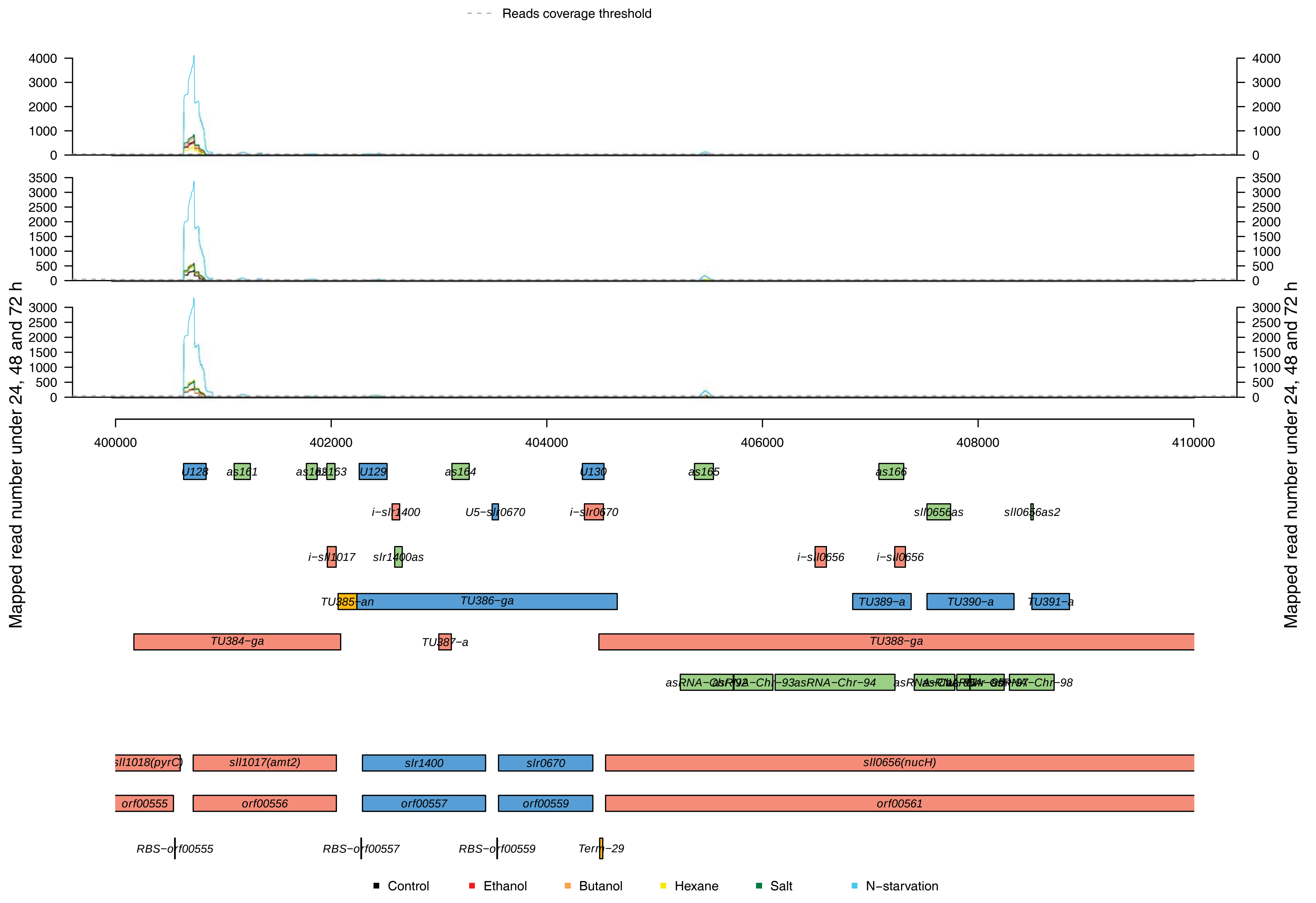
Reads coverage threshold
Mapped read number under 24, 48 and 72 h
400000
402000
404000
406000
408000
410000
U128
as161
as162
as163
U129
as164
U130
as165
as166
i−slr1400
U5−slr0670
i−slr0670
sll0656as
sll0656as2
i−sll1017
slr1400as
i−sll0656
i−sll0656
TU385−an
TU386−ga
TU389−a
TU390−a
TU391−a
TU384−ga
TU387−a
TU388−ga
asRNA−Chr−94
sll1018(pyrC)
sll1017(amt2)
slr1400
slr0670
sll0656(nucH)
orf00555
orf00556
orf00557
orf00559
orf00561
RBS−orf00555
RBS−orf00557
RBS−orf00559
Term−29
Control
Ethanol
Butanol
Hexane
Salt
N−starvation

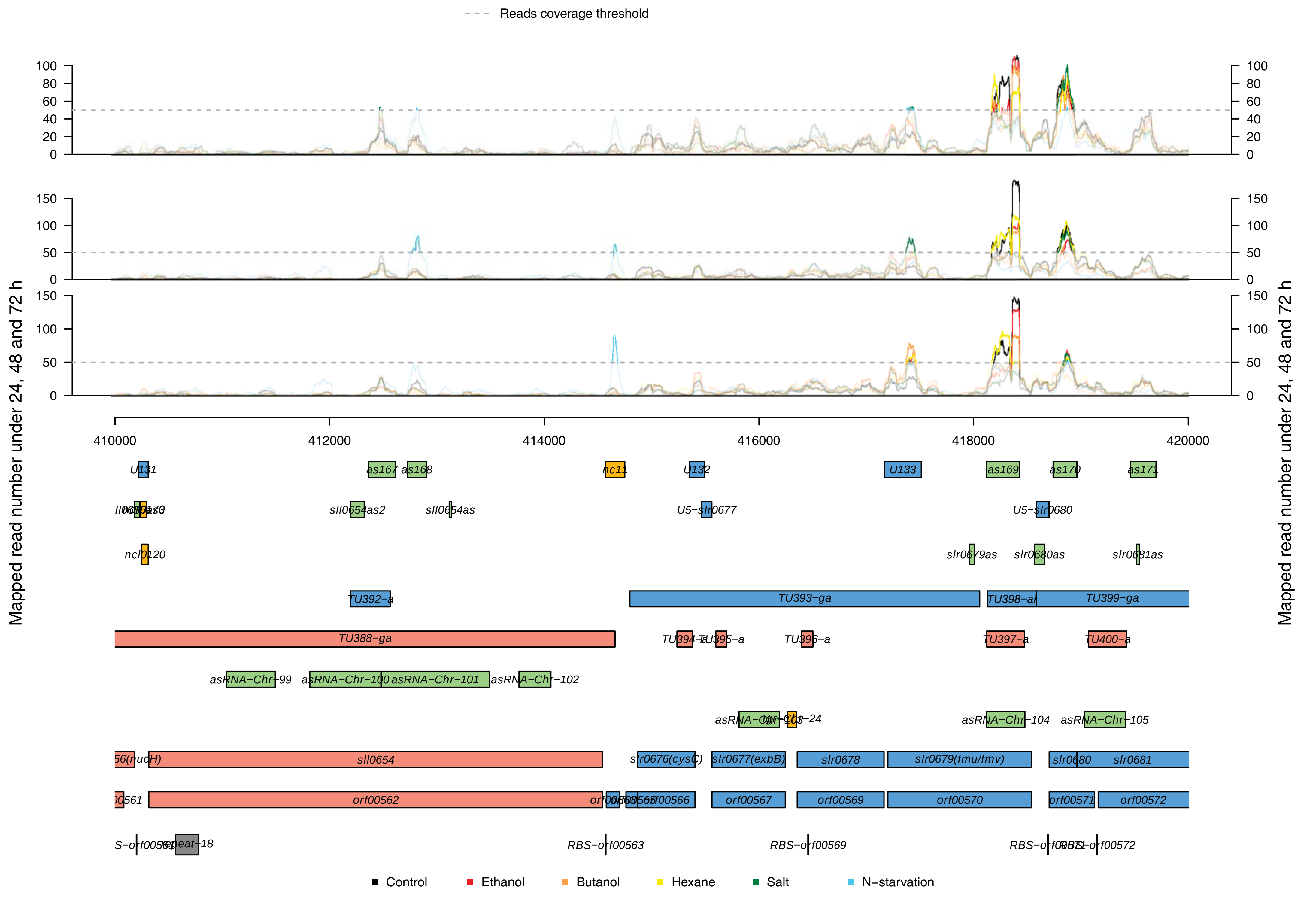

Reads coverage threshold
Mapped read number under 24, 48 and 72 h
410000
412000
414000
416000
418000
420000
U131
as167
as168
nc11
U132
U133
as169
as170
as171
U5−slr0677
U5−slr0680
ncl0120
slr0679as
slr0680as
slr0681as
TU392−a
TU393−ga
TU399−ga
TU388−ga
TU396−a
TU397−a
TU400−a
asRNA−Chr−99
asRNA−Chr−101
asRNA−Chr−102
asRNA−Chr−104
asRNA−Chr−105
sll0654
slr0676(cysC)
slr0677(exbB)
slr0678
slr0679(fmu/fmv)
slr0680
slr0681
orf00562
orf00566
orf00567
orf00569
orf00570
orf00571
orf00572
RBS−orf00563
RBS−orf00569
Control
Ethanol
Butanol
Hexane
Salt
N-starvation

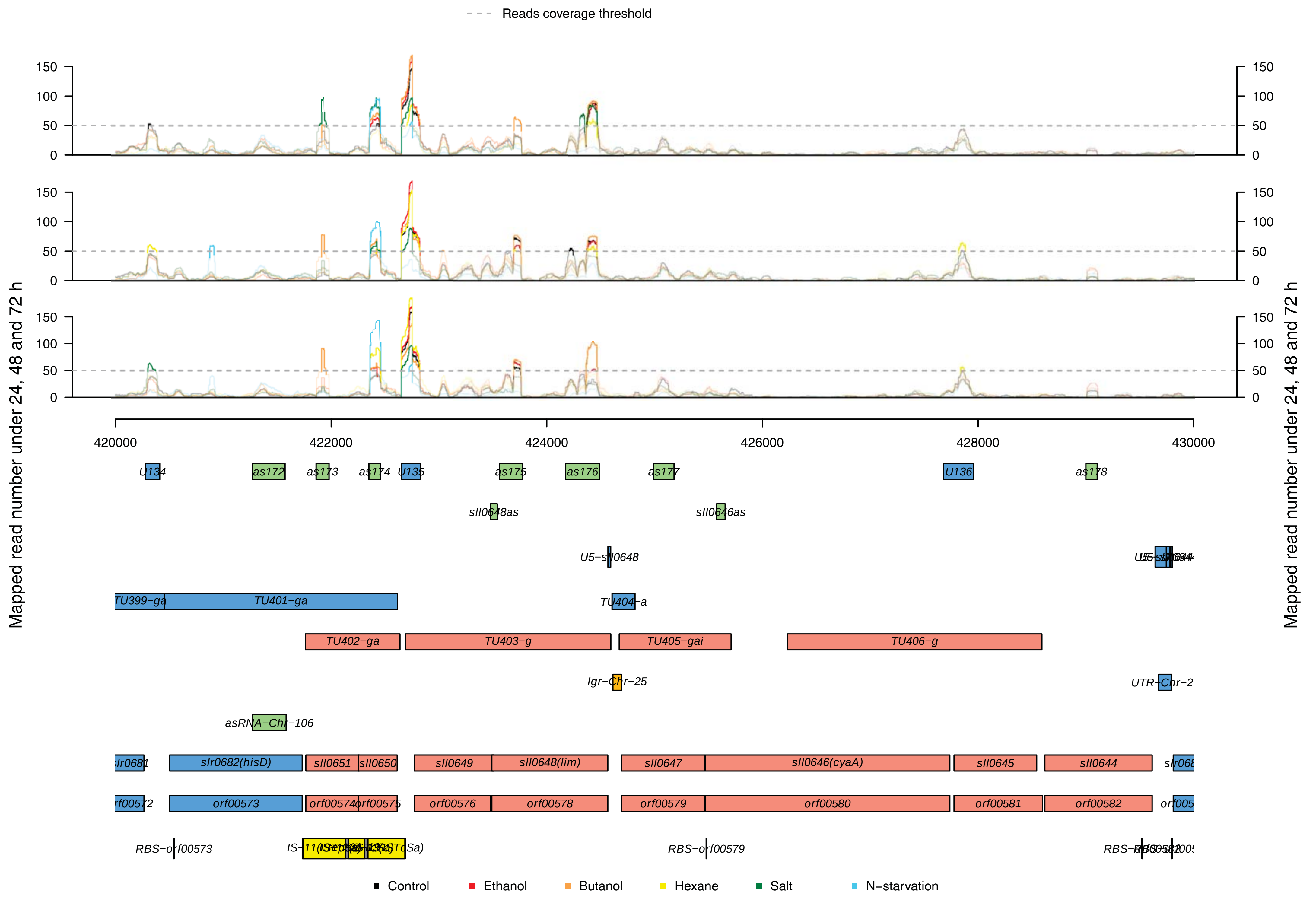

Reads coverage threshold
Mapped read number under 24, 48 and 72 h
420000
422000
424000
426000
428000
430000
U134
as172
as173
as174
U135
as175
as176
as177
U136
as178
sll0648as
sll0646as
U5−sll0648
TU399−ga
TU401−ga
TU404−a
TU402−ga
TU403−g
TU405−gai
TU406−g
Igr−Chr−25
UTR−Chr−2
asRNA−Chr−106
slr0682(hisD)
sll0651
sll0650
sll0649
sll0648(lim)
sll0647
sll0646(cyaA)
sll0645
sll0644
orf00573
orf00574
orf00575
orf00576
orf00578
orf00579
orf00580
orf00581
orf00582
RBS−orf00573
RBS−orf00579
Control
Ethanol
Butanol
Hexane
Salt
N−starvation

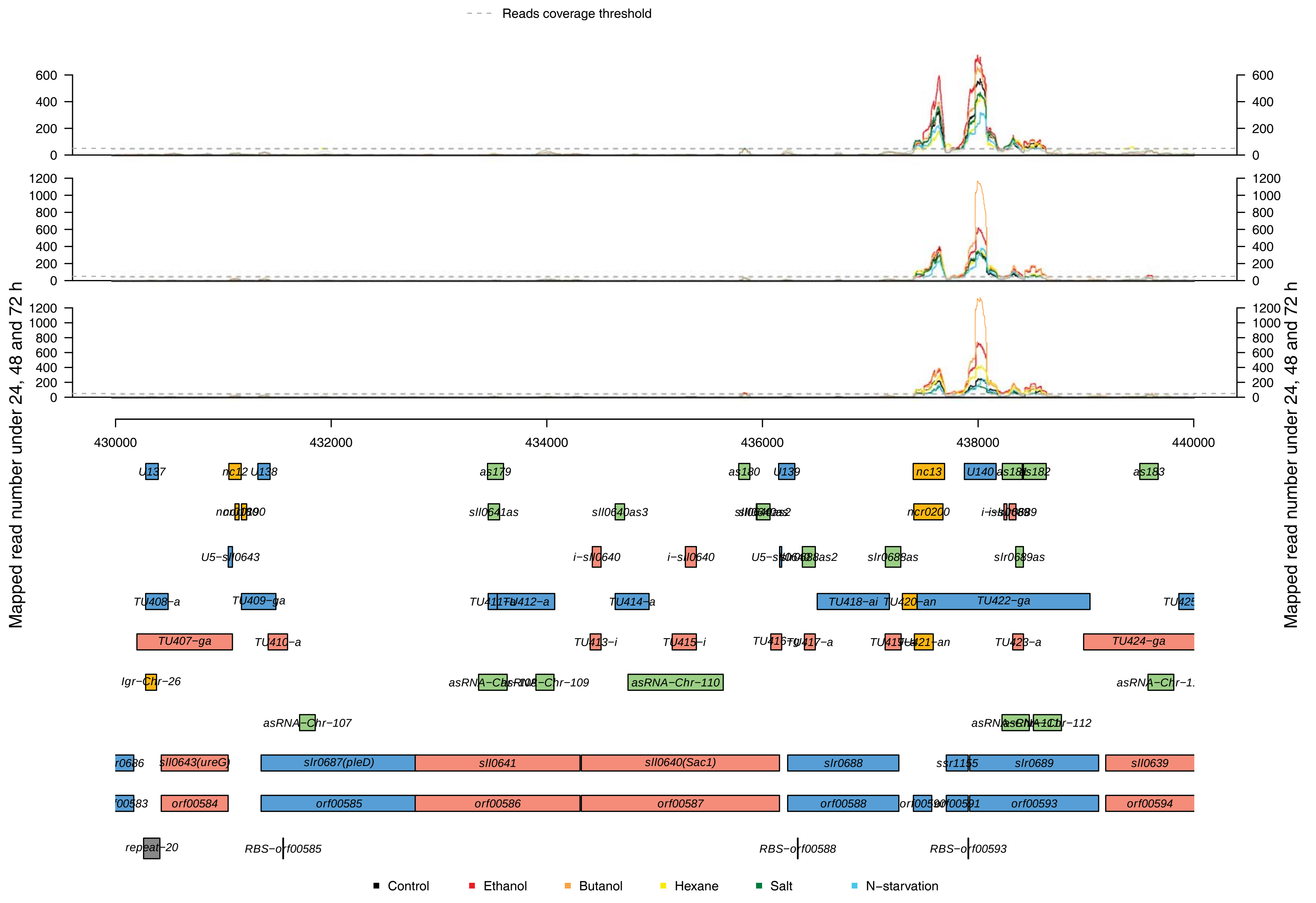

Reads coverage threshold
Mapped read number under 24, 48 and 72 h
430000
432000
434000
436000
438000
440000
nc13
TU407−ga
TU418−ai
TU422−ga
TU424−ga
asRNA−Chr−110
asRNA−Chr−107
sll0643(ureG)
slr0687(pleD)
sll0641
sll0640(Sac1)
slr0688
slr0689
sll0639
orf00584
orf00585
orf00586
orf00587
orf00588
orf00593
orf00594
RBS−orf00585
RBS−orf00588
RBS−orf00593
Control
Ethanol
Butanol
Hexane
Salt
N−starvation

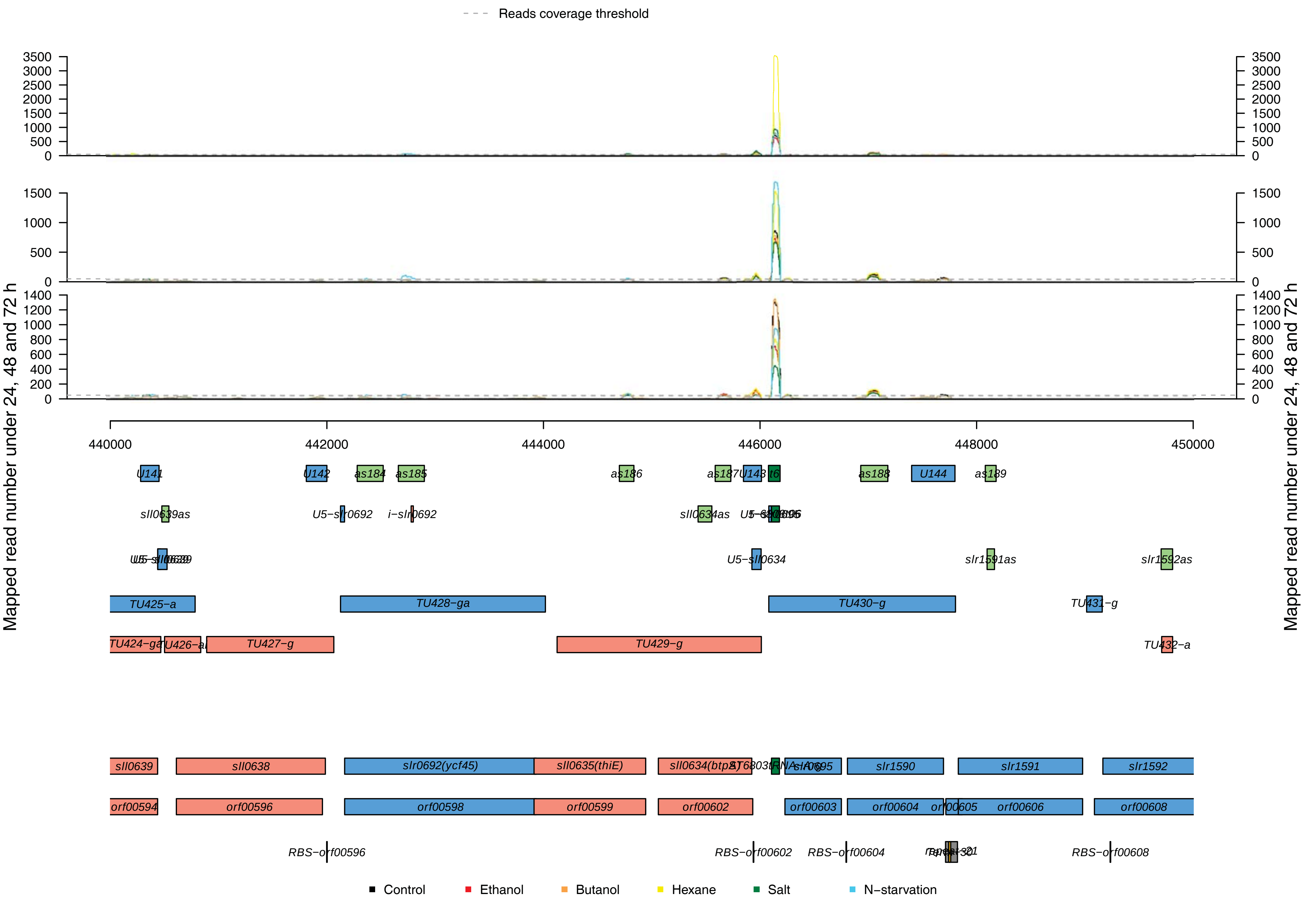

Reads coverage threshold
Mapped read number under 24, 48 and 72 h
440000
442000
444000
446000
448000
450000
U141
U142
as184
as185
as186
as187
U143
as188
U144
as189
sll0639as
U5−slr0692
i−slr0692
sll0634as
U5−sll0639
U5−sll0634
slr1591as
slr1592as
TU425−a
TU428−ga
TU430−g
TU431−g
TU424−ga
TU426−a
TU427−g
TU429−g
TU432−a
sll0639
sll0638
slr0692(ycf45)
sll0635(thiE)
sll0634(btpA)
slr0695
slr1590
slr1591
slr1592
orf00594
orf00596
orf00598
orf00599
orf00602
orf00603
orf00604
orf00605
orf00606
orf00608
RBS−orf00596
RBS−orf00602
RBS−orf00604
RBS−orf00608
Control
Ethanol
Butanol
Hexane
Salt
N−starvation

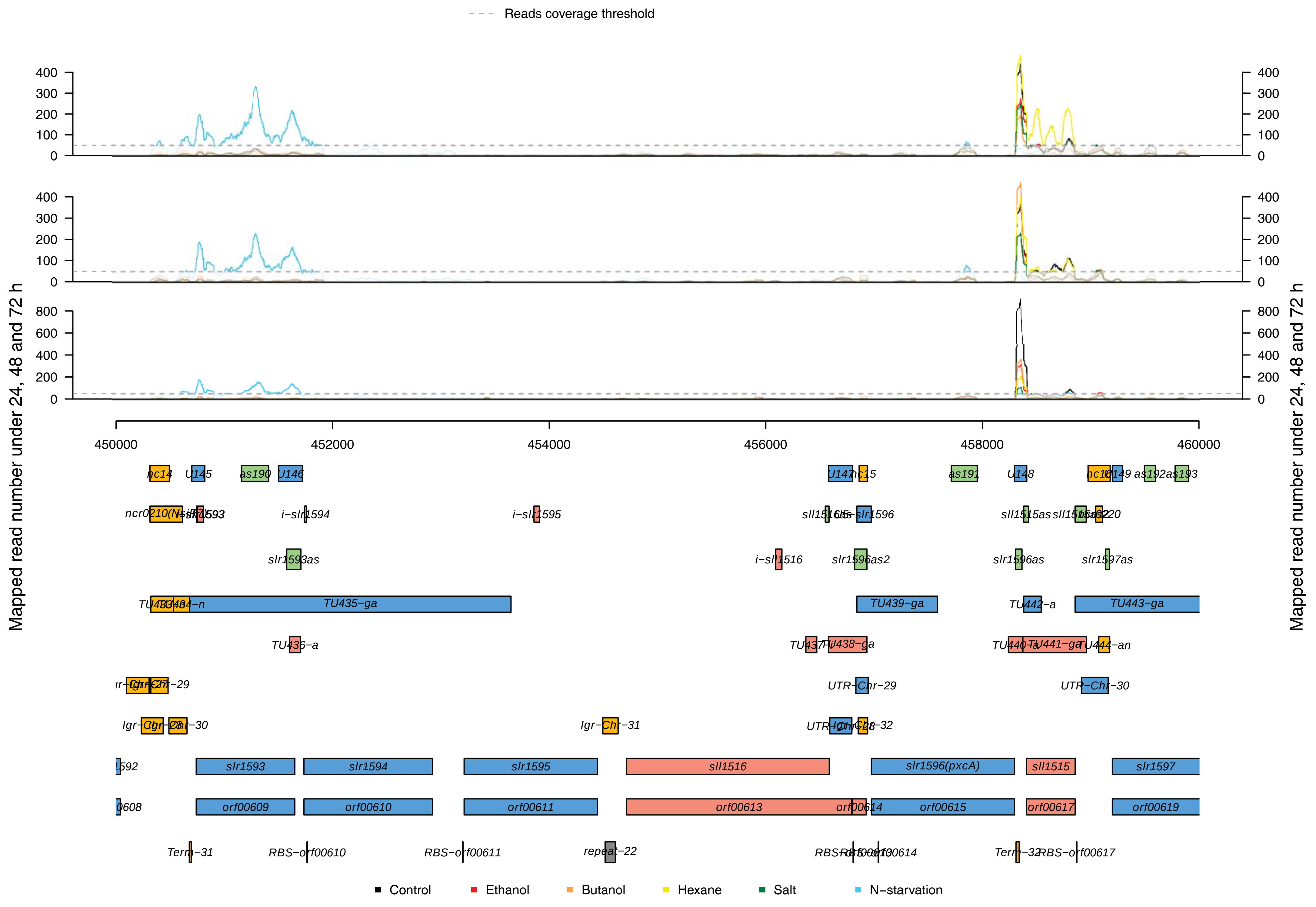

Reads coverage threshold
Mapped read number under 24, 48 and 72 h
450000
452000
454000
456000
458000
460000
TU435–ga
TU439–ga
TU443–ga
slr1593
slr1594
slr1595
sll1516
slr1596(pxcA)
sll1515
slr1597
orf00609
orf00610
orf00611
orf00613
orf00615
orf00617
orf00619
Control
Ethanol
Butanol
Hexane
Salt
N–starvation

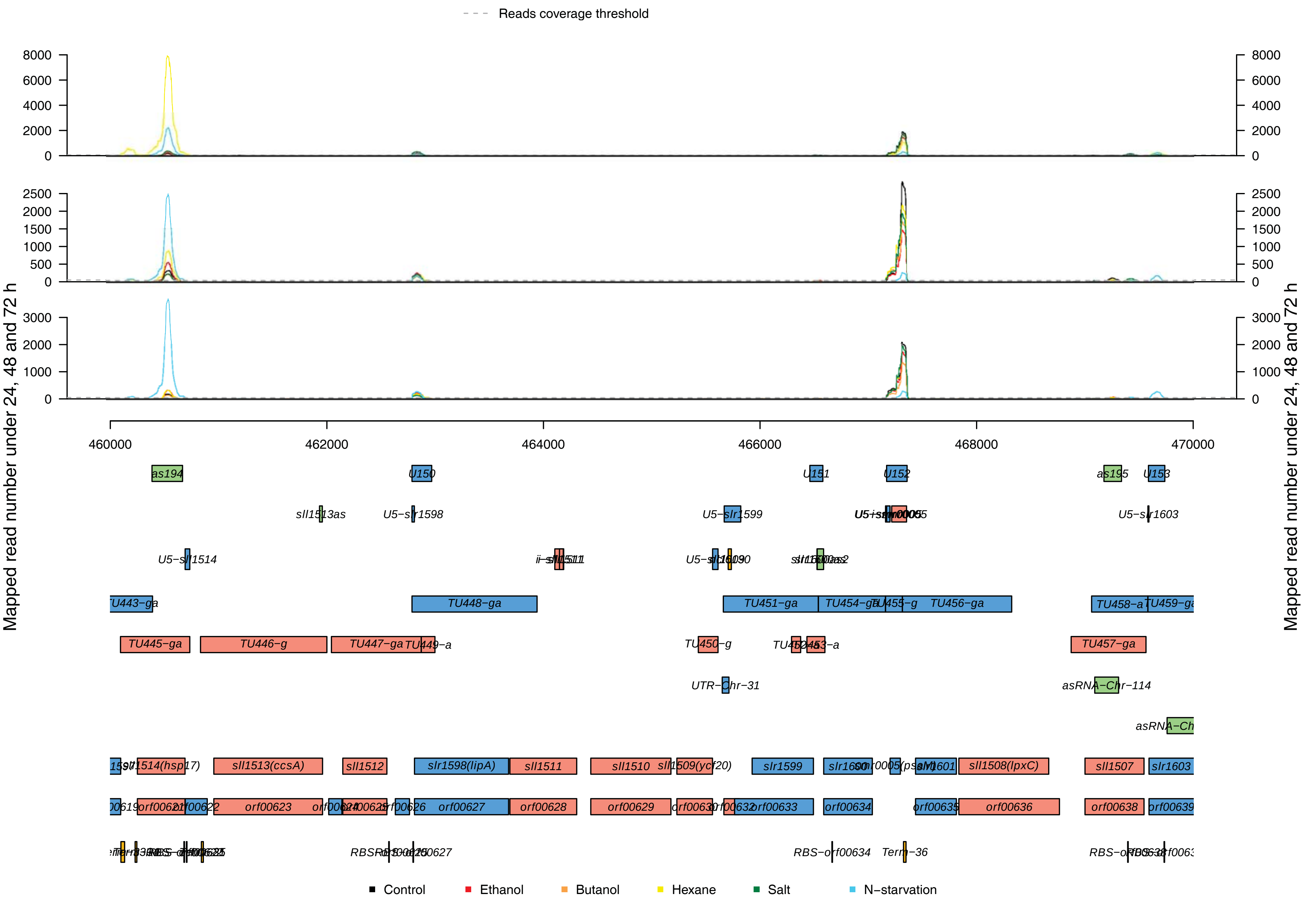

Reads coverage threshold
Mapped read number under 24, 48 and 72 h
460000
462000
464000
466000
468000
470000
as194
U150
U151
U152
as195
U153
sll1513as
U5−slr1598
U5−slr1599
U5−slr1603
U5−sll1514
TU443−ga
TU448−ga
TU451−ga
TU456−ga
TU458−a
TU445−ga
TU446−g
TU447−ga
TU457−ga
UTR−Chr−31
asRNA−Chr−114
sll1514(hsp17)
sll1513(ccsA)
sll1512
slr1598(lipA)
sll1511
sll1510
slr1599
sll1508(lpxC)
sll1507
slr1603
orf00623
orf00627
orf00628
orf00629
orf00633
orf00634
orf00636
orf00638
orf00639
RBS−orf00634
Term−36
Control
Ethanol
Butanol
Hexane
Salt
N−starvation

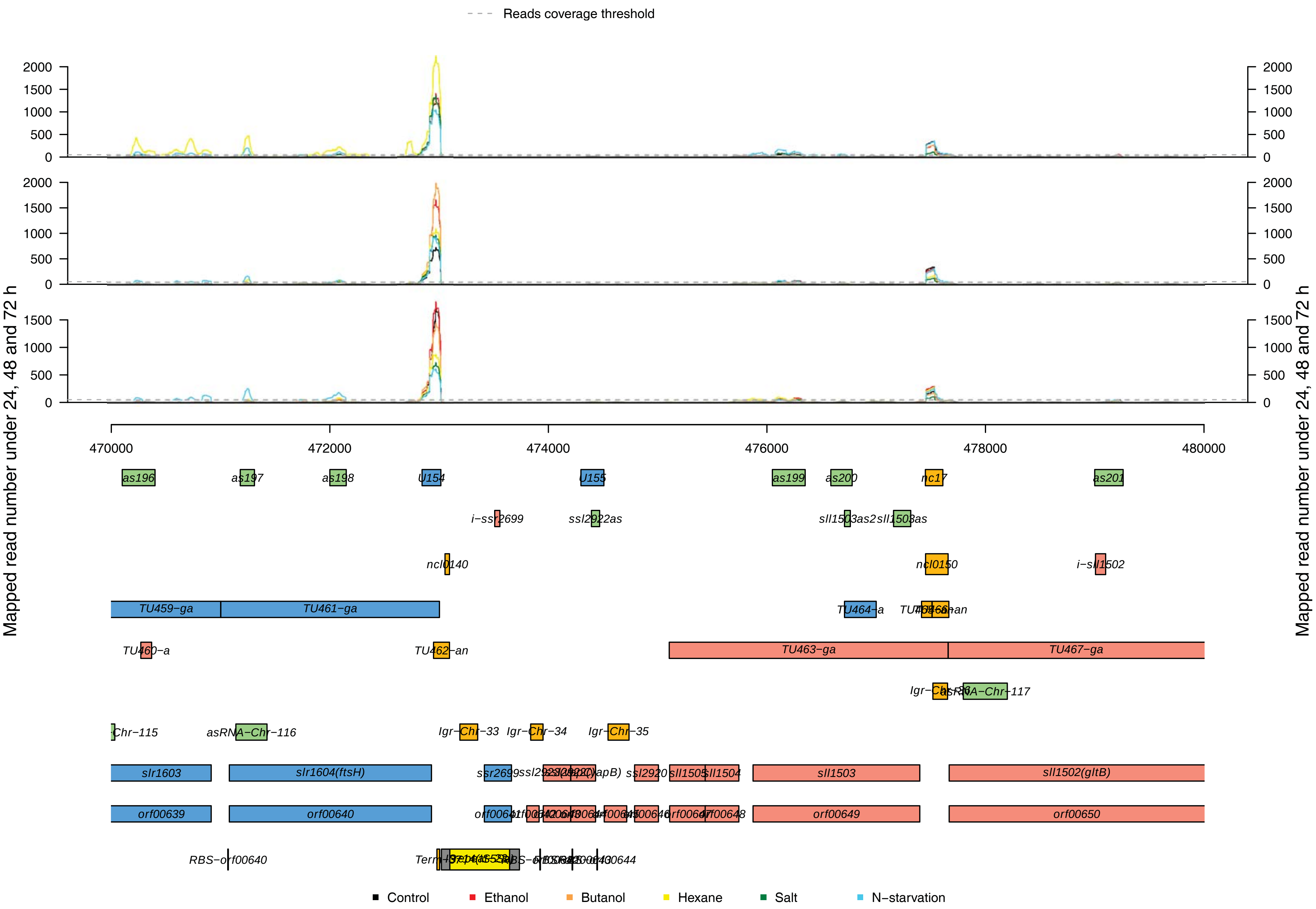

Reads coverage threshold
2000
1500
1000
500
0
Mapped read number under 24, 48 and 72 h
470000
472000
474000
476000
478000
480000
as196
as198
U154
U155
as199
as201
i−ssr2699
ssl2922as
ncl0140
ncl0150
TU459−ga
TU461−ga
TU460−a
TU462−an
TU463−ga
TU467−ga
asRNA−Chr−116
Igr−Chr−33
Igr−Chr−34
Igr−Chr−35
slr1603
slr1604(ftsH)
ssr2699
sll1503
sll1502(gltB)
orf00639
orf00640
orf00649
orf00650
RBS−orf00640
Control
Ethanol
Butanol
Hexane
Salt
N−starvation

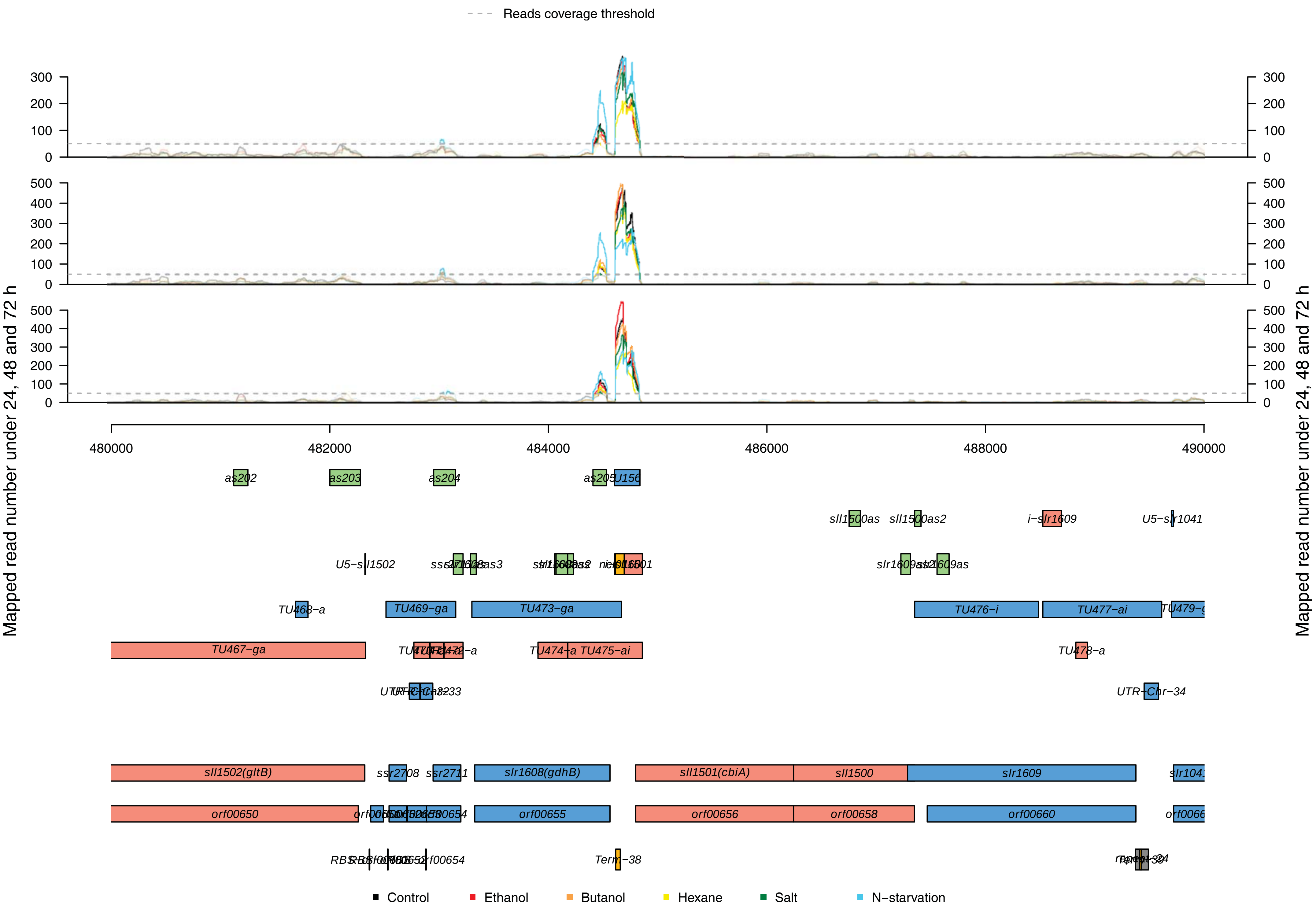
Reads coverage threshold
Mapped read number under 24, 48 and 72 h
300
200
100
0
500
400
300
200
100
0
480000
482000
484000
486000
488000
490000
as202
as203
as204
as205
U156
sll1500as
sll1500as2
i-slr1609
U5-slr1041
U5-sll1502
slr1609as
TU468-a
TU469-ga
TU473-ga
TU476-i
TU477-ai
TU467-ga
TU474-a
TU475-ai
TU478-a
UTR-Chr-34
sll1502(gltB)
ssr2708
ssr2711
slr1608(gdhB)
sll1501(cbiA)
sll1500
slr1609
orf00650
orf00655
orf00656
orf00658
orf00660
Term-38
Control
Ethanol
Butanol
Hexane
Salt
N-starvation

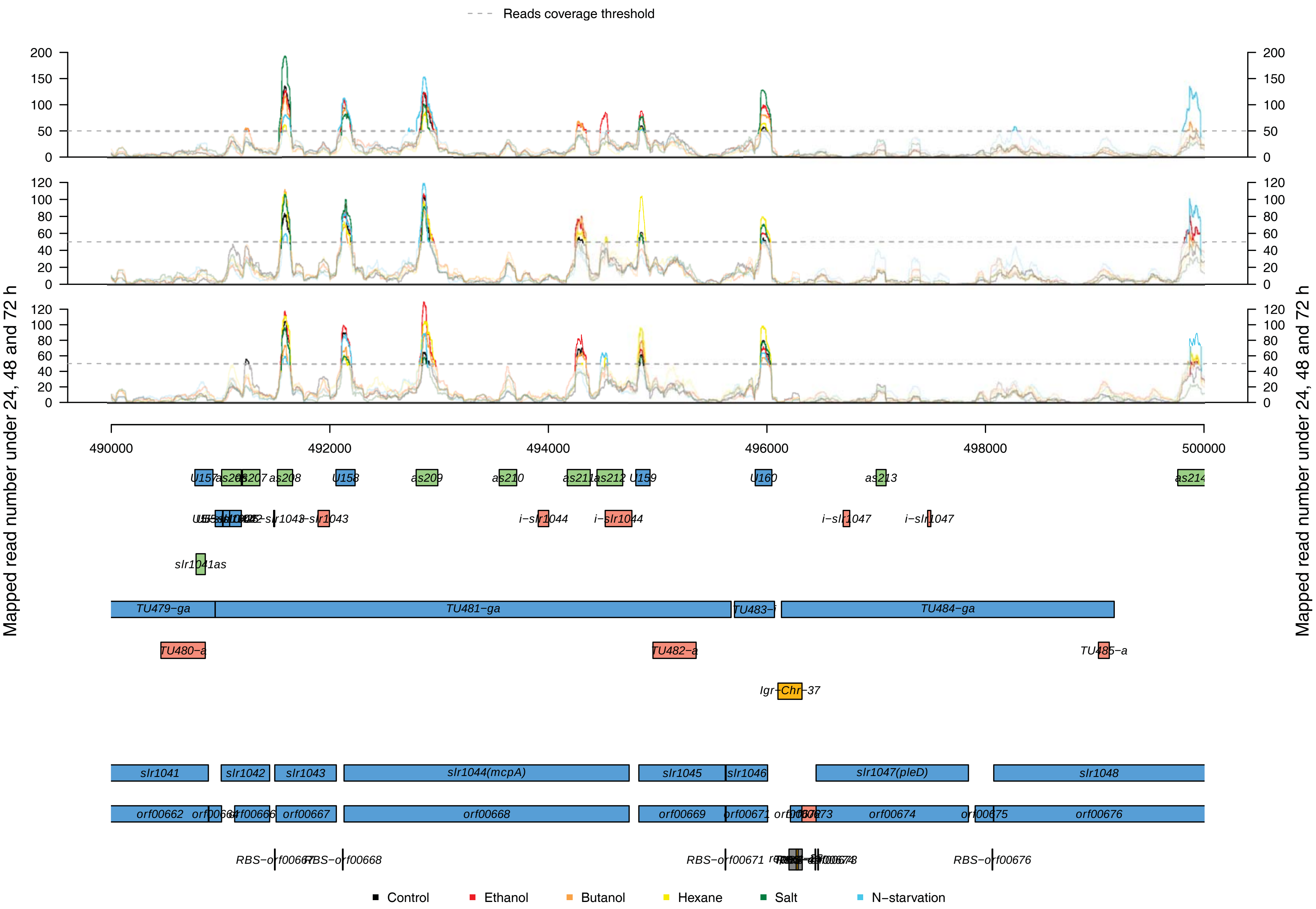

Reads coverage threshold
Mapped read number under 24, 48 and 72 h
490000
492000
494000
496000
498000
500000
U157
as207
as208
U158
as209
as210
as212
U159
U160
as213
as214
i−slr1043
i−slr1044
i−slr1047
slr1041as
TU479−ga
TU481−ga
TU484−ga
TU480−a
TU482−a
TU485−a
slr1041
slr1042
slr1043
slr1044(mcpA)
slr1045
slr1046
slr1047(pleD)
slr1048
orf00662
orf00667
orf00668
orf00669
orf00671
orf00674
orf00676
RBS−orf00668
RBS−orf00671
RBS−orf00676
Control
Ethanol
Butanol
Hexane
Salt
N−starvation

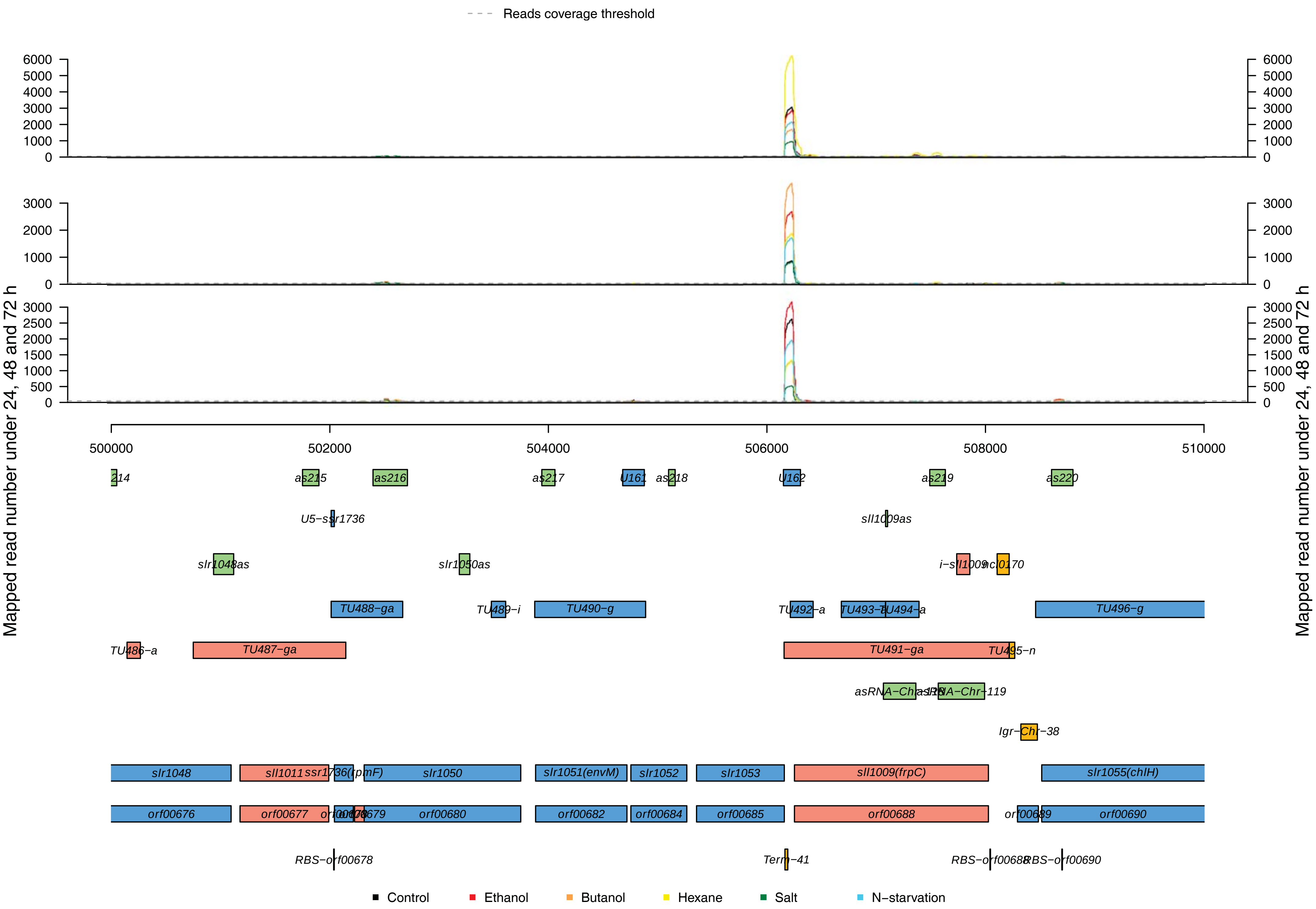

Reads coverage threshold
Mapped read number under 24, 48 and 72 h
500000
502000
504000
506000
508000
510000
as215
as216
as217
U161
U162
as219
U5−ssr1736
sll1009as
slr1048as
slr1050as
TU488−ga
TU490−g
TU496−g
TU487−ga
TU491−ga
Igr−Chr−38
slr1048
sll1011
slr1050
slr1051(envM)
slr1052
slr1053
sll1009(frpC)
slr1055(chlH)
orf00676
orf00677
orf00680
orf00682
orf00684
orf00685
orf00688
orf00690
RBS−orf00678
Term−41
Control
Ethanol
Butanol
Hexane
Salt
N−starvation

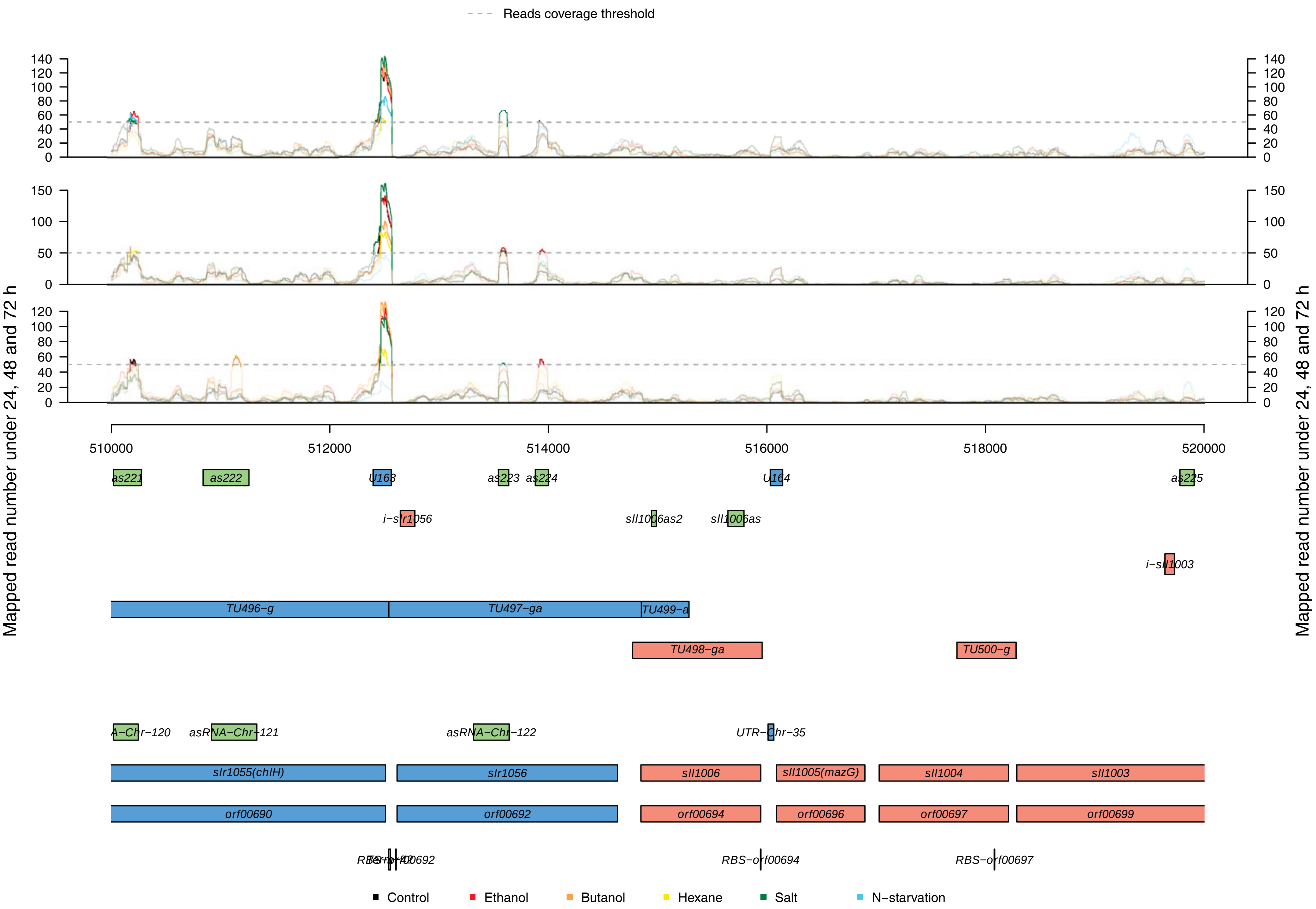

Reads coverage threshold
Mapped read number under 24, 48 and 72 h
140
120
100
80
60
40
20
0
150
100
50
0
510000
512000
514000
516000
518000
520000
as221
as222
U163
as223
as224
U164
as225
i−slr1056
sll1006as2
sll1006as
i−sll1003
TU496−g
TU497−ga
TU499−a
TU498−ga
TU500−g
A−Chr−120
asRNA−Chr−121
asRNA−Chr−122
UTR−Chr−35
slr1055(chlH)
slr1056
sll1006
sll1005(mazG)
sll1004
sll1003
orf00690
orf00692
orf00694
orf00696
orf00697
orf00699
RBS−orf00694
RBS−orf00697
Control
Ethanol
Butanol
Hexane
Salt
N−starvation

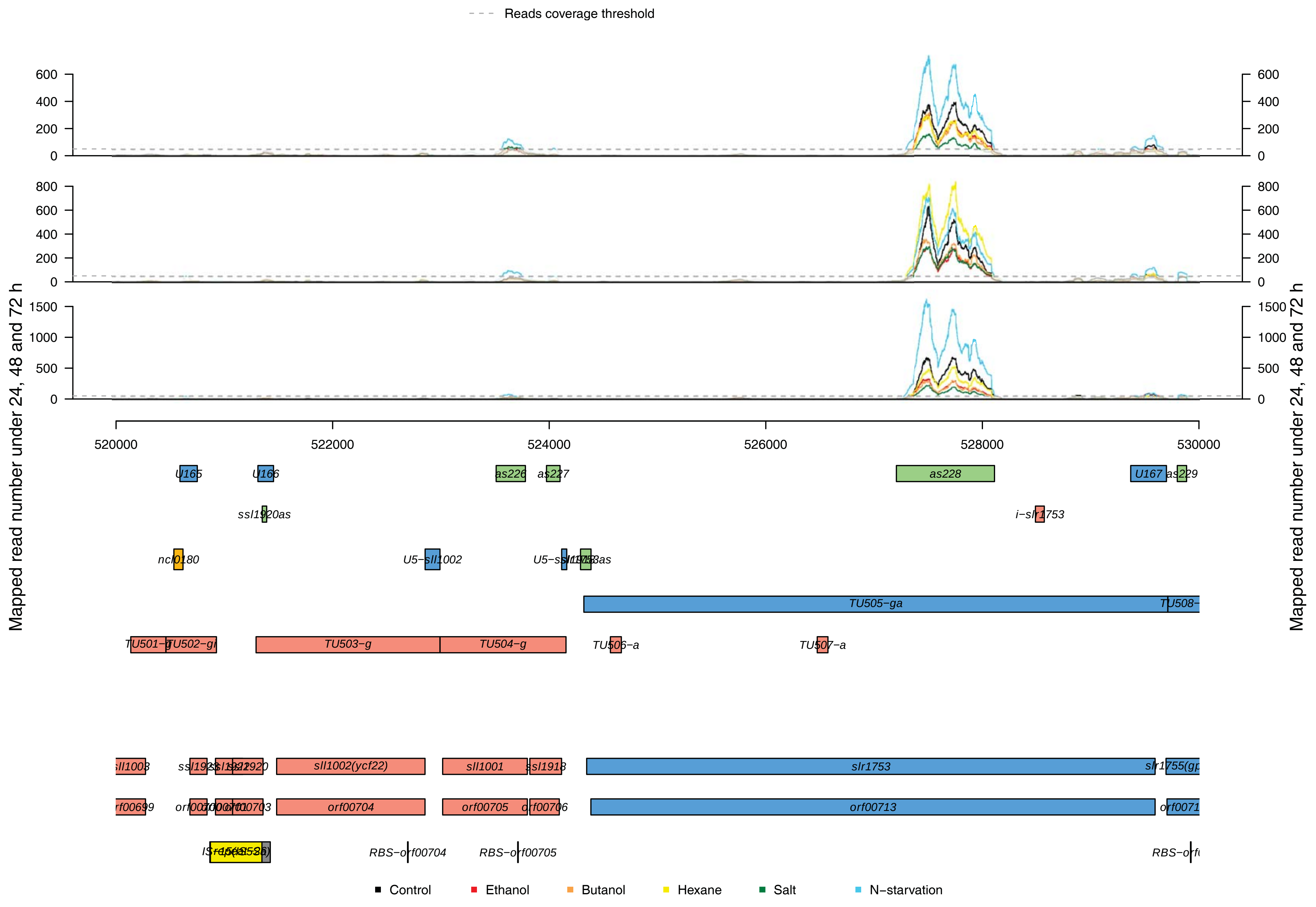

Reads coverage threshold
Mapped read number under 24, 48 and 72 h
520000
522000
524000
526000
528000
530000
as228
TU505–ga
slr1753
orf00713
sll1002(ycf22)
orf00704
sll1001
orf00705
TU503–g
TU504–g
Control
Ethanol
Butanol
Hexane
Salt
N–starvation

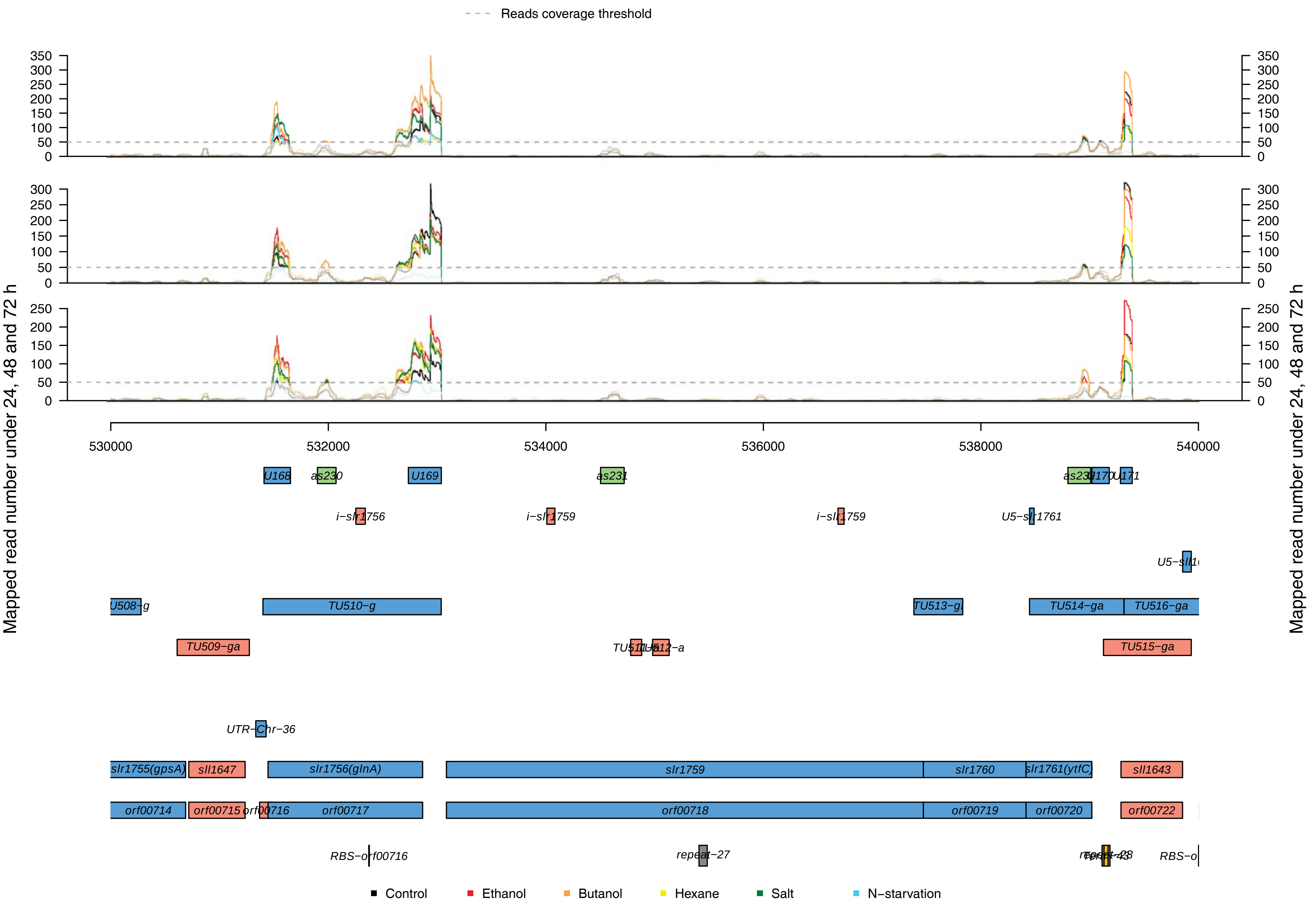
Reads coverage threshold
Mapped read number under 24, 48 and 72 h
530000
532000
534000
536000
538000
540000
U168
as230
U169
as231
i−slr1756
i−slr1759
U5−slr1761
TU510−g
TU513−g
TU514−ga
TU516−ga
TU509−ga
TU515−ga
UTR−Chr−36
slr1755(gpsA)
sll1647
slr1756(glnA)
slr1759
slr1760
slr1761(ytfC)
sll1643
orf00714
orf00715
orf00716
orf00717
orf00718
orf00719
orf00720
orf00722
RBS−orf00716
repeat−27
Control
Ethanol
Butanol
Hexane
Salt
N−starvation

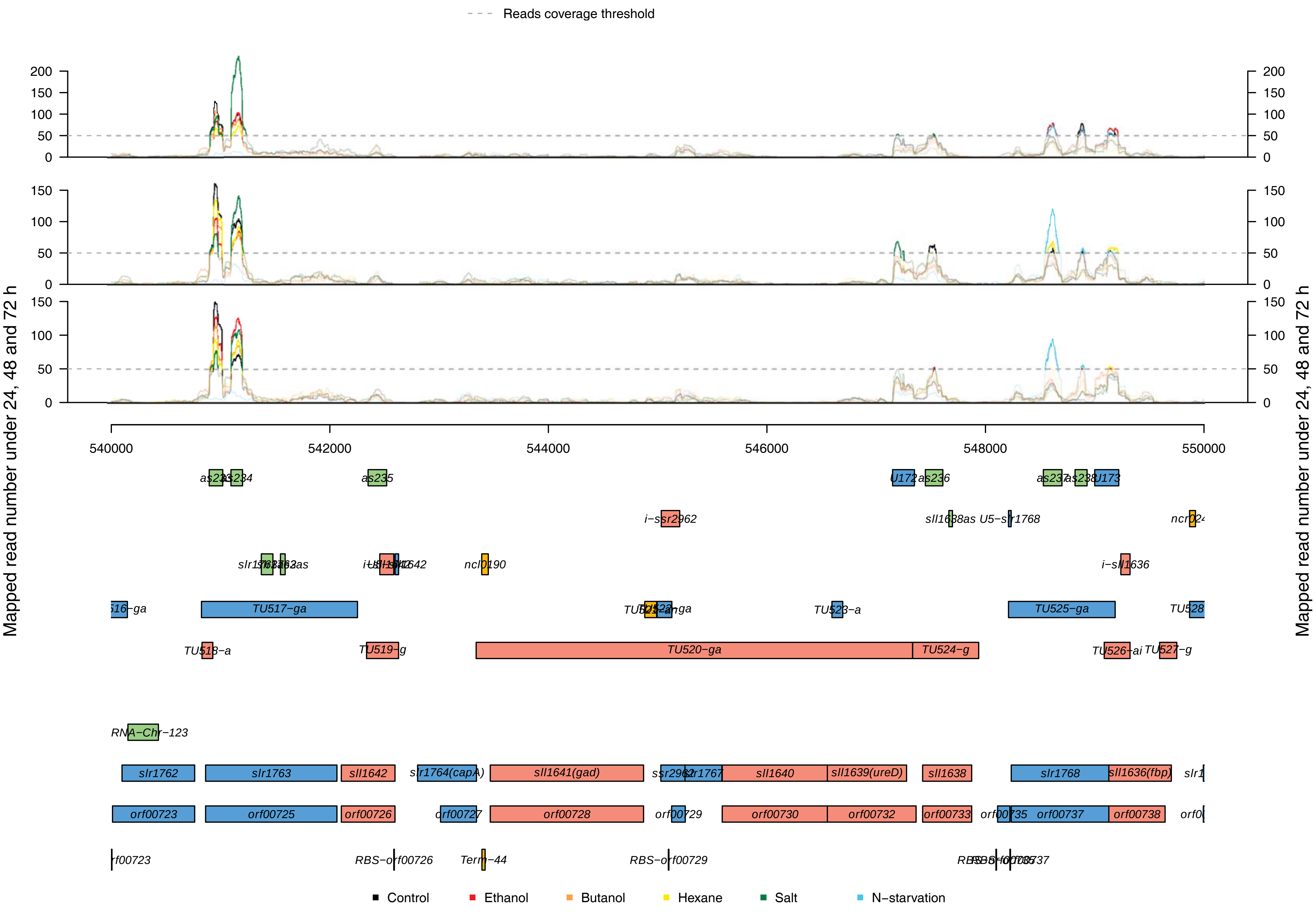
Reads coverage threshold
Mapped read number under 24, 48 and 72 h
540000
542000
544000
546000
548000
550000
TU517−ga
TU520−ga
TU524−g
TU525−ga
RNA−Chr−123
slr1762
slr1763
sll1642
slr1764(capA)
sll1641(gad)
sll1640
sll1639(ureD)
sll1638
slr1768
sll1636(fbp)
orf00723
orf00725
orf00726
orf00728
orf00730
orf00732
orf00733
orf00737
orf00738
Term−44
Control
Ethanol
Butanol
Hexane
Salt
N−starvation

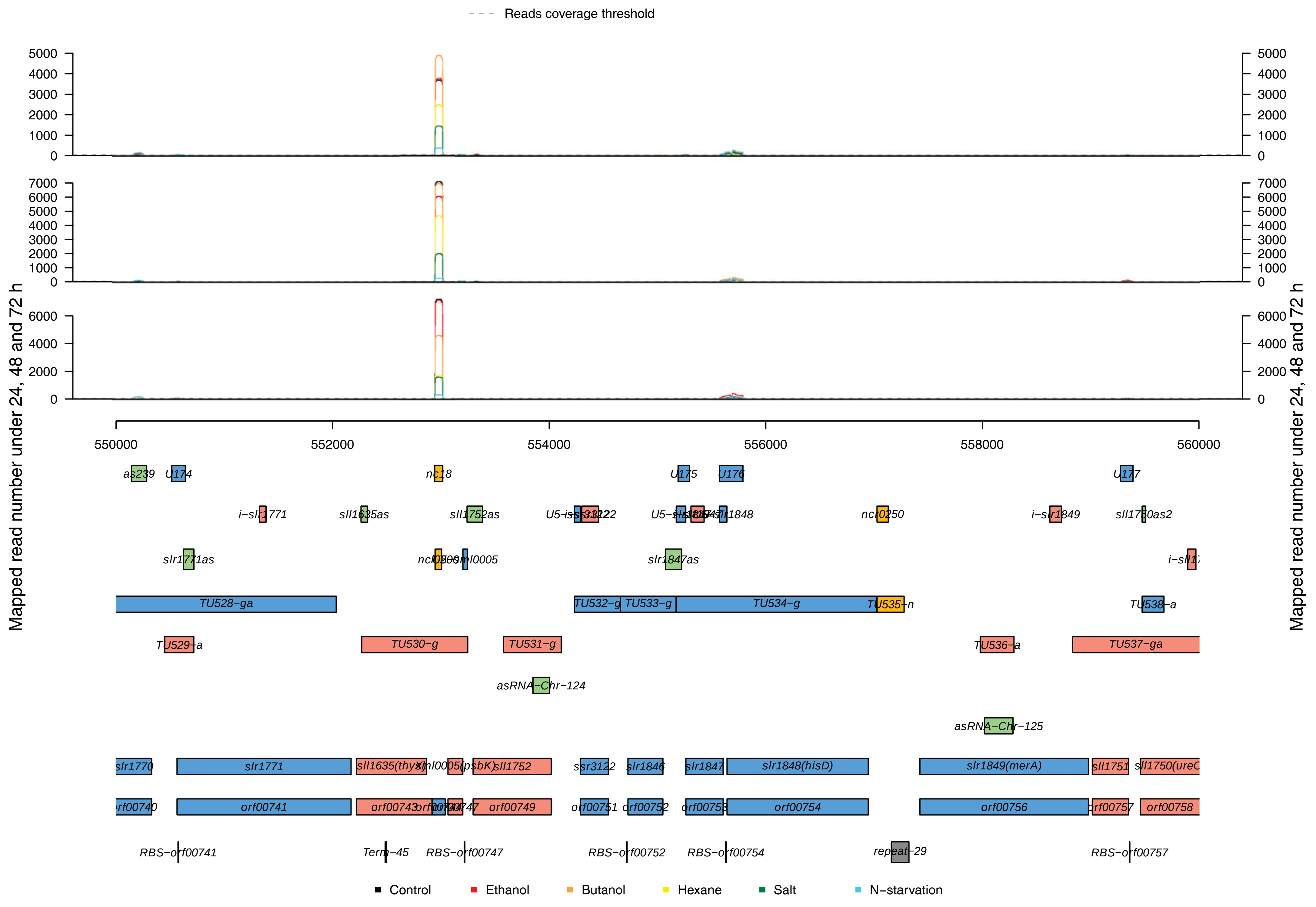

Reads coverage threshold
Mapped read number under 24, 48 and 72 h
550000
552000
554000
556000
558000
560000
as239
U174
nc18
U175
U176
U177
i−slr1771
sll1635as
sll1752as
ncl0250
i−slr1849
slr1771as
slr1847as
TU528−ga
TU532−g
TU533−g
TU534−g
TU535−n
TU538−a
TU529−a
TU530−g
TU531−g
TU536−a
TU537−ga
asRNA−Chr−124
asRNA−Chr−125
slr1771
sll1752
ssr3122
slr1846
slr1847
slr1848(hisD)
slr1849(merA)
sll1751
orf00741
orf00743
orf00749
orf00752
orf00754
orf00756
orf00758
RBS−orf00741
Term−45
RBS−orf00747
RBS−orf00752
RBS−orf00754
repeat−29
RBS−orf00757
Control
Ethanol
Butanol
Hexane
Salt
N−starvation

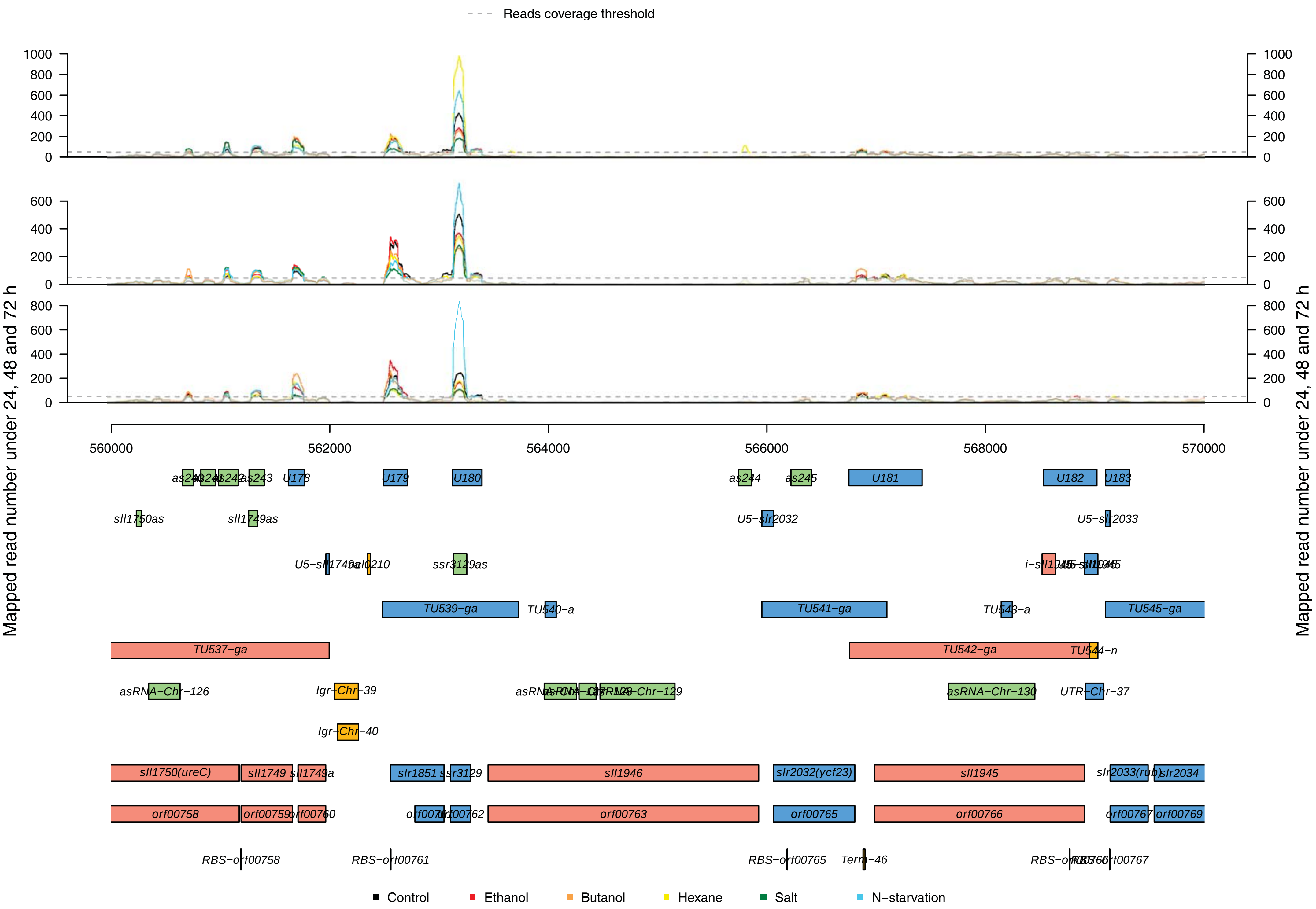

Reads coverage threshold
Mapped read number under 24, 48 and 72 h
1000
800
600
400
200
0
560000
562000
564000
566000
568000
570000
U178
U179
U180
as244
U181
U182
U183
sll1750as
sll1749as
U5−slr2032
U5−slr2033
ssr3129as
TU539−ga
TU540−a
TU541−ga
TU543−a
TU545−ga
TU537−ga
TU542−ga
TU544−n
asRNA−Chr−126
Igr−Chr−39
asRNA−Chr−130
UTR−Chr−37
Igr−Chr−40
sll1750(ureC)
sll1749
sll1749a
slr1851
ssr3129
sll1946
slr2032(ycf23)
sll1945
slr2034
orf00758
orf00759
orf00760
orf00763
orf00765
orf00766
orf00767
orf00769
RBS−orf00758
RBS−orf00761
RBS−orf00765
Term−46
Control
Ethanol
Butanol
Hexane
Salt
N−starvation

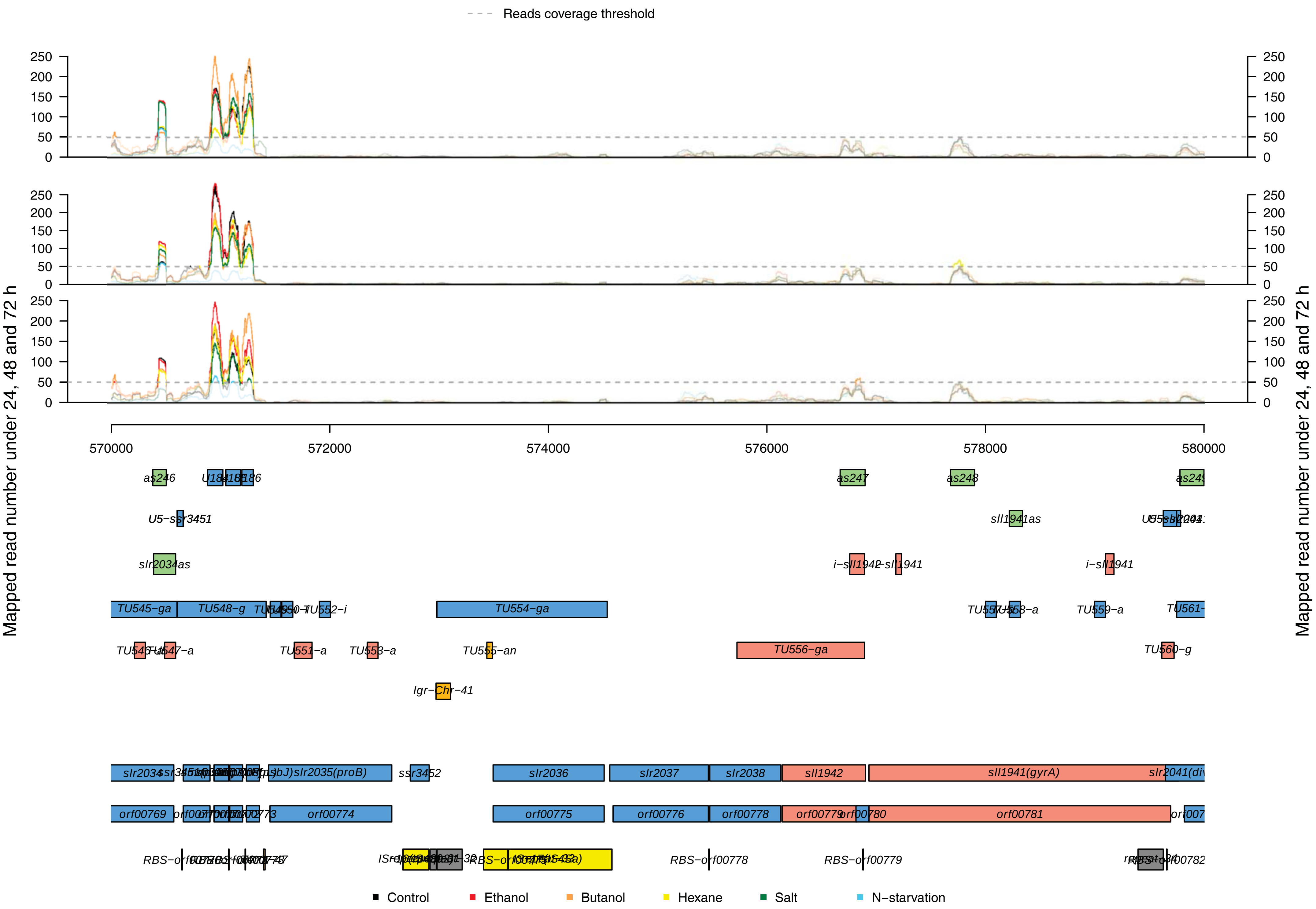

Reads coverage threshold
Mapped read number under 24, 48 and 72 h
250
200
150
100
50
0
570000
572000
574000
576000
578000
580000
as246
as247
as248
slr2034as
sll1941as
TU545−ga
TU548−g
TU552−i
TU554−ga
TU559−a
TU551−a
TU553−a
TU556−ga
TU560−g
Igr−Chr−41
slr2036
slr2037
slr2038
sll1942
sll1941(gyrA)
ssr3452
orf00769
orf00774
orf00775
orf00776
orf00778
orf00781
RBS−orf00778
RBS−orf00779
Control
Ethanol
Butanol
Hexane
Salt
N−starvation

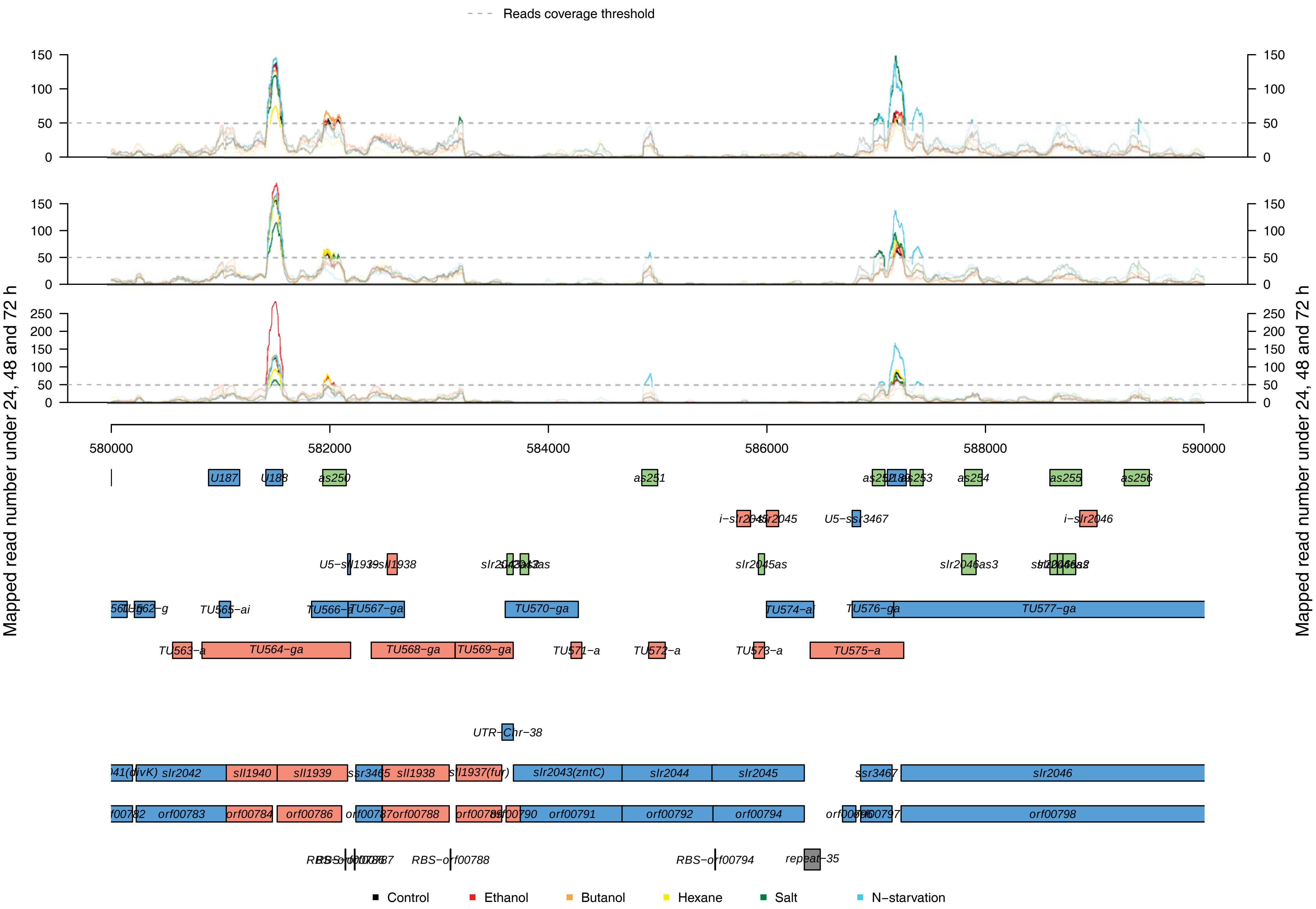

Reads coverage threshold
Mapped read number under 24, 48 and 72 h
580000
582000
584000
586000
588000
590000
U187
as250
as251
as254
as255
as256
ssr3467
slr2045as
slr2046as3
TU565–ai
TU567–ga
TU570–ga
TU574–ai
TU577–ga
TU564–ga
TU568–ga
TU569–ga
TU575–a
UTR–Chr–38
slr2042
sll1940
sll1939
sll1938
sll1937(fur)
slr2043(zntC)
slr2044
slr2045
slr2046
orf00783
orf00784
orf00786
orf00791
orf00792
orf00794
orf00798
RBS–orf00788
RBS–orf00794
repeat–35
Control
Ethanol
Butanol
Hexane
Salt
N–starvation

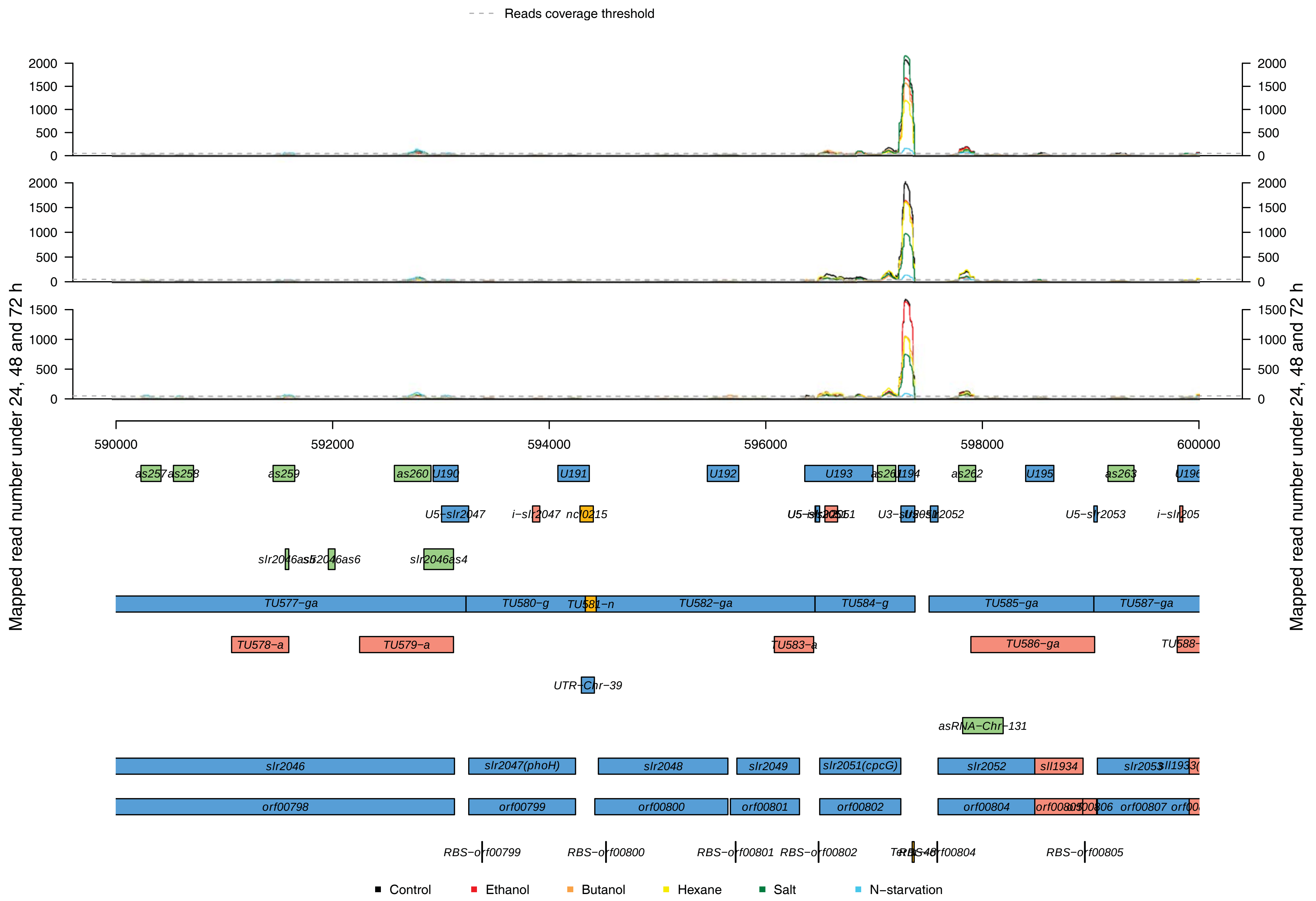
Reads coverage threshold
Mapped read number under 24, 48 and 72 h
590000
592000
594000
596000
598000
600000
TU577−ga
TU580−g
TU582−ga
TU584−g
TU585−ga
TU587−ga
TU578−a
TU579−a
TU586−ga
UTR−Chr−39
slr2046
slr2047(phoH)
slr2048
slr2049
slr2051(cpcG)
slr2052
sll1934
orf00798
orf00799
orf00800
orf00801
orf00802
orf00804
RBS−orf00799
RBS−orf00800
RBS−orf00801
RBS−orf00802
RBS−orf00805
Control
Ethanol
Butanol
Hexane
Salt
N−starvation

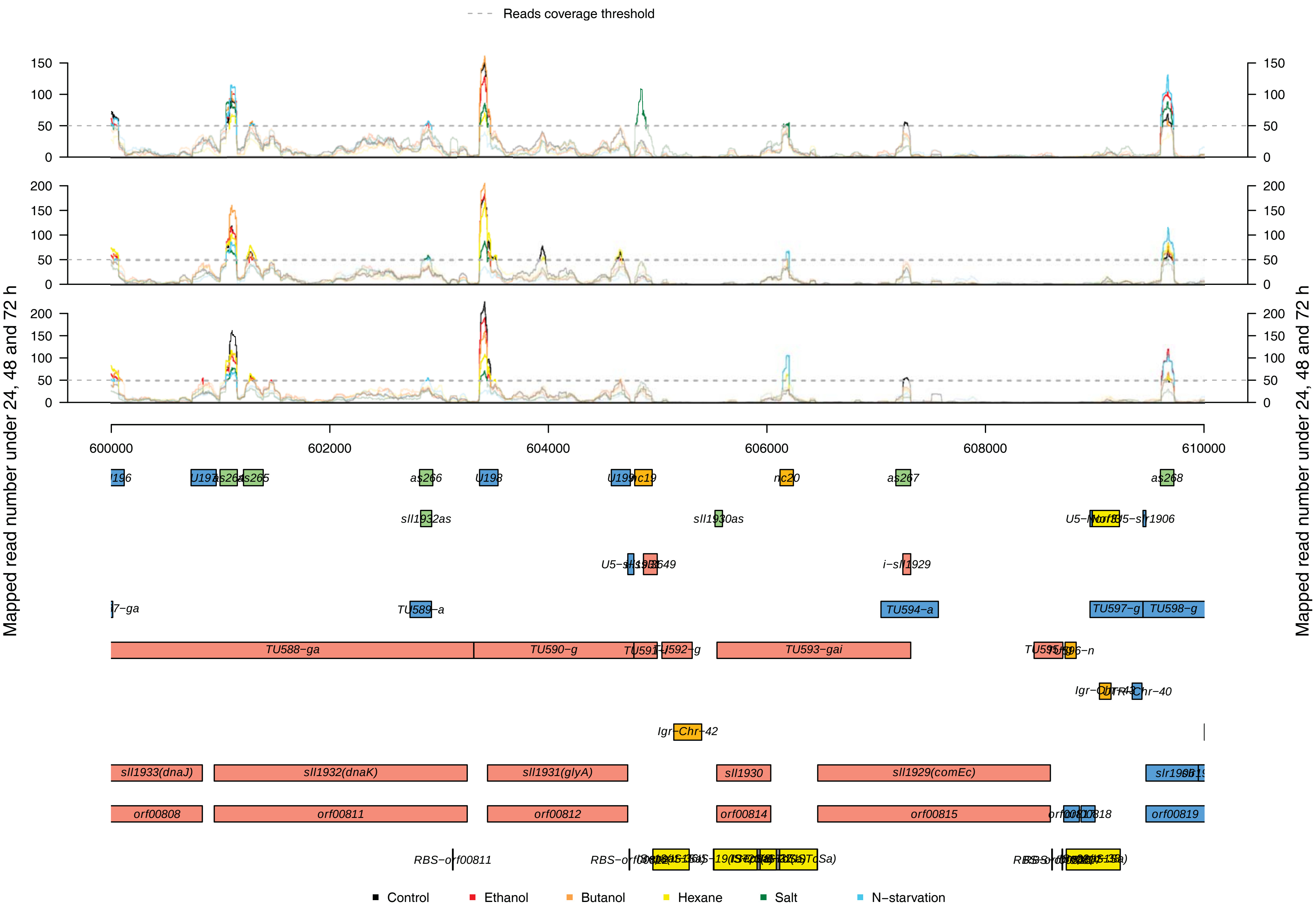

Reads coverage threshold
Mapped read number under 24, 48 and 72 h
600000
602000
604000
606000
608000
610000
as266
as267
as268
nc20
sll1932as
sll1930as
i−sll1929
TU589−a
TU594−a
TU597−g
TU598−g
TU588−ga
TU590−g
TU593−gai
Igr−Chr−42
sll1933(dnaJ)
sll1932(dnaK)
sll1931(glyA)
sll1930
sll1929(comEc)
orf00808
orf00811
orf00812
orf00814
orf00815
orf00819
RBS−orf00811
Control
Ethanol
Butanol
Hexane
Salt
N−starvation

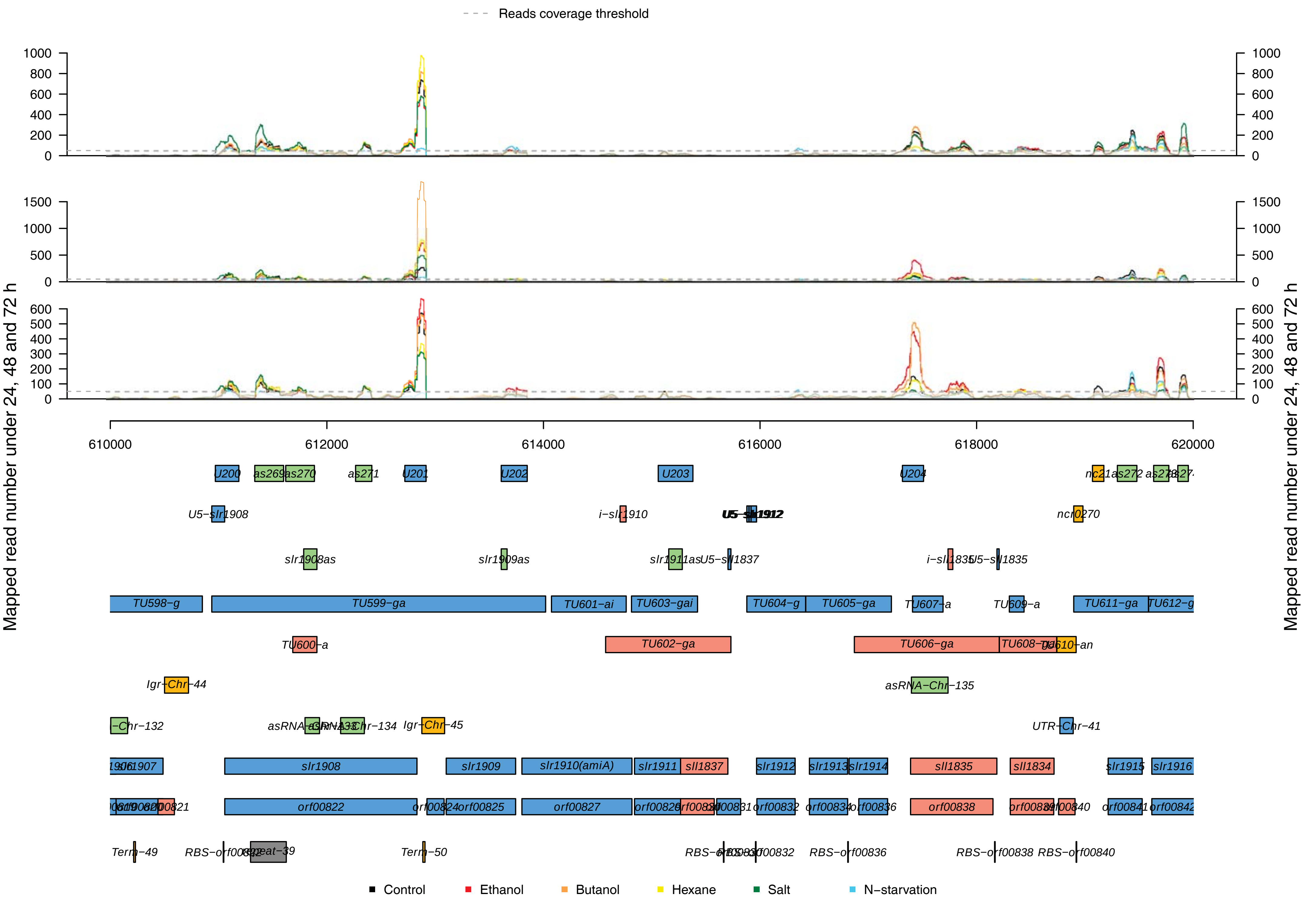

Reads coverage threshold
Mapped read number under 24, 48 and 72 h
610000
612000
614000
616000
618000
620000
U200
U201
U202
U203
U204
as271
U5−slr1908
i−slr1910
ncl0270
slr1908as
slr1909as
U5−sll1837
U5−sll1835
TU598−g
TU599−ga
TU601−ai
TU603−gai
TU604−g
TU605−ga
TU611−ga
TU600−a
TU602−ga
TU606−ga
Igr−Chr−44
asRNA−Chr−135
Igr−Chr−45
UTR−Chr−41
slr1908
slr1909
slr1910(amiA)
slr1911
sll1837
slr1912
sll1835
sll1834
slr1915
orf00822
orf00825
orf00827
orf00832
orf00838
orf00842
Term−49
Term−50
RBS−orf00836
RBS−orf00838
RBS−orf00840
Control
Ethanol
Butanol
Hexane
Salt
N−starvation

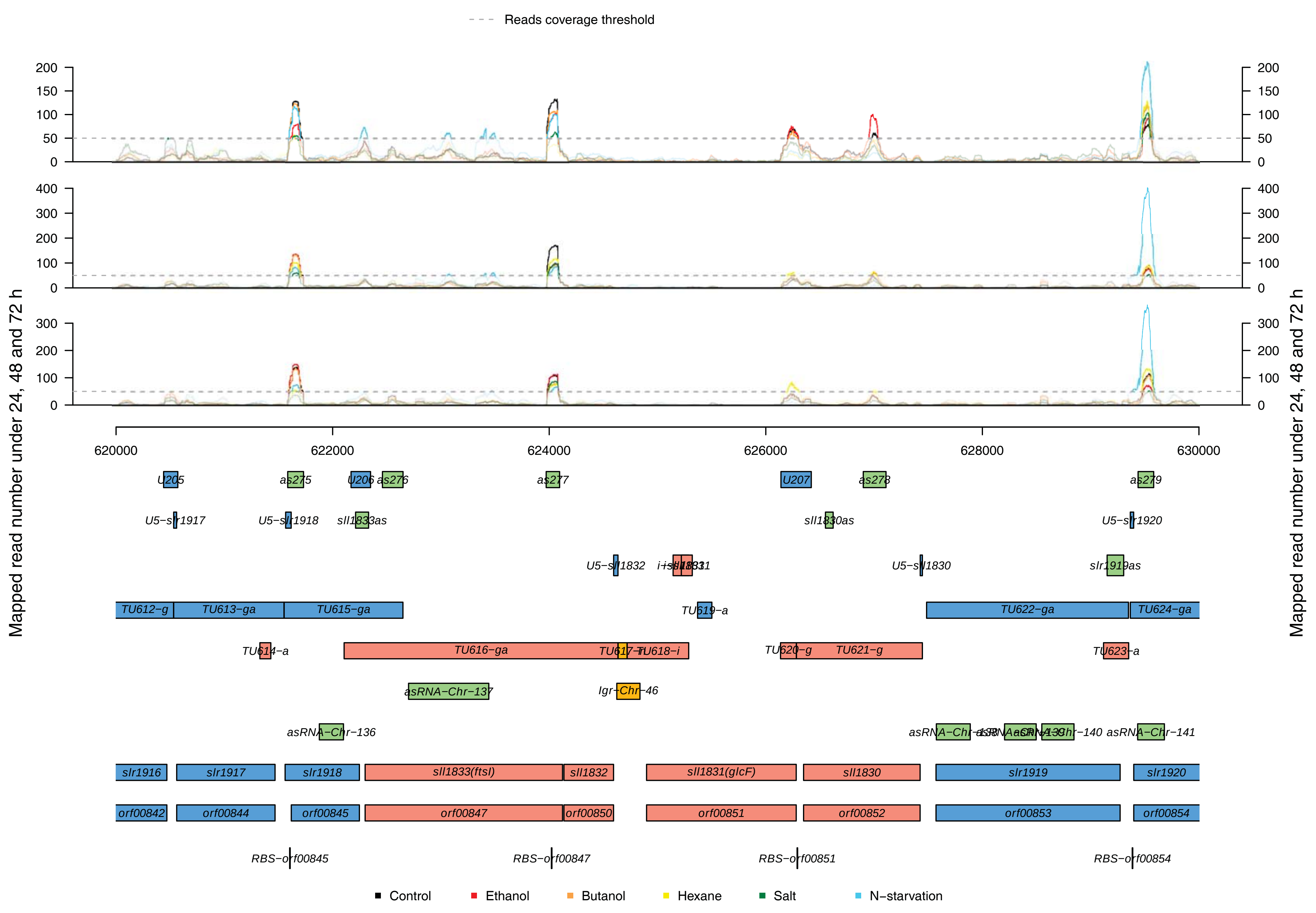

Reads coverage threshold
Mapped read number under 24, 48 and 72 h
620000
622000
624000
626000
628000
630000
U205
as275
U206
as276
as277
U207
as278
as279
U5−slr1917
U5−slr1918
sll1833as
sll1830as
U5−slr1920
U5−sll1832
U5−sll1830
slr1919as
TU612−g
TU613−ga
TU615−ga
TU619−a
TU622−ga
TU624−ga
TU614−a
TU616−ga
TU618−i
TU620−g
TU621−g
TU623−a
asRNA−Chr−137
Igr−Chr−46
asRNA−Chr−136
asRNA−Chr−140
asRNA−Chr−141
slr1916
slr1917
slr1918
sll1833(ftsI)
sll1832
sll1831(glcF)
sll1830
slr1919
slr1920
orf00842
orf00844
orf00845
orf00847
orf00850
orf00851
orf00852
orf00853
orf00854
RBS−orf00845
RBS−orf00847
RBS−orf00851
RBS−orf00854
Control
Ethanol
Butanol
Hexane
Salt
N−starvation

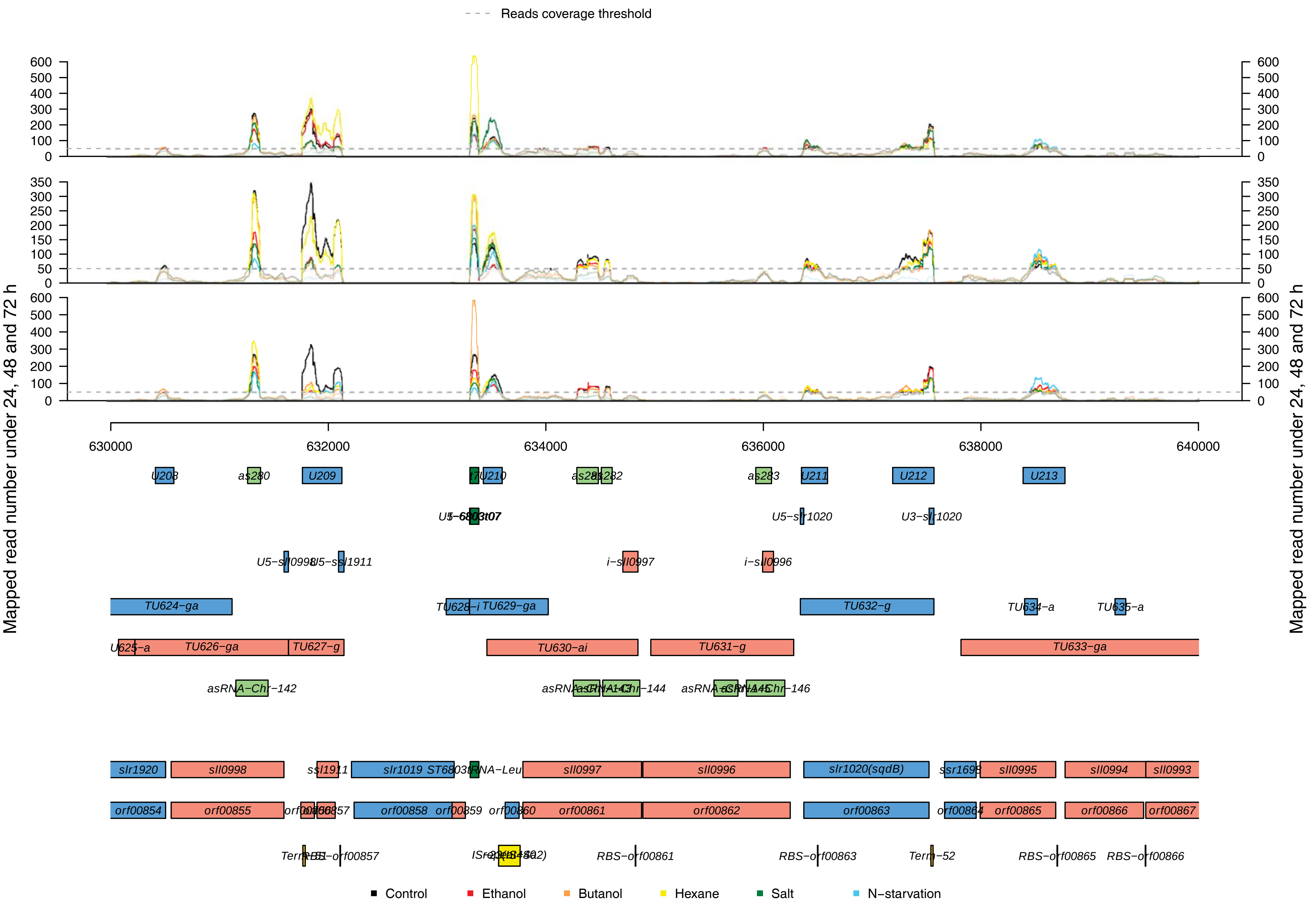

Reads coverage threshold
Mapped read number under 24, 48 and 72 h
630000
632000
634000
636000
638000
640000
U208
as280
U209
U210
U211
U212
U213
as283
U5−slr1020
U3−slr1020
i−sll0997
i−sll0996
TU624−ga
TU629−ga
TU632−g
TU626−ga
TU627−g
TU630−ai
TU631−g
TU633−ga
asRNA−Chr−142
slr1920
sll0998
ssl1911
slr1019
sll0997
sll0996
slr1020(sqdB)
sll0995
sll0994
sll0993
orf00854
orf00855
orf00858
orf00861
orf00862
orf00863
orf00865
orf00866
orf00867
RBS−orf00861
RBS−orf00863
Term−52
RBS−orf00865
RBS−orf00866
Control
Ethanol
Butanol
Hexane
Salt
N−starvation

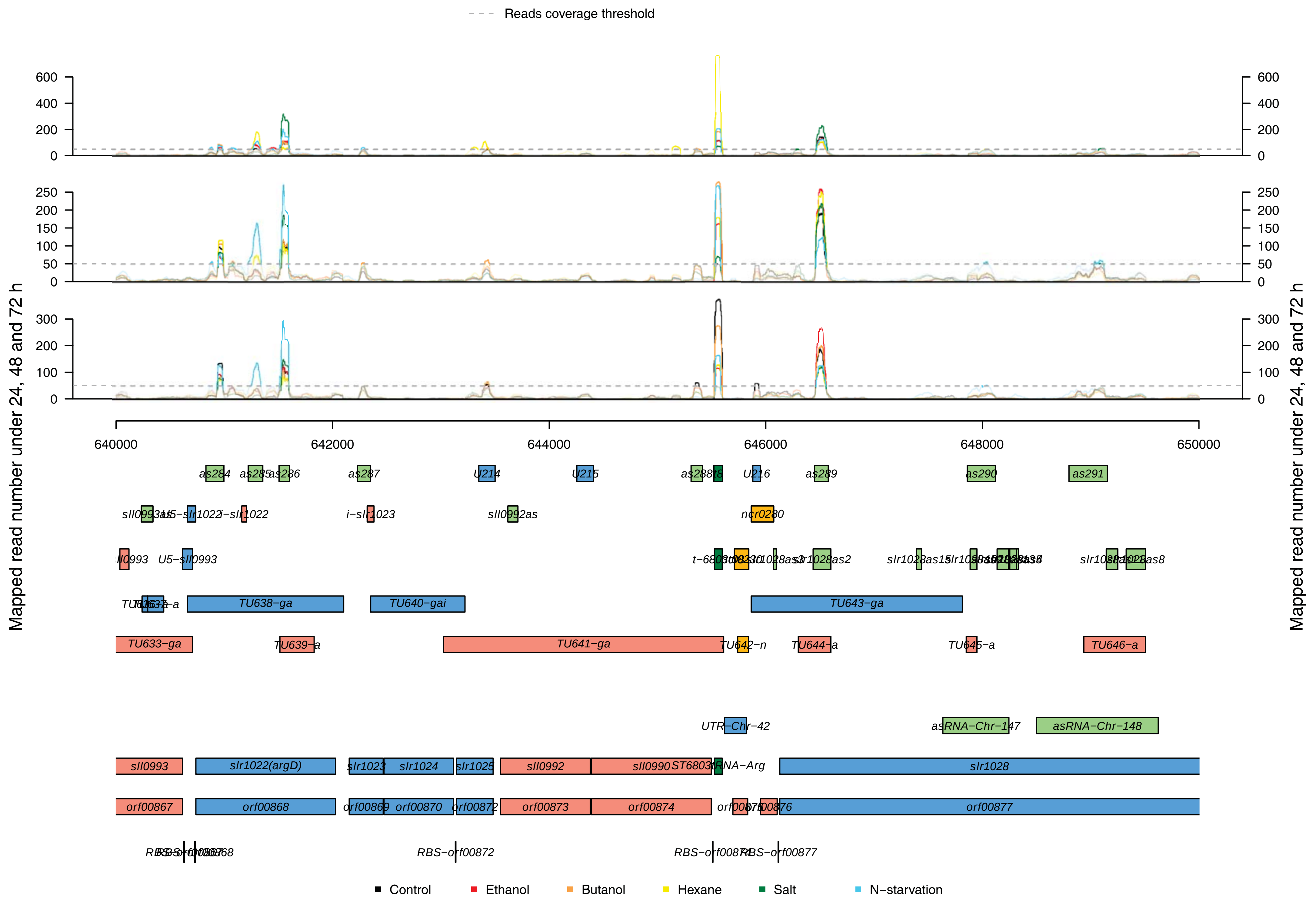

Reads coverage threshold
Mapped read number under 24, 48 and 72 h
640000
642000
644000
646000
648000
650000
TU638−ga
TU640−gai
TU643−ga
TU633−ga
TU639−a
TU641−ga
TU642−n
TU644−a
TU645−a
TU646−a
UTR−Chr−42
asRNA−Chr−147
asRNA−Chr−148
sll0993
slr1022(argD)
slr1023
slr1024
slr1025
sll0992
sll0990
ST6803t
tRNA−Arg
slr1028
orf00867
orf00868
orf00869
orf00870
orf00872
orf00873
orf00874
orf00877
RBS−orf00872
RBS−orf00877
Control
Ethanol
Butanol
Hexane
Salt
N−starvation

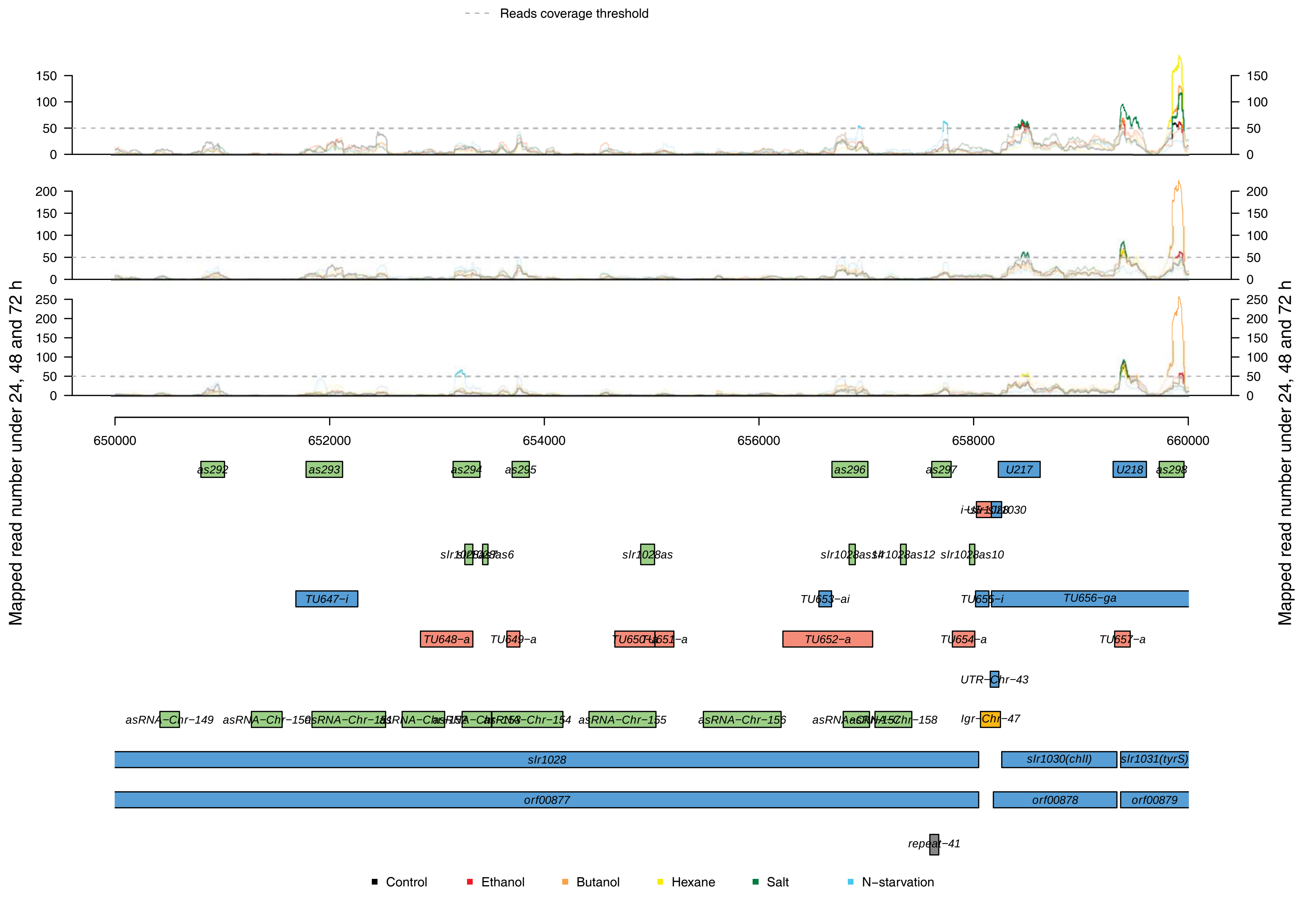
Reads coverage threshold
Mapped read number under 24, 48 and 72 h
650000
652000
654000
656000
658000
660000
as292
as293
as294
as295
as296
as297
U217
U218
as298
slr1028as
TU647−i
TU653−ai
TU656−ga
TU648−a
TU649−a
TU652−a
TU654−a
TU657−a
UTR−Chr−43
Igr−Chr−47
asRNA−Chr−149
asRNA−Chr−155
asRNA−Chr−156
slr1028
slr1030(chlI)
slr1031(tyrS)
orf00877
orf00878
orf00879
repeat−41
Control
Ethanol
Butanol
Hexane
Salt
N−starvation

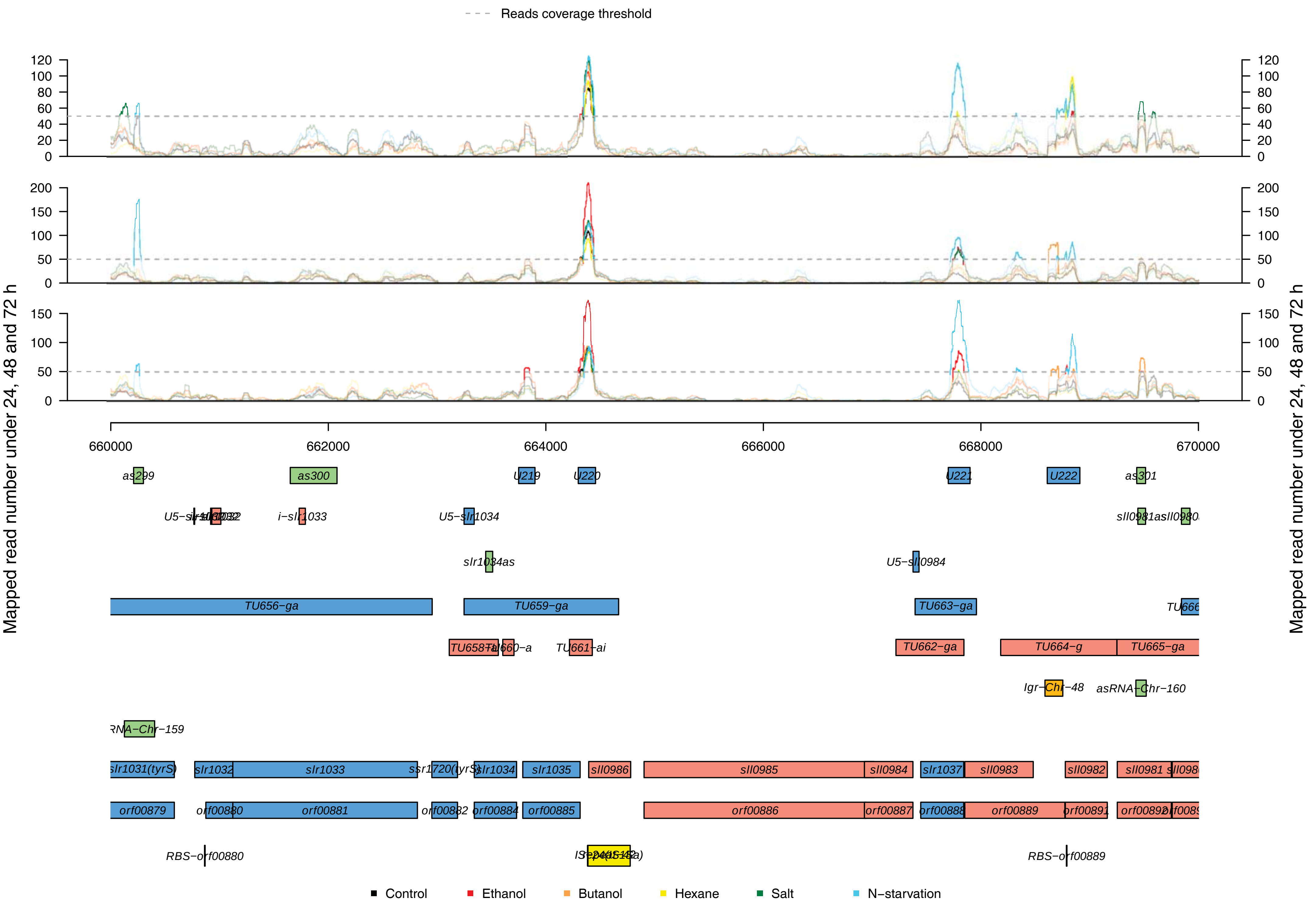

Reads coverage threshold
Mapped read number under 24, 48 and 72 h
660000
662000
664000
666000
668000
670000
as299
as300
U219
U220
U221
U222
as301
i−slr1033
U5−slr1034
slr1034as
U5−sll0984
TU656−ga
TU659−ga
TU663−ga
TU662−ga
TU664−g
TU665−ga
Igr−Chr−48
asRNA−Chr−160
slr1031(tyrS)
slr1032
slr1033
ssr1720(tyrS)
slr1034
slr1035
sll0986
sll0985
sll0984
slr1037
sll0983
sll0982
sll0981
orf00879
orf00880
orf00881
orf00882
orf00884
orf00885
orf00886
orf00887
orf00889
orf00891
RBS−orf00880
RBS−orf00889
Control
Ethanol
Butanol
Hexane
Salt
N−starvation

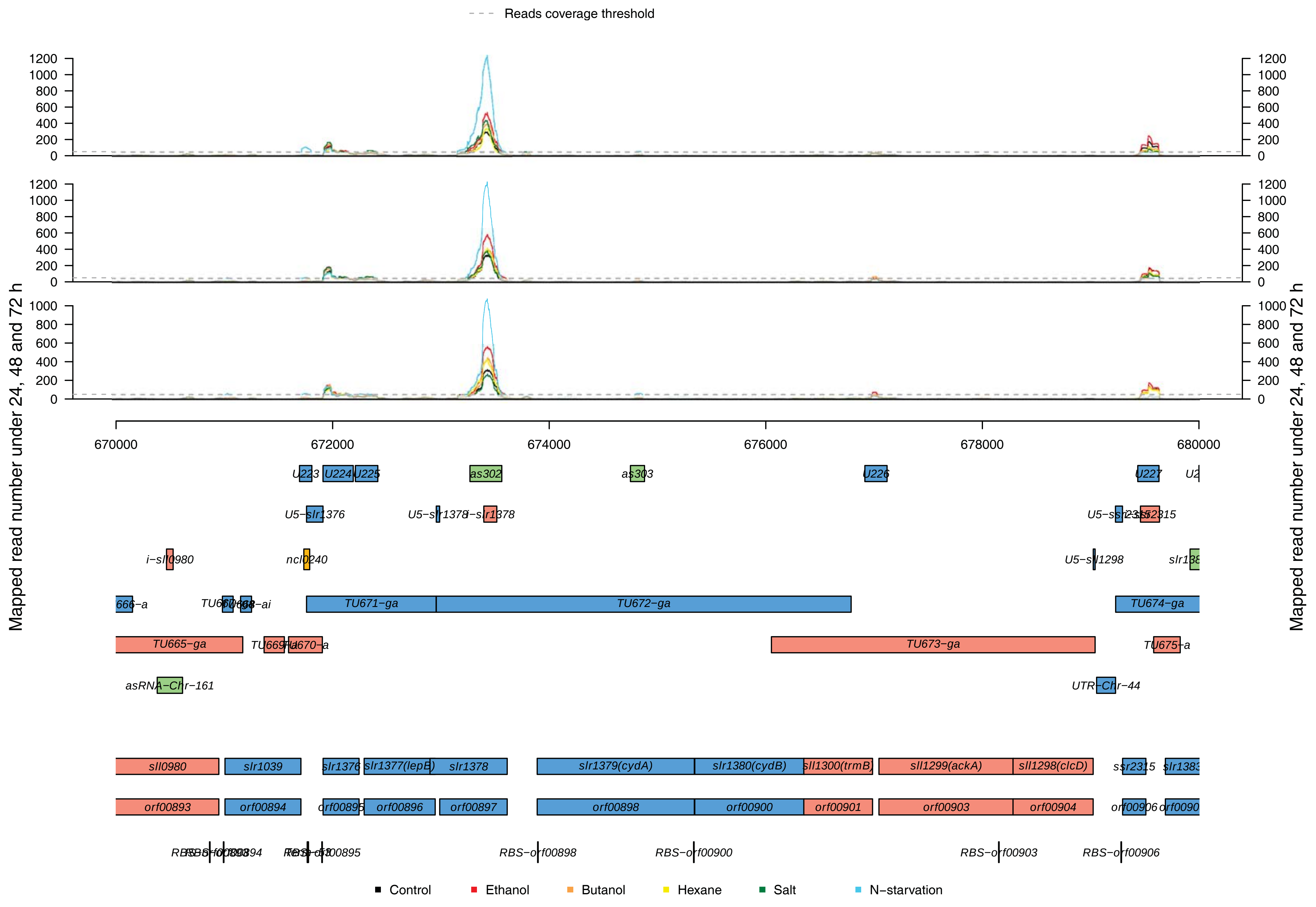

Reads coverage threshold
Mapped read number under 24, 48 and 72 h
670000
672000
674000
676000
678000
680000
TU671–ga
TU672–ga
TU673–ga
TU674–ga
TU665–ga
as302
as303
sll0980
slr1039
slr1377(lepB)
slr1378
slr1379(cydA)
slr1380(cydB)
sll1300(trmB)
sll1299(ackA)
sll1298(clcD)
orf00893
orf00894
orf00896
orf00897
orf00898
orf00900
orf00901
orf00903
orf00904
RBS–orf00898
RBS–orf00900
RBS–orf00903
RBS–orf00906
Control
Ethanol
Butanol
Hexane
Salt
N–starvation

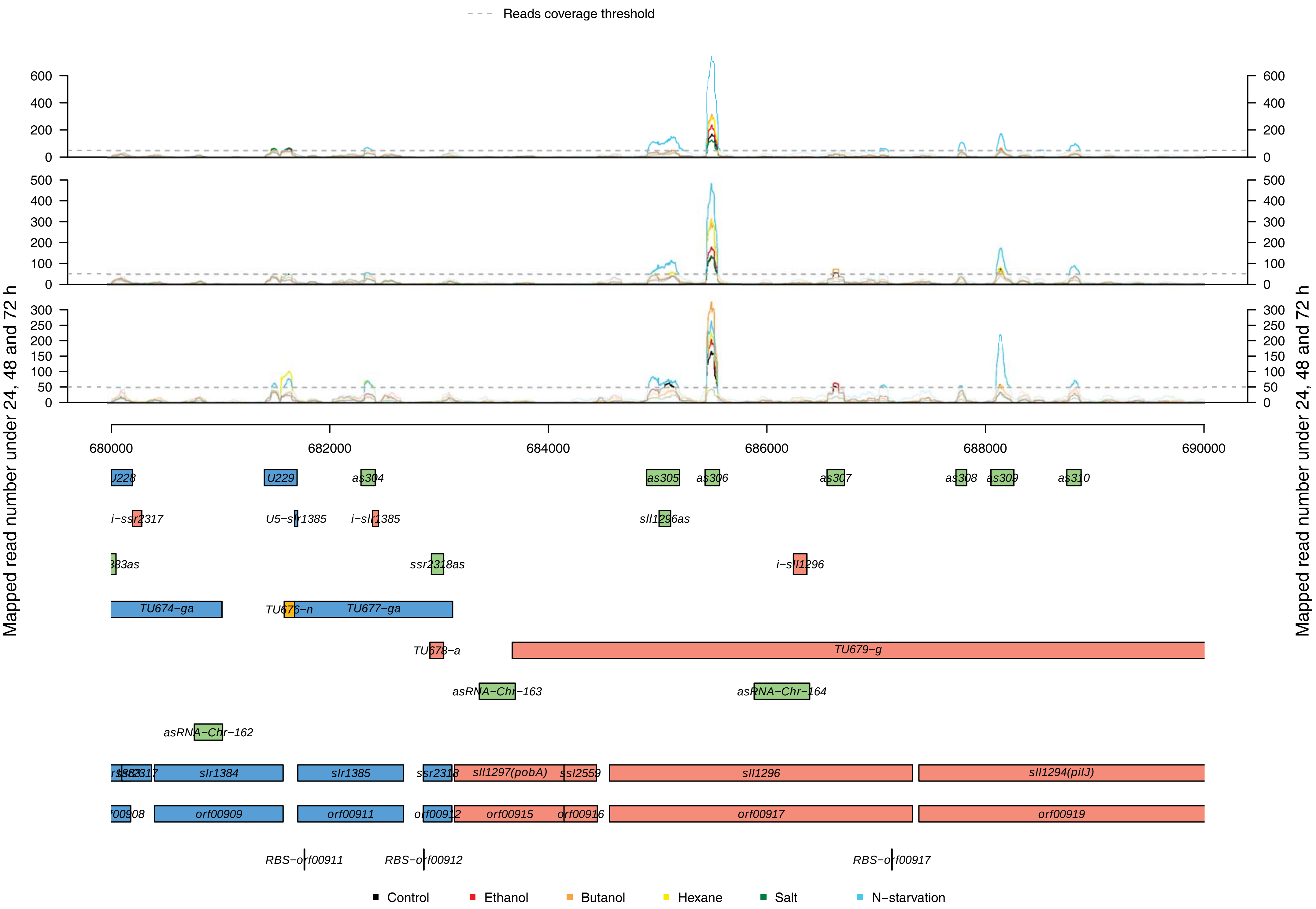

Reads coverage threshold
Mapped read number under 24, 48 and 72 h
680000
682000
684000
686000
688000
690000
U228
U229
as304
as305
as306
as307
as308
as309
as310
i−ssr2317
U5−slr1385
i−sll1385
sll1296as
ssr2318as
i−sll1296
TU674−ga
TU676−n
TU677−ga
TU678−a
TU679−g
asRNA−Chr−163
asRNA−Chr−164
asRNA−Chr−162
slr1384
slr1385
ssr2318
sll1297(pobA)
ssl2559
sll1296
sll1294(pilJ)
orf00909
orf00911
orf00912
orf00915
orf00916
orf00917
orf00919
RBS−orf00911
RBS−orf00912
RBS−orf00917
Control
Ethanol
Butanol
Hexane
Salt
N−starvation

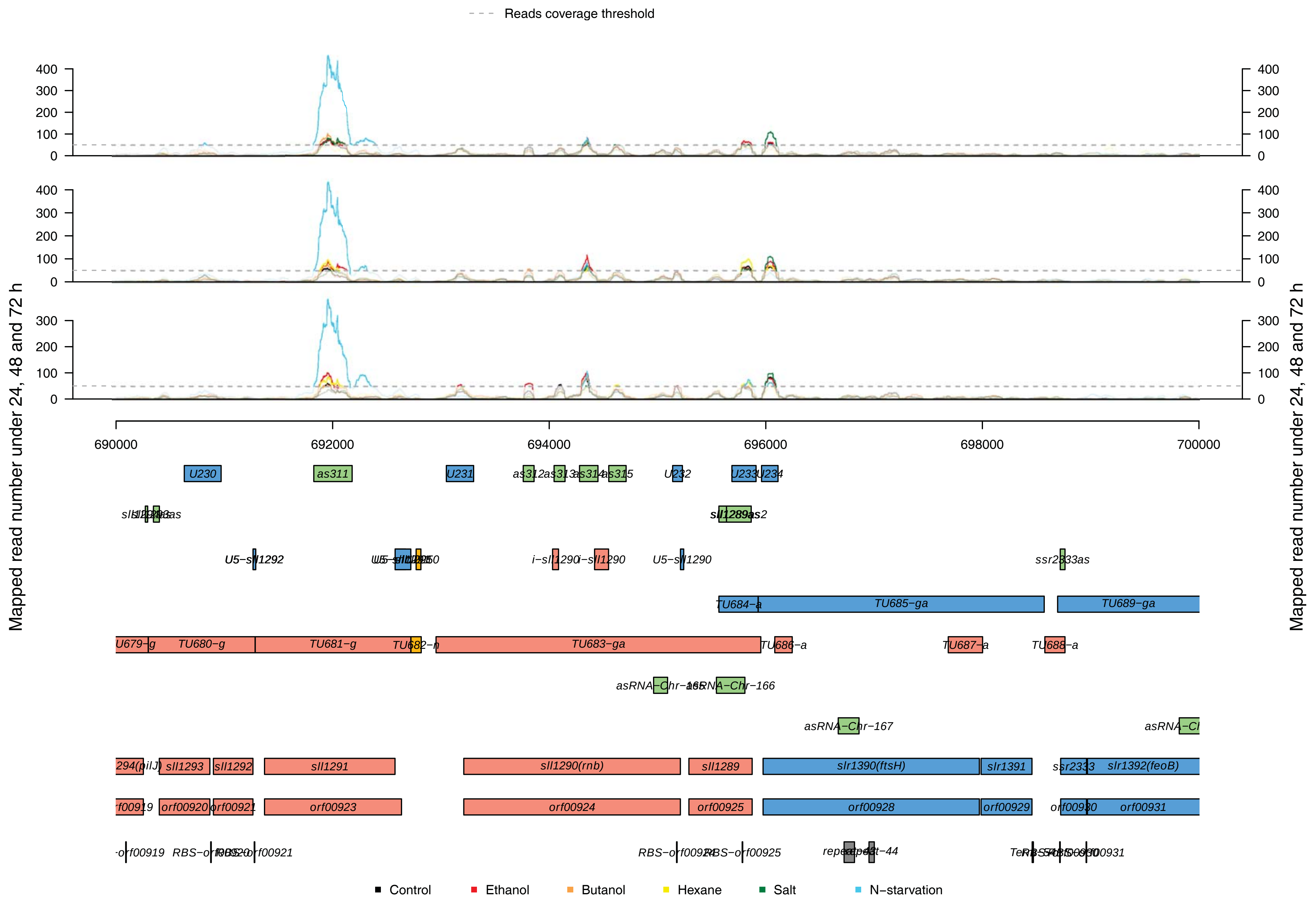

Reads coverage threshold
400
300
200
100
0
Mapped read number under 24, 48 and 72 h
690000
692000
694000
696000
698000
700000
U230
as311
U231
U232
slr1390(ftsH)
slr1391
slr1392(feoB)
orf00923
orf00924
orf00925
orf00928
orf00929
orf00931
sll1291
sll1290(rnb)
sll1289
sll1293
sll1292
TU680−g
TU681−g
TU683−ga
TU685−ga
TU689−ga
asRNA−Chr−167
Control
Ethanol
Butanol
Hexane
Salt
N−starvation

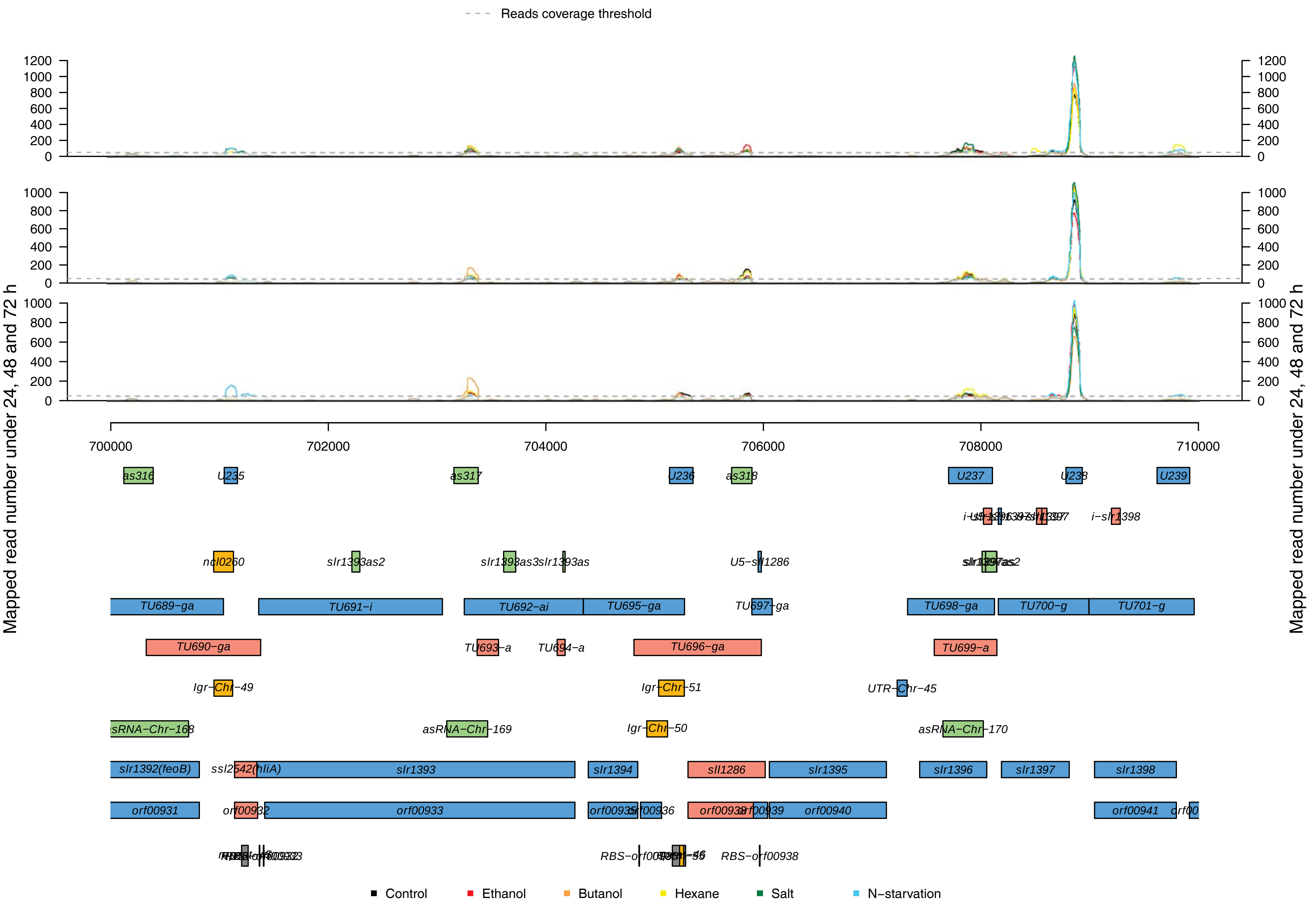

Reads coverage threshold
Mapped read number under 24, 48 and 72 h
700000
702000
704000
706000
708000
710000
Control
Ethanol
Butanol
Hexane
Salt
N−starvation

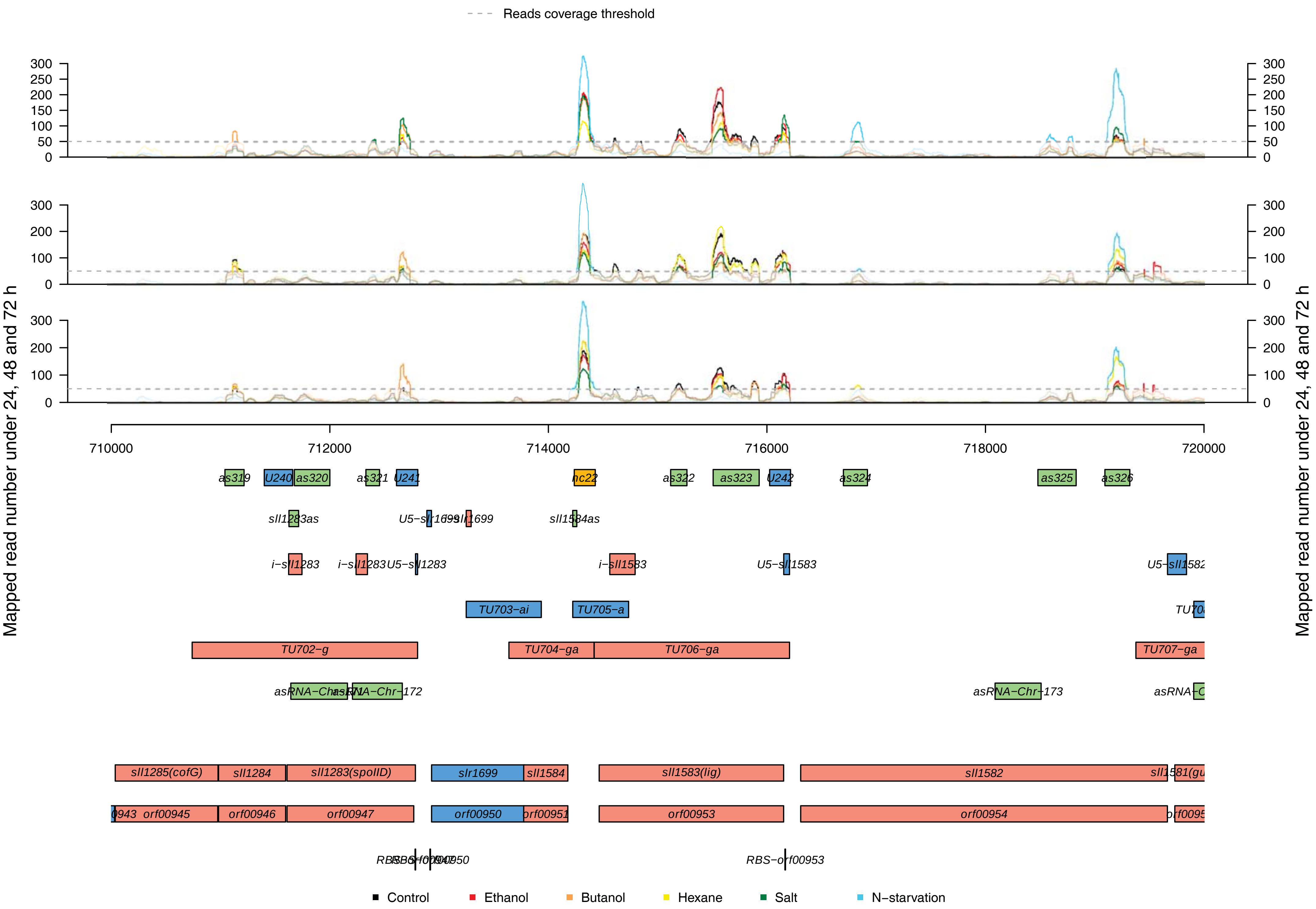

Reads coverage threshold
Mapped read number under 24, 48 and 72 h
710000
712000
714000
716000
718000
720000
Control
Ethanol
Butanol
Hexane
Salt
N−starvation

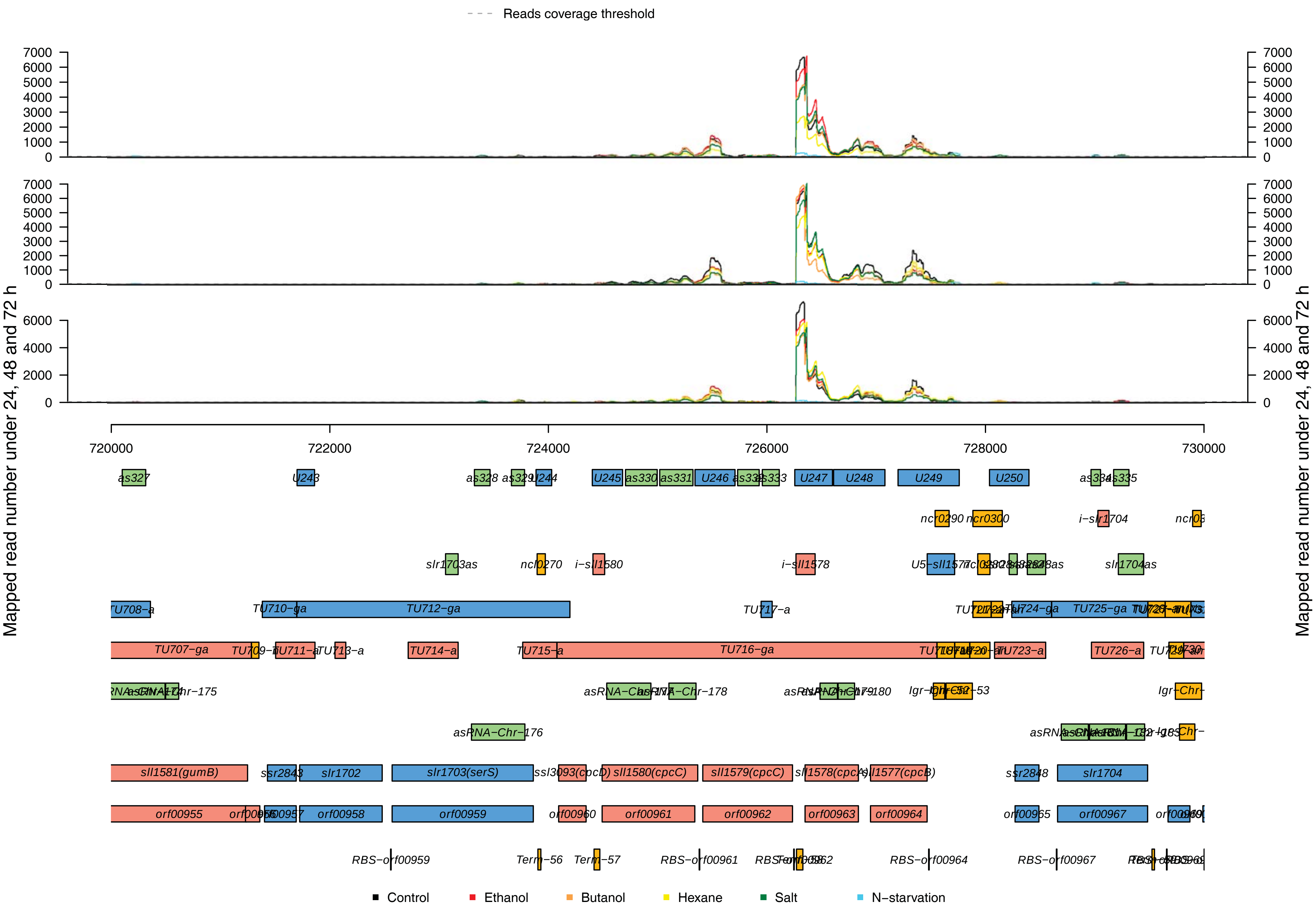

Reads coverage threshold
Mapped read number under 24, 48 and 72 h
720000
722000
724000
726000
728000
730000
TU712−ga
TU716−ga
TU707−ga
sll1581(gumB)
slr1703(serS)
sll1580(cpcC)
sll1579(cpcC)
slr1704
orf00955
orf00959
orf00961
orf00962
orf00967
Control
Ethanol
Butanol
Hexane
Salt
N−starvation

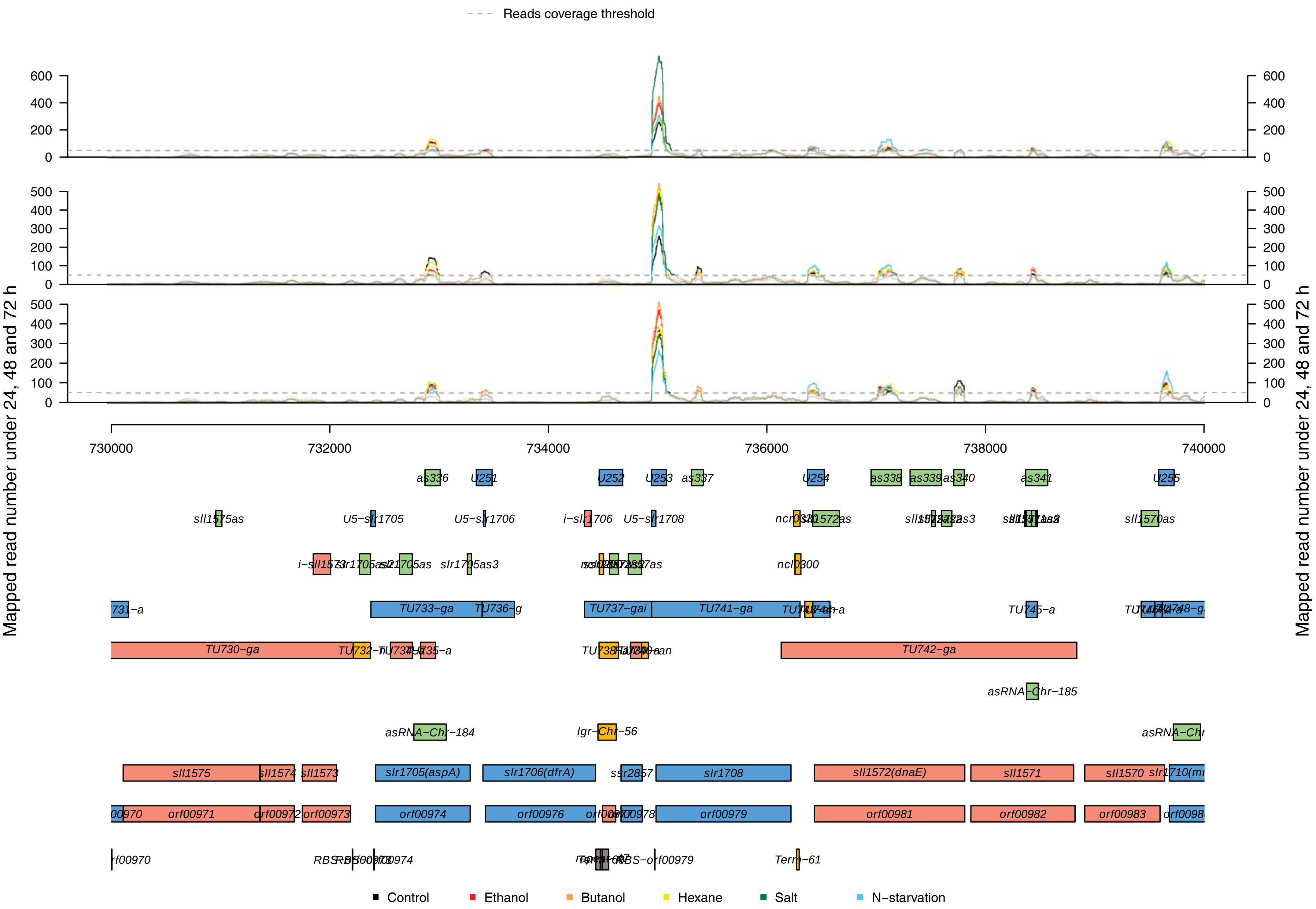

Reads coverage threshold
Mapped read number under 24, 48 and 72 h
730000
732000
734000
736000
738000
740000
as336
U251
U252
U253
as337
U254
as338
as339
as340
as341
U255
sll1575as
U5−slr1705
U5−slr1706
i−slr1706
U5−slr1708
sll1570as
slr1705as3
ncl0300
TU733−ga
TU737−gai
TU741−ga
TU745−a
TU730−ga
TU742−ga
asRNA−Chr−185
asRNA−Chr−184
Igr−Chr−56
sll1575
sll1574
sll1573
slr1705(aspA)
slr1706(dfrA)
ssr2857
slr1708
sll1572(dnaE)
sll1571
sll1570
orf00971
orf00973
orf00974
orf00976
orf00979
orf00981
orf00982
orf00983
RBS−orf00979
Term−61
Control
Ethanol
Butanol
Hexane
Salt
N−starvation

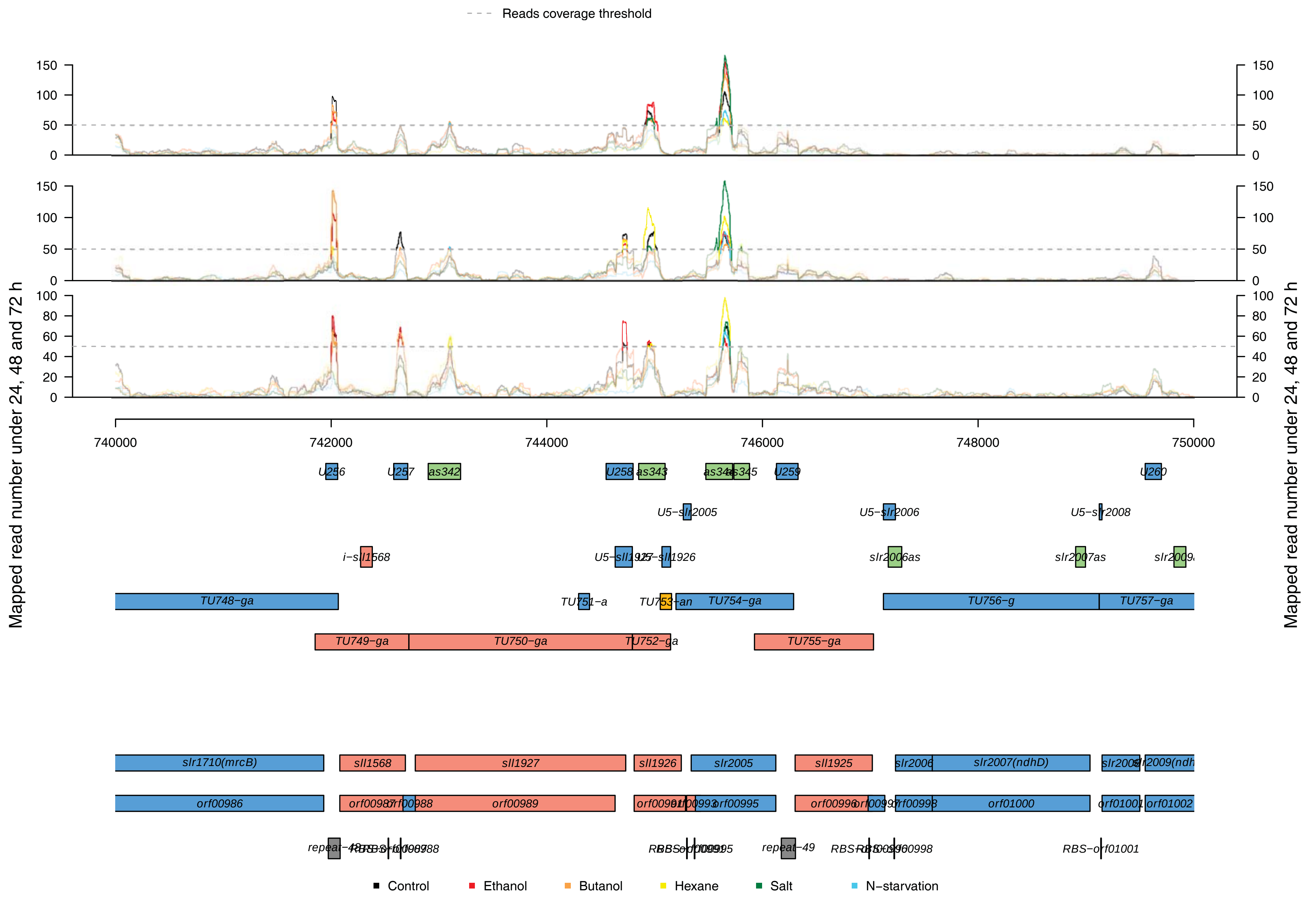

Reads coverage threshold
Mapped read number under 24, 48 and 72 h
740000
742000
744000
746000
748000
750000
U256
U257
as342
U258
as343
as345
U259
U260
U5−slr2005
U5−slr2006
U5−slr2008
i−sll1568
slr2006as
slr2007as
TU748−ga
TU751−a
TU754−ga
TU756−g
TU757−ga
TU749−ga
TU750−ga
TU752−ga
TU755−ga
slr1710(mrcB)
sll1568
sll1927
sll1926
slr2005
sll1925
slr2007(ndhD)
orf00986
orf00989
orf00995
orf01000
orf01001
orf01002
repeat−49
RBS−orf01001
Control
Ethanol
Butanol
Hexane
Salt
N−starvation

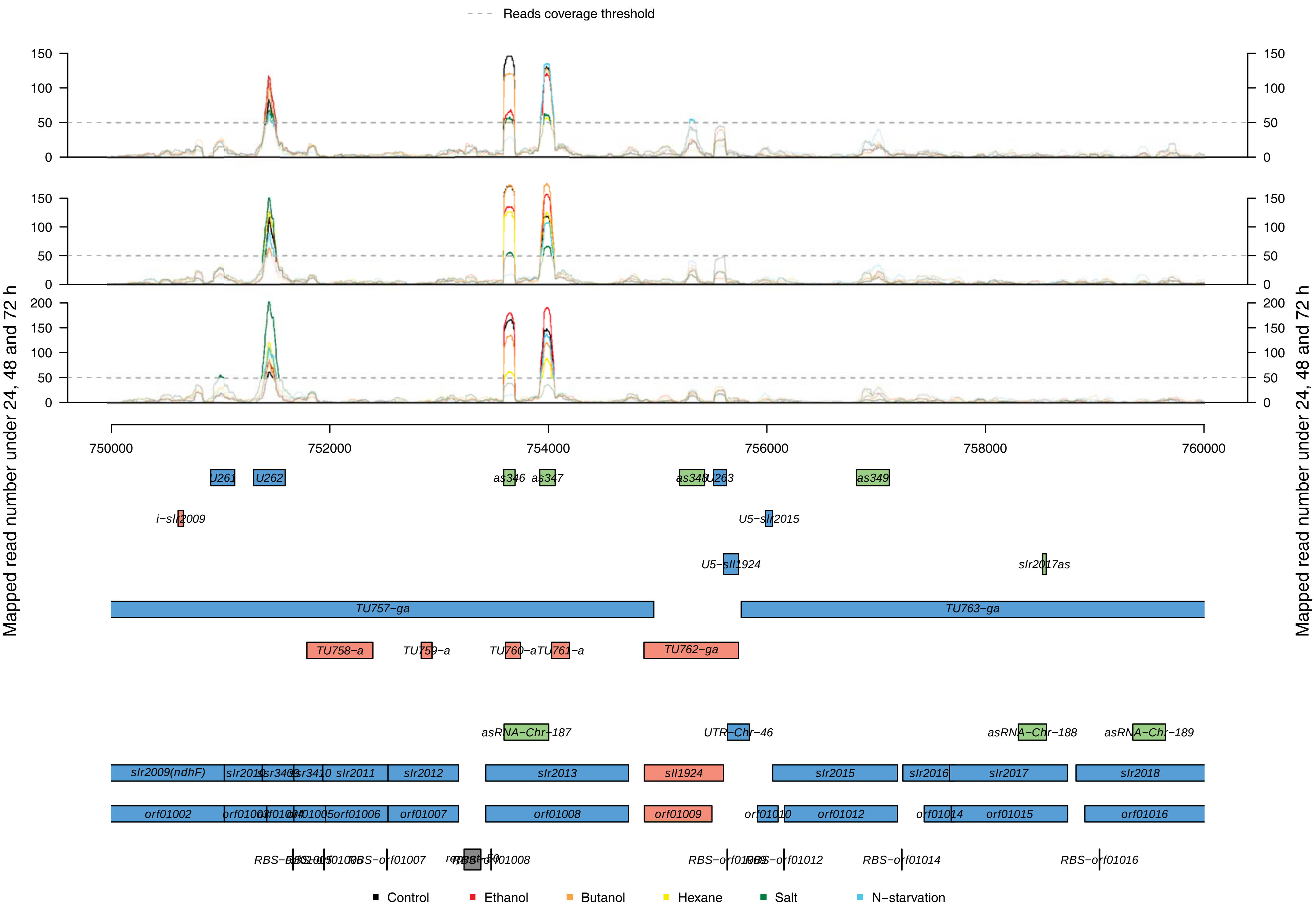

Reads coverage threshold
Mapped read number under 24, 48 and 72 h
750000
752000
754000
756000
758000
760000
TU757−ga
TU763−ga
TU758−a
TU762−ga
slr2013
slr2015
slr2017
slr2018
orf01002
orf01007
orf01008
orf01012
orf01015
orf01016
Control
Ethanol
Butanol
Hexane
Salt
N−starvation

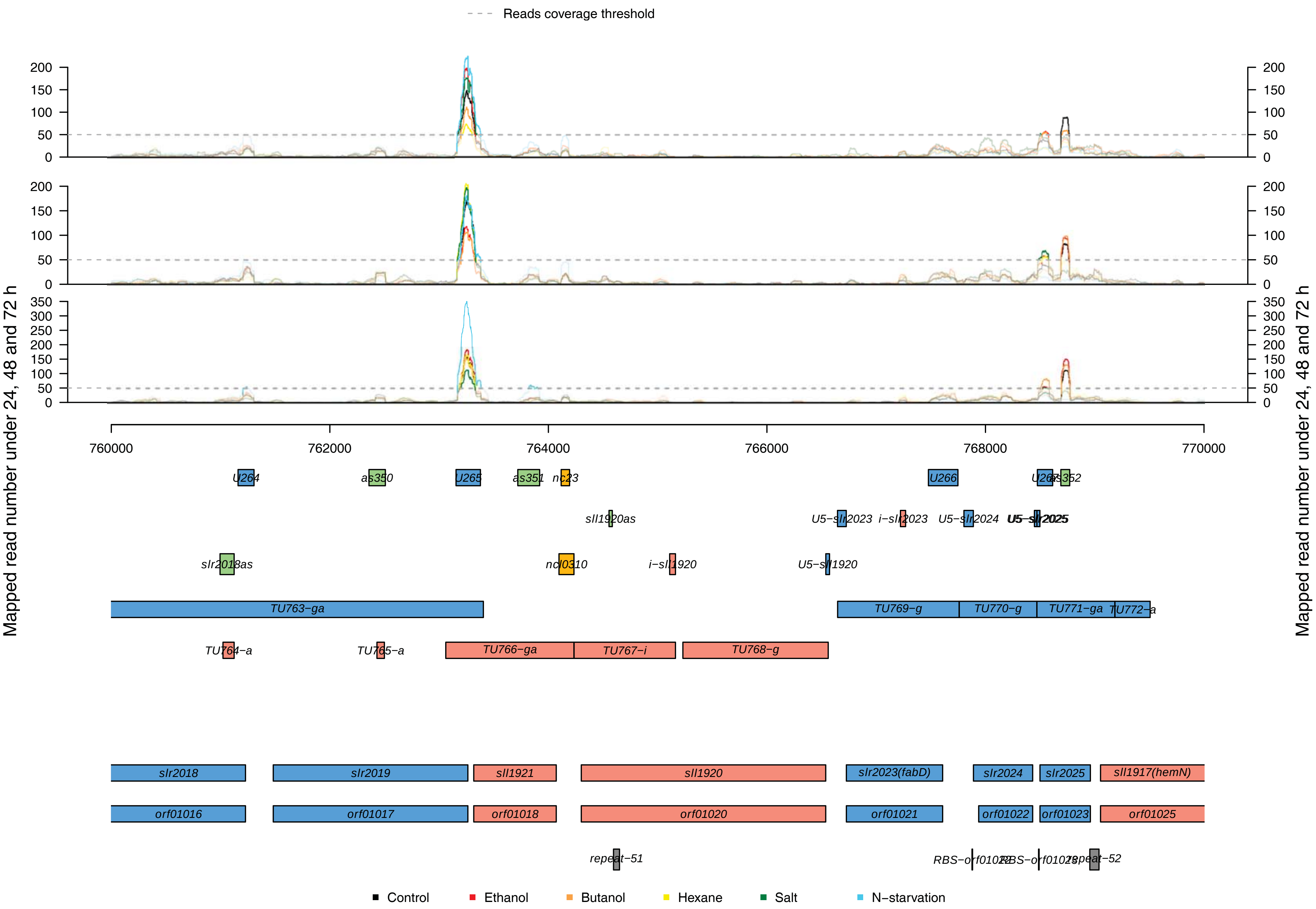

Reads coverage threshold
Mapped read number under 24, 48 and 72 h
760000
762000
764000
766000
768000
770000
U264
as350
U265
as351
nc23
U266
sll1920as
U5−slr2023
i−slr2023
U5−slr2024
U5−slr2025
slr2018as
ncl0310
i−sll1920
U5−sll1920
TU763−ga
TU769−g
TU770−g
TU771−ga
TU772−a
TU764−a
TU765−a
TU766−ga
TU767−i
TU768−g
slr2018
slr2019
sll1921
sll1920
slr2023(fabD)
slr2024
slr2025
sll1917(hemN)
orf01016
orf01017
orf01018
orf01020
orf01021
orf01022
orf01023
orf01025
repeat−51
repeat−52
Control
Ethanol
Butanol
Hexane
Salt
N−starvation

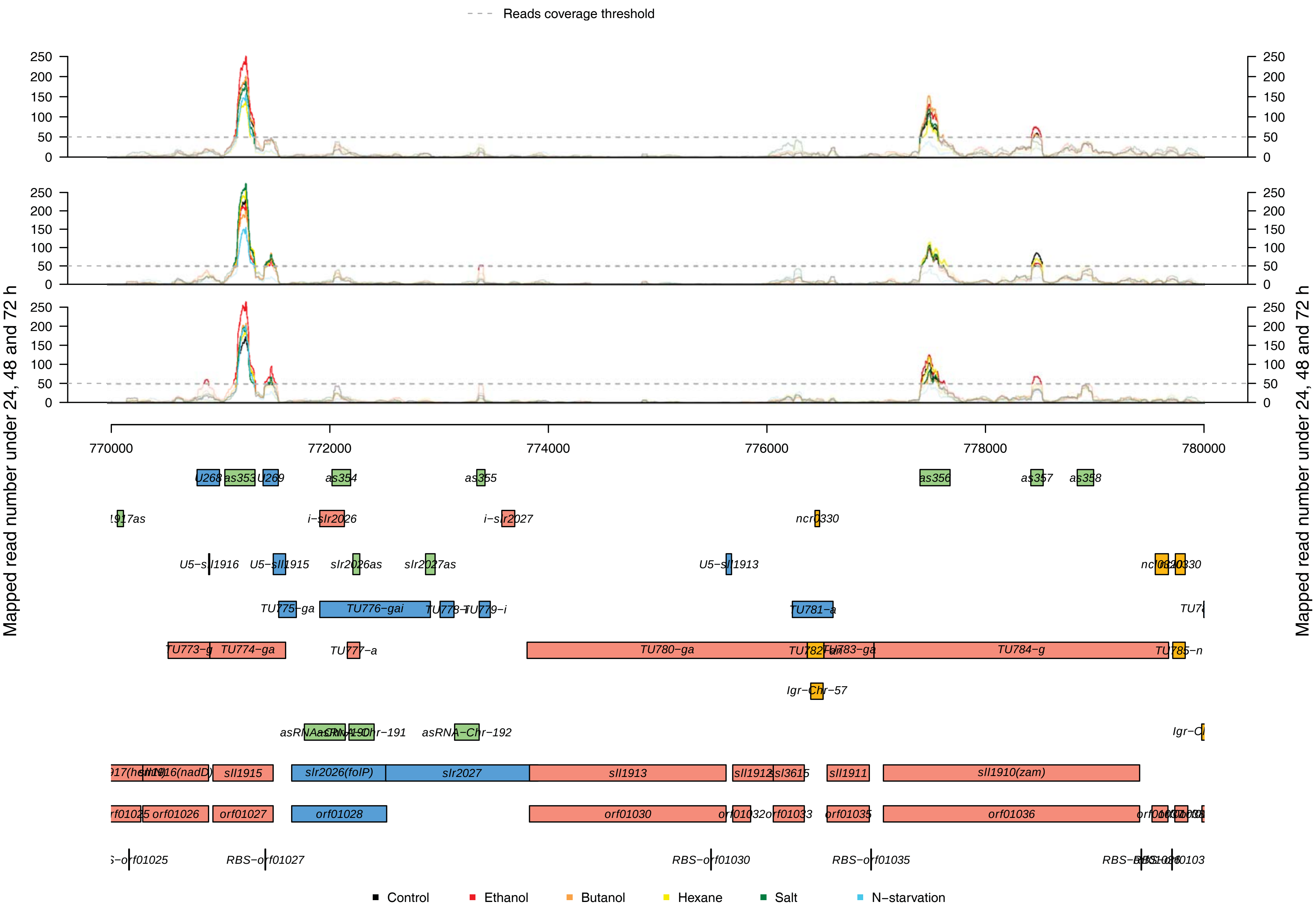

Reads coverage threshold
Mapped read number under 24, 48 and 72 h
770000
772000
774000
776000
778000
780000
as353
as354
as355
as356
as357
as358
i−slr2026
i−slr2027
ncr0330
U5−sll1916
U5−sll1915
slr2026as
slr2027as
U5−sll1913
TU776−gai
TU780−ga
TU784−g
TU774−ga
Igr−Chr−57
asRNA−Chr−192
sll1915
slr2026(folP)
slr2027
sll1913
sll1911
sll1910(zam)
orf01026
orf01027
orf01028
orf01030
orf01036
RBS−orf01027
RBS−orf01030
RBS−orf01035
Control
Ethanol
Butanol
Hexane
Salt
N−starvation

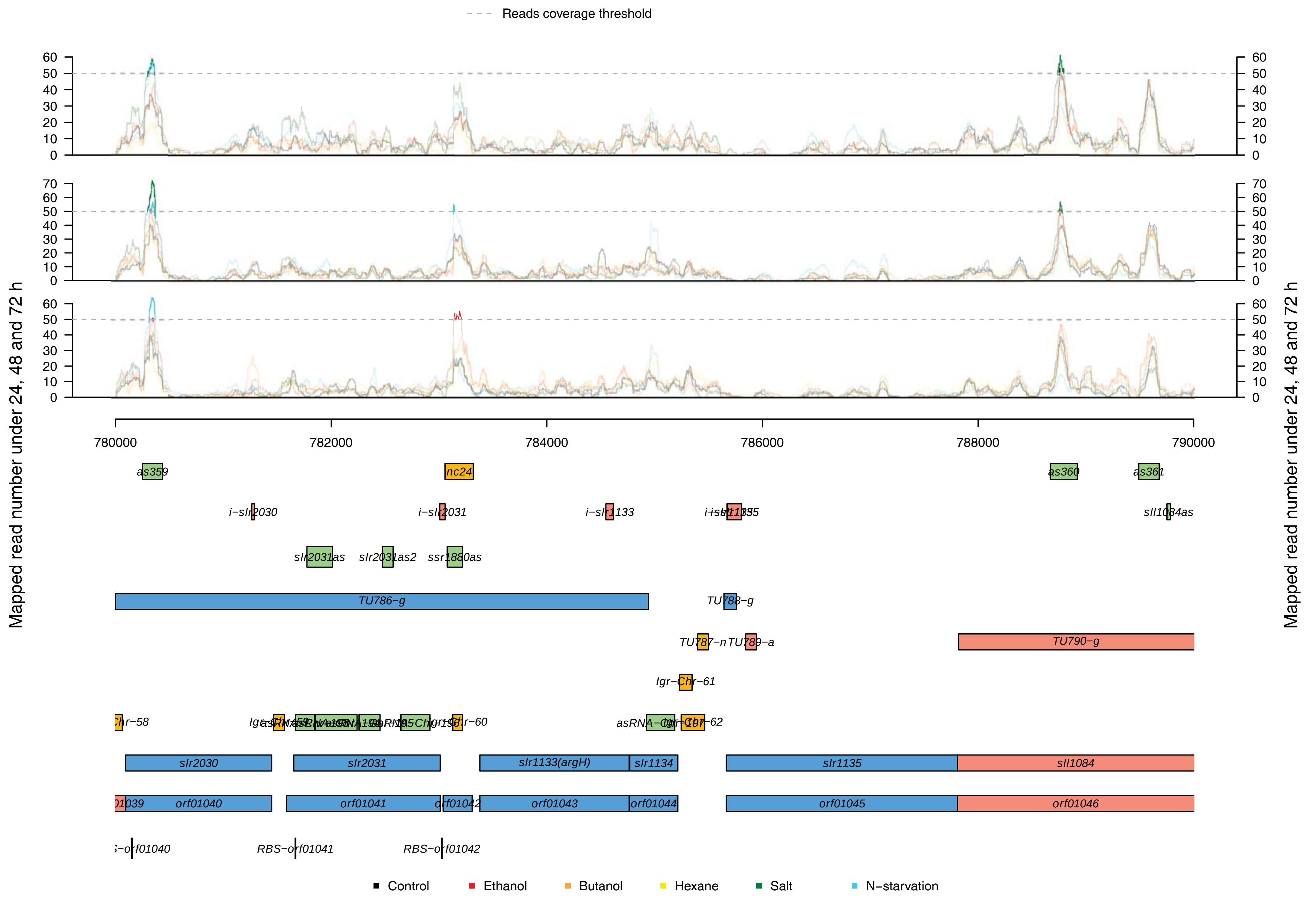

Reads coverage threshold
Mapped read number under 24, 48 and 72 h
780000
782000
784000
786000
788000
790000
as359
nc24
as360
as361
i−slr2030
i−slr2031
i−slr1133
sll1084as
slr2031as
slr2031as2
ssr1880as
TU786−g
TU788−g
TU787−n
TU789−a
TU790−g
Igr−Chr−61
slr2030
slr2031
slr1133(argH)
slr1134
slr1135
sll1084
orf01040
orf01041
orf01043
orf01044
orf01045
orf01046
RBS−orf01041
RBS−orf01042
Control
Ethanol
Butanol
Hexane
Salt
N−starvation

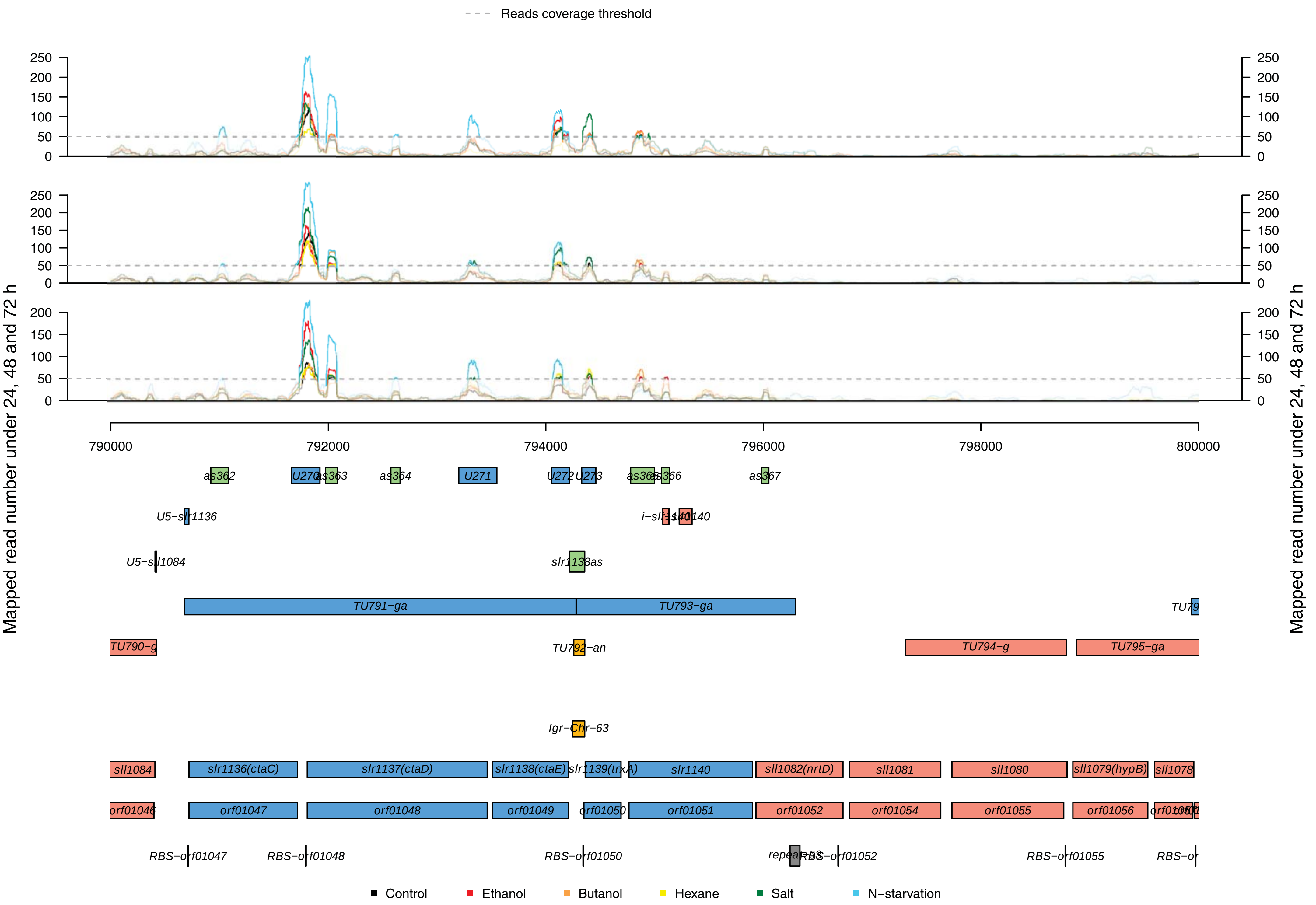
Reads coverage threshold
Mapped read number under 24, 48 and 72 h
790000
792000
794000
796000
798000
800000
as362
U270
as363
as364
U271
U272
U273
as365
as366
as367
U5−slr1136
i−slr1140
U5−sll1084
slr1138as
TU791−ga
TU793−ga
TU790−g
TU792−an
TU794−g
TU795−ga
Igr−Chr−63
sll1084
slr1136(ctaC)
slr1137(ctaD)
slr1138(ctaE)
slr1139(trxA)
slr1140
sll1082(nrtD)
sll1081
sll1080
sll1079(hypB)
sll1078
orf01046
orf01047
orf01048
orf01049
orf01050
orf01051
orf01052
orf01054
orf01055
orf01056
RBS−orf01047
RBS−orf01048
RBS−orf01050
RBS−orf01052
RBS−orf01055
Control
Ethanol
Butanol
Hexane
Salt
N−starvation

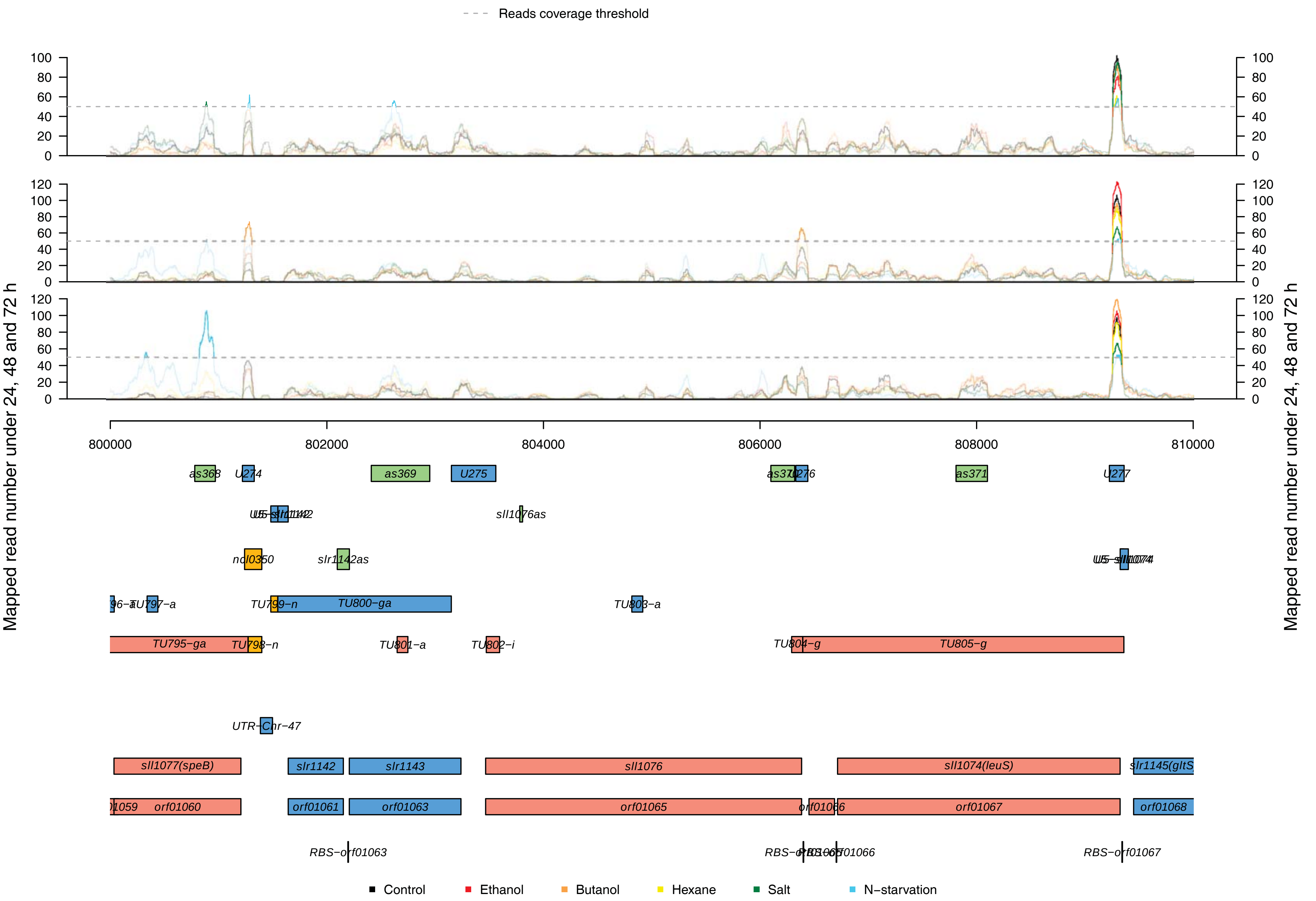
Reads coverage threshold
Mapped read number under 24, 48 and 72 h
800000
802000
804000
806000
808000
810000
as368
U274
as369
U275
as370
U276
as371
U277
sll1076as
ncl0350
slr1142as
TU797−a
TU799−n
TU800−ga
TU803−a
TU795−ga
TU798−n
TU801−a
TU802−i
TU804−g
TU805−g
UTR−Chr−47
sll1077(speB)
slr1142
slr1143
sll1076
sll1074(leuS)
orf01060
orf01061
orf01063
orf01065
orf01067
orf01068
RBS−orf01063
RBS−orf01066
RBS−orf01067
Control
Ethanol
Butanol
Hexane
Salt
N−starvation

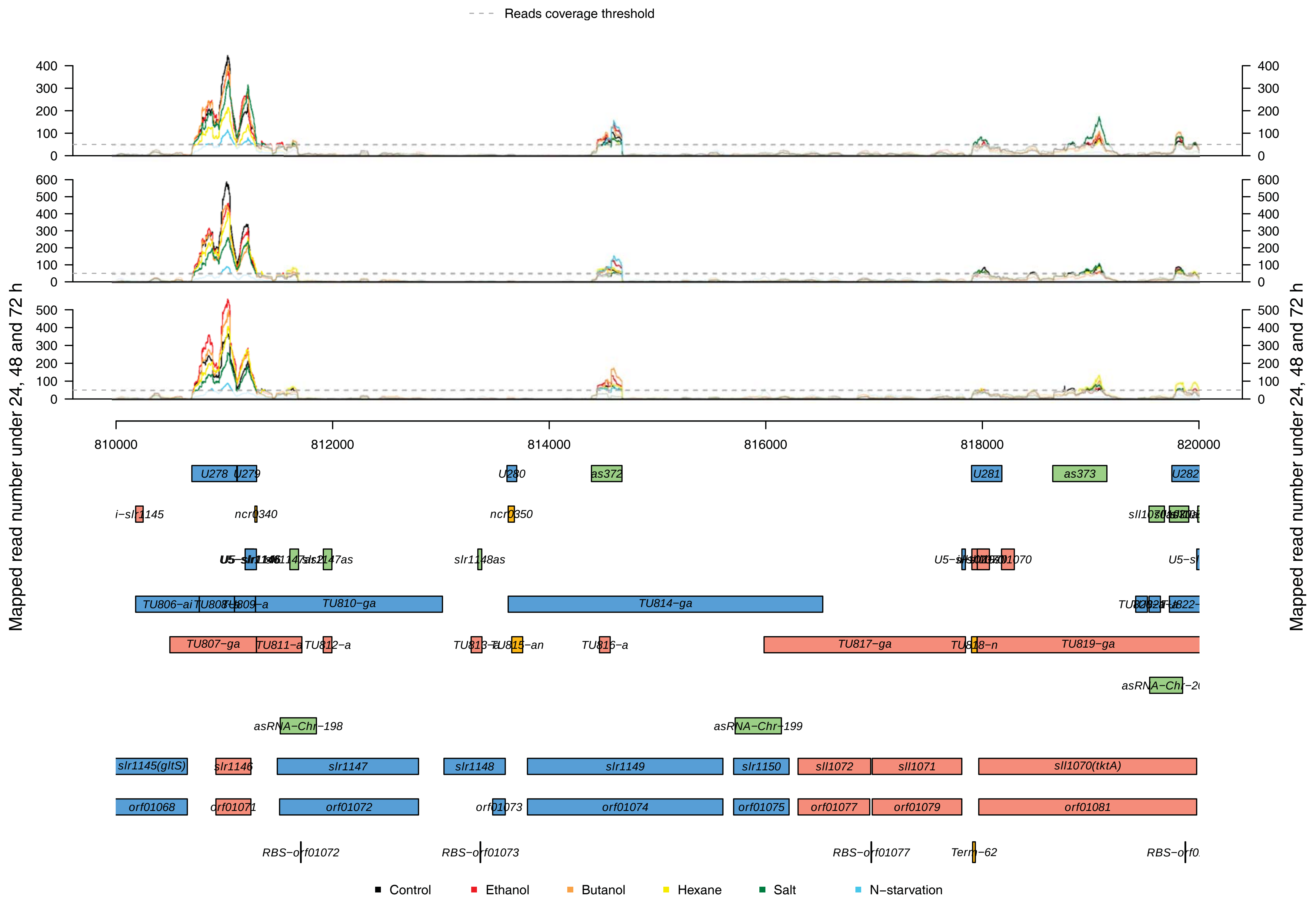

Reads coverage threshold
Mapped read number under 24, 48 and 72 h
810000
812000
814000
816000
818000
820000
U278
U279
U280
as372
U281
as373
U282
i−slr1145
ncr0340
ncr0350
slr1148as
TU806−ai
TU810−ga
TU814−ga
TU807−ga
TU811−a
TU812−a
TU816−a
TU817−ga
TU819−ga
asRNA−Chr−198
asRNA−Chr−199
slr1145(gltS)
slr1146
slr1147
slr1148
slr1149
slr1150
sll1072
sll1071
sll1070(tktA)
orf01068
orf01071
orf01072
orf01073
orf01074
orf01075
orf01077
orf01079
orf01081
RBS−orf01072
RBS−orf01073
RBS−orf01077
Term−62
Control
Ethanol
Butanol
Hexane
Salt
N−starvation

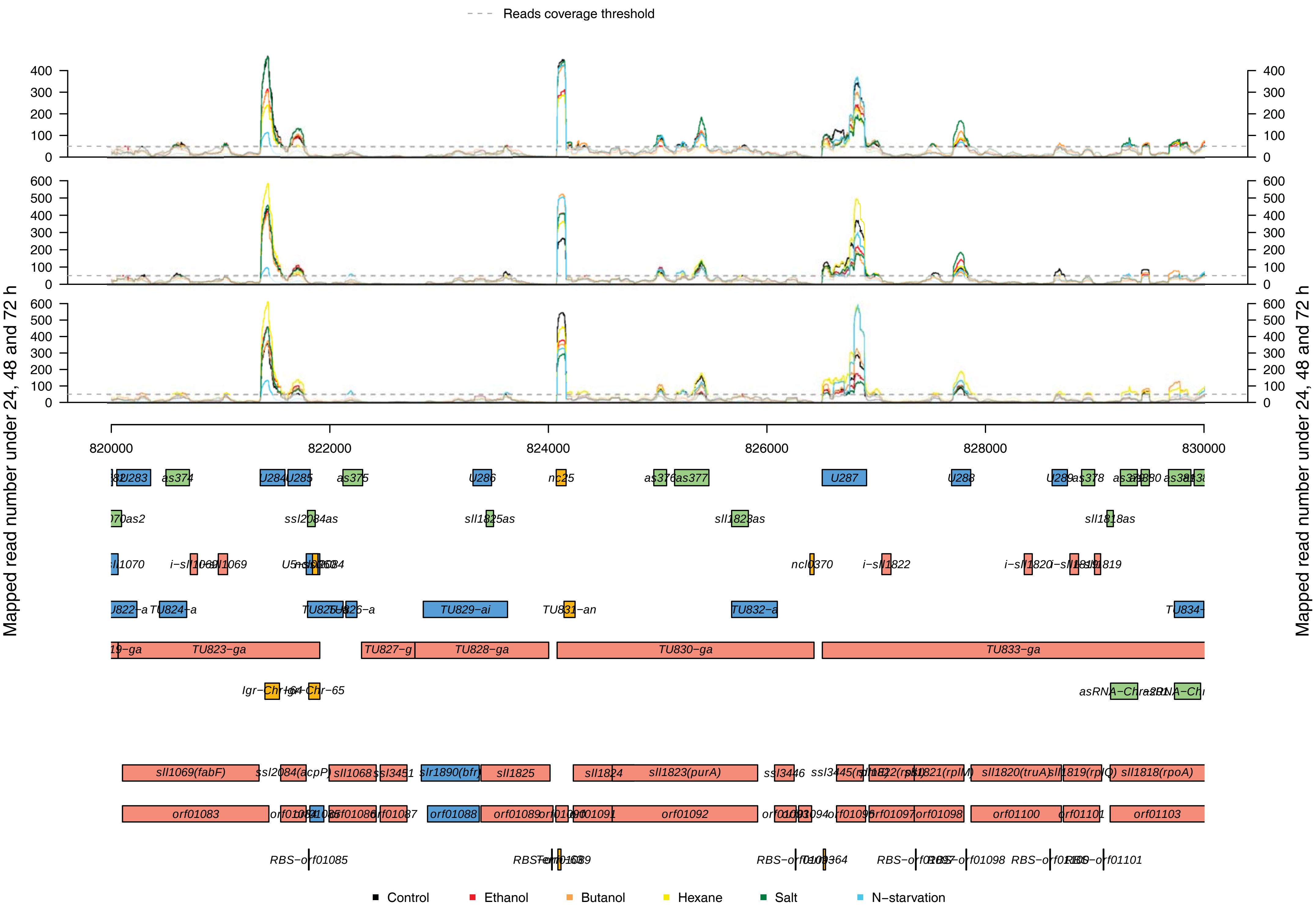

Reads coverage threshold
Mapped read number under 24, 48 and 72 h
820000
822000
824000
826000
828000
830000
as374
as375
U286
nc25
as377
U287
U288
ssl2084as
sll1825as
sll1823as
sll1818as
ncl0370
i−sll1822
TU824−a
TU829−ai
TU831−an
TU832−a
TU823−ga
TU828−ga
TU830−ga
TU833−ga
sll1069(fabF)
sll1068
ssl3451
slr1890(bfr)
sll1825
sll1824
sll1823(purA)
ssl3446
sll1820(truA)
sll1818(rpoA)
orf01083
orf01088
orf01092
orf01100
orf01103
RBS−orf01085
Control
Ethanol
Butanol
Hexane
Salt
N−starvation

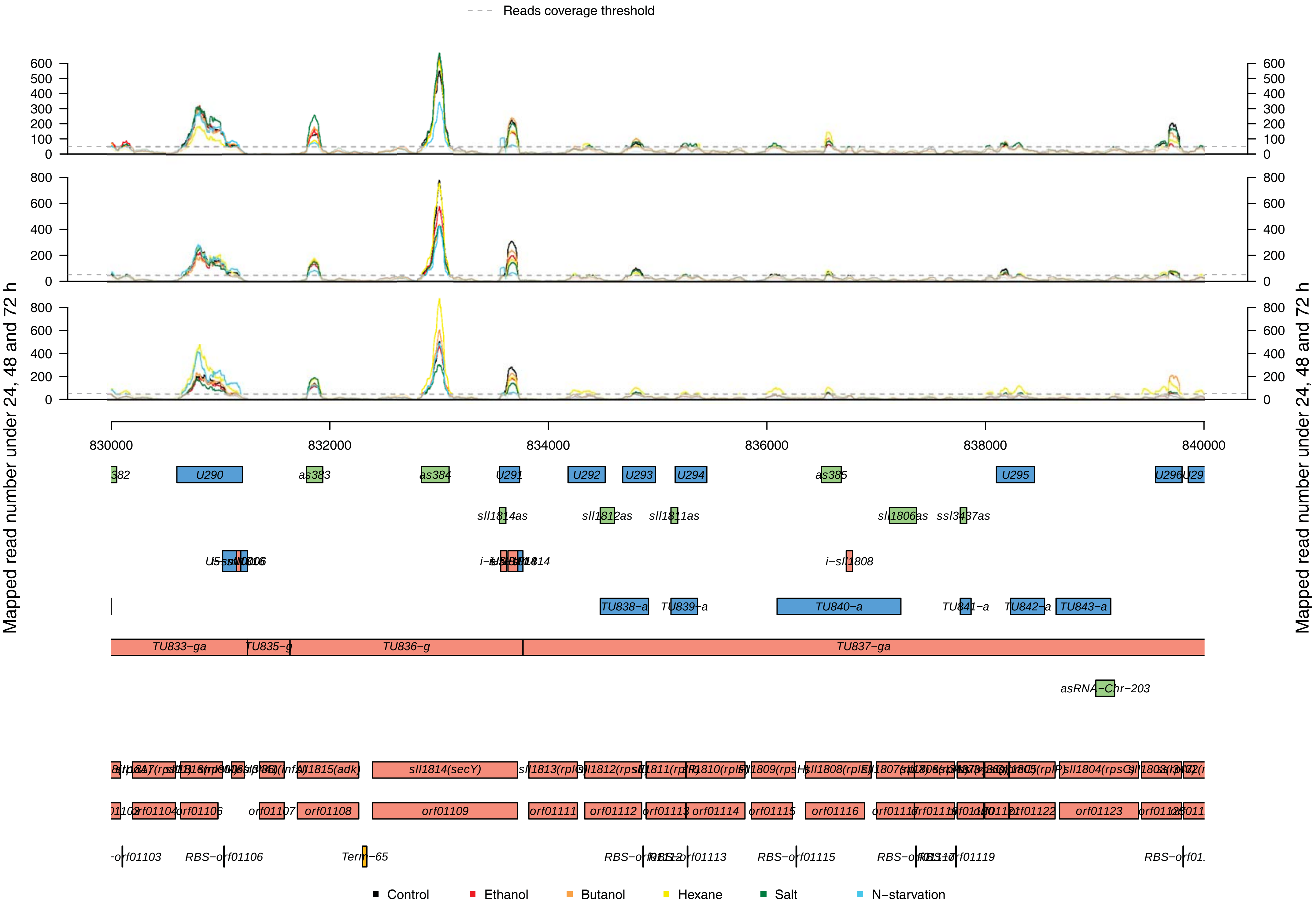

Reads coverage threshold
Mapped read number under 24, 48 and 72 h
0
100
200
300
400
500
600
800
830000
832000
834000
836000
838000
840000
U290
as383
as384
U291
U292
U293
U294
as385
U295
sll1814as
sll1812as
sll1811as
sll1806as
ssl3437as
i−sll1808
TU838−a
TU839−a
TU840−a
TU841−a
TU842−a
TU843−a
TU833−ga
TU835−g
TU836−g
TU837−ga
asRNA−Chr−203
sll1815(adk)
sll1814(secY)
sll1804(rpsC)
orf01104
orf01106
orf01107
orf01108
orf01109
orf01111
orf01112
orf01114
orf01115
orf01116
orf01122
orf01123
RBS−orf01106
Term−65
RBS−orf01115
RBS−orf01119
Control
Ethanol
Butanol
Hexane
Salt
N−starvation

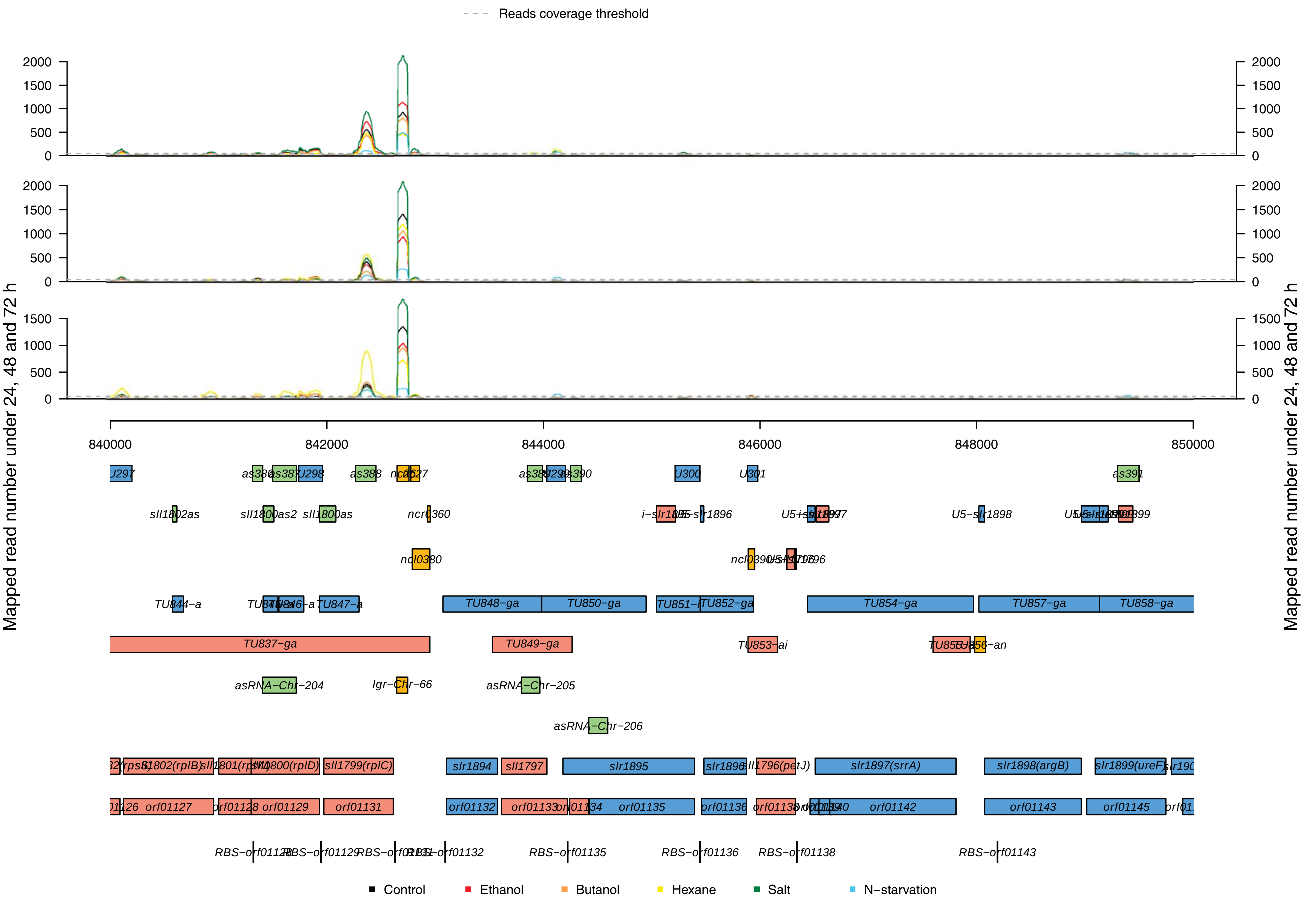

Reads coverage threshold
Mapped read number under 24, 48 and 72 h
2000
1500
1000
500
0
840000
842000
844000
846000
848000
850000
TU837−ga
TU848−ga
TU850−ga
TU854−ga
TU857−ga
TU858−ga
TU849−ga
asRNA−Chr−204
asRNA−Chr−205
asRNA−Chr−206
slr1894
sll1797
slr1895
slr1897(srrA)
slr1898(argB)
slr1899(ureF)
orf01127
orf01129
orf01131
orf01132
orf01135
orf01142
orf01143
orf01145
RBS−orf01135
RBS−orf01136
RBS−orf01138
RBS−orf01143
Control
Ethanol
Butanol
Hexane
Salt
N−starvation

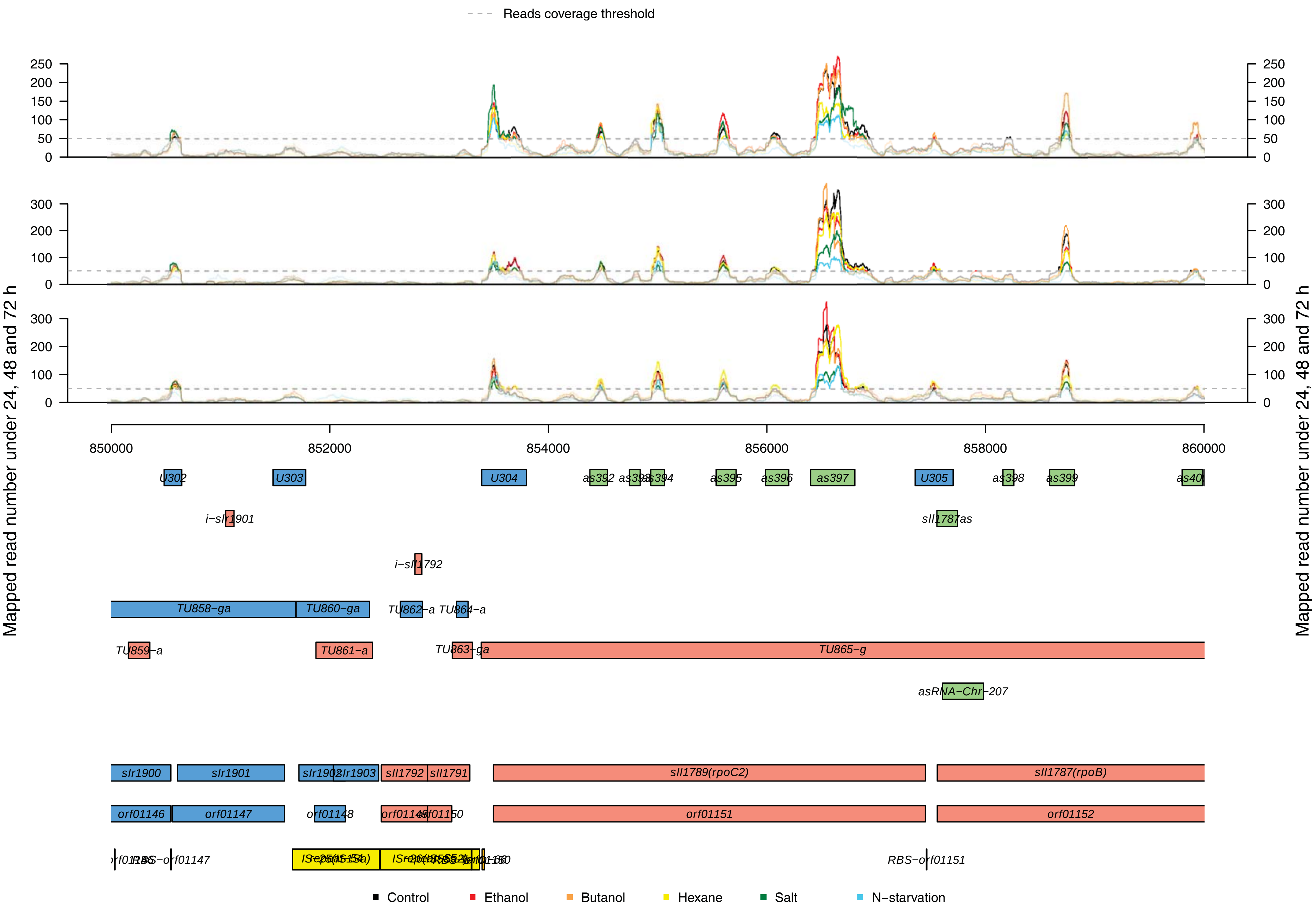

Reads coverage threshold
Mapped read number under 24, 48 and 72 h
250
200
150
100
50
0
300
200
100
0
850000
852000
854000
856000
858000
860000
U302
U303
U304
as392
as394
as395
as396
as397
U305
as398
as399
i−slr1901
sll1787as
i−sll1792
TU858−ga
TU860−ga
TU862−a
TU864−a
TU859−a
TU861−a
TU863−ga
TU865−g
asRNA−Chr−207
slr1900
slr1901
slr1902
slr1903
sll1792
sll1791
sll1789(rpoC2)
sll1787(rpoB)
orf01146
orf01147
orf01148
orf01151
orf01152
RBS−orf01147
RBS−orf01151
Control
Ethanol
Butanol
Hexane
Salt
N−starvation

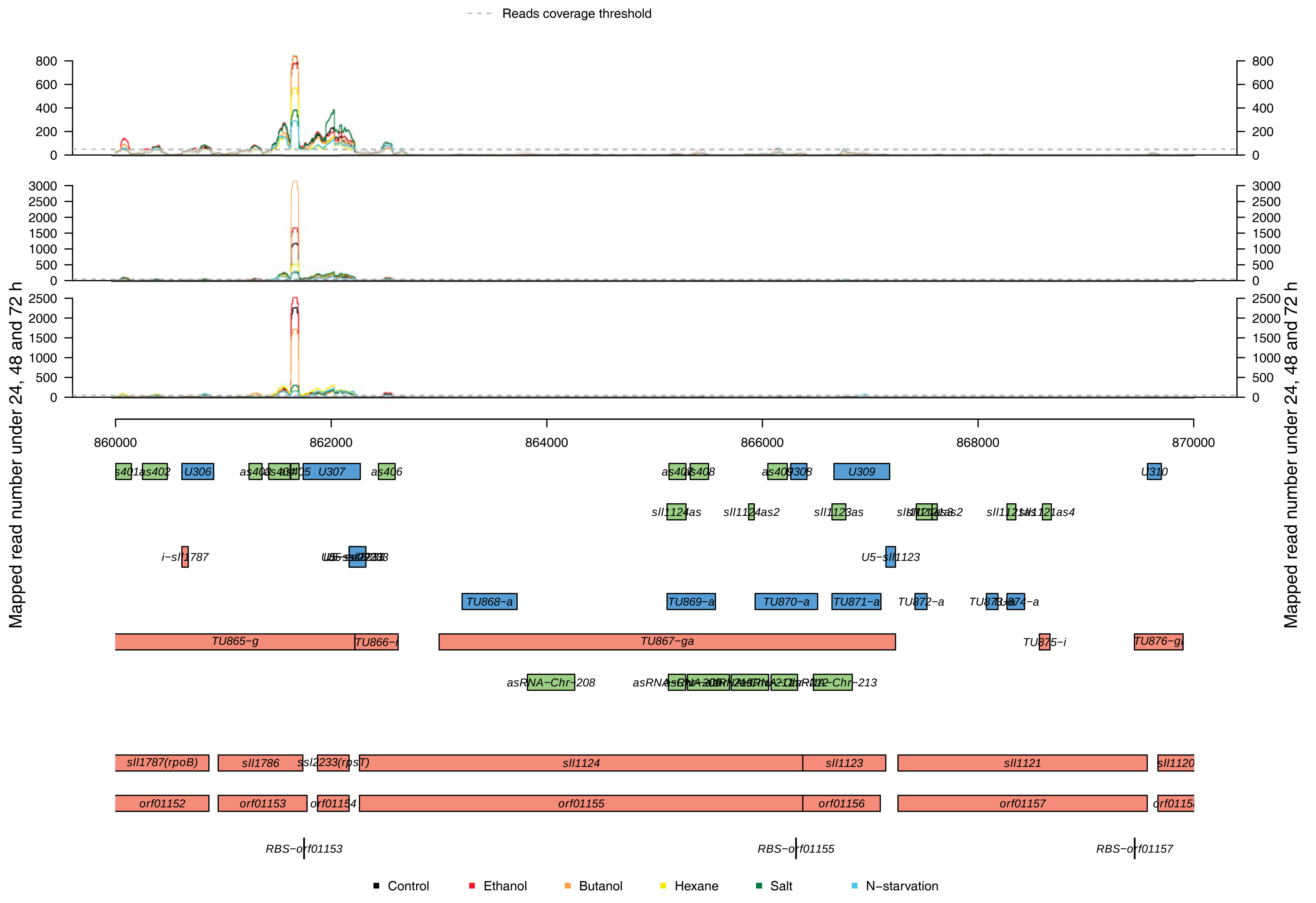

Reads coverage threshold
Mapped read number under 24, 48 and 72 h
860000
862000
864000
866000
868000
870000
U306
U307
U309
U310
i−sll1787
U5−sll1123
TU865−g
TU867−ga
TU868−a
TU869−a
TU870−a
TU871−a
asRNA−Chr−208
sll1124as
sll1123as
sll1787(rpoB)
sll1786
sll1124
sll1123
sll1121
orf01152
orf01153
orf01155
orf01156
orf01157
RBS−orf01153
RBS−orf01155
RBS−orf01157
Control
Ethanol
Butanol
Hexane
Salt
N−starvation

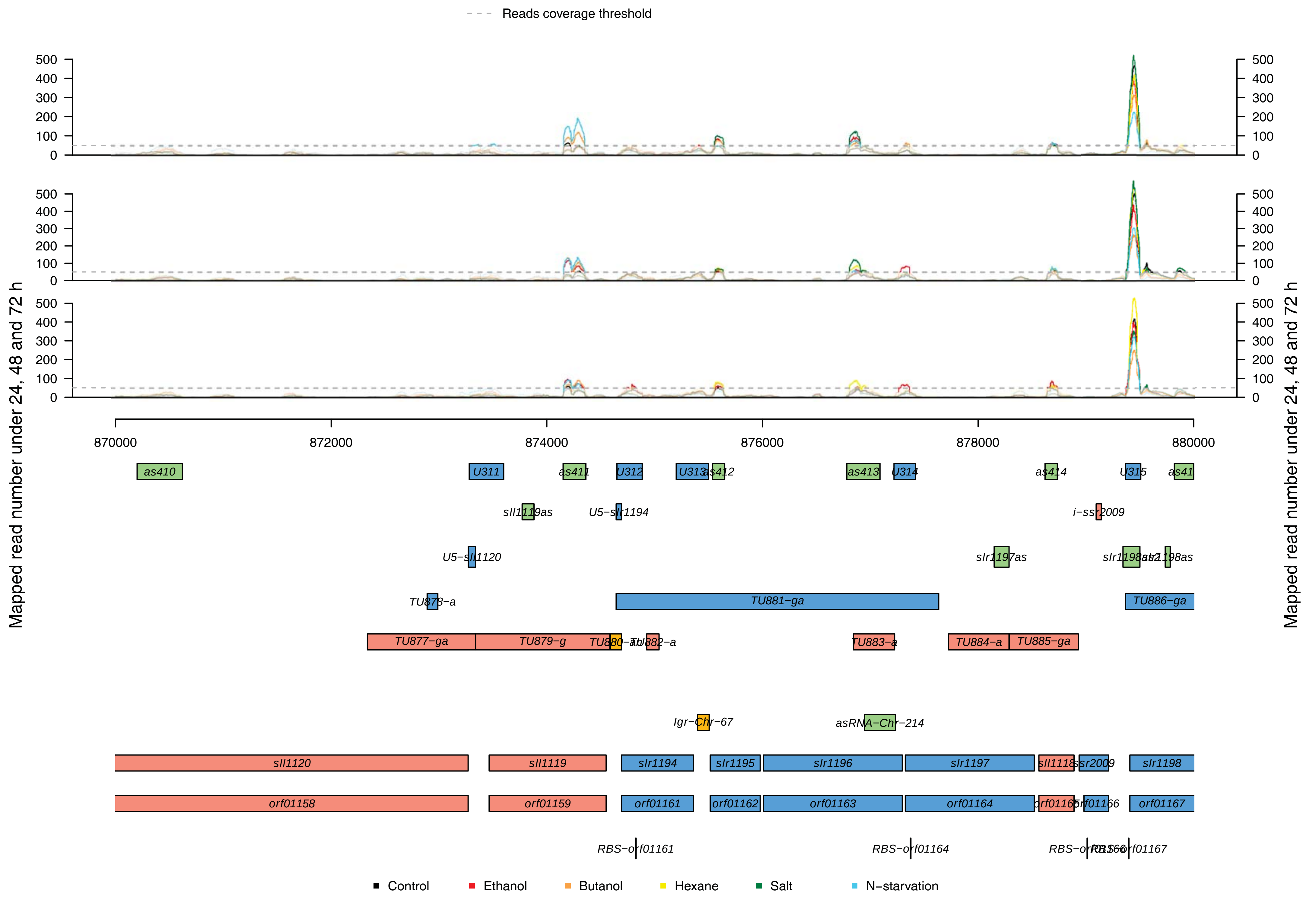

Reads coverage threshold
Mapped read number under 24, 48 and 72 h
870000
872000
874000
876000
878000
880000
as410
U311
as411
U312
U313
as412
as413
U314
as414
U315
sll1119as
U5−slr1194
i−ssr2009
U5−sll1120
slr1197as
TU878−a
TU881−ga
TU886−ga
TU877−ga
TU879−g
TU884−a
TU885−ga
Igr−Chr−67
asRNA−Chr−214
sll1120
sll1119
slr1194
slr1195
slr1196
slr1197
slr1198
orf01158
orf01159
orf01161
orf01162
orf01163
orf01164
orf01167
RBS−orf01161
RBS−orf01164
Control
Ethanol
Butanol
Hexane
Salt
N−starvation

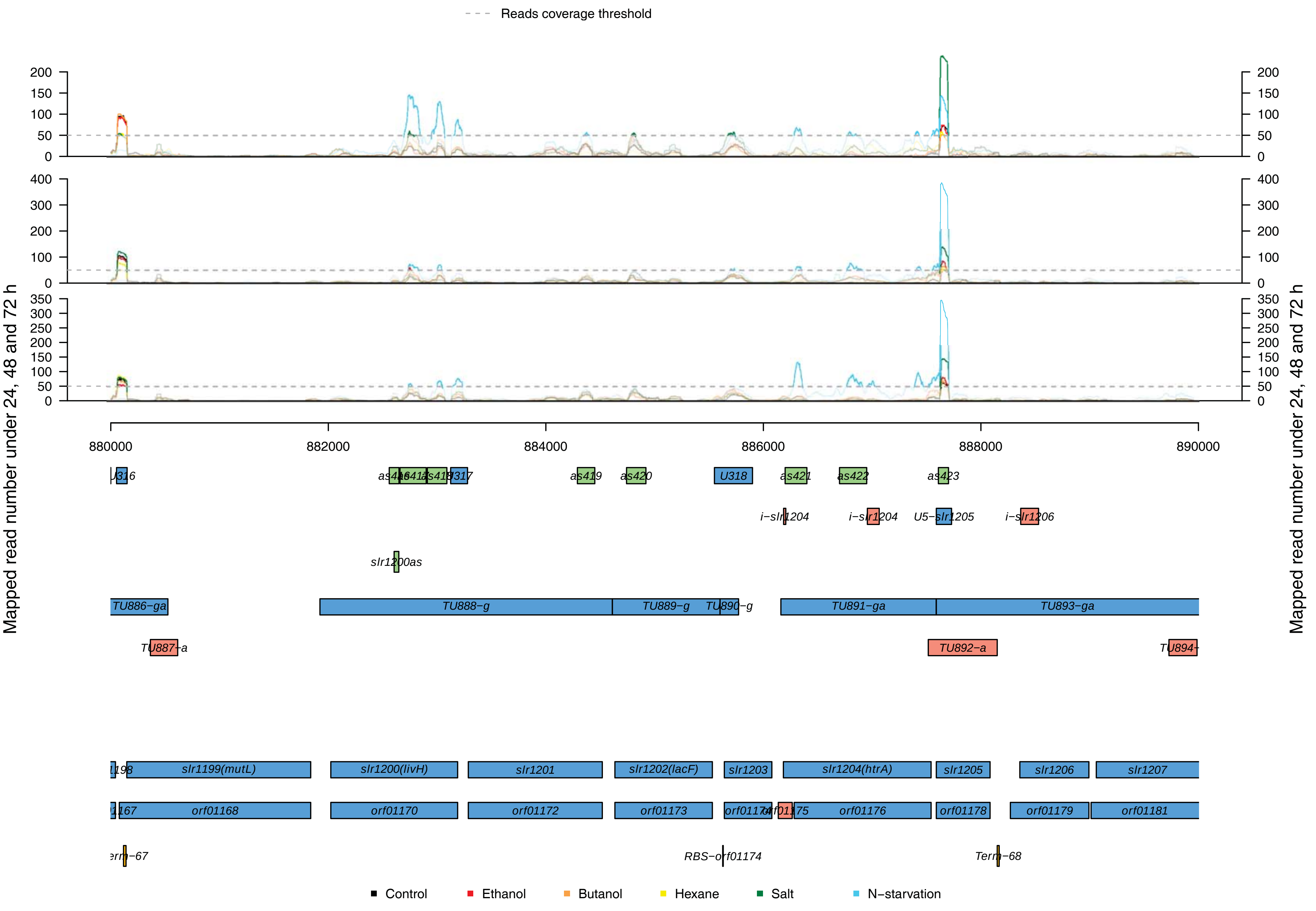

Reads coverage threshold
Mapped read number under 24, 48 and 72 h
880000
882000
884000
886000
888000
890000
U318
as419
as420
as421
as422
as423
i−slr1204
U5−slr1205
i−slr1206
slr1200as
TU886−ga
TU888−g
TU889−g
TU891−ga
TU893−ga
TU887−a
TU892−a
slr1199(mutL)
slr1200(livH)
slr1201
slr1202(lacF)
slr1203
slr1204(htrA)
slr1205
slr1206
slr1207
orf01168
orf01170
orf01172
orf01173
orf01176
orf01178
orf01179
orf01181
RBS−orf01174
Term−68
Control
Ethanol
Butanol
Hexane
Salt
N−starvation

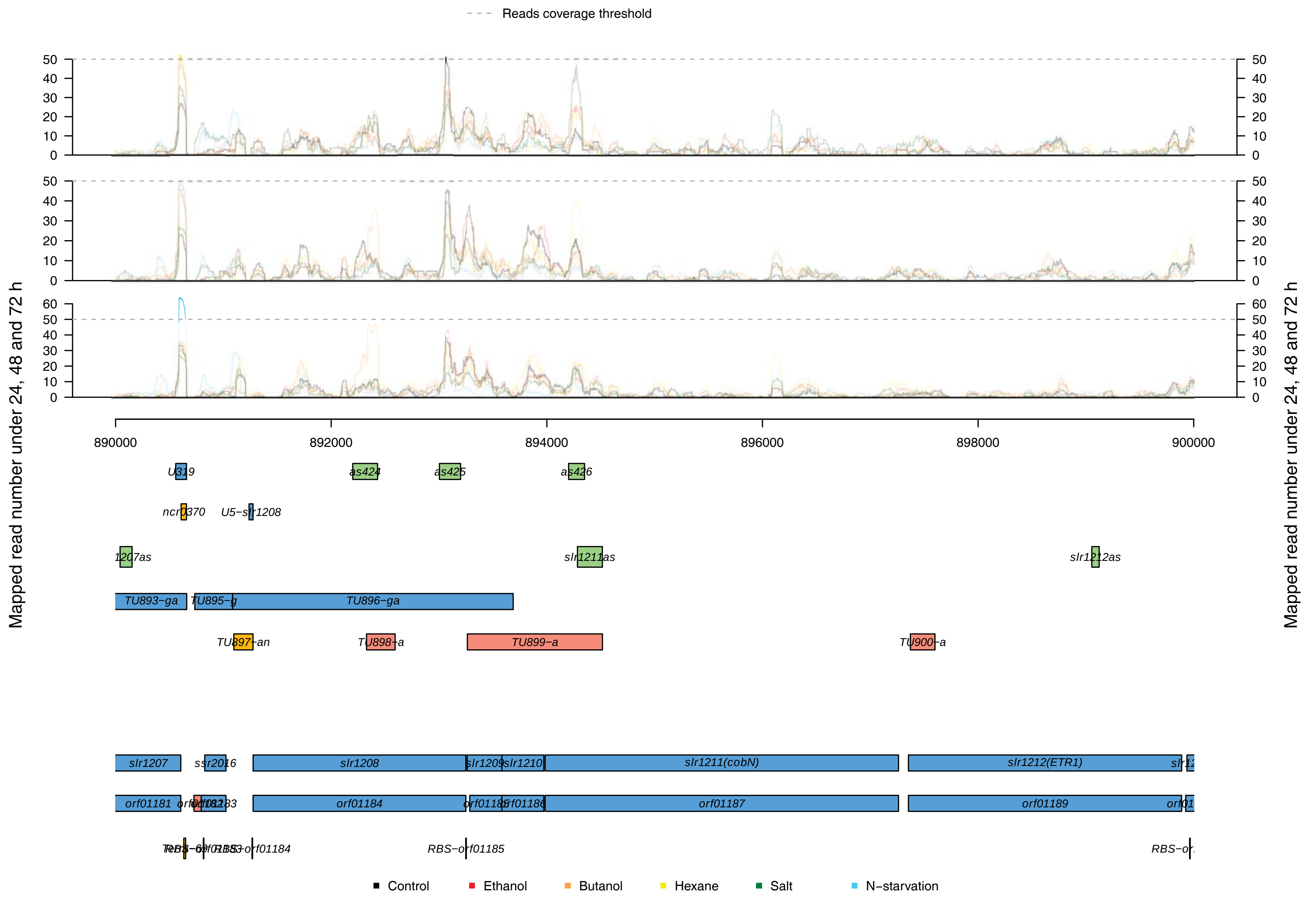

Reads coverage threshold
Mapped read number under 24, 48 and 72 h
890000
892000
894000
896000
898000
900000
U319
as424
as425
as426
ncr0370
U5−slr1208
1207as
slr1211as
slr1212as
TU893−ga
TU895−g
TU896−ga
TU897−an
TU898−a
TU899−a
TU900−a
slr1207
ssr2016
slr1208
slr1209
slr1210
slr1211(cobN)
slr1212(ETR1)
orf01181
orf01184
orf01187
orf01189
RBS−orf01184
RBS−orf01185
Control
Ethanol
Butanol
Hexane
Salt
N−starvation

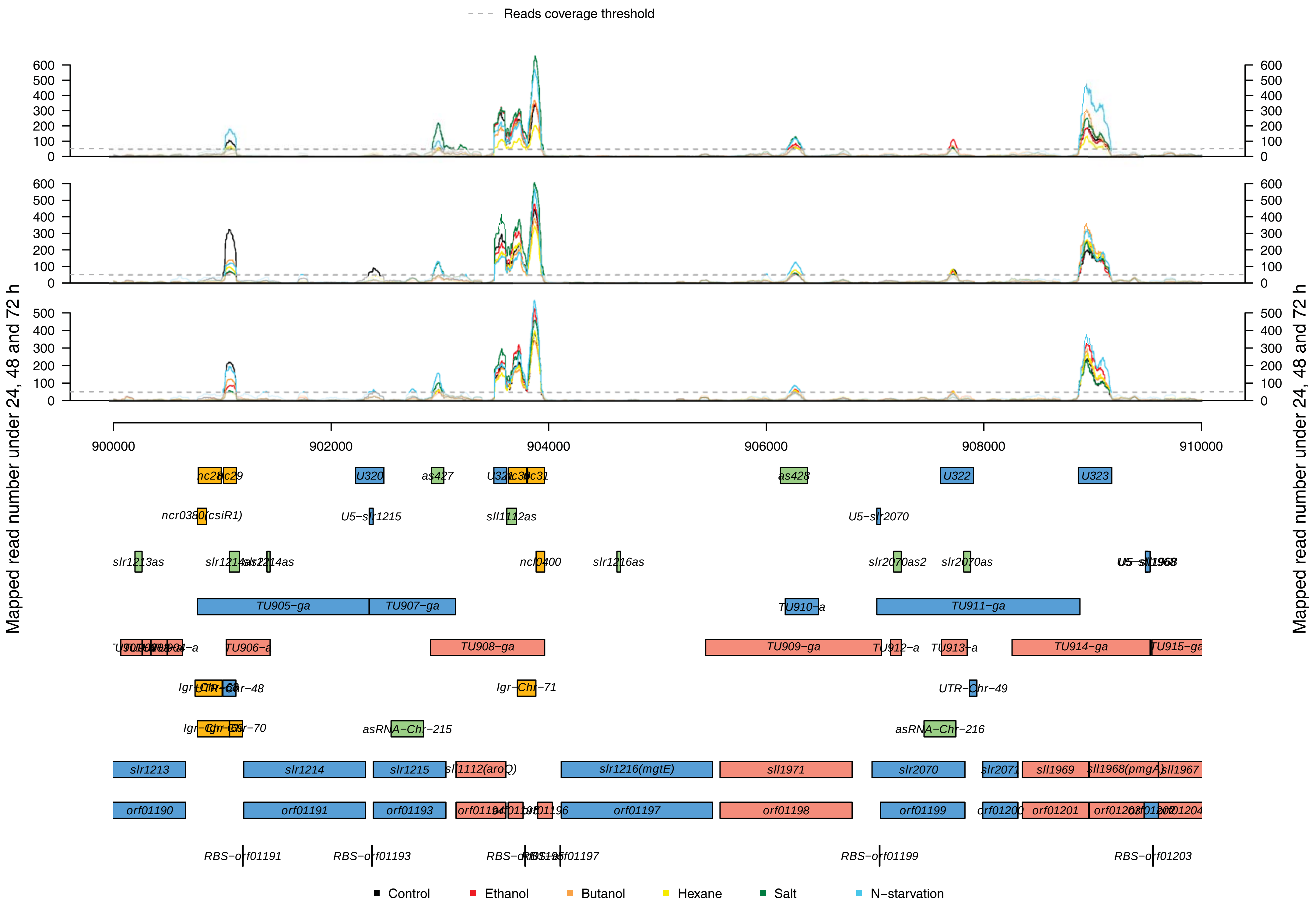

Reads coverage threshold
Mapped read number under 24, 48 and 72 h
900000
902000
904000
906000
908000
910000
U320
as427
as428
U322
U323
ncr0380(csiR1)
U5−slr1215
sll1112as
U5−slr2070
slr1213as
ncl0400
slr1216as
slr2070as2
slr2070as
TU905−ga
TU907−ga
TU910−a
TU911−ga
TU906−a
TU908−ga
TU909−ga
TU912−a
TU913−a
TU914−ga
TU915−ga
Igr−Chr−71
UTR−Chr−49
asRNA−Chr−215
asRNA−Chr−216
slr1213
slr1214
slr1215
sll1112(aroQ)
slr1216(mgtE)
sll1971
slr2070
slr2071
sll1969
sll1968(pmgA)
sll1967
orf01190
orf01191
orf01193
orf01197
orf01198
orf01199
orf01200
orf01201
orf01204
RBS−orf01191
RBS−orf01193
RBS−orf01199
RBS−orf01203
Control
Ethanol
Butanol
Hexane
Salt
N−starvation

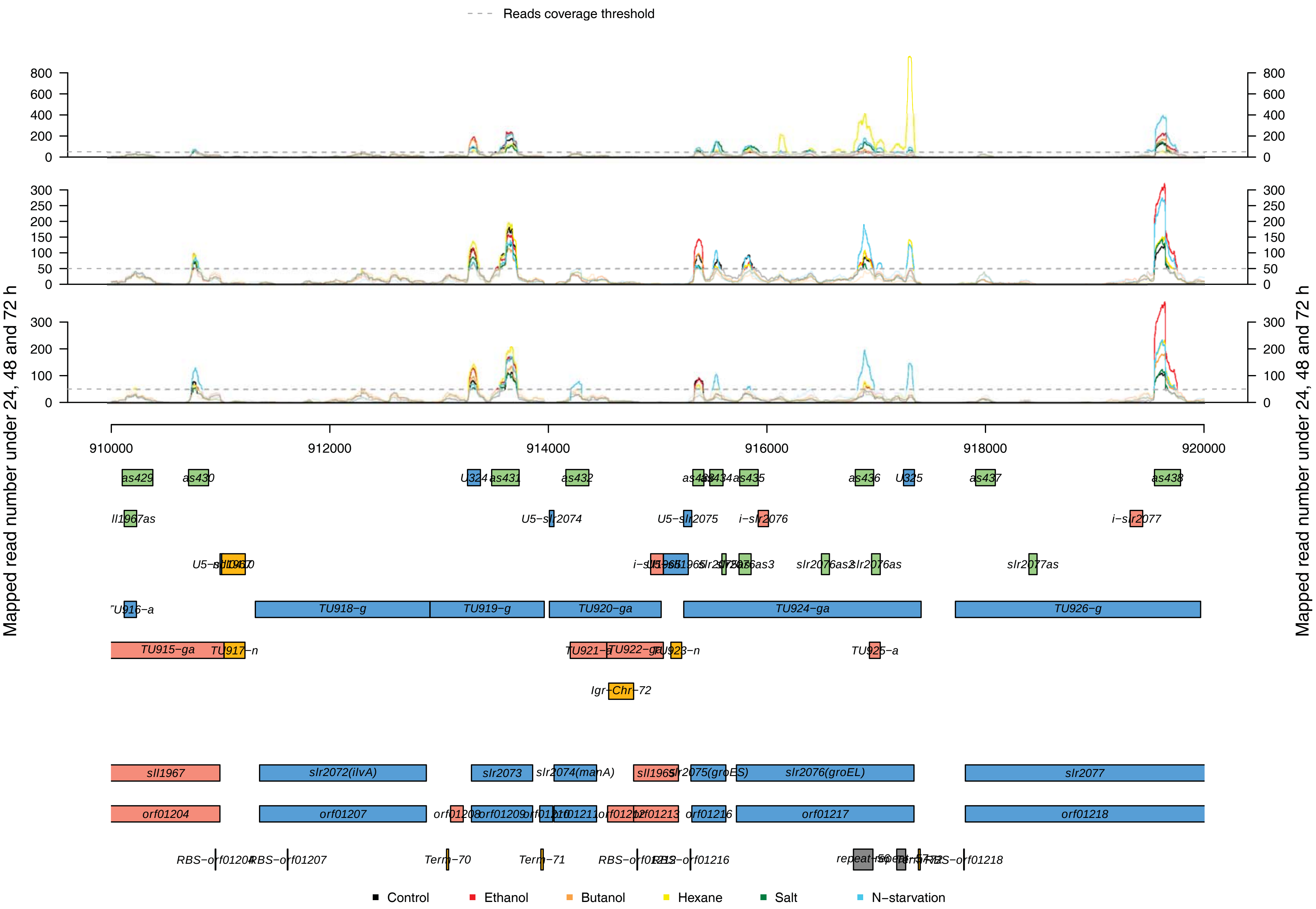

Reads coverage threshold
Mapped read number under 24, 48 and 72 h
800
600
400
200
0
300
250
200
150
100
50
0
300
200
100
0
910000
912000
914000
916000
918000
920000
as429
as430
U324
as431
as432
as435
as436
U325
as437
as438
ll1967as
U5−slr2074
U5−sll2075
i−slr2076
i−slr2077
slr2076as2
slr2076as
slr2077as
U916−a
TU918−g
TU919−g
TU920−ga
TU924−ga
TU926−g
TU915−ga
TU925−a
Igr−Chr−72
sll1967
slr2072(ilvA)
slr2073
slr2074(manA)
slr2076(groEL)
slr2077
orf01204
orf01207
orf01208
orf01209
orf01211
orf01213
orf01216
orf01217
orf01218
RBS−orf01204
RBS−orf01207
Term−70
Term−71
RBS−orf01216
RBS−orf01218
Control
Ethanol
Butanol
Hexane
Salt
N−starvation

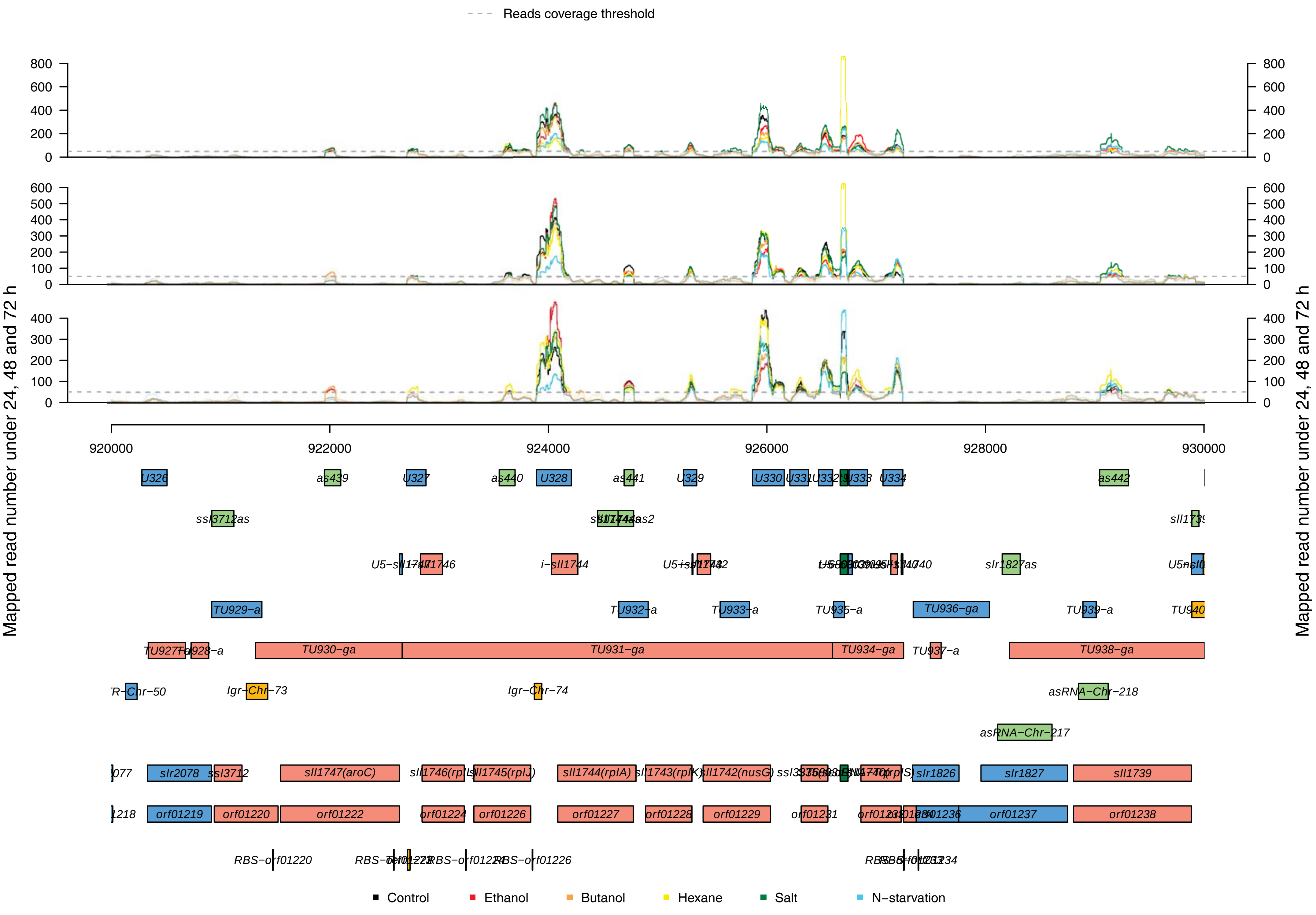

Reads coverage threshold
Mapped read number under 24, 48 and 72 h
920000
922000
924000
926000
928000
930000
U326
as439
U327
as440
U328
as441
U329
U330
U334
as442
ssl3712as
i−sll1744
slr1827as
TU929−a
TU932−a
TU933−a
TU935−a
TU936−ga
TU939−a
TU930−ga
TU931−ga
TU934−ga
TU938−ga
Igr−Chr−73
Igr−Chr−74
asRNA−Chr−218
asRNA−Chr−217
slr2078
ssl3712
sll1747(aroC)
sll1745(rplJ)
sll1744(rplA)
sll1742(nusG)
slr1826
slr1827
sll1739
orf01219
orf01220
orf01222
orf01224
orf01226
orf01227
orf01228
orf01229
orf01231
orf01237
orf01238
RBS−orf01220
RBS−orf01226
Control
Ethanol
Butanol
Hexane
Salt
N−starvation

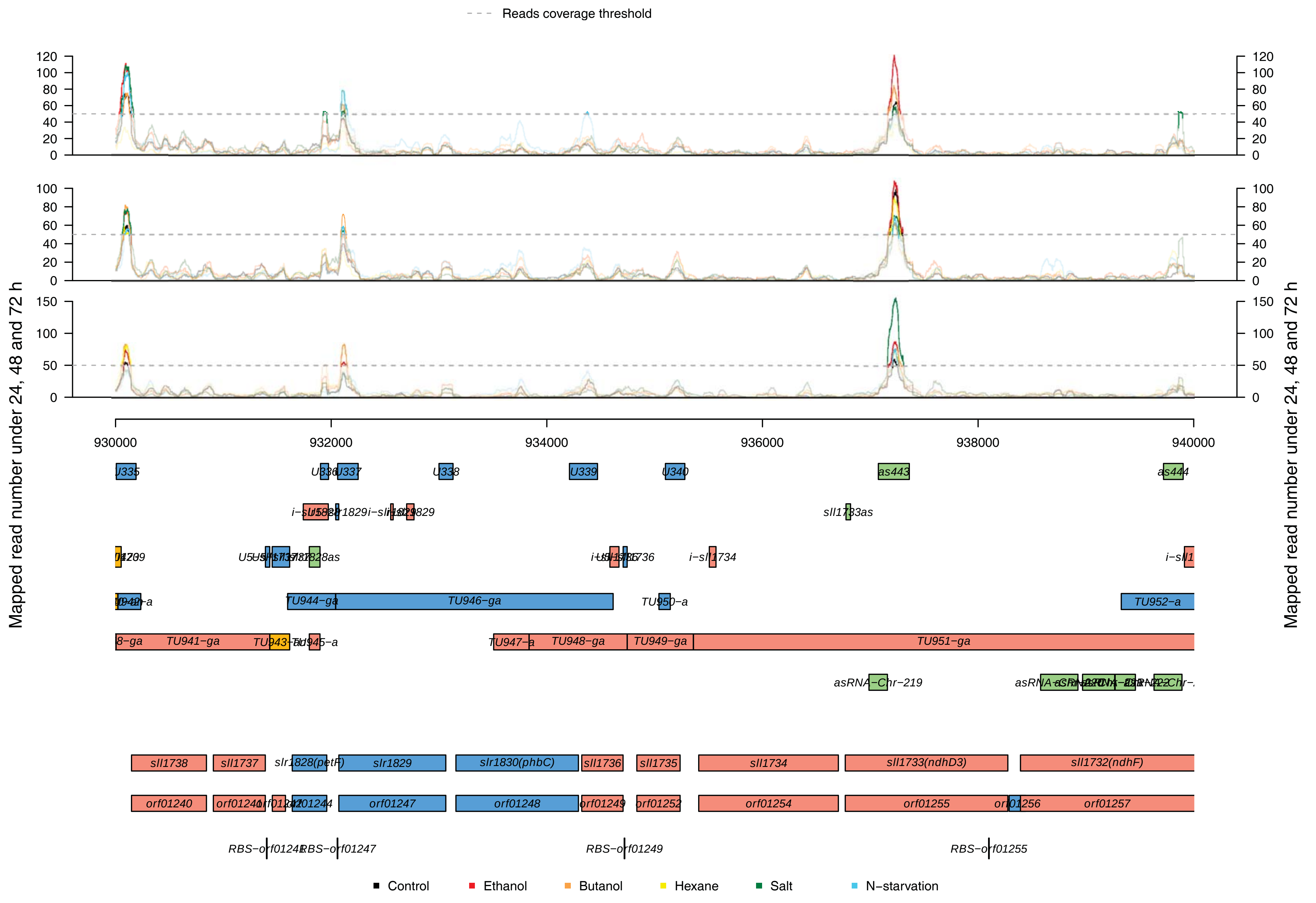

Reads coverage threshold
Mapped read number under 24, 48 and 72 h
930000
932000
934000
936000
938000
940000
sll1738
sll1737
slr1828(petF)
slr1829
slr1830(phbC)
sll1736
sll1735
sll1734
sll1733(ndhD3)
sll1732(ndhF)
orf01240
orf01247
orf01248
orf01252
orf01254
orf01255
orf01257
TU941−ga
TU944−ga
TU946−ga
TU948−ga
TU949−ga
TU951−ga
TU952−a
asRNA−Chr−219
Control
Ethanol
Butanol
Hexane
Salt
N−starvation

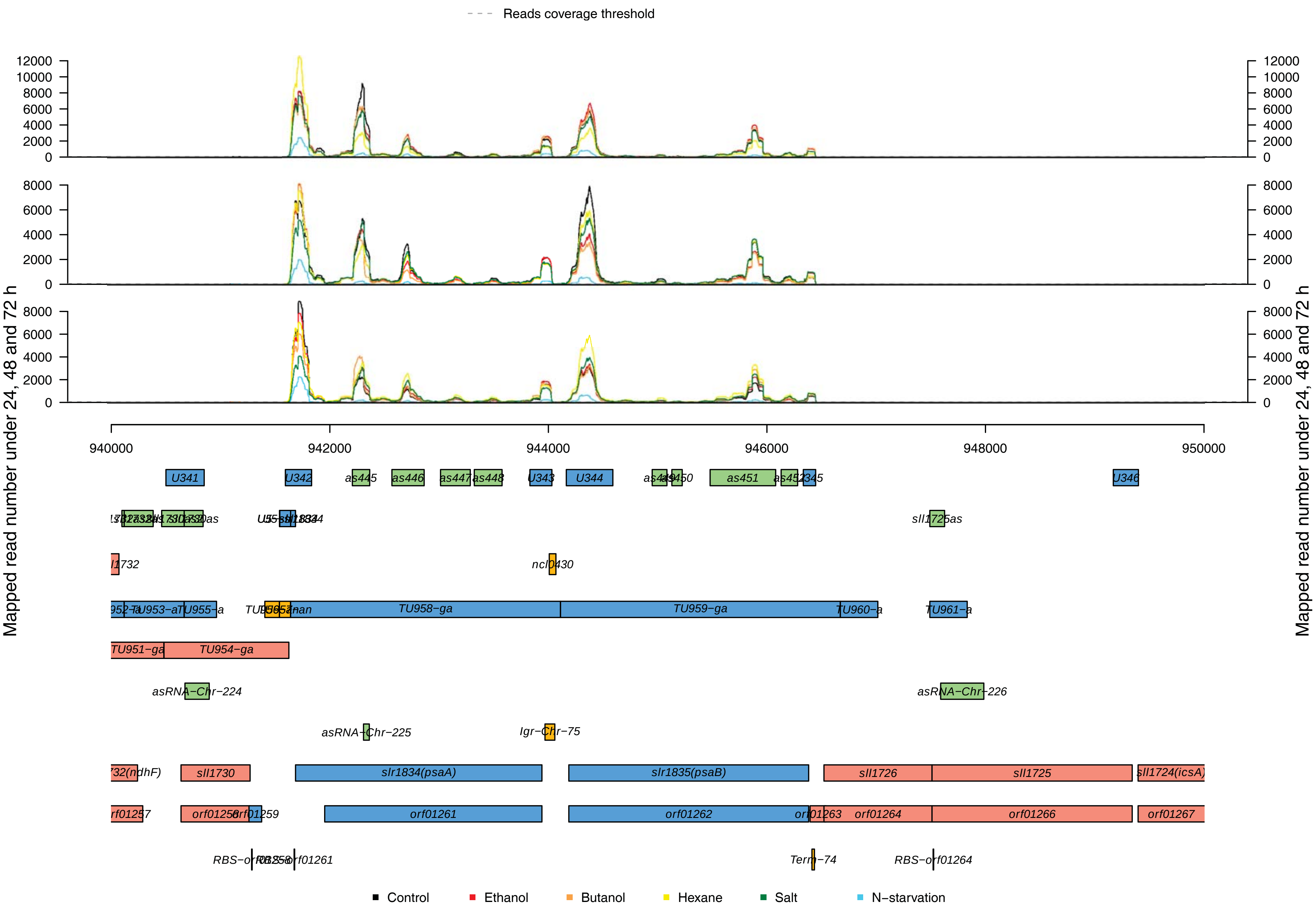

Reads coverage threshold
Mapped read number under 24, 48 and 72 h
940000
942000
944000
946000
948000
950000
U341
U342
as445
as446
as447
as448
U343
U344
as451
U346
sll1725as
ncl0430
TU958-ga
TU959-ga
TU960-a
TU961-a
TU951-ga
TU954-ga
asRNA-Chr-224
asRNA-Chr-226
asRNA-Chr-225
Igr-Chr-75
sll1730
slr1834(psaA)
slr1835(psaB)
sll1726
sll1725
sll1724(icsA)
orf01261
orf01262
orf01264
orf01266
orf01267
Term-74
RBS-orf01264
Control
Ethanol
Butanol
Hexane
Salt
N-starvation

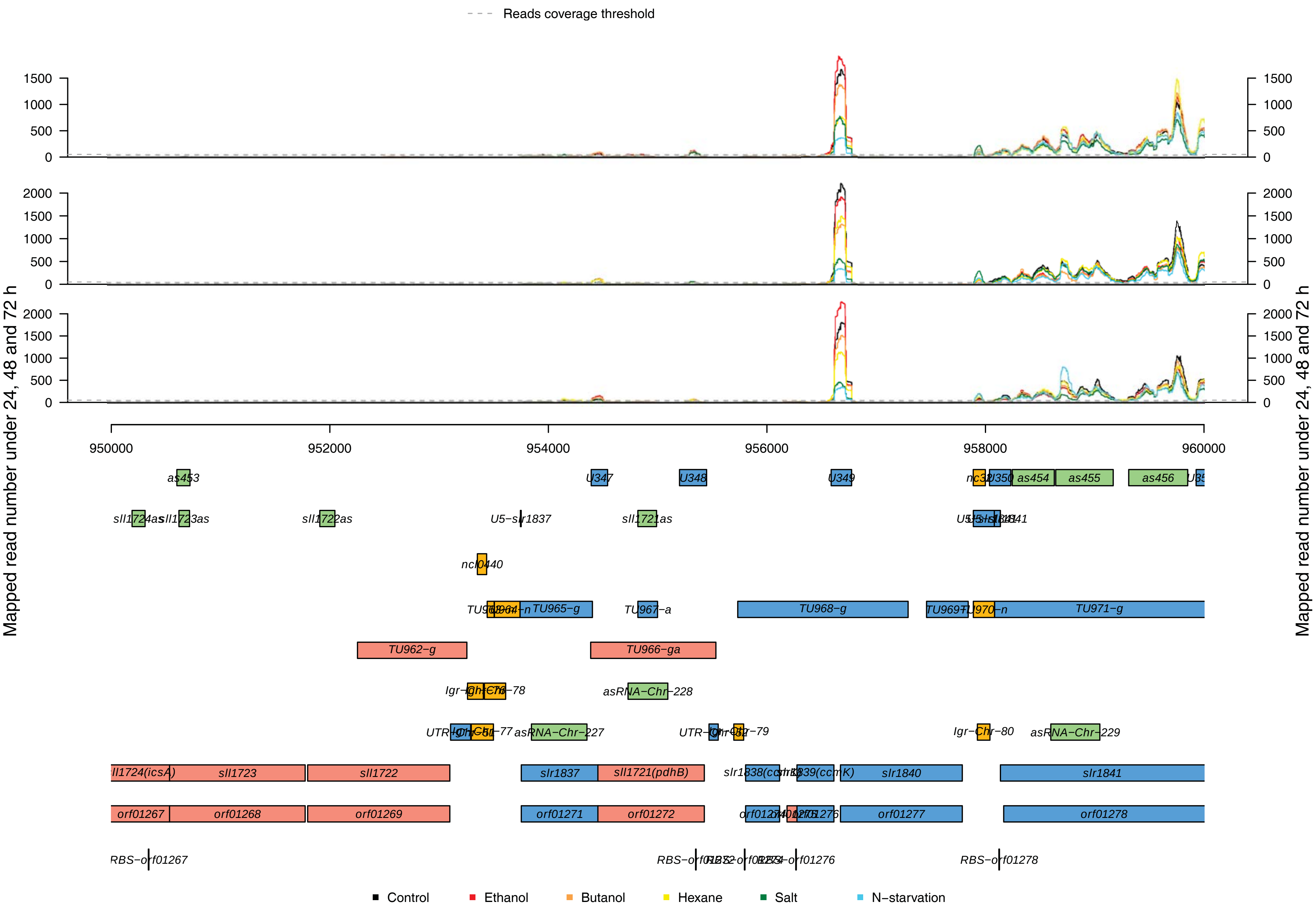

Reads coverage threshold
Mapped read number under 24, 48 and 72 h
0
500
1000
1500
2000
950000
952000
954000
956000
958000
960000
as453
U347
U348
U349
as454
as455
as456
sll1722as
U5−slr1837
sll1721as
ncl0440
TU965−g
TU967−a
TU968−g
TU971−g
TU962−g
TU966−ga
asRNA−Chr−228
asRNA−Chr−227
asRNA−Chr−229
Igr−Chr−80
sll1723
sll1722
slr1837
sll1721(pdhB)
slr1840
slr1841
orf01267
orf01268
orf01269
orf01271
orf01272
orf01277
orf01278
RBS−orf01267
RBS−orf01278
Control
Ethanol
Butanol
Hexane
Salt
N−starvation

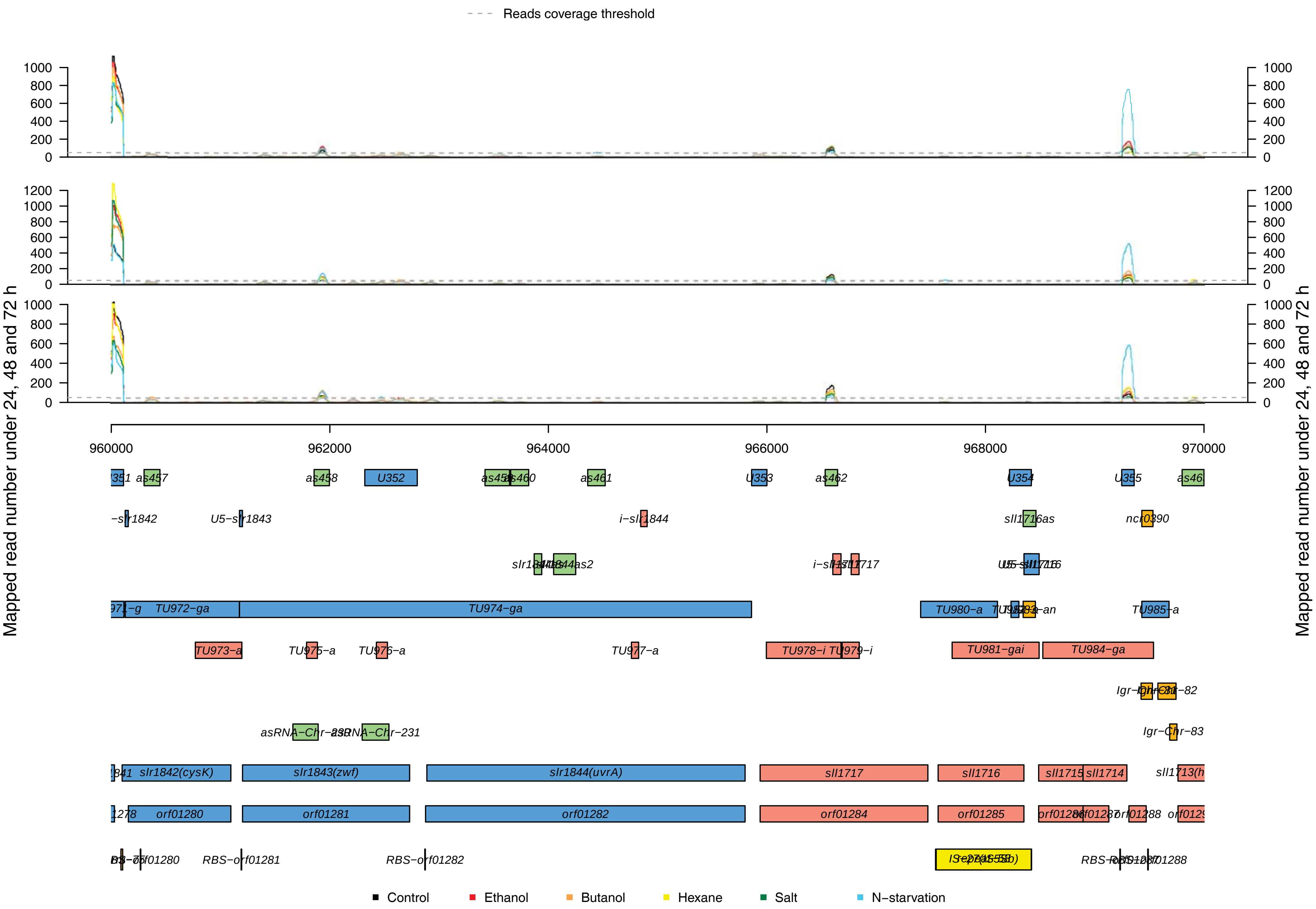

Reads coverage threshold
Mapped read number under 24, 48 and 72 h
1000
800
600
400
200
0
1200
960000
962000
964000
966000
968000
970000
as457
as458
U352
as460
as461
U353
as462
U354
U355
U5−slr1843
i−slr1844
sll1716as
ncl0390
TU972−ga
TU974−ga
TU980−a
TU985−a
TU973−a
TU975−a
TU976−a
TU977−a
TU978−i
TU979−i
TU981−gai
TU984−ga
Igr−Chr−83
asRNA−Chr−231
slr1842(cysK)
slr1843(zwf)
slr1844(uvrA)
sll1717
sll1716
sll1715
sll1714
orf01280
orf01281
orf01282
orf01284
orf01285
RBS−orf01281
RBS−orf01282
Control
Ethanol
Butanol
Hexane
Salt
N−starvation

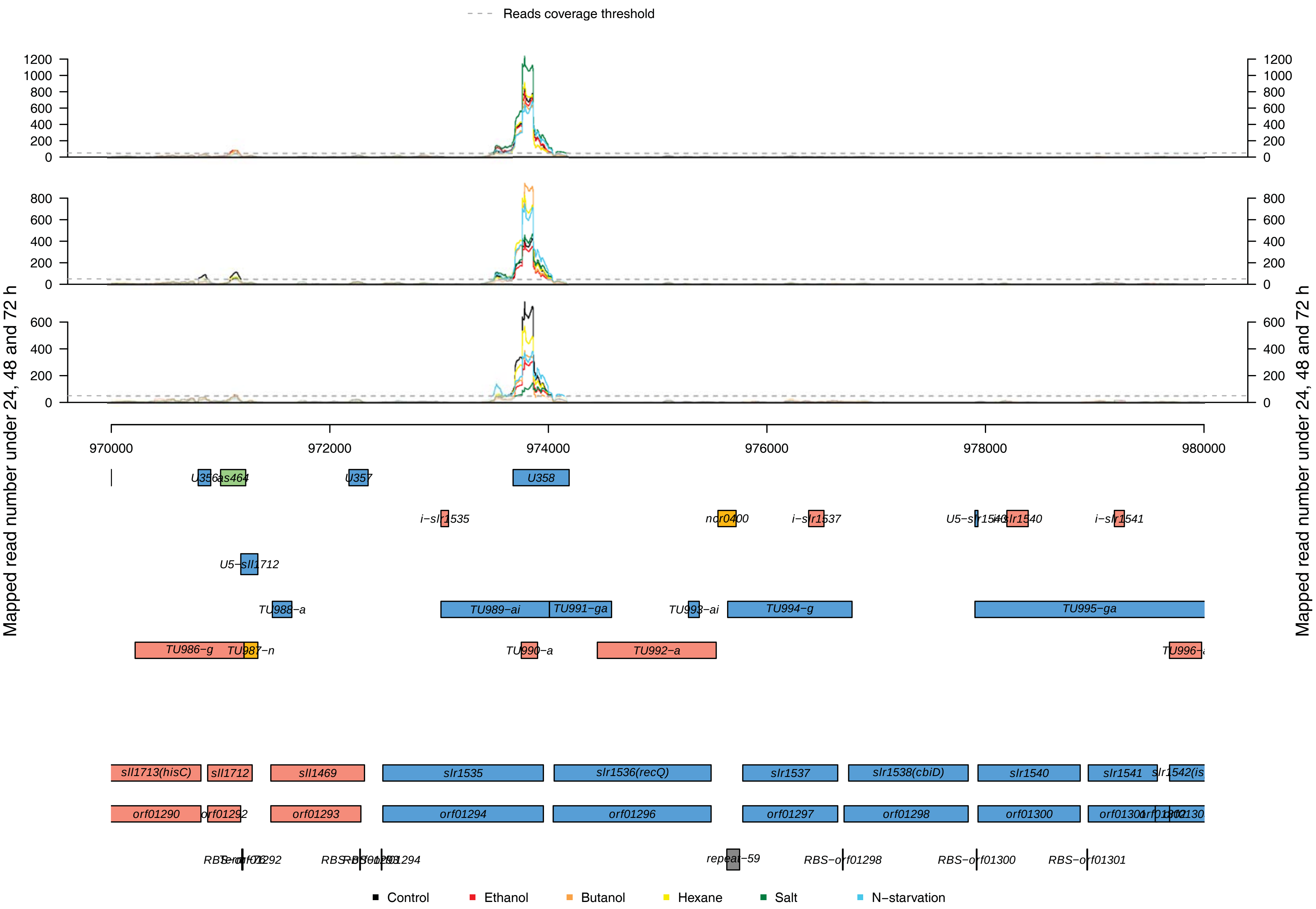

Reads coverage threshold
Mapped read number under 24, 48 and 72 h
970000
972000
974000
976000
978000
980000
U358
i−slr1535
ncr0400
i−slr1537
i−slr1541
U5−sll1712
TU988−a
TU989−ai
TU991−ga
TU994−g
TU995−ga
TU986−g
TU990−a
TU992−a
sll1713(hisC)
sll1712
sll1469
slr1535
slr1536(recQ)
slr1537
slr1538(cbiD)
slr1540
slr1541
orf01290
orf01293
orf01294
orf01296
orf01297
orf01298
orf01300
RBS−orf01298
RBS−orf01300
RBS−orf01301
Control
Ethanol
Butanol
Hexane
Salt
N−starvation

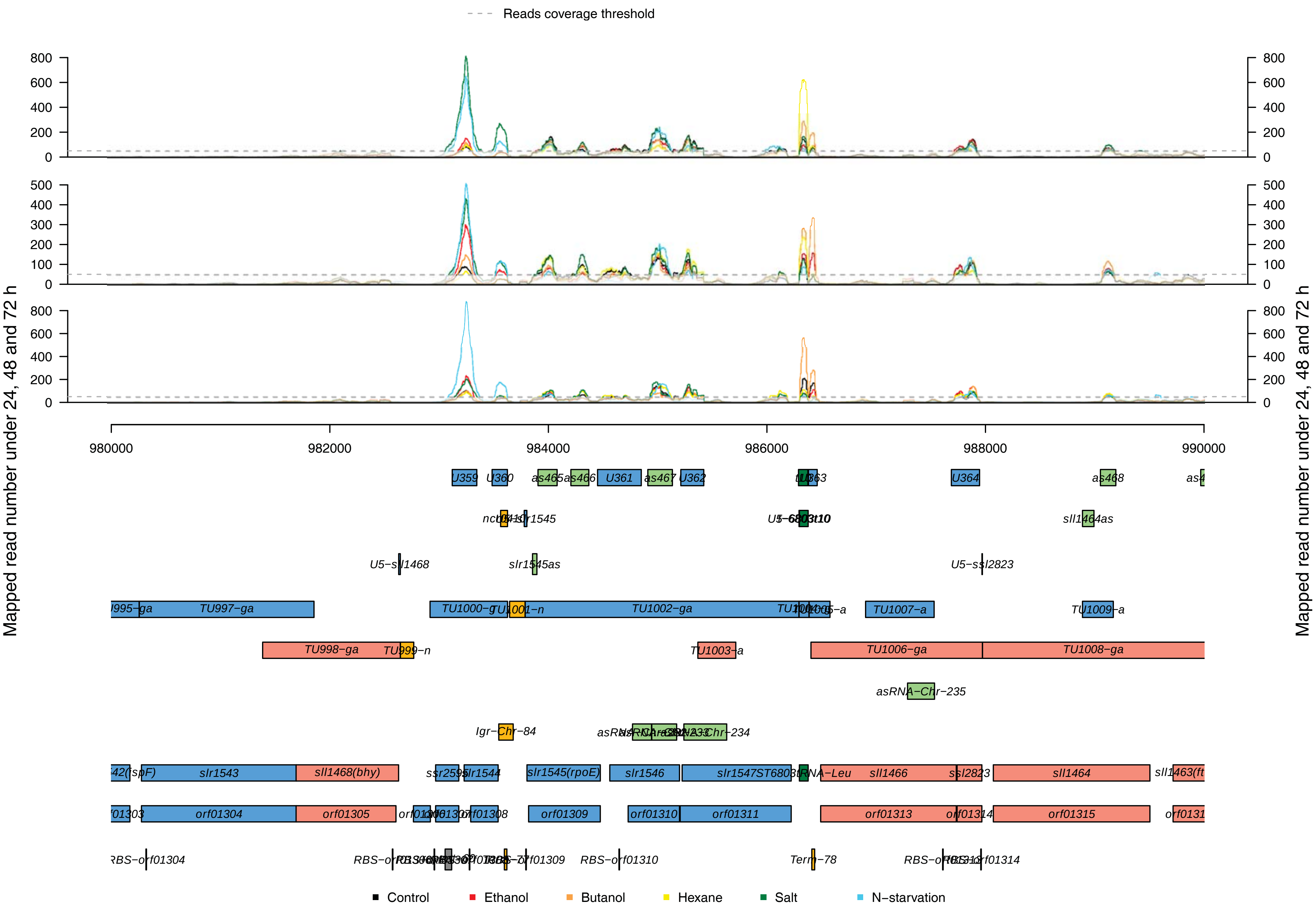

Reads coverage threshold
Mapped read number under 24, 48 and 72 h
980000
982000
984000
986000
988000
990000
TU997−ga
TU998−ga
TU1002−ga
TU1006−ga
TU1007−a
TU1008−ga
asRNA−Chr−235
Igr−Chr−84
slr1545as
U5−sll1468
U5−ssl2823
sll1464as
slr1543
sll1468(bhy)
slr1545(rpoE)
slr1546
sll1466
sll1464
orf01304
orf01305
orf01309
orf01311
orf01313
orf01315
RBS−orf01310
Term−78
Control
Ethanol
Butanol
Hexane
Salt
N−starvation

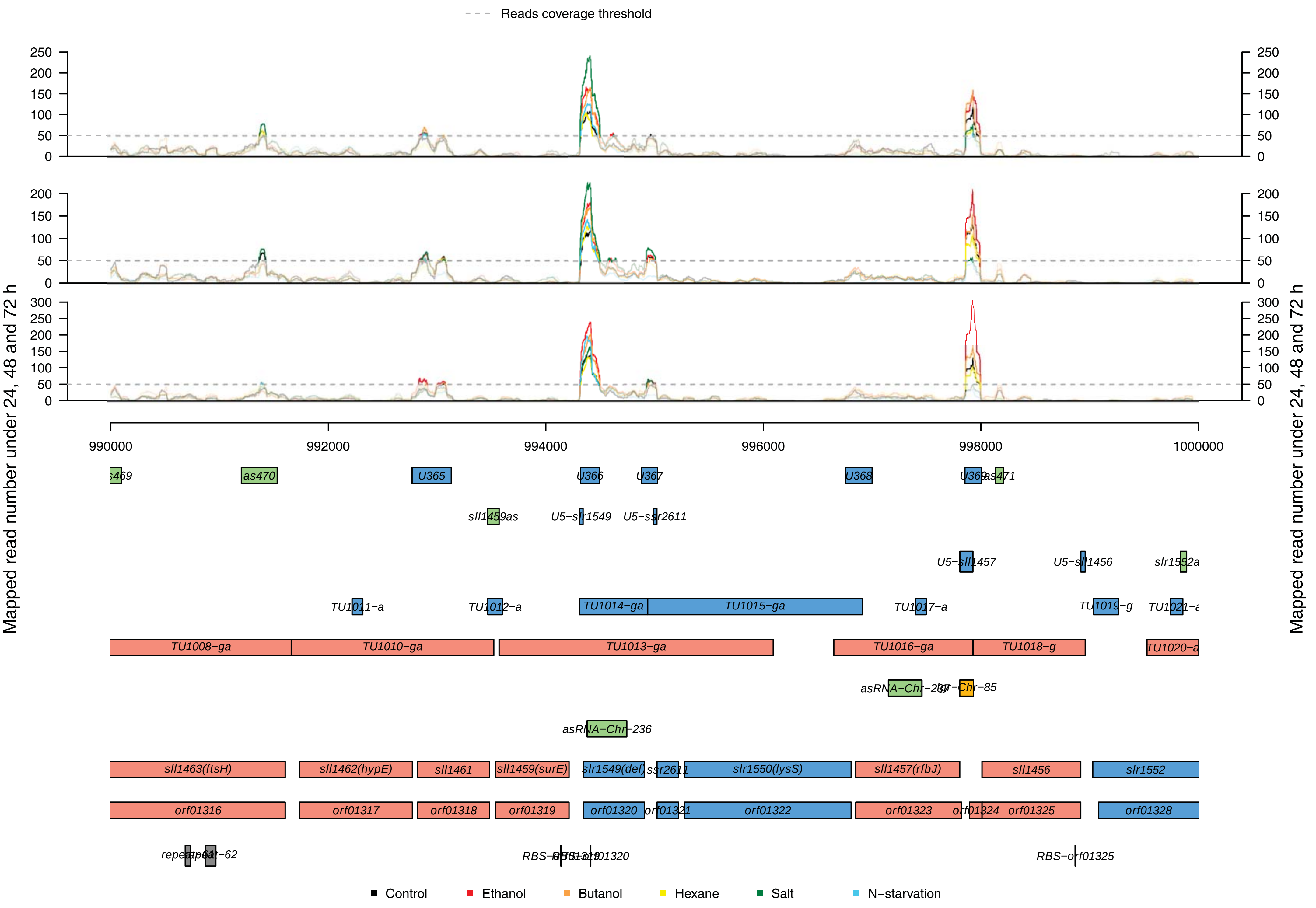

Reads coverage threshold
Mapped read number under 24, 48 and 72 h
990000
992000
994000
996000
998000
1000000
as470
U365
U366
U367
U368
sll1459as
U5−slr1549
U5−ssr2611
U5−sll1457
U5−sll1456
TU1011−a
TU1012−a
TU1014−ga
TU1015−ga
TU1017−a
TU1008−ga
TU1010−ga
TU1013−ga
TU1016−ga
TU1018−g
asRNA−Chr−236
sll1463(ftsH)
sll1462(hypE)
sll1461
sll1459(surE)
slr1550(lysS)
sll1457(rfbJ)
sll1456
slr1552
orf01316
orf01317
orf01318
orf01319
orf01320
orf01322
orf01323
orf01325
orf01328
RBS−orf01325
Control
Ethanol
Butanol
Hexane
Salt
N−starvation

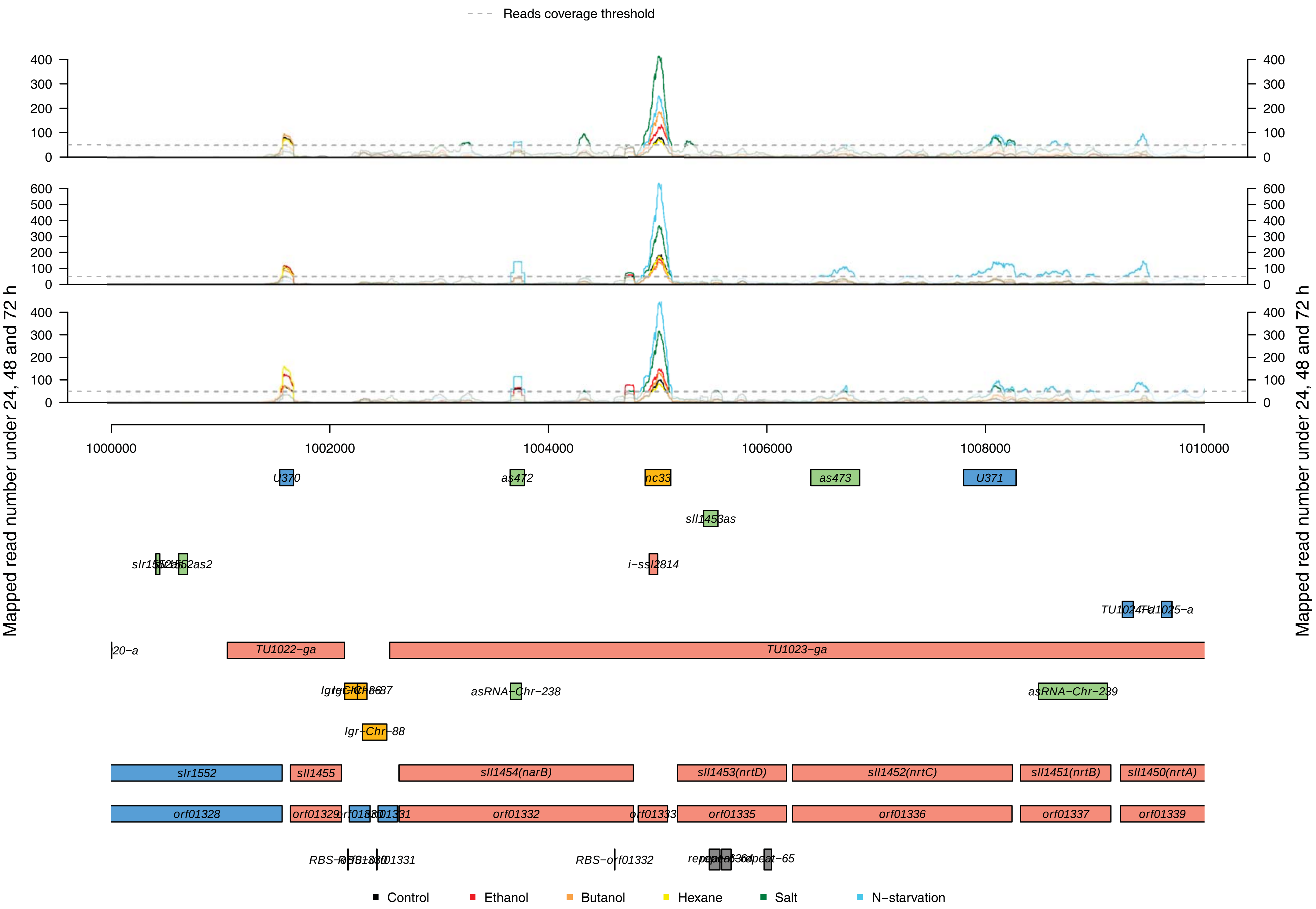

Reads coverage threshold
Mapped read number under 24, 48 and 72 h
1000000
1002000
1004000
1006000
1008000
1010000
U370
as472
nc33
as473
U371
sll1453as
i−ssl2814
TU1022−ga
TU1023−ga
asRNA−Chr−238
asRNA−Chr−239
Igr−Chr−88
slr1552
sll1455
sll1454(narB)
sll1453(nrtD)
sll1452(nrtC)
sll1451(nrtB)
sll1450(nrtA)
orf01328
orf01329
orf01332
orf01333
orf01335
orf01336
orf01337
orf01339
RBS−orf01332
Control
Ethanol
Butanol
Hexane
Salt
N−starvation

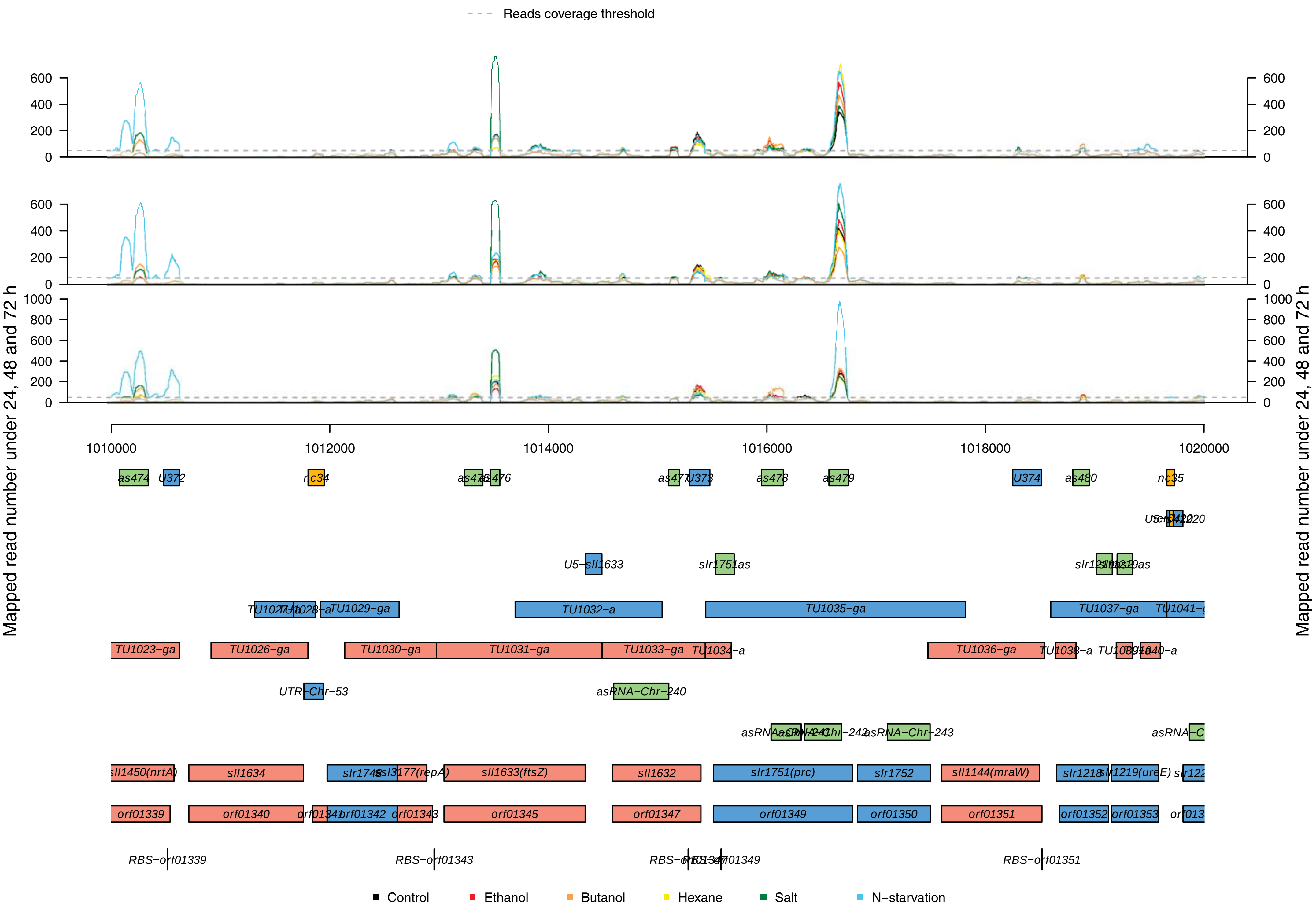

Reads coverage threshold
Mapped read number under 24, 48 and 72 h
1010000
1012000
1014000
1016000
1018000
1020000
as474
U372
nc34
as476
U373
as478
as479
U374
as480
nc35
U5-sll1633
slr1751as
TU1029-ga
TU1032-a
TU1035-ga
TU1037-ga
TU1023-ga
TU1026-ga
TU1030-ga
TU1031-ga
TU1033-ga
TU1034-a
TU1036-ga
TU1038-a
UTR-Chr-53
asRNA-Chr-240
asRNA-Chr-243
sll1450(nrtA)
sll1634
sll1633(ftsZ)
sll1632
slr1751(prc)
slr1752
sll1144(mraW)
orf01339
orf01340
orf01342
orf01345
orf01347
orf01349
orf01350
orf01351
orf01352
orf01353
RBS-orf01339
RBS-orf01343
RBS-orf01351
Control
Ethanol
Butanol
Hexane
Salt
N-starvation

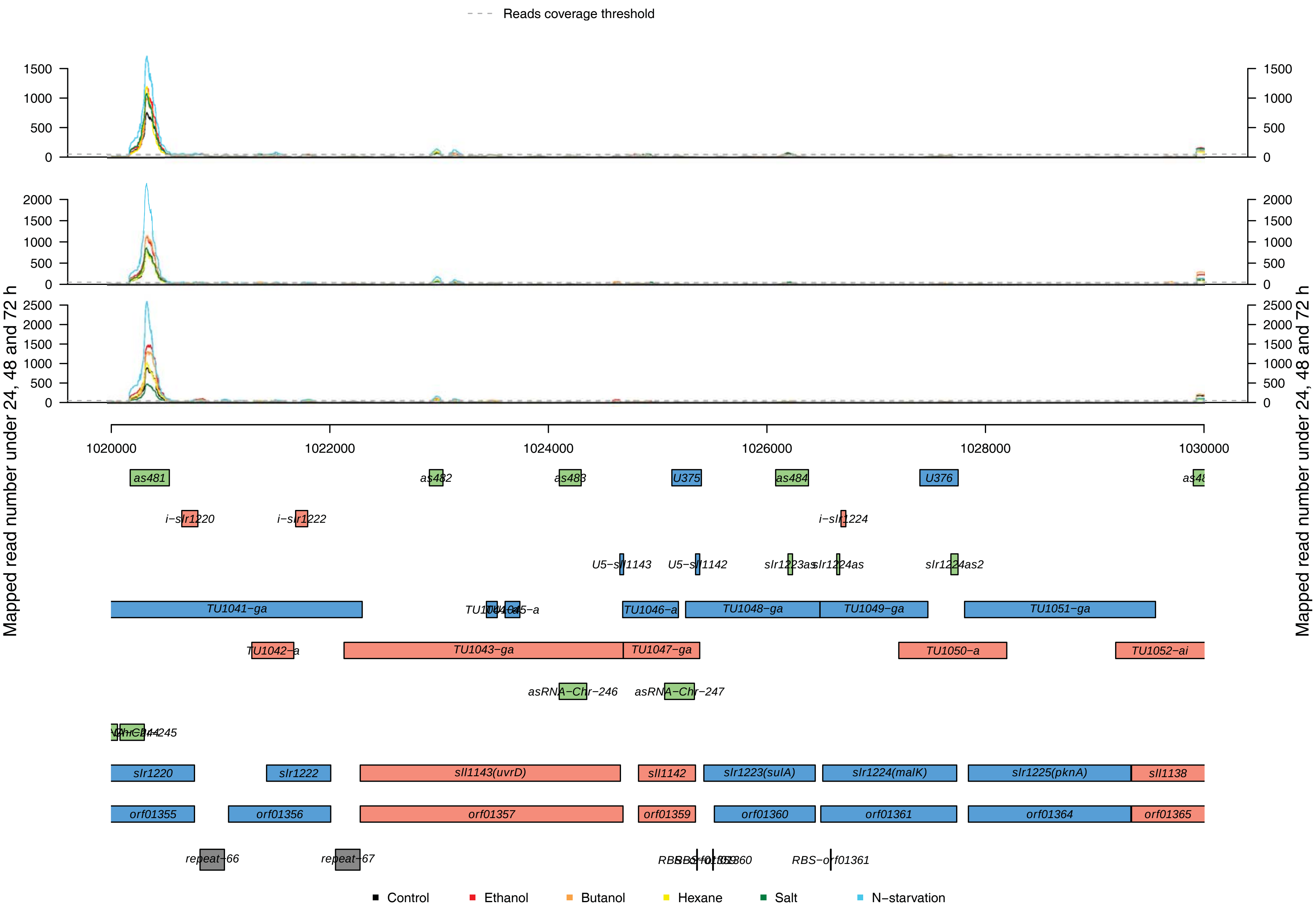

Reads coverage threshold
Mapped read number under 24, 48 and 72 h
1020000
1022000
1024000
1026000
1028000
1030000
as481
as482
as483
U375
as484
U376
i−slr1220
i−slr1222
i−slr1224
U5−sll1143
U5−sll1142
slr1223as
slr1224as
slr1224as2
TU1041−ga
TU1046−a
TU1048−ga
TU1049−ga
TU1051−ga
TU1042−a
TU1043−ga
TU1047−ga
TU1050−a
TU1052−ai
asRNA−Chr−246
asRNA−Chr−247
slr1220
slr1222
sll1143(uvrD)
sll1142
slr1223(sulA)
slr1224(malK)
slr1225(pknA)
sll1138
orf01355
orf01356
orf01357
orf01359
orf01360
orf01361
orf01364
orf01365
repeat−66
repeat−67
RBS−orf01361
Control
Ethanol
Butanol
Hexane
Salt
N−starvation

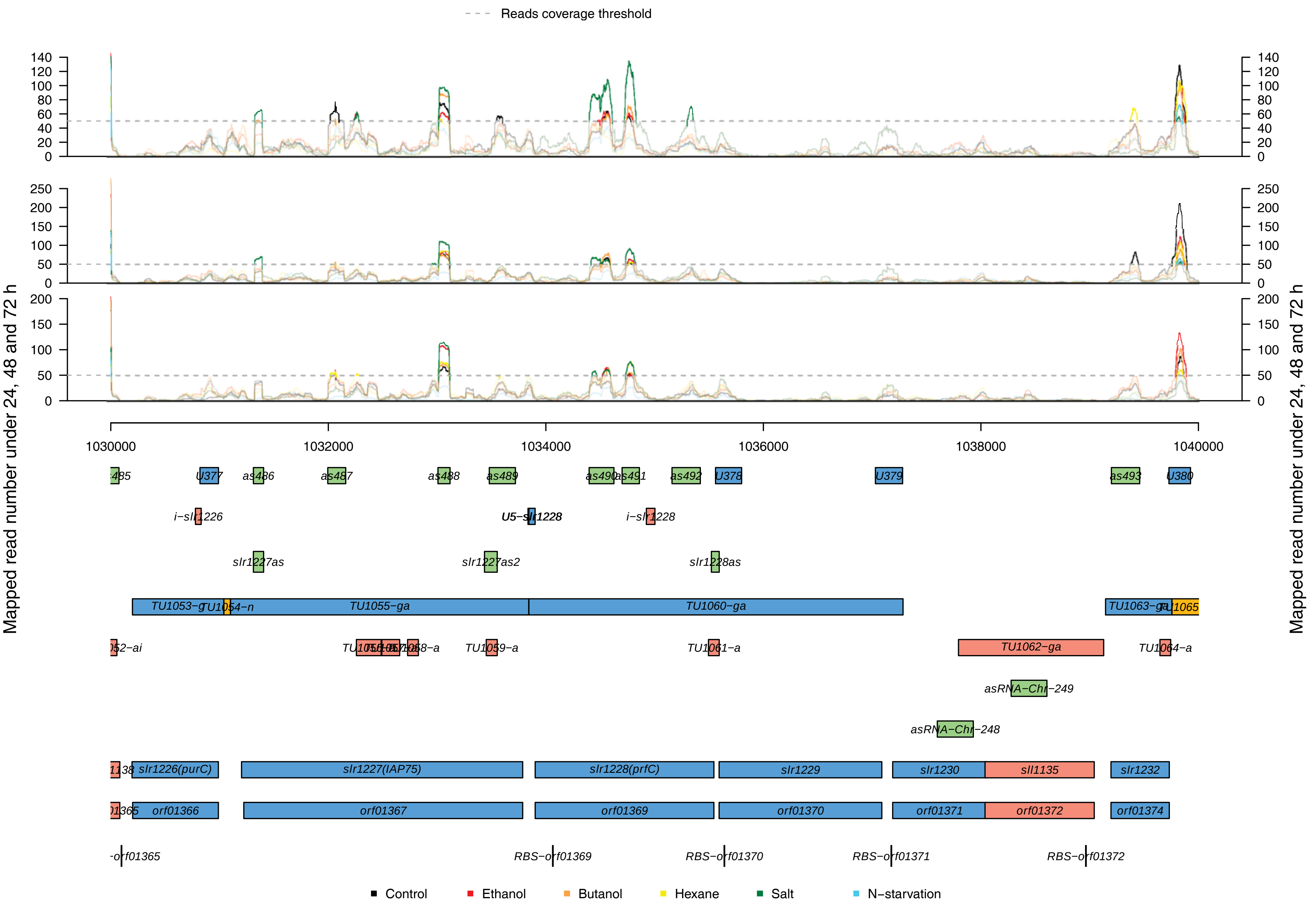

Reads coverage threshold
Mapped read number under 24, 48 and 72 h
1030000
1032000
1034000
1036000
1038000
1040000
U377
as486
as487
as488
as489
as490
as491
as492
U378
U379
as493
U380
i−slr1226
U5−slr1228
i−slr1228
slr1227as
slr1227as2
slr1228as
TU1055−ga
TU1060−ga
TU1059−a
TU1061−a
TU1062−ga
TU1064−a
asRNA−Chr−249
asRNA−Chr−248
slr1226(purC)
slr1227(IAP75)
slr1228(prfC)
slr1229
slr1230
sll1135
slr1232
orf01366
orf01367
orf01369
orf01370
orf01371
orf01372
orf01374
RBS−orf01369
RBS−orf01370
RBS−orf01371
RBS−orf01372
Control
Ethanol
Butanol
Hexane
Salt
N−starvation

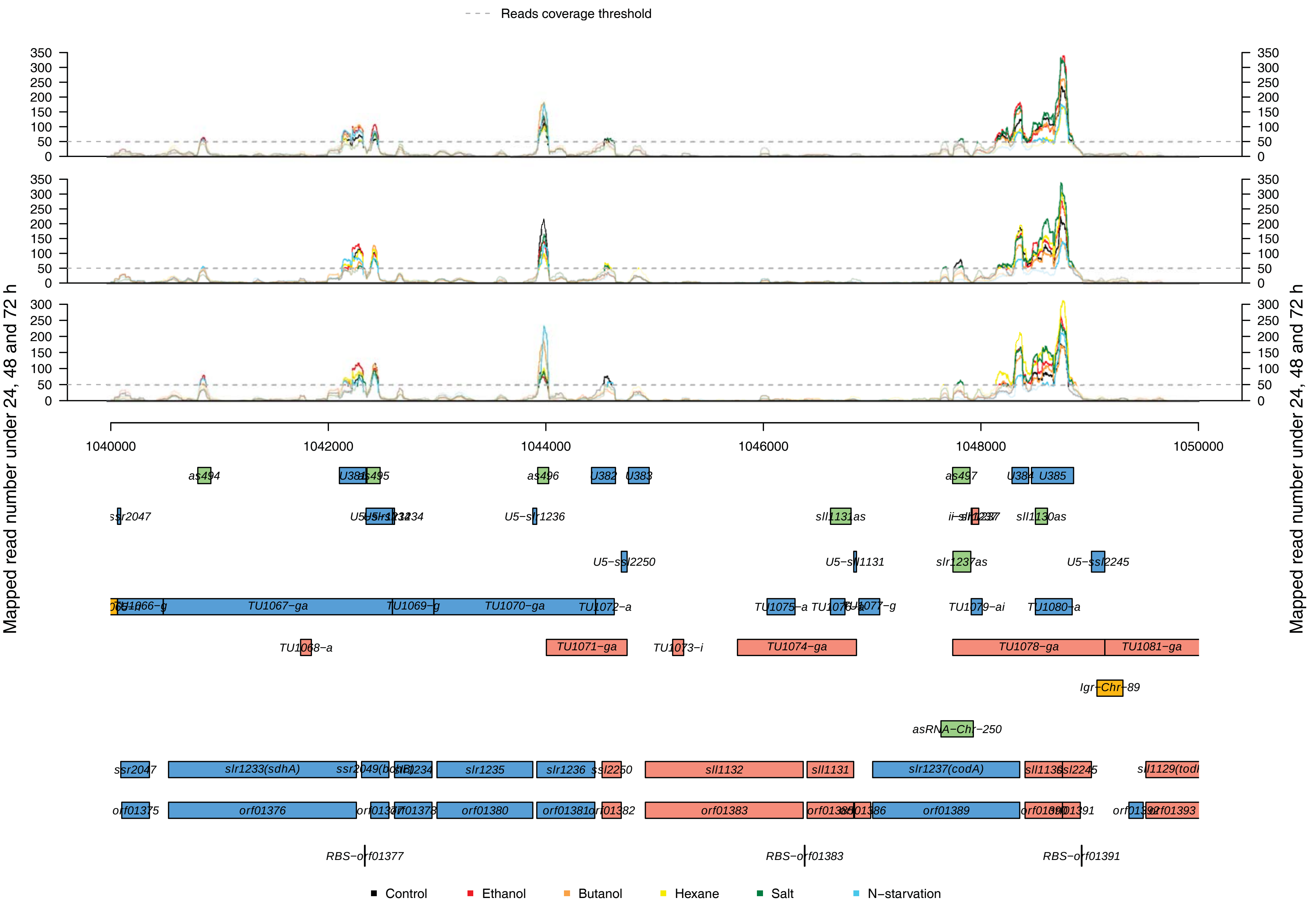

Reads coverage threshold
Mapped read number under 24, 48 and 72 h
1040000
1042000
1044000
1046000
1048000
1050000
as494
as495
as496
U382
U383
as497
U384
U385
ssr2047
U5−slr1236
sll1131as
sll1130as
U5−ssl2250
U5−sll1131
slr1237as
U5−ssl2245
TU1067−ga
TU1069−g
TU1070−ga
TU1072−a
TU1075−a
TU1077−g
TU1079−ai
TU1080−a
TU1068−a
TU1071−ga
TU1073−i
TU1074−ga
TU1078−ga
TU1081−ga
Igr−Chr−89
asRNA−Chr−250
ssr2047
slr1233(sdhA)
slr1235
slr1236
ssl2250
sll1132
sll1131
slr1237(codA)
orf01375
orf01376
orf01380
orf01382
orf01383
orf01386
orf01389
orf01391
orf01393
RBS−orf01377
RBS−orf01383
RBS−orf01391
Control
Ethanol
Butanol
Hexane
Salt
N−starvation

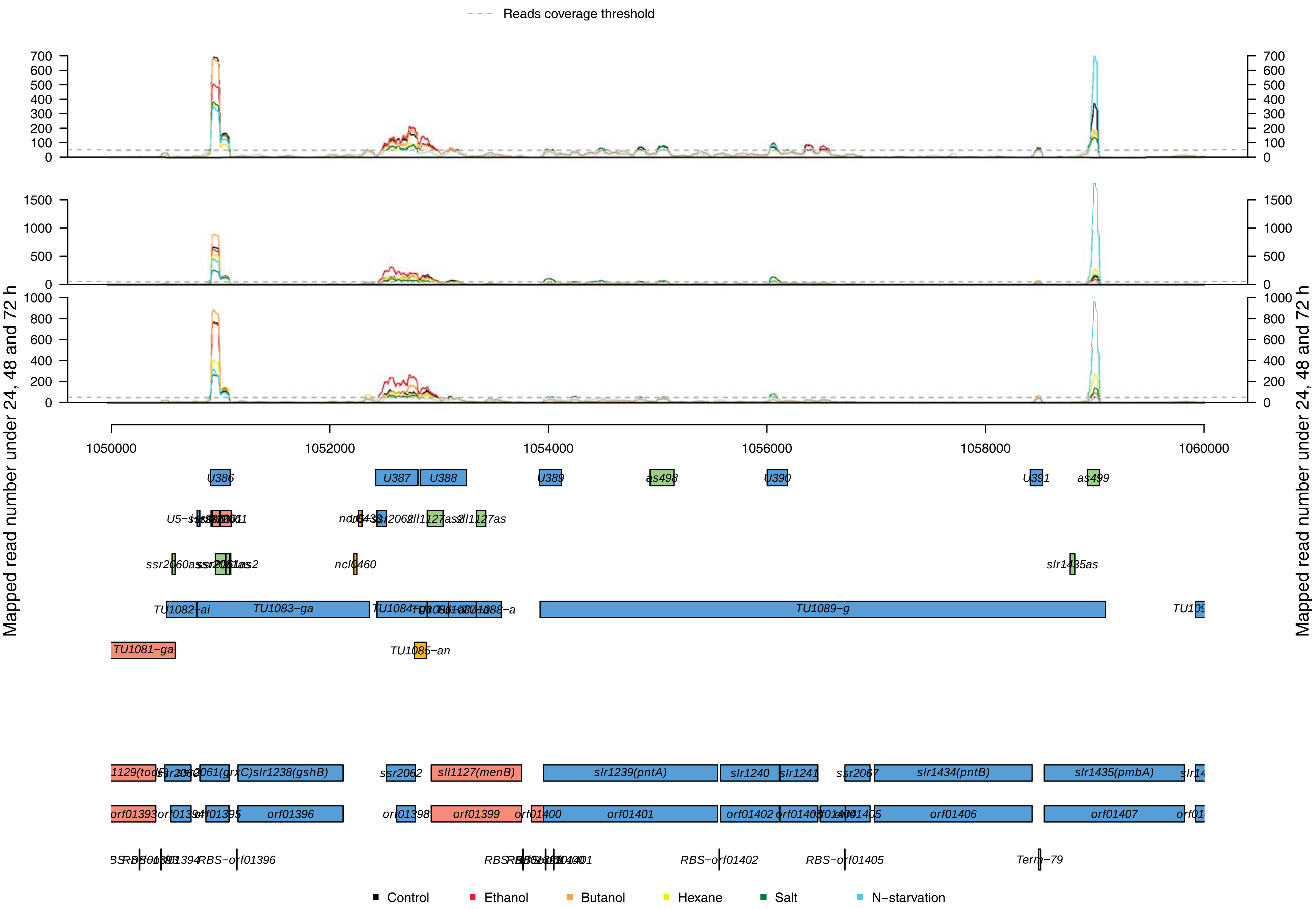

Reads coverage threshold
Mapped read number under 24, 48 and 72 h
1050000
1052000
1054000
1056000
1058000
1060000
U386
U387
U388
U389
as498
U390
U391
as499
slr1435as
TU1083−ga
TU1089−g
TU1081−ga
slr1238(gshB)
ssr2062
sll1127(menB)
slr1239(pntA)
slr1240
slr1241
slr1434(pntB)
slr1435(pmbA)
orf01393
orf01396
orf01399
orf01401
orf01402
orf01406
orf01407
RBS−orf01396
RBS−orf01402
RBS−orf01405
Term−79
Control
Ethanol
Butanol
Hexane
Salt
N−starvation

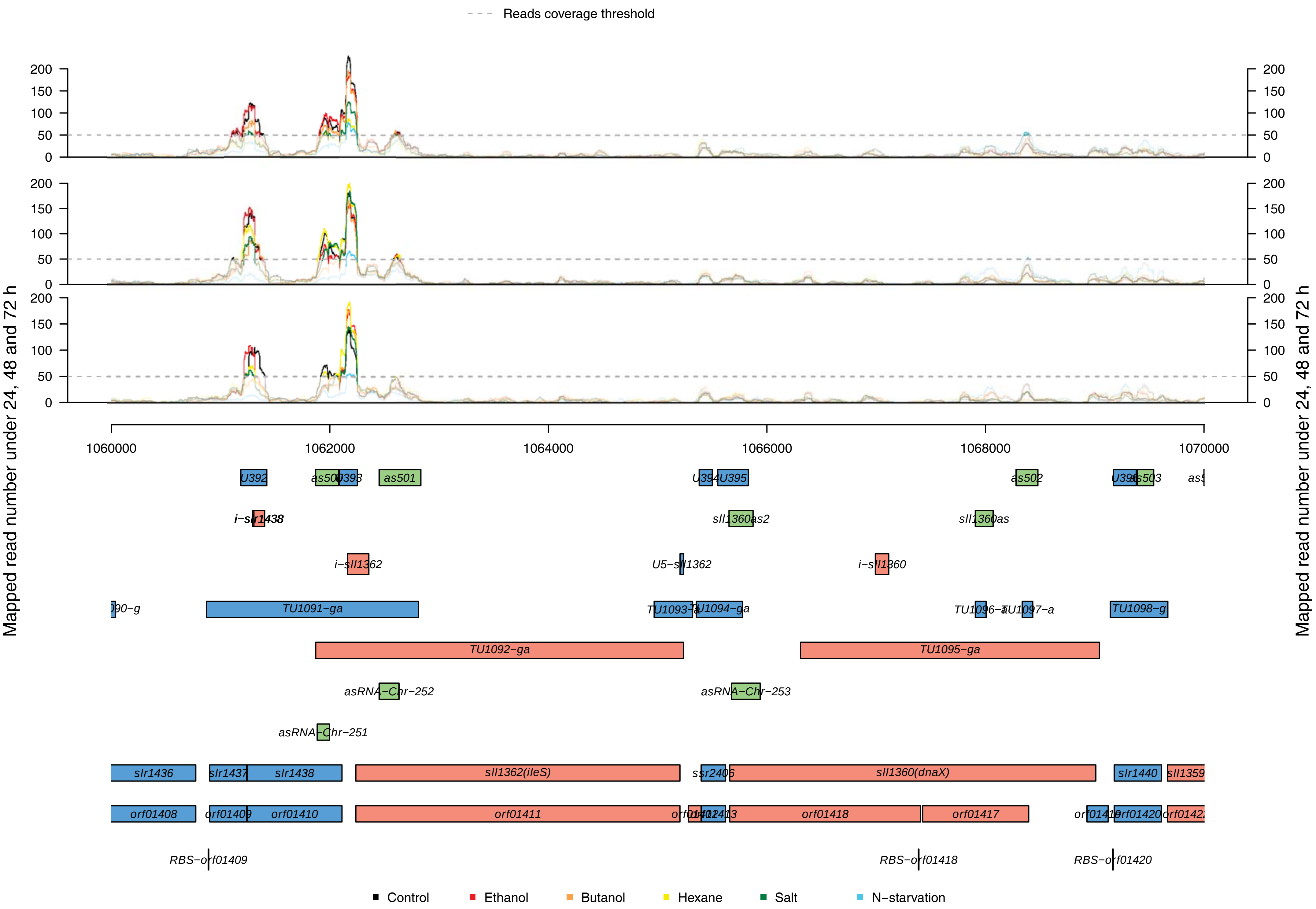

Reads coverage threshold
Mapped read number under 24, 48 and 72 h
200
150
100
50
0
1060000
1062000
1064000
1066000
1068000
1070000
U392
as500
U393
as501
U394
U395
as502
U396
as503
i−slr1438
sll1360as2
sll1360as
i−sll1362
U5−sll1362
i−sll1360
TU1091−ga
TU1093−a
TU1094−ga
TU1096−a
TU1097−a
TU1098−g
TU1092−ga
TU1095−ga
asRNA−Chr−252
asRNA−Chr−253
asRNA−Chr−251
slr1436
slr1437
slr1438
sll1362(ileS)
ssr2406
sll1360(dnaX)
slr1440
sll1359
orf01408
orf01409
orf01410
orf01411
orf01418
orf01417
orf01420
RBS−orf01409
RBS−orf01418
RBS−orf01420
Control
Ethanol
Butanol
Hexane
Salt
N−starvation

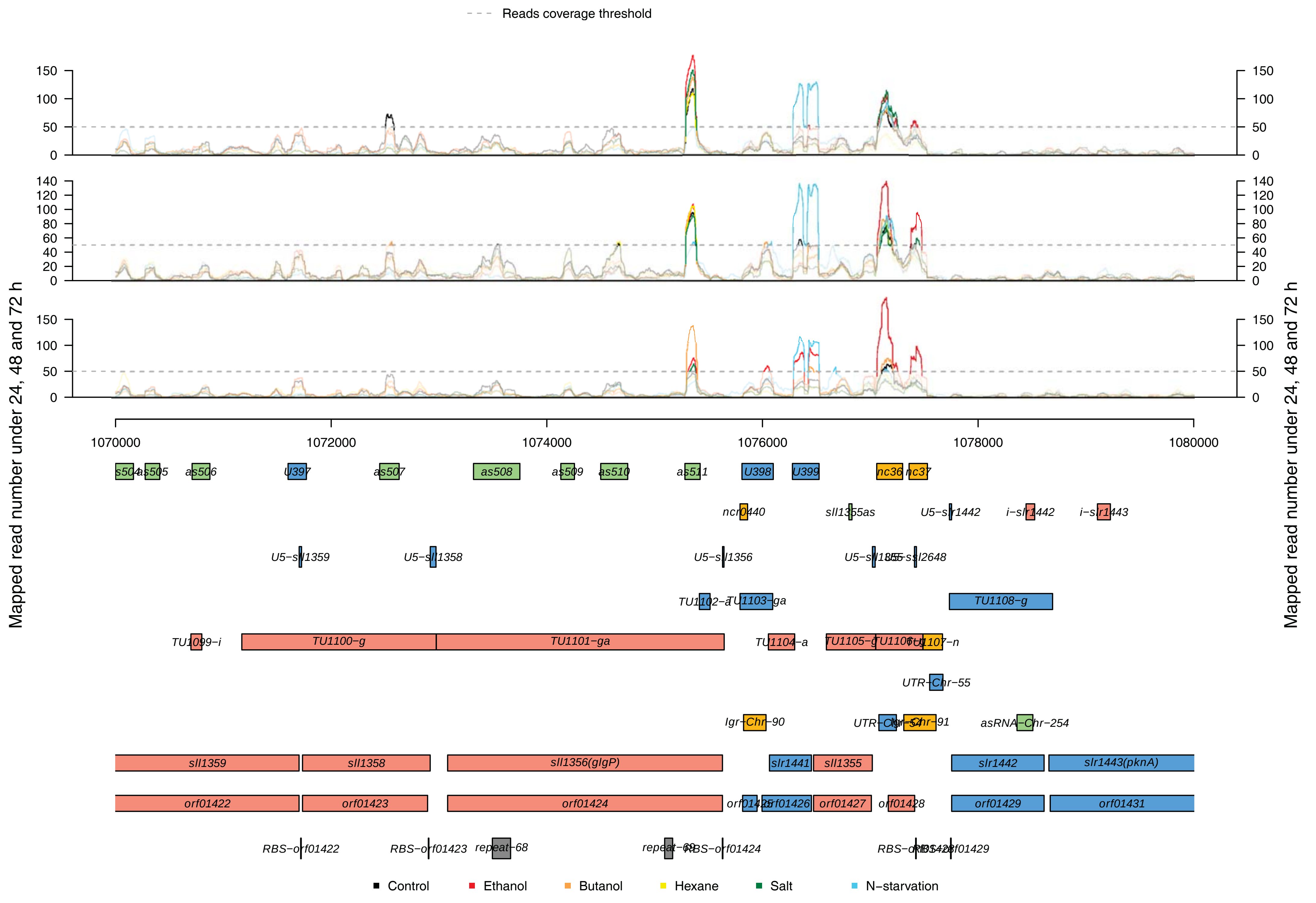

Reads coverage threshold
Mapped read number under 24, 48 and 72 h
1070000
1072000
1074000
1076000
1078000
1080000
Control
Ethanol
Butanol
Hexane
Salt
N−starvation

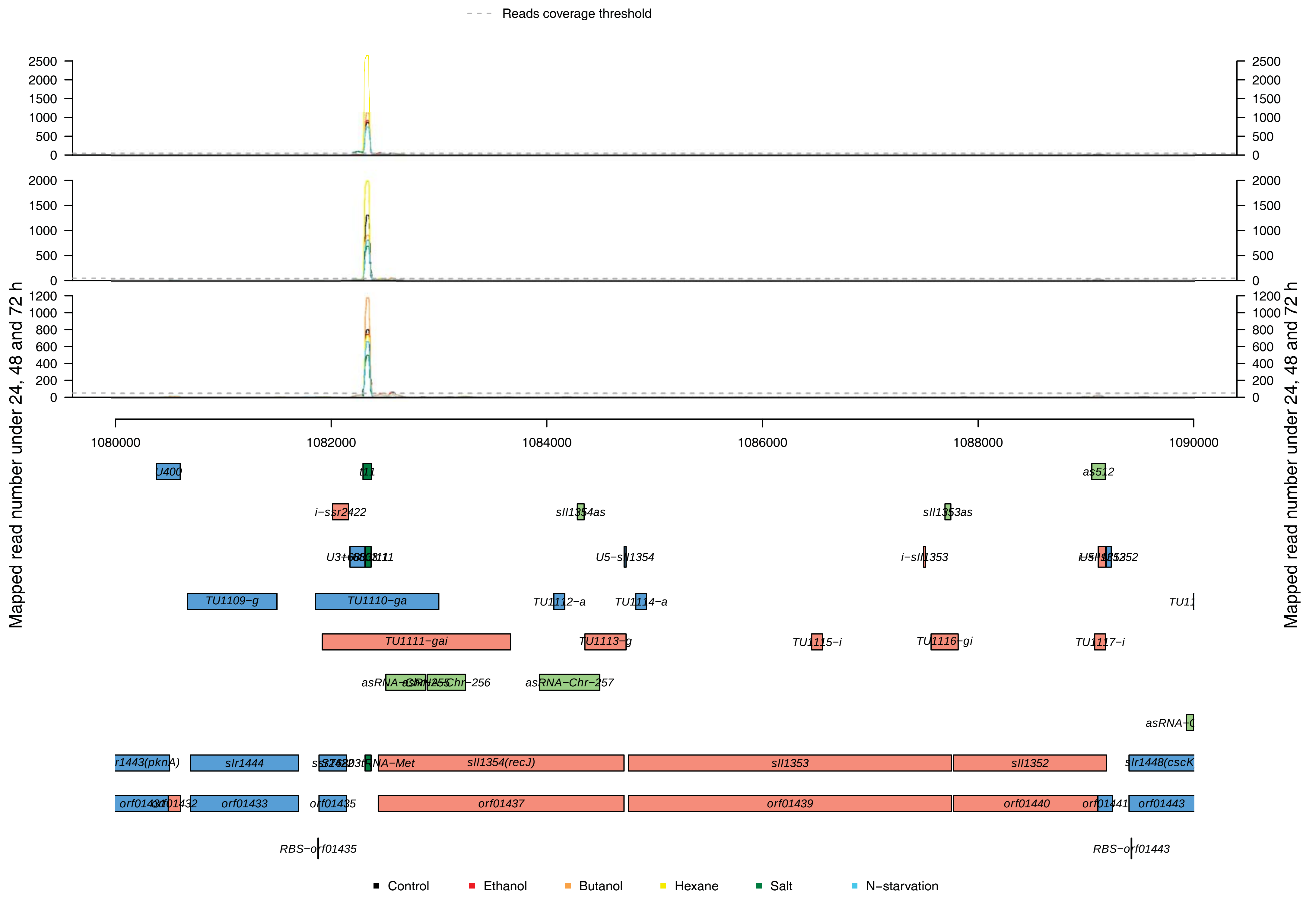
Reads coverage threshold
Mapped read number under 24, 48 and 72 h
1080000
1082000
1084000
1086000
1088000
1090000
TU1109−g
TU1110−ga
TU1111−gai
TU1112−a
TU1113−g
TU1114−a
TU1115−i
TU1116−gi
TU1117−i
i−ssr2422
sll1354as
sll1353as
U5−sll1354
i−sll1353
asRNA−Chr−256
asRNA−Chr−257
as512
slr1444
sll1354(recJ)
sll1353
sll1352
orf01433
orf01435
orf01437
orf01439
orf01440
orf01443
RBS−orf01435
RBS−orf01443
Control
Ethanol
Butanol
Hexane
Salt
N−starvation

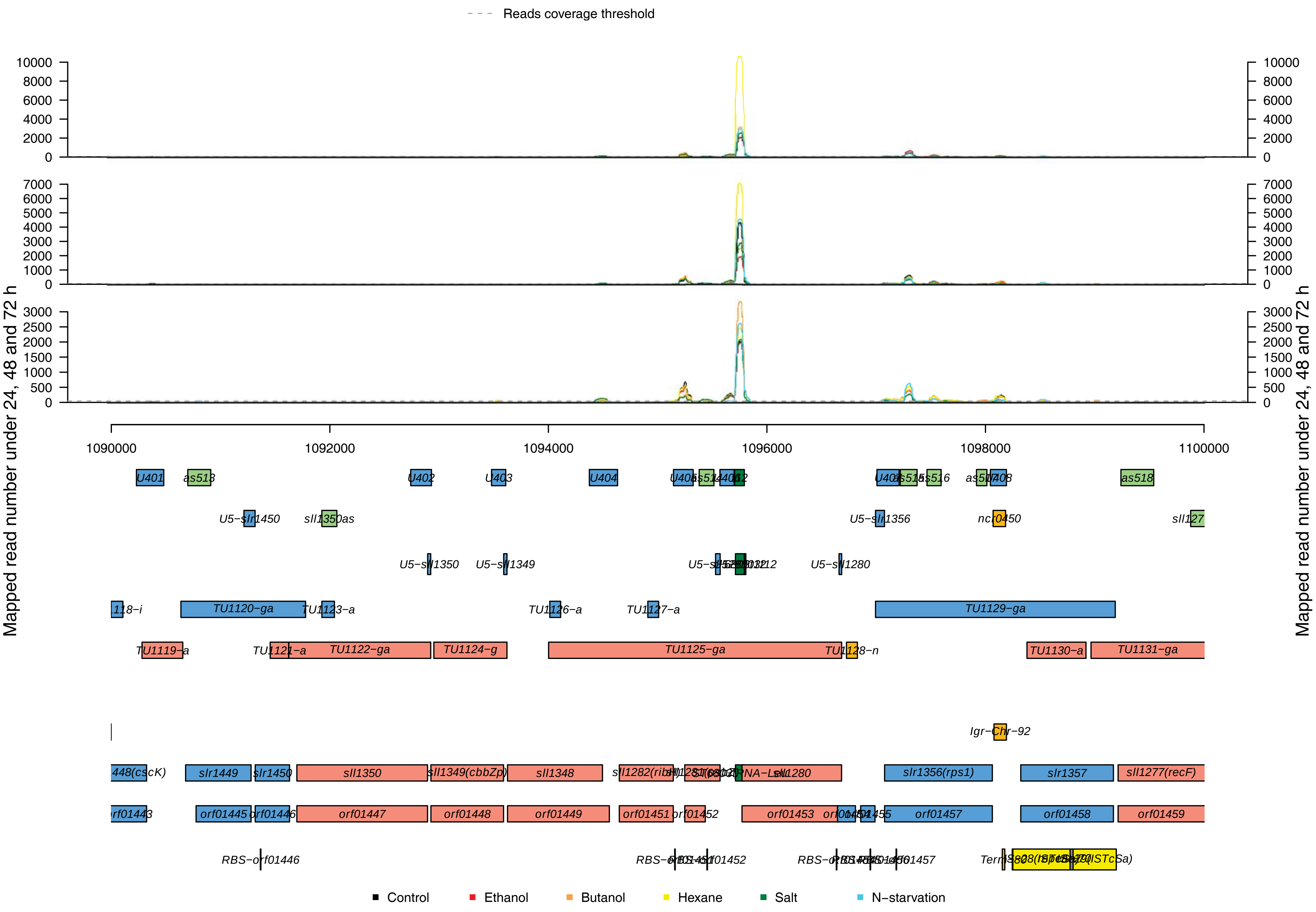

Reads coverage threshold
Mapped read number under 24, 48 and 72 h
1090000
1092000
1094000
1096000
1098000
1100000
TU1120−ga
TU1122−ga
TU1124−g
TU1125−ga
TU1129−ga
TU1130−a
TU1131−ga
slr1449
sll1350
sll1348
slr1356(rps1)
slr1357
sll1277(recF)
orf01445
orf01447
orf01448
orf01449
orf01451
orf01453
orf01457
orf01458
orf01459
Igr−Chr−92
Control
Ethanol
Butanol
Hexane
Salt
N−starvation

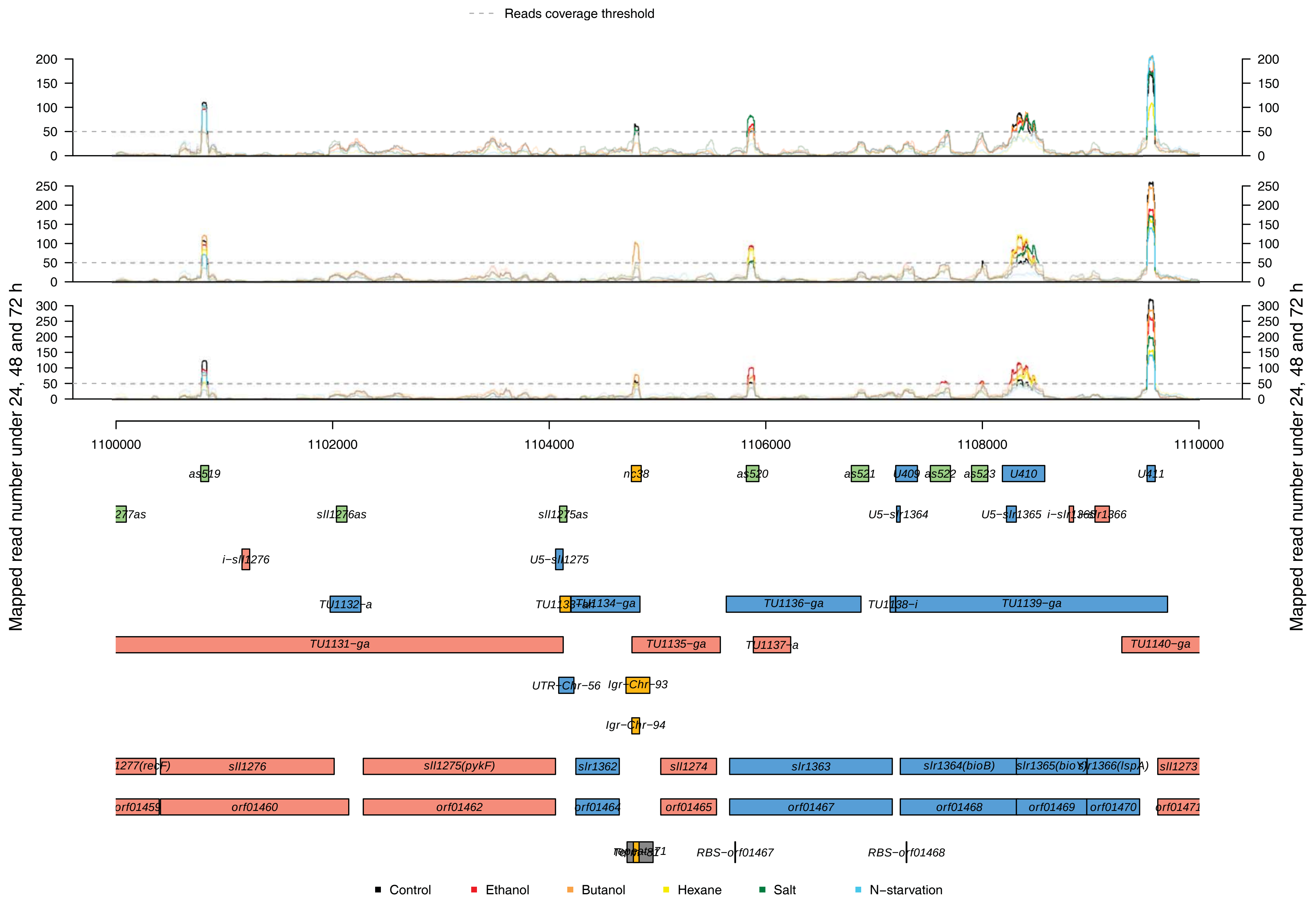
Reads coverage threshold
Mapped read number under 24, 48 and 72 h
1100000
1102000
1104000
1106000
1108000
1110000
as519
nc38
as520
as521
U409
as522
as523
U410
U411
277as
sll1276as
sll1275as
U5−slr1364
U5−slr1365
i−slr1365
slr1366
i−sll1276
U5−sll1275
TU1132−a
TU1133−an
TU1134−ga
TU1136−ga
TU1138−i
TU1139−ga
TU1131−ga
TU1135−ga
TU1137−a
TU1140−ga
UTR−Chr−56
Igr−Chr−93
Igr−Chr−94
1277(recF)
sll1276
sll1275(pykF)
slr1362
sll1274
slr1363
slr1364(bioB)
slr1365(bioY)
slr1366(lspA)
sll1273
orf01459
orf01460
orf01462
orf01464
orf01465
orf01467
orf01468
orf01469
orf01470
orf01471
RBS−orf01467
RBS−orf01468
Control
Ethanol
Butanol
Hexane
Salt
N−starvation

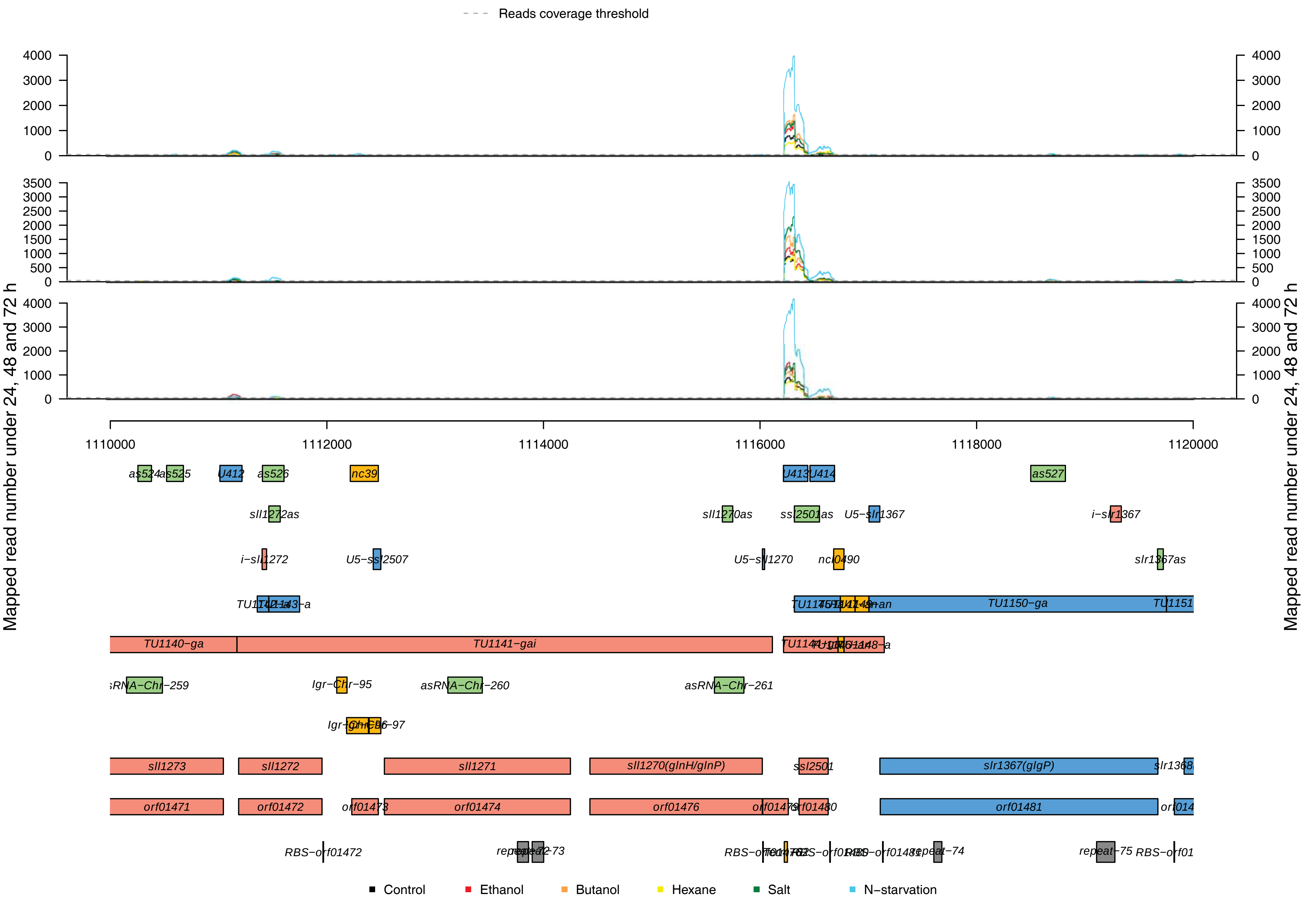

Reads coverage threshold
Mapped read number under 24, 48 and 72 h
1110000
1112000
1114000
1116000
1118000
1120000
TU1140−ga
TU1141−gai
TU1150−ga
sll1273
sll1272
sll1271
sll1270(glnH/glnP)
ssl2501
slr1367(glgP)
orf01471
orf01472
orf01474
orf01476
orf01481
asRNA−Chr−260
asRNA−Chr−261
Control
Ethanol
Butanol
Hexane
Salt
N−starvation

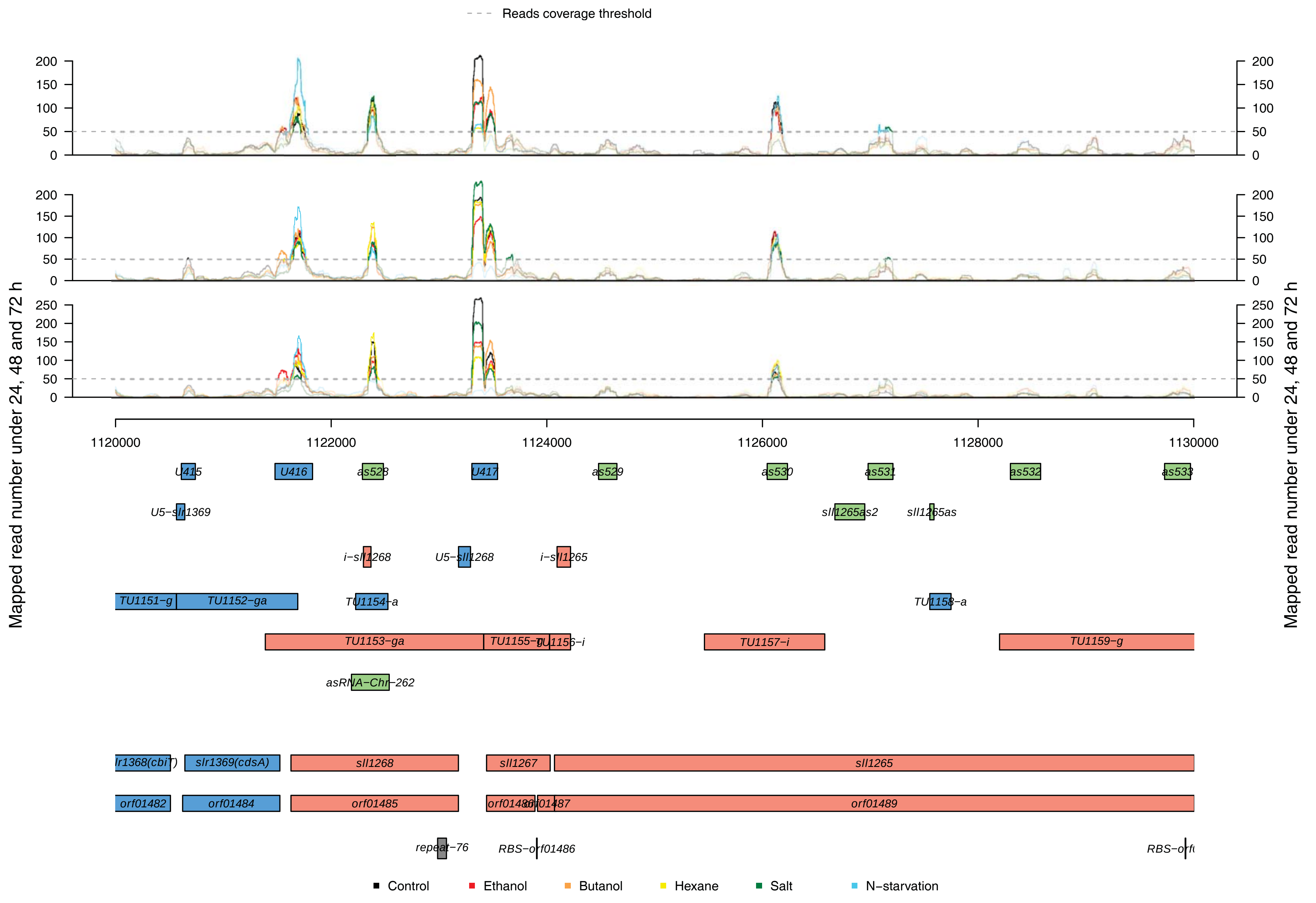

Reads coverage threshold
Mapped read number under 24, 48 and 72 h
1120000
1122000
1124000
1126000
1128000
1130000
U415
U416
as528
U417
as529
as530
as531
as532
as533
U5−slr1369
sll1265as2
sll1265as
i−sll1268
U5−sll1268
i−sll1265
TU1151−g
TU1152−ga
TU1154−a
TU1158−a
TU1153−ga
TU1155−i
TU1156−i
TU1157−i
TU1159−g
asRNA−Chr−262
slr1369(cdsA)
sll1268
sll1267
sll1265
orf01482
orf01484
orf01485
orf01489
repeat−76
RBS−orf01486
Control
Ethanol
Butanol
Hexane
Salt
N−starvation

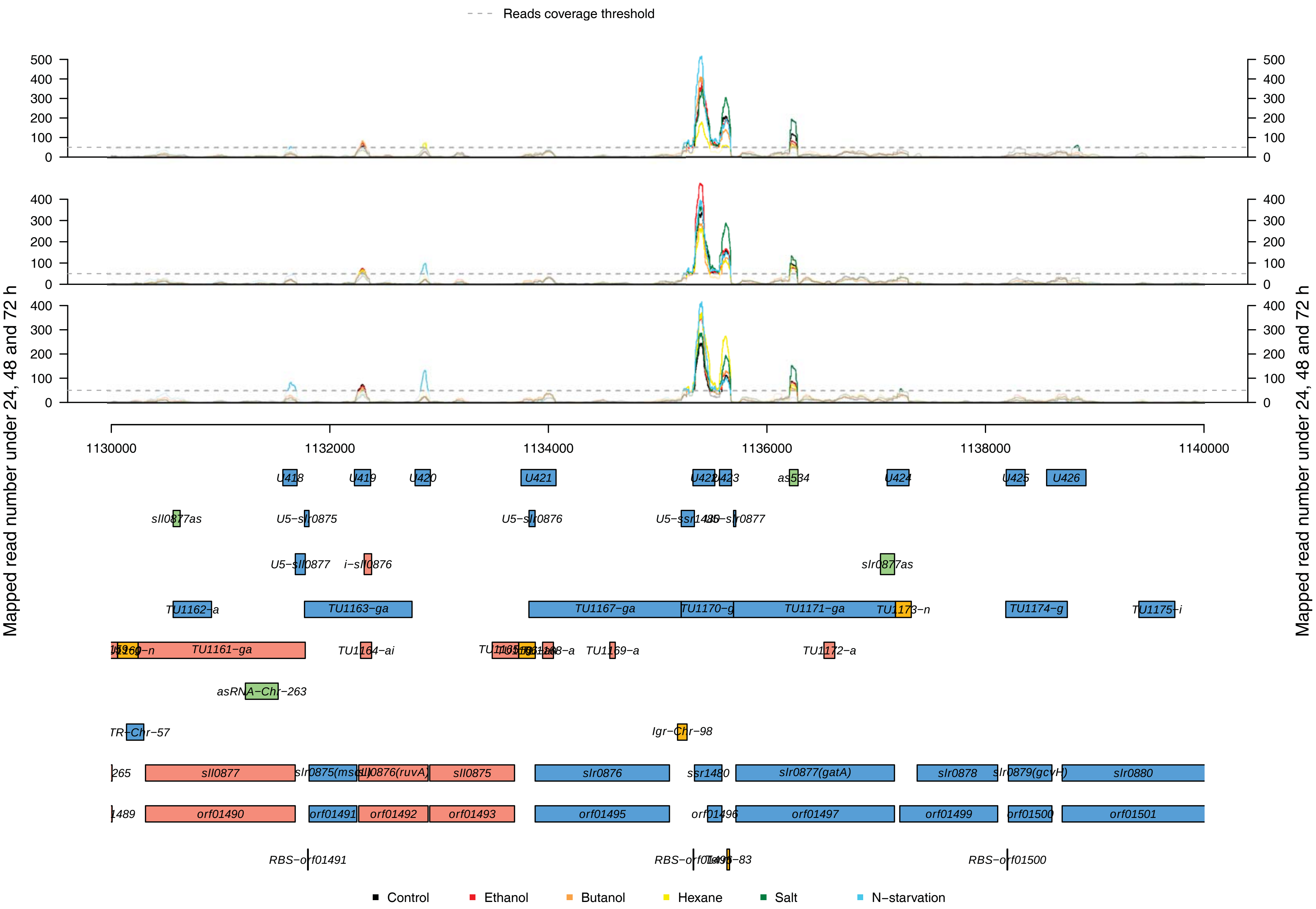

Reads coverage threshold
Mapped read number under 24, 48 and 72 h
1130000
1132000
1134000
1136000
1138000
1140000
U418
U419
U420
U421
U423
as534
U424
U425
U426
sll0877as
U5−slr0875
U5−slr0876
U5−sll0877
i−sll0876
slr0877as
TU1162−a
TU1163−ga
TU1167−ga
TU1170−g
TU1171−ga
TU1174−g
TU1175−i
TU1161−ga
TU1164−ai
TU1169−a
TU1172−a
asRNA−Chr−263
TR−Chr−57
Igr−Chr−98
sll0877
slr0876
ssr1480
slr0877(gatA)
slr0878
slr0880
orf01490
orf01491
orf01492
orf01493
orf01495
orf01497
orf01499
orf01501
RBS−orf01491
RBS−orf01500
Control
Ethanol
Butanol
Hexane
Salt
N−starvation

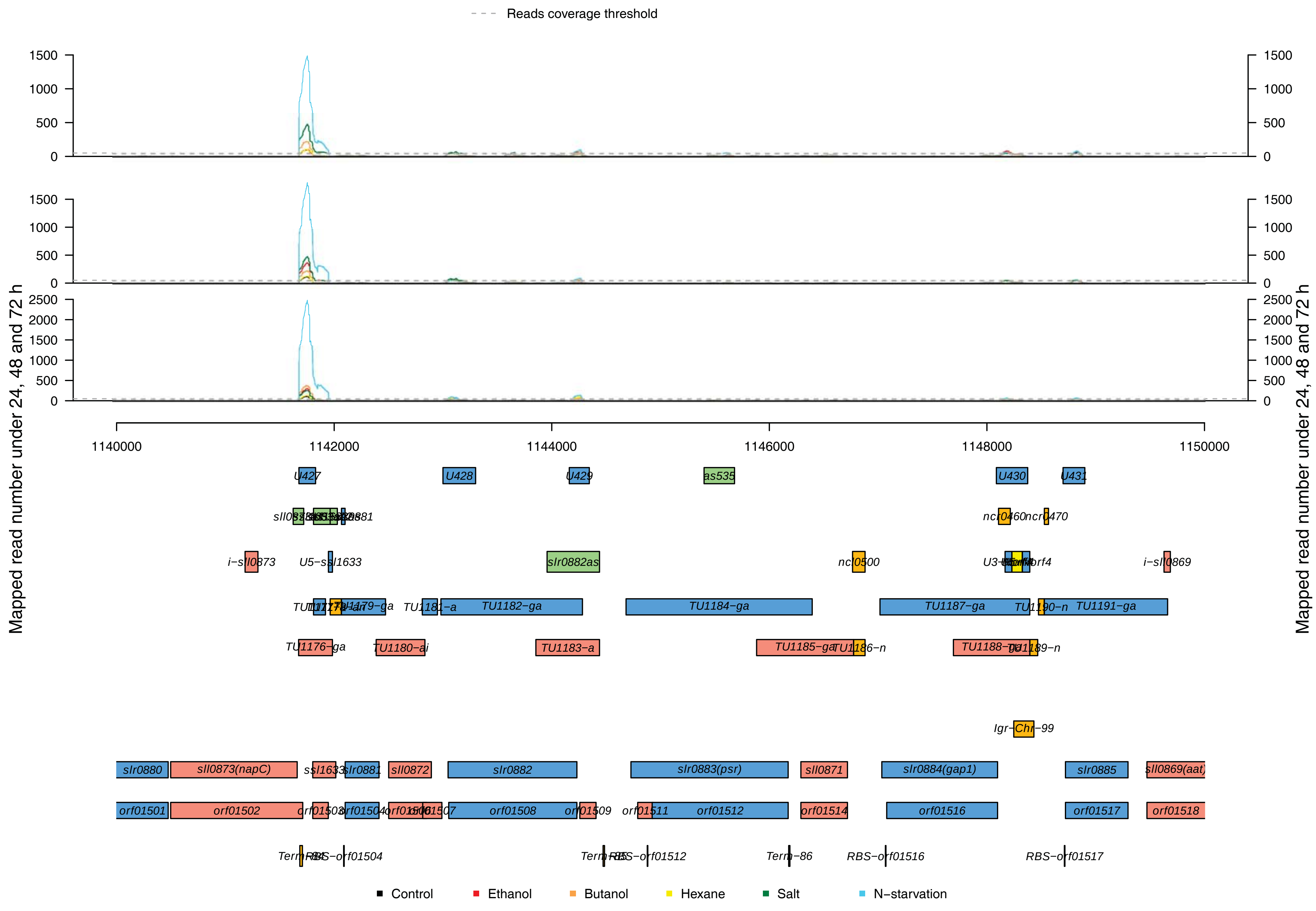

Reads coverage threshold
Mapped read number under 24, 48 and 72 h
1140000
1142000
1144000
1146000
1148000
1150000
U427
U428
U429
as535
U430
U431
i−sll0873
U5−ssl1633
slr0882as
ncl0500
i−sll0869
TU1182−ga
TU1184−ga
TU1187−ga
TU1191−ga
TU1176−ga
TU1180−ai
TU1183−a
Igr−Chr−99
slr0880
sll0873(napC)
slr0881
sll0872
slr0882
slr0883(psr)
sll0871
slr0884(gap1)
slr0885
sll0869(aat)
orf01501
orf01502
orf01508
orf01512
orf01514
orf01516
orf01517
orf01518
Term−86
RBS−orf01516
RBS−orf01517
Control
Ethanol
Butanol
Hexane
Salt
N−starvation

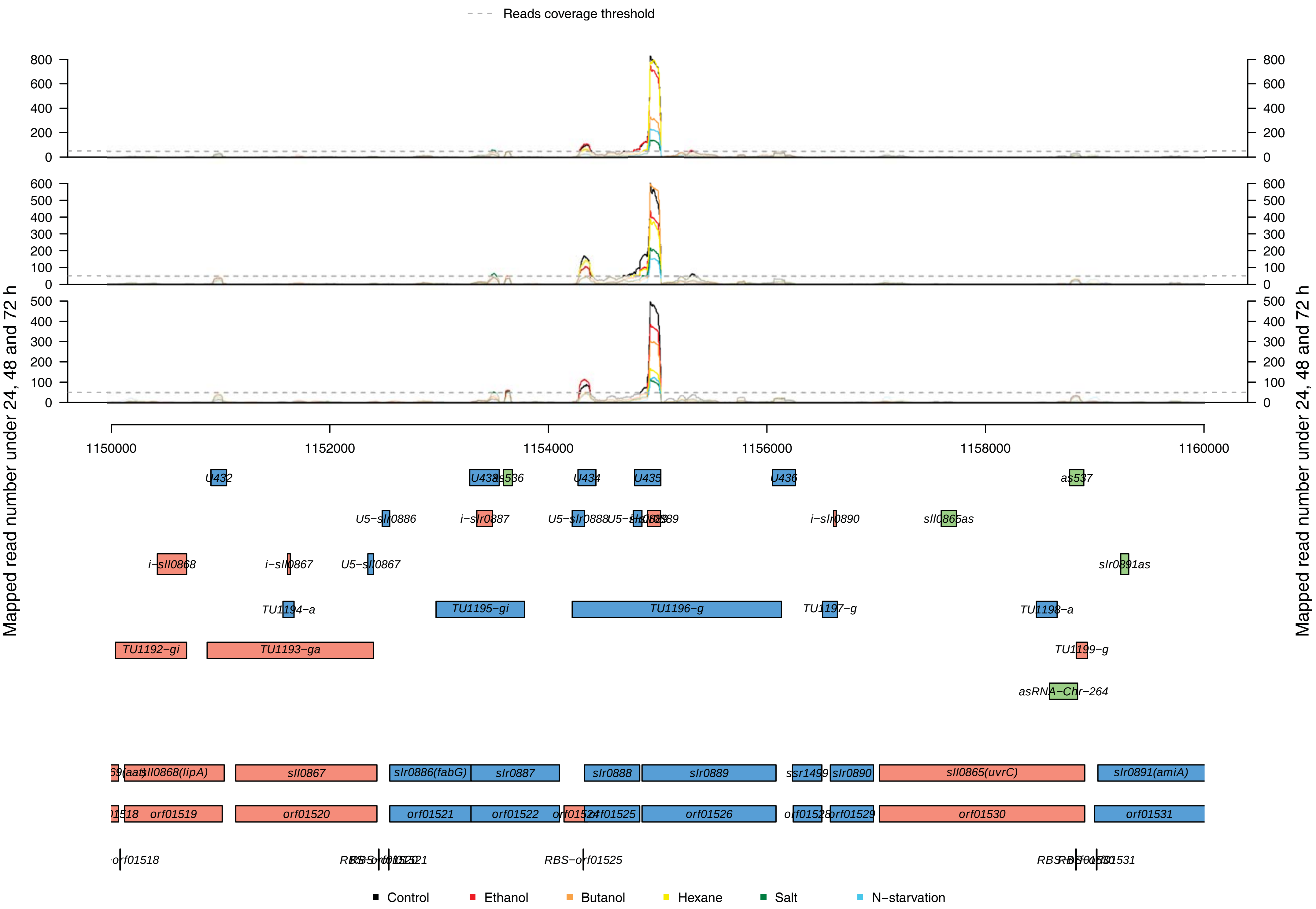

Reads coverage threshold
Mapped read number under 24, 48 and 72 h
1150000
1152000
1154000
1156000
1158000
1160000
U432
U434
U435
U436
as537
U5−sll0886
i−slr0887
i−slr0890
sll0865as
i−sll0868
i−sll0867
U5−sll0867
slr0891as
TU1194−a
TU1195−gi
TU1196−g
TU1197−g
TU1198−a
TU1192−gi
TU1193−ga
TU1199−g
asRNA−Chr−264
sll0868(lipA)
sll0867
slr0886(fabG)
slr0887
slr0888
slr0889
ssr1499
slr0890
sll0865(uvrC)
slr0891(amiA)
orf01519
orf01520
orf01521
orf01522
orf01526
orf01530
orf01531
RBS−orf01525
Control
Ethanol
Butanol
Hexane
Salt
N−starvation

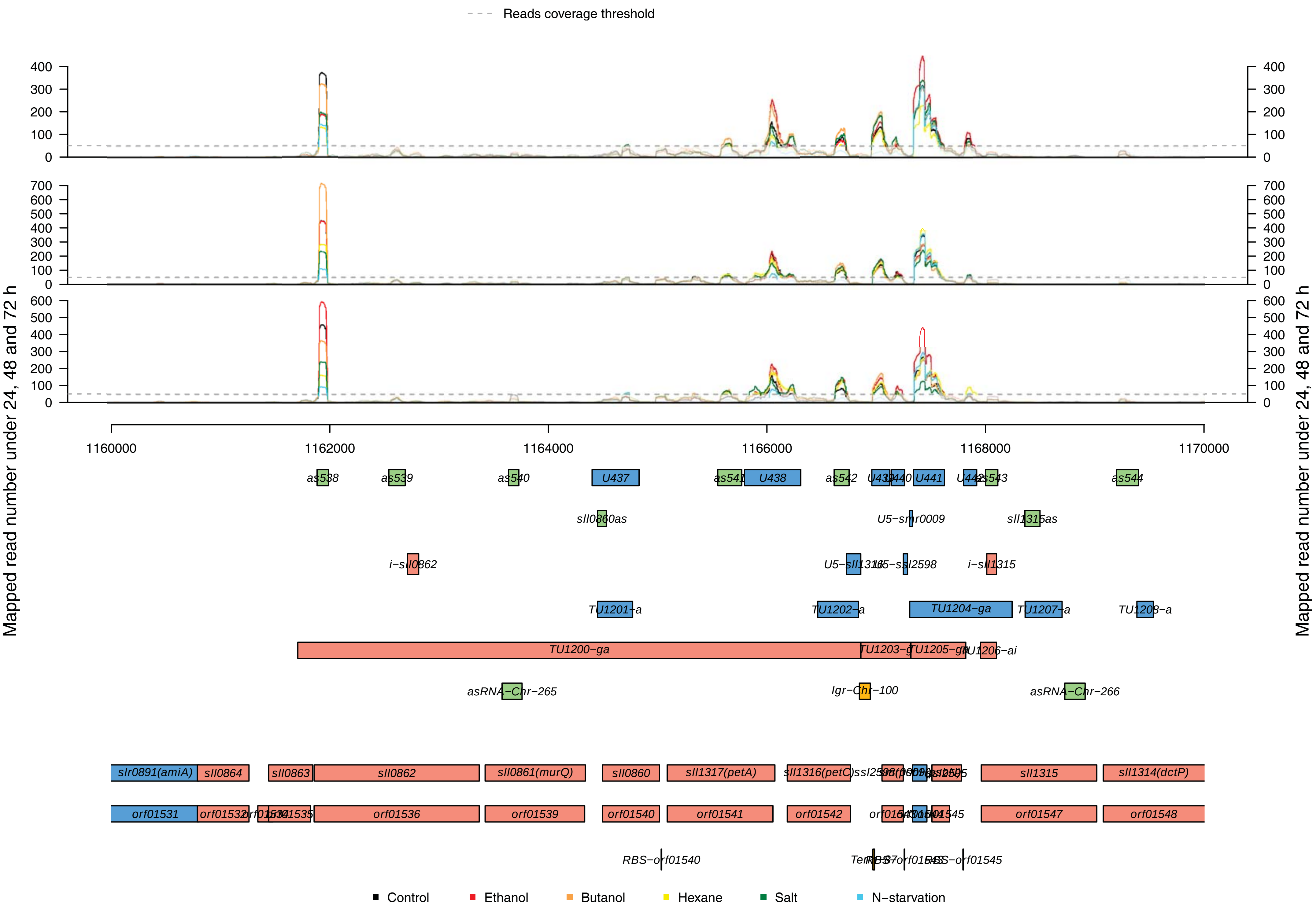

Reads coverage threshold
Mapped read number under 24, 48 and 72 h
1160000
1162000
1164000
1166000
1168000
1170000
as538
as539
as540
U437
U438
as542
U441
as544
sll0860as
U5−smr0009
sll1315as
i−sll0862
i−sll1315
TU1201−a
TU1202−a
TU1204−ga
TU1207−a
TU1208−a
TU1200−ga
TU1206−ai
asRNA−Chr−265
Igr−Chr−100
asRNA−Chr−266
slr0891(amiA)
sll0864
sll0863
sll0862
sll0861(murQ)
sll0860
sll1317(petA)
sll1316(petC)
sll1315
sll1314(dctP)
orf01531
orf01536
orf01539
orf01540
orf01541
orf01542
orf01547
orf01548
RBS−orf01540
Control
Ethanol
Butanol
Hexane
Salt
N−starvation

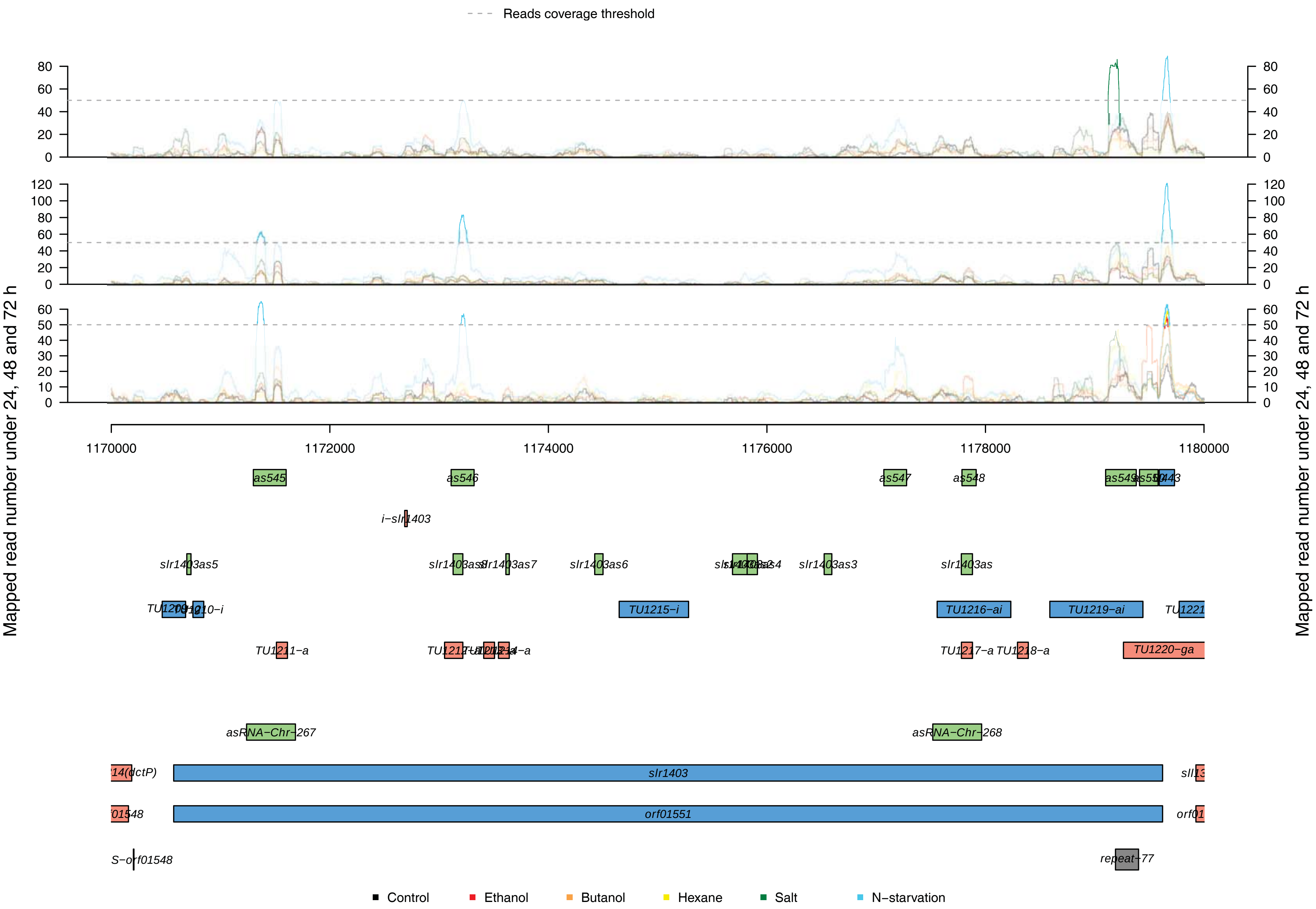

Reads coverage threshold
Mapped read number under 24, 48 and 72 h
1170000
1172000
1174000
1176000
1178000
1180000
as545
as546
as547
as548
i−slr1403
slr1403as5
slr1403as7
slr1403as6
slr1403as3
slr1403as
TU1210−i
TU1215−i
TU1216−ai
TU1219−ai
TU1211−a
TU1217−a
TU1218−a
TU1220−ga
asRNA−Chr−267
asRNA−Chr−268
slr1403
orf01551
S−orf01548
repeat−77
Control
Ethanol
Butanol
Hexane
Salt
N−starvation

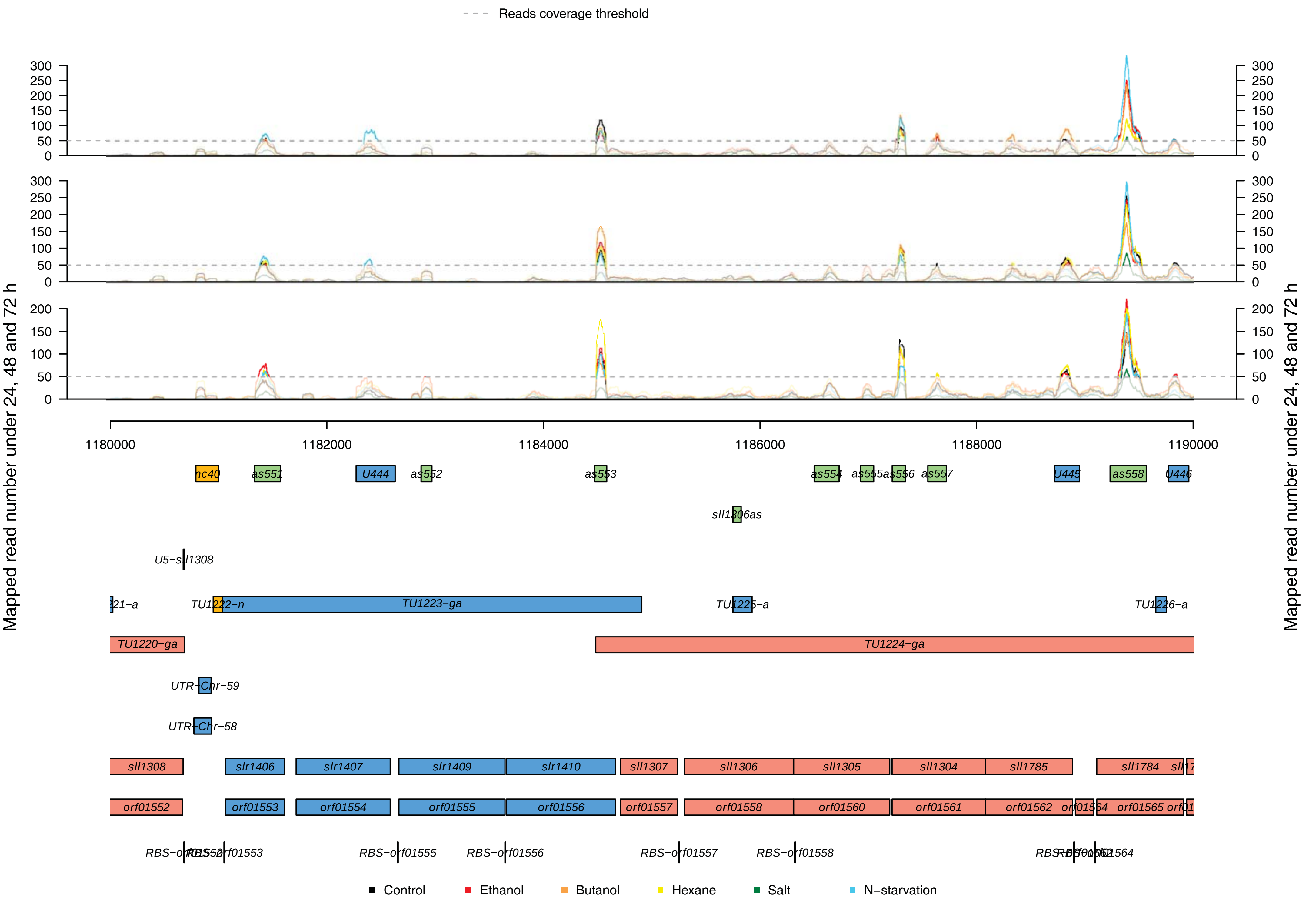

Reads coverage threshold
Mapped read number under 24, 48 and 72 h
300
250
200
150
100
50
0
1180000
1182000
1184000
1186000
1188000
1190000
as551
U444
as552
as553
as554
as557
U445
as558
U446
sll1306as
U5−sll1308
TU1222−n
TU1223−ga
TU1225−a
TU1226−a
TU1220−ga
TU1224−ga
UTR−Chr−59
UTR−Chr−58
sll1308
slr1406
slr1407
slr1409
slr1410
sll1307
sll1306
sll1305
sll1304
sll1785
sll1784
orf01552
orf01553
orf01554
orf01555
orf01556
orf01557
orf01558
orf01560
orf01561
orf01562
orf01565
RBS−orf01553
RBS−orf01555
RBS−orf01556
RBS−orf01557
RBS−orf01558
Control
Ethanol
Butanol
Hexane
Salt
N−starvation

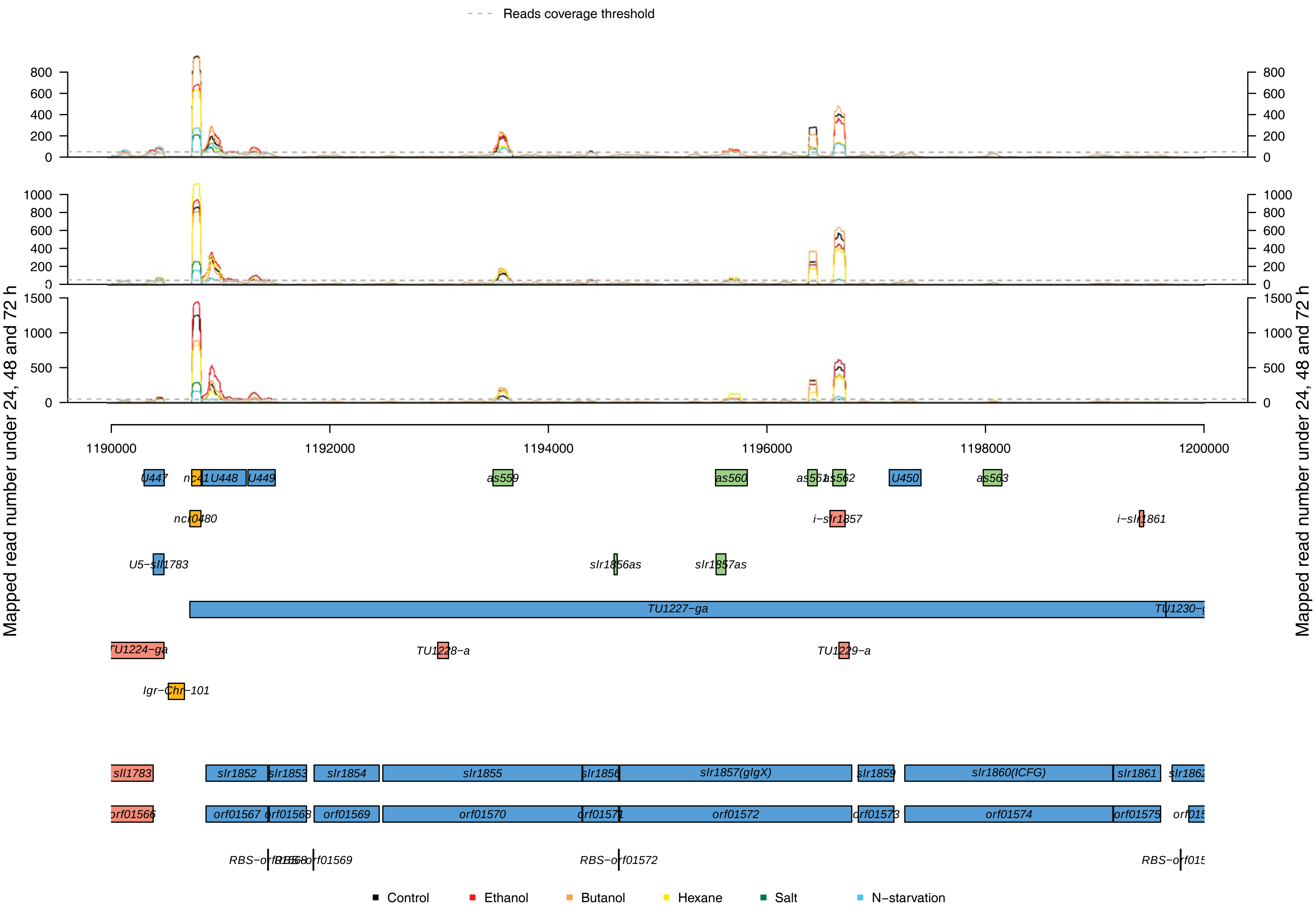

Reads coverage threshold
Mapped read number under 24, 48 and 72 h
800
600
400
200
0
1000
1500
500
1190000
1192000
1194000
1196000
1198000
1200000
U447
nc41
U448
U449
as559
as560
as561
as562
U450
as563
ncl0480
i−slr1857
i−slr1861
U5−sll1783
slr1856as
slr1857as
TU1227−ga
TU1224−ga
TU1228−a
TU1229−a
Igr−Chr−101
sll1783
slr1852
slr1853
slr1854
slr1855
slr1856
slr1857(glgX)
slr1859
slr1860(ICFG)
slr1861
orf01566
orf01567
orf01568
orf01569
orf01570
orf01571
orf01572
orf01573
orf01574
orf01575
RBS−orf01572
Control
Ethanol
Butanol
Hexane
Salt
N−starvation

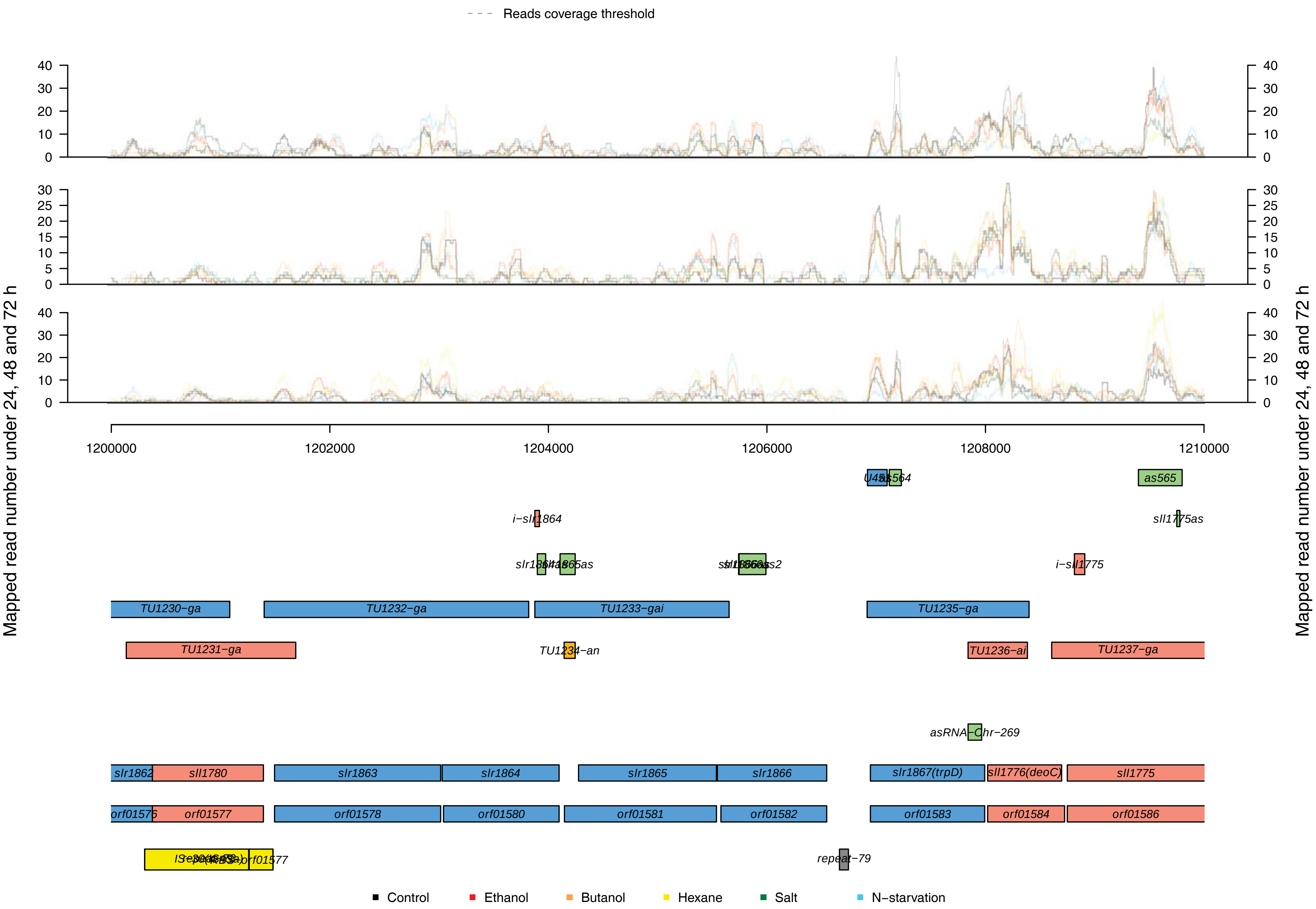

Reads coverage threshold
Mapped read number under 24, 48 and 72 h
1200000
1202000
1204000
1206000
1208000
1210000
as565
i−slr1864
sll1775as
i−sll1775
TU1230−ga
TU1232−ga
TU1233−gai
TU1235−ga
TU1231−ga
TU1234−an
TU1236−ai
TU1237−ga
asRNA−Chr−269
slr1862
sll1780
slr1863
slr1864
slr1865
slr1866
slr1867(trpD)
sll1776(deoC)
sll1775
orf01576
orf01577
orf01578
orf01580
orf01581
orf01582
orf01583
orf01584
orf01586
repeat−79
Control
Ethanol
Butanol
Hexane
Salt
N−starvation

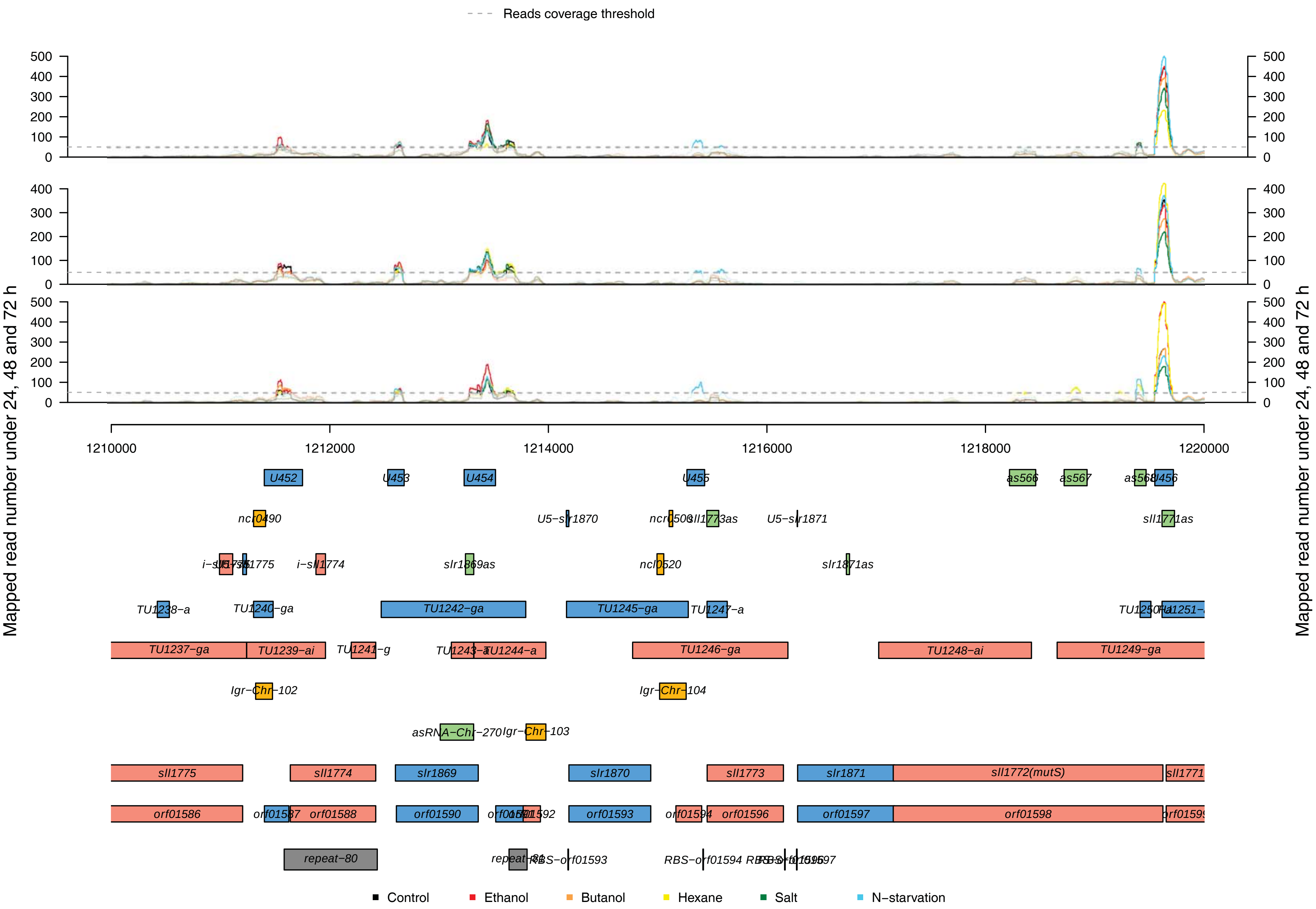

Reads coverage threshold
Mapped read number under 24, 48 and 72 h
1210000
1212000
1214000
1216000
1218000
1220000
U452
U453
U454
U455
as566
as567
ncl0490
U5−slr1870
sll1773as
U5−slr1871
sll1771as
i−sll1774
slr1869as
ncl0520
slr1871as
TU1238−a
TU1240−ga
TU1242−ga
TU1245−ga
TU1247−a
TU1237−ga
TU1239−ai
TU1241−g
TU1244−a
TU1246−ga
TU1248−ai
TU1249−ga
Igr−Chr−102
Igr−Chr−104
asRNA−Chr−270
Igr−Chr−103
sll1775
sll1774
slr1869
slr1870
sll1773
slr1871
sll1772(mutS)
sll1771
orf01586
orf01588
orf01590
orf01593
orf01596
orf01597
orf01598
repeat−80
RBS−orf01593
Control
Ethanol
Butanol
Hexane
Salt
N−starvation

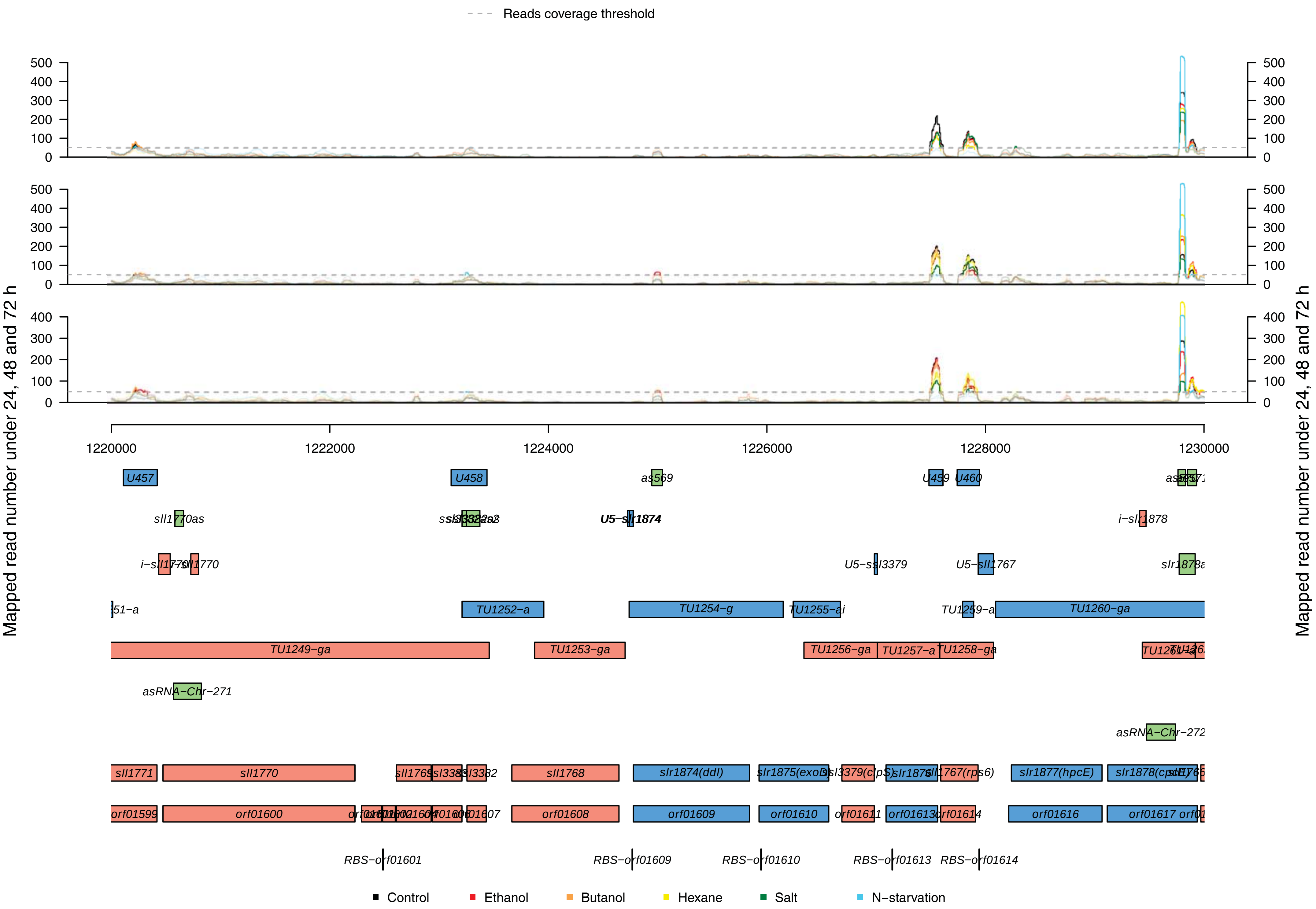
Reads coverage threshold
Mapped read number under 24, 48 and 72 h
1220000
1222000
1224000
1226000
1228000
1230000
U457
U458
as569
U459
U460
sll1770as
i-sll1770
sll1770
U5-slr1874
U5-ssl3379
U5-sll1767
i-slr1878
TU1252-a
TU1254-g
TU1260-ga
TU1249-ga
TU1253-ga
TU1256-ga
TU1257-a
TU1258-ga
asRNA-Chr-271
asRNA-Chr-272
sll1771
sll1770
sll1768
slr1874(ddl)
slr1877(hpcE)
orf01599
orf01600
orf01608
orf01609
orf01610
orf01611
orf01613
orf01614
orf01616
orf01617
RBS-orf01601
RBS-orf01609
RBS-orf01610
RBS-orf01613
RBS-orf01614
Control
Ethanol
Butanol
Hexane
Salt
N-starvation

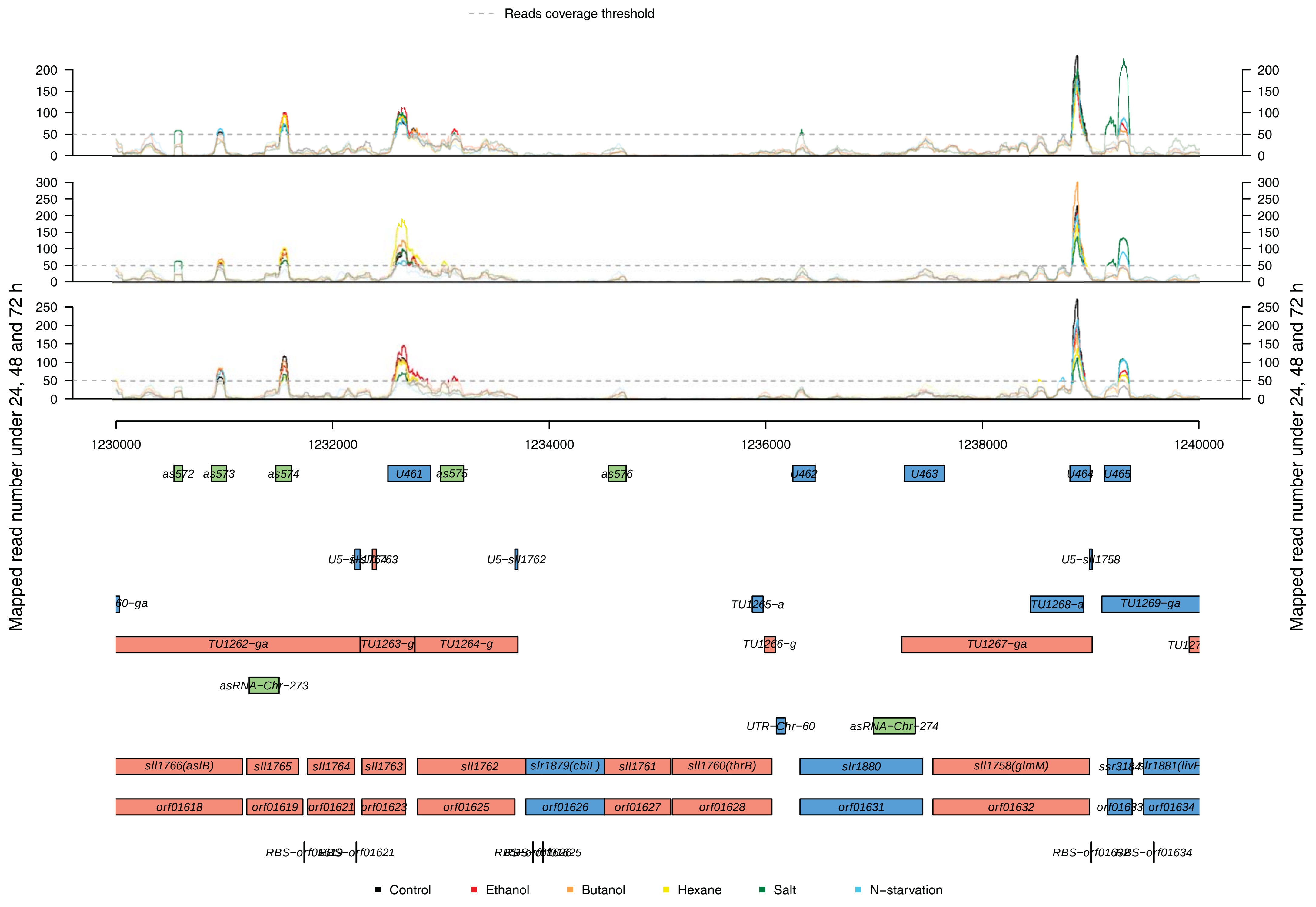

Reads coverage threshold
Mapped read number under 24, 48 and 72 h
1230000
1232000
1234000
1236000
1238000
1240000
as572
as573
as574
U461
as575
as576
U462
U463
U464
U465
U5−sll1762
U5−sll1758
TU1262−ga
TU1264−g
TU1265−a
TU1267−ga
TU1268−a
TU1269−ga
asRNA−Chr−273
UTR−Chr−60
asRNA−Chr−274
sll1766(asIB)
sll1765
sll1764
sll1763
sll1762
slr1879(cbiL)
sll1761
sll1760(thrB)
slr1880
sll1758(glmM)
orf01618
orf01619
orf01621
orf01623
orf01625
orf01626
orf01627
orf01628
orf01631
orf01632
orf01634
Control
Ethanol
Butanol
Hexane
Salt
N−starvation

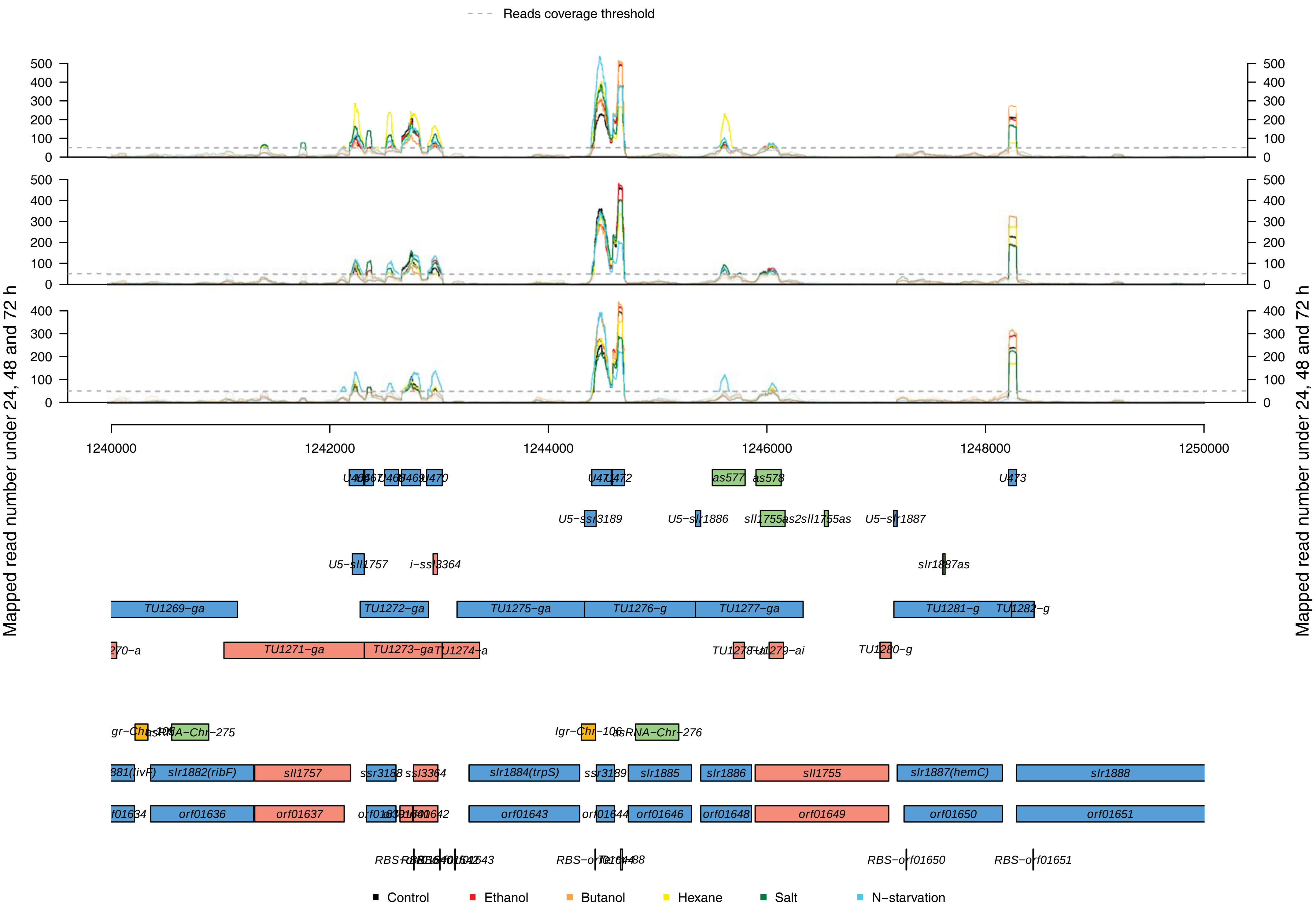

Reads coverage threshold
Mapped read number under 24, 48 and 72 h
1240000
1242000
1244000
1246000
1248000
1250000
TU1269−ga
TU1271−ga
TU1272−ga
TU1273−ga
TU1275−ga
TU1276−g
TU1277−ga
TU1281−g
TU1280−g
slr1882(ribF)
sll1757
slr1884(trpS)
slr1885
slr1886
sll1755
slr1887(hemC)
slr1888
orf01636
orf01637
orf01643
orf01646
orf01648
orf01649
orf01650
orf01651
RBS−orf01650
RBS−orf01651
Control
Ethanol
Butanol
Hexane
Salt
N−starvation

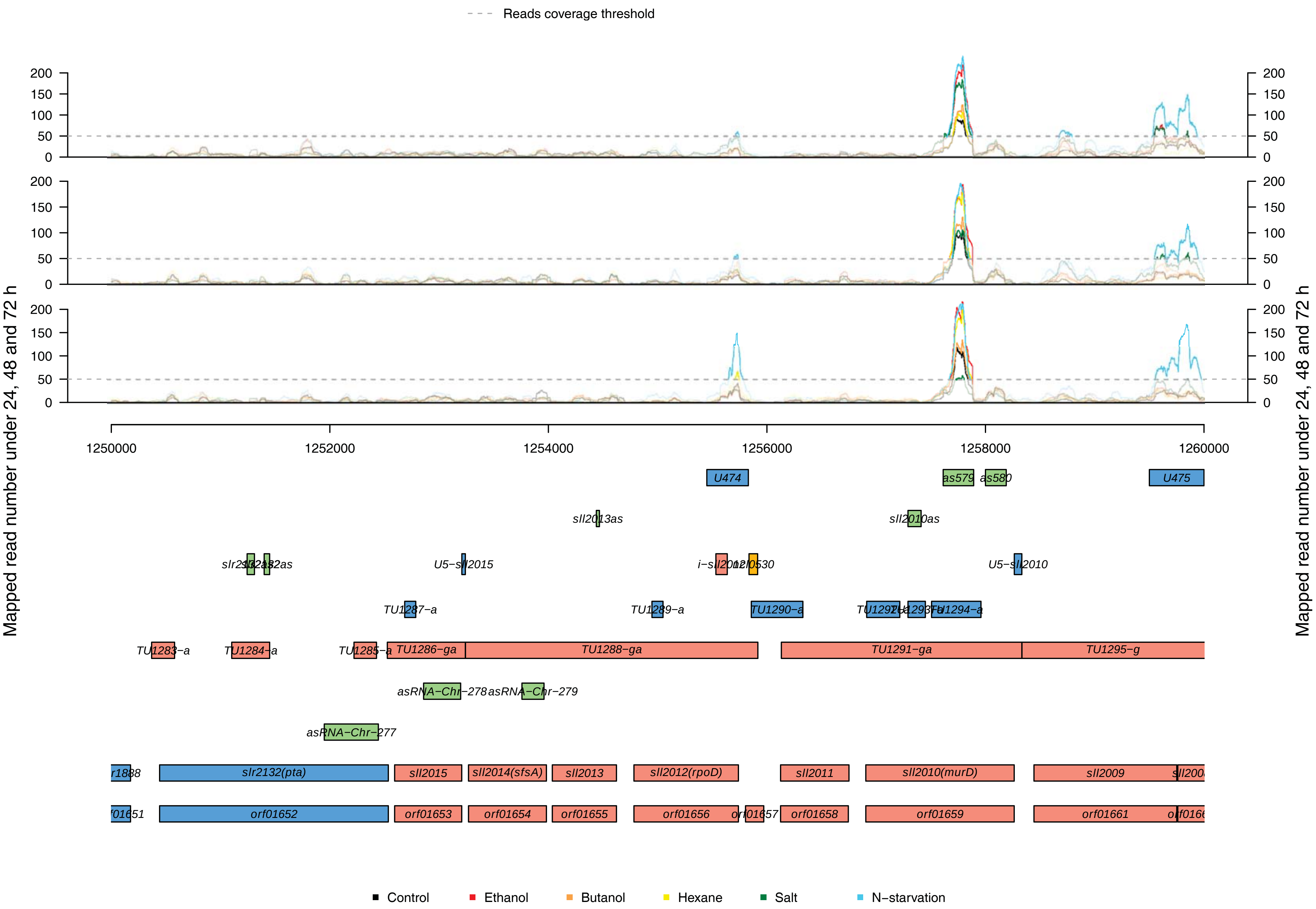

Reads coverage threshold
Mapped read number under 24, 48 and 72 h
1250000
1252000
1254000
1256000
1258000
1260000
U474
as579
as580
U475
sll2013as
sll2010as
U5−sll2015
U5−sll2010
TU1287−a
TU1289−a
TU1290−a
TU1283−a
TU1284−a
TU1285−a
TU1286−ga
TU1288−ga
TU1291−ga
TU1295−g
asRNA−Chr−278
asRNA−Chr−279
asRNA−Chr−277
slr2132(pta)
sll2015
sll2014(sfsA)
sll2013
sll2012(rpoD)
sll2011
sll2010(murD)
sll2009
orf01652
orf01653
orf01654
orf01655
orf01656
orf01658
orf01659
orf01661
Control
Ethanol
Butanol
Hexane
Salt
N−starvation

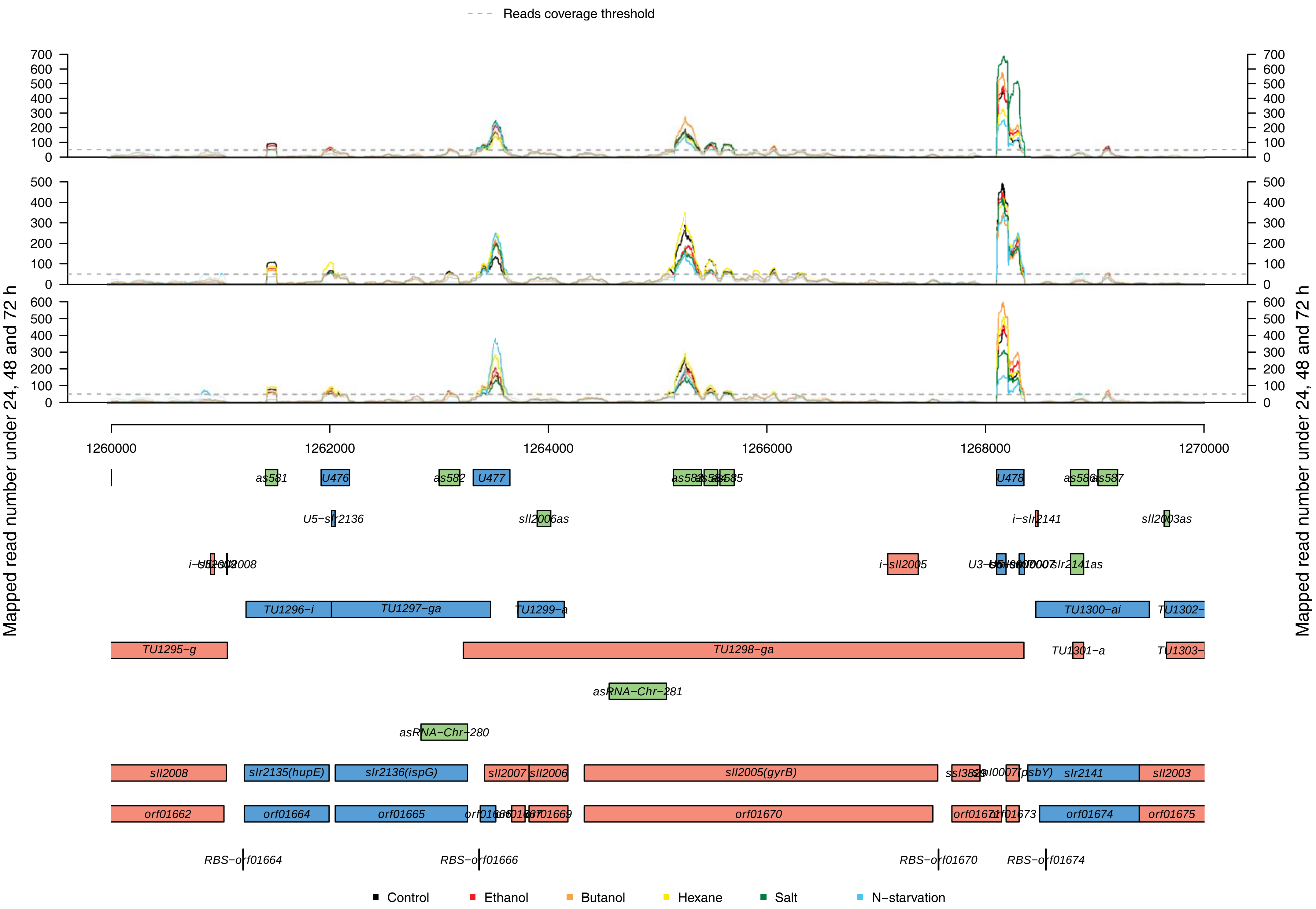

Reads coverage threshold
Mapped read number under 24, 48 and 72 h
1260000
1262000
1264000
1266000
1268000
1270000
as581
U476
as582
U477
U478
U5−slr2136
sll2006as
i−slr2141
sll2003as
i−sll2005
TU1296−i
TU1297−ga
TU1299−a
TU1300−ai
TU1302−
TU1295−g
TU1298−ga
TU1301−a
TU1303−
asRNA−Chr−281
asRNA−Chr−280
sll2008
slr2135(hupE)
slr2136(ispG)
sll2007
sll2006
sll2005(gyrB)
sml0007(psbY)
slr2141
sll2003
orf01662
orf01664
orf01665
orf01670
orf01673
orf01674
orf01675
RBS−orf01664
RBS−orf01666
RBS−orf01670
RBS−orf01674
Control
Ethanol
Butanol
Hexane
Salt
N−starvation

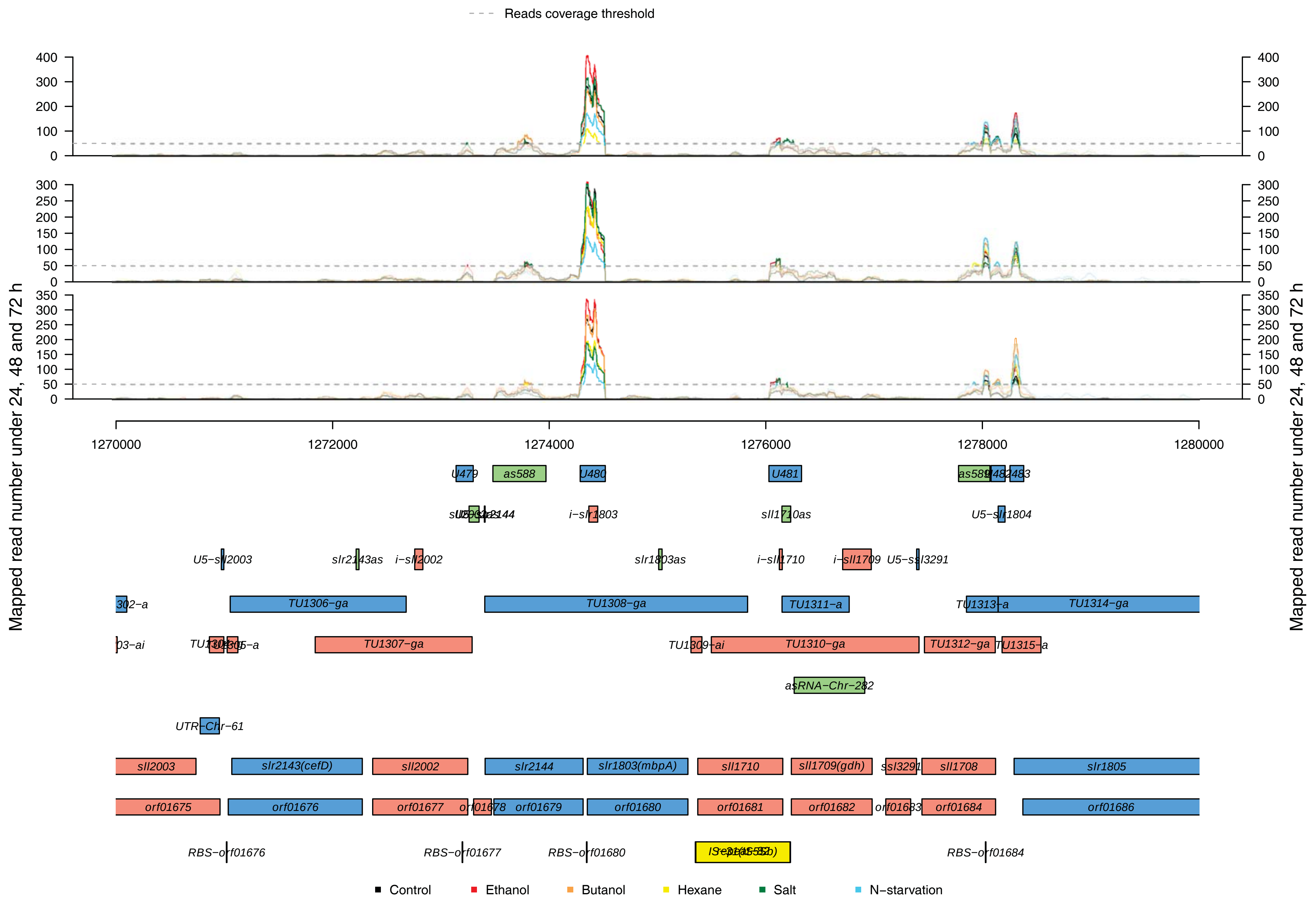

Reads coverage threshold
Mapped read number under 24, 48 and 72 h
1270000
1272000
1274000
1276000
1278000
1280000
U479
as588
U480
U481
i−slr1803
sll1710as
U5−slr1804
U5−sll2003
slr2143as
i−sll2002
slr1803as
i−sll1710
i−sll1709
U5−ssl3291
TU1306−ga
TU1308−ga
TU1311−a
TU1314−ga
TU1307−ga
TU1310−ga
TU1312−ga
asRNA−Chr−282
UTR−Chr−61
sll2003
slr2143(cefD)
sll2002
slr2144
slr1803(mbpA)
sll1710
sll1709(gdh)
ssl3291
sll1708
slr1805
orf01675
orf01676
orf01677
orf01679
orf01680
orf01681
orf01682
orf01684
orf01686
RBS−orf01676
RBS−orf01677
RBS−orf01680
RBS−orf01684
Control
Ethanol
Butanol
Hexane
Salt
N−starvation

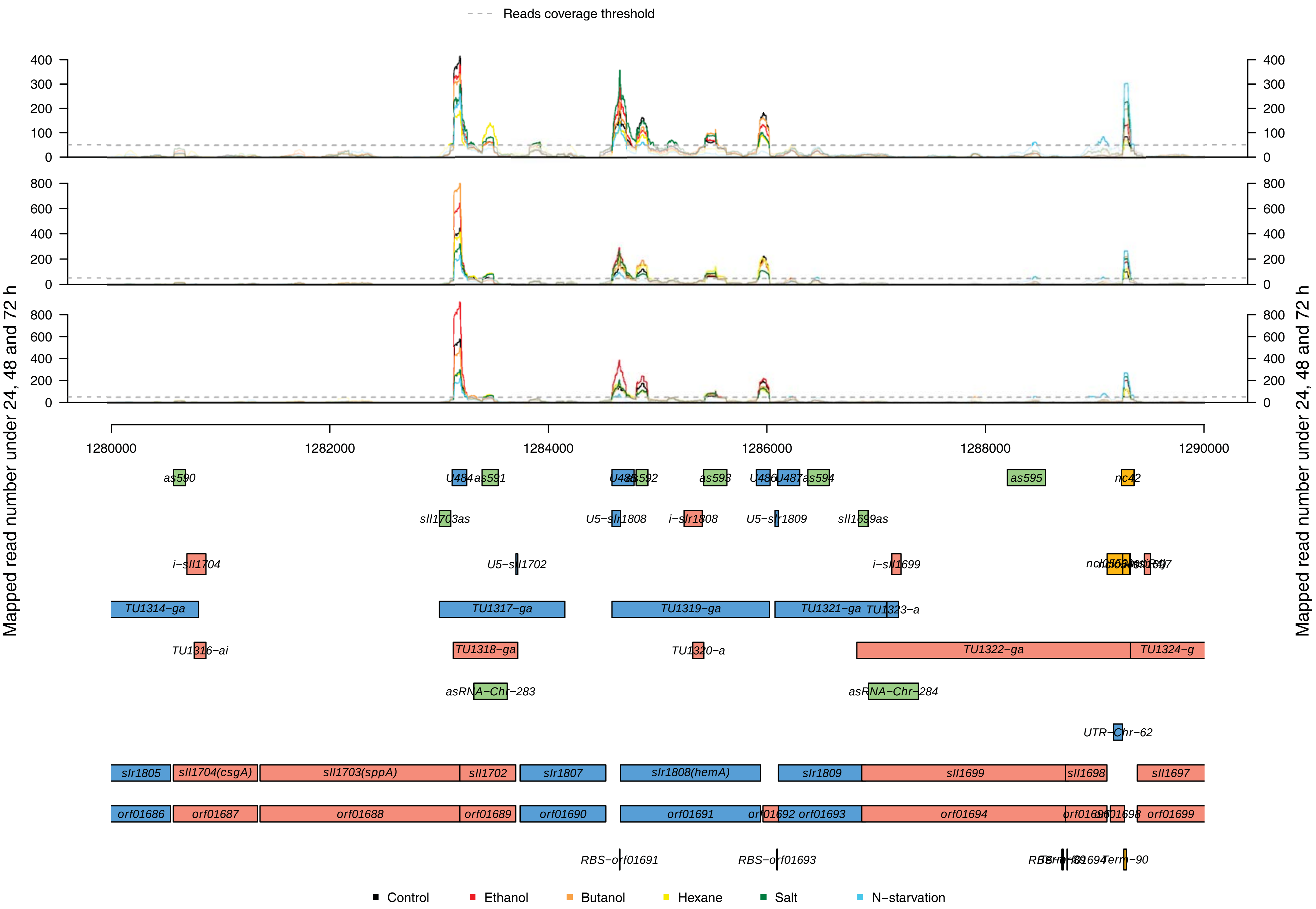

Reads coverage threshold
Mapped read number under 24, 48 and 72 h
400
300
200
100
0
800
600
400
200
0
1280000
1282000
1284000
1286000
1288000
1290000
as590
U484
as591
as592
as593
U487
as594
as595
nc42
sll1703as
U5−slr1808
i−slr1808
U5−slr1809
sll1699as
i−sll1704
U5−sll1702
i−sll1699
TU1314−ga
TU1317−ga
TU1319−ga
TU1321−ga
TU1323−a
TU1316−ai
TU1318−ga
TU1320−a
TU1322−ga
TU1324−g
asRNA−Chr−283
asRNA−Chr−284
UTR−Chr−62
slr1805
sll1704(csgA)
sll1703(sppA)
sll1702
slr1807
slr1808(hemA)
slr1809
sll1699
sll1698
sll1697
orf01686
orf01687
orf01688
orf01689
orf01690
orf01691
orf01693
orf01694
orf01699
RBS−orf01691
RBS−orf01693
Term−90
Control
Ethanol
Butanol
Hexane
Salt
N−starvation

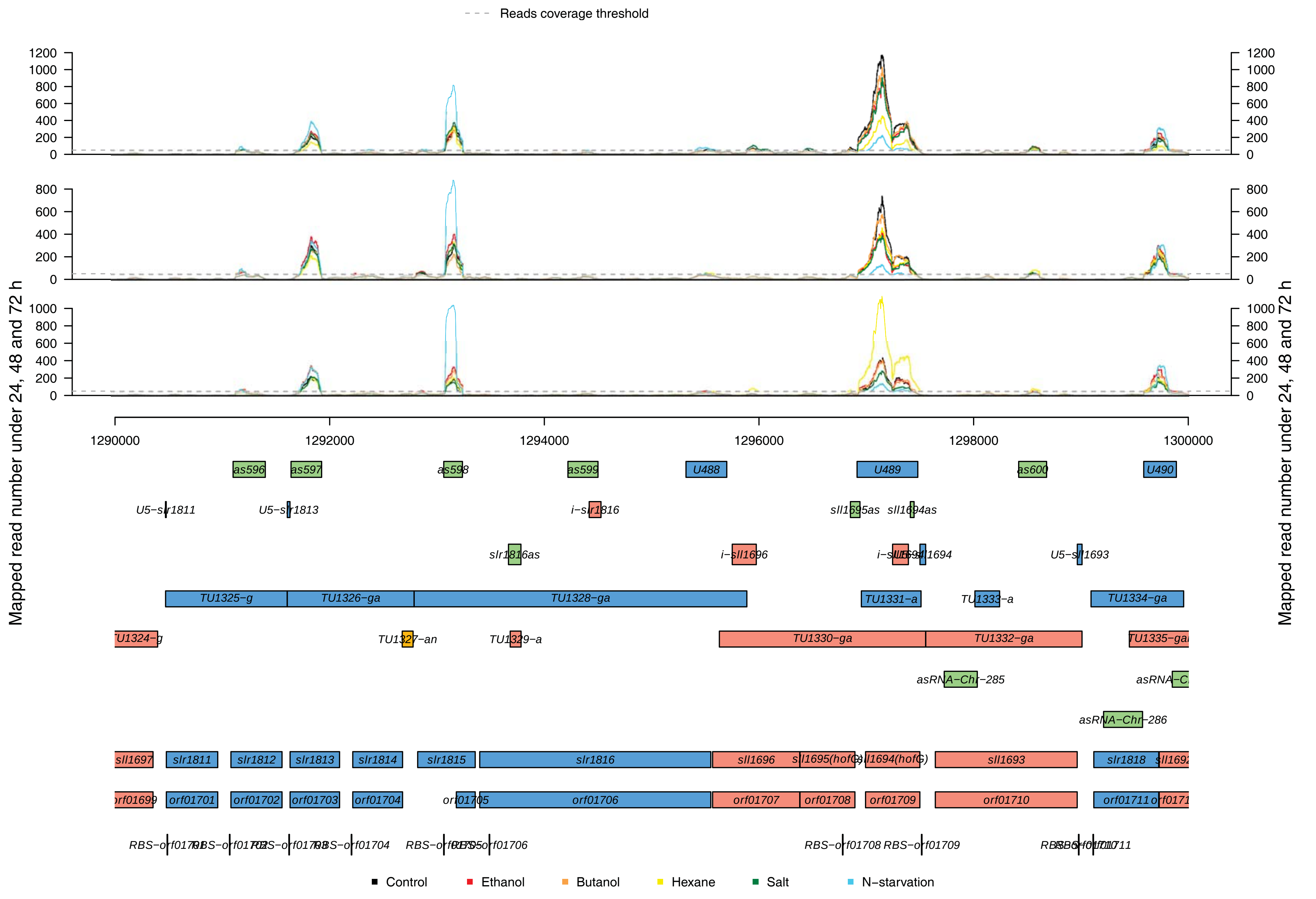

Reads coverage threshold
Mapped read number under 24, 48 and 72 h
1290000
1292000
1294000
1296000
1298000
1300000
as596
as597
as598
as599
U488
U489
as600
U490
U5−slr1811
U5−slr1813
i−slr1816
sll1695as
sll1694as
slr1816as
i−sll1696
U5−sll1693
TU1325−g
TU1326−ga
TU1328−ga
TU1331−a
TU1333−a
TU1334−ga
TU1330−ga
TU1332−ga
asRNA−Chr−285
asRNA−Chr−286
sll1697
slr1811
slr1812
slr1813
slr1814
slr1815
slr1816
sll1696
sll1693
slr1818
orf01701
orf01702
orf01703
orf01704
orf01706
orf01707
orf01708
orf01709
orf01710
orf01711
RBS−orf01704
RBS−orf01706
RBS−orf01708
RBS−orf01709
Control
Ethanol
Butanol
Hexane
Salt
N−starvation

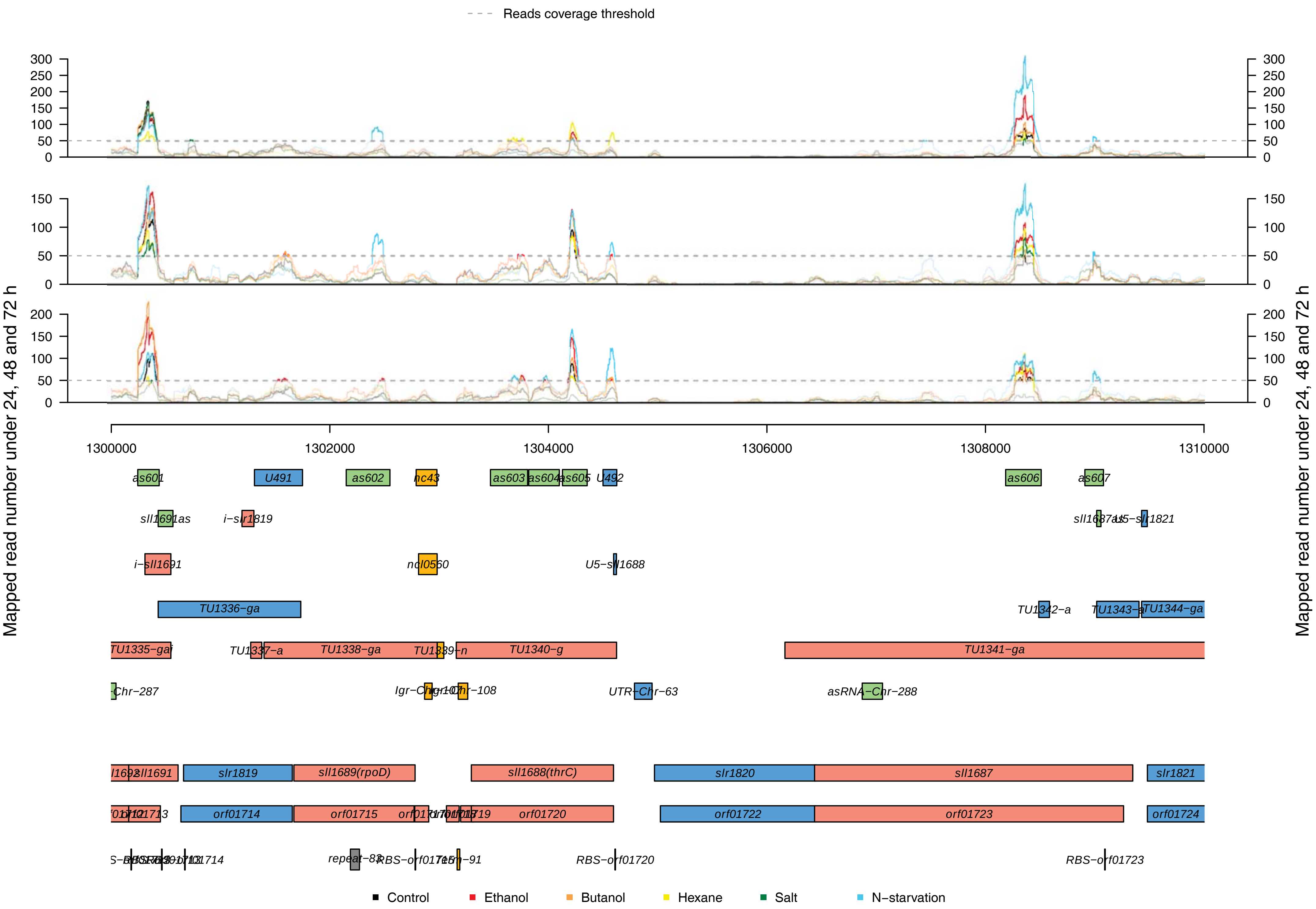
Reads coverage threshold
Mapped read number under 24, 48 and 72 h
1300000
1302000
1304000
1306000
1308000
1310000
as601
U491
as602
nc43
as603
as604
as605
U492
as606
as607
sll1691as
i−slr1819
i−sll1691
ncl0560
U5−sll1688
TU1336−ga
TU1342−a
TU1344−ga
TU1335−gai
TU1338−ga
TU1340−g
TU1341−ga
UTR−Chr−63
asRNA−Chr−288
sll1691
slr1819
sll1689(rpoD)
sll1688(thrC)
slr1820
sll1687
slr1821
orf01714
orf01715
orf01720
orf01722
orf01723
orf01724
RBS−orf01720
RBS−orf01723
Control
Ethanol
Butanol
Hexane
Salt
N−starvation

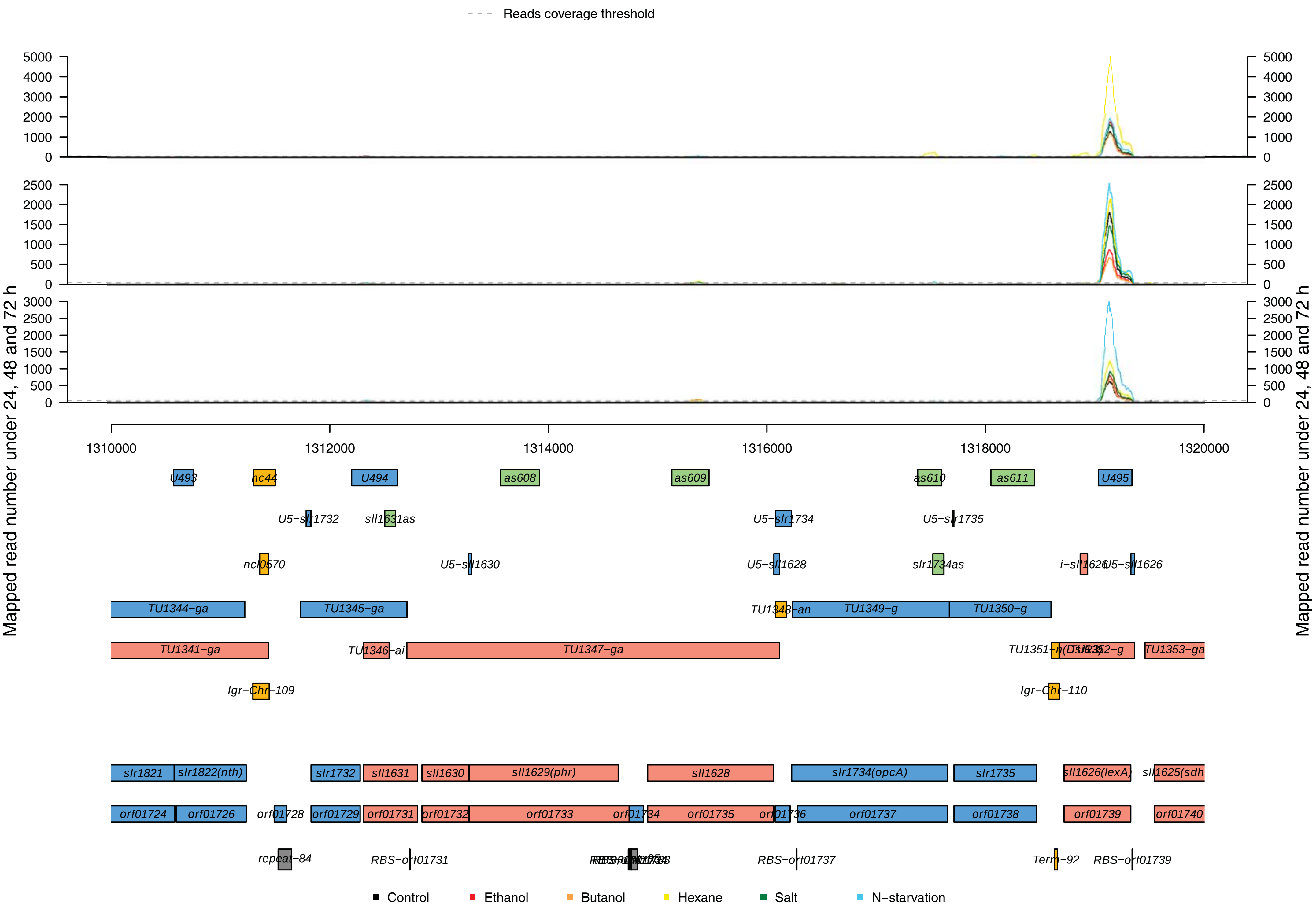
Reads coverage threshold
Mapped read number under 24, 48 and 72 h
5000
4000
3000
2000
1000
0
2500
1500
500
1310000
1312000
1314000
1316000
1318000
1320000
U493
nc44
U494
as608
as609
as610
as611
U495
U5−slr1732
sll1631as
U5−slr1734
U5−slr1735
ncl0570
U5−sll1630
U5−sll1628
slr1734as
i−sll1626
U5−sll1626
TU1344−ga
TU1345−ga
TU1348−an
TU1349−g
TU1350−g
TU1341−ga
TU1346−ai
TU1347−ga
TU1353−ga
Igr−Chr−109
Igr−Chr−110
slr1821
slr1822(nth)
slr1732
sll1631
sll1630
sll1629(phr)
sll1628
slr1734(opcA)
slr1735
sll1626(lexA)
sll1625(sdh
orf01724
orf01726
orf01728
orf01729
orf01731
orf01732
orf01733
orf01734
orf01735
orf01736
orf01737
orf01738
orf01739
orf01740
repeat−84
RBS−orf01731
RBS−orf01737
Term−92
RBS−orf01739
Control
Ethanol
Butanol
Hexane
Salt
N−starvation

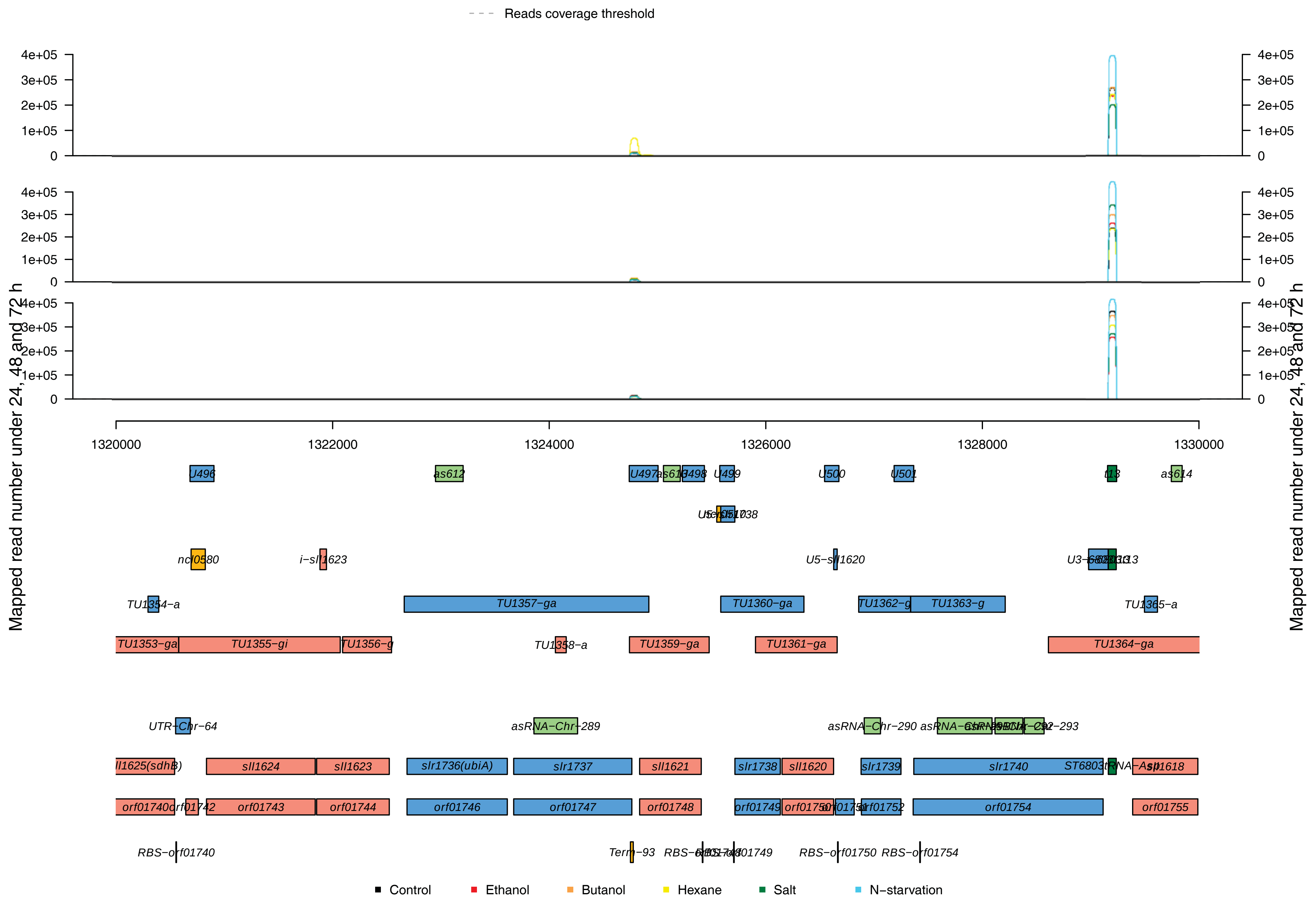

Reads coverage threshold
Mapped read number under 24, 48 and 72 h
4e+05
3e+05
2e+05
1e+05
0
1320000
1322000
1324000
1326000
1328000
1330000
U496
as612
U497
U498
U499
U500
U501
as614
ncl0580
i−sll1623
U5−sll1620
TU1354−a
TU1357−ga
TU1360−ga
TU1363−g
TU1365−a
TU1355−gi
TU1359−ga
TU1361−ga
TU1364−ga
UTR−Chr−64
asRNA−Chr−289
asRNA−Chr−290
sll1624
sll1623
slr1736(ubiA)
slr1737
sll1621
slr1738
sll1620
slr1739
slr1740
orf01743
orf01744
orf01746
orf01747
orf01748
orf01749
orf01754
orf01755
RBS−orf01740
Term−93
RBS−orf01750
RBS−orf01754
Control
Ethanol
Butanol
Hexane
Salt
N−starvation

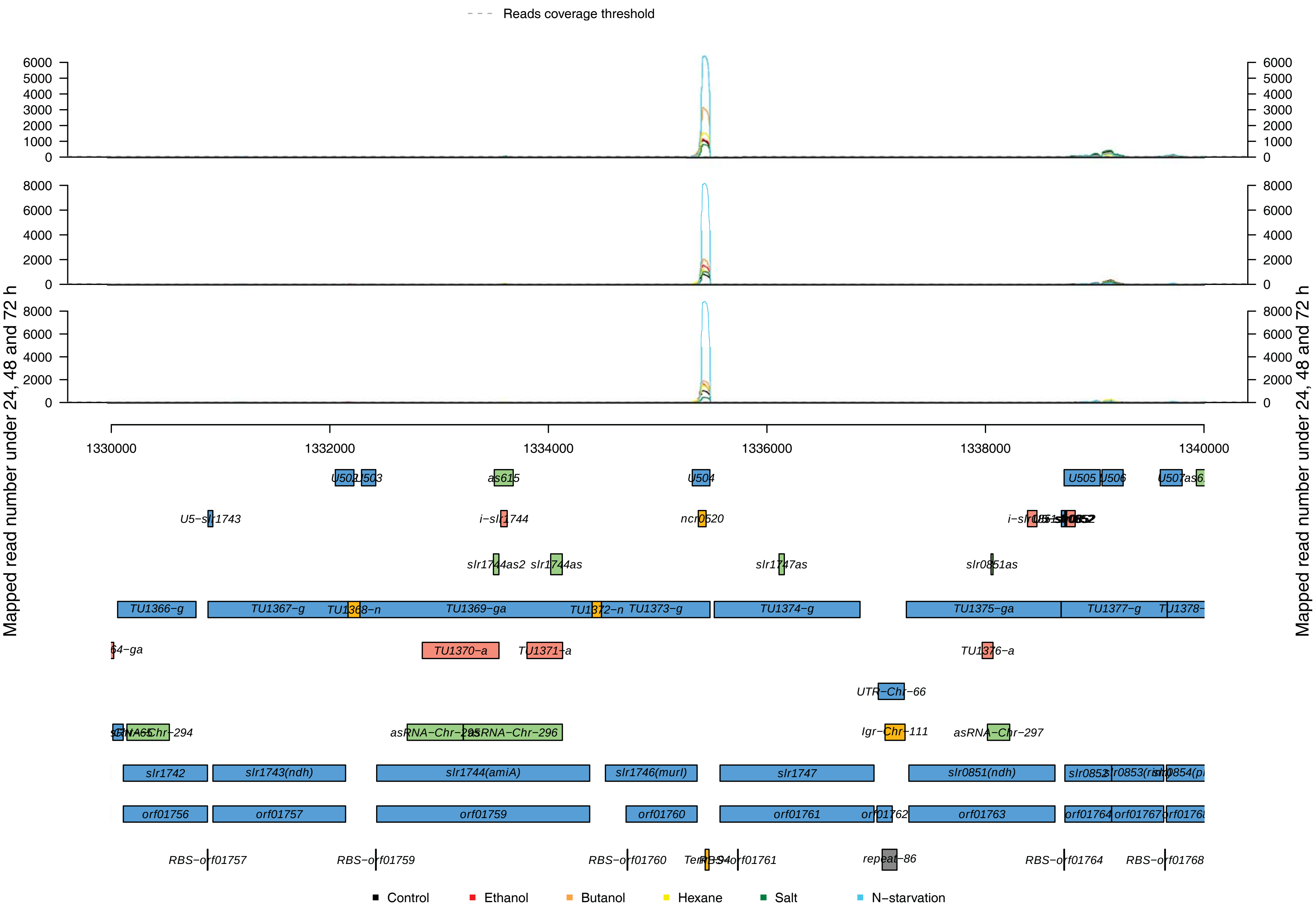

Reads coverage threshold
Mapped read number under 24, 48 and 72 h
1330000
1332000
1334000
1336000
1338000
1340000
U502
U503
as615
U504
U505
U506
U507
U5-slr1743
i-slr1744
ncr0520
slr1744as2
slr1744as
slr1747as
slr0851as
TU1366-g
TU1367-g
TU1368-n
TU1369-ga
TU1372-n
TU1373-g
TU1374-g
TU1375-ga
TU1377-g
TU1370-a
TU1371-a
TU1376-a
UTR-Chr-66
asRNA-Chr-295
asRNA-Chr-296
Igr-Chr-111
asRNA-Chr-297
slr1742
slr1743(ndh)
slr1744(amiA)
slr1746(murI)
slr1747
slr0851(ndh)
orf01756
orf01757
orf01759
orf01760
orf01761
orf01763
RBS-orf01757
RBS-orf01759
RBS-orf01760
RBS-orf01761
repeat-86
RBS-orf01764
RBS-orf01768
Control
Ethanol
Butanol
Hexane
Salt
N-starvation

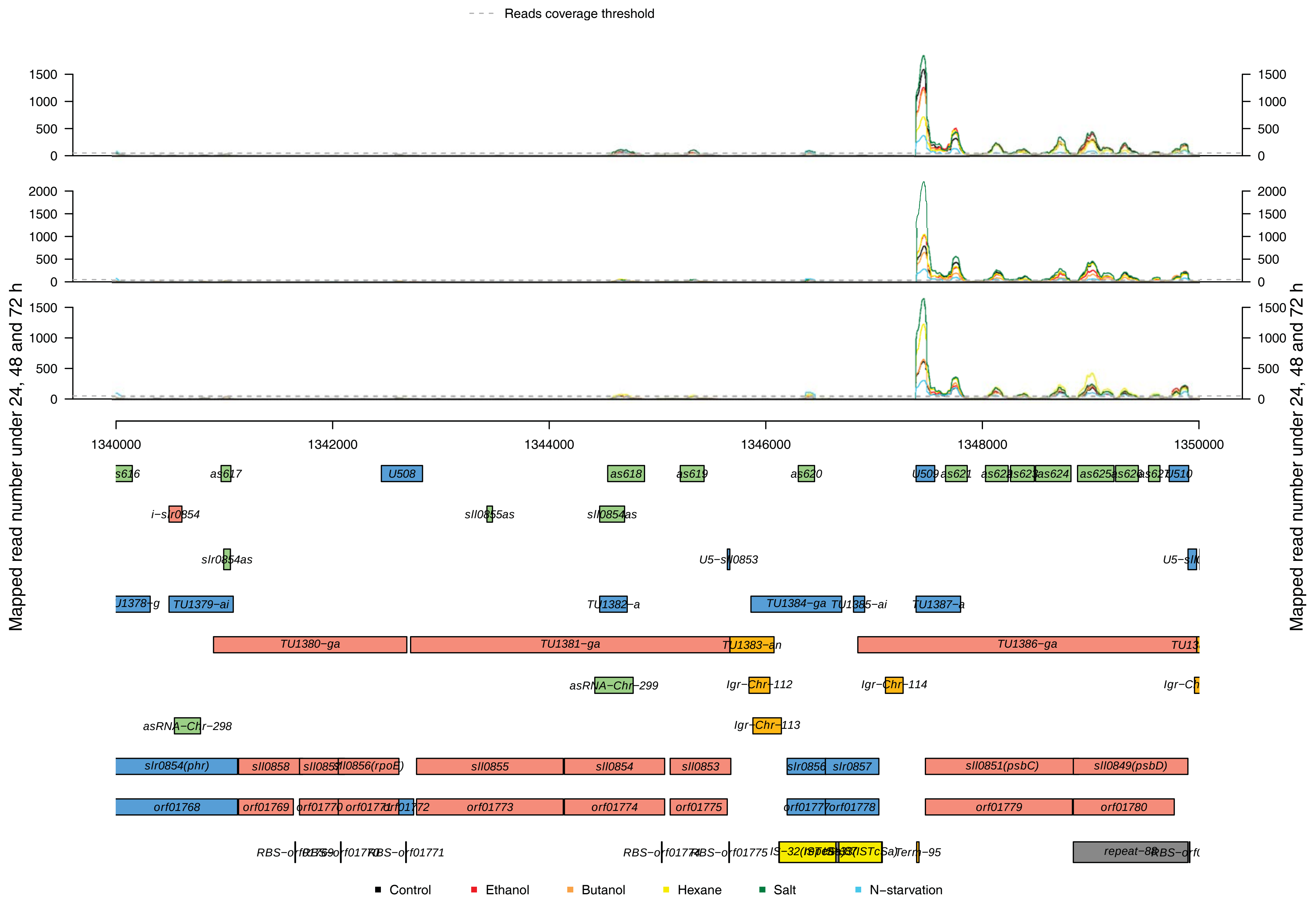
Reads coverage threshold
Mapped read number under 24, 48 and 72 h
1340000
1342000
1344000
1346000
1348000
1350000
TU1380−ga
TU1381−ga
TU1386−ga
slr0854(phr)
sll0858
sll0855
sll0854
sll0853
sll0851(psbC)
sll0849(psbD)
orf01768
orf01769
orf01773
orf01774
orf01775
orf01779
orf01780
Control
Ethanol
Butanol
Hexane
Salt
N−starvation

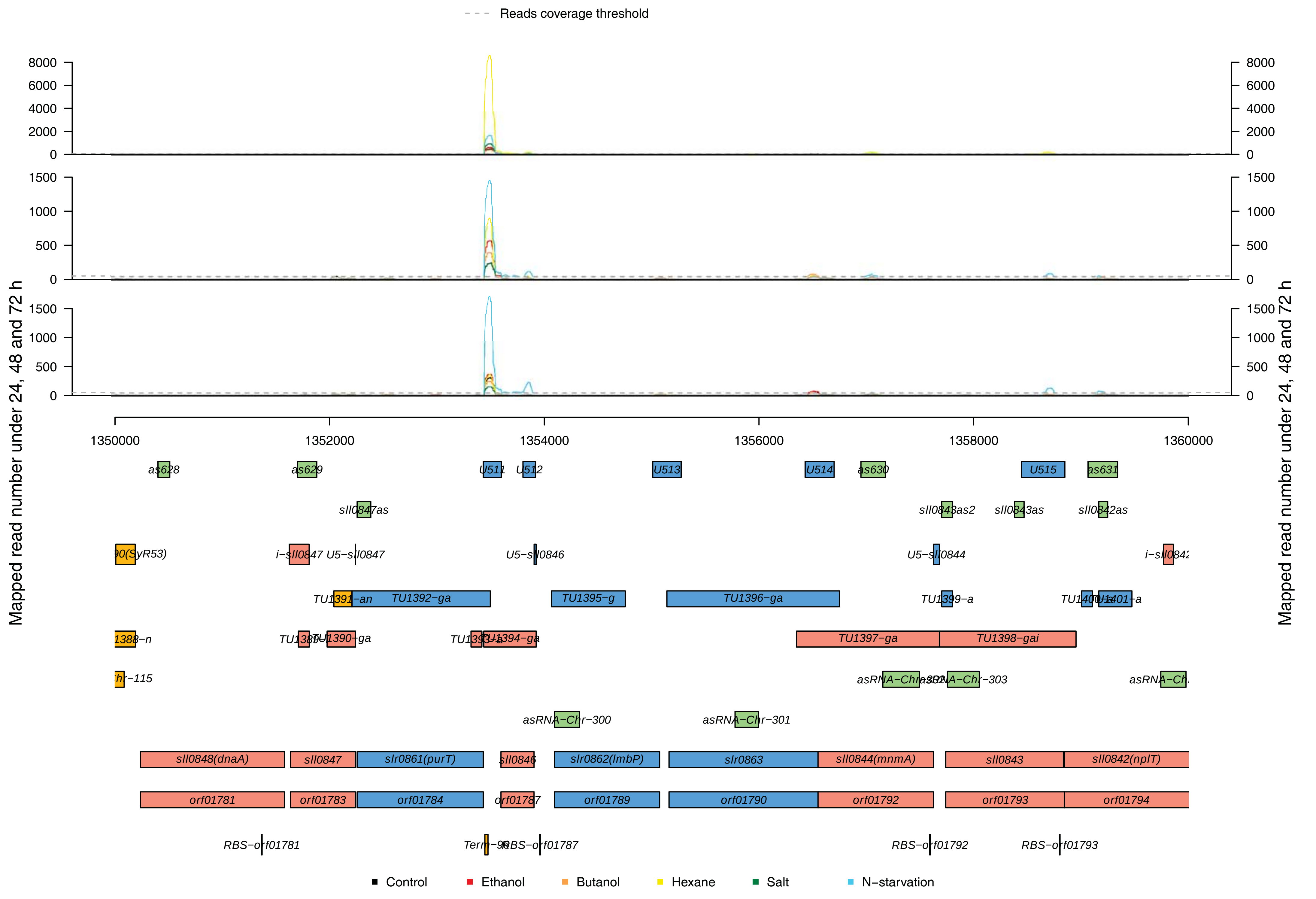

Reads coverage threshold
Mapped read number under 24, 48 and 72 h
1350000
1352000
1354000
1356000
1358000
1360000
as628
as629
U511
U512
U513
U514
as630
U515
as631
sll0847as
sll0843as2
sll0843as
sll0842as
i−sll0847
U5−sll0847
U5−sll0846
U5−sll0844
TU1392−ga
TU1395−g
TU1396−ga
TU1399−a
TU1397−ga
TU1398−gai
asRNA−Chr−303
asRNA−Chr−300
asRNA−Chr−301
sll0848(dnaA)
sll0847
slr0861(purT)
sll0846
slr0862(lmbP)
slr0863
sll0844(mnmA)
sll0843
sll0842(nplT)
orf01781
orf01783
orf01784
orf01787
orf01789
orf01790
orf01792
orf01793
orf01794
RBS−orf01781
RBS−orf01787
RBS−orf01792
RBS−orf01793
Control
Ethanol
Butanol
Hexane
Salt
N−starvation

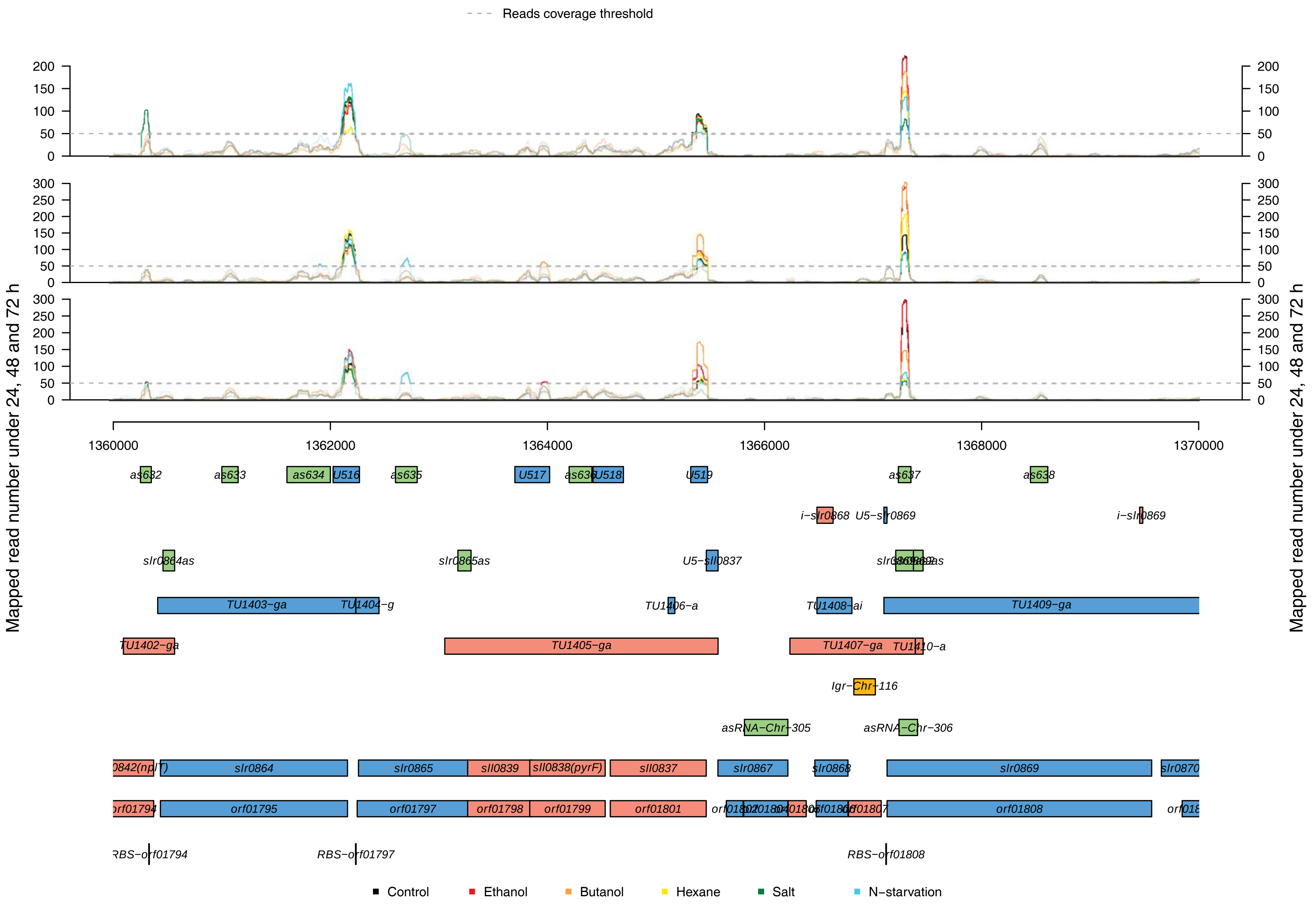
Reads coverage threshold
Mapped read number under 24, 48 and 72 h
1360000
1362000
1364000
1366000
1368000
1370000
as632
as633
as634
U516
as635
U517
as636
U518
U519
as637
as638
i−slr0868
U5−slr0869
i−slr0869
slr0864as
slr0865as
U5−sll0837
TU1403−ga
TU1404−g
TU1406−a
TU1408−ai
TU1409−ga
TU1402−ga
TU1405−ga
TU1407−ga
TU1410−a
Igr−Chr−116
asRNA−Chr−305
asRNA−Chr−306
slr0864
slr0865
sll0839
sll0838(pyrF)
sll0837
slr0867
slr0868
slr0869
orf01794
orf01795
orf01797
orf01798
orf01799
orf01801
orf01807
orf01808
RBS−orf01797
RBS−orf01808
Control
Ethanol
Butanol
Hexane
Salt
N−starvation

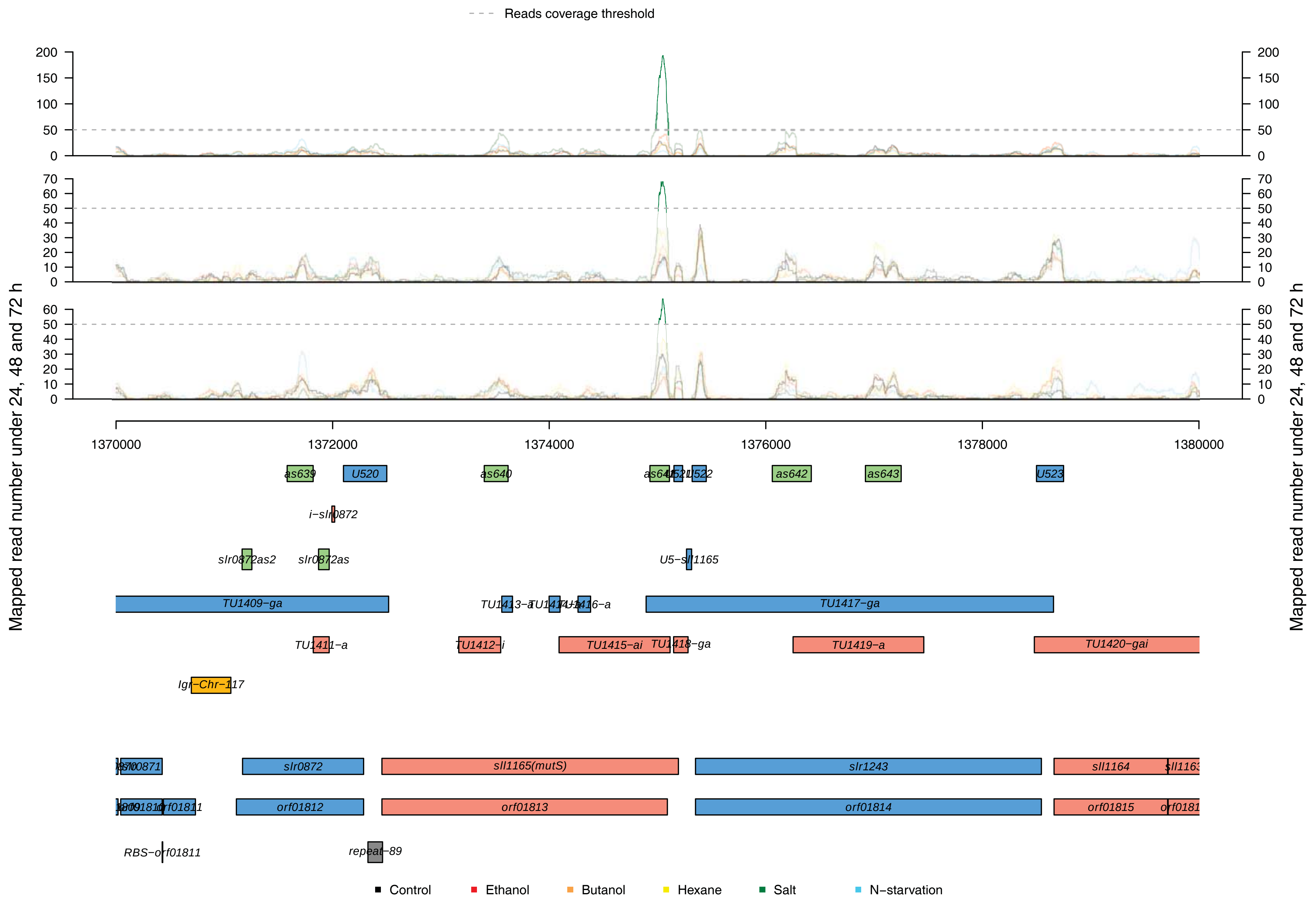

Reads coverage threshold
Mapped read number under 24, 48 and 72 h
1370000
1372000
1374000
1376000
1378000
1380000
as639
U520
as640
as642
as643
U523
U522
i−slr0872
slr0872as2
slr0872as
U5−sll1165
TU1409−ga
TU1417−ga
TU1416−a
TU1411−a
TU1412−i
TU1415−ai
TU1418−ga
TU1419−a
TU1420−gai
Igr−Chr−117
slr0872
sll1165(mutS)
slr1243
sll1164
orf01812
orf01813
orf01814
orf01815
RBS−orf01811
repeat−89
Control
Ethanol
Butanol
Hexane
Salt
N−starvation

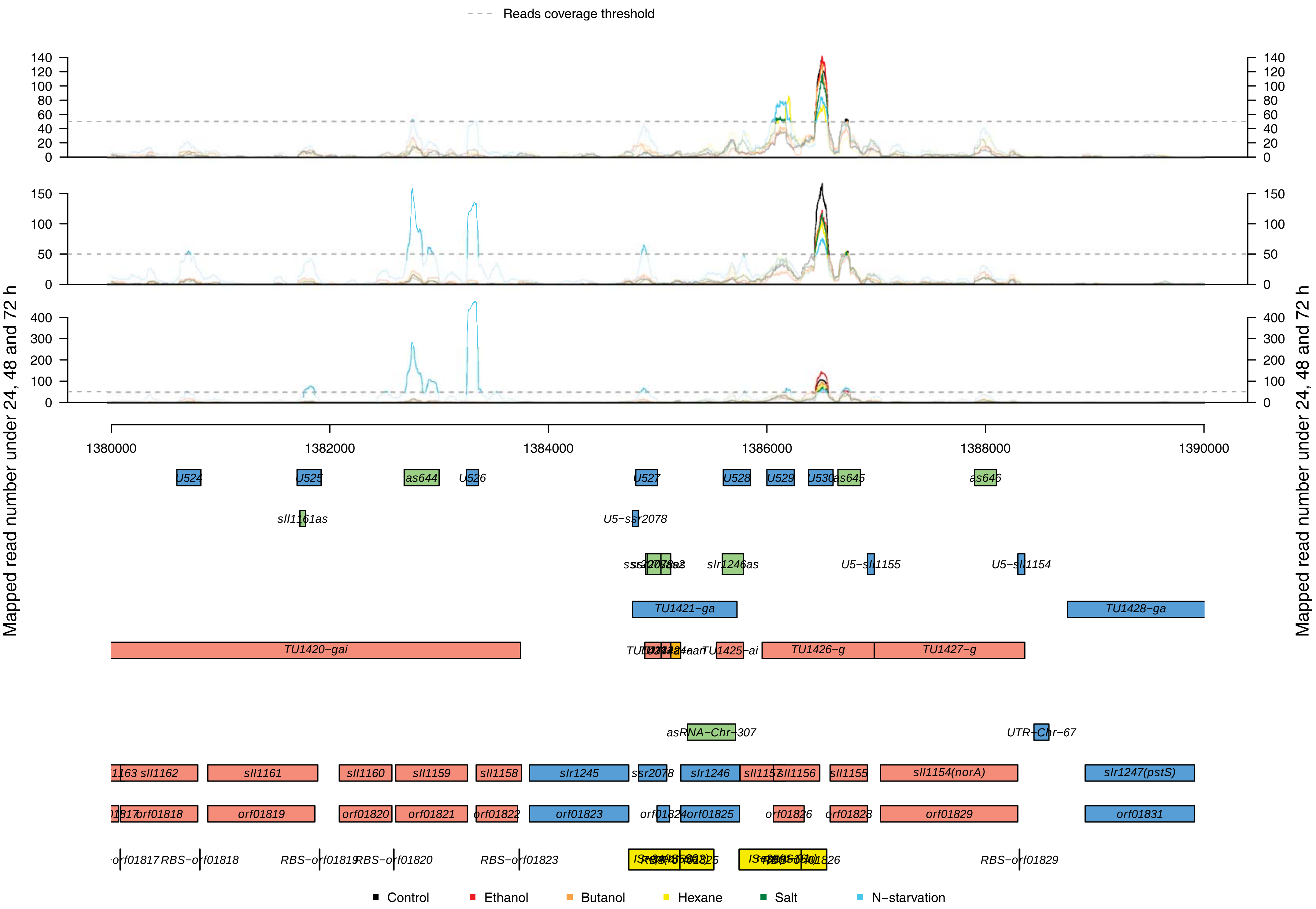

Reads coverage threshold
Mapped read number under 24, 48 and 72 h
0
20
40
60
80
100
120
140
150
200
300
400
1380000
1382000
1384000
1386000
1388000
1390000
U524
U525
as644
U526
U527
U528
U529
as645
as646
sll1161as
U5−ssr2078
slr1246as
U5−sll1155
U5−sll1154
TU1421−ga
TU1428−ga
TU1420−gai
TU1426−g
TU1427−g
asRNA−Chr−307
UTR−Chr−67
sll1162
sll1161
sll1160
sll1159
sll1158
slr1245
ssr2078
slr1246
sll1156
sll1155
sll1154(norA)
slr1247(pstS)
orf01818
orf01819
orf01820
orf01821
orf01822
orf01823
orf01825
orf01826
orf01828
orf01829
orf01831
RBS−orf01818
RBS−orf01819
RBS−orf01820
RBS−orf01823
RBS−orf01829
Control
Ethanol
Butanol
Hexane
Salt
N−starvation

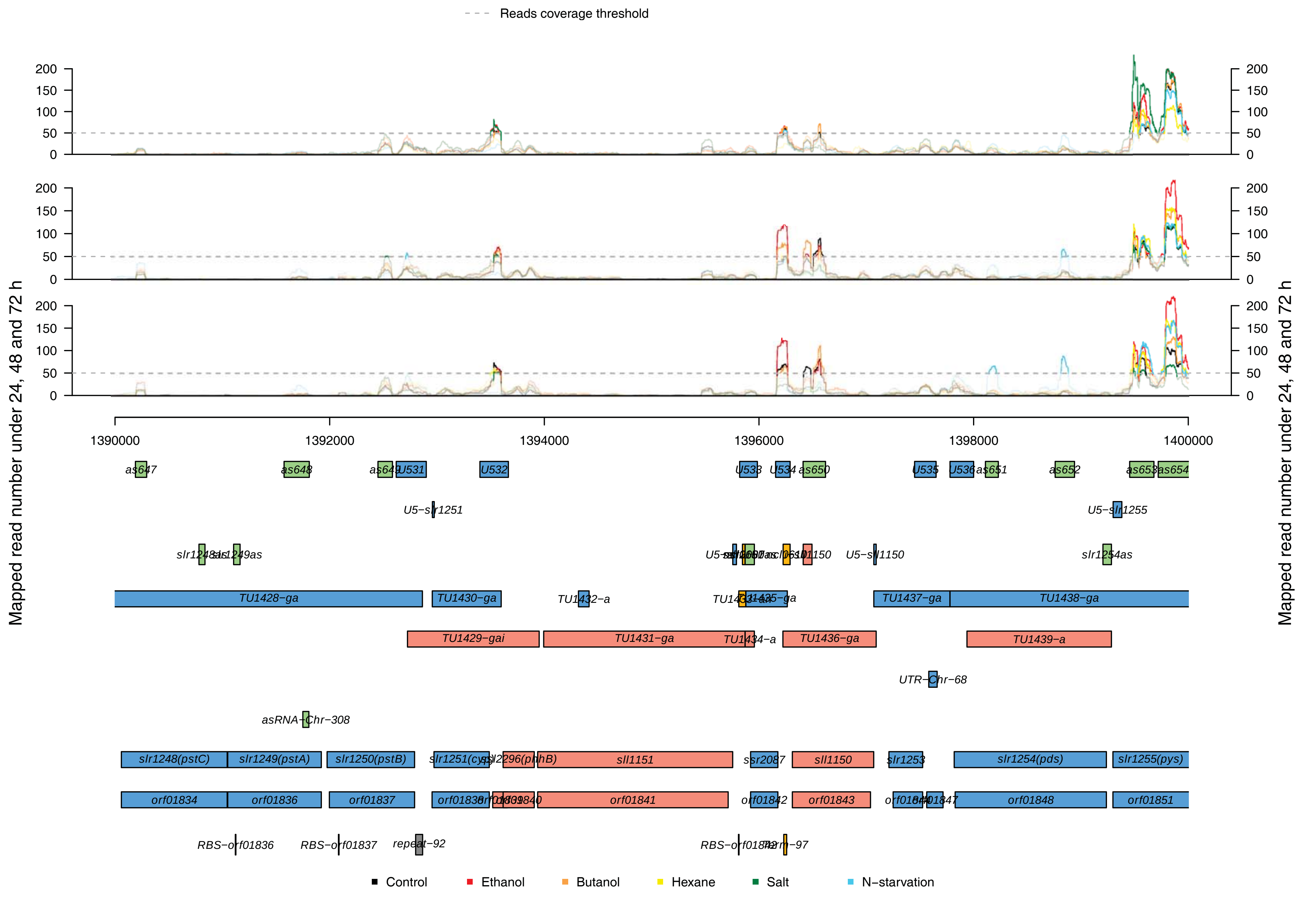

Reads coverage threshold
Mapped read number under 24, 48 and 72 h
0
50
100
150
200
1390000
1392000
1394000
1396000
1398000
1400000
as647
as648
as649
U531
U532
U533
U534
as650
U535
U536
as651
as652
as653
as654
U5−slr1251
U5−slr1255
slr1248as
slr1249as
U5−sll1150
slr1254as
TU1428−ga
TU1430−ga
TU1432−a
TU1437−ga
TU1438−ga
TU1429−gai
TU1431−ga
TU1436−ga
TU1439−a
UTR−Chr−68
asRNA−Chr−308
slr1248(pstC)
slr1249(pstA)
slr1250(pstB)
sll1151
ssr2087
sll1150
slr1253
slr1254(pds)
slr1255(pys)
orf01834
orf01836
orf01837
orf01841
orf01842
orf01843
orf01848
orf01851
RBS−orf01836
RBS−orf01837
repeat−92
Control
Ethanol
Butanol
Hexane
Salt
N−starvation

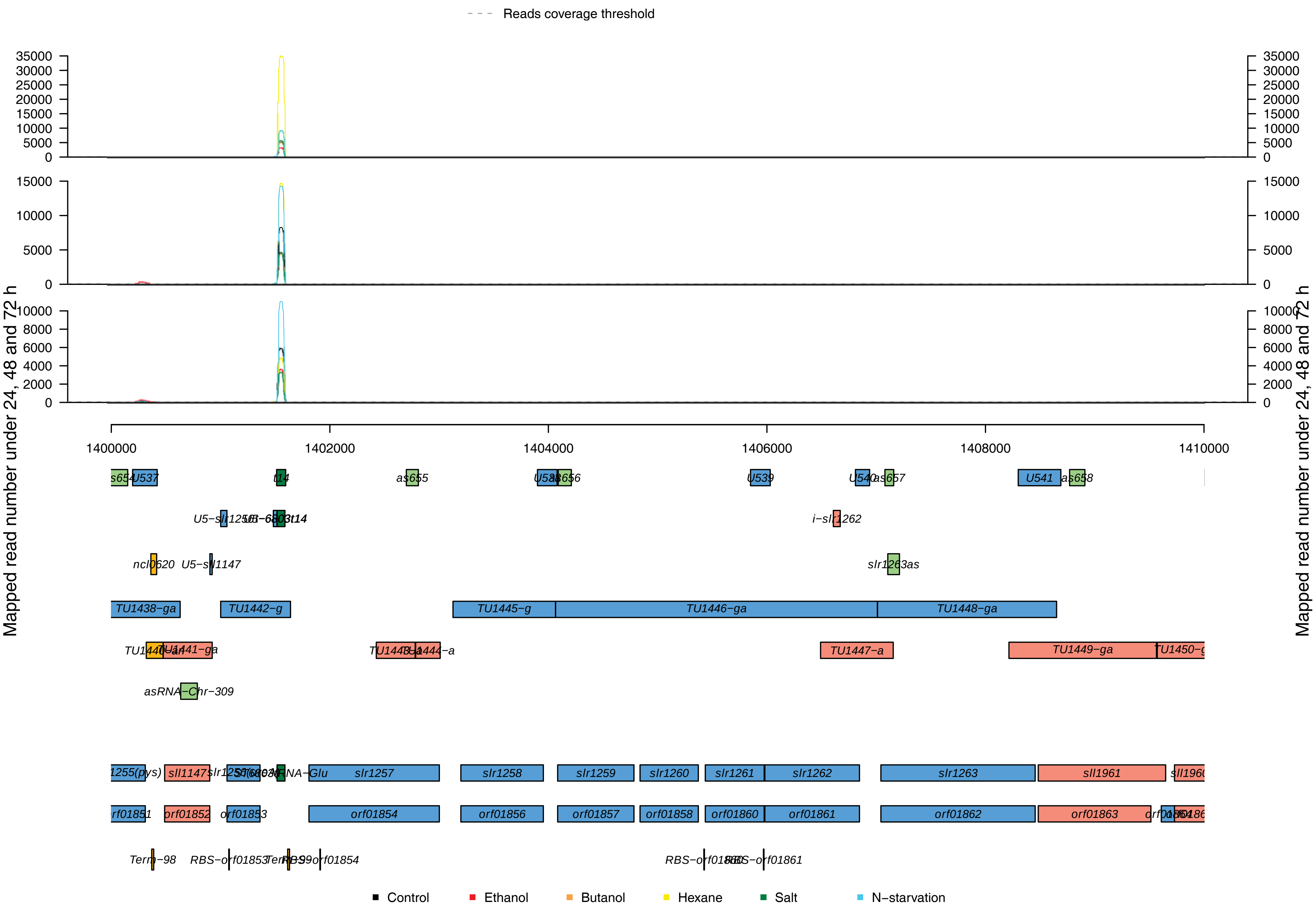

Reads coverage threshold
Mapped read number under 24, 48 and 72 h
1400000
1402000
1404000
1406000
1408000
1410000
U537
t14
as655
U539
U541
as658
i−sll1262
ncl0620
U5−sll1147
slr1263as
TU1438−ga
TU1442−g
TU1445−g
TU1446−ga
TU1448−ga
TU1447−a
TU1449−ga
asRNA−Chr−309
slr1257
slr1258
slr1259
slr1260
slr1261
slr1262
slr1263
sll1961
sll1147
orf01852
orf01853
orf01854
orf01856
orf01857
orf01858
orf01860
orf01861
orf01862
orf01863
Term−98
RBS−orf01853
RBS−orf01861
Control
Ethanol
Butanol
Hexane
Salt
N−starvation

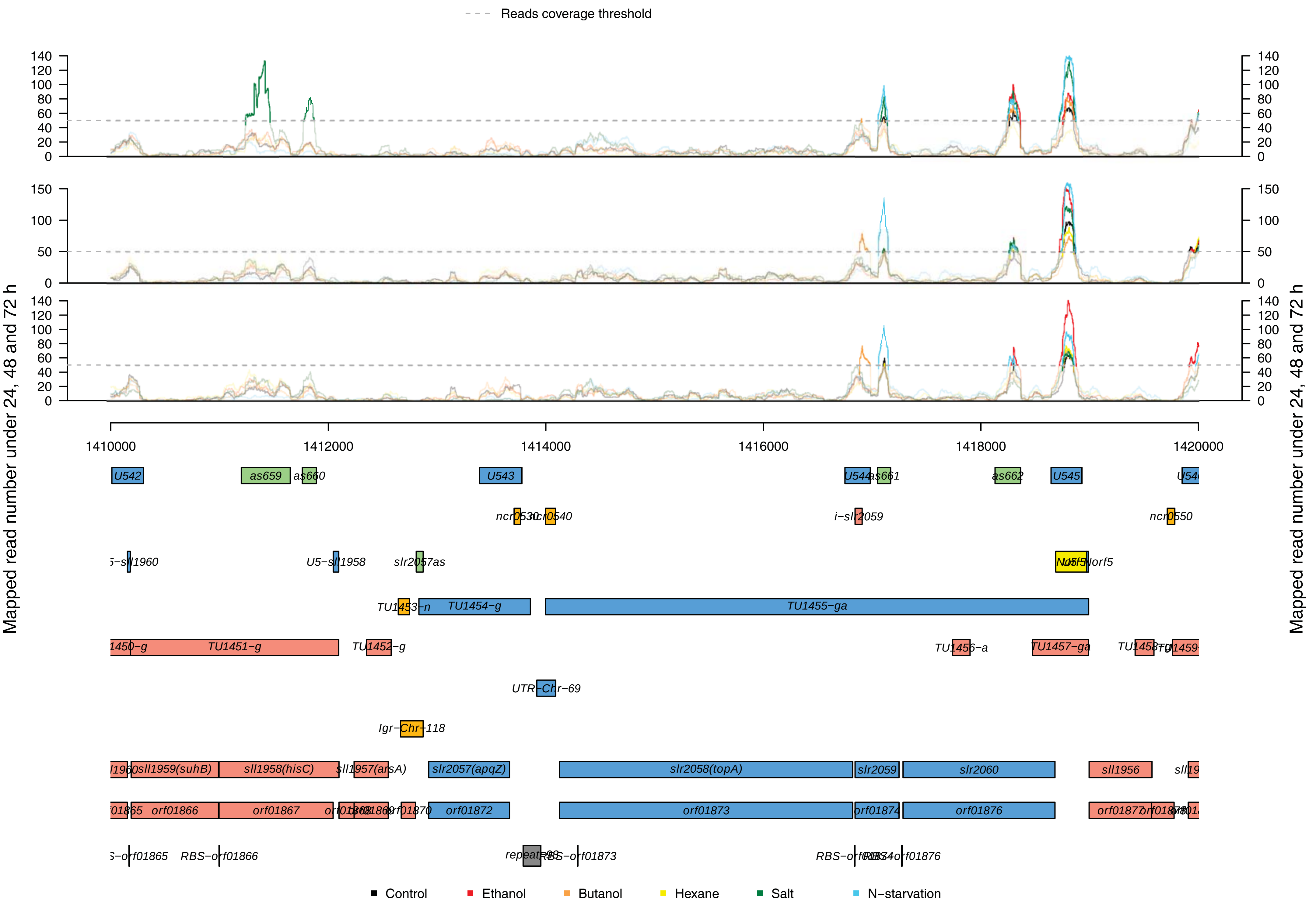

Reads coverage threshold
Mapped read number under 24, 48 and 72 h
1410000
1412000
1414000
1416000
1418000
1420000
U542
as659
as660
U543
U544
as661
as662
U545
ncr0530
ncr0540
i-slr2059
ncr0550
U5-sll1958
slr2057as
TU1453-n
TU1454-g
TU1455-ga
TU1451-g
TU1452-g
TU1456-a
TU1457-ga
UTR-Chr-69
Igr-Chr-118
sll1959(suhB)
sll1958(hisC)
sll1957(arsA)
slr2057(apqZ)
slr2058(topA)
slr2059
slr2060
sll1956
orf01866
orf01867
orf01872
orf01873
orf01874
orf01876
RBS-orf01866
RBS-orf01873
Control
Ethanol
Butanol
Hexane
Salt
N-starvation

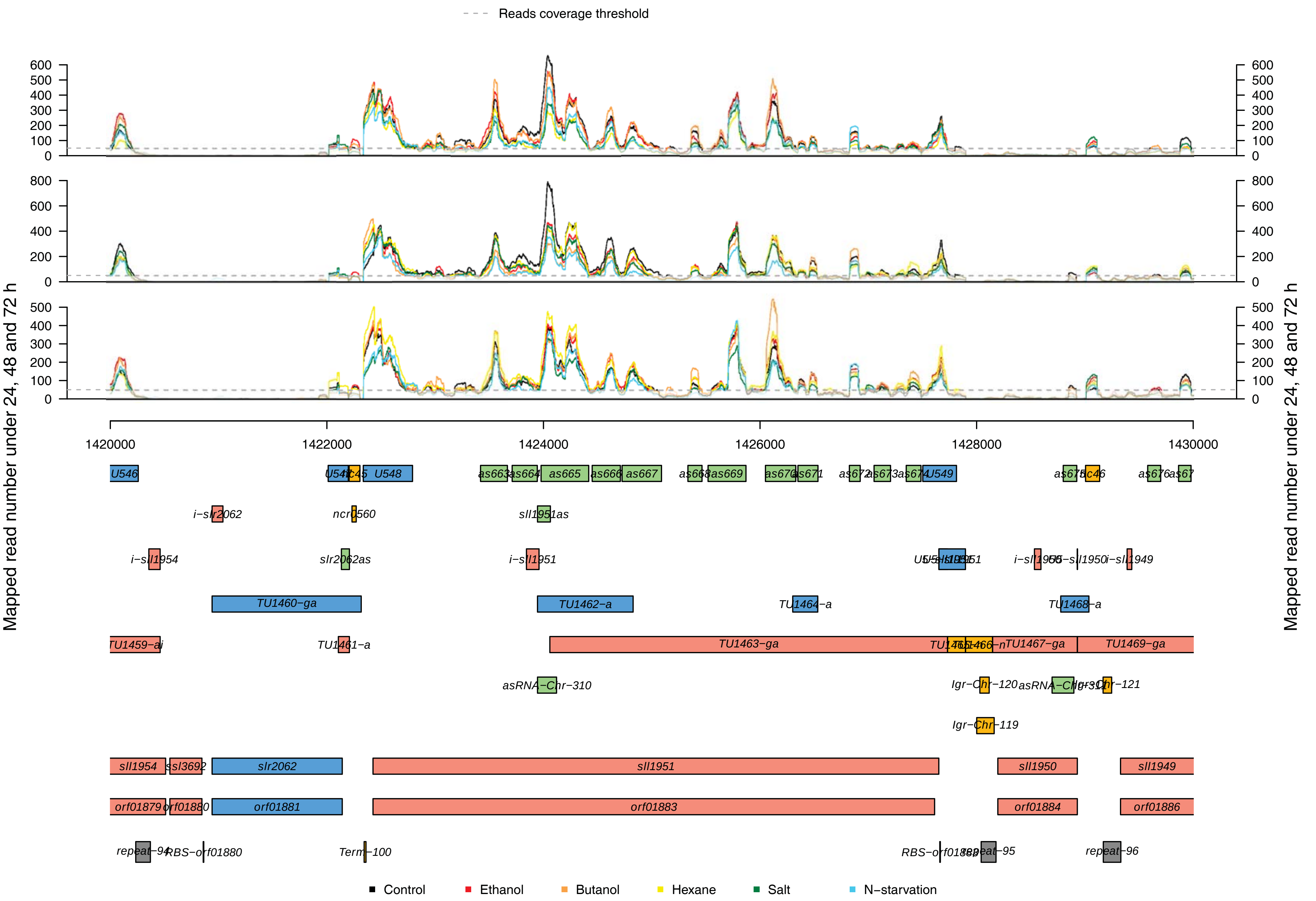

Reads coverage threshold
Mapped read number under 24, 48 and 72 h
1420000
1422000
1424000
1426000
1428000
1430000
Control
Ethanol
Butanol
Hexane
Salt
N−starvation

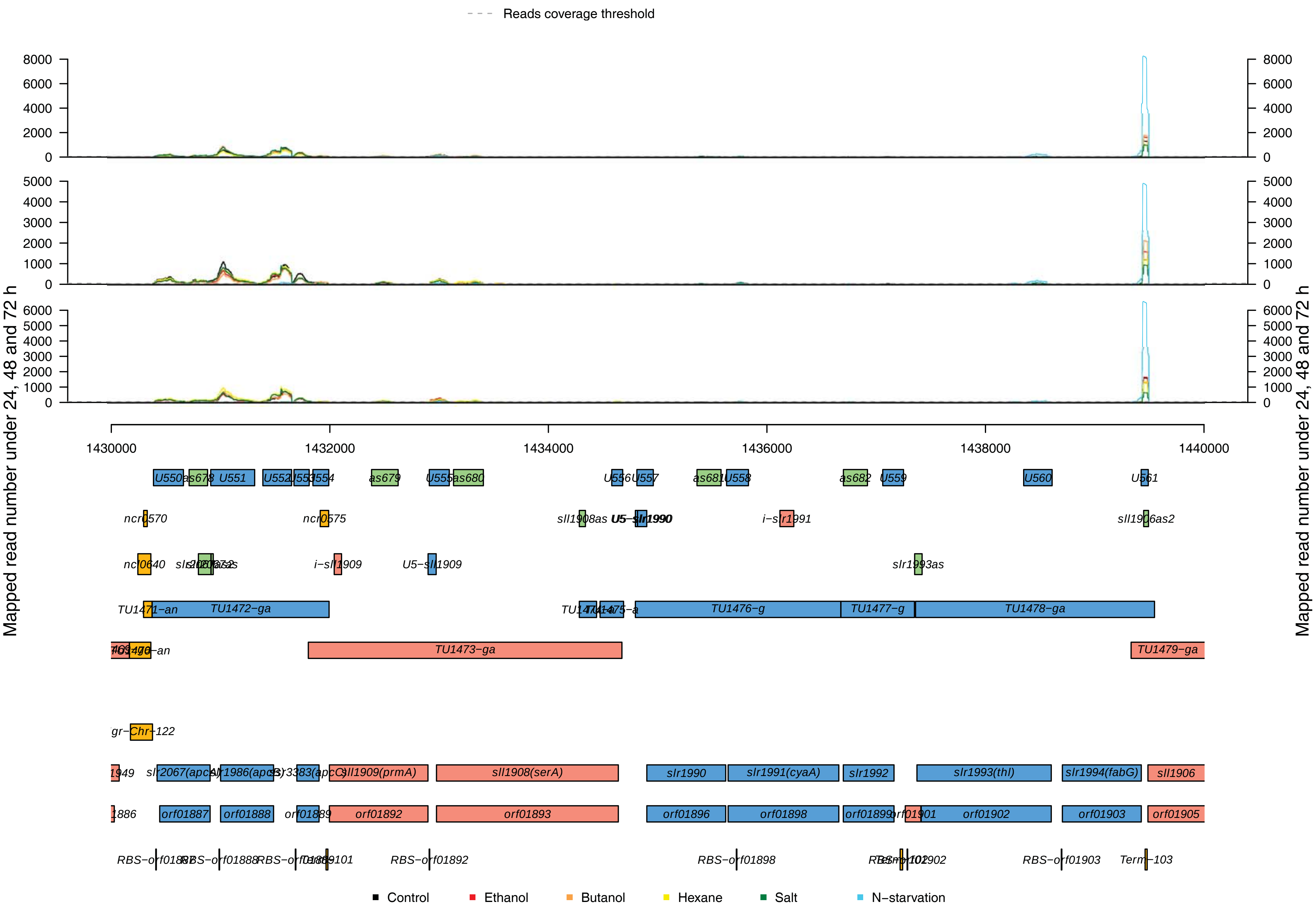

Reads coverage threshold
Mapped read number under 24, 48 and 72 h
8000
6000
4000
2000
0
5000
4000
3000
2000
1000
0
6000
5000
4000
3000
2000
1000
0
1430000
1432000
1434000
1436000
1438000
1440000
U550
U551
U552
U554
as679
as680
U556
U557
U558
as682
U559
U560
ncr0570
ncr0575
sll1908as
i−slr1991
sll1906as2
i−sll1909
U5−sll1909
slr1993as
TU1472−ga
TU1476−g
TU1477−g
TU1478−ga
TU1473−ga
TU1479−ga
sll1909(prmA)
sll1908(serA)
slr1990
slr1991(cyaA)
slr1992
slr1993(thl)
slr1994(fabG)
sll1906
orf01887
orf01888
orf01892
orf01893
orf01896
orf01898
orf01902
orf01903
orf01905
RBS−orf01892
RBS−orf01898
RBS−orf01903
Term−103
Control
Ethanol
Butanol
Hexane
Salt
N−starvation

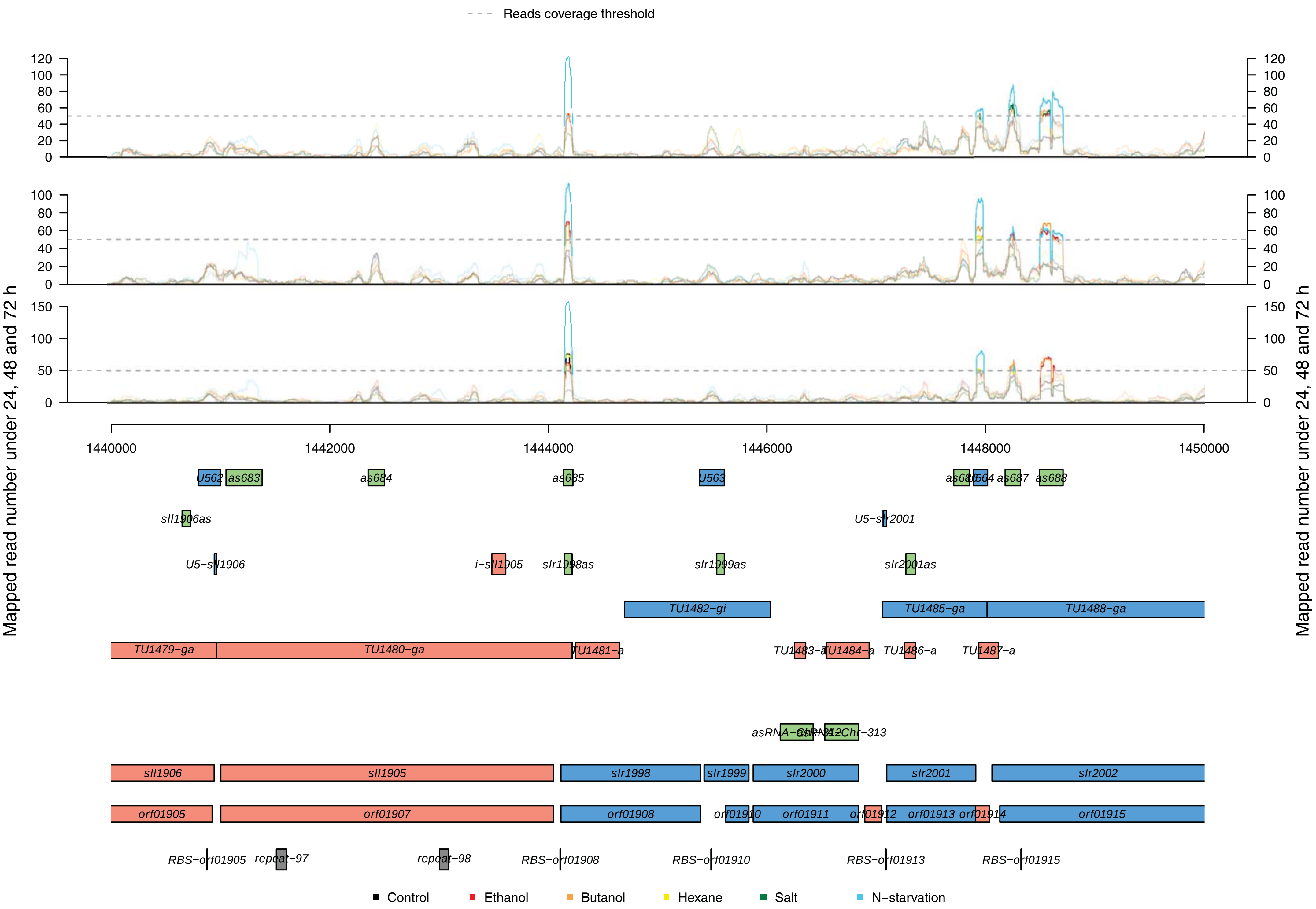

Reads coverage threshold
Mapped read number under 24, 48 and 72 h
1440000
1442000
1444000
1446000
1448000
1450000
U562
as683
as684
as685
U563
as686
U564
as687
as688
sll1906as
U5−slr2001
U5−sll1906
i−sll1905
slr1998as
slr1999as
slr2001as
TU1482−gi
TU1485−ga
TU1488−ga
TU1479−ga
TU1480−ga
TU1481−a
TU1483−a
TU1484−a
TU1486−a
TU1487−a
asRNA−CsRNA12
Chr−313
sll1906
sll1905
slr1998
slr1999
slr2000
slr2001
slr2002
orf01905
orf01907
orf01908
orf01910
orf01911
orf01912
orf01913
orf01914
orf01915
RBS−orf01905
repeat−97
repeat−98
RBS−orf01908
RBS−orf01910
RBS−orf01913
RBS−orf01915
Control
Ethanol
Butanol
Hexane
Salt
N−starvation

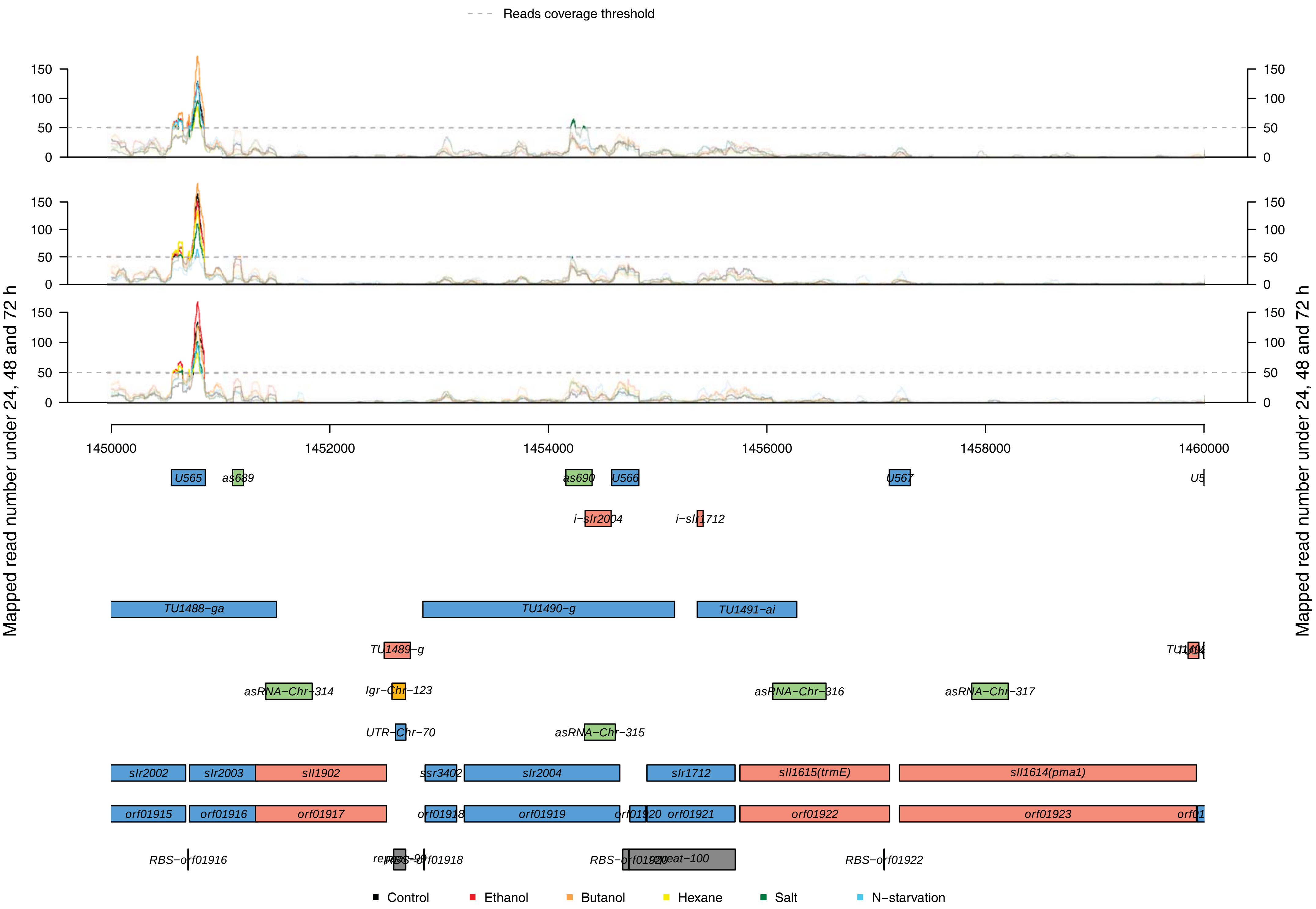

Reads coverage threshold
Mapped read number under 24, 48 and 72 h
150
100
50
0
1450000
1452000
1454000
1456000
1458000
1460000
U565
as689
as690
U566
U567
i−slr2004
i−sll1712
TU1488−ga
TU1490−g
TU1491−ai
TU1489−g
asRNA−Chr−314
Igr−Chr−123
asRNA−Chr−316
asRNA−Chr−317
UTR−Chr−70
asRNA−Chr−315
slr2002
slr2003
sll1902
ssr3402
slr2004
slr1712
sll1615(trmE)
sll1614(pma1)
orf01915
orf01916
orf01917
orf01918
orf01919
orf01920
orf01921
orf01922
orf01923
RBS−orf01916
RBS−orf01918
RBS−orf01920
repeat−100
RBS−orf01922
Control
Ethanol
Butanol
Hexane
Salt
N−starvation

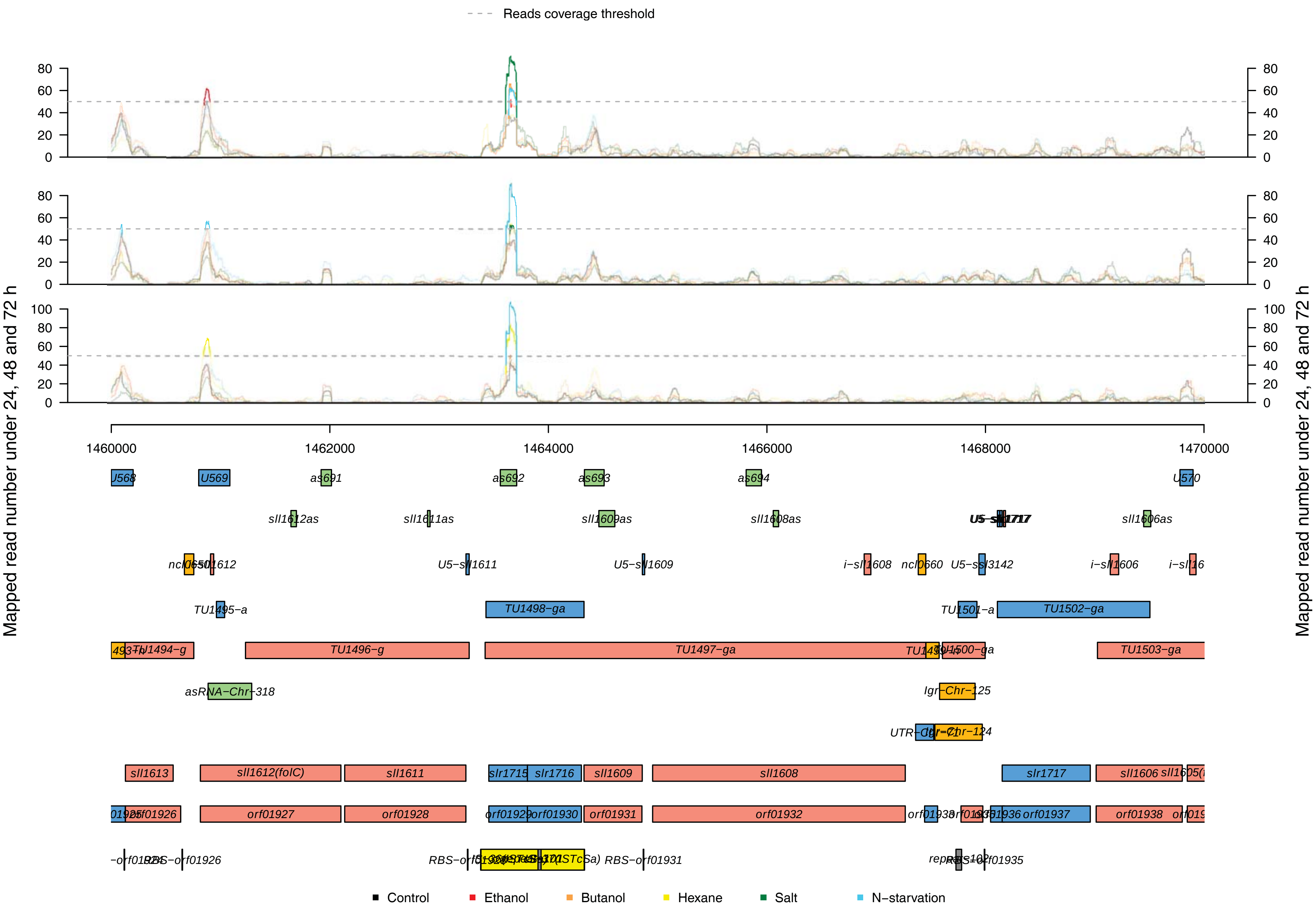

Reads coverage threshold
Mapped read number under 24, 48 and 72 h
1460000
1462000
1464000
1466000
1468000
1470000
U569
as691
as692
as693
as694
sll1612as
sll1611as
sll1609as
sll1608as
sll1606as
U5−sll1611
U5−sll1609
i−sll1608
ncl0660
U5−ssl3142
i−sll1606
TU1495−a
TU1498−ga
TU1502−ga
TU1494−g
TU1496−g
TU1497−ga
TU1503−ga
asRNA−Chr−318
Igr−Chr−125
sll1613
sll1612(folC)
sll1611
slr1715
slr1716
sll1609
sll1608
slr1717
orf01926
orf01927
orf01928
orf01930
orf01931
orf01932
orf01937
orf01938
RBS−orf01926
RBS−orf01931
Control
Ethanol
Butanol
Hexane
Salt
N−starvation

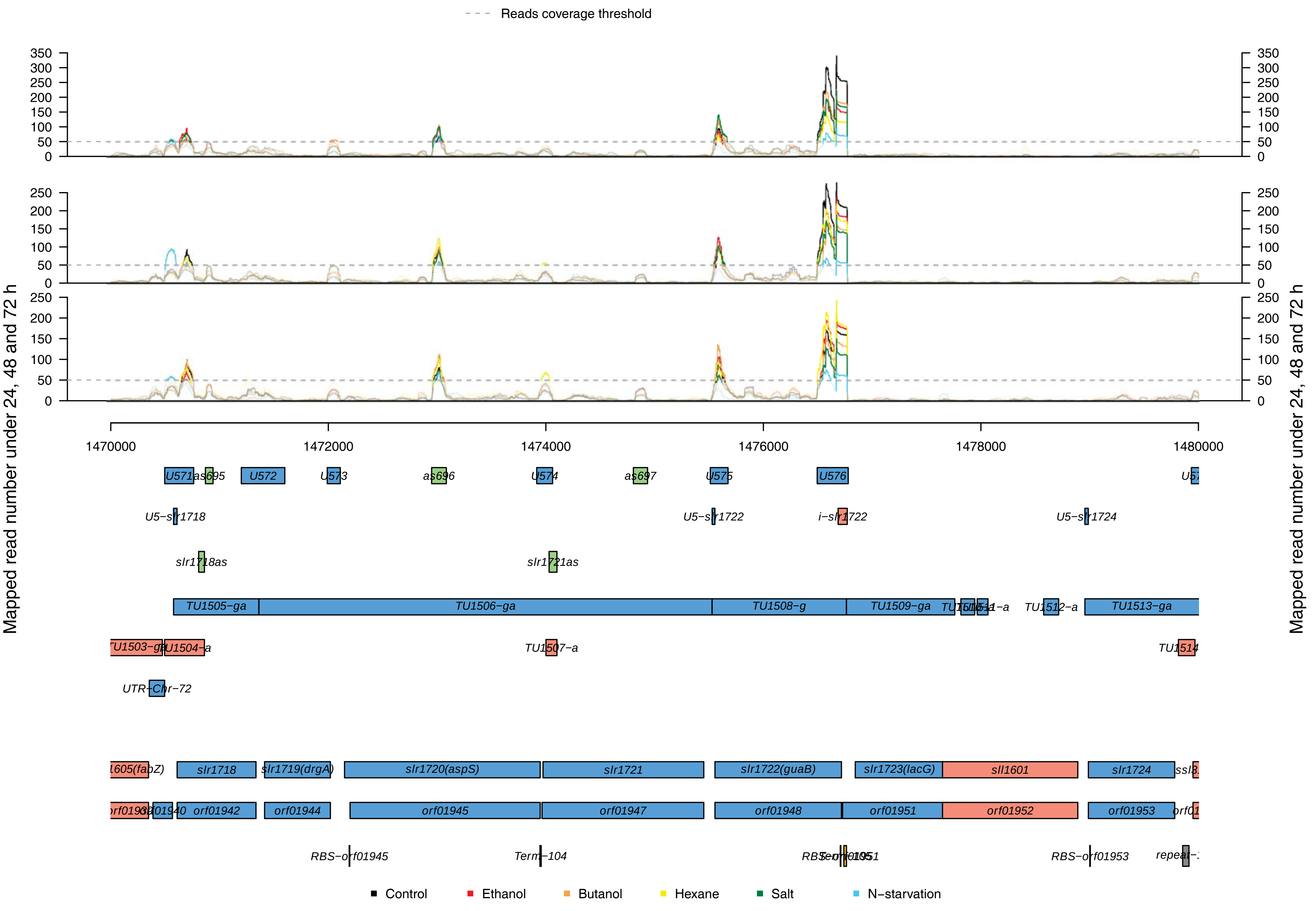

Reads coverage threshold
Mapped read number under 24, 48 and 72 h
1470000
1472000
1474000
1476000
1478000
1480000
U571
as695
U572
U573
as696
U574
as697
U575
U576
U5−slr1718
U5−slr1722
i−slr1722
U5−slr1724
slr1718as
slr1721as
TU1505−ga
TU1506−ga
TU1508−g
TU1509−ga
TU1512−a
TU1513−ga
TU1503−ga
TU1504−a
TU1507−a
UTR−Chr−72
slr1718
slr1719(drgA)
slr1720(aspS)
slr1721
slr1722(guaB)
slr1723(lacG)
sll1601
slr1724
orf01942
orf01944
orf01945
orf01947
orf01948
orf01951
orf01952
orf01953
RBS−orf01945
Term−104
RBS−orf01953
Control
Ethanol
Butanol
Hexane
Salt
N−starvation

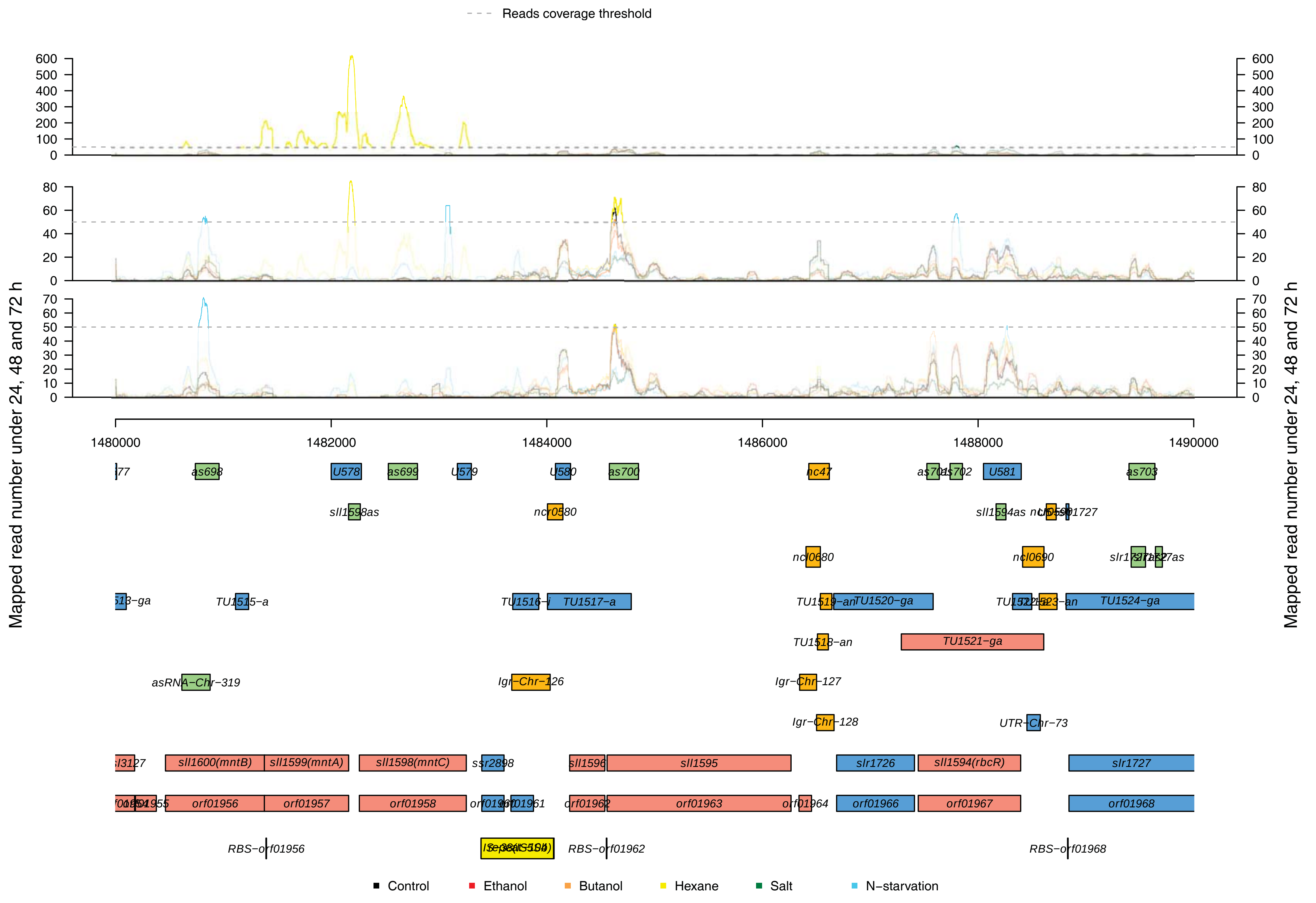

Reads coverage threshold
Mapped read number under 24, 48 and 72 h
1480000
1482000
1484000
1486000
1488000
1490000
as698
U578
as699
U579
U580
as700
nc47
U581
as703
sll1598as
ncr0580
sll1594as
ncl0680
ncl0690
TU1515−a
TU1517−a
TU1520−ga
TU1524−ga
TU1518−an
TU1521−ga
asRNA−Chr−319
Igr−Chr−126
Igr−Chr−127
Igr−Chr−128
UTR−Chr−73
sll1600(mntB)
sll1599(mntA)
sll1598(mntC)
ssr2898
sll1596
sll1595
slr1726
sll1594(rbcR)
slr1727
orf01956
orf01957
orf01958
orf01961
orf01962
orf01963
orf01964
orf01966
orf01967
orf01968
RBS−orf01956
RBS−orf01962
RBS−orf01968
Control
Ethanol
Butanol
Hexane
Salt
N−starvation

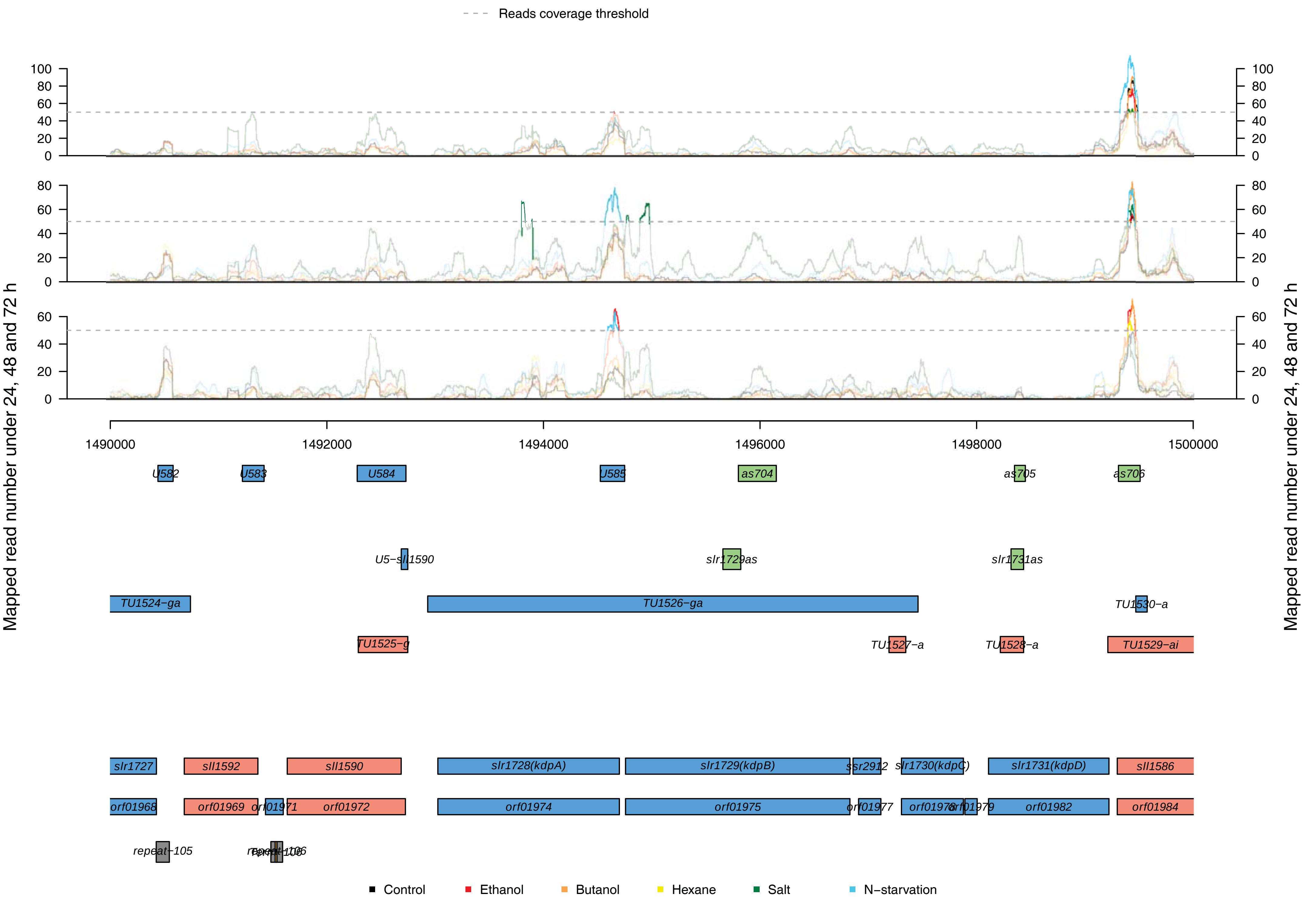

Reads coverage threshold
Mapped read number under 24, 48 and 72 h
1490000
1492000
1494000
1496000
1498000
1500000
U582
U583
U584
U585
as704
as705
as706
U5−sll1590
slr1729as
slr1731as
TU1524−ga
TU1526−ga
TU1530−a
TU1525−g
TU1527−a
TU1528−a
TU1529−ai
slr1727
sll1592
sll1590
slr1728(kdpA)
slr1729(kdpB)
ssr2912
slr1730(kdpC)
slr1731(kdpD)
sll1586
orf01968
orf01969
orf01971
orf01972
orf01974
orf01975
orf01977
orf01978
orf01979
orf01982
orf01984
repeat−105
Control
Ethanol
Butanol
Hexane
Salt
N−starvation

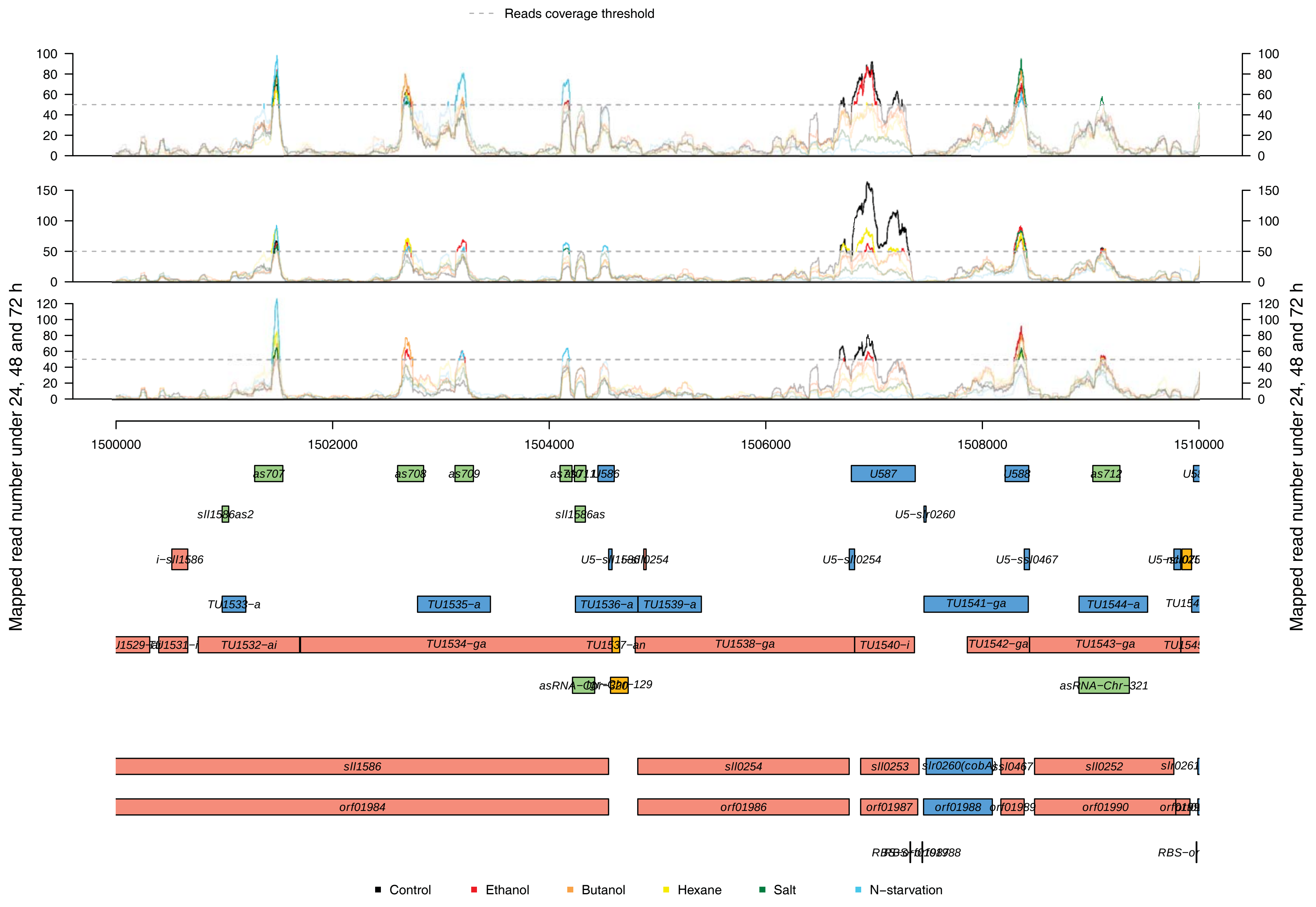

Reads coverage threshold
Mapped read number under 24, 48 and 72 h
1500000
1502000
1504000
1506000
1508000
1510000
as707
as708
as709
U586
U587
U588
as712
sll1586as2
sll1586as
U5−slr0260
i−sll1586
U5−sll0254
U5−sll0467
TU1533−a
TU1535−a
TU1536−a
TU1539−a
TU1541−ga
TU1544−a
TU1532−ai
TU1534−ga
TU1538−ga
TU1540−i
TU1542−ga
TU1543−ga
asRNA−Chr−321
sll1586
sll0254
sll0253
slr0260(cobA)
ssl0467
sll0252
orf01984
orf01986
orf01987
orf01988
orf01990
Control
Ethanol
Butanol
Hexane
Salt
N−starvation

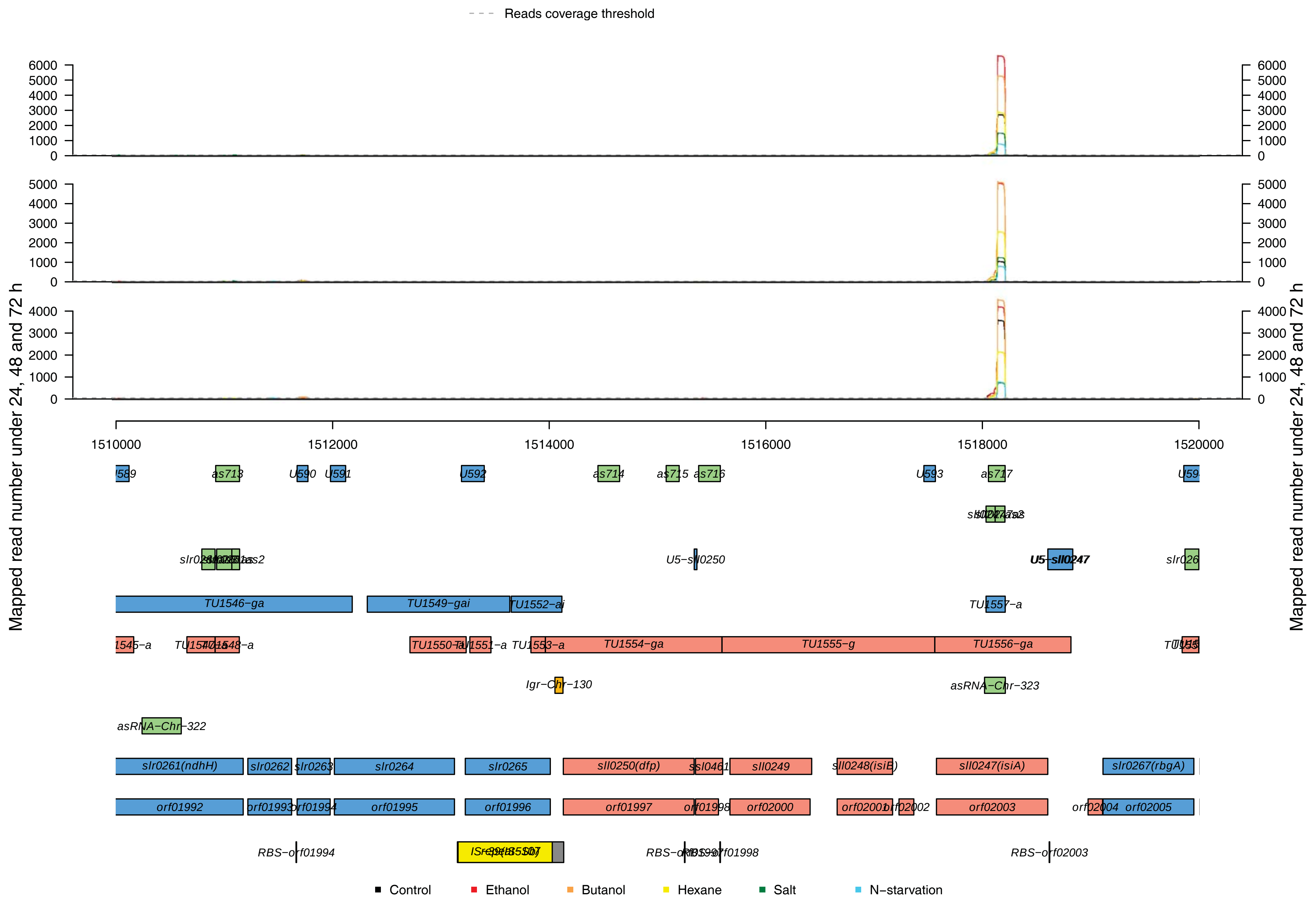
Reads coverage threshold
Mapped read number under 24, 48 and 72 h
1510000
1512000
1514000
1516000
1518000
1520000
TU1546-ga
TU1549-gai
TU1554-ga
TU1555-g
TU1556-ga
asRNA-Chr-322
asRNA-Chr-323
slr0261(ndhH)
slr0262
slr0264
slr0265
sll0250(dfp)
sll0249
sll0247(isiA)
slr0267(rbgA)
orf01992
orf01995
orf01996
orf01997
orf02000
orf02003
orf02005
RBS-orf01994
RBS-orf02003
Control
Ethanol
Butanol
Hexane
Salt
N-starvation

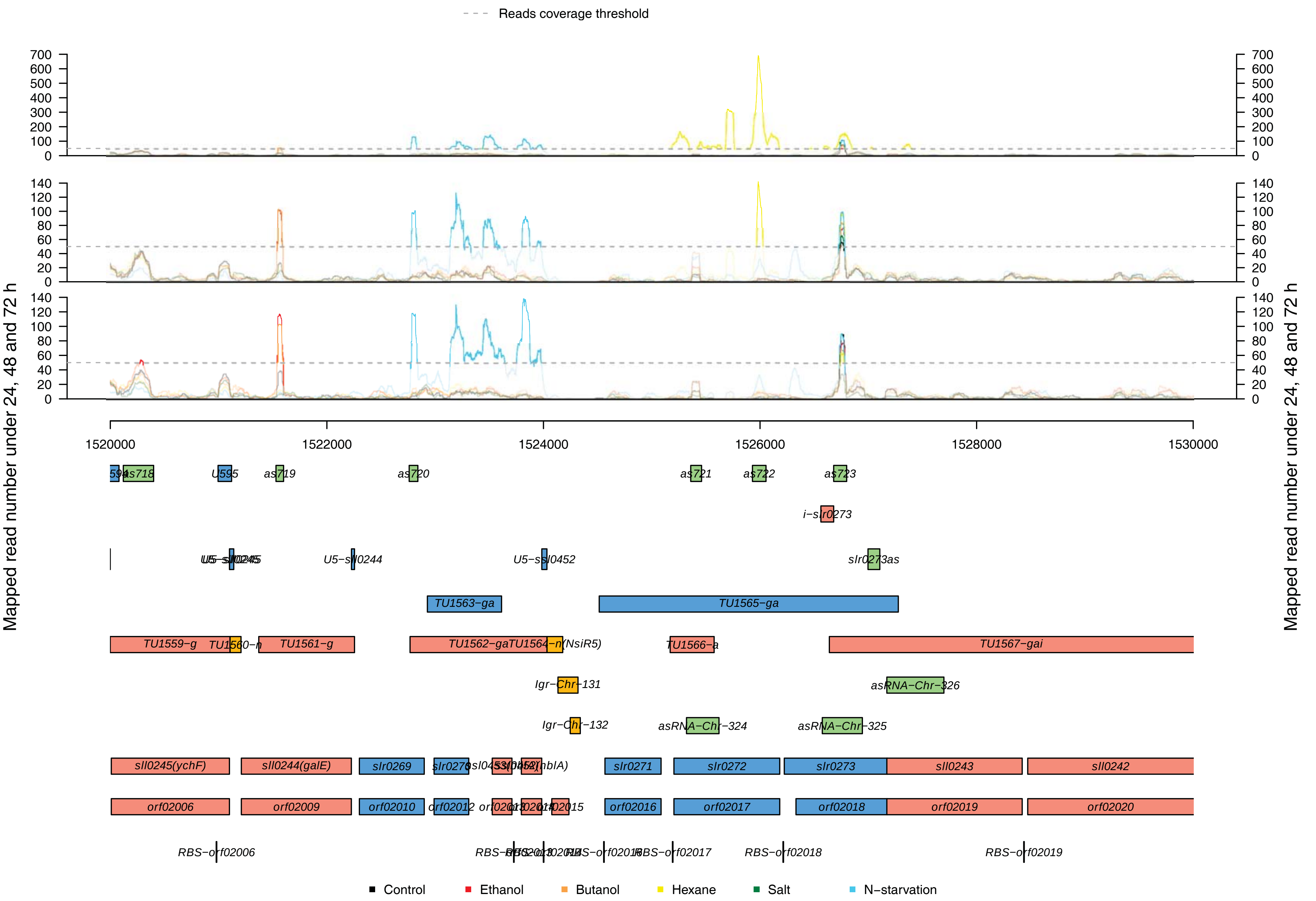

Reads coverage threshold
Mapped read number under 24, 48 and 72 h
1520000
1522000
1524000
1526000
1528000
1530000
as718
U595
as719
as720
as721
as722
as723
i−slr0273
U5−sll0244
U5−sll0452
slr0273as
TU1563−ga
TU1565−ga
TU1559−g
TU1561−g
TU1566−a
TU1567−gai
Igr−Chr−131
asRNA−Chr−326
Igr−Chr−132
asRNA−Chr−324
asRNA−Chr−325
sll0245(ychF)
sll0244(galE)
slr0269
slr0271
slr0272
slr0273
sll0243
sll0242
orf02006
orf02009
orf02010
orf02012
orf02015
orf02016
orf02017
orf02018
orf02019
orf02020
RBS−orf02006
RBS−orf02017
RBS−orf02018
RBS−orf02019
Control
Ethanol
Butanol
Hexane
Salt
N−starvation

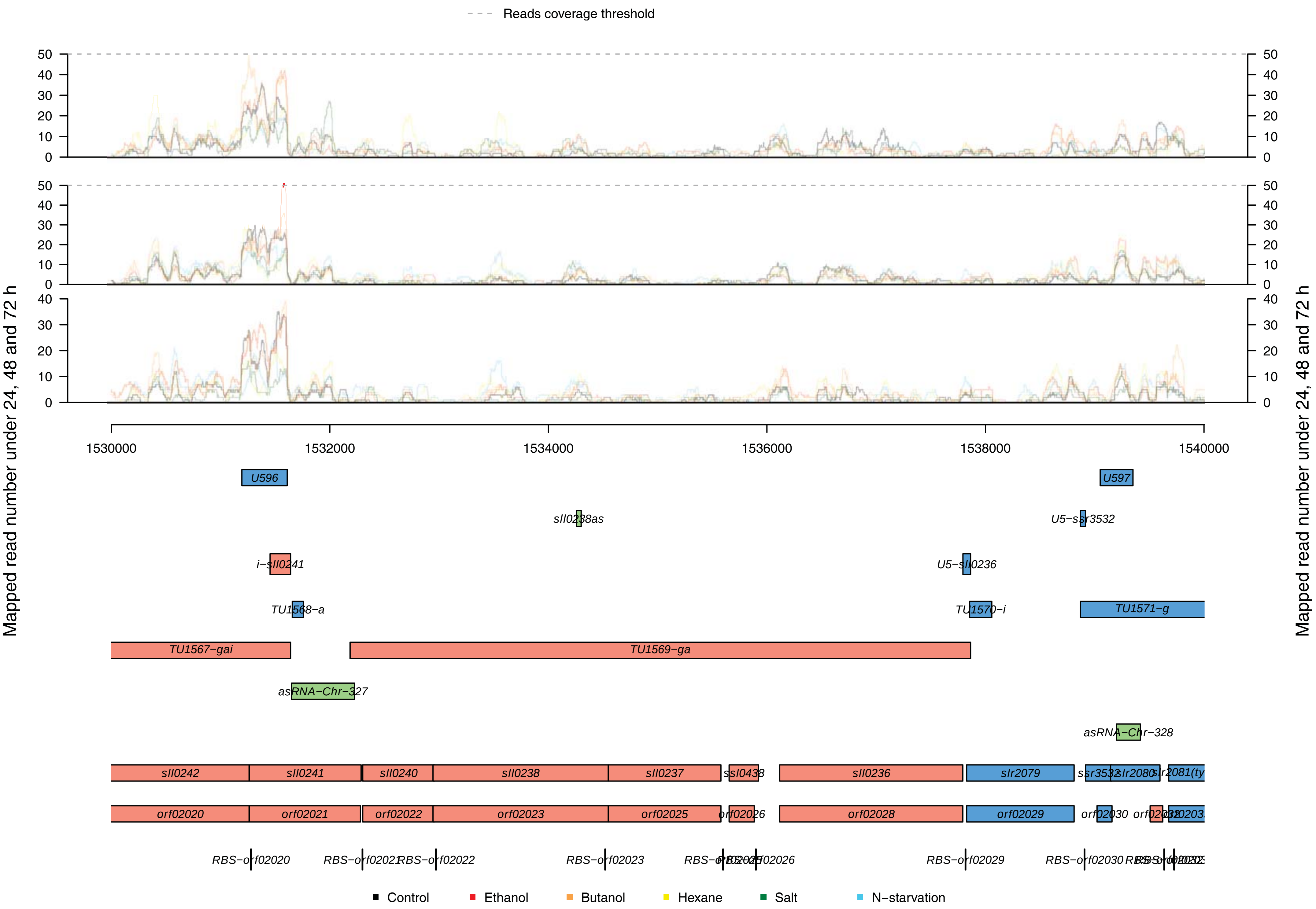

Reads coverage threshold
Mapped read number under 24, 48 and 72 h
1530000
1532000
1534000
1536000
1538000
1540000
U596
U597
sll0238as
U5−ssr3532
i−sll0241
U5−sll0236
TU1568−a
TU1570−i
TU1571−g
TU1567−gai
TU1569−ga
asRNA−Chr−327
asRNA−Chr−328
sll0242
sll0241
sll0240
sll0238
sll0237
ssl0438
sll0236
slr2079
orf02020
orf02021
orf02022
orf02023
orf02025
orf02026
orf02028
orf02029
orf02030
RBS−orf02020
RBS−orf02021
RBS−orf02022
RBS−orf02023
RBS−orf02029
RBS−orf02030
Control
Ethanol
Butanol
Hexane
Salt
N−starvation

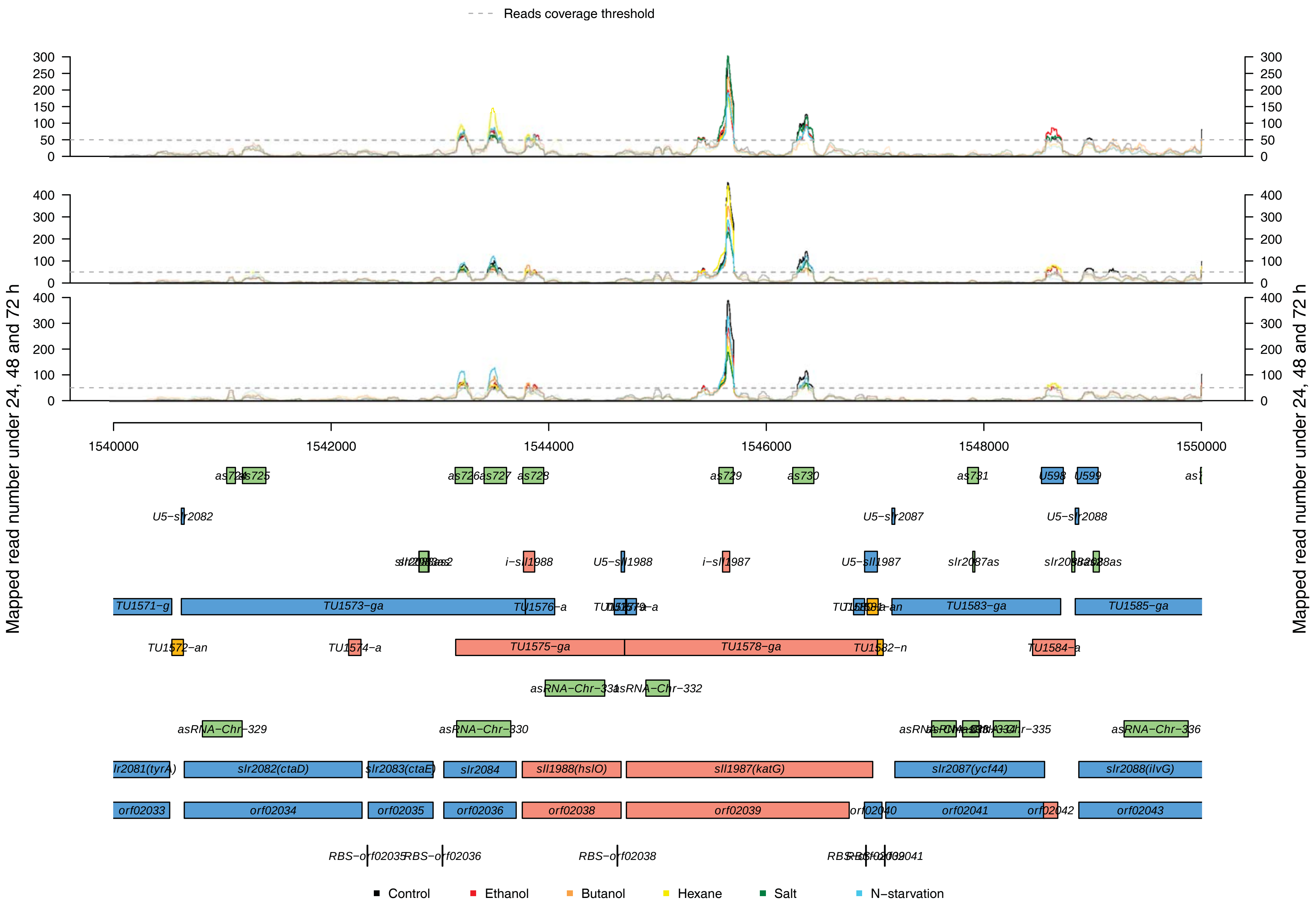

Reads coverage threshold
Mapped read number under 24, 48 and 72 h
1540000
1542000
1544000
1546000
1548000
1550000
TU1571−g
TU1573−ga
TU1576−a
TU1583−ga
TU1585−ga
TU1572−an
TU1574−a
TU1575−ga
TU1578−ga
TU1584−a
asRNA−Chr−329
asRNA−Chr−330
asRNA−Chr−336
asRNA−Chr−332
slr2082(ctaD)
slr2084
sll1988(hslO)
sll1987(katG)
slr2087(ycf44)
slr2088(ilvG)
orf02033
orf02034
orf02035
orf02036
orf02038
orf02039
orf02041
orf02043
U5−slr2082
U5−slr2087
U5−slr2088
U5−sll1988
U5−sll1987
i−sll1988
i−sll1987
slr2087as
RBS−orf02036
RBS−orf02038
Control
Ethanol
Butanol
Hexane
Salt
N−starvation

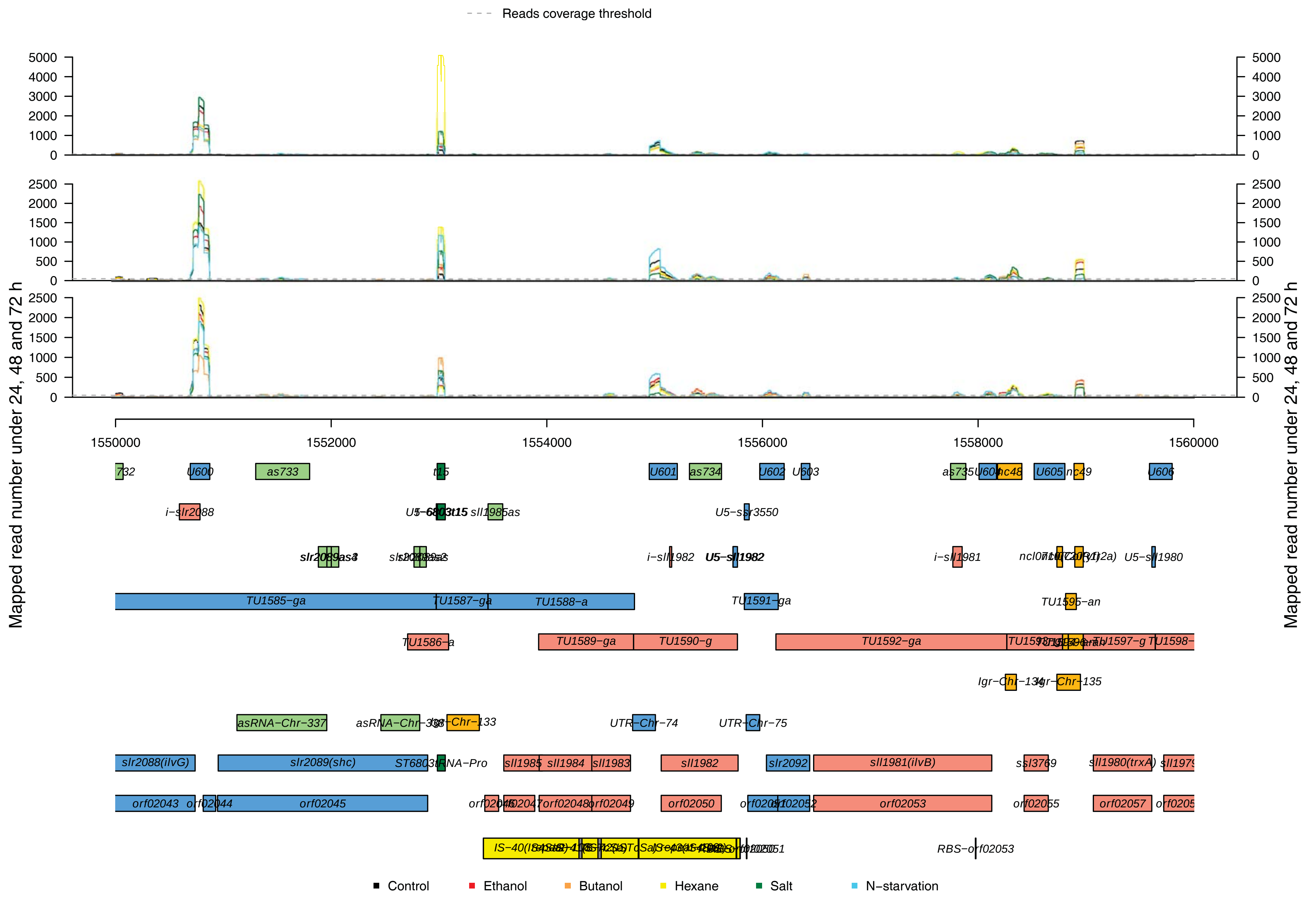

Reads coverage threshold
Mapped read number under 24, 48 and 72 h
1550000
1552000
1554000
1556000
1558000
1560000
U600
as733
U601
as734
U602
U603
U605
U606
i−slr2088
U5−ssr3550
i−sll1982
U5−sll1982
i−sll1981
U5−sll1980
TU1585−ga
TU1588−a
TU1586−a
TU1589−ga
TU1590−g
TU1592−ga
Igr−Chr−135
asRNA−Chr−337
UTR−Chr−74
UTR−Chr−75
slr2088(ilvG)
slr2089(shc)
sll1982
slr2092
sll1981(ilvB)
ssl3769
sll1980(trxA)
orf02043
orf02045
orf02050
orf02053
orf02057
RBS−orf02053
Control
Ethanol
Butanol
Hexane
Salt
N−starvation

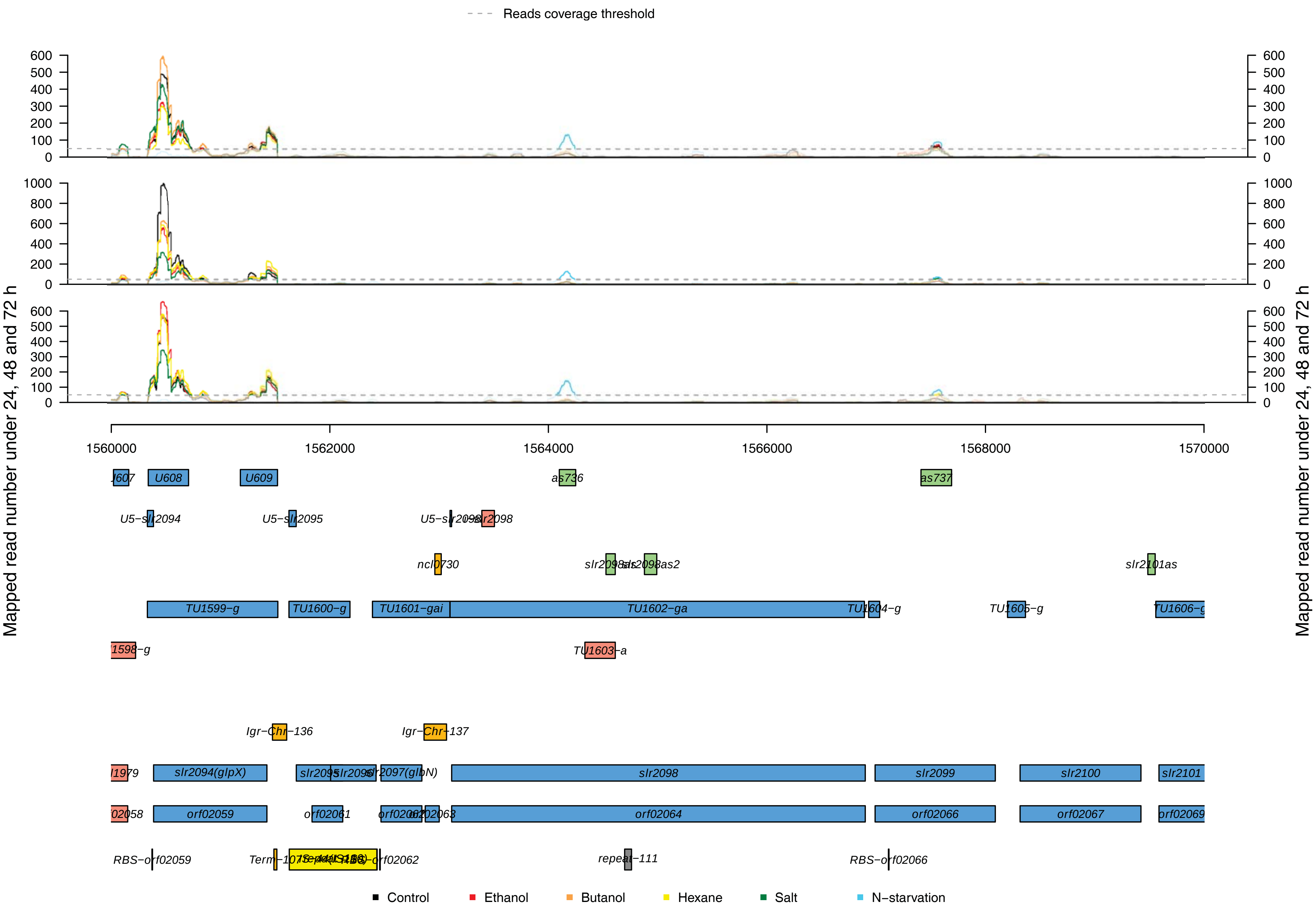

Reads coverage threshold
Mapped read number under 24, 48 and 72 h
1560000
1562000
1564000
1566000
1568000
1570000
U608
U609
as736
as737
U5−slr2094
U5−slr2095
ncl0730
slr2101as
TU1599−g
TU1600−g
TU1601−gai
TU1602−ga
TU1605−g
TU1603−a
Igr−Chr−136
Igr−Chr−137
slr2094(glpX)
slr2098
slr2099
slr2100
slr2101
orf02059
orf02061
orf02064
orf02066
orf02067
orf02069
RBS−orf02059
repeat−111
RBS−orf02066
Control
Ethanol
Butanol
Hexane
Salt
N−starvation

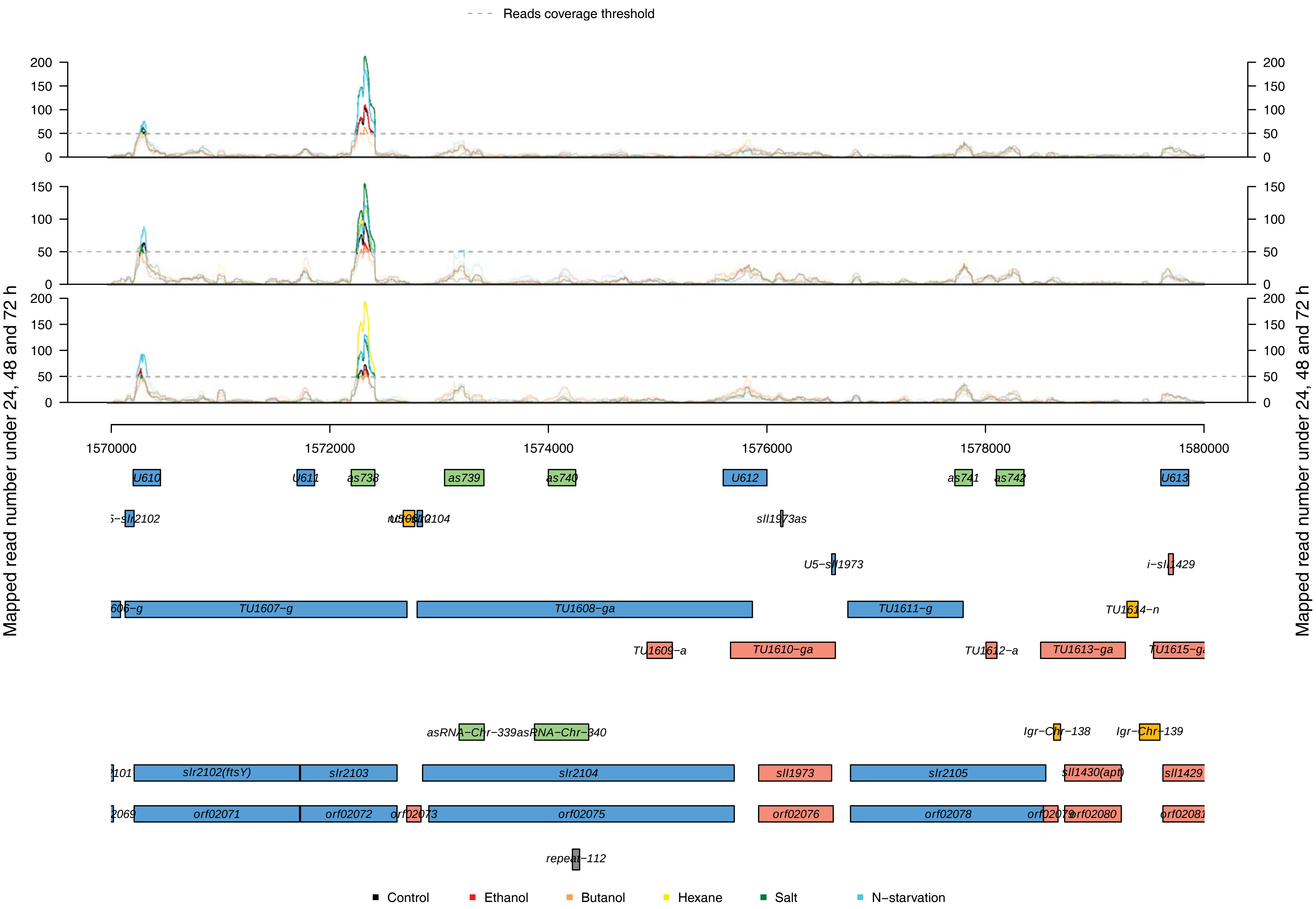

Reads coverage threshold
Mapped read number under 24, 48 and 72 h
1570000
1572000
1574000
1576000
1578000
1580000
U610
U611
as738
as739
as740
U612
as741
as742
U613
sll1973as
U5−sll1973
i−sll1429
TU1607−g
TU1608−ga
TU1611−g
TU1614−n
TU1609−a
TU1610−ga
TU1612−a
TU1613−ga
Igr−Chr−138
Igr−Chr−139
slr2102(ftsY)
slr2103
slr2104
sll1973
slr2105
sll1430(apt)
sll1429
orf02071
orf02072
orf02075
orf02076
orf02078
orf02080
repeat−112
Control
Ethanol
Butanol
Hexane
Salt
N−starvation

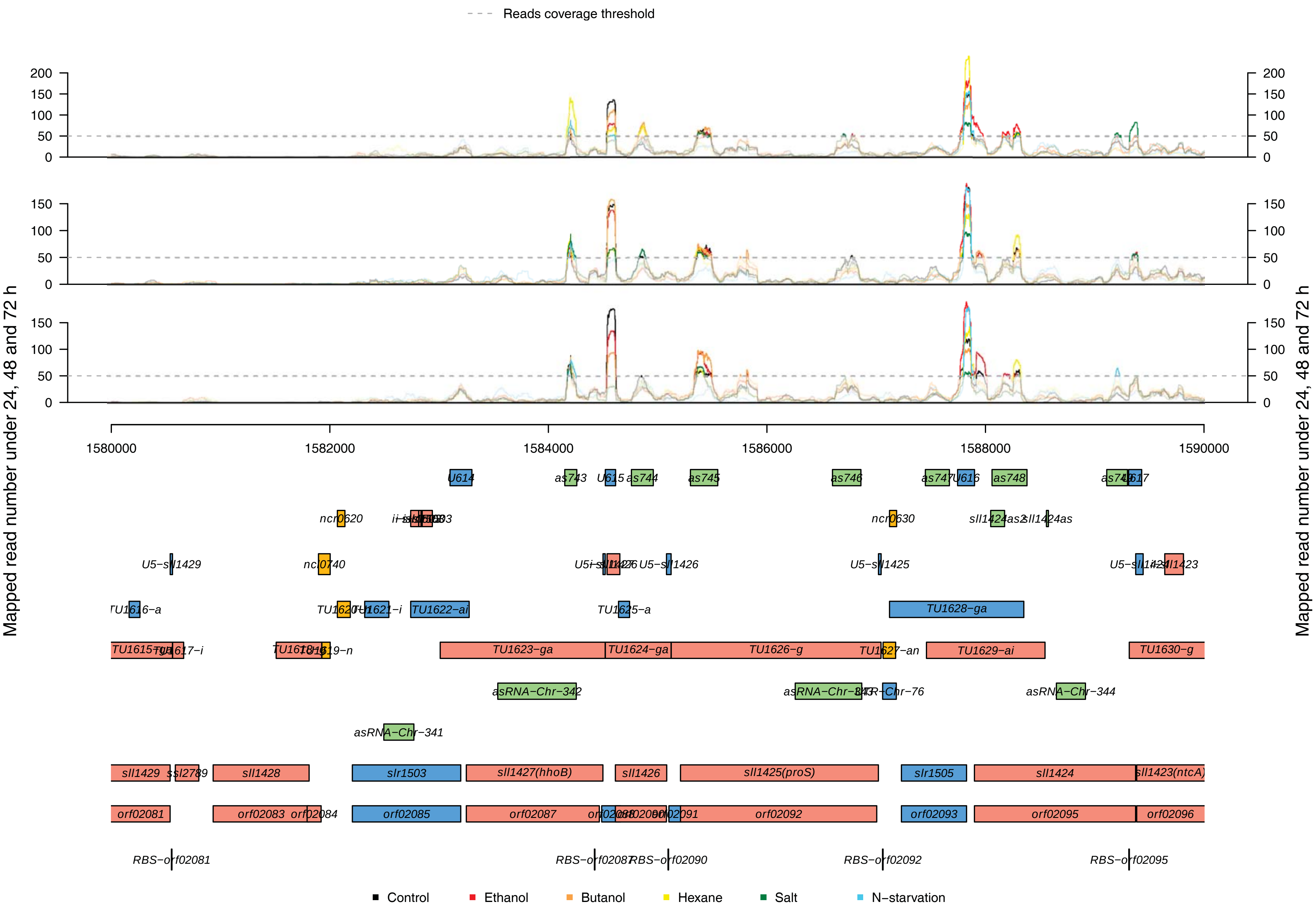

Reads coverage threshold
Mapped read number under 24, 48 and 72 h
200
150
100
50
0
1580000
1582000
1584000
1586000
1588000
1590000
U614
as743
as744
as745
as746
as748
ncr0620
ncr0630
U5−sll1429
U5−sll1426
U5−sll1425
TU1616−a
TU1622−ai
TU1625−a
TU1628−ga
TU1623−ga
TU1624−ga
TU1626−g
TU1629−ai
TU1630−g
asRNA−Chr−342
asRNA−Chr−344
asRNA−Chr−341
sll1429
ssl2789
sll1428
slr1503
sll1427(hhoB)
sll1426
sll1425(proS)
slr1505
sll1424
sll1423(ntcA)
orf02081
orf02083
orf02085
orf02087
orf02092
orf02093
orf02095
orf02096
RBS−orf02081
RBS−orf02092
RBS−orf02095
Control
Ethanol
Butanol
Hexane
Salt
N−starvation

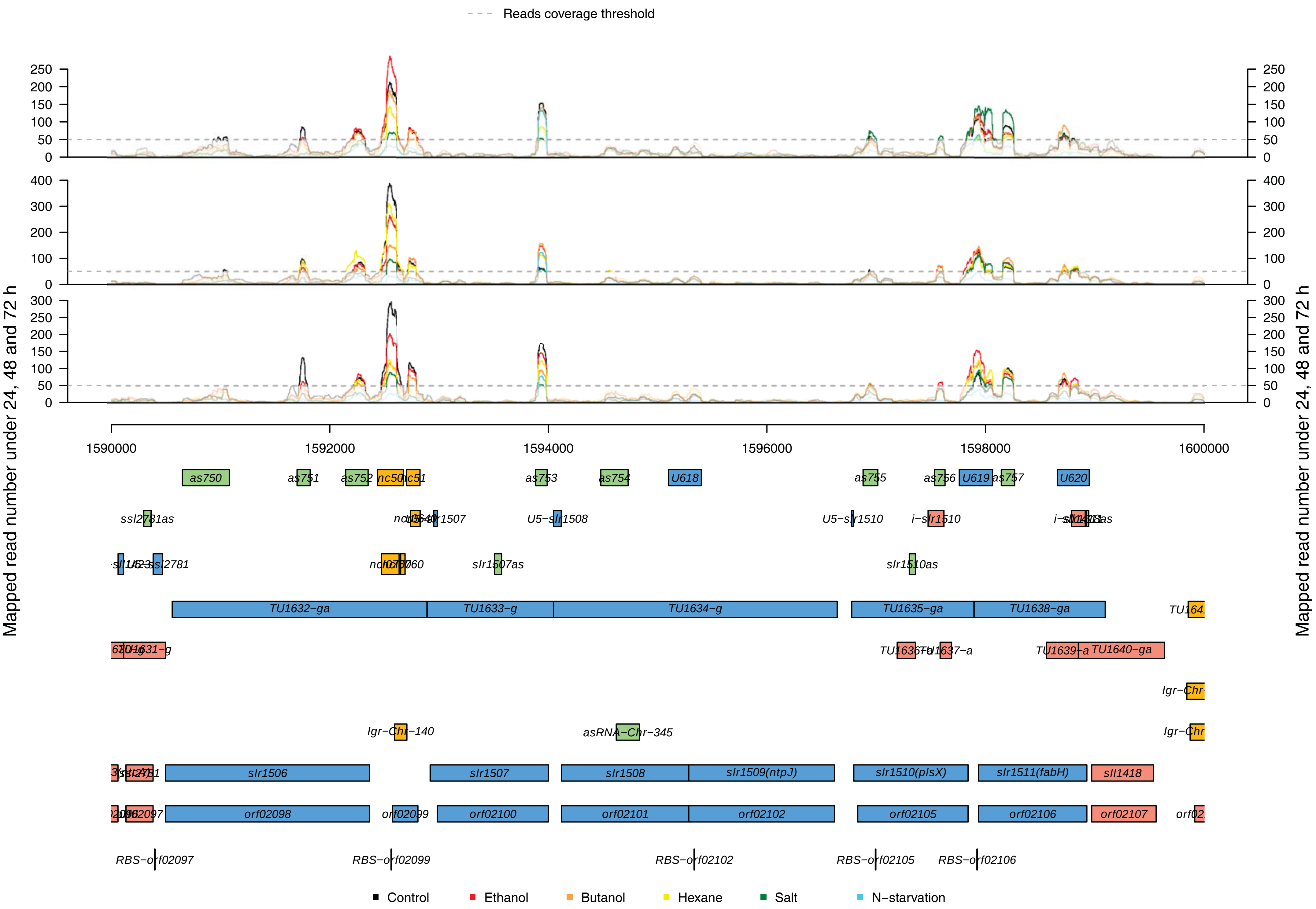

Reads coverage threshold
Mapped read number under 24, 48 and 72 h
1590000
1592000
1594000
1596000
1598000
1600000
as750
as751
as752
nc50
nc51
as753
as754
U618
as755
as756
U619
as757
U620
ssl2781as
U5−slr1510
i−slr1510
slr1507as
slr1510as
TU1632−ga
TU1633−g
TU1634−g
TU1635−ga
TU1638−ga
TU1640−ga
Igr−Chr−140
asRNA−Chr−345
slr1506
slr1507
slr1508
slr1509(ntpJ)
slr1510(plsX)
slr1511(fabH)
sll1418
orf02098
orf02100
orf02101
orf02102
orf02105
orf02106
orf02107
RBS−orf02097
RBS−orf02099
RBS−orf02102
RBS−orf02105
RBS−orf02106
Control
Ethanol
Butanol
Hexane
Salt
N−starvation

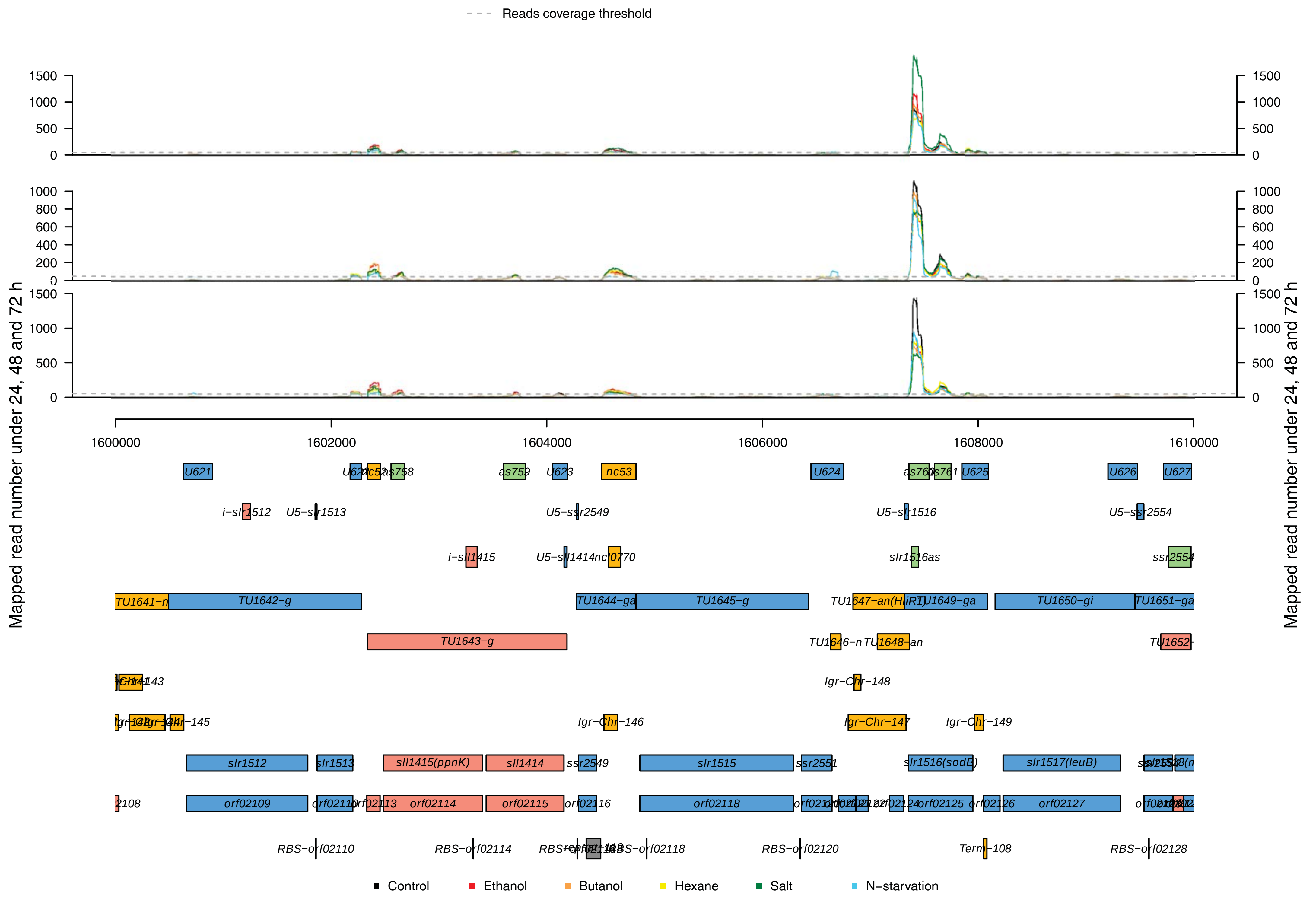
Reads coverage threshold
Mapped read number under 24, 48 and 72 h
1600000
1602000
1604000
1606000
1608000
1610000
U621
nc53
U623
U624
U625
U626
U627
as759
TU1642−g
TU1643−g
TU1645−g
TU1649−ga
TU1650−gi
TU1651−ga
slr1512
slr1513
sll1415(ppnK)
sll1414
slr1515
slr1516(sodB)
slr1517(leuB)
orf02109
orf02114
orf02115
orf02118
orf02125
orf02127
RBS−orf02110
RBS−orf02114
RBS−orf02118
RBS−orf02120
RBS−orf02128
Term−108
Igr−Chr−146
Igr−Chr−147
Igr−Chr−148
Igr−Chr−149
Control
Ethanol
Butanol
Hexane
Salt
N−starvation

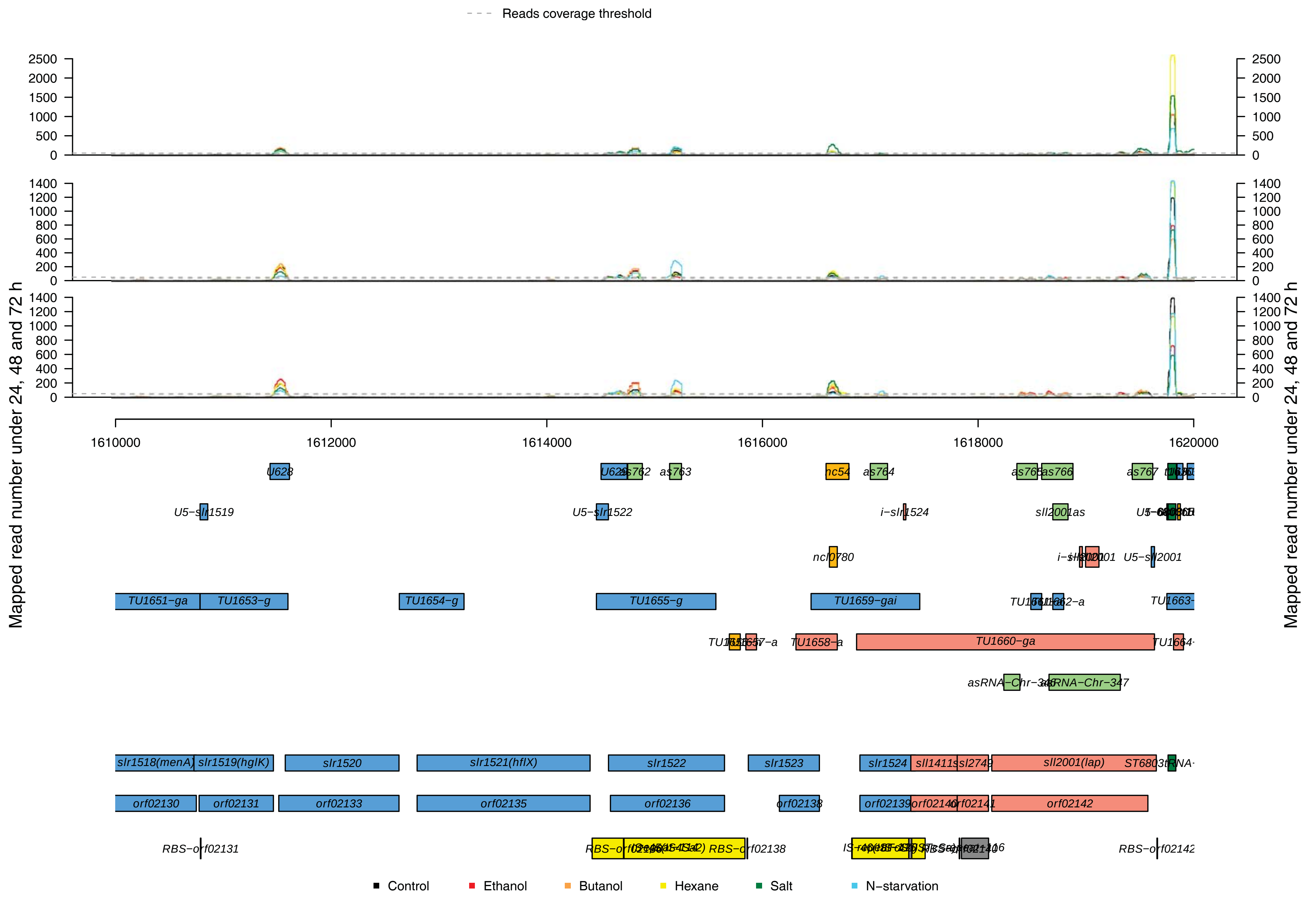

Reads coverage threshold
Mapped read number under 24, 48 and 72 h
1610000
1612000
1614000
1616000
1618000
1620000
U628
as762
as763
nc54
as764
as766
as767
U5−slr1519
U5−slr1522
i−slr1524
sll2001as
ncl0780
U5−sll2001
TU1651−ga
TU1653−g
TU1654−g
TU1655−g
TU1659−gai
TU1658−a
TU1660−ga
asRNA−Chr−347
slr1518(menA)
slr1519(hglK)
slr1520
slr1521(hflX)
slr1522
slr1523
slr1524
sll2001(lap)
orf02130
orf02131
orf02133
orf02135
orf02136
orf02138
orf02139
orf02142
RBS−orf02131
RBS−orf02138
RBS−orf02142
Control
Ethanol
Butanol
Hexane
Salt
N−starvation

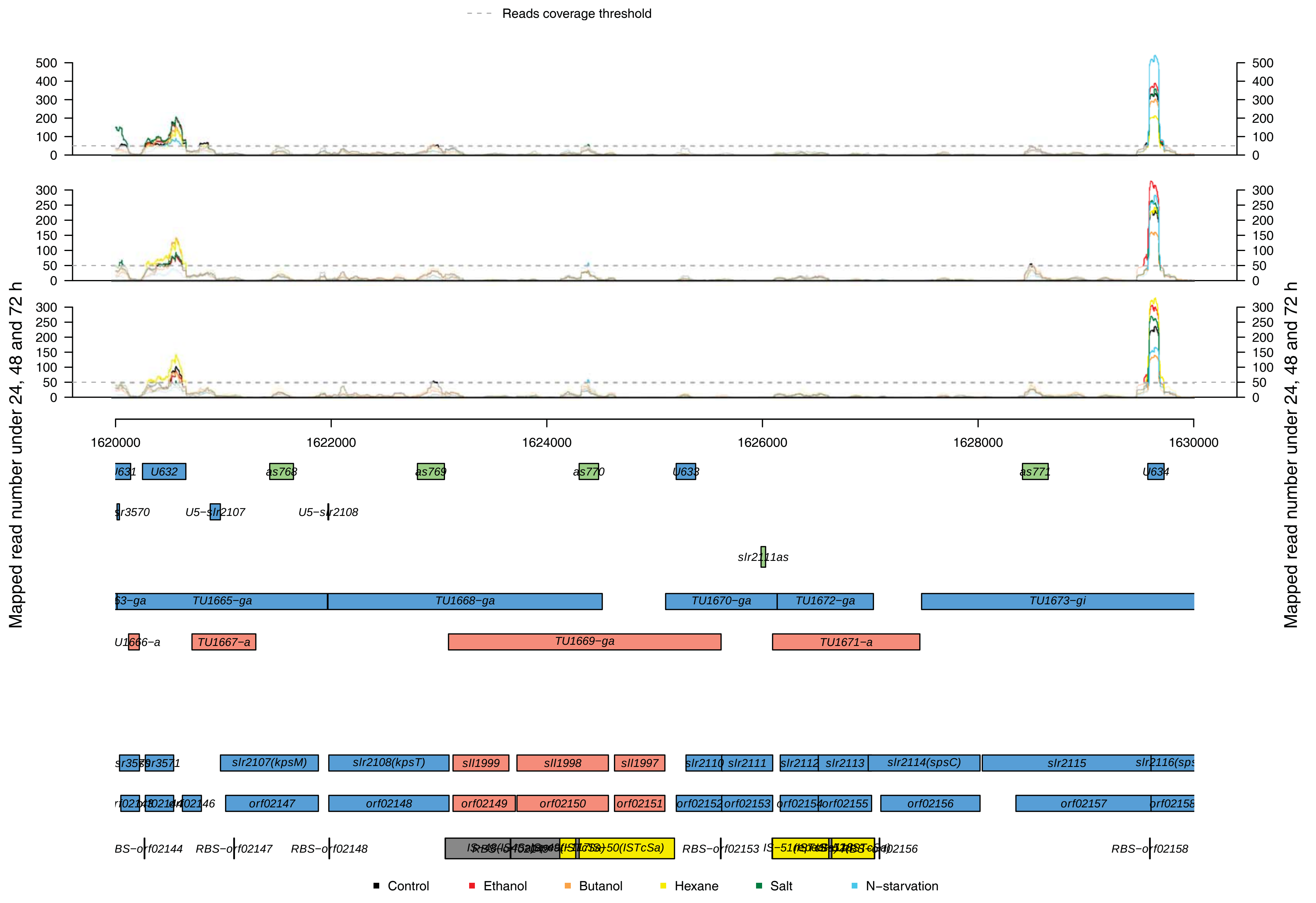

Reads coverage threshold
Mapped read number under 24, 48 and 72 h
1620000
1622000
1624000
1626000
1628000
1630000
U632
as768
as769
as770
U633
as771
U634
slr3570
U5−slr2107
U5−slr2108
slr2111as
TU1665−ga
TU1668−ga
TU1670−ga
TU1672−ga
TU1673−gi
TU1667−a
TU1669−ga
TU1671−a
slr2107(kpsM)
slr2108(kpsT)
sll1999
sll1998
sll1997
slr2110
slr2111
slr2112
slr2113
slr2114(spsC)
slr2115
orf02147
orf02148
orf02149
orf02150
orf02151
orf02152
orf02153
orf02155
orf02156
orf02157
RBS−orf02147
RBS−orf02148
RBS−orf02153
RBS−orf02158
IS−50(ISTcSa)
Control
Ethanol
Butanol
Hexane
Salt
N−starvation

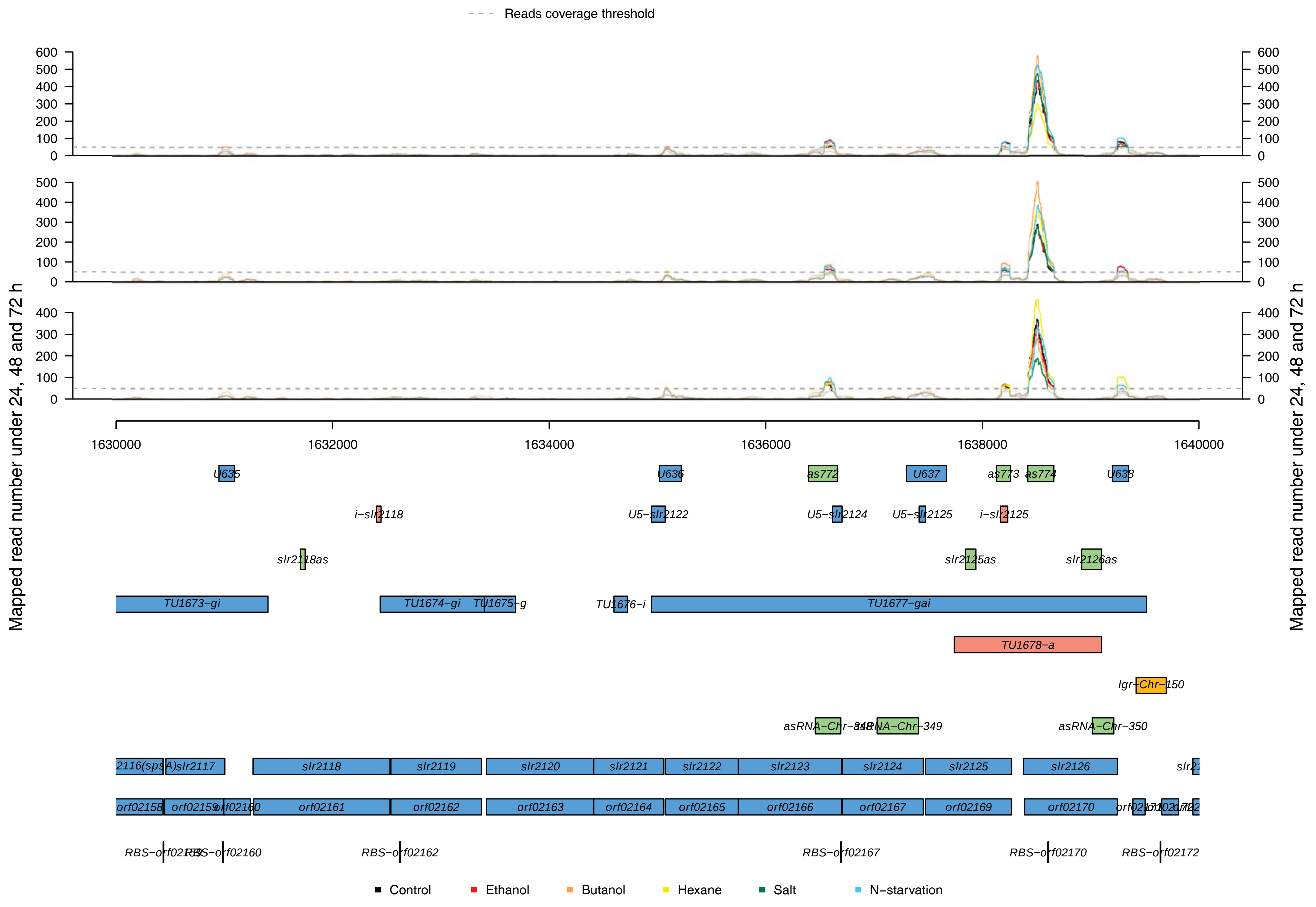

Reads coverage threshold
Mapped read number under 24, 48 and 72 h
1630000
1632000
1634000
1636000
1638000
1640000
U635
U636
as772
U637
as773
as774
U638
i−slr2118
U5−slr2122
U5−slr2124
U5−slr2125
i−slr2125
slr2118as
slr2125as
slr2126as
TU1673−gi
TU1674−gi
TU1675−g
TU1676−i
TU1677−gai
TU1678−a
Igr−Chr−150
asRNA−Chr−348
asRNA−Chr−349
asRNA−Chr−350
slr2117
slr2118
slr2119
slr2120
slr2121
slr2122
slr2123
slr2124
slr2125
slr2126
orf02158
orf02159
orf02160
orf02161
orf02162
orf02163
orf02164
orf02165
orf02166
orf02167
orf02169
orf02170
RBS−orf02160
RBS−orf02162
RBS−orf02167
RBS−orf02170
RBS−orf02172
Control
Ethanol
Butanol
Hexane
Salt
N−starvation

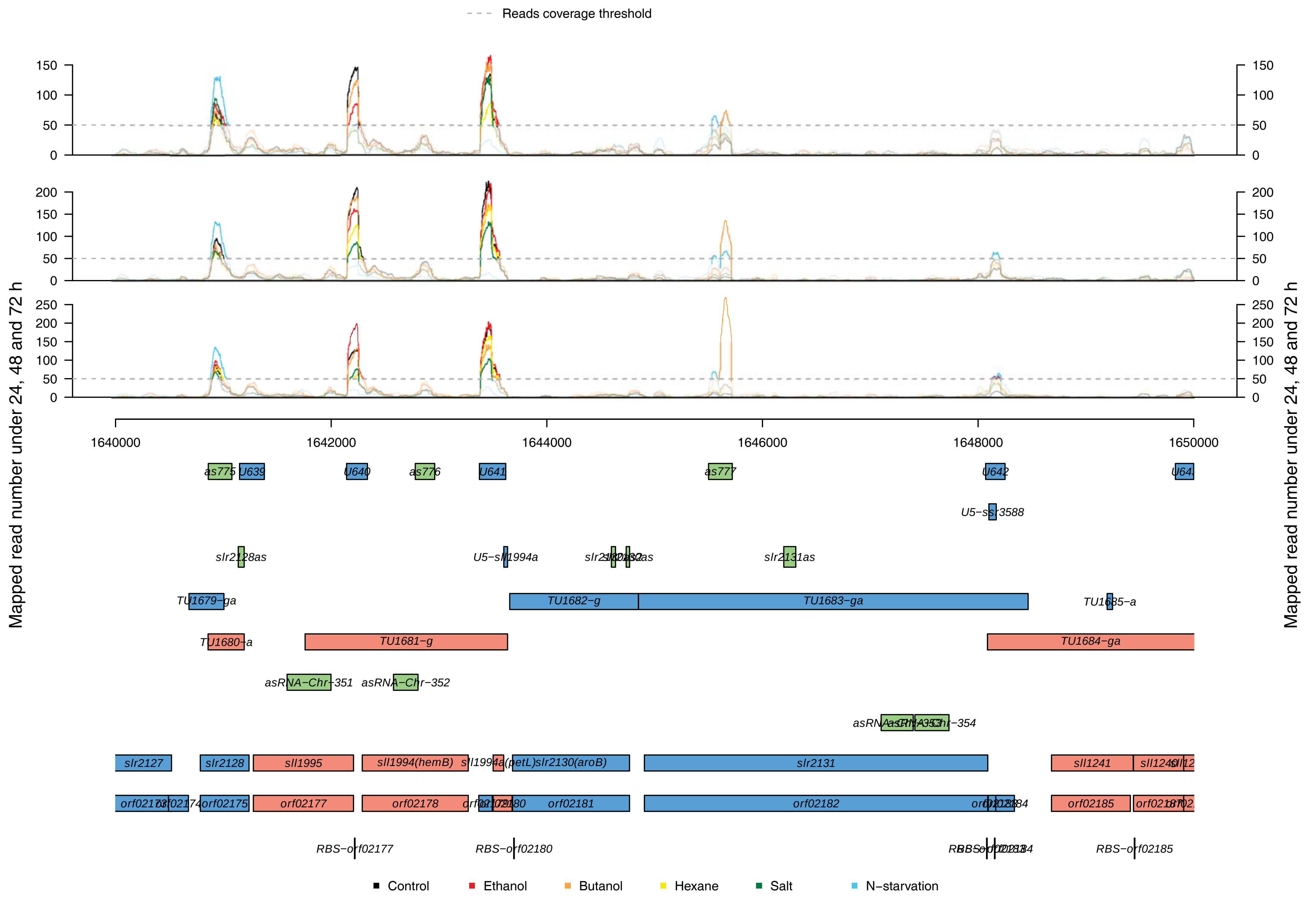
Reads coverage threshold
Mapped read number under 24, 48 and 72 h
1640000
1642000
1644000
1646000
1648000
1650000
as775
U639
U640
as776
U641
as777
U642
U5−ssr3588
slr2128as
U5−sll1994a
slr2131as
TU1679−ga
TU1682−g
TU1683−ga
TU1680−a
TU1681−g
TU1684−ga
asRNA−Chr−351
asRNA−Chr−352
slr2127
slr2128
sll1995
sll1994(hemB)
slr2130(aroB)
slr2131
sll1241
orf02175
orf02177
orf02178
orf02181
orf02182
orf02185
RBS−orf02177
RBS−orf02180
RBS−orf02185
Control
Ethanol
Butanol
Hexane
Salt
N−starvation

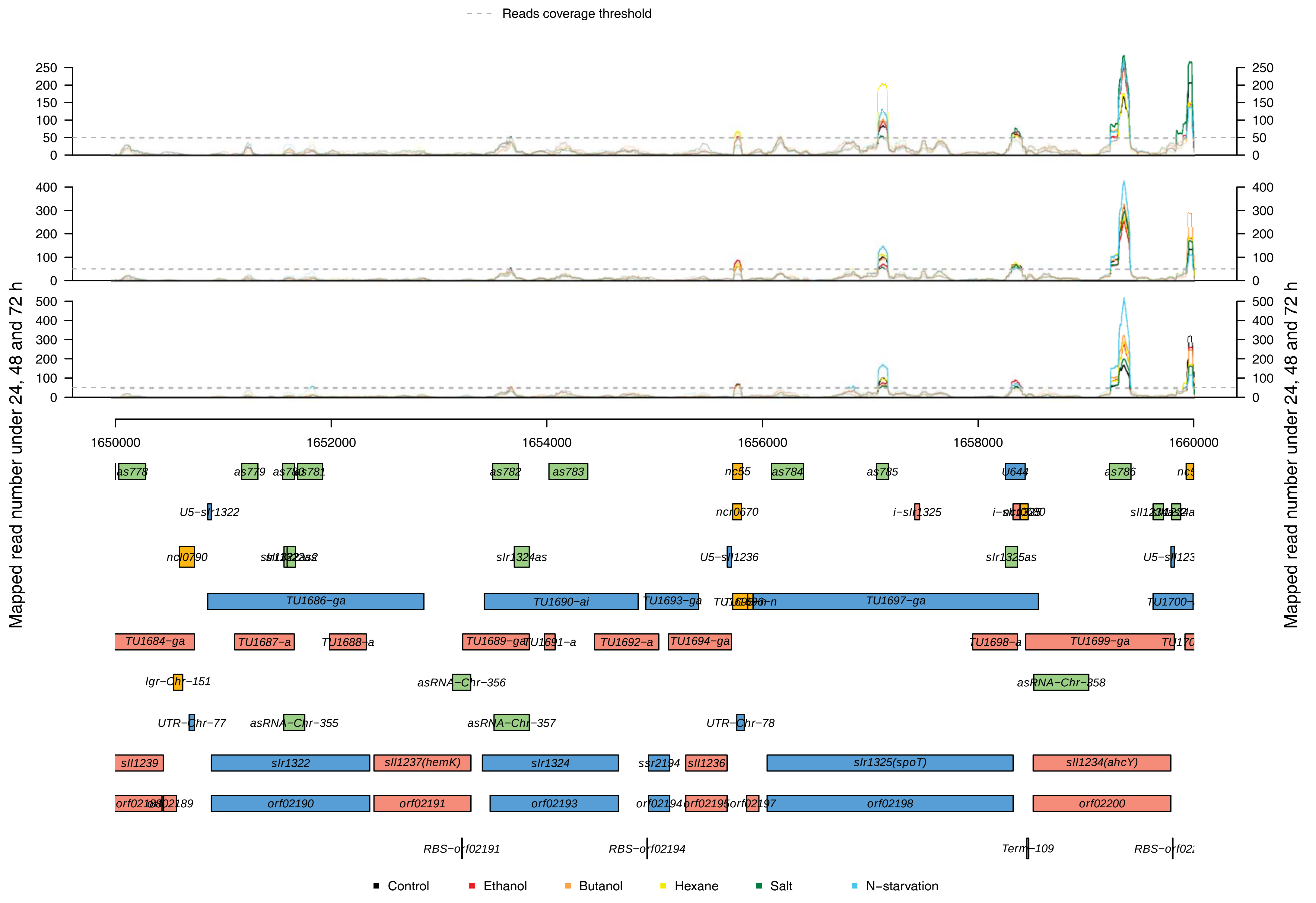

Reads coverage threshold
Mapped read number under 24, 48 and 72 h
1650000
1652000
1654000
1656000
1658000
1660000
as778
as779
as781
as782
as783
nc55
as784
as785
U644
as786
U5−slr1322
ncr0670
i−slr1325
ncl0790
slr1324as
U5−sll1236
slr1325as
TU1686−ga
TU1690−ai
TU1693−ga
TU1697−ga
TU1684−ga
TU1687−a
TU1688−a
TU1689−ga
TU1691−a
TU1692−a
TU1694−ga
TU1698−a
TU1699−ga
Igr−Chr−151
asRNA−Chr−356
asRNA−Chr−358
UTR−Chr−77
asRNA−Chr−355
asRNA−Chr−357
UTR−Chr−78
sll1239
slr1322
sll1237(hemK)
slr1324
ssr2194
sll1236
slr1325(spoT)
sll1234(ahcY)
orf02189
orf02190
orf02191
orf02193
orf02194
orf02195
orf02197
orf02198
orf02200
RBS−orf02191
RBS−orf02194
Term−109
Control
Ethanol
Butanol
Hexane
Salt
N−starvation

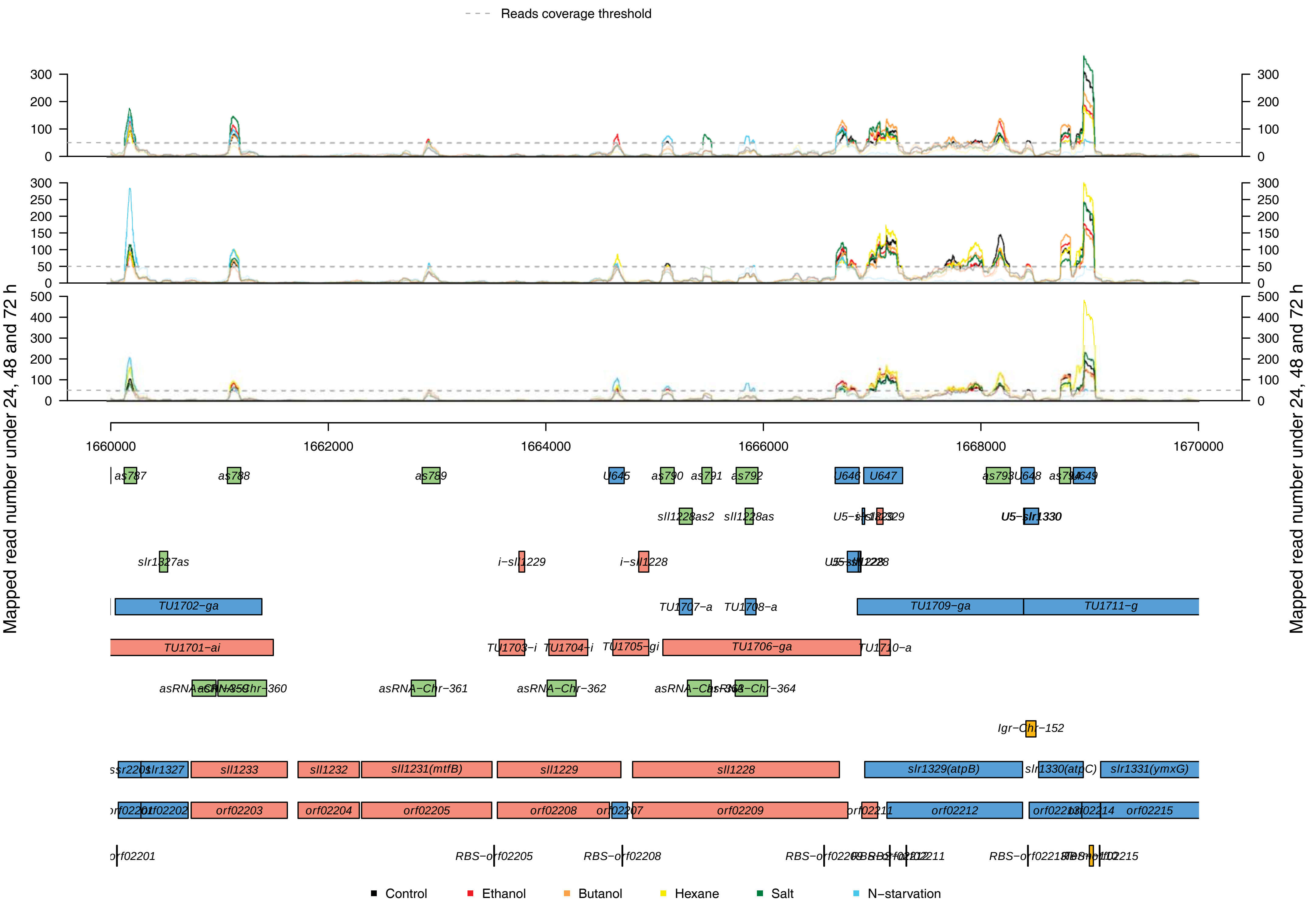
Reads coverage threshold
Mapped read number under 24, 48 and 72 h
1660000
1662000
1664000
1666000
1668000
1670000
TU1702−ga
TU1709−ga
TU1711−g
TU1701−ai
TU1706−ga
slr1329(atpB)
sll1228
sll1231(mtfB)
sll1233
sll1232
sll1229
slr1331(ymxG)
orf02203
orf02204
orf02205
orf02209
orf02212
orf02215
Igr−Chr−152
Control
Ethanol
Butanol
Hexane
Salt
N−starvation

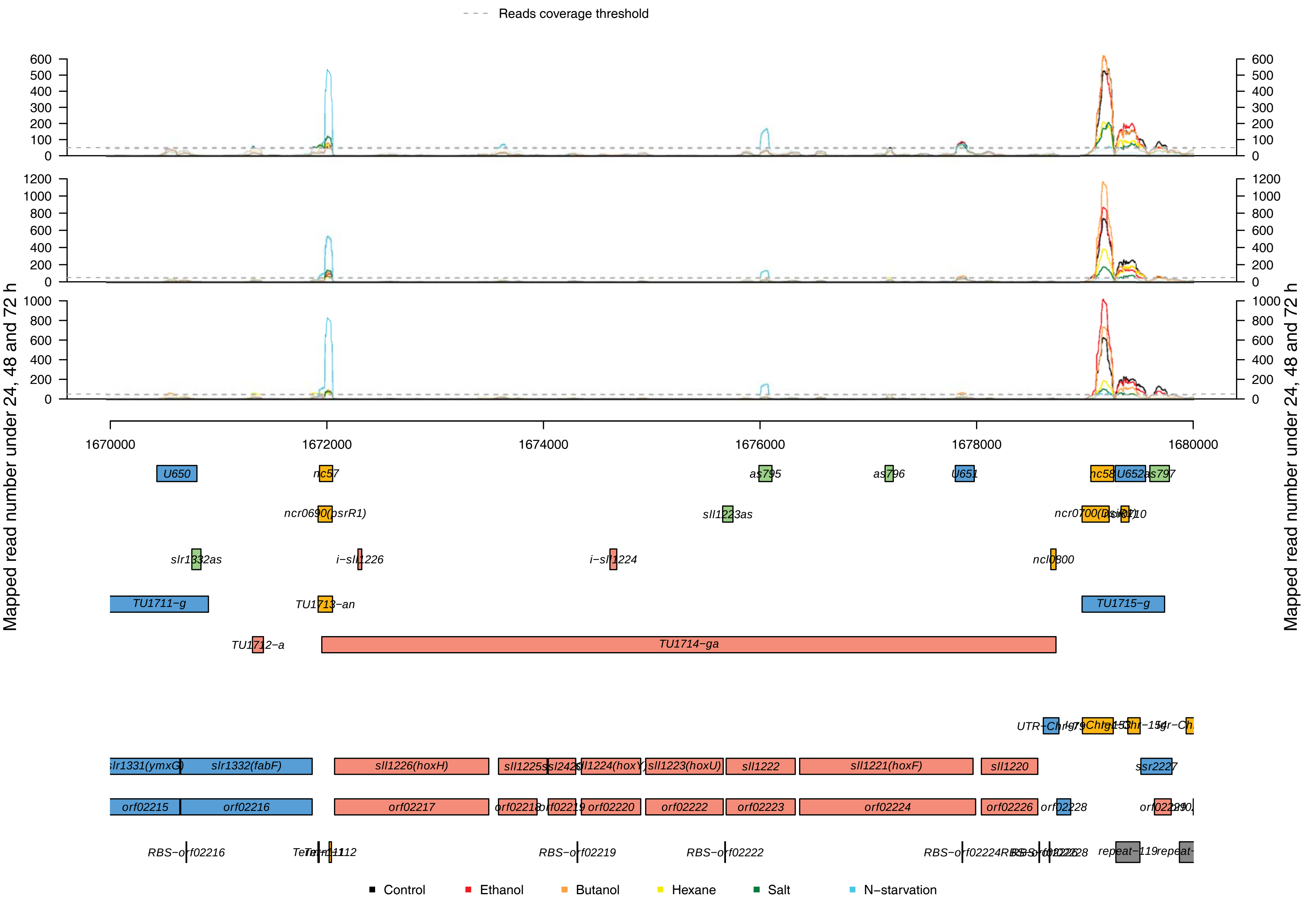

Reads coverage threshold
Mapped read number under 24, 48 and 72 h
1670000
1672000
1674000
1676000
1678000
1680000
U650
nc57
as795
as796
U651
ncr0690(psrR1)
sll1223as
slr1332as
i−sll1226
i−sll1224
ncl0800
TU1711−g
TU1713−an
TU1715−g
TU1712−a
TU1714−ga
slr1332(fabF)
sll1226(hoxH)
sll1223(hoxU)
sll1222
sll1221(hoxF)
sll1220
ssr2227
orf02215
orf02216
orf02217
orf02220
orf02222
orf02223
orf02224
orf02226
RBS−orf02216
RBS−orf02219
RBS−orf02222
Control
Ethanol
Butanol
Hexane
Salt
N−starvation

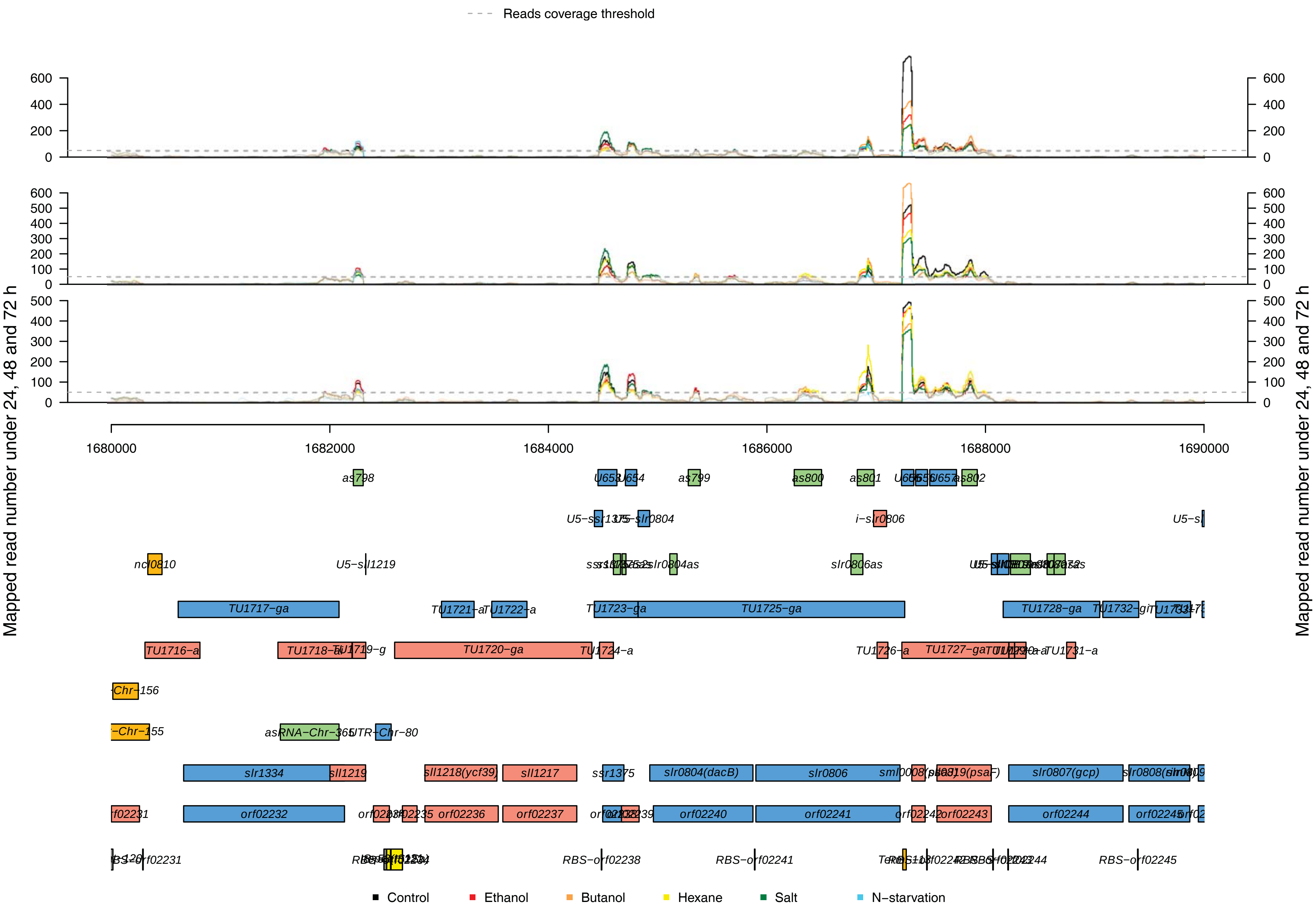

Reads coverage threshold
Mapped read number under 24, 48 and 72 h
1680000
1682000
1684000
1686000
1688000
1690000
as798
as799
as800
as801
as802
U654
i-slr0806
slr0806as
nc10810
U5-sll1219
TU1717-ga
TU1723-ga
TU1725-ga
TU1728-ga
TU1716-a
TU1720-ga
TU1731-a
asRNA-Chr-365
slr1334
sll1219
sll1218(ycf39)
sll1217
ssr1375
slr0804(dacB)
slr0806
slr0807(gcp)
orf02232
orf02236
orf02237
orf02240
orf02241
orf02243
orf02244
RBS-orf02238
RBS-orf02241
RBS-orf02245
Control
Ethanol
Butanol
Hexane
Salt
N-starvation

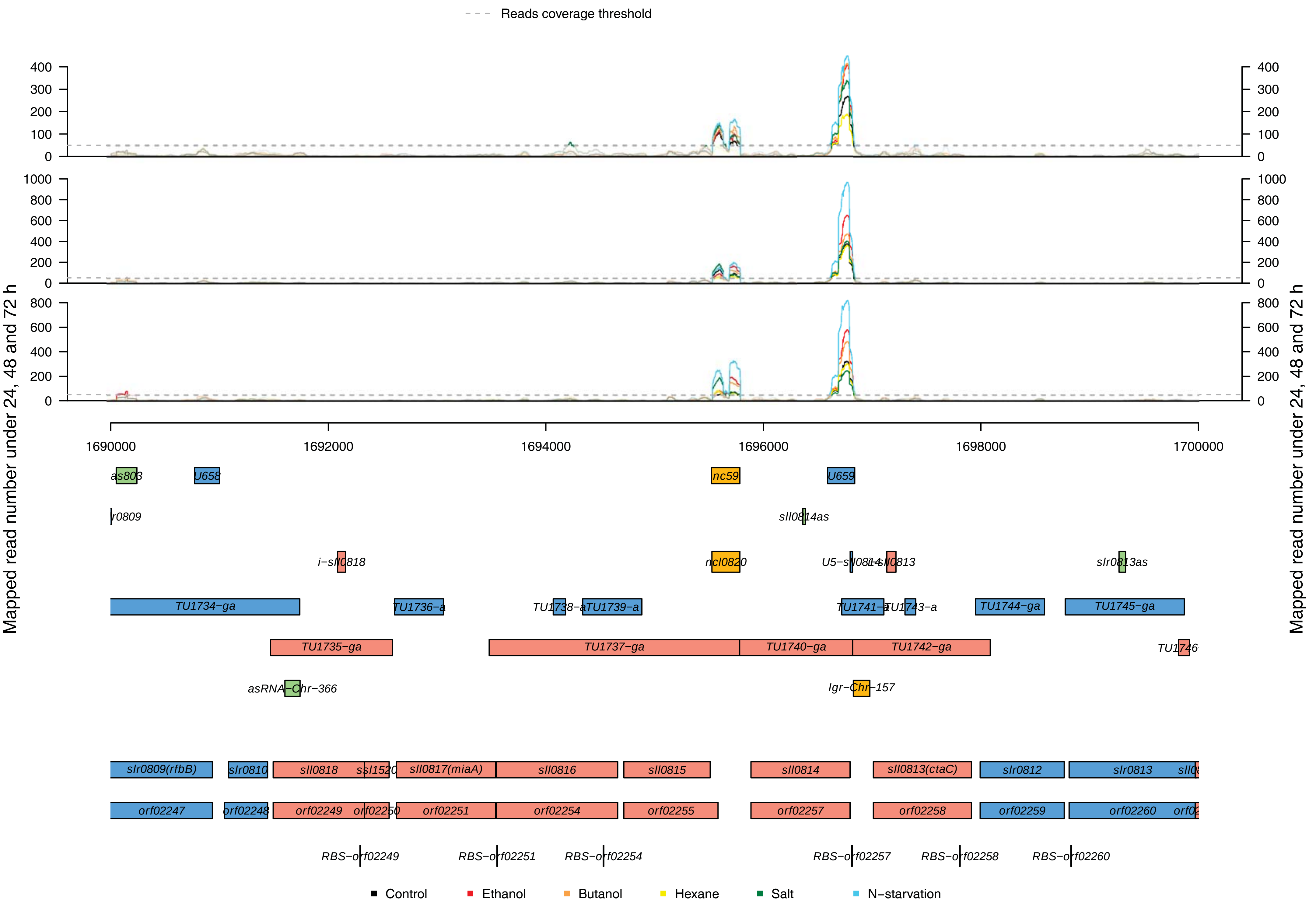

Reads coverage threshold
Mapped read number under 24, 48 and 72 h
1690000
1692000
1694000
1696000
1698000
1700000
as803
U658
nc59
U659
r0809
sll0814as
i-sll0818
ncl0820
U5-sll0814
sll0813
slr0813as
TU1734-ga
TU1736-a
TU1738-a
TU1739-a
TU1741-a
TU1743-a
TU1744-ga
TU1745-ga
TU1735-ga
TU1737-ga
TU1740-ga
TU1742-ga
asRNA-Chr-366
Igr-Chr-157
slr0809(rfbB)
slr0810
sll0818
sll0817(miaA)
sll0816
sll0815
sll0814
sll0813(ctaC)
slr0812
slr0813
orf02247
orf02248
orf02249
orf02251
orf02254
orf02255
orf02257
orf02258
orf02259
orf02260
RBS-orf02249
RBS-orf02251
RBS-orf02254
RBS-orf02257
RBS-orf02258
RBS-orf02260
Control
Ethanol
Butanol
Hexane
Salt
N-starvation

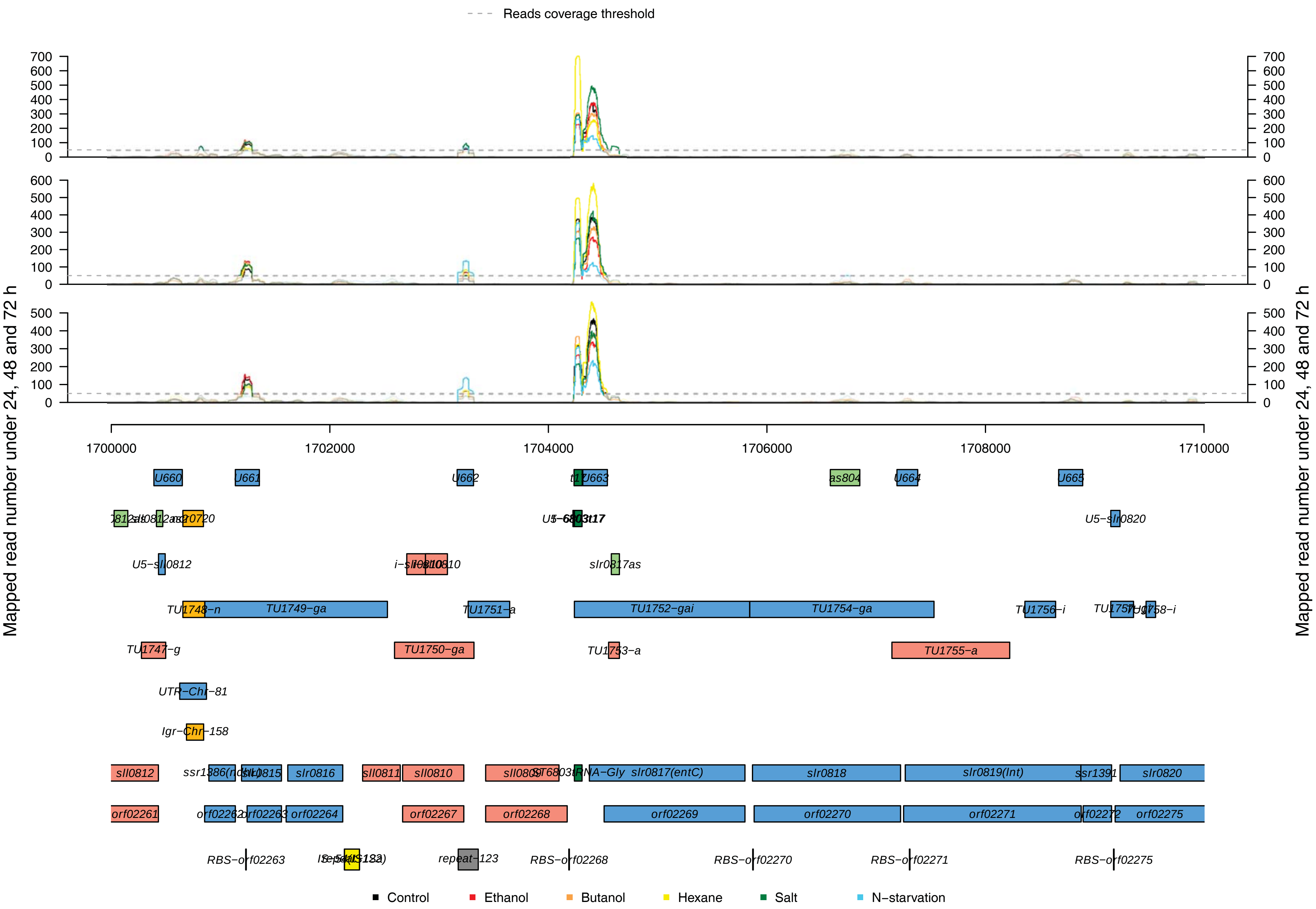

Reads coverage threshold
Mapped read number under 24, 48 and 72 h
1700000
1702000
1704000
1706000
1708000
1710000
U660
U661
U662
U663
as804
U664
U665
U5−sll0812
U5−slr0820
slr0817as
TU1748−n
TU1749−ga
TU1751−a
TU1752−gai
TU1754−ga
TU1756−i
TU1747−g
TU1750−ga
TU1753−a
TU1755−a
UTR−Chr−81
Igr−Chr−158
sll0812
slr0816
sll0811
sll0810
slr0817(entC)
slr0818
slr0819(Int)
ssr1391
slr0820
orf02261
orf02264
orf02267
orf02268
orf02269
orf02270
orf02271
orf02275
RBS−orf02263
repeat−123
RBS−orf02268
RBS−orf02270
RBS−orf02271
RBS−orf02275
Control
Ethanol
Butanol
Hexane
Salt
N−starvation

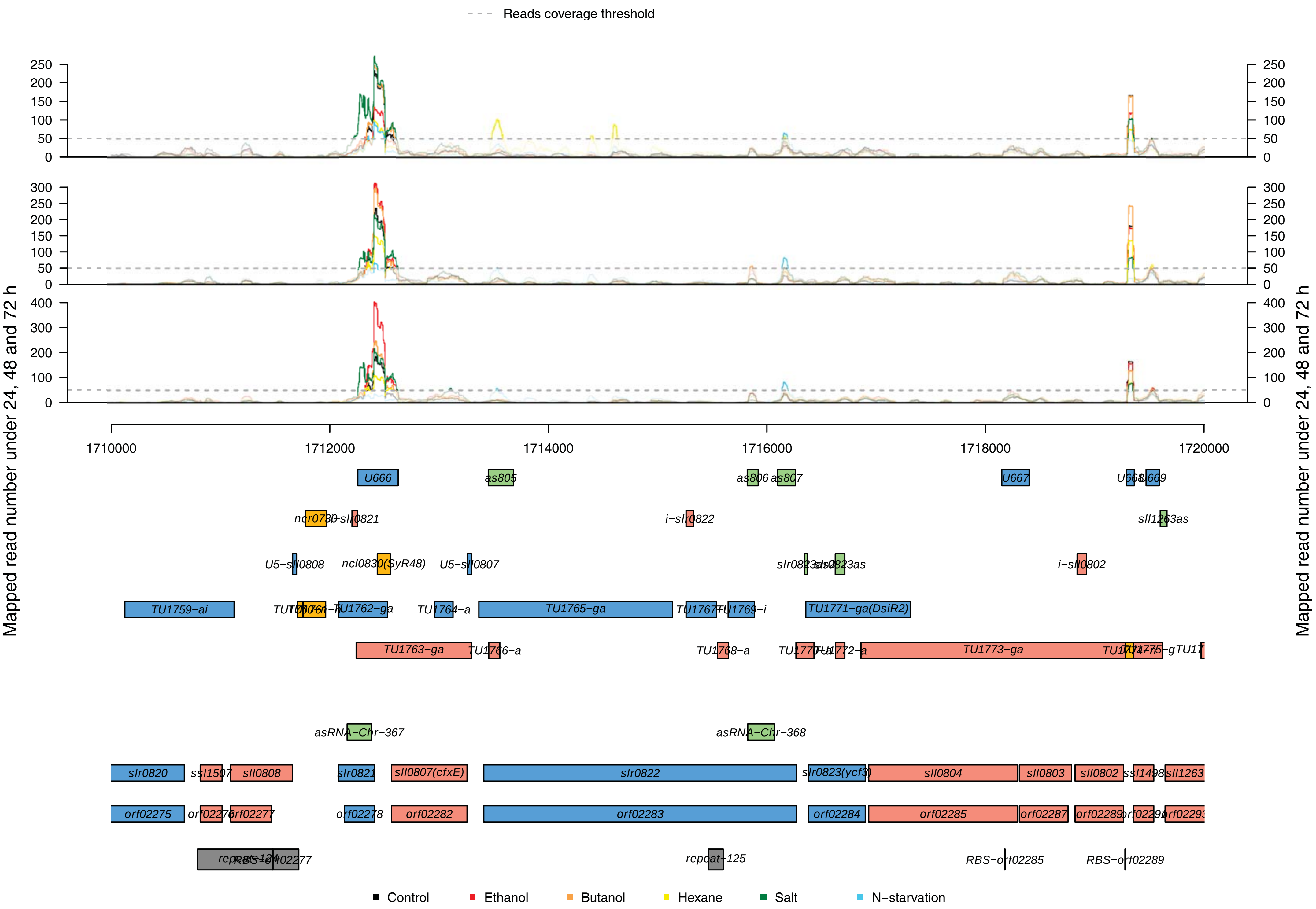

Reads coverage threshold
Mapped read number under 24, 48 and 72 h
1710000
1712000
1714000
1716000
1718000
1720000
U666
as805
as806
as807
U667
U669
ncr0730
i−slr0822
sll1263as
U5−sll0808
ncl0830(SyR48)
U5−sll0807
slr0823as
i−sll0802
TU1759−ai
TU1762−ga
TU1764−a
TU1765−ga
TU1769−i
TU1771−ga(DsiR2)
TU1763−ga
TU1766−a
TU1768−a
TU1773−ga
asRNA−Chr−367
asRNA−Chr−368
slr0820
ssl1507
sll0808
slr0821
sll0807(cfxE)
slr0822
slr0823(ycf3)
sll0804
sll0803
sll0802
ssl1498
sll1263
orf02275
orf02277
orf02278
orf02282
orf02283
orf02284
orf02285
orf02287
orf02289
repeat−125
RBS−orf02285
RBS−orf02289
Control
Ethanol
Butanol
Hexane
Salt
N−starvation

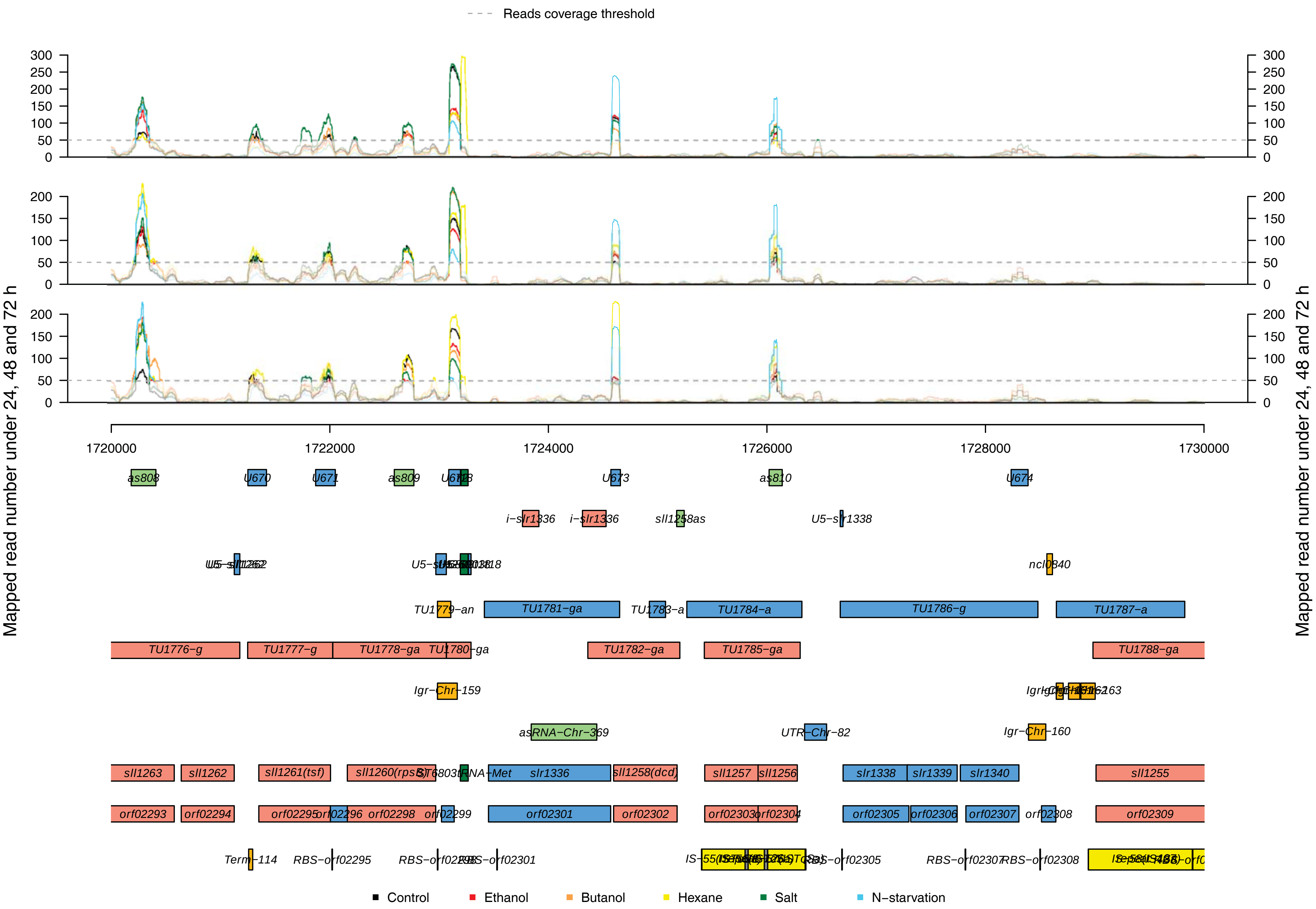

Reads coverage threshold
Mapped read number under 24, 48 and 72 h
1720000
1722000
1724000
1726000
1728000
1730000
as808
U670
U671
as809
U673
as810
U674
TU1779−an
TU1781−ga
TU1784−a
TU1786−g
TU1787−a
TU1776−g
TU1777−g
TU1778−ga
TU1782−ga
TU1785−ga
TU1788−ga
Igr−Chr−159
asRNA−Chr−369
UTR−Chr−82
Igr−Chr−160
sll1263
sll1262
sll1261(tsf)
slr1336
sll1258(dcd)
sll1257
sll1256
slr1338
slr1339
slr1340
sll1255
orf02293
orf02294
orf02295
orf02298
orf02301
orf02302
orf02305
orf02306
orf02307
orf02309
Term−114
RBS−orf02295
RBS−orf02301
RBS−orf02305
Control
Ethanol
Butanol
Hexane
Salt
N−starvation

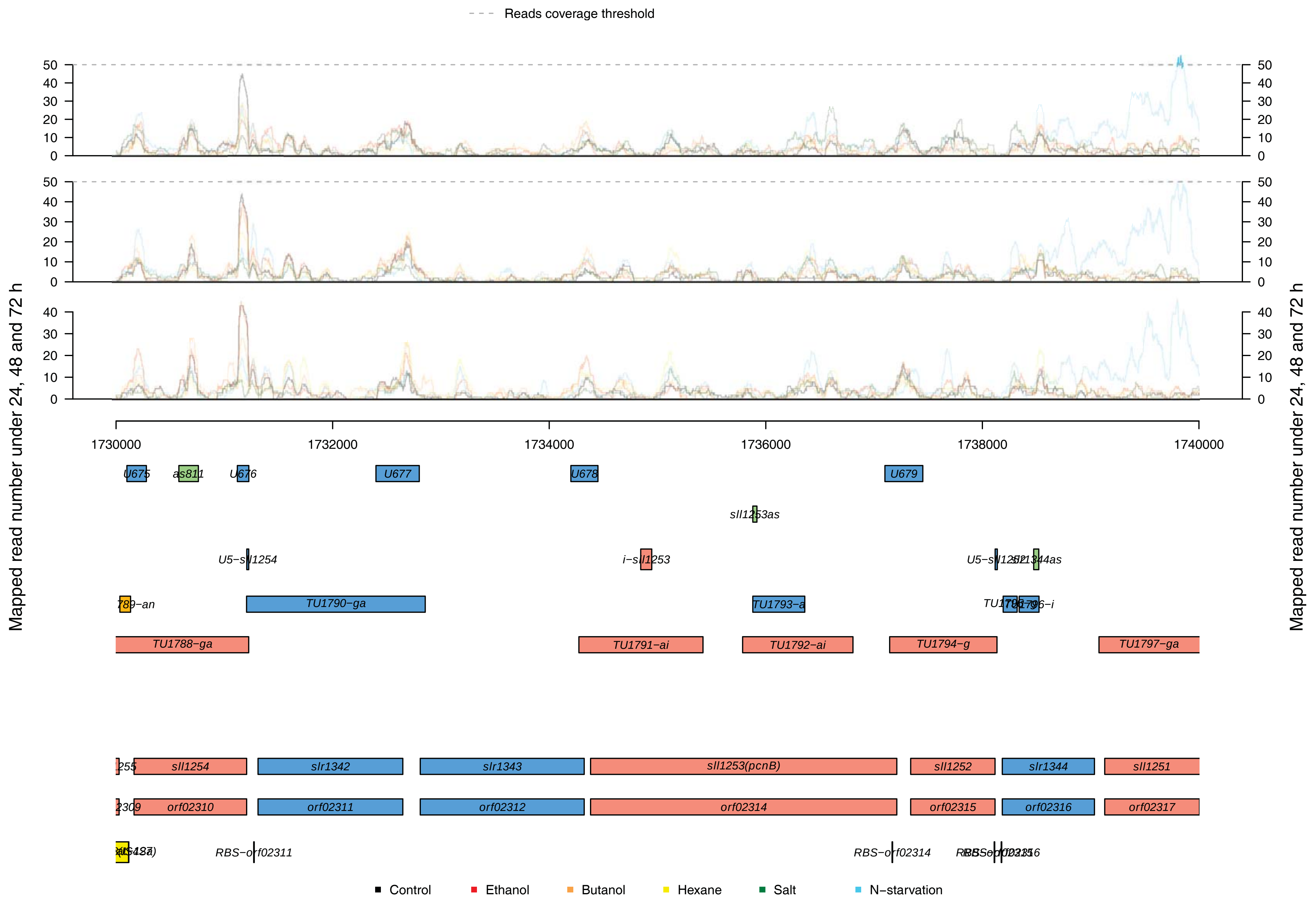

Reads coverage threshold
Mapped read number under 24, 48 and 72 h
1730000
1732000
1734000
1736000
1738000
1740000
U675
as811
U676
U677
U678
U679
sll1253as
U5−sll1254
i−sll1253
TU1790−ga
TU1793−a
TU1788−ga
TU1791−ai
TU1792−ai
TU1794−g
TU1797−ga
sll1254
slr1342
slr1343
sll1253(pcnB)
sll1252
slr1344
sll1251
orf02310
orf02311
orf02312
orf02314
orf02315
orf02316
orf02317
RBS−orf02311
RBS−orf02314
Control
Ethanol
Butanol
Hexane
Salt
N−starvation

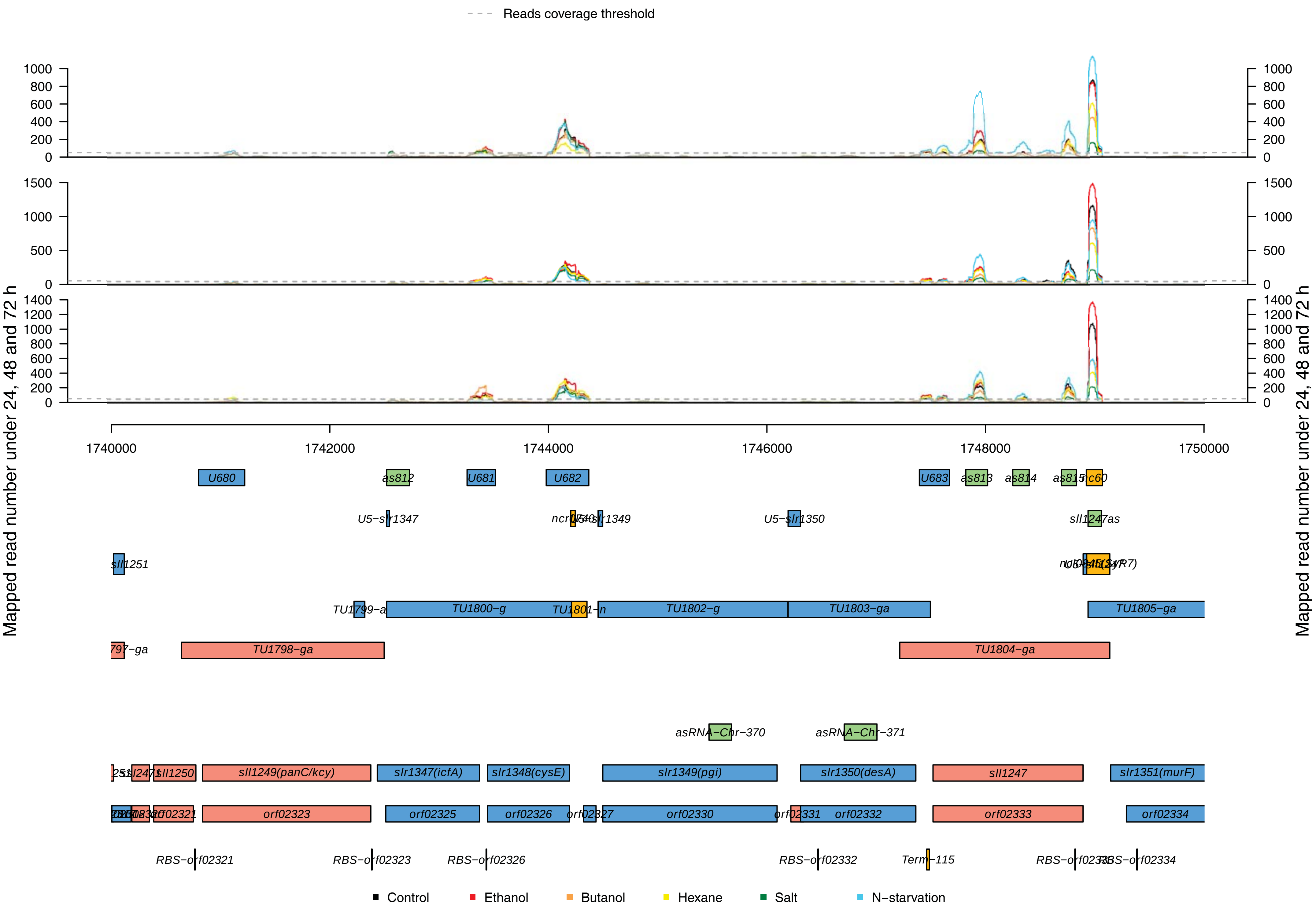

Reads coverage threshold
Mapped read number under 24, 48 and 72 h
1740000
1742000
1744000
1746000
1748000
1750000
U680
as812
U681
U682
U683
as813
as814
as815
U5−slr1347
U5−slr1350
sll1247as
sll1251
TU1799−a
TU1800−g
TU1802−g
TU1803−ga
TU1805−ga
TU1798−ga
TU1804−ga
asRNA−Chr−370
asRNA−Chr−371
sll1250
sll1249(panC/kcy)
slr1347(icfA)
slr1348(cysE)
slr1349(pgi)
slr1350(desA)
sll1247
slr1351(murF)
orf02323
orf02325
orf02326
orf02330
orf02332
orf02333
orf02334
RBS−orf02321
RBS−orf02323
RBS−orf02326
RBS−orf02332
Term−115
Control
Ethanol
Butanol
Hexane
Salt
N−starvation

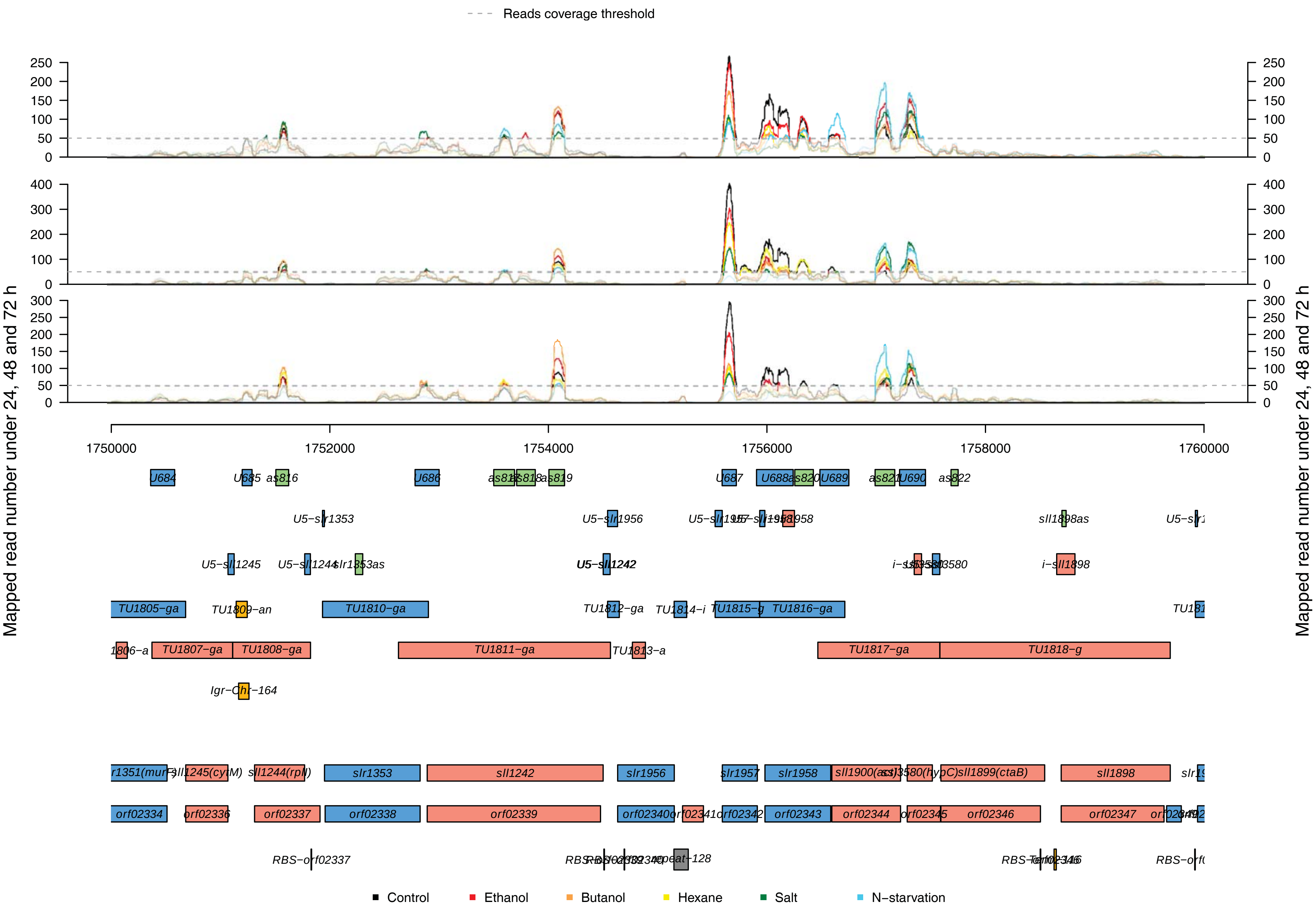

Reads coverage threshold
Mapped read number under 24, 48 and 72 h
1750000
1752000
1754000
1756000
1758000
1760000
TU1805−ga
TU1807−ga
TU1808−ga
TU1810−ga
TU1811−ga
TU1817−ga
TU1818−g
slr1353
sll1242
slr1956
slr1958
sll1898
orf02334
orf02337
orf02338
orf02339
orf02343
orf02344
orf02346
orf02347
RBS−orf02337
Control
Ethanol
Butanol
Hexane
Salt
N−starvation

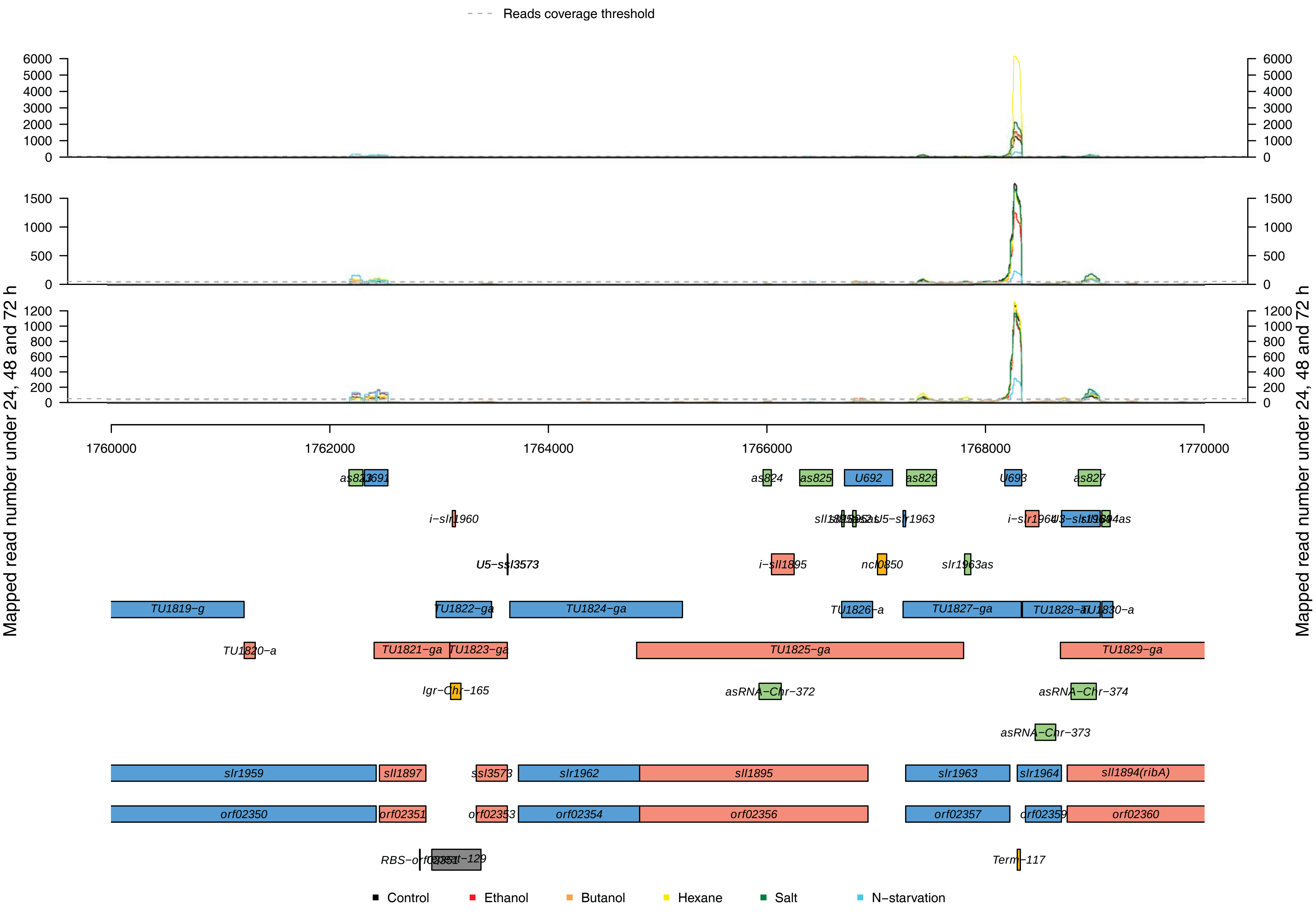
Reads coverage threshold
Mapped read number under 24, 48 and 72 h
1760000
1762000
1764000
1766000
1768000
1770000
as824
as825
U692
as826
as827
i−slr1960
U5−ssl3573
i−sll1895
ncl0850
slr1963as
TU1819−g
TU1822−ga
TU1824−ga
TU1827−ga
TU1820−a
TU1821−ga
TU1823−ga
TU1825−ga
TU1829−ga
Igr−Chr−165
asRNA−Chr−372
asRNA−Chr−374
asRNA−Chr−373
slr1959
sll1897
slr1962
sll1895
slr1963
slr1964
sll1894(ribA)
orf02350
orf02351
orf02354
orf02356
orf02357
orf02360
Term−117
Control
Ethanol
Butanol
Hexane
Salt
N−starvation

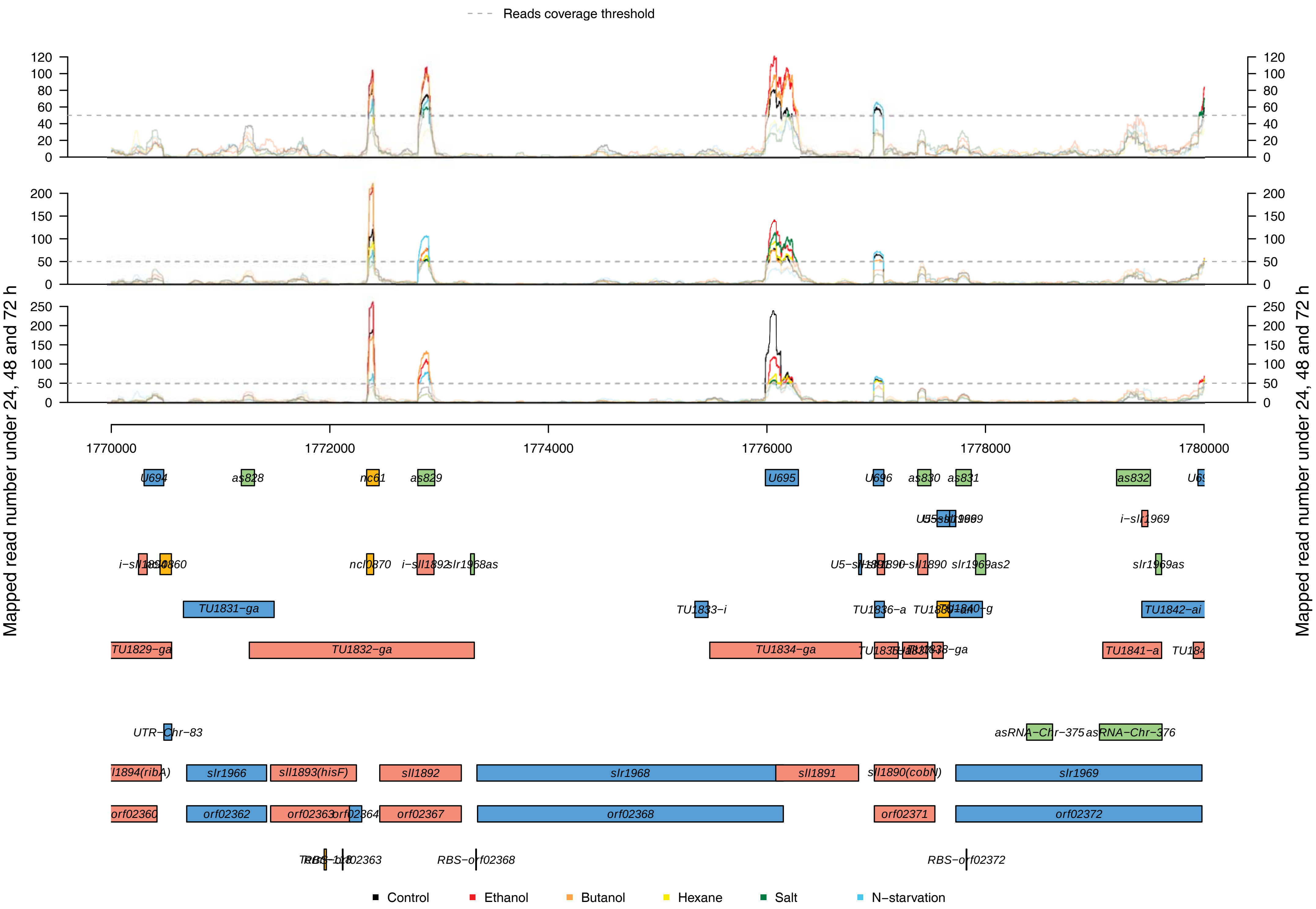

Reads coverage threshold
Mapped read number under 24, 48 and 72 h
1770000
1772000
1774000
1776000
1778000
1780000
U694
as828
nc61
as829
U695
U696
as830
as831
as832
i−slr1969
TU1831−ga
TU1833−i
TU1836−a
TU1842−ai
TU1829−ga
TU1832−ga
TU1834−ga
TU1841−a
UTR−Chr−83
asRNA−Chr−375
asRNA−Chr−376
slr1966
sll1893(hisF)
sll1892
slr1968
sll1891
sll1890(cobN)
slr1969
orf02360
orf02362
orf02367
orf02368
orf02371
orf02372
RBS−orf02368
RBS−orf02372
Control
Ethanol
Butanol
Hexane
Salt
N−starvation

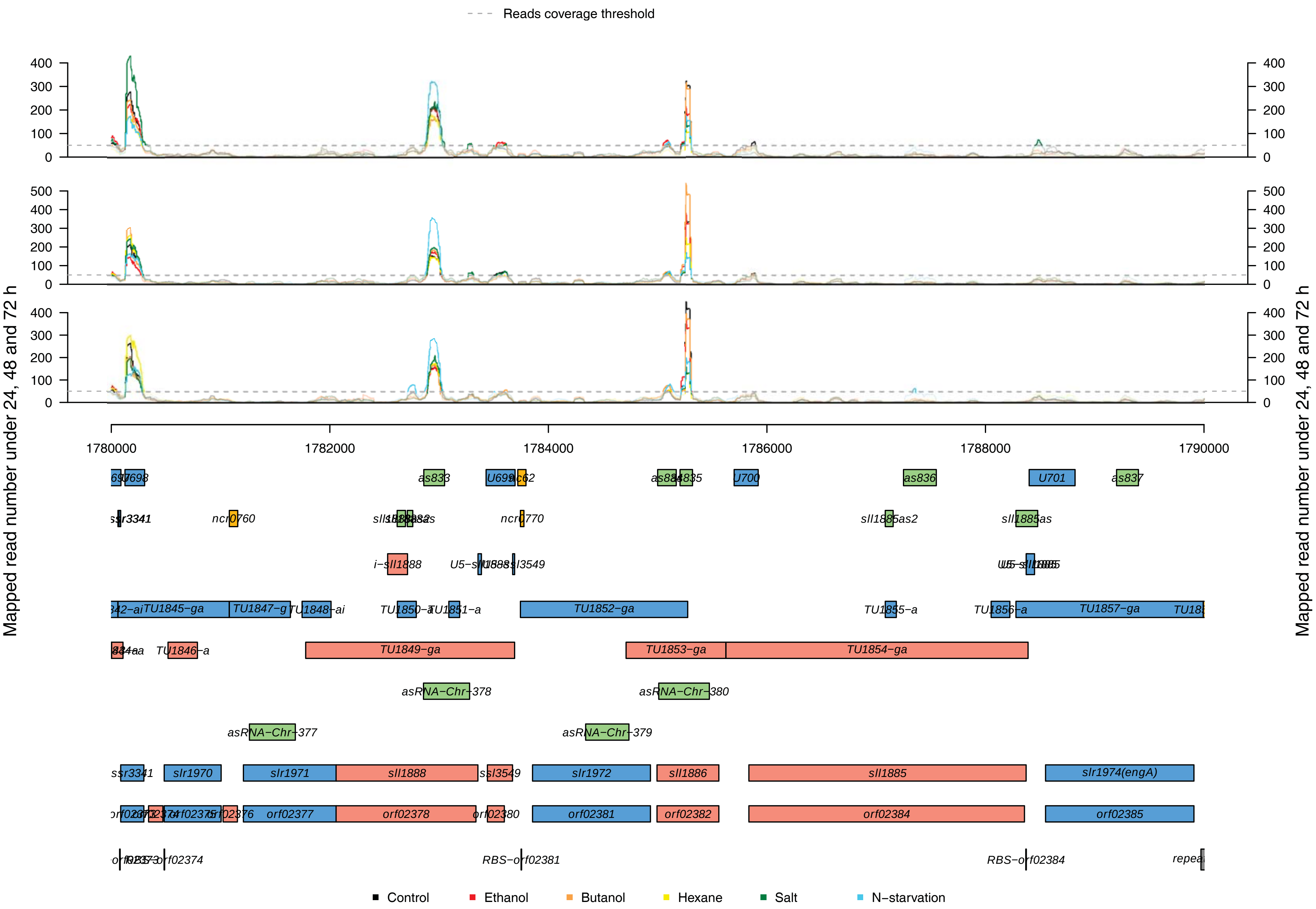

Reads coverage threshold
Mapped read number under 24, 48 and 72 h
1780000
1782000
1784000
1786000
1788000
1790000
as833
U699
as836
U701
as837
U700
ncr0760
ncr0770
sll1885as2
sll1885as
i−sll1888
TU1845−ga
TU1847−ga
TU1848−ai
TU1850−a
TU1851−a
TU1852−ga
TU1855−a
TU1856−a
TU1857−ga
TU1846−a
TU1849−ga
TU1853−ga
TU1854−ga
asRNA−Chr−378
asRNA−Chr−380
asRNA−Chr−377
asRNA−Chr−379
ssr3341
slr1970
slr1971
sll1888
ssl3549
slr1972
sll1886
sll1885
slr1974(engA)
orf02377
orf02378
orf02380
orf02381
orf02382
orf02384
orf02385
RBS−orf02374
RBS−orf02381
RBS−orf02384
Control
Ethanol
Butanol
Hexane
Salt
N−starvation

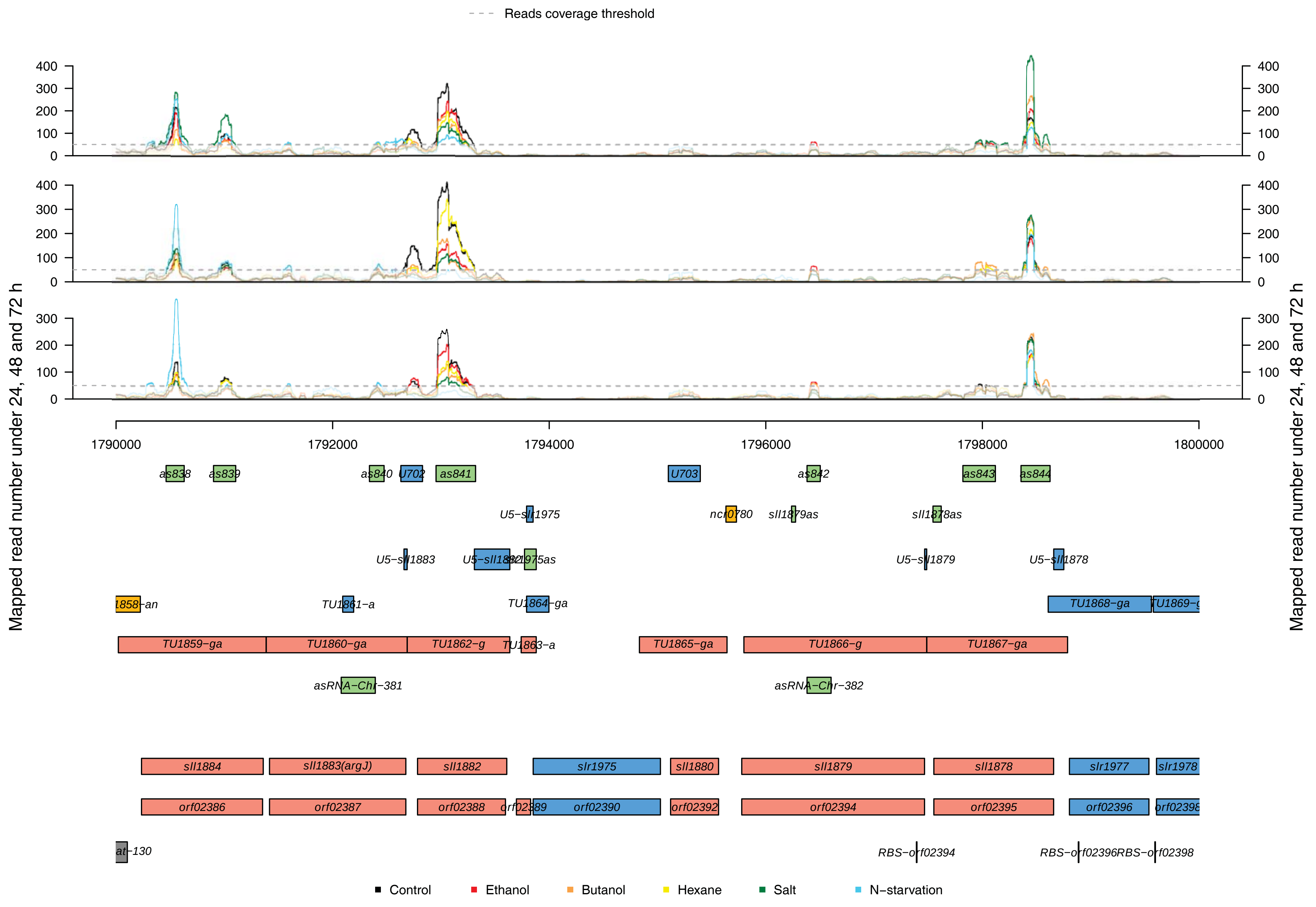

Reads coverage threshold
Mapped read number under 24, 48 and 72 h
1790000
1792000
1794000
1796000
1798000
1800000
as838
as839
as840
U702
as841
U703
as842
as843
as844
U5−slr1975
ncr0780
sll1879as
sll1878as
U5−sll1883
U5−sll1882
slr1975as
U5−sll1879
U5−sll1878
TU1861−a
TU1864−ga
TU1868−ga
TU1859−ga
TU1860−ga
TU1862−g
TU1863−a
TU1865−ga
TU1866−g
TU1867−ga
asRNA−Chr−381
asRNA−Chr−382
sll1884
sll1883(argJ)
sll1882
slr1975
sll1880
sll1879
sll1878
slr1977
slr1978
orf02386
orf02387
orf02388
orf02389
orf02390
orf02392
orf02394
orf02395
orf02396
orf02398
RBS−orf02394
RBS−orf02396
RBS−orf02398
Control
Ethanol
Butanol
Hexane
Salt
N−starvation

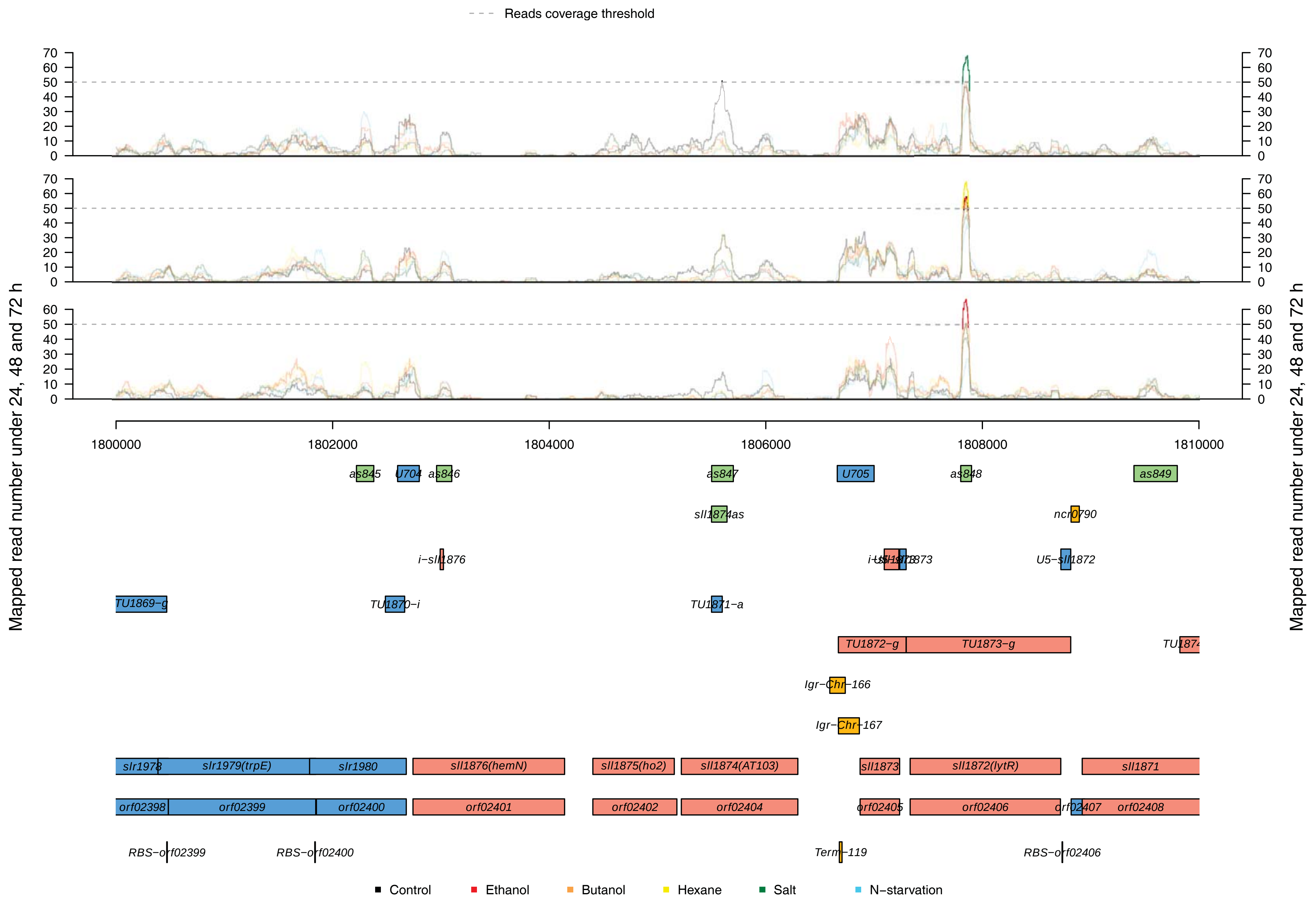

Reads coverage threshold
Mapped read number under 24, 48 and 72 h
1800000
1802000
1804000
1806000
1808000
1810000
as845
U704
as846
as847
U705
as848
as849
sll1874as
ncr0790
i−sll1876
U5−sll1872
TU1869−g
TU1870−i
TU1871−a
TU1872−g
TU1873−g
Igr−Chr−166
Igr−Chr−167
slr1978
slr1979(trpE)
slr1980
sll1876(hemN)
sll1875(ho2)
sll1874(AT103)
sll1873
sll1872(lytR)
sll1871
orf02398
orf02399
orf02400
orf02401
orf02402
orf02404
orf02405
orf02406
orf02407
orf02408
RBS−orf02399
RBS−orf02400
Term−119
RBS−orf02406
Control
Ethanol
Butanol
Hexane
Salt
N−starvation

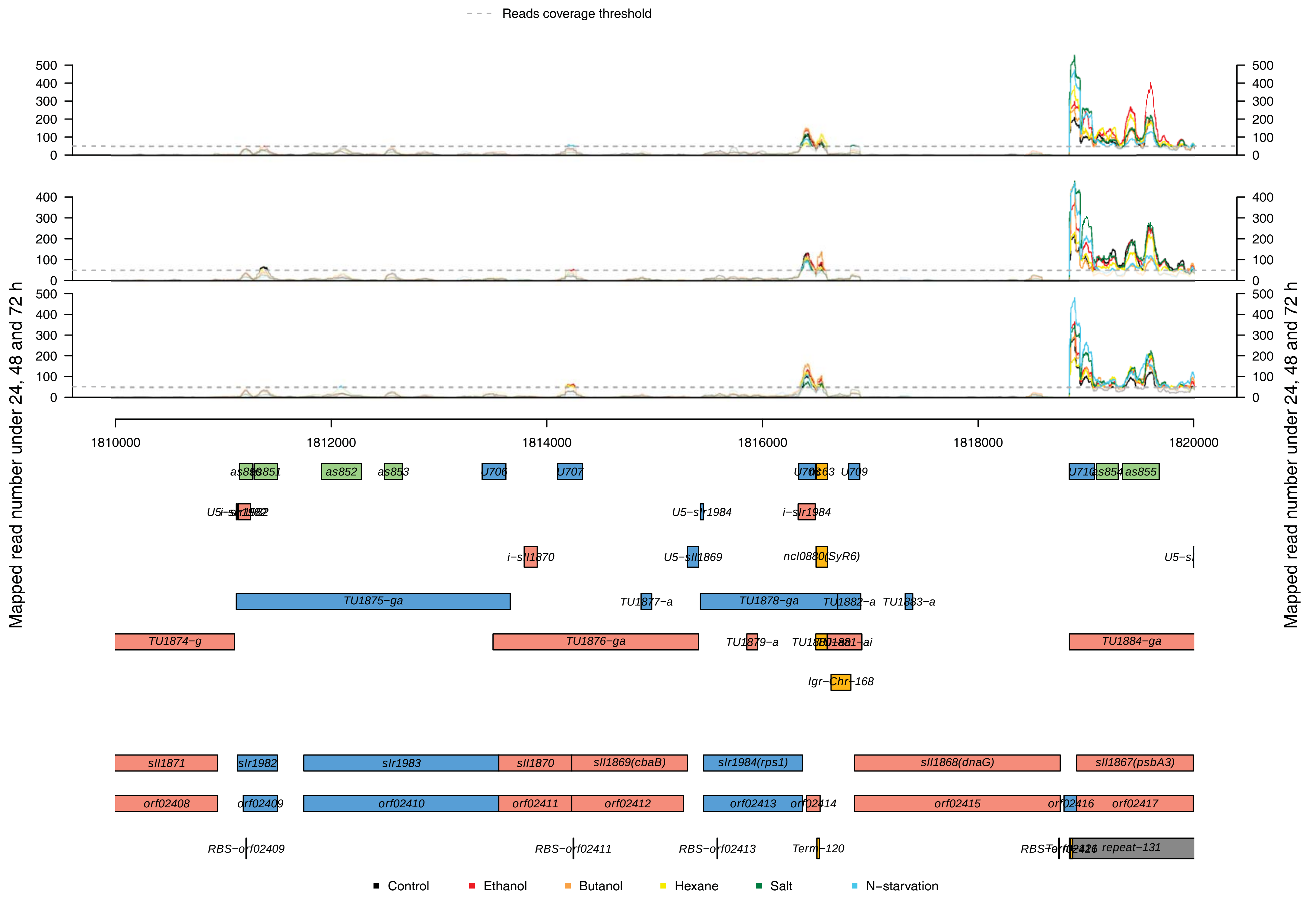

Reads coverage threshold
Mapped read number under 24, 48 and 72 h
1810000
1812000
1814000
1816000
1818000
1820000
TU1875−ga
TU1874−g
TU1876−ga
TU1878−ga
TU1884−ga
sll1871
slr1982
slr1983
sll1870
sll1869(cbaB)
slr1984(rps1)
sll1868(dnaG)
sll1867(psbA3)
orf02408
orf02410
orf02411
orf02412
orf02413
orf02415
orf02417
Control
Ethanol
Butanol
Hexane
Salt
N−starvation

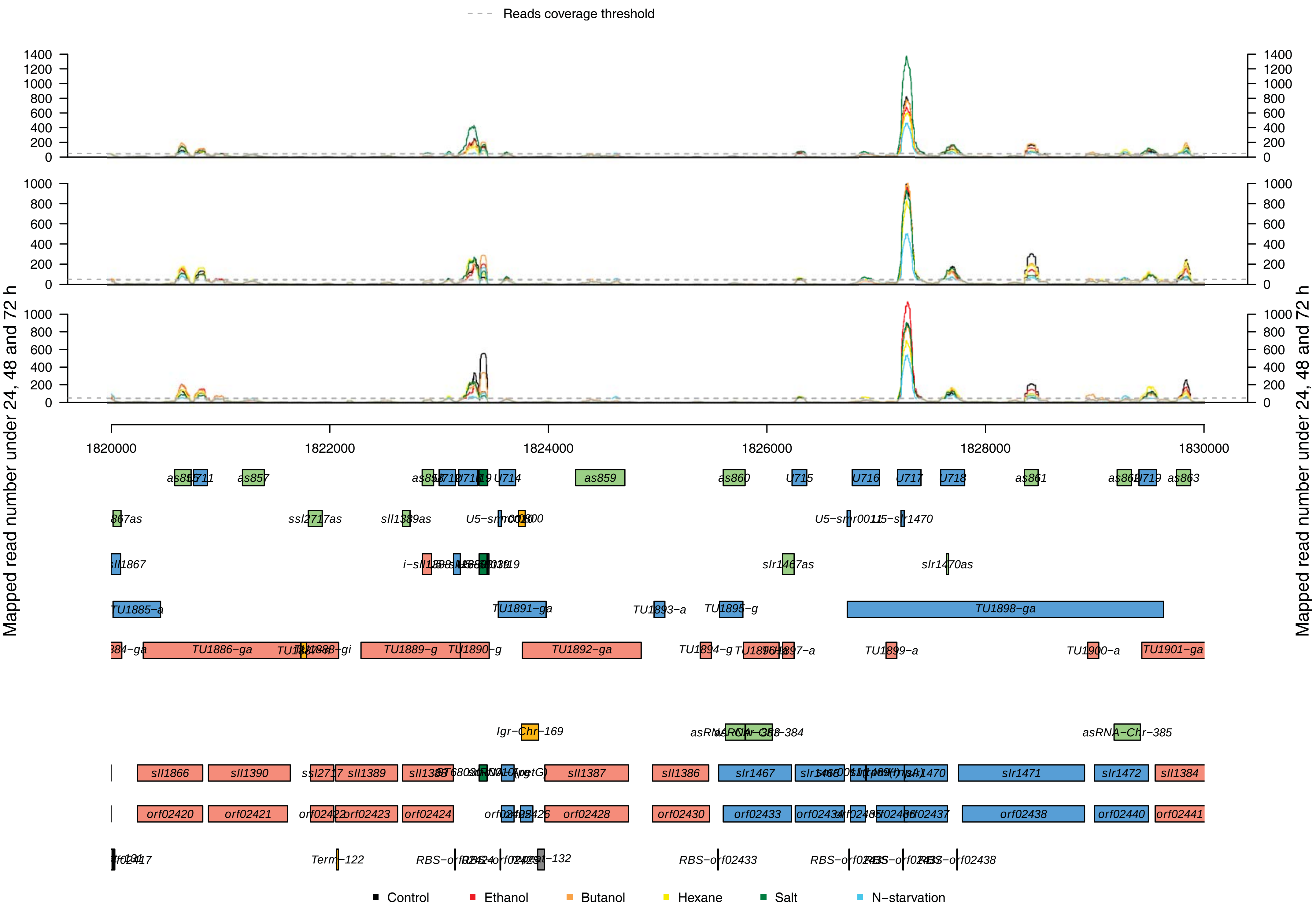

Reads coverage threshold
Mapped read number under 24, 48 and 72 h
1820000
1822000
1824000
1826000
1828000
1830000
as857
as859
as860
U715
U716
U717
U718
as861
as863
ssl2717as
sll1389as
slr1467as
slr1470as
TU1885−a
TU1891−ga
TU1893−a
TU1895−g
TU1898−ga
TU1886−ga
TU1889−g
TU1890−g
TU1892−ga
TU1894−g
TU1899−a
TU1900−a
TU1901−ga
Igr−Chr−169
asRNA−Chr−385
sll1866
sll1390
sll1389
sll1387
sll1386
slr1467
slr1471
slr1472
sll1384
orf02420
orf02421
orf02423
orf02424
orf02428
orf02430
orf02433
orf02437
orf02438
orf02440
orf02441
Term−122
RBS−orf02433
Control
Ethanol
Butanol
Hexane
Salt
N−starvation

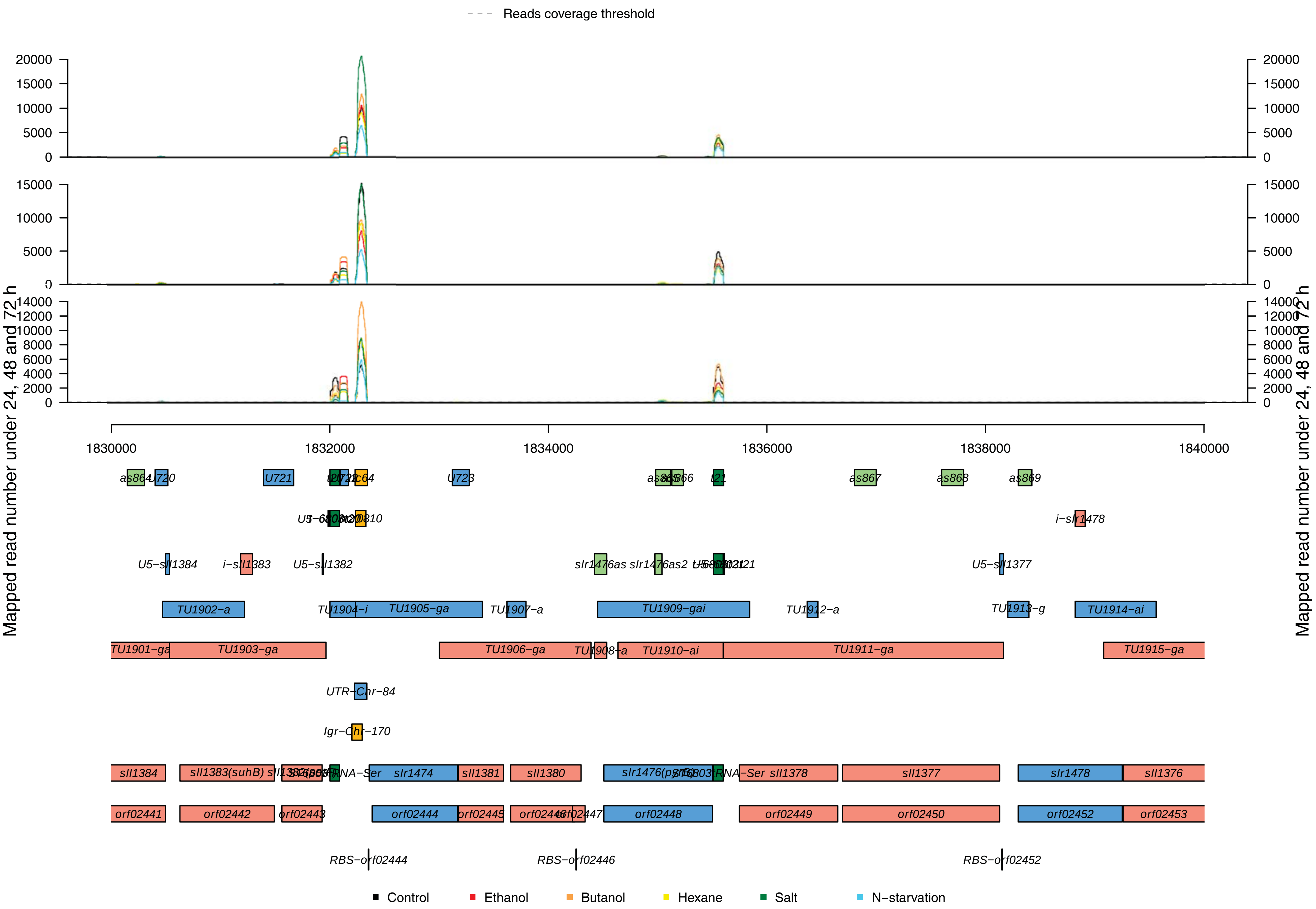
Reads coverage threshold
20000
15000
10000
5000
0
14000
12000
8000
6000
4000
2000
Mapped read number under 24, 48 and 72 h
1830000
1832000
1834000
1836000
1838000
1840000
U720
U721
U723
as867
as868
as869
i−slr1478
U5−sll1384
i−sll1383
U5−sll1382
U5−sll1377
TU1902−a
TU1905−ga
TU1909−gai
TU1912−a
TU1914−ai
TU1901−ga
TU1903−ga
TU1906−ga
TU1910−ai
TU1911−ga
TU1915−ga
UTR−Chr−84
Igr−Chr−170
sll1384
sll1383(suhB)
slr1474
sll1381
sll1380
sll1378
sll1377
slr1478
sll1376
orf02441
orf02442
orf02444
orf02448
orf02449
orf02450
orf02452
orf02453
RBS−orf02444
RBS−orf02446
RBS−orf02452
Control
Ethanol
Butanol
Hexane
Salt
N−starvation

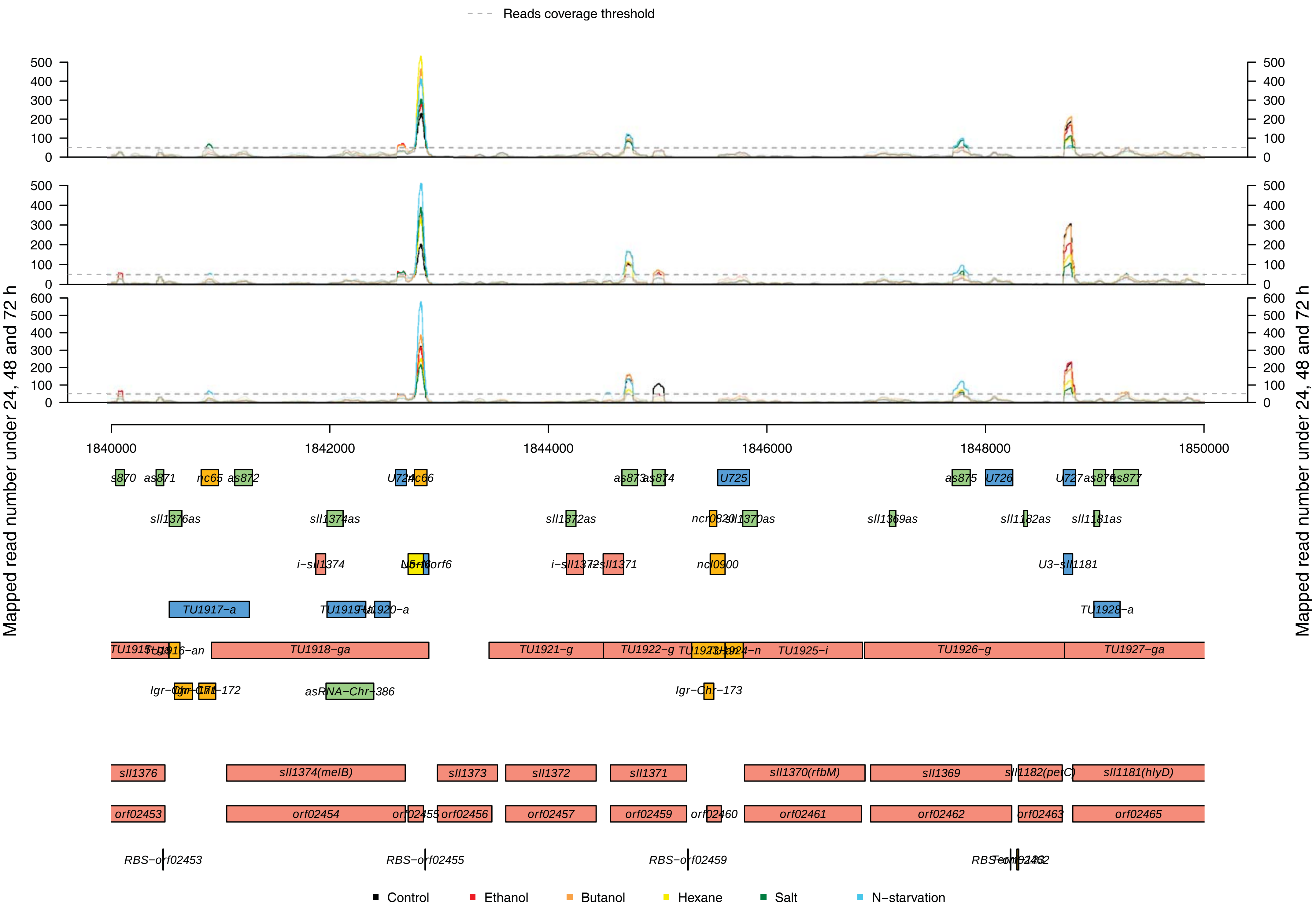

Reads coverage threshold
Mapped read number under 24, 48 and 72 h
1840000
1842000
1844000
1846000
1848000
1850000
TU1917−a
TU1918−ga
TU1921−g
TU1922−g
TU1925−i
TU1926−g
TU1927−ga
TU1928−a
asRNA−Chr−386
sll1376
sll1374(melB)
sll1373
sll1372
sll1371
sll1370(rfbM)
sll1369
sll1181(hlyD)
orf02453
orf02454
orf02456
orf02457
orf02459
orf02461
orf02462
orf02465
RBS−orf02453
RBS−orf02455
RBS−orf02459
Control
Ethanol
Butanol
Hexane
Salt
N−starvation

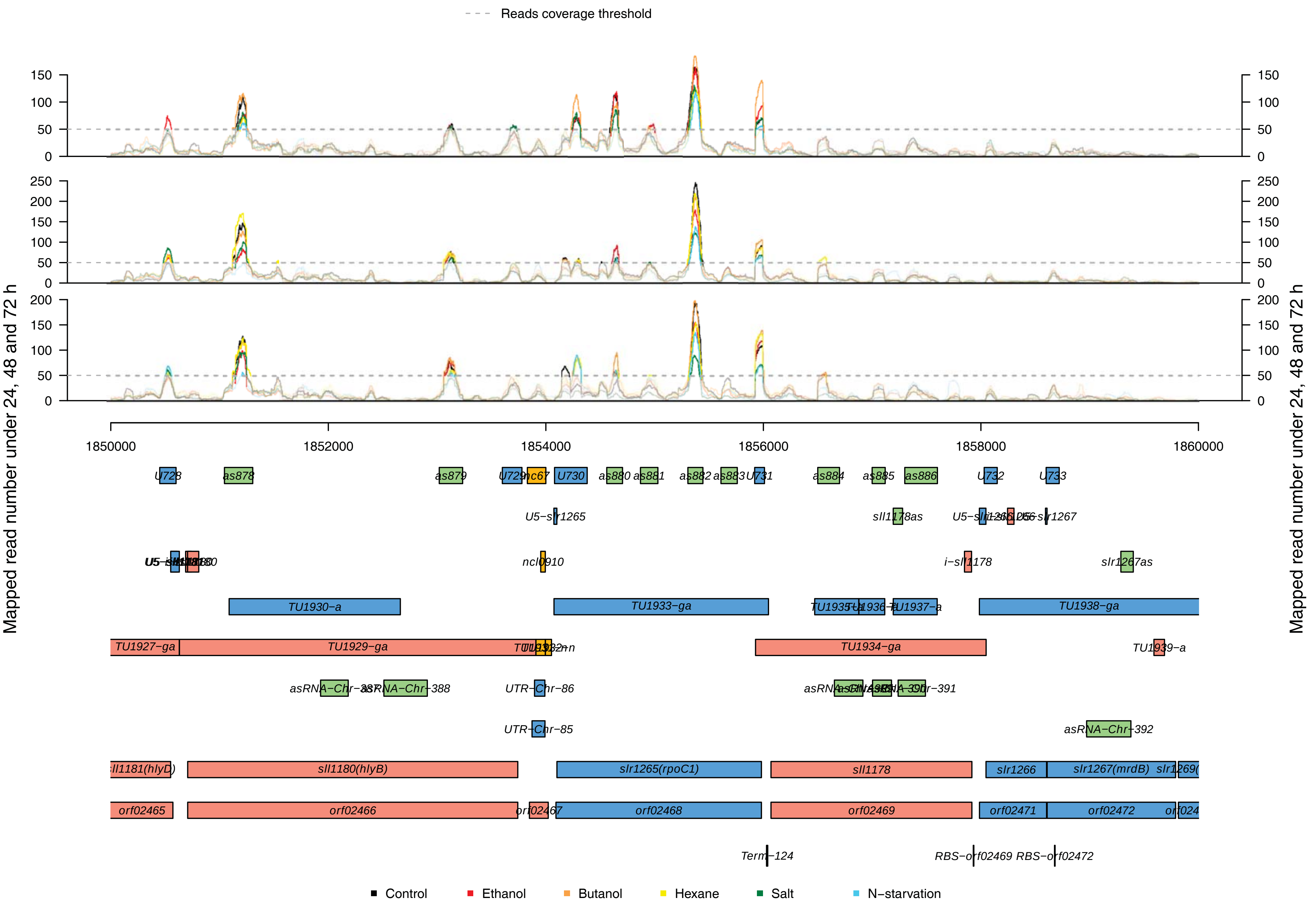

Reads coverage threshold
Mapped read number under 24, 48 and 72 h
1850000
1852000
1854000
1856000
1858000
1860000
U728
as878
as879
U729
nc67
U730
as880
as881
as882
as883
U731
as884
as885
as886
U732
U733
U5−slr1265
sll1178as
i−sll1178
slr1267as
ncl0910
TU1930−a
TU1933−ga
TU1938−ga
TU1927−ga
TU1929−ga
TU1934−ga
TU1939−a
asRNA−Chr−388
UTR−Chr−86
UTR−Chr−85
asRNA−Chr−391
asRNA−Chr−392
sll1180(hlyB)
slr1265(rpoC1)
sll1178
slr1266
slr1267(mrdB)
orf02465
orf02466
orf02468
orf02469
orf02471
orf02472
Term−124
RBS−orf02469
RBS−orf02472
Control
Ethanol
Butanol
Hexane
Salt
N−starvation

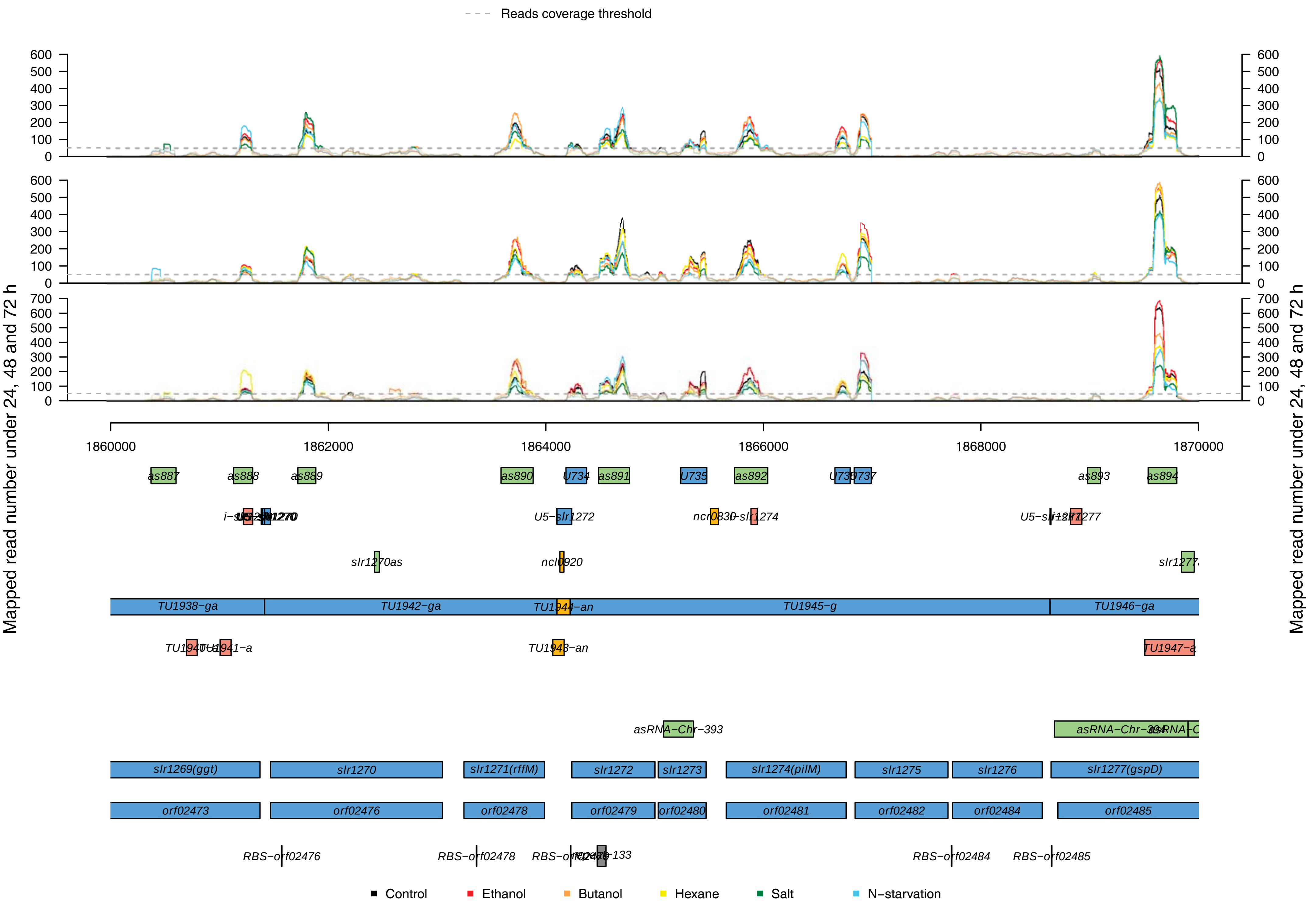

Reads coverage threshold
Mapped read number under 24, 48 and 72 h
0
100
200
300
400
500
600
700
1860000
1862000
1864000
1866000
1868000
1870000
as887
as888
as889
as890
U734
as891
U735
as892
as893
as894
U5-slr1272
slr1270as
ncl0920
TU1938-ga
TU1942-ga
TU1945-g
TU1946-ga
TU1947-a
asRNA-Chr-393
slr1269(ggt)
slr1270
slr1271(rffM)
slr1272
slr1273
slr1274(pilM)
slr1275
slr1276
slr1277(gspD)
orf02473
orf02476
orf02478
orf02479
orf02480
orf02481
orf02482
orf02484
orf02485
RBS-orf02476
RBS-orf02478
RBS-orf02484
RBS-orf02485
Control
Ethanol
Butanol
Hexane
Salt
N-starvation

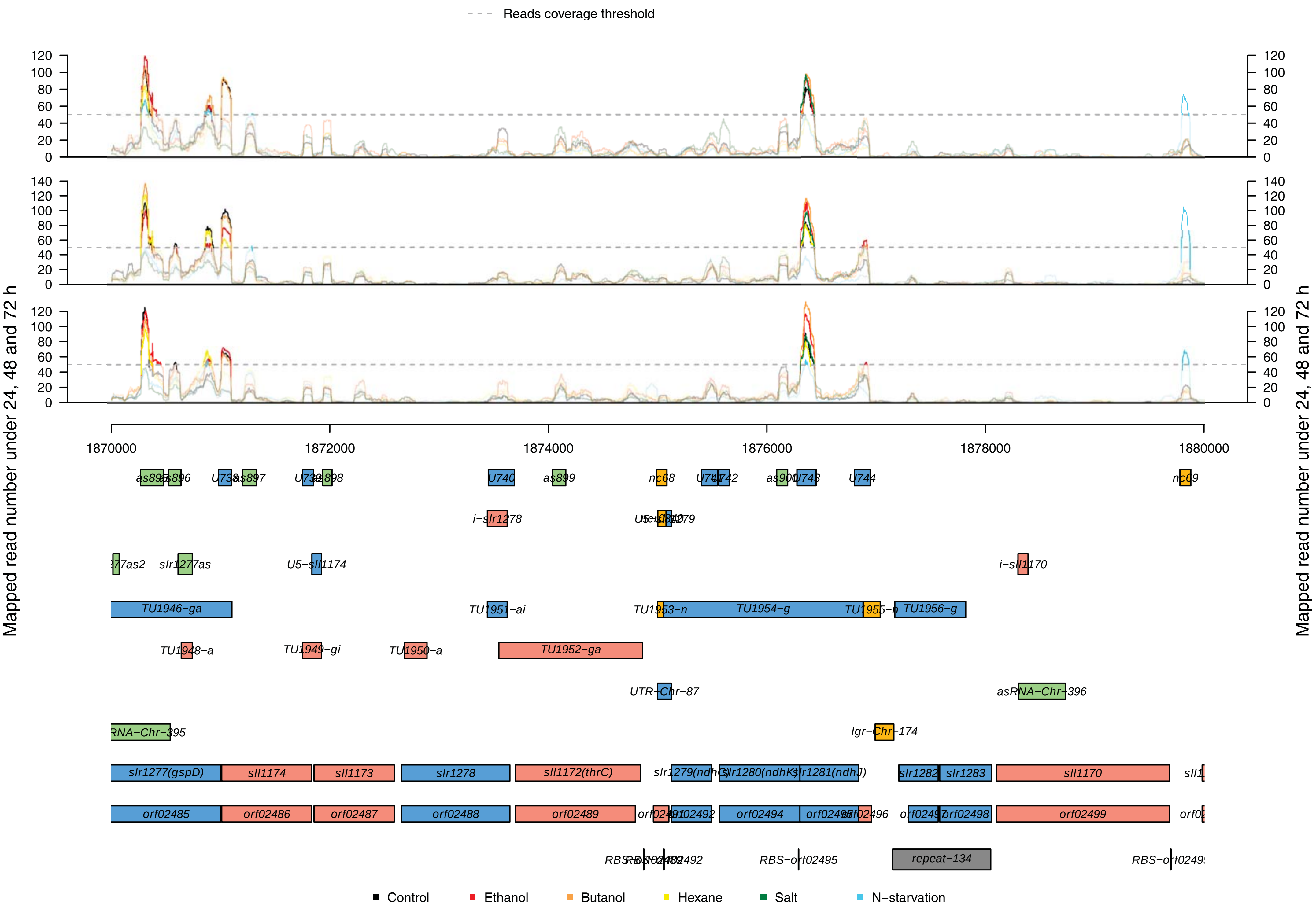

Reads coverage threshold
Mapped read number under 24, 48 and 72 h
1870000
1872000
1874000
1876000
1878000
1880000
TU1946−ga
TU1948−a
TU1949−gi
TU1950−a
TU1951−ai
TU1952−ga
TU1954−g
TU1956−g
UTR−Chr−87
asRNA−Chr−396
Igr−Chr−174
slr1277(gspD)
sll1174
sll1173
slr1278
sll1172(thrC)
sll1170
orf02485
orf02486
orf02487
orf02488
orf02489
orf02494
orf02499
RBS−orf02495
repeat−134
Control
Ethanol
Butanol
Hexane
Salt
N−starvation

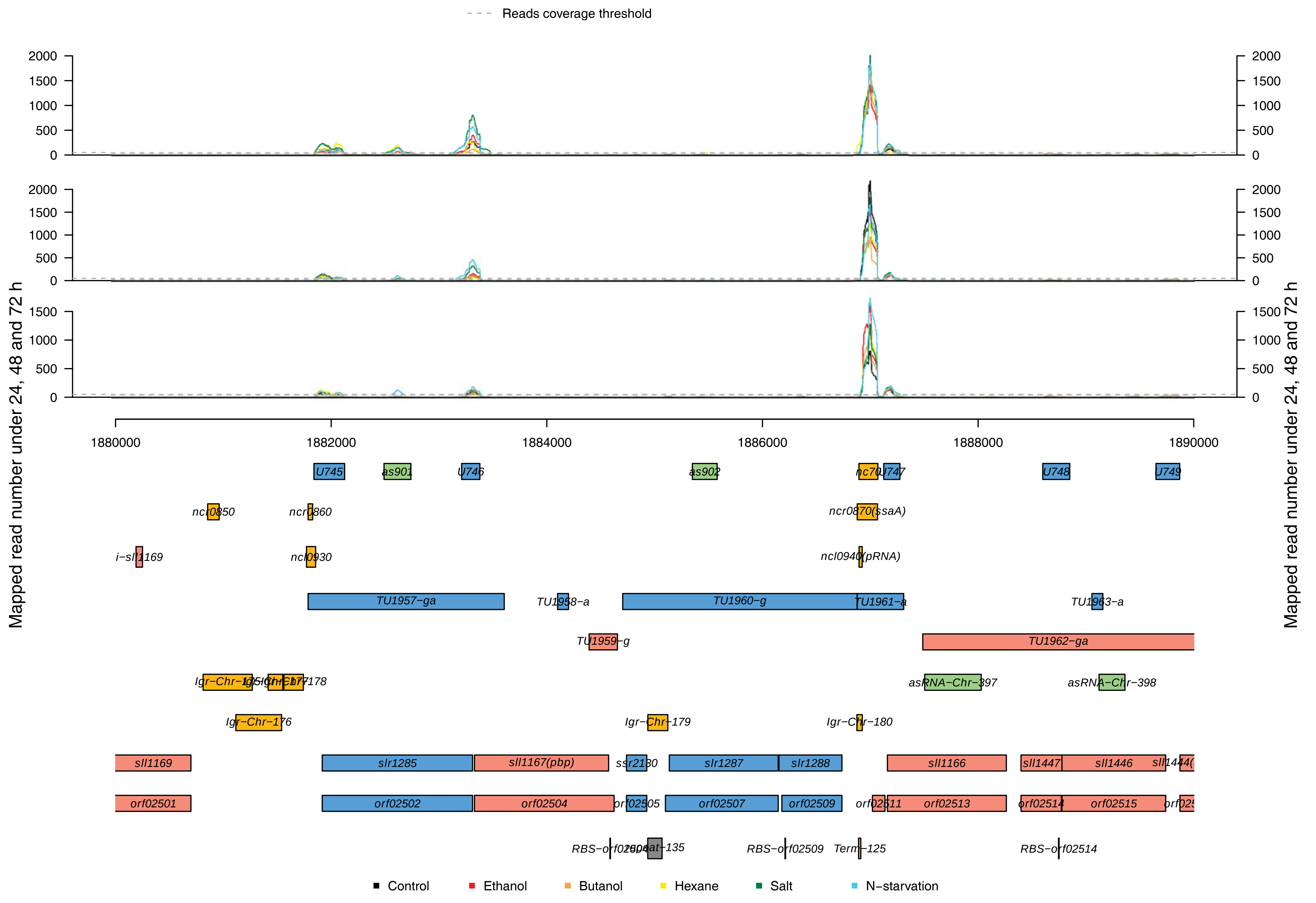

Reads coverage threshold
Mapped read number under 24, 48 and 72 h
1880000
1882000
1884000
1886000
1888000
1890000
U745
as901
U746
as902
U747
U748
U749
ncr0850
ncr0860
ncr0870(ssaA)
i-sll1169
ncl0930
ncl0940(pRNA)
TU1957-ga
TU1958-a
TU1960-g
TU1961-a
TU1963-a
TU1959-g
TU1962-ga
asRNA-Chr-397
asRNA-Chr-398
Igr-Chr-176
Igr-Chr-179
Igr-Chr-180
sll1169
slr1285
sll1167(pbp)
ssr2130
slr1287
slr1288
sll1166
sll1447
sll1446
orf02501
orf02502
orf02504
orf02505
orf02507
orf02509
orf02513
orf02514
orf02515
RBS-orf02509
Term-125
RBS-orf02514
Control
Ethanol
Butanol
Hexane
Salt
N-starvation

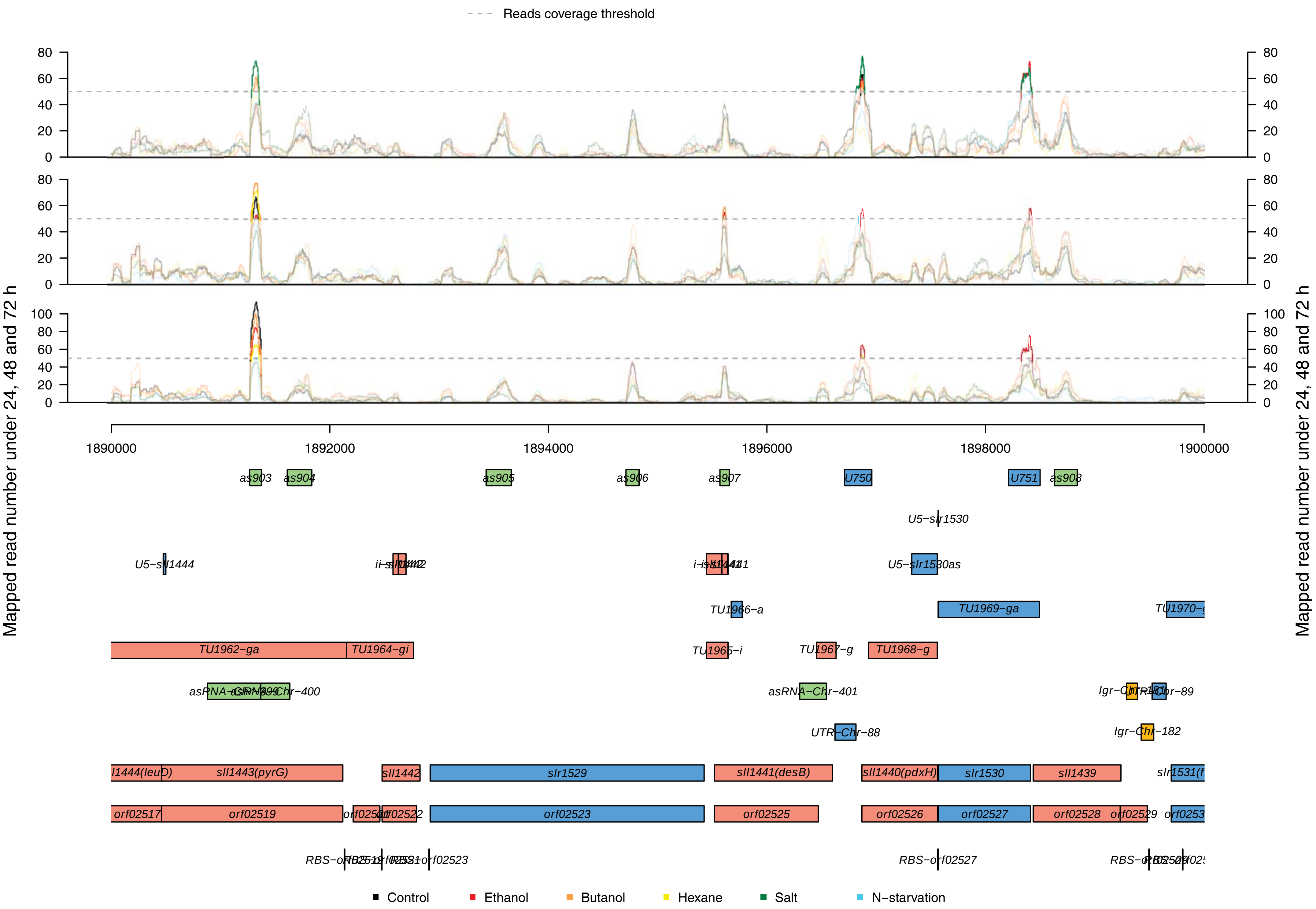

Reads coverage threshold
Mapped read number under 24, 48 and 72 h
1890000
1892000
1894000
1896000
1898000
1900000
as903
as904
as905
as906
as907
U750
U751
as908
U5−slr1530
U5−sll1444
U5−slr1530as
TU1966−a
TU1969−ga
TU1962−ga
TU1964−gi
TU1965−i
TU1967−g
TU1968−g
asRNA−Chr−401
UTR−Chr−88
Igr−Chr−182
sll1443(pyrG)
sll1442
slr1529
sll1441(desB)
sll1440(pdxH)
slr1530
sll1439
orf02517
orf02519
orf02523
orf02525
orf02526
orf02527
orf02528
RBS−orf02527
Control
Ethanol
Butanol
Hexane
Salt
N−starvation

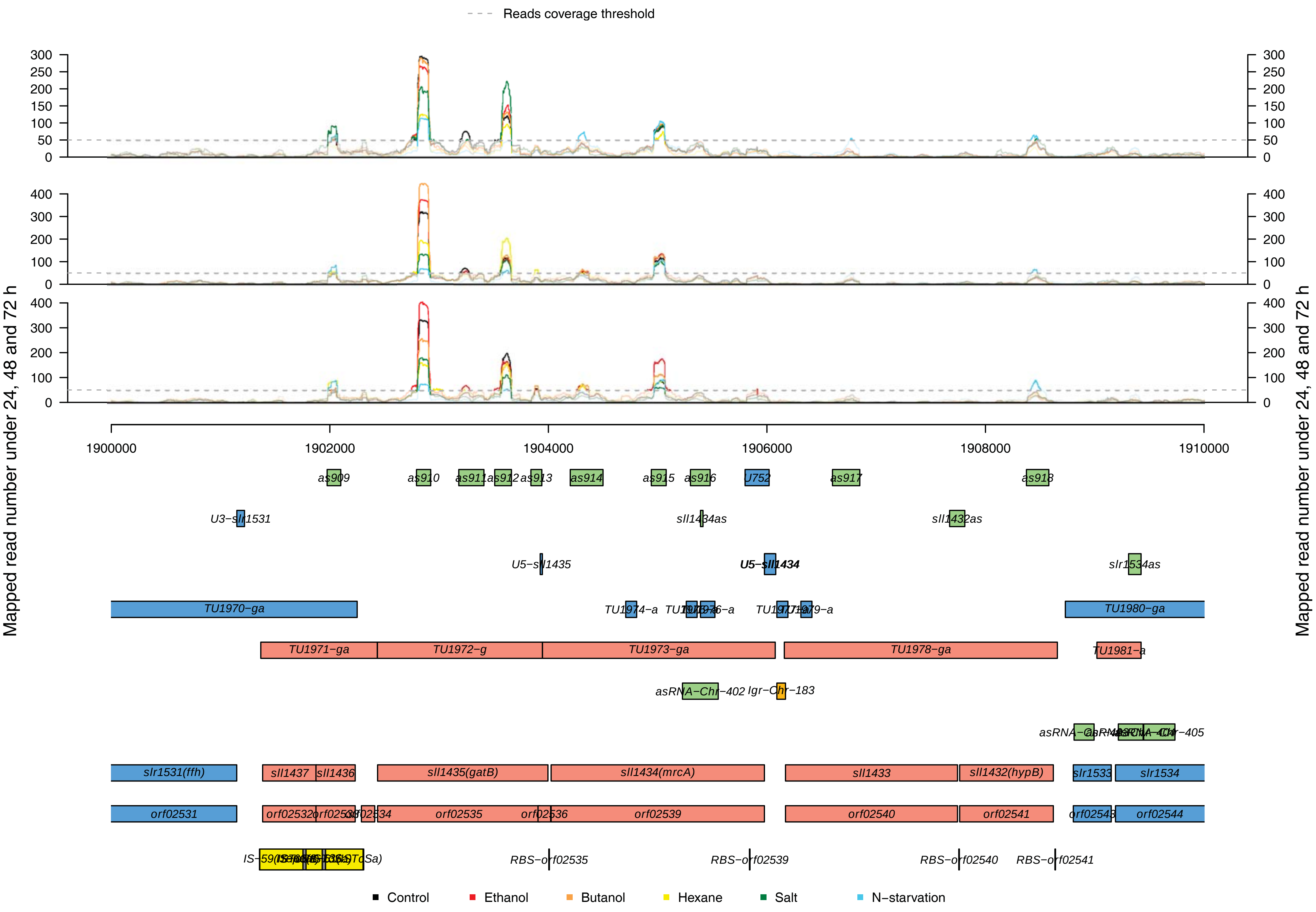

Reads coverage threshold
Mapped read number under 24, 48 and 72 h
1900000
1902000
1904000
1906000
1908000
1910000
TU1970−ga
TU1971−ga
TU1972−g
TU1973−ga
TU1978−ga
TU1980−ga
slr1531(ffh)
sll1437
sll1436
sll1435(gatB)
sll1434(mrcA)
sll1433
sll1432(hypB)
slr1533
slr1534
orf02531
orf02535
orf02539
orf02540
orf02541
orf02544
RBS−orf02535
RBS−orf02539
RBS−orf02540
RBS−orf02541
Control
Ethanol
Butanol
Hexane
Salt
N−starvation

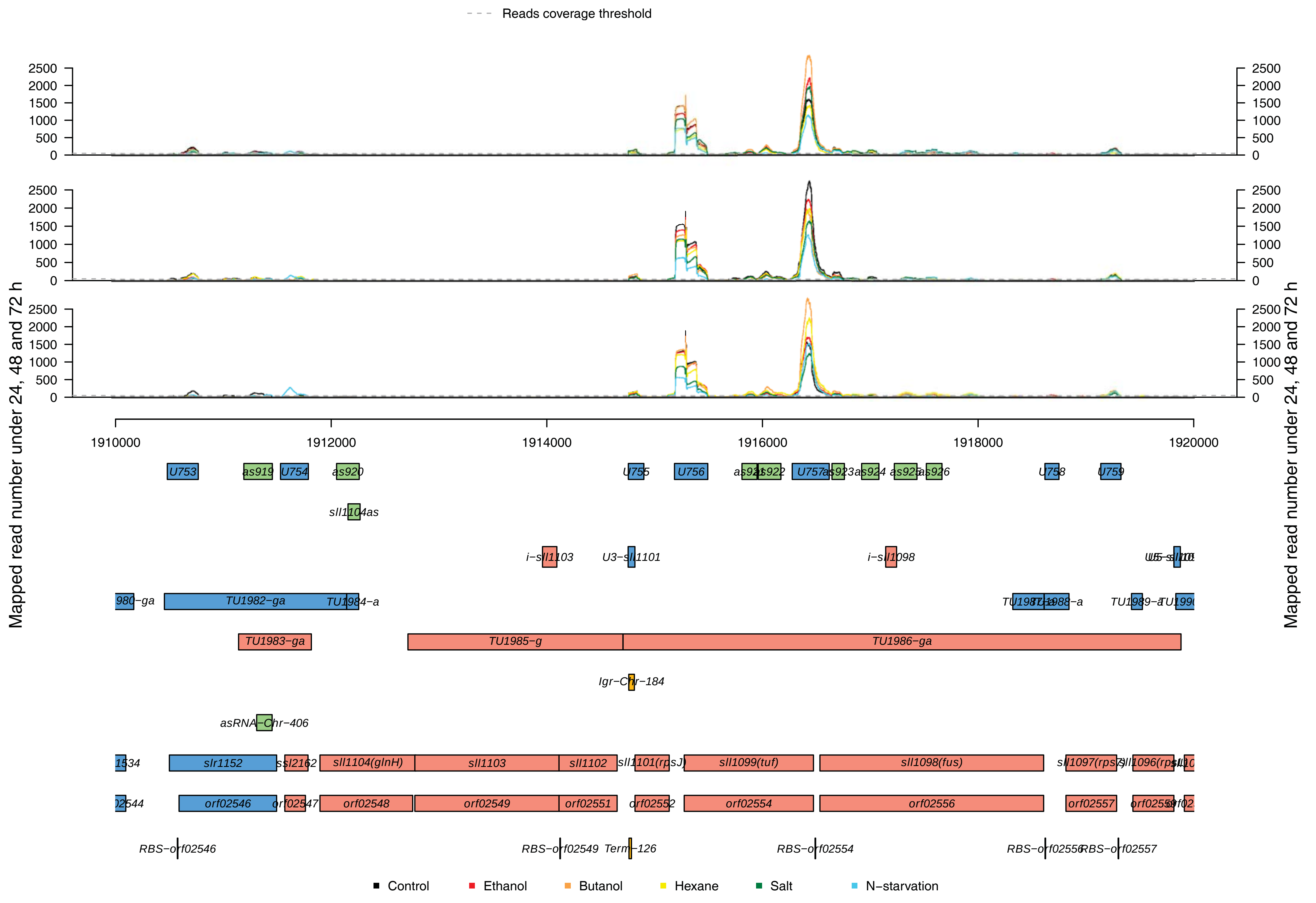

Reads coverage threshold
Mapped read number under 24, 48 and 72 h
1910000
1912000
1914000
1916000
1918000
1920000
U753
as919
U754
as920
U755
U756
U757
U758
U759
sll1104as
i−sll1103
U3−sll1101
i−sll1098
TU1982−ga
TU1984−a
TU1983−ga
TU1985−g
TU1986−ga
Igr−Chr−184
asRNA−Chr−406
slr1152
ssl2162
sll1104(glnH)
sll1103
sll1102
sll1101(rpsJ)
sll1099(tuf)
sll1098(fus)
sll1097(rps7)
orf02546
orf02547
orf02548
orf02549
orf02551
orf02552
orf02554
orf02556
orf02557
RBS−orf02546
RBS−orf02549
Term−126
RBS−orf02554
RBS−orf02556
RBS−orf02557
Control
Ethanol
Butanol
Hexane
Salt
N−starvation

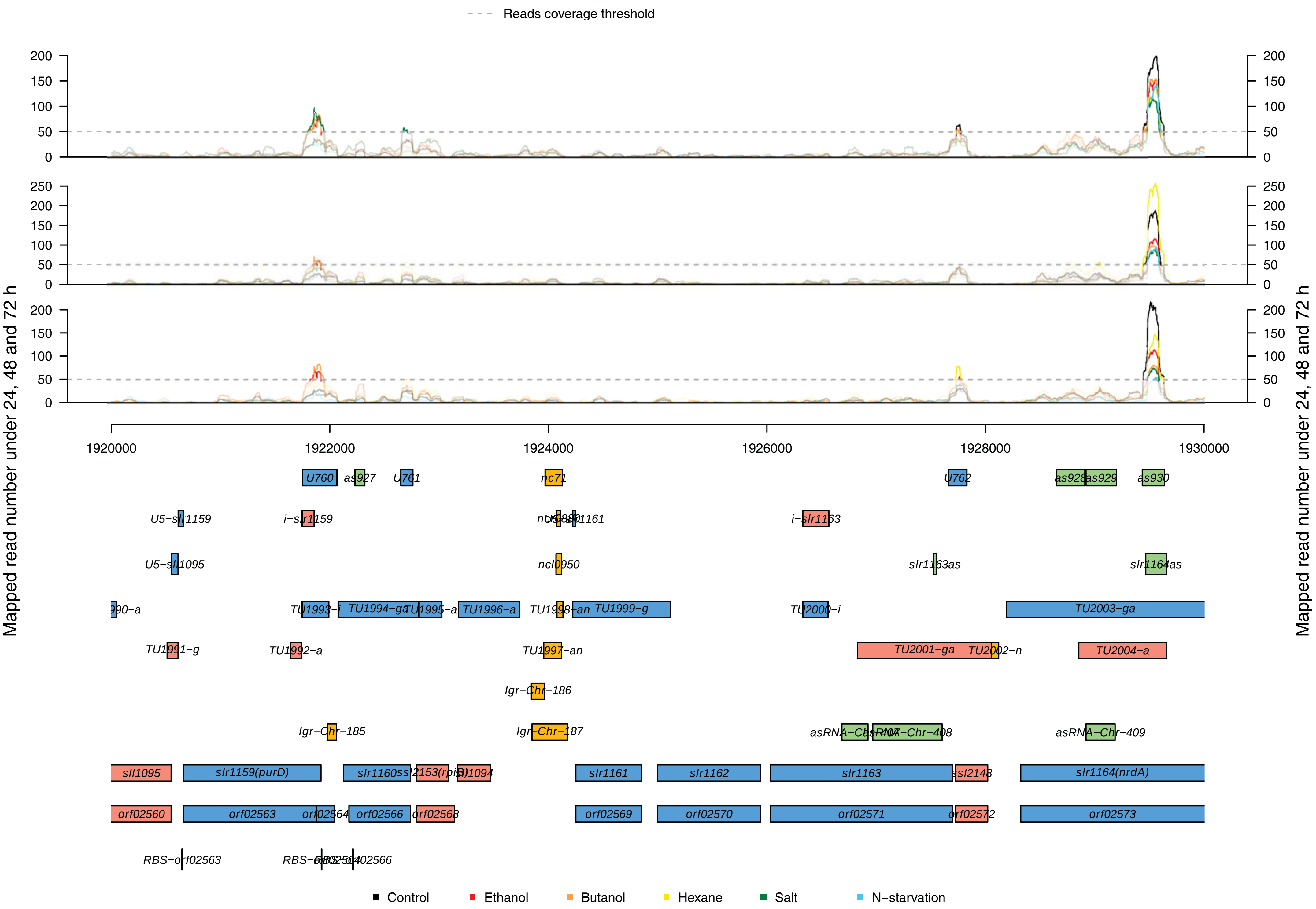

Reads coverage threshold
Mapped read number under 24, 48 and 72 h
1920000
1922000
1924000
1926000
1928000
1930000
U760
as927
U761
nc71
U762
as928
as929
as930
U5−slr1159
i−slr1159
i−slr1163
U5−sll1095
ncl0950
slr1163as
slr1164as
TU1991−g
TU1992−a
TU1993−i
TU1994−ga
TU1995−a
TU1996−a
TU1997−an
TU1998−an
TU1999−g
TU2000−i
TU2001−ga
TU2002−n
TU2003−ga
TU2004−a
Igr−Chr−185
Igr−Chr−186
Igr−Chr−187
asRNA−Chr−408
asRNA−Chr−409
sll1095
slr1159(purD)
slr1161
slr1162
slr1163
ssl2148
slr1164(nrdA)
orf02560
orf02563
orf02566
orf02569
orf02570
orf02571
orf02572
orf02573
RBS−orf02563
Control
Ethanol
Butanol
Hexane
Salt
N−starvation

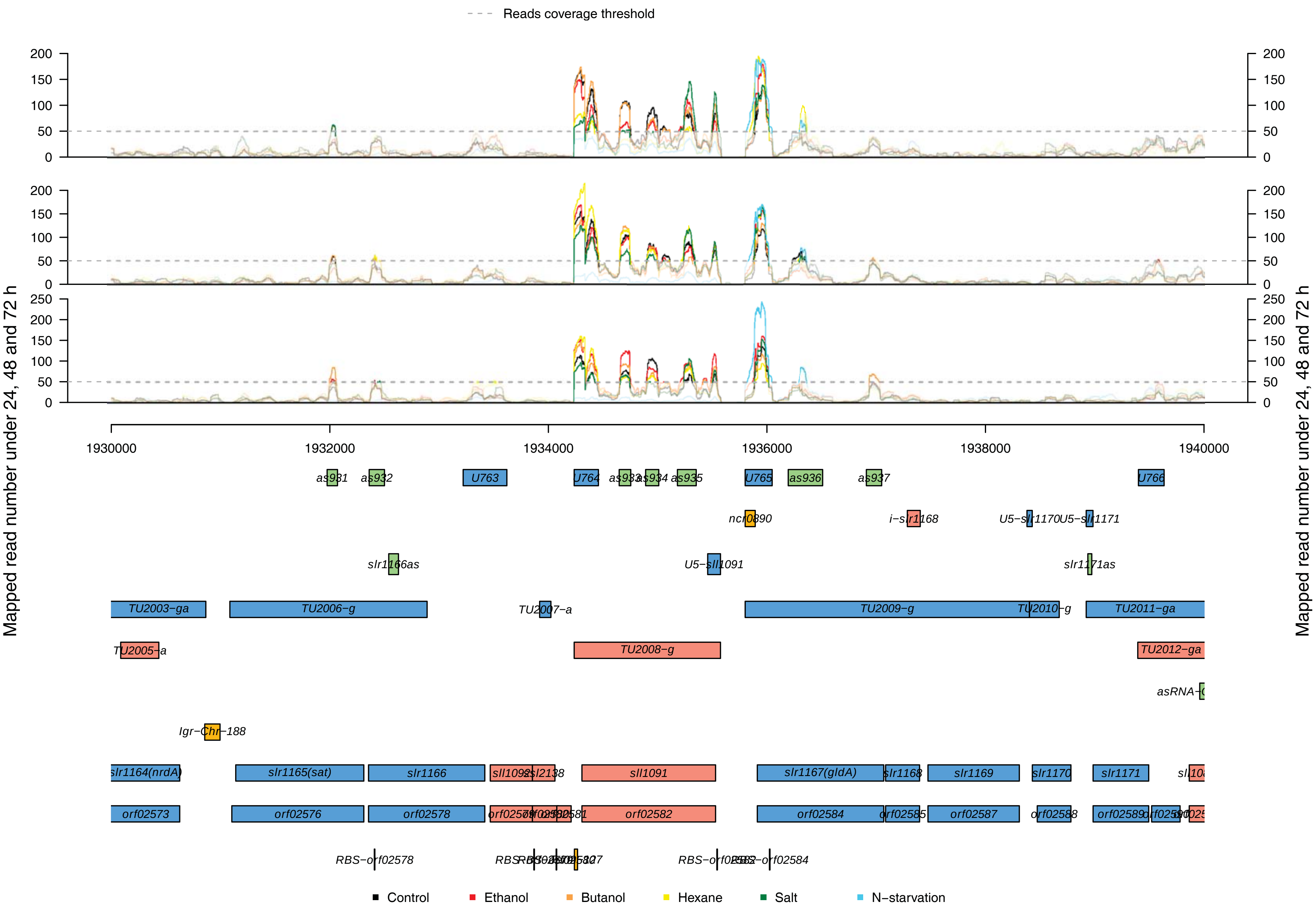

Reads coverage threshold
Mapped read number under 24, 48 and 72 h
1930000
1932000
1934000
1936000
1938000
1940000
as931
as932
U763
U764
as933
as934
as935
U765
as936
as937
U766
ncl0890
i-slr1168
U5-slr1170
U5-slr1171
slr1166as
U5-sll1091
slr1171as
TU2003-ga
TU2006-g
TU2007-a
TU2009-g
TU2010-g
TU2011-ga
TU2005-a
TU2008-g
TU2012-ga
Igr-Chr-188
slr1164(nrdA)
slr1165(sat)
slr1166
sll2138
sll1091
slr1167(gldA)
slr1168
slr1169
slr1170
slr1171
orf02573
orf02576
orf02578
orf02582
orf02584
orf02585
orf02587
orf02588
orf02589
RBS-orf02578
Control
Ethanol
Butanol
Hexane
Salt
N-starvation

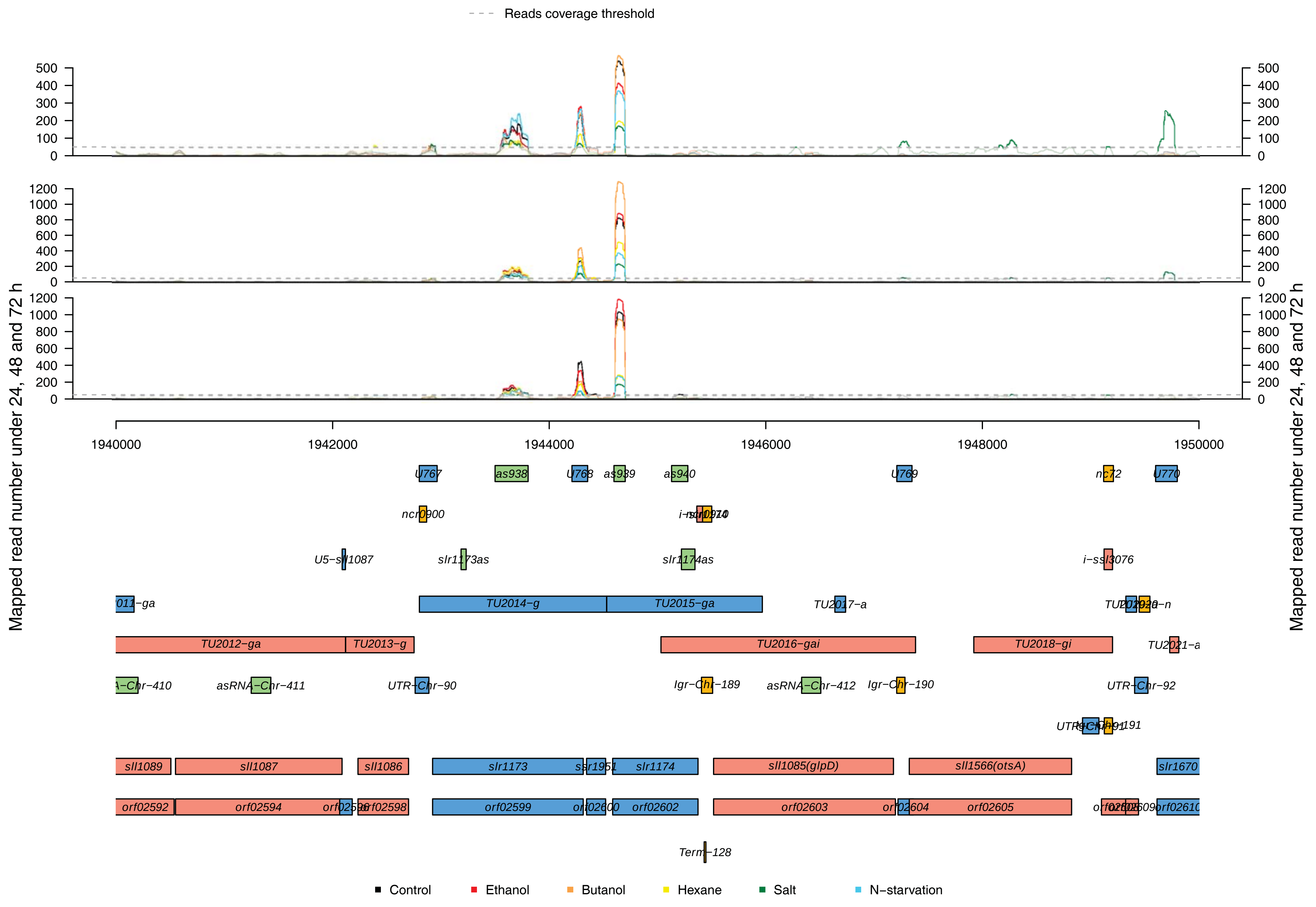

Reads coverage threshold
Mapped read number under 24, 48 and 72 h
1940000
1942000
1944000
1946000
1948000
1950000
U767
as938
U768
as939
as940
U769
nc72
U770
ncr0900
U5−sll1087
slr1173as
slr1174as
i−ssl3076
TU2014−g
TU2015−ga
TU2017−a
TU2012−ga
TU2013−g
TU2016−gai
TU2018−gi
asRNA−Chr−411
UTR−Chr−90
Igr−Chr−189
asRNA−Chr−412
Igr−Chr−190
UTR−Chr−92
sll1089
sll1087
sll1086
slr1173
slr1174
sll1085(glpD)
sll1566(otsA)
orf02592
orf02594
orf02598
orf02599
orf02602
orf02603
orf02605
Term−128
Control
Ethanol
Butanol
Hexane
Salt
N−starvation

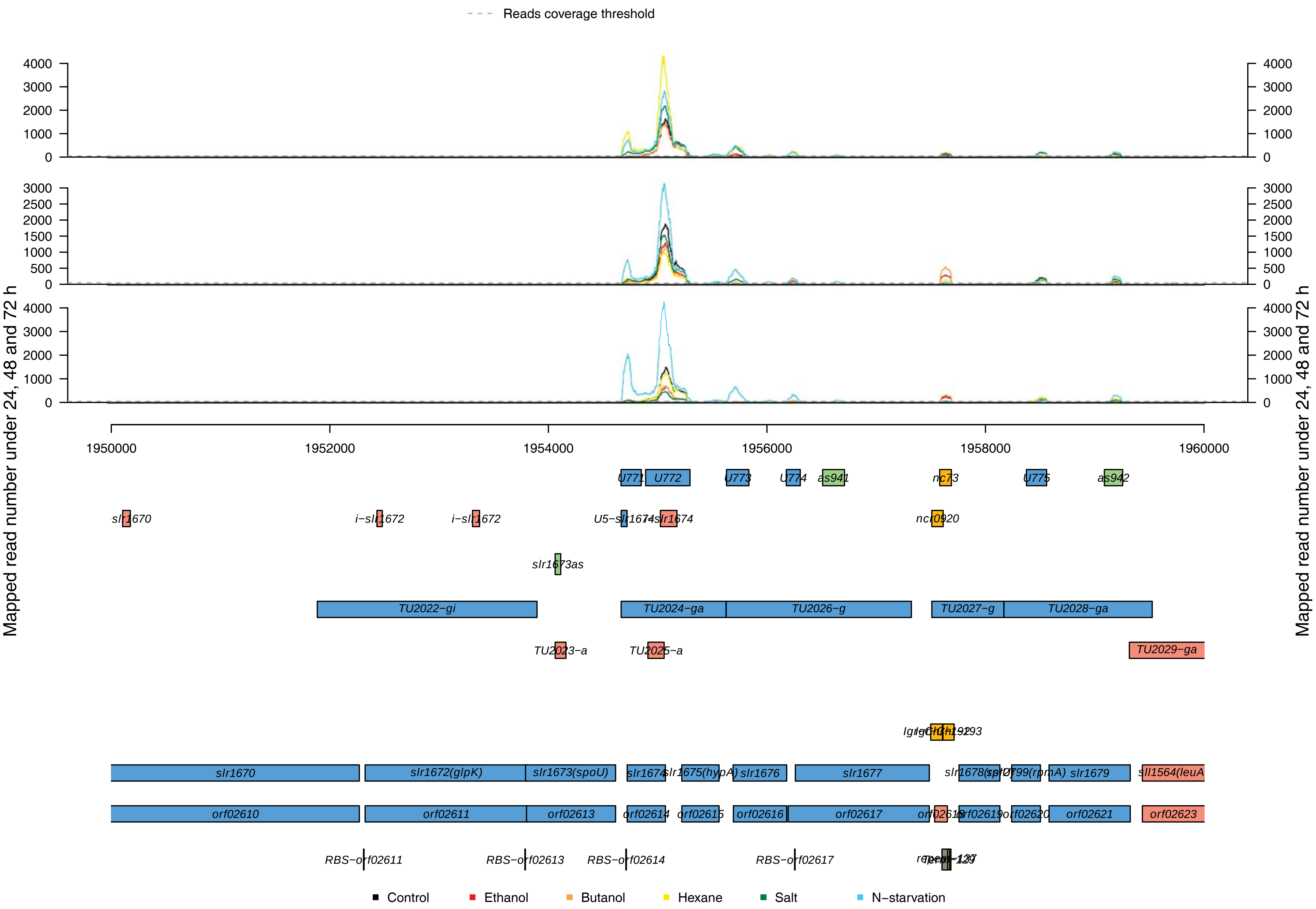

Reads coverage threshold
Mapped read number under 24, 48 and 72 h
1950000
1952000
1954000
1956000
1958000
1960000
U771
U772
U773
U774
as941
nc73
U775
as942
slr1670
i−slr1672
i−slr1672
U5−slr1674
as−slr1674
ncl0920
slr1673as
TU2022−gi
TU2024−ga
TU2026−g
TU2027−g
TU2028−ga
TU2023−a
TU2025−a
TU2029−ga
slr1670
slr1672(glpK)
slr1673(spoU)
slr1674
slr1675(hypA)
slr1676
slr1677
slr1679
sll1564(leuA
orf02610
orf02611
orf02613
orf02614
orf02615
orf02616
orf02617
orf02621
orf02623
RBS−orf02611
RBS−orf02613
RBS−orf02614
RBS−orf02617
Control
Ethanol
Butanol
Hexane
Salt
N−starvation

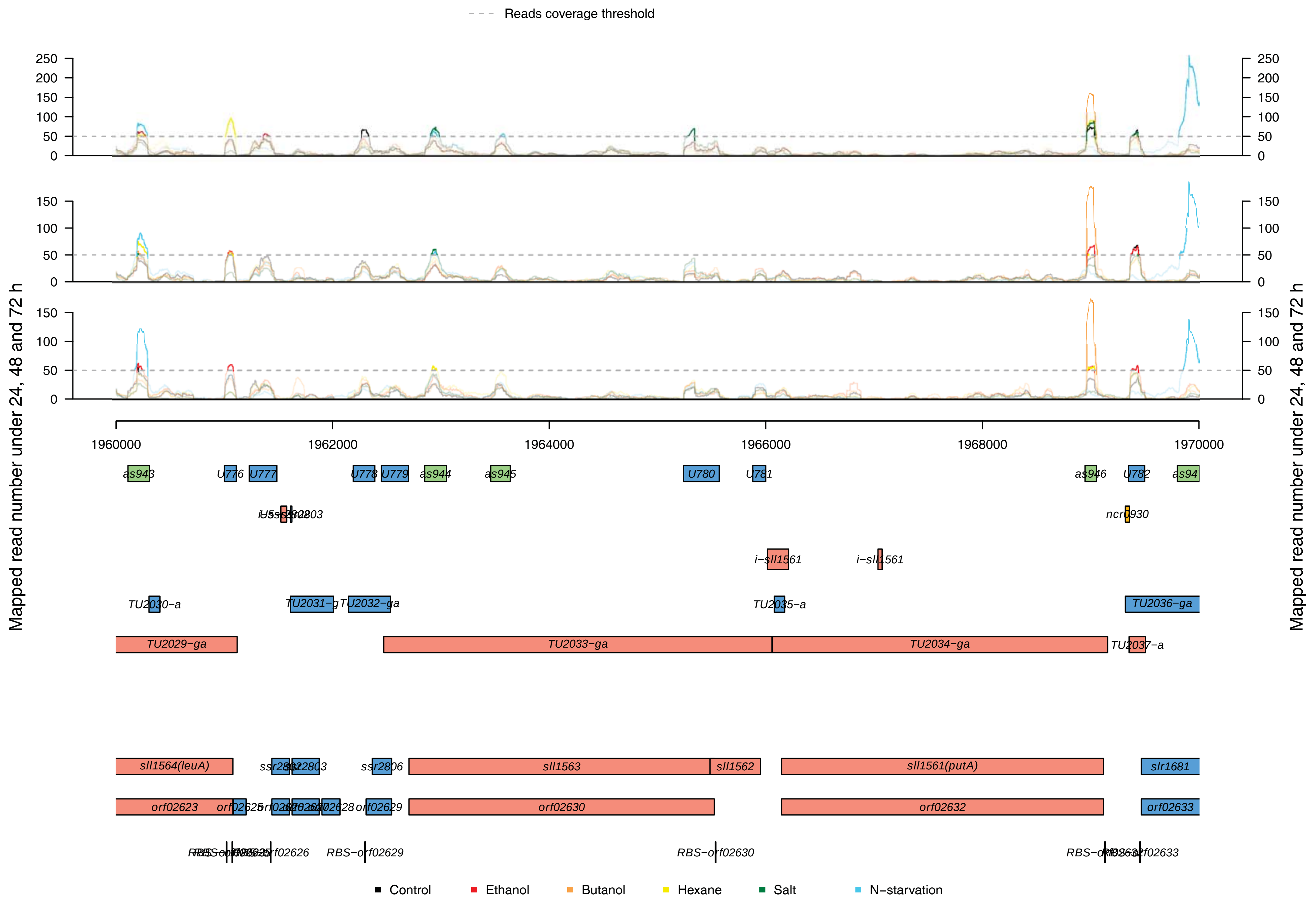

Reads coverage threshold
Mapped read number under 24, 48 and 72 h
1960000
1962000
1964000
1966000
1968000
1970000
as943
U776
U777
U778
U779
as944
as945
U780
U781
as946
U782
ncr0930
i-sll1561
TU2030-a
TU2031-g
TU2032-ga
TU2035-a
TU2036-ga
TU2029-ga
TU2033-ga
TU2034-ga
TU2037-a
sll1564(leuA)
ssr2806
sll1563
sll1562
sll1561(putA)
slr1681
orf02623
orf02629
orf02630
orf02632
orf02633
RBS-orf02629
RBS-orf02630
Control
Ethanol
Butanol
Hexane
Salt
N-starvation

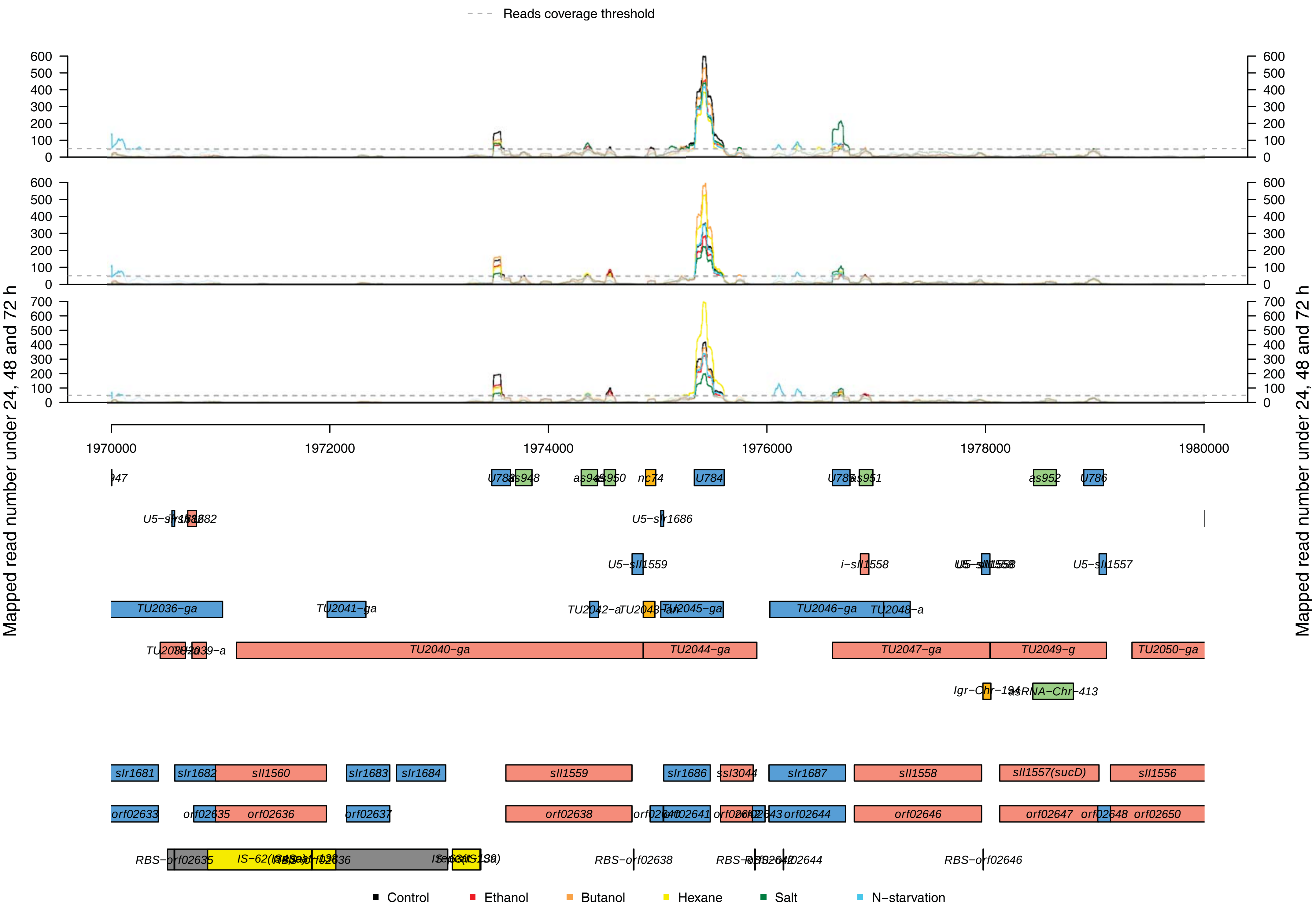

Reads coverage threshold
Mapped read number under 24, 48 and 72 h
1970000
1972000
1974000
1976000
1978000
1980000
U784
as952
U786
U5−sll1559
i−sll1558
U5−sll1557
TU2036−ga
TU2041−ga
TU2046−ga
TU2040−ga
TU2044−ga
TU2047−ga
TU2049−g
TU2050−ga
slr1681
slr1682
sll1560
slr1683
slr1684
sll1559
slr1686
ssl3044
slr1687
sll1558
sll1557(sucD)
sll1556
orf02633
orf02636
orf02637
orf02638
orf02644
orf02646
orf02647
orf02650
RBS−orf02638
RBS−orf02646
Control
Ethanol
Butanol
Hexane
Salt
N−starvation

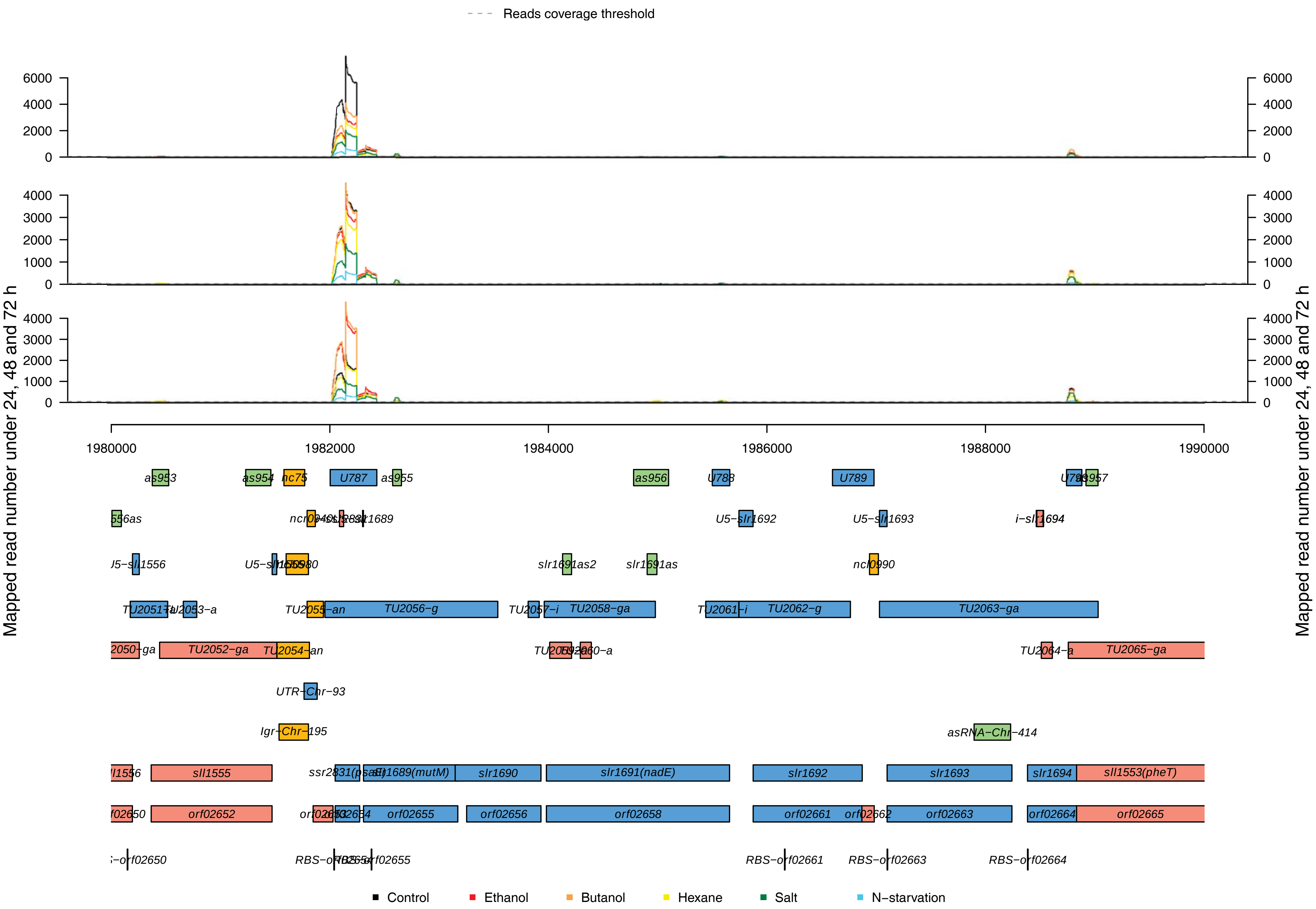

Reads coverage threshold
Mapped read number under 24, 48 and 72 h
6000
4000
2000
0
3000
1000
1980000
1982000
1984000
1986000
1988000
1990000
as953
as954
U787
as955
as956
U789
U5−slr1692
U5−slr1693
i−slr1694
slr1691as2
slr1691as
ncl0990
TU2056−g
TU2058−ga
TU2062−g
TU2063−ga
TU2052−ga
TU2065−ga
UTR−Chr−93
Igr−Chr−195
asRNA−Chr−414
sll1555
slr1690
slr1691(nadE)
slr1692
slr1693
slr1694
sll1553(pheT)
orf02652
orf02655
orf02656
orf02658
orf02661
orf02663
orf02664
orf02665
RBS−orf02661
RBS−orf02663
RBS−orf02664
Control
Ethanol
Butanol
Hexane
Salt
N−starvation

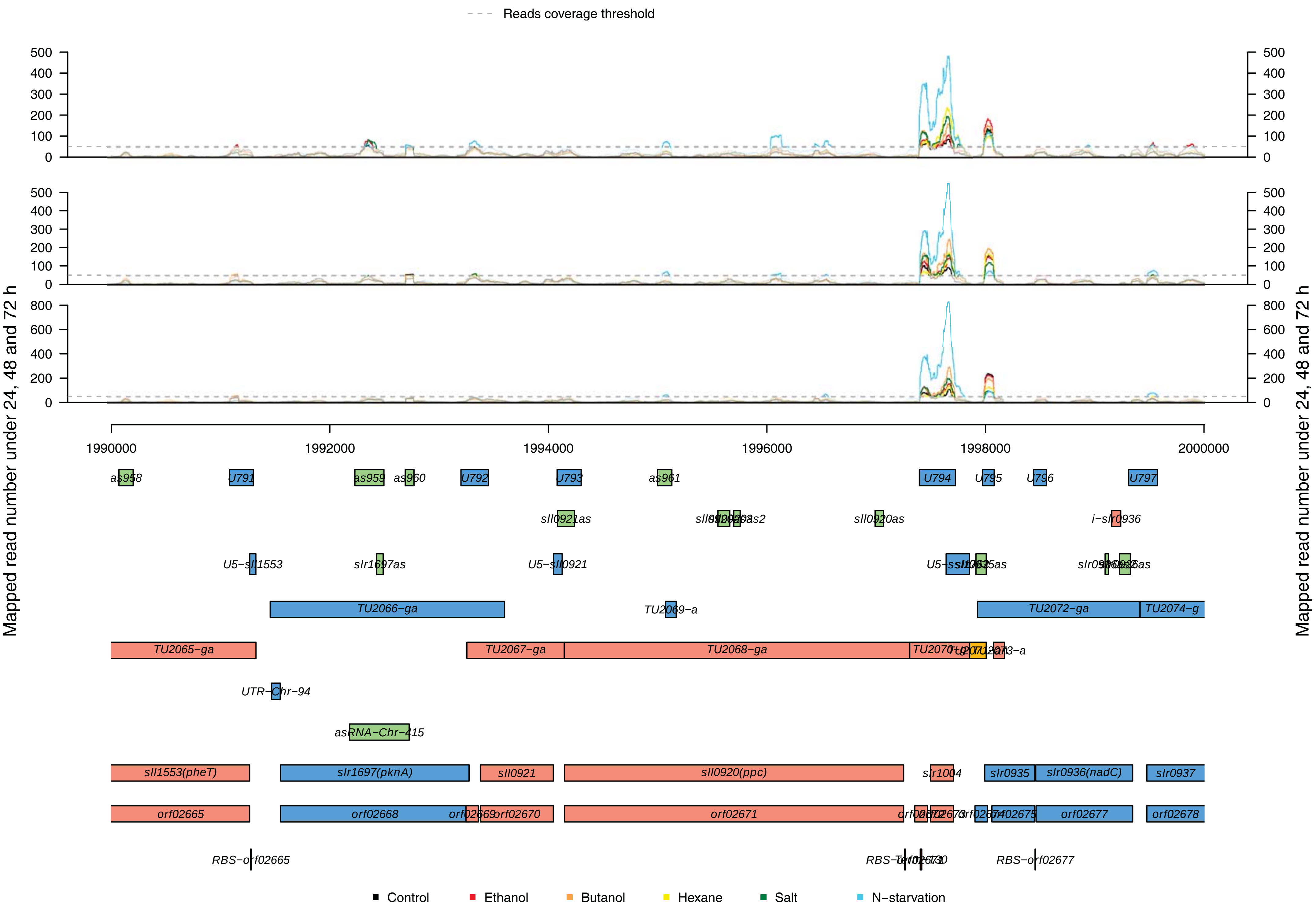

Reads coverage threshold
Mapped read number under 24, 48 and 72 h
1990000
1992000
1994000
1996000
1998000
2000000
as958
U791
as959
as960
U792
U793
as961
U794
U795
U796
U797
sll0921as
sll0920as
i−slr0936
U5−sll1553
slr1697as
U5−sll0921
TU2066−ga
TU2069−a
TU2072−ga
TU2074−g
TU2065−ga
TU2067−ga
TU2068−ga
UTR−Chr−94
asRNA−Chr−415
sll1553(pheT)
slr1697(pknA)
sll0921
sll0920(ppc)
slr1004
slr0935
slr0936(nadC)
slr0937
orf02665
orf02668
orf02670
orf02671
orf02677
orf02678
RBS−orf02665
RBS−orf02677
Control
Ethanol
Butanol
Hexane
Salt
N−starvation

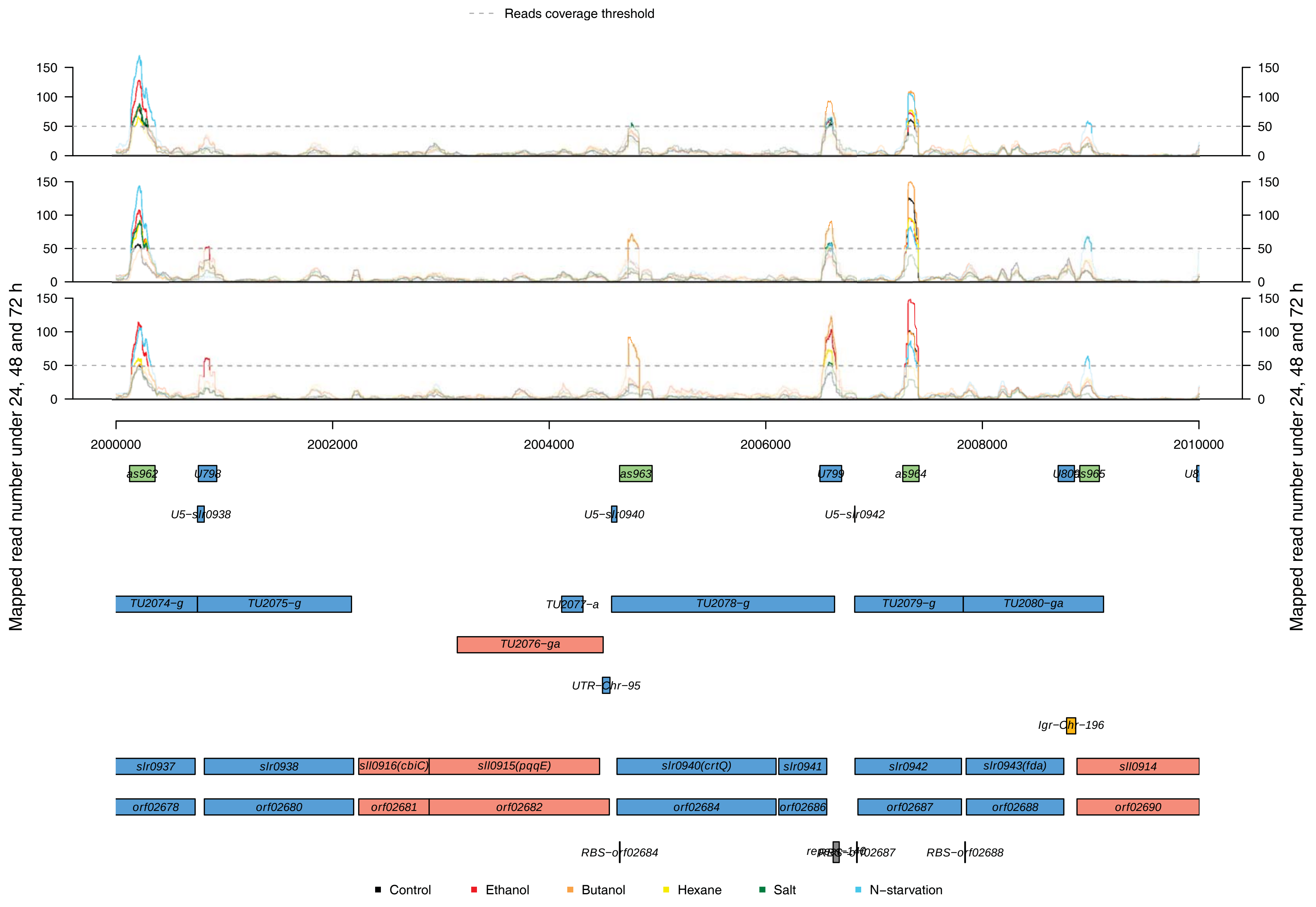

Reads coverage threshold
Mapped read number under 24, 48 and 72 h
150
100
50
0
2000000
2002000
2004000
2006000
2008000
2010000
as962
U798
as963
U799
as964
U800
as965
U5−slr0938
U5−slr0940
U5−slr0942
TU2074−g
TU2075−g
TU2077−a
TU2078−g
TU2079−g
TU2080−ga
TU2076−ga
UTR−Chr−95
Igr−Chr−196
slr0937
slr0938
sll0916(cbiC)
sll0915(pqqE)
slr0940(crtQ)
slr0941
slr0942
slr0943(fda)
sll0914
orf02678
orf02680
orf02681
orf02682
orf02684
orf02686
orf02687
orf02688
orf02690
RBS−orf02684
RBS−orf02687
RBS−orf02688
Control
Ethanol
Butanol
Hexane
Salt
N−starvation

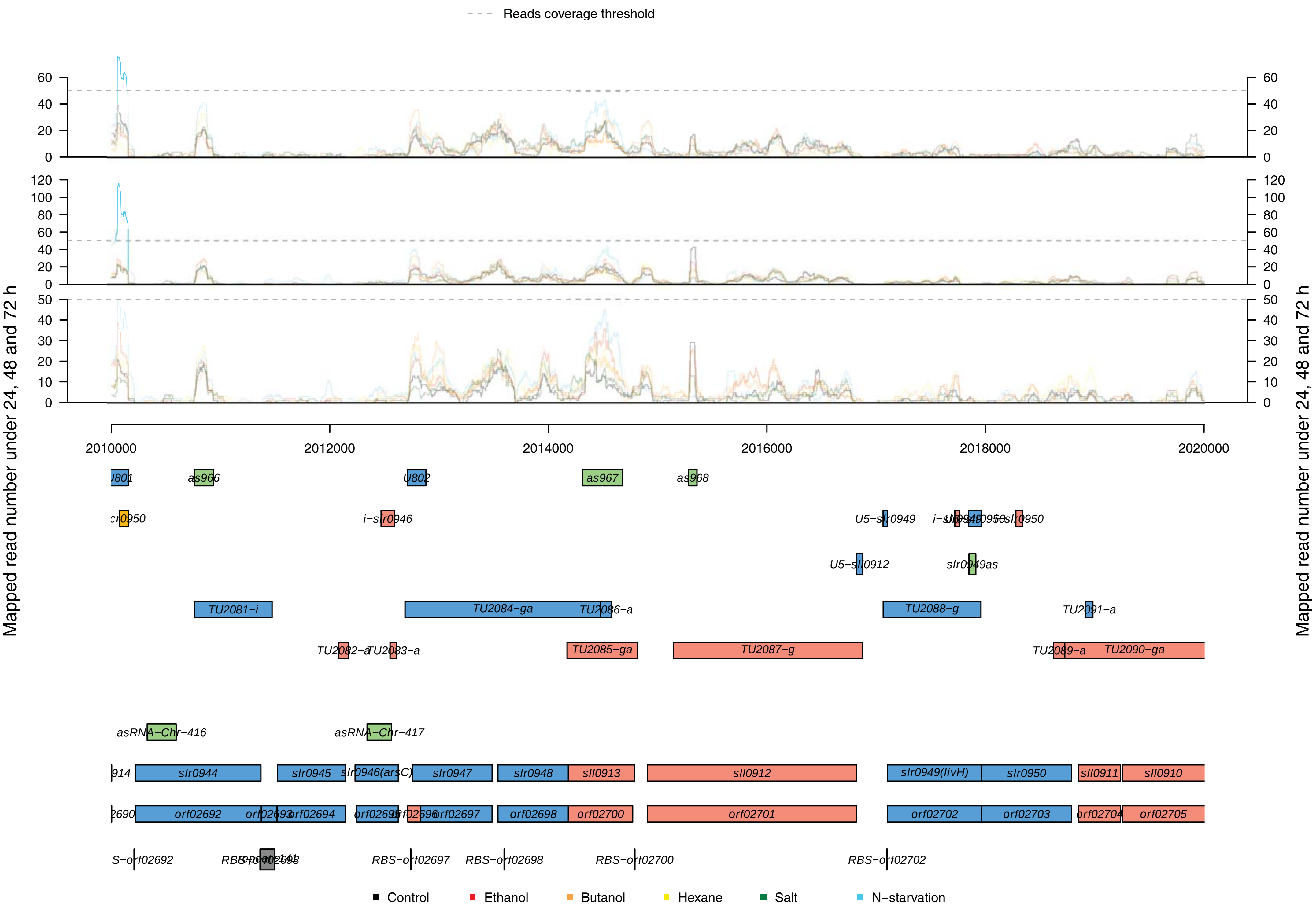
Reads coverage threshold
Mapped read number under 24, 48 and 72 h
2010000
2012000
2014000
2016000
2018000
2020000
as966
U802
as967
as968
i−slr0946
U5−slr0949
U5−sll0912
slr0949as
TU2081−i
TU2084−ga
TU2086−a
TU2088−g
TU2091−a
TU2082−a
TU2083−a
TU2085−ga
TU2087−g
TU2089−a
TU2090−ga
asRNA−Chr−416
asRNA−Chr−417
slr0944
slr0945
slr0946(arsC)
slr0947
slr0948
sll0913
sll0912
slr0949(livH)
slr0950
sll0911
sll0910
orf02692
orf02694
orf02697
orf02698
orf02700
orf02701
orf02702
orf02703
orf02704
orf02705
RBS−orf02697
RBS−orf02698
RBS−orf02700
RBS−orf02702
Control
Ethanol
Butanol
Hexane
Salt
N−starvation

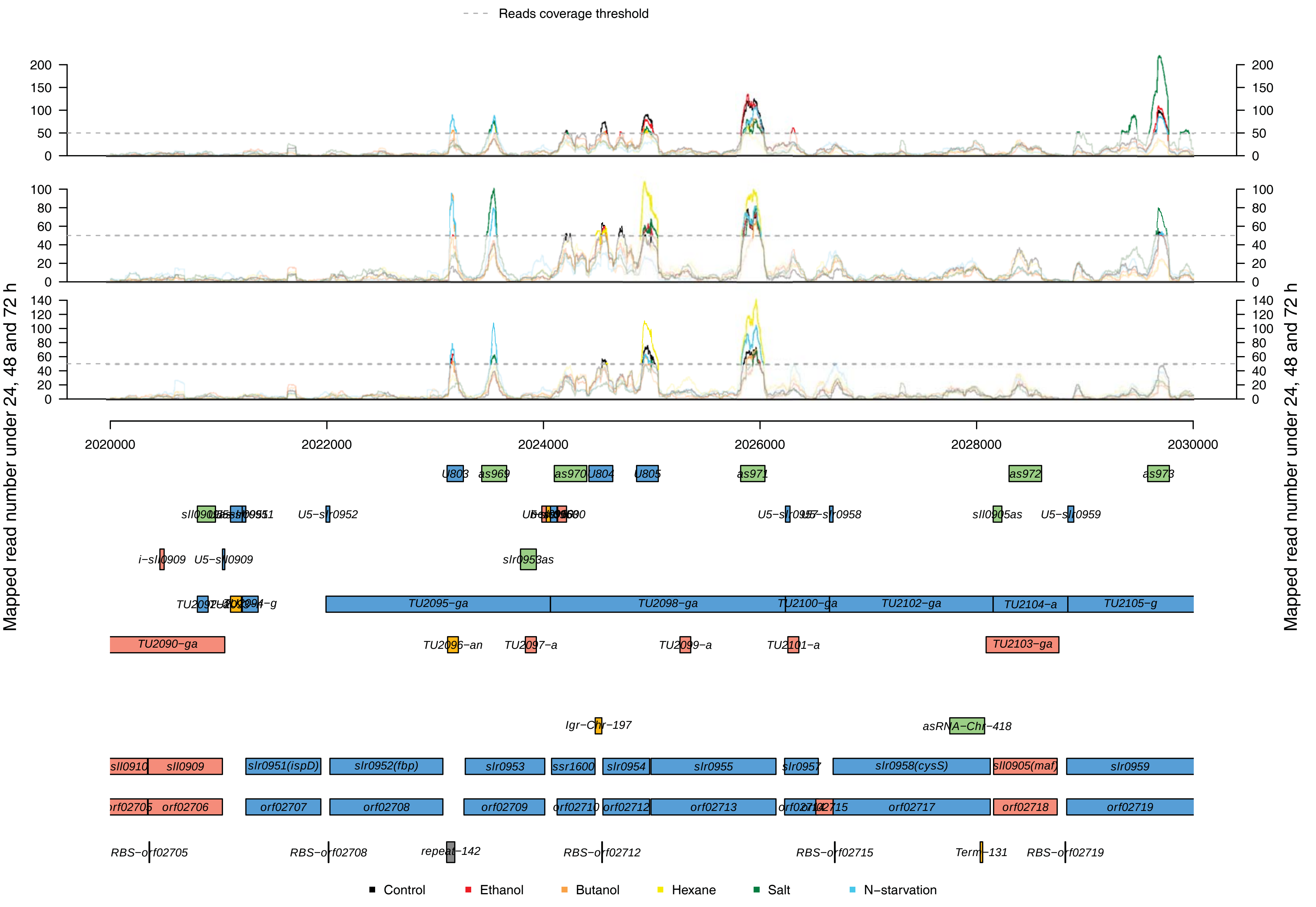

Reads coverage threshold
Mapped read number under 24, 48 and 72 h
2020000
2022000
2024000
2026000
2028000
2030000
U803
as969
as970
U804
U805
as971
as972
as973
U5−slr0952
U5−sll0909
i−sll0909
slr0953as
sll0905as
U5−slr0959
TU2095−ga
TU2098−ga
TU2100−ga
TU2102−ga
TU2104−a
TU2105−g
TU2090−ga
TU2096−an
TU2097−a
TU2099−a
TU2101−a
TU2103−ga
Igr−Chr−197
asRNA−Chr−418
sll0910
sll0909
slr0951(ispD)
slr0952(fbp)
slr0953
ssr1600
slr0954
slr0955
slr0957
slr0958(cysS)
sll0905(maf)
slr0959
orf02706
orf02707
orf02708
orf02709
orf02710
orf02712
orf02713
orf02717
orf02718
orf02719
RBS−orf02705
RBS−orf02708
repeat−142
RBS−orf02712
RBS−orf02715
Term−131
RBS−orf02719
Control
Ethanol
Butanol
Hexane
Salt
N−starvation

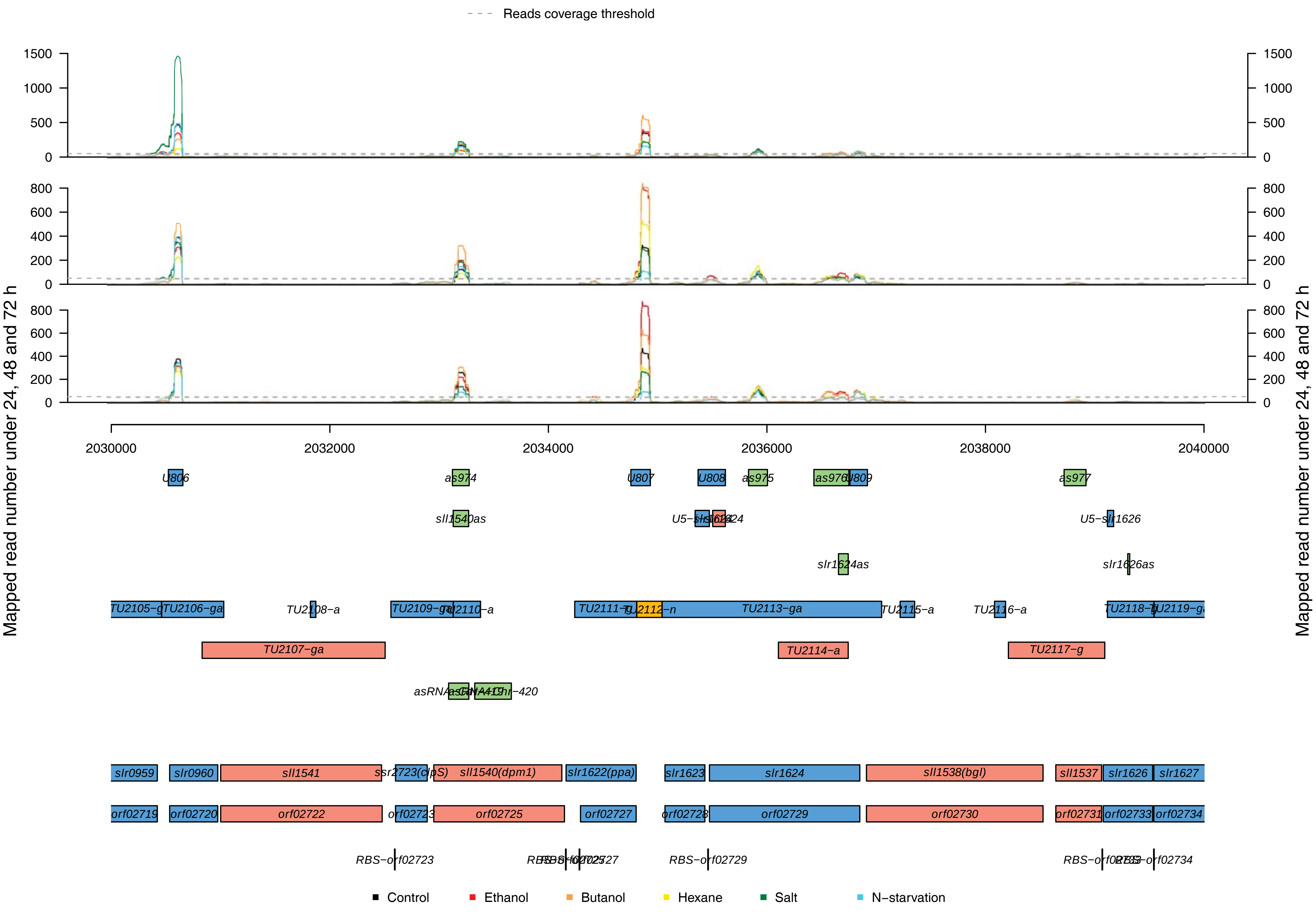

Reads coverage threshold
Mapped read number under 24, 48 and 72 h
1500
1000
500
0
800
600
400
200
2030000
2032000
2034000
2036000
2038000
2040000
U806
as974
U807
U808
as975
as977
sll1540as
U5−slr1626
slr1624as
slr1626as
TU2107−ga
TU2113−ga
TU2114−a
TU2117−g
slr0959
slr0960
sll1541
sll1540(dpm1)
slr1622(ppa)
slr1623
slr1624
sll1538(bgl)
sll1537
slr1626
slr1627
orf02719
orf02720
orf02722
orf02725
orf02727
orf02729
orf02730
orf02731
orf02733
orf02734
RBS−orf02723
RBS−orf02729
Control
Ethanol
Butanol
Hexane
Salt
N−starvation

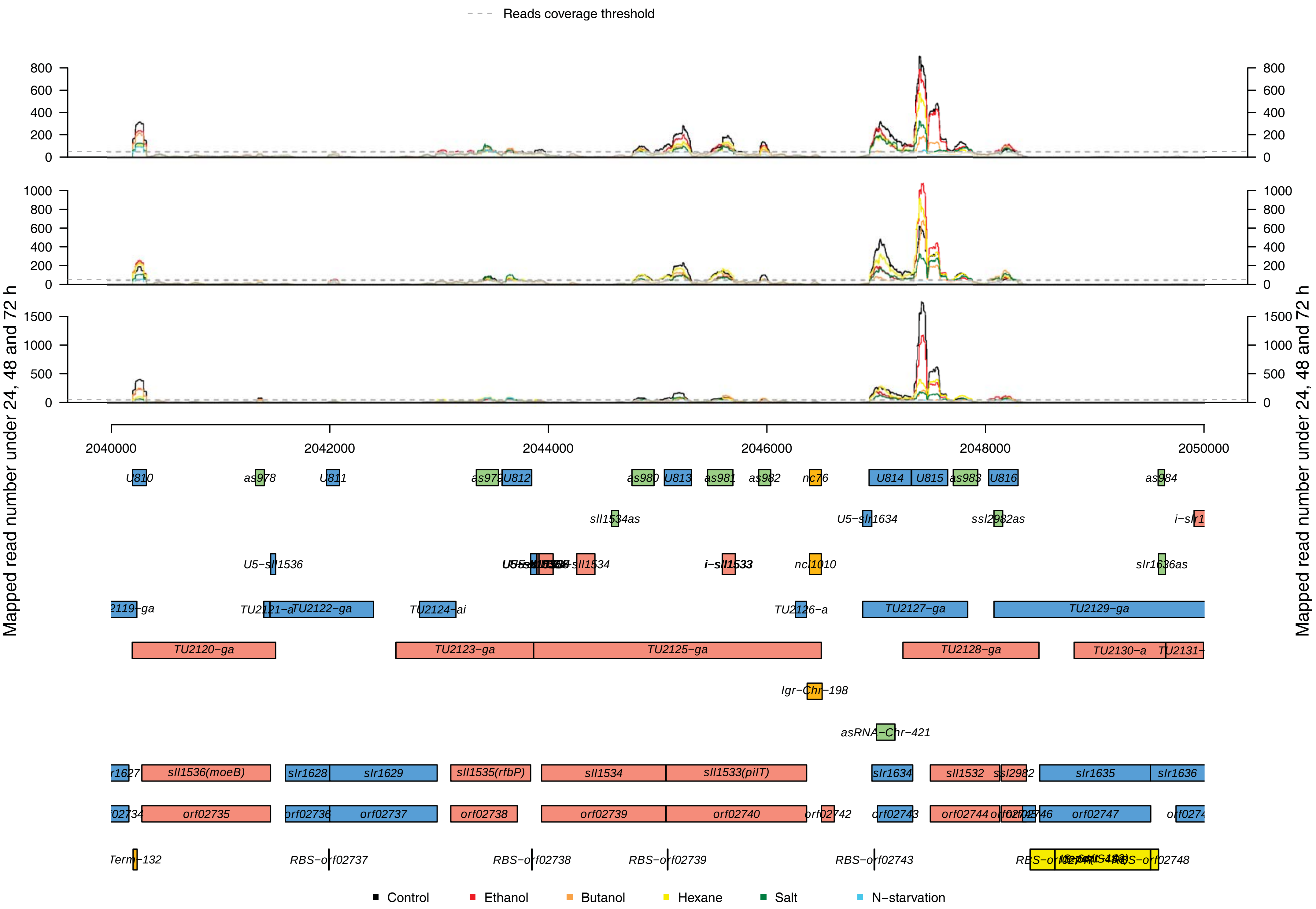
Reads coverage threshold
Mapped read number under 24, 48 and 72 h
2040000
2042000
2044000
2046000
2048000
2050000
U810
as978
U811
U812
as980
U813
as981
as982
nc76
U814
U815
as983
U816
as984
sll1534as
U5−slr1634
ssl2982as
U5−sll1536
i−sll1533
ncl1010
slr1636as
TU2122−ga
TU2124−ai
TU2126−a
TU2127−ga
TU2129−ga
TU2120−ga
TU2123−ga
TU2125−ga
TU2128−ga
TU2130−a
Igr−Chr−198
asRNA−Chr−421
sll1536(moeB)
slr1628
slr1629
sll1535(rfbP)
sll1534
sll1533(pilT)
slr1634
sll1532
slr1635
slr1636
orf02735
orf02737
orf02738
orf02739
orf02740
orf02747
RBS−orf02737
RBS−orf02738
RBS−orf02739
RBS−orf02743
Control
Ethanol
Butanol
Hexane
Salt
N−starvation

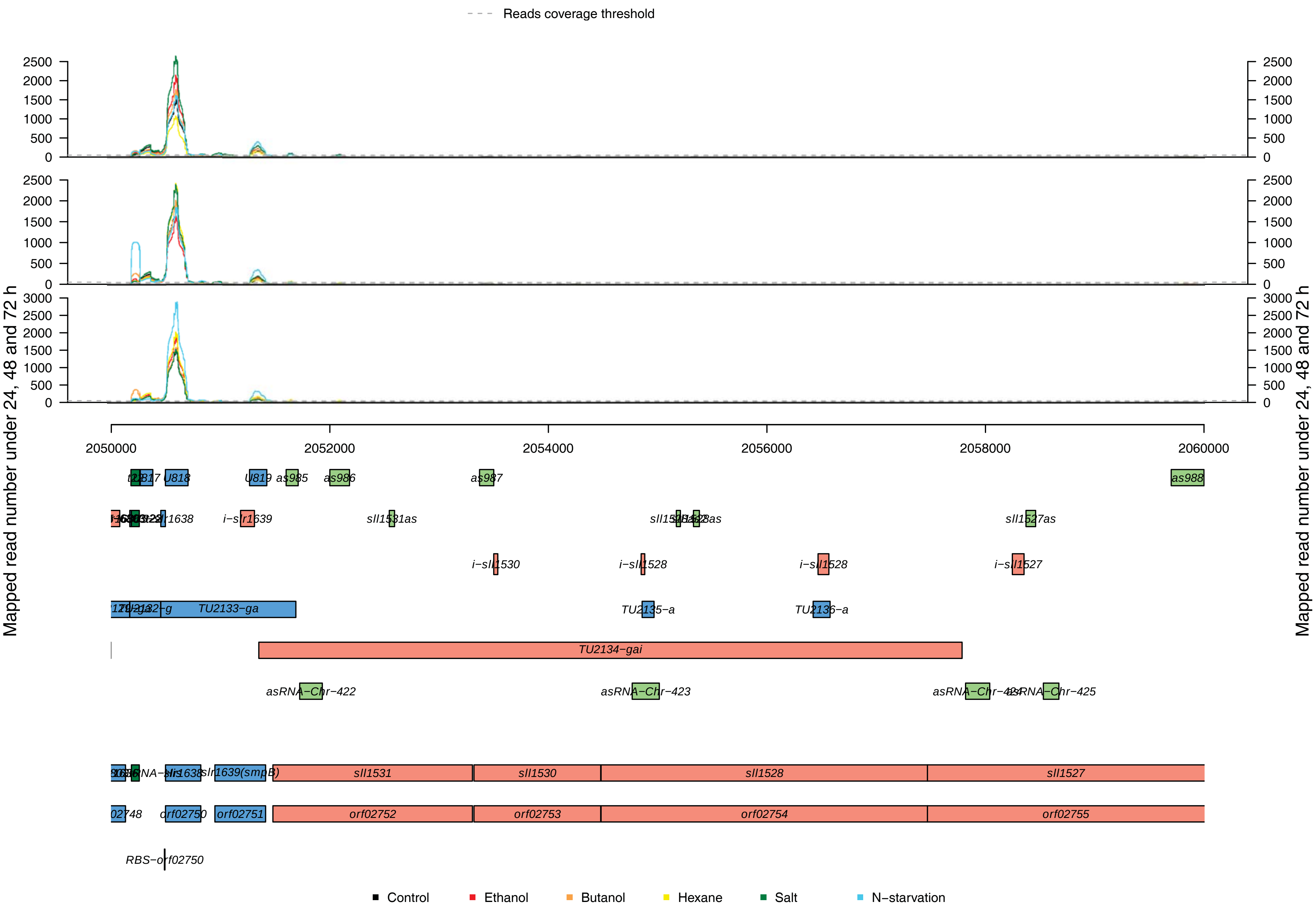

Reads coverage threshold
Mapped read number under 24, 48 and 72 h
2050000
2052000
2054000
2056000
2058000
2060000
TU2134−gai
TU2133−ga
TU2135−a
TU2136−a
i−sll1530
i−sll1528
i−sll1527
i−slr1639
sll1531as
sll1527as
asRNA−Chr−422
asRNA−Chr−423
asRNA−Chr−425
sll1531
sll1530
sll1528
sll1527
orf02751
orf02752
orf02753
orf02754
orf02755
RBS−orf02750
as985
as986
as987
as988
Control
Ethanol
Butanol
Hexane
Salt
N−starvation

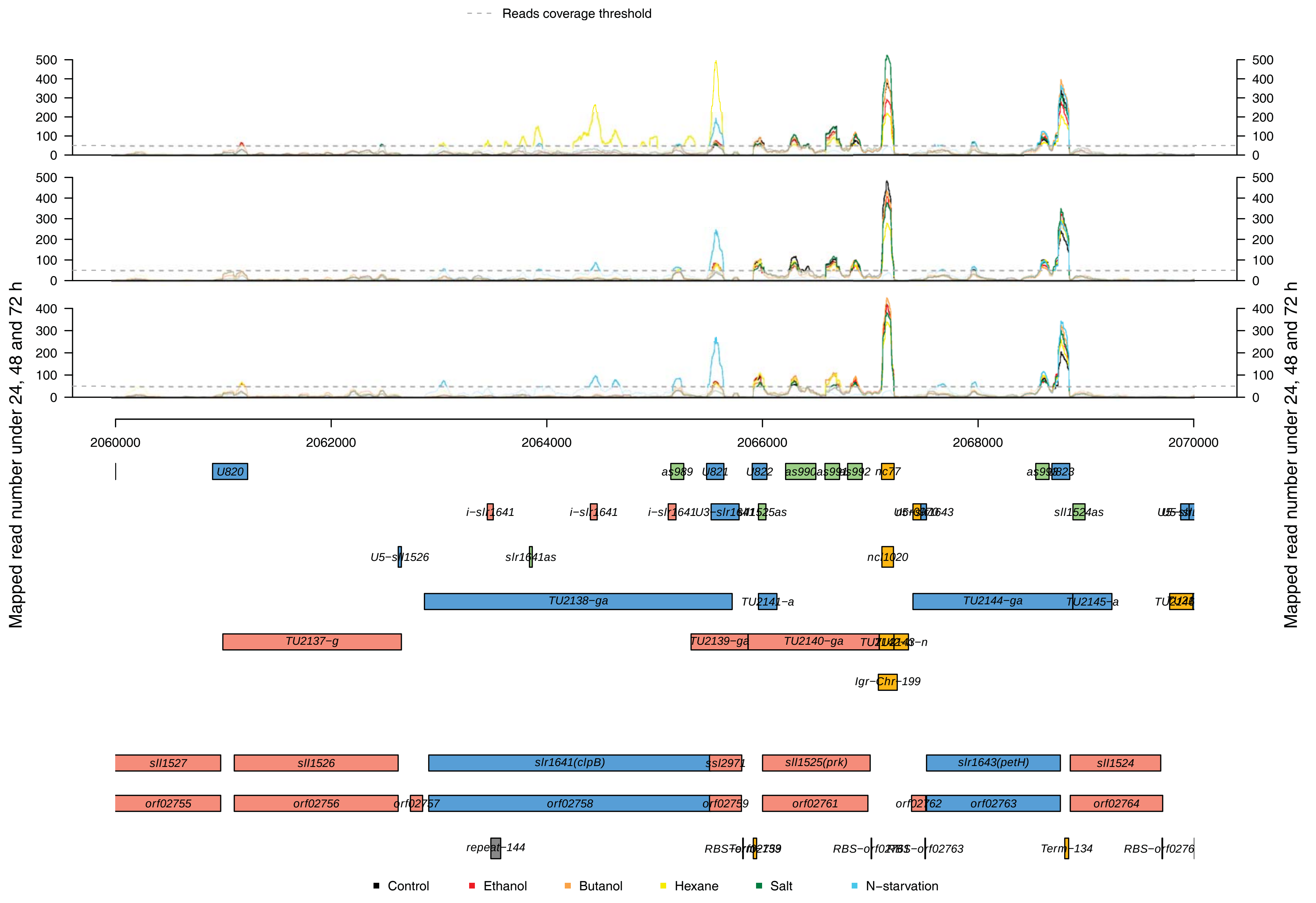

Reads coverage threshold
Mapped read number under 24, 48 and 72 h
2060000
2062000
2064000
2066000
2068000
2070000
U820
U821
U822
U823
TU2137−g
TU2138−ga
TU2139−ga
TU2140−ga
TU2141−a
TU2144−ga
TU2145−a
sll1527
sll1526
slr1641(clpB)
ssl2971
sll1525(prk)
slr1643(petH)
sll1524
orf02755
orf02756
orf02758
orf02761
orf02763
orf02764
repeat−144
Term−134
Control
Ethanol
Butanol
Hexane
Salt
N−starvation

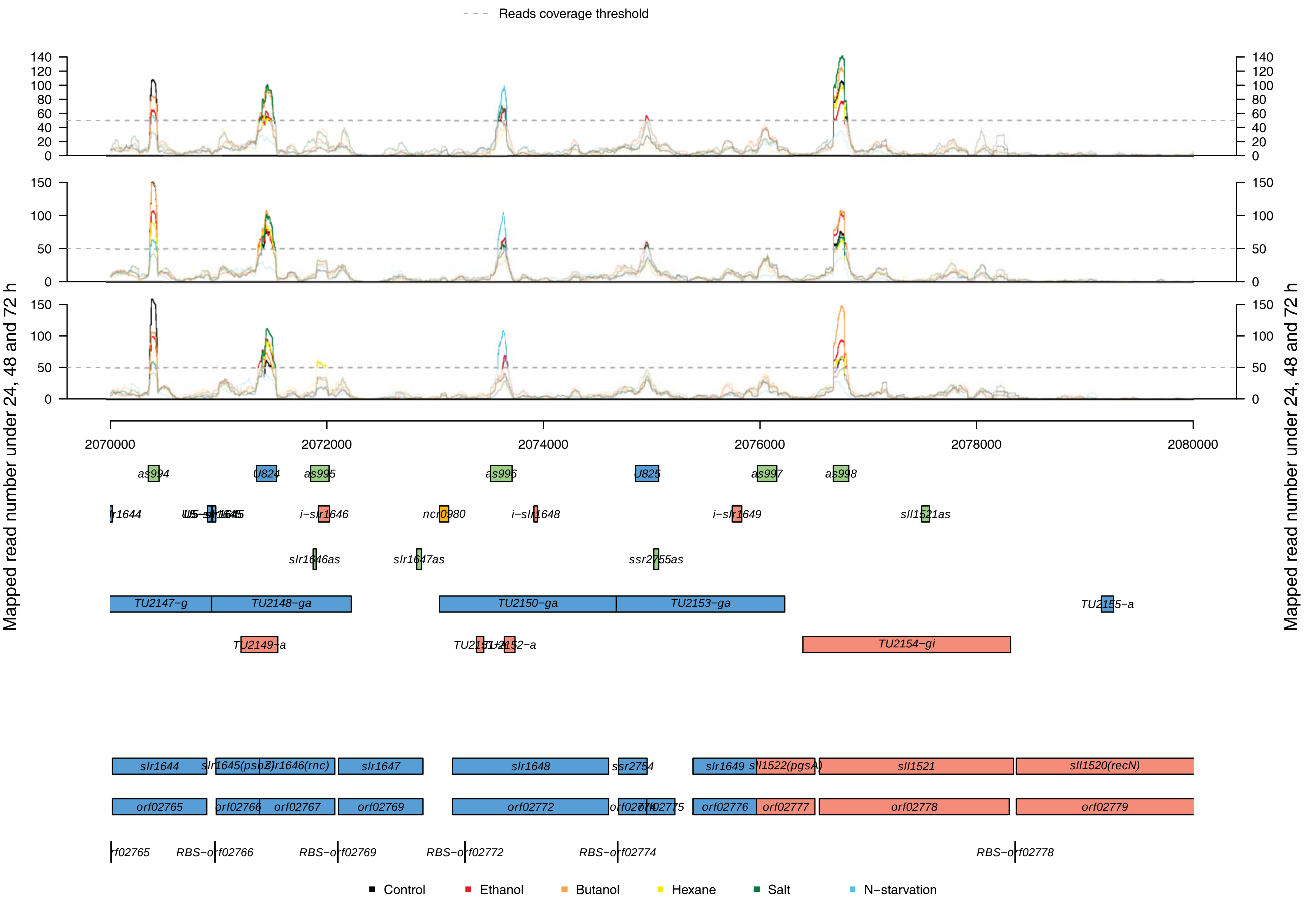

Reads coverage threshold
Mapped read number under 24, 48 and 72 h
2070000
2072000
2074000
2076000
2078000
2080000
as994
U824
as995
as996
U825
as997
as998
i-slr1646
ncr0980
i-slr1648
i-slr1649
sll1521as
slr1646as
slr1647as
ssr2755as
TU2147-g
TU2148-ga
TU2150-ga
TU2153-ga
TU2155-a
TU2149-a
TU2154-gi
slr1644
slr1645(psb2)
slr1646(rnc)
slr1647
slr1648
ssr2754
slr1649
sll1522(pgsA)
sll1521
sll1520(recN)
orf02765
orf02766
orf02767
orf02769
orf02772
orf02776
orf02777
orf02778
orf02779
RBS-orf02766
RBS-orf02769
RBS-orf02772
RBS-orf02774
RBS-orf02778
Control
Ethanol
Butanol
Hexane
Salt
N-starvation

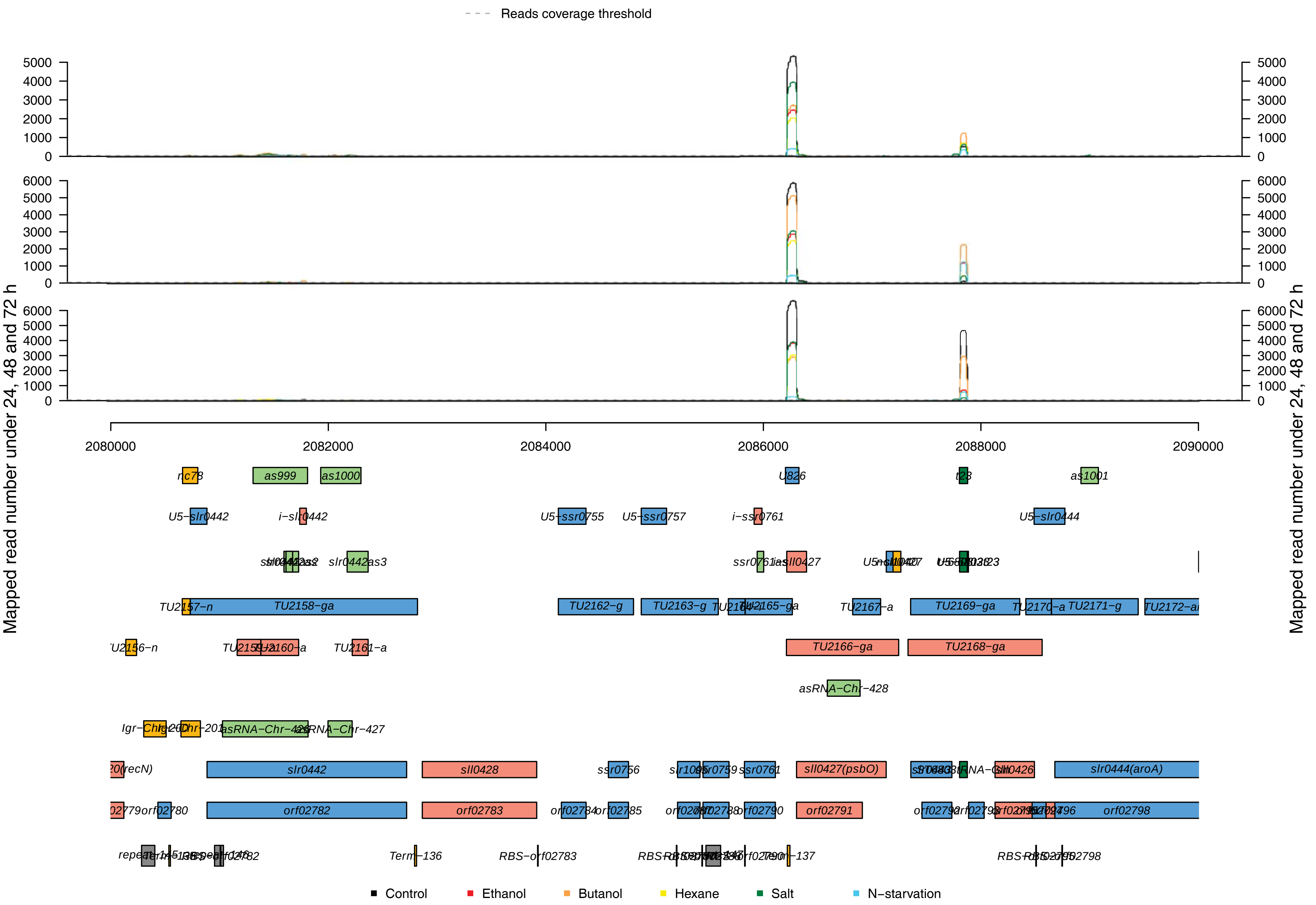

Reads coverage threshold
Mapped read number under 24, 48 and 72 h
2080000
2082000
2084000
2086000
2088000
2090000
as999
as1000
U826
as1001
U5−slr0442
i−slr0442
U5−ssr0755
U5−ssr0757
i−ssr0761
U5−slr0444
slr0442as3
TU2158−ga
TU2162−g
TU2163−g
TU2167−a
TU2169−ga
TU2171−g
TU2166−ga
TU2168−ga
asRNA−Chr−428
asRNA−Chr−427
slr0442
sll0428
ssr0756
ssr0761
sll0427(psbO)
slr0444(aroA)
orf02782
orf02783
orf02785
orf02790
orf02791
orf02798
Term−136
RBS−orf02783
Term−137
Control
Ethanol
Butanol
Hexane
Salt
N−starvation

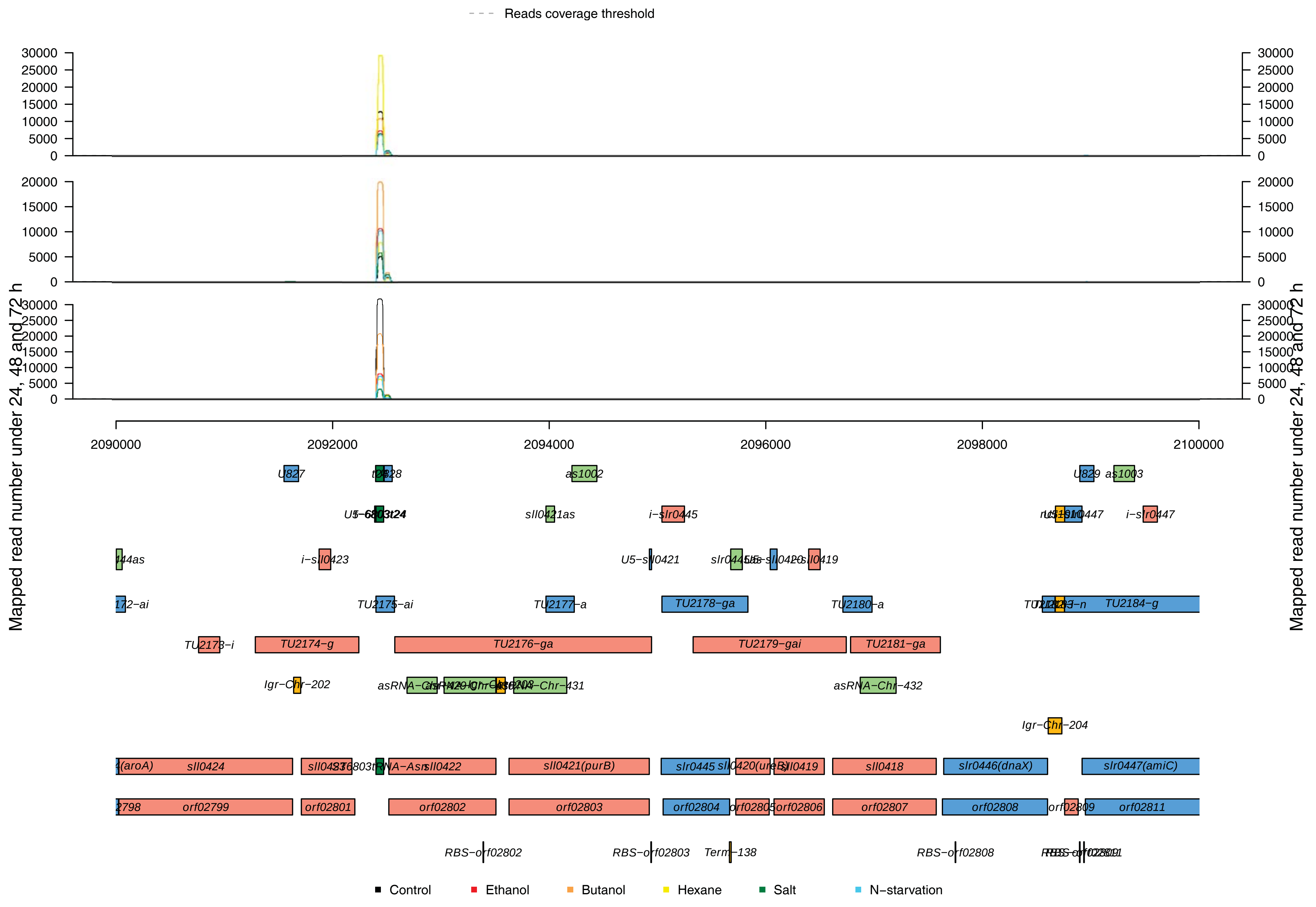

Reads coverage threshold
Mapped read number under 24, 48 and 72 h
2090000
2092000
2094000
2096000
2098000
2100000
U827
U828
as1002
U829
as1003
sll0421as
i−slr0445
i−slr0447
i−sll0423
U5−sll0421
TU2175−ai
TU2177−a
TU2178−ga
TU2180−a
TU2184−g
TU2174−g
TU2176−ga
TU2179−gai
TU2181−ga
Igr−Chr−202
asRNA−Chr−431
asRNA−Chr−432
Igr−Chr−204
sll0424
sll0422
sll0421(purB)
slr0445
sll0419
sll0418
slr0446(dnaX)
slr0447(amiC)
orf02799
orf02801
orf02802
orf02803
orf02804
orf02806
orf02807
orf02808
orf02811
RBS−orf02802
RBS−orf02803
Term−138
RBS−orf02808
Control
Ethanol
Butanol
Hexane
Salt
N−starvation

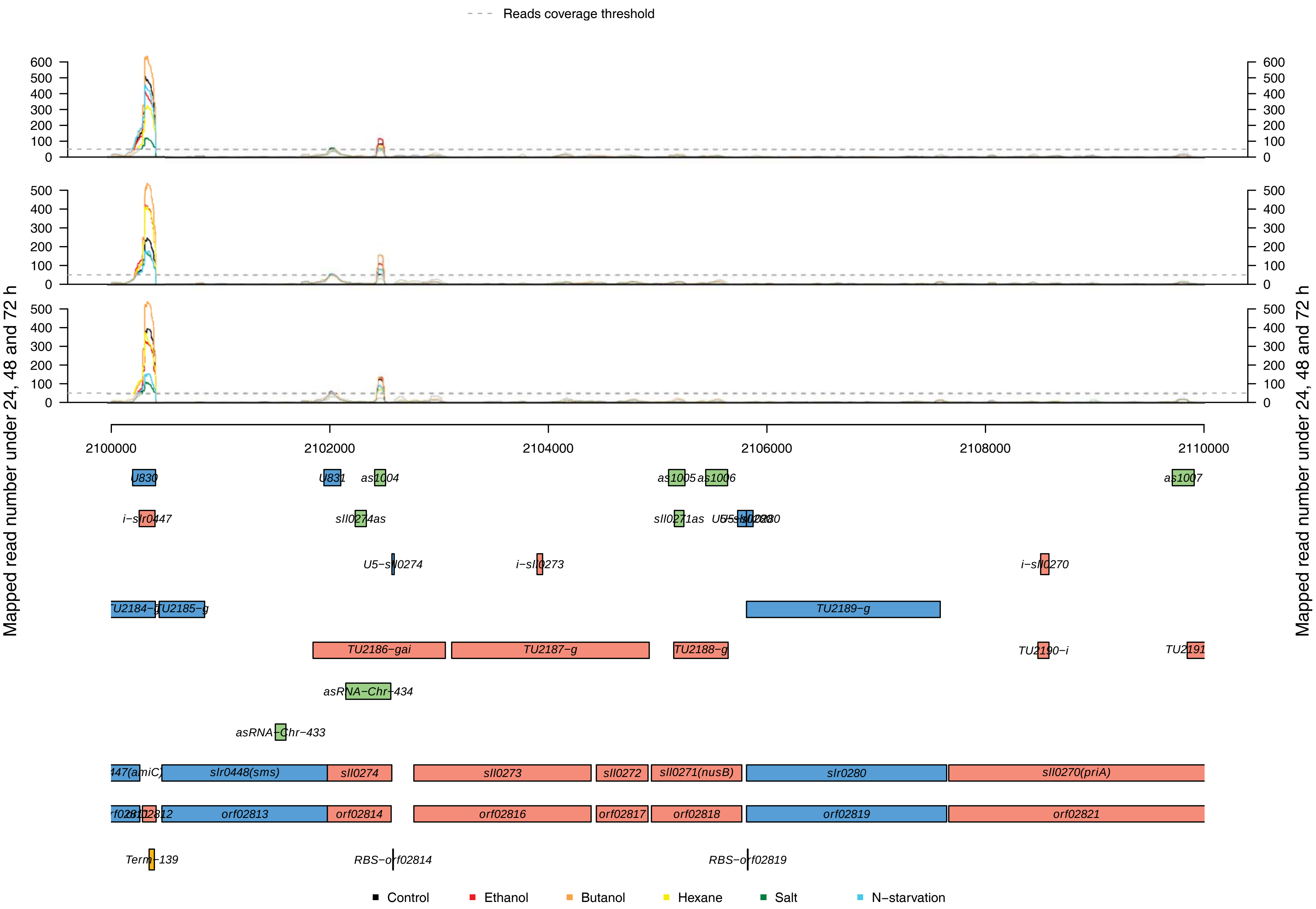

Reads coverage threshold
Mapped read number under 24, 48 and 72 h
2100000
2102000
2104000
2106000
2108000
2110000
U830
U831
as1004
as1005
as1006
as1007
i−slr0447
sll0274as
sll0271as
U5−sll0274
i−sll0273
i−sll0270
TU2189−g
TU2186−gai
TU2187−g
TU2188−g
TU2190−i
asRNA−Chr−434
asRNA−Chr−433
slr0448(sms)
sll0274
sll0273
sll0272
sll0271(nusB)
slr0280
sll0270(priA)
orf02813
orf02814
orf02816
orf02817
orf02818
orf02819
orf02821
Term−139
RBS−orf02814
RBS−orf02819
Control
Ethanol
Butanol
Hexane
Salt
N−starvation

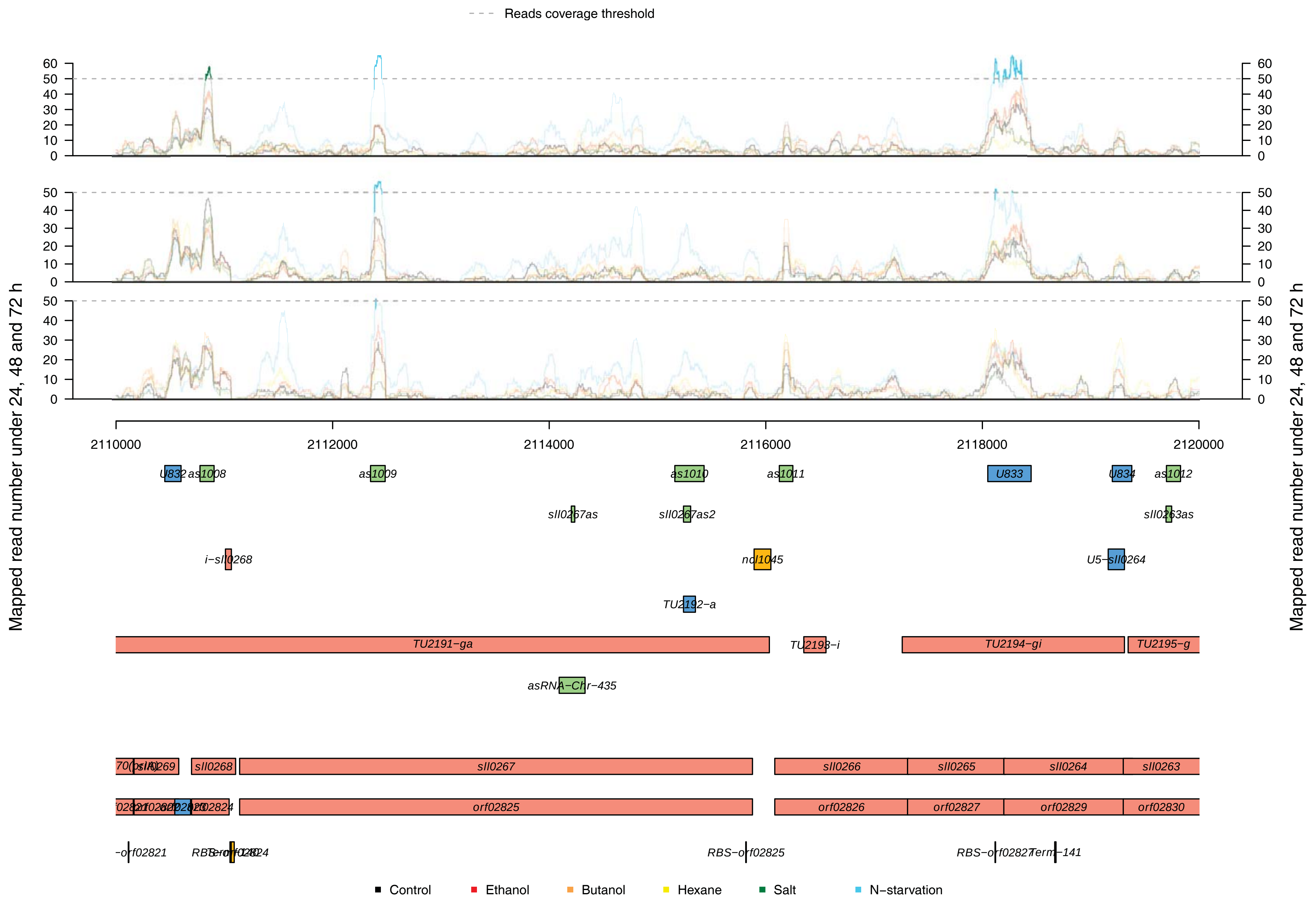

Reads coverage threshold
Mapped read number under 24, 48 and 72 h
2110000
2112000
2114000
2116000
2118000
2120000
U832
as1008
as1009
as1010
as1011
U833
U834
as1012
sll0267as
sll0267as2
sll0263as
i−sll0268
ncl1045
U5−sll0264
TU2192−a
TU2191−ga
TU2193−i
TU2194−gi
TU2195−g
asRNA−Chr−435
sll0268
sll0267
sll0266
sll0265
sll0264
sll0263
orf02825
orf02826
orf02827
orf02829
orf02830
RBS−orf02825
Term−141
Control
Ethanol
Butanol
Hexane
Salt
N−starvation

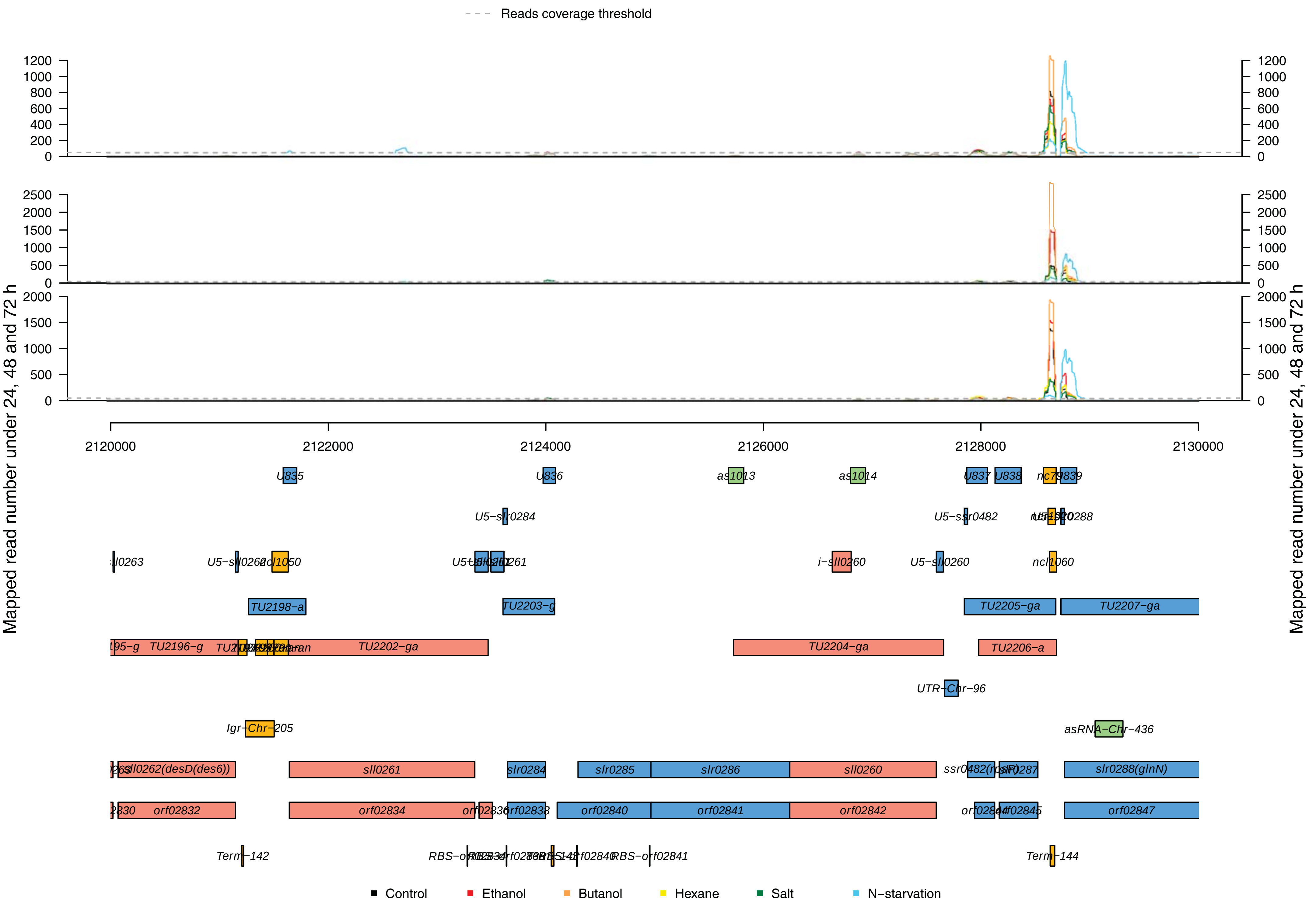

Reads coverage threshold
Mapped read number under 24, 48 and 72 h
2120000
2122000
2124000
2126000
2128000
2130000
U835
U836
as1013
as1014
U837
U838
U5−slr0284
U5−ssr0482
U5−sll0260
i−sll0260
ncl1060
TU2198−a
TU2203−g
TU2205−ga
TU2207−ga
TU2196−g
TU2202−ga
TU2204−ga
TU2206−a
UTR−Chr−96
Igr−Chr−205
asRNA−Chr−436
sll0262(desD(des6))
sll0261
slr0284
slr0285
slr0286
sll0260
slr0287
slr0288(glnN)
orf02832
orf02834
orf02838
orf02840
orf02841
orf02842
orf02845
orf02847
Term−142
Term−144
RBS−orf02841
Control
Ethanol
Butanol
Hexane
Salt
N−starvation

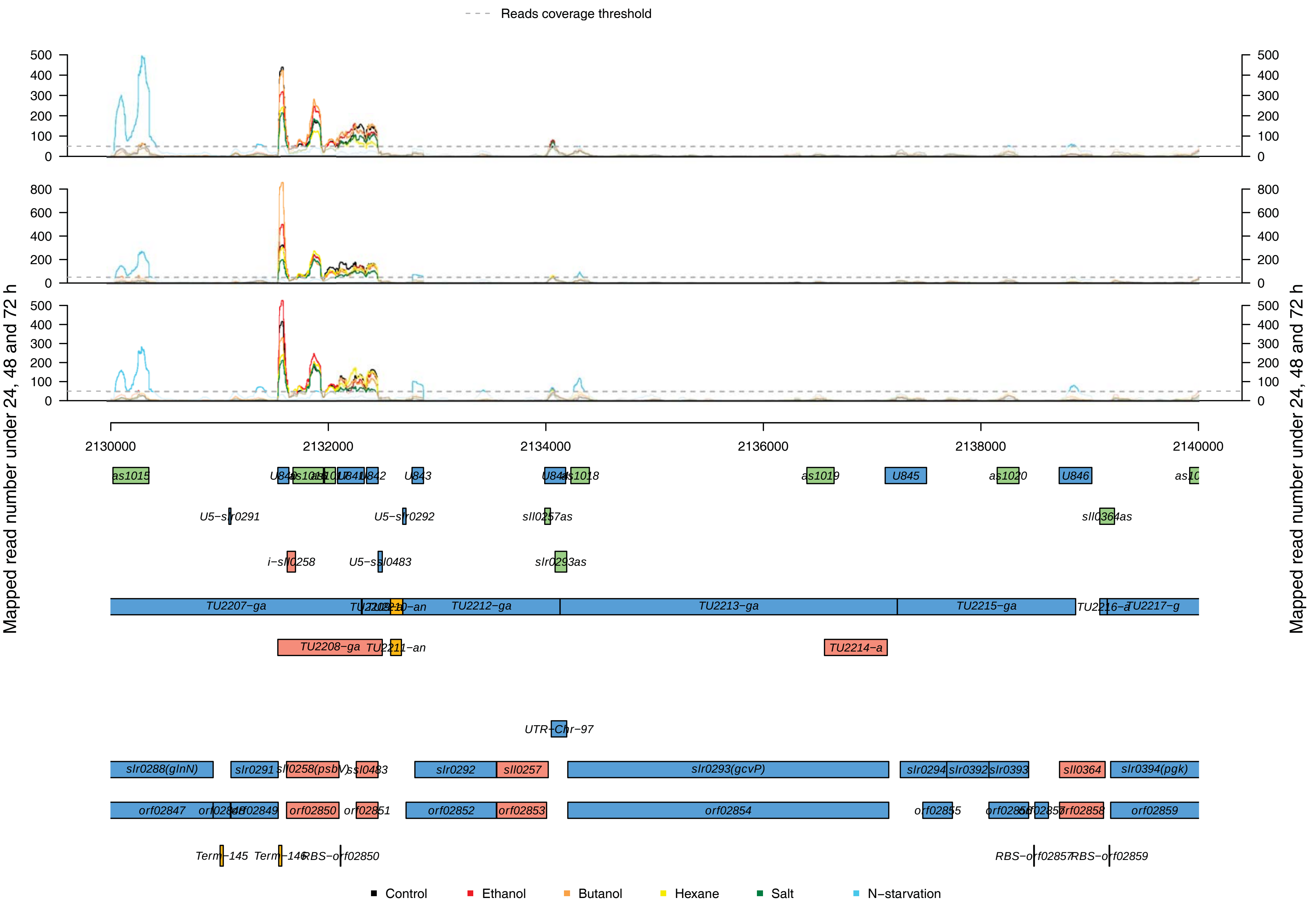

Reads coverage threshold
Mapped read number under 24, 48 and 72 h
2130000
2132000
2134000
2136000
2138000
2140000
as1015
U843
U845
U846
as1019
as1020
U5−slr0291
U5−slr0292
sll0257as
sll0364as
i−sll0258
U5−ssl0483
slr0293as
TU2207−ga
TU2212−ga
TU2213−ga
TU2215−ga
TU2208−ga
TU2214−a
UTR−Chr−97
slr0288(glnN)
slr0291
ssl0483
slr0292
sll0257
slr0293(gcvP)
slr0294
slr0392
slr0393
sll0364
slr0394(pgk)
orf02847
orf02850
orf02852
orf02853
orf02854
orf02855
orf02859
Term−145
RBS−orf02850
Control
Ethanol
Butanol
Hexane
Salt
N−starvation

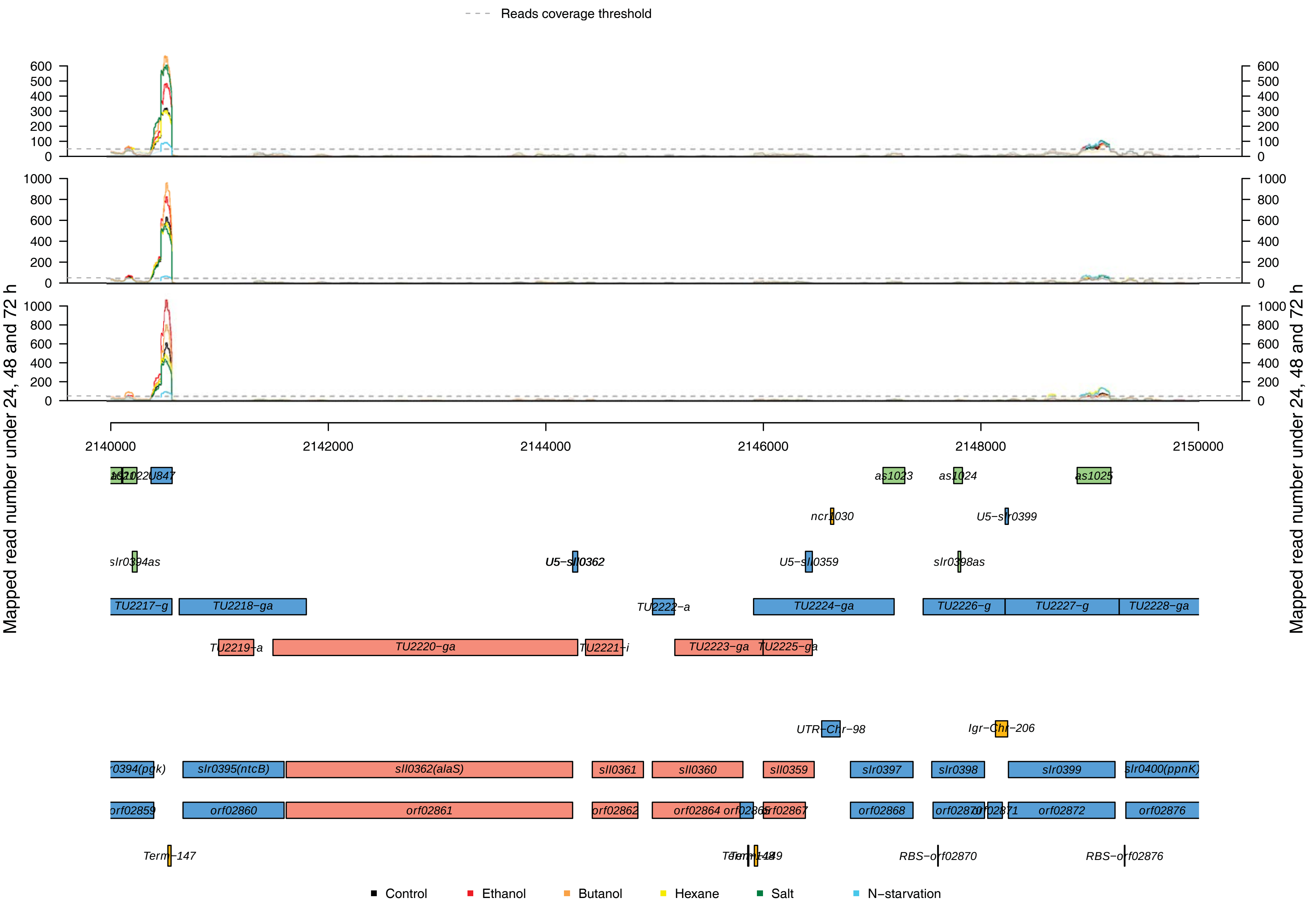

Reads coverage threshold
Mapped read number under 24, 48 and 72 h
2140000
2142000
2144000
2146000
2148000
2150000
as1023
as1024
as1025
ncr1030
U5−slr0399
slr0394as
U5−sll0362
U5−sll0359
slr0398as
TU2217−g
TU2218−ga
TU2222−a
TU2224−ga
TU2226−g
TU2227−g
TU2228−ga
TU2219−a
TU2220−ga
TU2221−i
TU2223−ga
TU2225−ga
UTR−Chr−98
Igr−Chr−206
slr0395(ntcB)
sll0362(alaS)
sll0361
sll0360
sll0359
slr0397
slr0398
slr0399
slr0400(ppnK)
orf02859
orf02860
orf02861
orf02862
orf02864
orf02867
orf02868
orf02872
orf02876
Term−147
RBS−orf02870
RBS−orf02876
Control
Ethanol
Butanol
Hexane
Salt
N−starvation

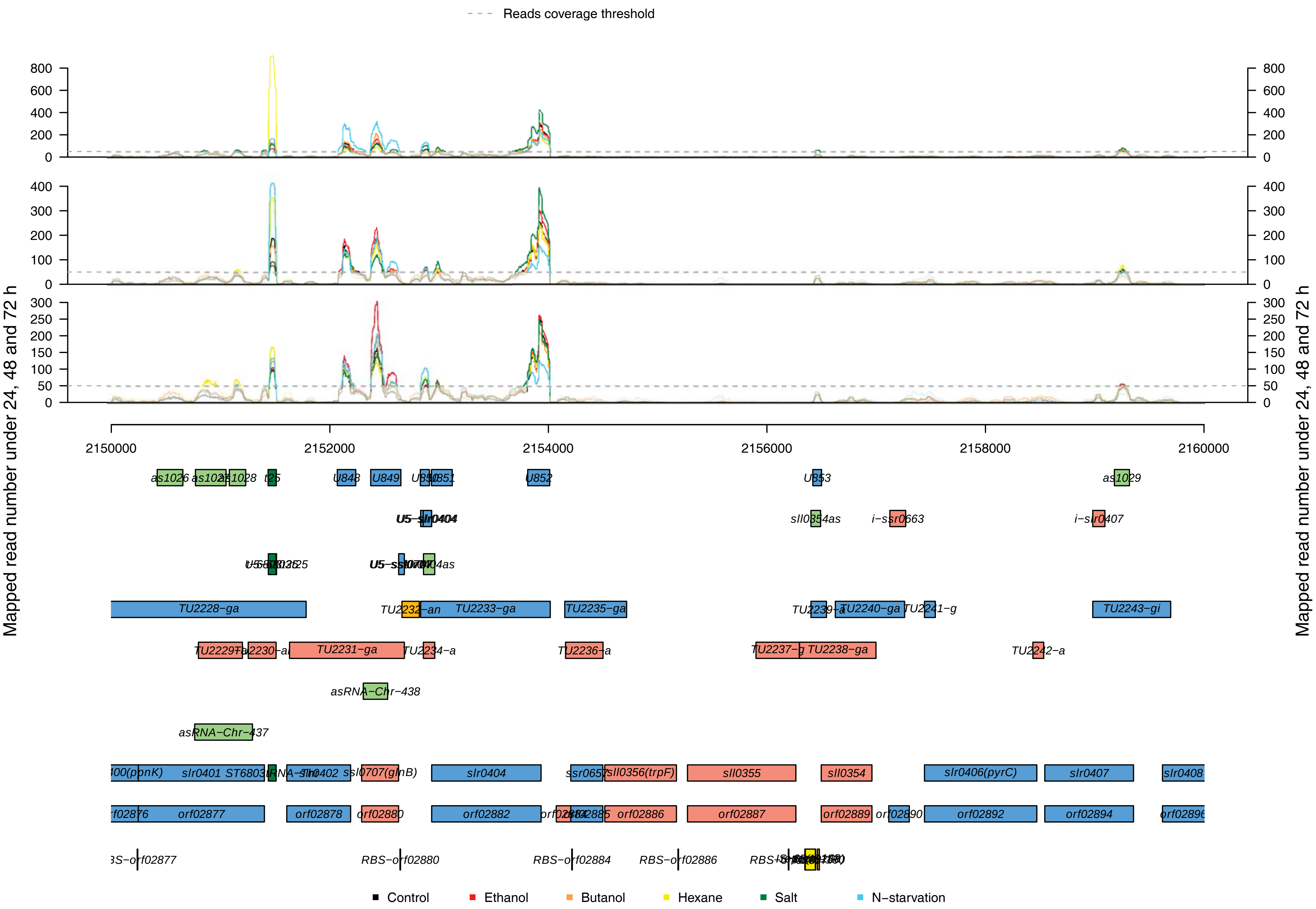

Reads coverage threshold
Mapped read number under 24, 48 and 72 h
2150000
2152000
2154000
2156000
2158000
2160000
as1026
t25
U848
U849
U852
U853
as1029
sll0354as
i-ssr0663
i-slr0407
TU2228-ga
TU2233-ga
TU2235-ga
TU2240-ga
TU2243-gi
TU2231-ga
TU2236-a
TU2238-ga
TU2242-a
asRNA-Chr-438
asRNA-Chr-437
slr0401
slr0404
sll0356(trpF)
sll0355
sll0354
slr0406(pyrC)
slr0407
slr0408
orf02877
orf02878
orf02880
orf02882
orf02886
orf02887
orf02889
orf02892
orf02894
RBS-orf02880
RBS-orf02884
RBS-orf02886
Control
Ethanol
Butanol
Hexane
Salt
N-starvation

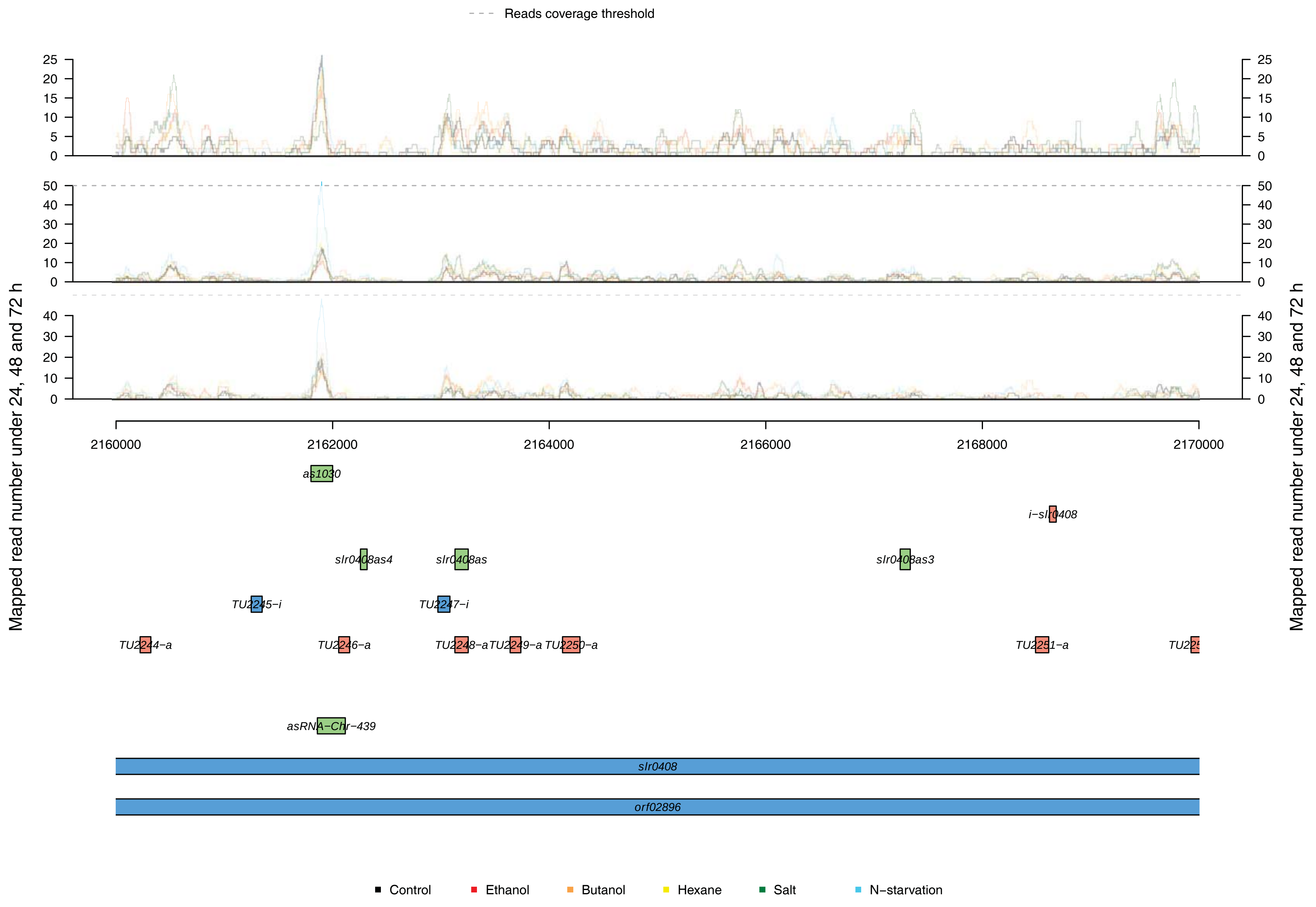

Reads coverage threshold
Mapped read number under 24, 48 and 72 h
2160000
2162000
2164000
2166000
2168000
2170000
as1030
i−slr0408
slr0408as4
slr0408as
slr0408as3
TU2245−i
TU2247−i
TU2244−a
TU2246−a
TU2248−a
TU2249−a
TU2250−a
TU2251−a
asRNA−Chr−439
slr0408
orf02896
Control
Ethanol
Butanol
Hexane
Salt
N−starvation

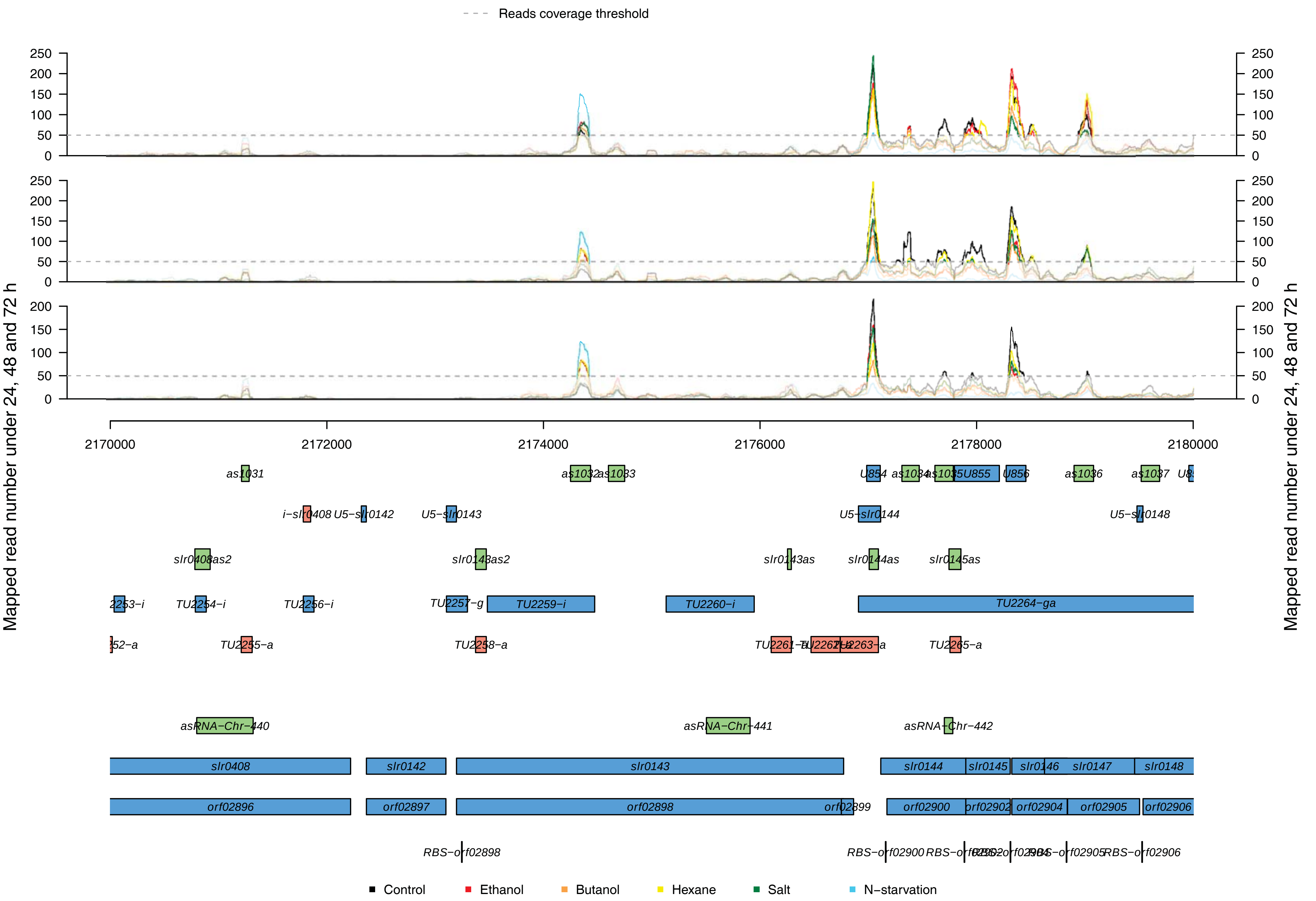

Reads coverage threshold
Mapped read number under 24, 48 and 72 h
2170000
2172000
2174000
2176000
2178000
2180000
as1031
as1032
as1033
as1036
as1037
U5−slr0142
U5−slr0143
U5−slr0144
U5−slr0148
slr0408as2
slr0143as2
slr0143as
slr0144as
slr0145as
TU2254−i
TU2256−i
TU2259−i
TU2260−i
TU2264−ga
TU2255−a
TU2258−a
TU2265−a
asRNA−Chr−440
asRNA−Chr−441
asRNA−Chr−442
slr0408
slr0142
slr0143
slr0144
slr0145
slr0146
slr0147
slr0148
orf02896
orf02897
orf02898
orf02899
orf02900
orf02902
orf02904
orf02905
orf02906
RBS−orf02898
RBS−orf02900
RBS−orf02905
RBS−orf02906
Control
Ethanol
Butanol
Hexane
Salt
N−starvation

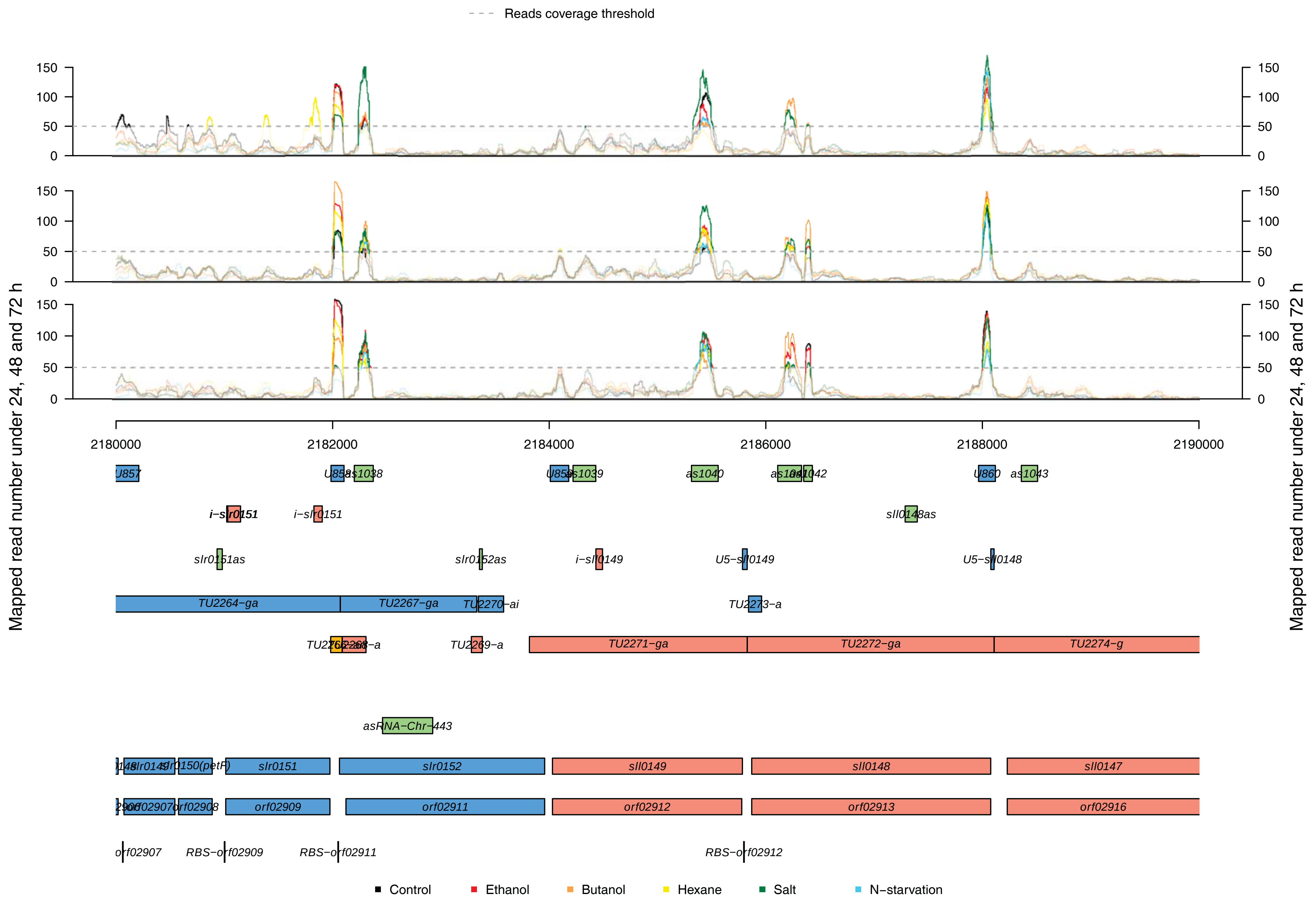

Reads coverage threshold
Mapped read number under 24, 48 and 72 h
150
100
50
0
2180000
2182000
2184000
2186000
2188000
2190000
as1040
as1043
i−slr0151
sll0148as
slr0151as
slr0152as
i−sll0149
U5−sll0149
U5−sll0148
TU2264−ga
TU2267−ga
TU2273−a
TU2269−a
TU2271−ga
TU2272−ga
TU2274−g
asRNA−Chr−443
slr0151
slr0152
sll0149
sll0148
sll0147
orf02909
orf02911
orf02912
orf02913
orf02916
RBS−orf02909
RBS−orf02911
RBS−orf02912
Control
Ethanol
Butanol
Hexane
Salt
N−starvation

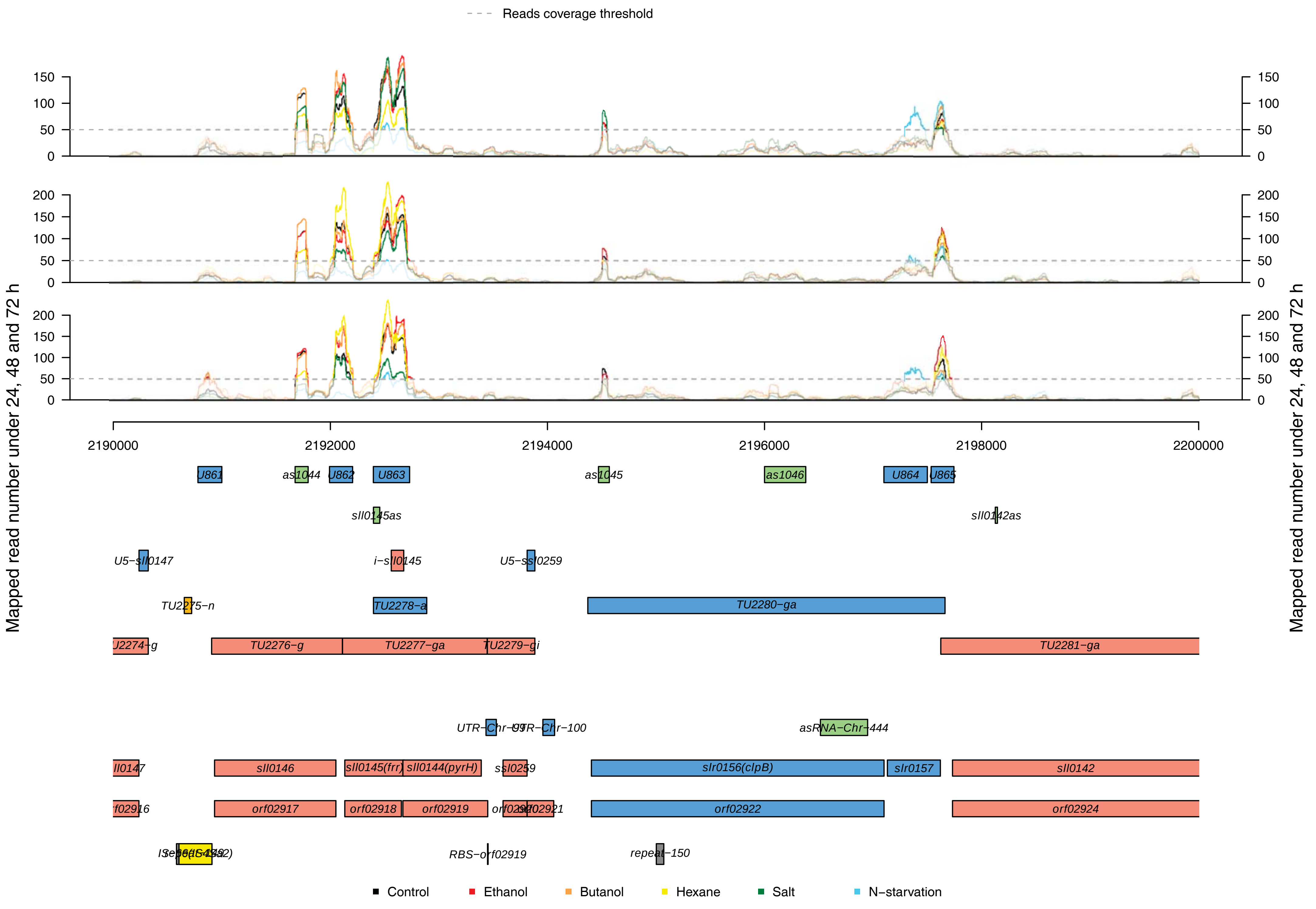

Reads coverage threshold
Mapped read number under 24, 48 and 72 h
2190000
2192000
2194000
2196000
2198000
2200000
U861
as1044
U862
U863
as1045
as1046
U864
U865
sll0145as
sll0142as
U5−sll0147
i−sll0145
U5−ssl0259
TU2275−n
TU2278−a
TU2280−ga
TU2276−g
TU2277−ga
TU2279−gi
TU2281−ga
UTR−Chr−99
UTR−Chr−100
asRNA−Chr−444
sll0146
sll0145(frr)
sll0144(pyrH)
ssl0259
slr0156(clpB)
slr0157
sll0142
orf02917
orf02918
orf02919
orf02922
orf02924
RBS−orf02919
repeat−150
Control
Ethanol
Butanol
Hexane
Salt
N−starvation

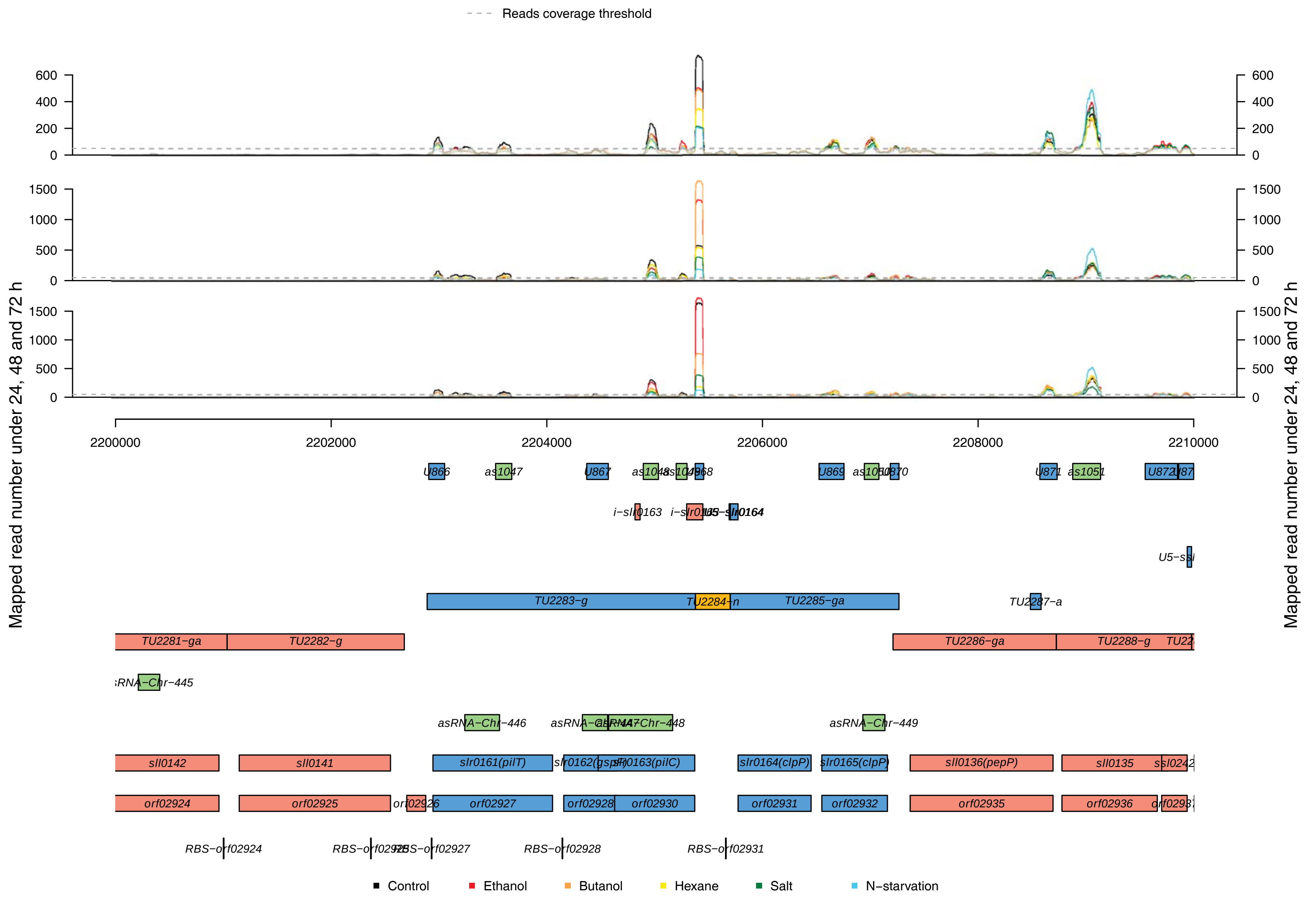

Reads coverage threshold
Mapped read number under 24, 48 and 72 h
2200000
2202000
2204000
2206000
2208000
2210000
TU2283−g
TU2284−n
TU2285−ga
TU2287−a
TU2281−ga
TU2282−g
TU2286−ga
TU2288−g
asRNA−Chr−446
asRNA−Chr−449
sll0142
sll0141
slr0161(pilT)
slr0163(pilC)
slr0164(clpP)
slr0165(clpP)
sll0136(pepP)
sll0135
orf02924
orf02925
orf02927
orf02928
orf02930
orf02931
orf02932
orf02935
orf02936
RBS−orf02924
RBS−orf02927
RBS−orf02928
RBS−orf02931
Control
Ethanol
Butanol
Hexane
Salt
N−starvation

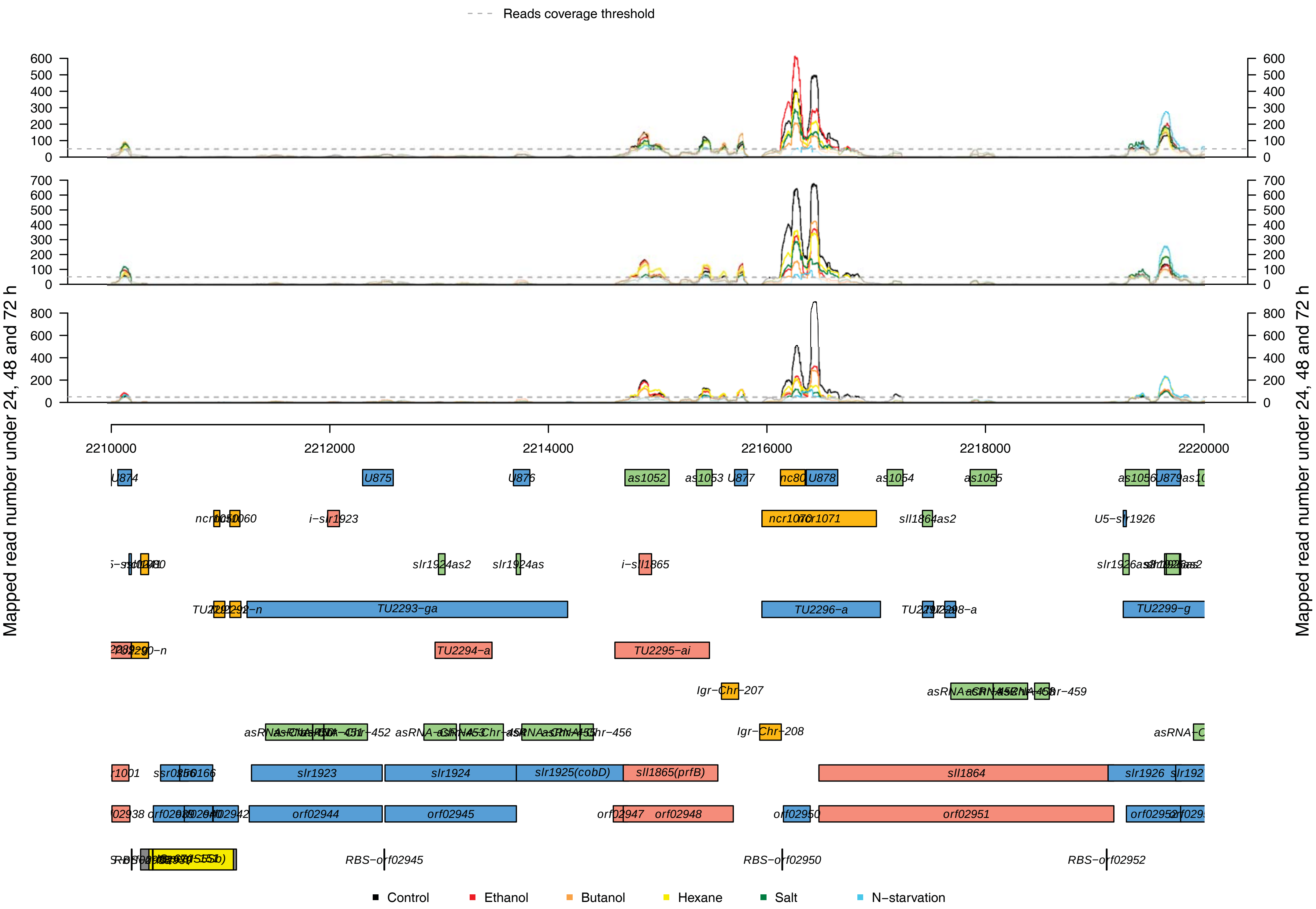
Reads coverage threshold
Mapped read number under 24, 48 and 72 h
2210000
2212000
2214000
2216000
2218000
2220000
U874
U875
U876
as1052
U877
U878
as1054
as1055
U879
i−slr1923
ncr1071
sll1864as2
U5−slr1926
slr1924as2
slr1924as
i−sll1865
TU2293−ga
TU2296−a
TU2299−g
TU2294−a
TU2295−ai
Igr−Chr−207
Igr−Chr−208
asRNA−Chr−459
asRNA−Chr−452
asRNA−Chr−456
slr1923
slr1924
slr1925(cobD)
sll1865(prfB)
sll1864
slr1926
orf02944
orf02945
orf02948
orf02951
RBS−orf02945
RBS−orf02950
RBS−orf02952
Control
Ethanol
Butanol
Hexane
Salt
N−starvation

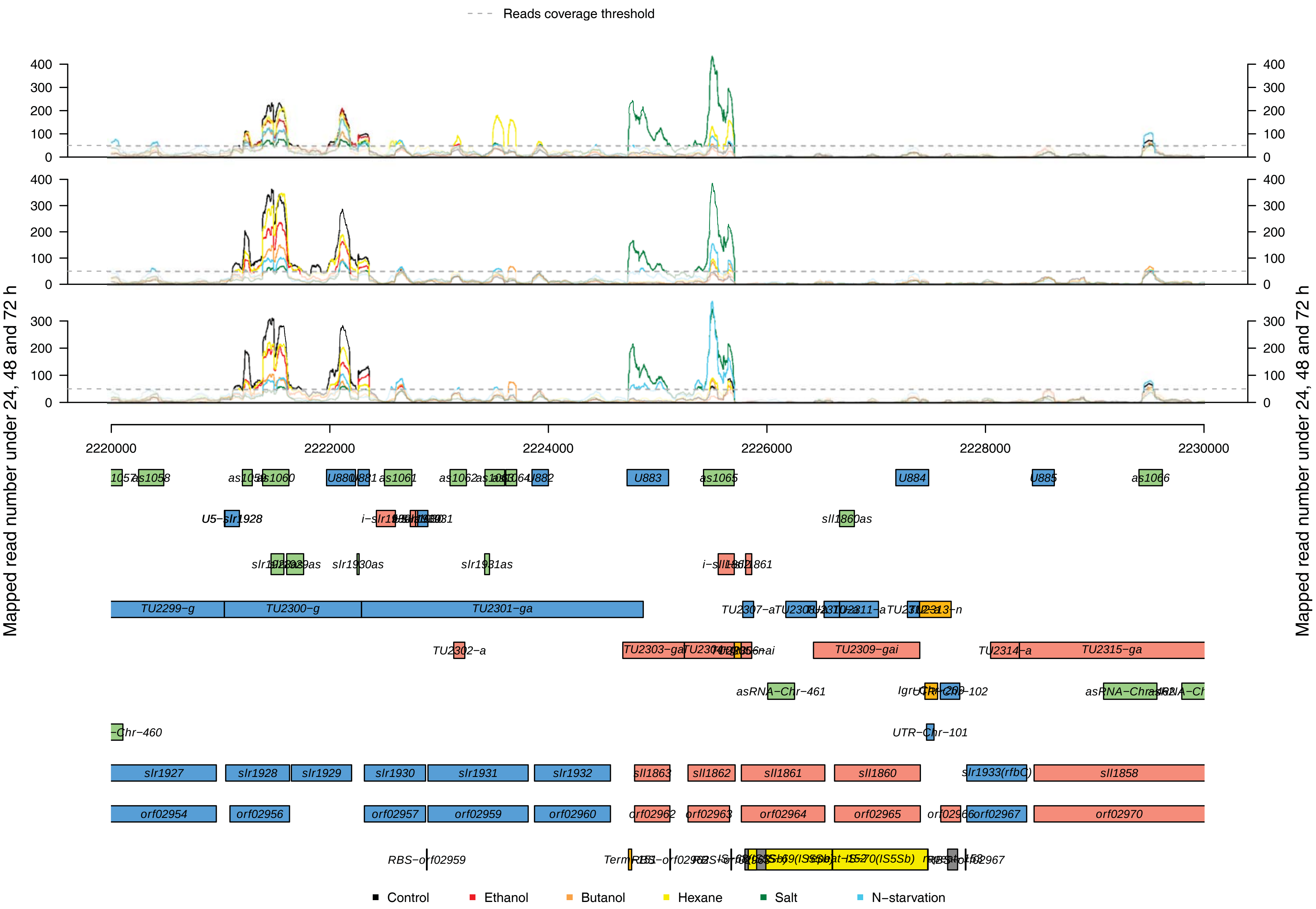
Reads coverage threshold
Mapped read number under 24, 48 and 72 h
400
300
200
100
0
2220000
2222000
2224000
2226000
2228000
2230000
as1058
as1060
as1061
as1062
U880
U882
U883
as1065
U884
U885
as1066
U5−slr1928
sll1860as
slr1930as
slr1931as
TU2299−g
TU2300−g
TU2301−ga
TU2302−a
TU2303−ga
TU2309−gai
TU2314−a
TU2315−ga
asRNA−Chr−461
UTR−Chr−101
slr1927
slr1928
slr1929
slr1930
slr1931
slr1932
sll1863
sll1862
sll1861
sll1860
slr1933(rfbC)
sll1858
orf02954
orf02956
orf02957
orf02959
orf02960
orf02962
orf02963
orf02964
orf02965
orf02967
orf02970
RBS−orf02959
Control
Ethanol
Butanol
Hexane
Salt
N−starvation

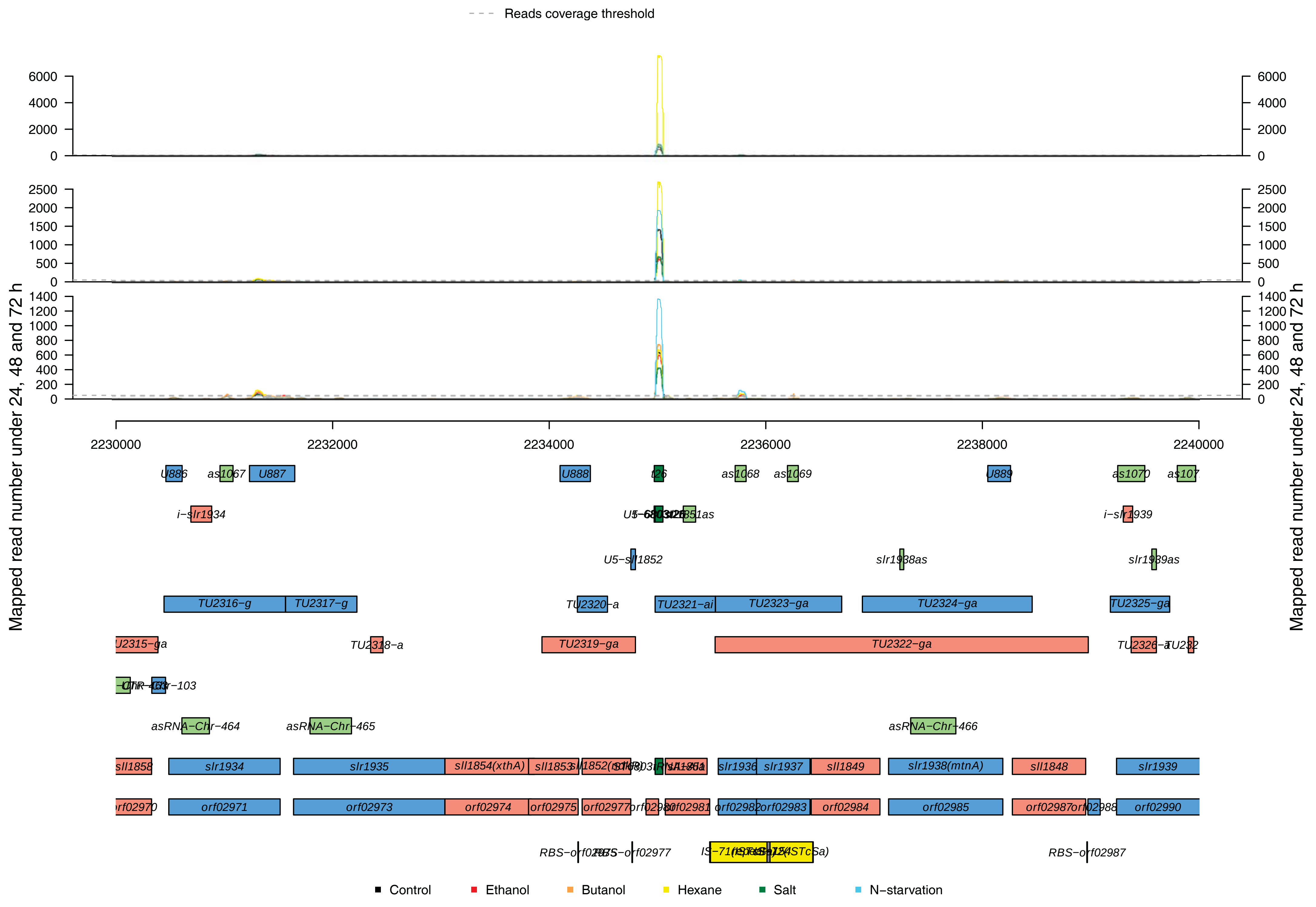

Reads coverage threshold
Mapped read number under 24, 48 and 72 h
2230000
2232000
2234000
2236000
2238000
2240000
TU2316−g
TU2317−g
TU2322−ga
TU2324−ga
TU2323−ga
TU2319−ga
asRNA−Chr−464
asRNA−Chr−465
asRNA−Chr−466
slr1934
slr1935
sll1854(xthA)
slr1938(mtnA)
sll1848
slr1939
sll1849
orf02971
orf02973
orf02974
orf02985
orf02990
orf02984
Control
Ethanol
Butanol
Hexane
Salt
N−starvation

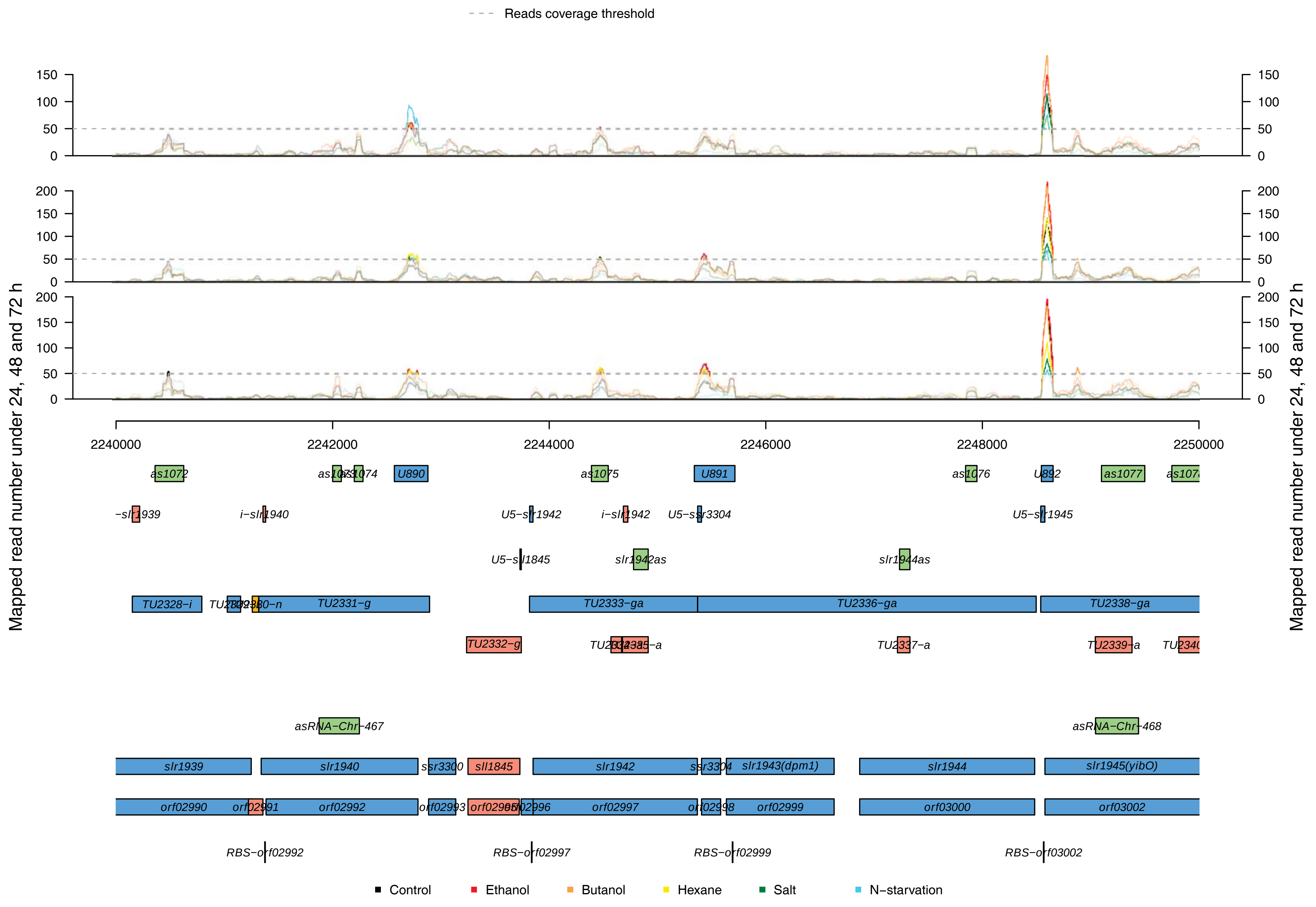

Reads coverage threshold
Mapped read number under 24, 48 and 72 h
2240000
2242000
2244000
2246000
2248000
2250000
as1072
as1074
U890
as1075
U891
as1076
U892
as1077
U5−sll1845
slr1942as
slr1944as
TU2328−i
TU2331−g
TU2333−ga
TU2336−ga
TU2338−ga
TU2332−g
TU2337−a
TU2339−a
asRNA−Chr−467
asRNA−Chr−468
slr1939
slr1940
ssr3300
sll1845
slr1942
ssr3304
slr1943(dpm1)
slr1944
slr1945(yibO)
orf02990
orf02992
orf02993
orf02997
orf02998
orf02999
orf03000
orf03002
RBS−orf02992
RBS−orf02997
RBS−orf02999
RBS−orf03002
Control
Ethanol
Butanol
Hexane
Salt
N−starvation

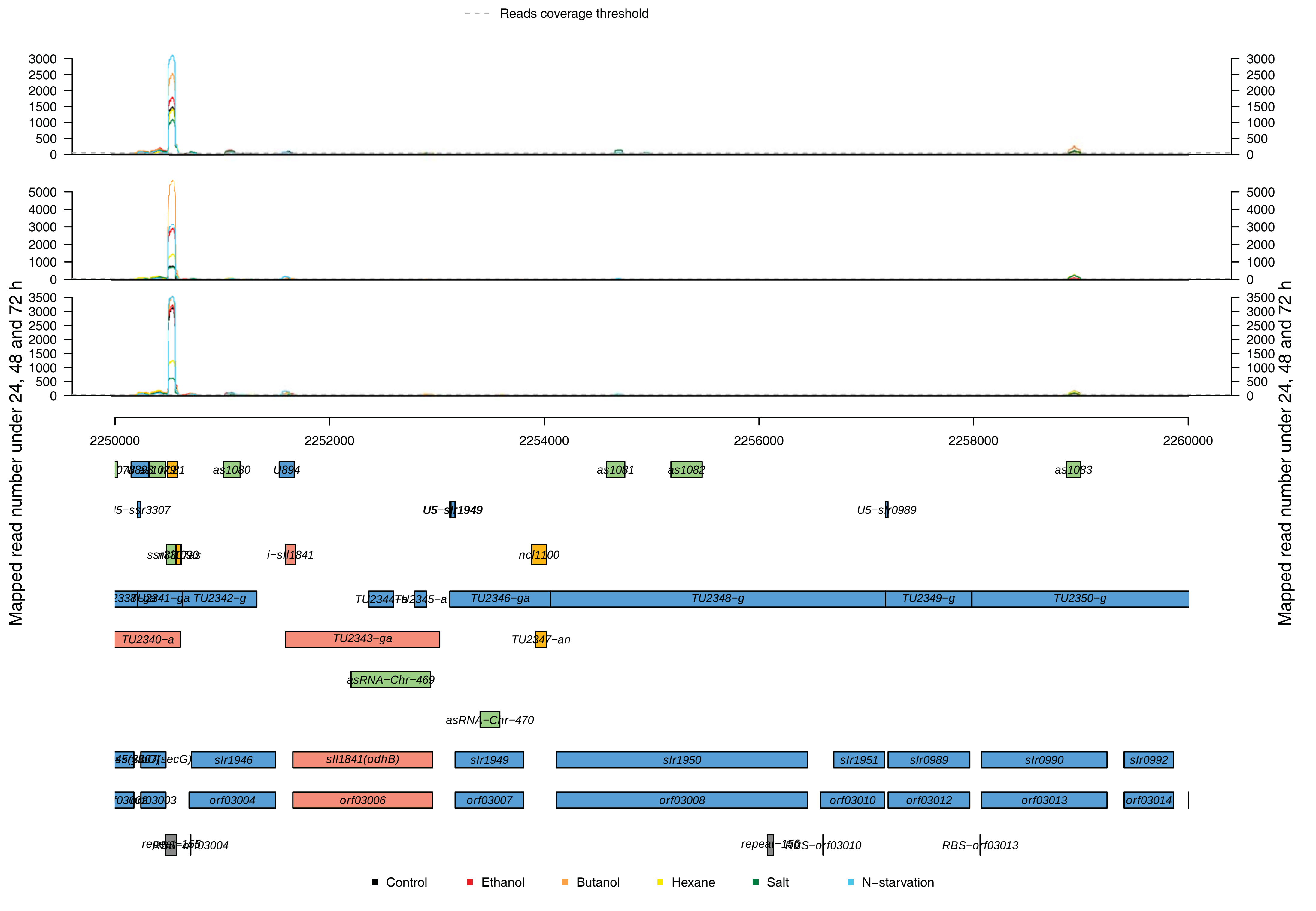

Reads coverage threshold
Mapped read number under 24, 48 and 72 h
2250000
2252000
2254000
2256000
2258000
2260000
as1080
U894
as1081
as1082
as1083
U5−ssr3307
U5−slr1949
U5−slr0989
i−sll1841
ncl1100
TU2341−ga
TU2342−g
TU2345−a
TU2346−ga
TU2348−g
TU2349−g
TU2350−g
TU2340−a
TU2343−ga
TU2347−an
asRNA−Chr−469
asRNA−Chr−470
slr1946
sll1841(odhB)
slr1949
slr1950
slr1951
slr0989
slr0990
slr0992
orf03004
orf03006
orf03007
orf03008
orf03010
orf03012
orf03013
orf03014
RBS−orf03010
RBS−orf03013
Control
Ethanol
Butanol
Hexane
Salt
N−starvation

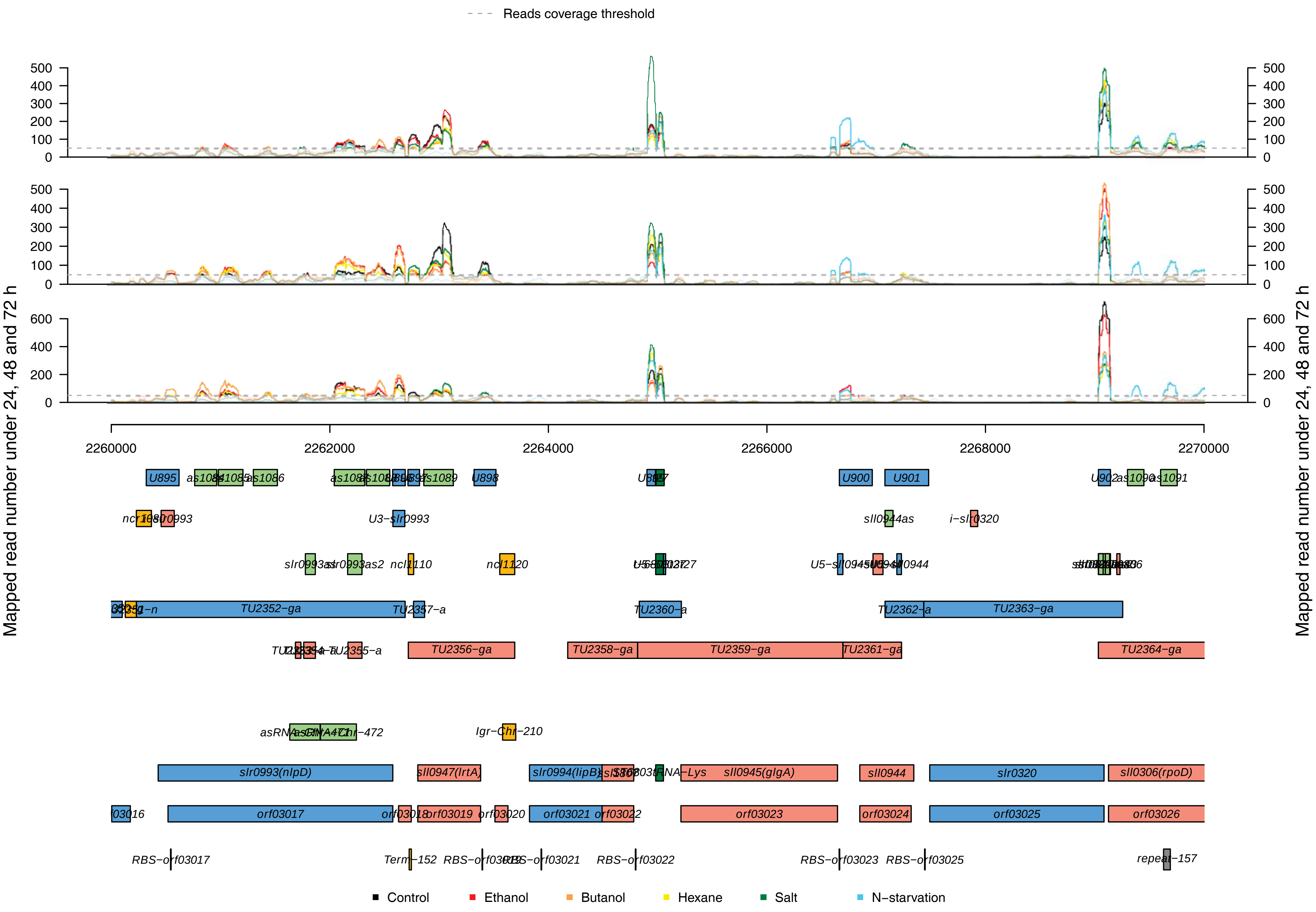
Reads coverage threshold
Mapped read number under 24, 48 and 72 h
2260000
2262000
2264000
2266000
2268000
2270000
U895
as1086
as1089
U898
U900
U901
ncl1110
ncl1120
sll0944as
i−slr0320
U3−slr0993
slr0993as2
TU2352−ga
TU2357−a
TU2360−a
TU2362−a
TU2363−ga
TU2355−a
TU2356−ga
TU2358−ga
TU2359−ga
TU2361−ga
TU2364−ga
Igr−Chr−210
slr0993(nlpD)
sll0947(lrtA)
sll0945(glgA)
sll0944
slr0320
sll0306(rpoD)
orf03017
orf03019
orf03021
orf03023
orf03024
orf03025
orf03026
RBS−orf03017
Term−152
RBS−orf03022
RBS−orf03023
RBS−orf03025
repeat−157
Control
Ethanol
Butanol
Hexane
Salt
N−starvation

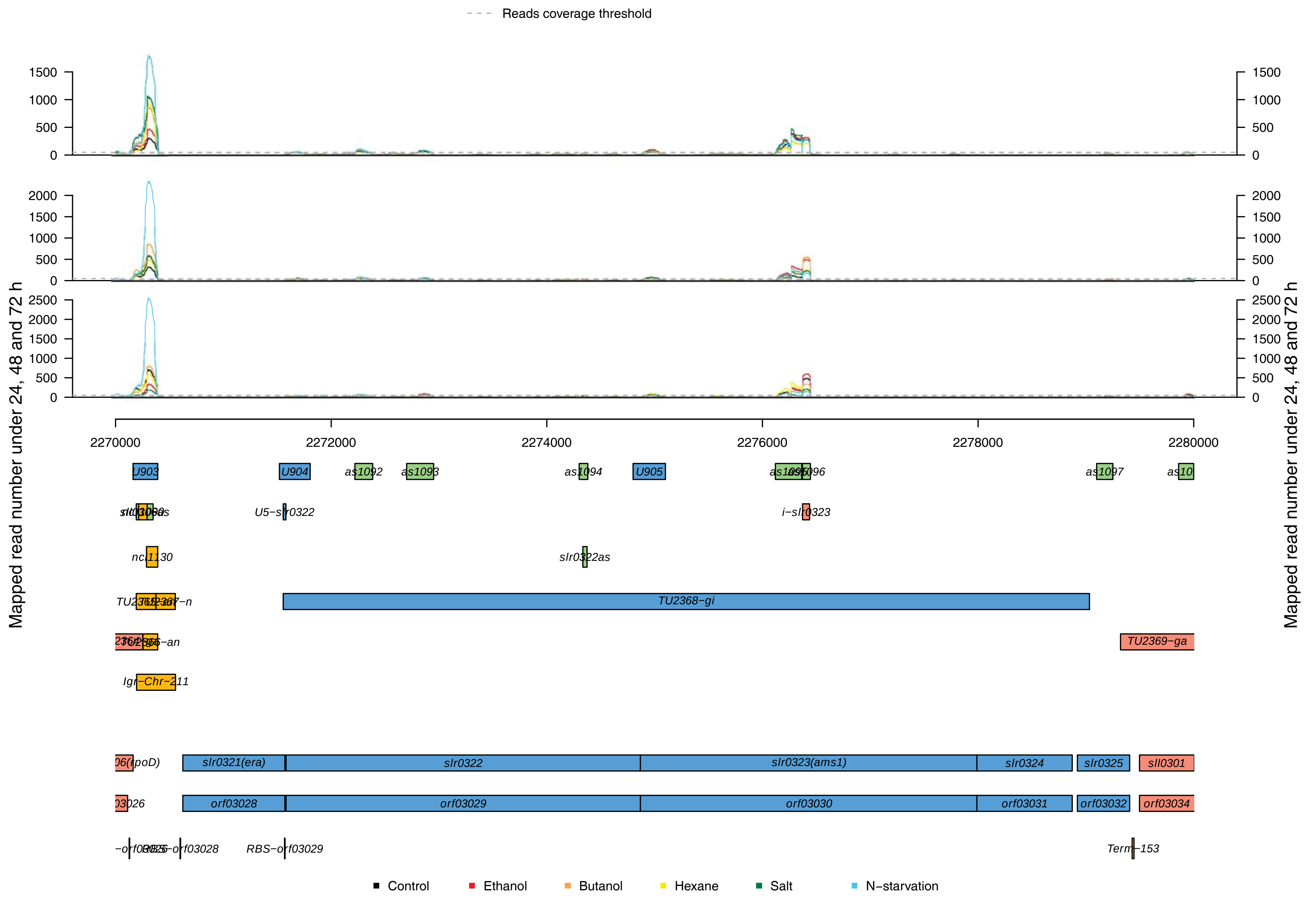

Reads coverage threshold
Mapped read number under 24, 48 and 72 h
2270000
2272000
2274000
2276000
2278000
2280000
U903
U904
as1092
as1093
as1094
U905
as1097
i−slr0323
U5−slr0322
ncl1130
slr0322as
TU2368−gi
TU2369−ga
Igr−Chr−211
slr0321(era)
slr0322
slr0323(ams1)
slr0324
slr0325
sll0301
orf03028
orf03029
orf03030
orf03031
orf03032
orf03034
RBS−orf03029
Term−153
Control
Ethanol
Butanol
Hexane
Salt
N−starvation

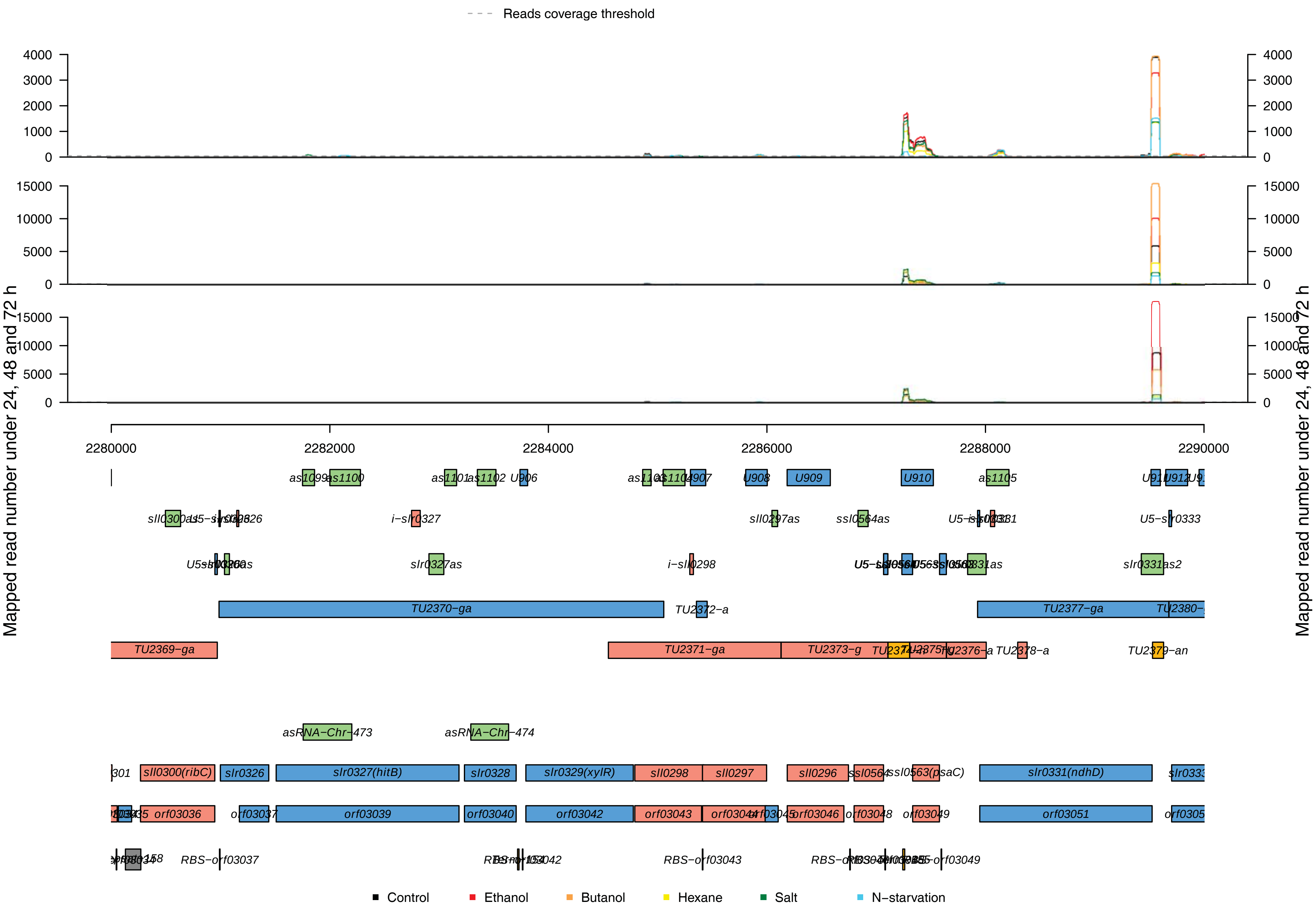

Reads coverage threshold
Mapped read number under 24, 48 and 72 h
2280000
2282000
2284000
2286000
2288000
2290000
as1100
U906
U908
U909
U910
as1105
i−slr0327
sll0297as
ssl0564as
U5−slr0333
slr0327as
i−sll0298
slr0331as2
TU2370−ga
TU2372−a
TU2377−ga
TU2369−ga
TU2371−ga
TU2373−g
TU2378−a
TU2379−an
asRNA−Chr−473
asRNA−Chr−474
sll0300(ribC)
slr0326
slr0327(hitB)
slr0328
slr0329(xylR)
sll0298
sll0297
sll0296
ssl0564
ssl0563(psaC)
slr0331(ndhD)
orf03036
orf03037
orf03039
orf03040
orf03042
orf03043
orf03046
orf03048
orf03049
orf03051
RBS−orf03037
RBS−orf03043
RBS−orf03049
Control
Ethanol
Butanol
Hexane
Salt
N−starvation

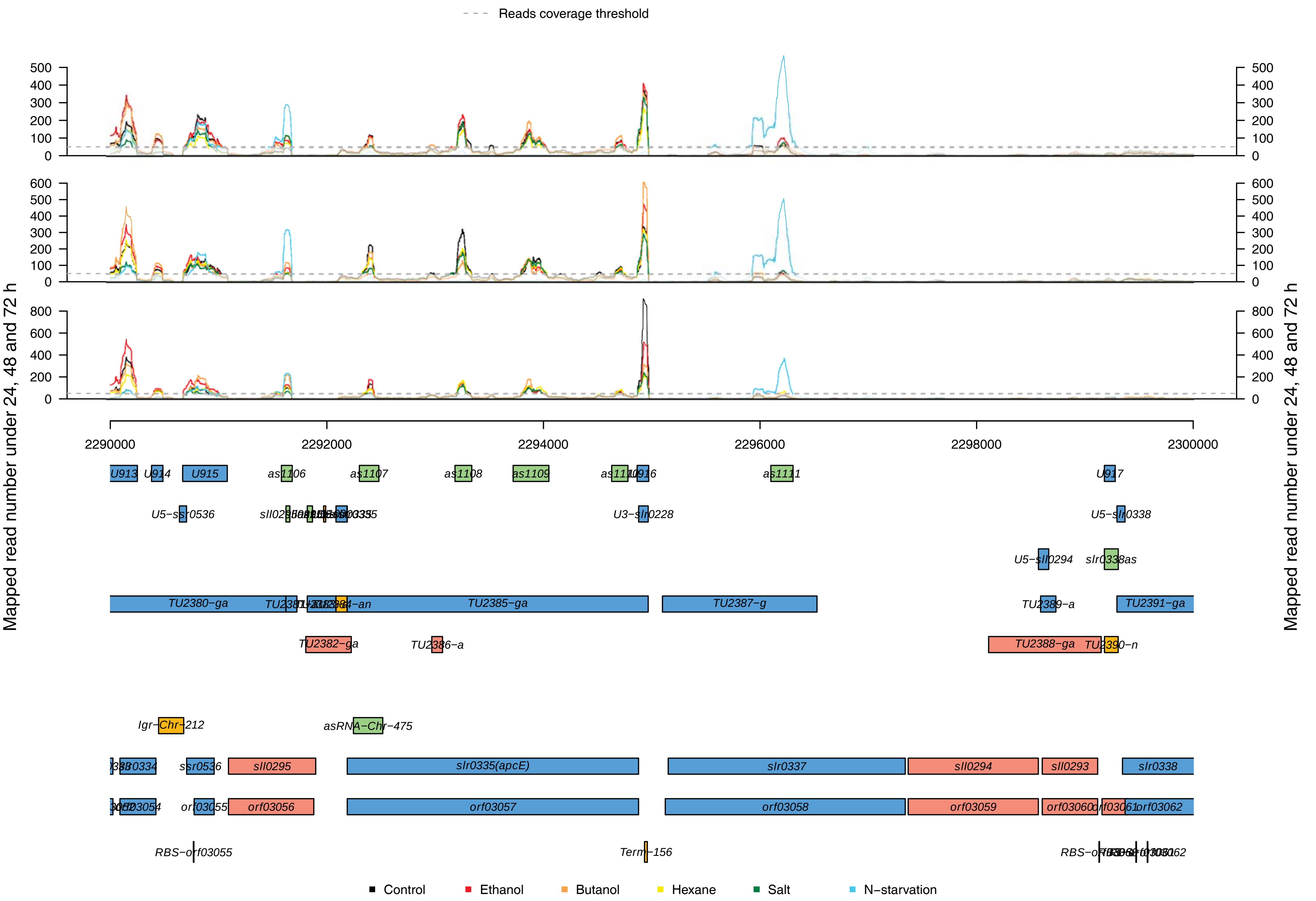

Reads coverage threshold
Mapped read number under 24, 48 and 72 h
0
100
200
300
400
500
600
800
2290000
2292000
2294000
2296000
2298000
2300000
U913
U914
U915
as1106
as1107
as1108
as1109
U916
as1111
U917
U5−ssr0536
U3−slr0228
U5−slr0338
U5−sll0294
slr0338as
TU2380−ga
TU2385−ga
TU2387−g
TU2389−a
TU2391−ga
TU2382−ga
TU2386−a
TU2388−ga
TU2390−n
Igr−Chr−212
asRNA−Chr−475
slr0334
ssr0536
sll0295
slr0335(apcE)
slr0337
sll0294
sll0293
slr0338
orf03054
orf03055
orf03056
orf03057
orf03058
orf03059
orf03060
orf03062
RBS−orf03055
Term−156
Control
Ethanol
Butanol
Hexane
Salt
N−starvation

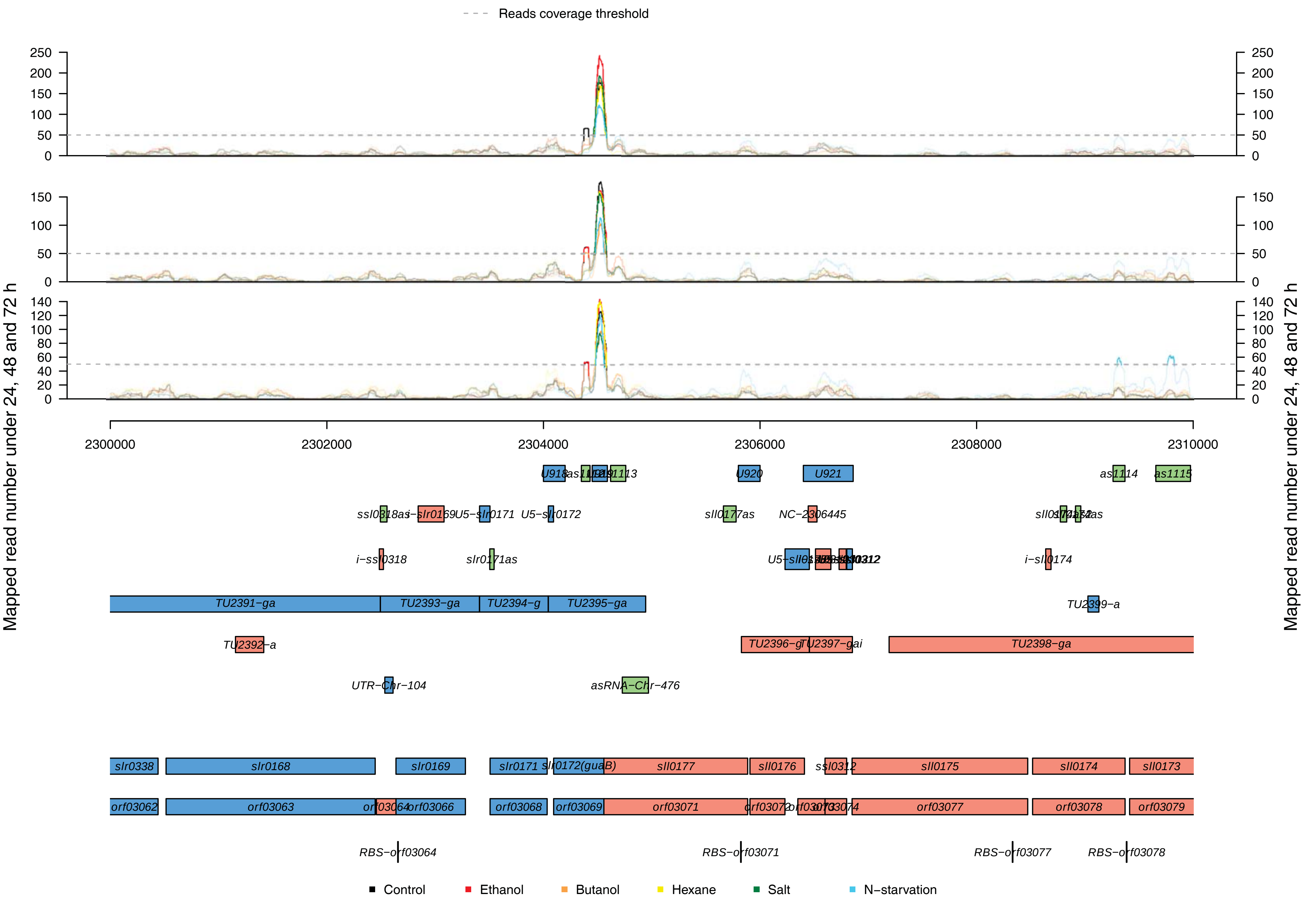

Reads coverage threshold
Mapped read number under 24, 48 and 72 h
2300000
2302000
2304000
2306000
2308000
2310000
U918
U920
U921
as1114
as1115
ssl0318as
U5−slr0171
U5−slr0172
sll0177as
NC−2306445
i−ssl0318
slr0171as
i−sll0174
TU2391−ga
TU2393−ga
TU2394−g
TU2395−ga
TU2399−a
TU2392−a
TU2398−ga
UTR−Chr−104
asRNA−Chr−476
slr0338
slr0168
slr0169
slr0171
slr0172(guaB)
sll0177
sll0176
ssl0312
sll0175
sll0174
sll0173
orf03062
orf03063
orf03066
orf03068
orf03069
orf03071
orf03077
orf03078
orf03079
RBS−orf03064
RBS−orf03071
RBS−orf03077
RBS−orf03078
Control
Ethanol
Butanol
Hexane
Salt
N−starvation

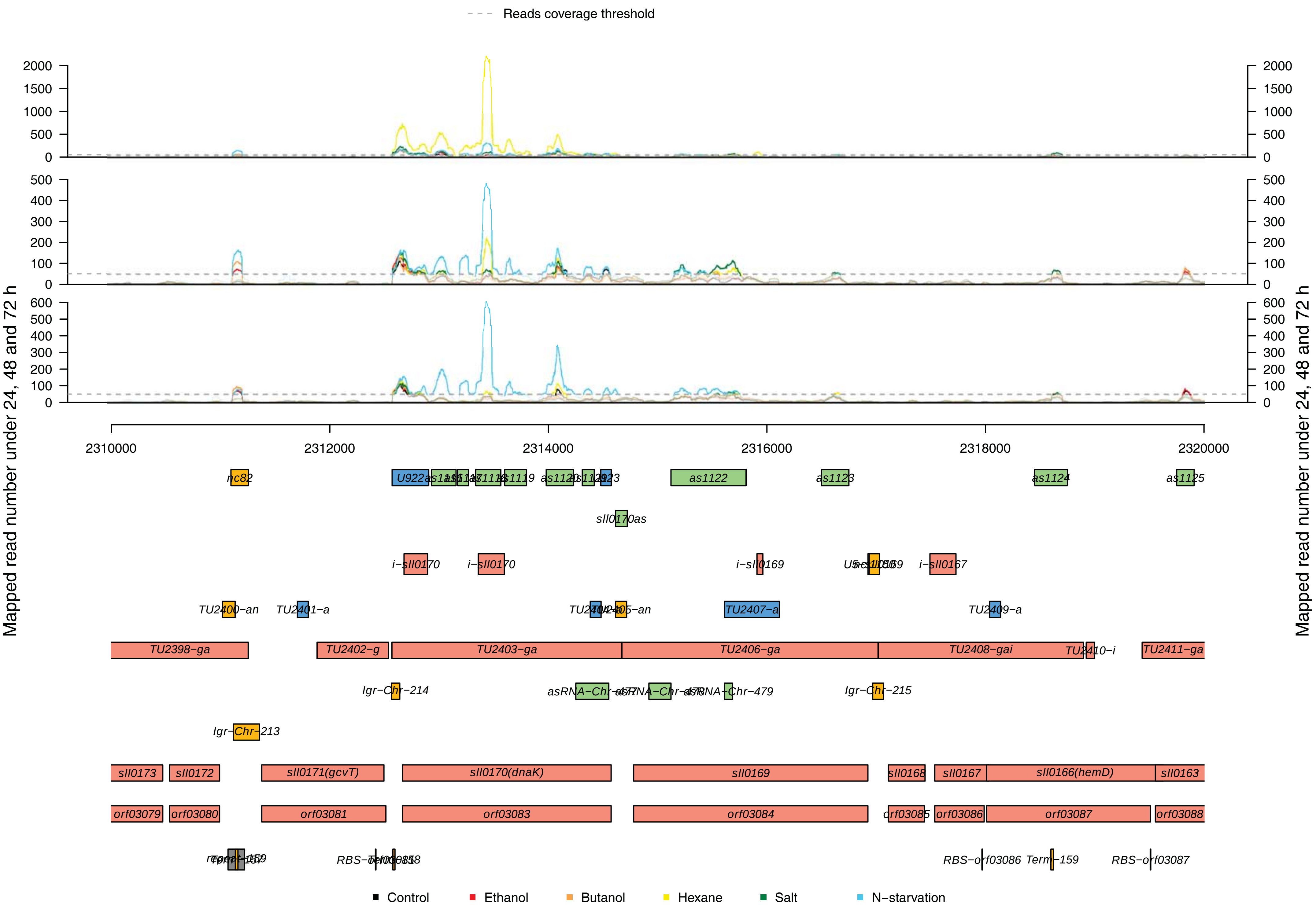
Reads coverage threshold
Mapped read number under 24, 48 and 72 h
2310000
2312000
2314000
2316000
2318000
2320000
nc82
U922a
as1122
as1123
as1124
as1125
sll0170as
i−sll0170
i−sll0169
i−sll0167
TU2400−an
TU2401−a
TU2407−a
TU2409−a
TU2398−ga
TU2402−g
TU2403−ga
TU2406−ga
TU2408−gai
TU2411−ga
Igr−Chr−214
Igr−Chr−215
Igr−Chr−213
sll0173
sll0172
sll0171(gcvT)
sll0170(dnaK)
sll0169
sll0168
sll0167
sll0166(hemD)
sll0163
orf03079
orf03080
orf03081
orf03083
orf03084
orf03085
orf03086
orf03087
orf03088
RBS−orf03086
Term−159
RBS−orf03087
Control
Ethanol
Butanol
Hexane
Salt
N−starvation

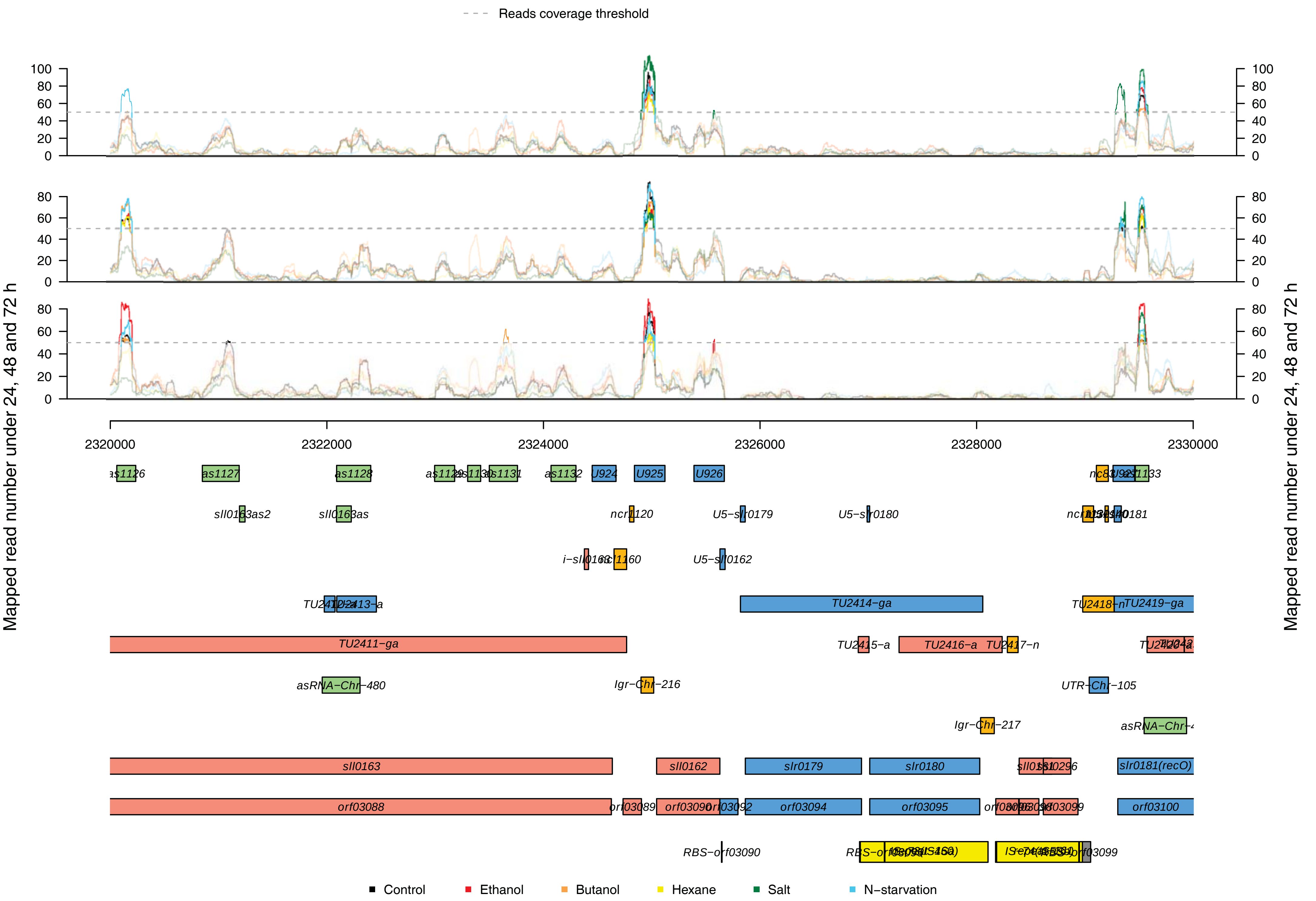

Reads coverage threshold
Mapped read number under 24, 48 and 72 h
2320000
2322000
2324000
2326000
2328000
2330000
TU2414–ga
TU2411–ga
TU2416–a
TU2419–ga
asRNA–Chr–480
Igr–Chr–216
Igr–Chr–217
UTR–Chr–105
sll0163
sll0162
slr0179
slr0180
orf03088
orf03094
orf03095
orf03100
RBS–orf03090
Control
Ethanol
Butanol
Hexane
Salt
N–starvation

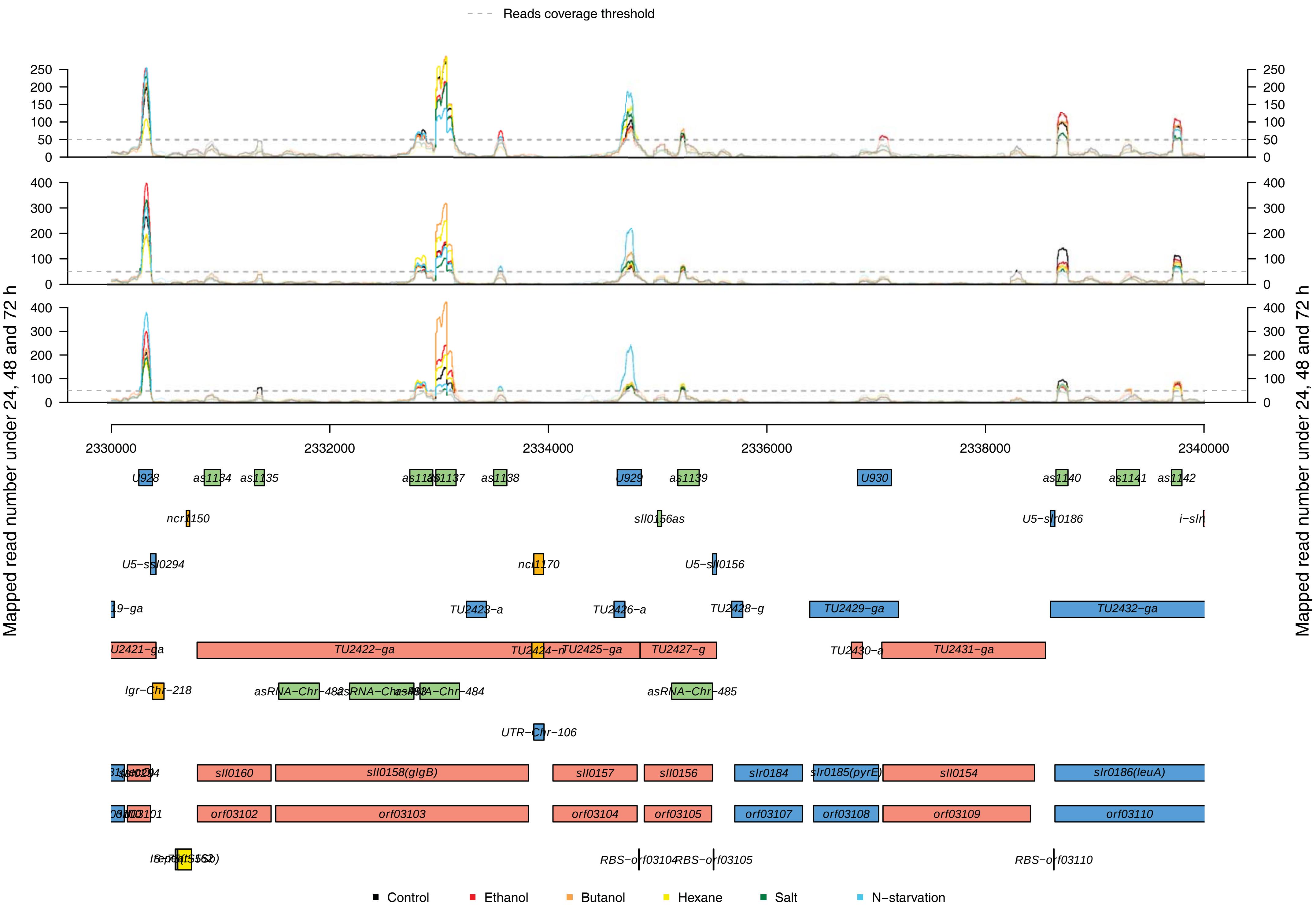

Reads coverage threshold
Mapped read number under 24, 48 and 72 h
2330000
2332000
2334000
2336000
2338000
2340000
U928
as1134
as1135
as1137
as1138
U929
as1139
U930
as1140
as1141
as1142
ncr1150
sll0156as
U5−slr0186
U5−ssl0294
ncl1170
U5−sll0156
TU2423−a
TU2426−a
TU2428−g
TU2429−ga
TU2432−ga
TU2422−ga
TU2425−ga
TU2427−g
TU2431−ga
Igr−Chr−218
asRNA−Chr−485
UTR−Chr−106
sll0160
sll0158(glgB)
sll0157
sll0156
slr0184
slr0185(pyrE)
sll0154
slr0186(leuA)
orf03102
orf03103
orf03104
orf03105
orf03107
orf03108
orf03109
orf03110
RBS−orf03110
Control
Ethanol
Butanol
Hexane
Salt
N−starvation

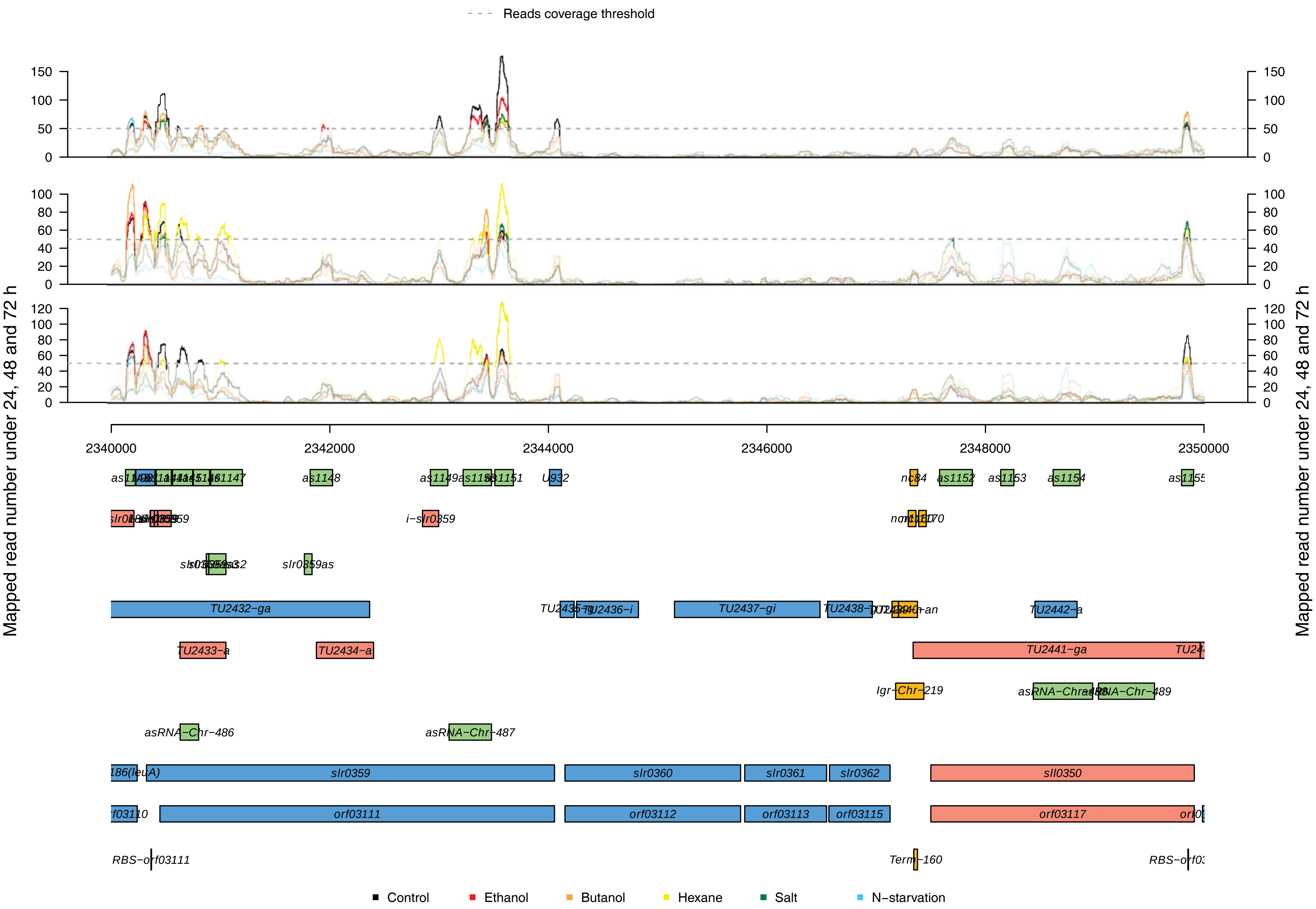
Reads coverage threshold
Mapped read number under 24, 48 and 72 h
2340000
2342000
2344000
2346000
2348000
2350000
as1148
as1152
as1153
as1154
i−slr0359
slr0359as
TU2432−ga
TU2436−i
TU2437−gi
TU2442−a
TU2433−a
TU2434−a
TU2441−ga
Igr−Chr−219
asRNA−Chr−486
asRNA−Chr−487
slr0359
slr0360
slr0361
slr0362
sll0350
orf03111
orf03112
orf03113
orf03115
orf03117
RBS−orf03111
Term−160
Control
Ethanol
Butanol
Hexane
Salt
N−starvation

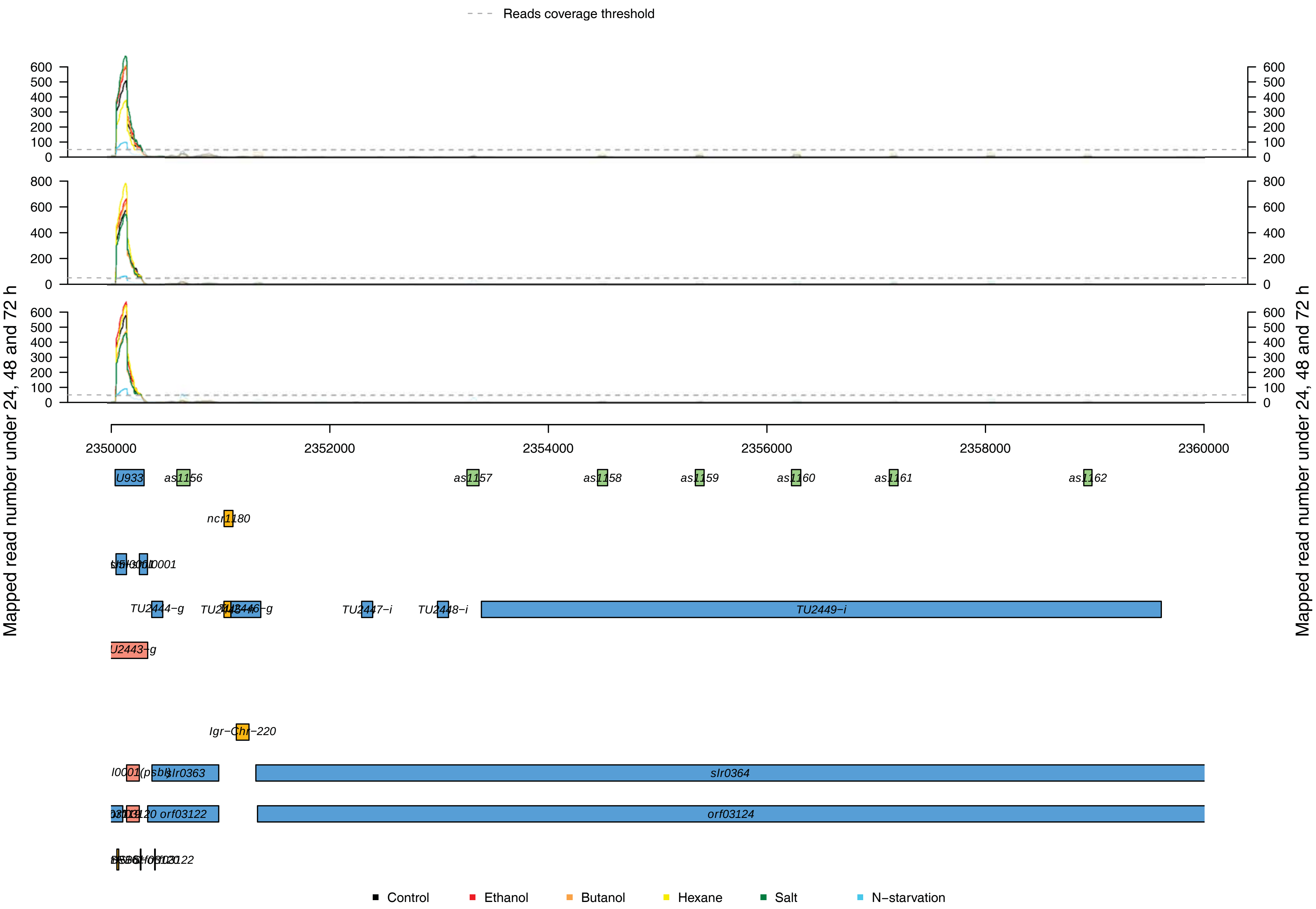

Reads coverage threshold
Mapped read number under 24, 48 and 72 h
2350000
2352000
2354000
2356000
2358000
2360000
as1156
as1157
as1158
as1159
as1160
as1161
as1162
ncr1180
TU2444−g
TU2447−i
TU2448−i
TU2449−i
Igr−Chr−220
slr0363
slr0364
orf03122
orf03124
Control
Ethanol
Butanol
Hexane
Salt
N−starvation

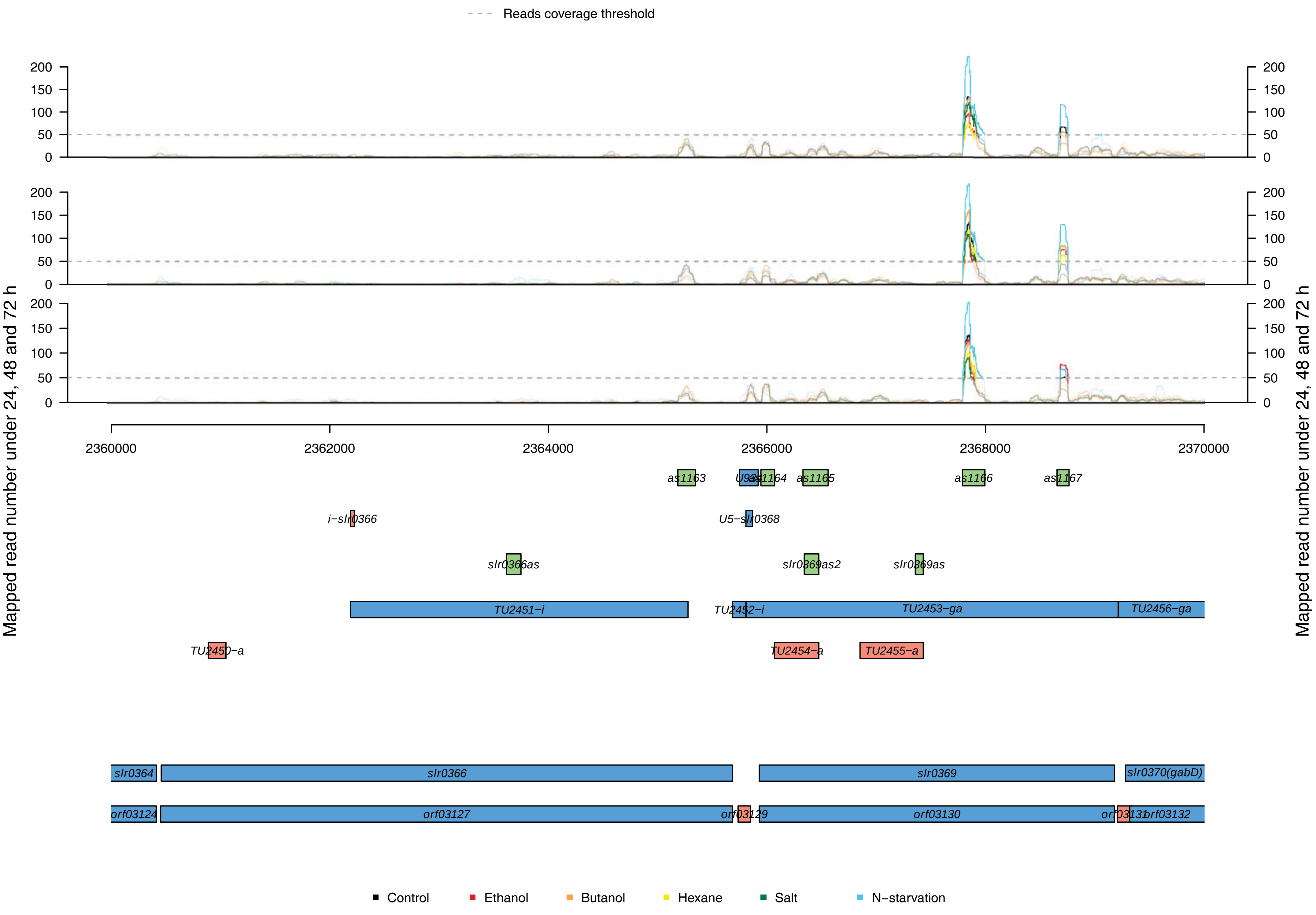

Reads coverage threshold
Mapped read number under 24, 48 and 72 h
2360000
2362000
2364000
2366000
2368000
2370000
as1163
as1164
as1165
as1166
as1167
i−slr0366
U5−slr0368
slr0366as
slr0369as2
slr0369as
TU2451−i
TU2452−i
TU2453−ga
TU2456−ga
TU2450−a
TU2454−a
TU2455−a
slr0364
slr0366
slr0369
slr0370(gabD)
orf03124
orf03127
orf03129
orf03130
orf03131
orf03132
Control
Ethanol
Butanol
Hexane
Salt
N−starvation

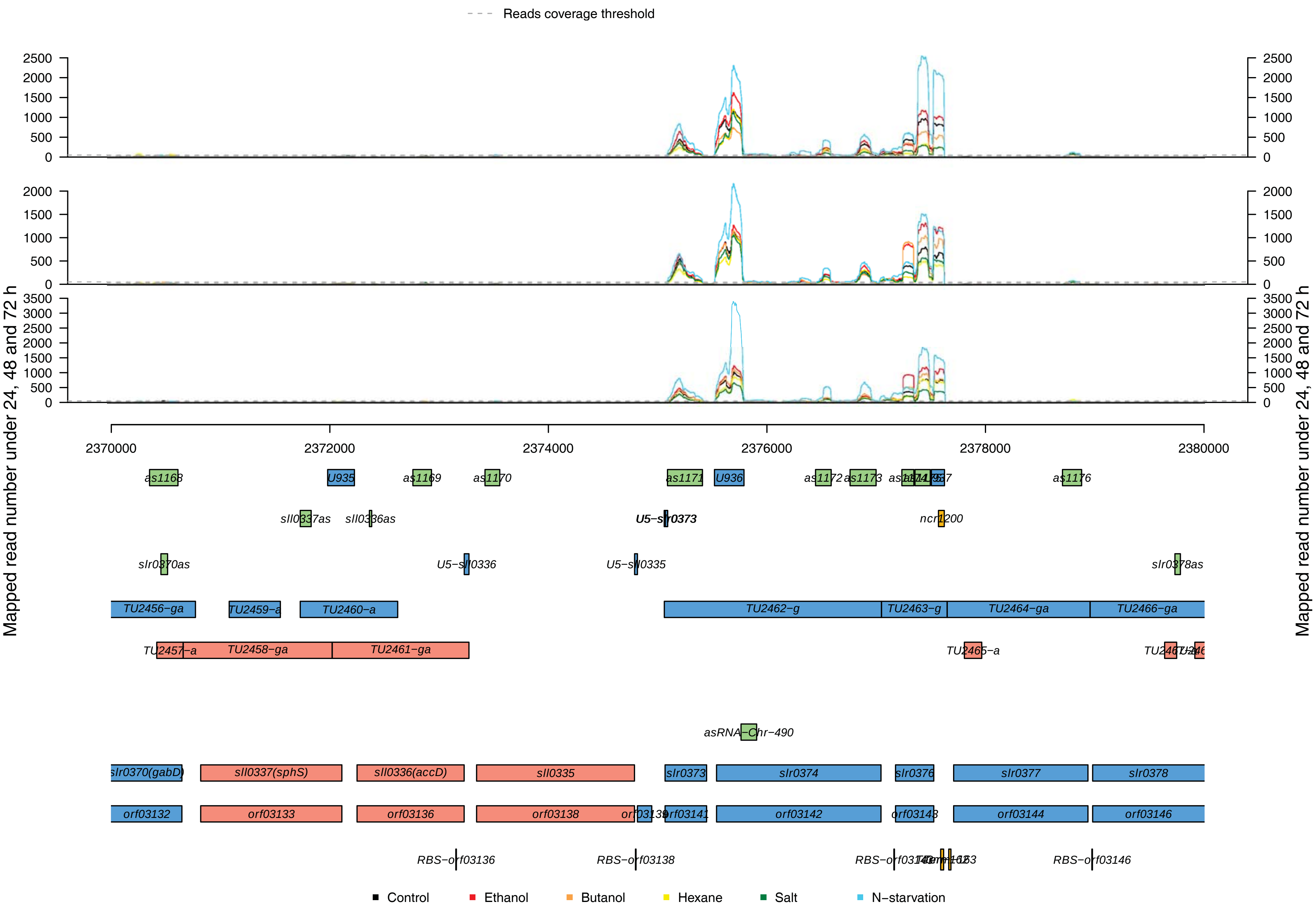

Reads coverage threshold
Mapped read number under 24, 48 and 72 h
2370000
2372000
2374000
2376000
2378000
2380000
as1168
U935
as1169
as1170
as1171
U936
as1172
as1173
as1176
sll0337as
sll0336as
U5−slr0373
ncr1200
slr0370as
U5−sll0336
U5−sll0335
slr0378as
TU2456−ga
TU2459−a
TU2460−a
TU2462−g
TU2463−g
TU2464−ga
TU2466−ga
TU2457−a
TU2458−ga
TU2461−ga
TU2465−a
asRNA−Chr−490
slr0370(gabD)
sll0337(sphS)
sll0336(accD)
sll0335
slr0373
slr0374
slr0376
slr0377
slr0378
orf03132
orf03133
orf03136
orf03138
orf03141
orf03142
orf03143
orf03144
orf03146
RBS−orf03136
RBS−orf03138
RBS−orf03146
Control
Ethanol
Butanol
Hexane
Salt
N−starvation

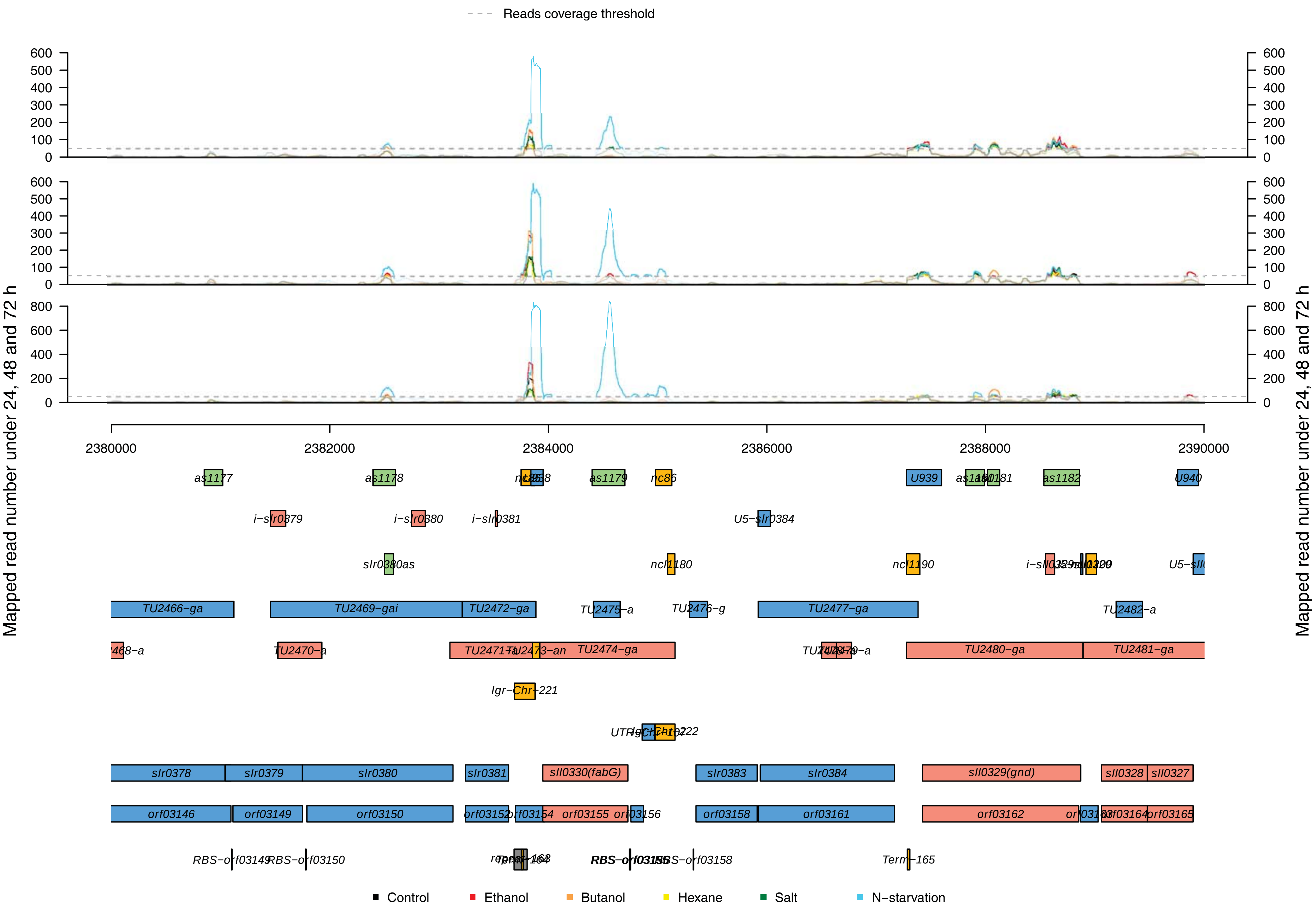

Reads coverage threshold
Mapped read number under 24, 48 and 72 h
600
500
400
300
200
100
0
800
2380000
2382000
2384000
2386000
2388000
2390000
as1177
as1178
as1179
nc86
U939
as1182
U940
i−slr0379
i−slr0380
i−slr0381
U5−slr0384
slr0380as
ncl1180
ncl1190
TU2466−ga
TU2469−gai
TU2472−ga
TU2475−a
TU2476−g
TU2477−ga
TU2482−a
TU2470−a
TU2474−ga
TU2480−ga
TU2481−ga
Igr−Chr−221
slr0378
slr0379
slr0380
slr0381
sll0330(fabG)
slr0383
slr0384
sll0329(gnd)
sll0328
sll0327
orf03146
orf03149
orf03150
orf03155
orf03158
orf03161
orf03162
orf03165
RBS−orf03150
RBS−orf03158
Term−165
Control
Ethanol
Butanol
Hexane
Salt
N−starvation

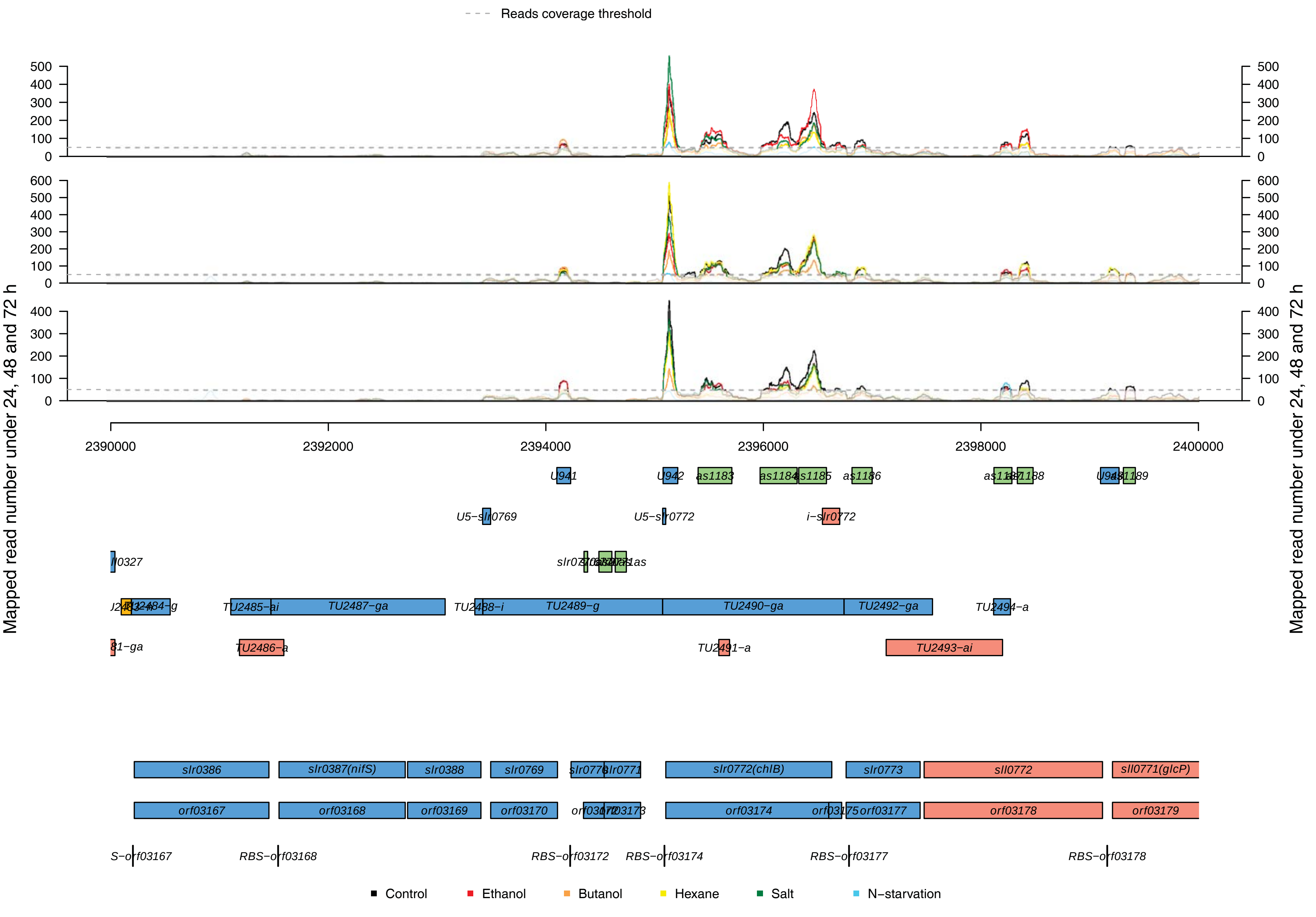

Reads coverage threshold
Mapped read number under 24, 48 and 72 h
2390000
2392000
2394000
2396000
2398000
2400000
U941
U942
as1183
as1184
as1185
as1186
as1187
as1188
U943
as1189
U5−slr0769
U5−slr0772
i−slr0772
TU2485−ai
TU2487−ga
TU2488−i
TU2489−g
TU2490−ga
TU2492−ga
TU2494−a
TU2486−a
TU2491−a
TU2493−ai
slr0386
slr0387(nifS)
slr0388
slr0769
slr0770
slr0771
slr0772(chlB)
slr0773
sll0772
sll0771(glcP)
orf03167
orf03168
orf03169
orf03170
orf03174
orf03177
orf03178
orf03179
RBS−orf03168
RBS−orf03172
RBS−orf03174
RBS−orf03177
RBS−orf03178
Control
Ethanol
Butanol
Hexane
Salt
N−starvation

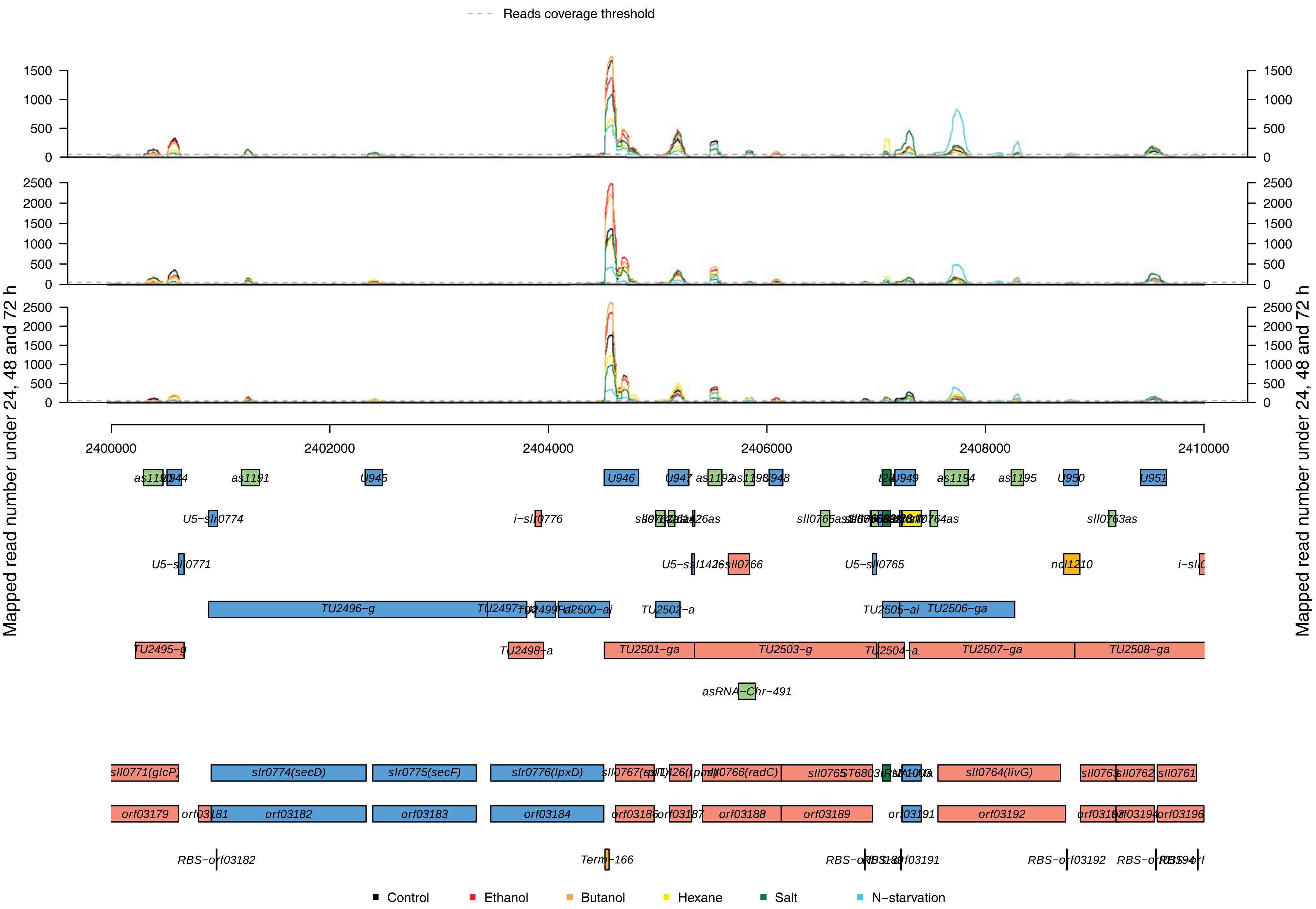

Reads coverage threshold
Mapped read number under 24, 48 and 72 h
2400000
2402000
2404000
2406000
2408000
2410000
U945
U946
U947
U949
U950
U951
as1191
as1194
as1195
U5−slr0774
i−slr0776
U5−sll0771
U5−sll0765
TU2496−g
TU2502−a
TU2506−ga
TU2495−g
TU2498−a
TU2501−ga
TU2503−g
TU2507−ga
TU2508−ga
asRNA−Chr−491
sll0771(glcP)
slr0774(secD)
slr0775(secF)
slr0776(lpxD)
sll0764(livG)
sll0762
sll0761
orf03179
orf03182
orf03183
orf03184
orf03188
orf03189
orf03192
RBS−orf03182
RBS−orf03192
Control
Ethanol
Butanol
Hexane
Salt
N−starvation

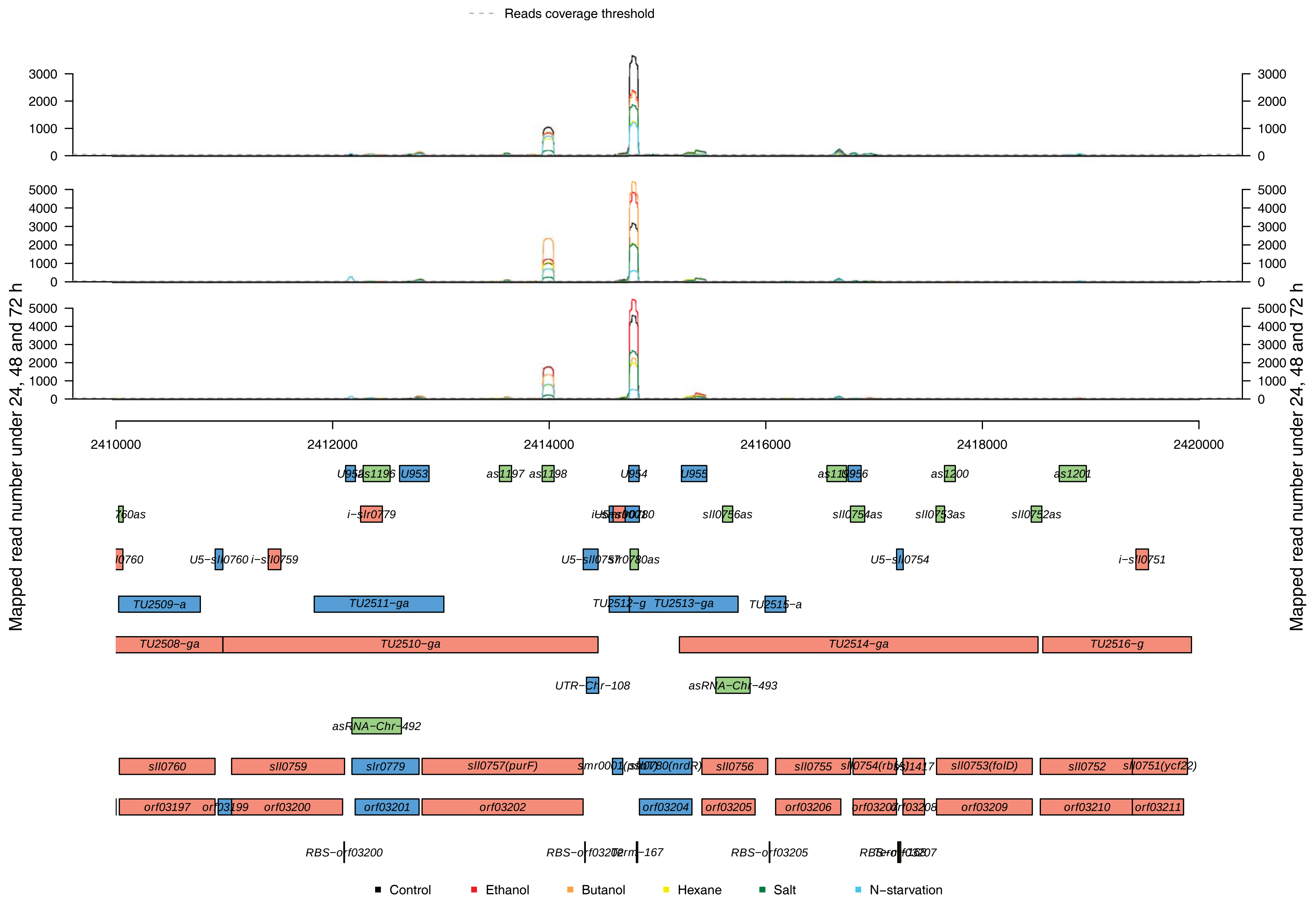

Reads coverage threshold
Mapped read number under 24, 48 and 72 h
2410000
2412000
2414000
2416000
2418000
2420000
TU2509-a
TU2511-ga
TU2513-ga
TU2508-ga
TU2510-ga
TU2514-ga
TU2516-g
UTR-Chr-108
asRNA-Chr-493
asRNA-Chr-492
sll0760
sll0759
slr0779
sll0757(purF)
sll0756
sll0755
sll0753(folD)
sll0752
sll0751(ycf22)
orf03197
orf03200
orf03201
orf03202
orf03204
orf03205
orf03206
orf03209
orf03210
orf03211
RBS-orf03200
RBS-orf03205
Control
Ethanol
Butanol
Hexane
Salt
N-starvation

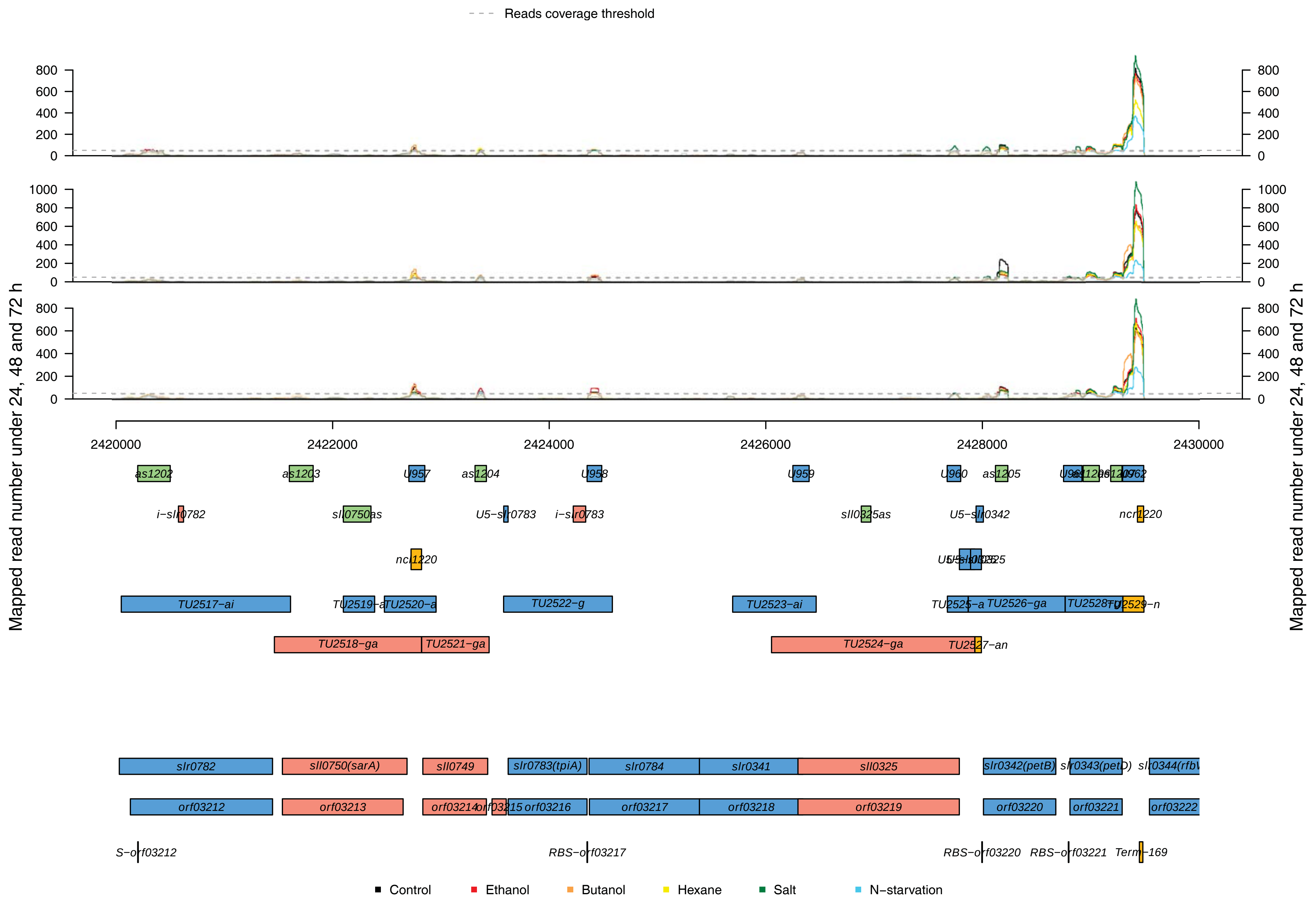

Reads coverage threshold
Mapped read number under 24, 48 and 72 h
2420000
2422000
2424000
2426000
2428000
2430000
as1202
as1203
U957
as1204
U958
U959
U960
as1205
i−sll0782
sll0750as
U5−slr0783
i−slr0783
sll0325as
U5−slr0342
ncr1220
ncl1220
TU2517−ai
TU2519−a
TU2520−a
TU2522−g
TU2523−ai
TU2526−ga
TU2518−ga
TU2521−ga
TU2524−ga
slr0782
sll0750(sarA)
sll0749
slr0783(tpiA)
slr0784
slr0341
sll0325
slr0342(petB)
slr0343(petD)
orf03212
orf03213
orf03216
orf03217
orf03218
orf03219
orf03220
orf03221
orf03222
RBS−orf03217
RBS−orf03220
RBS−orf03221
Term−169
Control
Ethanol
Butanol
Hexane
Salt
N−starvation

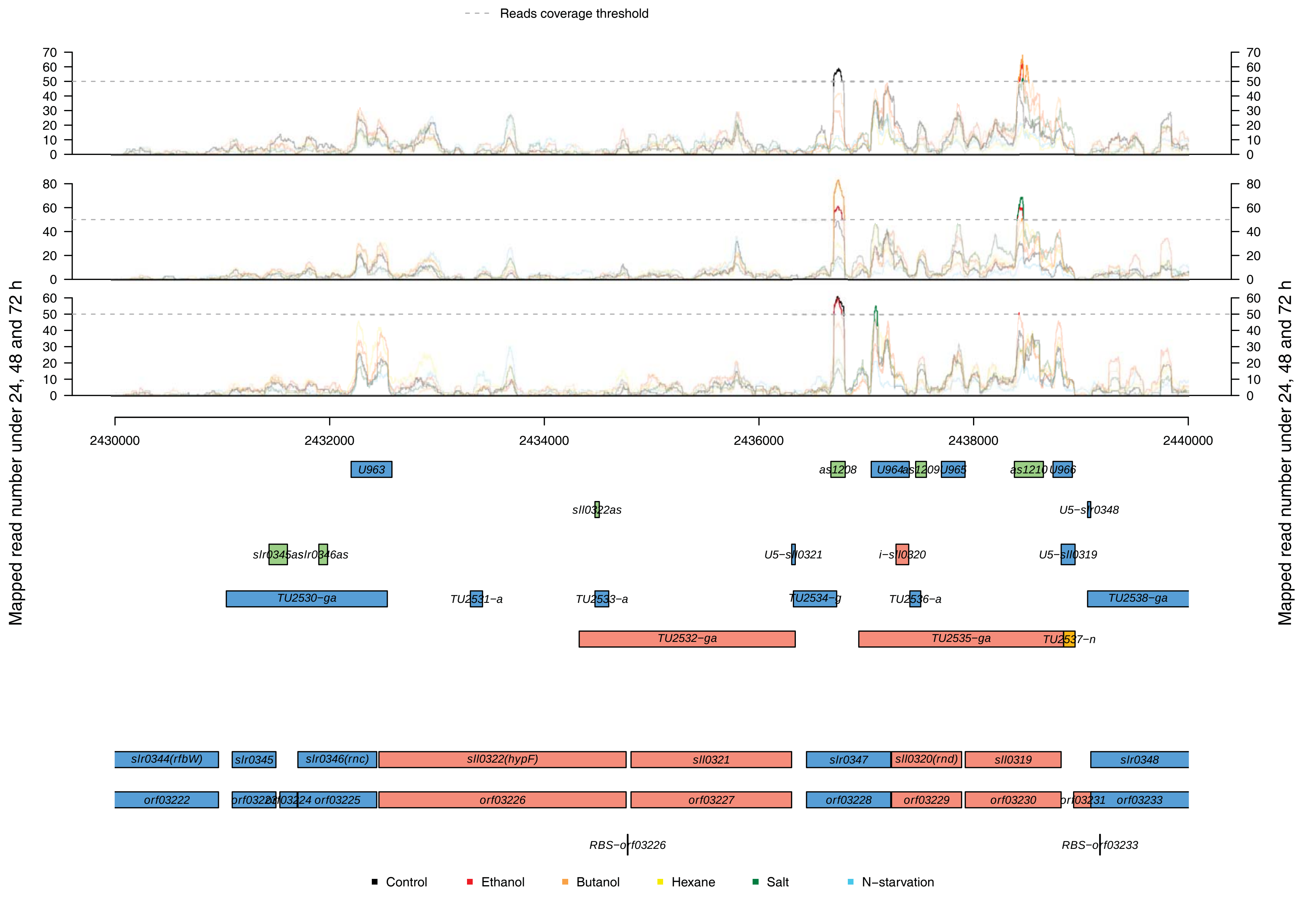

Reads coverage threshold
Mapped read number under 24, 48 and 72 h
2430000
2432000
2434000
2436000
2438000
2440000
U963
as1208
U964
as1209
U965
as1210
U966
sll0322as
U5−slr0348
slr0345as
slr0346as
U5−sll0321
i−sll0320
U5−sll0319
TU2530−ga
TU2531−a
TU2533−a
TU2534−g
TU2536−a
TU2538−ga
TU2532−ga
TU2535−ga
TU2537−n
slr0344(rfbW)
slr0345
slr0346(rnc)
sll0322(hypF)
sll0321
slr0347
sll0320(rnd)
sll0319
slr0348
orf03222
orf03223
orf03224
orf03225
orf03226
orf03227
orf03228
orf03229
orf03230
orf03231
orf03233
RBS−orf03226
RBS−orf03233
Control
Ethanol
Butanol
Hexane
Salt
N−starvation

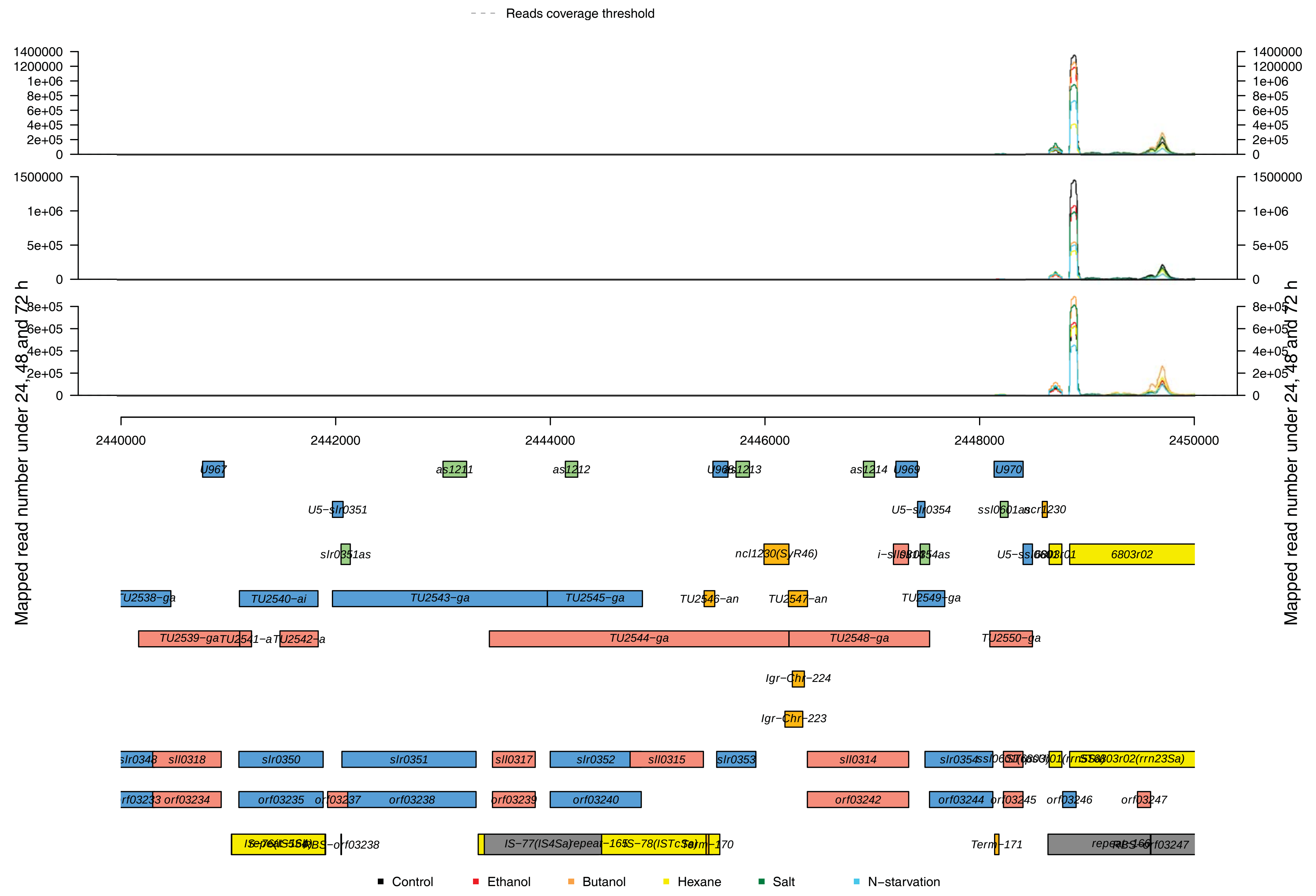

Reads coverage threshold
Mapped read number under 24, 48 and 72 h
1400000
1200000
1e+06
8e+05
6e+05
4e+05
2e+05
0
1500000
5e+05
2440000
2442000
2444000
2446000
2448000
2450000
U967
as1211
as1212
as1213
as1214
U969
U970
U5−slr0351
U5−slr0354
ncl1230
slr0351as
ncl1230(SyR46)
6803r02
TU2538−ga
TU2540−ai
TU2543−ga
TU2545−ga
TU2546−an
TU2547−an
TU2549−ga
TU2539−ga
TU2542−a
TU2544−ga
TU2548−ga
TU2550−ga
Igr−Chr−224
Igr−Chr−223
slr0348
sll0318
slr0350
slr0351
sll0317
slr0352
sll0315
slr0353
sll0314
slr0354
orf03234
orf03235
orf03237
orf03238
orf03239
orf03240
orf03242
orf03244
orf03245
orf03246
orf03247
IS−77(IS4Sa)
Term−170
Term−171
Control
Ethanol
Butanol
Hexane
Salt
N−starvation

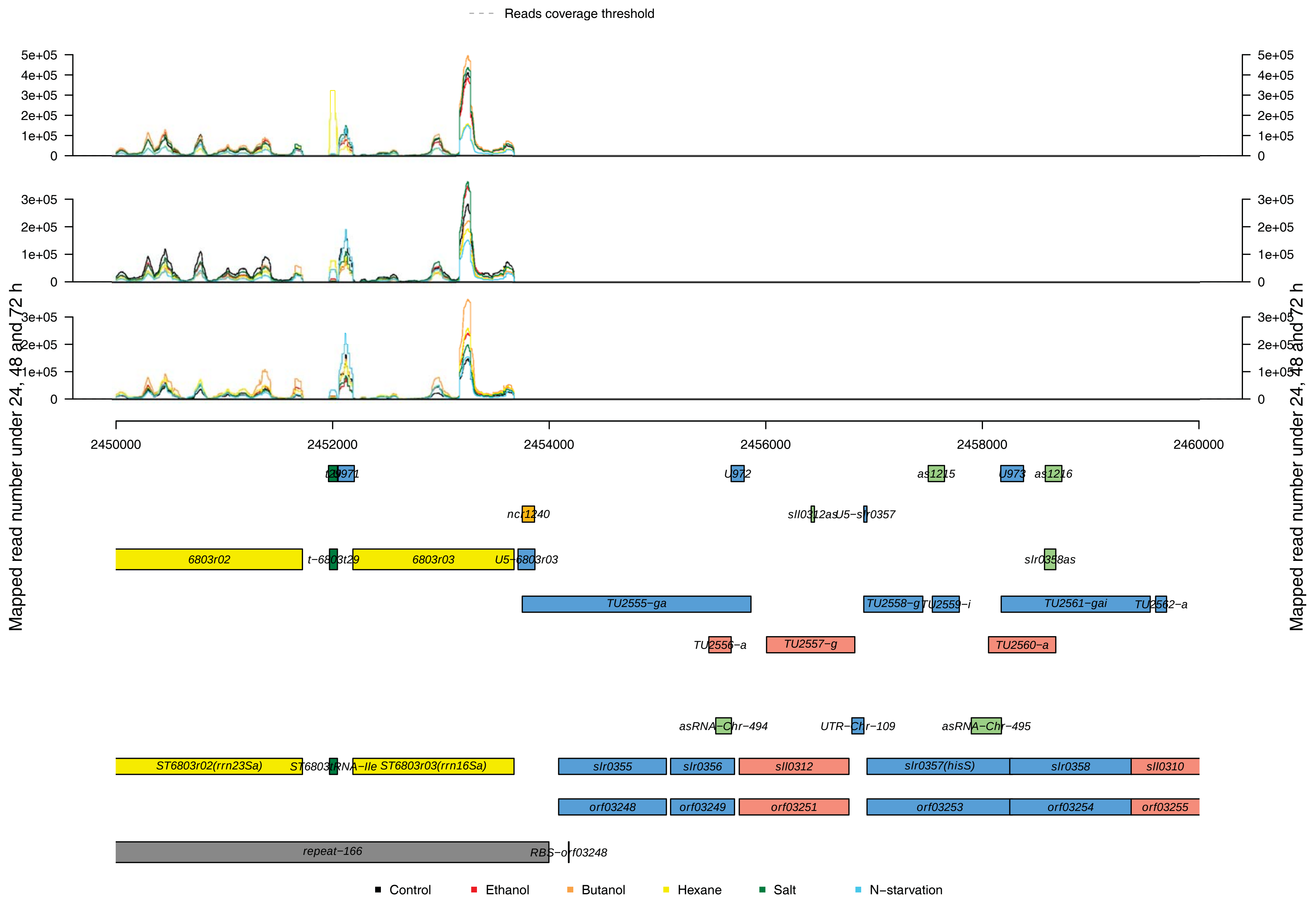

Reads coverage threshold
Mapped read number under 24, 48 and 72 h
5e+05
4e+05
3e+05
2e+05
1e+05
0
2450000
2452000
2454000
2456000
2458000
2460000
6803r02
t−6803t29
6803r03
U5−6803r03
ncl1240
TU2555−ga
TU2556−a
TU2557−g
TU2558−g
TU2559−i
TU2560−a
TU2561−gai
TU2562−a
U972
U973
as1215
as1216
sll0312as
U5−slr0357
slr0358as
asRNA−Chr−494
UTR−Chr−109
asRNA−Chr−495
ST6803r02(rrn23Sa)
ST6803tRNA−Ile
ST6803r03(rrn16Sa)
slr0355
slr0356
sll0312
slr0357(hisS)
slr0358
sll0310
orf03248
orf03249
orf03251
orf03253
orf03254
orf03255
repeat−166
RBS−orf03248
Control
Ethanol
Butanol
Hexane
Salt
N−starvation

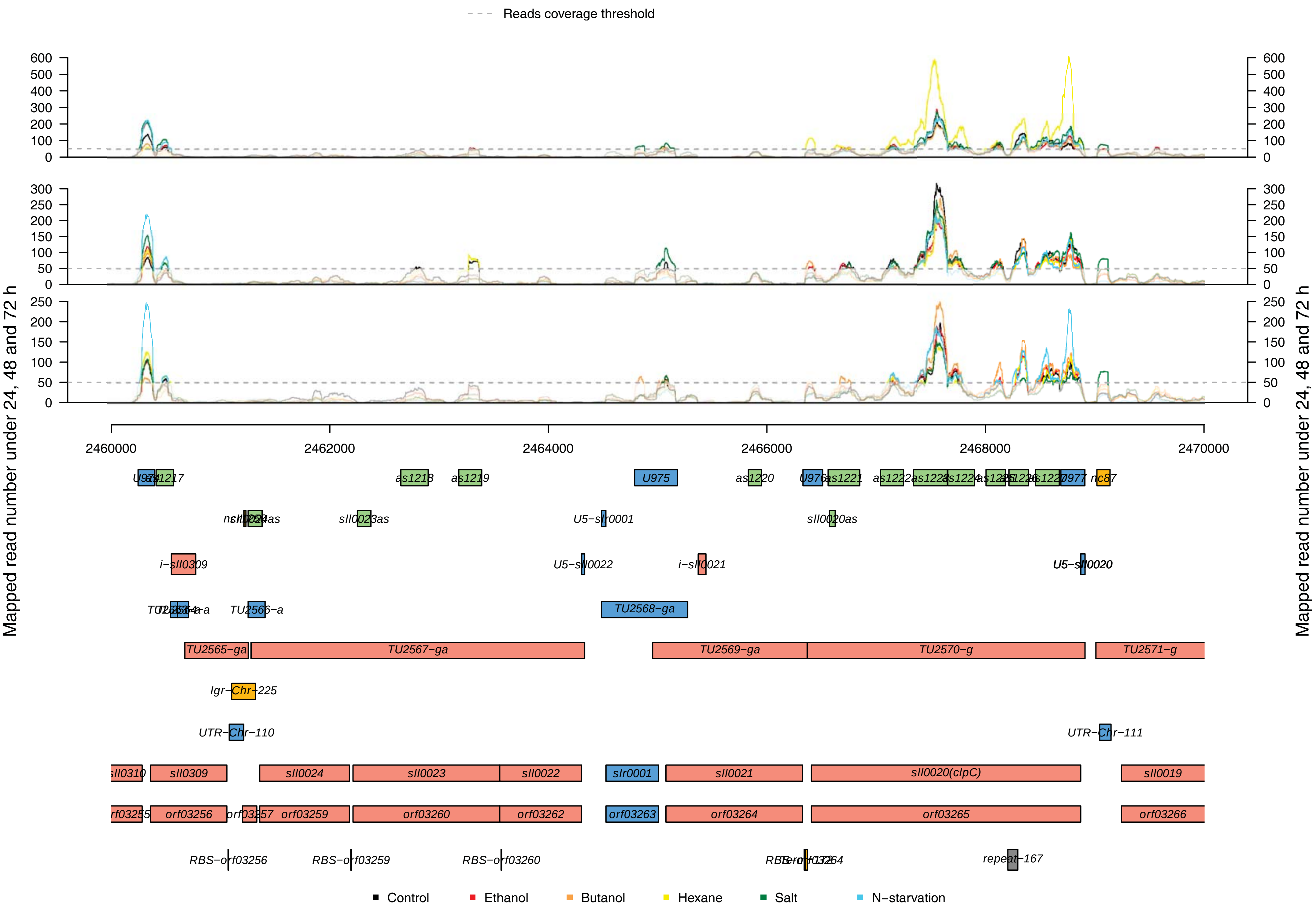
Reads coverage threshold
Mapped read number under 24, 48 and 72 h
2460000
2462000
2464000
2466000
2468000
2470000
U974
as1217
as1218
as1219
U975
as1220
U976
as1221
nc87
sll0023as
U5−slr0001
sll0020as
i−sll0309
U5−sll0022
i−sll0021
U5−sll0020
TU2566−a
TU2568−ga
TU2565−ga
TU2567−ga
TU2569−ga
TU2570−g
TU2571−g
Igr−Chr−225
UTR−Chr−110
UTR−Chr−111
sll0309
sll0024
sll0023
sll0022
slr0001
sll0021
sll0020(clpC)
sll0019
orf03256
orf03259
orf03260
orf03262
orf03263
orf03264
orf03265
orf03266
RBS−orf03256
RBS−orf03259
RBS−orf03260
repeat−167
Control
Ethanol
Butanol
Hexane
Salt
N−starvation

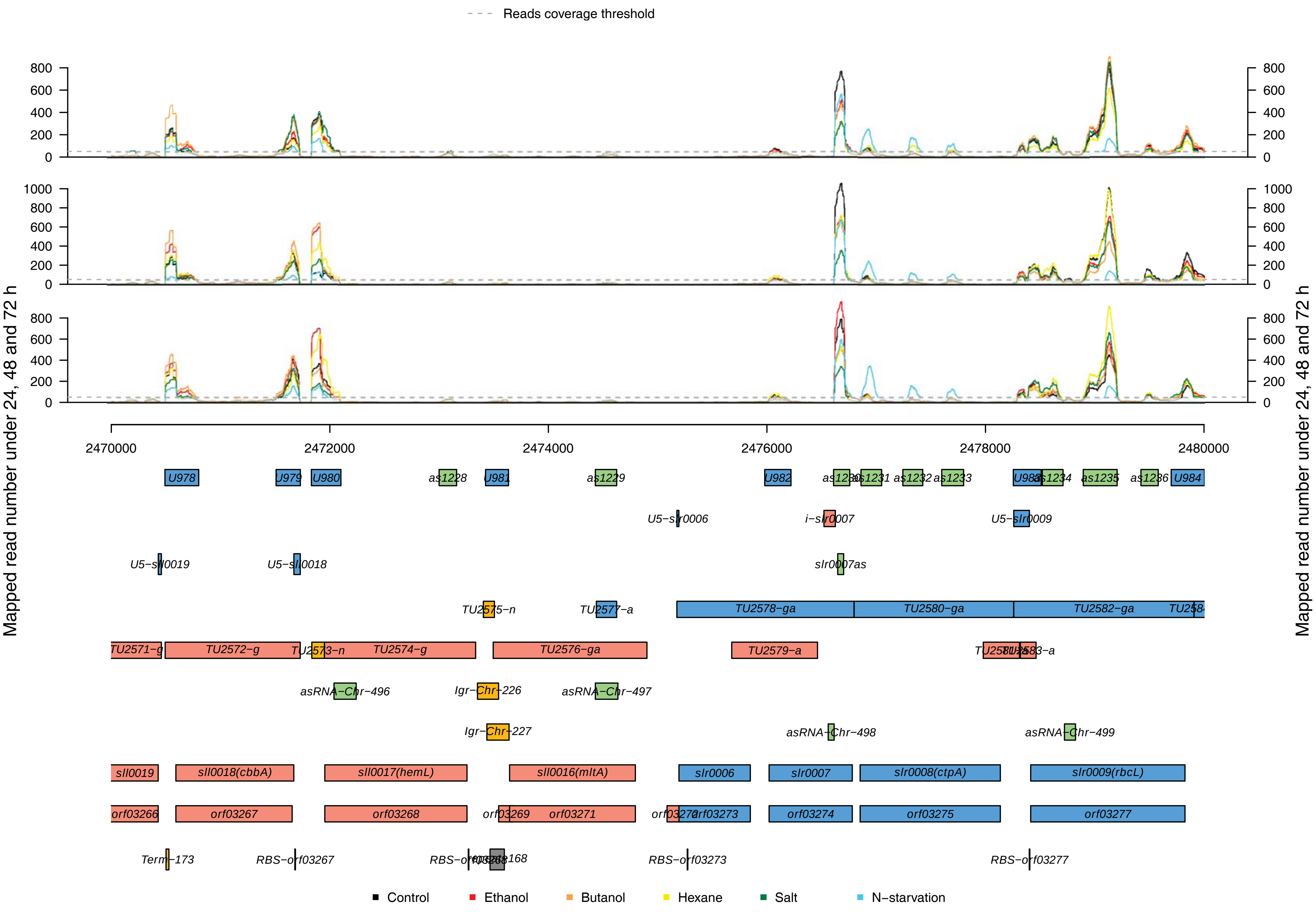
Reads coverage threshold
Mapped read number under 24, 48 and 72 h
2470000
2472000
2474000
2476000
2478000
2480000
U978
U979
U980
as1228
U981
as1229
U982
as1232
as1233
as1234
as1235
U984
U5−slr0006
i−slr0007
U5−slr0009
U5−sll0019
U5−sll0018
slr0007as
TU2575−n
TU2577−a
TU2578−ga
TU2580−ga
TU2582−ga
TU2571−g
TU2572−g
TU2573−n
TU2574−g
TU2576−ga
TU2579−a
asRNA−Chr−496
Igr−Chr−226
asRNA−Chr−497
Igr−Chr−227
asRNA−Chr−498
asRNA−Chr−499
sll0019
sll0018(cbbA)
sll0017(hemL)
sll0016(mltA)
slr0006
slr0007
slr0008(ctpA)
slr0009(rbcL)
orf03266
orf03267
orf03268
orf03271
orf03273
orf03274
orf03275
orf03277
Term−173
RBS−orf03267
RBS−orf03273
RBS−orf03277
Control
Ethanol
Butanol
Hexane
Salt
N−starvation

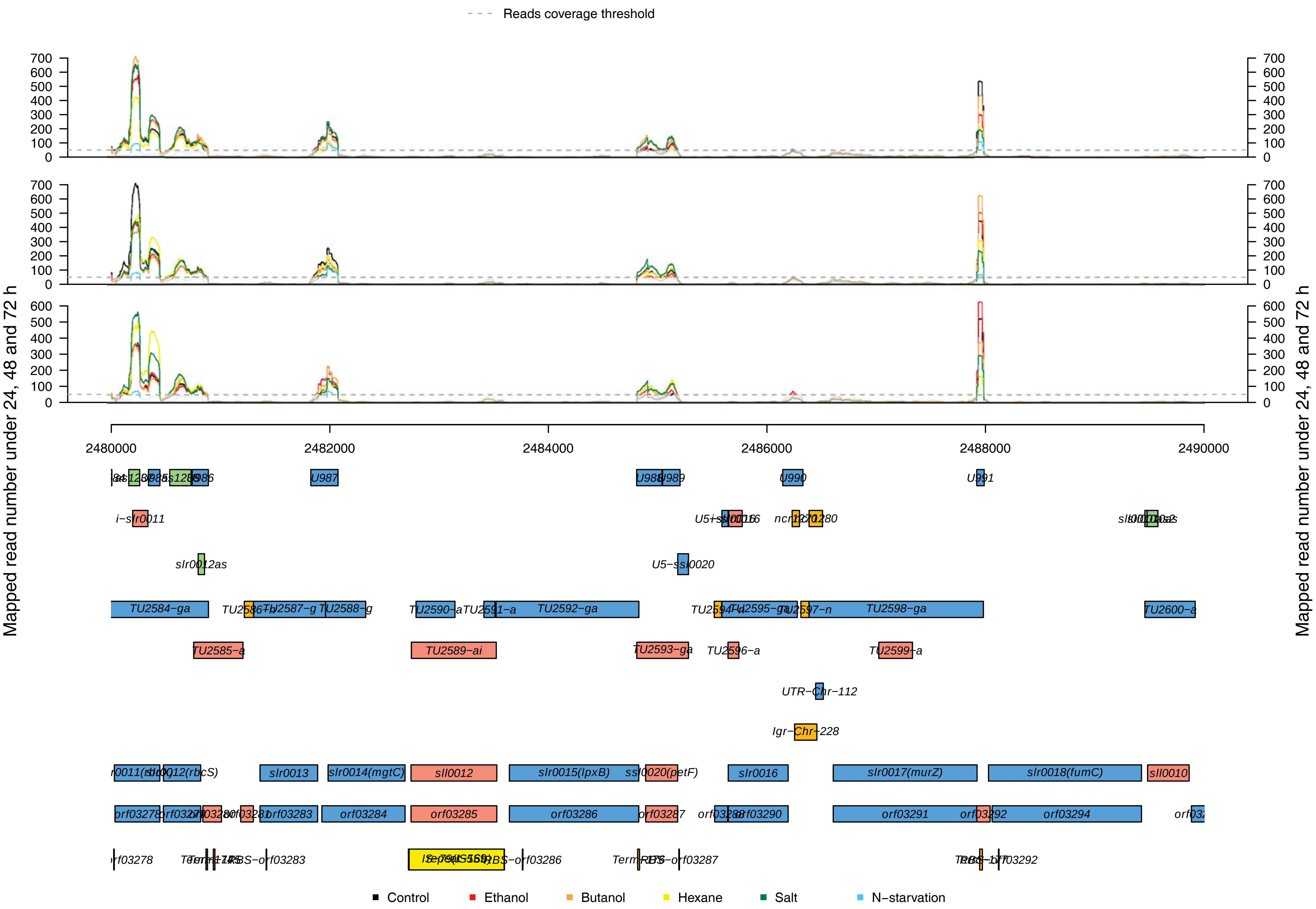

Reads coverage threshold
Mapped read number under 24, 48 and 72 h
2480000
2482000
2484000
2486000
2488000
2490000
U987
U990
i−slr0011
slr0012as
U5−ssl0020
TU2584−ga
TU2592−ga
TU2598−ga
TU2585−a
TU2589−ai
TU2593−ga
TU2596−a
TU2599−a
UTR−Chr−112
Igr−Chr−228
slr0013
slr0014(mgtC)
sll0012
slr0015(lpxB)
slr0016
slr0017(murZ)
slr0018(fumC)
sll0010
orf03283
orf03284
orf03285
orf03286
orf03291
orf03294
Control
Ethanol
Butanol
Hexane
Salt
N−starvation

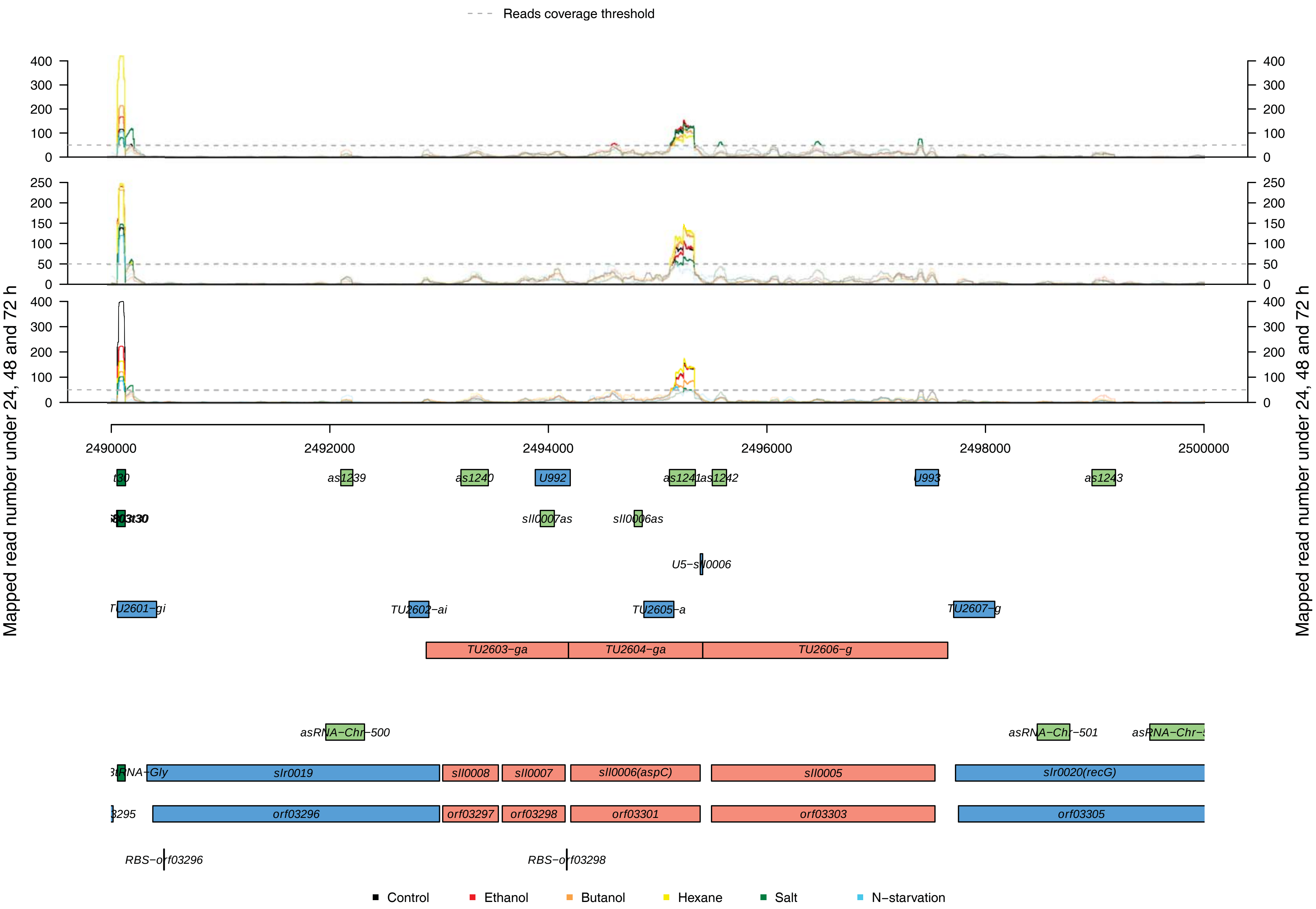

Reads coverage threshold
Mapped read number under 24, 48 and 72 h
2490000
2492000
2494000
2496000
2498000
2500000
as1239
as1240
U992
as1241
as1242
U993
as1243
sll0007as
sll0006as
U5−sll0006
TU2601−gi
TU2602−ai
TU2605−a
TU2607−g
TU2603−ga
TU2604−ga
TU2606−g
asRNA−Chr−500
asRNA−Chr−501
slr0019
sll0008
sll0007
sll0006(aspC)
sll0005
slr0020(recG)
orf03296
orf03297
orf03298
orf03301
orf03303
orf03305
RBS−orf03296
RBS−orf03298
Control
Ethanol
Butanol
Hexane
Salt
N−starvation

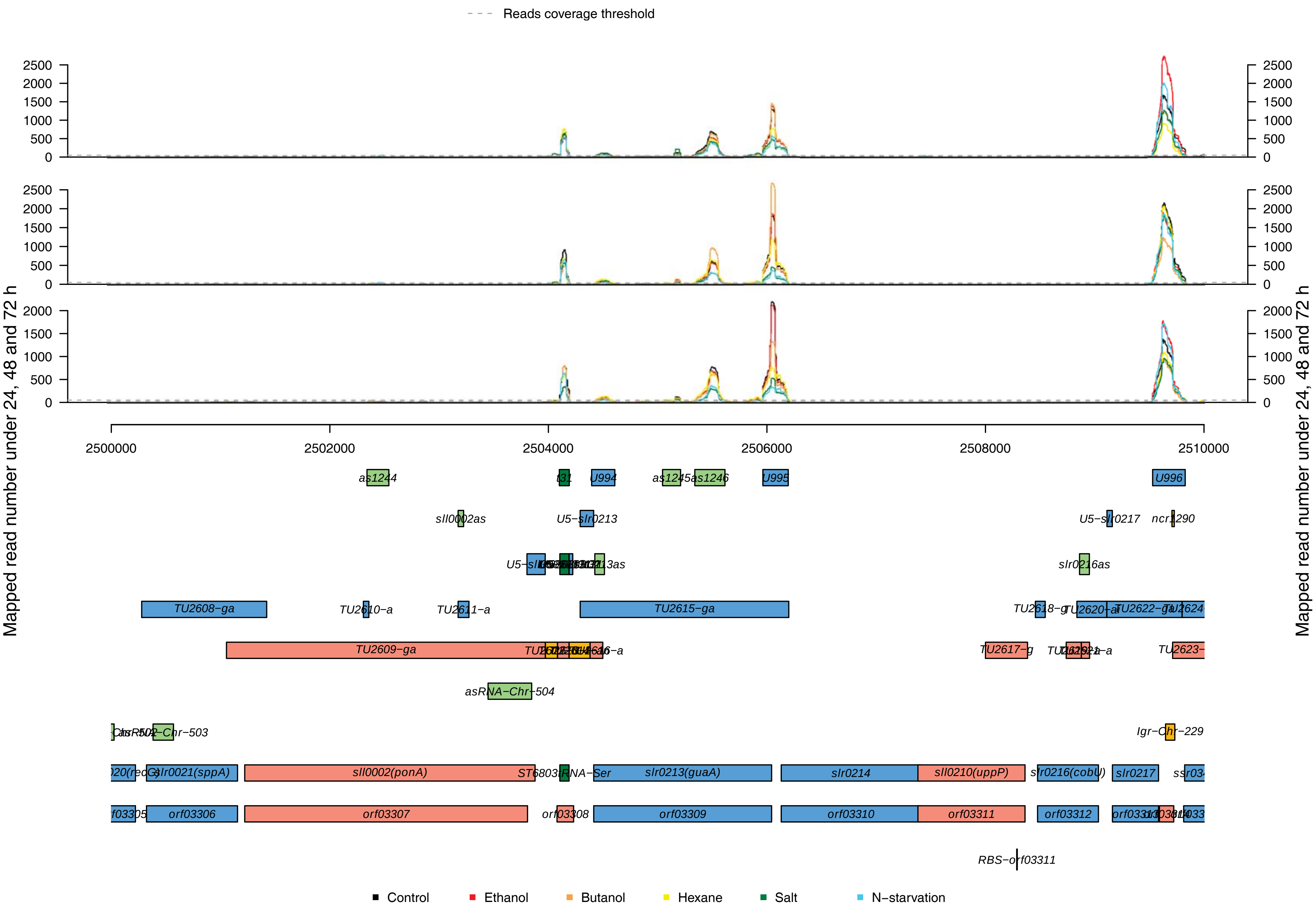

Reads coverage threshold
Mapped read number under 24, 48 and 72 h
2500
2000
1500
1000
500
0
2500000
2502000
2504000
2506000
2508000
2510000
as1244
U994
as1245
as1246
U995
U996
sll0002as
U5−slr0213
U5−slr0217
ncr1290
slr0216as
TU2608−ga
TU2610−a
TU2611−a
TU2615−ga
TU2609−ga
asRNA−Chr−504
Igr−Chr−229
sll0002(ponA)
slr0213(guaA)
slr0214
sll0210(uppP)
slr0217
orf03306
orf03307
orf03309
orf03310
orf03311
orf03312
RBS−orf03311
Control
Ethanol
Butanol
Hexane
Salt
N−starvation

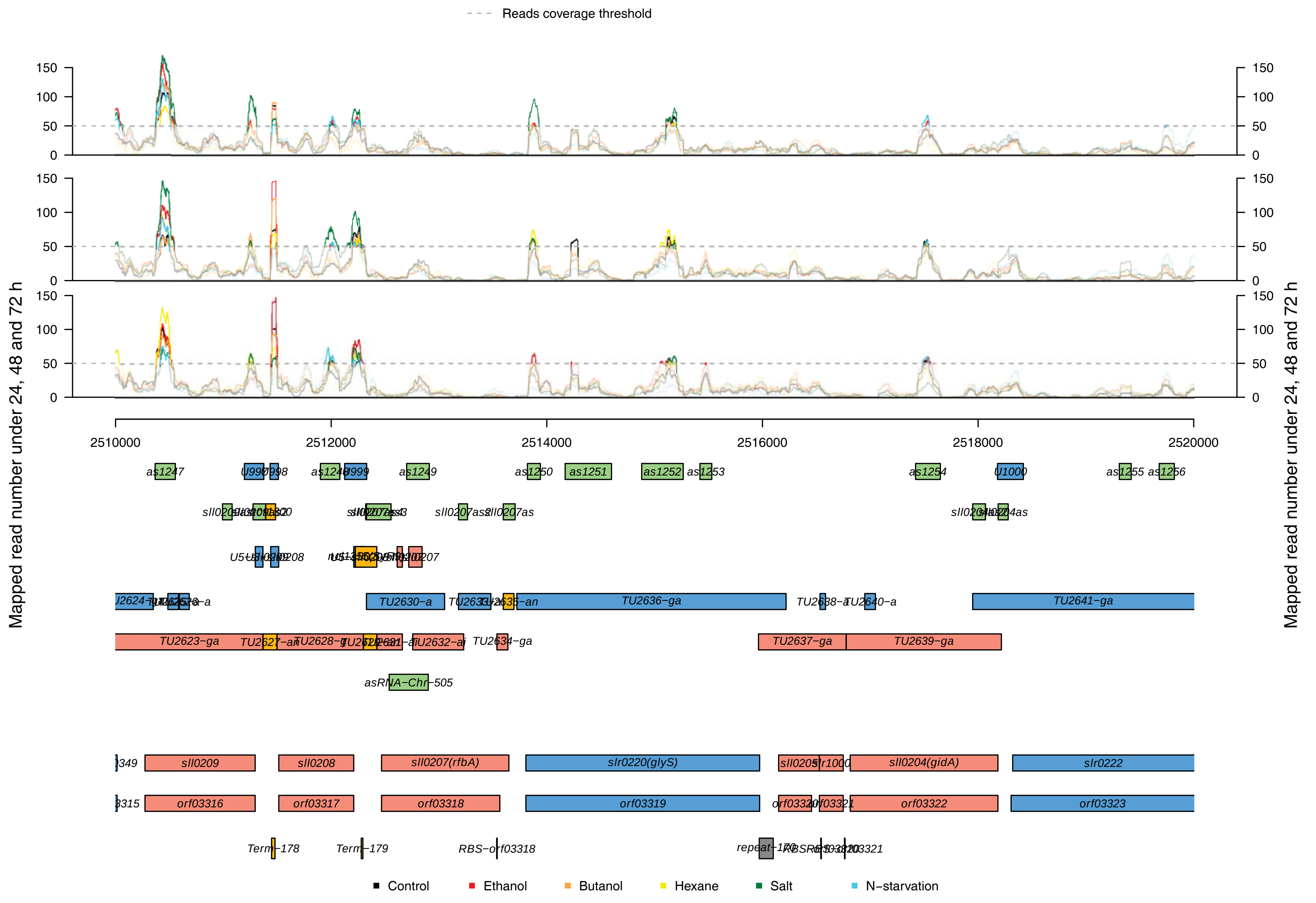

Reads coverage threshold
Mapped read number under 24, 48 and 72 h
2510000
2512000
2514000
2516000
2518000
2520000
as1247
as1249
as1250
as1251
as1252
as1253
as1254
as1255
as1256
TU2630−a
TU2636−ga
TU2641−ga
TU2623−ga
TU2632−ai
TU2634−ga
TU2637−ga
TU2639−ga
asRNA−Chr−505
sll0209
sll0208
sll0207(rfbA)
slr0220(glyS)
sll0204(gidA)
slr0222
orf03316
orf03317
orf03318
orf03319
orf03322
orf03323
Term−178
Term−179
RBS−orf03318
Control
Ethanol
Butanol
Hexane
Salt
N−starvation

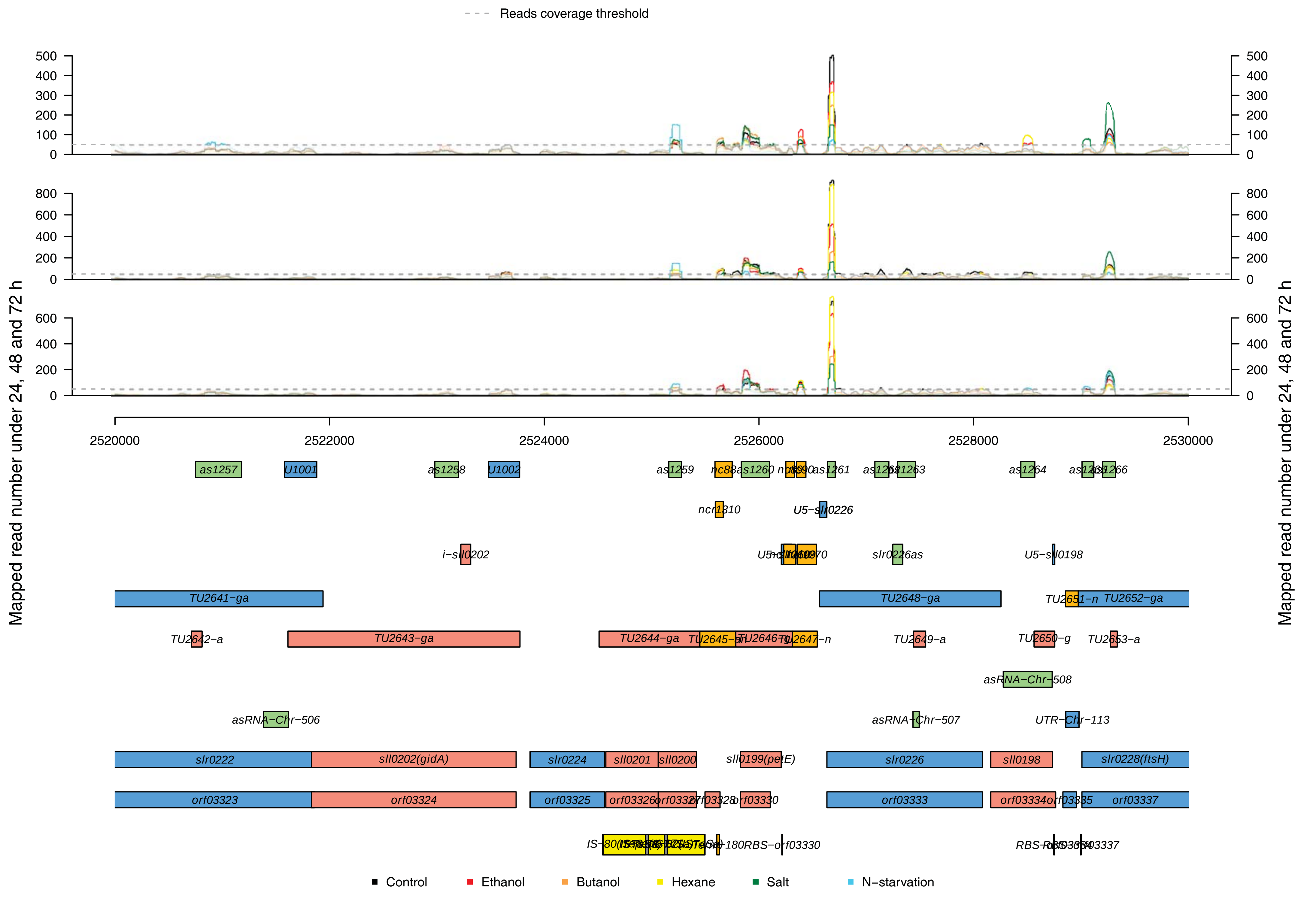

Reads coverage threshold
Mapped read number under 24, 48 and 72 h
2520000
2522000
2524000
2526000
2528000
2530000
as1257
U1001
as1258
U1002
as1259
as1260
as1261
as1264
ncr1310
U5−slr0226
i−sll0202
slr0226as
U5−sll0198
TU2641−ga
TU2648−ga
TU2652−ga
TU2642−a
TU2643−ga
TU2644−ga
TU2649−a
TU2650−g
TU2653−a
asRNA−Chr−508
asRNA−Chr−506
asRNA−Chr−507
UTR−Chr−113
slr0222
sll0202(gidA)
slr0224
sll0201
sll0200
sll0199(petE)
slr0226
sll0198
slr0228(ftsH)
orf03323
orf03324
orf03325
orf03333
orf03337
RBS−orf03330
Control
Ethanol
Butanol
Hexane
Salt
N−starvation

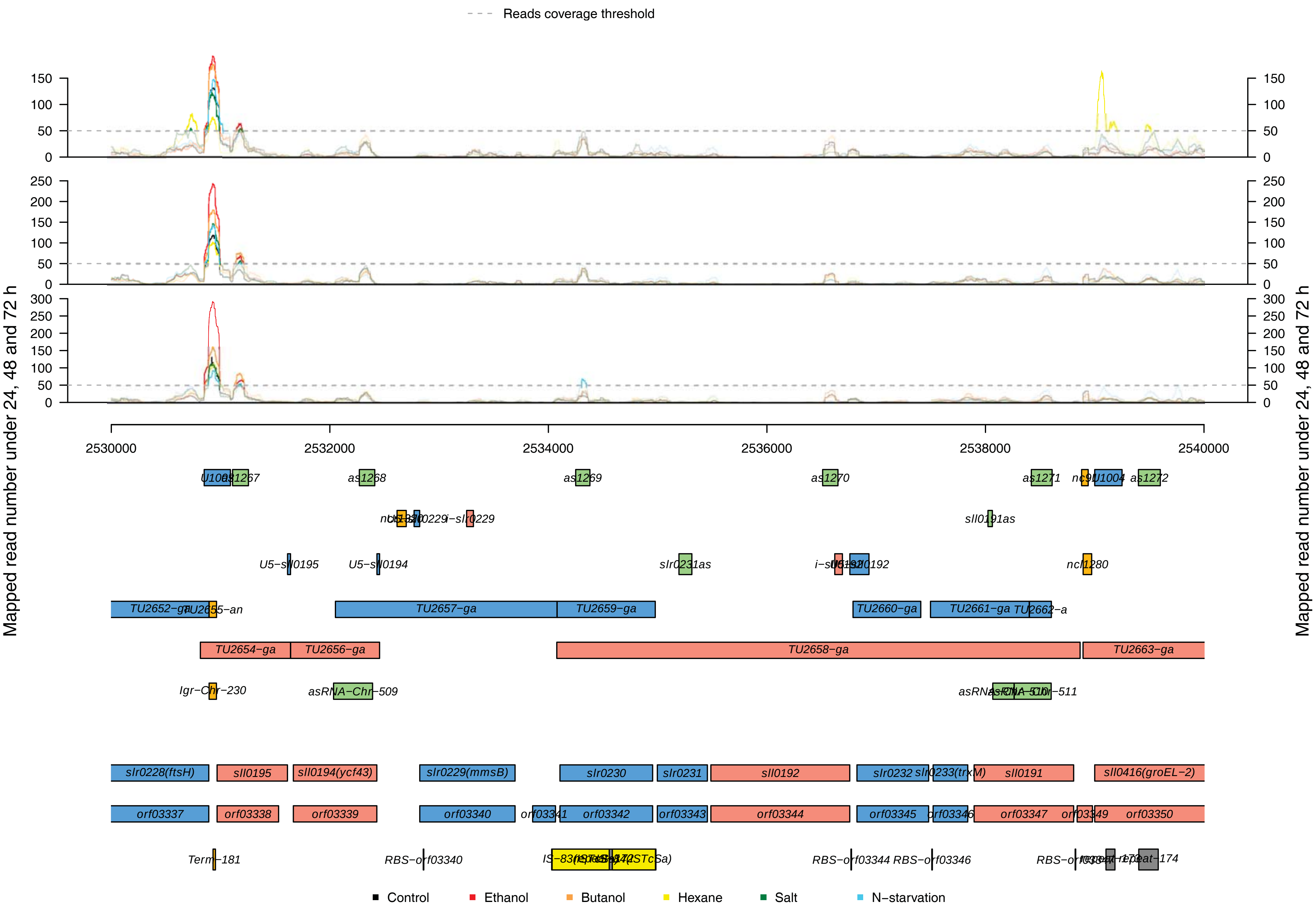

Reads coverage threshold
Mapped read number under 24, 48 and 72 h
2530000
2532000
2534000
2536000
2538000
2540000
as1267
as1268
as1269
as1270
as1271
as1272
U1004
sll0191as
U5−sll0195
U5−sll0194
slr0231as
ncl1280
TU2652−ga
TU2657−ga
TU2659−ga
TU2660−ga
TU2661−ga
TU2654−ga
TU2656−ga
TU2658−ga
TU2663−ga
Igr−Chr−230
asRNA−Chr−509
slr0228(ftsH)
sll0195
sll0194(ycf43)
slr0229(mmsB)
slr0230
slr0231
sll0192
sll0191
sll0416(groEL−2)
orf03337
orf03338
orf03339
orf03340
orf03342
orf03343
orf03344
orf03345
orf03347
orf03350
Term−181
RBS−orf03340
RBS−orf03344
RBS−orf03346
repeat−174
Control
Ethanol
Butanol
Hexane
Salt
N−starvation

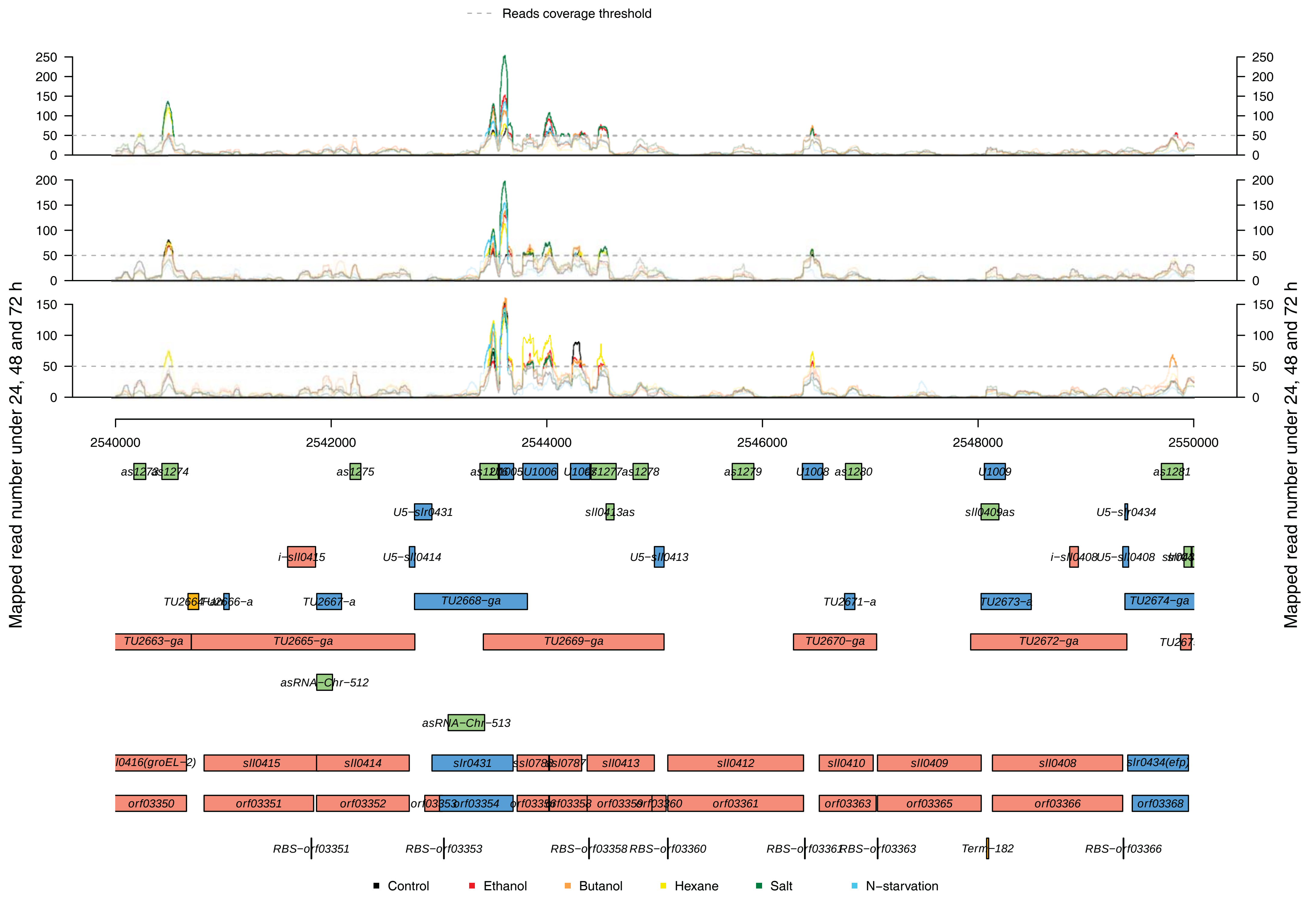

Reads coverage threshold
Mapped read number under 24, 48 and 72 h
2540000
2542000
2544000
2546000
2548000
2550000
TU2665−ga
TU2668−ga
TU2669−ga
TU2670−ga
TU2672−ga
TU2674−ga
asRNA−Chr−512
asRNA−Chr−513
sll0415
sll0414
slr0431
sll0413
sll0412
sll0410
sll0409
sll0408
slr0434(efp)
orf03350
orf03351
orf03352
orf03354
orf03361
orf03363
orf03365
orf03366
orf03368
RBS−orf03351
RBS−orf03353
RBS−orf03358
RBS−orf03360
RBS−orf03363
RBS−orf03366
Control
Ethanol
Butanol
Hexane
Salt
N−starvation

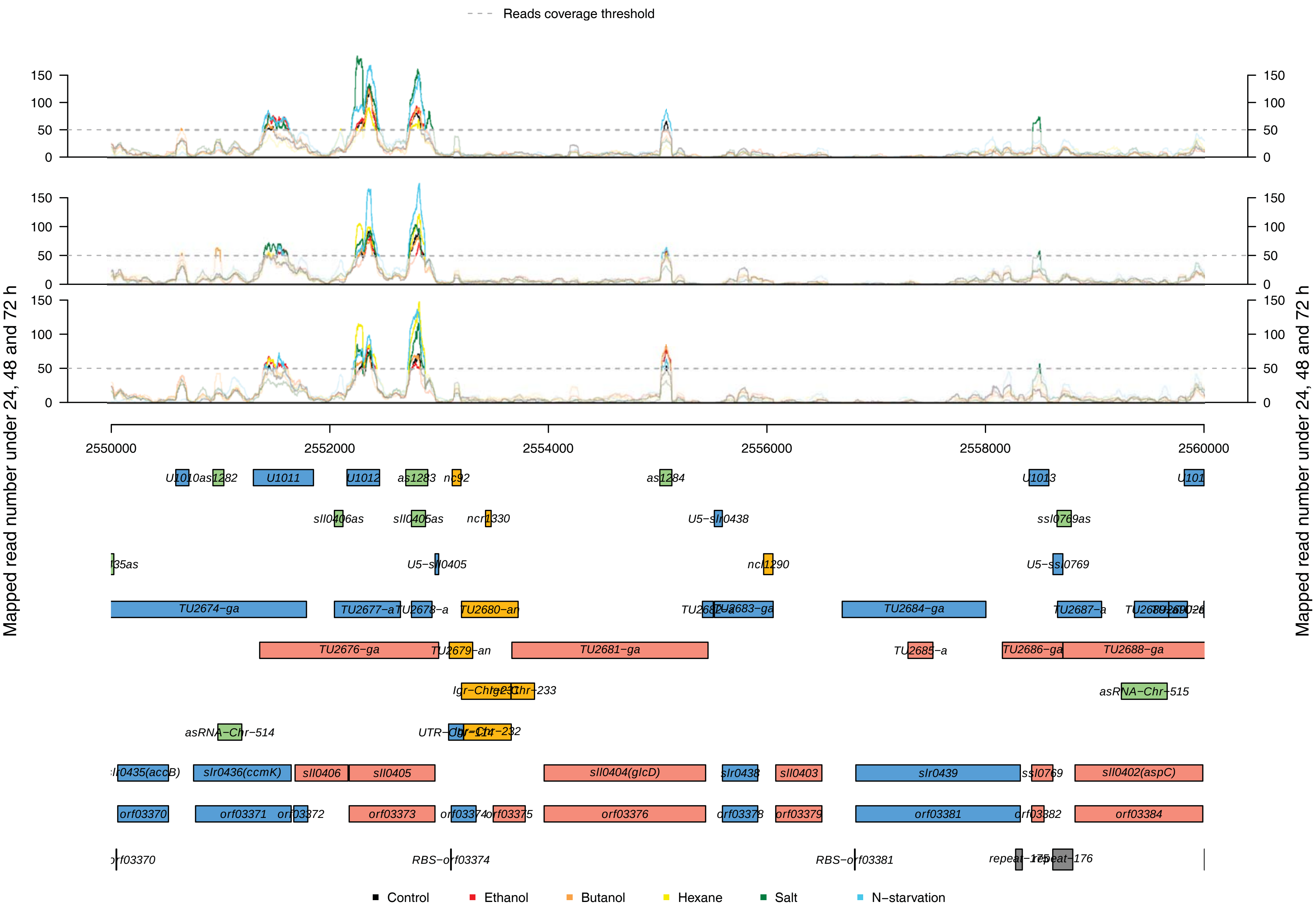

Reads coverage threshold
Mapped read number under 24, 48 and 72 h
2550000
2552000
2554000
2556000
2558000
2560000
TU2674−ga
TU2676−ga
TU2681−ga
TU2684−ga
TU2688−ga
slr0436(ccmK)
sll0404(glcD)
slr0439
sll0402(aspC)
orf03370
orf03371
orf03373
orf03376
orf03381
orf03384
asRNA−Chr−514
asRNA−Chr−515
RBS−orf03374
RBS−orf03381
Control
Ethanol
Butanol
Hexane
Salt
N−starvation

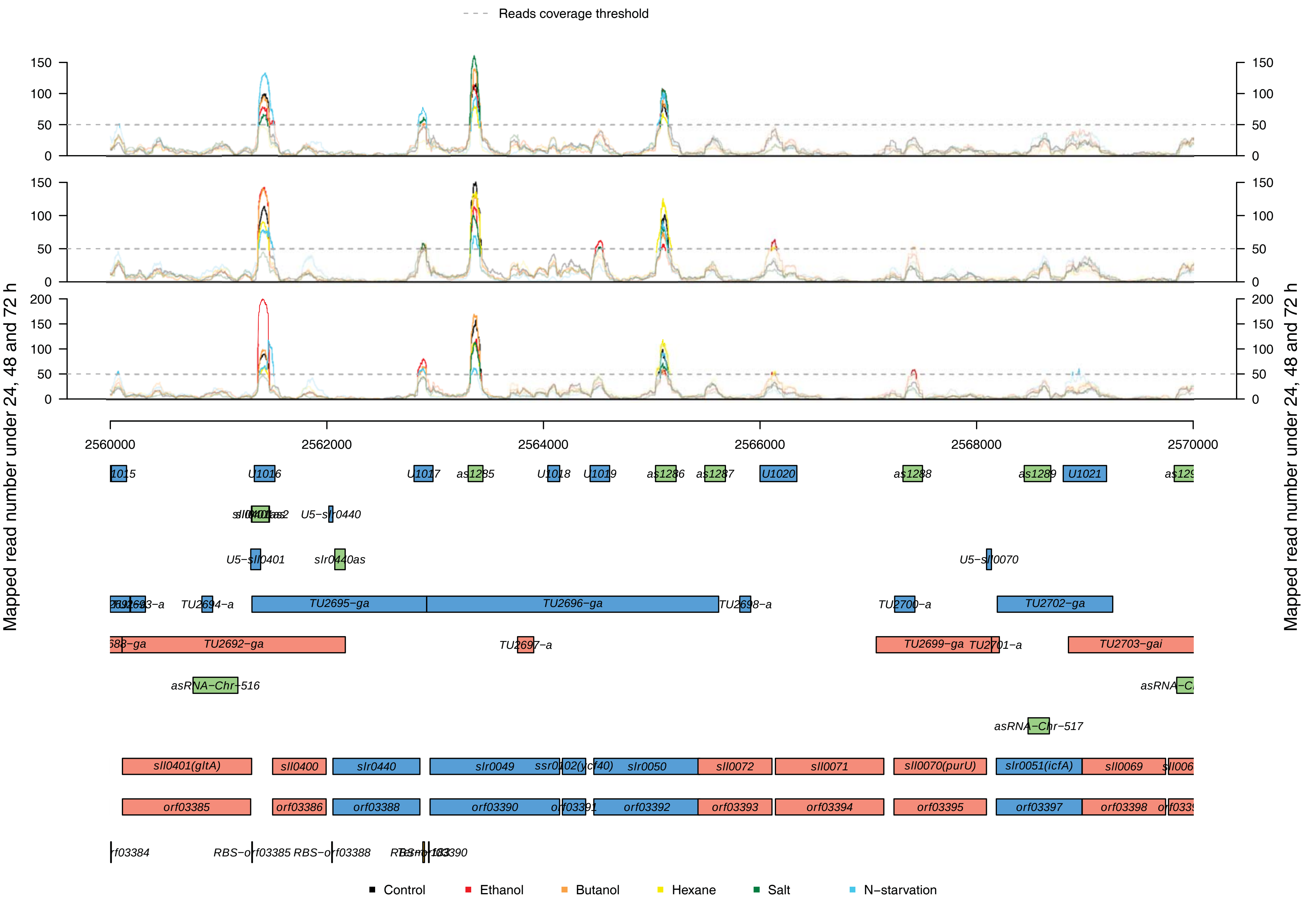

Reads coverage threshold
Mapped read number under 24, 48 and 72 h
2560000
2562000
2564000
2566000
2568000
2570000
TU2695−ga
TU2696−ga
TU2702−ga
TU2692−ga
TU2699−ga
TU2703−gai
asRNA−Chr−516
asRNA−Chr−517
sll0401(gltA)
sll0400
slr0440
slr0049
slr0050
sll0072
sll0071
sll0070(purU)
slr0051(icfA)
sll0069
orf03385
orf03386
orf03388
orf03390
orf03392
orf03393
orf03394
orf03395
orf03397
orf03398
Control
Ethanol
Butanol
Hexane
Salt
N−starvation

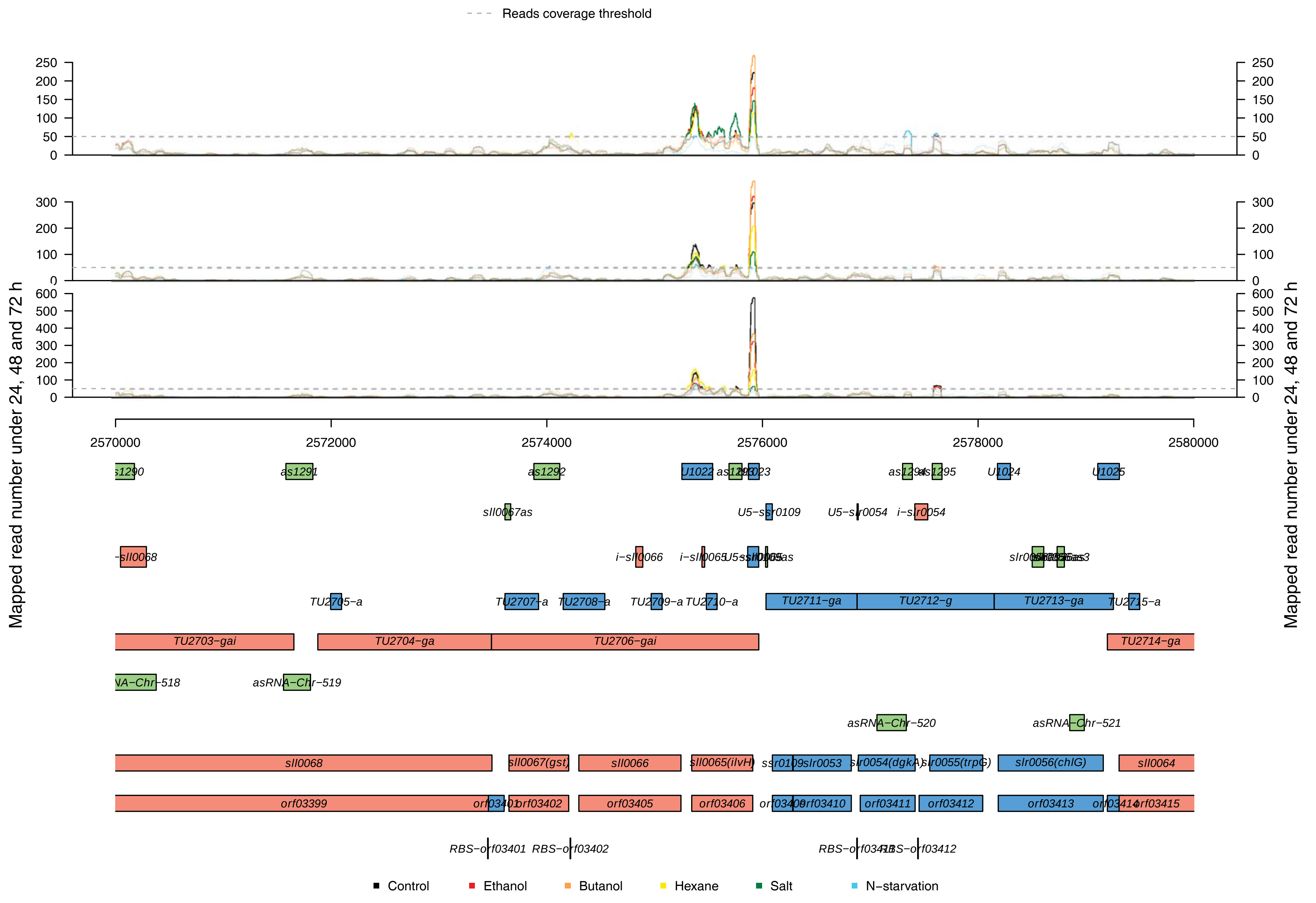

Reads coverage threshold
Mapped read number under 24, 48 and 72 h
2570000
2572000
2574000
2576000
2578000
2580000
TU2703–gai
TU2704–ga
TU2706–gai
TU2711–ga
TU2712–g
TU2713–ga
TU2714–ga
sll0068
sll0067(gst)
sll0066
sll0065(ilvH)
slr0053
slr0054(dgkA)
slr0055(trpG)
slr0056(chlG)
sll0064
orf03399
orf03402
orf03405
orf03406
orf03410
orf03411
orf03412
orf03413
orf03415
asRNA–Chr–519
asRNA–Chr–520
asRNA–Chr–521
RBS–orf03401
RBS–orf03402
RBS–orf03412
Control
Ethanol
Butanol
Hexane
Salt
N–starvation

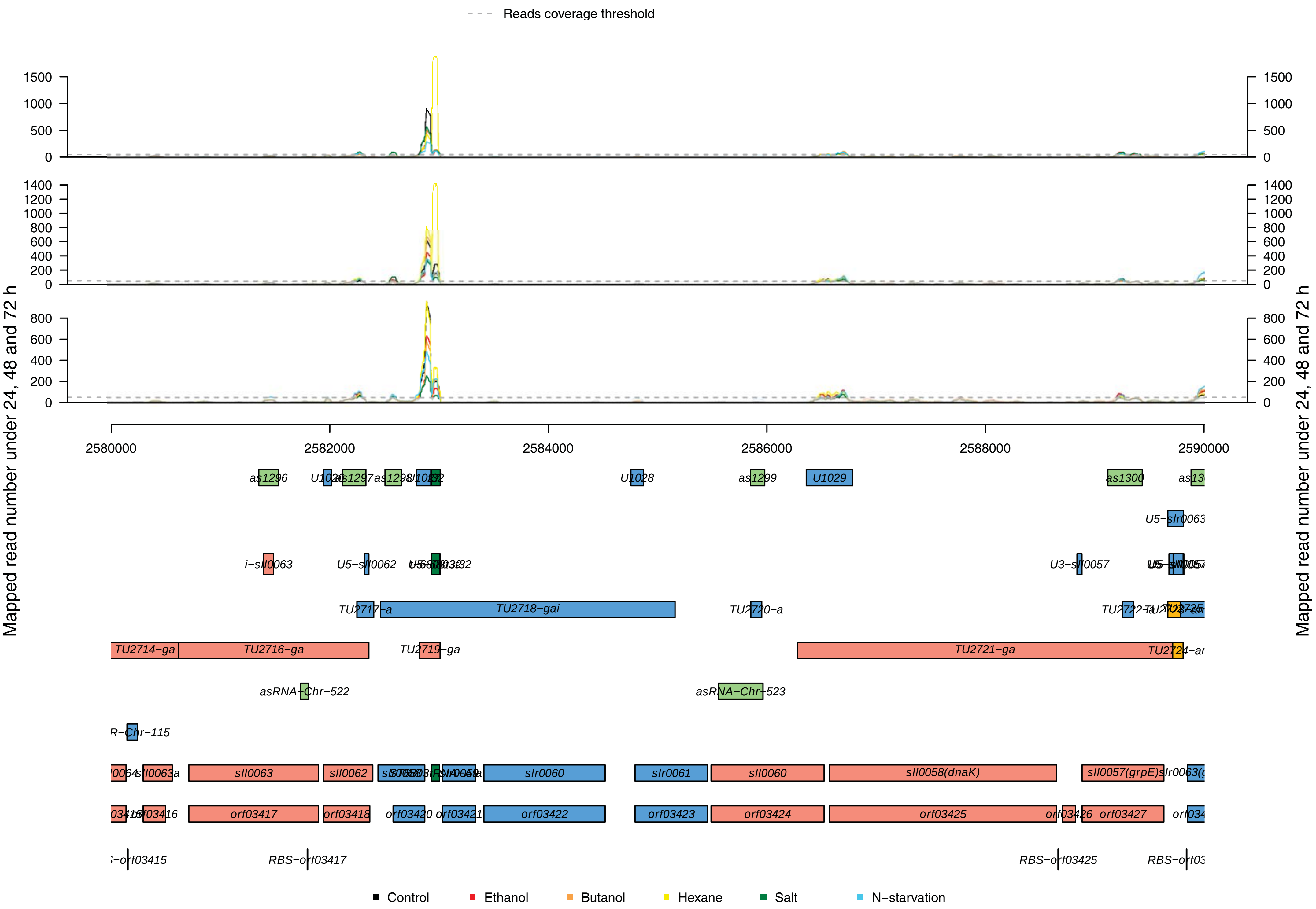

Reads coverage threshold
Mapped read number under 24, 48 and 72 h
2580000
2582000
2584000
2586000
2588000
2590000
as1296
as1297
as1298
U1028
as1299
U1029
as1300
U5−slr0063
i−sll0063
U5−sll0062
U3−sll0057
TU2717−a
TU2718−gai
TU2720−a
TU2714−ga
TU2716−ga
TU2719−ga
TU2721−ga
asRNA−Chr−522
asRNA−Chr−523
sll0063
sll0062
slr0060
slr0061
sll0060
sll0058(dnaK)
sll0057(grpE)
orf03417
orf03418
orf03420
orf03421
orf03422
orf03423
orf03424
orf03425
orf03427
RBS−orf03417
RBS−orf03425
Control
Ethanol
Butanol
Hexane
Salt
N−starvation

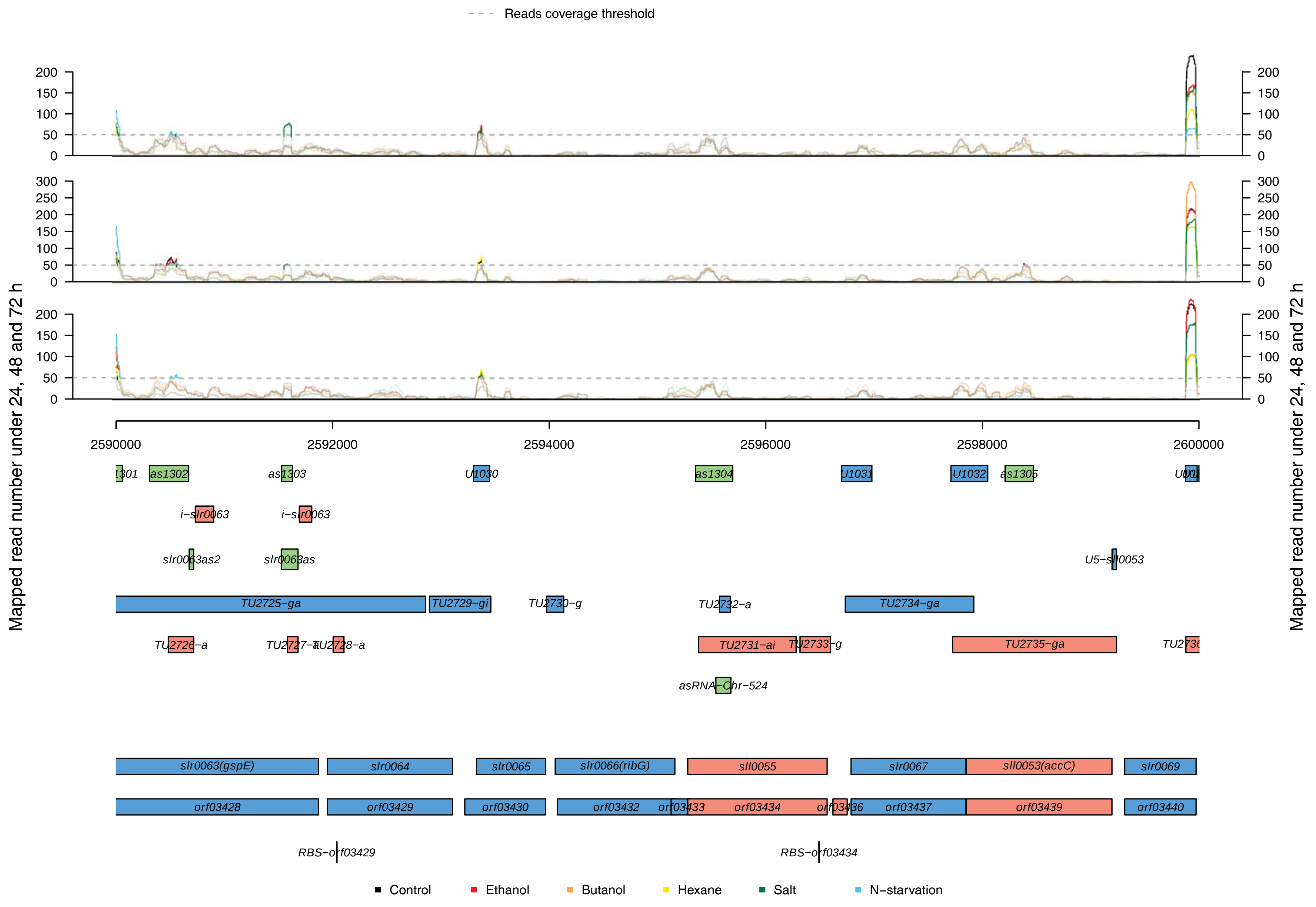

Reads coverage threshold
Mapped read number under 24, 48 and 72 h
2590000
2592000
2594000
2596000
2598000
2600000
as1302
as1303
U1030
as1304
U1031
U1032
as1305
i−slr0063
i−slr0063
slr0063as2
slr0063as
U5−sll0053
TU2725−ga
TU2729−gi
TU2730−g
TU2732−a
TU2734−ga
TU2726−a
TU2727−a
TU2728−a
TU2731−ai
TU2733−g
TU2735−ga
asRNA−Chr−524
slr0063(gspE)
slr0064
slr0065
slr0066(ribG)
sll0055
slr0067
sll0053(accC)
slr0069
orf03428
orf03429
orf03430
orf03432
orf03433
orf03434
orf03436
orf03437
orf03439
orf03440
RBS−orf03429
RBS−orf03434
Control
Ethanol
Butanol
Hexane
Salt
N−starvation

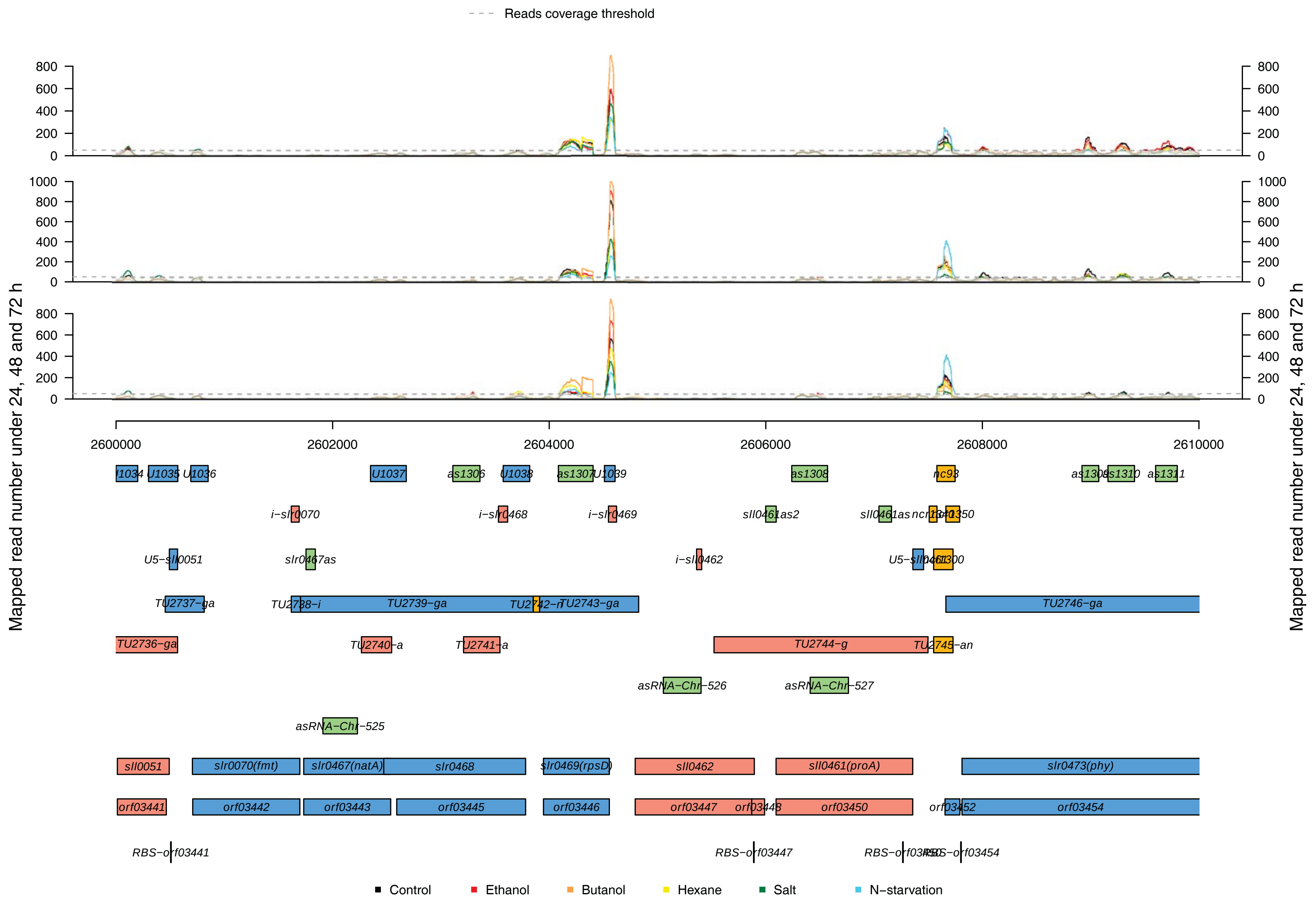

Reads coverage threshold
Mapped read number under 24, 48 and 72 h
2600000
2602000
2604000
2606000
2608000
2610000
Control
Ethanol
Butanol
Hexane
Salt
N−starvation

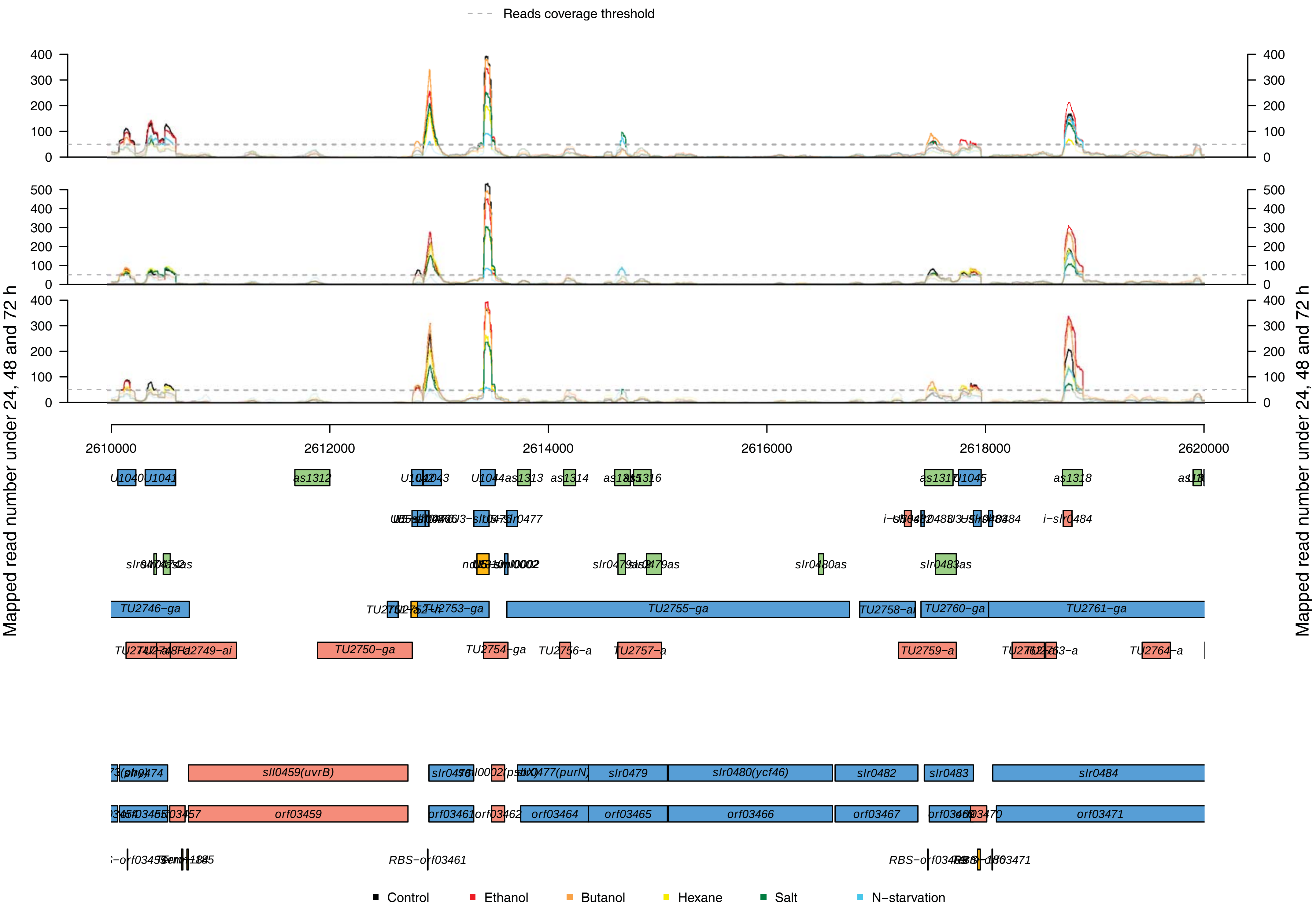

Reads coverage threshold
Mapped read number under 24, 48 and 72 h
2610000
2612000
2614000
2616000
2618000
2620000
TU2746−ga
TU2750−ga
TU2755−ga
TU2759−a
TU2760−ga
TU2761−ga
TU2764−a
sll0459(uvrB)
slr0479
slr0480(ycf46)
slr0482
slr0483
slr0484
orf03459
orf03464
orf03465
orf03466
orf03467
orf03471
Control
Ethanol
Butanol
Hexane
Salt
N−starvation

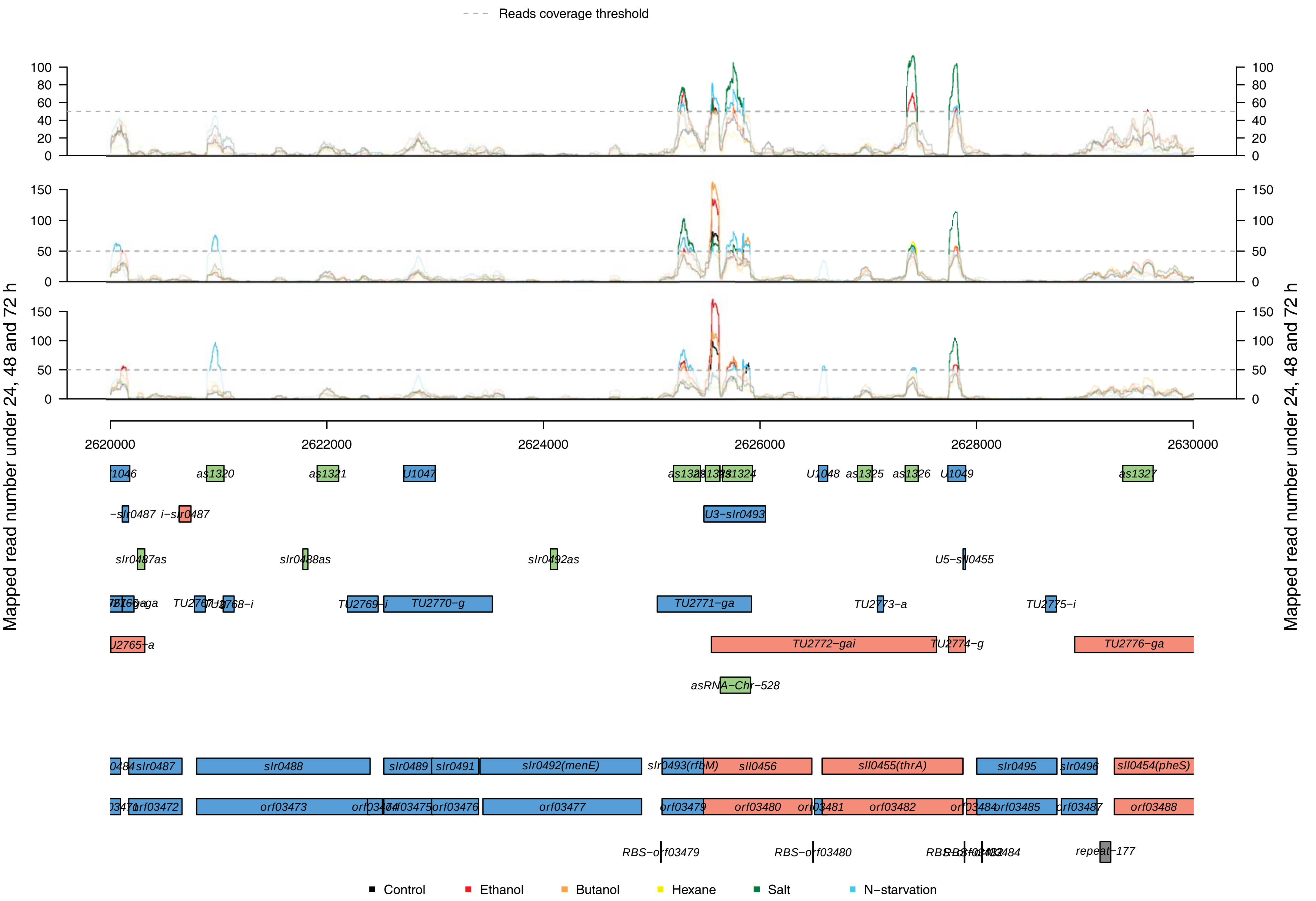

Reads coverage threshold
Mapped read number under 24, 48 and 72 h
2620000
2622000
2624000
2626000
2628000
2630000
as1320
as1321
U1047
U1048
as1325
as1326
U1049
as1327
U3−slr0493
slr0487as
slr0488as
slr0492as
U5−sll0455
TU2769−i
TU2770−g
TU2771−ga
TU2773−a
TU2775−i
TU2772−gai
TU2774−g
TU2776−ga
asRNA−Chr−528
slr0487
slr0488
slr0489
slr0491
slr0492(menE)
slr0493(rfbM)
sll0456
sll0455(thrA)
slr0495
slr0496
sll0454(pheS)
orf03472
orf03473
orf03476
orf03477
orf03479
orf03480
orf03481
orf03482
orf03485
orf03487
orf03488
RBS−orf03479
RBS−orf03480
repeat−177
Control
Ethanol
Butanol
Hexane
Salt
N−starvation

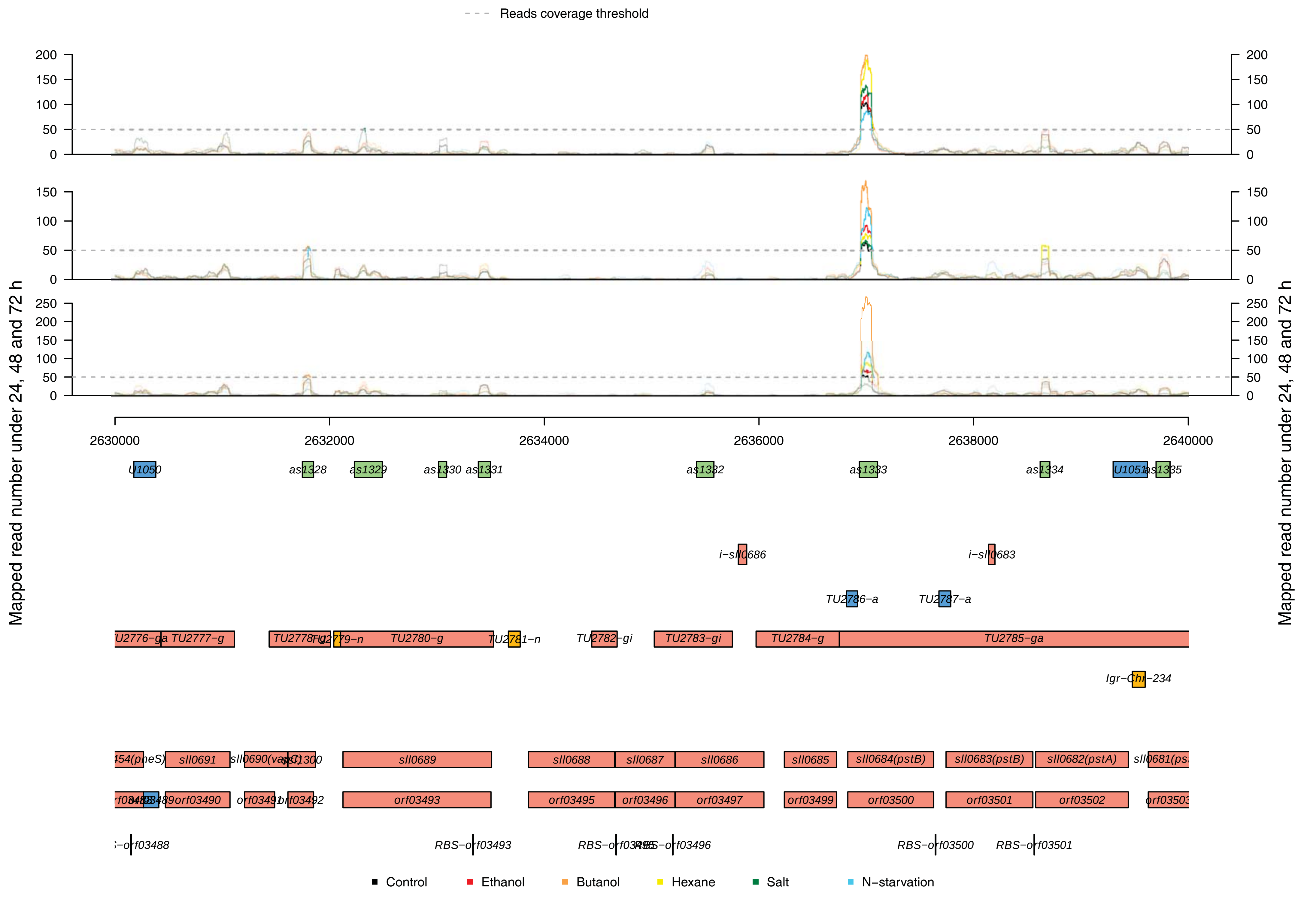

Reads coverage threshold
Mapped read number under 24, 48 and 72 h
2630000
2632000
2634000
2636000
2638000
2640000
as1328
as1329
as1330
as1331
as1332
as1333
as1334
as1335
i−sll0686
i−sll0683
TU2786−a
TU2787−a
TU2777−g
TU2780−g
TU2783−gi
TU2784−g
TU2785−ga
sll0691
sll0689
sll0688
sll0687
sll0686
sll0685
sll0684(pstB)
sll0683(pstB)
sll0682(pstA)
orf03490
orf03493
orf03495
orf03496
orf03497
orf03499
orf03500
orf03501
orf03502
RBS−orf03493
RBS−orf03500
RBS−orf03501
Control
Ethanol
Butanol
Hexane
Salt
N−starvation

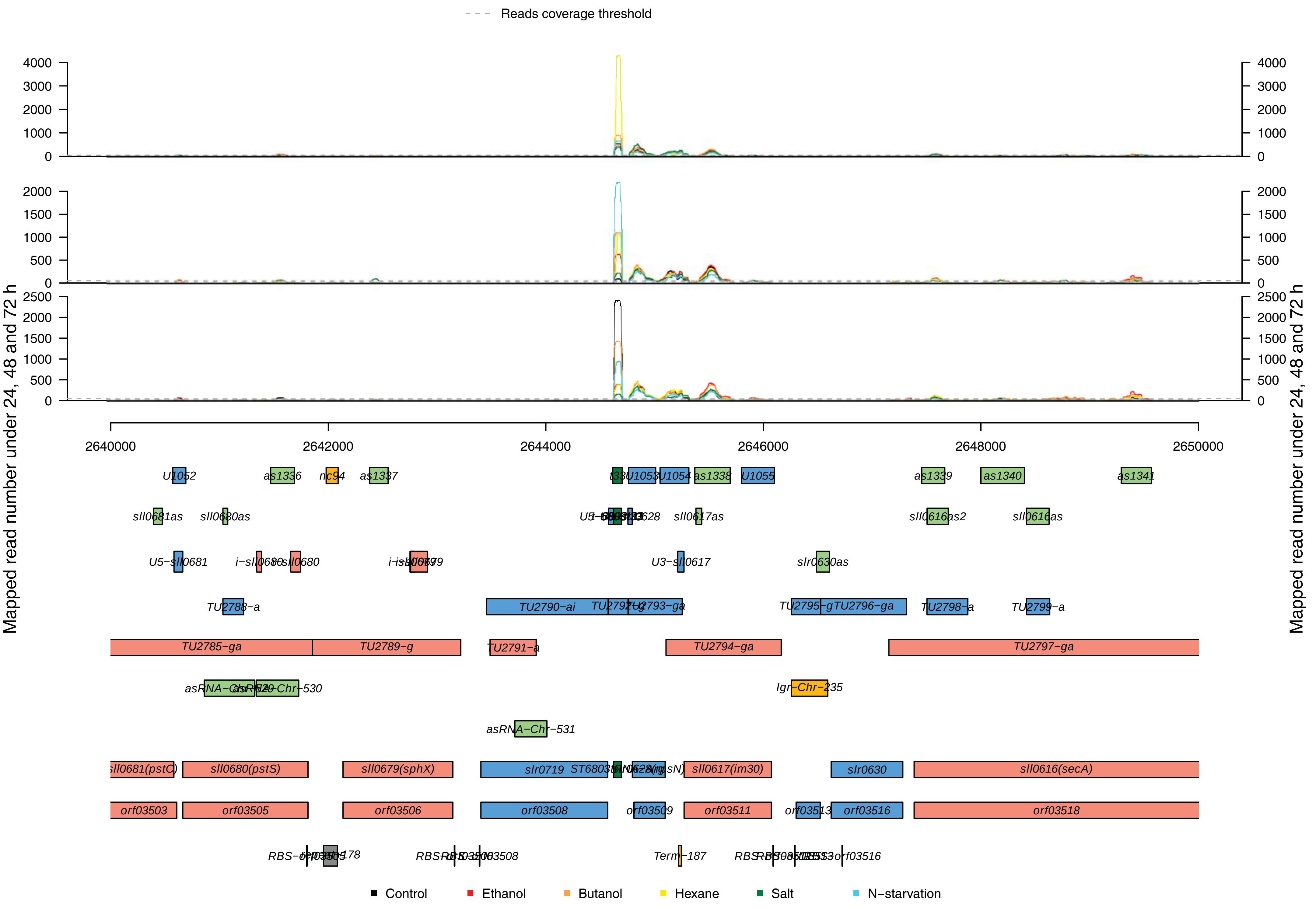
Reads coverage threshold
Mapped read number under 24, 48 and 72 h
2640000
2642000
2644000
2646000
2648000
2650000
TU2785-ga
TU2789-g
TU2790-ai
TU2791-a
TU2794-ga
TU2797-ga
sll0681(pstC)
sll0680(pstS)
sll0679(sphX)
slr0719
sll0617(im30)
slr0630
sll0616(secA)
orf03503
orf03505
orf03506
orf03508
orf03509
orf03511
orf03516
orf03518
Control
Ethanol
Butanol
Hexane
Salt
N-starvation

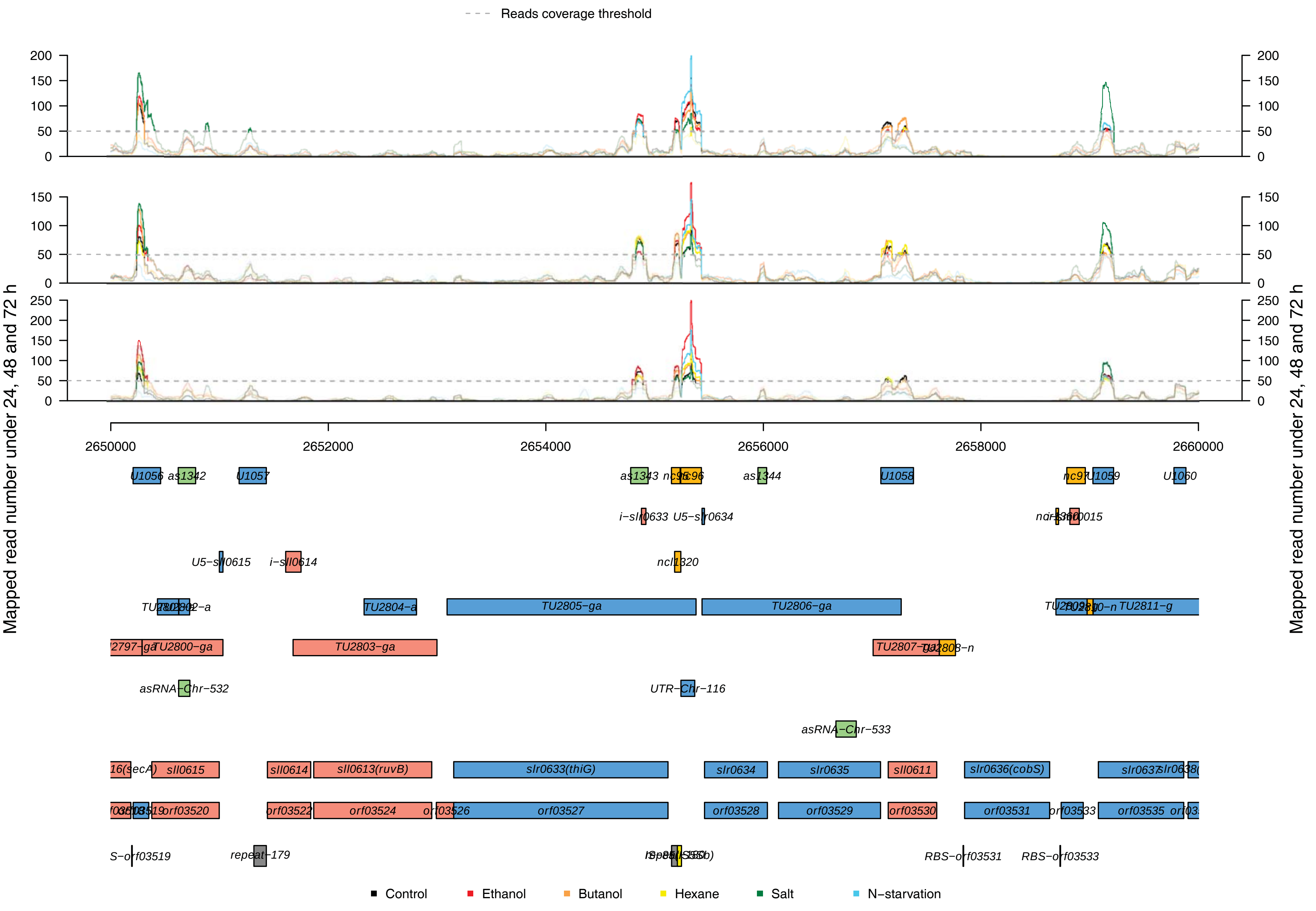
Reads coverage threshold
Mapped read number under 24, 48 and 72 h
2650000
2652000
2654000
2656000
2658000
2660000
Control
Ethanol
Butanol
Hexane
Salt
N−starvation
TU2805−ga
TU2806−ga
TU2803−ga
TU2800−ga
TU2804−a
slr0633(thiG)
sll0613(ruvB)
slr0636(cobS)
orf03527
orf03524
asRNA−Chr−532
asRNA−Chr−533
UTR−Chr−116
repeat−179
RBS−orf03531
RBS−orf03533

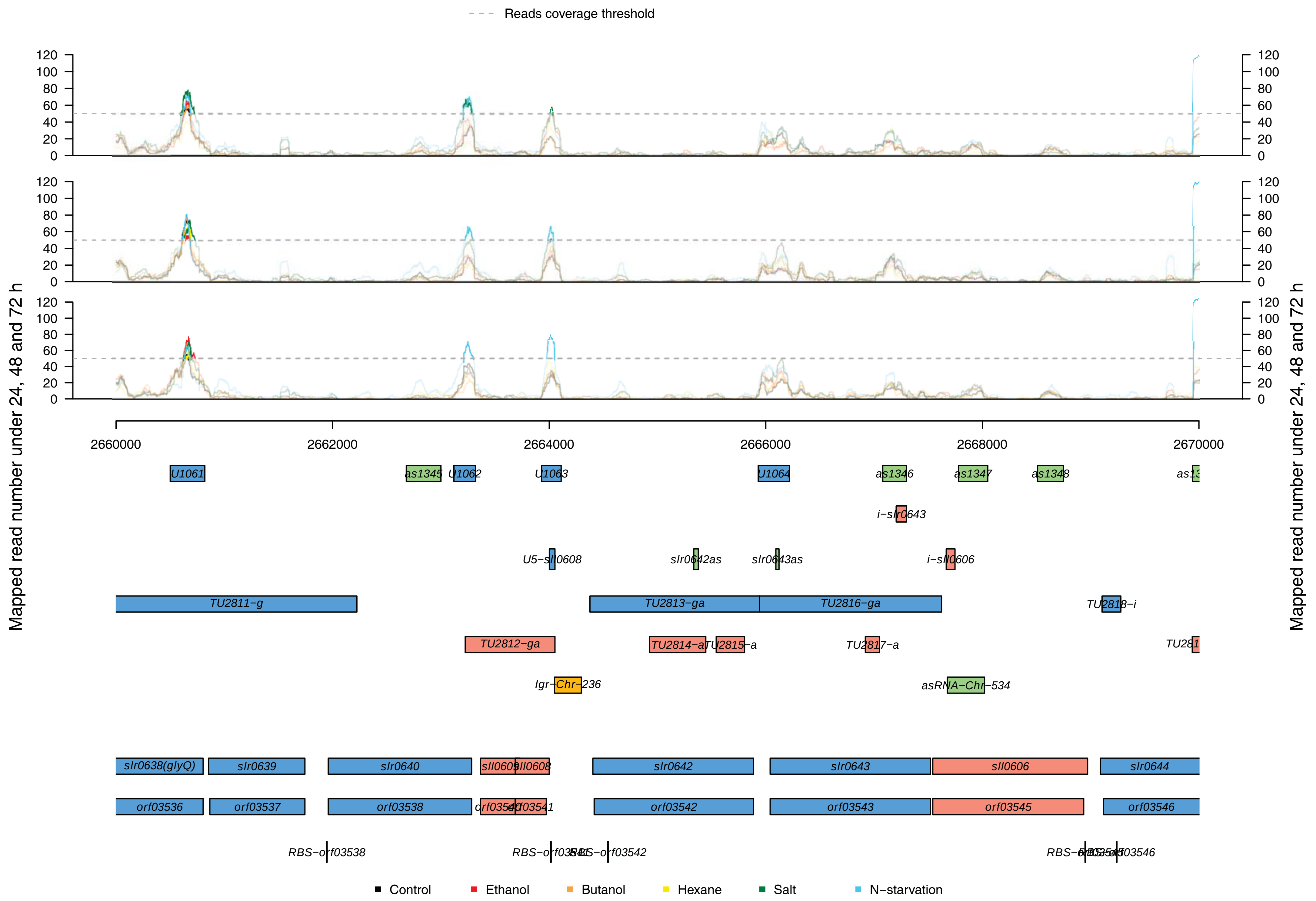

Reads coverage threshold
Mapped read number under 24, 48 and 72 h
2660000
2662000
2664000
2666000
2668000
2670000
U1061
as1345
U1062
U1063
U1064
as1346
as1347
as1348
i−slr0643
U5−sll0608
slr0642as
slr0643as
i−sll0606
TU2811−g
TU2813−ga
TU2816−ga
TU2818−i
TU2812−ga
TU2814−a
TU2815−a
TU2817−a
Igr−Chr−236
asRNA−Chr−534
slr0638(glyQ)
slr0639
slr0640
sll0609
sll0608
slr0642
slr0643
sll0606
slr0644
orf03536
orf03537
orf03538
orf03540
orf03541
orf03542
orf03543
orf03545
orf03546
RBS−orf03538
RBS−orf03542
Control
Ethanol
Butanol
Hexane
Salt
N−starvation

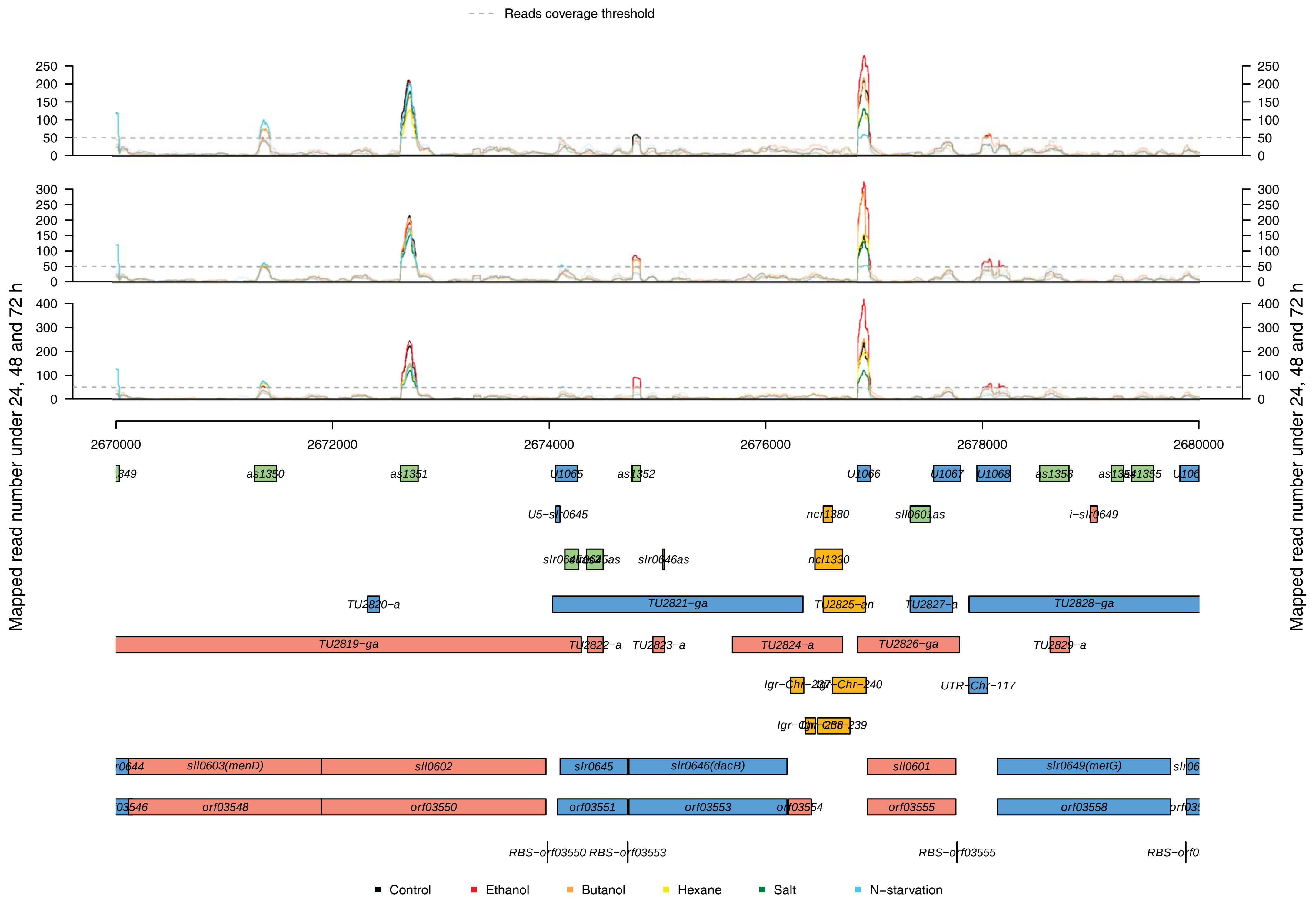

Reads coverage threshold
Mapped read number under 24, 48 and 72 h
2670000
2672000
2674000
2676000
2678000
2680000
as1350
as1351
U1065
as1352
U1066
U1067
U1068
as1353
U5−slr0645
ncl1380
sll0601as
i−slr0649
slr0646as
ncl1330
TU2820−a
TU2821−ga
TU2825−an
TU2827−a
TU2828−ga
TU2819−ga
TU2822−a
TU2823−a
TU2824−a
TU2826−ga
TU2829−a
Igr−Chr−240
UTR−Chr−117
sll0603(menD)
sll0602
slr0645
slr0646(dacB)
sll0601
slr0649(metG)
orf03548
orf03550
orf03551
orf03553
orf03555
orf03558
RBS−orf03550
RBS−orf03553
RBS−orf03555
Control
Ethanol
Butanol
Hexane
Salt
N−starvation

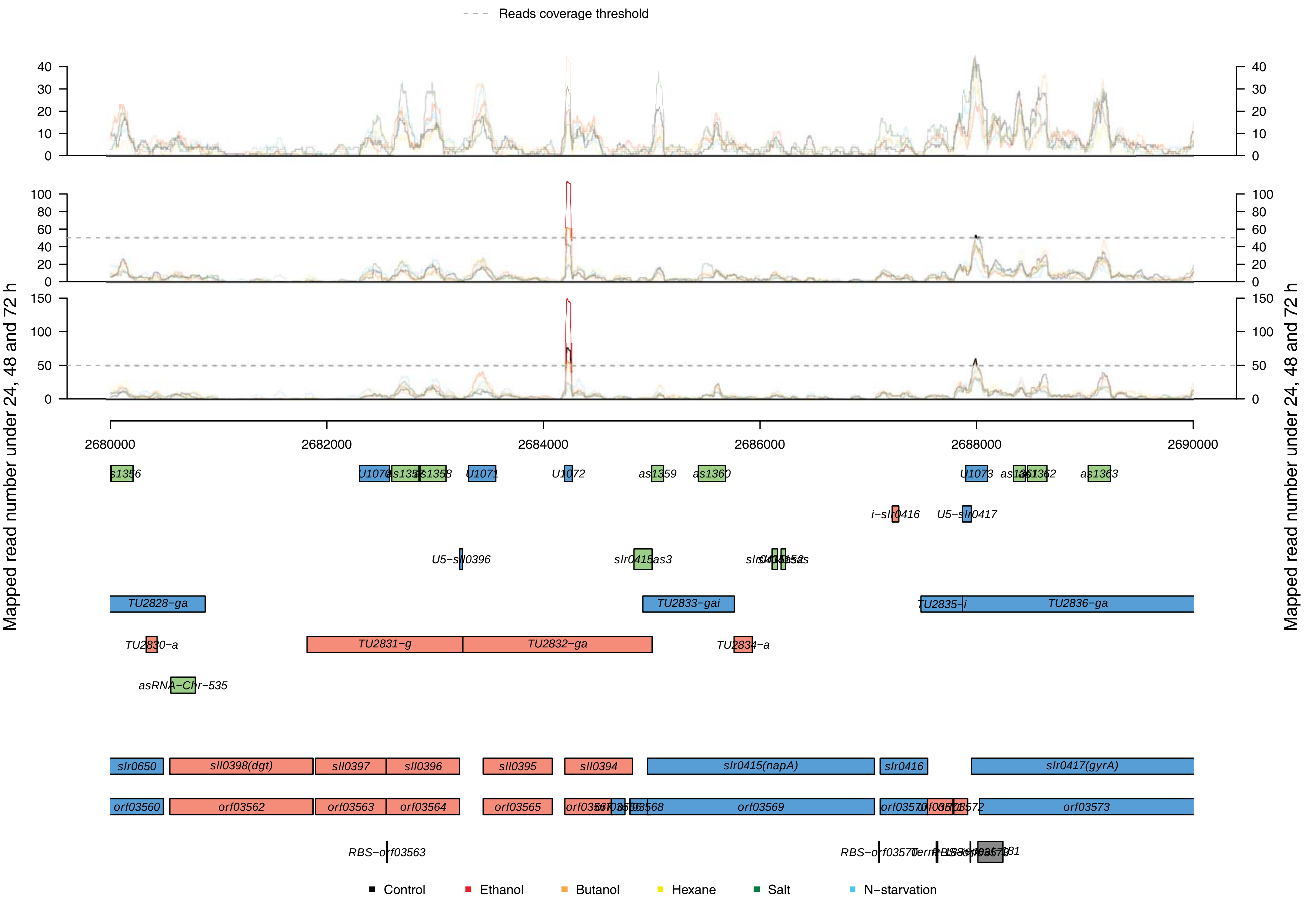

Reads coverage threshold
Mapped read number under 24, 48 and 72 h
2680000
2682000
2684000
2686000
2688000
2690000
TU2828−ga
TU2830−a
TU2831−g
TU2832−ga
TU2833−gai
TU2834−a
TU2836−ga
asRNA−Chr−535
U5−sll0396
slr0415as3
i−slr0416
U5−slr0417
slr0650
sll0398(dgt)
sll0397
sll0396
sll0395
sll0394
slr0415(napA)
slr0416
slr0417(gyrA)
orf03560
orf03562
orf03563
orf03564
orf03565
orf03569
orf03573
RBS−orf03563
Control
Ethanol
Butanol
Hexane
Salt
N−starvation

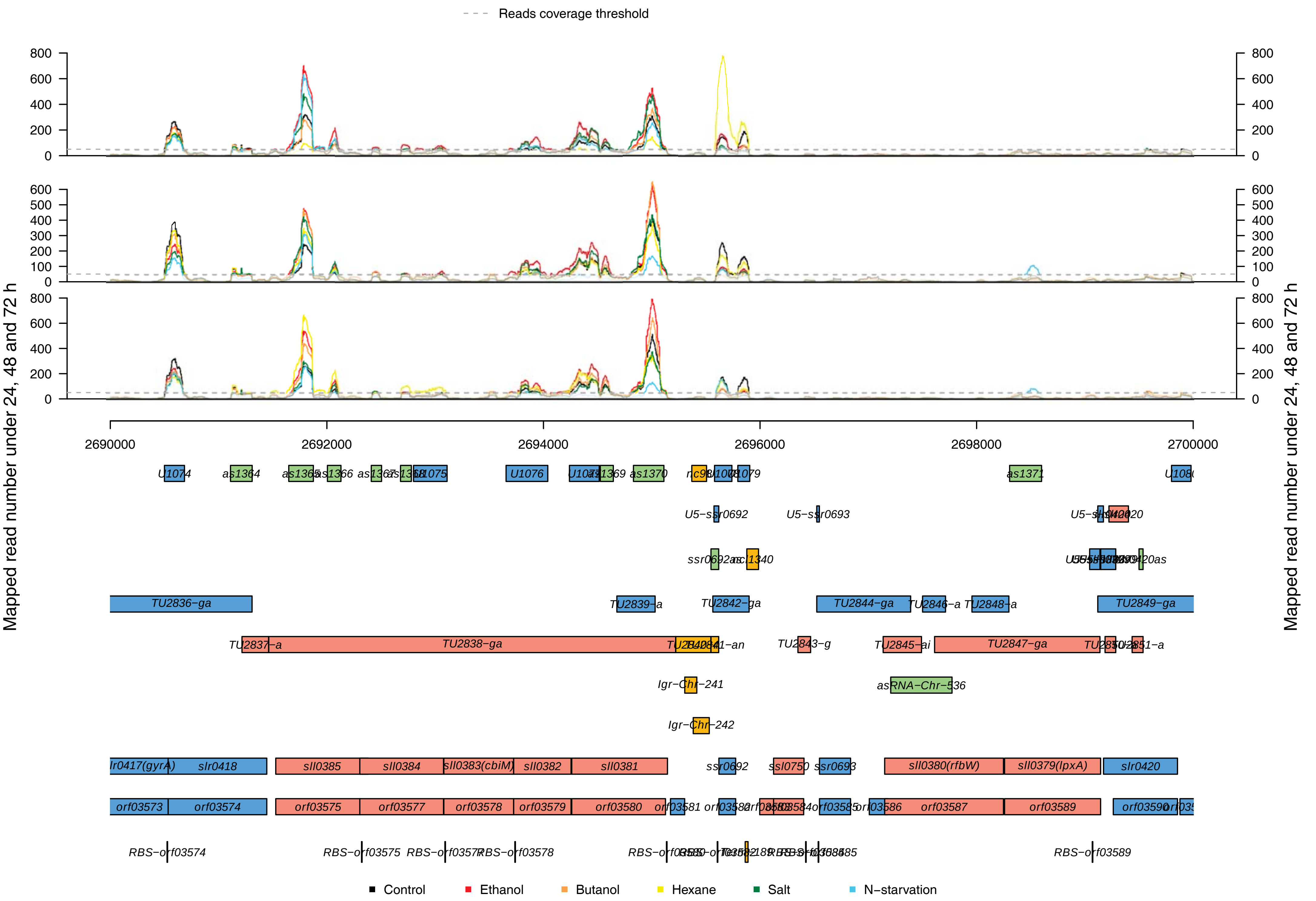

Reads coverage threshold
Mapped read number under 24, 48 and 72 h
2690000
2692000
2694000
2696000
2698000
2700000
TU2836−ga
TU2838−ga
TU2844−ga
TU2847−ga
TU2849−ga
asRNA−Chr−536
Igr−Chr−241
Igr−Chr−242
slr0418
sll0385
sll0384
sll0383(cbiM)
sll0382
sll0381
sll0380(rfbW)
sll0379(lpxA)
slr0420
orf03573
orf03574
orf03575
orf03577
orf03578
orf03579
orf03580
orf03587
orf03589
RBS−orf03574
RBS−orf03575
RBS−orf03578
RBS−orf03589
Control
Ethanol
Butanol
Hexane
Salt
N−starvation

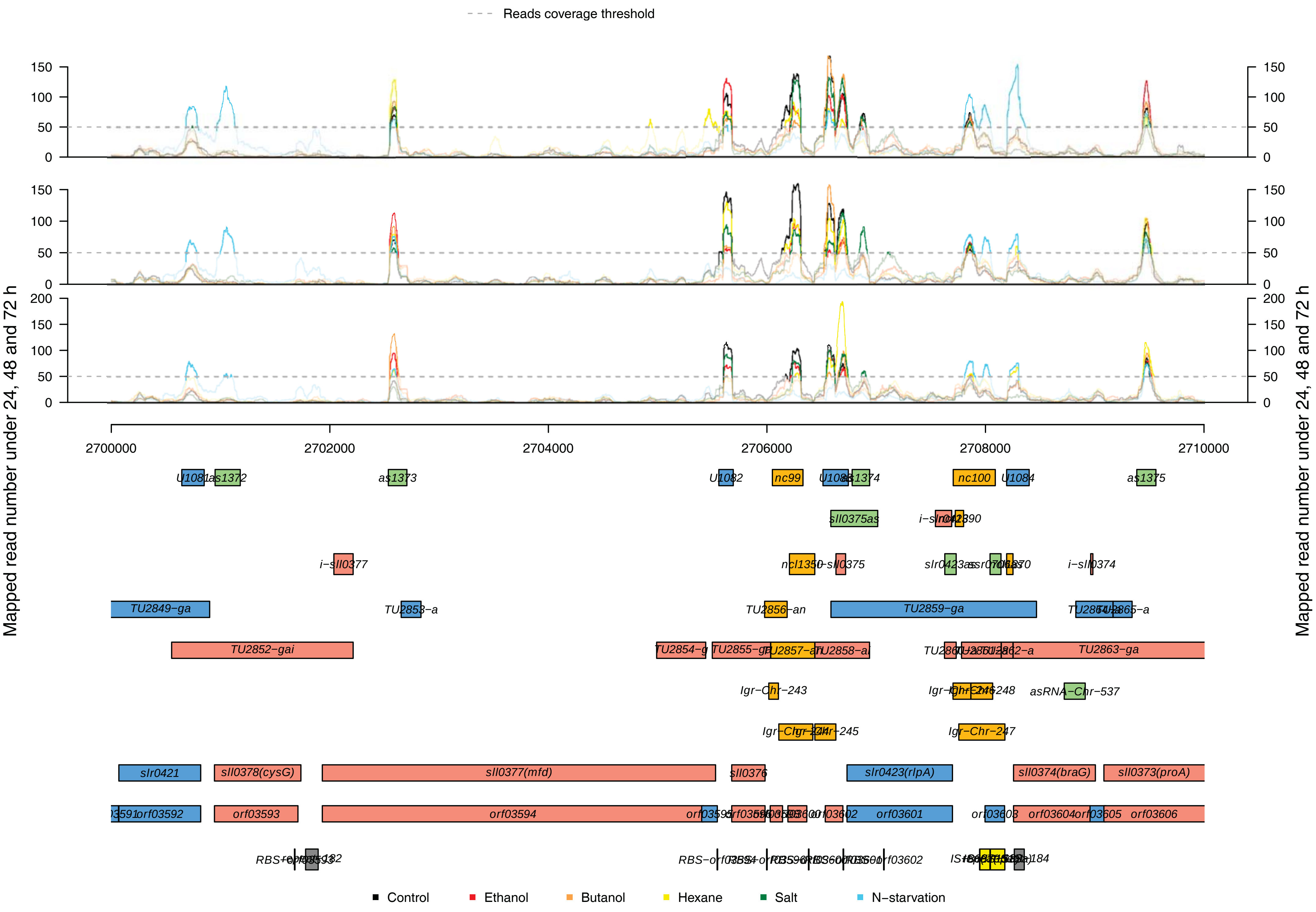

Reads coverage threshold
Mapped read number under 24, 48 and 72 h
0
50
100
150
200
2700000
2702000
2704000
2706000
2708000
2710000
as1372
as1373
nc99
as1374
nc100
as1375
sll0375as
i−sll0377
i−sll0374
TU2849−ga
TU2853−a
TU2856−an
TU2859−ga
TU2852−gai
TU2863−ga
Igr−Chr−243
Igr−Chr−247
asRNA−Chr−537
slr0421
sll0378(cysG)
sll0377(mfd)
sll0376
slr0423(rlpA)
sll0374(braG)
sll0373(proA)
orf03592
orf03593
orf03594
orf03601
orf03606
Control
Ethanol
Butanol
Hexane
Salt
N−starvation

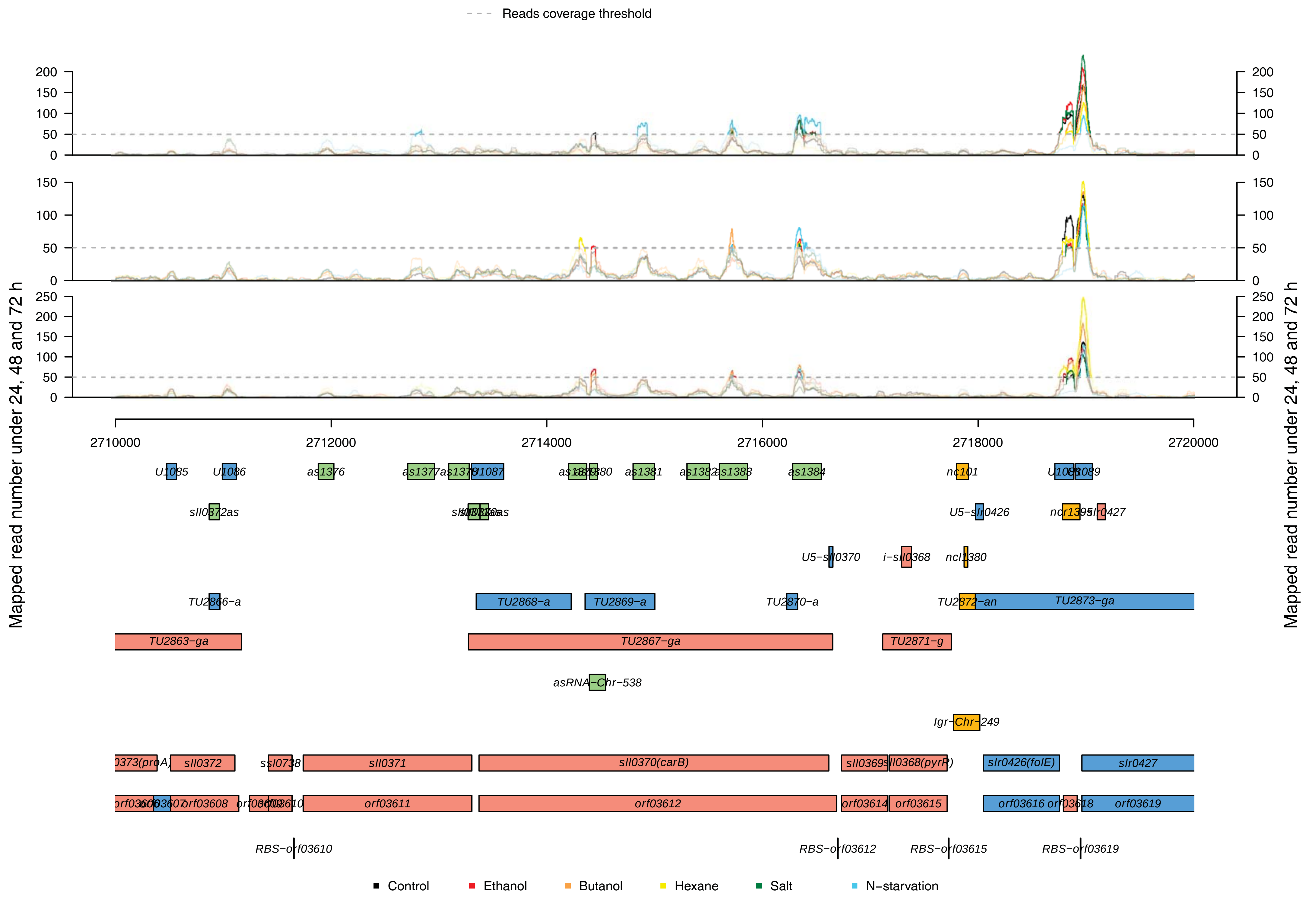

Reads coverage threshold
Mapped read number under 24, 48 and 72 h
2710000
2712000
2714000
2716000
2718000
2720000
U1085
U1086
as1376
as1377
U1087
as1381
as1382
as1383
as1384
nc101
sll0372as
U5−slr0426
slr0427
U5−sll0370
i−sll0368
ncl1380
TU2866−a
TU2868−a
TU2869−a
TU2870−a
TU2873−ga
TU2863−ga
TU2867−ga
TU2871−g
asRNA−Chr−538
Igr−Chr−249
sll0372
sll0371
sll0370(carB)
slr0426(folE)
slr0427
orf03608
orf03611
orf03612
orf03614
orf03615
orf03619
RBS−orf03610
RBS−orf03612
RBS−orf03615
RBS−orf03619
Control
Ethanol
Butanol
Hexane
Salt
N−starvation

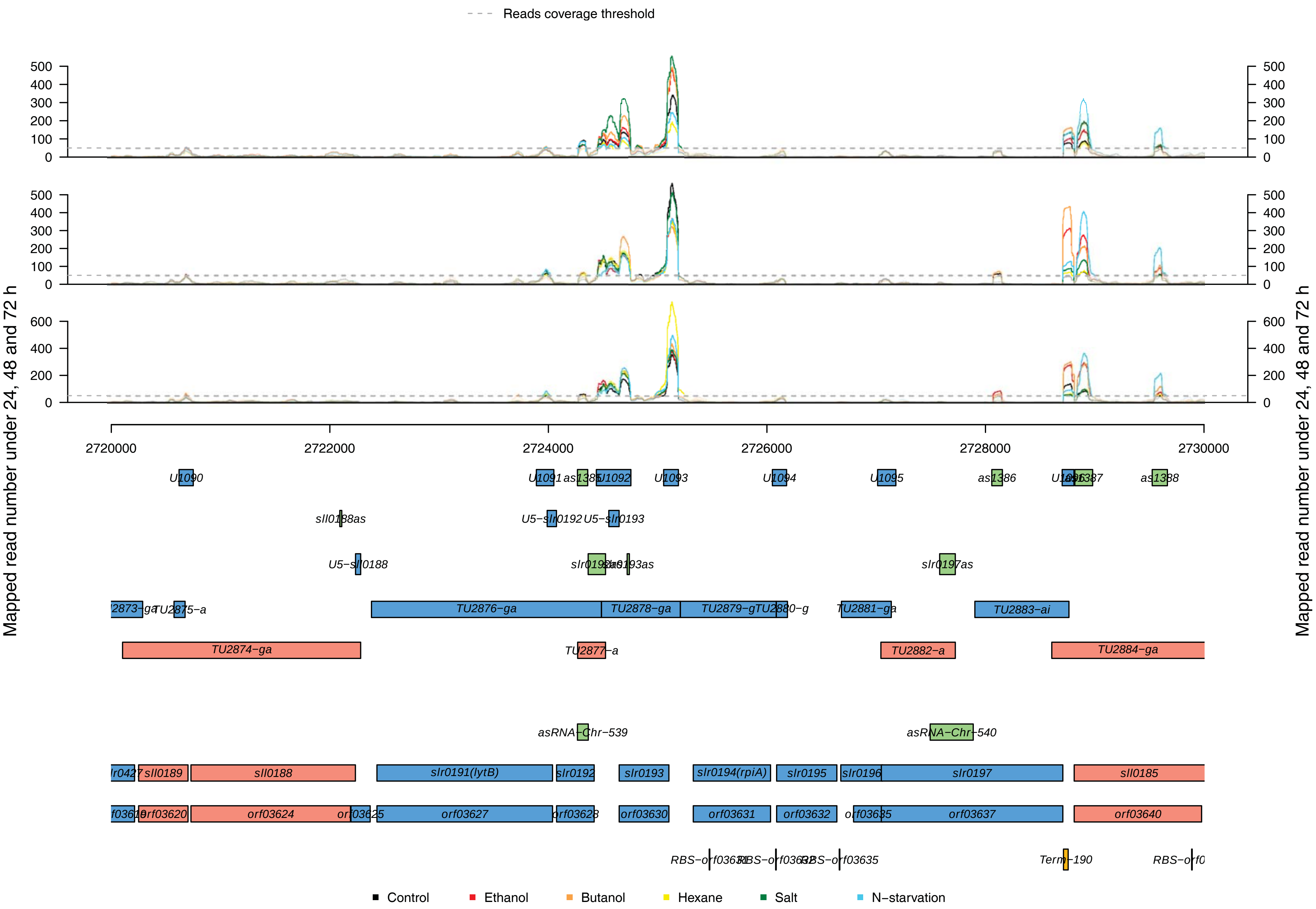

Reads coverage threshold
Mapped read number under 24, 48 and 72 h
2720000
2722000
2724000
2726000
2728000
2730000
TU2874−ga
TU2876−ga
TU2878−ga
TU2882−a
TU2883−ai
TU2884−ga
asRNA−Chr−539
asRNA−Chr−540
sll0189
sll0188
slr0191(lytB)
slr0192
slr0193
slr0194(rpiA)
slr0195
slr0196
slr0197
sll0185
orf03620
orf03624
orf03627
orf03630
orf03631
orf03632
orf03637
orf03640
Control
Ethanol
Butanol
Hexane
Salt
N−starvation

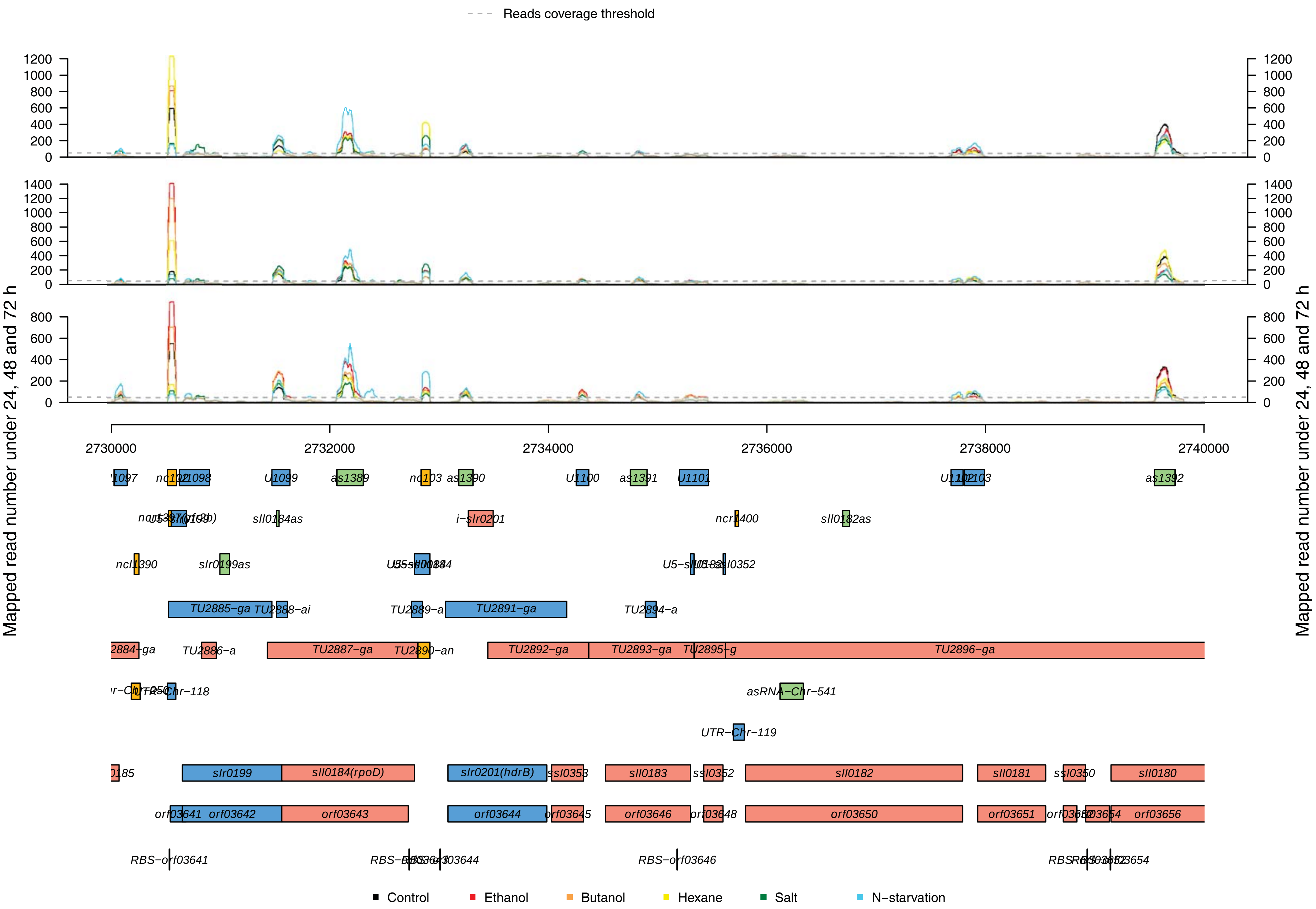

Reads coverage threshold
Mapped read number under 24, 48 and 72 h
2730000
2732000
2734000
2736000
2738000
2740000
TU2885−ga
TU2887−ga
TU2891−ga
TU2892−ga
TU2893−ga
TU2896−ga
asRNA−Chr−541
UTR−Chr−119
slr0199
sll0184(rpoD)
slr0201(hdrB)
sll0183
sll0182
sll0181
sll0180
orf03643
orf03644
orf03646
orf03650
orf03651
orf03656
RBS−orf03641
RBS−orf03646
Control
Ethanol
Butanol
Hexane
Salt
N−starvation

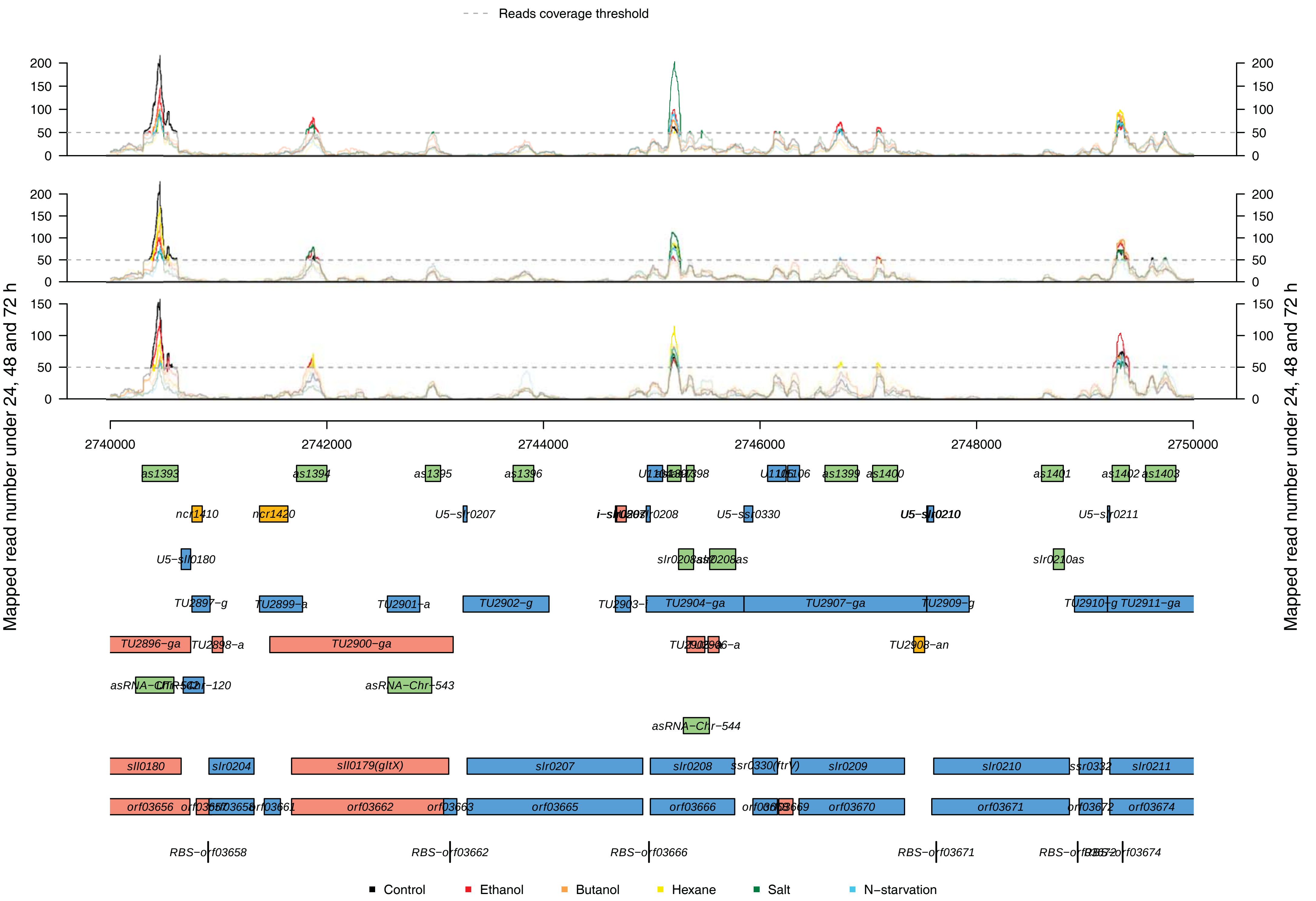
Reads coverage threshold
Mapped read number under 24, 48 and 72 h
200
150
100
50
0
2740000
2742000
2744000
2746000
2748000
2750000
as1393
as1394
as1395
as1396
as1399
as1400
as1401
as1402
as1403
ncr1410
ncr1420
U5−slr0207
U5−ssr0330
U5−slr0210
U5−slr0211
U5−sll0180
slr0210as
TU2897−g
TU2899−a
TU2901−a
TU2902−g
TU2904−ga
TU2907−ga
TU2909−g
TU2910−g
TU2911−ga
TU2896−ga
TU2898−a
TU2900−ga
TU2908−an
asRNA−Chr−543
asRNA−Chr−544
sll0180
slr0204
sll0179(gltX)
slr0207
slr0208
ssr0330(ftrV)
slr0209
slr0210
ssr0332
slr0211
orf03656
orf03662
orf03665
orf03666
orf03670
orf03671
orf03672
orf03674
RBS−orf03658
RBS−orf03662
RBS−orf03666
RBS−orf03671
RBS−orf03674
Control
Ethanol
Butanol
Hexane
Salt
N−starvation

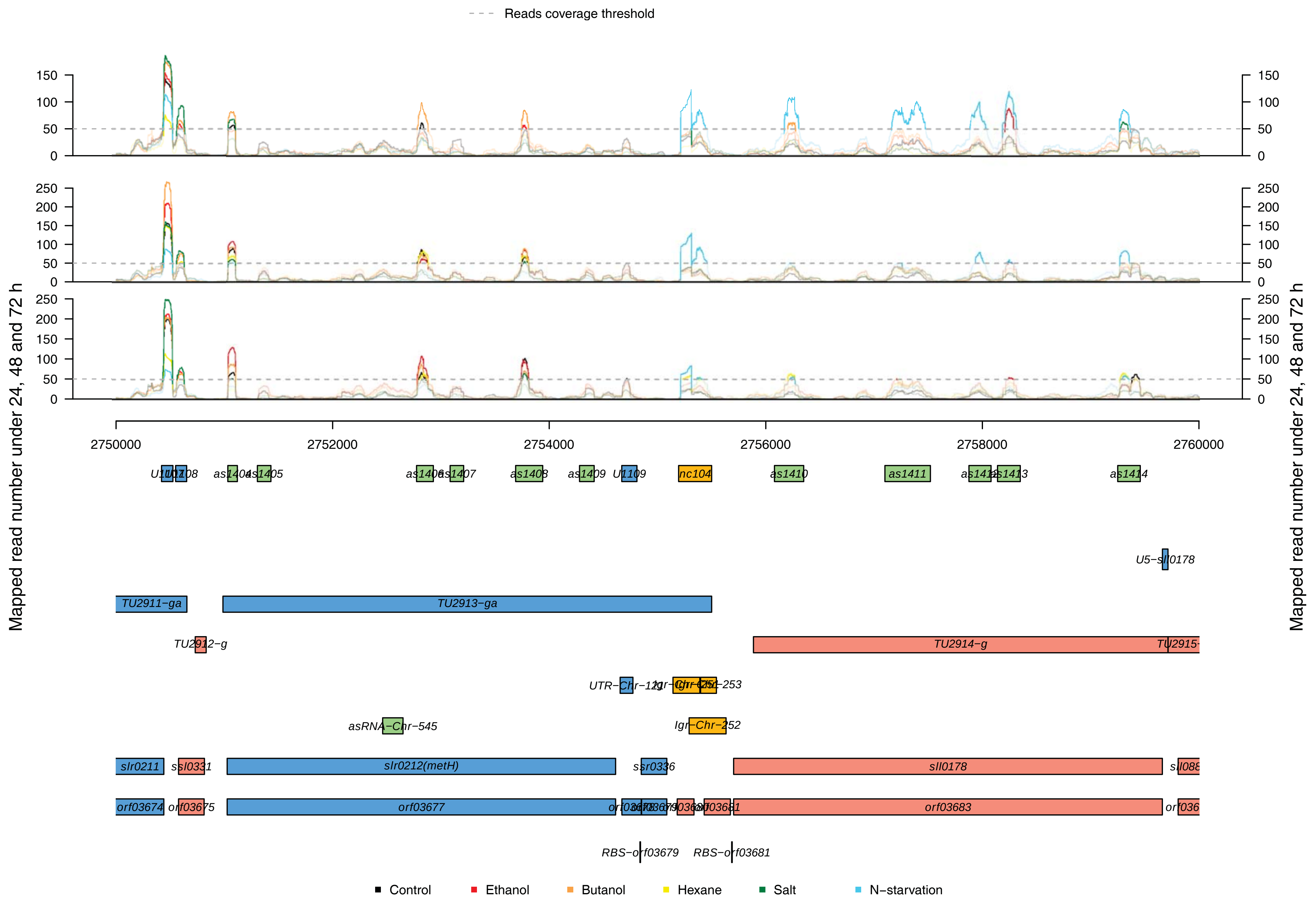

Reads coverage threshold
Mapped read number under 24, 48 and 72 h
150
100
50
0
250
200
150
100
50
0
2750000
2752000
2754000
2756000
2758000
2760000
as1404
as1405
as1406
as1407
as1408
as1409
U1109
nc104
as1410
as1411
as1413
as1414
U5−sll0178
TU2911−ga
TU2913−ga
TU2912−g
TU2914−g
UTR−Chr−129
Igr−Chr−253
asRNA−Chr−545
Igr−Chr−252
slr0211
ssl0331
slr0212(metH)
ssr0336
sll0178
orf03674
orf03675
orf03677
orf03681
orf03683
RBS−orf03679
RBS−orf03681
Control
Ethanol
Butanol
Hexane
Salt
N−starvation

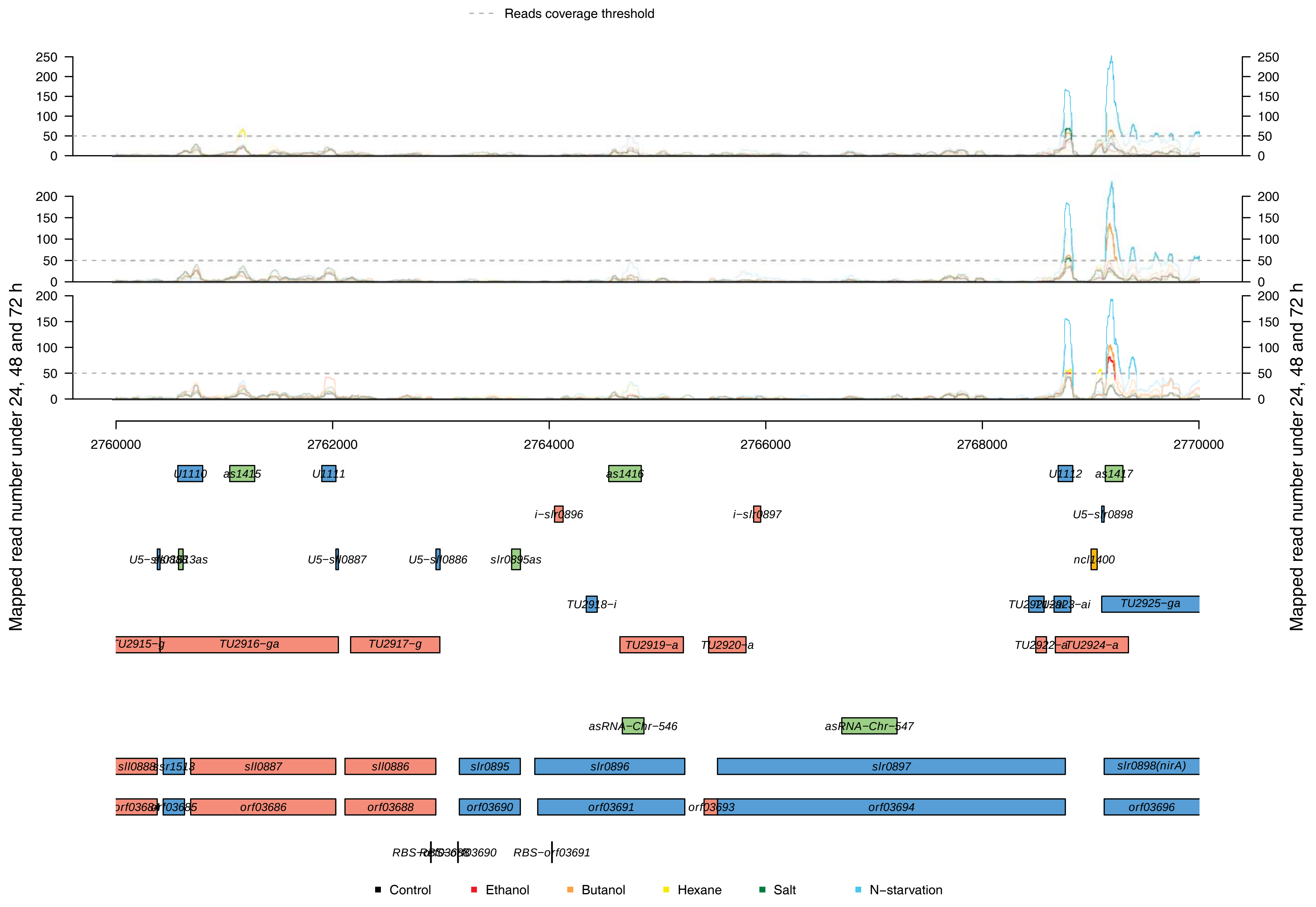

Reads coverage threshold
Mapped read number under 24, 48 and 72 h
2760000
2762000
2764000
2766000
2768000
2770000
U1110
as1415
U1111
as1416
U1112
as1417
i−slr0896
i−slr0897
U5−slr0898
U5−sll0887
U5−sll0886
slr0895as
ncl1400
TU2918−i
TU2925−ga
TU2916−ga
TU2917−g
TU2919−a
TU2920−a
TU2924−a
asRNA−Chr−546
asRNA−Chr−547
sll0887
sll0886
slr0895
slr0896
slr0897
slr0898(nirA)
orf03686
orf03688
orf03690
orf03691
orf03694
orf03696
RBS−orf03691
Control
Ethanol
Butanol
Hexane
Salt
N−starvation

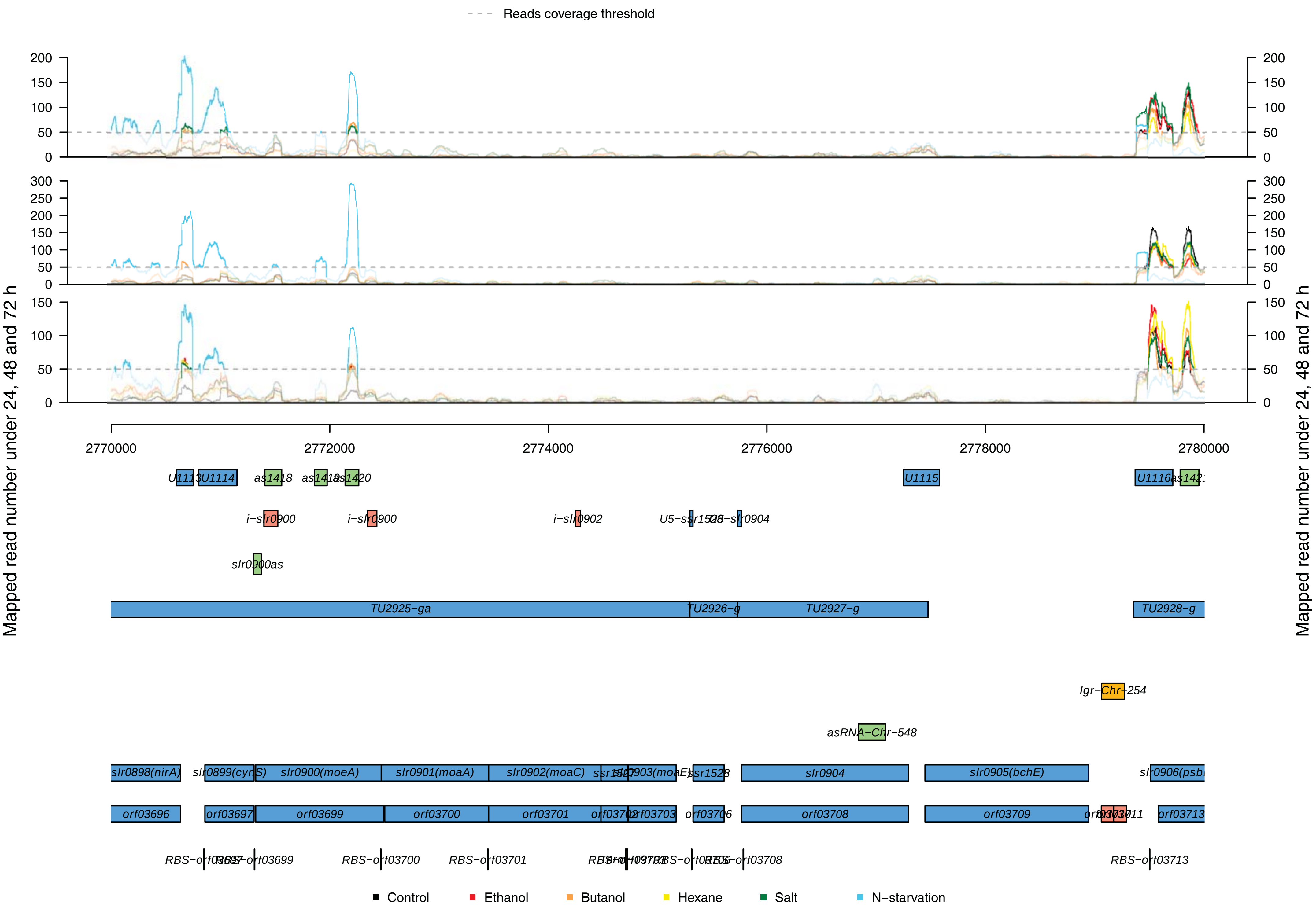

Reads coverage threshold
Mapped read number under 24, 48 and 72 h
2770000
2772000
2774000
2776000
2778000
2780000
TU2925−ga
TU2926−g
TU2927−g
TU2928−g
slr0900as
Igr−Chr−254
asRNA−Chr−548
slr0898(nirA)
slr0899(cynS)
slr0900(moeA)
slr0901(moaA)
slr0902(moaC)
slr0904
slr0905(bchE)
orf03696
orf03697
orf03699
orf03700
orf03701
orf03703
orf03706
orf03708
orf03709
orf03713
RBS−orf03700
RBS−orf03701
RBS−orf03708
RBS−orf03713
Control
Ethanol
Butanol
Hexane
Salt
N−starvation

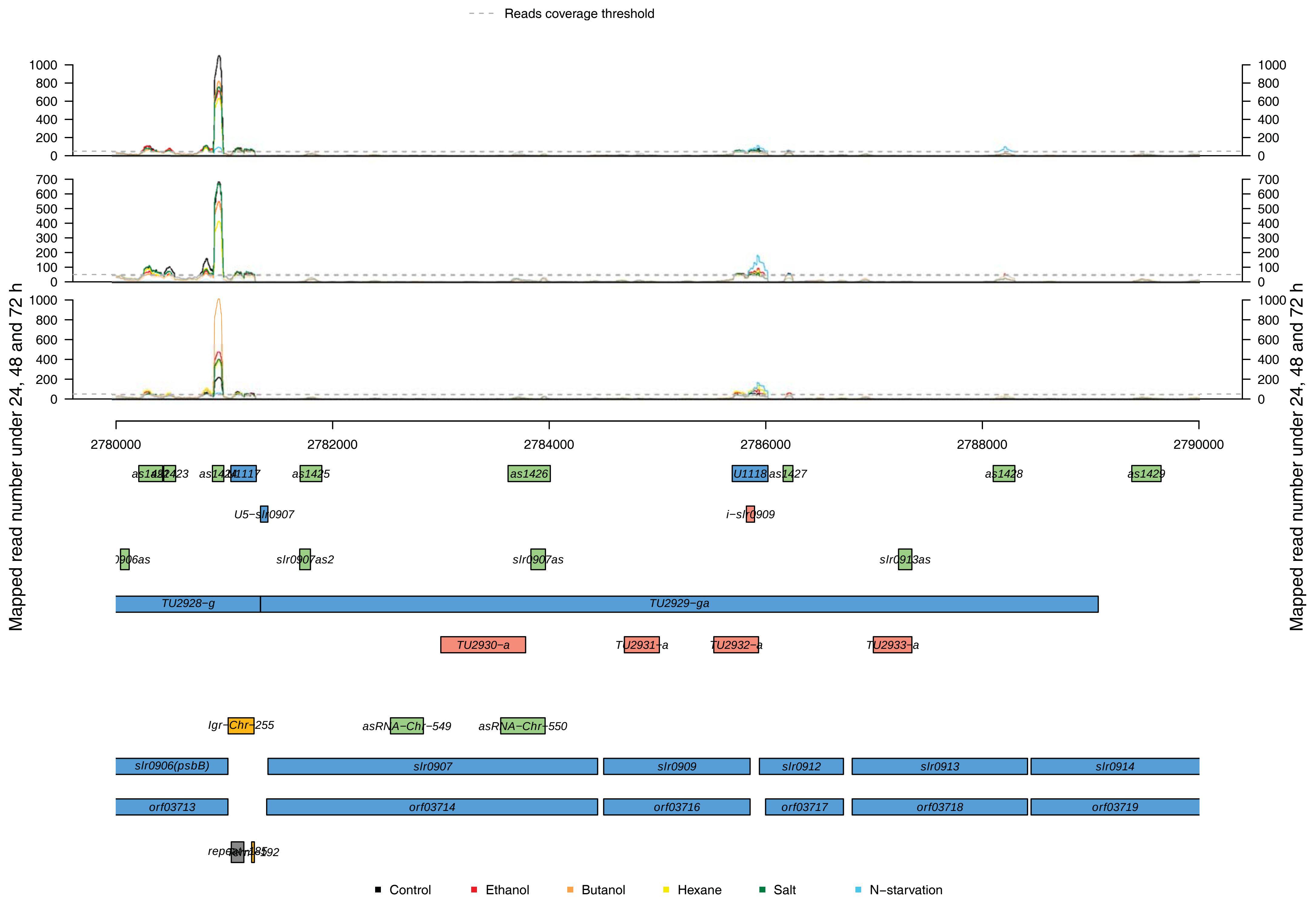

Reads coverage threshold
Mapped read number under 24, 48 and 72 h
2780000
2782000
2784000
2786000
2788000
2790000
TU2928−g
TU2929−ga
TU2930−a
slr0906(psbB)
slr0907
slr0909
slr0912
slr0913
slr0914
orf03713
orf03714
orf03716
orf03717
orf03718
orf03719
Control
Ethanol
Butanol
Hexane
Salt
N−starvation

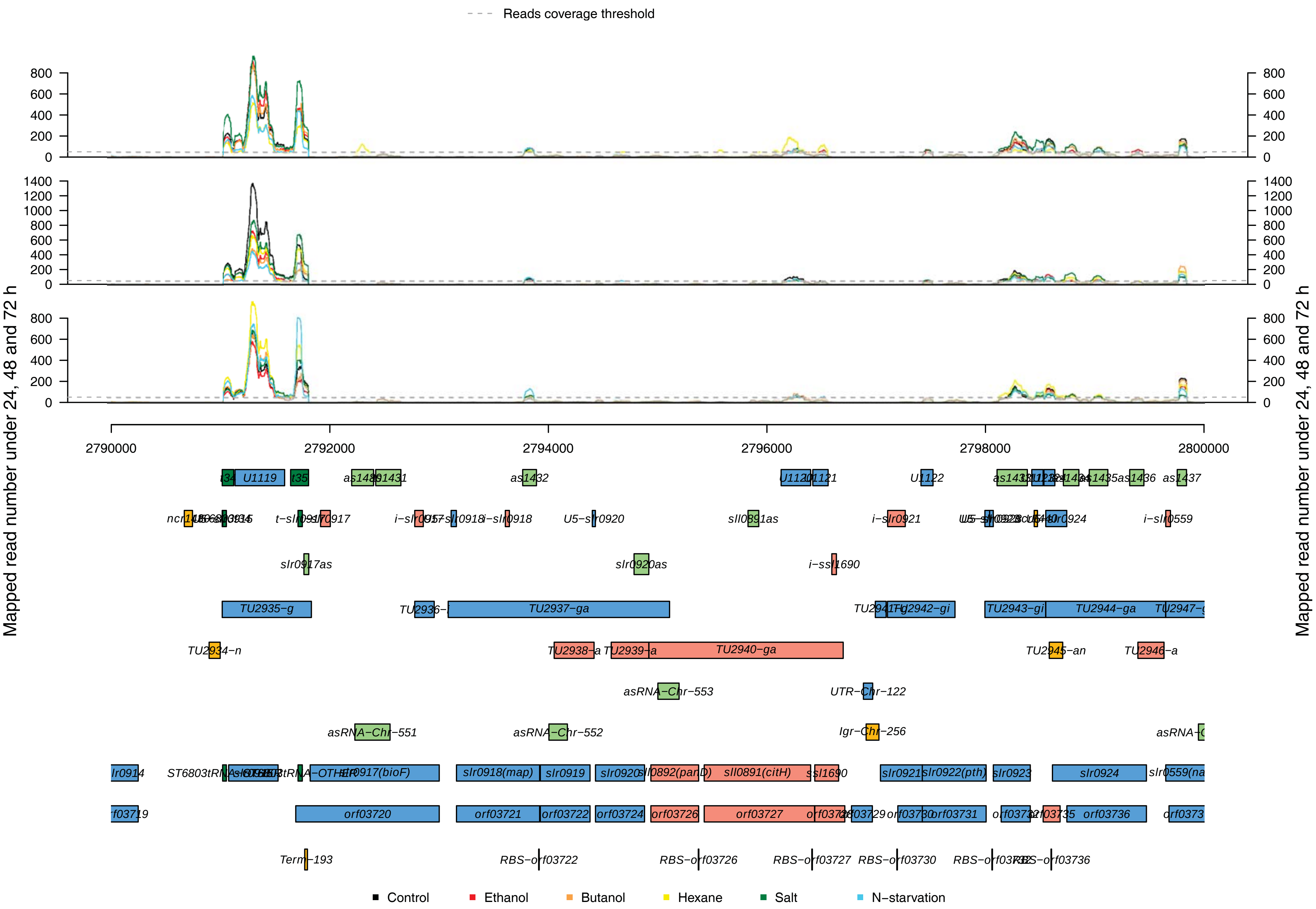

Reads coverage threshold
Mapped read number under 24, 48 and 72 h
0
200
400
600
800
1000
1200
1400
2790000
2792000
2794000
2796000
2798000
2800000
U1119
t35
as1432
U1122
as1436
as1437
t−sll0917
i−slr0921
U5−slr0920
sll0891as
i−slr0559
slr0917as
slr0920as
i−ssl1690
TU2935−g
TU2937−ga
TU2942−gi
TU2943−gi
TU2944−ga
TU2934−n
TU2940−ga
TU2945−an
TU2946−a
asRNA−Chr−553
UTR−Chr−122
asRNA−Chr−551
asRNA−Chr−552
Igr−Chr−256
slr0917(bioF)
slr0918(map)
slr0919
sll0892(panD)
sll0891(citH)
ssl1690
slr0922(pth)
slr0923
slr0924
orf03720
orf03721
orf03722
orf03724
orf03726
orf03727
orf03731
orf03736
Term−193
RBS−orf03722
RBS−orf03726
RBS−orf03727
RBS−orf03730
Control
Ethanol
Butanol
Hexane
Salt
N−starvation

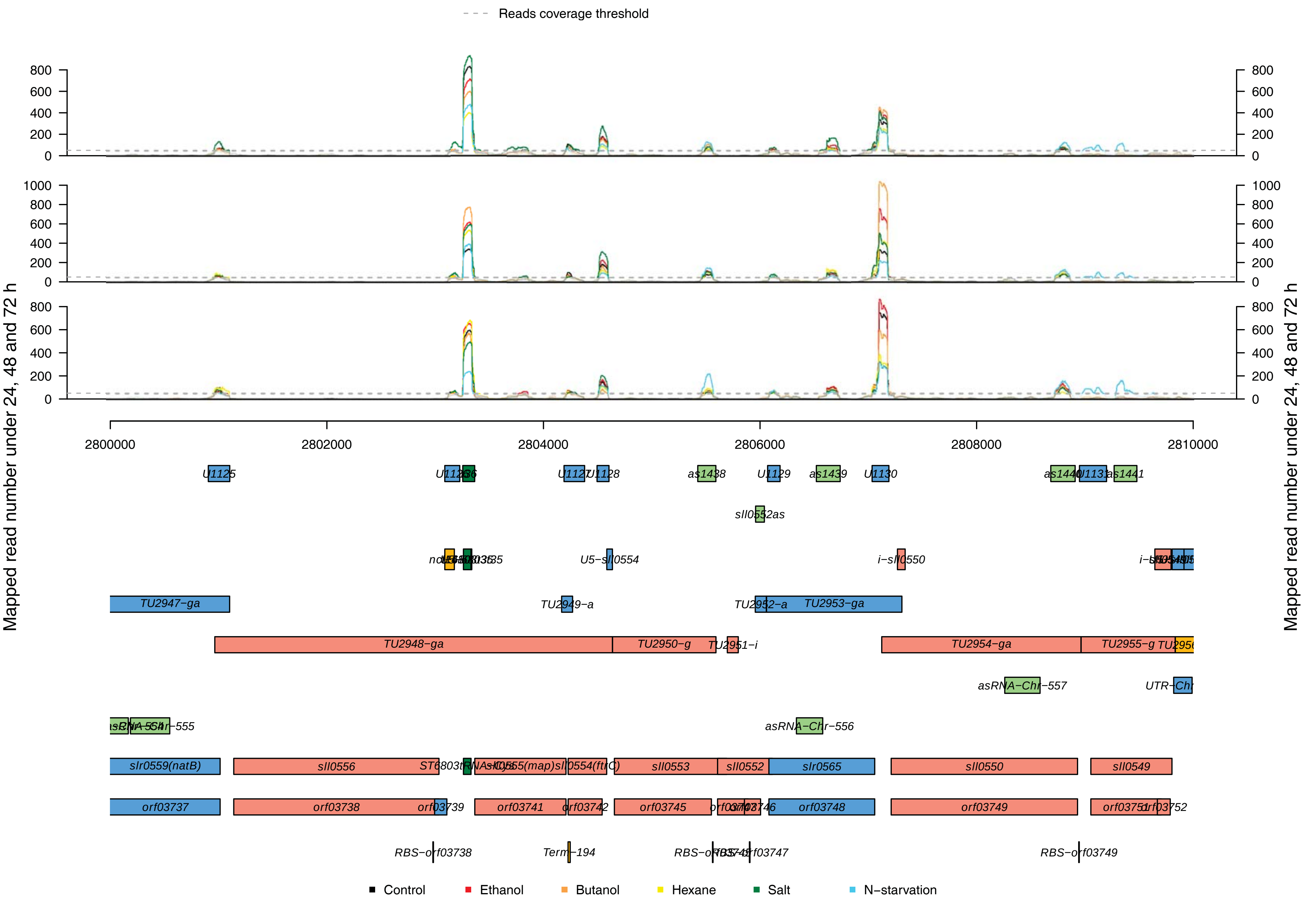

Reads coverage threshold
Mapped read number under 24, 48 and 72 h
2800000
2802000
2804000
2806000
2808000
2810000
TU2947−ga
TU2948−ga
TU2949−a
TU2950−g
TU2952−a
TU2953−ga
TU2954−ga
asRNA−Chr−557
asRNA−Chr−556
sll0552as
i−sll0550
U5−sll0554
slr0559(natB)
sll0556
sll0553
sll0552
slr0565
sll0550
sll0549
orf03737
orf03738
orf03741
orf03742
orf03745
orf03748
orf03749
RBS−orf03738
Term−194
RBS−orf03749
Control
Ethanol
Butanol
Hexane
Salt
N−starvation

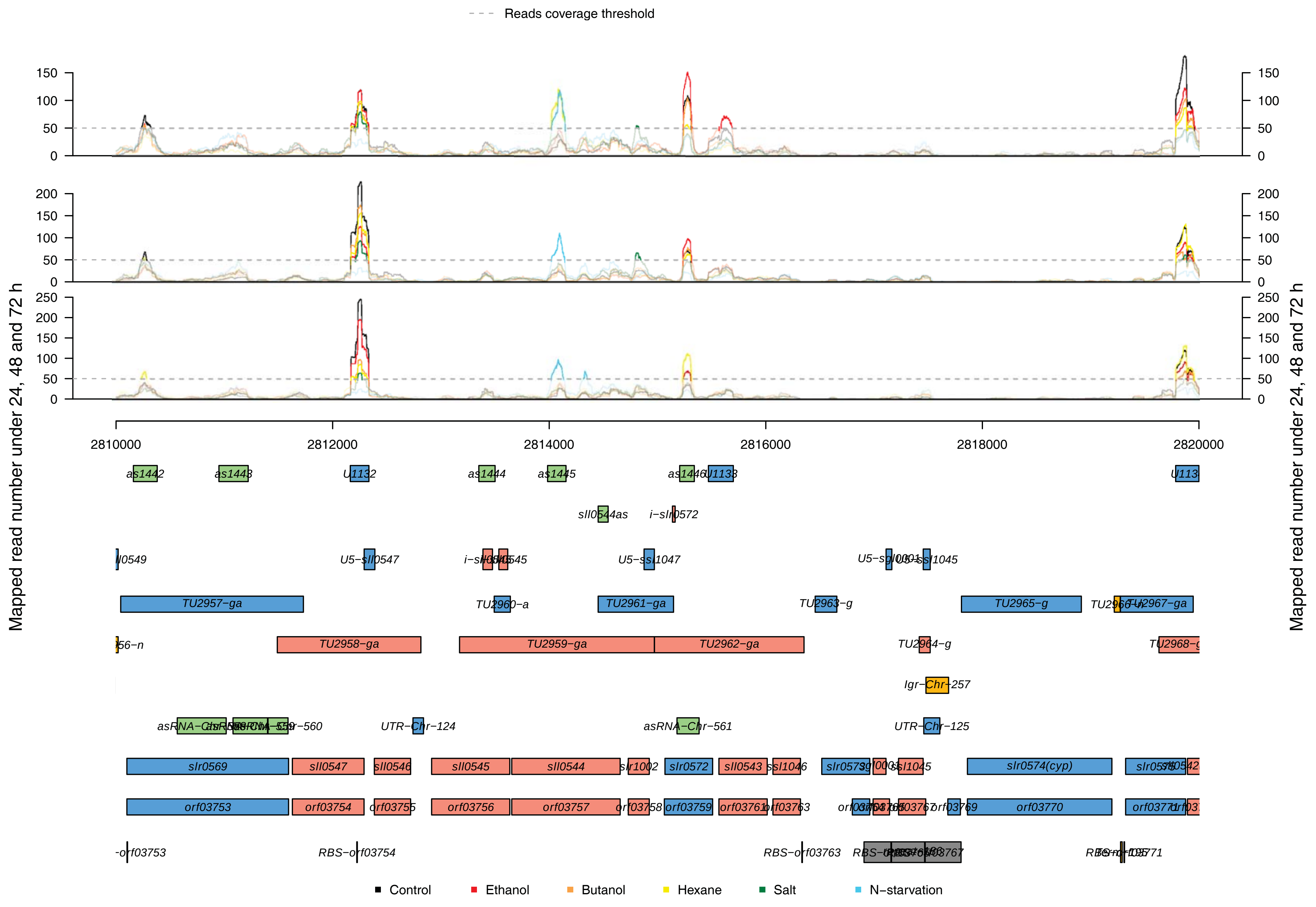

Reads coverage threshold
Mapped read number under 24, 48 and 72 h
2810000
2812000
2814000
2816000
2818000
2820000
as1442
as1443
as1444
as1445
TU2957−ga
TU2958−ga
TU2959−ga
TU2961−ga
TU2962−ga
TU2965−g
slr0569
sll0547
sll0545
sll0544
slr0572
sll0543
slr0574(cyp)
orf03753
orf03754
orf03756
orf03757
orf03759
orf03770
UTR−Chr−124
asRNA−Chr−561
UTR−Chr−125
Igr−Chr−257
RBS−orf03754
RBS−orf03763
Control
Ethanol
Butanol
Hexane
Salt
N−starvation

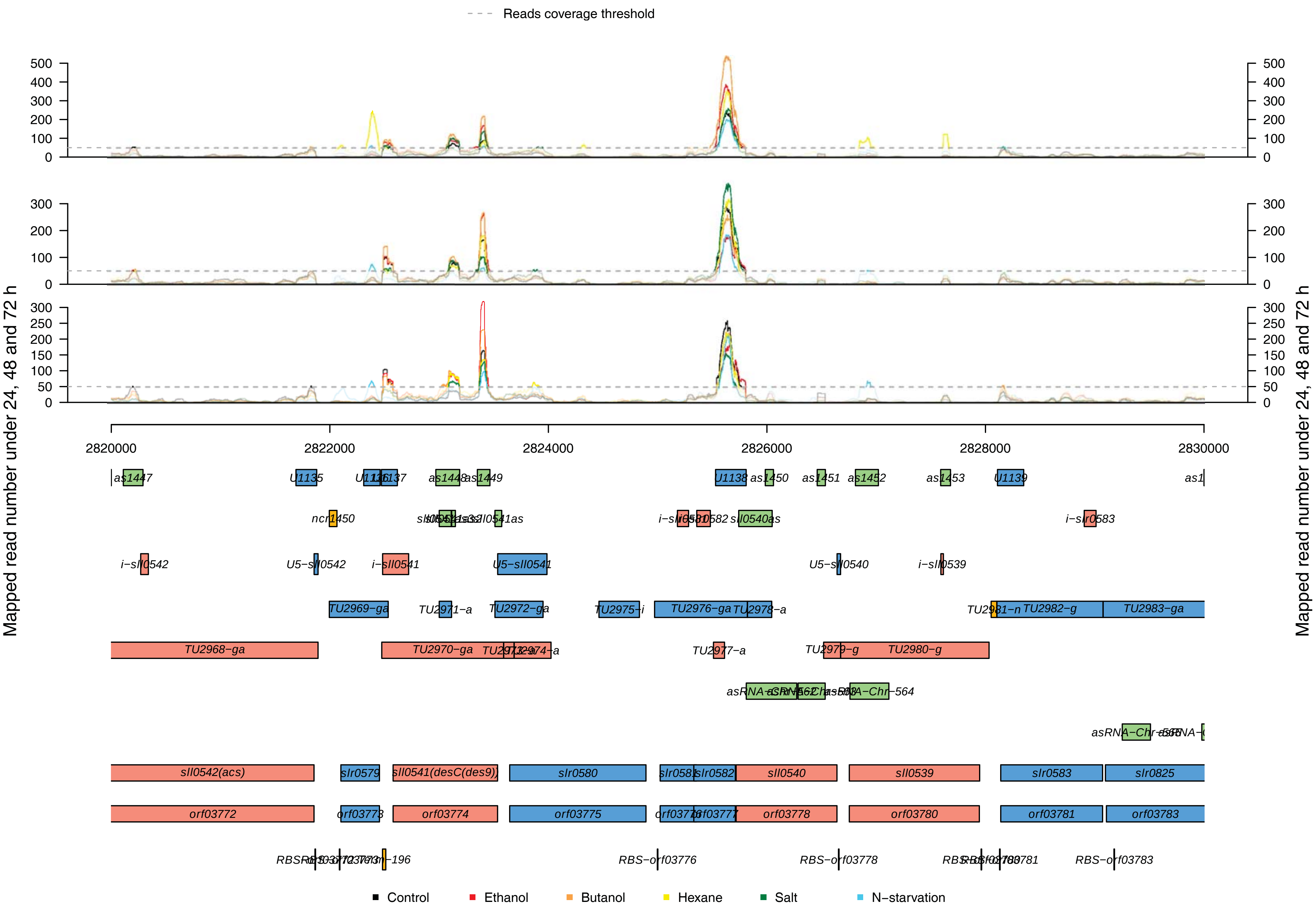

Reads coverage threshold
Mapped read number under 24, 48 and 72 h
500
400
300
200
100
0
300
250
150
50
2820000
2822000
2824000
2826000
2828000
2830000
as1447
U1135
as1448
as1449
U1138
as1450
as1451
as1452
as1453
U1139
ncr1450
i−slr0583
i−sll0542
U5−sll0542
i−sll0541
U5−sll0541
U5−sll0540
i−sll0539
TU2969−ga
TU2971−a
TU2972−ga
TU2976−ga
TU2978−a
TU2982−g
TU2983−ga
TU2968−ga
TU2970−ga
TU2977−a
TU2980−g
asRNA−Chr−564
sll0542(acs)
slr0579
sll0541(desC(des9))
slr0580
slr0582
sll0540
sll0539
slr0583
slr0825
orf03772
orf03774
orf03775
orf03778
orf03780
orf03781
orf03783
RBS−orf03776
RBS−orf03778
RBS−orf03783
Control
Ethanol
Butanol
Hexane
Salt
N−starvation

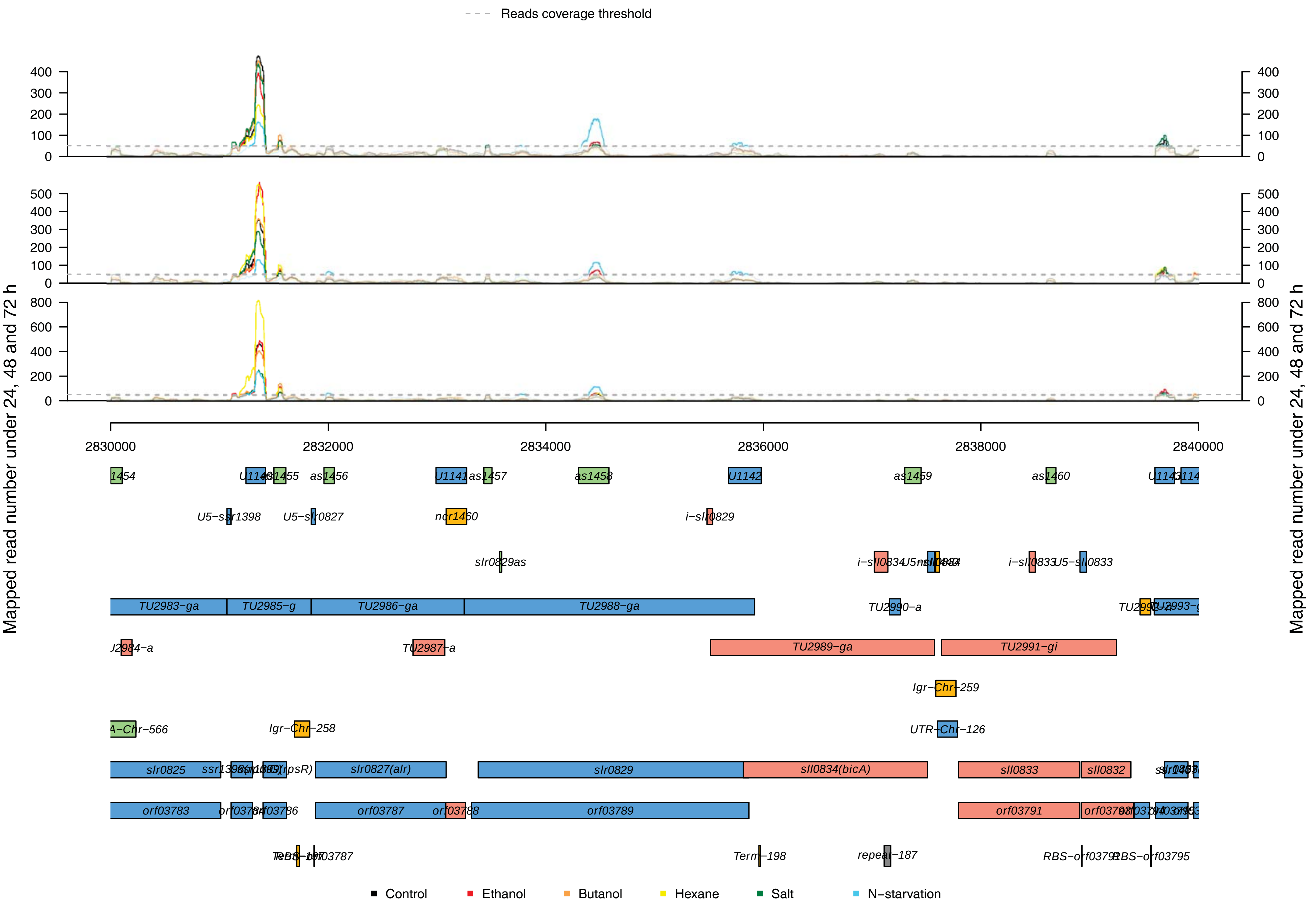

Reads coverage threshold
Mapped read number under 24, 48 and 72 h
2830000
2832000
2834000
2836000
2838000
2840000
TU2983−ga
TU2985−g
TU2986−ga
TU2988−ga
TU2989−ga
TU2991−gi
TU2990−a
TU2987−a
slr0825
slr0827(alr)
slr0829
sll0834(bicA)
sll0833
sll0832
orf03783
orf03787
orf03789
orf03791
ncr1460
slr0829as
Igr−Chr−259
Igr−Chr−258
UTR−Chr−126
Term−198
repeat−187
Control
Ethanol
Butanol
Hexane
Salt
N−starvation

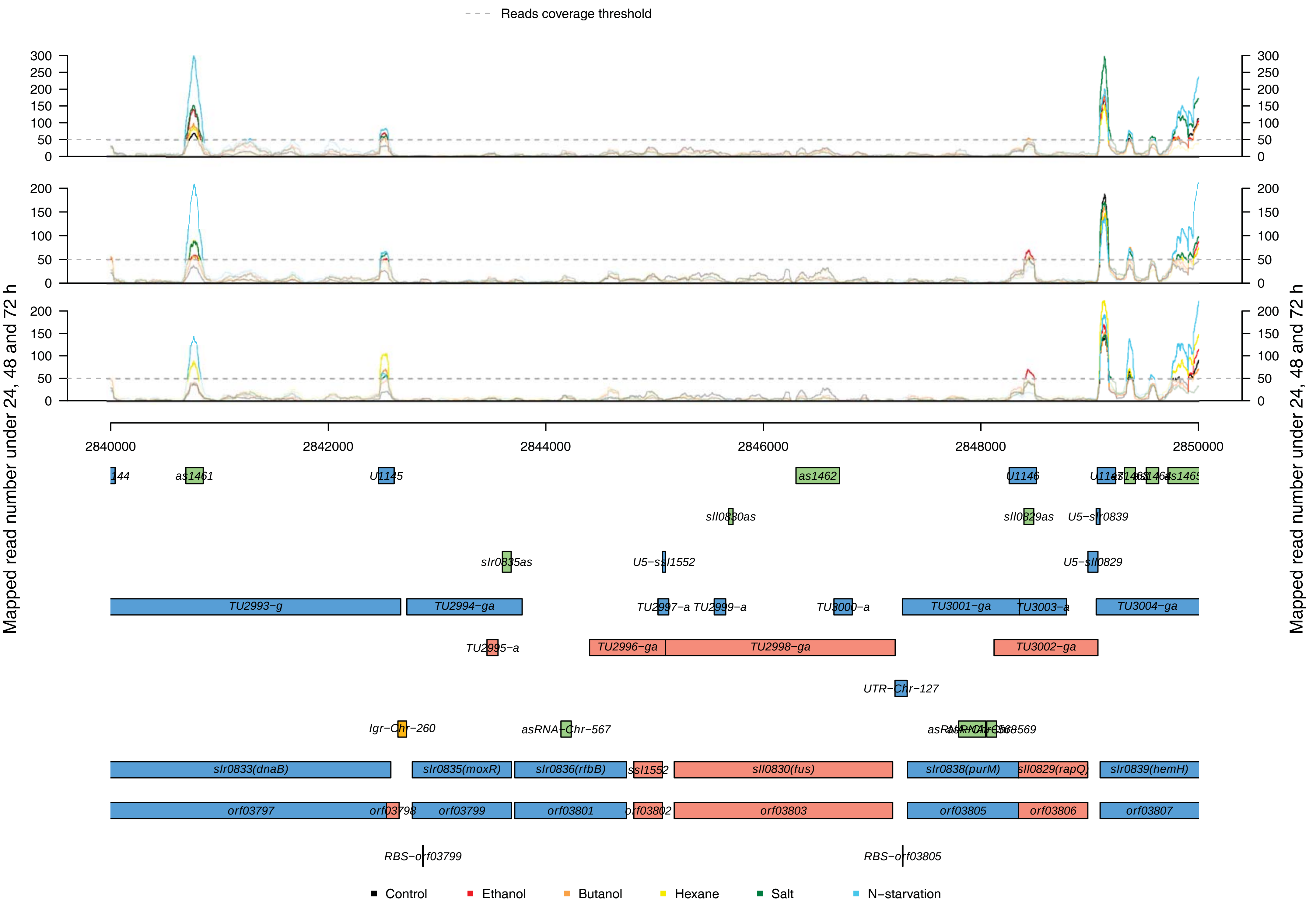

Reads coverage threshold
Mapped read number under 24, 48 and 72 h
2840000
2842000
2844000
2846000
2848000
2850000
as1461
U1145
as1462
U1146
sll0830as
sll0829as
U5−slr0839
slr0835as
U5−ssl1552
U5−sll0829
TU2993−g
TU2994−ga
TU2997−a
TU2999−a
TU3000−a
TU3001−ga
TU3003−a
TU3004−ga
TU2995−a
TU2996−ga
TU2998−ga
TU3002−ga
UTR−Chr−127
Igr−Chr−260
asRNA−Chr−567
slr0833(dnaB)
slr0835(moxR)
slr0836(rfbB)
ssl1552
sll0830(fus)
slr0838(purM)
sll0829(rapQ)
slr0839(hemH)
orf03797
orf03798
orf03799
orf03801
orf03802
orf03803
orf03805
orf03806
orf03807
RBS−orf03799
RBS−orf03805
Control
Ethanol
Butanol
Hexane
Salt
N−starvation

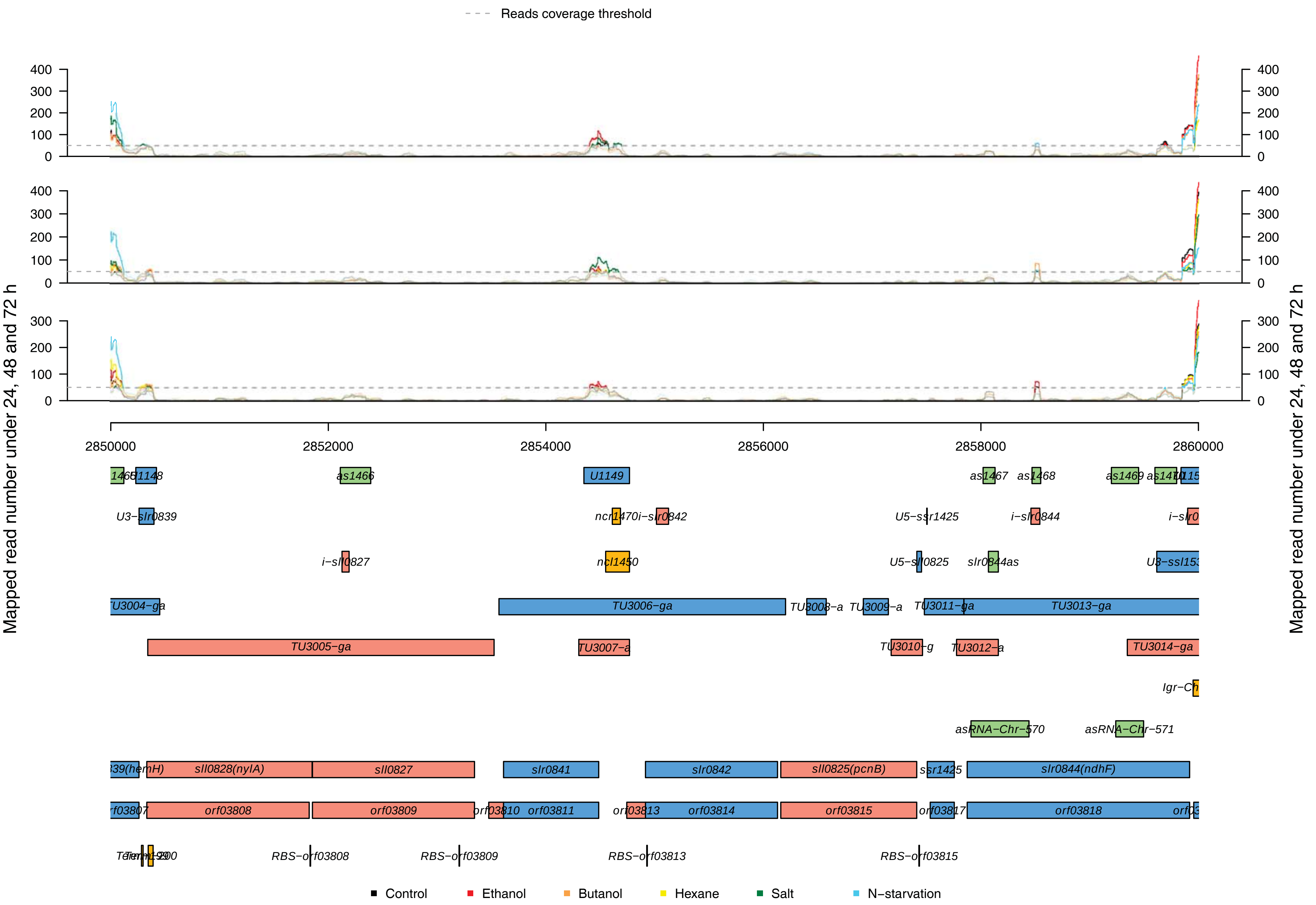

Reads coverage threshold
Mapped read number under 24, 48 and 72 h
2850000
2852000
2854000
2856000
2858000
2860000
U1149
as1466
TU3006−ga
TU3005−ga
TU3013−ga
slr0842
sll0825(pcnB)
slr0844(ndhF)
orf03818
Control
Ethanol
Butanol
Hexane
Salt
N−starvation

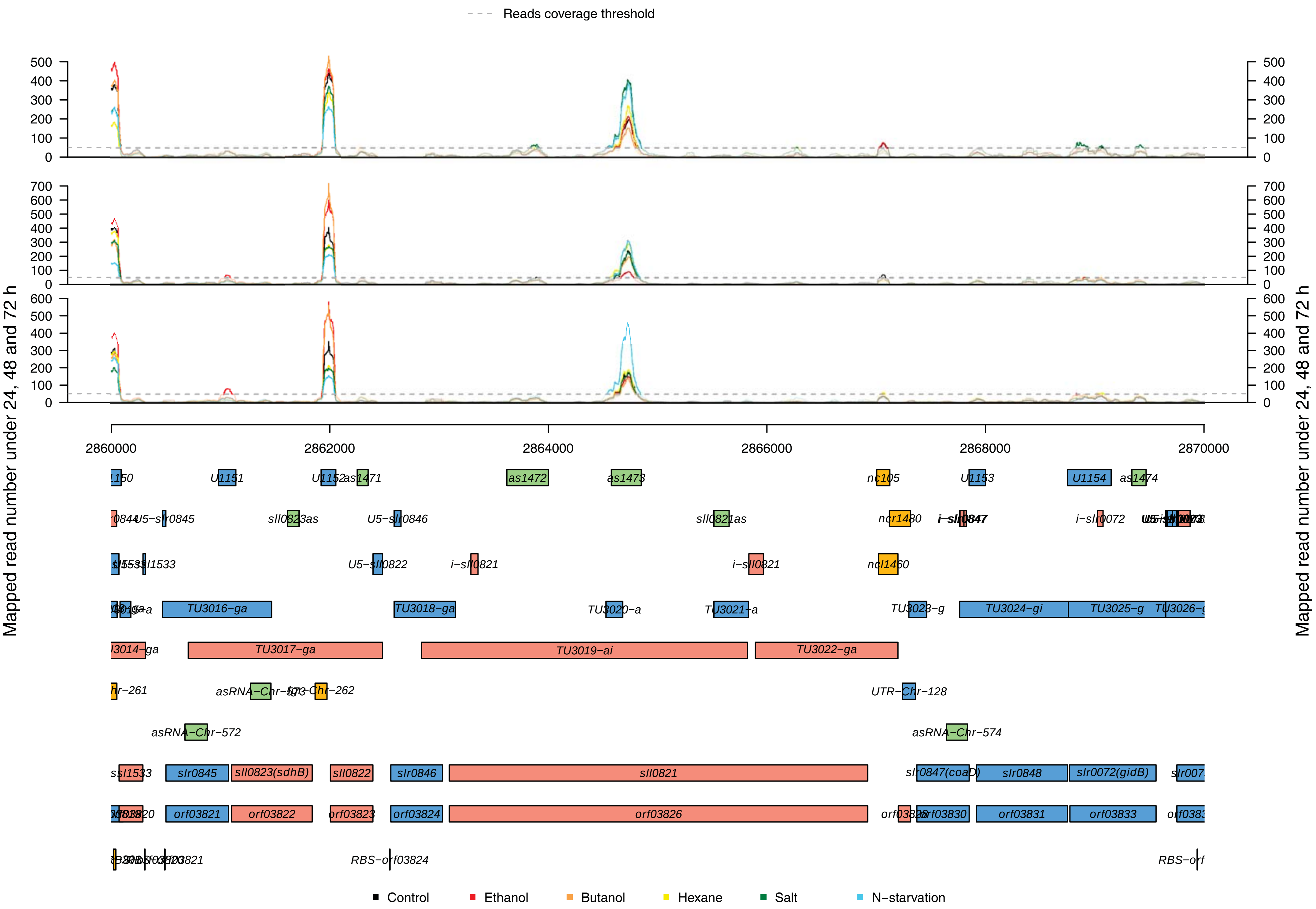

Reads coverage threshold
Mapped read number under 24, 48 and 72 h
2860000
2862000
2864000
2866000
2868000
2870000
as1472
TU3016−ga
TU3018−ga
TU3024−gi
TU3025−g
TU3017−ga
TU3019−ai
TU3022−ga
asRNA−Chr−572
asRNA−Chr−574
UTR−Chr−128
slr0845
sll0823(sdhB)
sll0822
slr0846
sll0821
slr0848
slr0072(gidB)
orf03821
orf03822
orf03824
orf03826
orf03831
orf03833
RBS−orf03824
Control
Ethanol
Butanol
Hexane
Salt
N−starvation

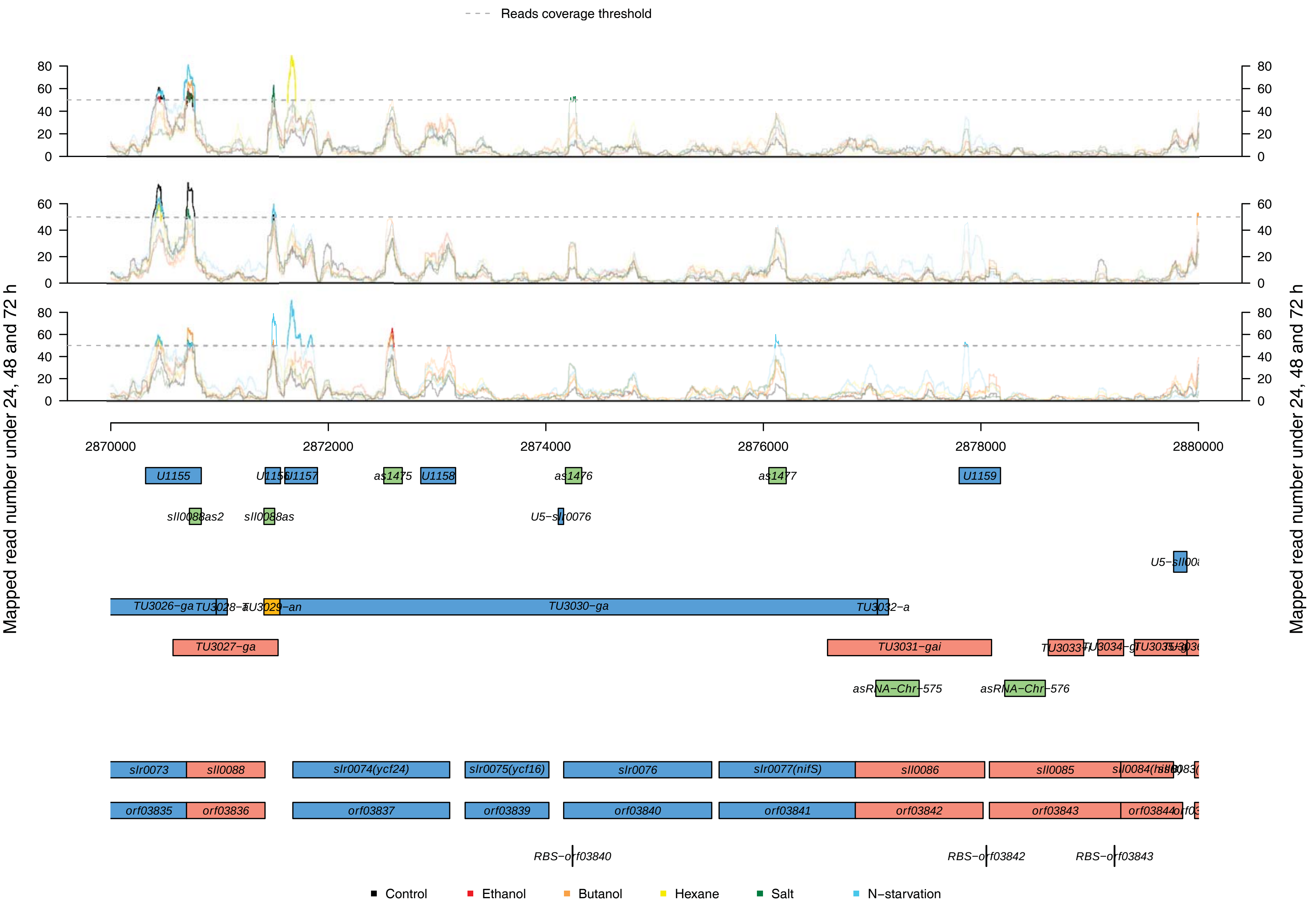

Reads coverage threshold
Mapped read number under 24, 48 and 72 h
2870000
2872000
2874000
2876000
2878000
2880000
U1155
U1156
U1157
as1475
U1158
as1476
as1477
U1159
sll0088as2
sll0088as
U5−slr0076
TU3026−ga
TU3028−a
TU3029−an
TU3030−ga
TU3032−a
TU3027−ga
TU3031−gai
asRNA−Chr−575
asRNA−Chr−576
slr0073
sll0088
slr0074(ycf24)
slr0075(ycf16)
slr0076
slr0077(nifS)
sll0086
sll0085
orf03835
orf03836
orf03837
orf03839
orf03840
orf03841
orf03842
orf03843
orf03844
RBS−orf03840
RBS−orf03842
RBS−orf03843
Control
Ethanol
Butanol
Hexane
Salt
N−starvation

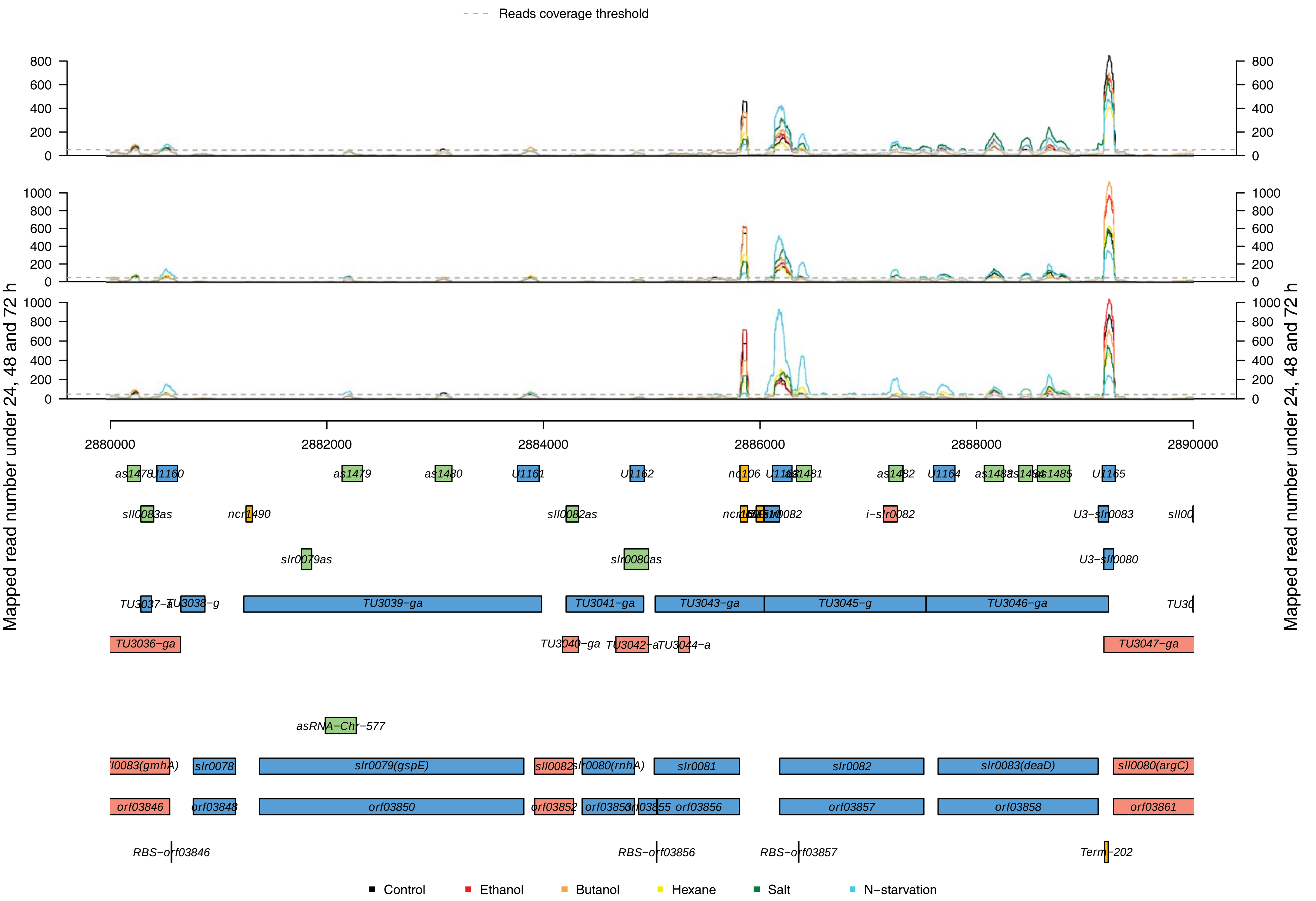

Reads coverage threshold
Mapped read number under 24, 48 and 72 h
2880000
2882000
2884000
2886000
2888000
2890000
TU3039−ga
TU3041−ga
TU3043−ga
TU3045−g
TU3046−ga
TU3036−ga
TU3047−ga
asRNA−Chr−577
slr0079(gspE)
slr0081
slr0082
slr0083(deaD)
sll0080(argC)
orf03846
orf03848
orf03850
orf03856
orf03857
orf03858
orf03861
RBS−orf03846
RBS−orf03856
RBS−orf03857
Control
Ethanol
Butanol
Hexane
Salt
N−starvation

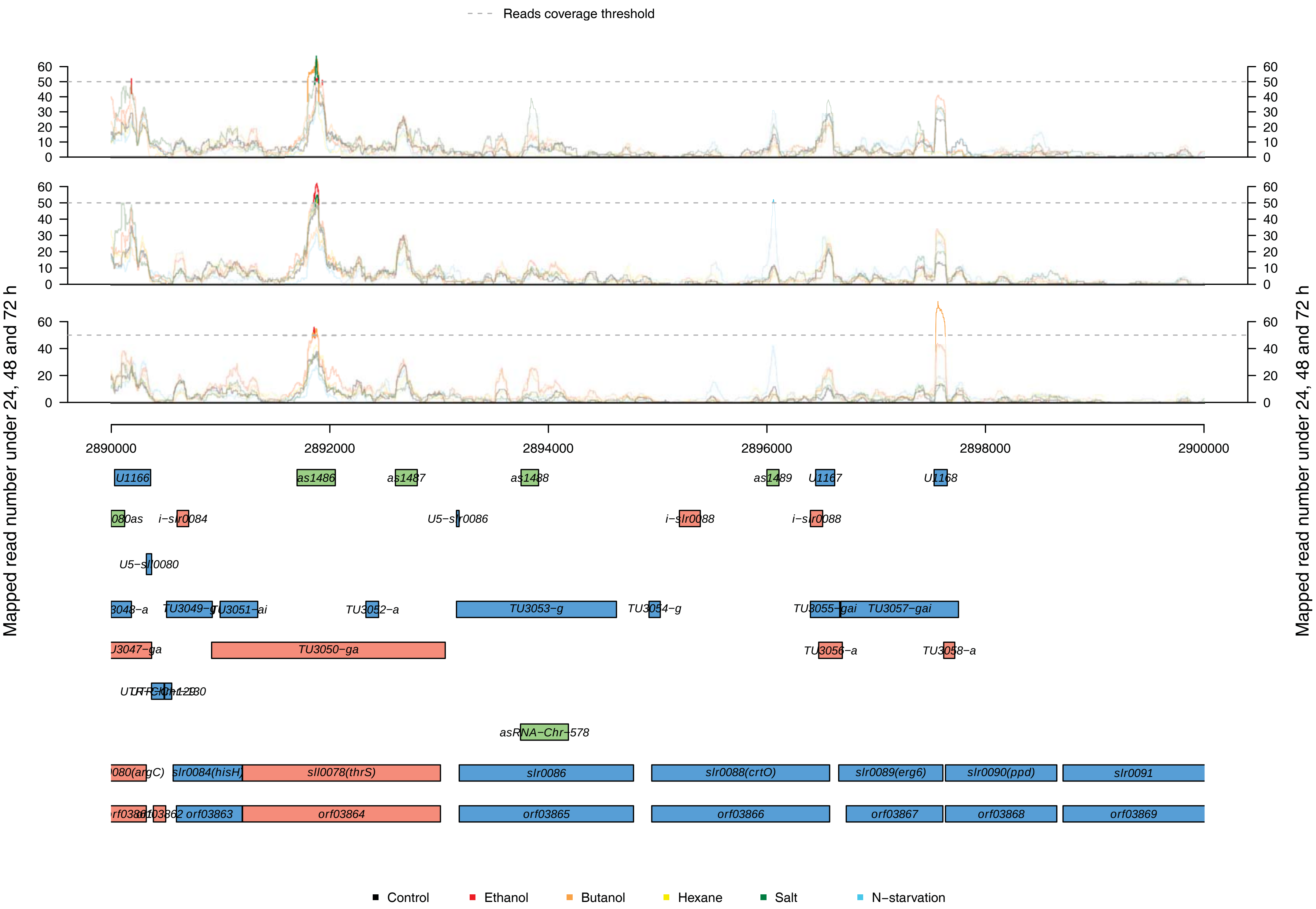
Reads coverage threshold
Mapped read number under 24, 48 and 72 h
2890000
2892000
2894000
2896000
2898000
2900000
U1166
as1486
as1487
as1488
as1489
U1167
U1168
i−slr0084
U5−slr0086
i−slr0088
U5−sll0080
TU3049
TU3051
TU3052−a
TU3053−g
TU3054−g
TU3055−gai
TU3057−gai
TU3050−ga
TU3056−a
TU3058−a
asRNA−Chr−578
slr0084(hisH)
sll0078(thrS)
slr0086
slr0088(crtO)
slr0089(erg6)
slr0090(ppd)
slr0091
orf03863
orf03864
orf03865
orf03866
orf03867
orf03868
orf03869
Control
Ethanol
Butanol
Hexane
Salt
N−starvation

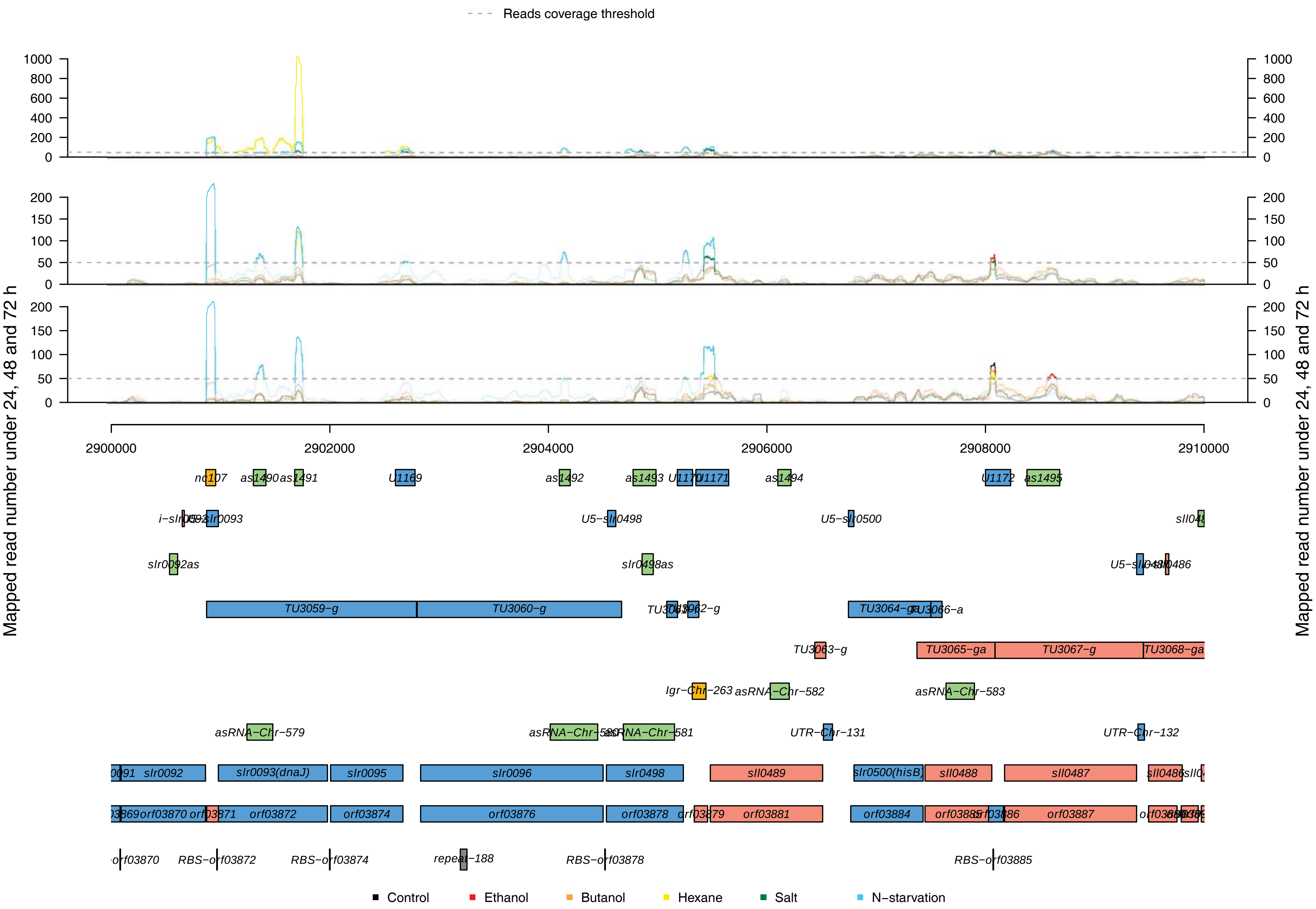
Reads coverage threshold
Mapped read number under 24, 48 and 72 h
1000
800
600
400
200
0
150
100
50
2900000
2902000
2904000
2906000
2908000
2910000
as1490
as1491
U1169
as1492
as1493
U1171
as1494
as1495
U5−slr0498
U5−slr0500
slr0092as
slr0498as
TU3059−g
TU3060−g
TU3063−g
TU3065−ga
TU3067−g
TU3068−ga
asRNA−Chr−582
asRNA−Chr−583
asRNA−Chr−579
asRNA−Chr−581
UTR−Chr−131
UTR−Chr−132
slr0092
slr0093(dnaJ)
slr0095
slr0096
slr0498
sll0489
slr0500(hisB)
sll0488
sll0487
orf03870
orf03872
orf03874
orf03876
orf03878
orf03881
orf03884
orf03887
RBS−orf03872
RBS−orf03874
repeat−188
RBS−orf03878
RBS−orf03885
Control
Ethanol
Butanol
Hexane
Salt
N−starvation

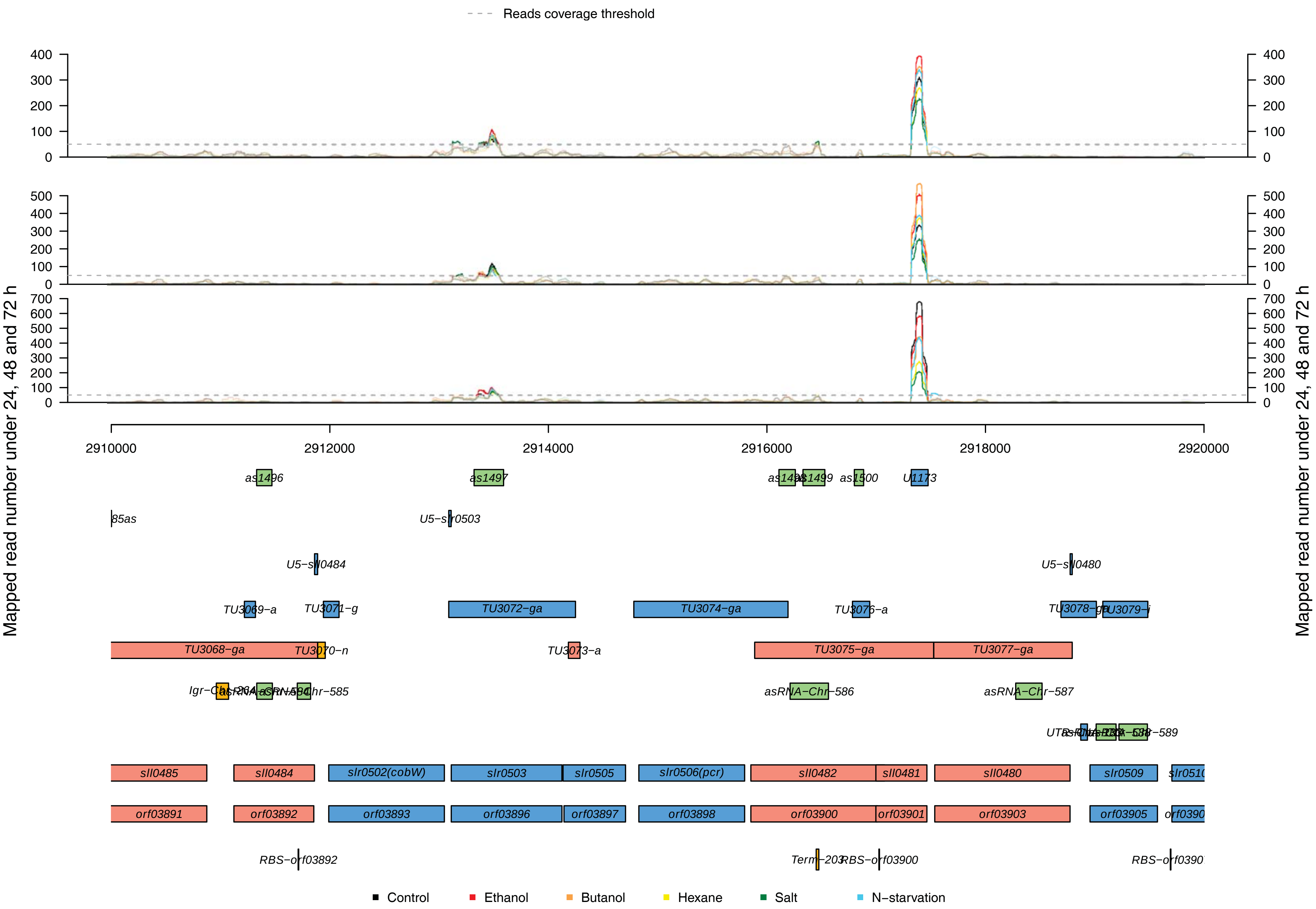
Reads coverage threshold
Mapped read number under 24, 48 and 72 h
2910000
2912000
2914000
2916000
2918000
2920000
as1496
as1497
as1499
as1500
U1173
U5−slr0503
U5−sll0484
U5−sll0480
TU3069−a
TU3071−g
TU3072−ga
TU3074−ga
TU3076−a
TU3068−ga
TU3070−n
TU3073−a
TU3075−ga
TU3077−ga
asRNA−Chr−585
asRNA−Chr−586
asRNA−Chr−587
sll0485
sll0484
slr0502(cobW)
slr0503
slr0505
slr0506(pcr)
sll0482
sll0481
sll0480
slr0509
orf03891
orf03892
orf03893
orf03896
orf03897
orf03898
orf03900
orf03901
orf03903
orf03905
RBS−orf03892
RBS−orf03900
Control
Ethanol
Butanol
Hexane
Salt
N−starvation

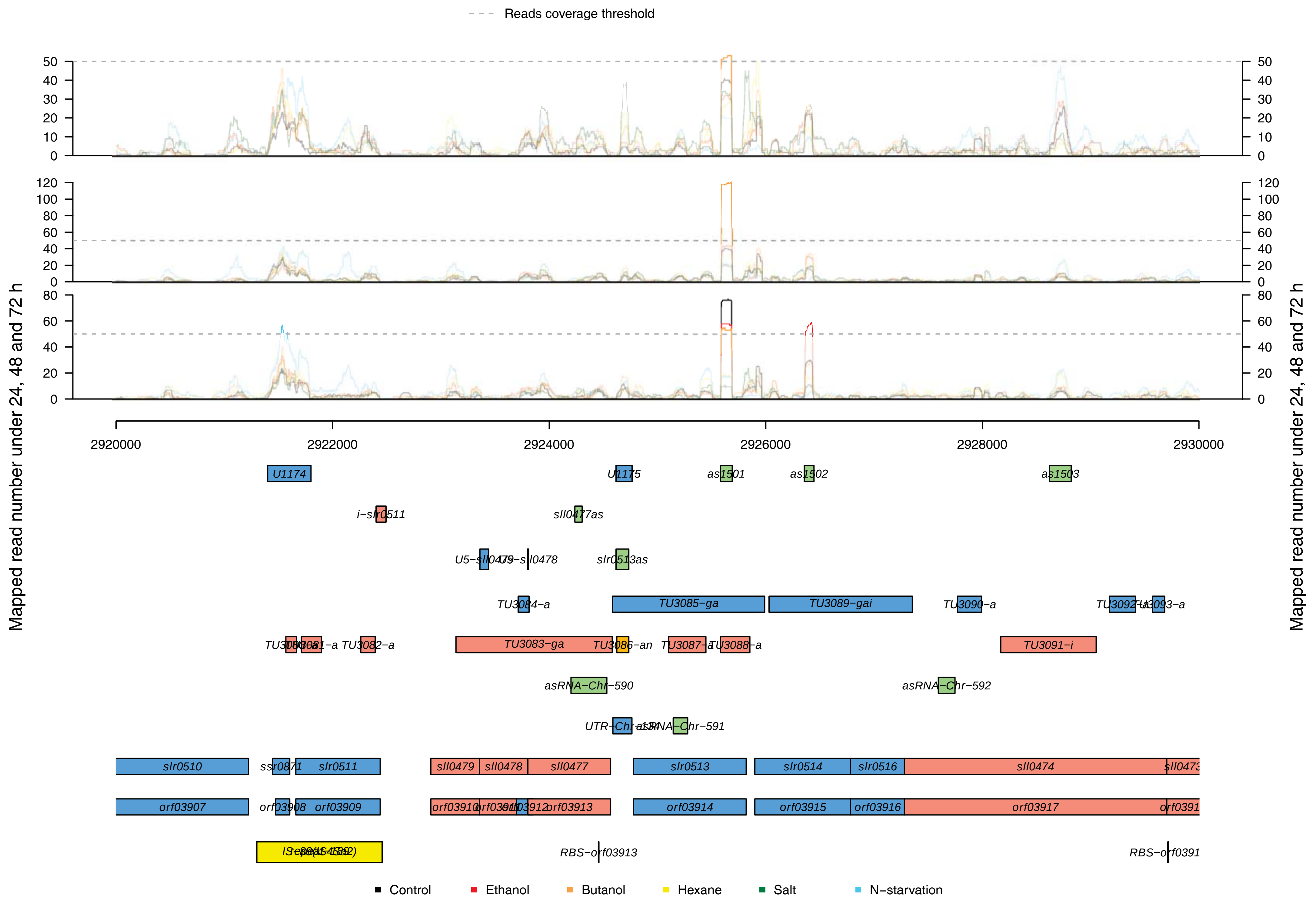

Reads coverage threshold
Mapped read number under 24, 48 and 72 h
2920000
2922000
2924000
2926000
2928000
2930000
U1174
U1175
as1501
as1502
as1503
i−slr0511
sll0477as
slr0513as
TU3084−a
TU3085−ga
TU3089−gai
TU3090−a
TU3083−ga
TU3082−a
TU3087−a
TU3088−a
TU3091−i
asRNA−Chr−590
asRNA−Chr−592
asRNA−Chr−591
slr0510
slr0511
sll0479
sll0478
sll0477
slr0513
slr0514
slr0516
sll0474
orf03907
orf03909
orf03913
orf03914
orf03915
orf03916
orf03917
RBS−orf03913
Control
Ethanol
Butanol
Hexane
Salt
N−starvation

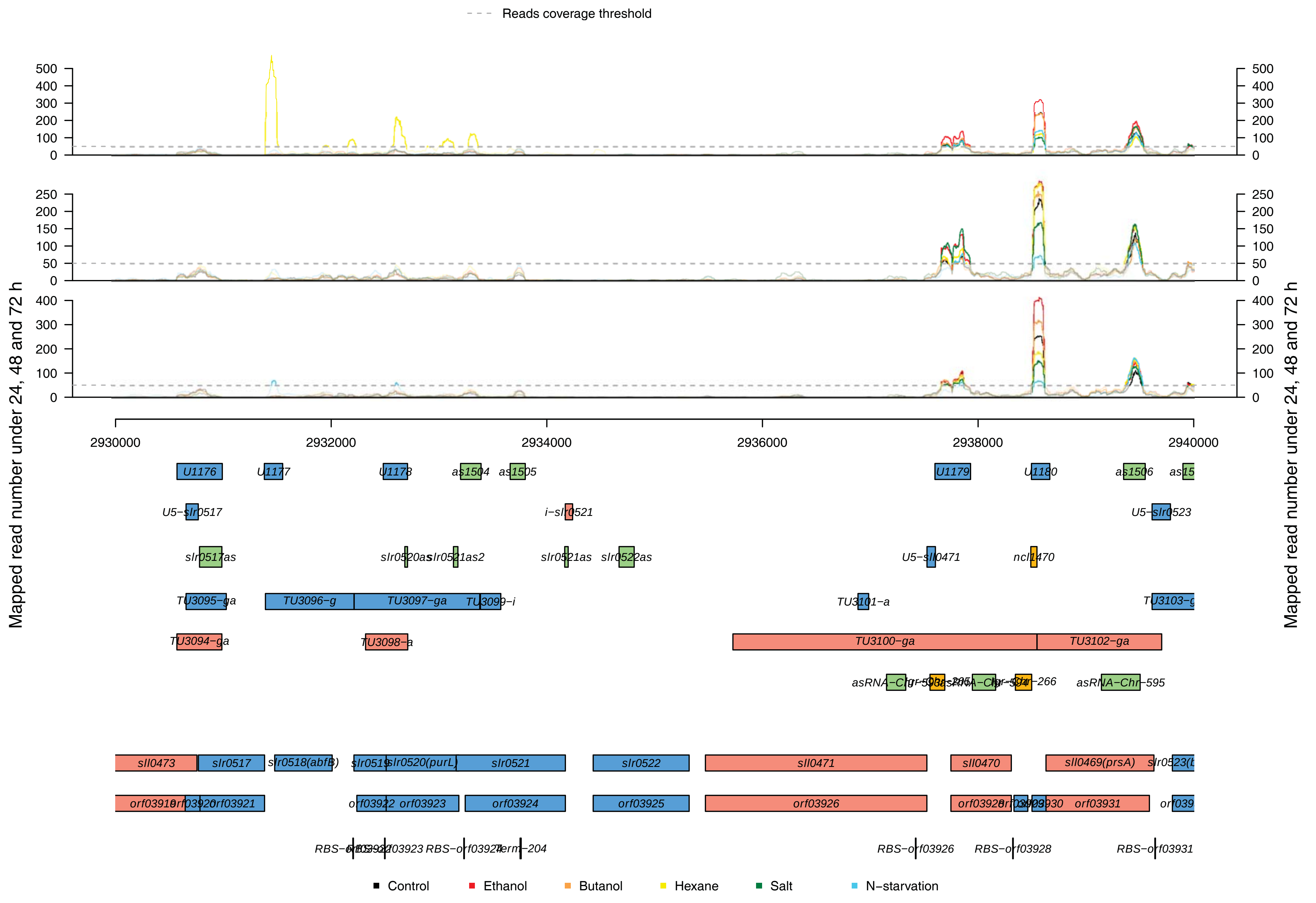

Reads coverage threshold
Mapped read number under 24, 48 and 72 h
2930000
2932000
2934000
2936000
2938000
2940000
U1176
U1177
U1178
as1504
as1505
U1179
U1180
as1506
U5−slr0517
i−slr0521
U5−slr0523
slr0517as
slr0520as
slr0521as2
slr0521as
slr0522as
U5−sll0471
ncl1470
TU3095−ga
TU3096−g
TU3097−ga
TU3099−i
TU3101−a
TU3094−ga
TU3098−a
TU3100−ga
TU3102−ga
asRNA−Chr−595
sll0473
slr0517
slr0518(abfB)
slr0519
slr0520(purL)
slr0521
slr0522
sll0471
sll0470
sll0469(prsA)
orf03921
orf03922
orf03923
orf03924
orf03925
orf03926
orf03930
orf03931
RBS−orf03923
RBS−orf03924
term−204
RBS−orf03926
RBS−orf03928
RBS−orf03931
Control
Ethanol
Butanol
Hexane
Salt
N−starvation

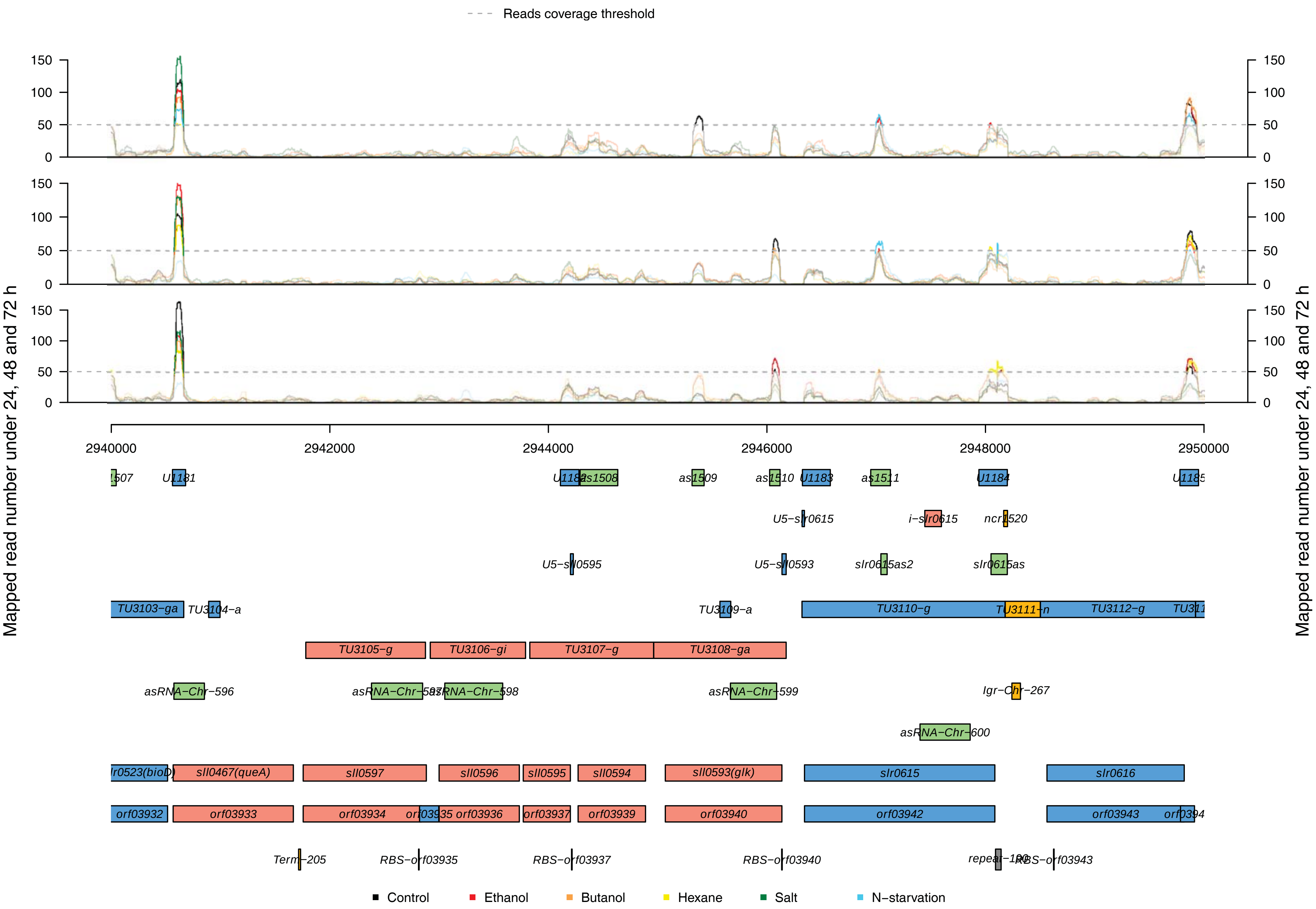

Reads coverage threshold
Mapped read number under 24, 48 and 72 h
150
100
50
0
2940000
2942000
2944000
2946000
2948000
2950000
U1181
as1508
as1509
as1510
U1183
as1511
U1184
U5−slr0615
i−slr0615
ncr1520
U5−sll0595
U5−sll0593
slr0615as2
slr0615as
TU3103−ga
TU3104−a
TU3109−a
TU3110−g
TU3112−g
TU3105−g
TU3106−gi
TU3107−g
TU3108−ga
asRNA−Chr−596
asRNA−Chr−599
Igr−Chr−267
asRNA−Chr−600
sll0467(queA)
sll0597
sll0596
sll0595
sll0594
sll0593(glk)
slr0615
slr0616
orf03932
orf03933
orf03934
orf03936
orf03937
orf03939
orf03940
orf03942
orf03943
Term−205
RBS−orf03935
RBS−orf03937
RBS−orf03940
RBS−orf03943
Control
Ethanol
Butanol
Hexane
Salt
N−starvation

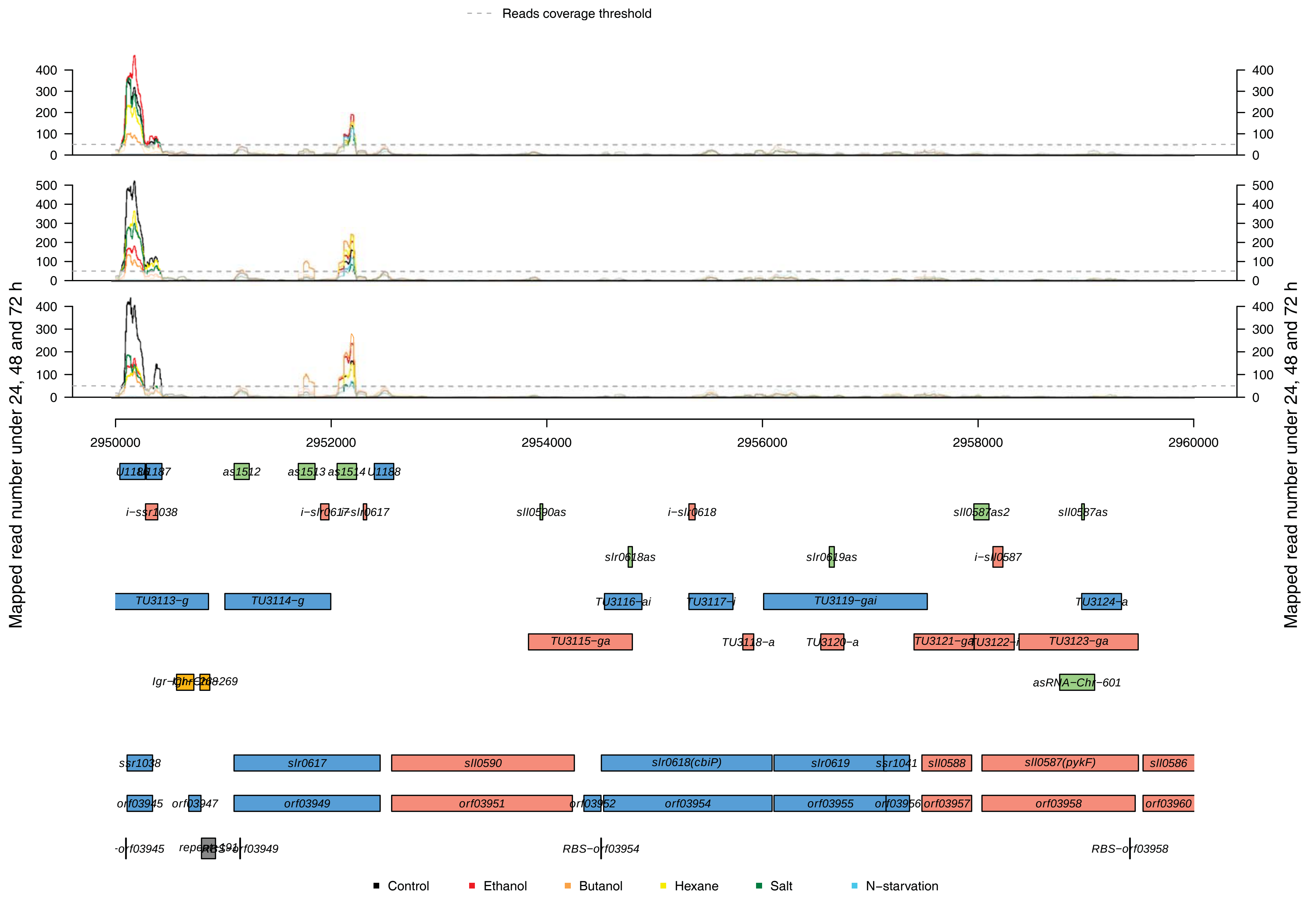
Reads coverage threshold
Mapped read number under 24, 48 and 72 h
2950000
2952000
2954000
2956000
2958000
2960000
TU3113–g
TU3114–g
TU3115–ga
TU3119–gai
TU3123–ga
slr0617
sll0590
slr0618(cbiP)
slr0619
sll0588
sll0587(pykF)
sll0586
orf03949
orf03951
orf03954
orf03955
orf03957
orf03958
orf03960
Control
Ethanol
Butanol
Hexane
Salt
N–starvation

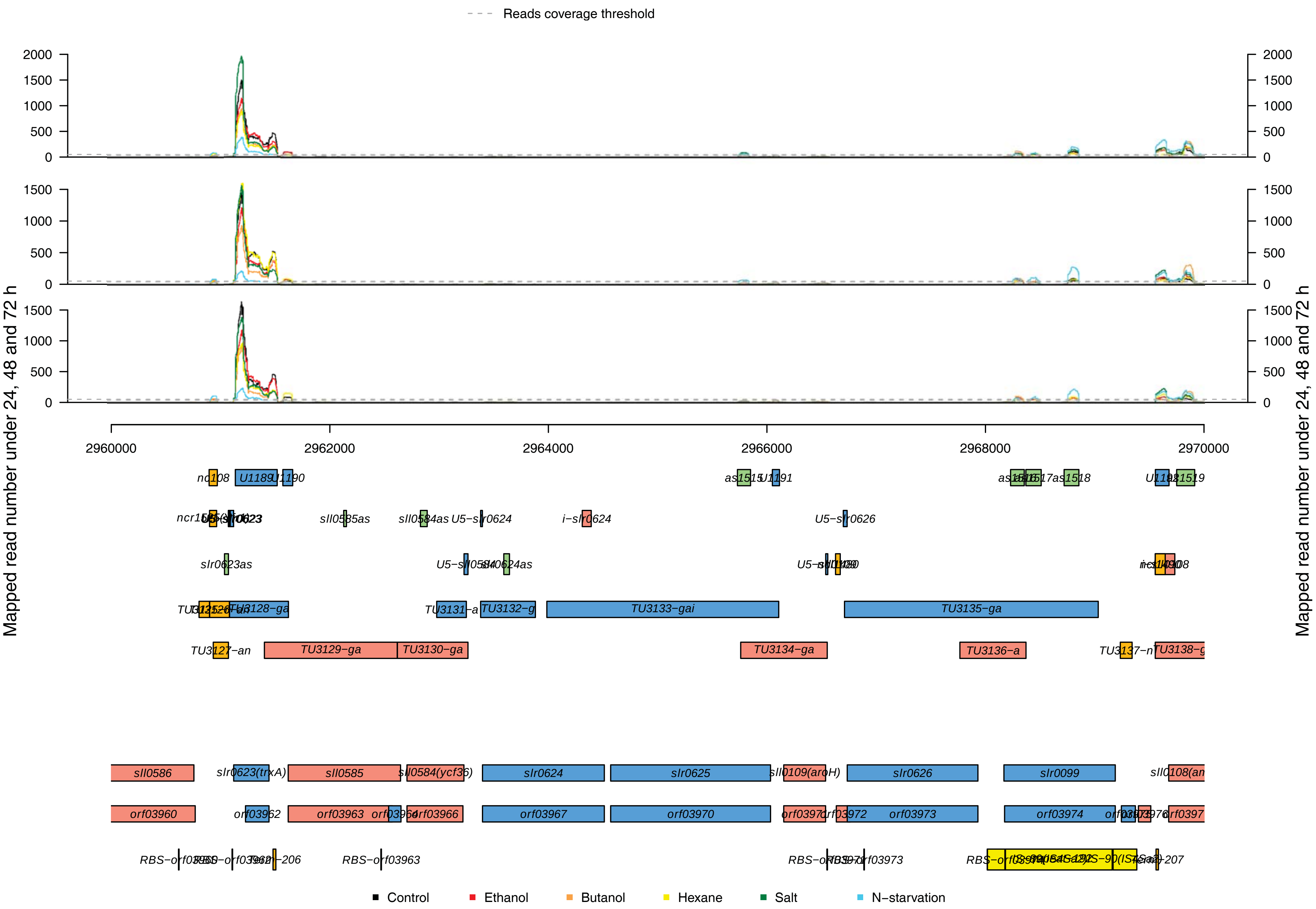

Reads coverage threshold
Mapped read number under 24, 48 and 72 h
2960000
2962000
2964000
2966000
2968000
2970000
TU3129−ga
TU3130−ga
TU3133−gai
TU3135−ga
TU3134−ga
TU3136−a
TU3128−ga
TU3132−g
U1189
sll0586
sll0585
slr0623(trxA)
sll0584(ycf36)
slr0624
slr0625
slr0626
slr0099
orf03960
orf03962
orf03963
orf03966
orf03967
orf03970
orf03973
orf03974
RBS−orf03963
slr0623as
sll0585as
i−slr0624
U5−slr0624
U5−slr0626
Control
Ethanol
Butanol
Hexane
Salt
N−starvation

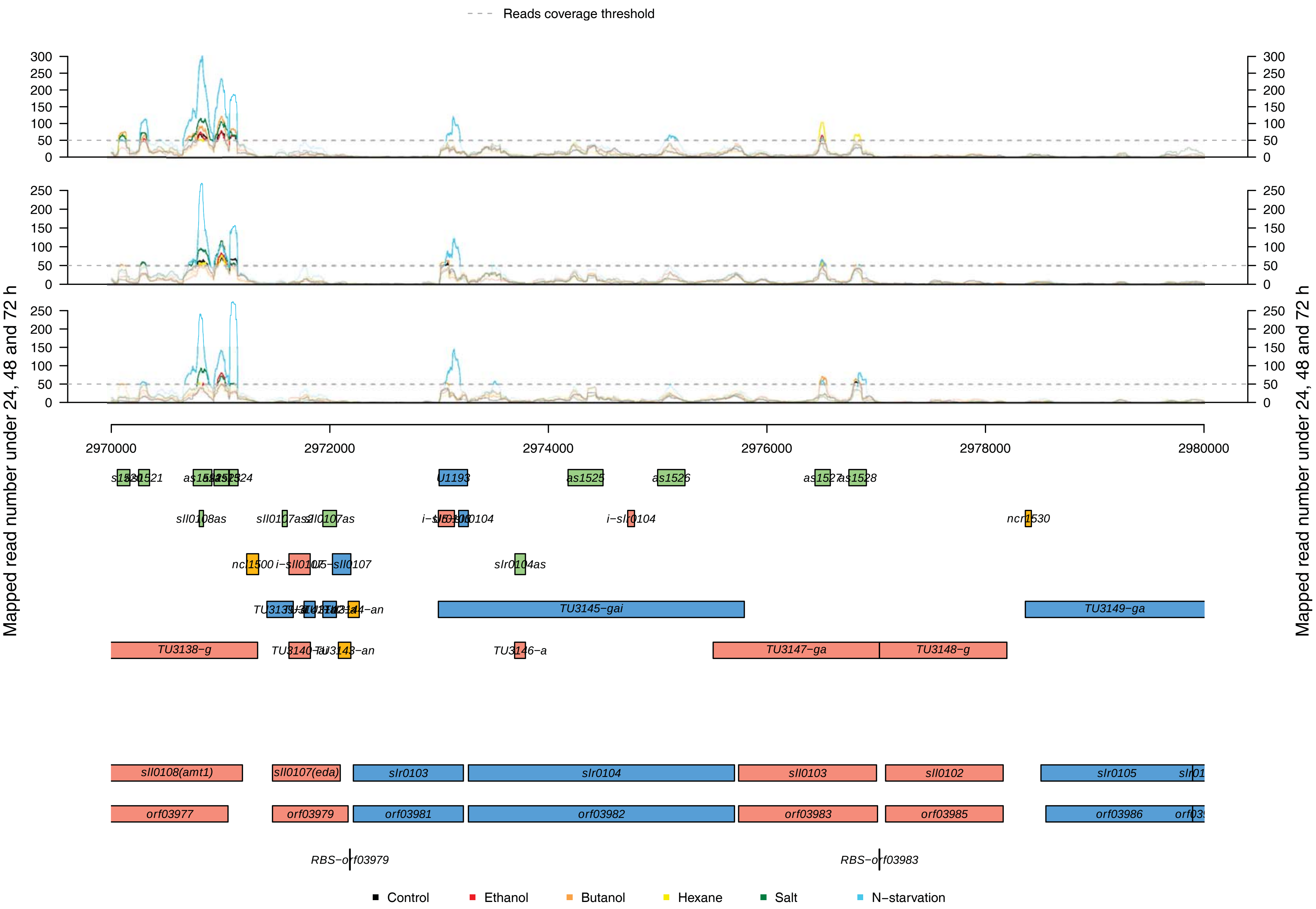

Reads coverage threshold
Mapped read number under 24, 48 and 72 h
2970000
2972000
2974000
2976000
2978000
2980000
as1525
as1526
ncr1530
slr0104as
TU3145−gai
TU3149−ga
TU3138−g
TU3146−a
TU3147−ga
TU3148−g
sll0108(amt1)
sll0107(eda)
slr0103
slr0104
sll0103
sll0102
slr0105
orf03977
orf03979
orf03981
orf03982
orf03983
orf03985
orf03986
RBS−orf03979
RBS−orf03983
Control
Ethanol
Butanol
Hexane
Salt
N−starvation

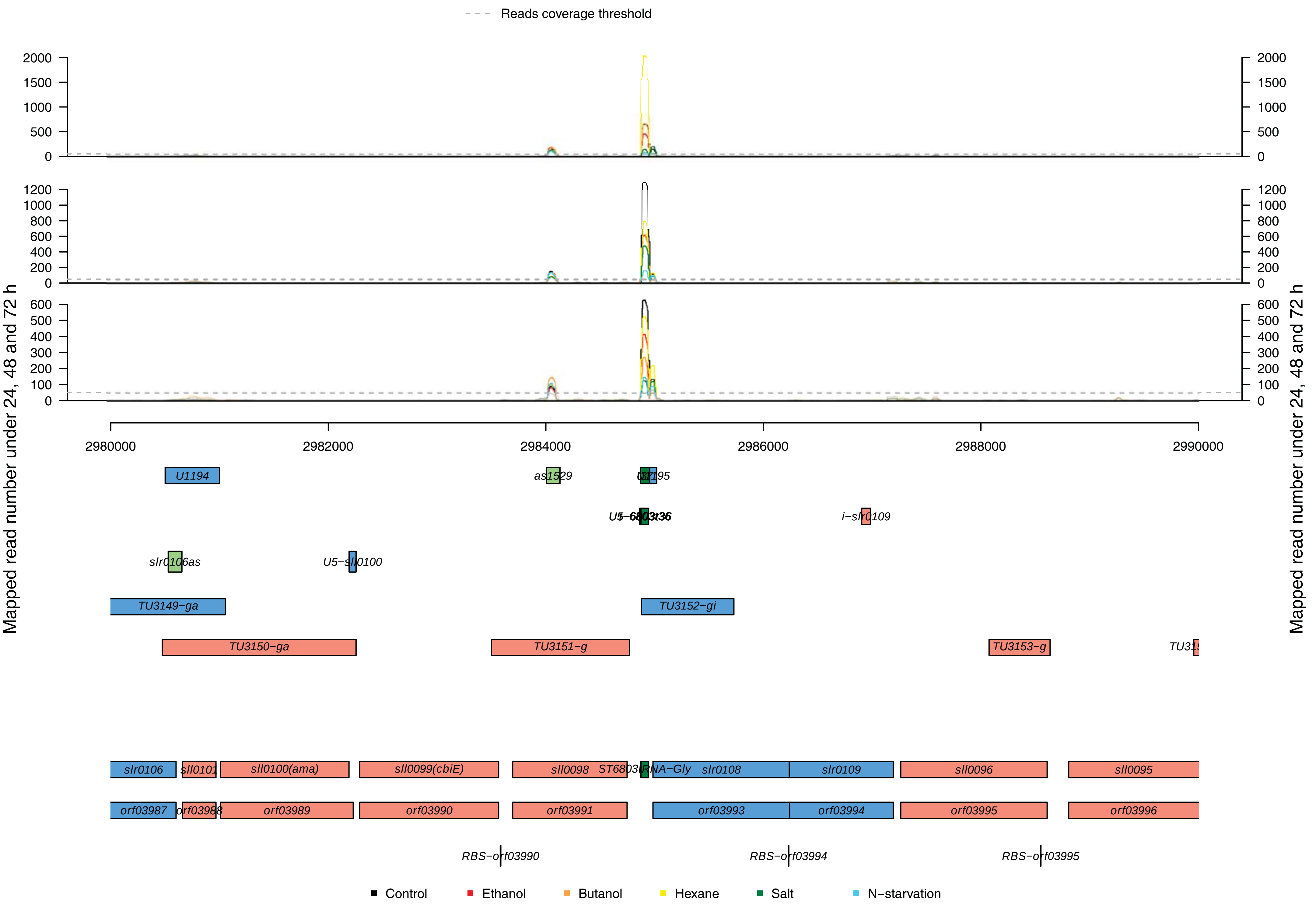

Reads coverage threshold
Mapped read number under 24, 48 and 72 h
2980000
2982000
2984000
2986000
2988000
2990000
U1194
as1529
slr0106as
U5−sll0100
i−slr0109
TU3149−ga
TU3152−gi
TU3150−ga
TU3151−g
TU3153−g
slr0106
sll0101
sll0100(ama)
sll0099(cbiE)
sll0098
tRNA−Gly
slr0108
slr0109
sll0096
sll0095
orf03987
orf03988
orf03989
orf03990
orf03991
orf03993
orf03994
orf03995
orf03996
RBS−orf03990
RBS−orf03994
RBS−orf03995
Control
Ethanol
Butanol
Hexane
Salt
N−starvation

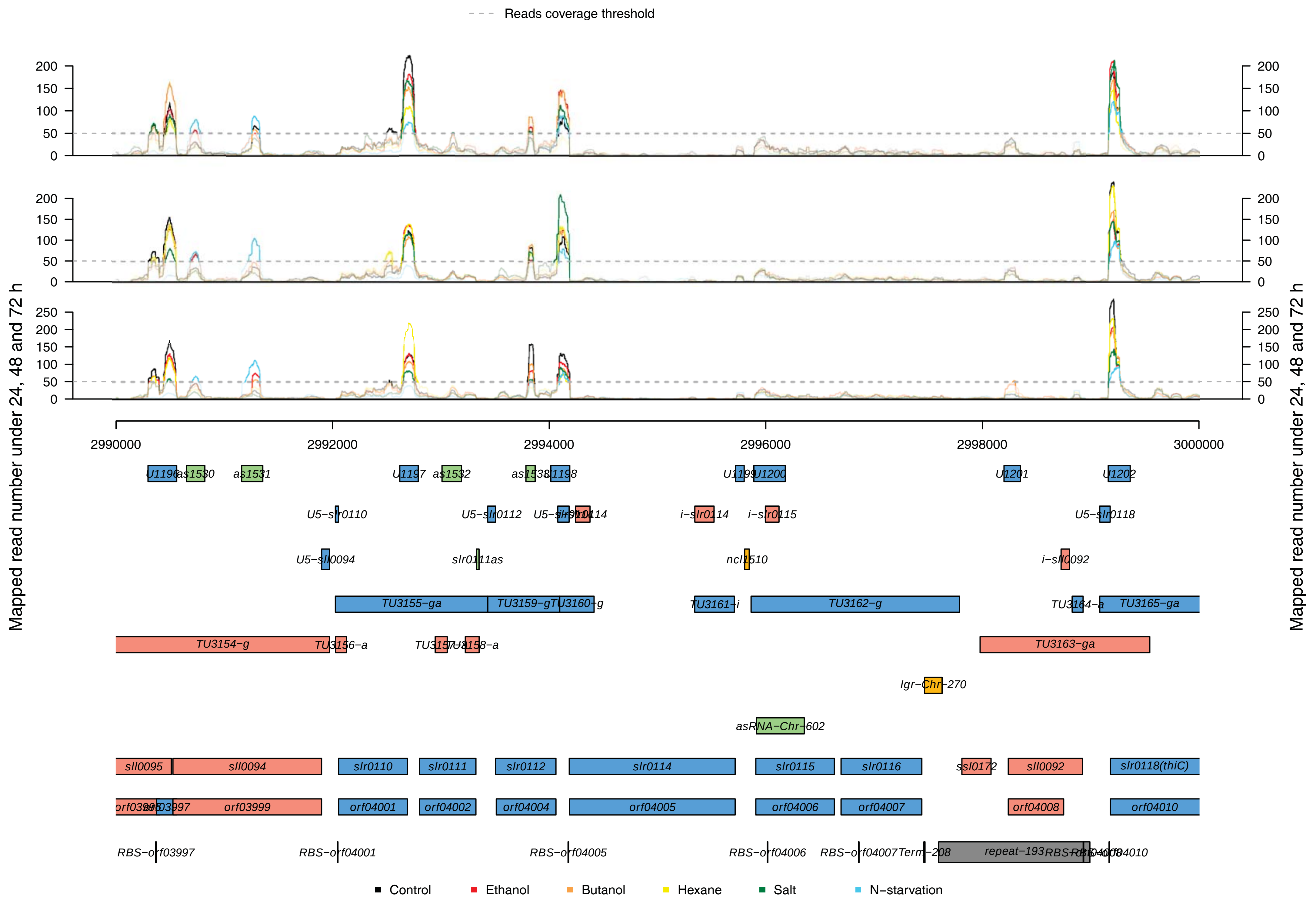

Reads coverage threshold
Mapped read number under 24, 48 and 72 h
2990000
2992000
2994000
2996000
2998000
3000000
TU3154−g
TU3155−ga
TU3162−g
TU3163−ga
TU3165−ga
sll0095
sll0094
slr0110
slr0111
slr0112
slr0114
slr0115
slr0116
sll0092
slr0118(thiC)
orf03999
orf04001
orf04002
orf04004
orf04005
orf04006
orf04007
orf04008
orf04010
Control
Ethanol
Butanol
Hexane
Salt
N−starvation

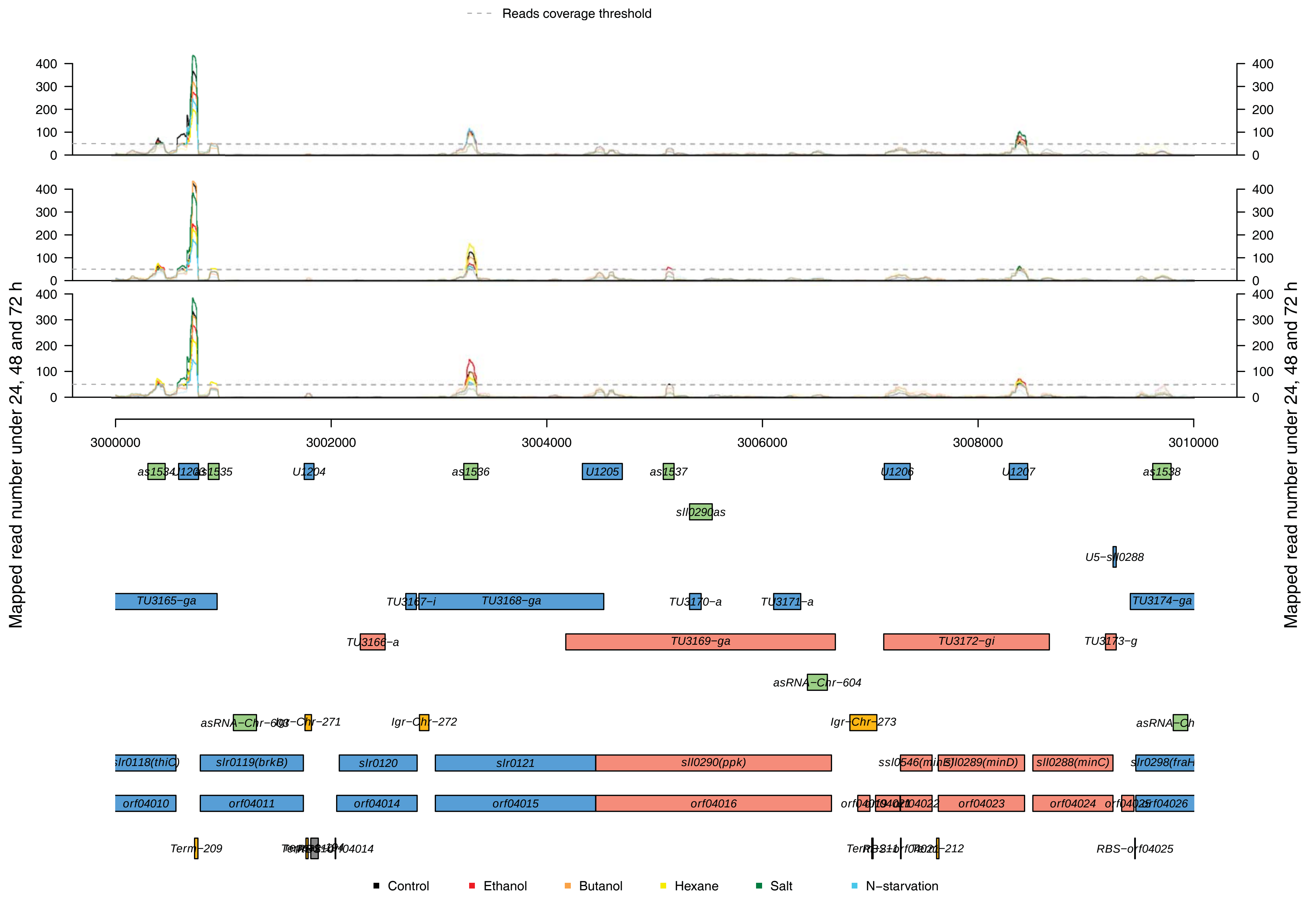
Reads coverage threshold
Mapped read number under 24, 48 and 72 h
3000000
3002000
3004000
3006000
3008000
3010000
TU3165−ga
TU3166−a
TU3167−i
TU3168−ga
TU3169−ga
TU3170−a
TU3171−a
TU3172−gi
TU3173−g
TU3174−ga
sll0290as
U5−sll0288
asRNA−Chr−604
Igr−Chr−271
Igr−Chr−272
Igr−Chr−273
slr0118(thiC)
slr0119(brkB)
slr0120
slr0121
sll0290(ppk)
sll0289(minD)
sll0288(minC)
orf04010
orf04011
orf04014
orf04015
orf04016
orf04023
orf04024
Term−209
Term−212
RBS−orf04025
Control
Ethanol
Butanol
Hexane
Salt
N−starvation

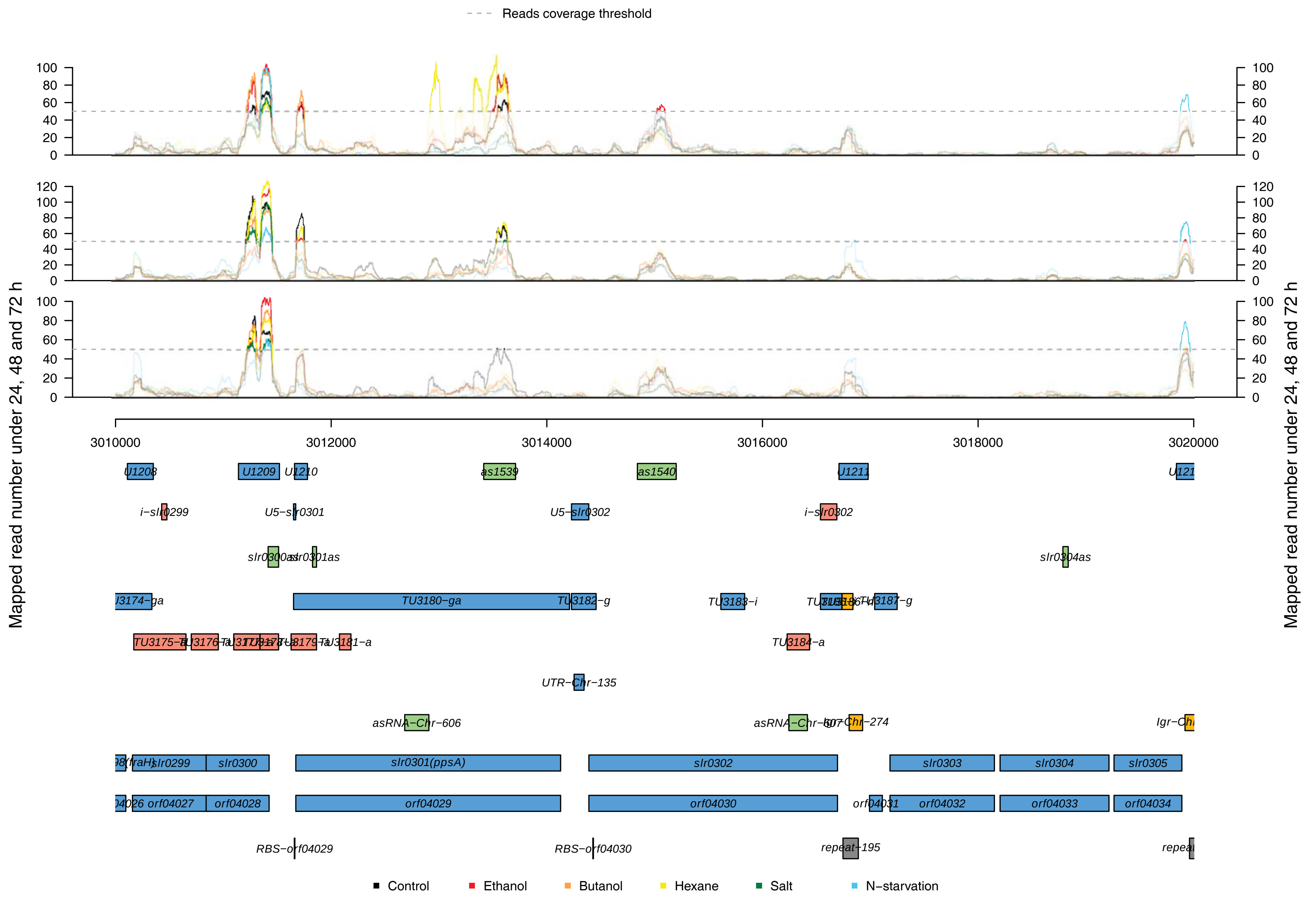

Reads coverage threshold
Mapped read number under 24, 48 and 72 h
3010000
3012000
3014000
3016000
3018000
3020000
U1208
U1209
U1210
as1539
as1540
U1211
i−slr0299
U5−slr0301
U5−slr0302
i−slr0302
slr0300as
slr0301as
slr0304as
TU3180−ga
TU3182−g
TU3183−i
TU3187−g
TU3184−a
TU3181−a
UTR−Chr−135
asRNA−Chr−606
slr0299
slr0300
slr0301(ppsA)
slr0302
slr0303
slr0304
slr0305
orf04027
orf04028
orf04029
orf04030
orf04031
orf04032
orf04033
orf04034
RBS−orf04029
RBS−orf04030
repeat−195
Control
Ethanol
Butanol
Hexane
Salt
N−starvation

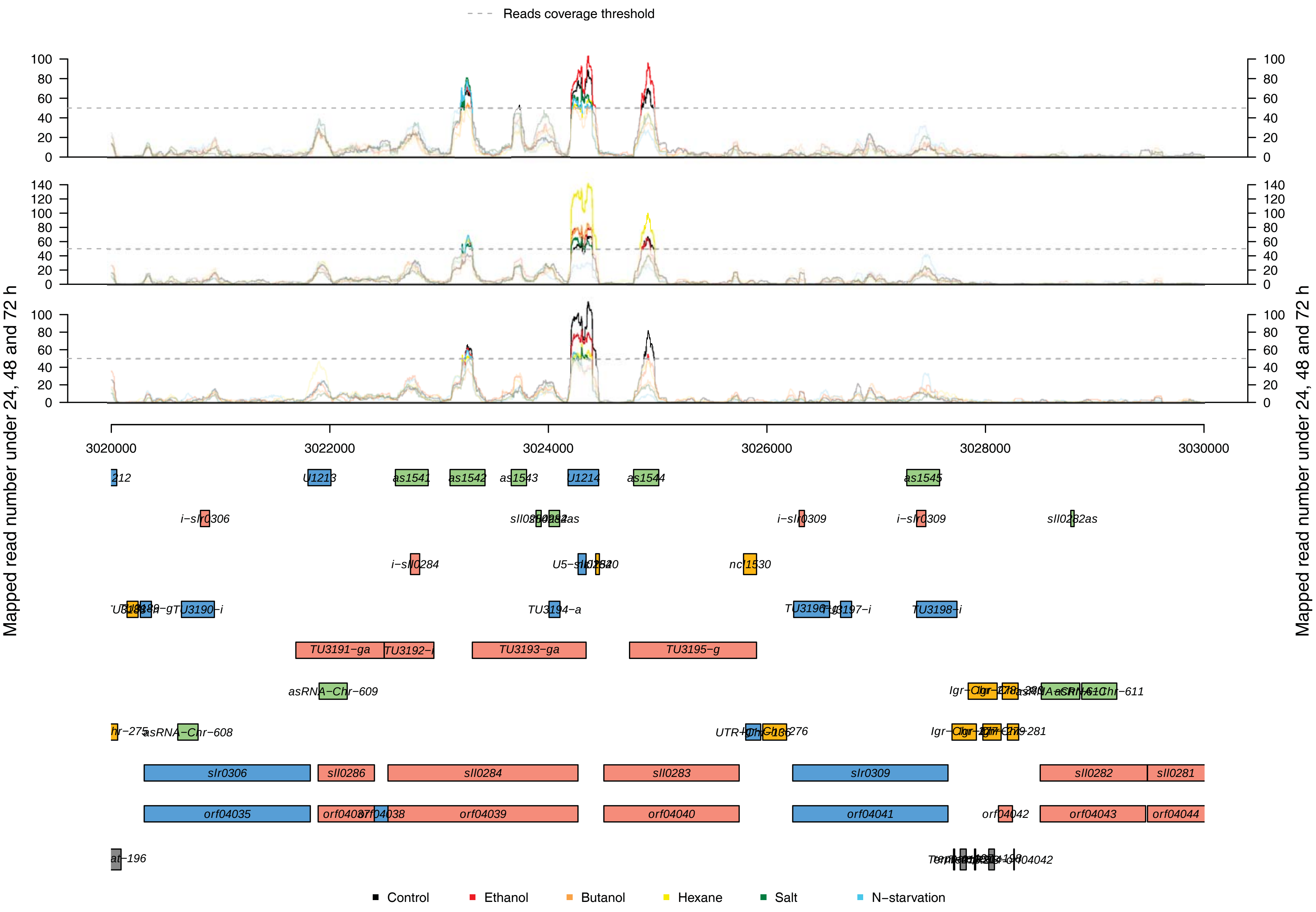
Reads coverage threshold
Mapped read number under 24, 48 and 72 h
3020000
3022000
3024000
3026000
3028000
3030000
as1541
as1542
as1545
TU3191−ga
TU3192−i
TU3193−ga
TU3195−g
TU3194−a
TU3198−i
asRNA−Chr−609
asRNA−Chr−608
slr0306
sll0286
sll0284
sll0283
slr0309
sll0282
sll0281
orf04035
orf04039
orf04040
orf04041
orf04043
orf04044
Control
Ethanol
Butanol
Hexane
Salt
N−starvation

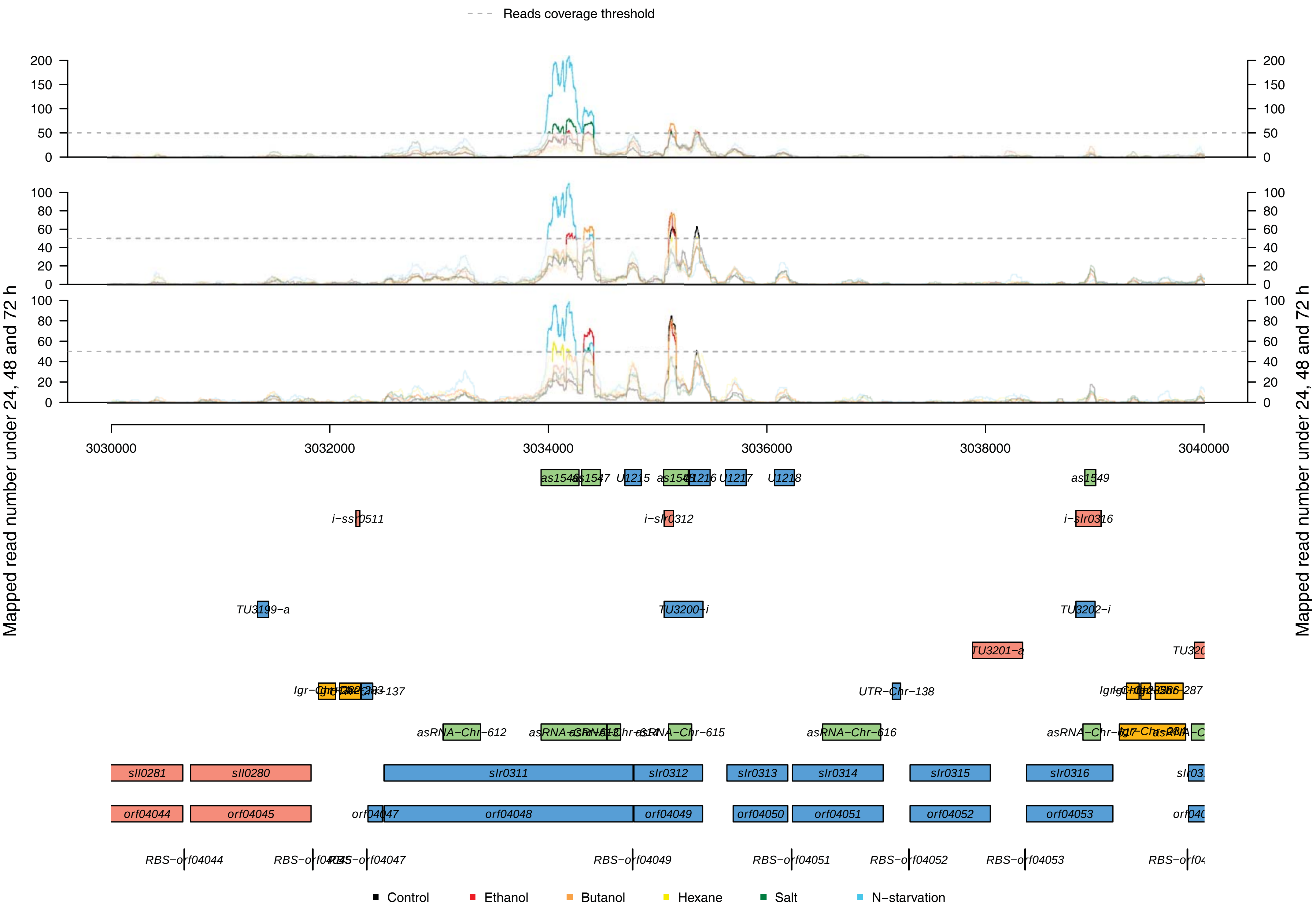

Reads coverage threshold
Mapped read number under 24, 48 and 72 h
3030000
3032000
3034000
3036000
3038000
3040000
as1549
U1215
U1217
U1218
i−sll0511
i−slr0312
i−slr0316
TU3199−a
TU3200−i
TU3202−i
TU3201−a
UTR−Chr−138
asRNA−Chr−612
asRNA−Chr−615
asRNA−Chr−616
sll0281
sll0280
slr0311
slr0312
slr0313
slr0314
slr0315
slr0316
orf04044
orf04045
orf04048
orf04049
orf04050
orf04051
orf04052
orf04053
RBS−orf04044
RBS−orf04049
RBS−orf04051
RBS−orf04052
RBS−orf04053
Control
Ethanol
Butanol
Hexane
Salt
N−starvation

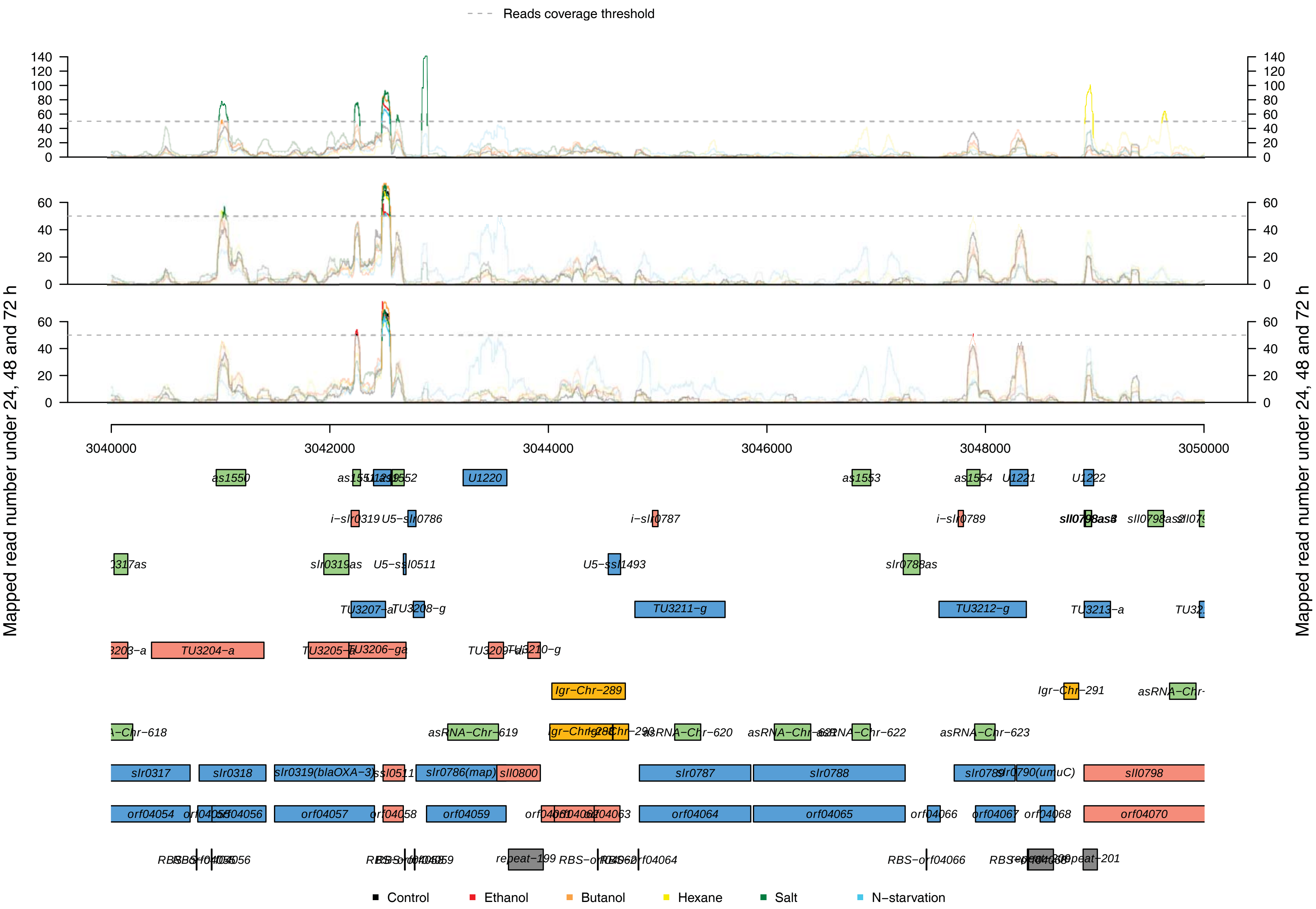

Reads coverage threshold
Mapped read number under 24, 48 and 72 h
3040000
3042000
3044000
3046000
3048000
3050000
as1550
U1220
as1553
as1554
U1221
U1222
i−slr0319
U5−slr0786
i−slr0787
i−slr0789
slr0319as
U5−ssl0511
U5−ssl1493
slr0788as
TU3204−a
TU3211−g
TU3212−g
TU3213−a
Igr−Chr−289
Igr−Chr−291
asRNA−Chr−619
asRNA−Chr−620
asRNA−Chr−623
slr0317
slr0318
slr0319(blaOXA−3)
slr0786(map)
sll0800
slr0787
slr0788
sll0798
orf04054
orf04057
orf04059
orf04064
orf04065
orf04066
orf04070
repeat−199
repeat−201
RBS−orf04066
Control
Ethanol
Butanol
Hexane
Salt
N−starvation

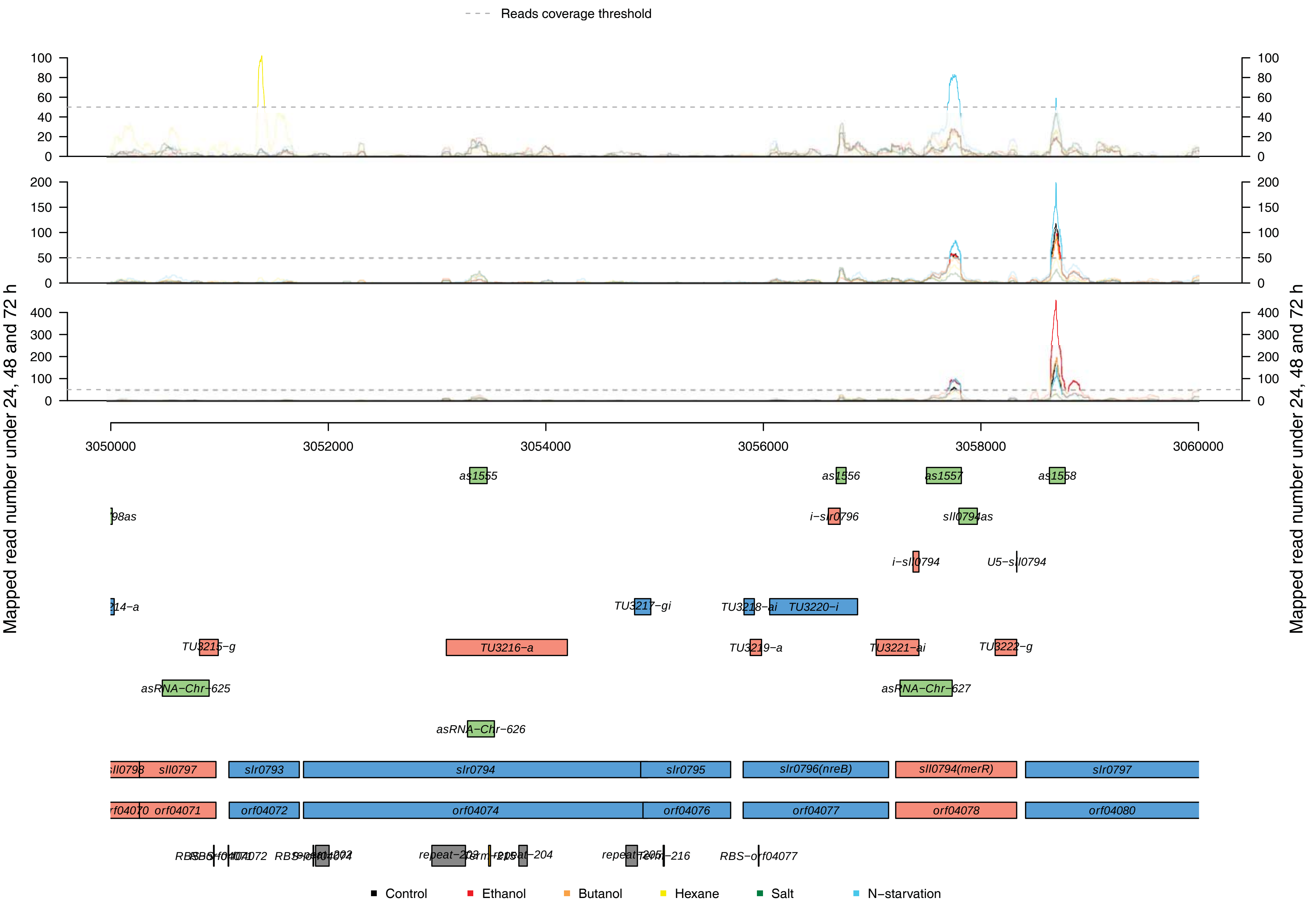

Reads coverage threshold
Mapped read number under 24, 48 and 72 h
3050000
3052000
3054000
3056000
3058000
3060000
as1555
as1556
as1557
as1558
i−slr0796
sll0794as
i−sll0794
U5−sll0794
TU3217−gi
TU3218−ai
TU3220−i
TU3215−g
TU3216−a
TU3219−a
TU3221−ai
TU3222−g
asRNA−Chr−625
asRNA−Chr−627
asRNA−Chr−626
sll0797
slr0793
slr0794
slr0795
slr0796(nreB)
sll0794(merR)
slr0797
orf04071
orf04072
orf04074
orf04076
orf04077
orf04078
orf04080
repeat−204
RBS−orf04077
Control
Ethanol
Butanol
Hexane
Salt
N−starvation

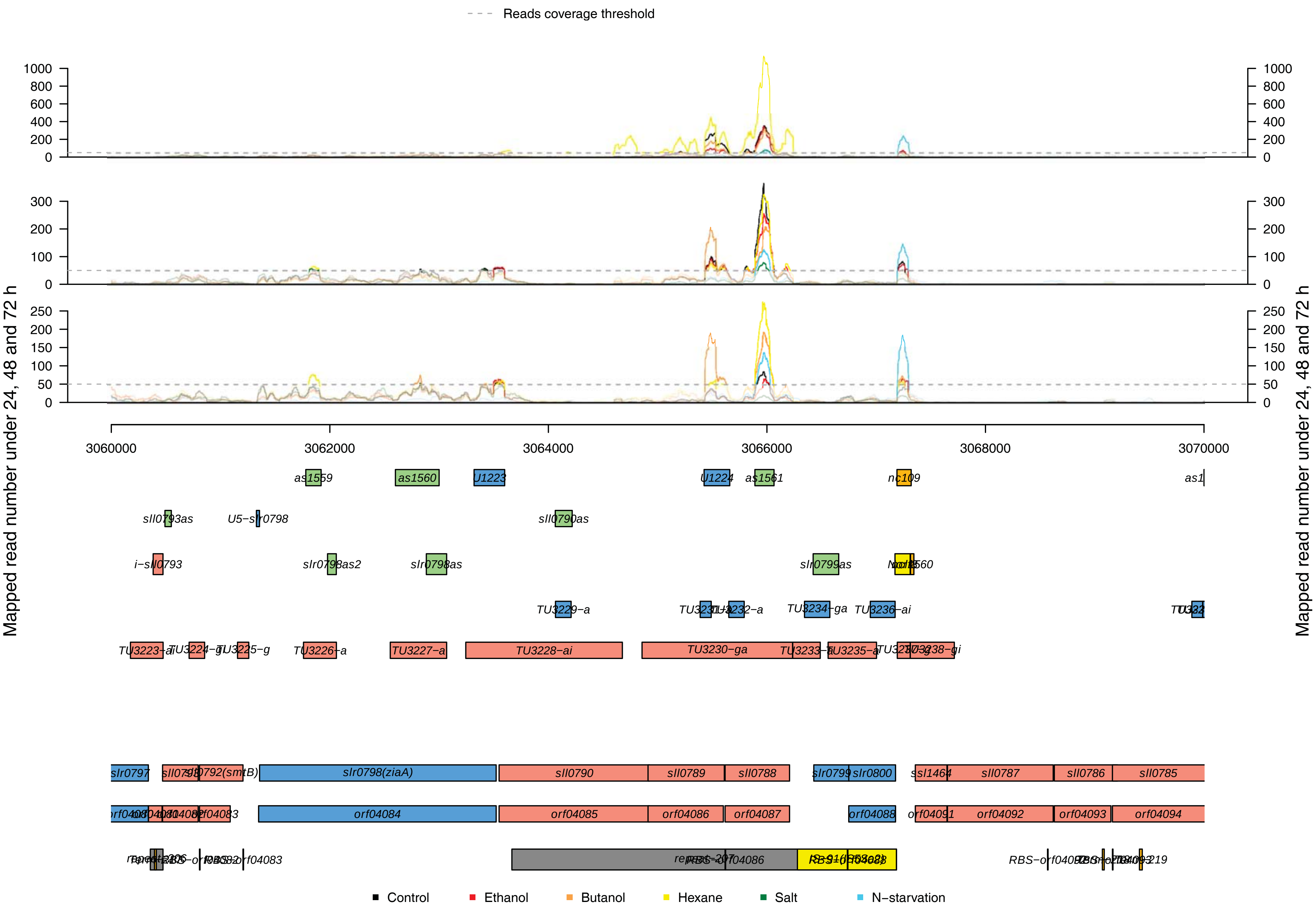

Reads coverage threshold
Mapped read number under 24, 48 and 72 h
1000
800
600
400
200
0
300
200
100
250
150
50
3060000
3062000
3064000
3066000
3068000
3070000
as1559
as1560
as1561
nc109
sll0793as
U5−slr0798
sll0790as
i−sll0793
slr0798as2
slr0798as
slr0799as
TU3229−a
TU3234−ga
TU3236−ai
TU3223−ai
TU3226−a
TU3227−a
TU3228−ai
TU3230−ga
TU3235−a
slr0797
slr0798(ziaA)
sll0790
sll0789
sll0788
slr0799
slr0800
ssl1464
sll0787
sll0786
sll0785
orf04084
orf04085
orf04086
orf04087
orf04088
orf04091
orf04092
orf04093
orf04094
Control
Ethanol
Butanol
Hexane
Salt
N−starvation

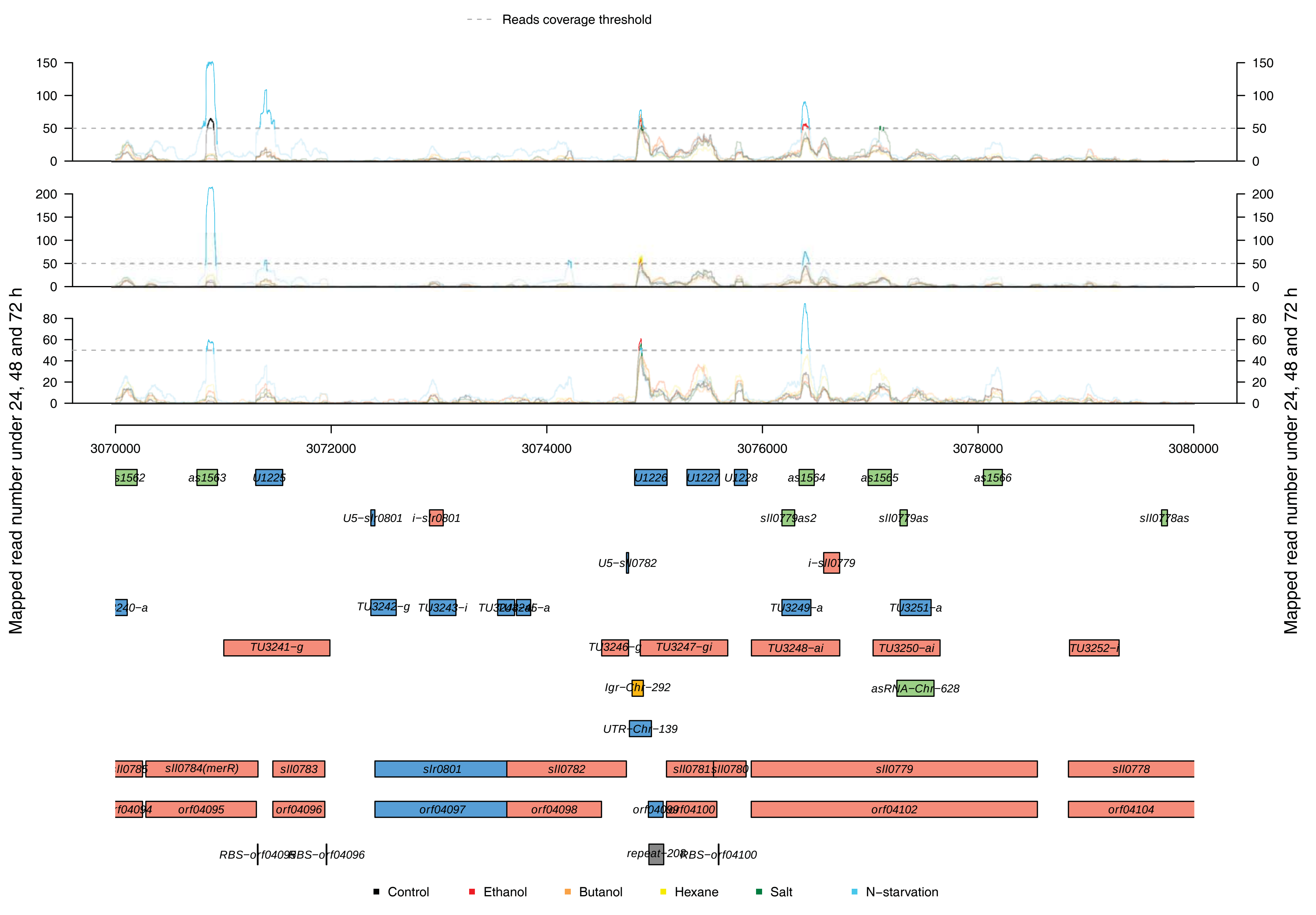
Reads coverage threshold
Mapped read number under 24, 48 and 72 h
3070000
3072000
3074000
3076000
3078000
3080000
as1563
U1225
U1226
U1227
U1228
as1564
as1565
as1566
U5−slr0801
i−slr0801
sll0779as2
sll0779as
sll0778as
U5−sll0782
i−sll0779
TU3242−g
TU3243−i
TU3249−a
TU3251−a
TU3241−g
TU3247−gi
TU3248−ai
TU3250−ai
Igr−Chr−292
asRNA−Chr−628
UTR−Chr−139
sll0784(merR)
sll0783
slr0801
sll0782
sll0779
sll0778
orf04095
orf04096
orf04097
orf04098
orf04100
orf04102
orf04104
RBS−orf04096
RBS−orf04100
Control
Ethanol
Butanol
Hexane
Salt
N−starvation

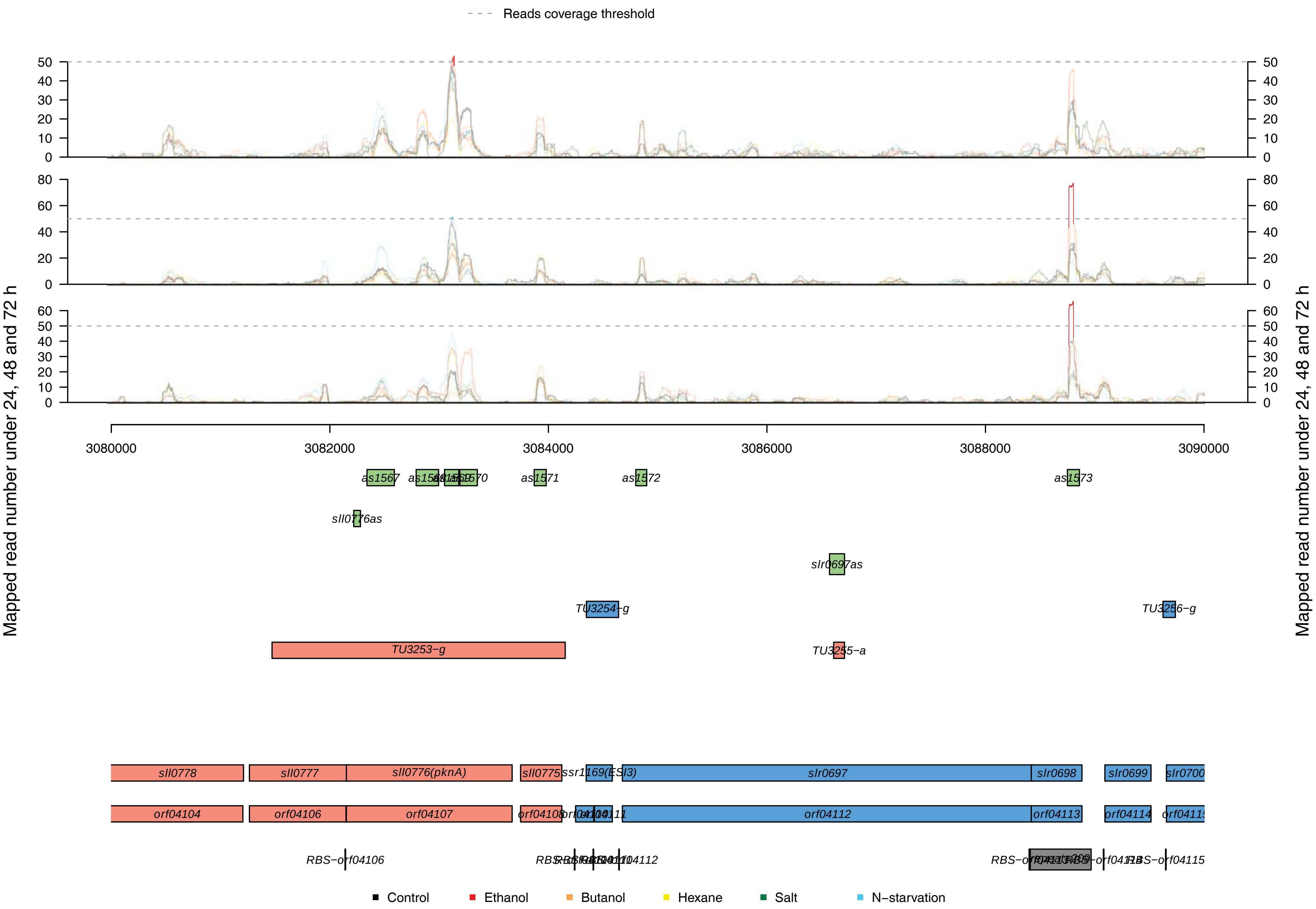

Reads coverage threshold
Mapped read number under 24, 48 and 72 h
3080000
3082000
3084000
3086000
3088000
3090000
as1567
as1571
as1572
as1573
sll0776as
slr0697as
TU3254−g
TU3256−g
TU3253−g
TU3255−a
sll0778
sll0777
sll0776(pknA)
sll0775
ssr1169(ESI3)
slr0697
slr0698
slr0699
slr0700
orf04104
orf04106
orf04107
orf04108
orf04112
orf04113
orf04114
RBS−orf04106
RBS−orf04115
Control
Ethanol
Butanol
Hexane
Salt
N−starvation

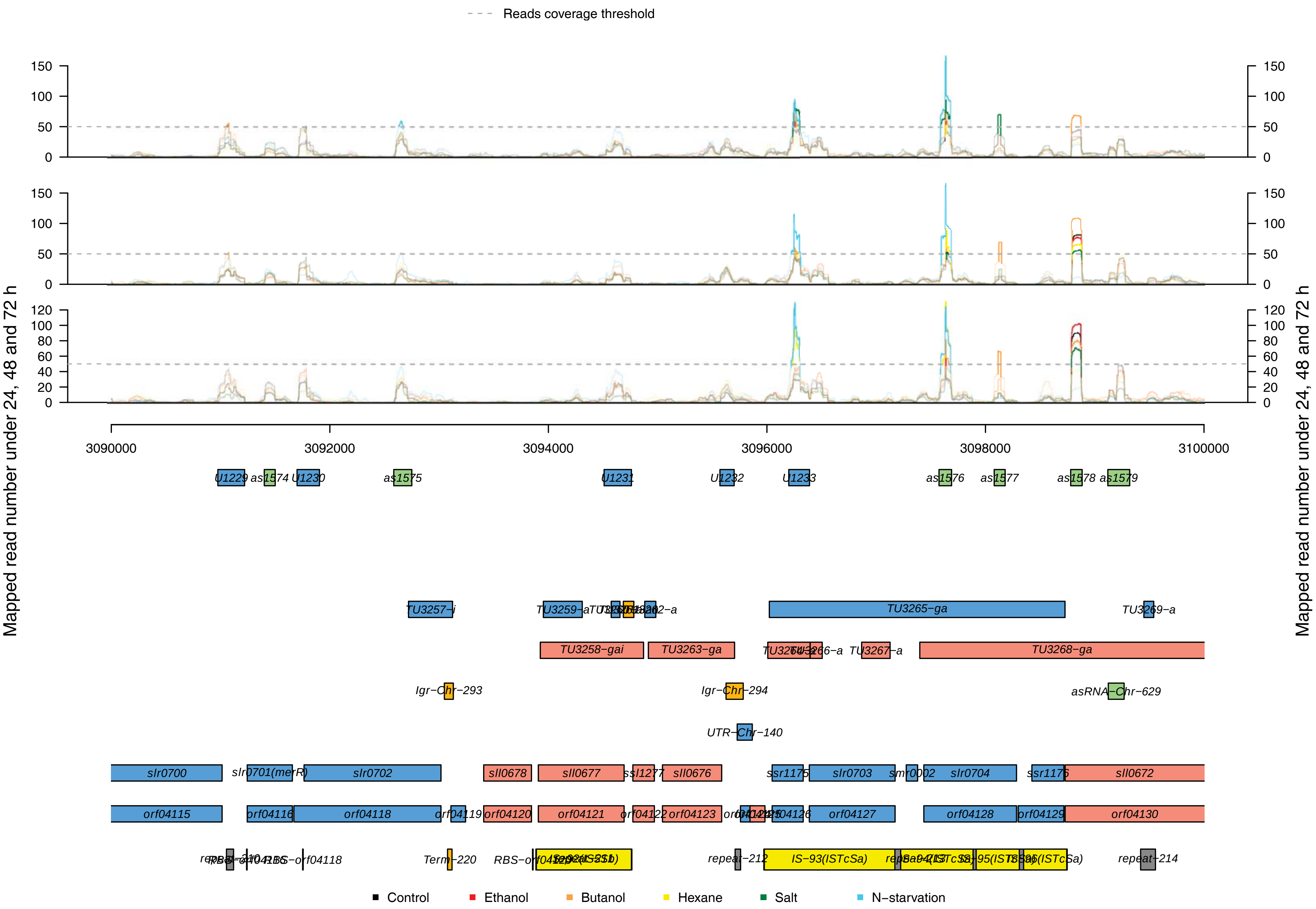

Reads coverage threshold
Mapped read number under 24, 48 and 72 h
3090000
3092000
3094000
3096000
3098000
3100000
as1574
as1575
as1576
as1577
as1578
as1579
TU3257−i
TU3259−a
TU3265−ga
TU3269−a
TU3258−gai
TU3263−ga
TU3267−a
TU3268−ga
Igr−Chr−293
Igr−Chr−294
asRNA−Chr−629
UTR−Chr−140
slr0700
slr0701(merR)
slr0702
sll0678
sll0677
ssl1277
sll0676
ssr1175
slr0703
smr0002
slr0704
ssr1176
sll0672
orf04115
orf04116
orf04118
orf04119
orf04120
orf04121
orf04122
orf04123
orf04127
orf04128
orf04129
orf04130
RBS−orf04118
Term−220
repeat−212
IS−93(ISTcSa)
repeat−214
Control
Ethanol
Butanol
Hexane
Salt
N−starvation

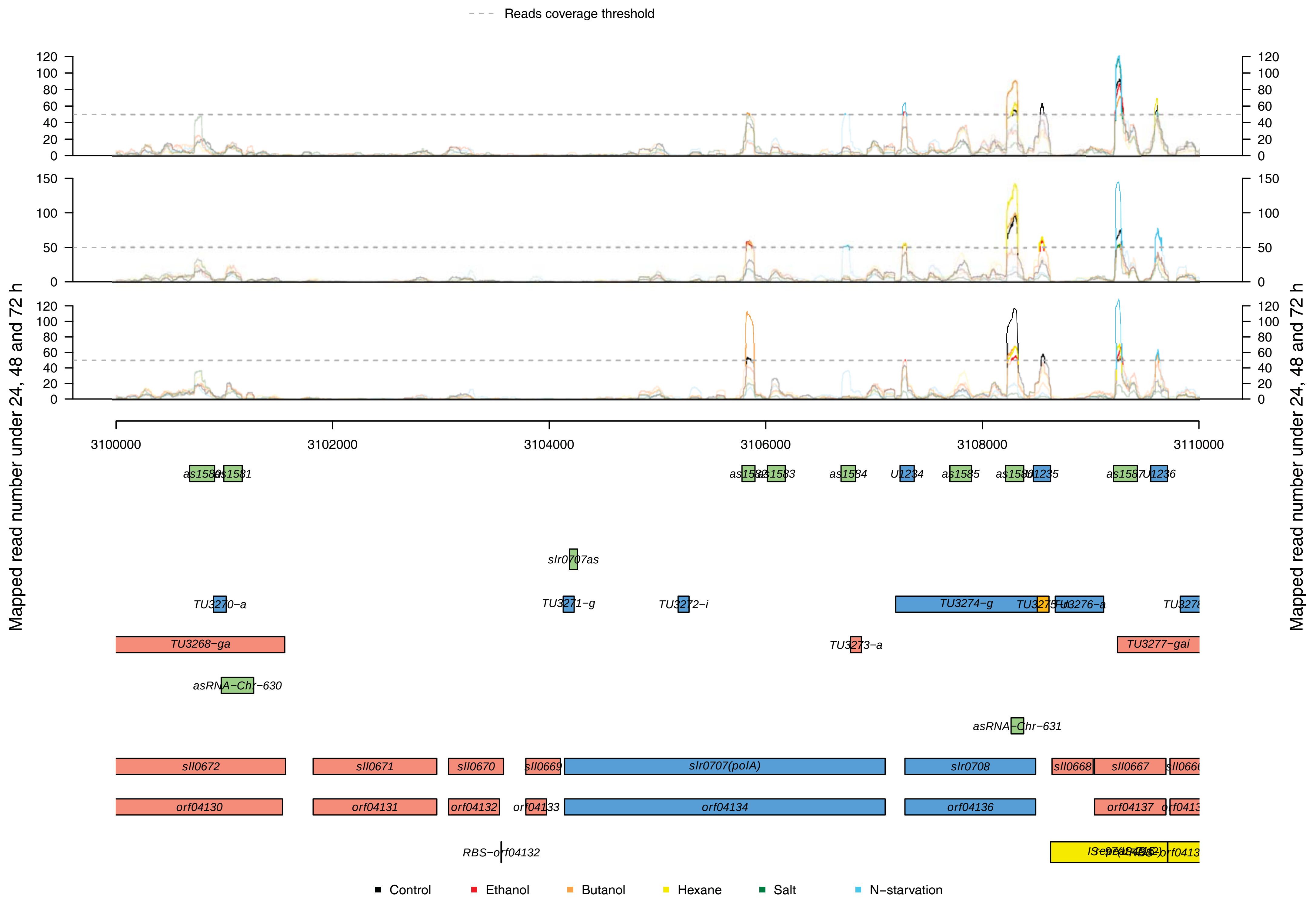

Reads coverage threshold
Mapped read number under 24, 48 and 72 h
3100000
3102000
3104000
3106000
3108000
3110000
TU3270−a
TU3271−g
TU3272−i
TU3274−g
TU3268−ga
TU3277−gai
asRNA−Chr−630
asRNA−Chr−631
slr0707as
sll0672
sll0671
sll0670
slr0707(polA)
slr0708
sll0668
sll0667
orf04130
orf04131
orf04132
orf04134
orf04136
orf04137
RBS−orf04132
Control
Ethanol
Butanol
Hexane
Salt
N−starvation

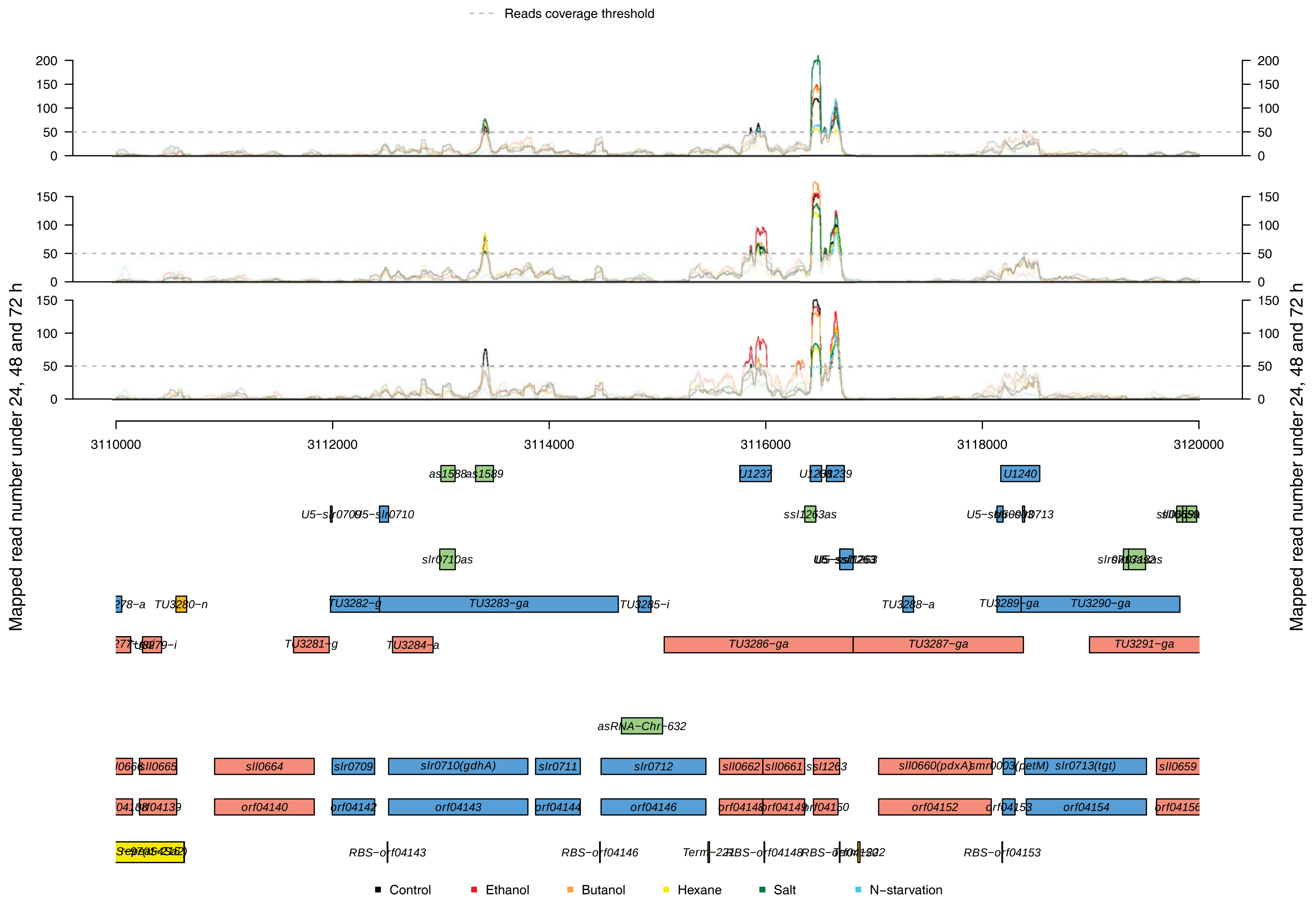

Reads coverage threshold
Mapped read number under 24, 48 and 72 h
3110000
3112000
3114000
3116000
3118000
3120000
TU3283−ga
TU3290−ga
TU3286−ga
TU3287−ga
TU3291−ga
asRNA−Chr−632
sll0664
slr0709
slr0710(gdhA)
slr0711
slr0712
sll0662
sll0661
ssl1263
sll0660(pdxA)
slr0713(tgt)
sll0659
orf04140
orf04142
orf04143
orf04144
orf04146
orf04152
orf04154
RBS−orf04143
RBS−orf04146
RBS−orf04153
Control
Ethanol
Butanol
Hexane
Salt
N−starvation

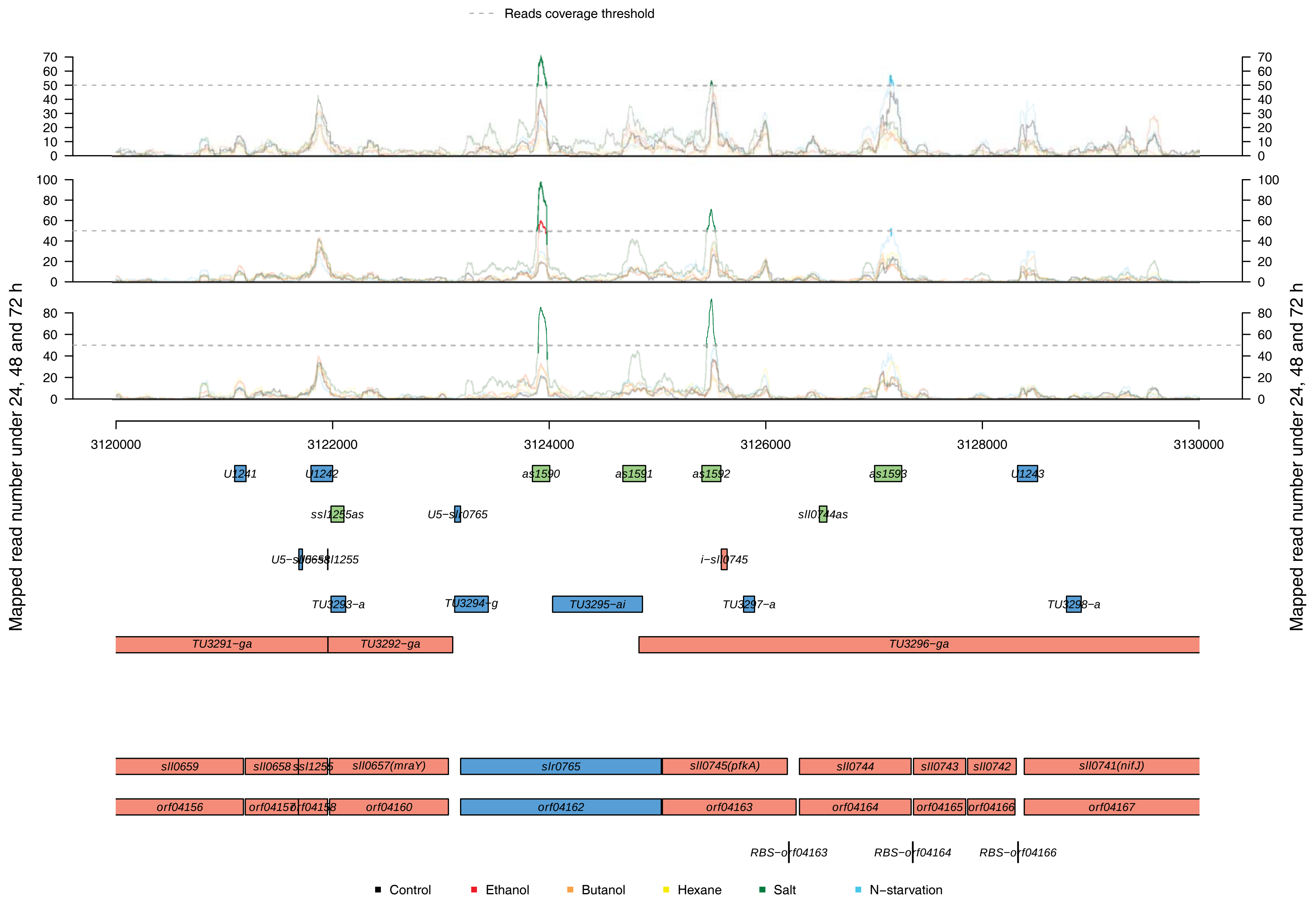
Reads coverage threshold
Mapped read number under 24, 48 and 72 h
3120000
3122000
3124000
3126000
3128000
3130000
U1241
U1242
as1590
as1591
as1592
as1593
U1243
ssl1255as
U5−slr0765
sll0744as
U5−sll0658
ssl1255
i−sll0745
TU3293−a
TU3294−g
TU3295−ai
TU3297−a
TU3298−a
TU3291−ga
TU3292−ga
TU3296−ga
sll0659
sll0658
ssl1255
sll0657(mraY)
slr0765
sll0745(pfkA)
sll0744
sll0743
sll0742
sll0741(nifJ)
orf04156
orf04157
orf04158
orf04160
orf04162
orf04163
orf04164
orf04165
orf04166
orf04167
RBS−orf04163
RBS−orf04164
RBS−orf04166
Control
Ethanol
Butanol
Hexane
Salt
N−starvation

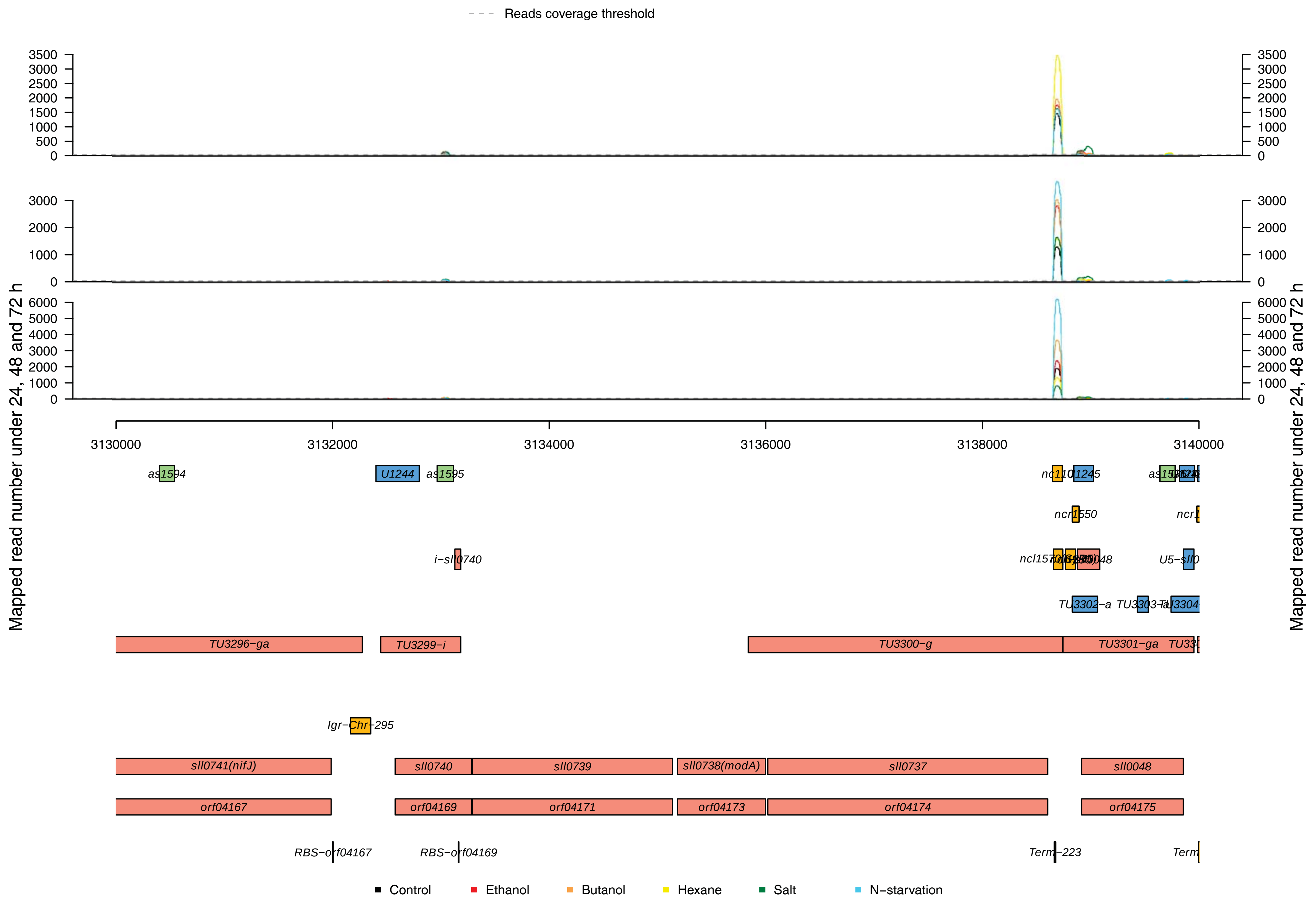

Reads coverage threshold
Mapped read number under 24, 48 and 72 h
3130000
3132000
3134000
3136000
3138000
3140000
as1594
U1244
as1595
ncr1550
i-sll0740
TU3296-ga
TU3299-i
TU3300-g
TU3301-ga
Igr-Chr-295
sll0741(nifJ)
sll0740
sll0739
sll0738(modA)
sll0737
sll0048
orf04167
orf04169
orf04171
orf04173
orf04174
orf04175
RBS-orf04167
RBS-orf04169
Term-223
Control
Ethanol
Butanol
Hexane
Salt
N-starvation

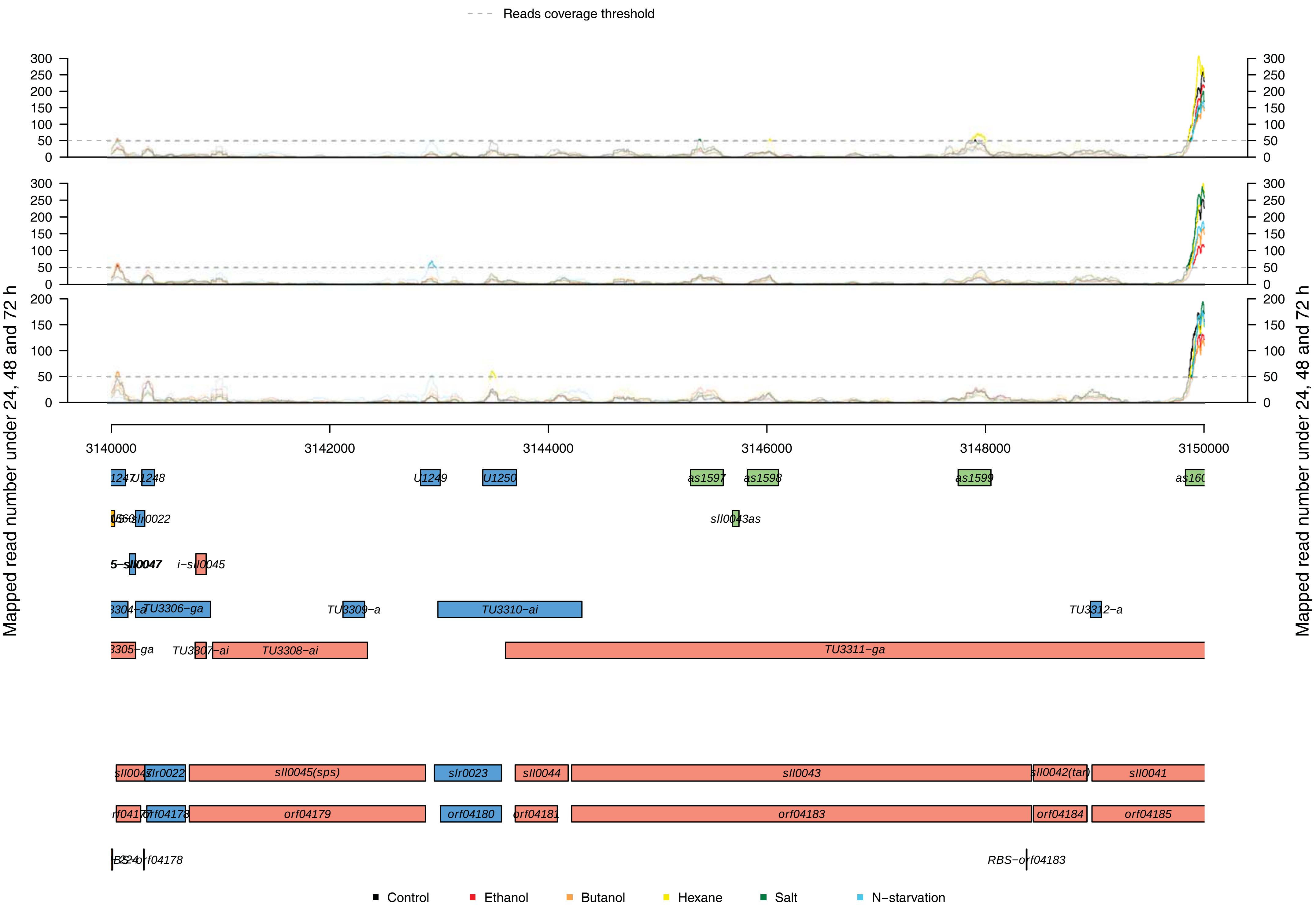
Reads coverage threshold
Mapped read number under 24, 48 and 72 h
3140000
3142000
3144000
3146000
3148000
3150000
U1248
U1249
U1250
as1597
as1598
as1599
slr0022
sll0043as
i−sll0045
TU3306−ga
TU3309−a
TU3310−ai
TU3312−a
TU3308−ai
TU3311−ga
sll0045(sps)
slr0023
sll0044
sll0043
sll0041
orf04179
orf04180
orf04183
orf04184
orf04185
RBS−orf04183
Control
Ethanol
Butanol
Hexane
Salt
N−starvation

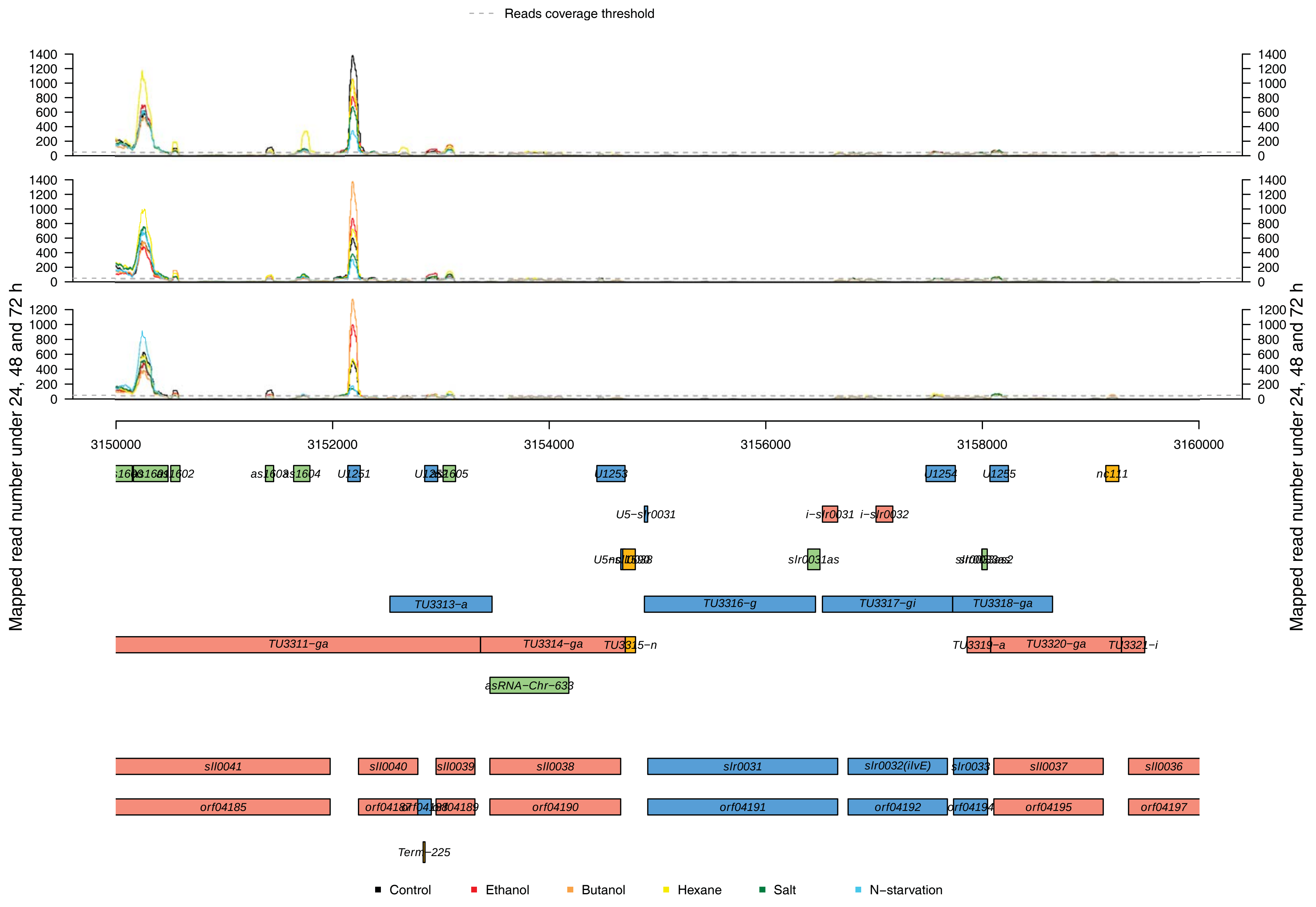
Reads coverage threshold
Mapped read number under 24, 48 and 72 h
3150000
3152000
3154000
3156000
3158000
3160000
as1602
as1603
as1604
U1251
U1252
as1605
U1253
U1254
U1255
nc111
U5−slr0031
i−slr0031
i−slr0032
slr0031as
TU3313−a
TU3316−g
TU3317−gi
TU3318−ga
TU3311−ga
TU3314−ga
TU3315−n
TU3319−a
TU3320−ga
TU3321−i
asRNA−Chr−633
sll0041
sll0040
sll0039
sll0038
slr0031
slr0032(ilvE)
slr0033
sll0037
sll0036
orf04185
orf04187
orf04189
orf04190
orf04191
orf04192
orf04194
orf04195
orf04197
Term−225
Control
Ethanol
Butanol
Hexane
Salt
N−starvation

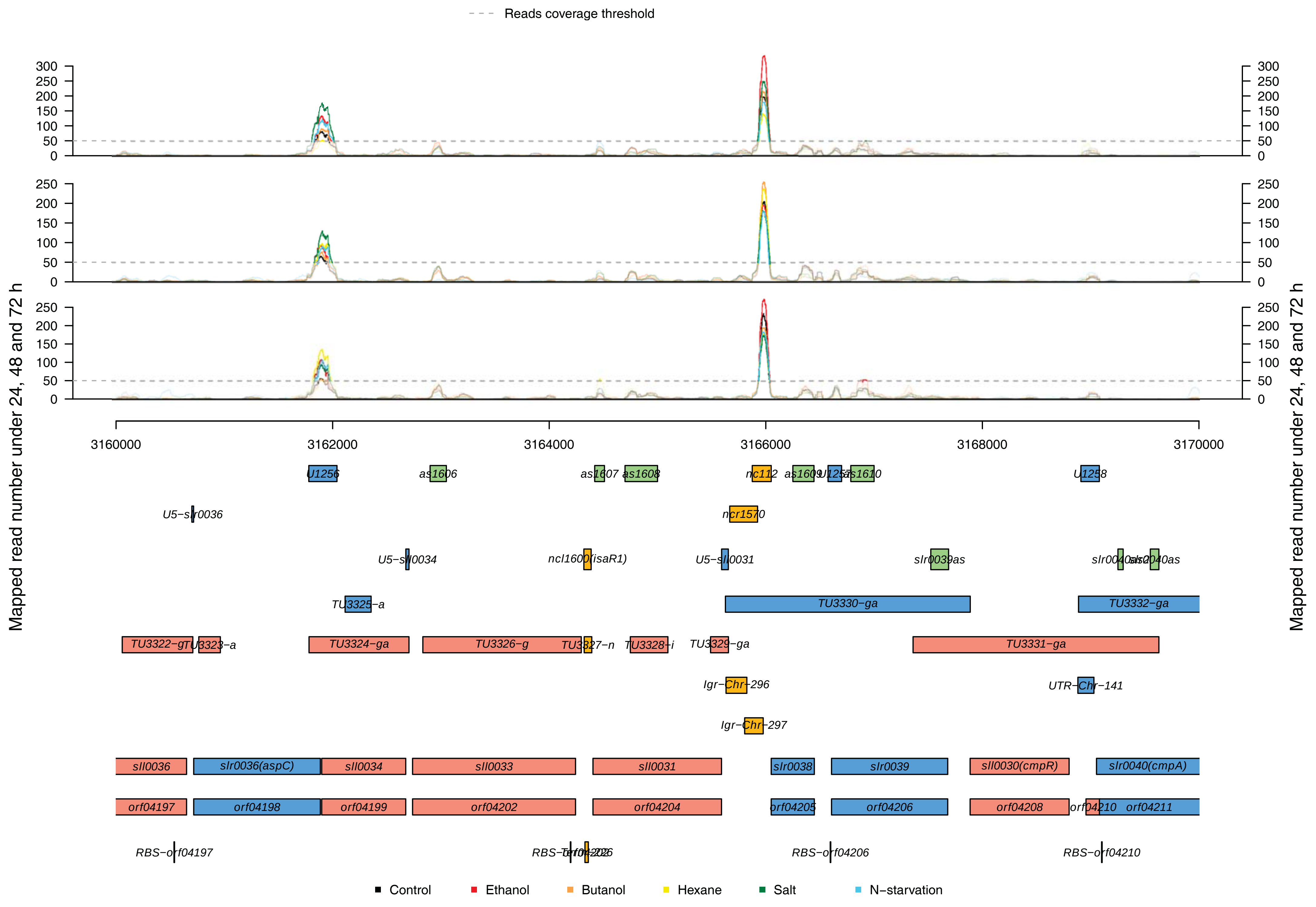

Reads coverage threshold
Mapped read number under 24, 48 and 72 h
3160000
3162000
3164000
3166000
3168000
3170000
U1256
as1606
as1607
as1608
nc112
as1609
U1257
as1610
U1258
U5−slr0036
ncr1570
U5−sll0034
ncl1600(isaR1)
U5−sll0031
slr0039as
slr0040as
TU3325−a
TU3330−ga
TU3332−ga
TU3324−ga
TU3326−g
TU3328−i
TU3329−ga
TU3331−ga
Igr−Chr−296
UTR−Chr−141
Igr−Chr−297
sll0036
slr0036(aspC)
sll0034
sll0033
sll0031
slr0038
slr0039
sll0030(cmpR)
slr0040(cmpA)
orf04197
orf04198
orf04199
orf04202
orf04204
orf04205
orf04206
orf04208
orf04210
orf04211
RBS−orf04197
RBS−orf04206
RBS−orf04210
Control
Ethanol
Butanol
Hexane
Salt
N−starvation

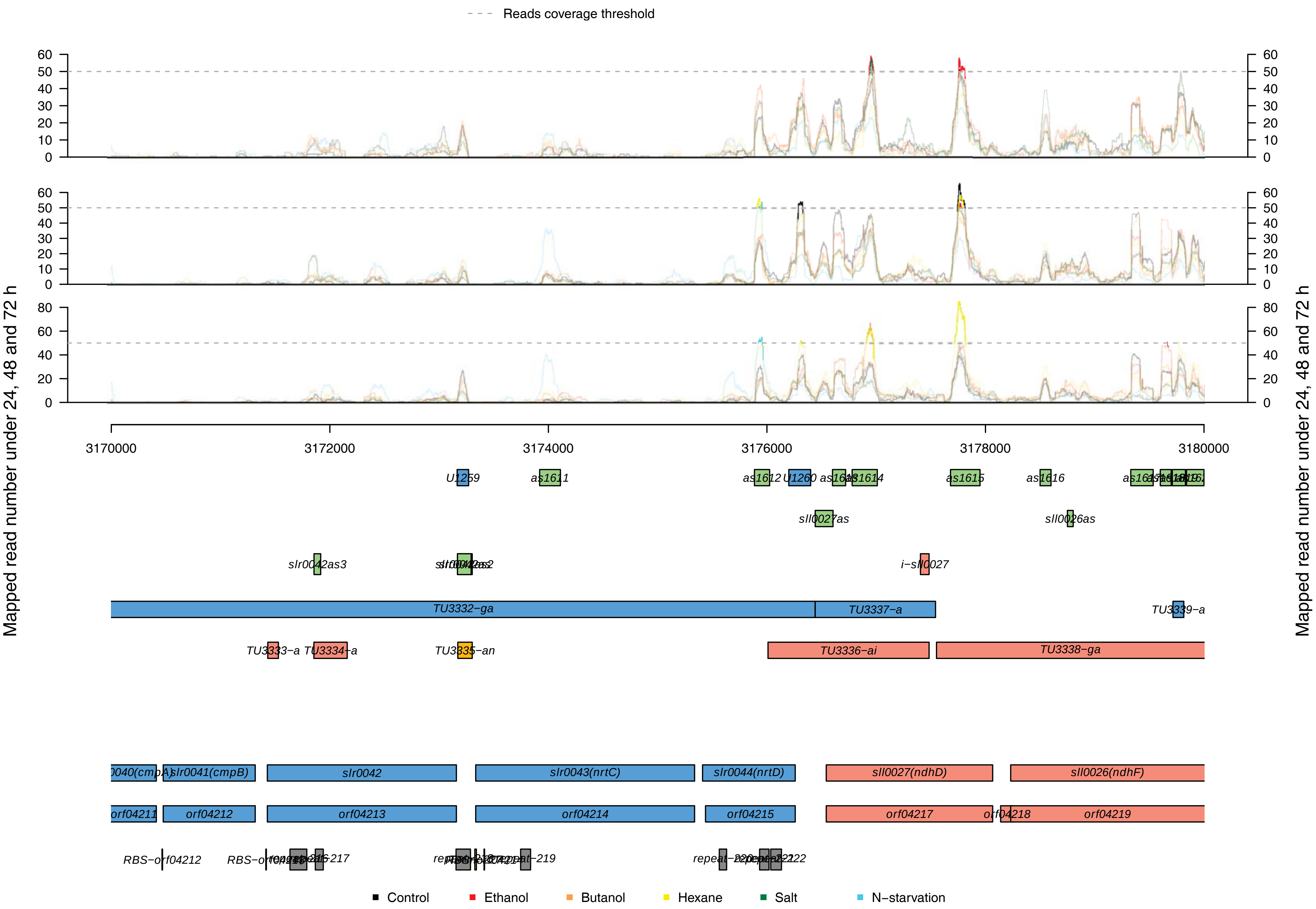

Reads coverage threshold
Mapped read number under 24, 48 and 72 h
3170000
3172000
3174000
3176000
3178000
3180000
U1259
as1611
as1612
U1260
as1614
as1615
as1616
sll0027as
sll0026as
slr0042as3
i−sll0027
TU3332−ga
TU3337−a
TU3339−a
TU3333−a
TU3334−a
TU3335−an
TU3336−ai
TU3338−ga
slr0041(cmpB)
slr0042
slr0043(nrtC)
slr0044(nrtD)
sll0027(ndhD)
sll0026(ndhF)
orf04211
orf04212
orf04213
orf04214
orf04215
orf04217
orf04218
orf04219
RBS−orf04212
repeat−219
Control
Ethanol
Butanol
Hexane
Salt
N−starvation

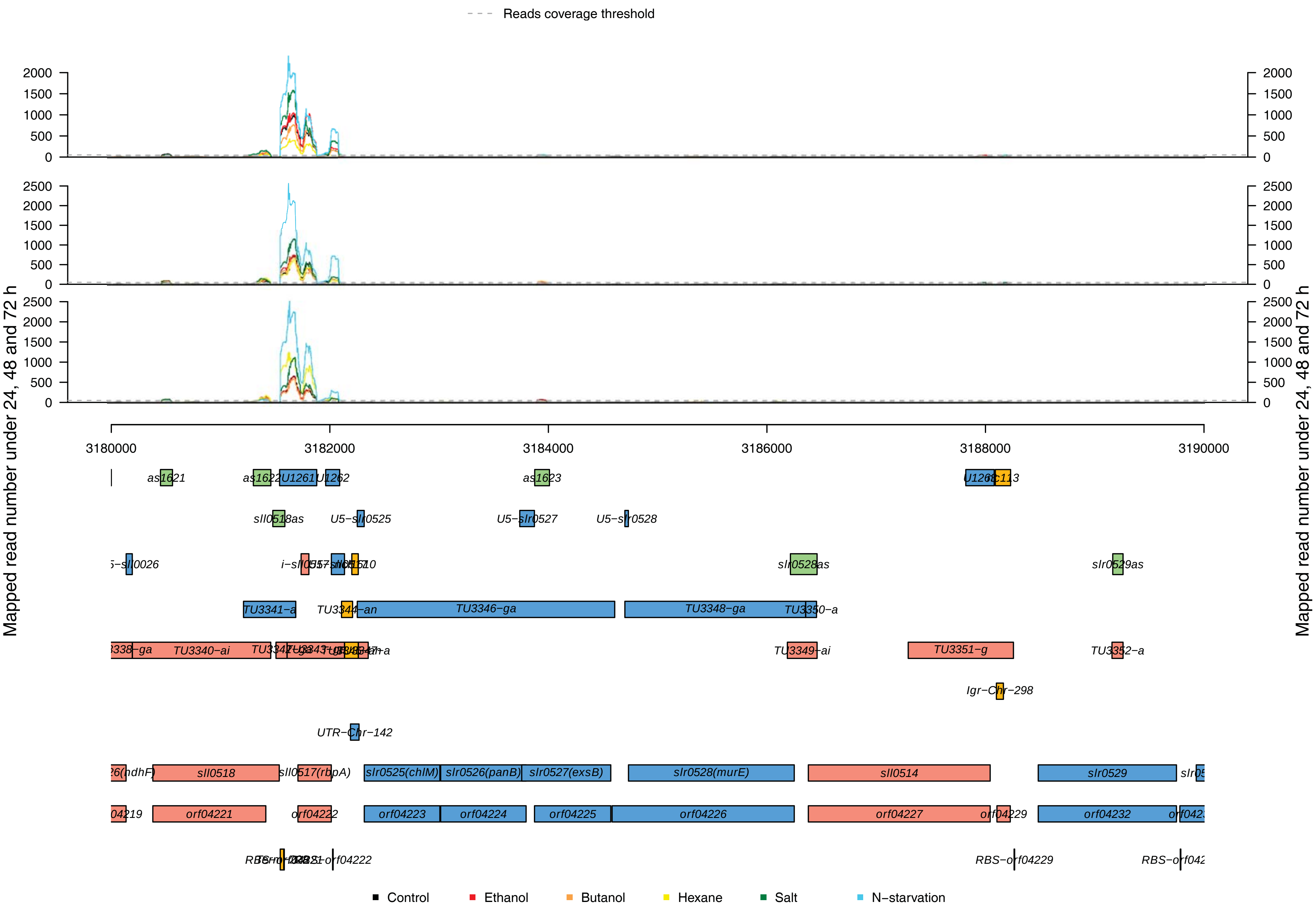

Reads coverage threshold
Mapped read number under 24, 48 and 72 h
3180000
3182000
3184000
3186000
3188000
3190000
as1621
as1623
U5−slr0525
U5−slr0527
U5−slr0528
slr0529as
TU3341−a
TU3346−ga
TU3348−ga
TU3340−ai
TU3349−ai
TU3351−g
TU3352−a
Igr−Chr−298
UTR−Chr−142
sll0518
sll0517(rbpA)
slr0525(chlM)
slr0526(panB)
slr0527(exsB)
slr0528(murE)
sll0514
slr0529
orf04221
orf04222
orf04223
orf04224
orf04225
orf04226
orf04227
orf04232
RBS−orf04229
Control
Ethanol
Butanol
Hexane
Salt
N−starvation

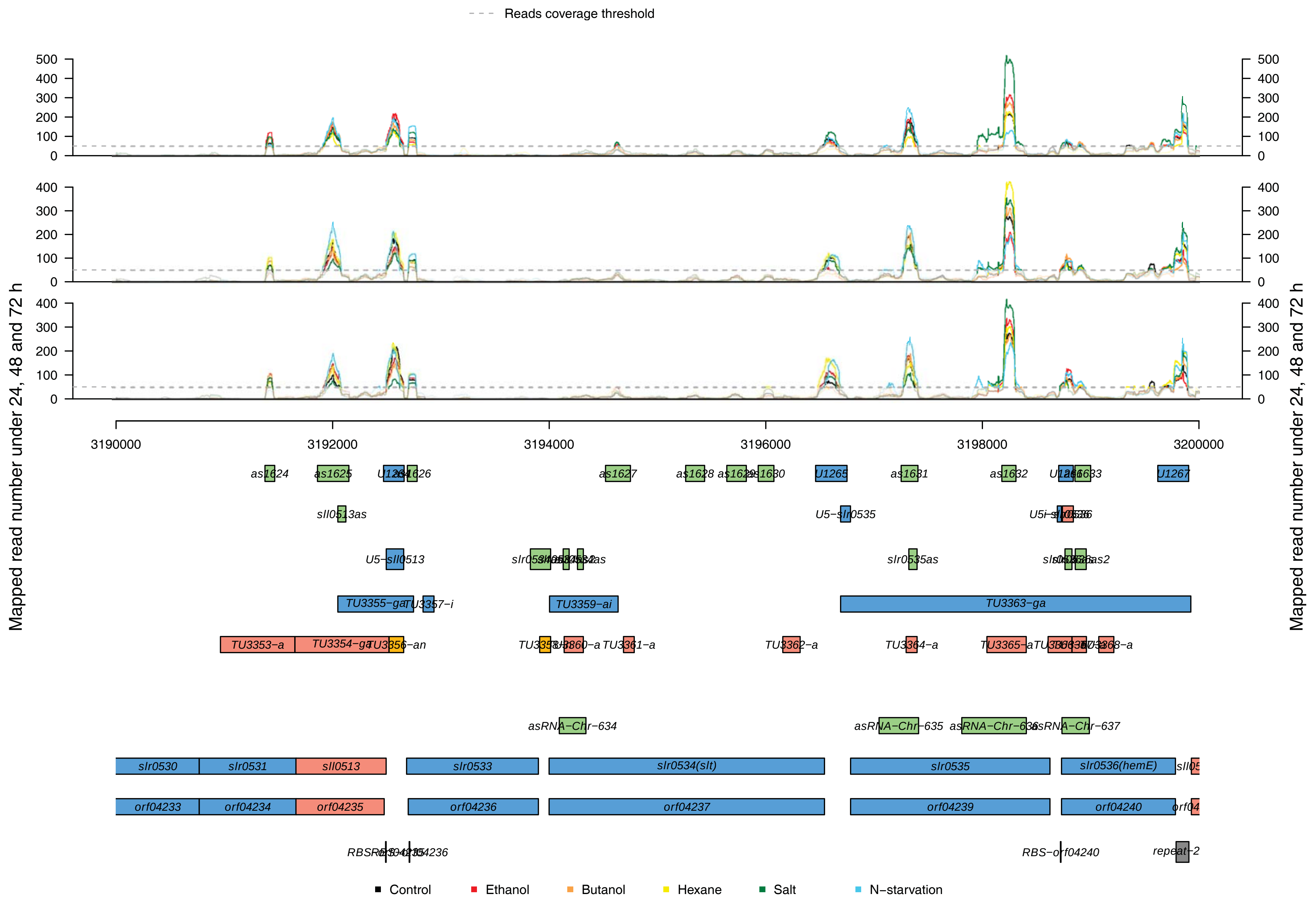

Reads coverage threshold
Mapped read number under 24, 48 and 72 h
3190000
3192000
3194000
3196000
3198000
3200000
as1624
as1625
as1627
as1631
as1632
U1265
U1267
sll0513as
U5−slr0535
U5−sll0513
slr0535as
TU3355−ga
TU3359−ai
TU3363−ga
TU3353−a
TU3361−a
TU3362−a
TU3364−a
asRNA−Chr−634
asRNA−Chr−635
asRNA−Chr−637
slr0530
slr0531
sll0513
slr0533
slr0534(slt)
slr0535
slr0536(hemE)
orf04233
orf04234
orf04235
orf04236
orf04237
orf04239
orf04240
RBS−orf04240
Control
Ethanol
Butanol
Hexane
Salt
N−starvation

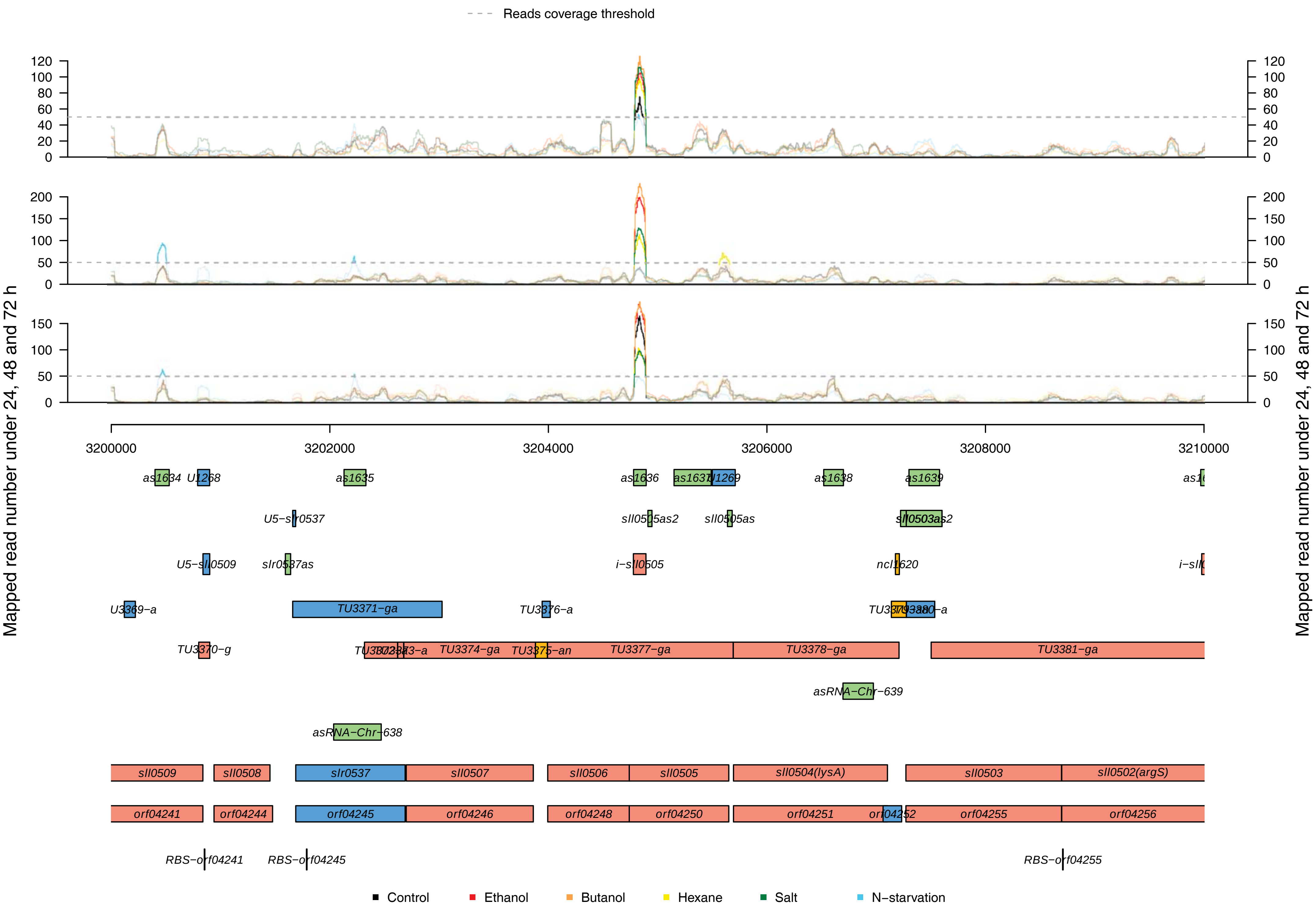
Reads coverage threshold
Mapped read number under 24, 48 and 72 h
3200000
3202000
3204000
3206000
3208000
3210000
Control
Ethanol
Butanol
Hexane
Salt
N−starvation

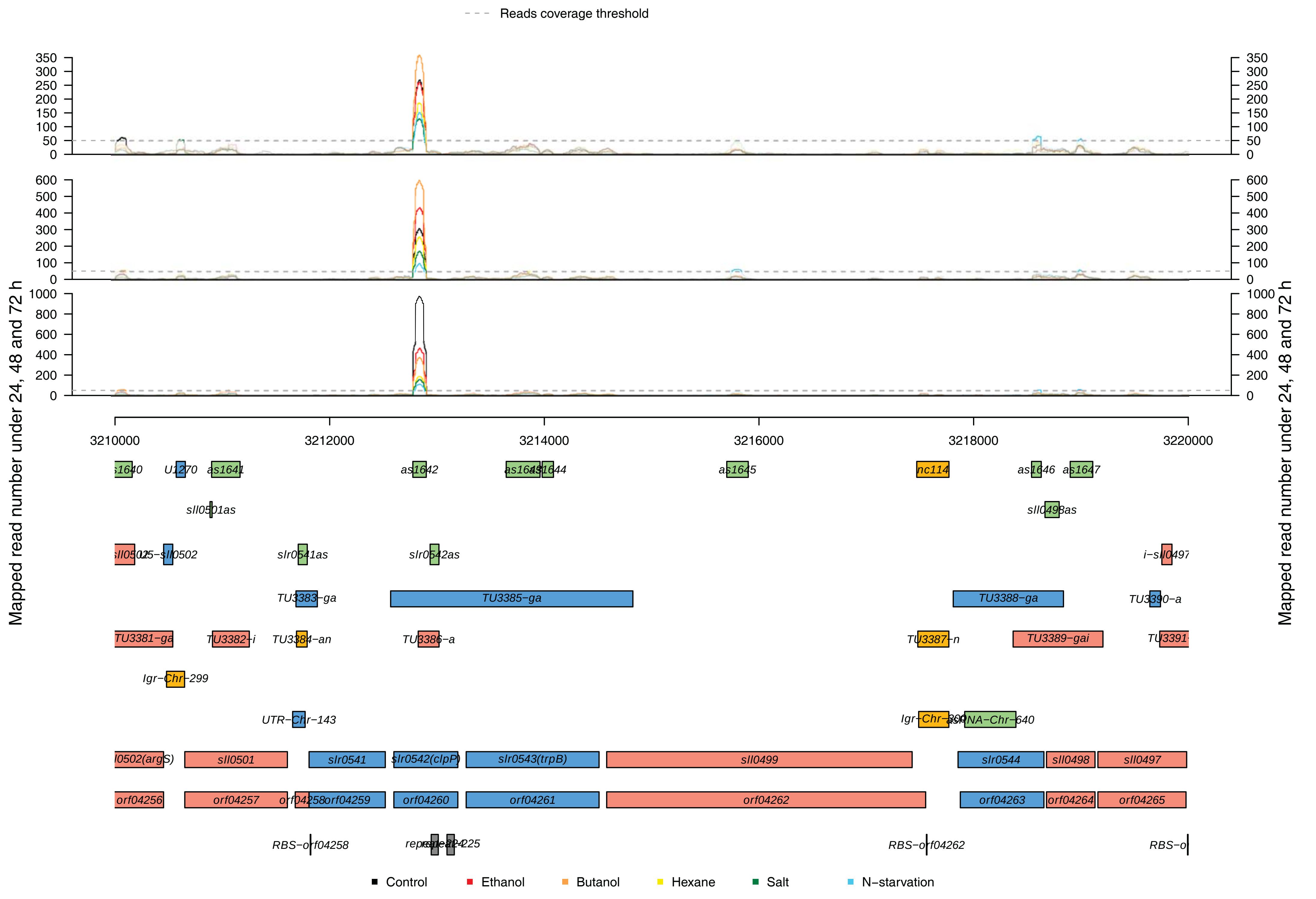

Reads coverage threshold
Mapped read number under 24, 48 and 72 h
3210000
3212000
3214000
3216000
3218000
3220000
U1270
as1641
as1642
as1645
nc114
as1646
as1647
sll0501as
sll0498as
slr0541as
slr0542as
TU3383−ga
TU3385−ga
TU3388−ga
TU3390−a
TU3381−ga
TU3384−an
TU3386−a
TU3387−n
TU3389−gai
Igr−Chr−299
UTR−Chr−143
sll0501
slr0541
slr0542(clpP)
slr0543(trpB)
sll0499
slr0544
sll0498
sll0497
orf04256
orf04257
orf04259
orf04260
orf04261
orf04262
orf04263
orf04264
orf04265
RBS−orf04258
RBS−orf04262
Control
Ethanol
Butanol
Hexane
Salt
N−starvation

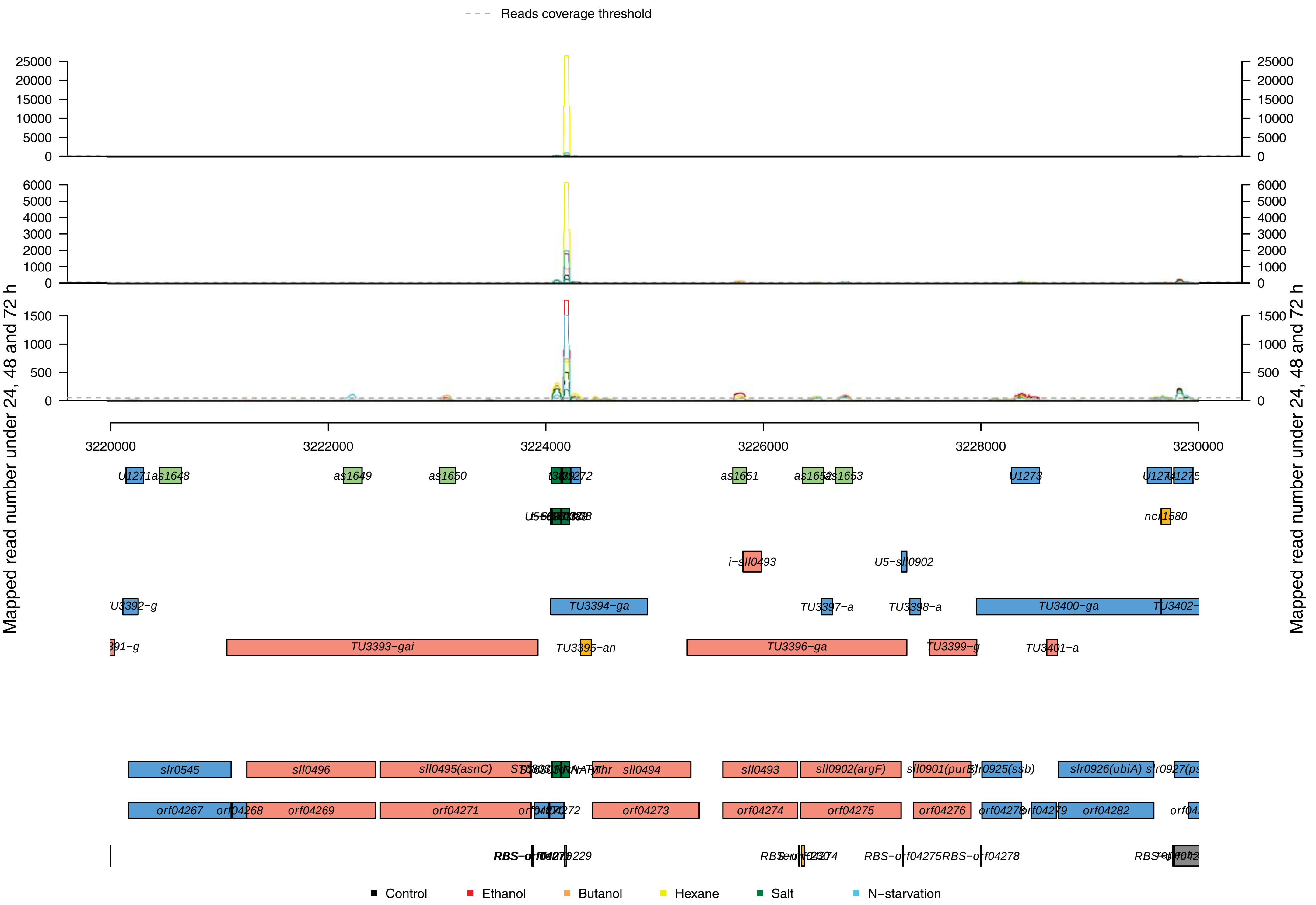

Reads coverage threshold
Mapped read number under 24, 48 and 72 h
3220000
3222000
3224000
3226000
3228000
3230000
TU3393−gai
TU3394−ga
TU3396−ga
TU3400−ga
slr0545
sll0496
sll0495(asnC)
sll0494
sll0493
sll0902(argF)
slr0926(ubiA)
orf04267
orf04269
orf04271
orf04273
orf04274
orf04275
orf04276
orf04282
Control
Ethanol
Butanol
Hexane
Salt
N−starvation

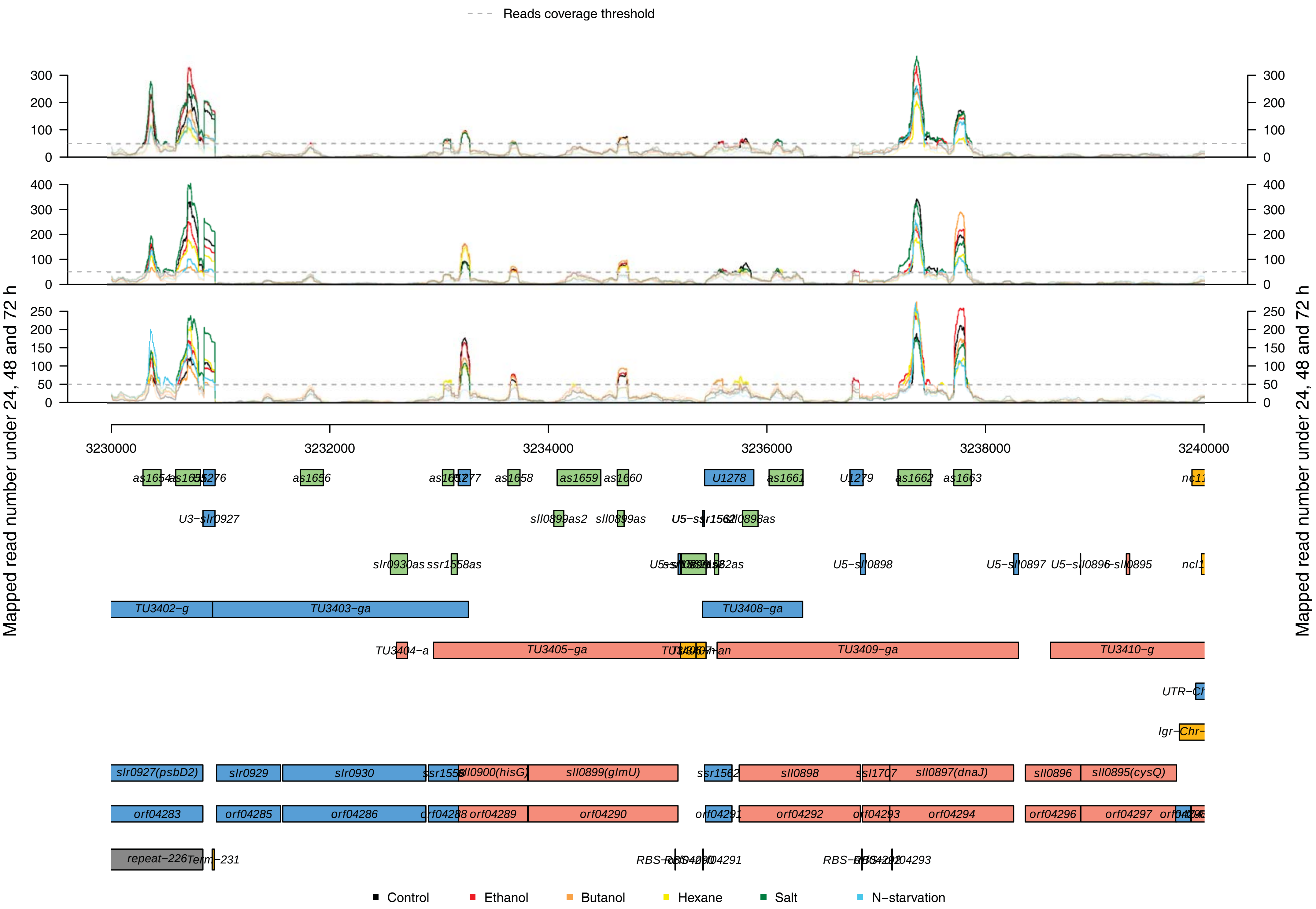

Reads coverage threshold
Mapped read number under 24, 48 and 72 h
3230000
3232000
3234000
3236000
3238000
3240000
as1656
as1658
as1659
as1660
U1278
as1661
U1279
as1662
as1663
U3−slr0927
sll0899as2
sll0899as
slr0930as
ssr1558as
U5−sll0898
U5−sll0897
TU3402−g
TU3403−ga
TU3408−ga
TU3404−a
TU3405−ga
TU3409−ga
TU3410−g
slr0927(psbD2)
slr0929
slr0930
sll0899(glmU)
sll0898
sll0897(dnaJ)
sll0896
sll0895(cysQ)
orf04283
orf04285
orf04286
orf04289
orf04290
orf04292
orf04294
orf04296
orf04297
Control
Ethanol
Butanol
Hexane
Salt
N−starvation

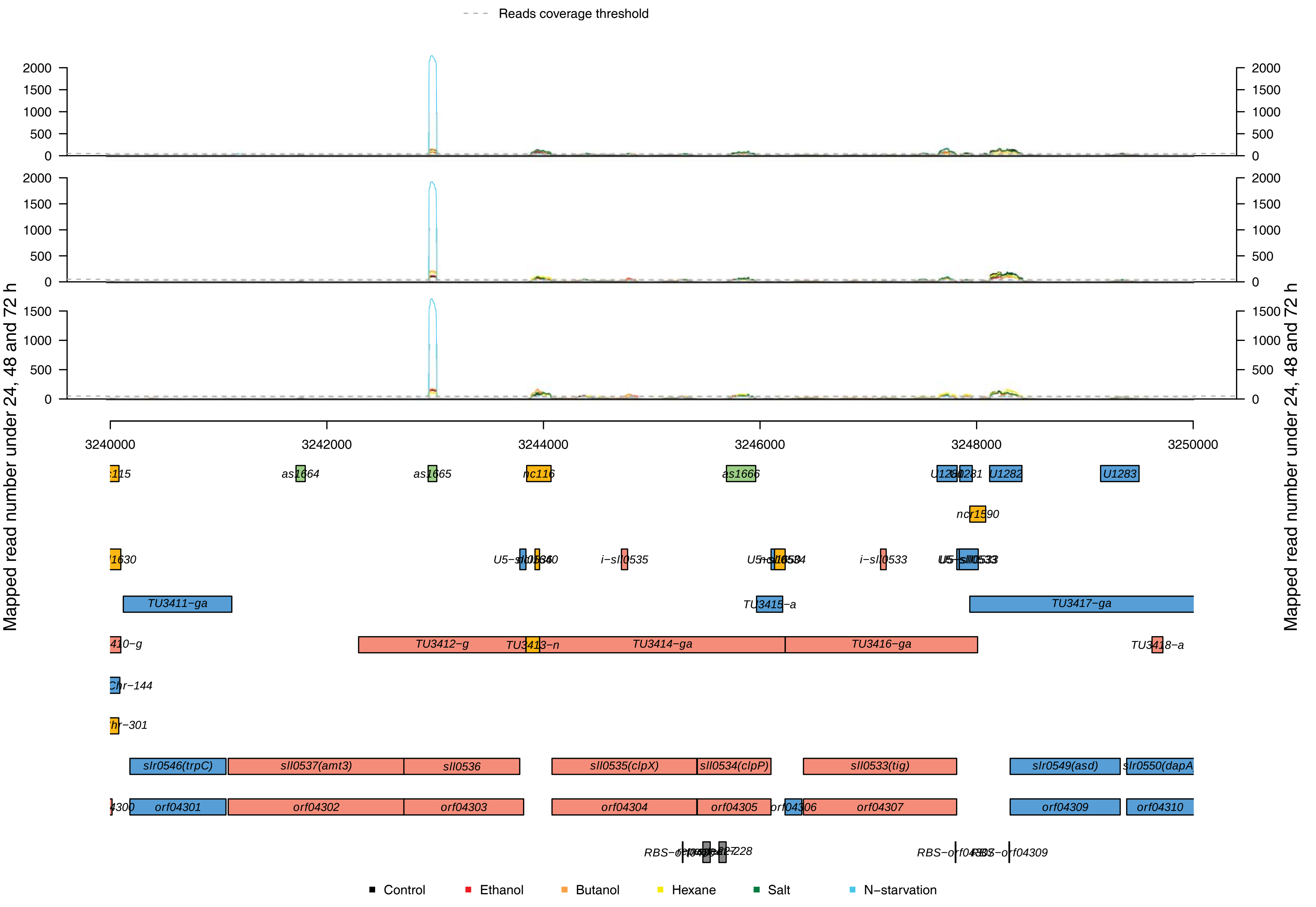

Reads coverage threshold
Mapped read number under 24, 48 and 72 h
3240000
3242000
3244000
3246000
3248000
3250000
TU3411-ga
TU3412-g
TU3414-ga
TU3416-ga
TU3417-ga
slr0546(trpC)
sll0537(amt3)
sll0536
sll0535(clpX)
sll0534(clpP)
sll0533(tig)
slr0549(asd)
orf04301
orf04302
orf04303
orf04304
orf04305
orf04307
orf04309
orf04310
Control
Ethanol
Butanol
Hexane
Salt
N-starvation

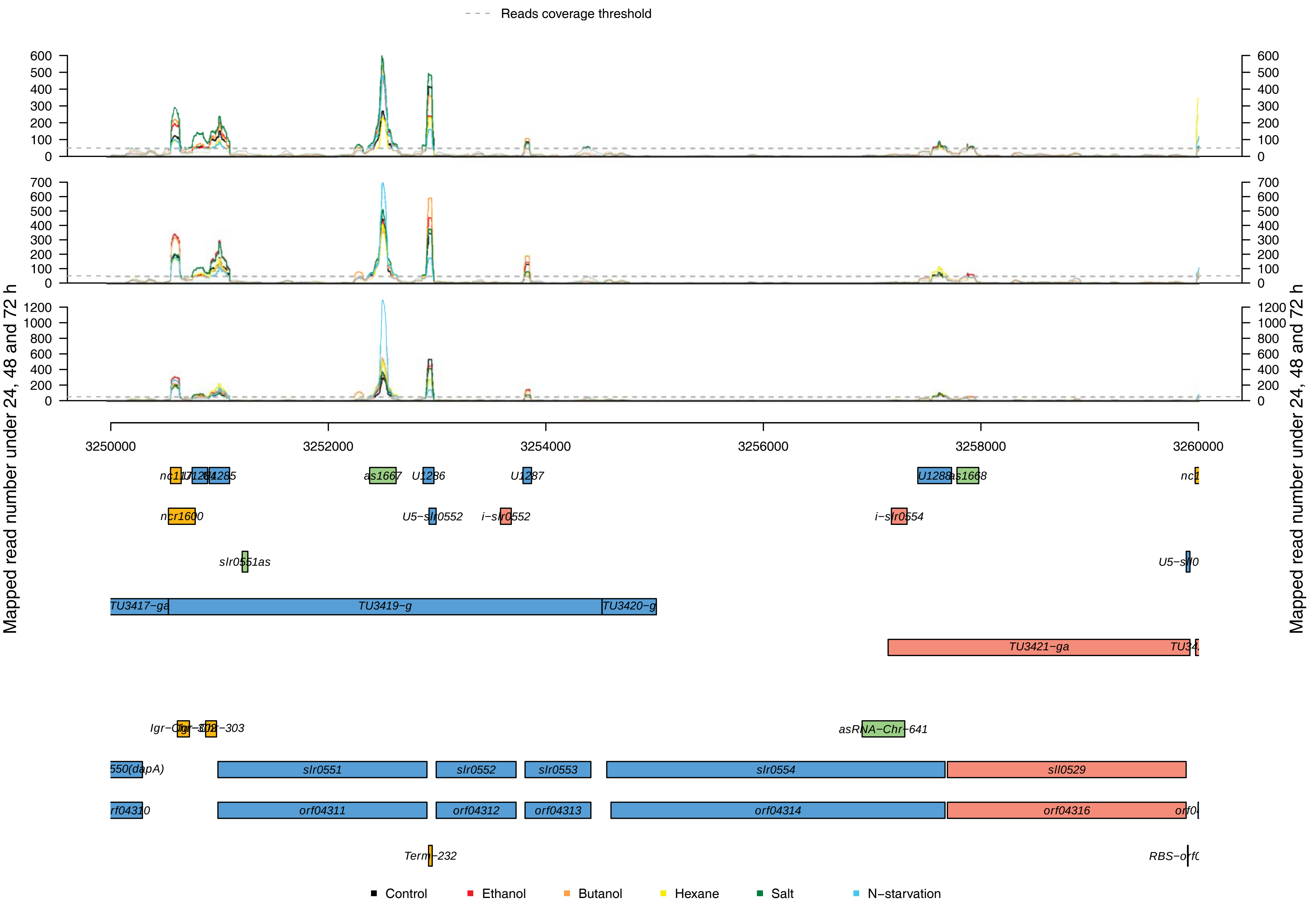

Reads coverage threshold
Mapped read number under 24, 48 and 72 h
3250000
3252000
3254000
3256000
3258000
3260000
ncr1600
U5−slr0552
i−slr0552
i−slr0554
slr0551as
TU3419−g
TU3421−ga
asRNA−Chr−641
slr0551
slr0552
slr0553
slr0554
sll0529
orf04311
orf04312
orf04313
orf04314
orf04316
Term−232
Control
Ethanol
Butanol
Hexane
Salt
N−starvation

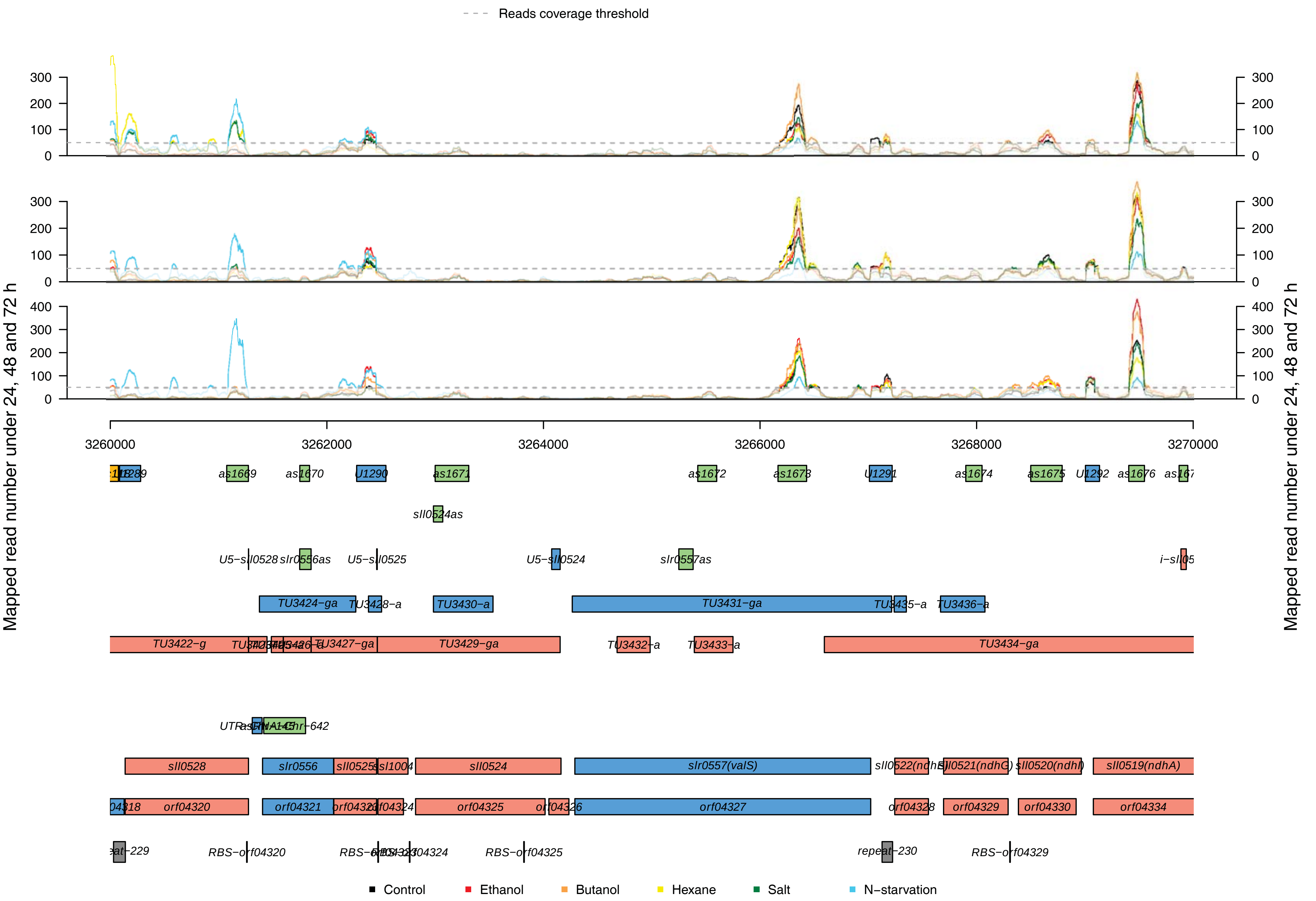

Reads coverage threshold
Mapped read number under 24, 48 and 72 h
3260000
3262000
3264000
3266000
3268000
3270000
TU3424−ga
TU3430−a
TU3431−ga
TU3422−g
TU3427−ga
TU3429−ga
TU3434−ga
sll0528
slr0556
sll0524
slr0557(valS)
sll0519(ndhA)
orf04320
orf04321
orf04325
orf04327
orf04329
orf04330
orf04334
Control
Ethanol
Butanol
Hexane
Salt
N−starvation

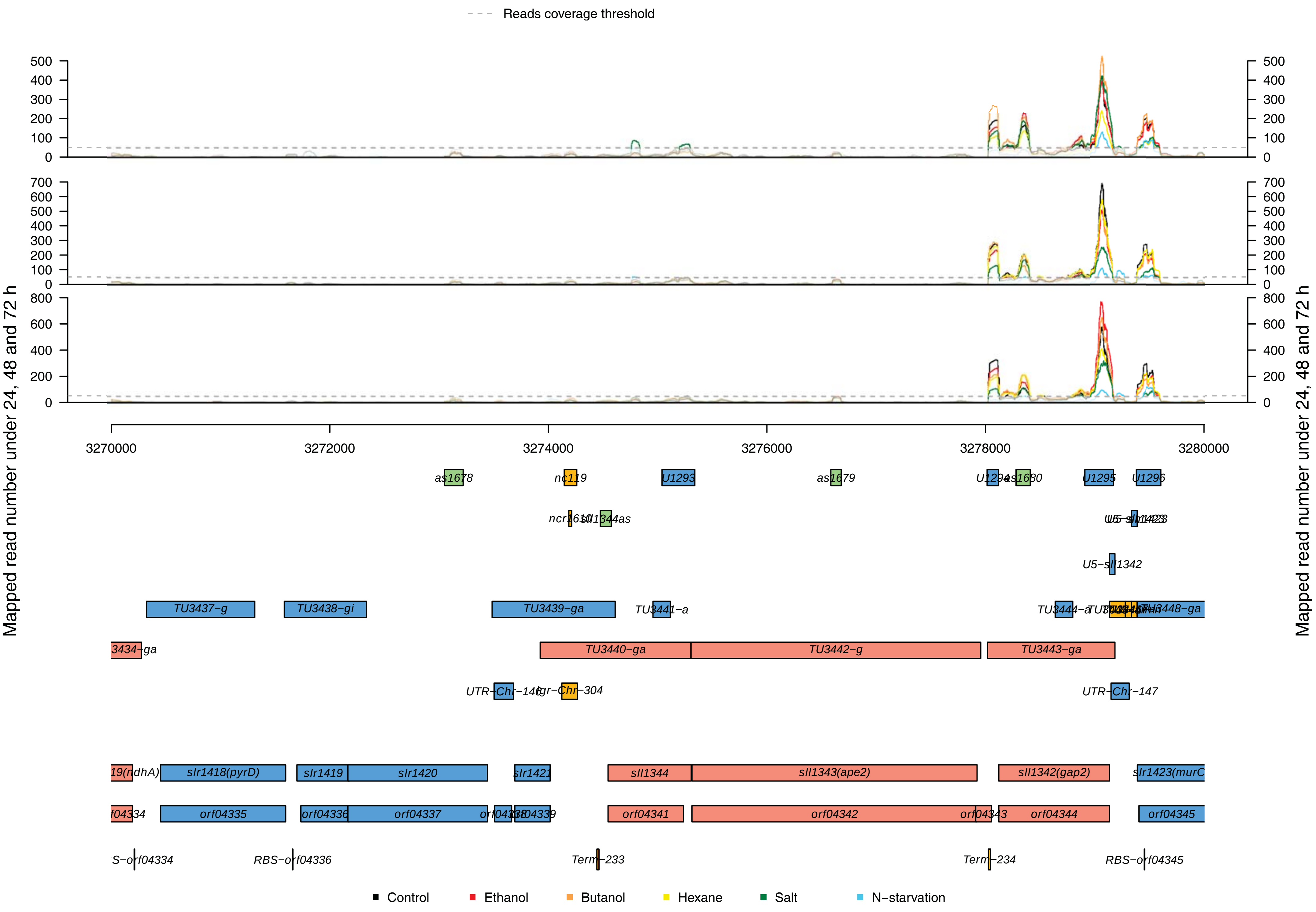

Reads coverage threshold
Mapped read number under 24, 48 and 72 h
3270000
3272000
3274000
3276000
3278000
3280000
as1678
nc119
U1293
as1679
U1294
as1680
U1295
U1296
U5−sll1342
TU3437−g
TU3438−gi
TU3439−ga
TU3441−a
TU3444−a
TU3448−ga
TU3440−ga
TU3442−g
TU3443−ga
UTR−Chr−147
slr1418(pyrD)
slr1419
slr1420
slr1421
sll1344
sll1343(ape2)
sll1342(gap2)
orf04335
orf04336
orf04337
orf04341
orf04342
orf04344
orf04345
RBS−orf04336
Term−233
Term−234
RBS−orf04345
Control
Ethanol
Butanol
Hexane
Salt
N−starvation

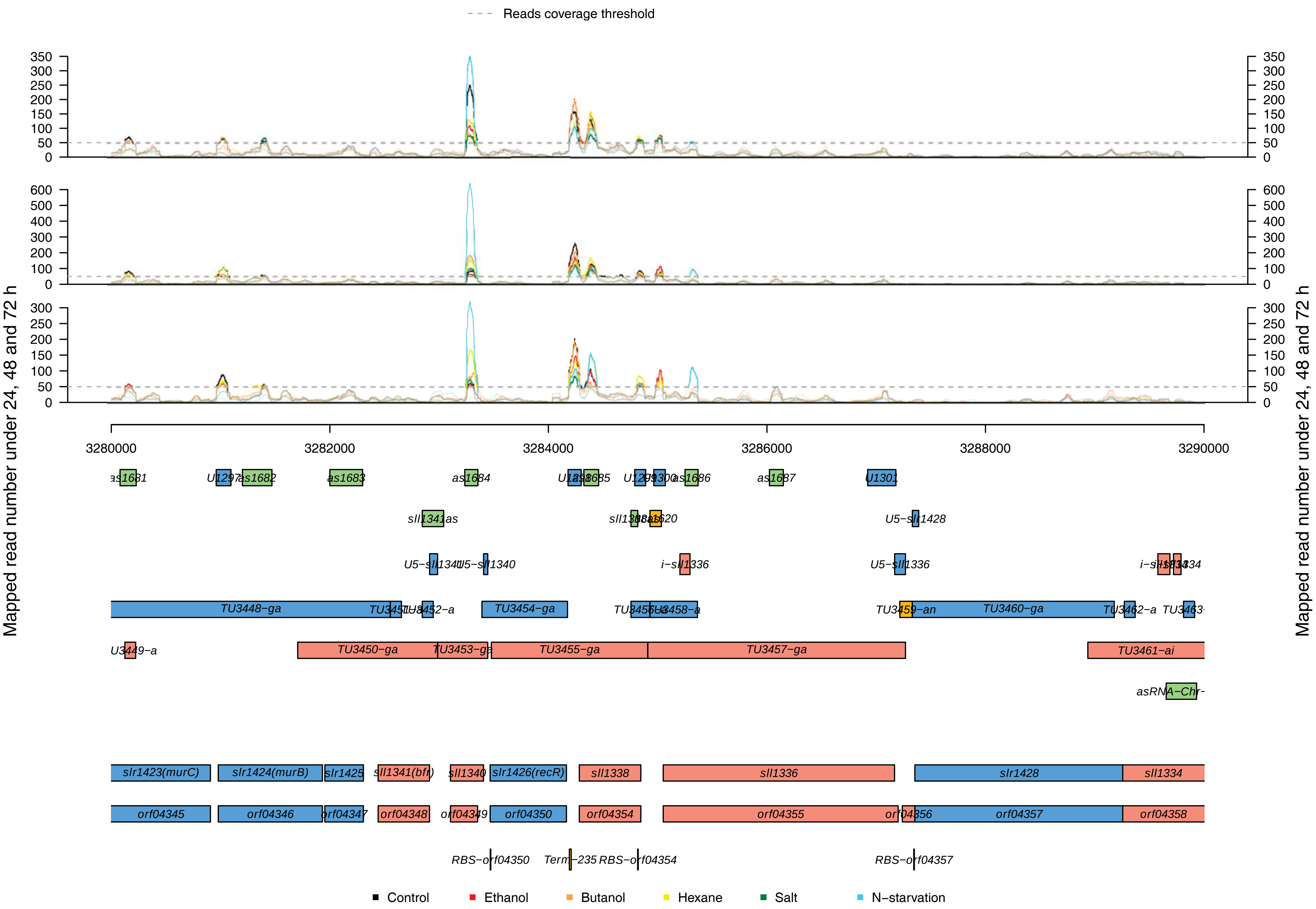
Reads coverage threshold
Mapped read number under 24, 48 and 72 h
3280000
3282000
3284000
3286000
3288000
3290000
TU3448−ga
TU3450−ga
TU3453−ga
TU3454−ga
TU3455−ga
TU3457−ga
TU3460−ga
TU3461−ai
slr1423(murC)
slr1424(murB)
slr1425
sll1341(bfr)
sll1340
slr1426(recR)
sll1338
sll1336
slr1428
sll1334
orf04345
orf04346
orf04347
orf04348
orf04349
orf04350
orf04354
orf04355
orf04356
orf04357
orf04358
RBS−orf04350
RBS−orf04354
RBS−orf04357
Control
Ethanol
Butanol
Hexane
Salt
N−starvation

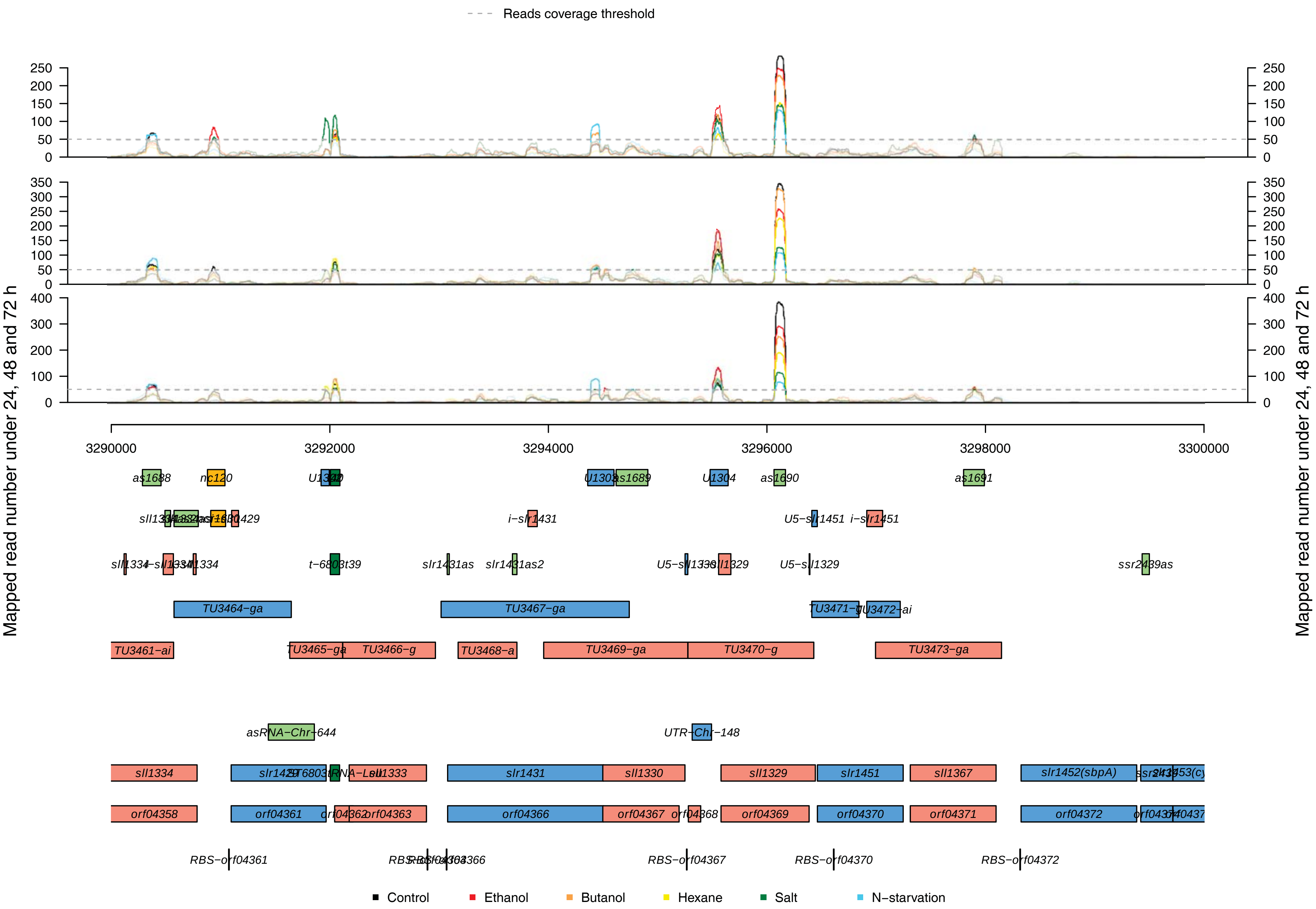

Reads coverage threshold
Mapped read number under 24, 48 and 72 h
3290000
3292000
3294000
3296000
3298000
3300000
TU3464-ga
TU3467-ga
TU3461-ai
TU3469-ga
TU3470-g
TU3473-ga
asRNA-Chr-644
UTR-Chr-148
sll1334
slr1431
sll1330
sll1329
slr1451
sll1367
slr1452(sbpA)
orf04358
orf04361
orf04366
orf04369
orf04370
orf04371
orf04372
RBS-orf04361
RBS-orf04367
RBS-orf04370
RBS-orf04372
Control
Ethanol
Butanol
Hexane
Salt
N-starvation

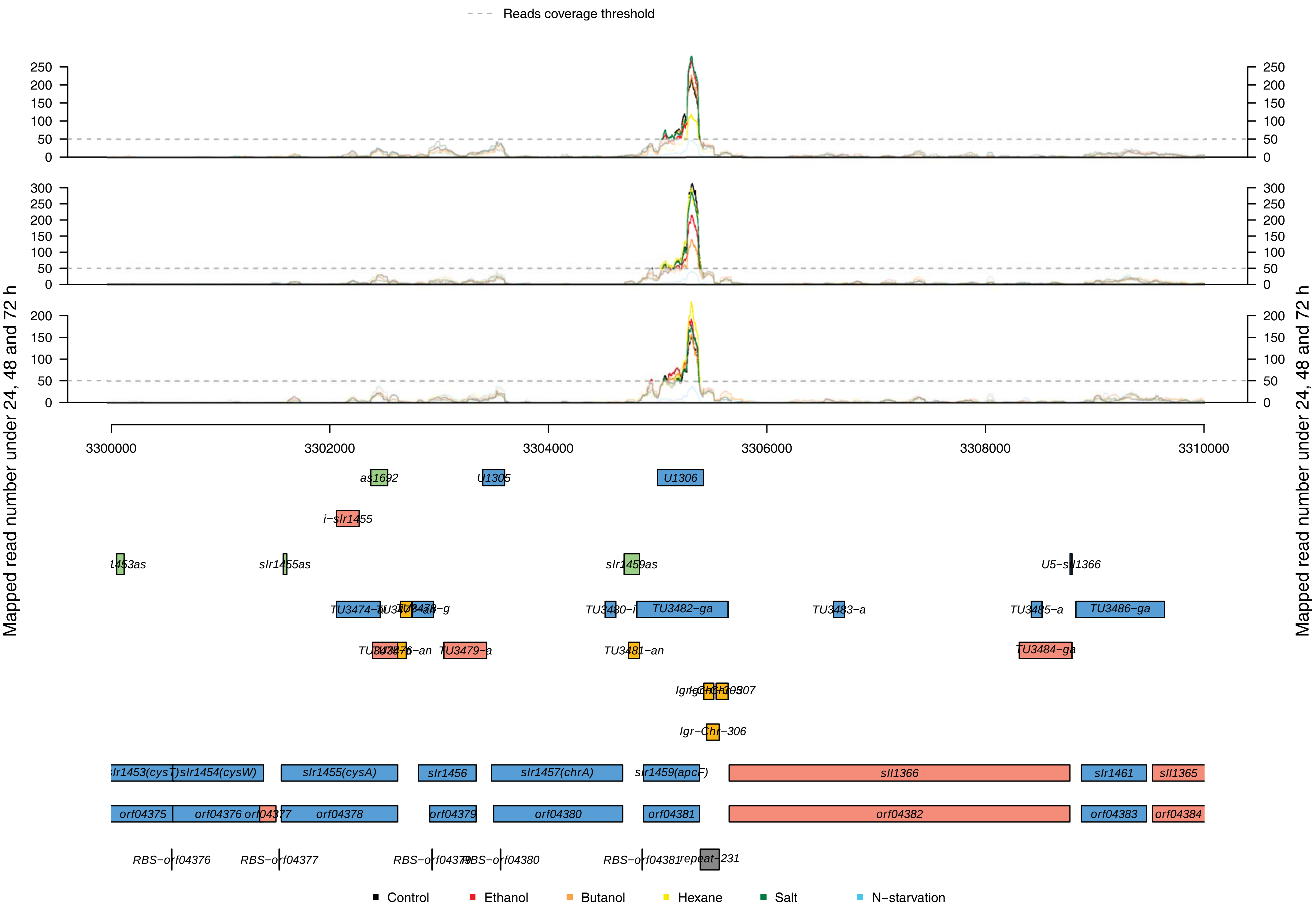

Reads coverage threshold
Mapped read number under 24, 48 and 72 h
3300000
3302000
3304000
3306000
3308000
3310000
as1692
U1305
U1306
i−slr1455
slr1455as
slr1459as
TU3474−a
TU3482−ga
TU3483−a
TU3485−a
TU3486−ga
TU3479−a
TU3481−an
TU3484−ga
Igr−Chr−306
slr1454(cysW)
slr1455(cysA)
slr1456
slr1457(chrA)
slr1459(apcF)
sll1366
slr1461
sll1365
orf04375
orf04376
orf04378
orf04379
orf04380
orf04381
orf04382
orf04383
orf04384
RBS−orf04376
RBS−orf04377
RBS−orf04380
RBS−orf04381
repeat−231
Control
Ethanol
Butanol
Hexane
Salt
N−starvation

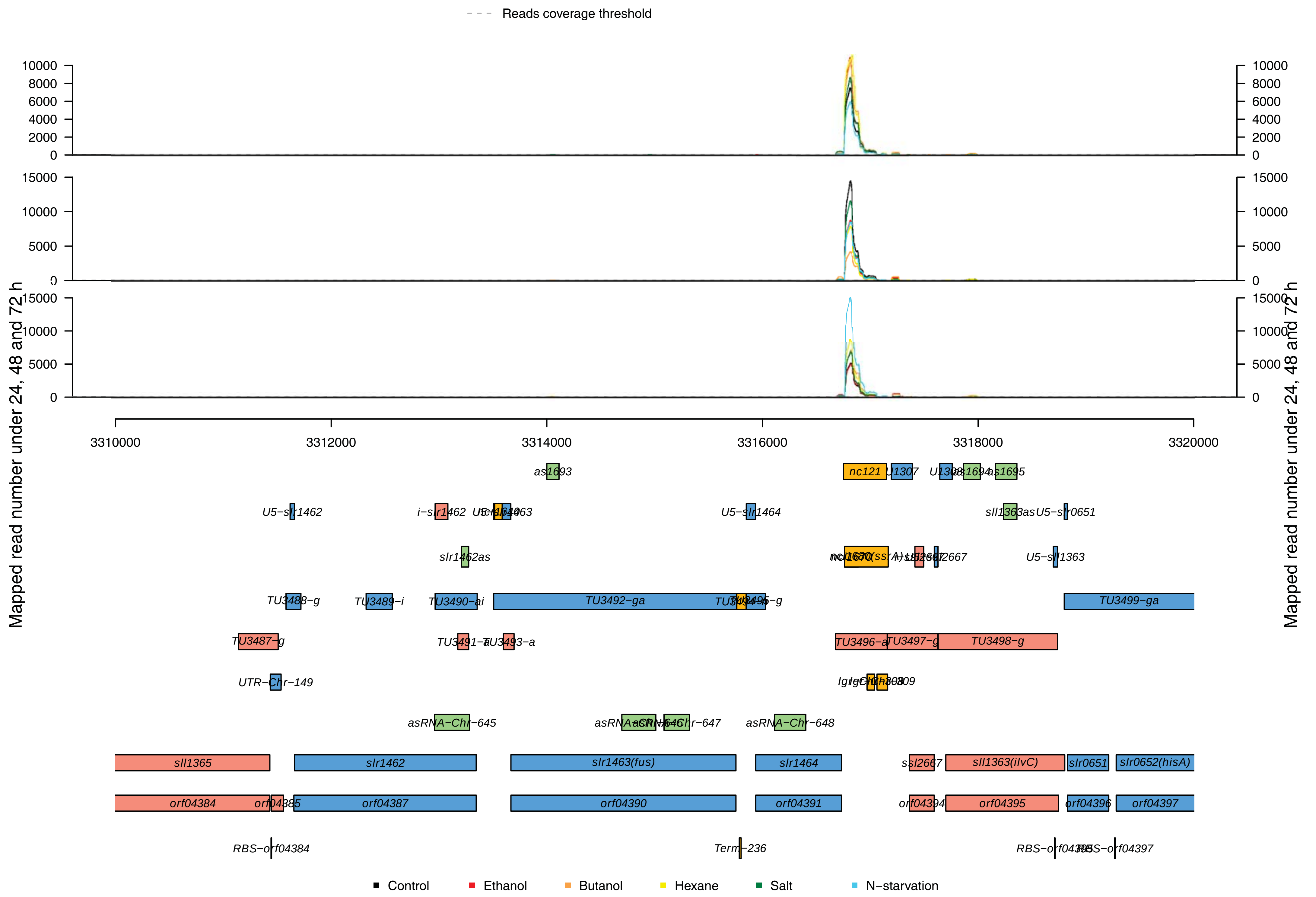

Reads coverage threshold
Mapped read number under 24, 48 and 72 h
3310000
3312000
3314000
3316000
3318000
3320000
nc121
sll1363(ilvC)
slr0651
slr0652(hisA)
sll1365
slr1462
slr1463(fus)
slr1464
ssl2667
orf04384
orf04387
orf04390
orf04391
orf04395
orf04397
TU3492−ga
TU3499−ga
TU3498−g
TU3497−g
TU3496−a
TU3487−g
TU3488−g
TU3489−i
TU3490−ai
UTR−Chr−149
asRNA−Chr−645
asRNA−Chr−648
slr1462as
U5−slr1462
U5−slr1464
U5−sll1363
as1693
as1695
RBS−orf04384
Term−236
Control
Ethanol
Butanol
Hexane
Salt
N−starvation

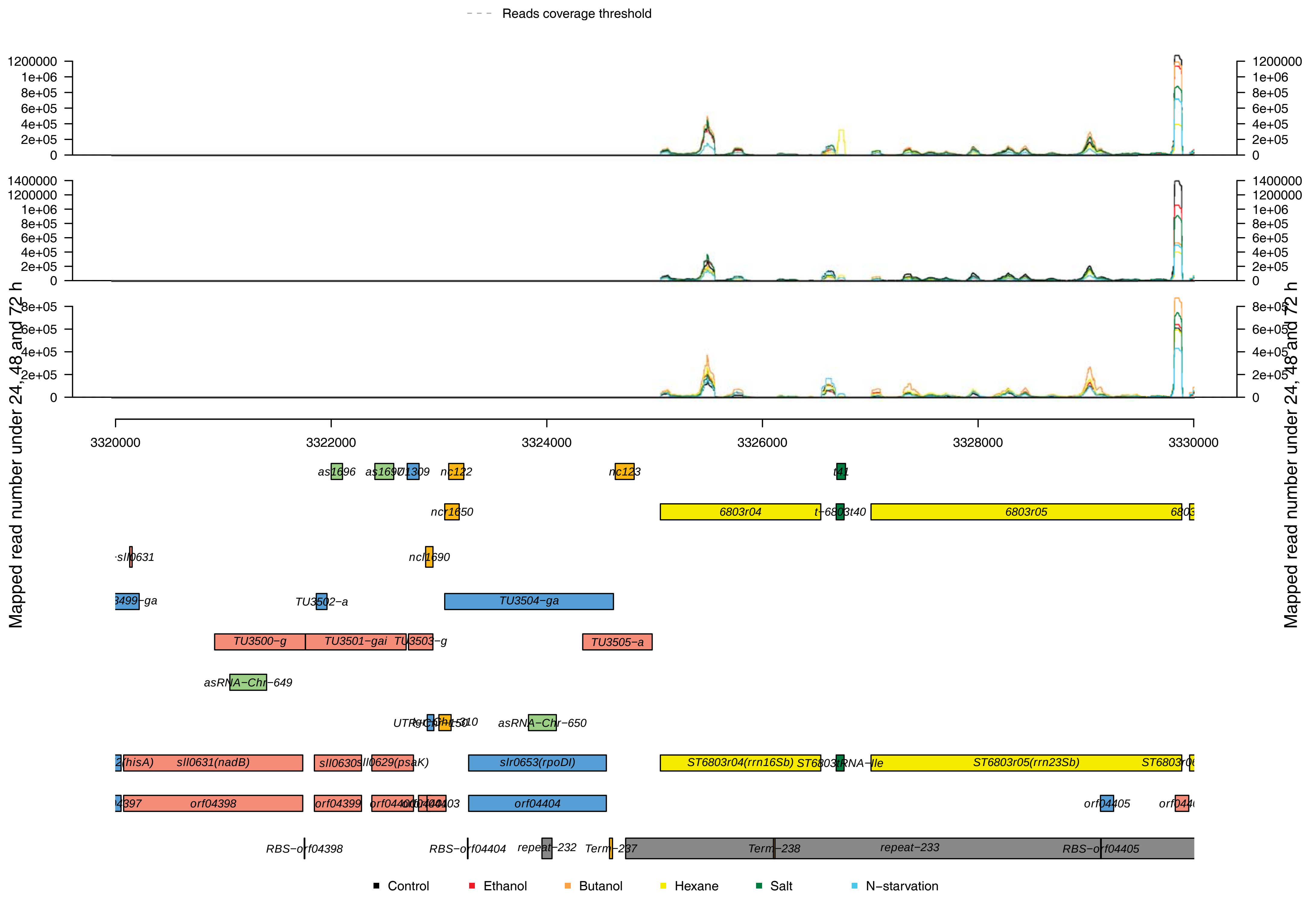

Reads coverage threshold
Mapped read number under 24, 48 and 72 h
3320000
3322000
3324000
3326000
3328000
3330000
6803r04
6803r05
TU3504−ga
TU3500−g
TU3505−a
asRNA−Chr−649
asRNA−Chr−650
sll0631(nadB)
slr0653(rpoDI)
ST6803r04(rrn16Sb)
ST6803r05(rrn23Sb)
orf04398
orf04399
orf04404
RBS−orf04398
RBS−orf04404
RBS−orf04405
repeat−233
Control
Ethanol
Butanol
Hexane
Salt
N−starvation

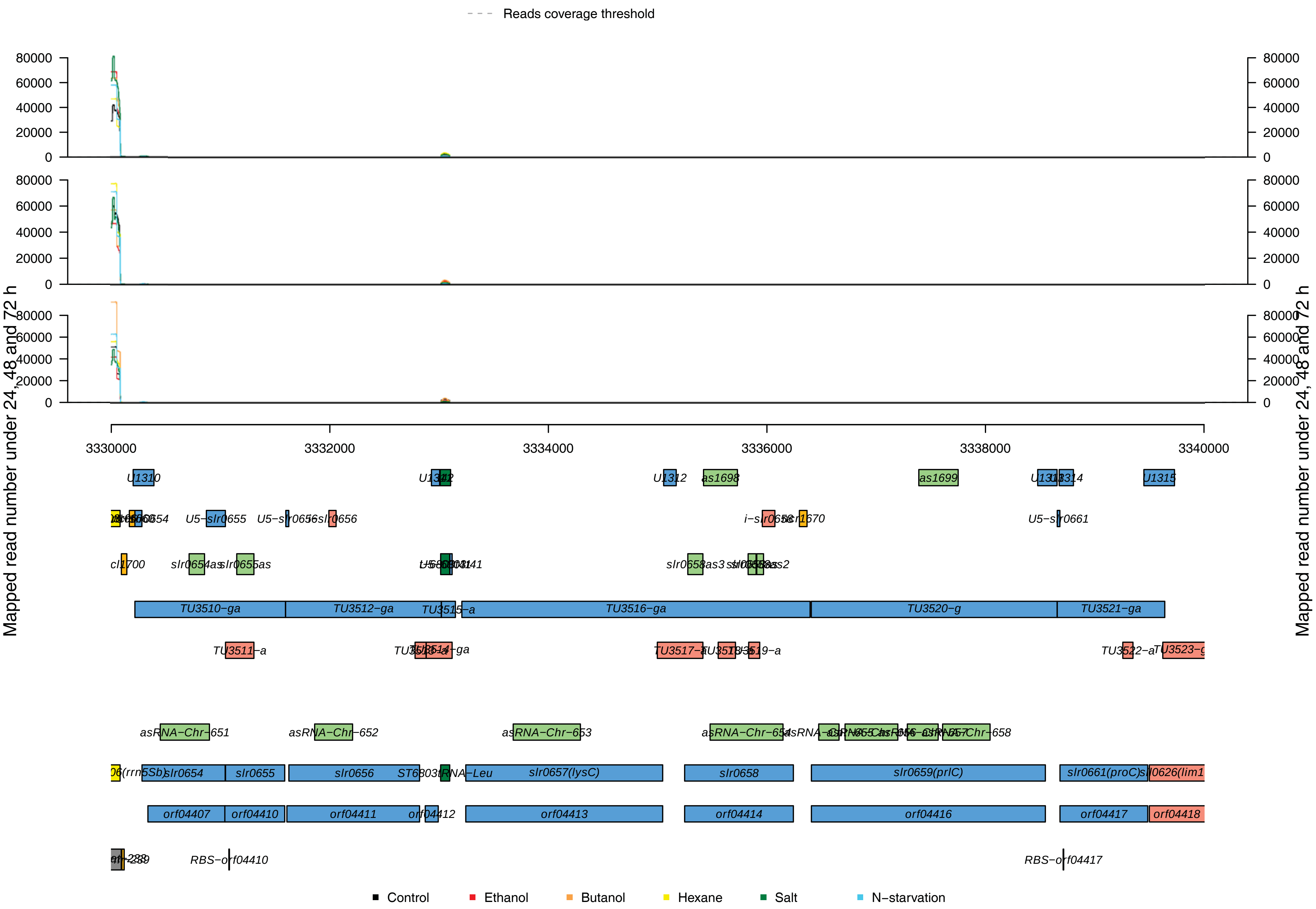
Reads coverage threshold
Mapped read number under 24, 48 and 72 h
3330000
3332000
3334000
3336000
3338000
3340000
TU3510−ga
TU3512−ga
TU3516−ga
TU3520−g
TU3521−ga
asRNA−Chr−651
asRNA−Chr−652
asRNA−Chr−653
slr0654
slr0655
slr0656
slr0657(lysC)
slr0658
slr0659(prlC)
slr0661(proC)
orf04407
orf04410
orf04411
orf04413
orf04414
orf04416
orf04417
orf04418
RBS−orf04410
RBS−orf04417
Control
Ethanol
Butanol
Hexane
Salt
N−starvation

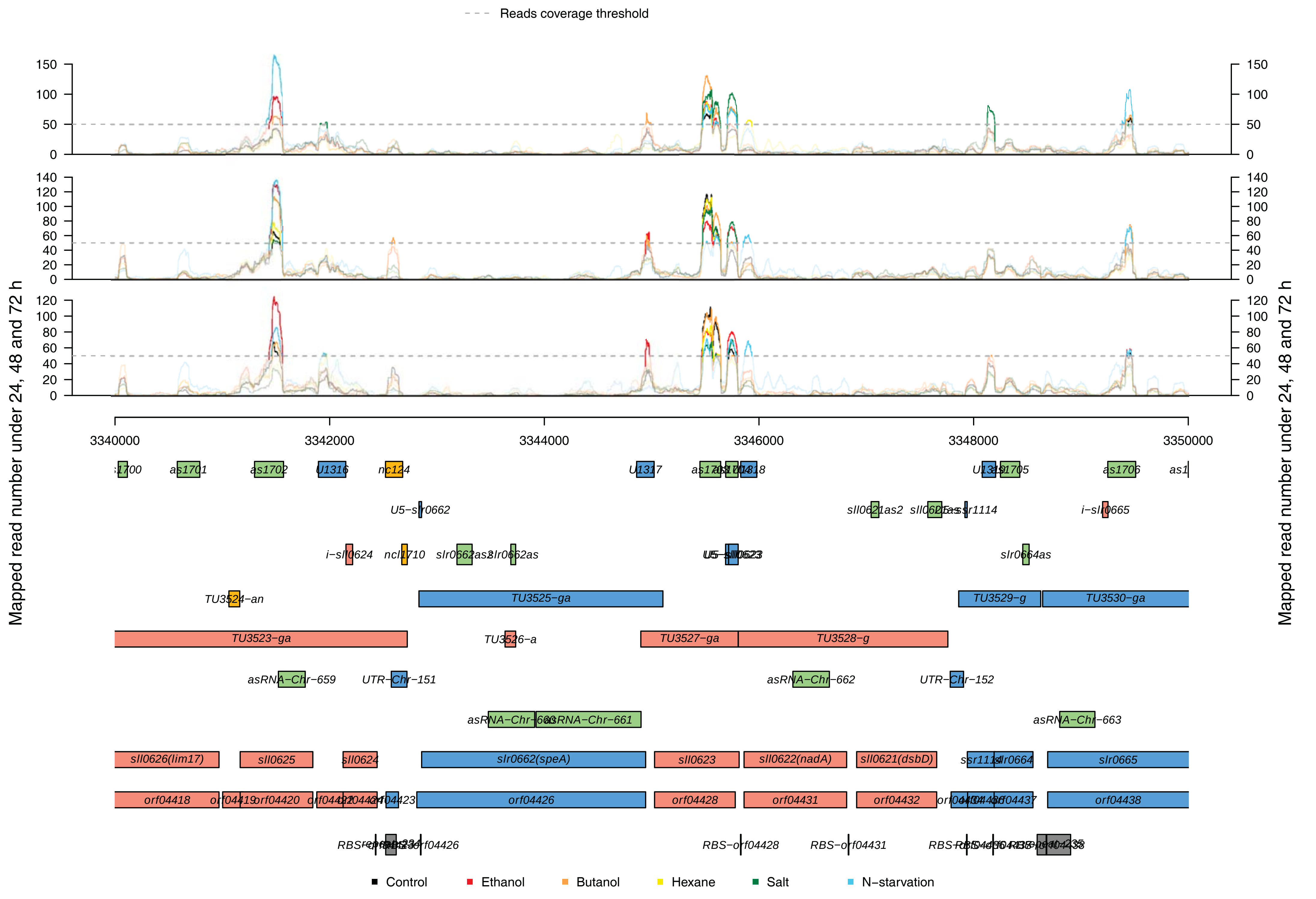

Reads coverage threshold
Mapped read number under 24, 48 and 72 h
150
100
50
0
140
120
100
80
60
40
20
0
120
100
80
60
40
20
0
3340000
3342000
3344000
3346000
3348000
3350000
as1701
as1702
U1316
nc124
U1317
as1705
as1706
U5−slr0662
sll0621as2
i−sll0665
i−sll0624
ncl1710
slr0662as
slr0664as
TU3524−an
TU3525−ga
TU3529−g
TU3530−ga
TU3523−ga
TU3526−a
TU3527−ga
TU3528−g
asRNA−Chr−659
UTR−Chr−151
asRNA−Chr−662
UTR−Chr−152
asRNA−Chr−661
asRNA−Chr−663
sll0626(lim17)
sll0625
sll0624
slr0662(speA)
sll0623
sll0622(nadA)
sll0621(dsbD)
slr0665
orf04418
orf04420
orf04423
orf04426
orf04428
orf04431
orf04432
orf04437
orf04438
RBS−orf04426
RBS−orf04428
RBS−orf04431
Control
Ethanol
Butanol
Hexane
Salt
N−starvation

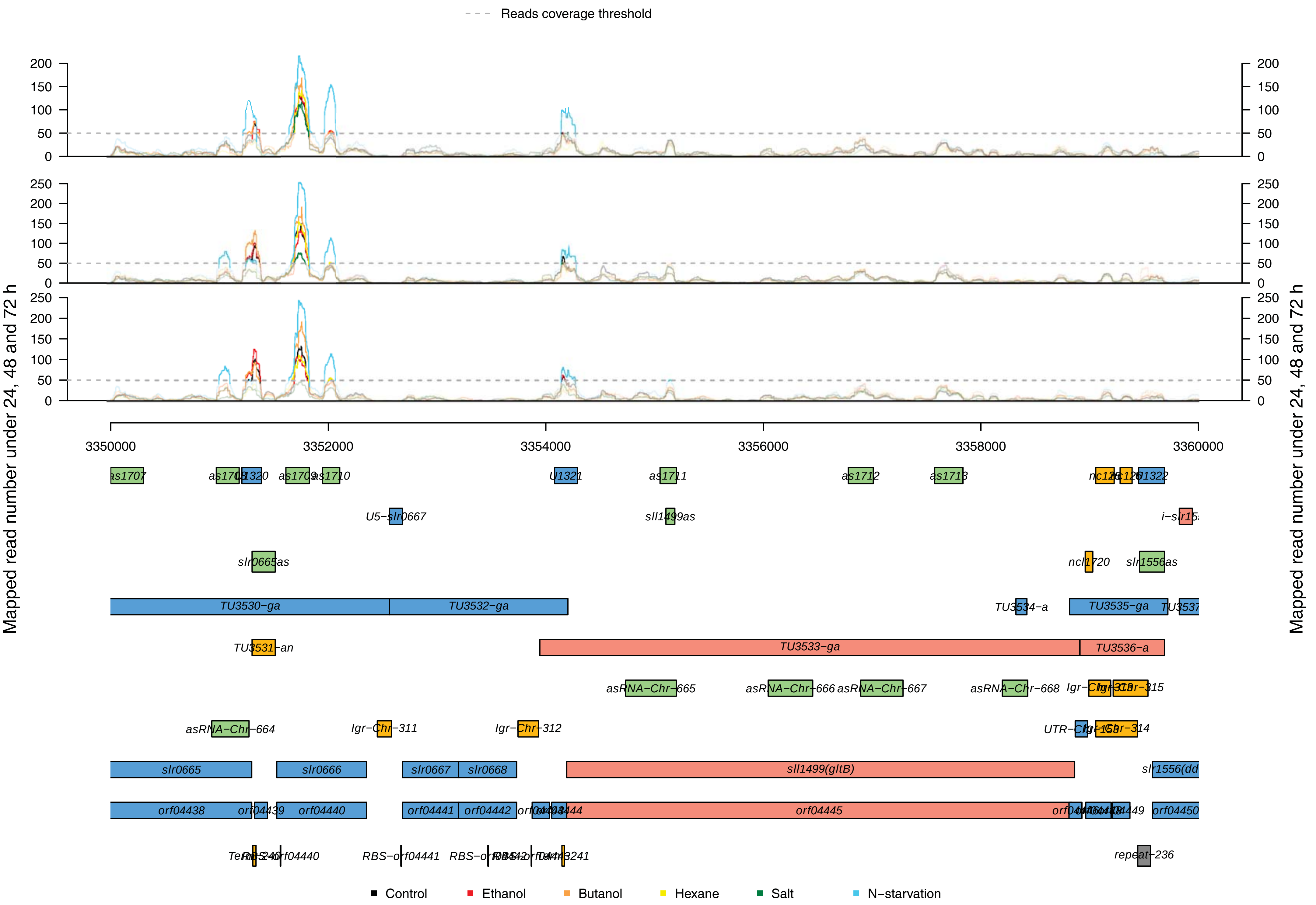

Reads coverage threshold
Mapped read number under 24, 48 and 72 h
3350000
3352000
3354000
3356000
3358000
3360000
TU3530−ga
TU3532−ga
TU3533−ga
TU3536−a
TU3535−ga
TU3534−a
TU3531−an
sll1499(gltB)
slr0665
slr0666
slr0667
slr0668
orf04438
orf04440
orf04441
orf04442
orf04445
orf04450
asRNA−Chr−664
asRNA−Chr−665
asRNA−Chr−666
asRNA−Chr−667
asRNA−Chr−668
Igr−Chr−311
Igr−Chr−312
Igr−Chr−314
Igr−Chr−315
U5−slr0667
sll1499as
slr0665as
slr1556as
ncl1720
U1321
as1711
as1712
as1713
as1710
RBS−orf04441
repeat−236
Control
Ethanol
Butanol
Hexane
Salt
N−starvation

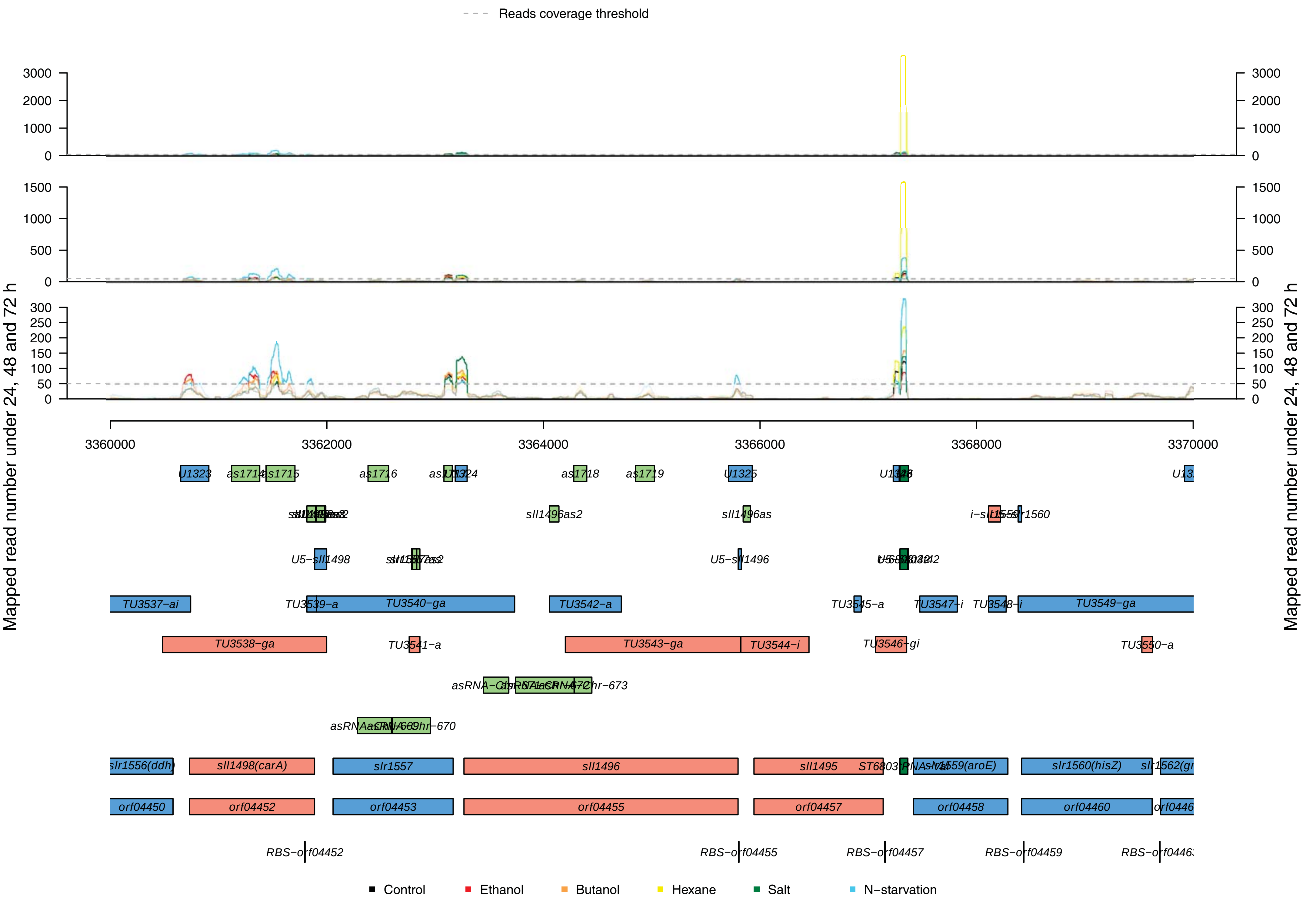

Reads coverage threshold
Mapped read number under 24, 48 and 72 h
3360000
3362000
3364000
3366000
3368000
3370000
TU3537−ai
TU3538−ga
TU3540−ga
TU3542−a
TU3543−ga
TU3544−i
TU3549−ga
slr1556(ddh)
sll1498(carA)
slr1557
sll1496
sll1495
slr1559(aroE)
slr1560(hisZ)
orf04450
orf04452
orf04453
orf04455
orf04457
orf04458
orf04460
RBS−orf04452
RBS−orf04455
RBS−orf04457
RBS−orf04459
Control
Ethanol
Butanol
Hexane
Salt
N−starvation

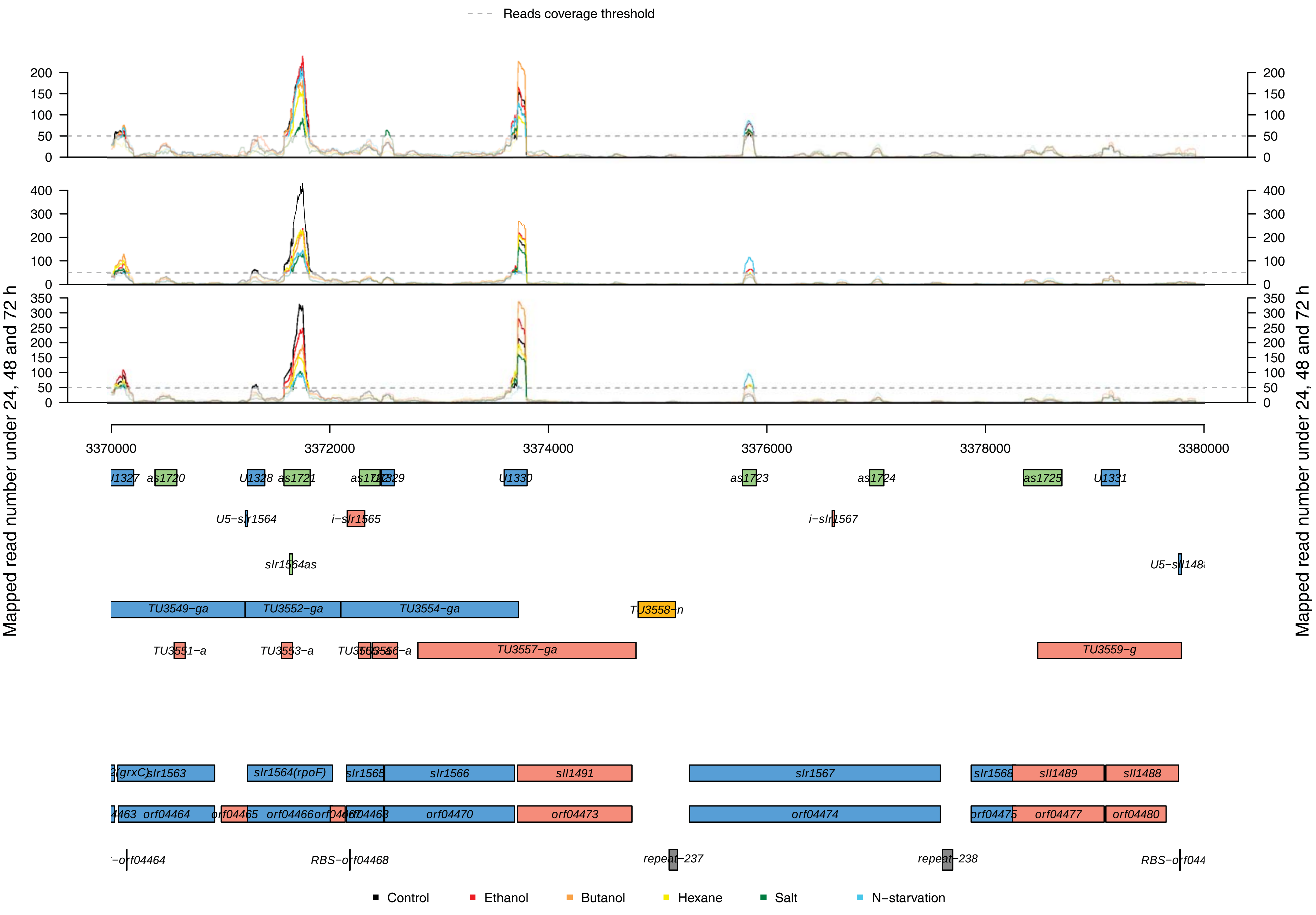

Reads coverage threshold
Mapped read number under 24, 48 and 72 h
3370000
3372000
3374000
3376000
3378000
3380000
as1720
as1721
as1723
as1724
as1725
U5−slr1564
i−slr1565
i−slr1567
slr1564as
TU3549−ga
TU3552−ga
TU3554−ga
TU3551−a
TU3553−a
TU3557−ga
TU3559−g
slr1563
slr1564(rpoF)
slr1566
sll1491
slr1567
sll1489
sll1488
orf04464
orf04470
orf04473
orf04474
orf04477
orf04480
RBS−orf04468
repeat−237
repeat−238
Control
Ethanol
Butanol
Hexane
Salt
N−starvation

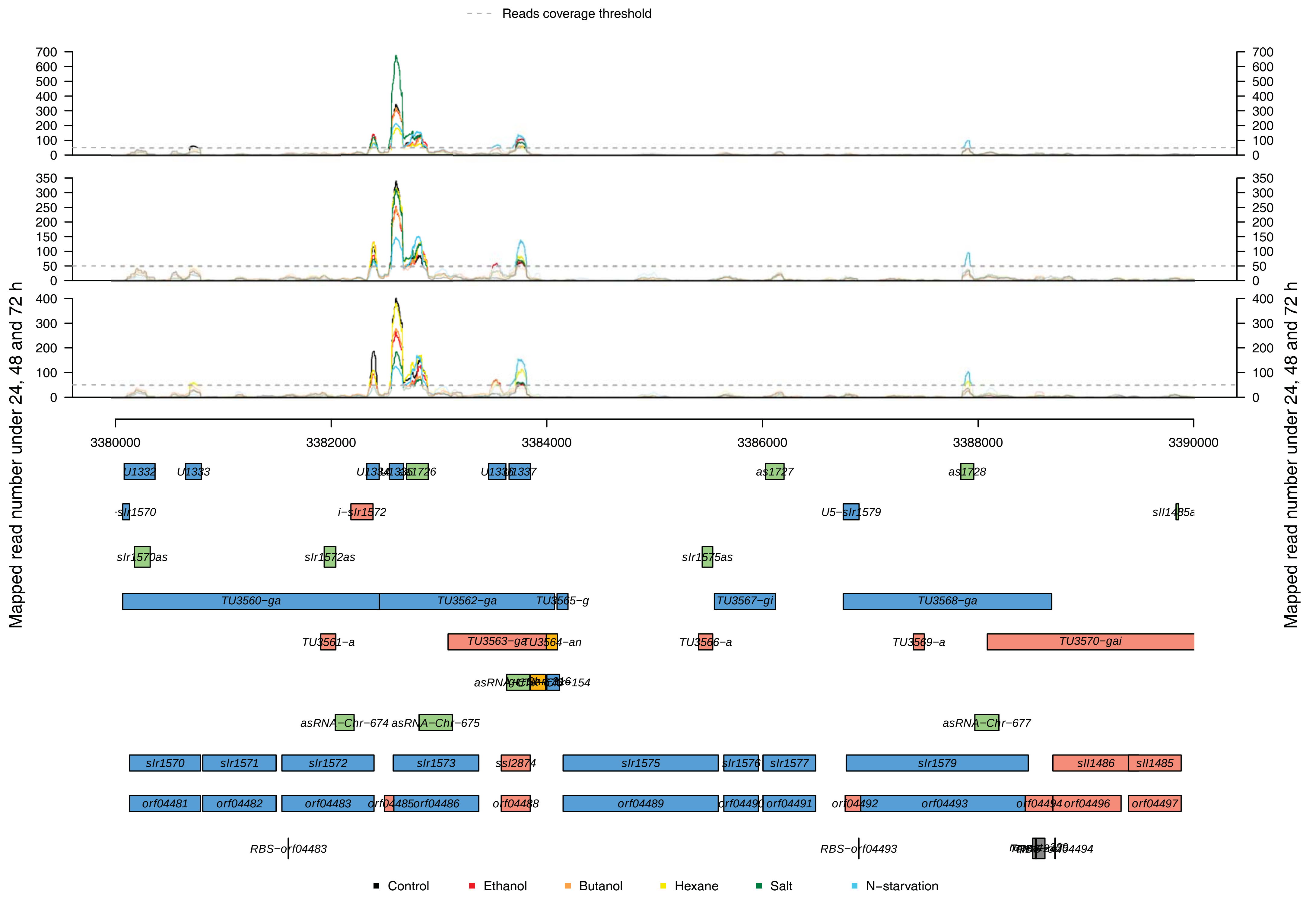

Reads coverage threshold
Mapped read number under 24, 48 and 72 h
3380000
3382000
3384000
3386000
3388000
3390000
U1332
U1333
as1726
as1727
as1728
i-slr1572
U5-slr1579
slr1570as
slr1572as
slr1575as
TU3560-ga
TU3562-ga
TU3567-gi
TU3568-ga
TU3561-a
TU3566-a
TU3569-a
TU3570-gai
asRNA-Chr-674
asRNA-Chr-675
asRNA-Chr-677
slr1570
slr1571
slr1572
slr1573
ssl2874
slr1575
slr1576
slr1577
slr1579
sll1486
sll1485
orf04481
orf04482
orf04483
orf04486
orf04488
orf04489
orf04491
orf04493
orf04496
orf04497
RBS-orf04483
RBS-orf04493
Control
Ethanol
Butanol
Hexane
Salt
N-starvation

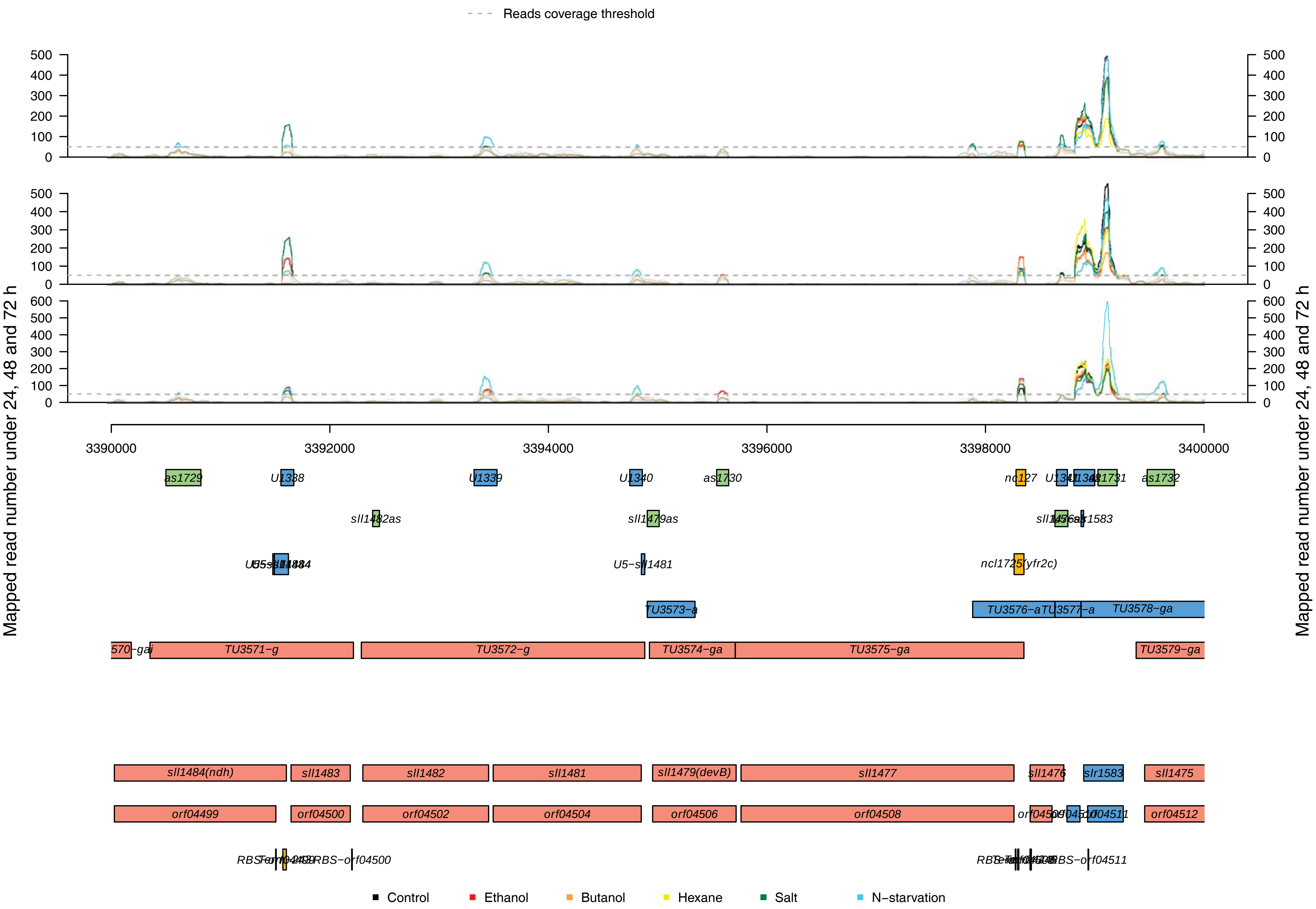

Reads coverage threshold
Mapped read number under 24, 48 and 72 h
3390000
3392000
3394000
3396000
3398000
3400000
as1729
U1338
U1339
U1340
as1730
as1732
sll1482as
sll1479as
U5−sll1481
ncl1725(yfr2c)
TU3573−a
TU3576−a
TU3578−ga
TU3571−g
TU3572−g
TU3574−ga
TU3575−ga
TU3579−ga
sll1484(ndh)
sll1483
sll1482
sll1481
sll1479(devB)
sll1477
sll1476
slr1583
sll1475
orf04499
orf04500
orf04502
orf04504
orf04506
orf04508
orf04511
orf04512
RBS−orf04500
RBS−orf04511
Control
Ethanol
Butanol
Hexane
Salt
N−starvation

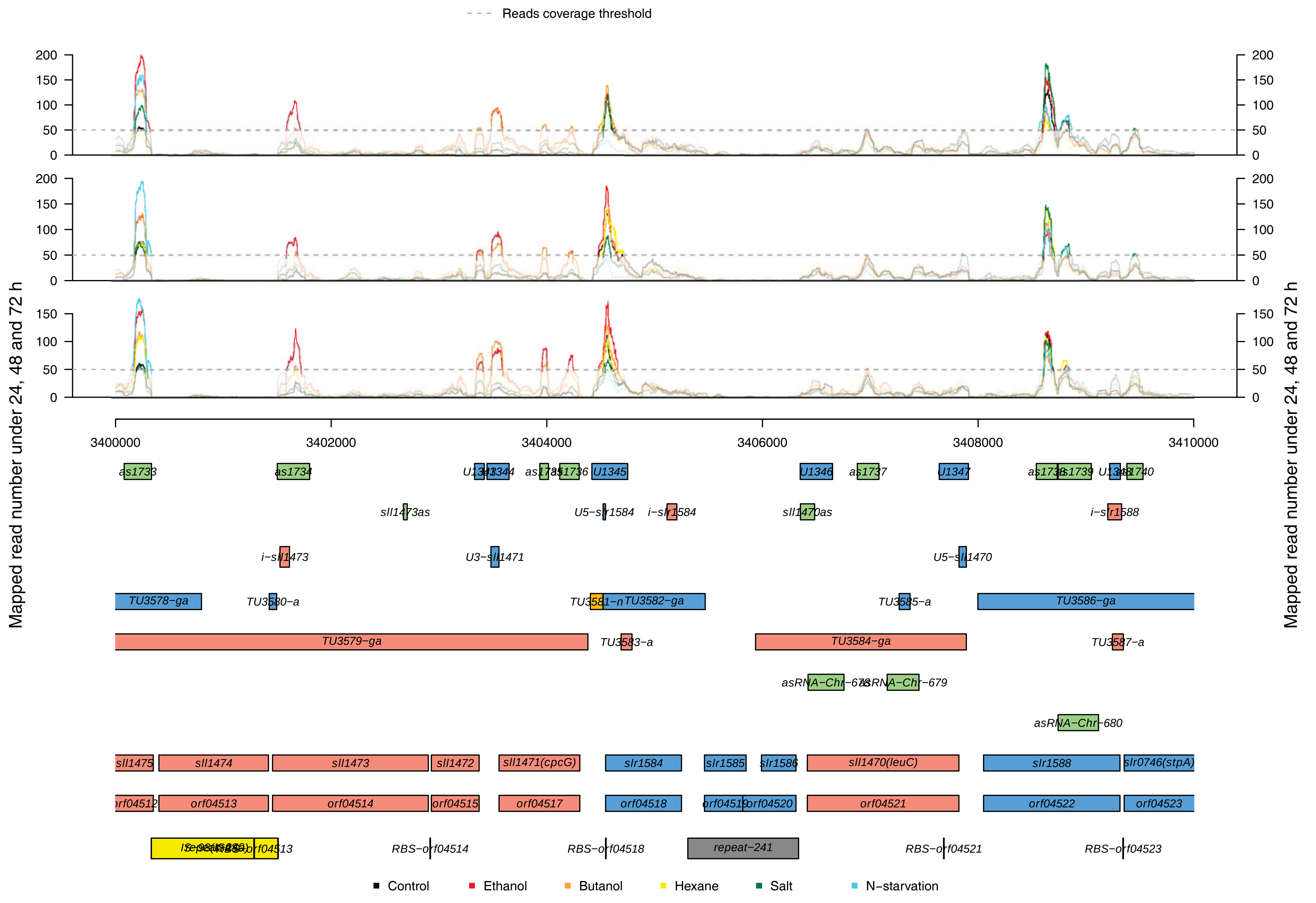

Reads coverage threshold
Mapped read number under 24, 48 and 72 h
3400000
3402000
3404000
3406000
3408000
3410000
as1733
as1734
U1343
U1344
as1735
as1736
U1345
U1346
as1737
U1347
as1738
as1739
U1348
as1740
sll1473as
U5−slr1584
i−slr1584
sll1470as
i−slr1588
i−sll1473
U3−sll1471
U5−sll1470
TU3578−ga
TU3580−a
TU3581−n
TU3582−ga
TU3585−a
TU3586−ga
TU3579−ga
TU3583−a
TU3584−ga
TU3587−a
asRNA−Chr−678
asRNA−Chr−679
asRNA−Chr−680
sll1475
sll1474
sll1473
sll1472
sll1471(cpcG)
slr1584
slr1585
slr1586
sll1470(leuC)
slr1588
slr0746(stpA)
orf04512
orf04513
orf04514
orf04515
orf04517
orf04518
orf04519
orf04520
orf04521
orf04522
orf04523
RBS−orf04513
RBS−orf04514
RBS−orf04518
repeat−241
RBS−orf04521
RBS−orf04523
Control
Ethanol
Butanol
Hexane
Salt
N−starvation

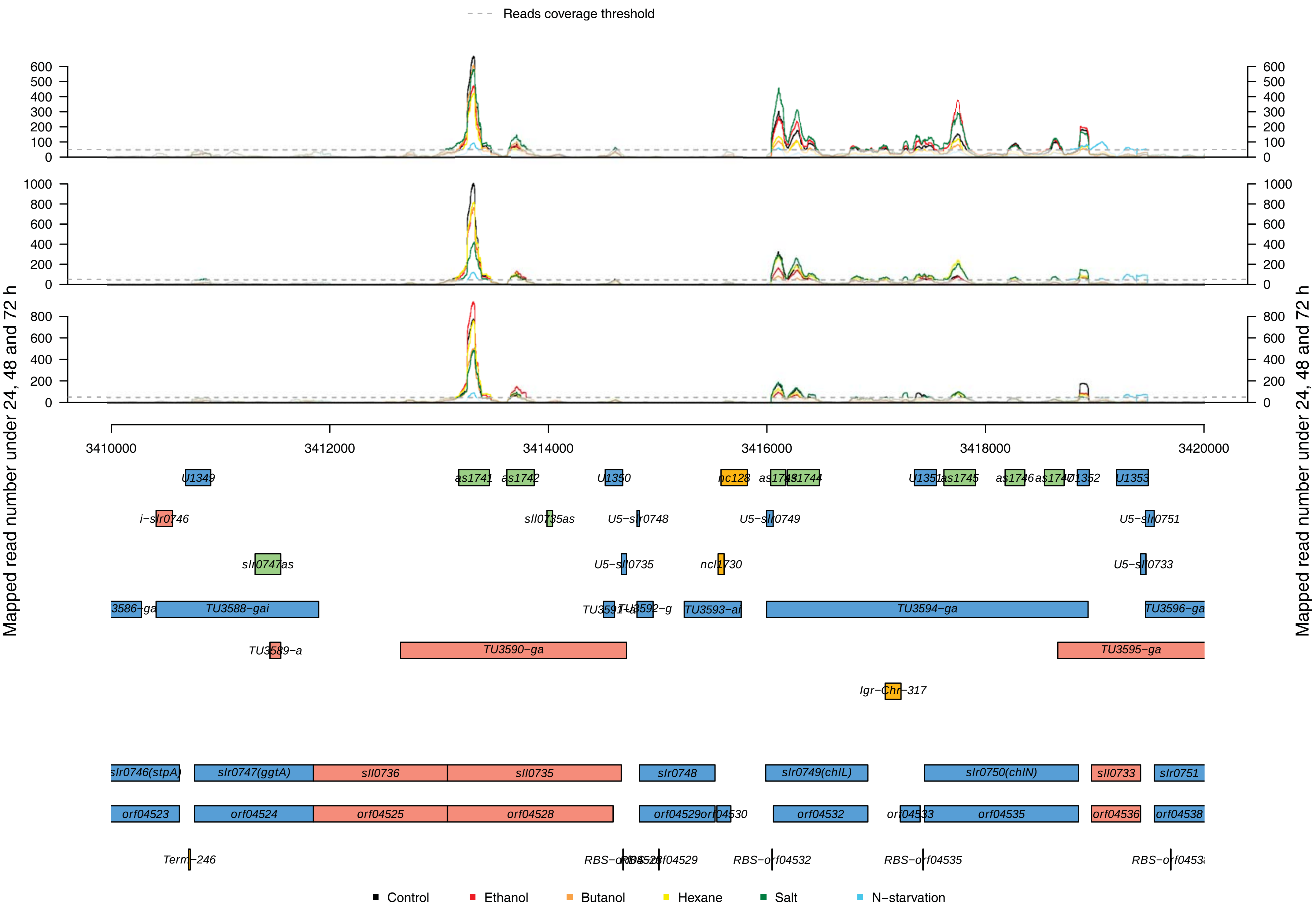

Reads coverage threshold
Mapped read number under 24, 48 and 72 h
3410000
3412000
3414000
3416000
3418000
3420000
U1349
as1741
as1742
U1350
nc128
U1351
as1745
U1353
i−slr0746
sll0735as
U5−slr0748
U5−slr0749
U5−slr0751
slr0747as
U5−sll0735
ncl1730
U5−sll0733
TU3588−gai
TU3593−ai
TU3594−ga
TU3590−ga
TU3595−ga
TU3589−a
Igr−Chr−317
slr0746(stpA)
slr0747(ggtA)
sll0736
sll0735
slr0748
slr0749(chlL)
slr0750(chlN)
sll0733
slr0751
orf04523
orf04524
orf04525
orf04528
orf04532
orf04535
orf04536
orf04538
Term−246
RBS−orf04532
RBS−orf04535
Control
Ethanol
Butanol
Hexane
Salt
N−starvation

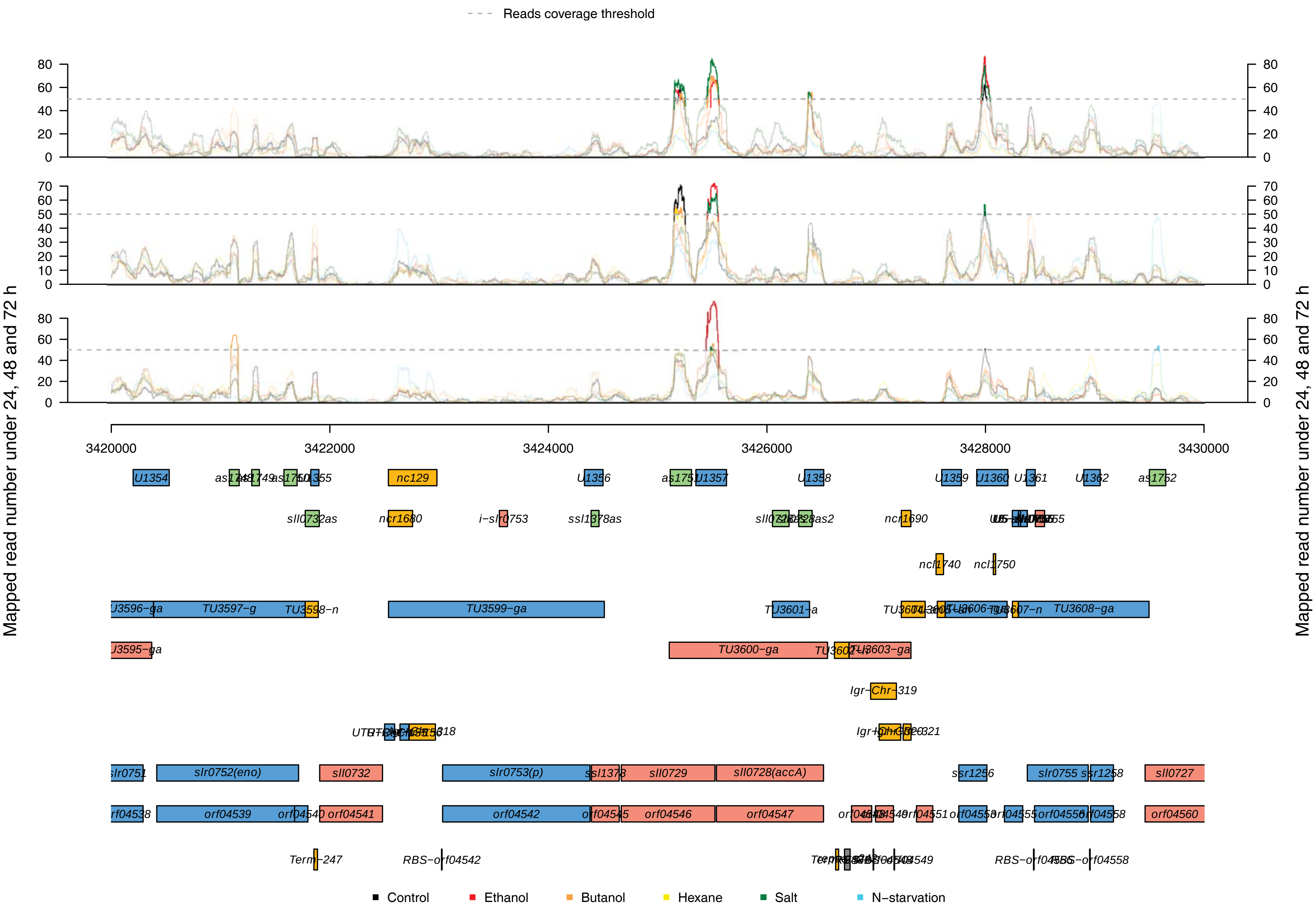

Reads coverage threshold
Mapped read number under 24, 48 and 72 h
3420000
3422000
3424000
3426000
3428000
3430000
nc129
ncr1680
i−slr0753
ssl1378as
sll0732as
ncl1690
ncl1740
ncl1750
TU3597−g
TU3599−ga
TU3600−ga
TU3608−ga
Igr−Chr−319
slr0752(eno)
sll0732
slr0753(p)
sll0729
sll0728(accA)
ssr1256
sll0727
orf04539
orf04541
orf04542
orf04546
orf04547
orf04560
Term−247
RBS−orf04542
Control
Ethanol
Butanol
Hexane
Salt
N−starvation

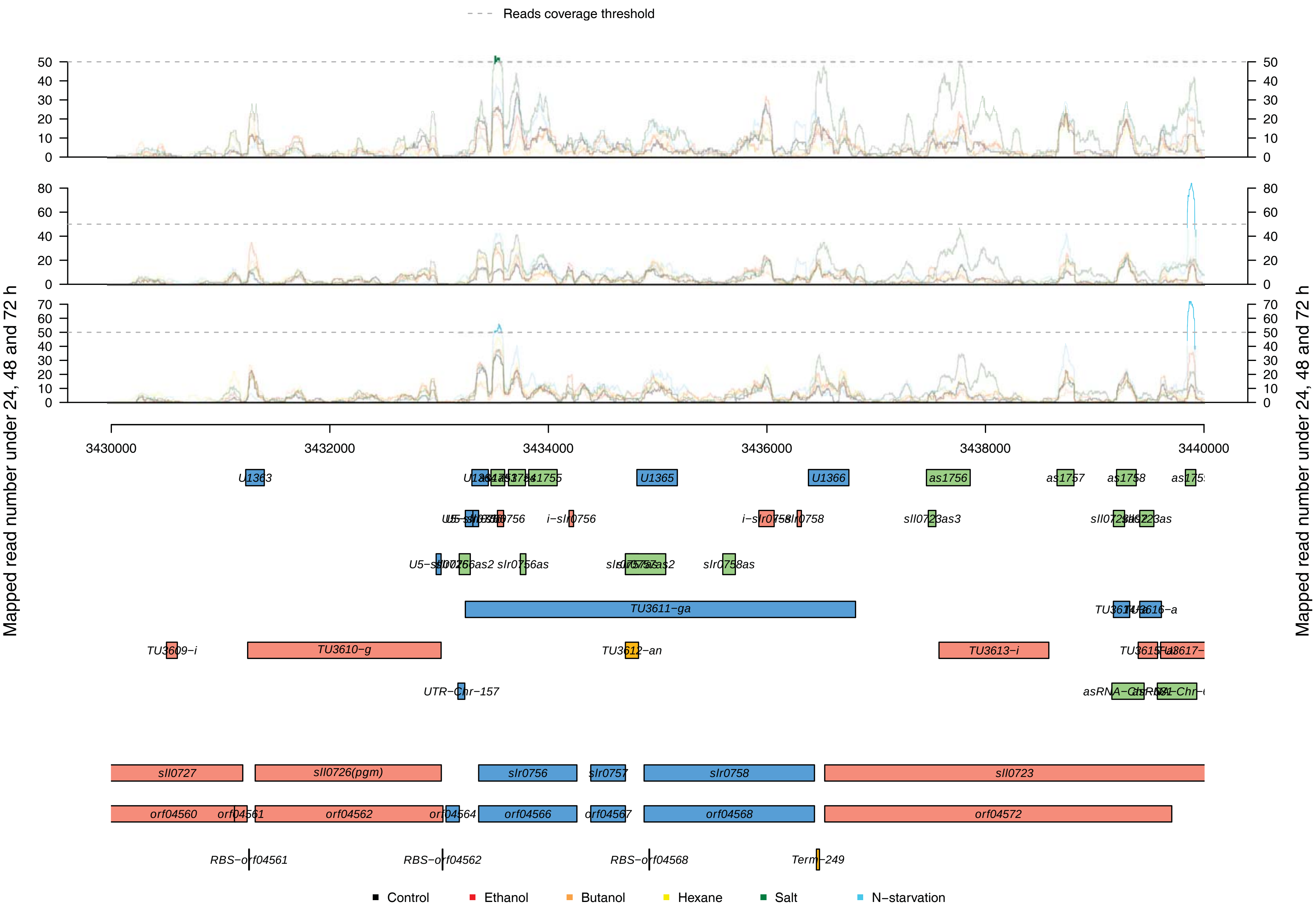

Reads coverage threshold
Mapped read number under 24, 48 and 72 h
3430000
3432000
3434000
3436000
3438000
3440000
U1363
U1365
U1366
as1756
as1757
as1758
i−slr0756
i−slr0758
slr0758
sll0723as3
slr0758as
TU3611−ga
TU3609−i
TU3610−g
TU3612−an
TU3613−i
UTR−Chr−157
sll0727
sll0726(pgm)
slr0756
slr0757
slr0758
sll0723
orf04560
orf04562
orf04566
orf04567
orf04568
orf04572
RBS−orf04561
RBS−orf04562
RBS−orf04568
Term−249
Control
Ethanol
Butanol
Hexane
Salt
N−starvation

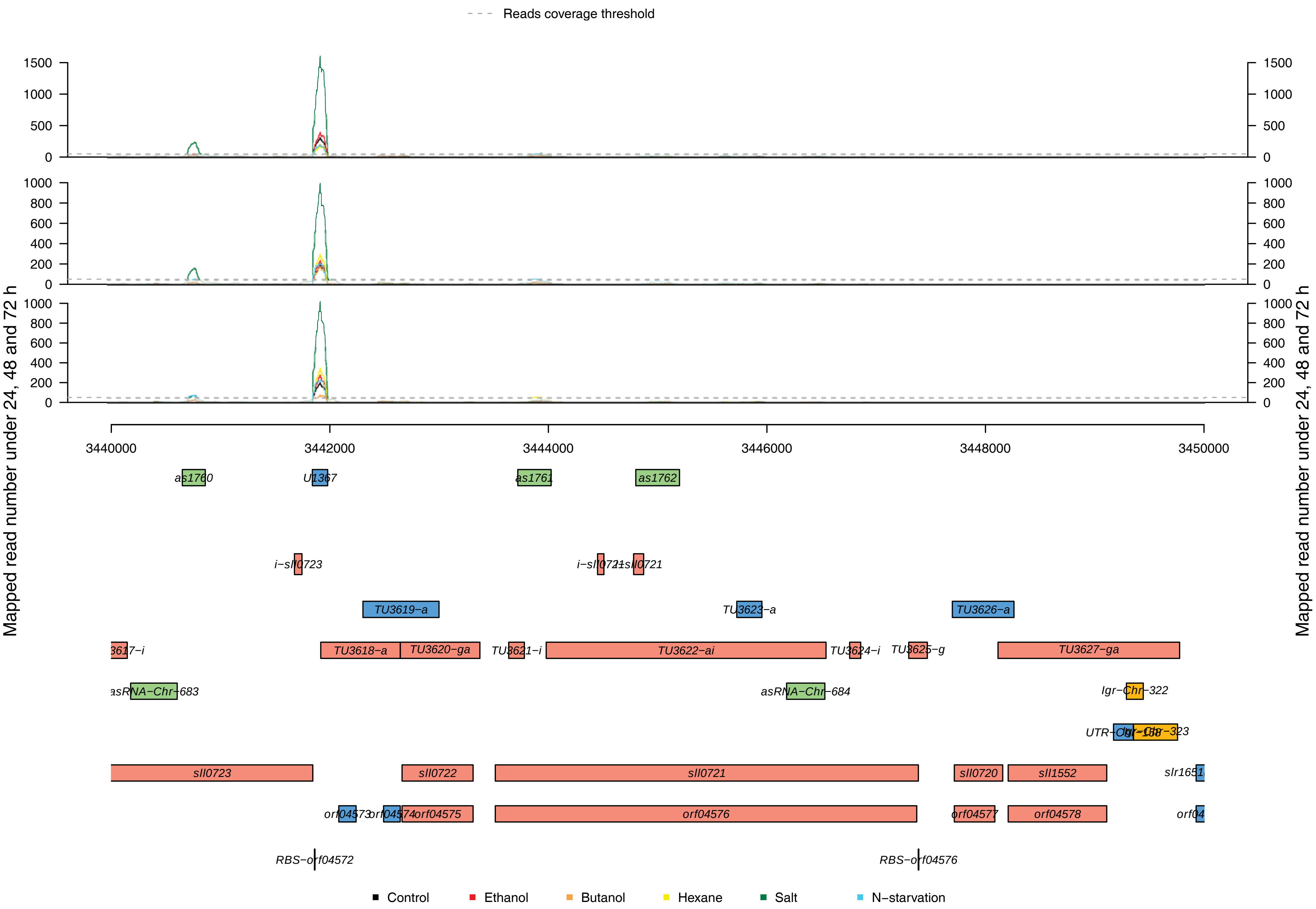

Reads coverage threshold
Mapped read number under 24, 48 and 72 h
1500
1000
500
0
800
600
400
200
3440000
3442000
3444000
3446000
3448000
3450000
as1760
U1367
as1761
as1762
i−sll0723
i−sll0721
i−sll0721
TU3619−a
TU3623−a
TU3626−a
3617−i
TU3618−a
TU3620−ga
TU3621−i
TU3622−ai
TU3624−i
TU3625−g
TU3627−ga
asRNA−Chr−683
asRNA−Chr−684
Igr−Chr−322
sll0723
sll0722
sll0721
sll0720
sll1552
orf04573
orf04574
orf04575
orf04576
orf04577
orf04578
RBS−orf04572
RBS−orf04576
Control
Ethanol
Butanol
Hexane
Salt
N−starvation

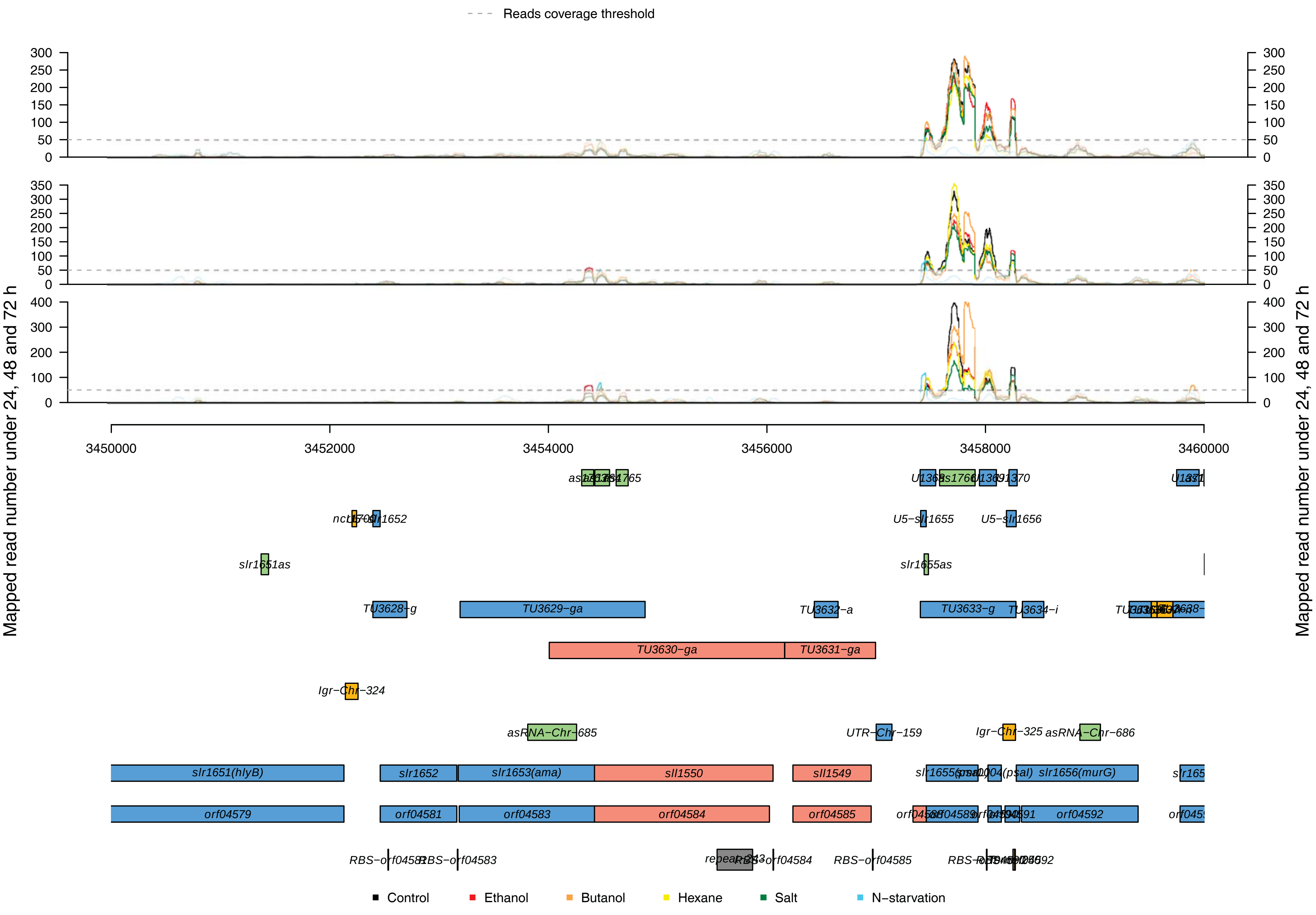

Reads coverage threshold
Mapped read number under 24, 48 and 72 h
3450000
3452000
3454000
3456000
3458000
3460000
TU3629−ga
TU3630−ga
TU3631−ga
TU3633−g
slr1651as
slr1655as
Igr−Chr−324
asRNA−Chr−685
UTR−Chr−159
asRNA−Chr−686
slr1651(hlyB)
slr1652
slr1653(ama)
sll1550
sll1549
slr1656(murG)
orf04579
orf04581
orf04583
orf04584
orf04585
orf04592
RBS−orf04585
Control
Ethanol
Butanol
Hexane
Salt
N−starvation

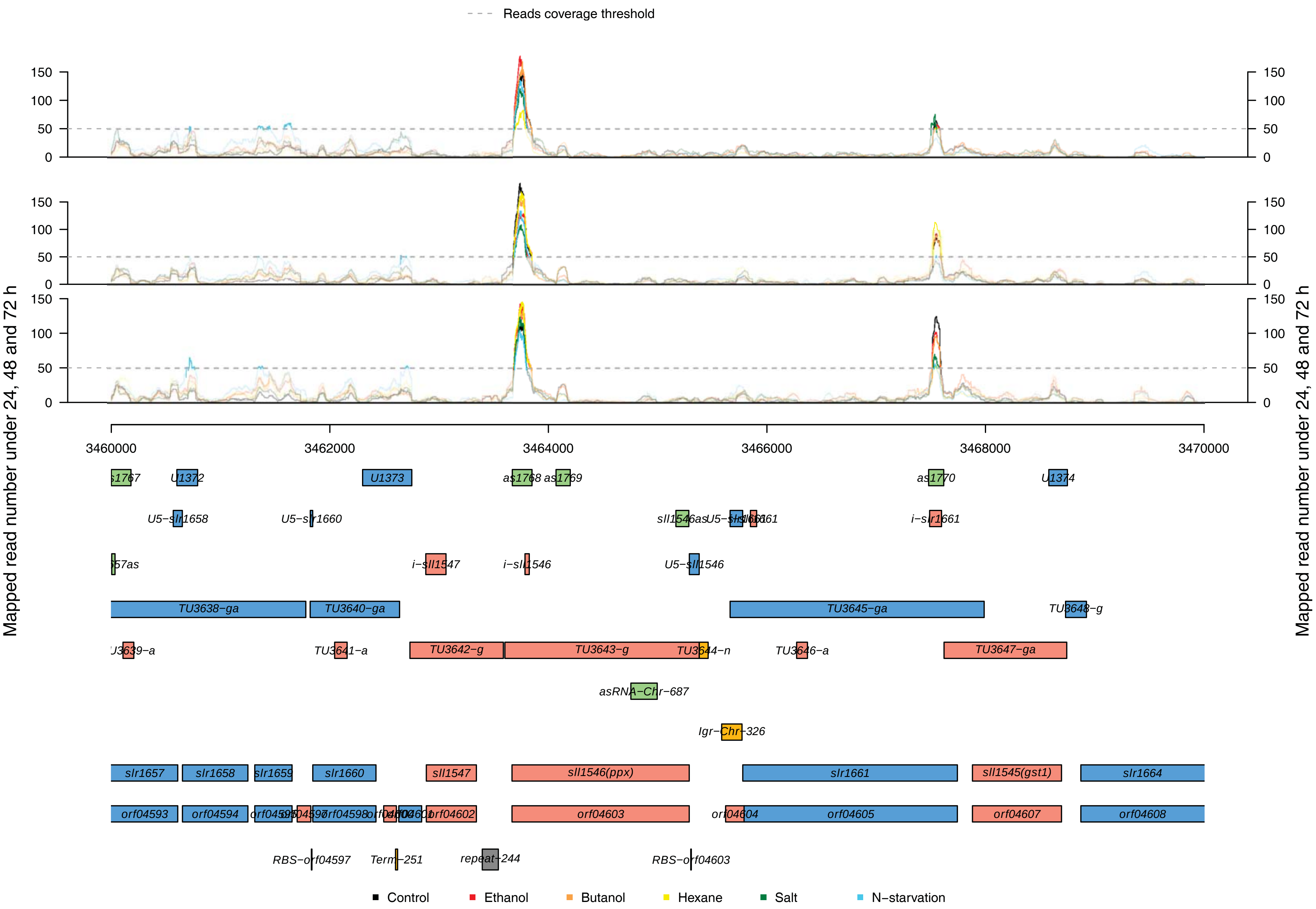

Reads coverage threshold
Mapped read number under 24, 48 and 72 h
3460000
3462000
3464000
3466000
3468000
3470000
TU3638−ga
TU3640−ga
TU3645−ga
TU3642−g
TU3643−g
TU3647−ga
asRNA−Chr−687
Igr−Chr−326
slr1657
slr1658
slr1660
sll1547
sll1546(ppx)
slr1661
sll1545(gst1)
slr1664
orf04593
orf04594
orf04603
orf04605
orf04607
orf04608
RBS−orf04597
RBS−orf04603
Control
Ethanol
Butanol
Hexane
Salt
N−starvation

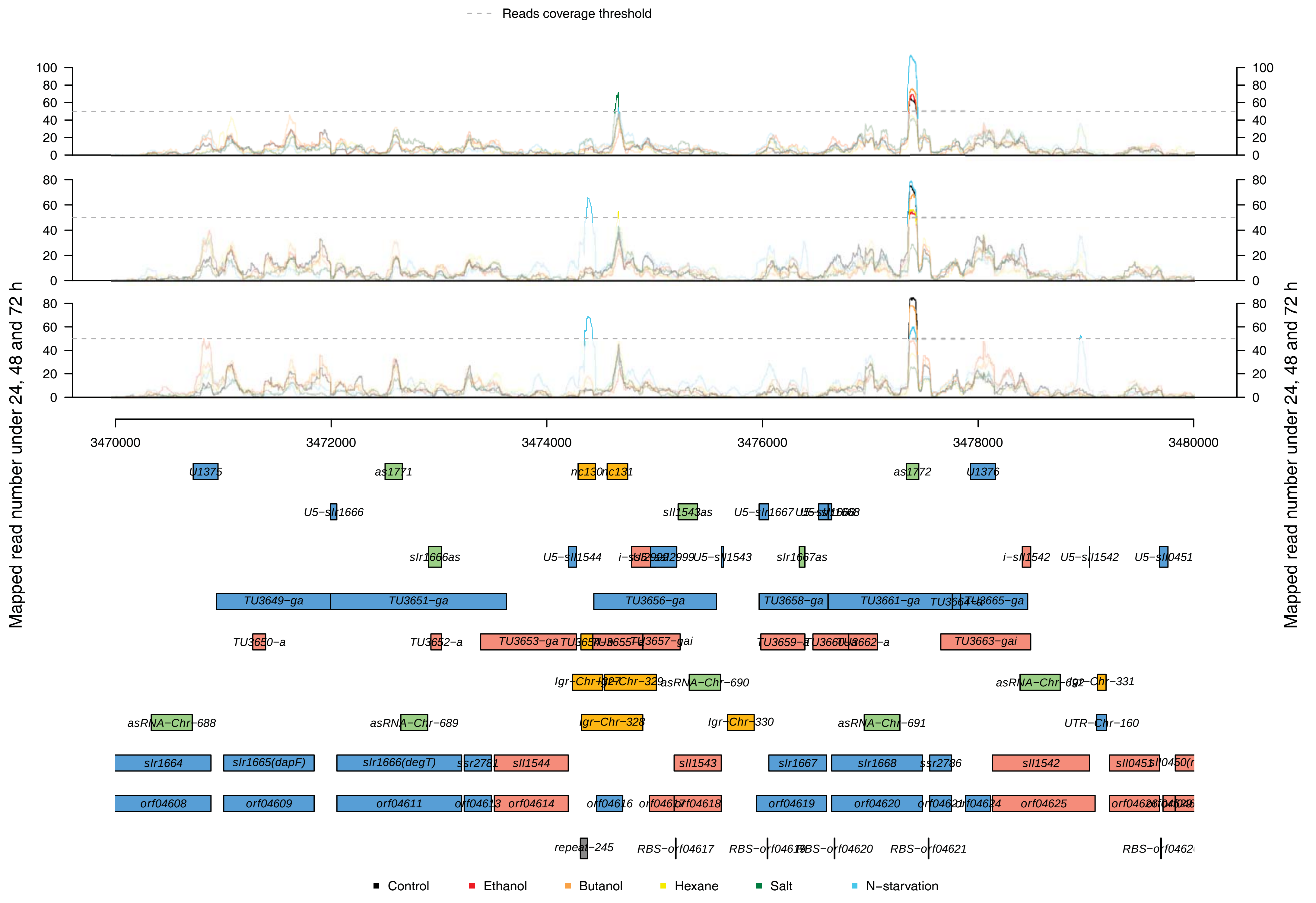

Reads coverage threshold
Mapped read number under 24, 48 and 72 h
3470000
3472000
3474000
3476000
3478000
3480000
Control
Ethanol
Butanol
Hexane
Salt
N−starvation

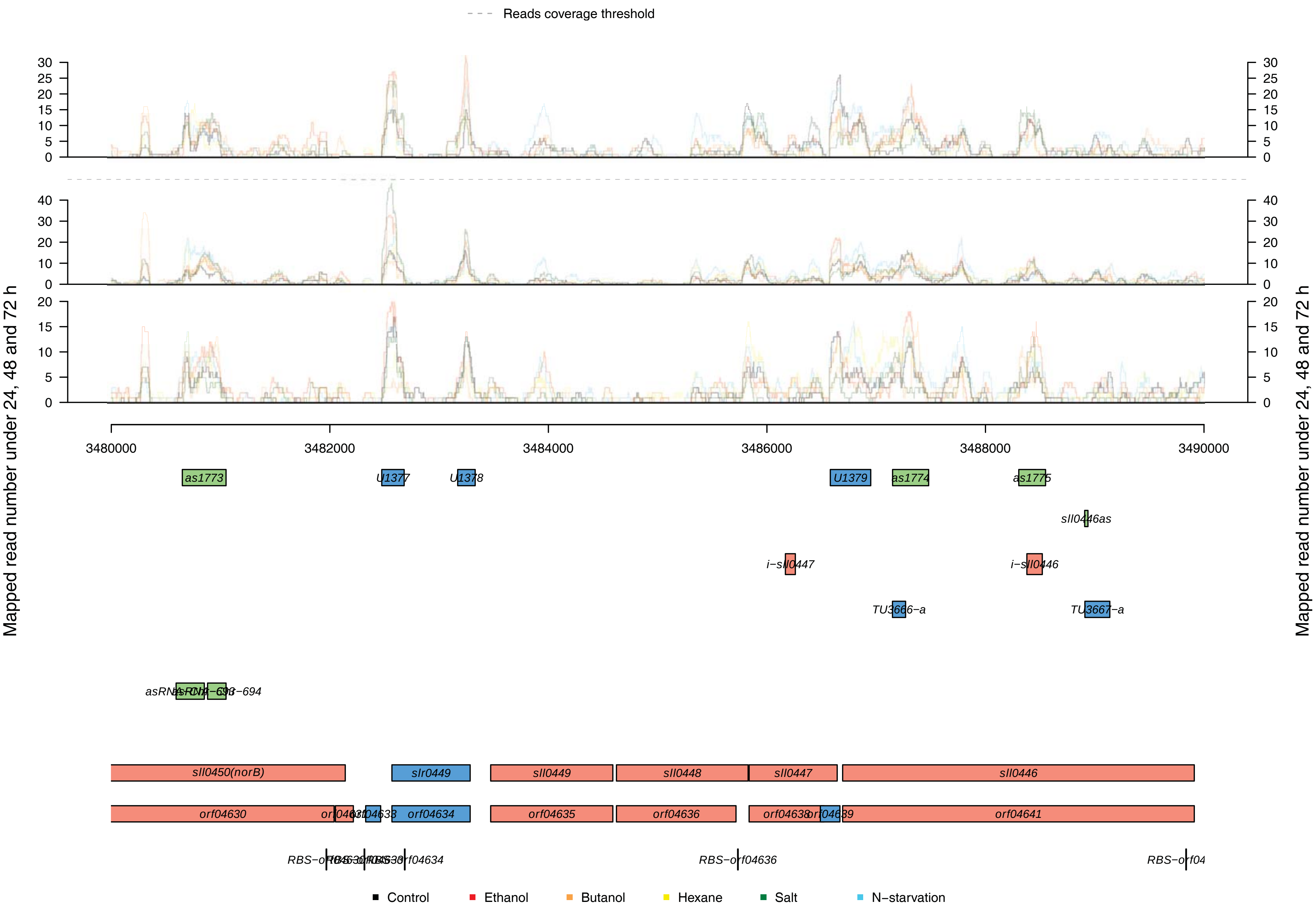
Reads coverage threshold
Mapped read number under 24, 48 and 72 h
3480000
3482000
3484000
3486000
3488000
3490000
as1773
U1377
U1378
U1379
as1774
as1775
sll0446as
i−sll0447
i−sll0446
TU3666−a
TU3667−a
sll0450(norB)
slr0449
sll0449
sll0448
sll0447
sll0446
orf04630
orf04634
orf04635
orf04636
orf04641
RBS−orf04636
Control
Ethanol
Butanol
Hexane
Salt
N−starvation

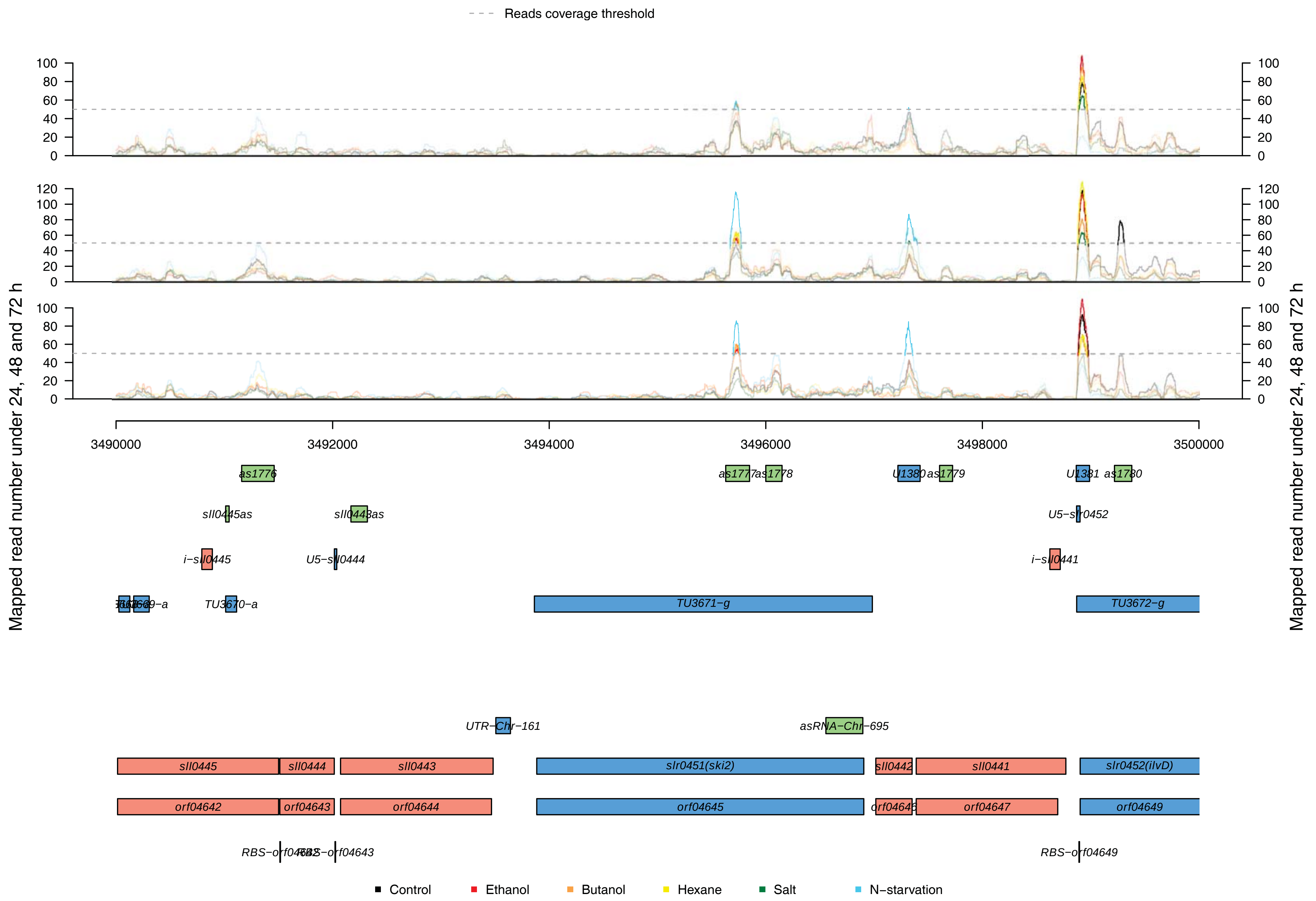
Reads coverage threshold
Mapped read number under 24, 48 and 72 h
3490000
3492000
3494000
3496000
3498000
3500000
as1776
as1777
as1778
U1380
as1779
U1381
as1780
sll0445as
sll0443as
U5−slr0452
i−sll0445
U5−sll0444
i−sll0441
TU3670−a
TU3671−g
TU3672−g
UTR−Chr−161
asRNA−Chr−695
sll0445
sll0444
sll0443
slr0451(ski2)
sll0442
sll0441
slr0452(ilvD)
orf04642
orf04643
orf04644
orf04645
orf04647
orf04649
RBS−orf04643
RBS−orf04649
Control
Ethanol
Butanol
Hexane
Salt
N−starvation

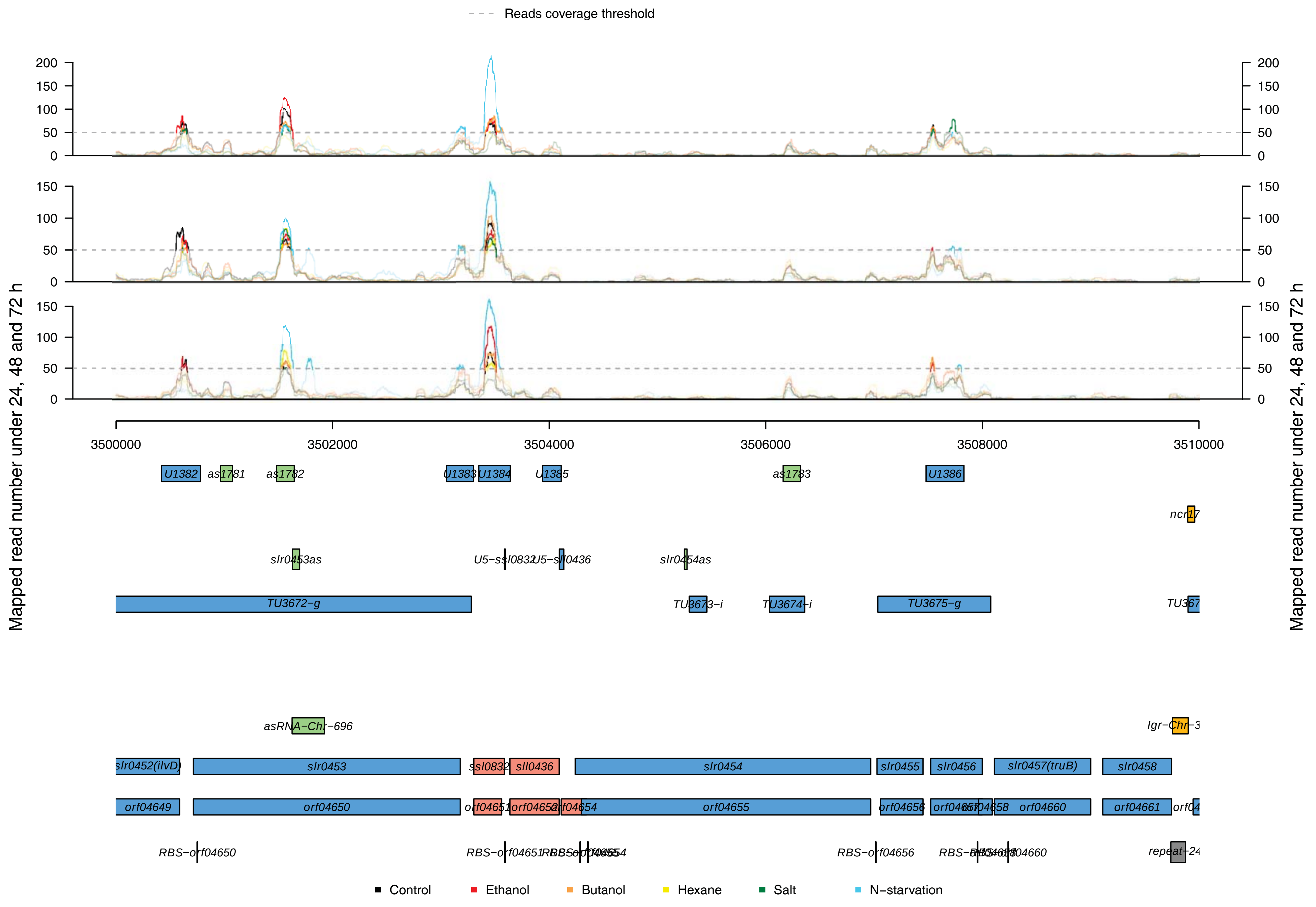

Reads coverage threshold
Mapped read number under 24, 48 and 72 h
3500000
3502000
3504000
3506000
3508000
3510000
U1382
as1781
as1782
U1383
U1384
U1385
as1783
U1386
ncr17
slr0453as
U5−ssl0832
U5−sll0436
slr0454as
TU3672−g
TU3673−i
TU3674−i
TU3675−g
asRNA−Chr−696
slr0452(ilvD)
slr0453
ssl0832
sll0436
slr0454
slr0455
slr0456
slr0457(truB)
slr0458
orf04649
orf04650
orf04651
orf04652
orf04654
orf04655
orf04656
orf04660
orf04661
RBS−orf04650
RBS−orf04656
Control
Ethanol
Butanol
Hexane
Salt
N−starvation

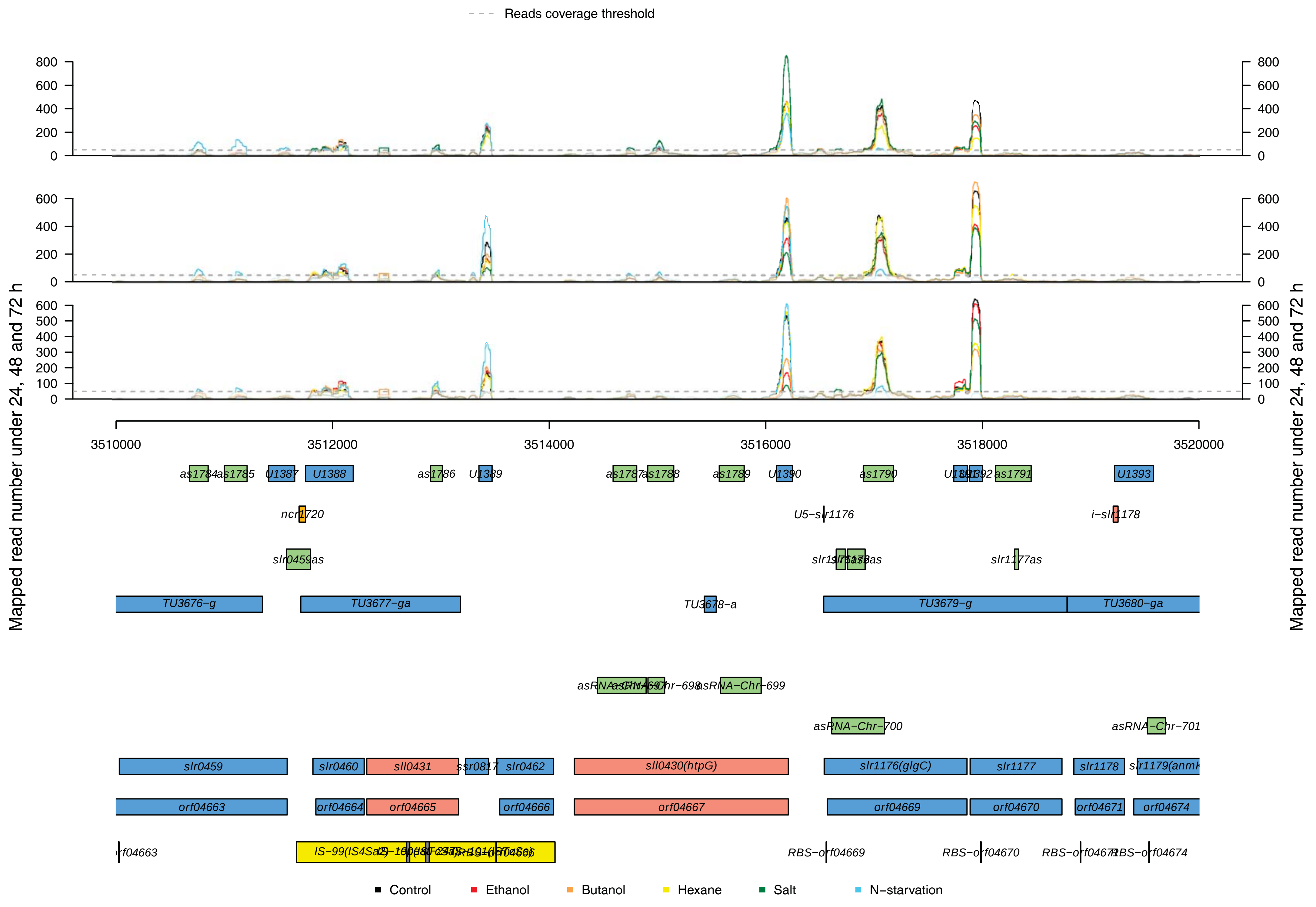

Reads coverage threshold
Mapped read number under 24, 48 and 72 h
3510000
3512000
3514000
3516000
3518000
3520000
TU3676−g
TU3677−ga
TU3679−g
TU3680−ga
slr0459
slr0460
sll0431
slr0462
sll0430(htpG)
slr1176(glgC)
slr1177
slr1178
orf04663
orf04664
orf04665
orf04666
orf04667
orf04669
orf04670
orf04671
orf04674
Control
Ethanol
Butanol
Hexane
Salt
N−starvation

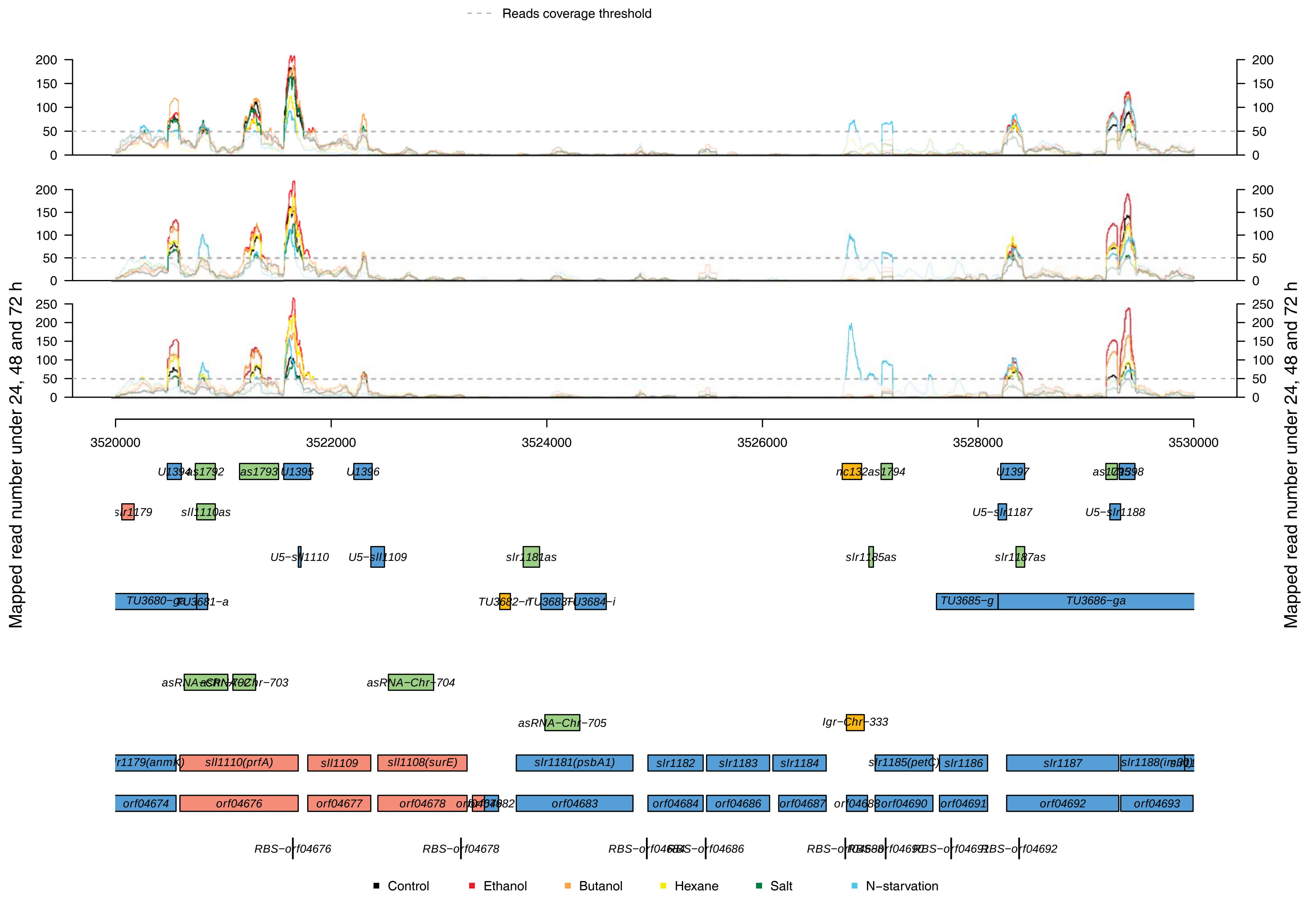
Reads coverage threshold
Mapped read number under 24, 48 and 72 h
3520000
3522000
3524000
3526000
3528000
3530000
as1793
U1395
U1396
nc132
as1794
U1397
slr1179
sll1110as
U5−sll1110
U5−sll1109
slr1181as
slr1185as
slr1187as
U5−slr1187
U5−slr1188
TU3680−ga
TU3681−a
TU3685−g
TU3686−ga
asRNA−Chr−703
asRNA−Chr−704
asRNA−Chr−705
Igr−Chr−333
sll1110(prfA)
sll1109
sll1108(surE)
slr1181(psbA1)
slr1182
slr1183
slr1184
slr1185(petC)
slr1186
slr1187
orf04674
orf04676
orf04677
orf04678
orf04683
orf04684
orf04686
orf04687
orf04690
orf04691
orf04692
orf04693
RBS−orf04676
RBS−orf04678
RBS−orf04686
RBS−orf04692
Control
Ethanol
Butanol
Hexane
Salt
N−starvation

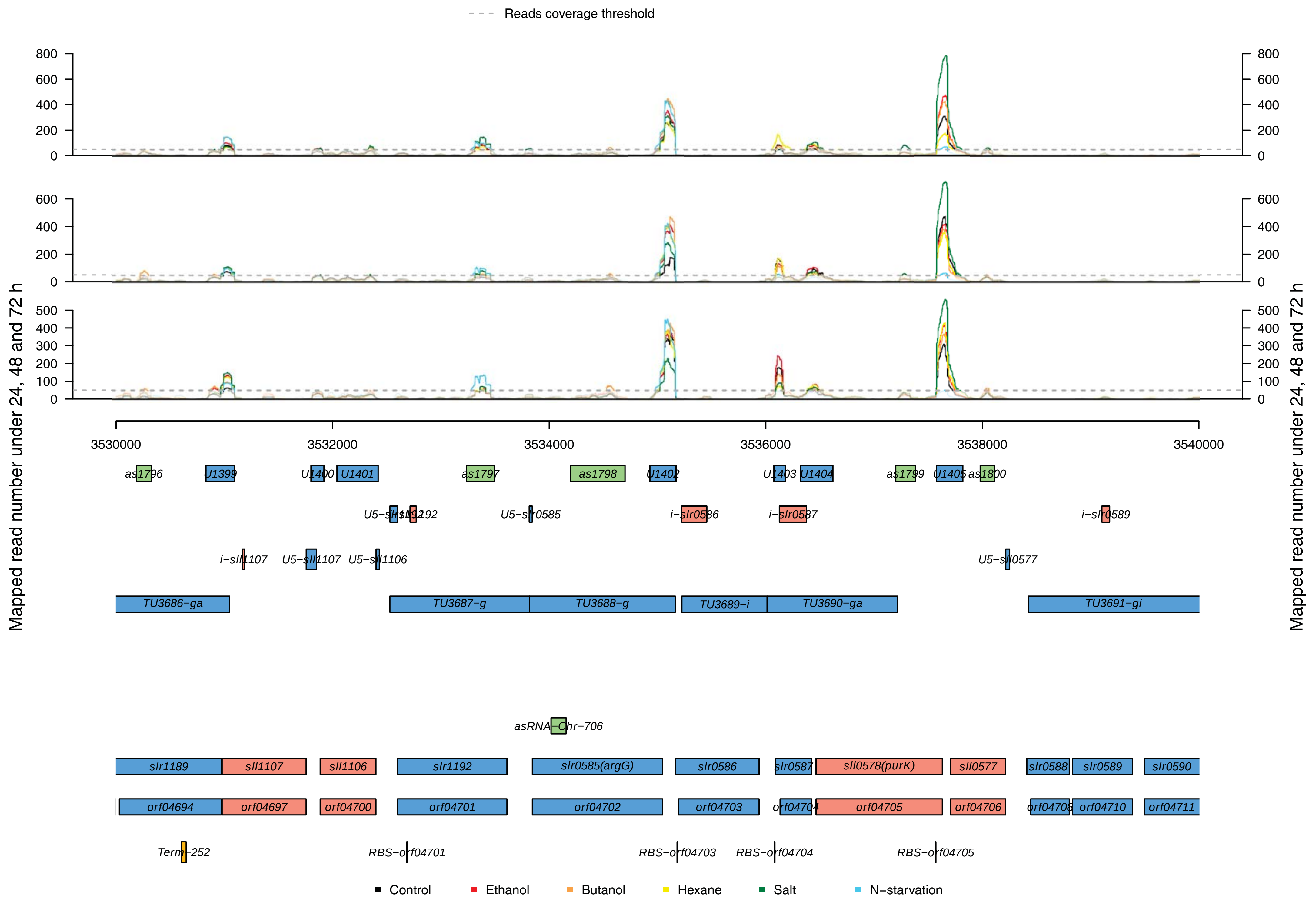

Reads coverage threshold
Mapped read number under 24, 48 and 72 h
3530000
3532000
3534000
3536000
3538000
3540000
as1796
U1399
U1400
U1401
as1797
as1798
U1402
U1403
U1404
as1799
U1405
as1800
U5-slr1192
U5-slr0585
i-slr0586
i-slr0587
i-slr0589
i-sll1107
U5-sll1107
U5-sll1106
U5-sll0577
TU3686-ga
TU3687-g
TU3688-g
TU3689-i
TU3690-ga
TU3691-gi
asRNA-Chr-706
slr1189
sll1107
sll1106
slr1192
slr0585(argG)
slr0586
slr0587
sll0578(purK)
sll0577
slr0588
slr0589
slr0590
orf04694
orf04697
orf04700
orf04701
orf04702
orf04703
orf04704
orf04705
orf04706
orf04708
orf04710
orf04711
Term-252
RBS-orf04701
RBS-orf04703
RBS-orf04704
RBS-orf04705
Control
Ethanol
Butanol
Hexane
Salt
N-starvation

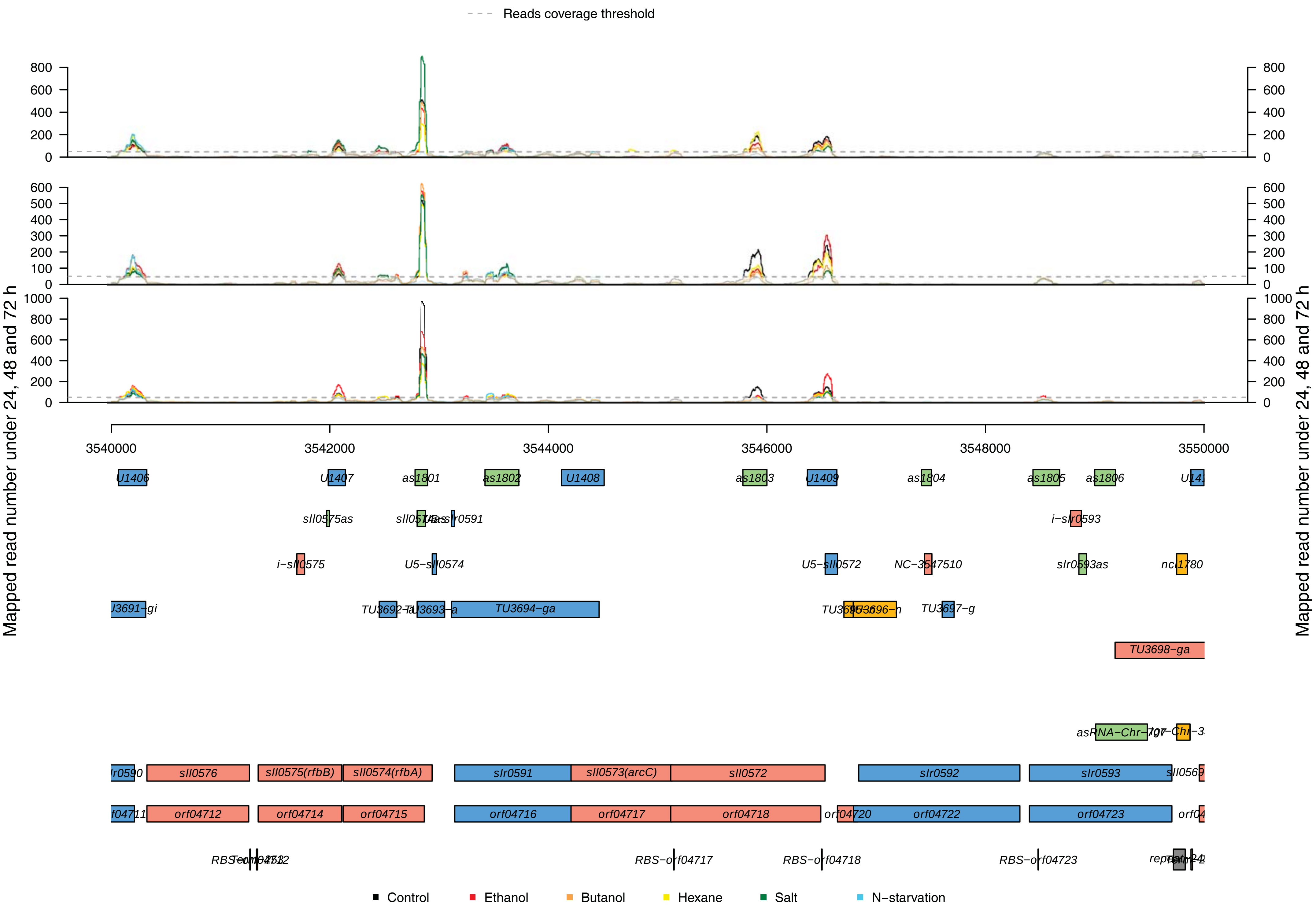
Reads coverage threshold
Mapped read number under 24, 48 and 72 h
3540000
3542000
3544000
3546000
3548000
3550000
Control
Ethanol
Butanol
Hexane
Salt
N−starvation

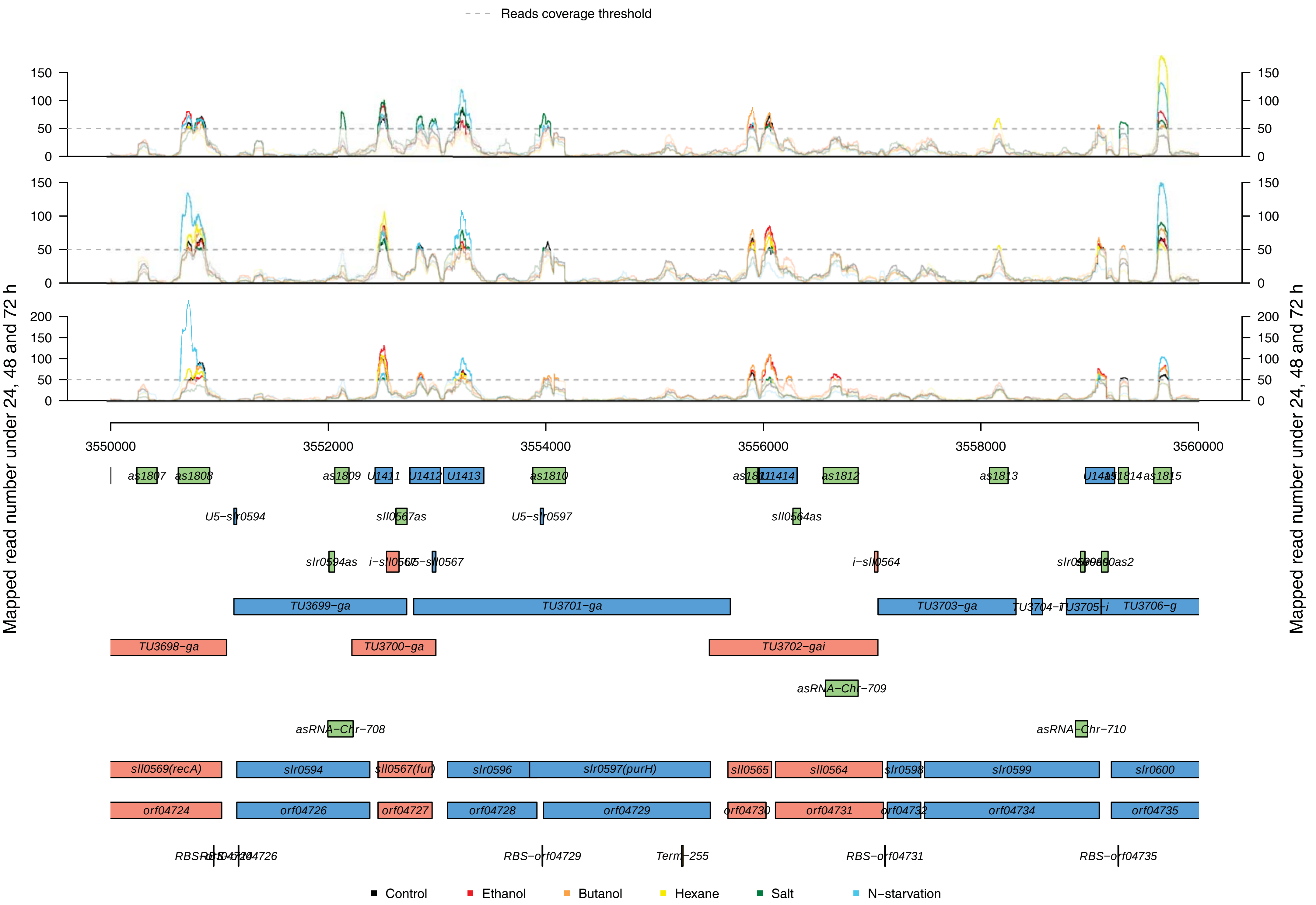

Reads coverage threshold
Mapped read number under 24, 48 and 72 h
3550000
3552000
3554000
3556000
3558000
3560000
as1807
as1808
as1809
as1810
as1812
as1813
as1815
U5−slr0594
sll0567as
U5−slr0597
sll0564as
slr0594as
i−sll0567
U5−sll0567
i−sll0564
TU3699−ga
TU3701−ga
TU3703−ga
TU3698−ga
TU3700−ga
TU3702−gai
asRNA−Chr−709
asRNA−Chr−708
asRNA−Chr−710
sll0569(recA)
slr0594
sll0567(fur)
slr0596
slr0597(purH)
sll0565
sll0564
slr0598
slr0599
slr0600
orf04724
orf04726
orf04727
orf04728
orf04729
orf04730
orf04731
orf04732
orf04734
orf04735
RBS−orf04729
Term−255
RBS−orf04731
RBS−orf04735
Control
Ethanol
Butanol
Hexane
Salt
N−starvation

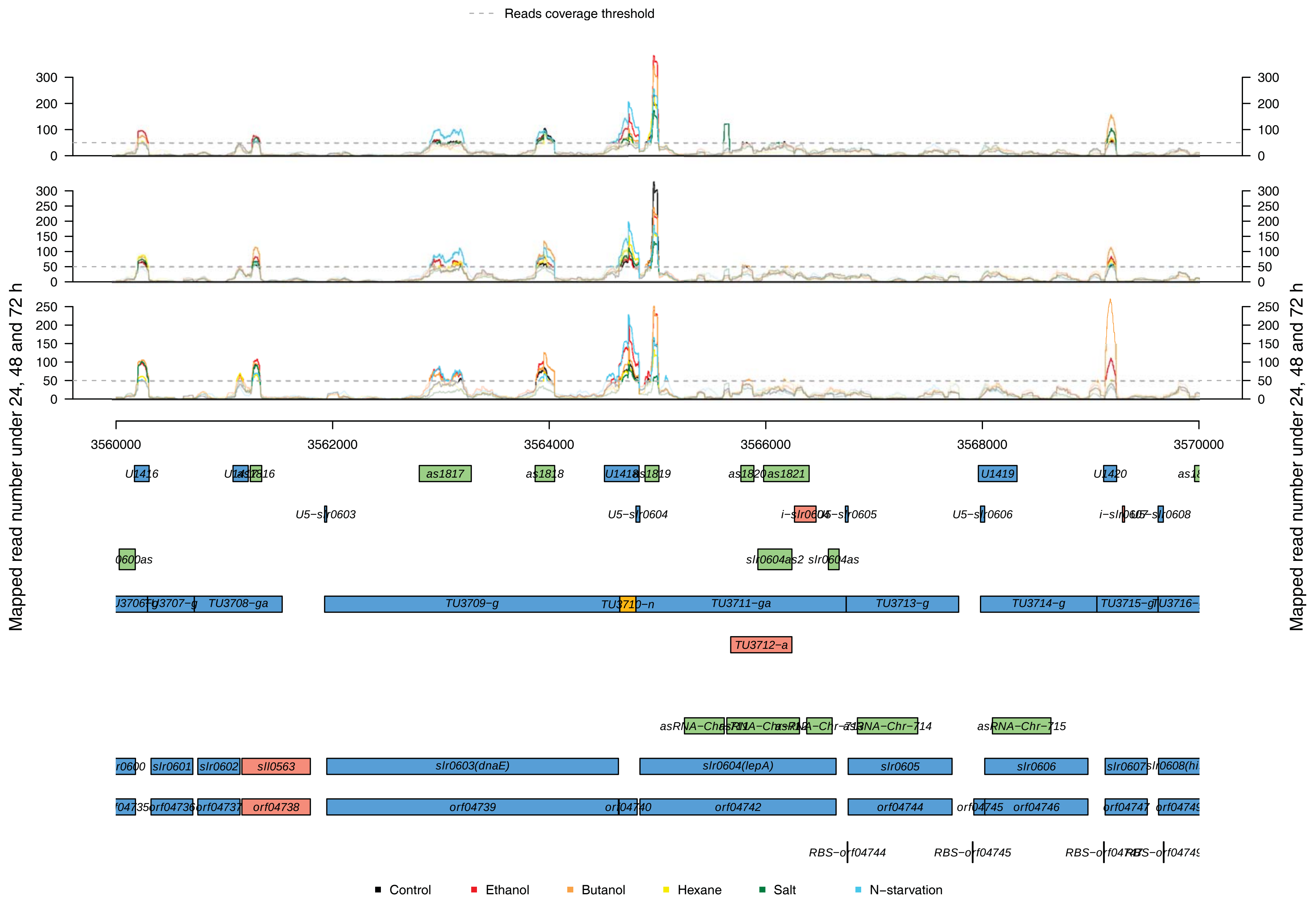

Reads coverage threshold
Mapped read number under 24, 48 and 72 h
3560000
3562000
3564000
3566000
3568000
3570000
as1817
U1419
TU3709-g
TU3711-ga
TU3713-g
TU3714-g
TU3712-a
slr0603(dnaE)
slr0604(lepA)
slr0605
slr0606
sll0563
orf04739
orf04742
orf04744
orf04746
Control
Ethanol
Butanol
Hexane
Salt
N-starvation

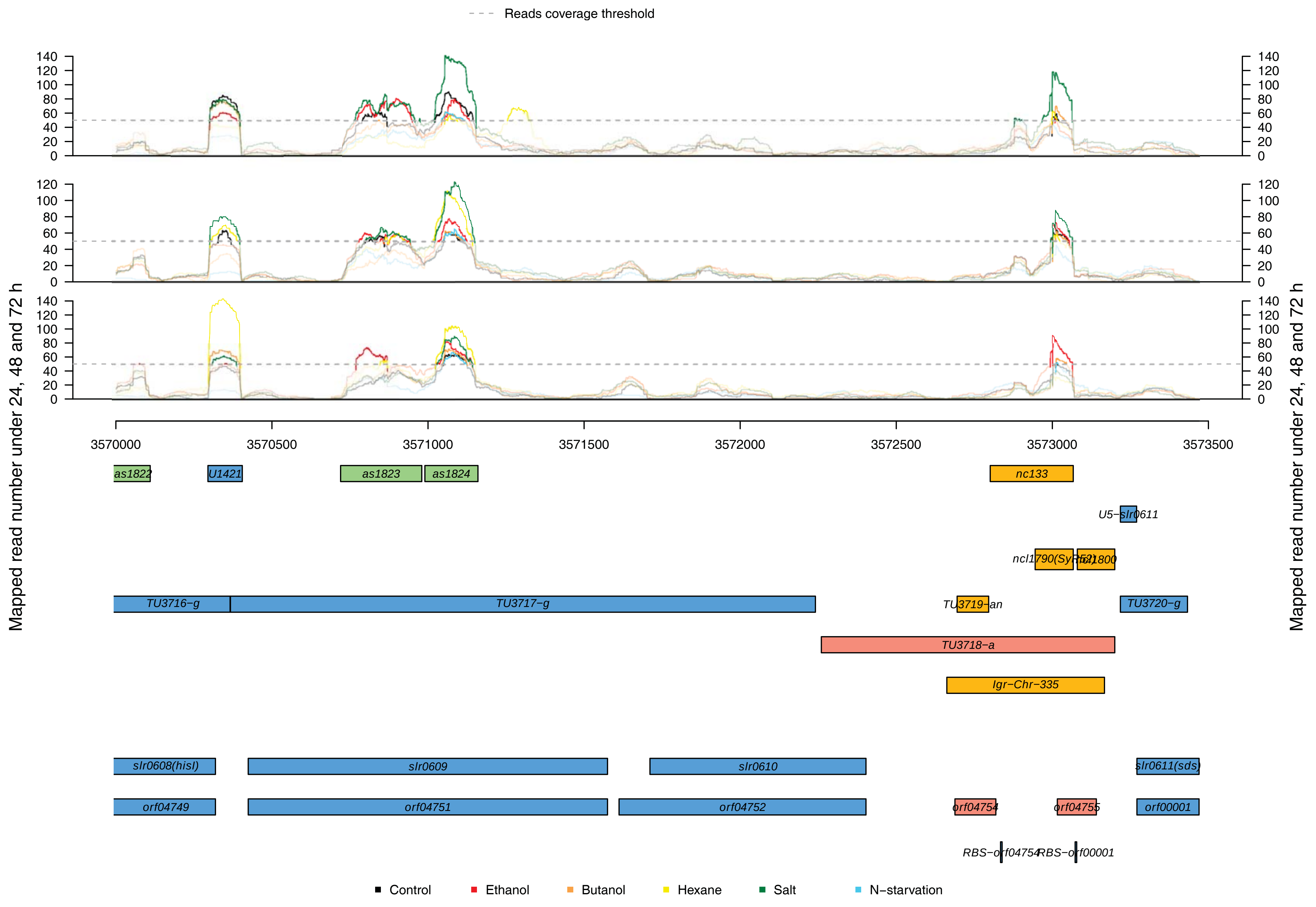

Reads coverage threshold
Mapped read number under 24, 48 and 72 h
3570000
3570500
3571000
3571500
3572000
3572500
3573000
3573500
as1822
U1421
as1823
as1824
nc133
U5−slr0611
ncl1790(SyR52)
ncl1800
TU3716−g
TU3717−g
TU3719−an
TU3720−g
TU3718−a
Igr−Chr−335
slr0608(hisI)
slr0609
slr0610
slr0611(sds)
orf04749
orf04751
orf04752
orf04754
orf04755
orf00001
RBS−orf04754
RBS−orf00001
Control
Ethanol
Butanol
Hexane
Salt
N−starvation
