# Supplementary material for: Systematic and functional identification of small non-coding RNAs associated with exogenous biofuel stress in cyanobacterium Synechocystis sp. PCC 6803
Source: Biotechnol Biofuels. 2017 Mar 7;10:57. doi: 10.1186/s13068-017-0743-y (PMC5341163; doi:10.1186/s13068-017-0743-y)
Supplement: Supplementary file 5 — Additional file 5: Figure S2. Detailed visualization of sRNA mapping data of 133 trans-encoded sRNAs in Synechocystis. Detailed description is the same as Additional file 2: Figure S1. [file 13068_2017_743_MOESM5_ESM.pdf]

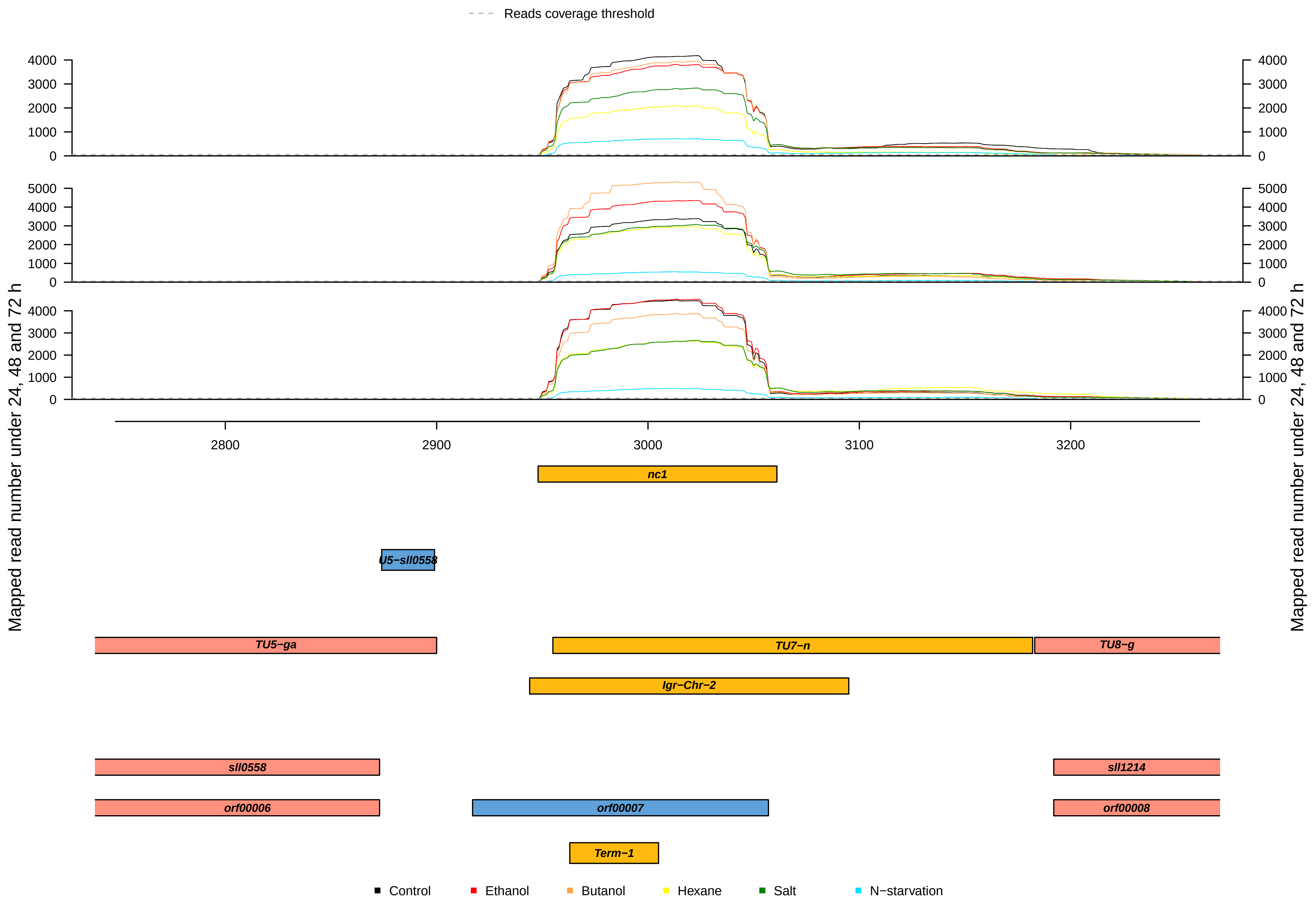

Reads coverage threshold
Mapped read number under 24, 48 and 72 h
2800
2900
3000
3100
3200
nc1
U5−sll0558
TU5−ga
TU7−n
TU8−g
Igr−Chr−2
sll0558
sll1214
orf00006
orf00007
orf00008
Term−1
Control
Ethanol
Butanol
Hexane
Salt
N−starvation

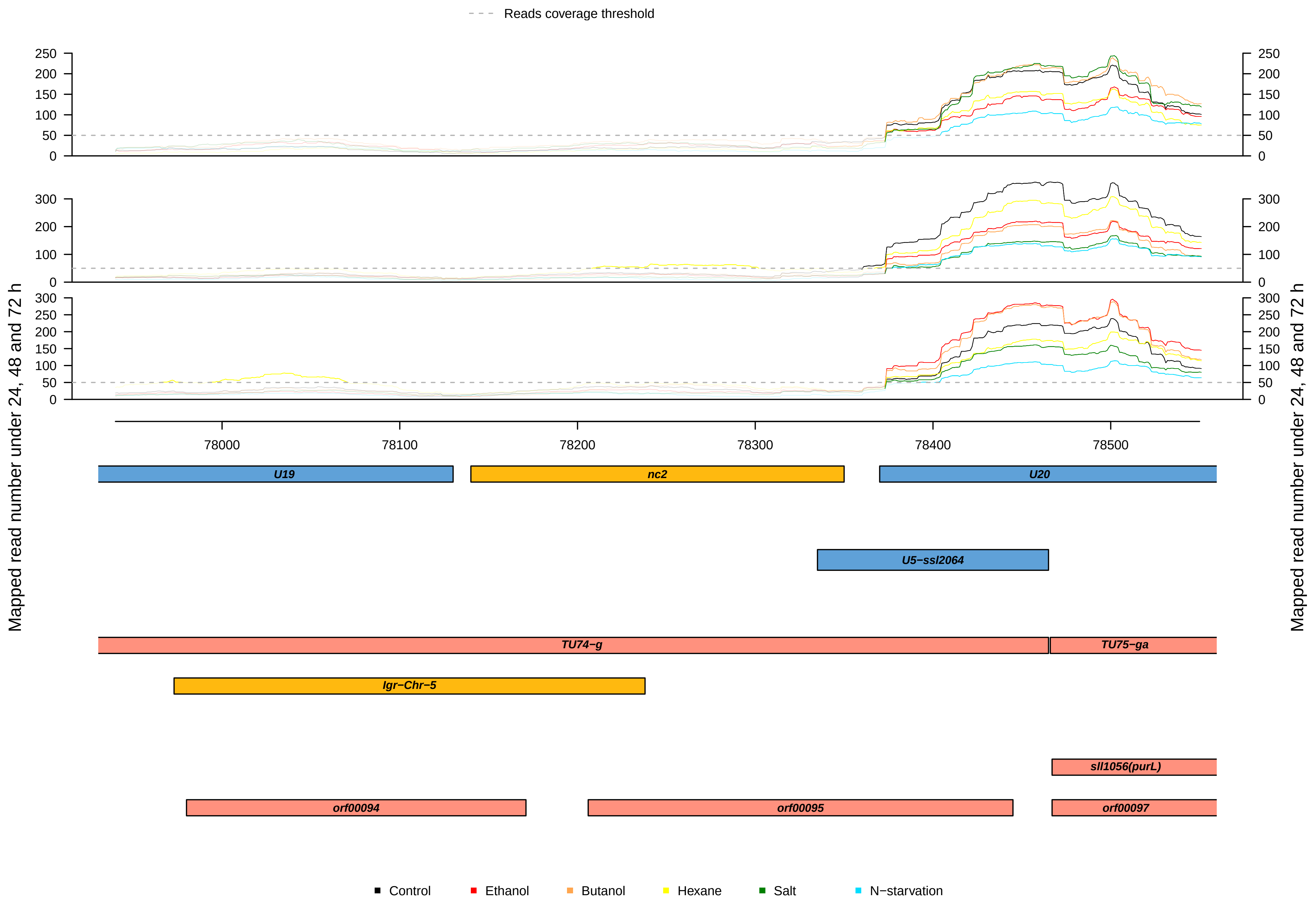

Reads coverage threshold
Mapped read number under 24, 48 and 72 h
250
200
150
100
50
0
300
200
100
0
300
250
200
150
100
50
0
78000
78100
78200
78300
78400
78500
U19
nc2
U20
U5−ssl2064
TU74−g
TU75−ga
Igr−Chr−5
sll1056(purL)
orf00094
orf00095
orf00097
Control
Ethanol
Butanol
Hexane
Salt
N−starvation

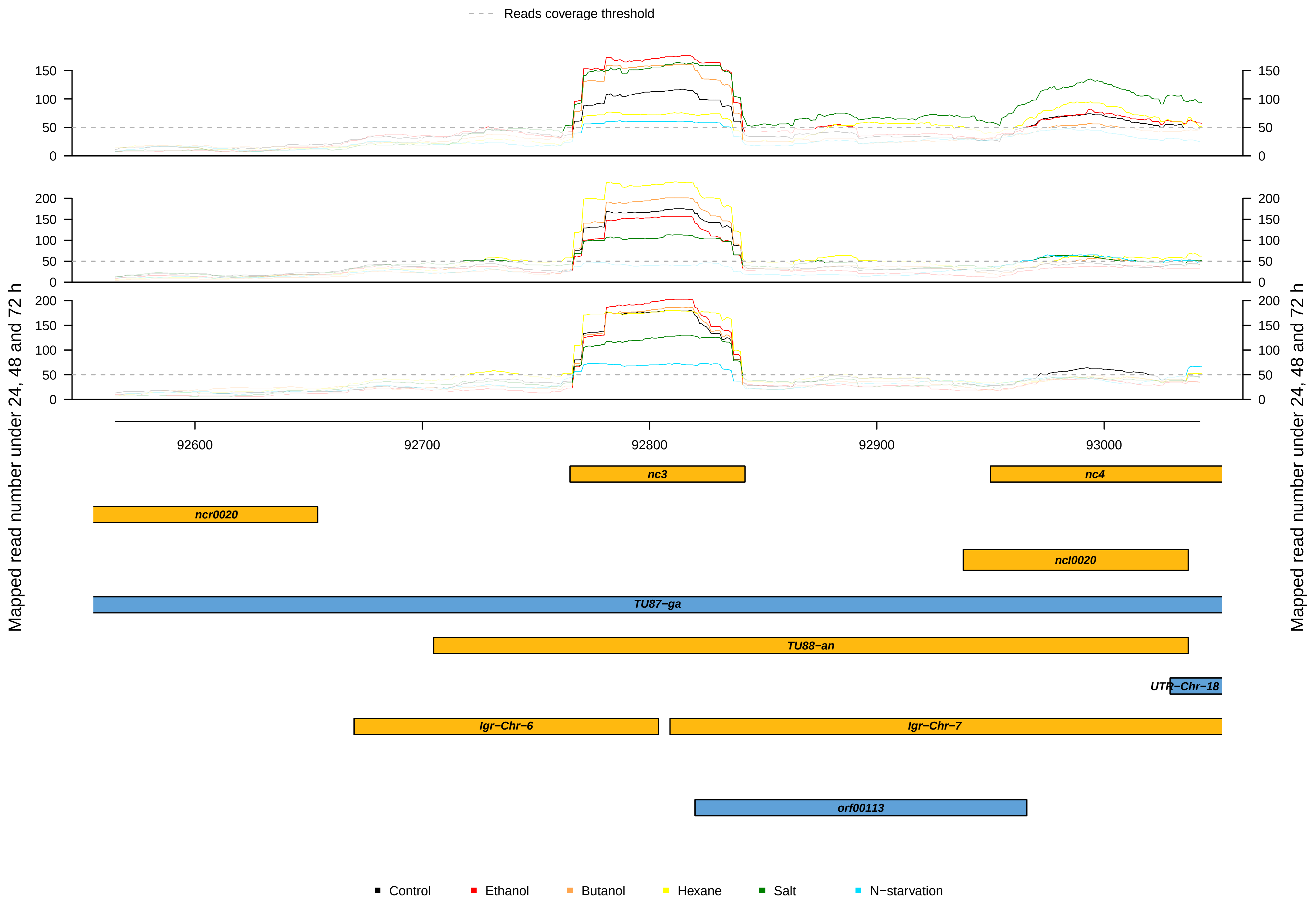

Reads coverage threshold
150
100
50
0
200
150
100
50
0
200
150
100
50
0
Mapped read number under 24, 48 and 72 h
92600
92700
92800
92900
93000
nc3
nc4
ncr0020
ncl0020
TU87−ga
TU88−an
UTR−Chr−18
Igr−Chr−6
Igr−Chr−7
orf00113
Control
Ethanol
Butanol
Hexane
Salt
N−starvation

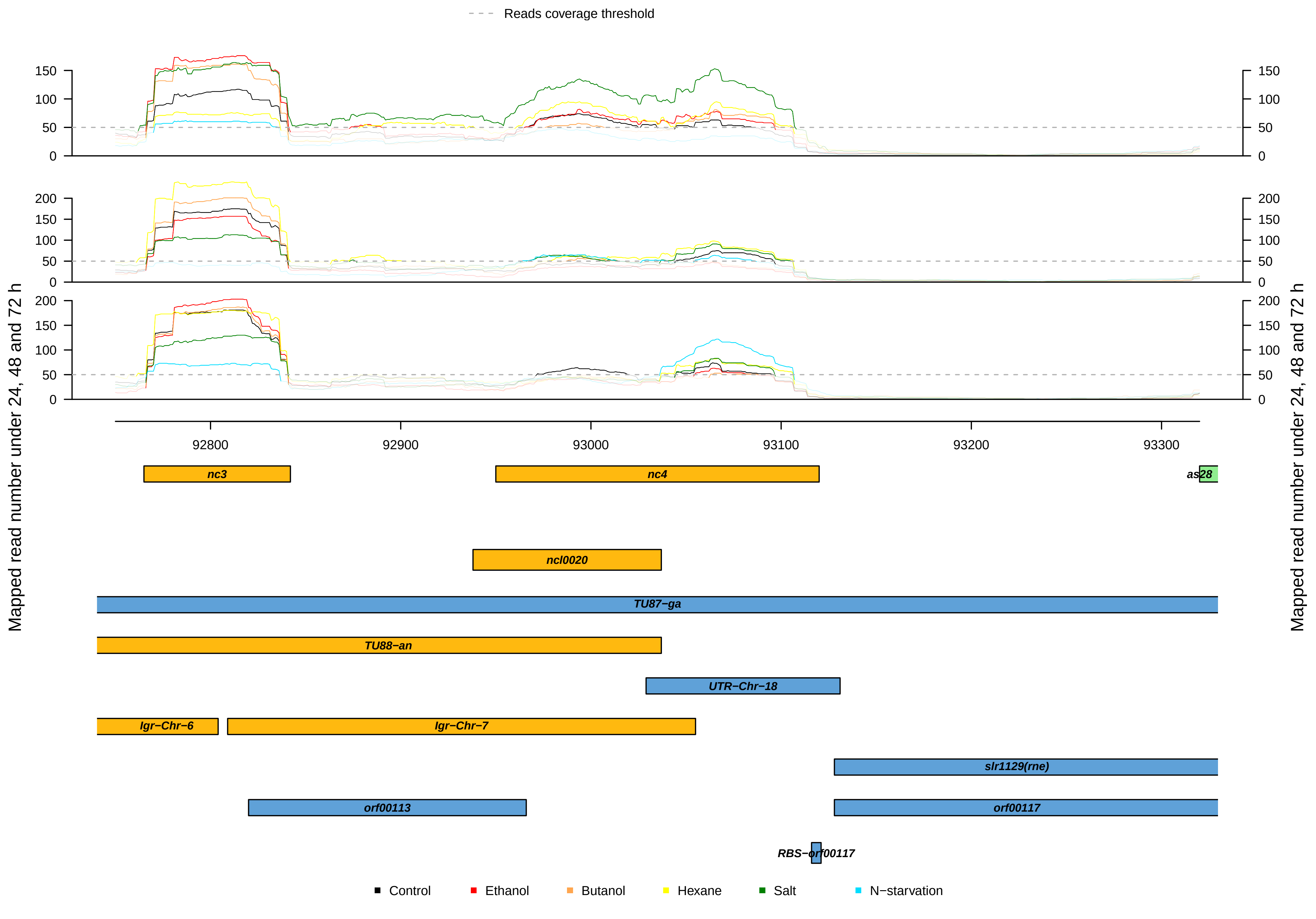

Reads coverage threshold
Mapped read number under 24, 48 and 72 h
150
100
50
0
200
150
100
50
0
200
150
100
50
0
92800
92900
93000
93100
93200
93300
nc3
nc4
as28
ncl0020
TU87−ga
TU88−an
UTR−Chr−18
Igr−Chr−6
Igr−Chr−7
slr1129(rne)
orf00113
orf00117
RBS−orf00117
Control
Ethanol
Butanol
Hexane
Salt
N−starvation

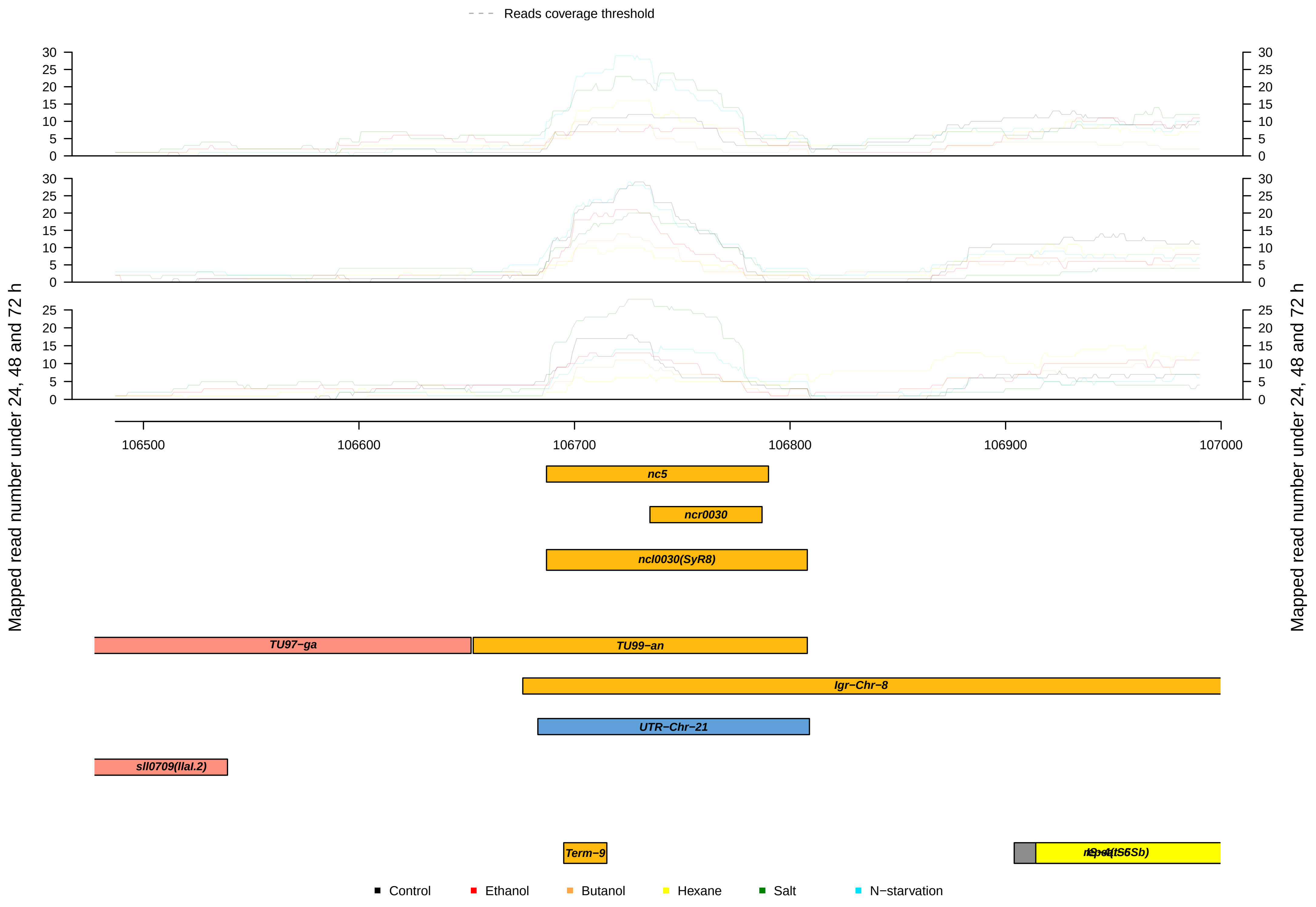

Reads coverage threshold
Mapped read number under 24, 48 and 72 h
106500
106600
106700
106800
106900
107000
nc5
ncr0030
ncl0030(SyR8)
TU97−ga
TU99−an
Igr−Chr−8
UTR−Chr−21
sll0709(llaI.2)
Term−9
Control
Ethanol
Butanol
Hexane
Salt
N−starvation

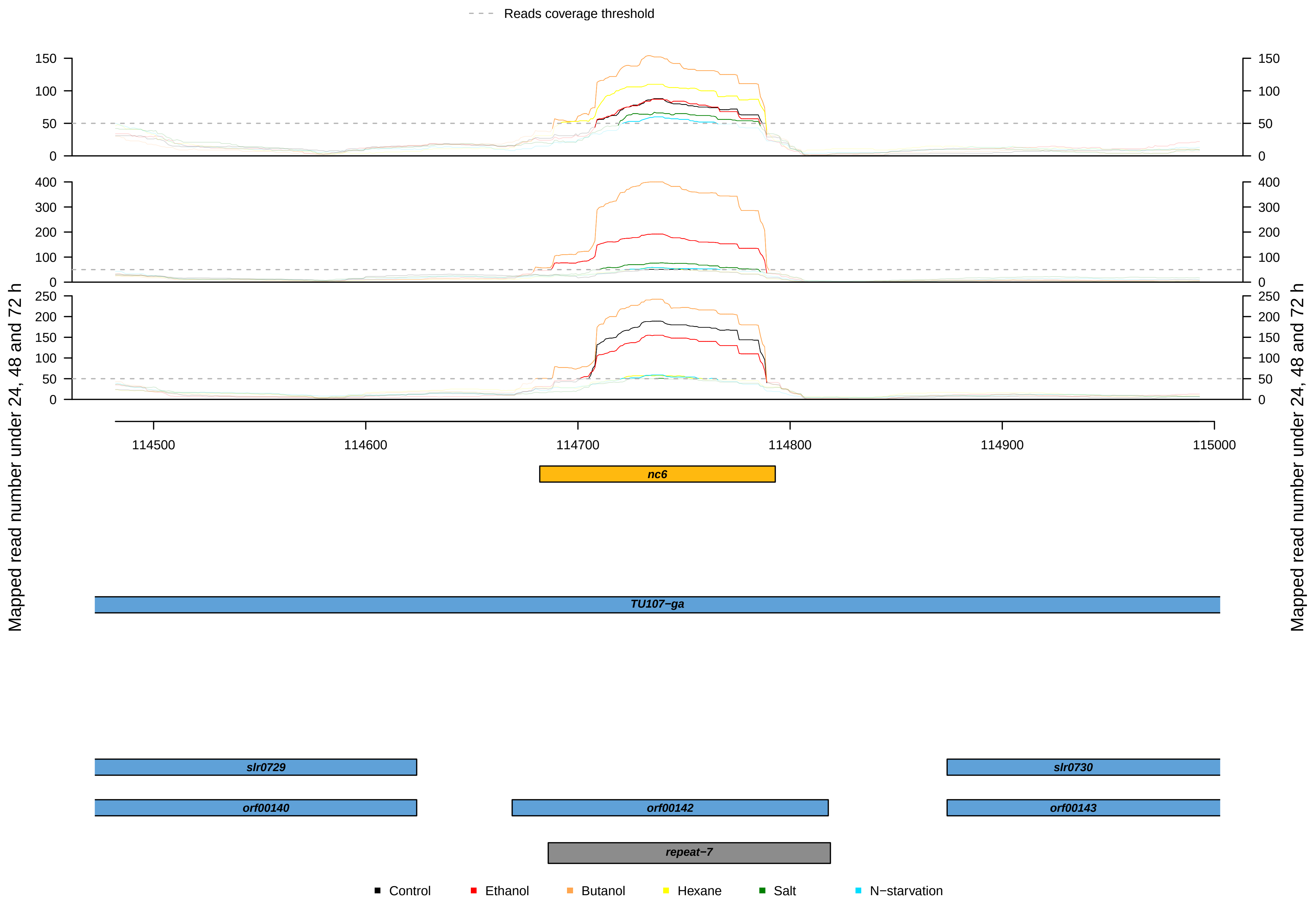

Reads coverage threshold
Mapped read number under 24, 48 and 72 h
114500
114600
114700
114800
114900
115000
nc6
TU107−ga
slr0729
slr0730
orf00140
orf00142
orf00143
repeat−7
Control
Ethanol
Butanol
Hexane
Salt
N−starvation

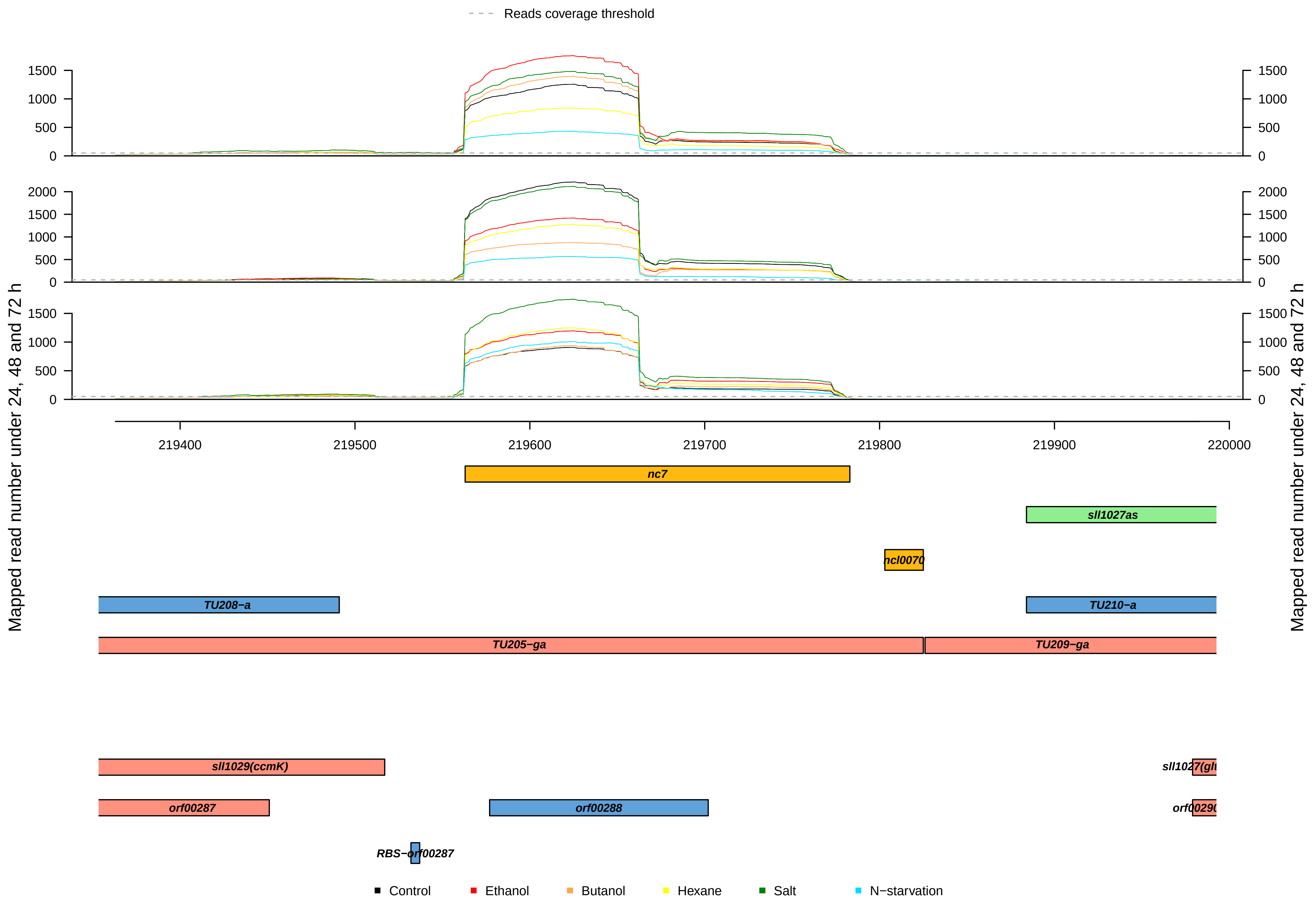

Reads coverage threshold
1500
1000
500
0
2000
1500
1000
500
0
Mapped read number under 24, 48 and 72 h
219400
219500
219600
219700
219800
219900
220000
nc7
sll1027as
ncl0070
TU208−a
TU210−a
TU205−ga
TU209−ga
sll1029(ccmK)
sll1027(glt
orf00287
orf00288
orf0029(
RBS−orf00287
Control
Ethanol
Butanol
Hexane
Salt
N−starvation

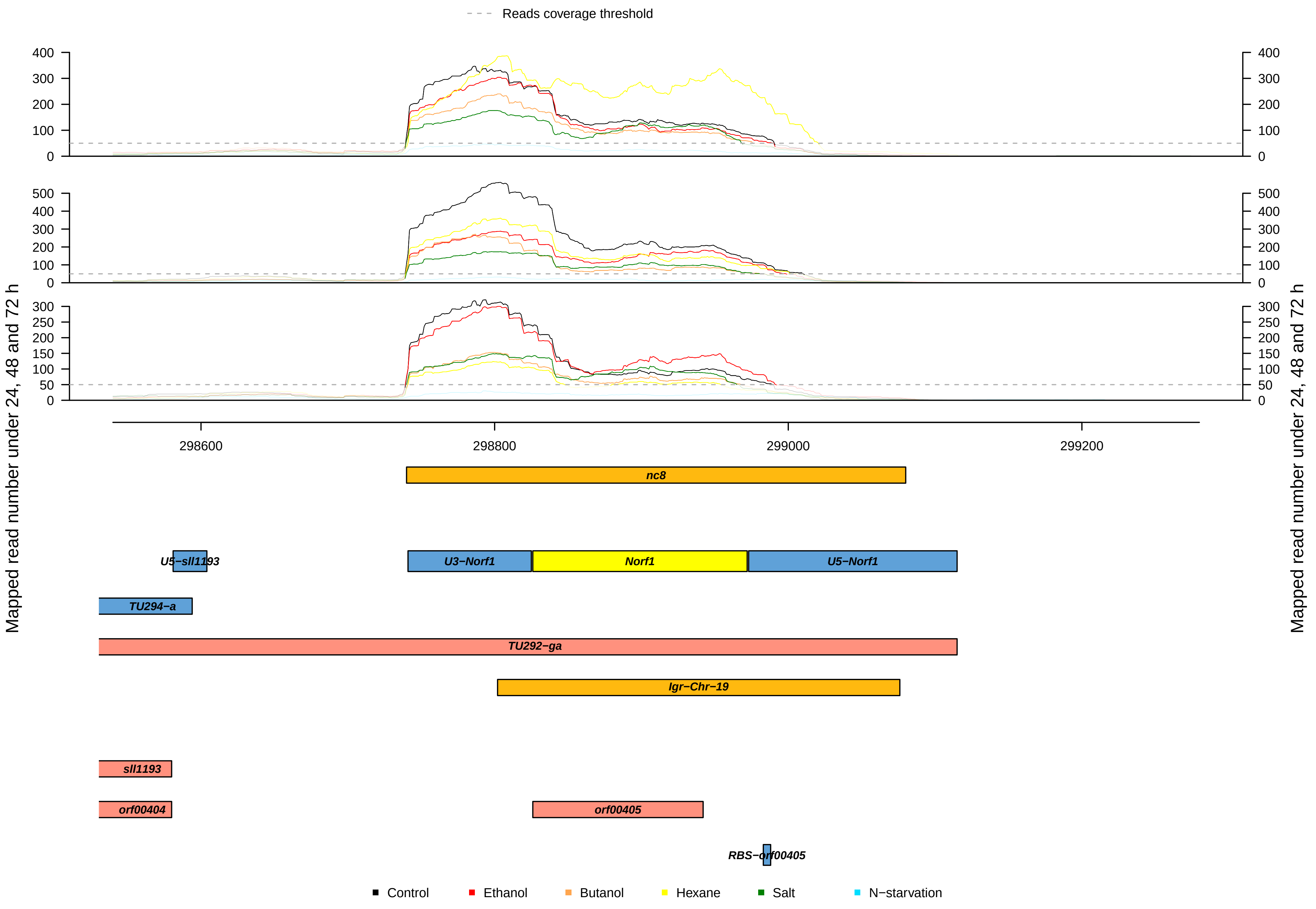

Reads coverage threshold
400
300
200
100
0
500
400
300
200
100
0
300
250
200
150
100
50
0
Mapped read number under 24, 48 and 72 h
298600
298800
299000
299200
nc8
U5−sll1193
U3−Norf1
Norf1
U5−Norf1
TU294−a
TU292−ga
Igr−Chr−19
sll1193
orf00404
orf00405
RBS−orf00405
Control
Ethanol
Butanol
Hexane
Salt
N−starvation

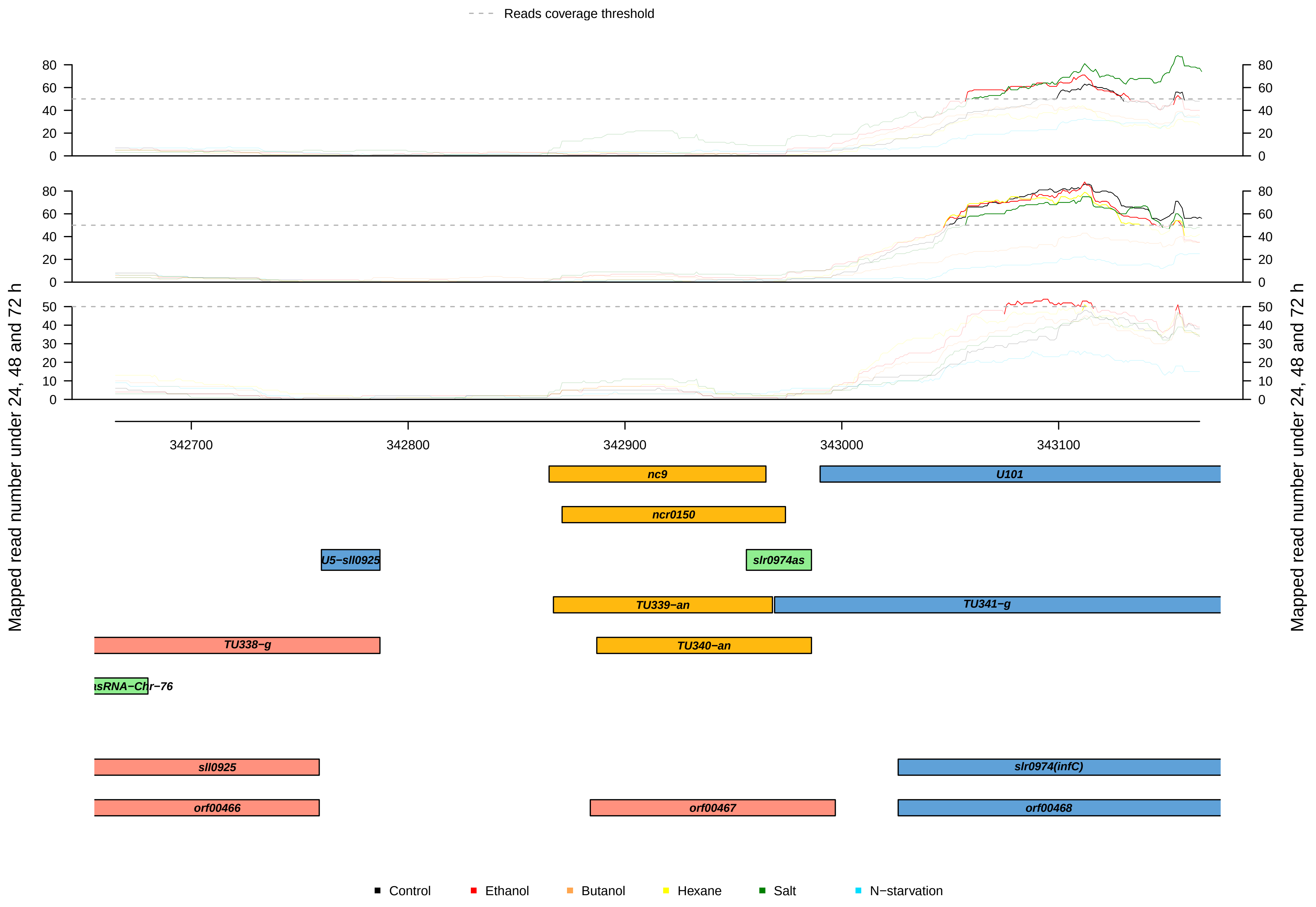

Reads coverage threshold
Mapped read number under 24, 48 and 72 h
342700
342800
342900
343000
343100
nc9
U101
ncr0150
U5−sll0925
slr0974as
TU339−an
TU341−g
TU338−g
TU340−an
asRNA−Chr−76
sll0925
slr0974(infC)
orf00466
orf00467
orf00468
Control
Ethanol
Butanol
Hexane
Salt
N−starvation

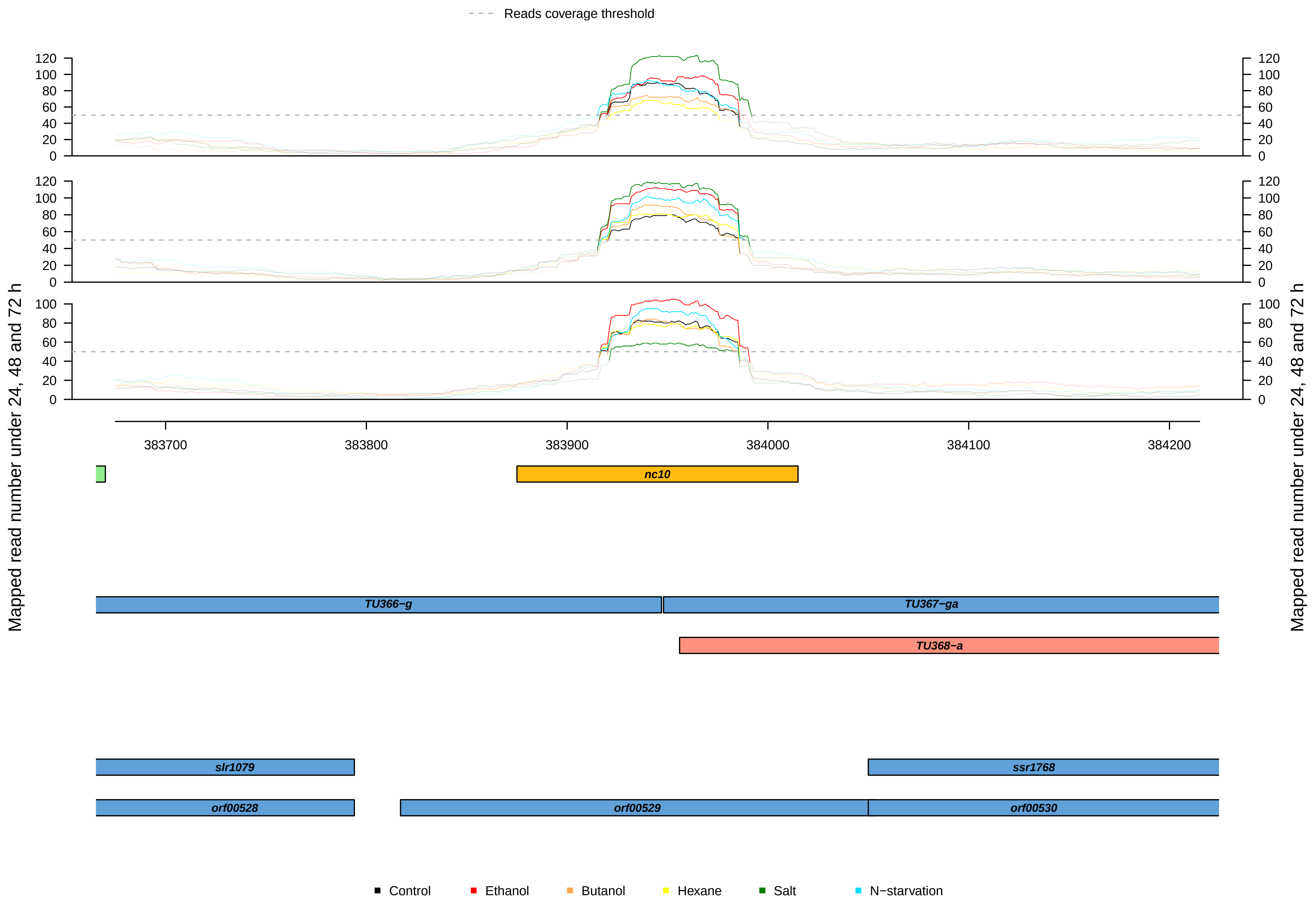

Reads coverage threshold
Mapped read number under 24, 48 and 72 h
0
20
40
60
80
100
120
383700
383800
383900
384000
384100
384200
nc10
TU366−g
TU367−ga
TU368−a
slr1079
ssr1768
orf00528
orf00529
orf00530
Control
Ethanol
Butanol
Hexane
Salt
N−starvation

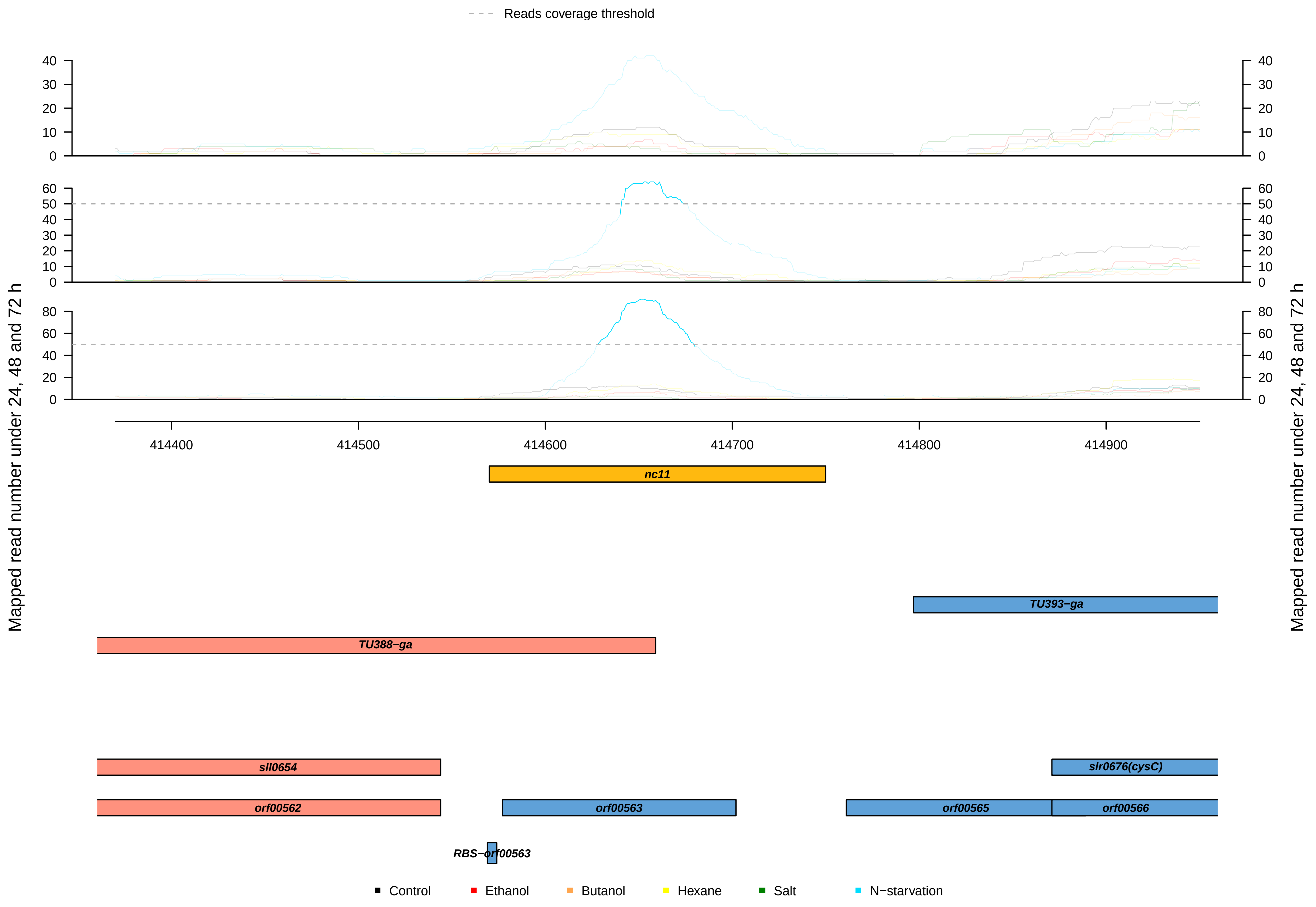

Reads coverage threshold
Mapped read number under 24, 48 and 72 h
414400
414500
414600
414700
414800
414900
nc11
TU393−ga
TU388−ga
sll0654
slr0676(cysC)
orf00562
orf00563
orf00565
orf00566
RBS−orf00563
Control
Ethanol
Butanol
Hexane
Salt
N−starvation

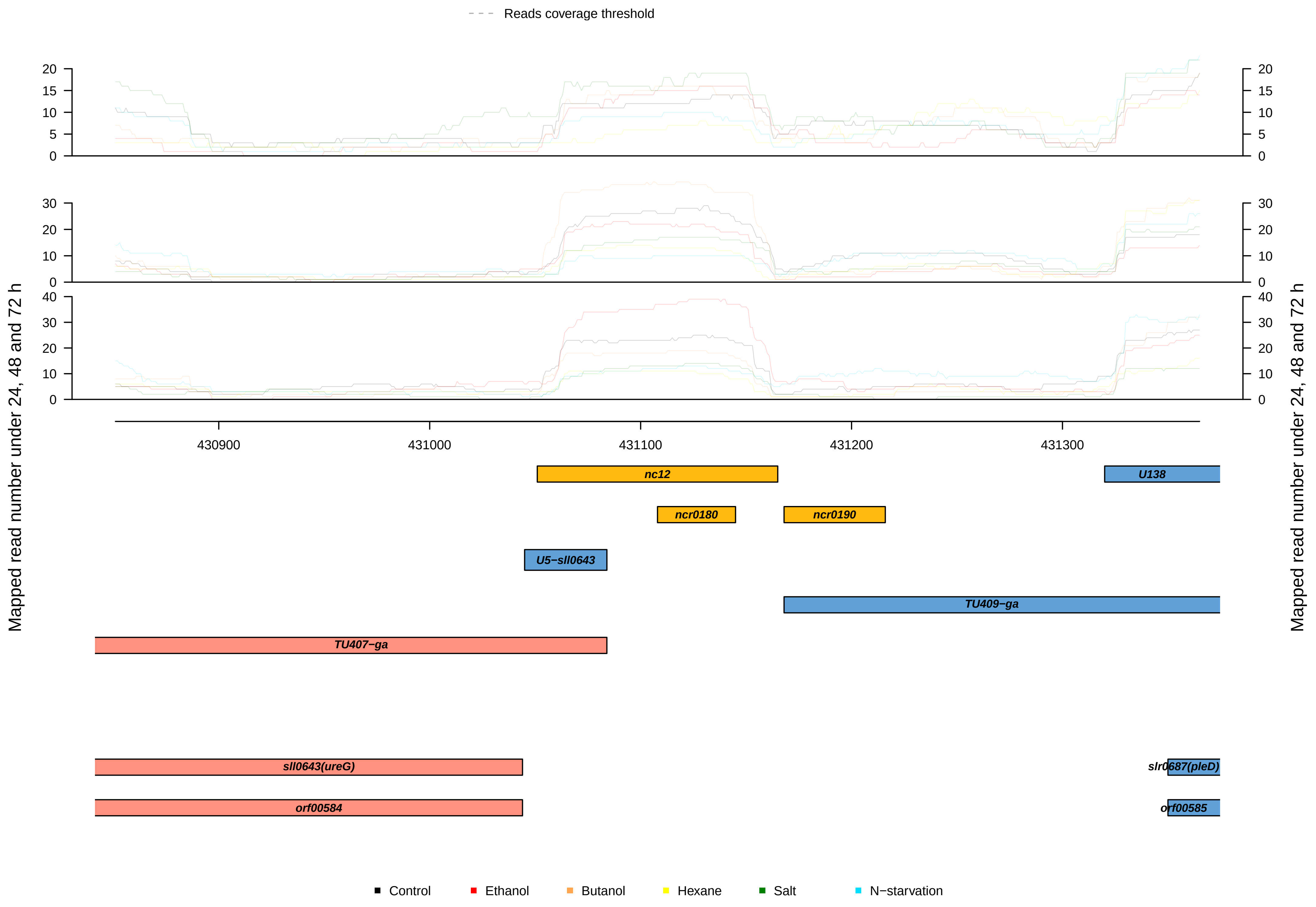

Reads coverage threshold
Mapped read number under 24, 48 and 72 h
430900
431000
431100
431200
431300
nc12
U138
ncr0180
ncr0190
U5−sll0643
TU409−ga
TU407−ga
sll0643(ureG)
slr0687(pleD)
orf00584
orf00585
Control
Ethanol
Butanol
Hexane
Salt
N−starvation

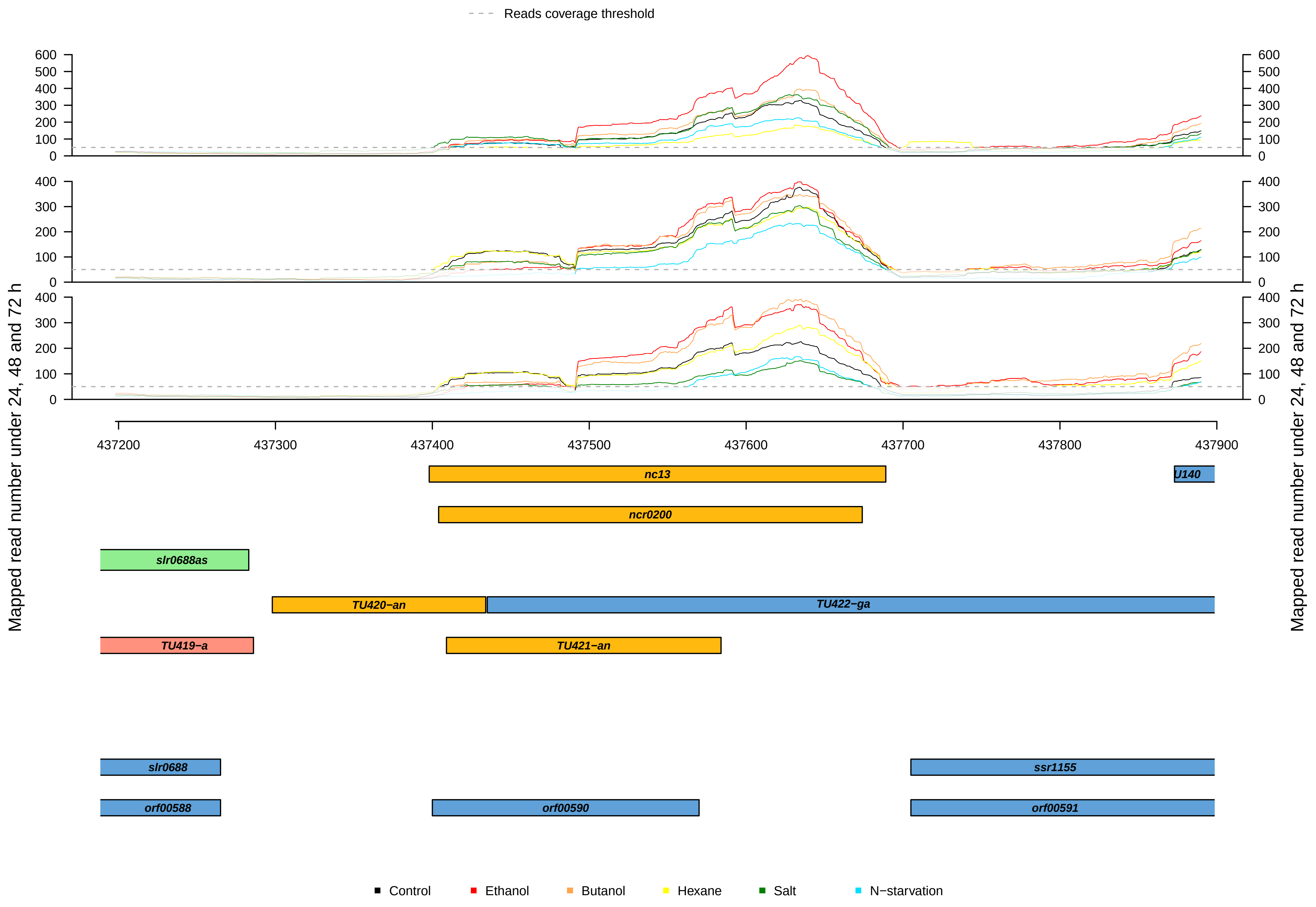

Reads coverage threshold
Mapped read number under 24, 48 and 72 h
600
500
400
300
200
100
0
437200
437300
437400
437500
437600
437700
437800
437900
nc13
U140
ncr0200
slr0688as
TU420−an
TU422−ga
TU419−a
TU421−an
slr0688
ssr1155
orf00588
orf00590
orf00591
Control
Ethanol
Butanol
Hexane
Salt
N−starvation

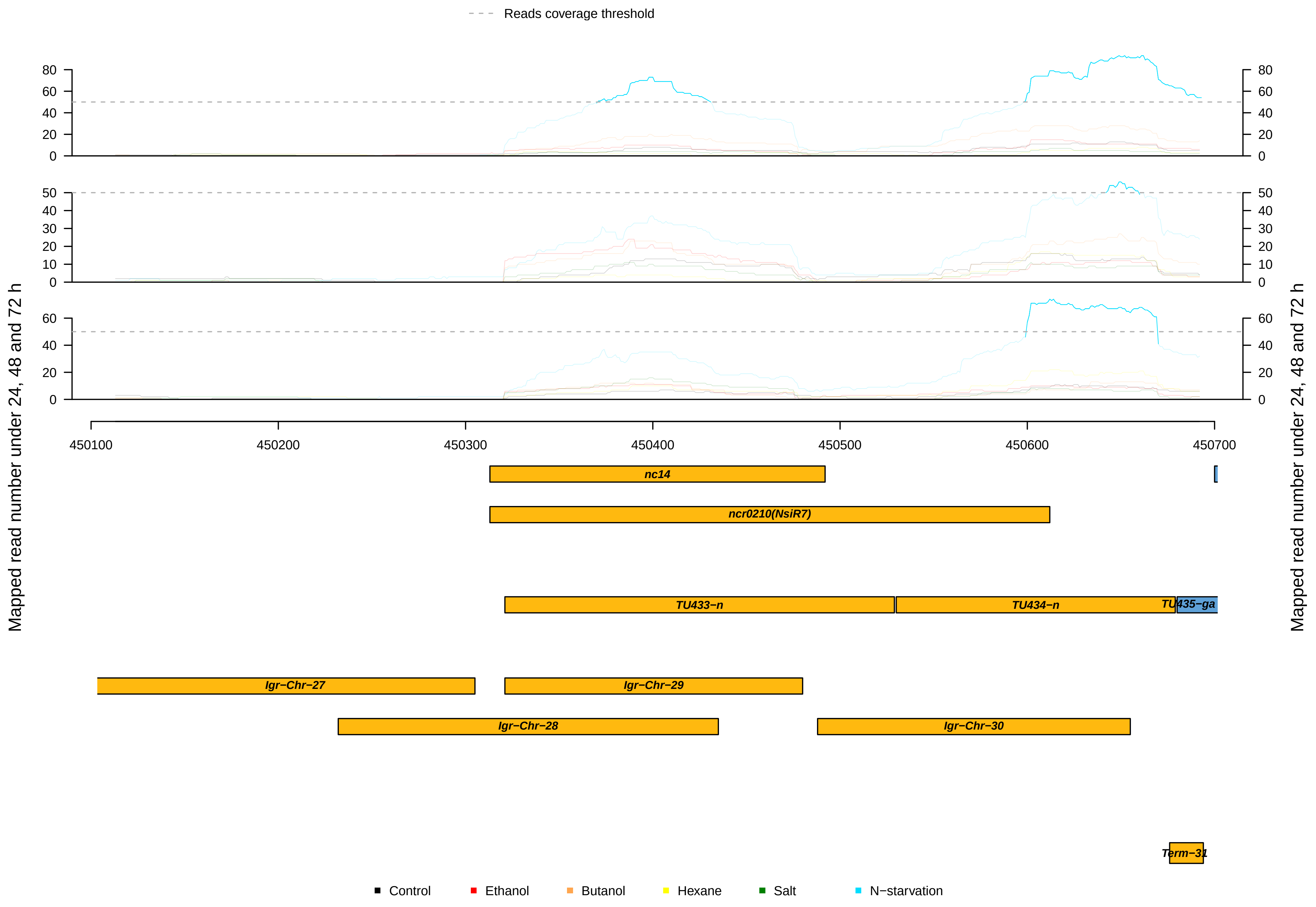

Reads coverage threshold
Mapped read number under 24, 48 and 72 h
80
60
40
20
0
50
40
30
20
10
0
60
40
20
0
450100
450200
450300
450400
450500
450600
450700
nc14
ncr0210(NsiR7)
TU433−n
TU434−n
TU435−ga
Igr−Chr−27
Igr−Chr−29
Igr−Chr−28
Igr−Chr−30
Term−31
Control
Ethanol
Butanol
Hexane
Salt
N−starvation

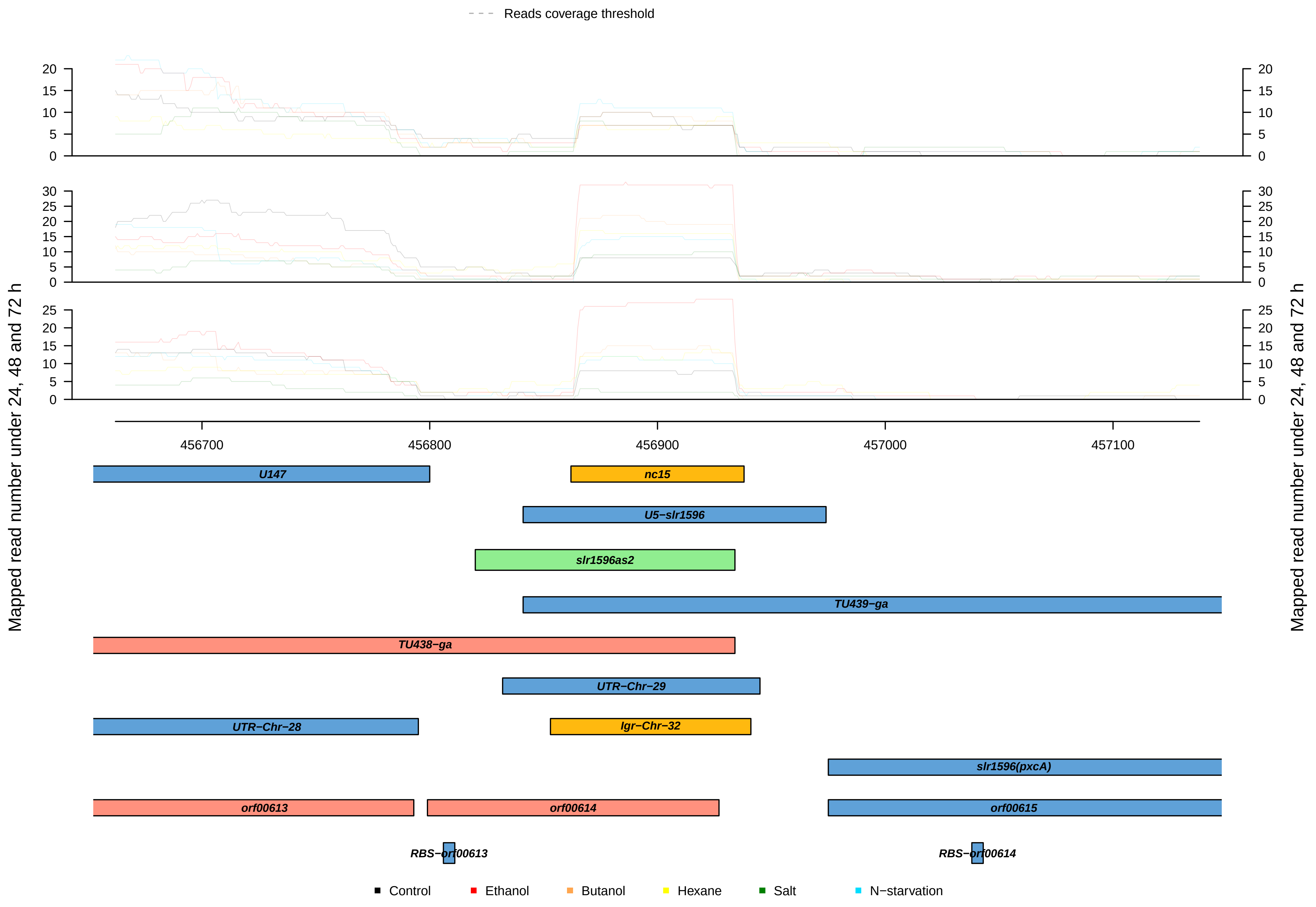
Reads coverage threshold
Mapped read number under 24, 48 and 72 h
456700
456800
456900
457000
457100
U147
nc15
U5−slr1596
slr1596as2
TU439−ga
TU438−ga
UTR−Chr−29
UTR−Chr−28
Igr−Chr−32
slr1596(pxcA)
orf00613
orf00614
orf00615
RBS−orf00613
RBS−orf00614
Control
Ethanol
Butanol
Hexane
Salt
N−starvation

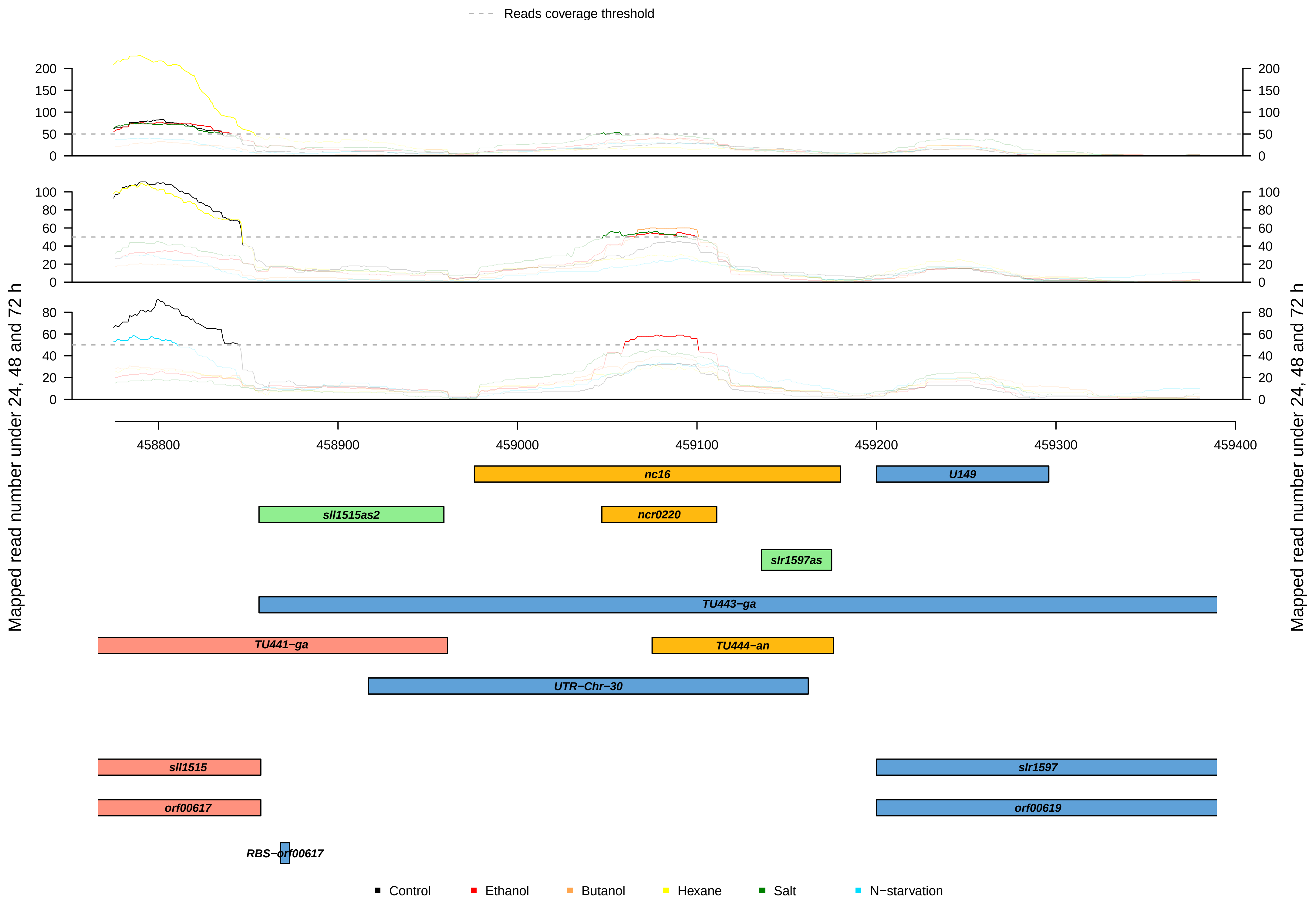

Reads coverage threshold
Mapped read number under 24, 48 and 72 h
200
150
100
50
0
80
60
40
20
458800
458900
459000
459100
459200
459300
459400
nc16
U149
sll1515as2
ncr0220
slr1597as
TU443−ga
TU441−ga
TU444−an
UTR−Chr−30
sll1515
slr1597
orf00617
orf00619
RBS−orf00617
Control
Ethanol
Butanol
Hexane
Salt
N−starvation

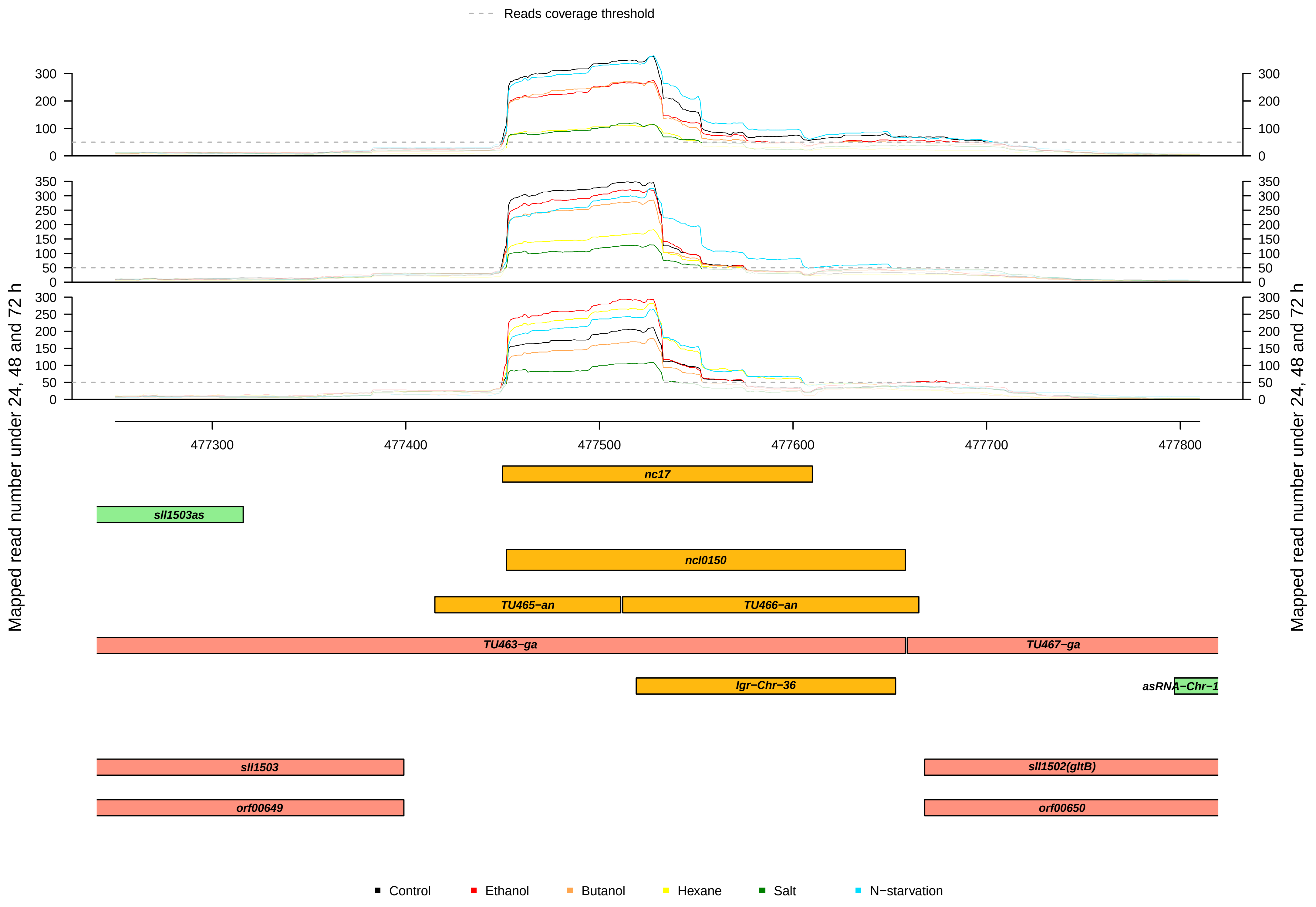

Reads coverage threshold
Mapped read number under 24, 48 and 72 h
477300
477400
477500
477600
477700
477800
nc17
sll1503as
ncl0150
TU465−an
TU466−an
TU463−ga
TU467−ga
Igr−Chr−36
asRNA−Chr−1
sll1503
sll1502(gltB)
orf00649
orf00650
Control
Ethanol
Butanol
Hexane
Salt
N−starvation

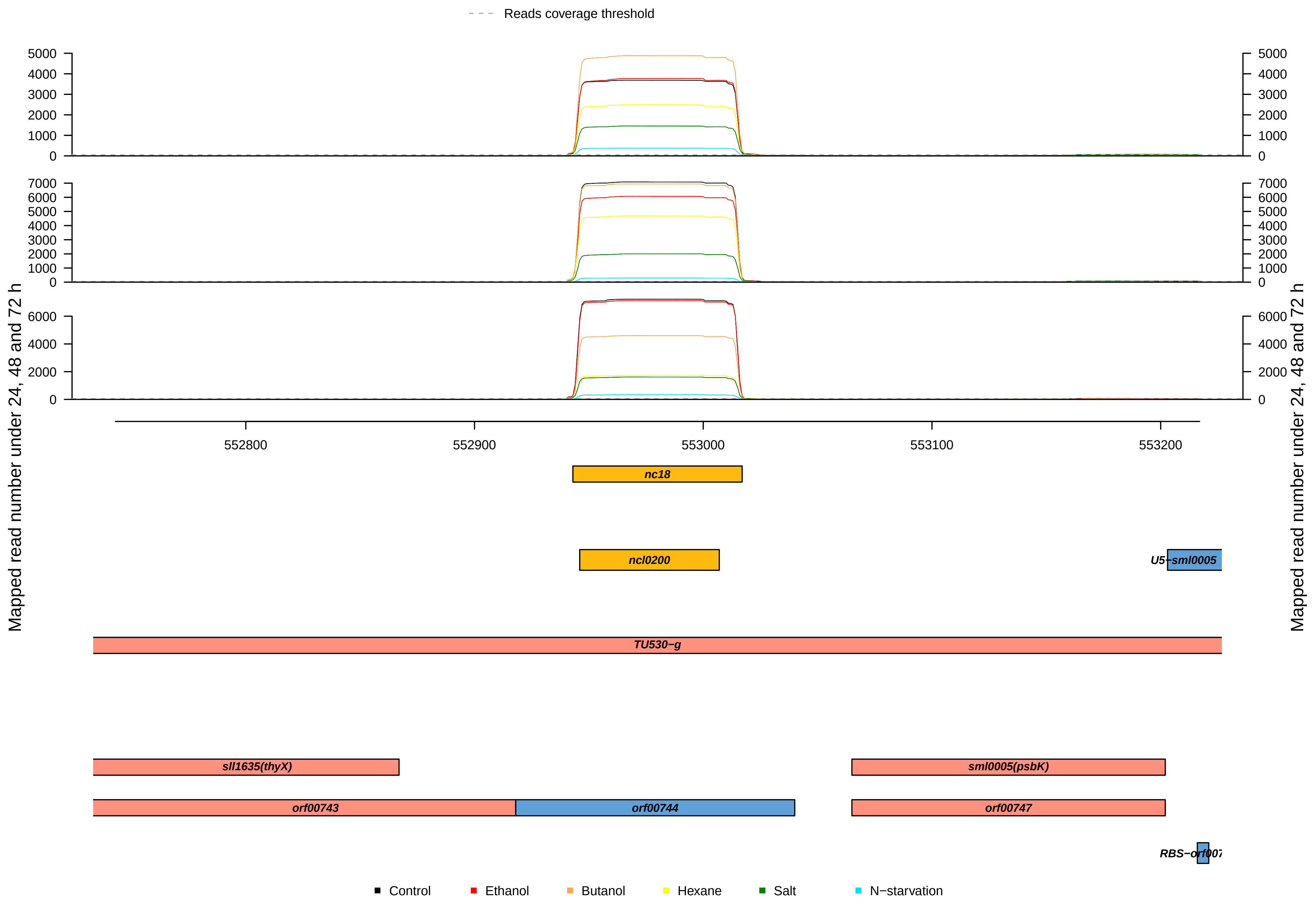

Reads coverage threshold
5000
4000
3000
2000
1000
0
7000
6000
5000
4000
3000
2000
1000
0
6000
4000
2000
0
Mapped read number under 24, 48 and 72 h
552800
552900
553000
553100
553200
nc18
ncl0200
U5−sml0005
TU530−g
sll1635(thyX)
sml0005(psbK)
orf00743
orf00744
orf00747
Control
Ethanol
Butanol
Hexane
Salt
N−starvation

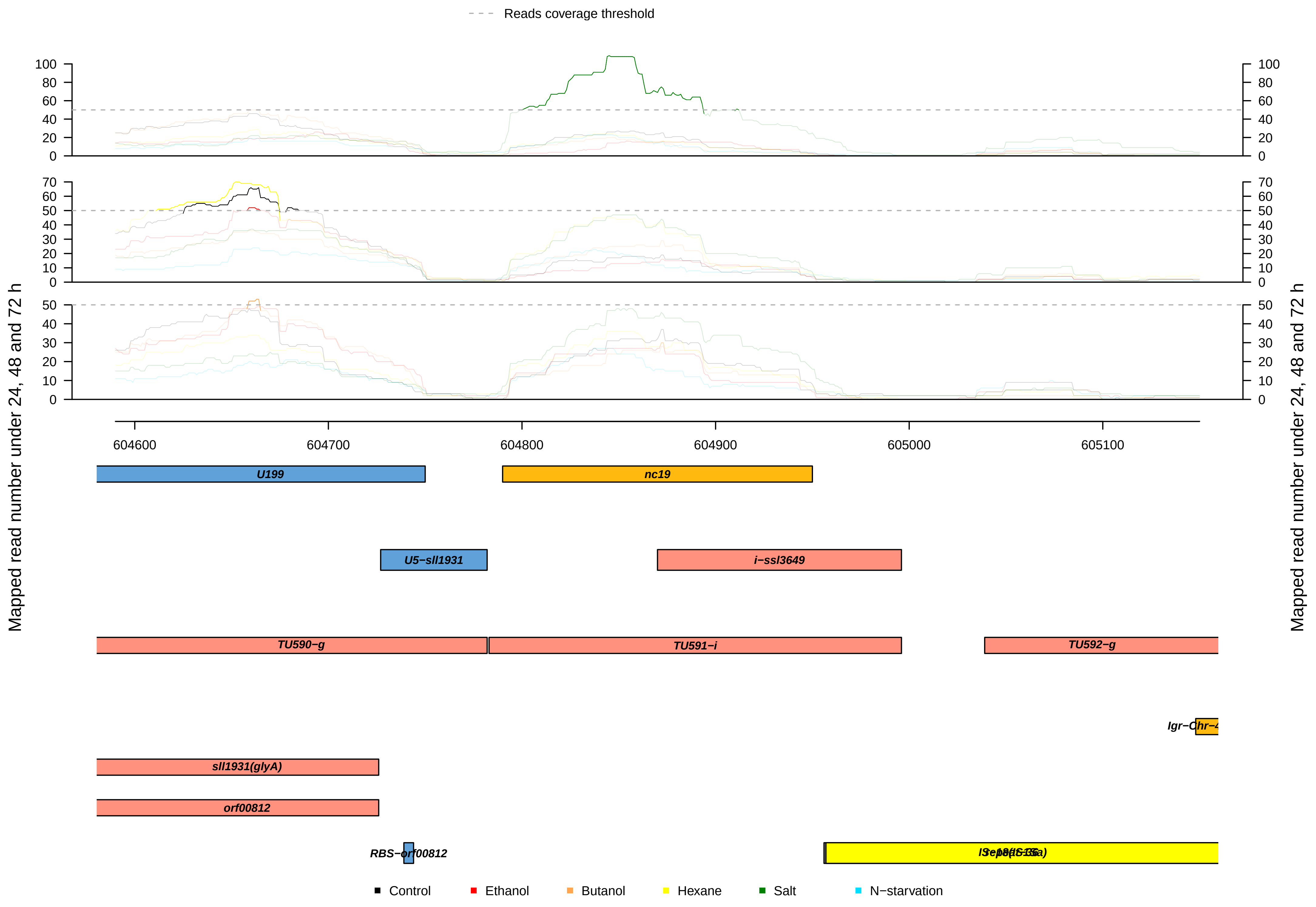
Reads coverage threshold
Mapped read number under 24, 48 and 72 h
604600
604700
604800
604900
605000
605100
U199
nc19
U5−sll1931
i−ssl3649
TU590−g
TU591−i
TU592−g
sll1931(glyA)
orf00812
RBS−orf00812
Control
Ethanol
Butanol
Hexane
Salt
N−starvation

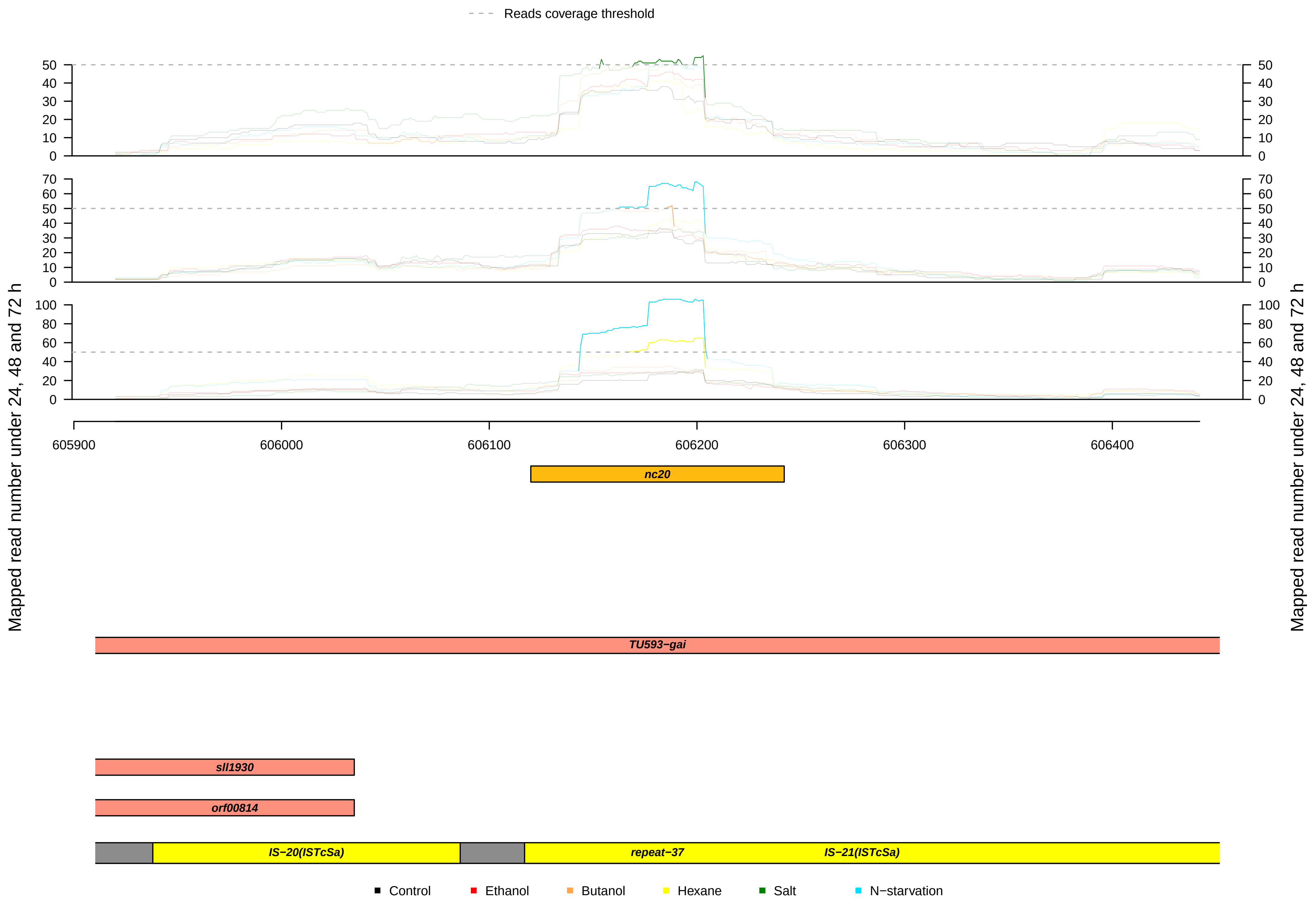
Reads coverage threshold
Mapped read number under 24, 48 and 72 h
605900
606000
606100
606200
606300
606400
nc20
TU593−gai
sll1930
orf00814
IS−20(ISTcSa)
repeat−37
IS−21(ISTcSa)
Control
Ethanol
Butanol
Hexane
Salt
N−starvation

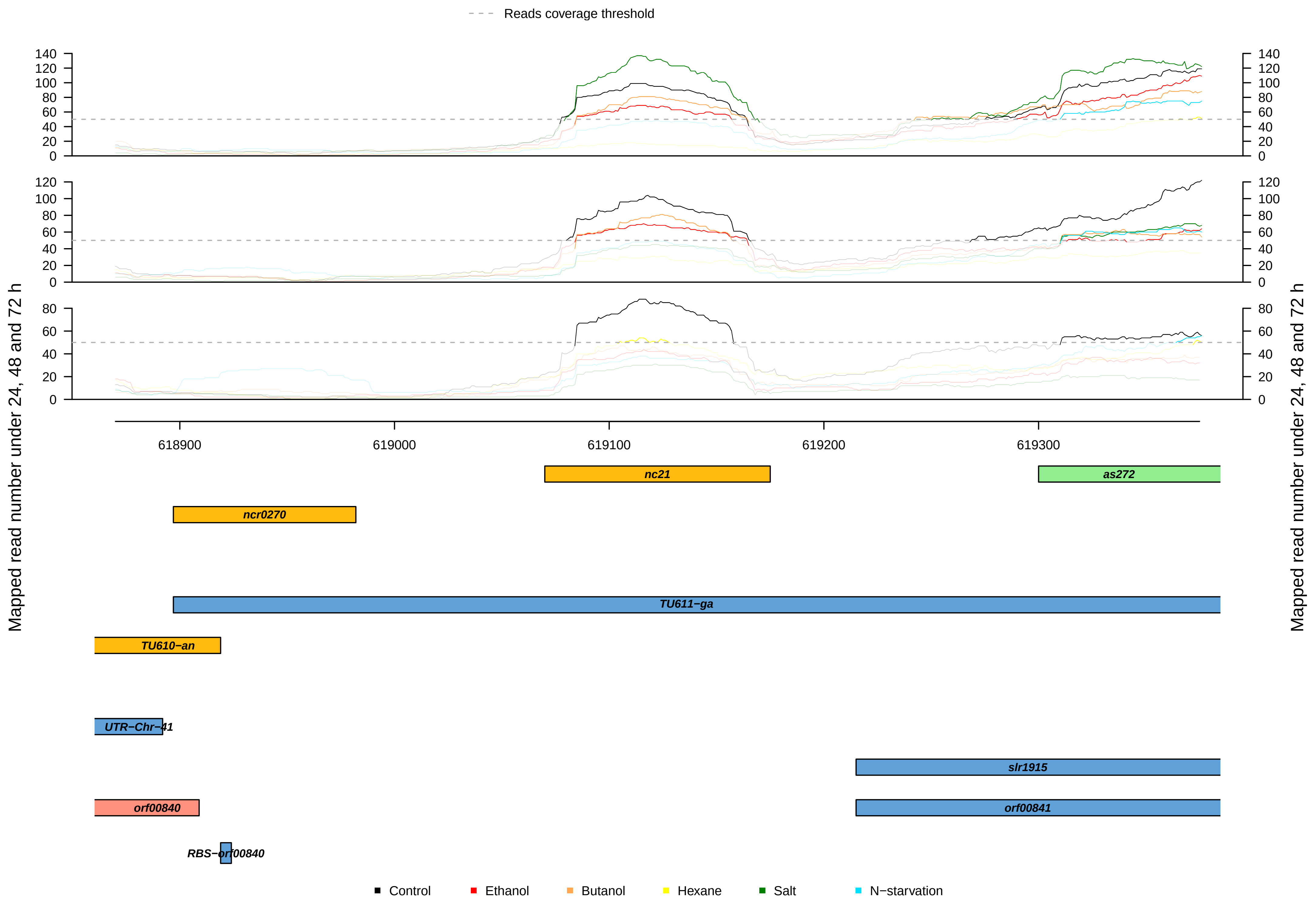

Reads coverage threshold
Mapped read number under 24, 48 and 72 h
618900
619000
619100
619200
619300
nc21
as272
ncr0270
TU611−ga
TU610−an
UTR−Chr−41
slr1915
orf00840
orf00841
RBS−orf00840
Control
Ethanol
Butanol
Hexane
Salt
N−starvation

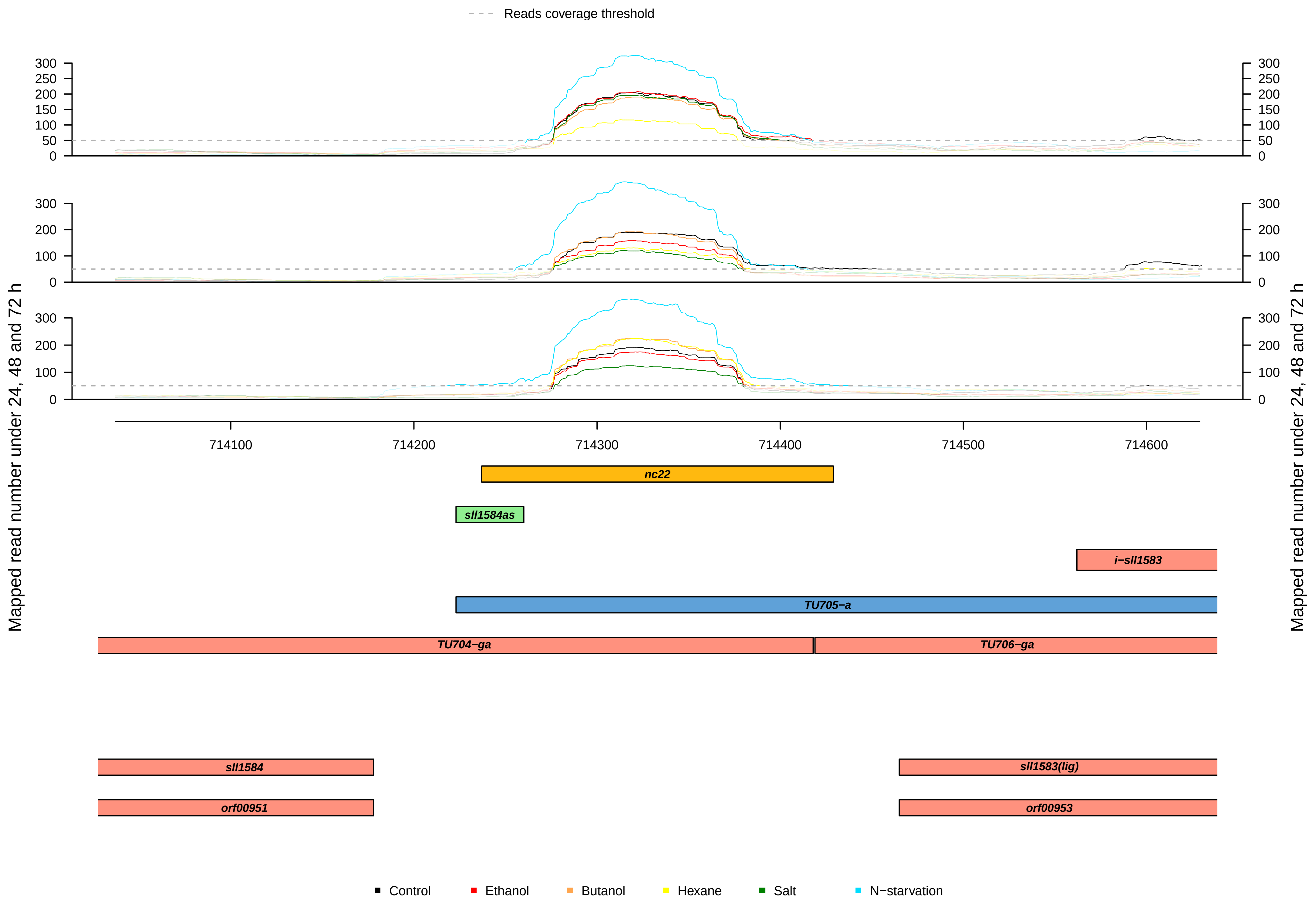

Reads coverage threshold
Mapped read number under 24, 48 and 72 h
0
50
100
150
200
250
300
714100
714200
714300
714400
714500
714600
nc22
sll1584as
i−sll1583
TU705−a
TU704−ga
TU706−ga
sll1584
sll1583(lig)
orf00951
orf00953
Control
Ethanol
Butanol
Hexane
Salt
N−starvation

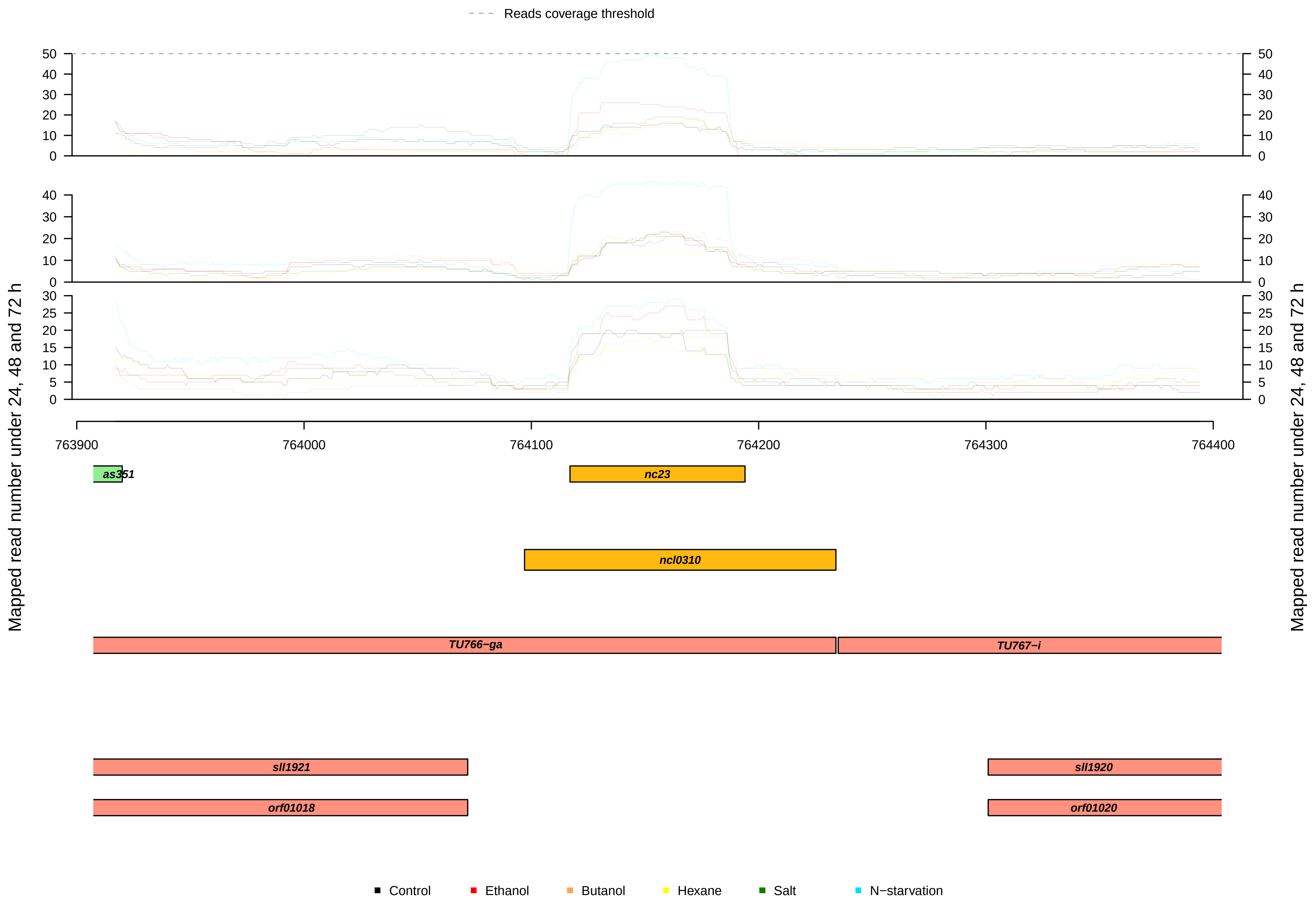

Reads coverage threshold
Mapped read number under 24, 48 and 72 h
763900
764000
764100
764200
764300
764400
as351
nc23
ncl0310
TU766−ga
TU767−i
sll1921
sll1920
orf01018
orf01020
Control
Ethanol
Butanol
Hexane
Salt
N−starvation

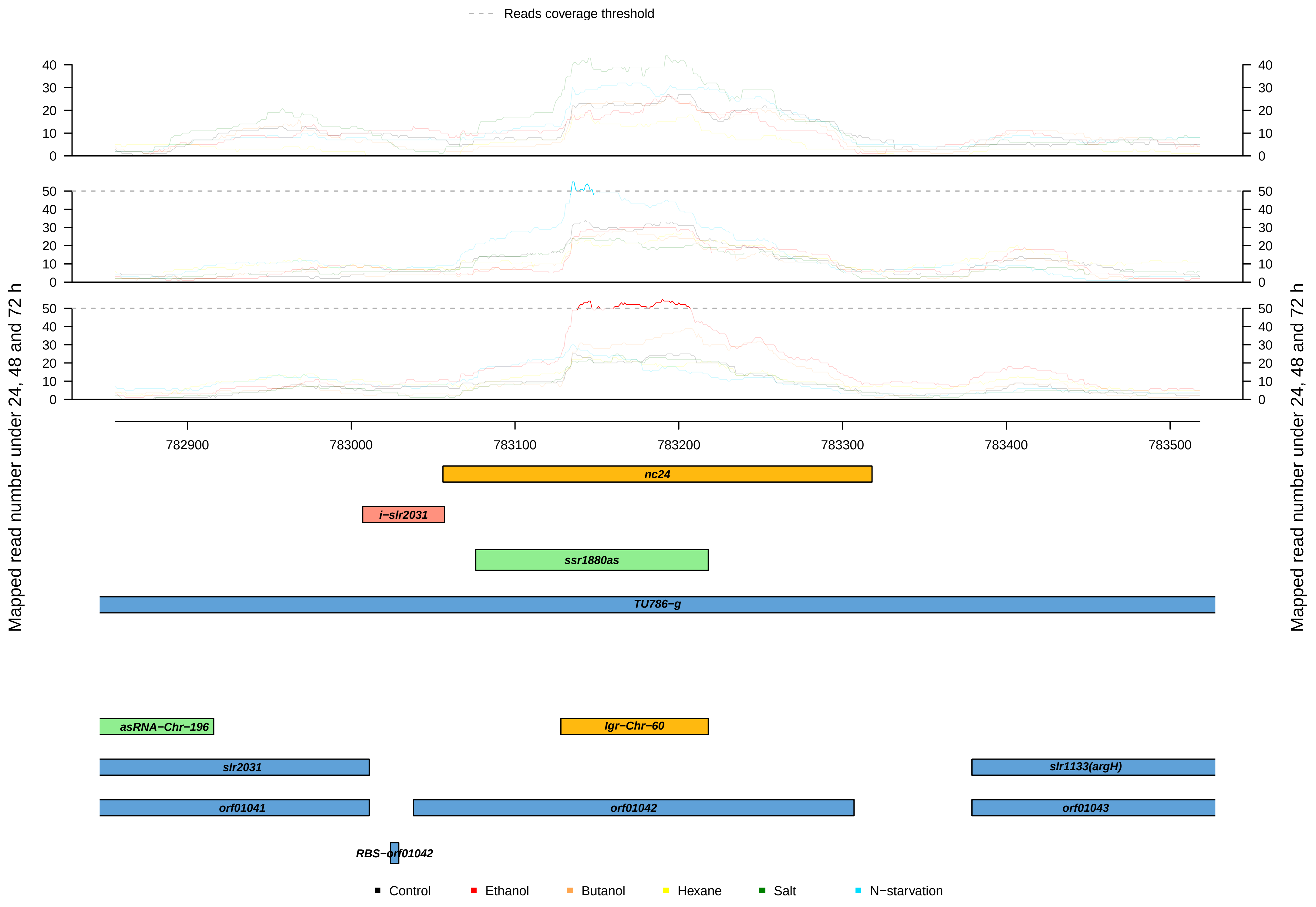

Reads coverage threshold
Mapped read number under 24, 48 and 72 h
782900
783000
783100
783200
783300
783400
783500
nc24
i−slr2031
ssr1880as
TU786−g
asRNA−Chr−196
Igr−Chr−60
slr2031
slr1133(argH)
orf01041
orf01042
orf01043
RBS−orf01042
Control
Ethanol
Butanol
Hexane
Salt
N−starvation

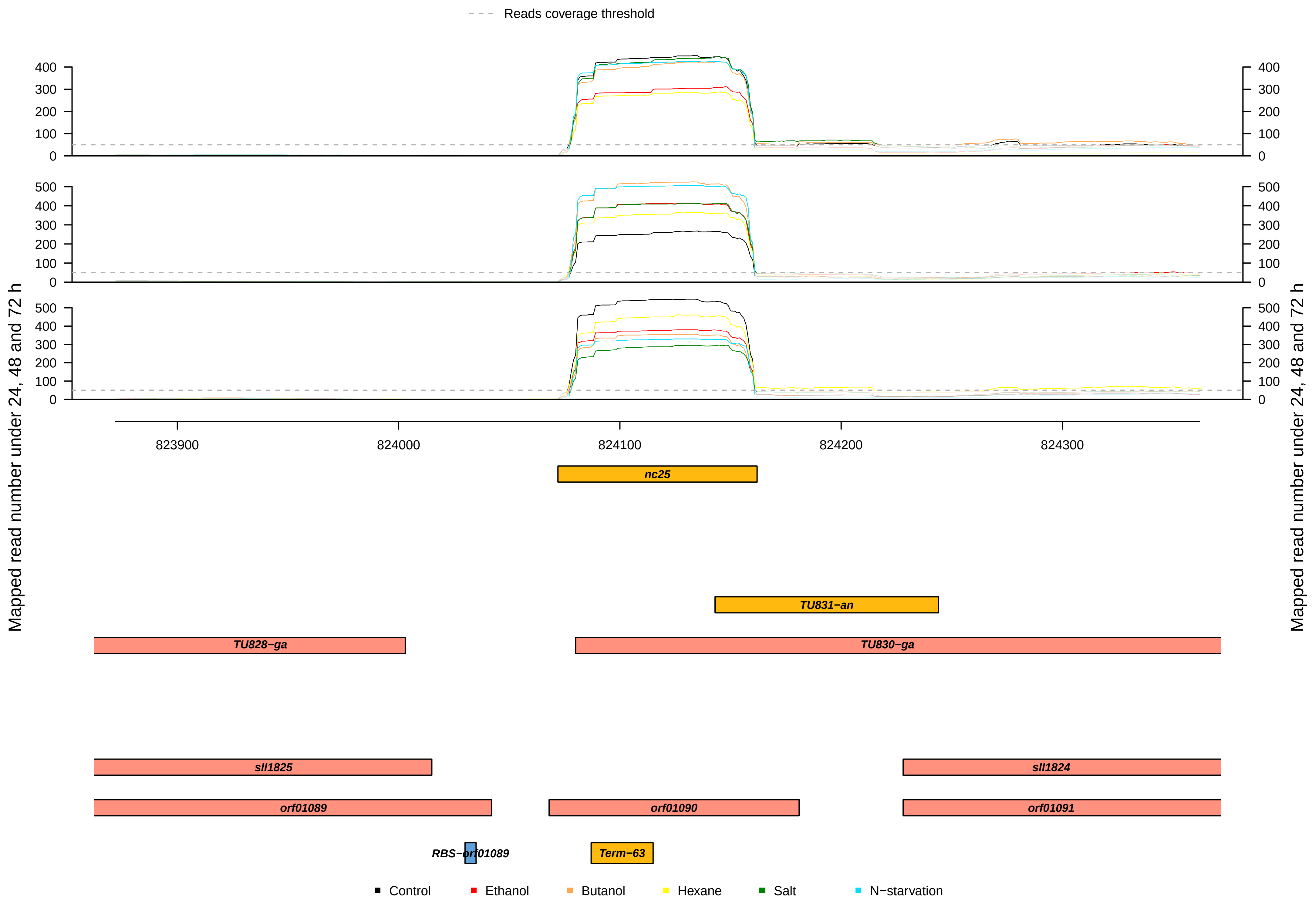

Reads coverage threshold
400
300
200
100
0
500
Mapped read number under 24, 48 and 72 h
823900
824000
824100
824200
824300
nc25
TU831−an
TU828−ga
TU830−ga
sll1825
sll1824
orf01089
orf01090
orf01091
RBS−orf01089
Term−63
Control
Ethanol
Butanol
Hexane
Salt
N−starvation

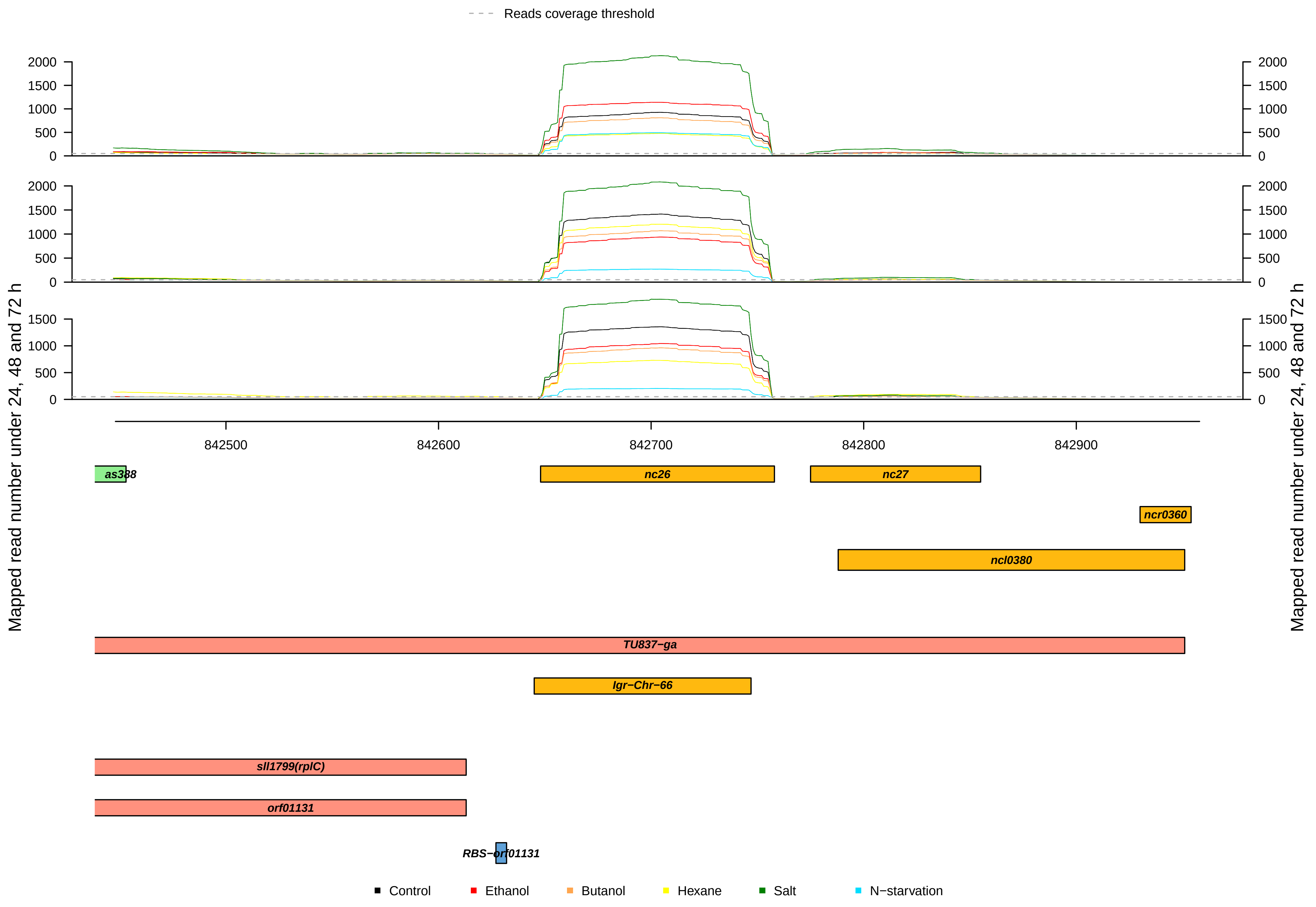

Reads coverage threshold
2000
1500
1000
500
0
Mapped read number under 24, 48 and 72 h
842500
842600
842700
842800
842900
as388
nc26
nc27
ncr0360
ncl0380
TU837−ga
Igr−Chr−66
sll1799(rplC)
orf01131
RBS−orf01131
Control
Ethanol
Butanol
Hexane
Salt
N−starvation

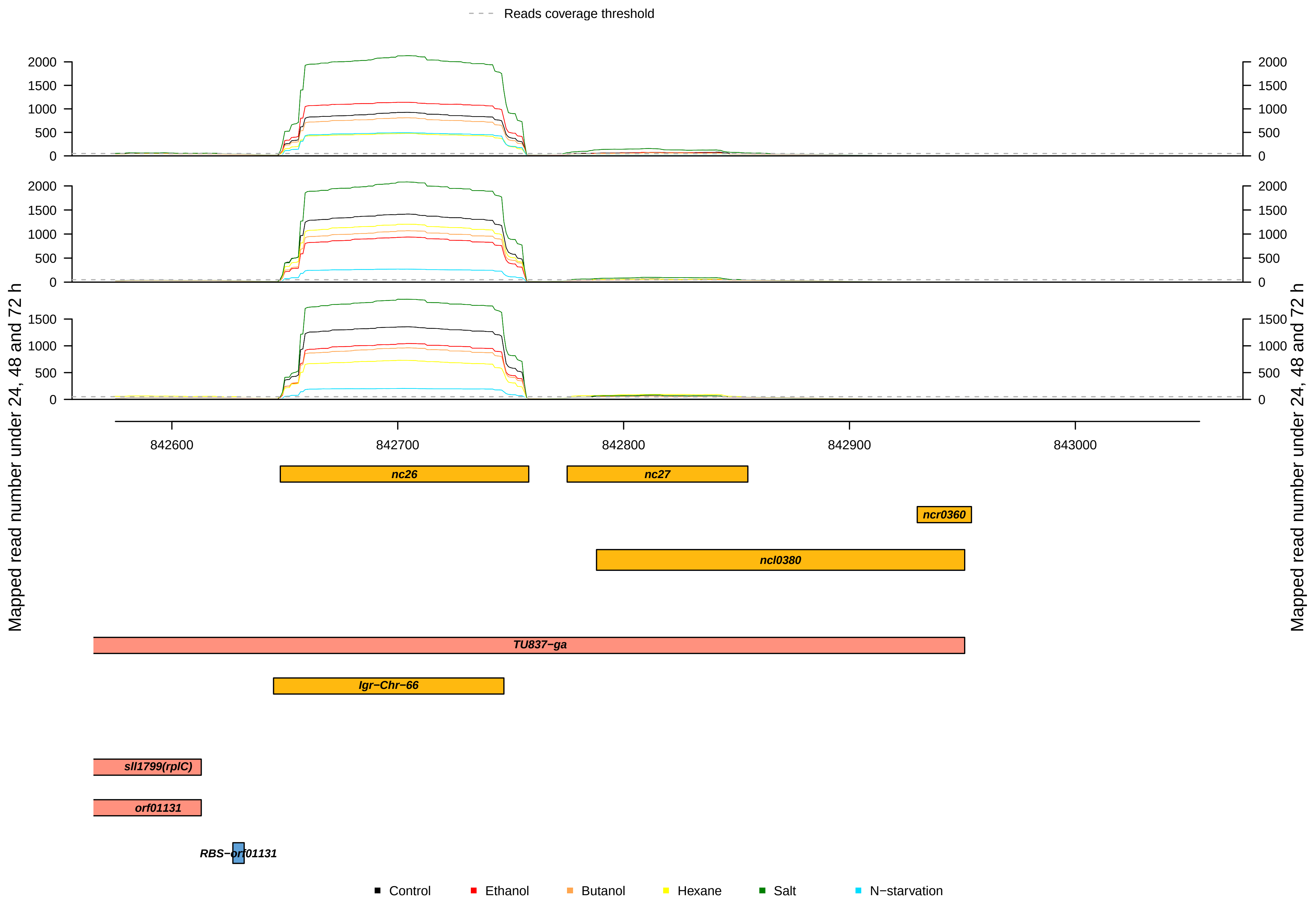

Reads coverage threshold
2000
1500
1000
500
0
Mapped read number under 24, 48 and 72 h
842600
842700
842800
842900
843000
nc26
nc27
ncr0360
ncl0380
TU837−ga
Igr−Chr−66
sll1799(rplC)
orf01131
RBS−orf01131
Control
Ethanol
Butanol
Hexane
Salt
N−starvation

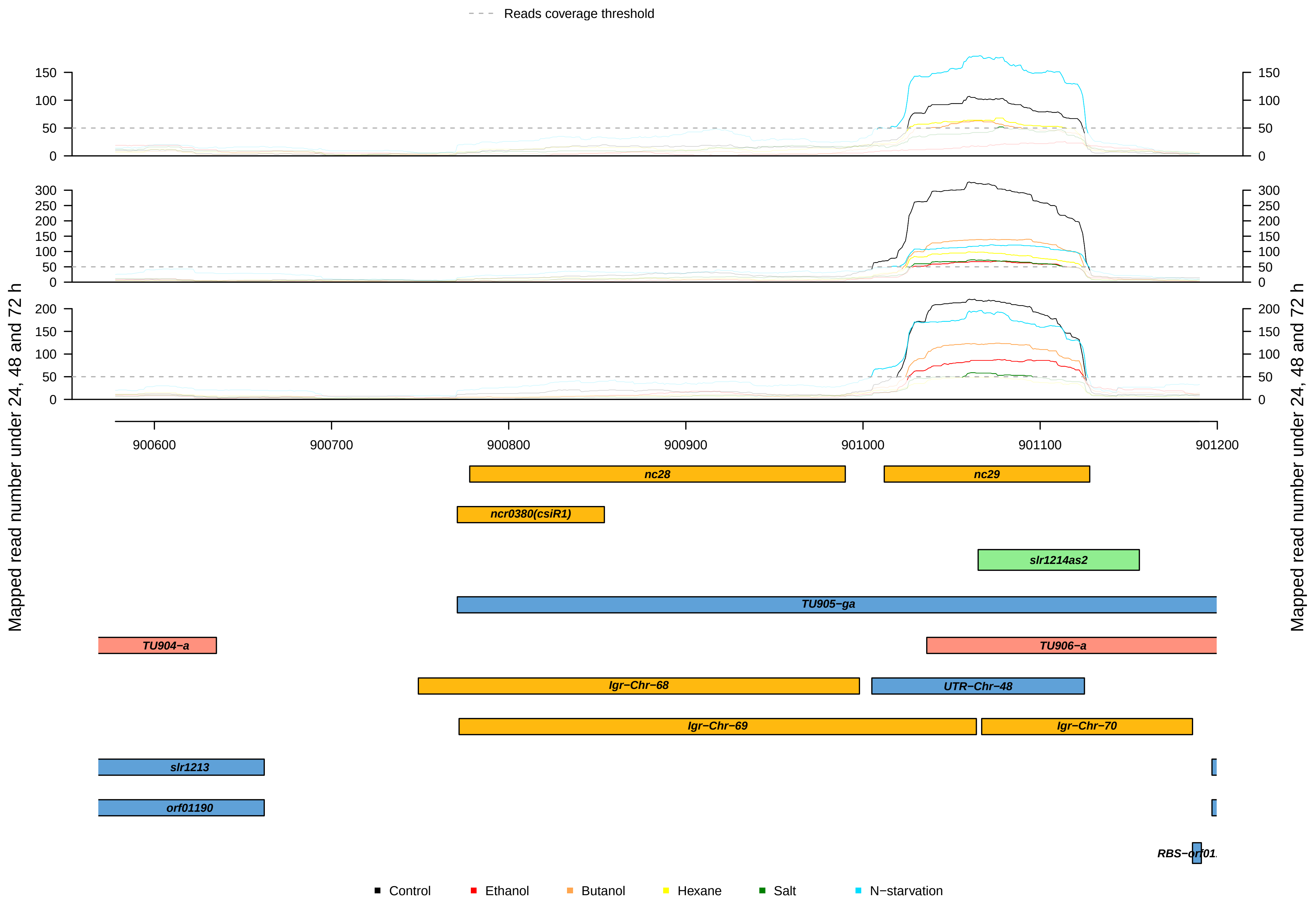

Reads coverage threshold
Mapped read number under 24, 48 and 72 h
900600
900700
900800
900900
901000
901100
901200
nc28
nc29
ncr0380(csiR1)
slr1214as2
TU905−ga
TU904−a
TU906−a
Igr−Chr−68
UTR−Chr−48
Igr−Chr−69
Igr−Chr−70
slr1213
orf01190
Control
Ethanol
Butanol
Hexane
Salt
N−starvation

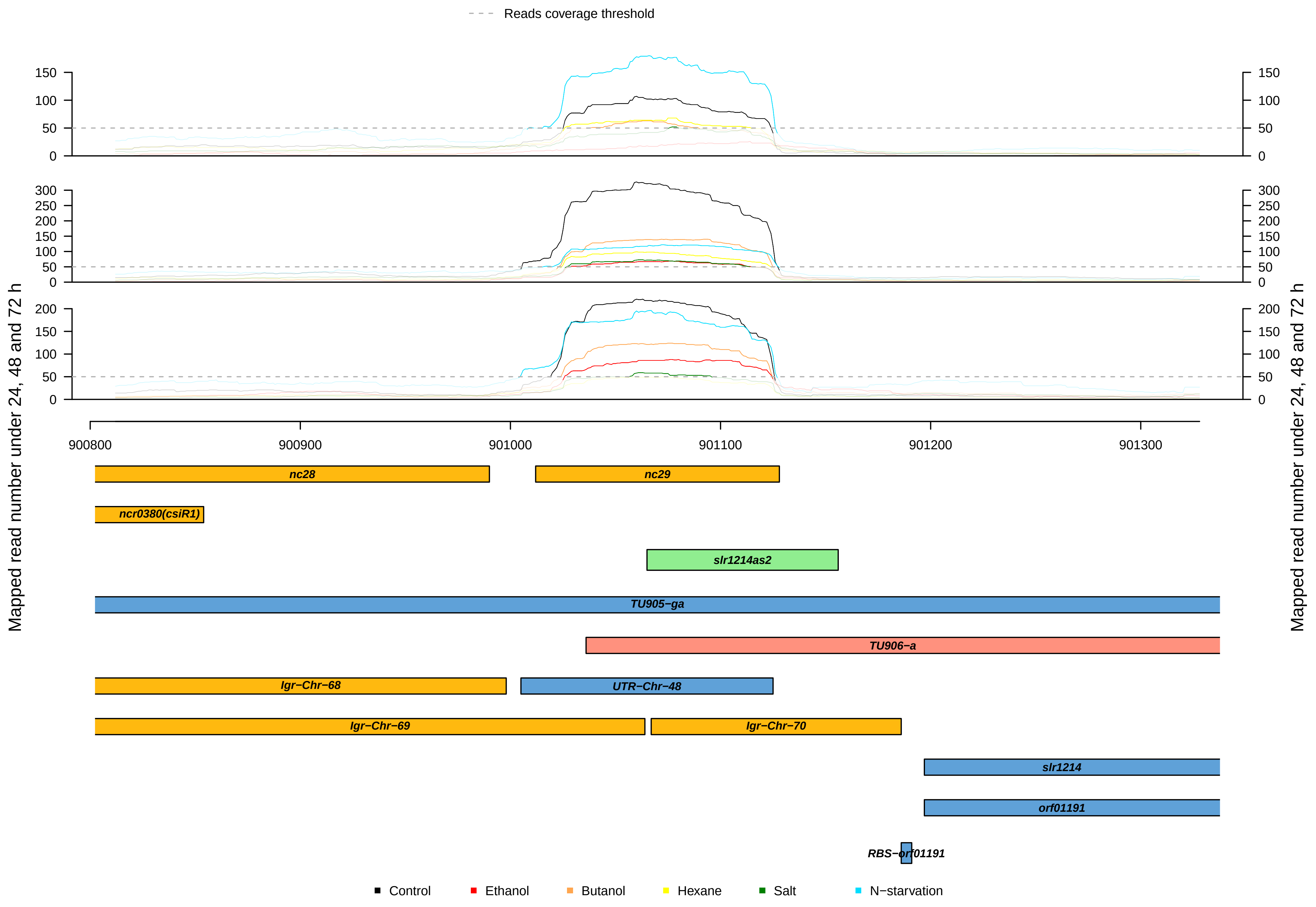
Reads coverage threshold
Mapped read number under 24, 48 and 72 h
150
100
50
0
300
250
200
150
100
50
0
200
150
100
50
0
900800
900900
901000
901100
901200
901300
nc28
nc29
ncr0380(csiR1)
slr1214as2
TU905−ga
TU906−a
Igr−Chr−68
UTR−Chr−48
Igr−Chr−69
Igr−Chr−70
slr1214
orf01191
RBS−orf01191
Control
Ethanol
Butanol
Hexane
Salt
N−starvation

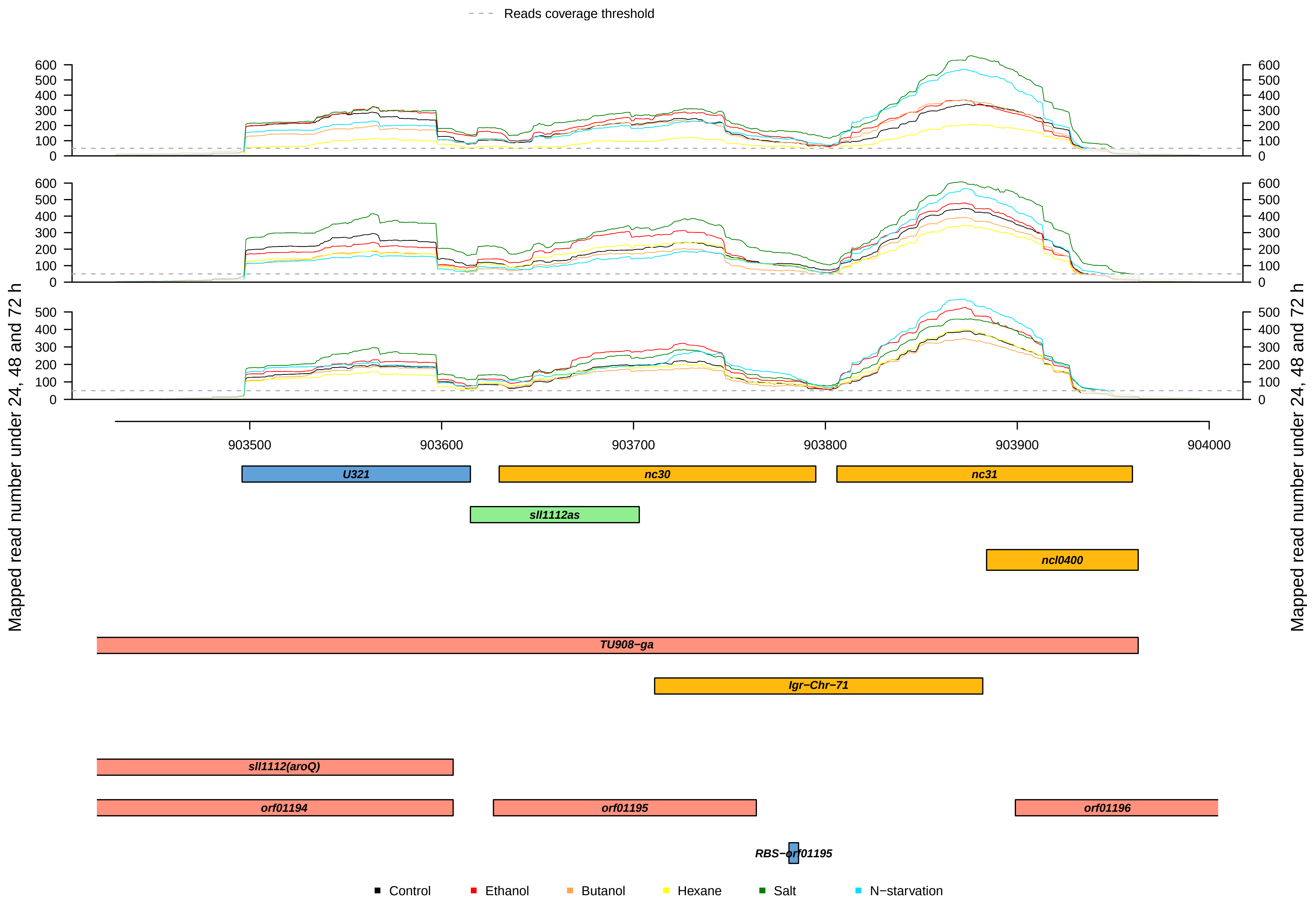

Reads coverage threshold
Mapped read number under 24, 48 and 72 h
600
500
400
300
200
100
0
903500
903600
903700
903800
903900
904000
U321
nc30
nc31
sll1112as
ncl0400
TU908−ga
Igr−Chr−71
sll1112(aroQ)
orf01194
orf01195
orf01196
RBS−orf01195
Control
Ethanol
Butanol
Hexane
Salt
N−starvation

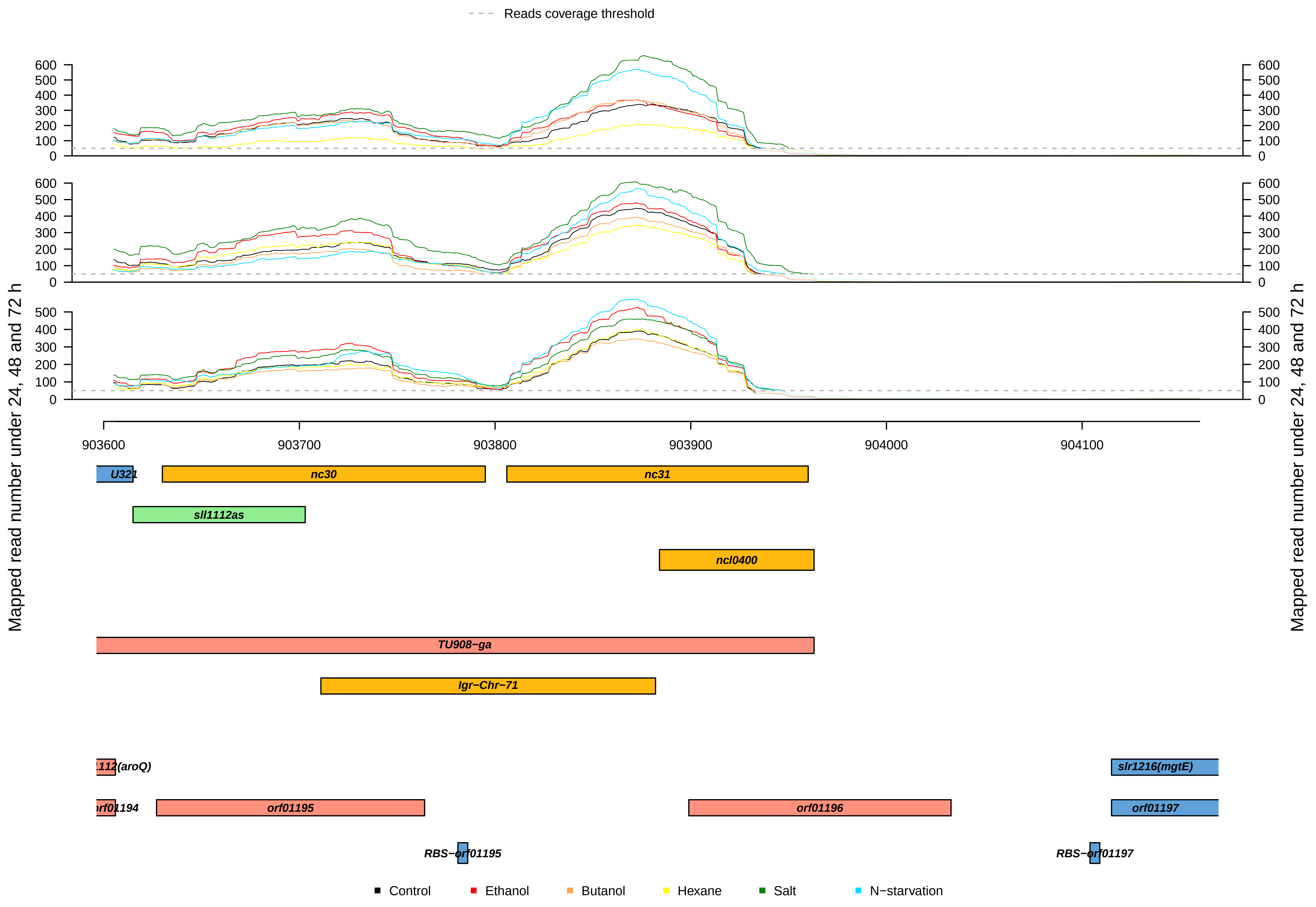
Reads coverage threshold
600
500
400
300
200
100
0
Mapped read number under 24, 48 and 72 h
903600
903700
903800
903900
904000
904100
U321
nc30
nc31
sll1112as
ncl0400
TU908−ga
Igr−Chr−71
112(aroQ)
slr1216(mgtE)
rf01194
orf01195
orf01196
orf01197
RBS−orf01195
RBS−orf01197
Control
Ethanol
Butanol
Hexane
Salt
N−starvation

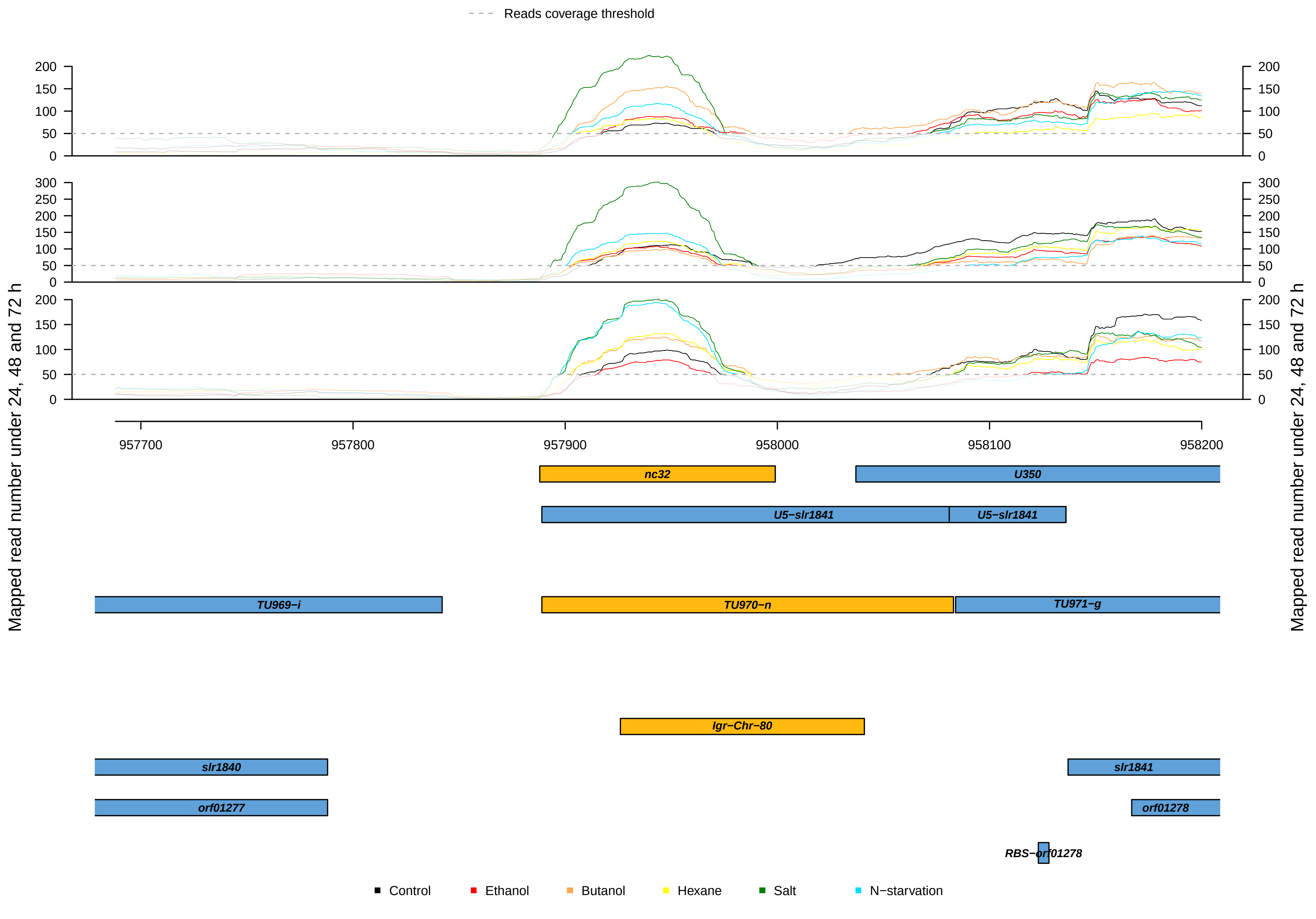
Reads coverage threshold
Mapped read number under 24, 48 and 72 h
957700
957800
957900
958000
958100
958200
nc32
U350
U5−slr1841
U5−slr1841
TU969−i
TU970−n
TU971−g
Igr−Chr−80
slr1840
slr1841
orf01277
orf01278
RBS−orf01278
Control
Ethanol
Butanol
Hexane
Salt
N−starvation

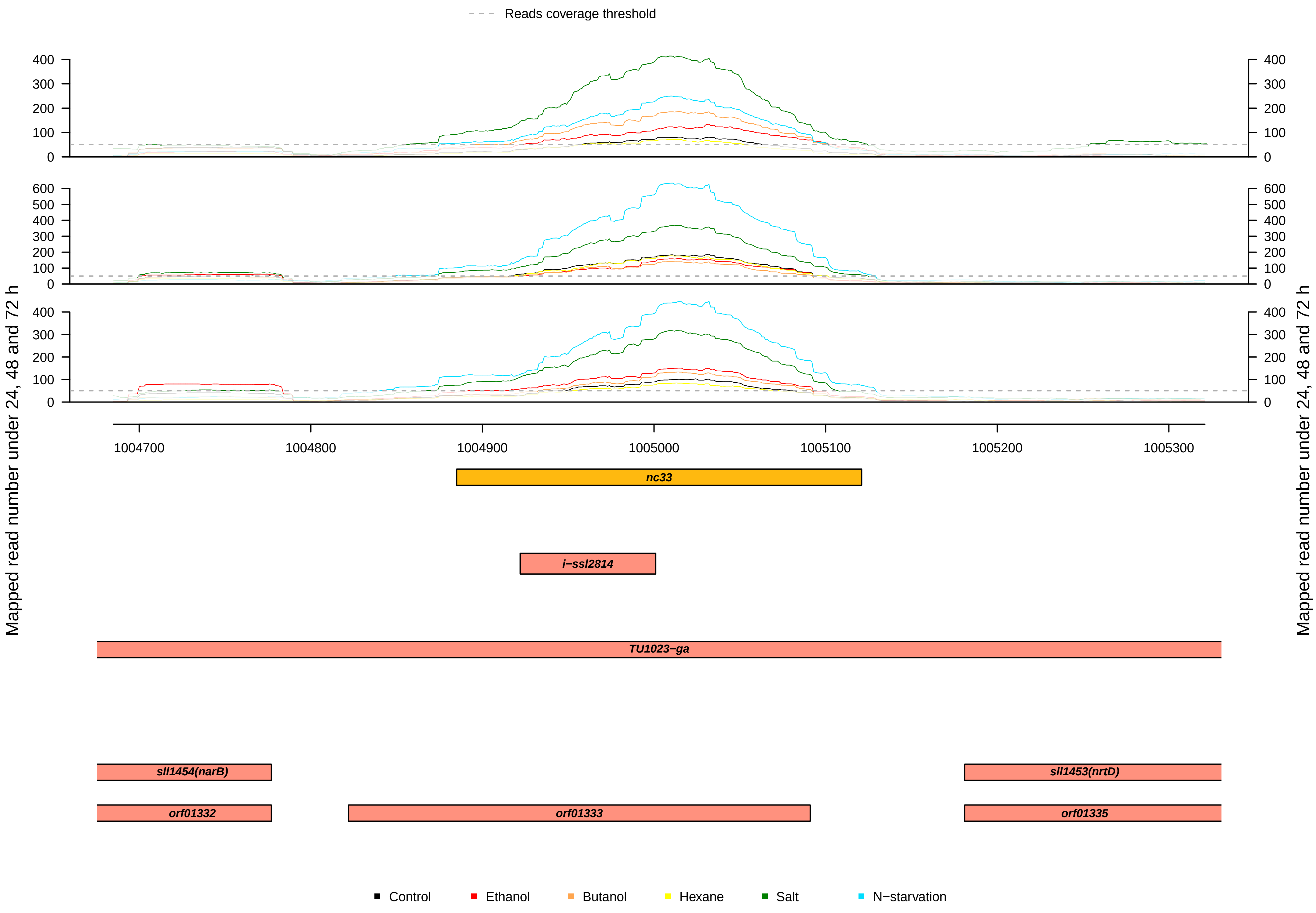

Reads coverage threshold
Mapped read number under 24, 48 and 72 h
400
300
200
100
0
600
500
400
300
200
100
0
400
300
200
100
0
1004700
1004800
1004900
1005000
1005100
1005200
1005300
nc33
i−ssl2814
TU1023−ga
sll1454(narB)
sll1453(nrtD)
orf01332
orf01333
orf01335
Control
Ethanol
Butanol
Hexane
Salt
N−starvation

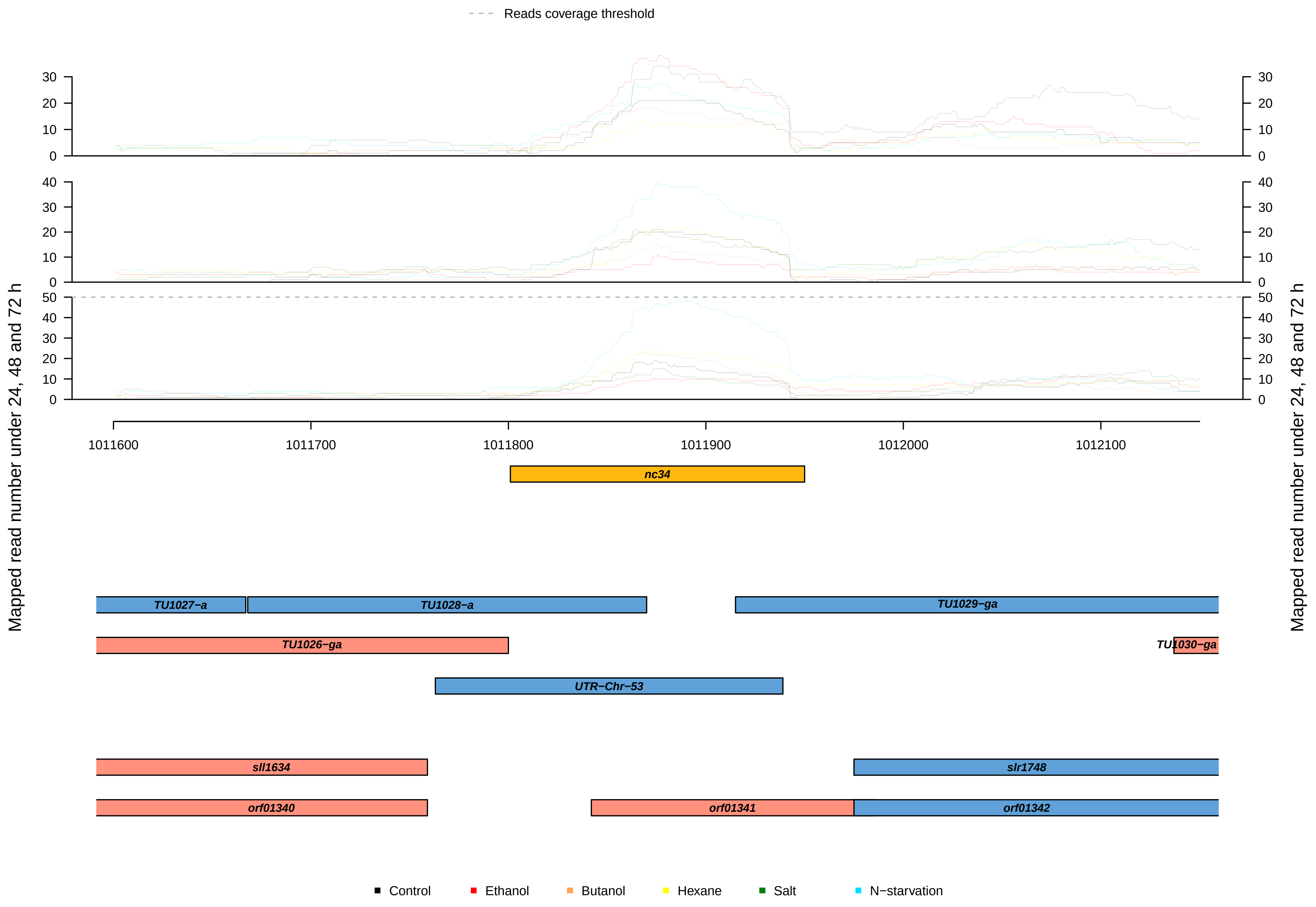

Reads coverage threshold
Mapped read number under 24, 48 and 72 h
1011600
1011700
1011800
1011900
1012000
1012100
nc34
TU1027−a
TU1028−a
TU1029−ga
TU1026−ga
TU1030−ga
UTR−Chr−53
sll1634
slr1748
orf01340
orf01341
orf01342
Control
Ethanol
Butanol
Hexane
Salt
N−starvation

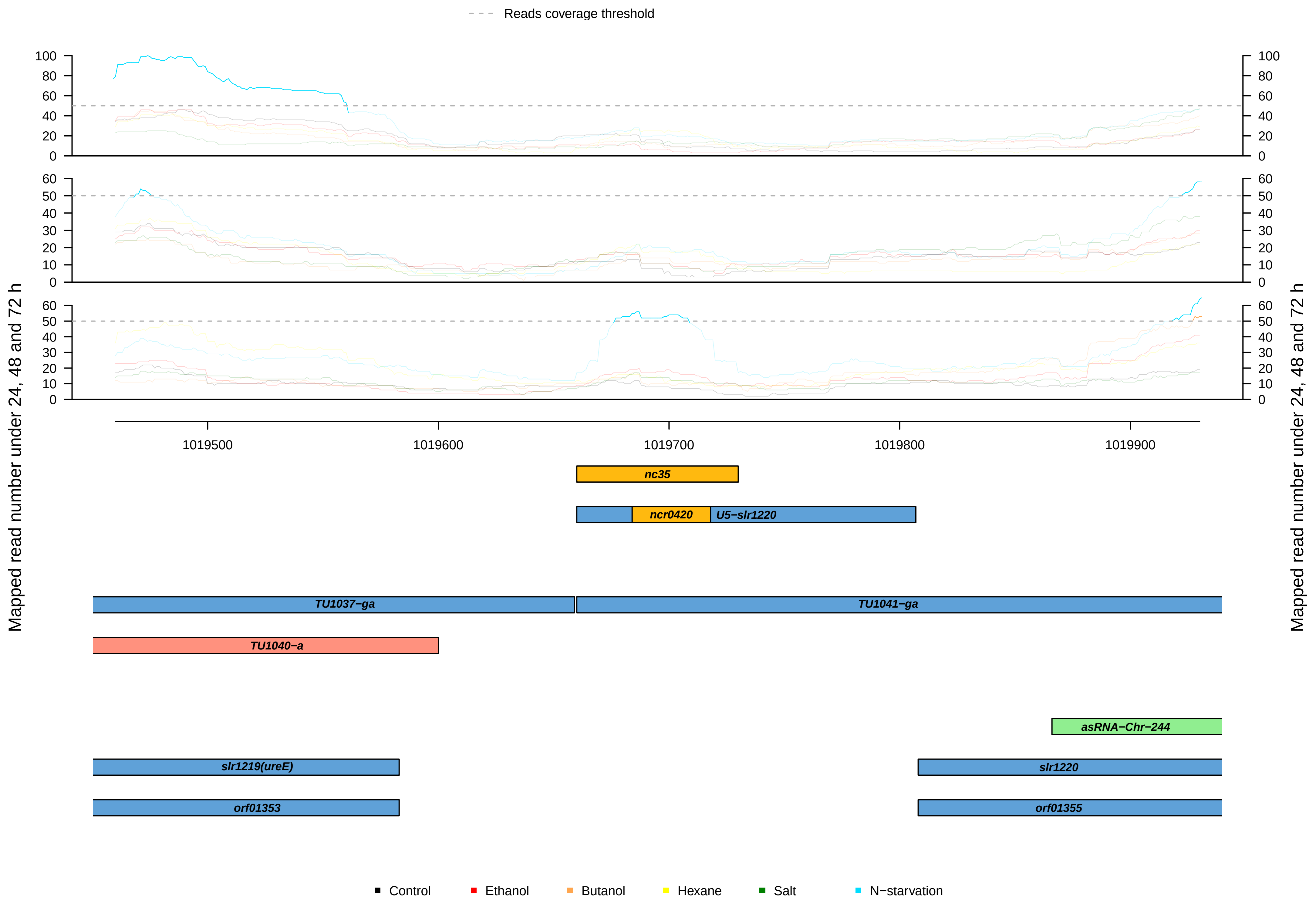

Reads coverage threshold
Mapped read number under 24, 48 and 72 h
100
80
60
40
20
0
60
50
40
30
20
10
0
1019500
1019600
1019700
1019800
1019900
nc35
ncr0420
U5−slr1220
TU1037−ga
TU1041−ga
TU1040−a
asRNA−Chr−244
slr1219(ureE)
slr1220
orf01353
orf01355
Control
Ethanol
Butanol
Hexane
Salt
N−starvation

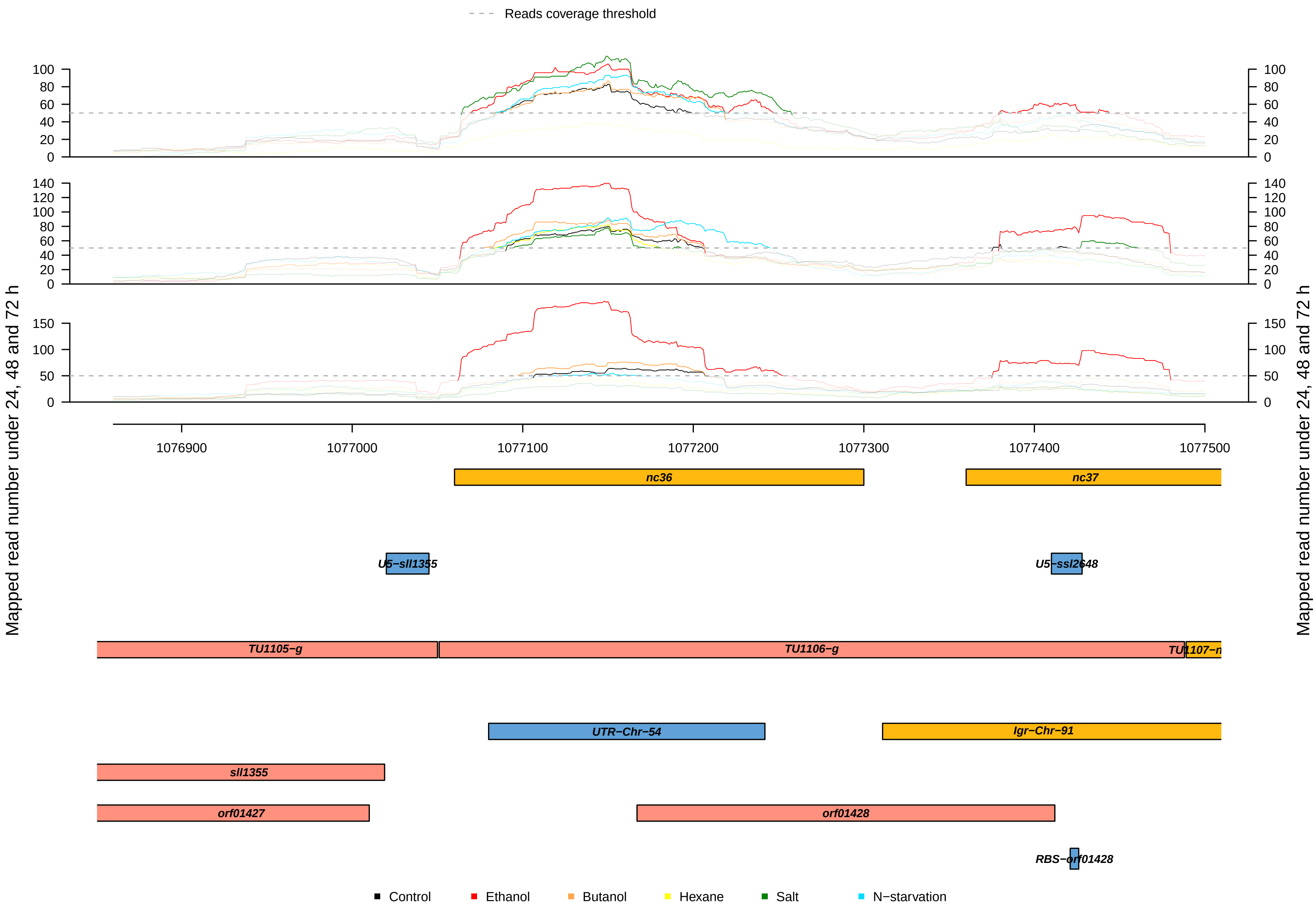

Reads coverage threshold
Mapped read number under 24, 48 and 72 h
100
80
60
40
20
0
140
120
150
50
1076900
1077000
1077100
1077200
1077300
1077400
1077500
nc36
nc37
U5−sll1355
U5−ssl2648
TU1105−g
TU1106−g
TU1107−n
UTR−Chr−54
Igr−Chr−91
sll1355
orf01427
orf01428
RBS−orf01428
Control
Ethanol
Butanol
Hexane
Salt
N−starvation

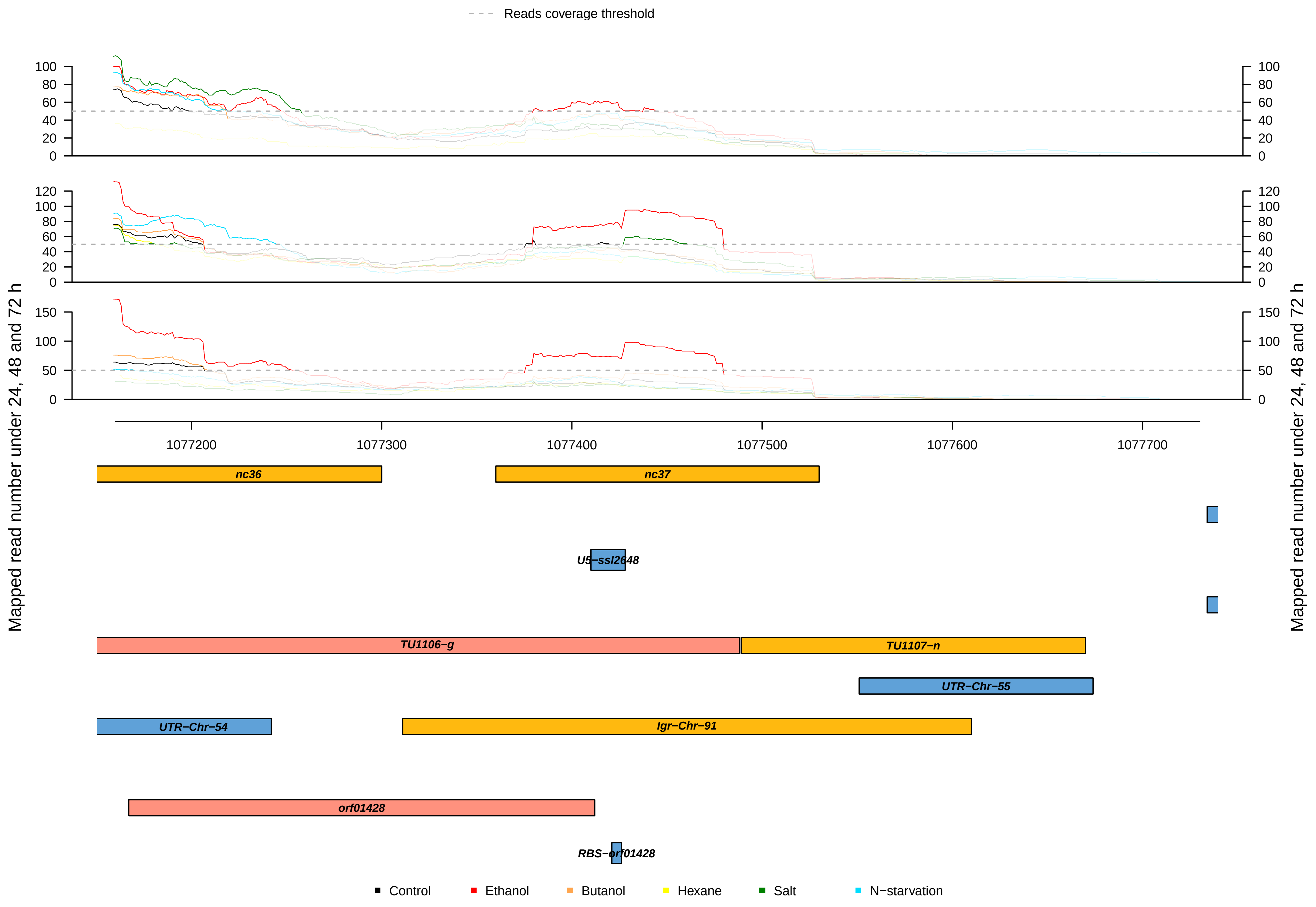

Reads coverage threshold
Mapped read number under 24, 48 and 72 h
1077200
1077300
1077400
1077500
1077600
1077700
nc36
nc37
U5−ssl2648
TU1106−g
TU1107−n
UTR−Chr−55
UTR−Chr−54
Igr−Chr−91
orf01428
RBS−orf01428
Control
Ethanol
Butanol
Hexane
Salt
N−starvation

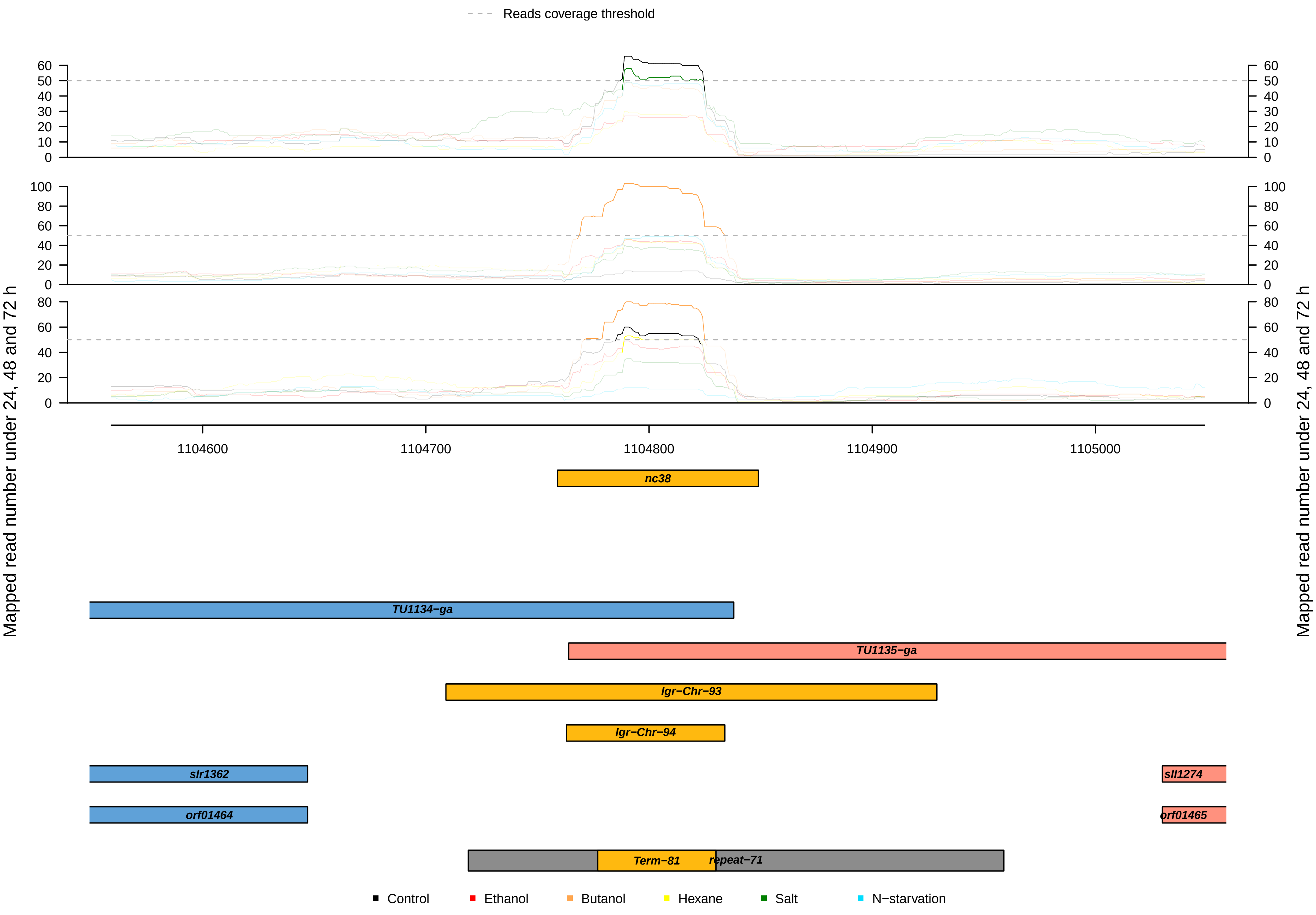

Reads coverage threshold
Mapped read number under 24, 48 and 72 h
1104600
1104700
1104800
1104900
1105000
nc38
TU1134−ga
TU1135−ga
Igr−Chr−93
Igr−Chr−94
slr1362
sll1274
orf01464
orf01465
Term−81
repeat−71
Control
Ethanol
Butanol
Hexane
Salt
N−starvation

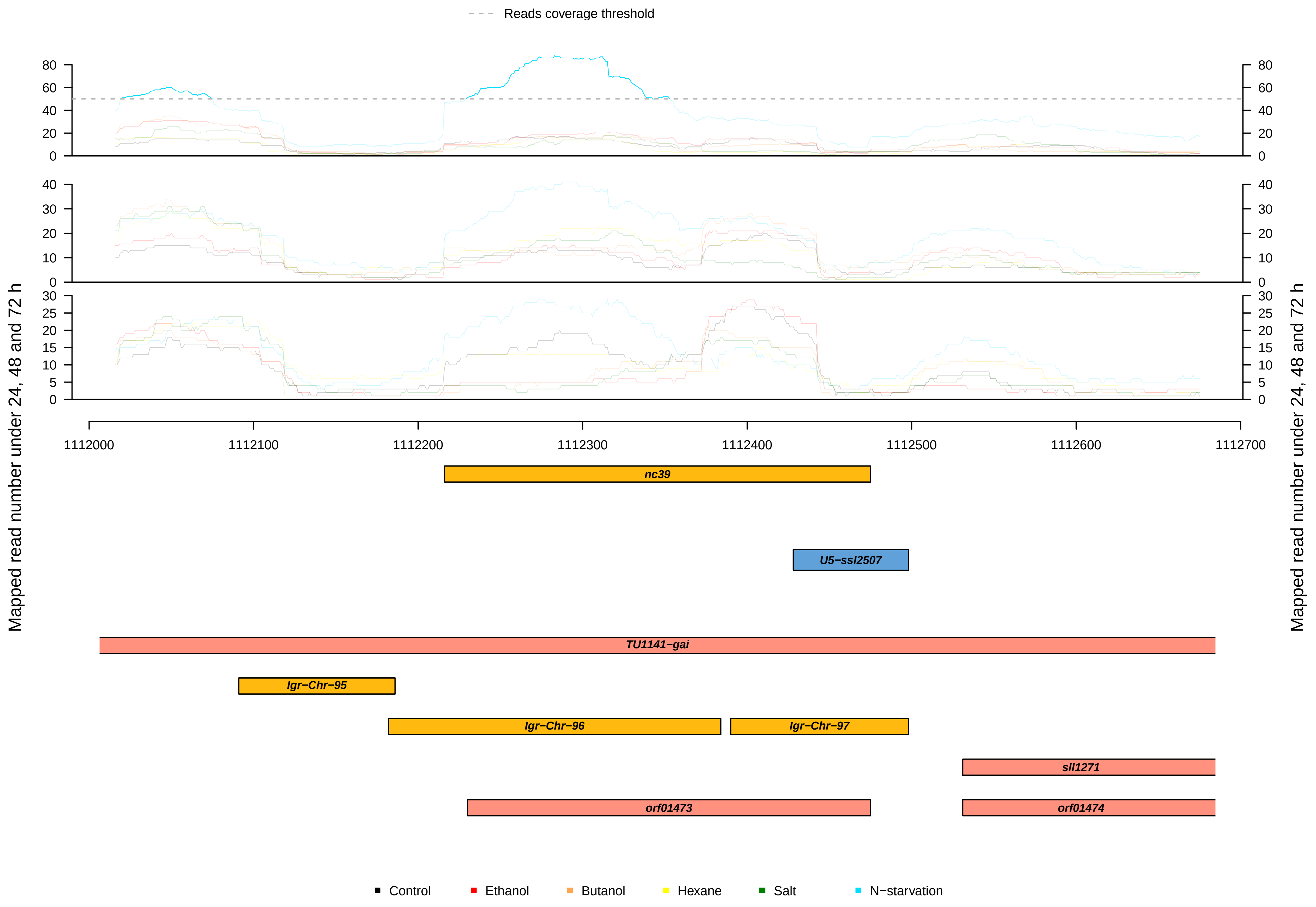

Reads coverage threshold
Mapped read number under 24, 48 and 72 h
1112000
1112100
1112200
1112300
1112400
1112500
1112600
1112700
nc39
U5−ssl2507
TU1141−gai
Igr−Chr−95
Igr−Chr−96
Igr−Chr−97
sll1271
orf01473
orf01474
Control
Ethanol
Butanol
Hexane
Salt
N−starvation

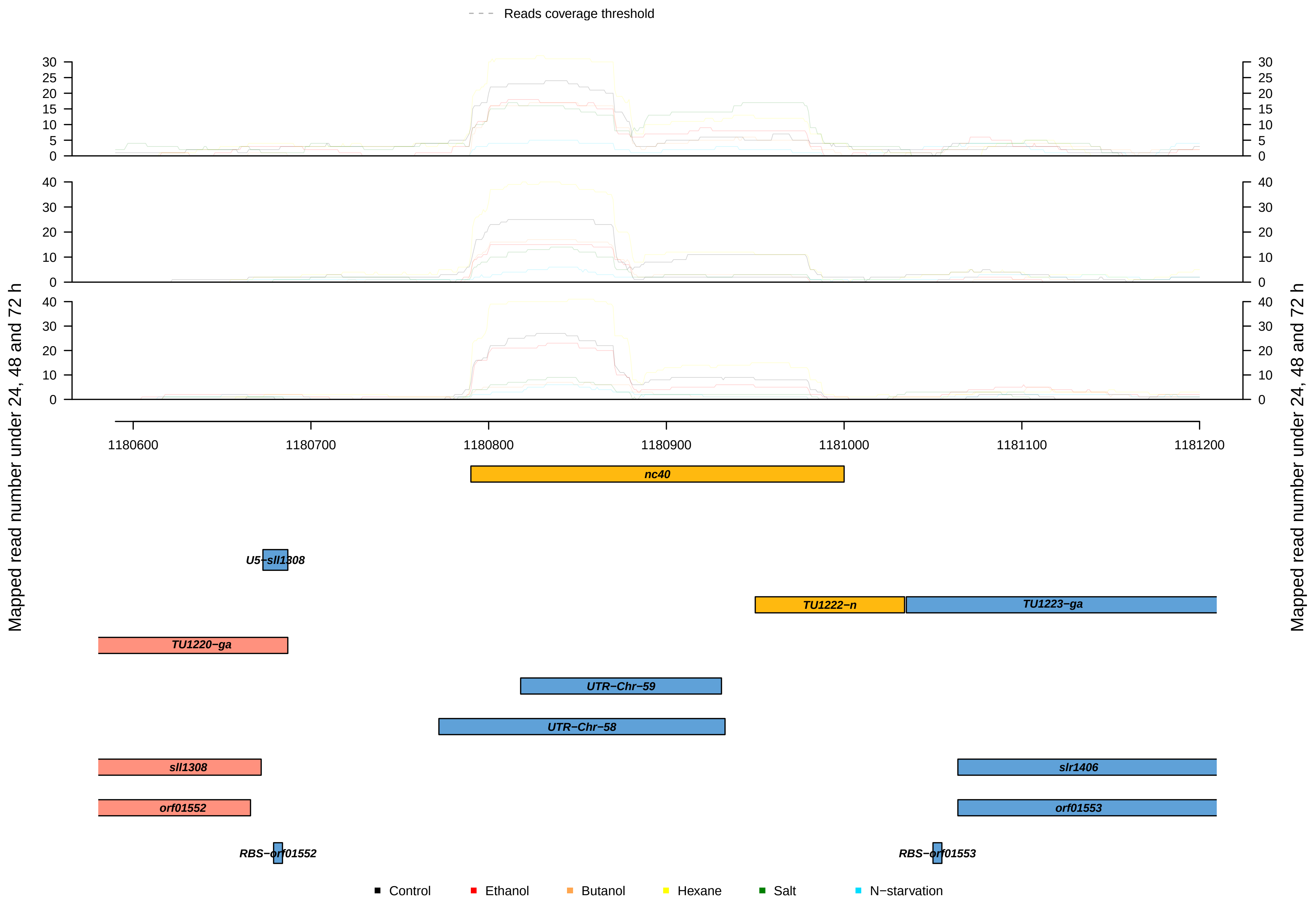

Reads coverage threshold
Mapped read number under 24, 48 and 72 h
1180600
1180700
1180800
1180900
1181000
1181100
1181200
nc40
U5−sll1308
TU1222−n
TU1223−ga
TU1220−ga
UTR−Chr−59
UTR−Chr−58
sll1308
slr1406
orf01552
orf01553
RBS−orf01552
RBS−orf01553
Control
Ethanol
Butanol
Hexane
Salt
N−starvation

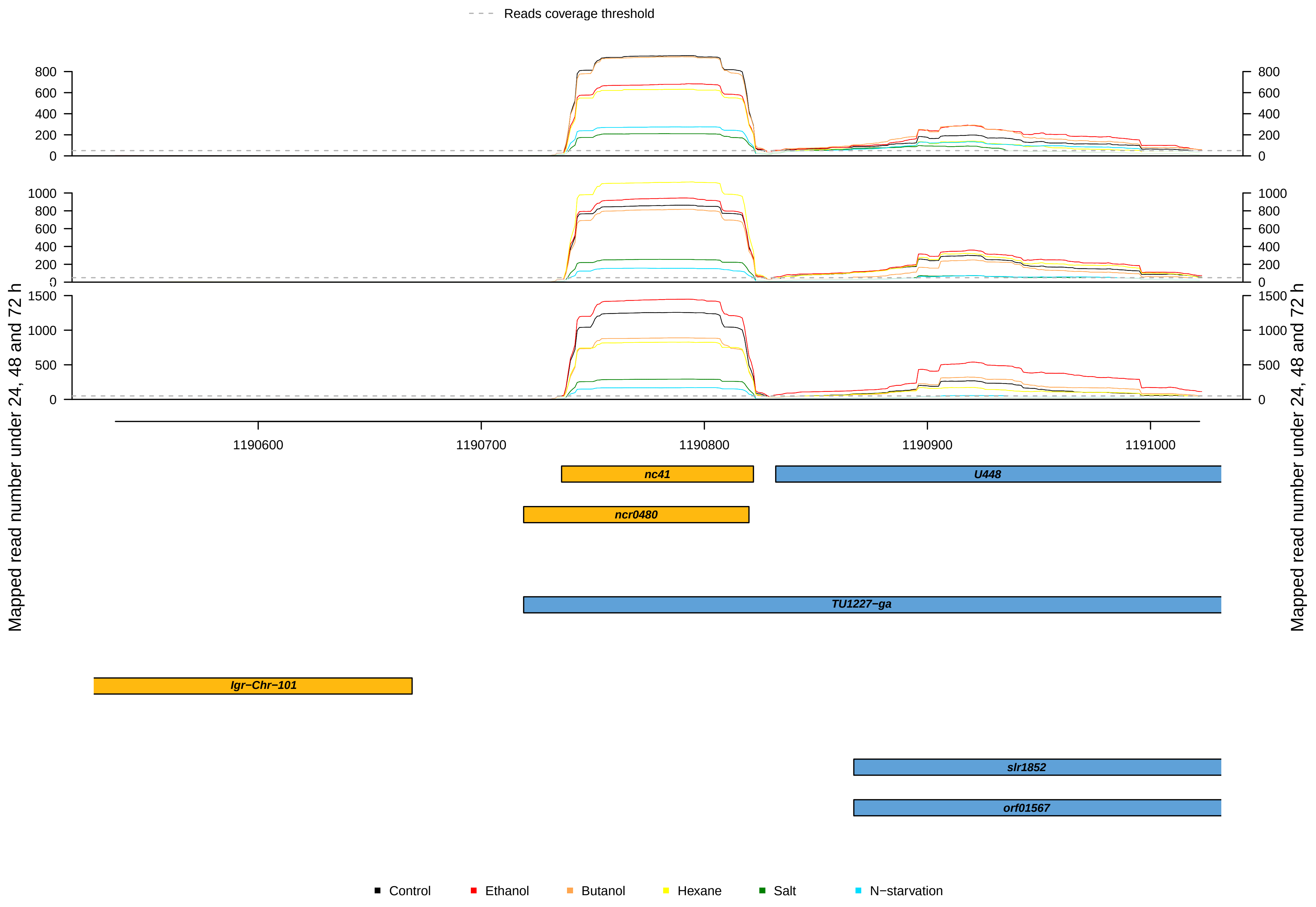
Reads coverage threshold
800
600
400
200
0
1000
1500
500
Mapped read number under 24, 48 and 72 h
1190600
1190700
1190800
1190900
1191000
nc41
U448
ncr0480
TU1227−ga
Igr−Chr−101
slr1852
orf01567
Control
Ethanol
Butanol
Hexane
Salt
N−starvation

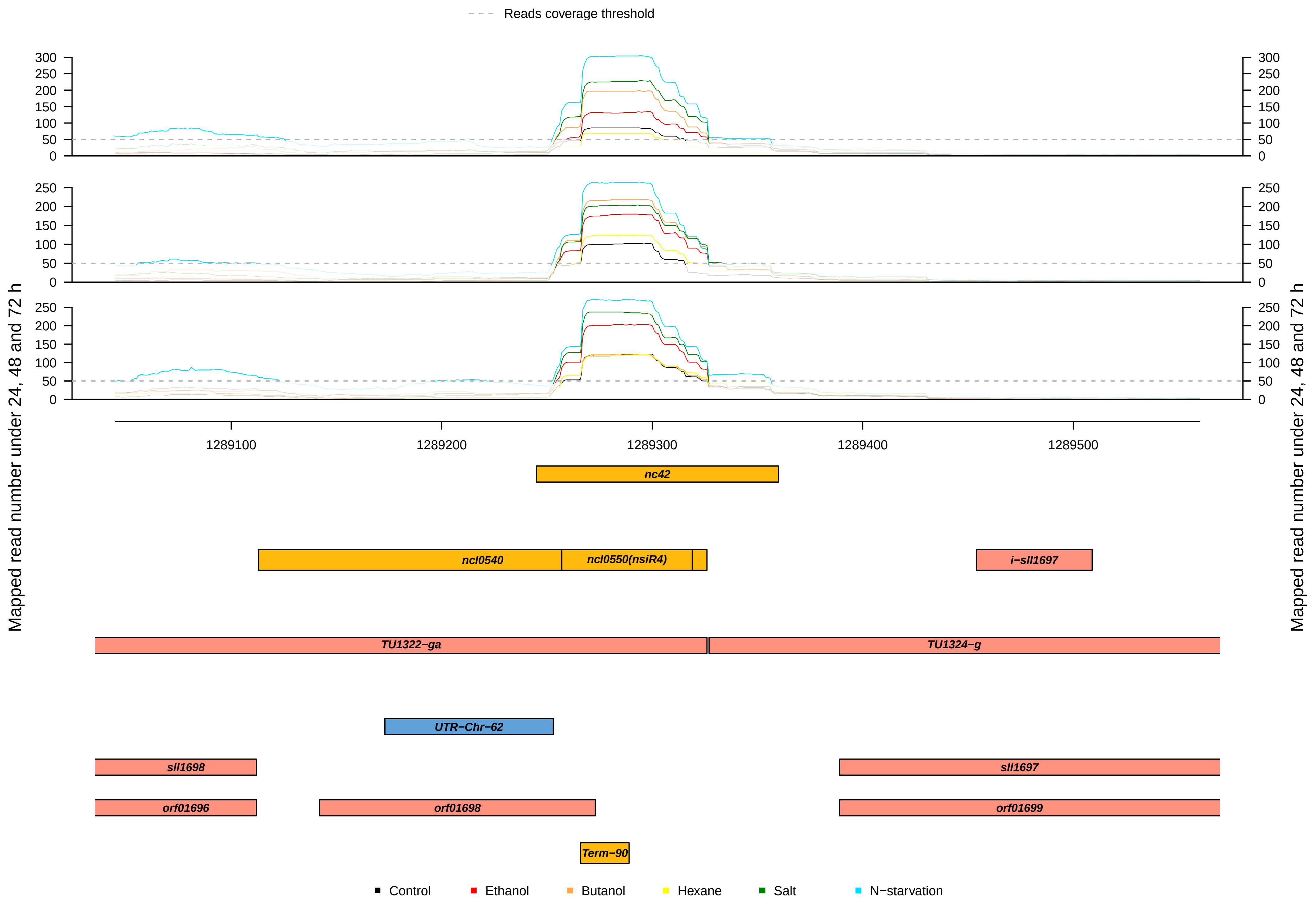

Reads coverage threshold
Mapped read number under 24, 48 and 72 h
300
250
200
150
100
50
0
1289100
1289200
1289300
1289400
1289500
nc42
ncl0540
ncl0550(nsiR4)
i−sll1697
TU1322−ga
TU1324−g
UTR−Chr−62
sll1698
sll1697
orf01696
orf01698
orf01699
Term−90
Control
Ethanol
Butanol
Hexane
Salt
N−starvation

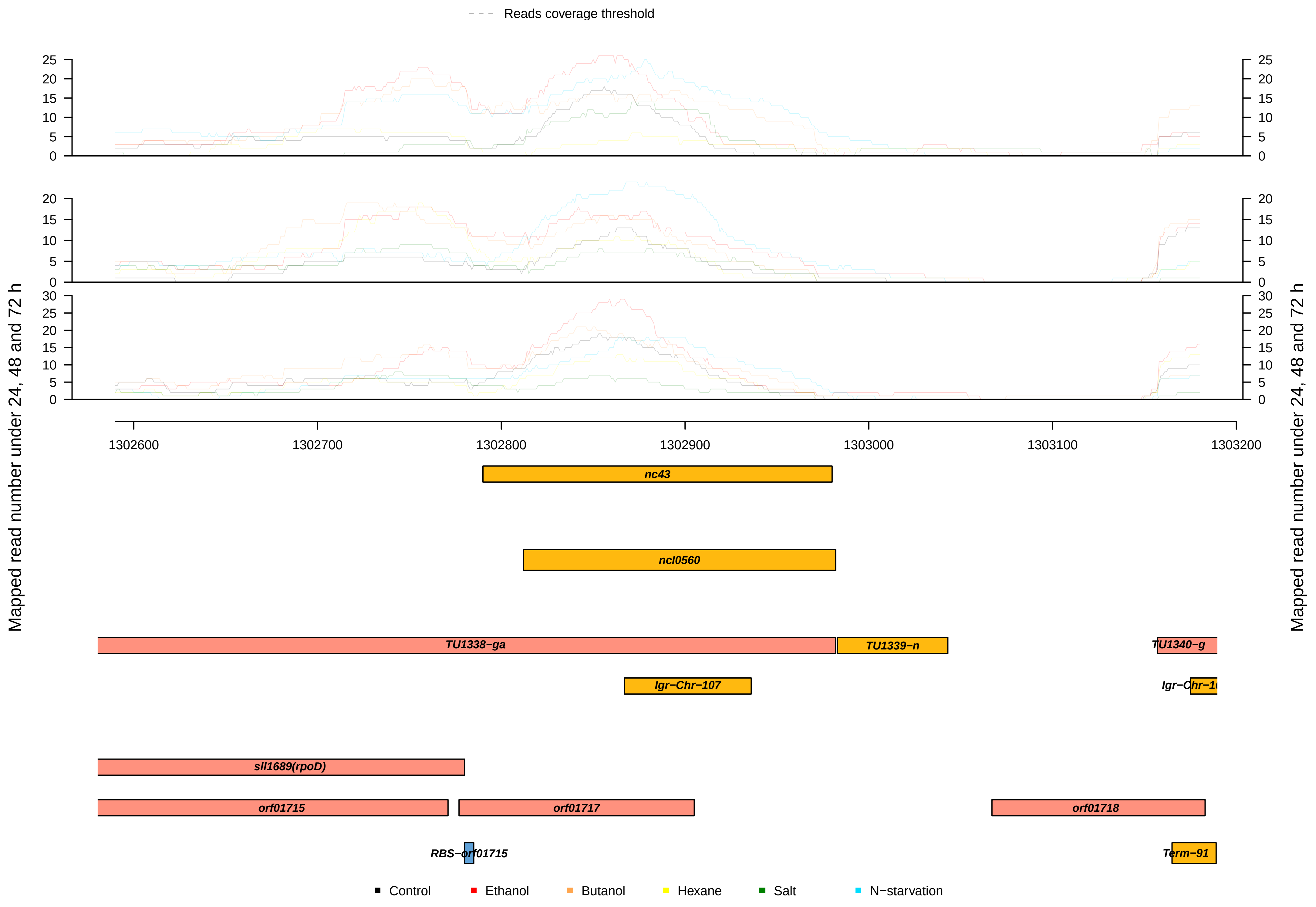

Reads coverage threshold
Mapped read number under 24, 48 and 72 h
1302600
1302700
1302800
1302900
1303000
1303100
1303200
nc43
ncl0560
TU1338−ga
TU1339−n
TU1340−g
Igr−Chr−107
sll1689(rpoD)
orf01715
orf01717
orf01718
RBS−orf01715
Term−91
Control
Ethanol
Butanol
Hexane
Salt
N−starvation

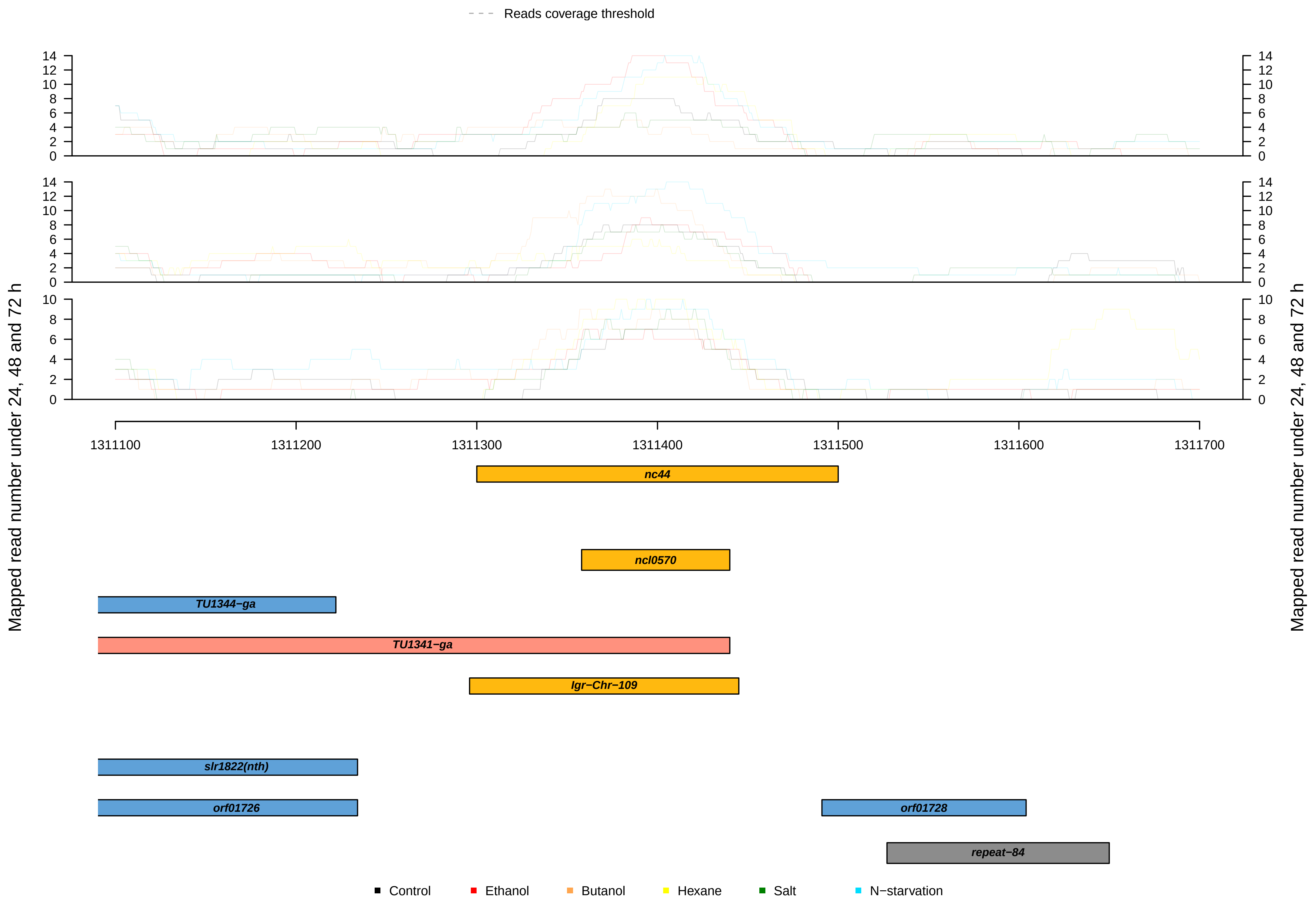

Reads coverage threshold
Mapped read number under 24, 48 and 72 h
1311100
1311200
1311300
1311400
1311500
1311600
1311700
nc44
ncl0570
TU1344−ga
TU1341−ga
Igr−Chr−109
slr1822(nth)
orf01726
orf01728
repeat−84
Control
Ethanol
Butanol
Hexane
Salt
N−starvation

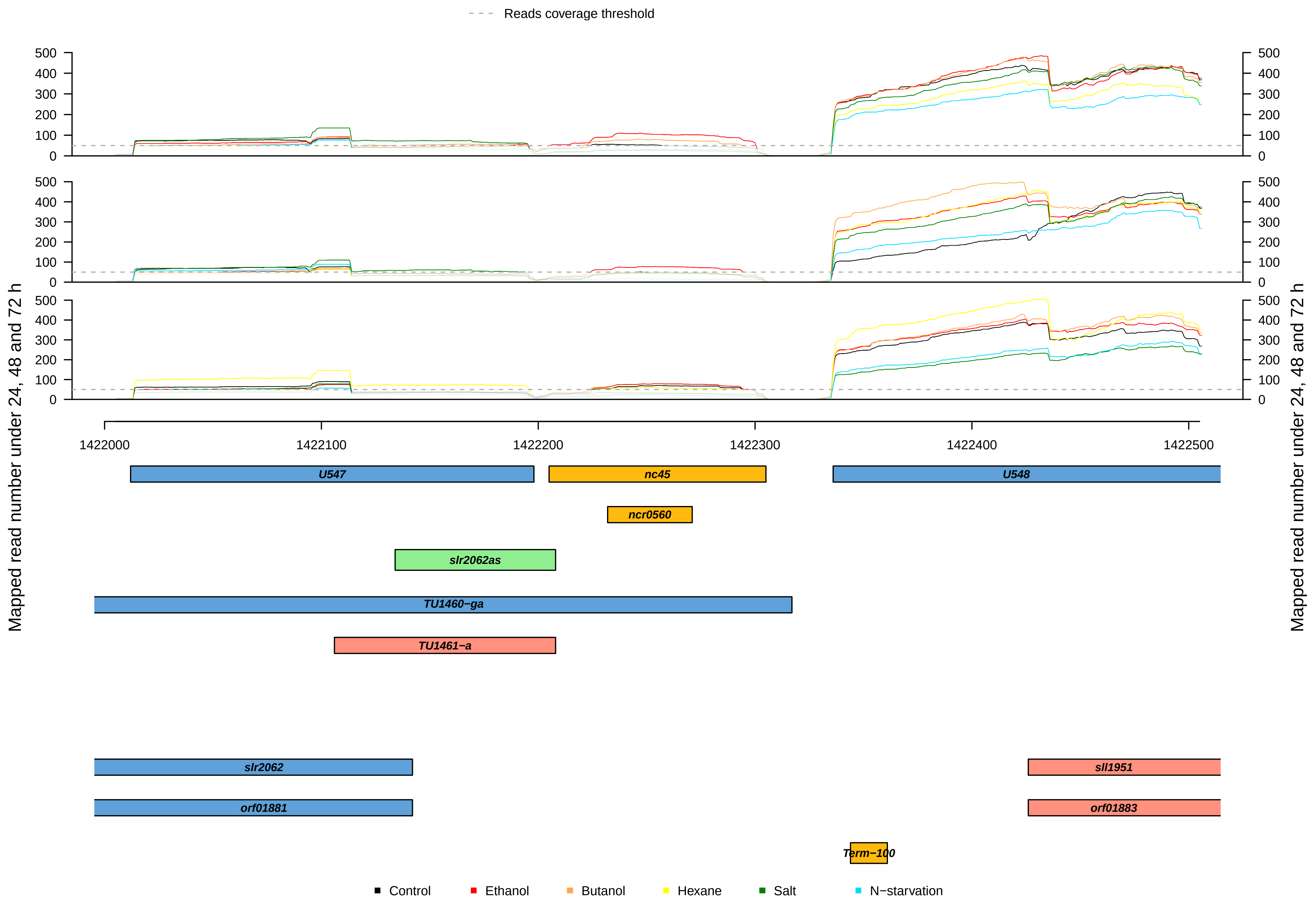

Reads coverage threshold
500
400
300
200
100
0
Mapped read number under 24, 48 and 72 h
1422000
1422100
1422200
1422300
1422400
1422500
U547
nc45
U548
ncr0560
slr2062as
TU1460−ga
TU1461−a
slr2062
sll1951
orf01881
orf01883
Term−100
Control
Ethanol
Butanol
Hexane
Salt
N−starvation

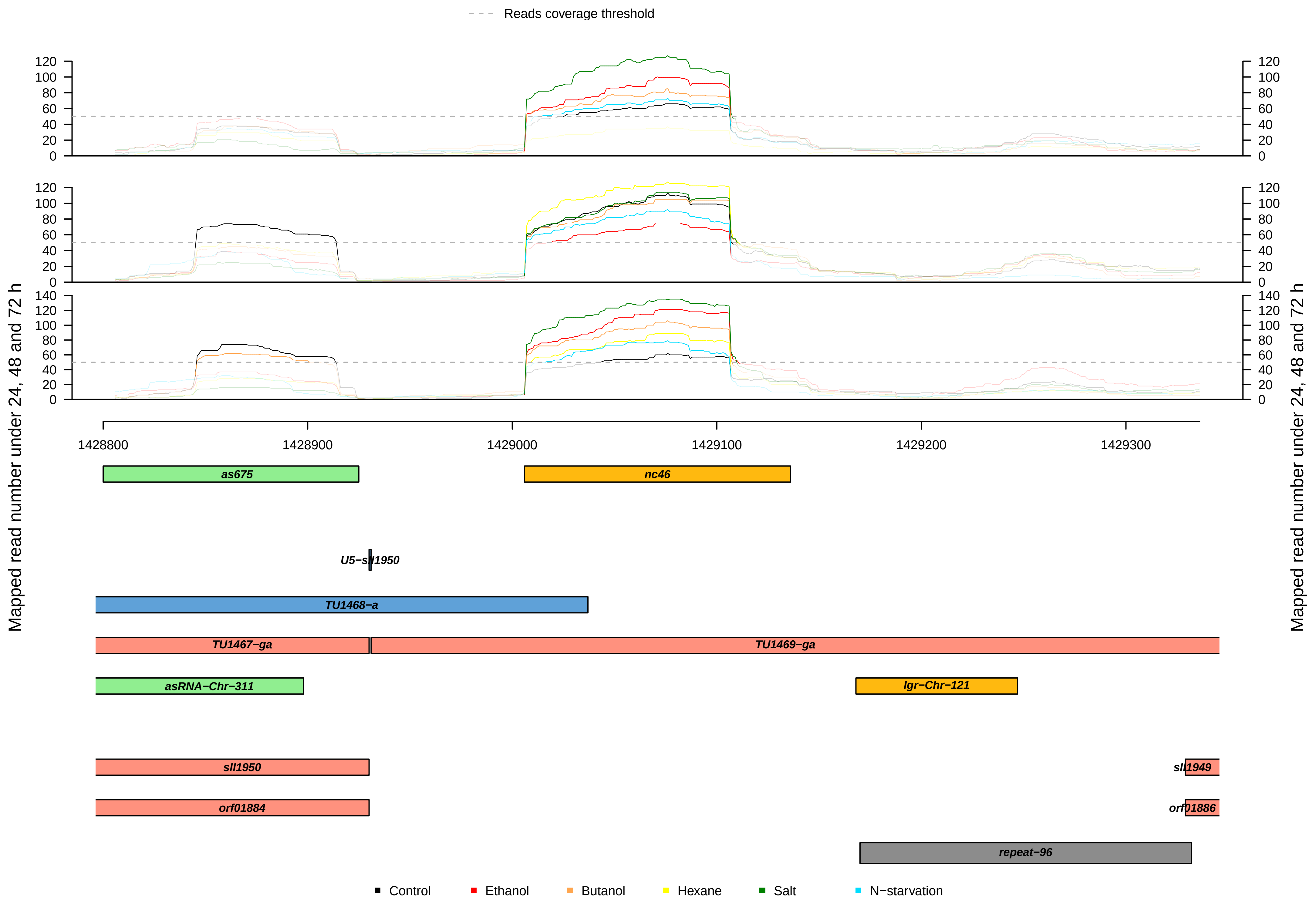

Reads coverage threshold
Mapped read number under 24, 48 and 72 h
120
100
80
60
40
20
0
140
1428800
1428900
1429000
1429100
1429200
1429300
as675
nc46
U5−sll1950
TU1468−a
TU1467−ga
TU1469−ga
asRNA−Chr−311
Igr−Chr−121
sll1950
sll1949
orf01884
orf01886
repeat−96
Control
Ethanol
Butanol
Hexane
Salt
N−starvation

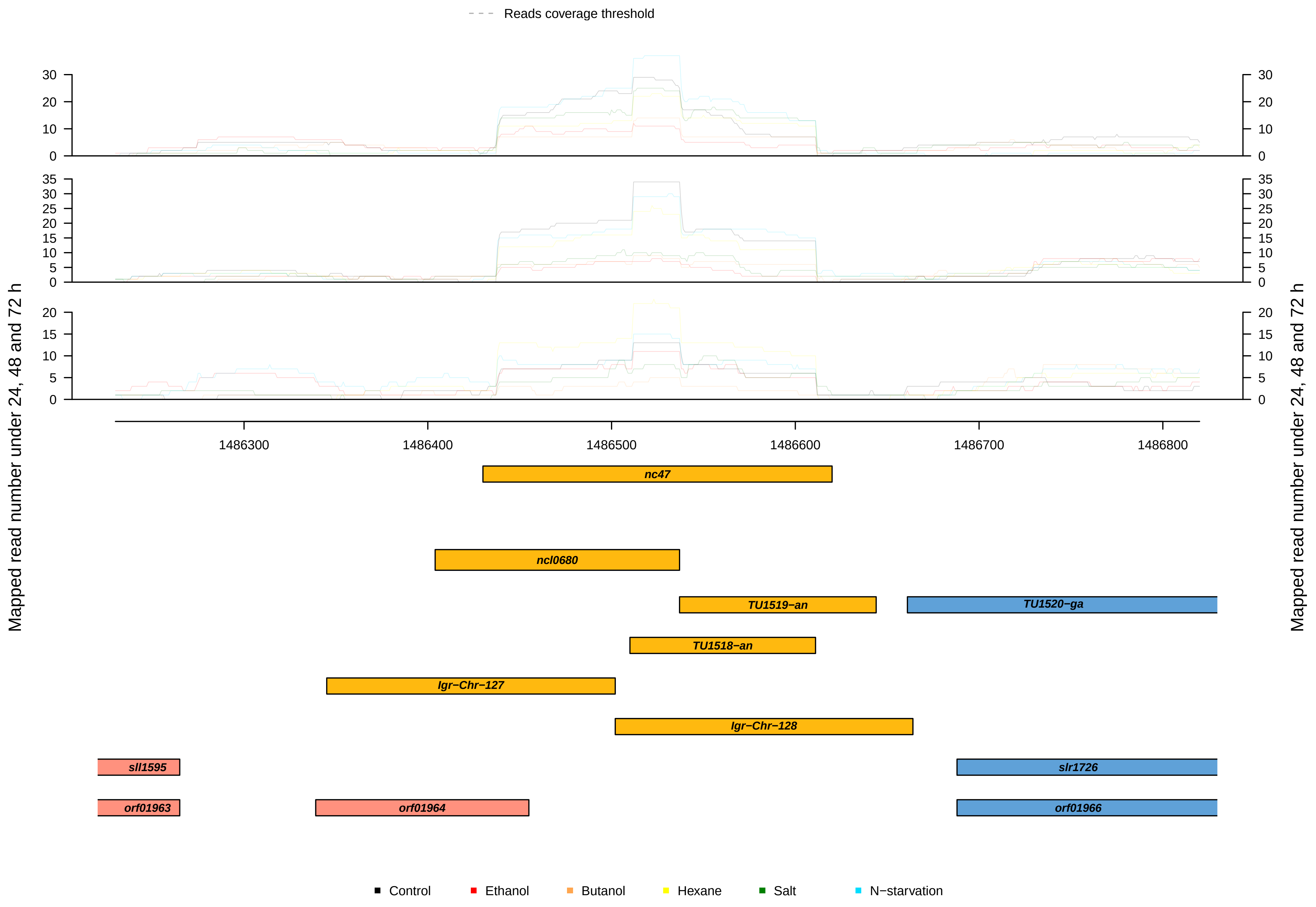

Reads coverage threshold
Mapped read number under 24, 48 and 72 h
1486300
1486400
1486500
1486600
1486700
1486800
nc47
ncl0680
TU1519−an
TU1520−ga
TU1518−an
Igr−Chr−127
Igr−Chr−128
sll1595
slr1726
orf01963
orf01964
orf01966
Control
Ethanol
Butanol
Hexane
Salt
N−starvation

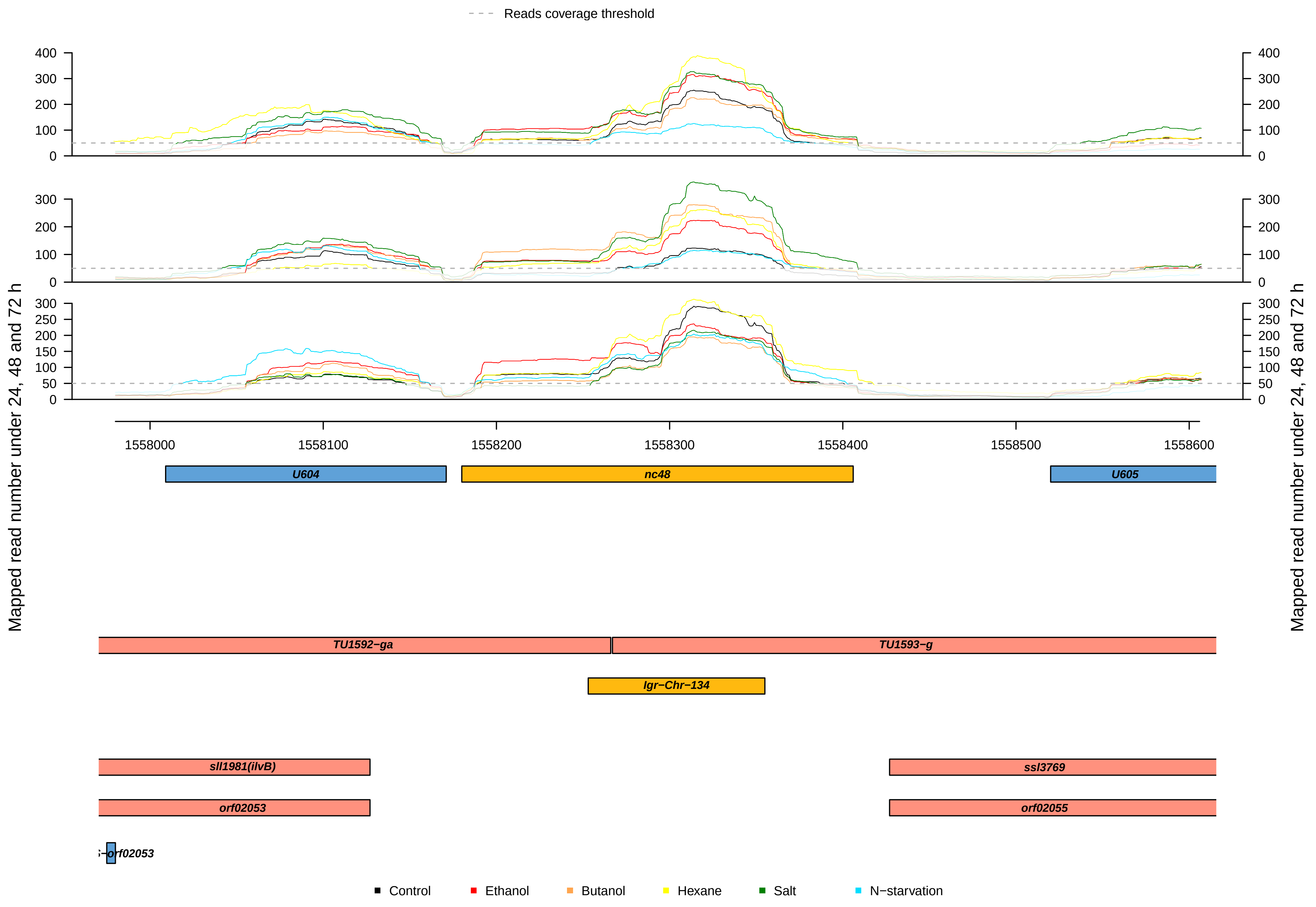

Reads coverage threshold
400
300
200
100
0
Mapped read number under 24, 48 and 72 h
1558000
1558100
1558200
1558300
1558400
1558500
1558600
U604
nc48
U605
TU1592−ga
TU1593−g
Igr−Chr−134
sll1981(ilvB)
ssl3769
orf02053
orf02055
Control
Ethanol
Butanol
Hexane
Salt
N−starvation

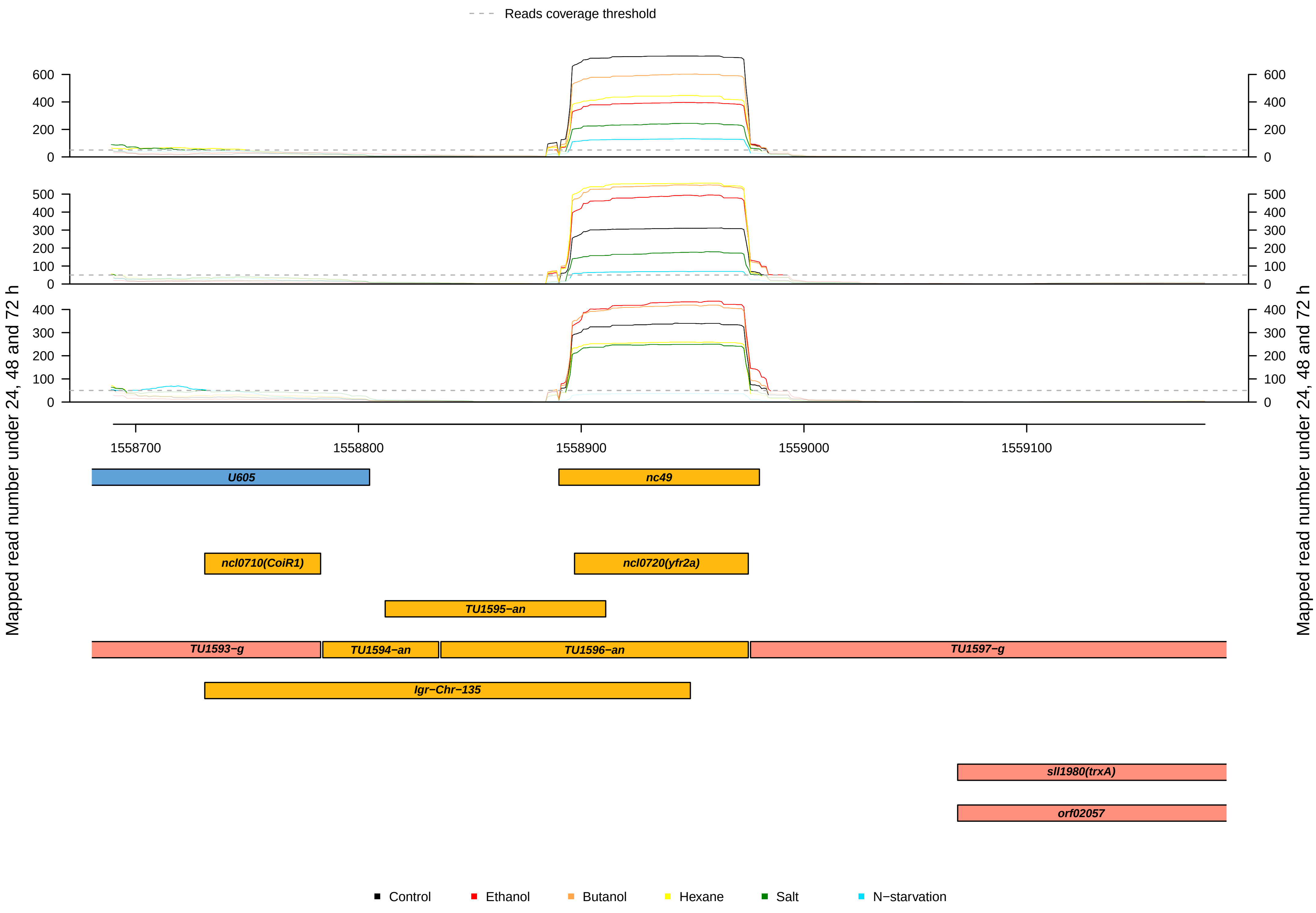

Reads coverage threshold
600
400
200
0
500
400
300
200
100
0
400
300
200
100
0
Mapped read number under 24, 48 and 72 h
1558700
1558800
1558900
1559000
1559100
U605
nc49
ncl0710(CoiR1)
ncl0720(yfr2a)
TU1595−an
TU1593−g
TU1594−an
TU1596−an
TU1597−g
Igr−Chr−135
sll1980(trxA)
orf02057
Control
Ethanol
Butanol
Hexane
Salt
N−starvation

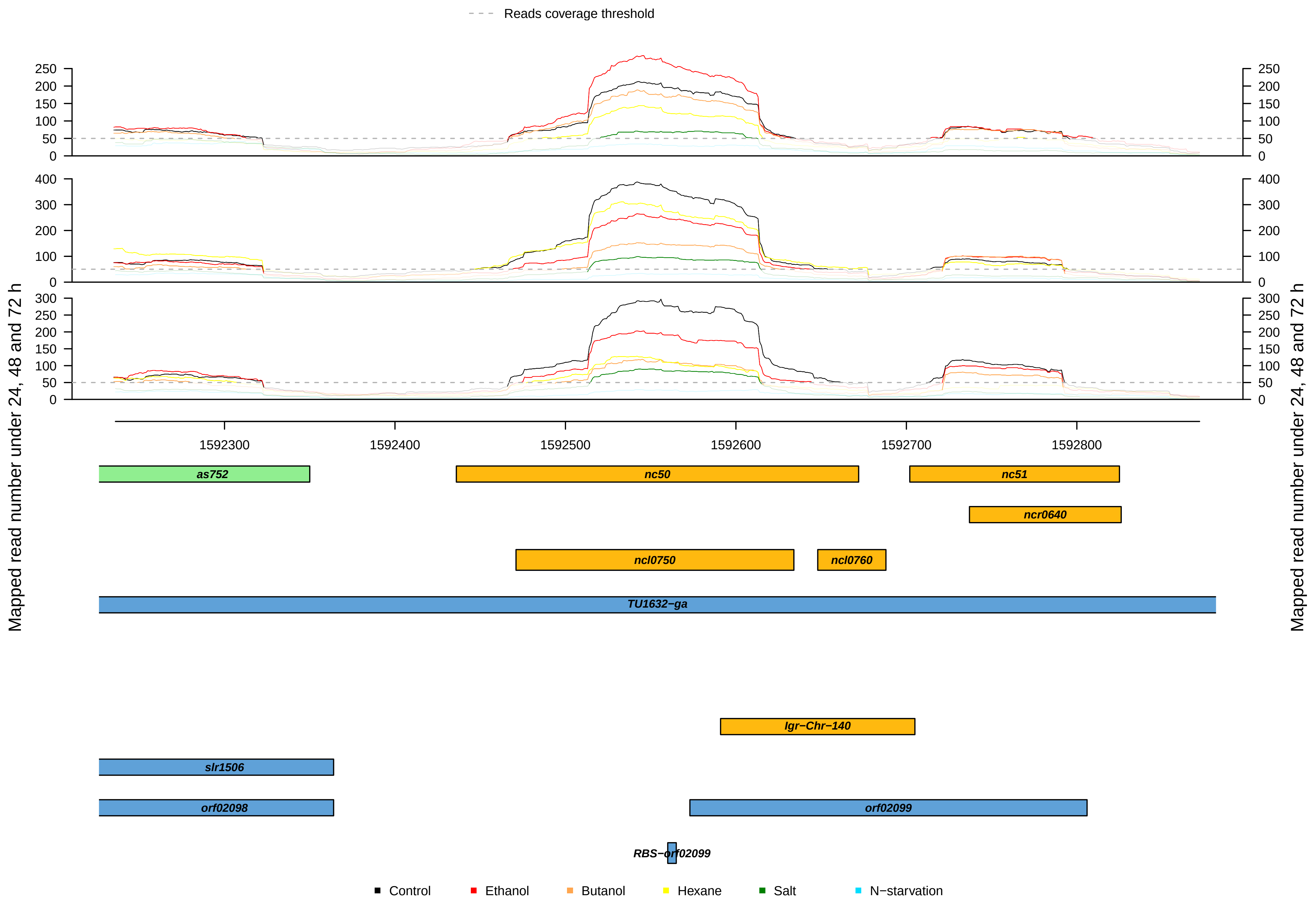

Reads coverage threshold
Mapped read number under 24, 48 and 72 h
250
200
150
100
50
0
400
300
200
100
0
300
250
200
150
100
50
0
1592300
1592400
1592500
1592600
1592700
1592800
as752
nc50
nc51
ncr0640
ncl0750
ncl0760
TU1632−ga
Igr−Chr−140
slr1506
orf02098
orf02099
RBS−orf02099
Control
Ethanol
Butanol
Hexane
Salt
N−starvation

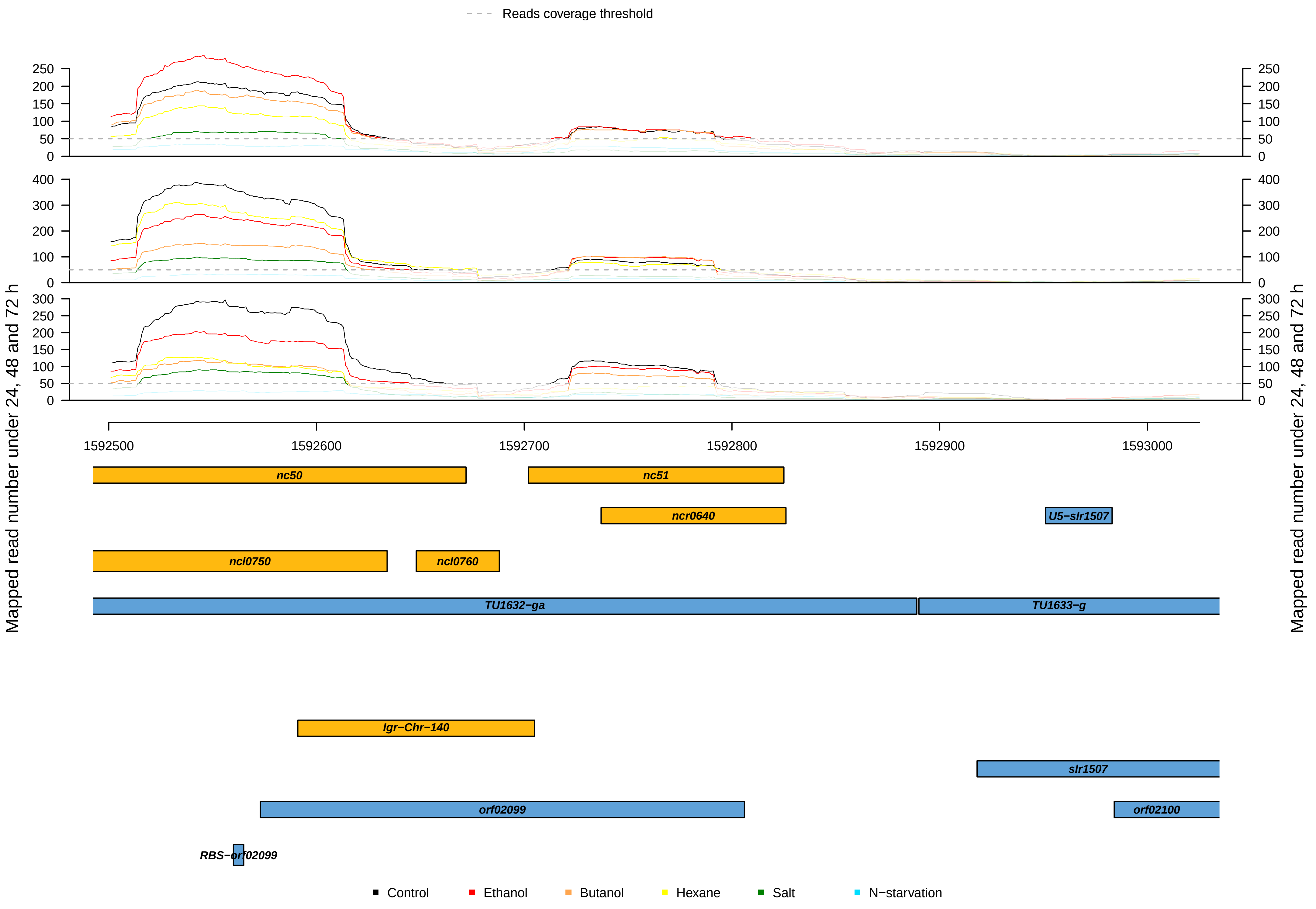

Reads coverage threshold
Mapped read number under 24, 48 and 72 h
250
200
150
100
50
0
400
300
200
100
0
300
250
200
150
100
50
0
1592500
1592600
1592700
1592800
1592900
1593000
nc50
nc51
ncr0640
U5−slr1507
ncl0750
ncl0760
TU1632−ga
TU1633−g
Igr−Chr−140
slr1507
orf02099
orf02100
RBS−orf02099
Control
Ethanol
Butanol
Hexane
Salt
N−starvation

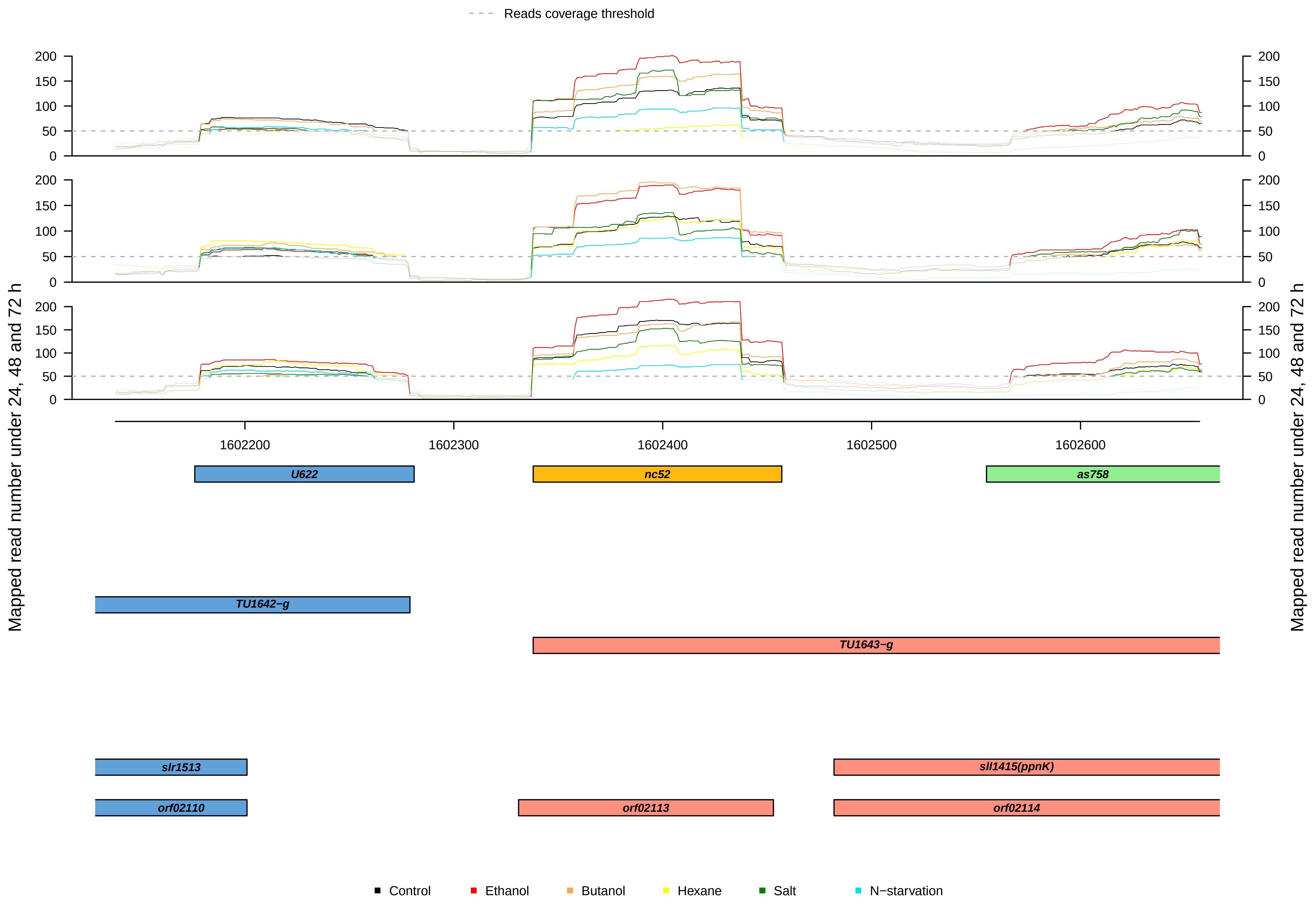
Reads coverage threshold
Mapped read number under 24, 48 and 72 h
0
50
100
150
200
1602200
1602300
1602400
1602500
1602600
U622
nc52
as758
TU1642−g
TU1643−g
slr1513
sll1415(ppnK)
orf02110
orf02113
orf02114
Control
Ethanol
Butanol
Hexane
Salt
N−starvation

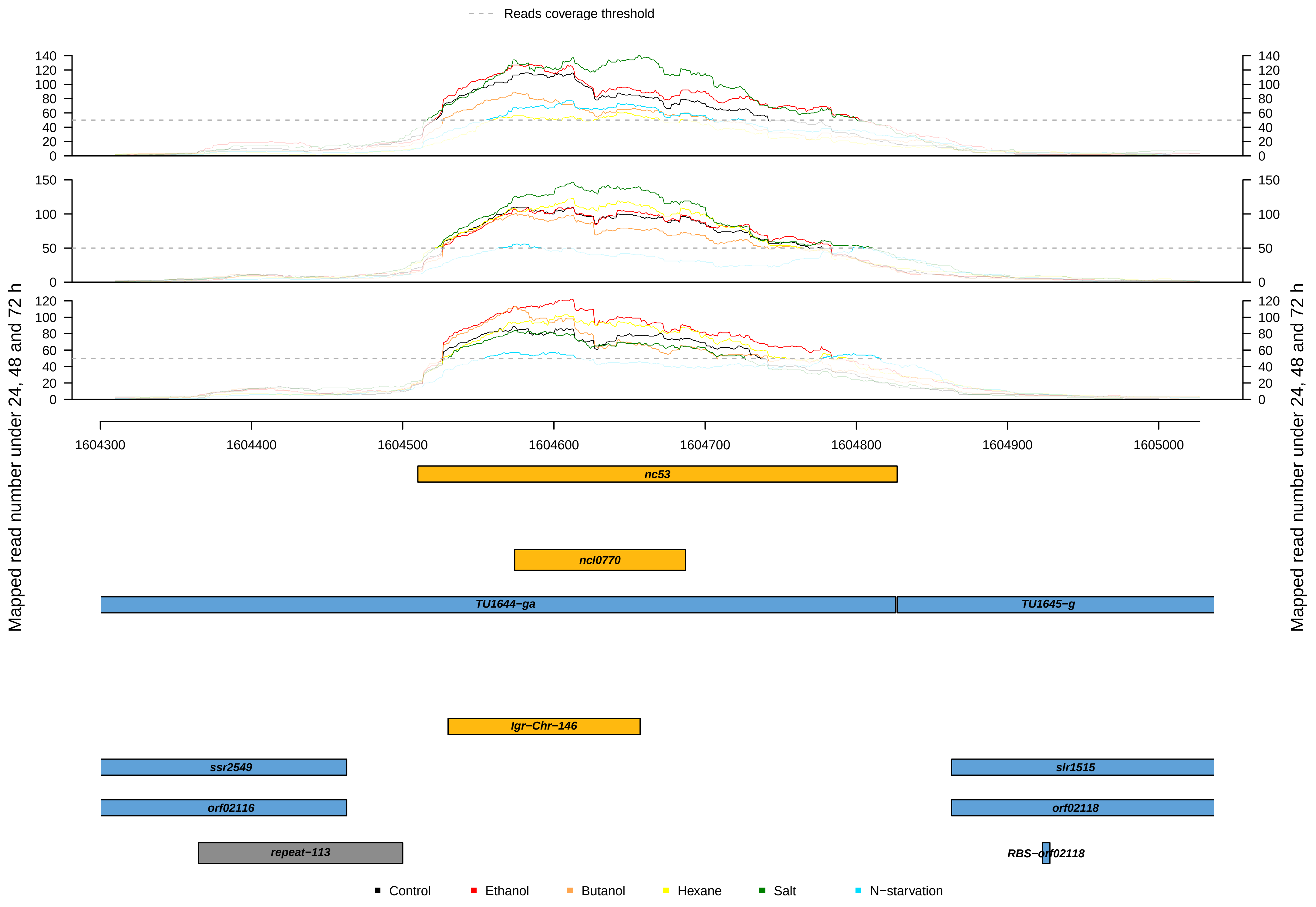

Reads coverage threshold
Mapped read number under 24, 48 and 72 h
1604300
1604400
1604500
1604600
1604700
1604800
1604900
1605000
nc53
ncl0770
TU1644−ga
TU1645−g
Igr−Chr−146
ssr2549
slr1515
orf02116
orf02118
repeat−113
RBS−orf02118
Control
Ethanol
Butanol
Hexane
Salt
N−starvation

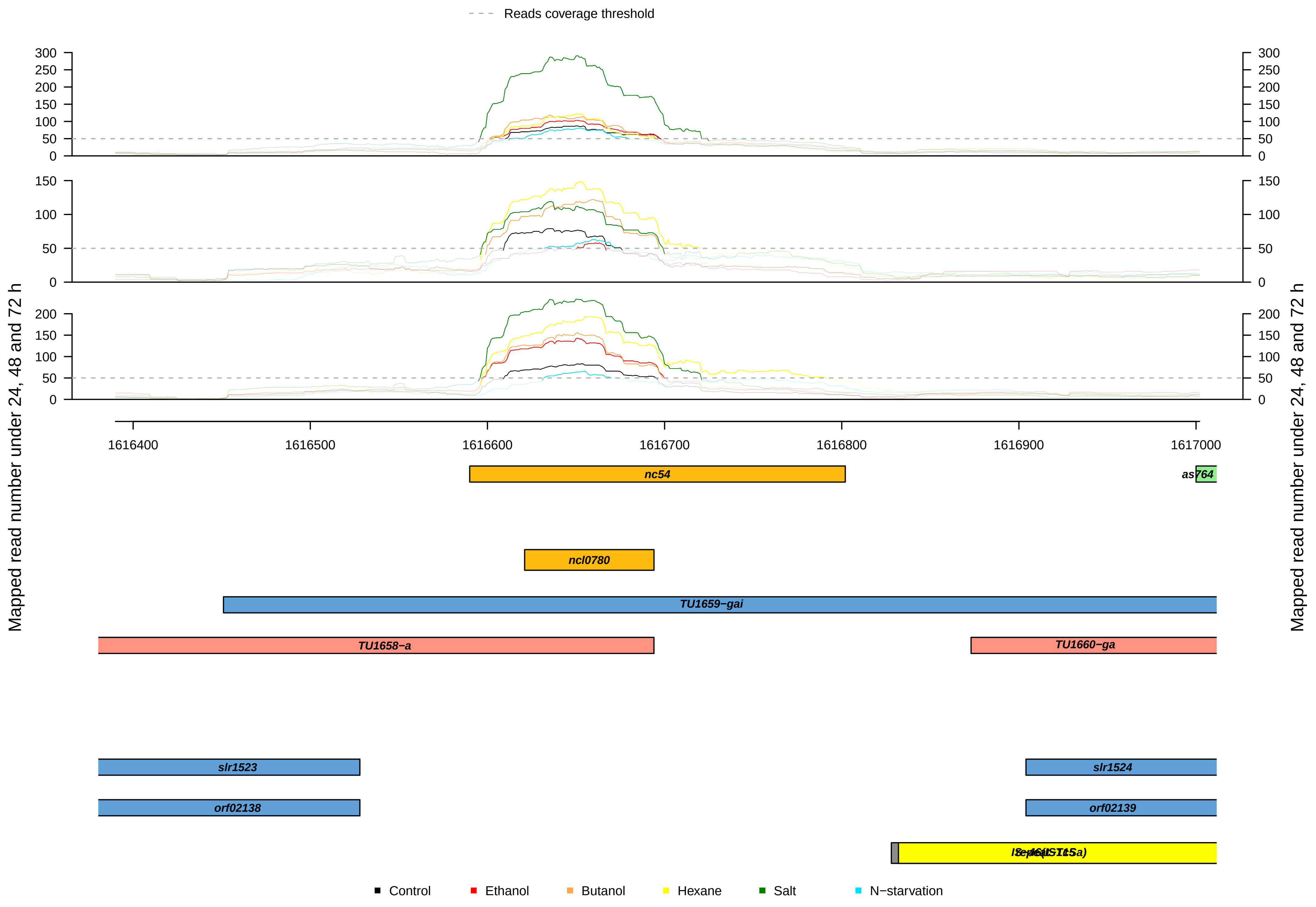
Reads coverage threshold
Mapped read number under 24, 48 and 72 h
300
250
200
150
100
50
0
150
100
50
0
200
150
100
50
0
1616400
1616500
1616600
1616700
1616800
1616900
1617000
nc54
as764
ncl0780
TU1659−gai
TU1658−a
TU1660−ga
slr1523
slr1524
orf02138
orf02139
Control
Ethanol
Butanol
Hexane
Salt
N−starvation

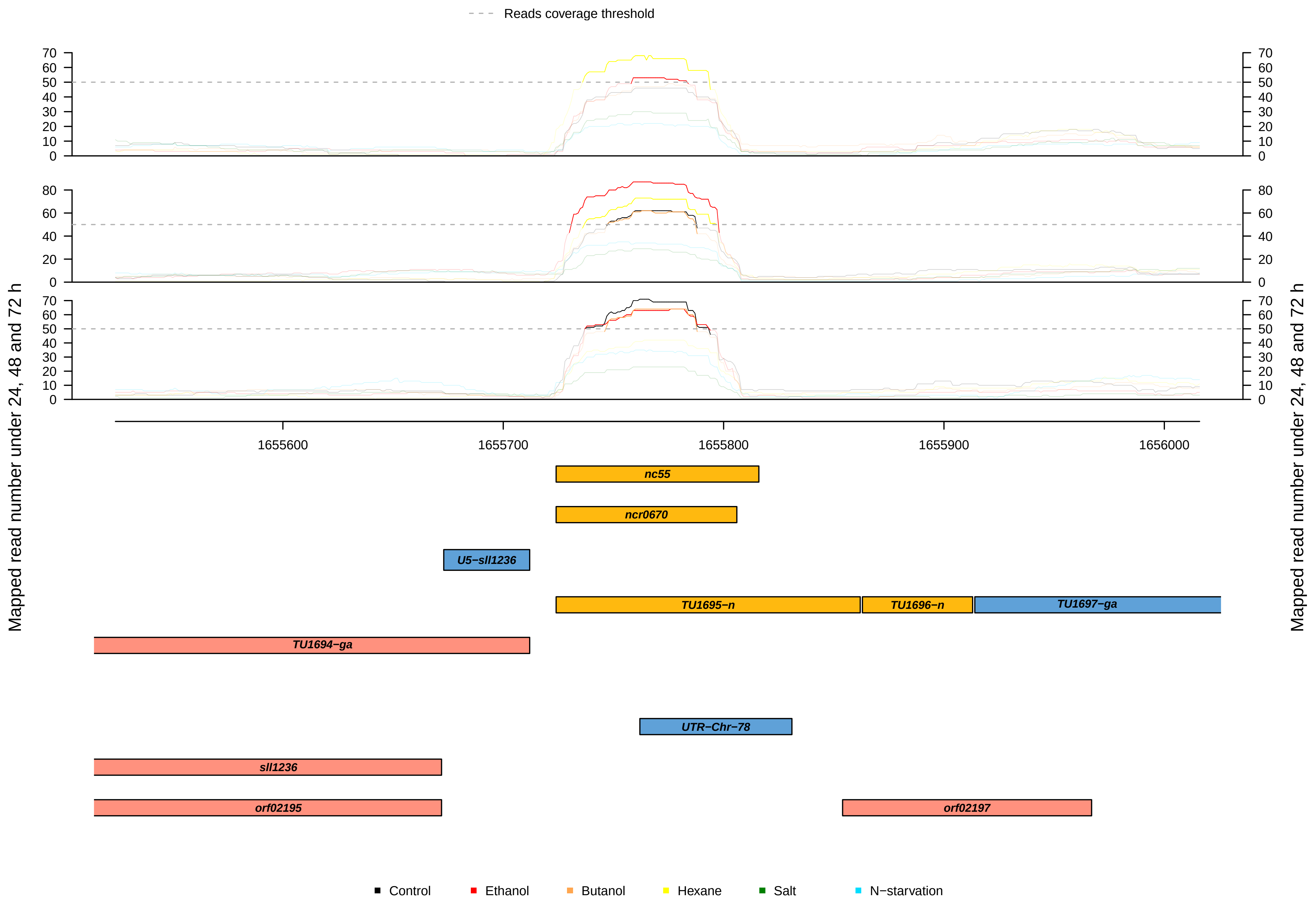

Reads coverage threshold
Mapped read number under 24, 48 and 72 h
1655600
1655700
1655800
1655900
1656000
nc55
ncr0670
U5−sll1236
TU1695−n
TU1696−n
TU1697−ga
TU1694−ga
UTR−Chr−78
sll1236
orf02195
orf02197
Control
Ethanol
Butanol
Hexane
Salt
N−starvation

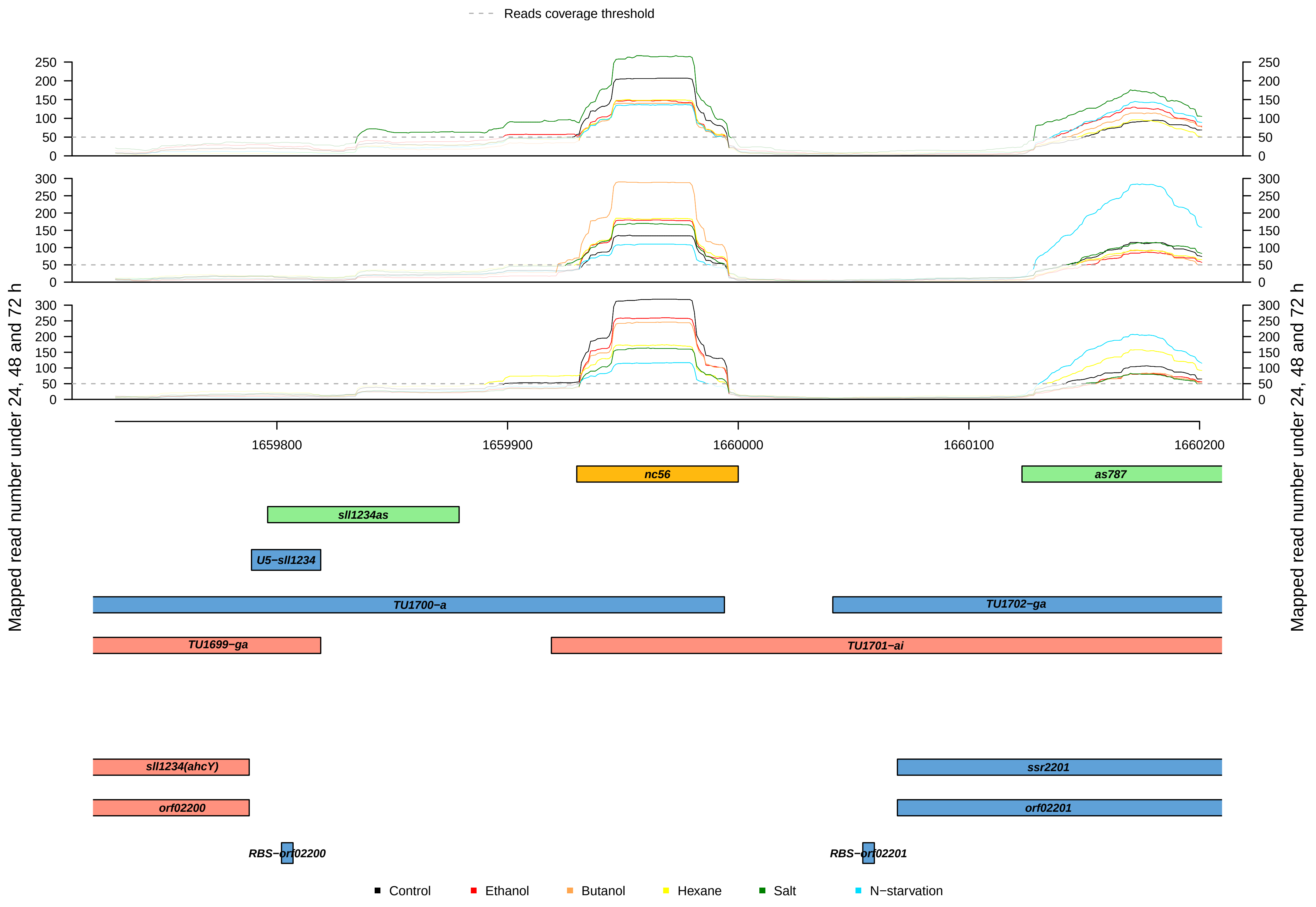

Reads coverage threshold
Mapped read number under 24, 48 and 72 h
1659800
1659900
1660000
1660100
1660200
nc56
as787
sll1234as
U5−sll1234
TU1700−a
TU1702−ga
TU1699−ga
TU1701−ai
sll1234(ahcY)
ssr2201
orf02200
orf02201
RBS−orf02200
RBS−orf02201
Control
Ethanol
Butanol
Hexane
Salt
N−starvation

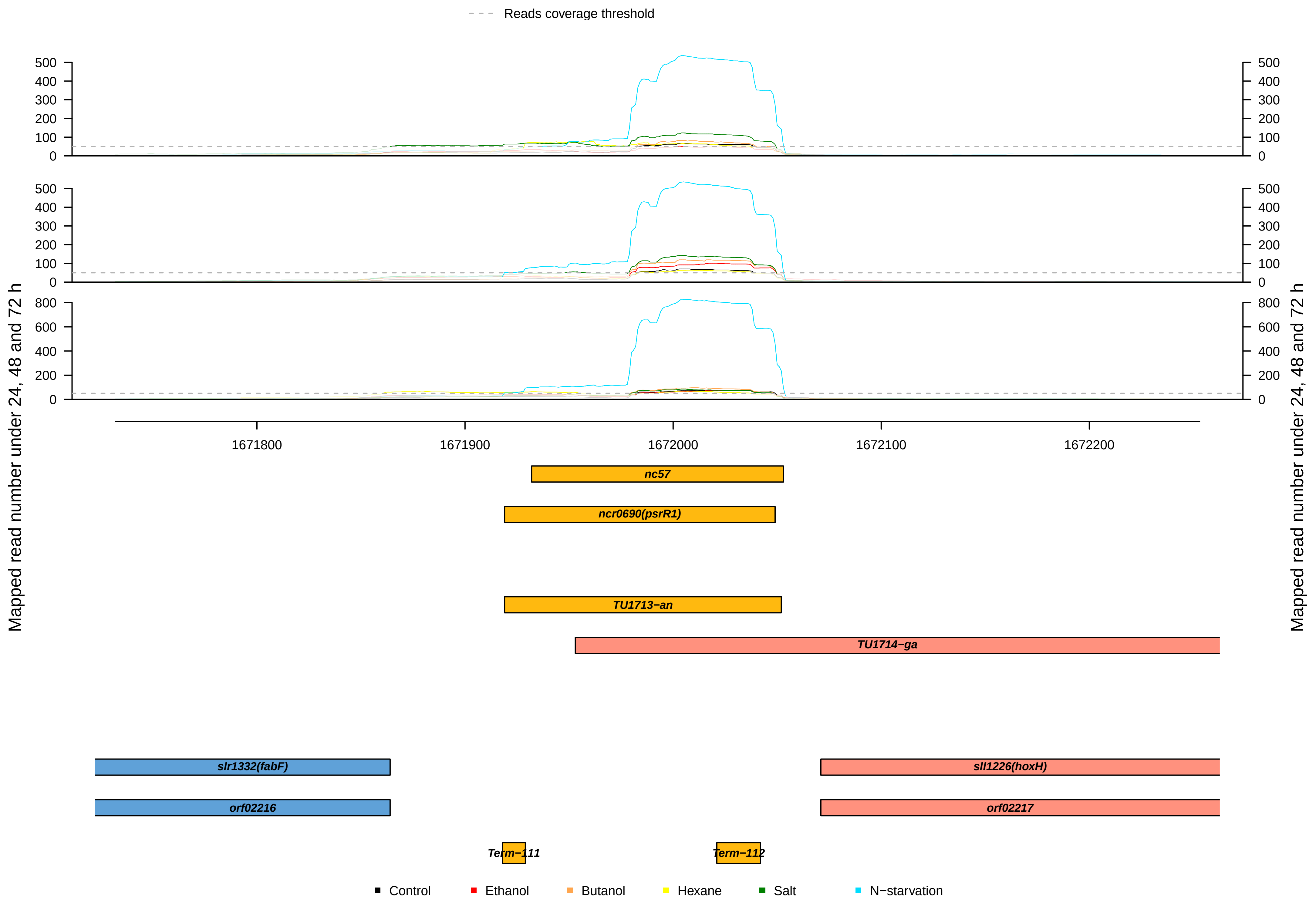

Reads coverage threshold
Mapped read number under 24, 48 and 72 h
1671800
1671900
1672000
1672100
1672200
nc57
ncr0690(psrR1)
TU1713−an
TU1714−ga
slr1332(fabF)
sll1226(hoxH)
orf02216
orf02217
Term−111
Term−112
Control
Ethanol
Butanol
Hexane
Salt
N−starvation

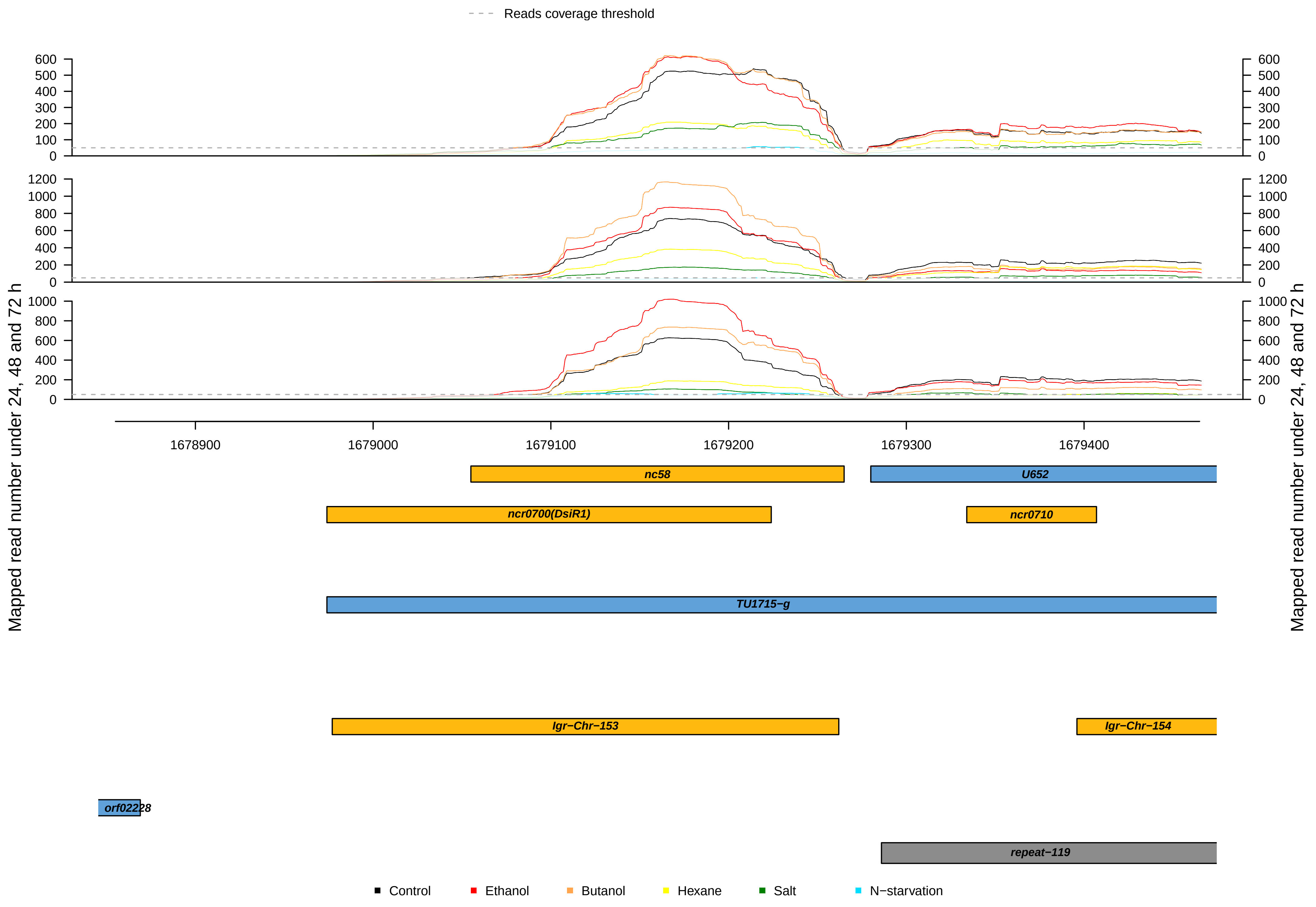

Reads coverage threshold
Mapped read number under 24, 48 and 72 h
600
500
400
300
200
100
0
1200
1000
800
600
400
200
0
1000
800
600
400
200
0
1678900
1679000
1679100
1679200
1679300
1679400
nc58
U652
ncr0700(DsiR1)
ncr0710
TU1715−g
Igr−Chr−153
Igr−Chr−154
orf02228
repeat−119
Control
Ethanol
Butanol
Hexane
Salt
N−starvation

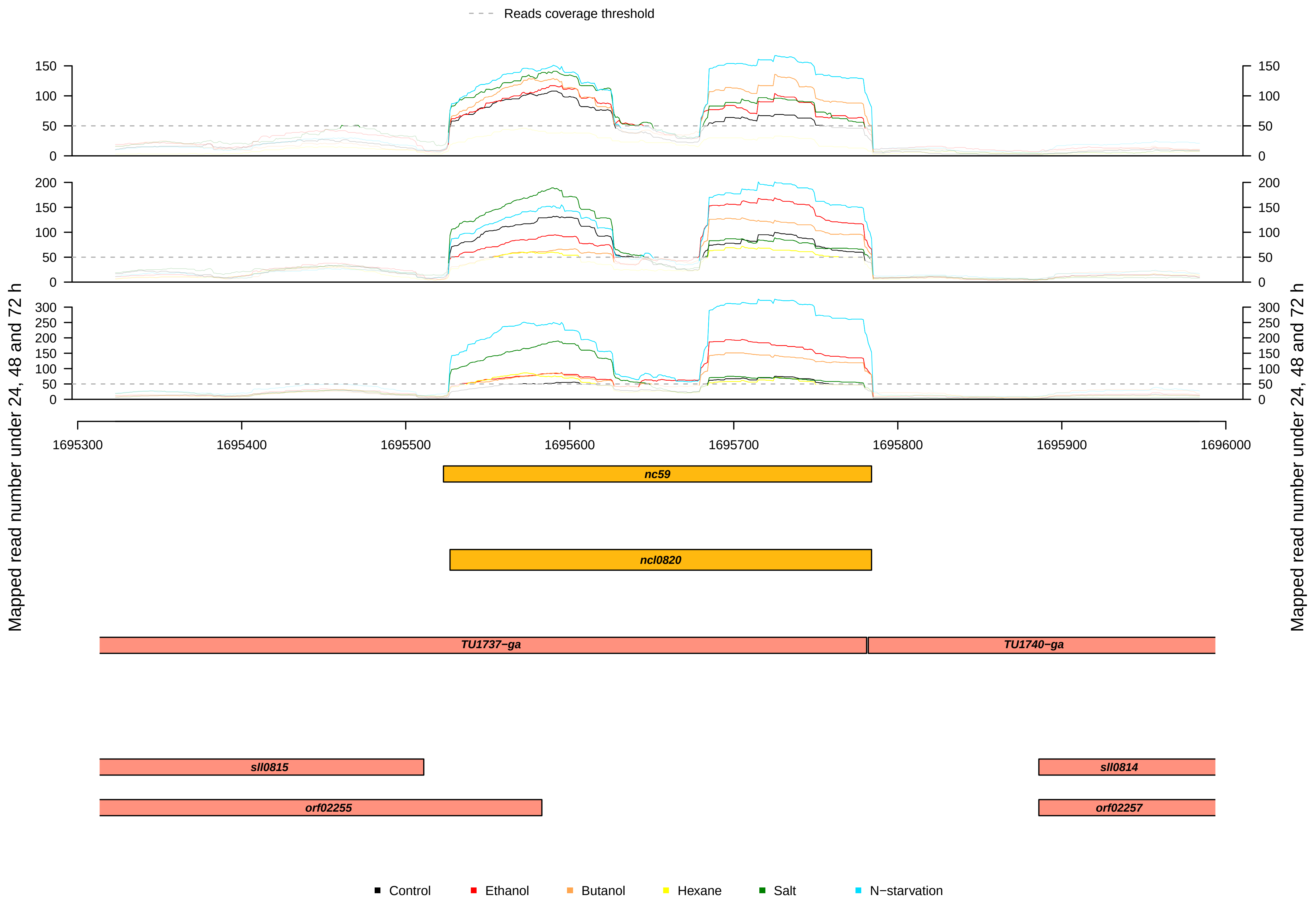

Reads coverage threshold
Mapped read number under 24, 48 and 72 h
150
100
50
0
200
150
100
50
0
300
250
200
150
100
50
0
1695300
1695400
1695500
1695600
1695700
1695800
1695900
1696000
nc59
ncl0820
TU1737−ga
TU1740−ga
sll0815
sll0814
orf02255
orf02257
Control
Ethanol
Butanol
Hexane
Salt
N−starvation

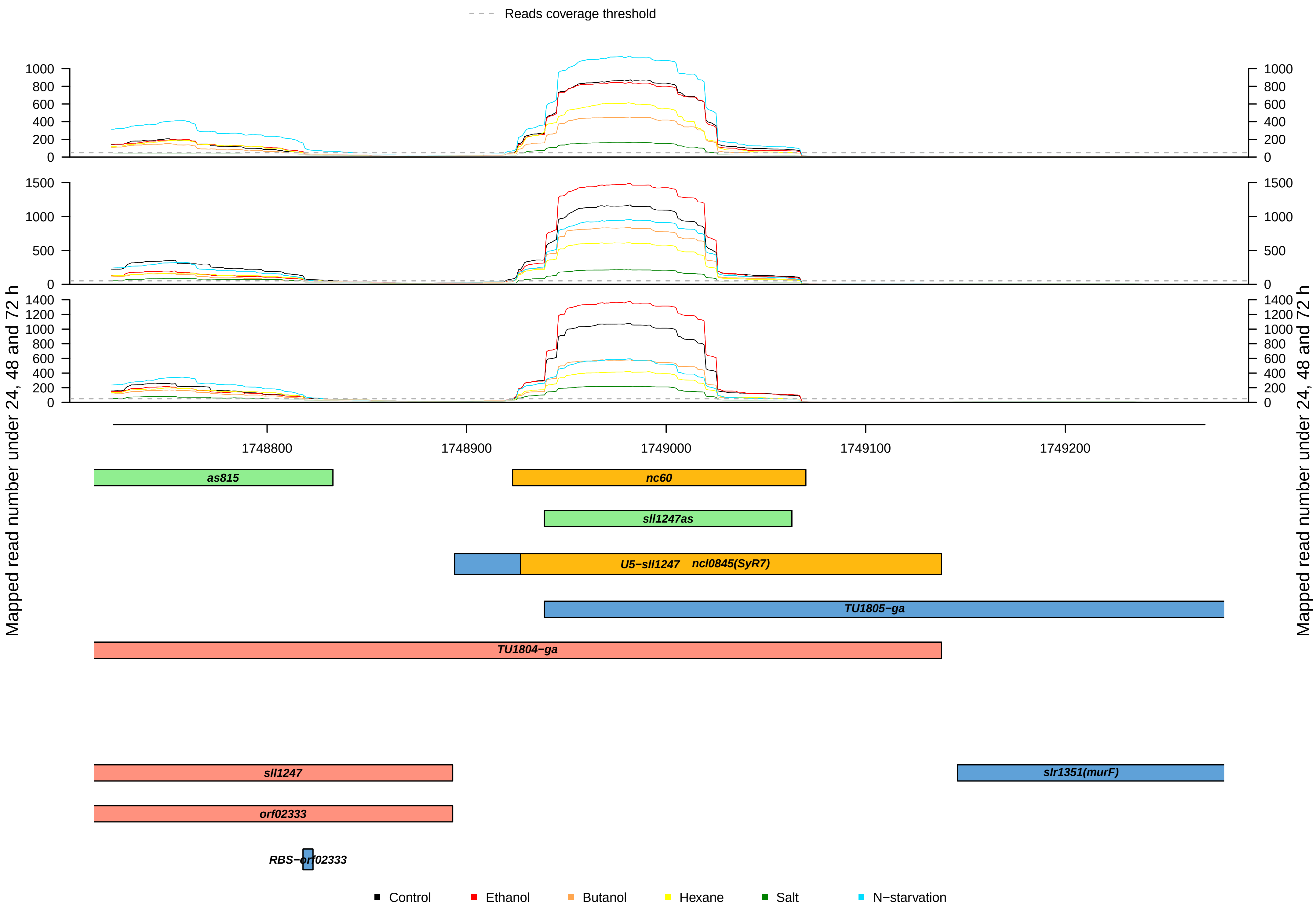
Reads coverage threshold
Mapped read number under 24, 48 and 72 h
1000
800
600
400
200
0
1500
1000
500
0
1400
1200
1000
800
600
400
200
0
1748800
1748900
1749000
1749100
1749200
as815
nc60
sll1247as
U5−sll1247
ncl0845(SyR7)
TU1805−ga
TU1804−ga
sll1247
slr1351(murF)
orf02333
RBS−orf02333
Control
Ethanol
Butanol
Hexane
Salt
N−starvation

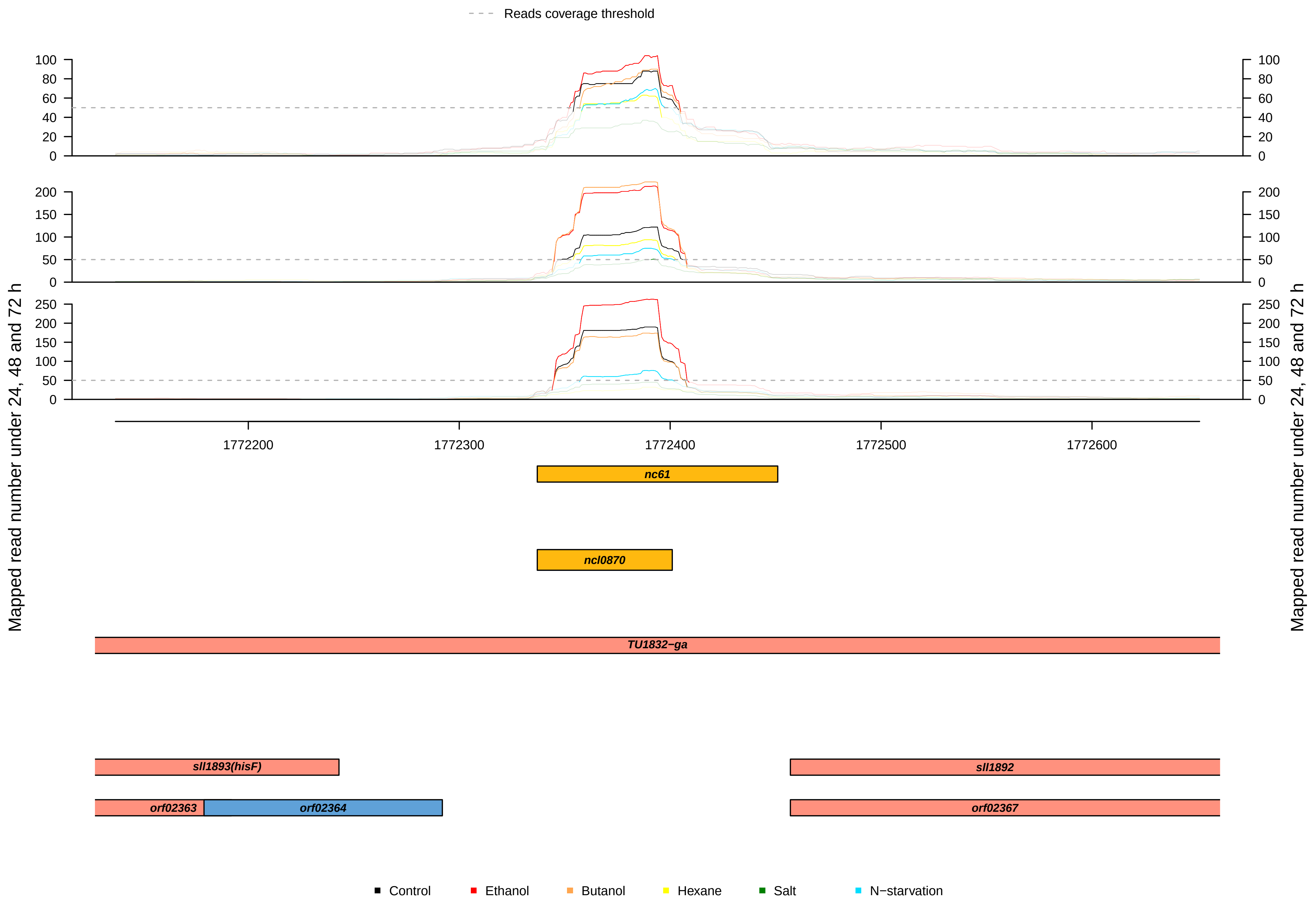

Reads coverage threshold
Mapped read number under 24, 48 and 72 h
1772200
1772300
1772400
1772500
1772600
nc61
ncl0870
TU1832−ga
sll1893(hisF)
sll1892
orf02363
orf02364
orf02367
Control
Ethanol
Butanol
Hexane
Salt
N−starvation

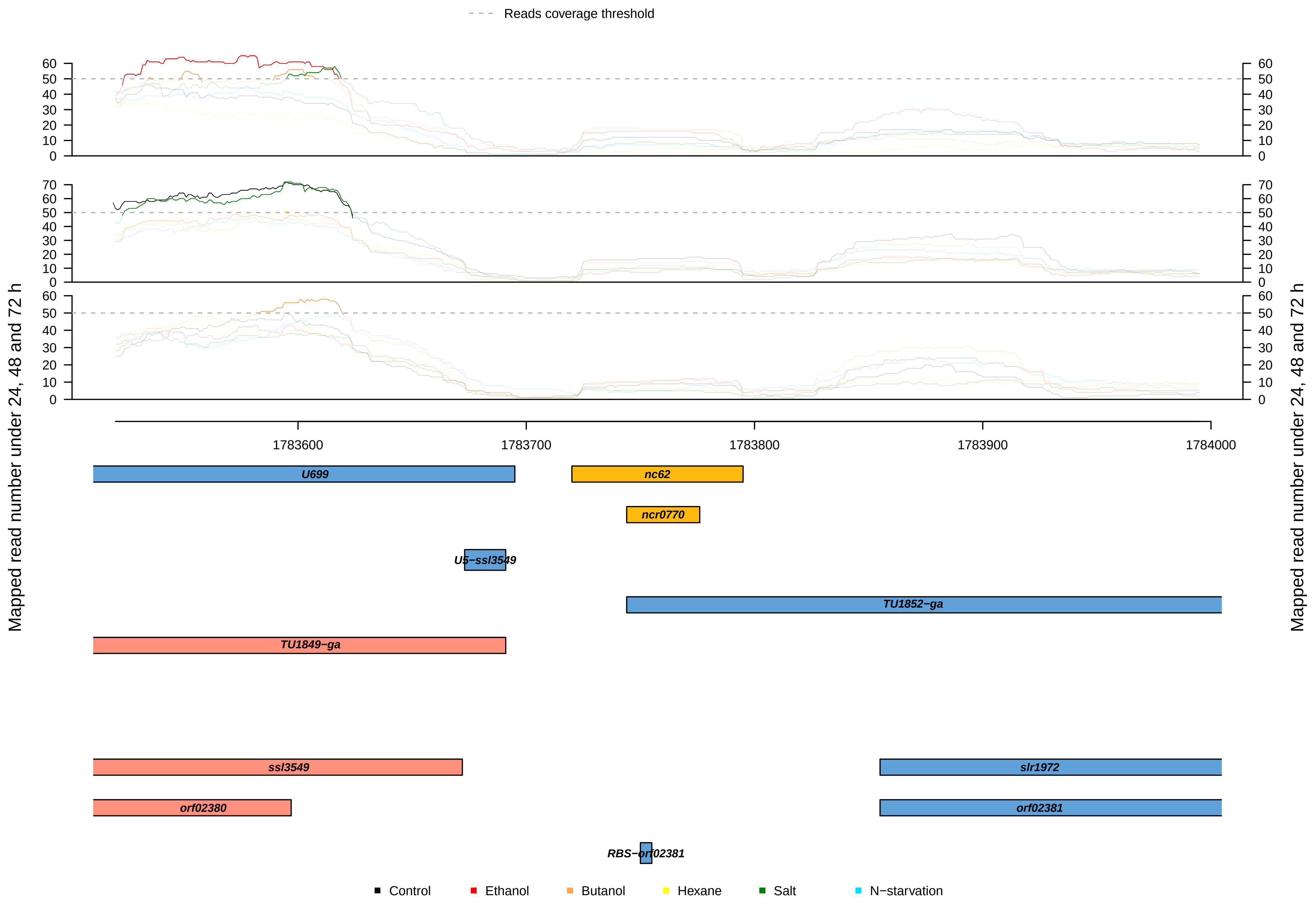

Reads coverage threshold
Mapped read number under 24, 48 and 72 h
1783600
1783700
1783800
1783900
1784000
U699
nc62
ncr0770
U5−ssl3549
TU1852−ga
TU1849−ga
ssl3549
slr1972
orf02380
orf02381
RBS−orf02381
Control
Ethanol
Butanol
Hexane
Salt
N−starvation

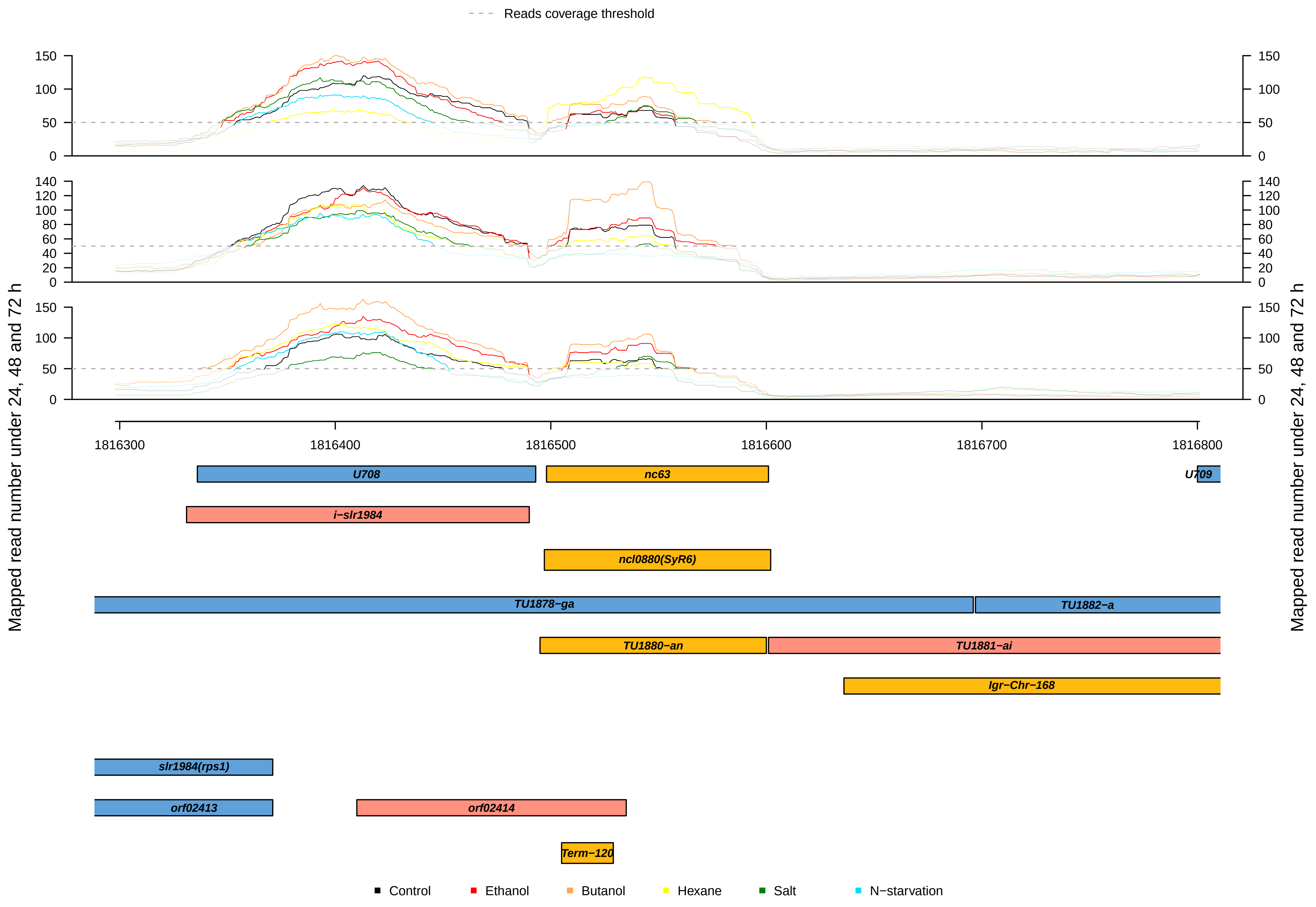

Reads coverage threshold
Mapped read number under 24, 48 and 72 h
150
100
50
0
140
120
100
80
60
40
20
0
1816300
1816400
1816500
1816600
1816700
1816800
U708
nc63
U709
i−slr1984
ncl0880(SyR6)
TU1878−ga
TU1882−a
TU1880−an
TU1881−ai
Igr−Chr−168
slr1984(rps1)
orf02413
orf02414
Term−120
Control
Ethanol
Butanol
Hexane
Salt
N−starvation

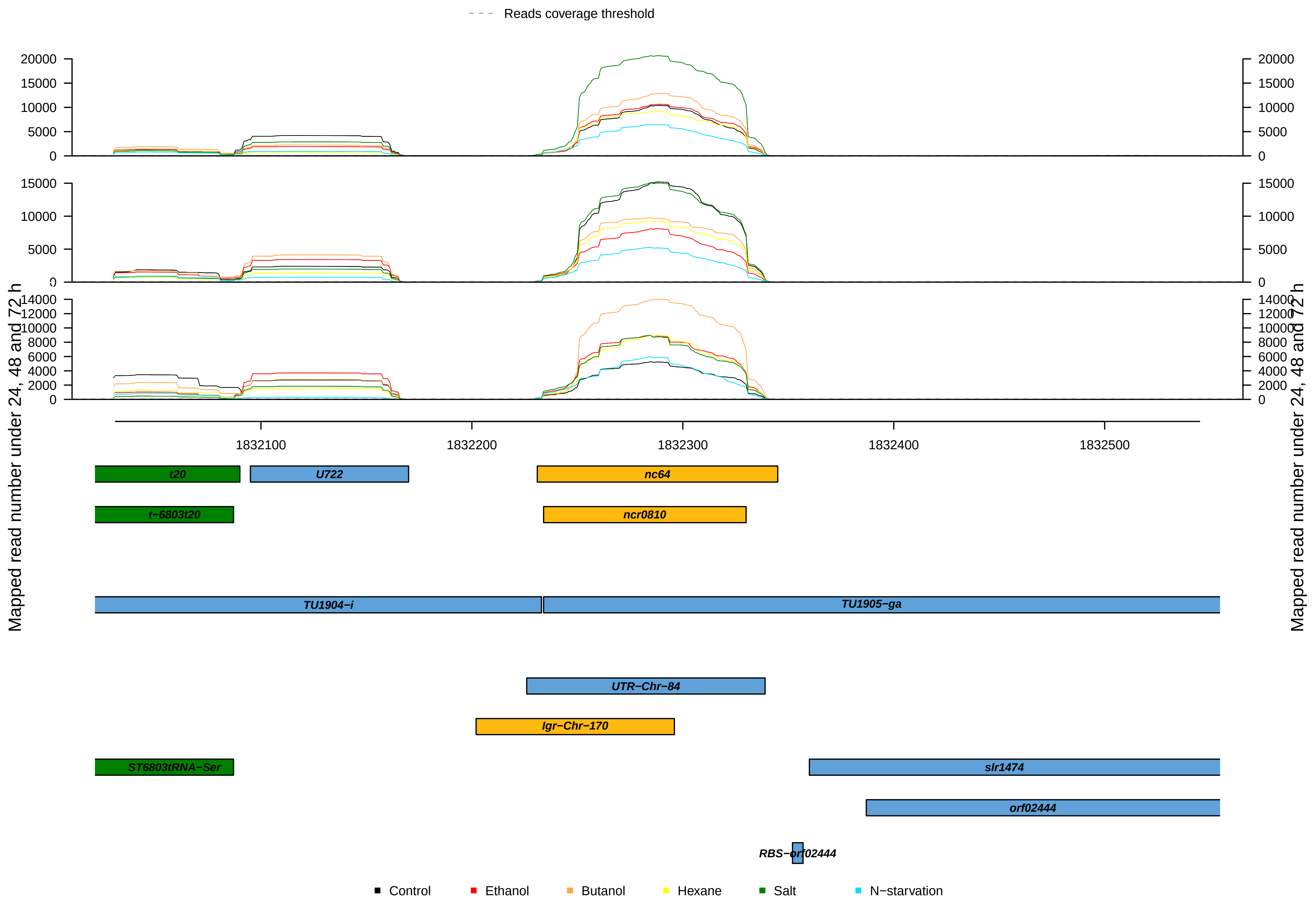

Reads coverage threshold
20000
15000
10000
5000
0
15000
10000
5000
0
14000
12000
10000
8000
6000
4000
2000
0
Mapped read number under 24, 48 and 72 h
1832100
1832200
1832300
1832400
1832500
t20
U722
nc64
t−6803t20
ncr0810
TU1904−i
TU1905−ga
UTR−Chr−84
Igr−Chr−170
ST6803tRNA−Ser
slr1474
orf02444
RBS−orf02444
Control
Ethanol
Butanol
Hexane
Salt
N−starvation

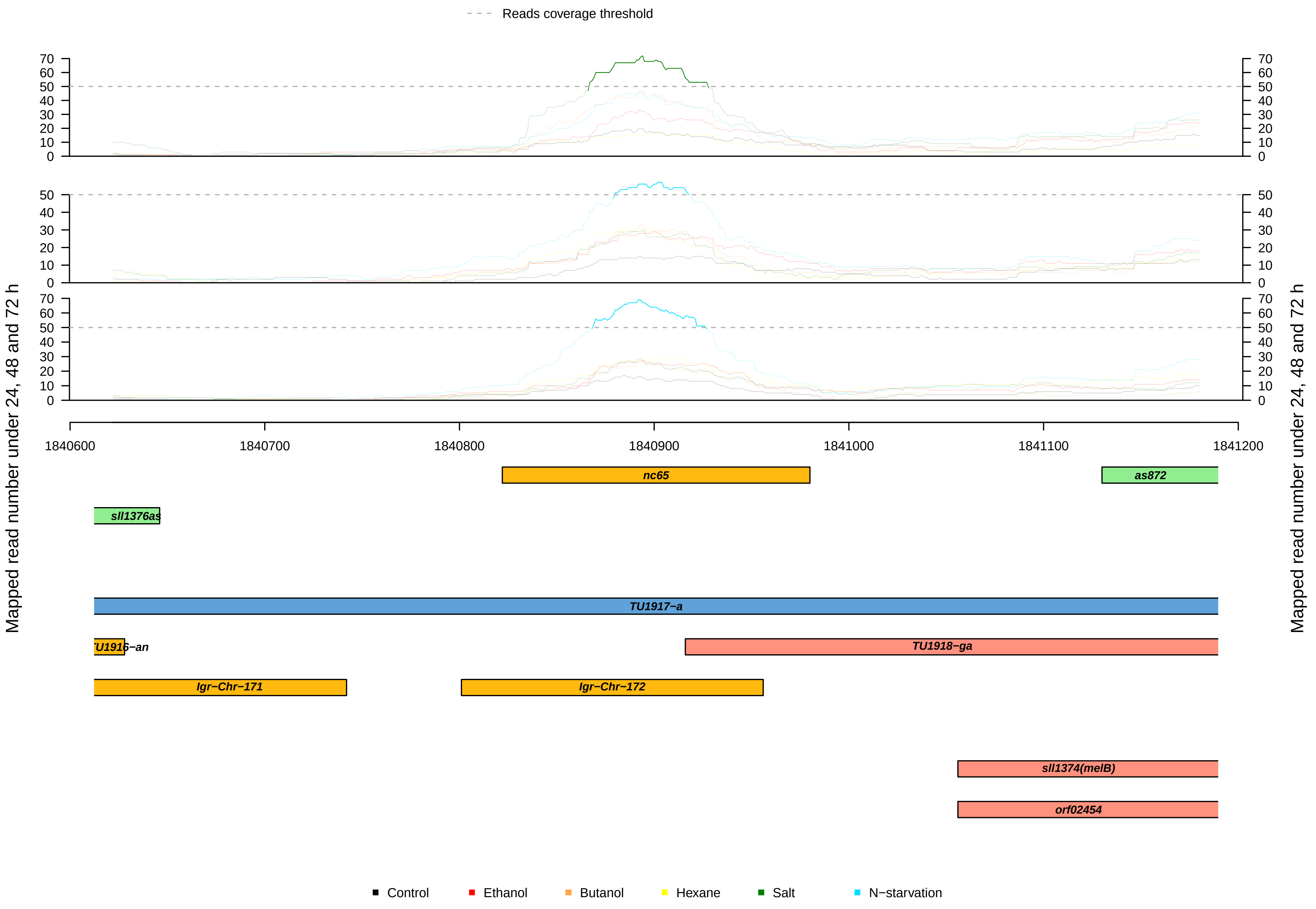

Reads coverage threshold
Mapped read number under 24, 48 and 72 h
1840600
1840700
1840800
1840900
1841000
1841100
1841200
nc65
as872
sll1376as
TU1917−a
TU1916−an
TU1918−ga
Igr−Chr−171
Igr−Chr−172
sll1374(melB)
orf02454
Control
Ethanol
Butanol
Hexane
Salt
N−starvation

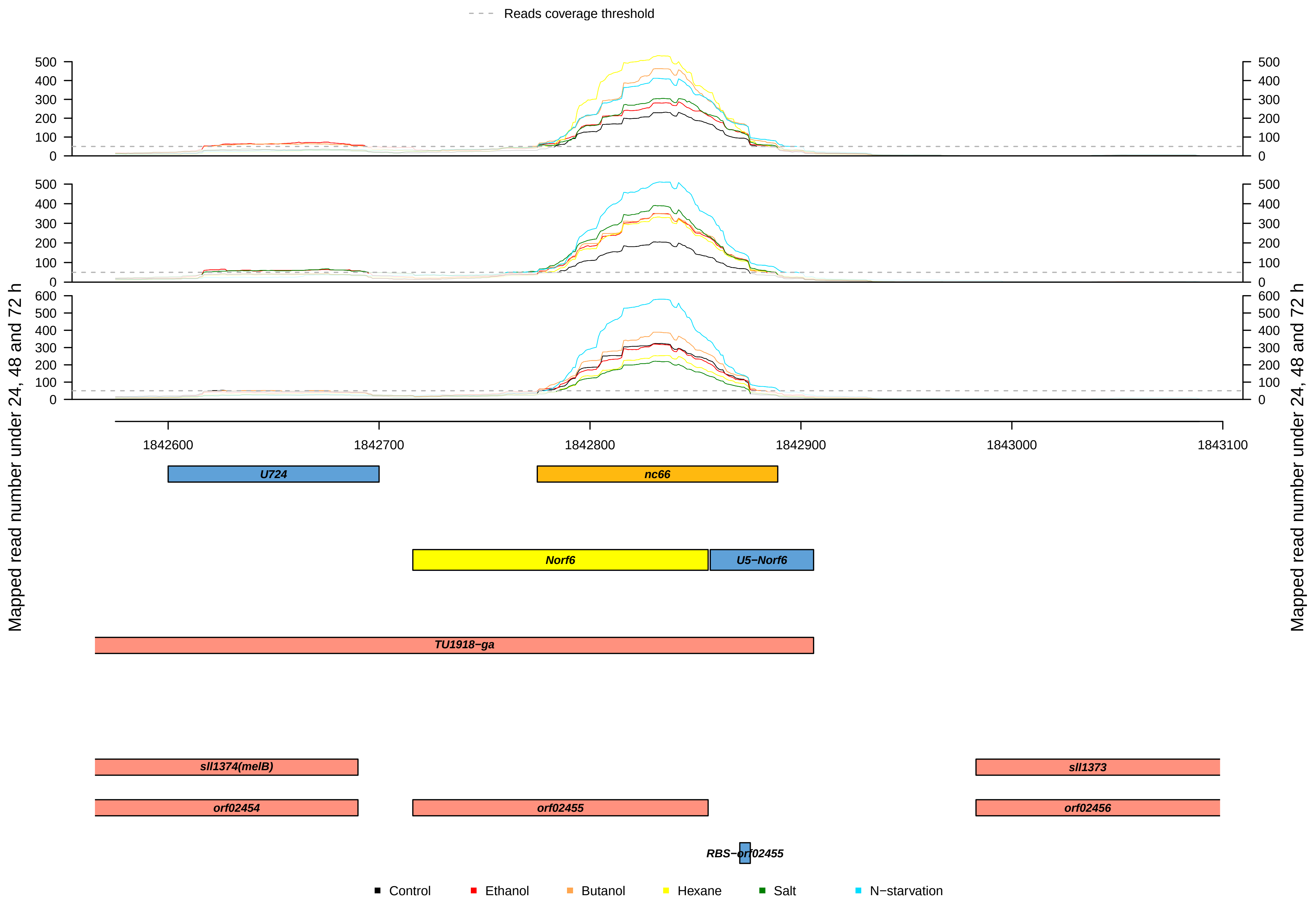
Reads coverage threshold
Mapped read number under 24, 48 and 72 h
500
400
300
200
100
0
600
1842600
1842700
1842800
1842900
1843000
1843100
U724
nc66
Norf6
U5−Norf6
TU1918−ga
sll1374(melB)
sll1373
orf02454
orf02455
orf02456
RBS−orf02455
Control
Ethanol
Butanol
Hexane
Salt
N−starvation

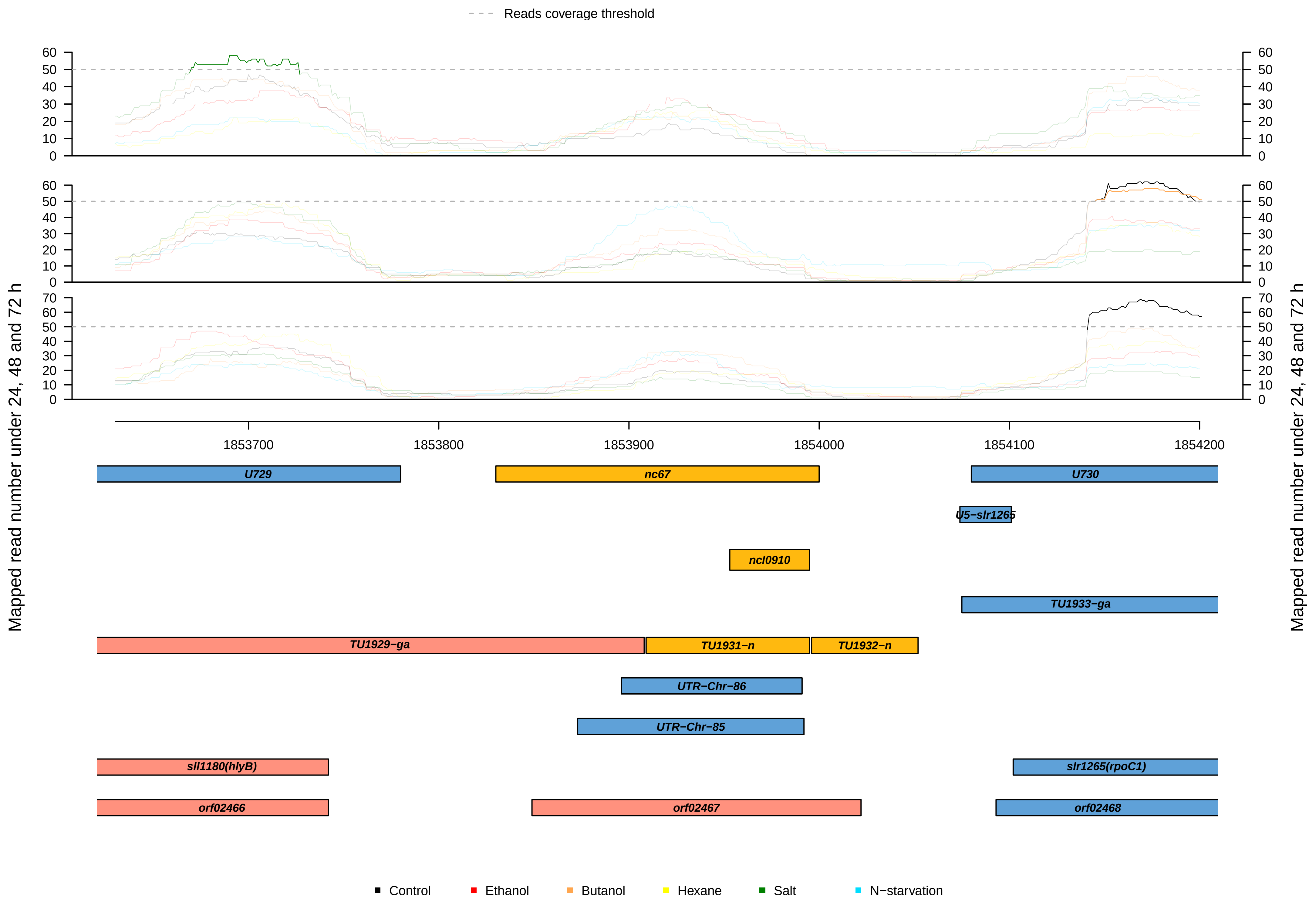

Reads coverage threshold
Mapped read number under 24, 48 and 72 h
1853700
1853800
1853900
1854000
1854100
1854200
U729
nc67
U730
U5−slr1265
ncl0910
TU1933−ga
TU1929−ga
TU1931−n
TU1932−n
UTR−Chr−86
UTR−Chr−85
sll1180(hlyB)
slr1265(rpoC1)
orf02466
orf02467
orf02468
Control
Ethanol
Butanol
Hexane
Salt
N−starvation

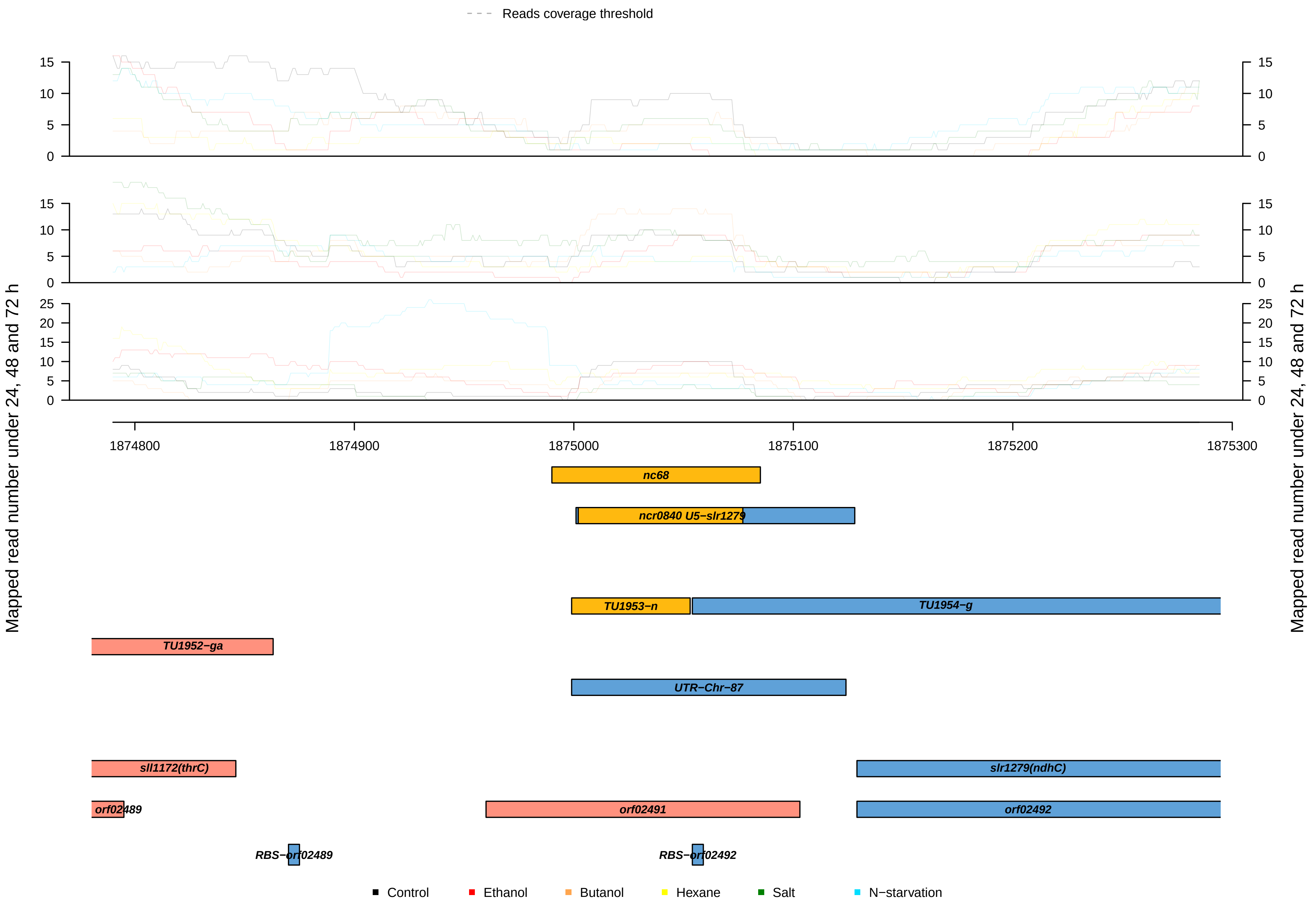
Reads coverage threshold
Mapped read number under 24, 48 and 72 h
1874800
1874900
1875000
1875100
1875200
1875300
nc68
ncr0840 U5−slr1279
TU1953−n
TU1954−g
TU1952−ga
UTR−Chr−87
sll1172(thrC)
slr1279(ndhC)
orf02489
orf02491
orf02492
RBS−orf02489
RBS−orf02492
Control
Ethanol
Butanol
Hexane
Salt
N−starvation

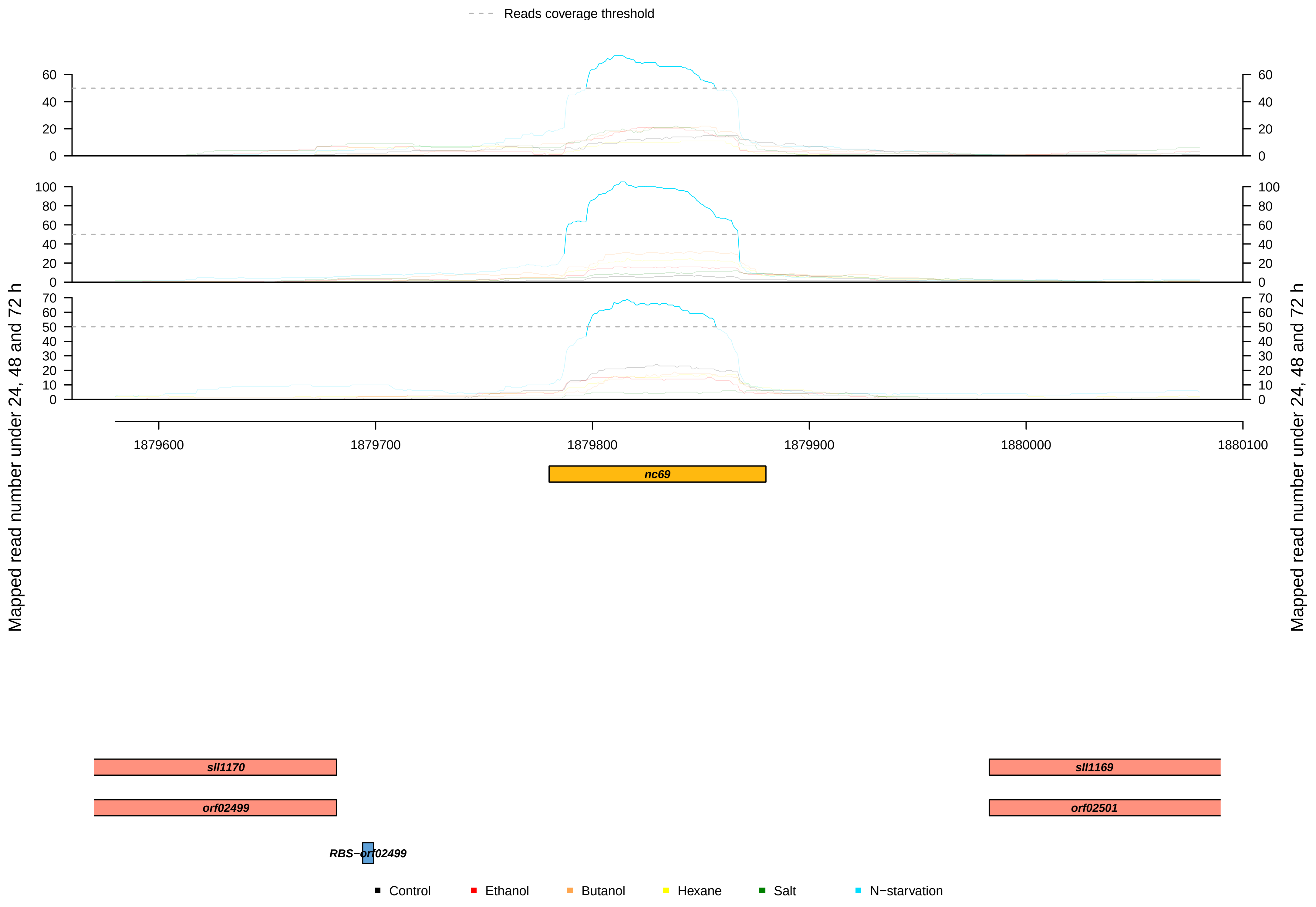

Reads coverage threshold
0
20
40
60
0
20
40
60
80
100
0
10
20
30
40
50
60
70
Mapped read number under 24, 48 and 72 h
Mapped read number under 24, 48 and 72 h
1879600
1879700
1879800
1879900
1880000
1880100
nc69
sll1170
sll1169
orf02499
orf02501
RBS−orf02499
Control
Ethanol
Butanol
Hexane
Salt
N−starvation

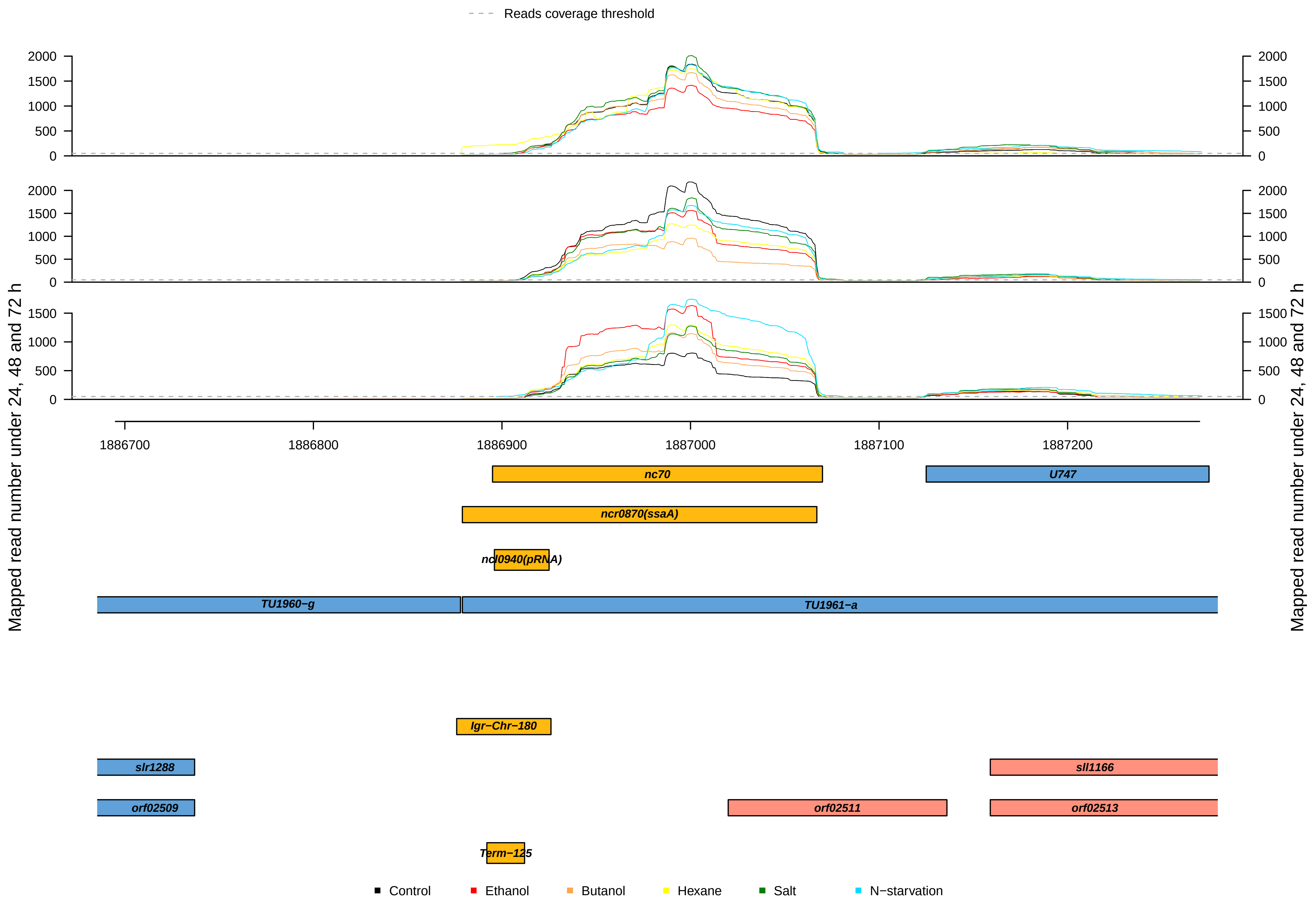

Reads coverage threshold
2000
1500
1000
500
0
Mapped read number under 24, 48 and 72 h
1886700
1886800
1886900
1887000
1887100
1887200
nc70
U747
ncr0870(ssaA)
ncl0940(pRNA)
TU1960−g
TU1961−a
Igr−Chr−180
slr1288
sll1166
orf02509
orf02511
orf02513
Term−125
Control
Ethanol
Butanol
Hexane
Salt
N−starvation

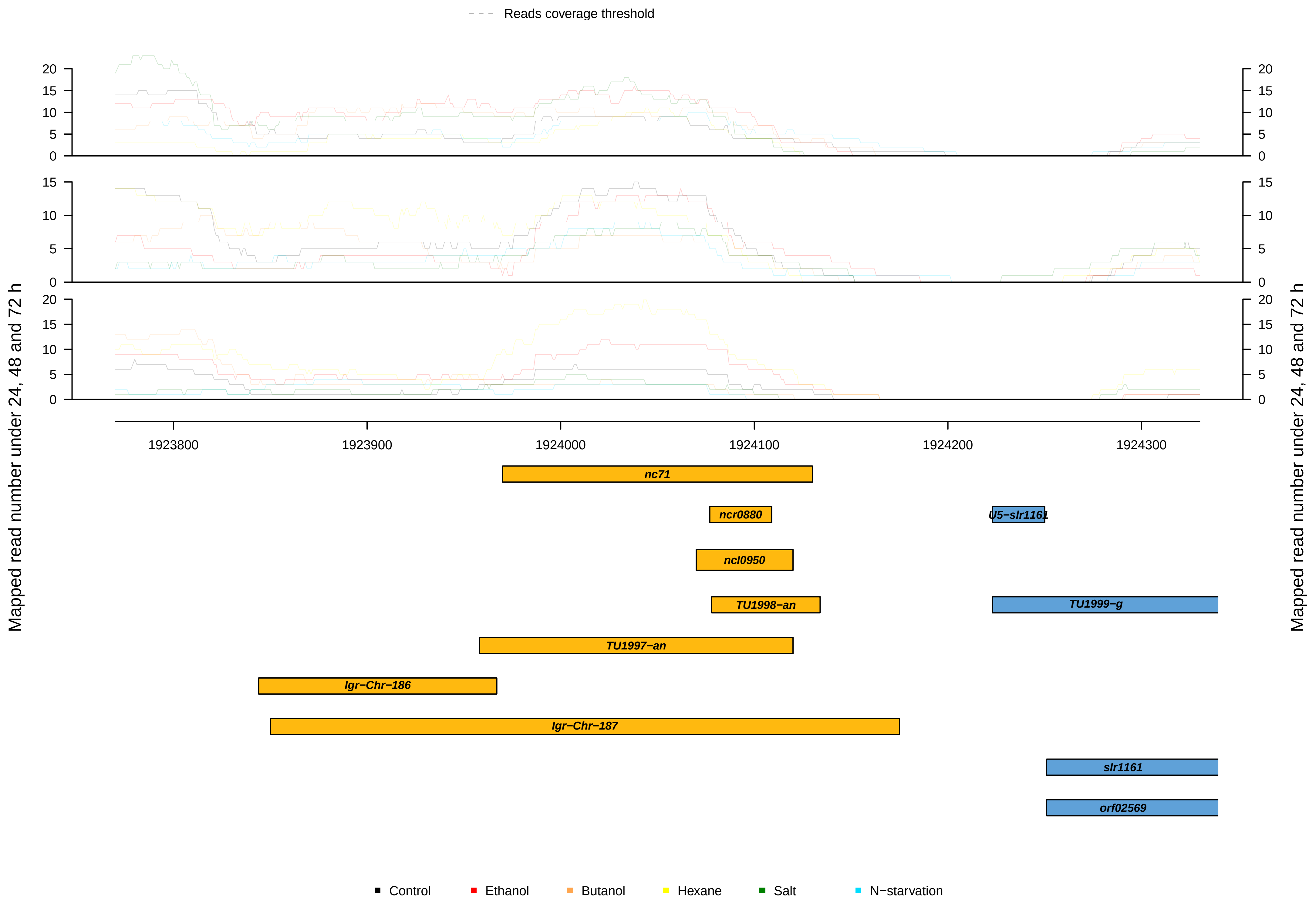

Reads coverage threshold
Mapped read number under 24, 48 and 72 h
20
15
10
5
0
1923800
1923900
1924000
1924100
1924200
1924300
nc71
ncr0880
U5−slr1161
ncl0950
TU1998−an
TU1999−g
TU1997−an
Igr−Chr−186
Igr−Chr−187
slr1161
orf02569
Control
Ethanol
Butanol
Hexane
Salt
N−starvation

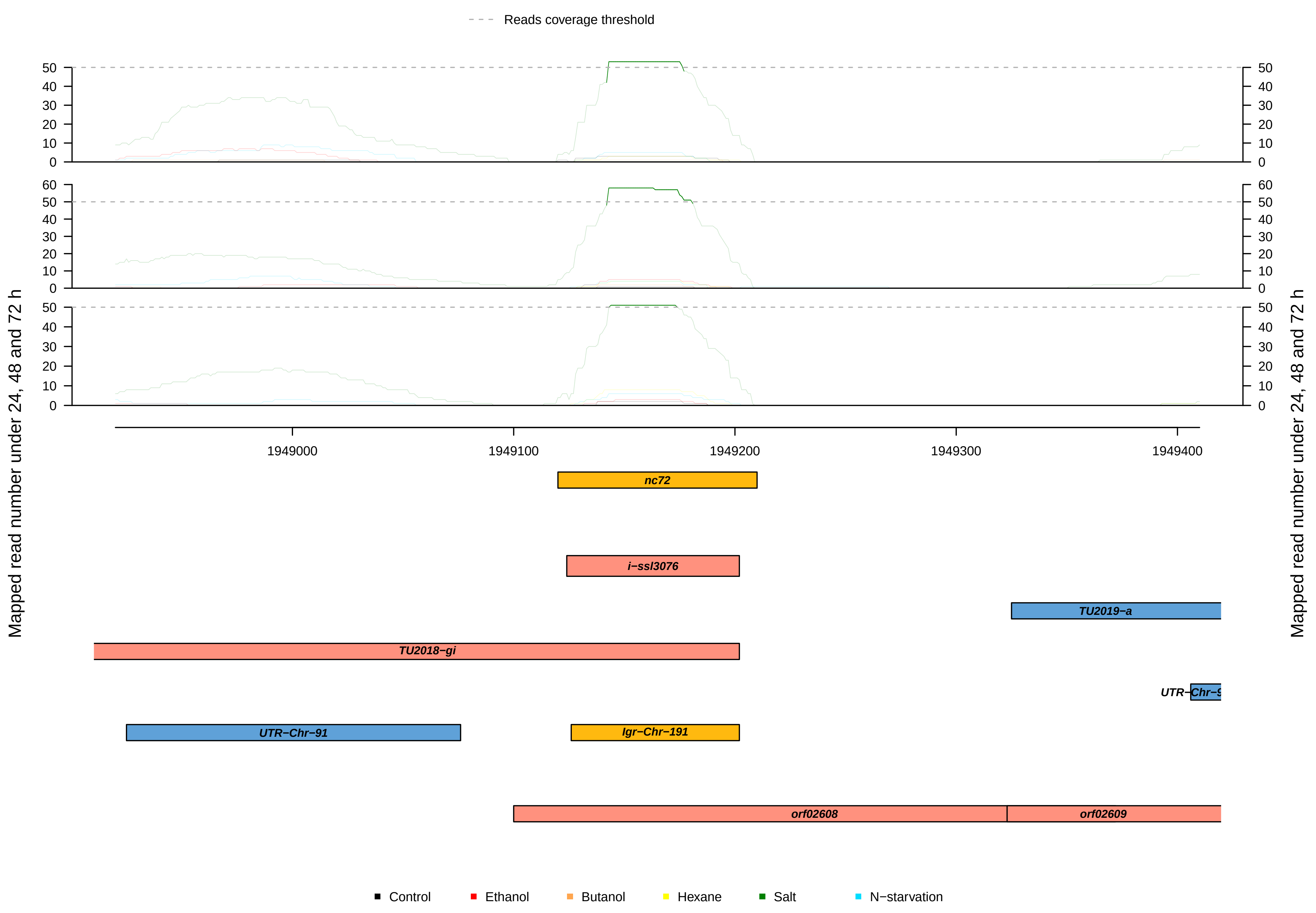

Reads coverage threshold
Mapped read number under 24, 48 and 72 h
1949000
1949100
1949200
1949300
1949400
nc72
i−ssl3076
TU2019−a
TU2018−gi
UTR−Chr−9
UTR−Chr−91
Igr−Chr−191
orf02608
orf02609
Control
Ethanol
Butanol
Hexane
Salt
N−starvation

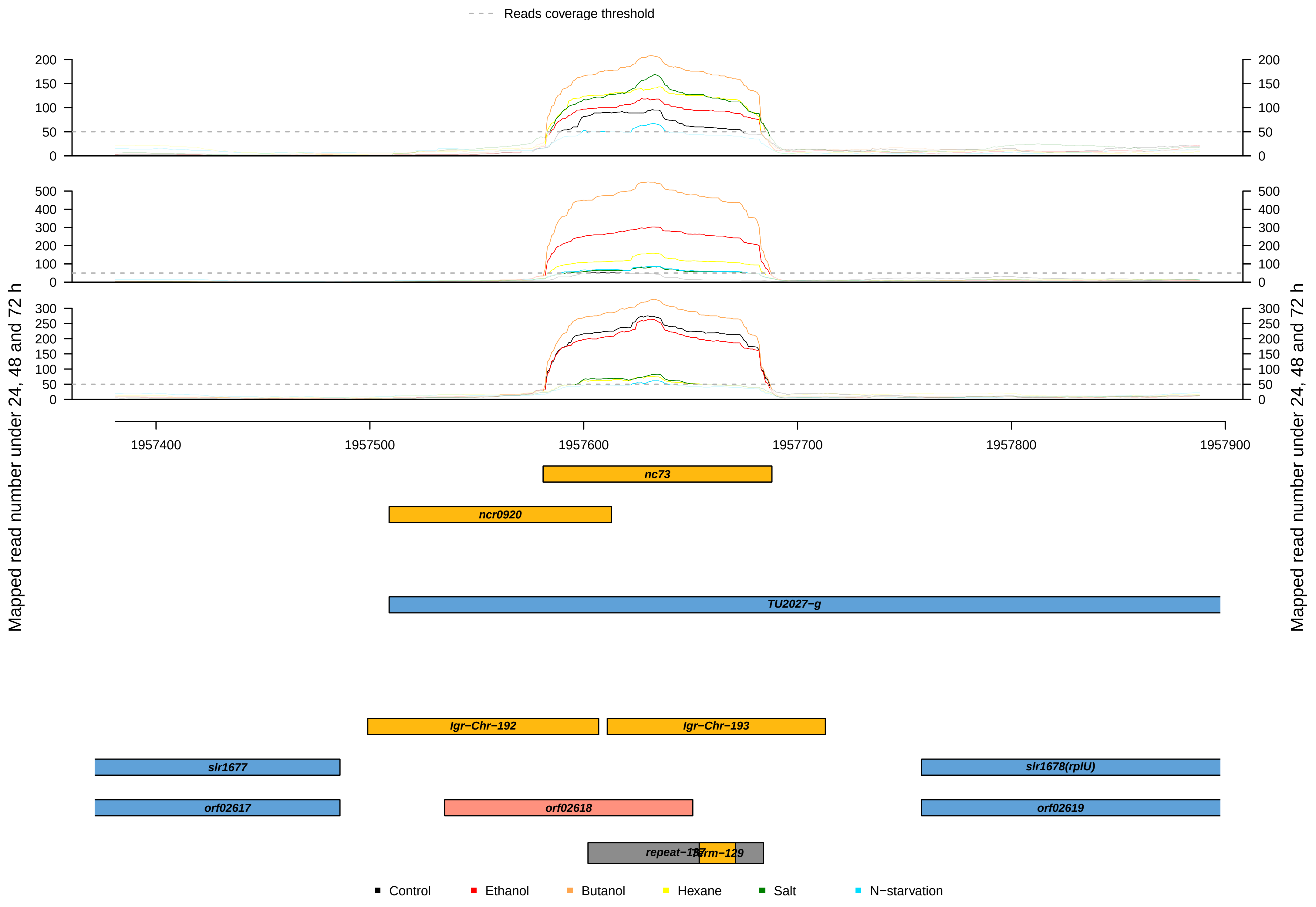

Reads coverage threshold
Mapped read number under 24, 48 and 72 h
1957400
1957500
1957600
1957700
1957800
1957900
nc73
ncr0920
TU2027−g
Igr−Chr−192
Igr−Chr−193
slr1677
slr1678(rplU)
orf02617
orf02618
orf02619
repeat−137
Term−129
Control
Ethanol
Butanol
Hexane
Salt
N−starvation

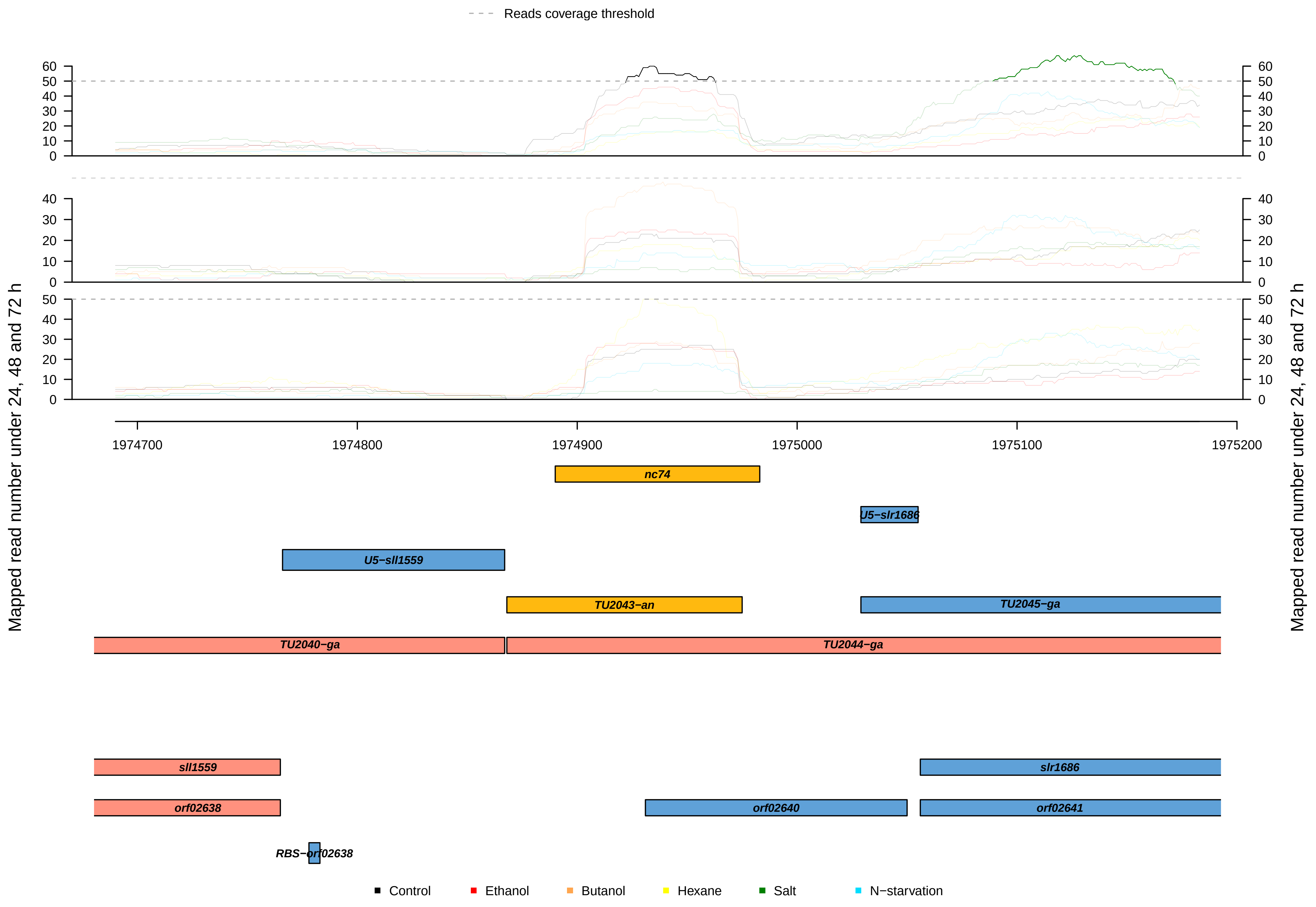

Reads coverage threshold
Mapped read number under 24, 48 and 72 h
1974700
1974800
1974900
1975000
1975100
1975200
nc74
U5−slr1686
U5−sll1559
TU2043−an
TU2045−ga
TU2040−ga
TU2044−ga
sll1559
slr1686
orf02638
orf02640
orf02641
RBS−orf02638
Control
Ethanol
Butanol
Hexane
Salt
N−starvation

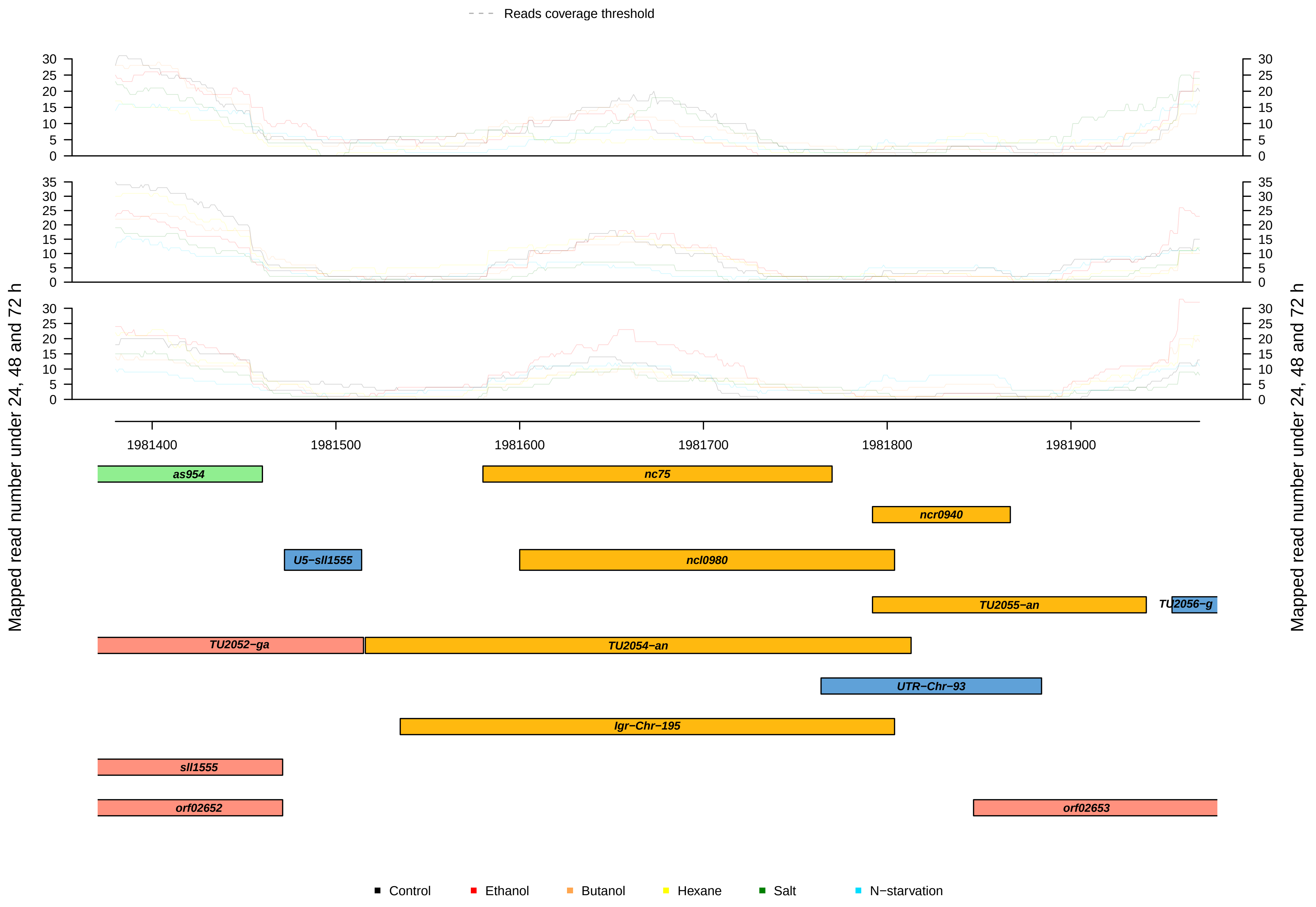

Reads coverage threshold
Mapped read number under 24, 48 and 72 h
1981400
1981500
1981600
1981700
1981800
1981900
as954
nc75
ncr0940
U5−sll1555
ncl0980
TU2055−an
TU2056−g
TU2052−ga
TU2054−an
UTR−Chr−93
Igr−Chr−195
sll1555
orf02652
orf02653
Control
Ethanol
Butanol
Hexane
Salt
N−starvation

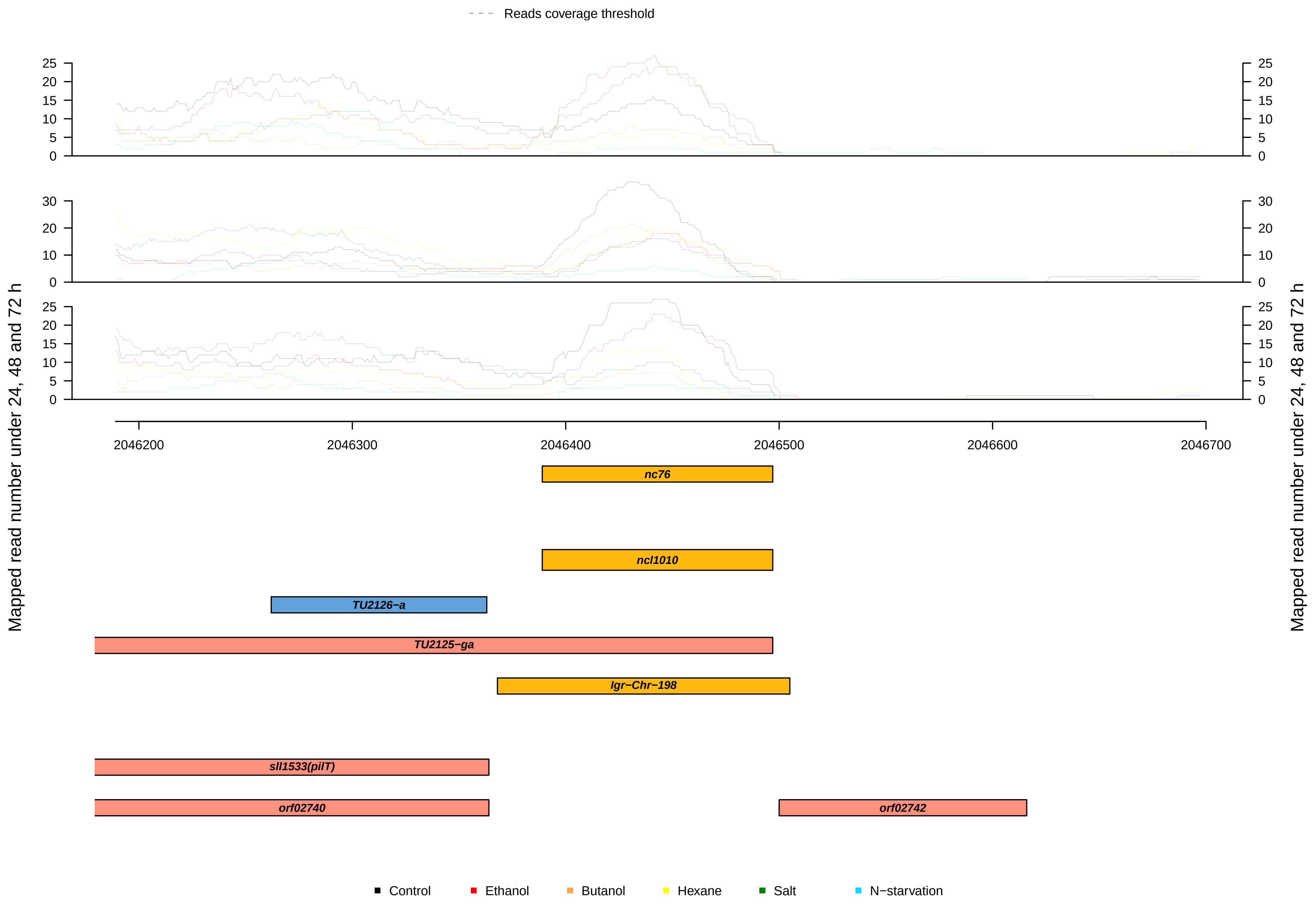

Reads coverage threshold
Mapped read number under 24, 48 and 72 h
2046200
2046300
2046400
2046500
2046600
2046700
nc76
ncl1010
TU2126−a
TU2125−ga
Igr−Chr−198
sll1533(pilT)
orf02740
orf02742
Control
Ethanol
Butanol
Hexane
Salt
N−starvation

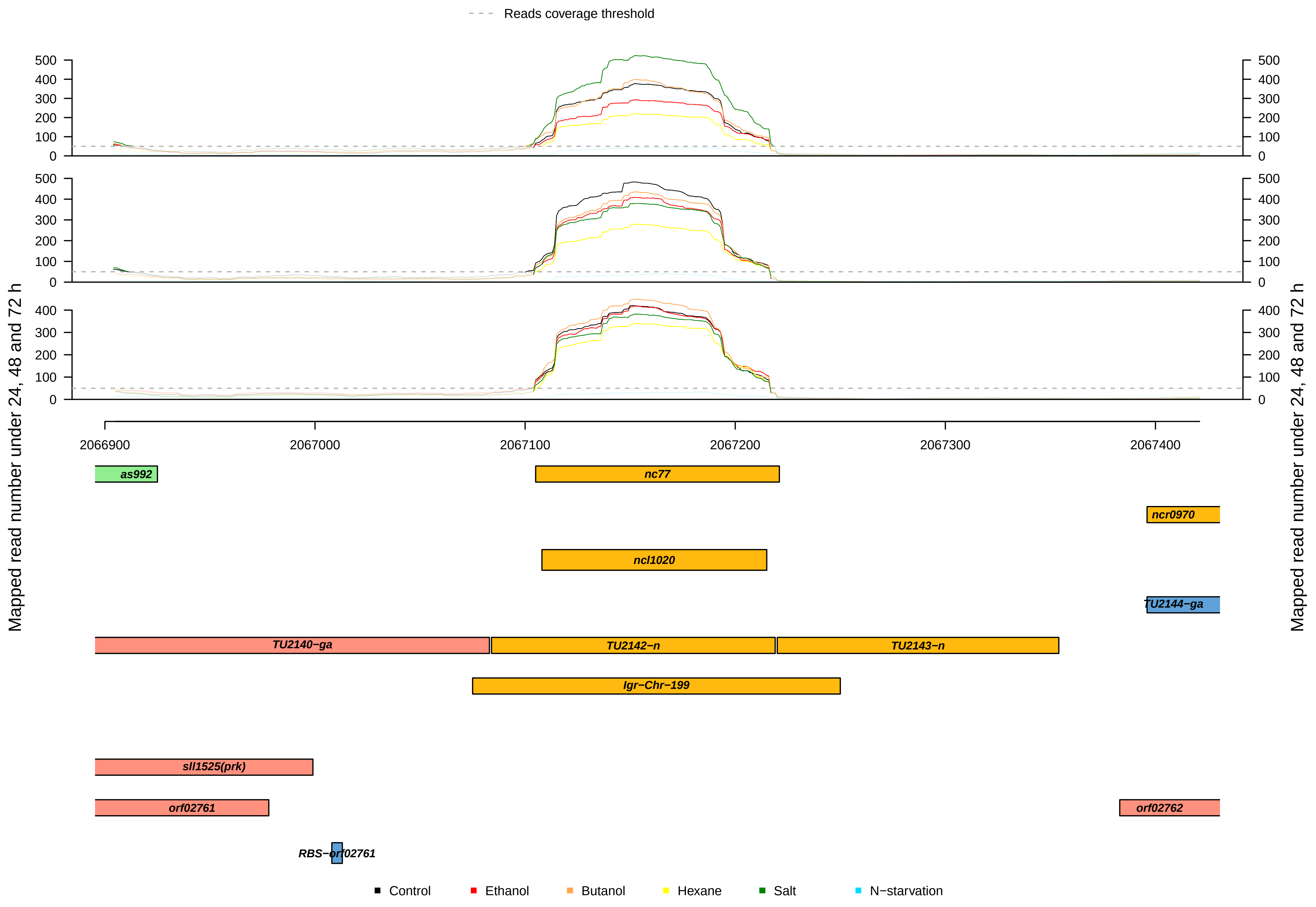

Reads coverage threshold
Mapped read number under 24, 48 and 72 h
500
400
300
200
100
0
2066900
2067000
2067100
2067200
2067300
2067400
as992
nc77
ncr0970
ncl1020
TU2144−ga
TU2140−ga
TU2142−n
TU2143−n
Igr−Chr−199
sll1525(prk)
orf02761
orf02762
RBS−orf02761
Control
Ethanol
Butanol
Hexane
Salt
N−starvation

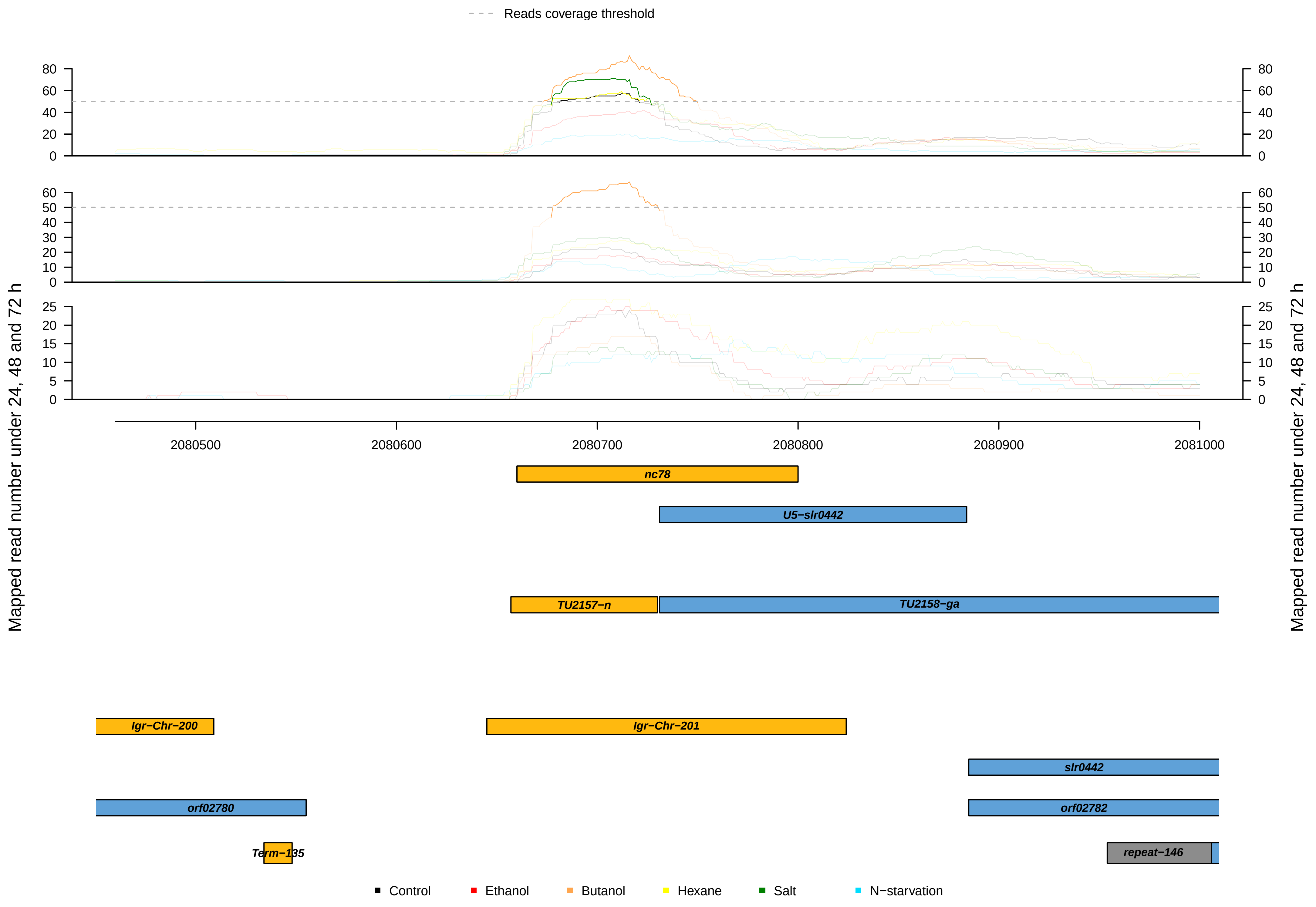

Reads coverage threshold
Mapped read number under 24, 48 and 72 h
80
60
40
20
0
60
50
40
30
20
10
0
25
20
15
10
5
0
2080500
2080600
2080700
2080800
2080900
2081000
nc78
U5−slr0442
TU2157−n
TU2158−ga
Igr−Chr−200
Igr−Chr−201
slr0442
orf02780
orf02782
Term−135
repeat−146
Control
Ethanol
Butanol
Hexane
Salt
N−starvation

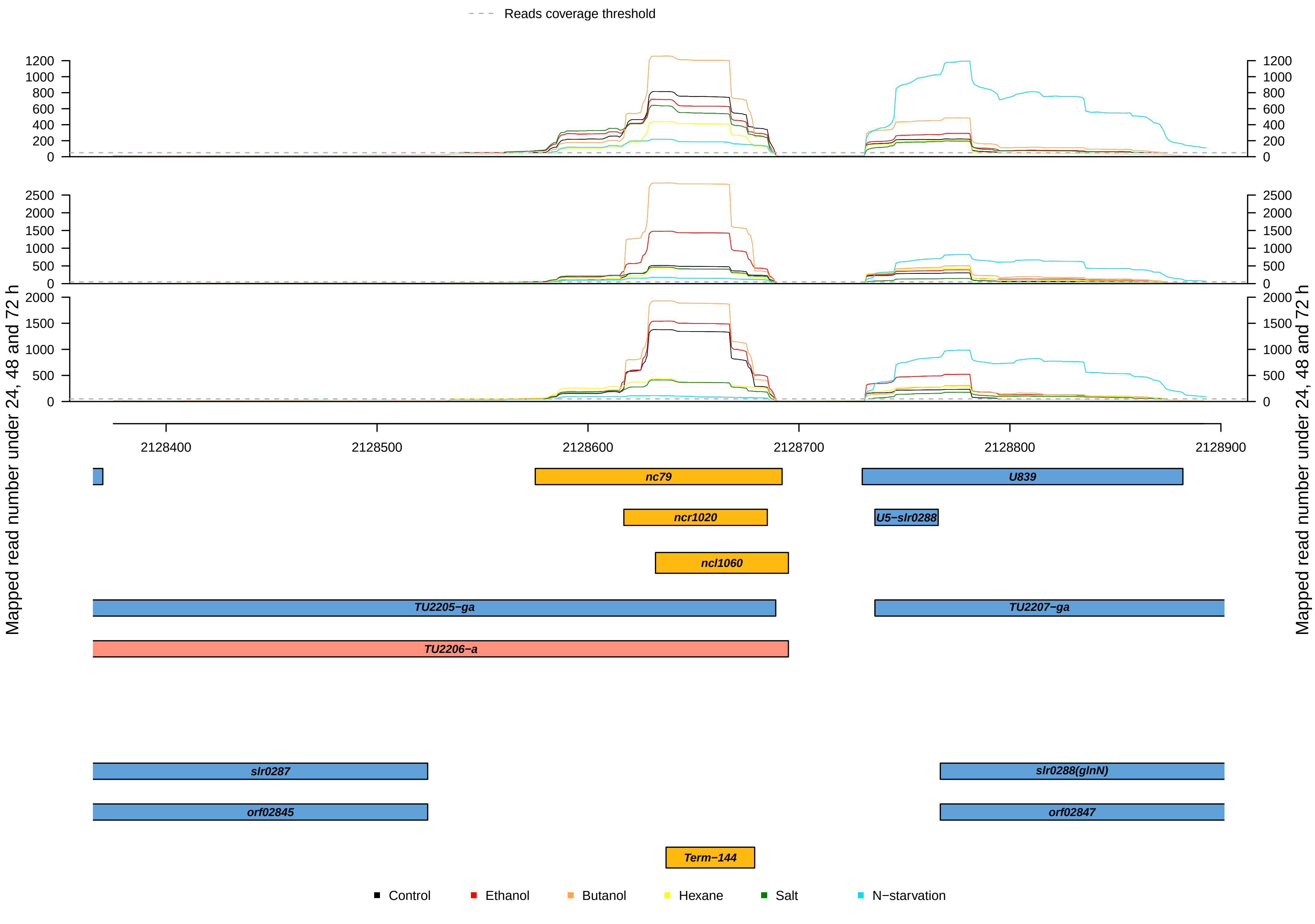

Reads coverage threshold
Mapped read number under 24, 48 and 72 h
1200
1000
800
600
400
200
0
2500
2000
1500
1000
500
0
2000
1500
1000
500
0
2128400
2128500
2128600
2128700
2128800
2128900
nc79
U839
ncr1020
U5−slr0288
ncl1060
TU2205−ga
TU2207−ga
TU2206−a
slr0287
slr0288(glnN)
orf02845
orf02847
Term−144
Control
Ethanol
Butanol
Hexane
Salt
N−starvation

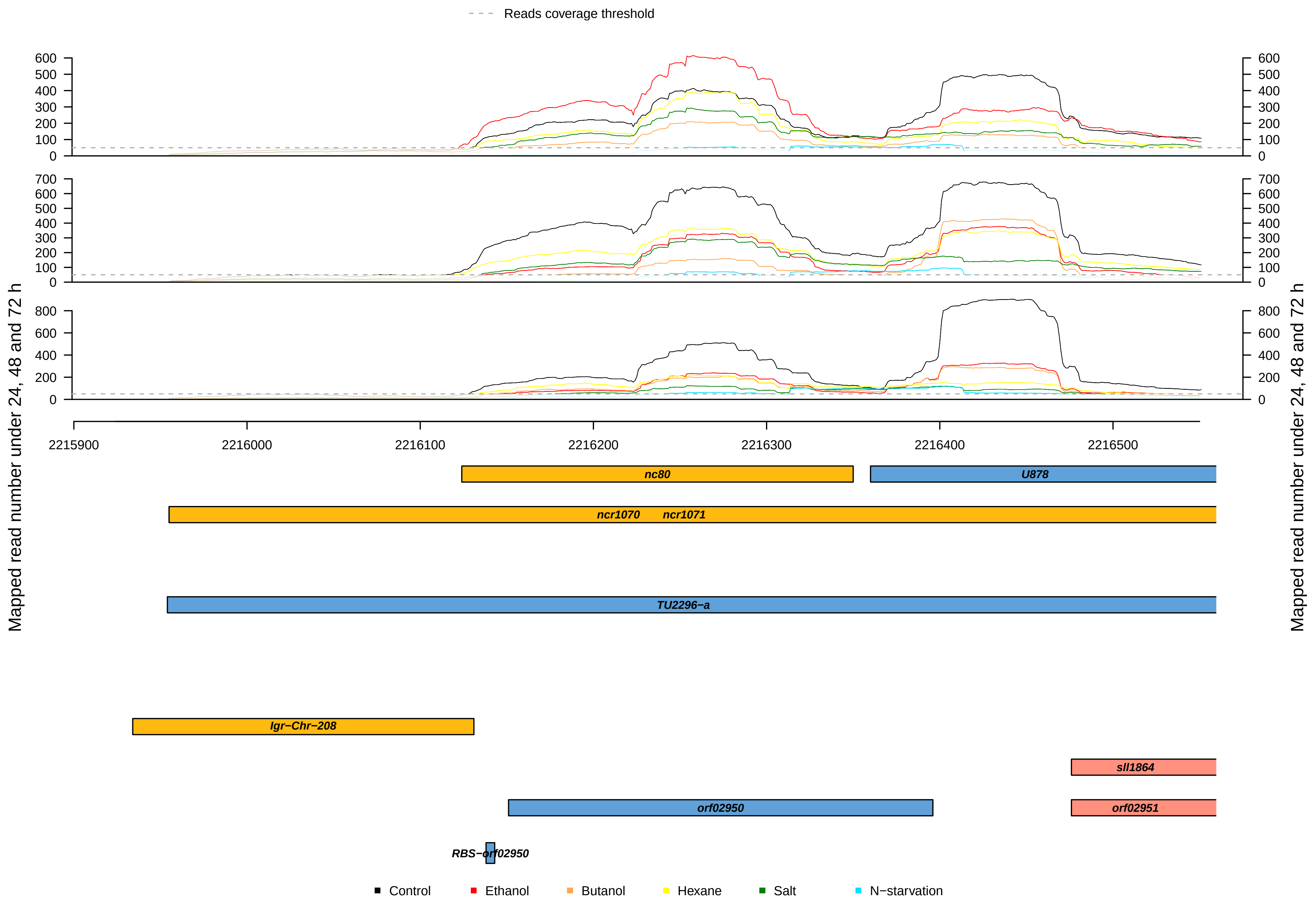

Reads coverage threshold
600
500
400
300
200
100
0
700
800
Mapped read number under 24, 48 and 72 h
2215900
2216000
2216100
2216200
2216300
2216400
2216500
nc80
U878
ncr1070
ncr1071
TU2296−a
Igr−Chr−208
sll1864
orf02950
orf02951
RBS−orf02950
Control
Ethanol
Butanol
Hexane
Salt
N−starvation

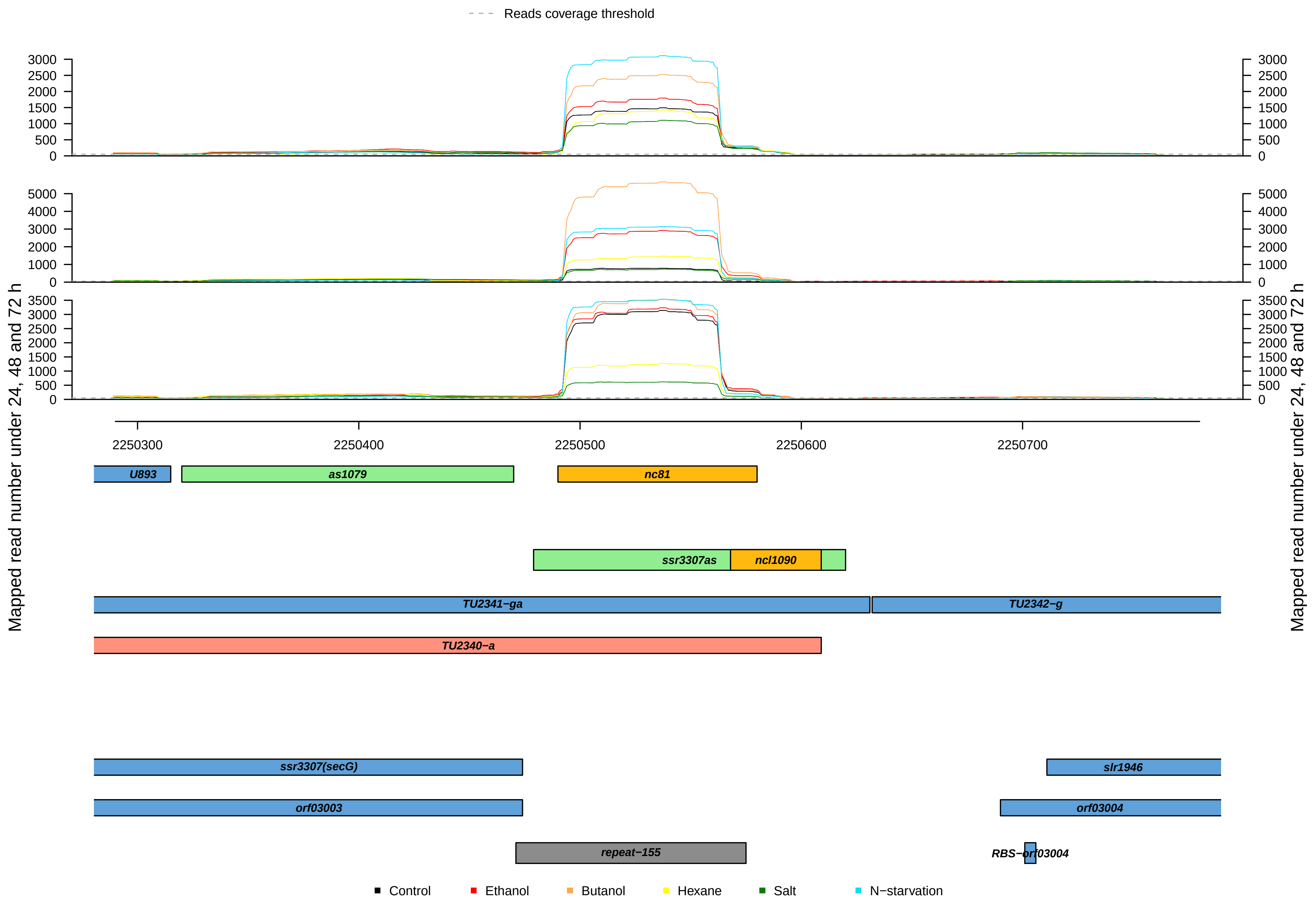
Reads coverage threshold
Mapped read number under 24, 48 and 72 h
3000
2500
2000
1500
1000
500
0
5000
4000
3000
2000
1000
0
3500
3000
2500
2000
1500
1000
500
0
2250300
2250400
2250500
2250600
2250700
U893
as1079
nc81
ssr3307as
ncl1090
TU2341−ga
TU2342−g
TU2340−a
ssr3307(secG)
slr1946
orf03003
orf03004
repeat−155
RBS−orf03004
Control
Ethanol
Butanol
Hexane
Salt
N−starvation

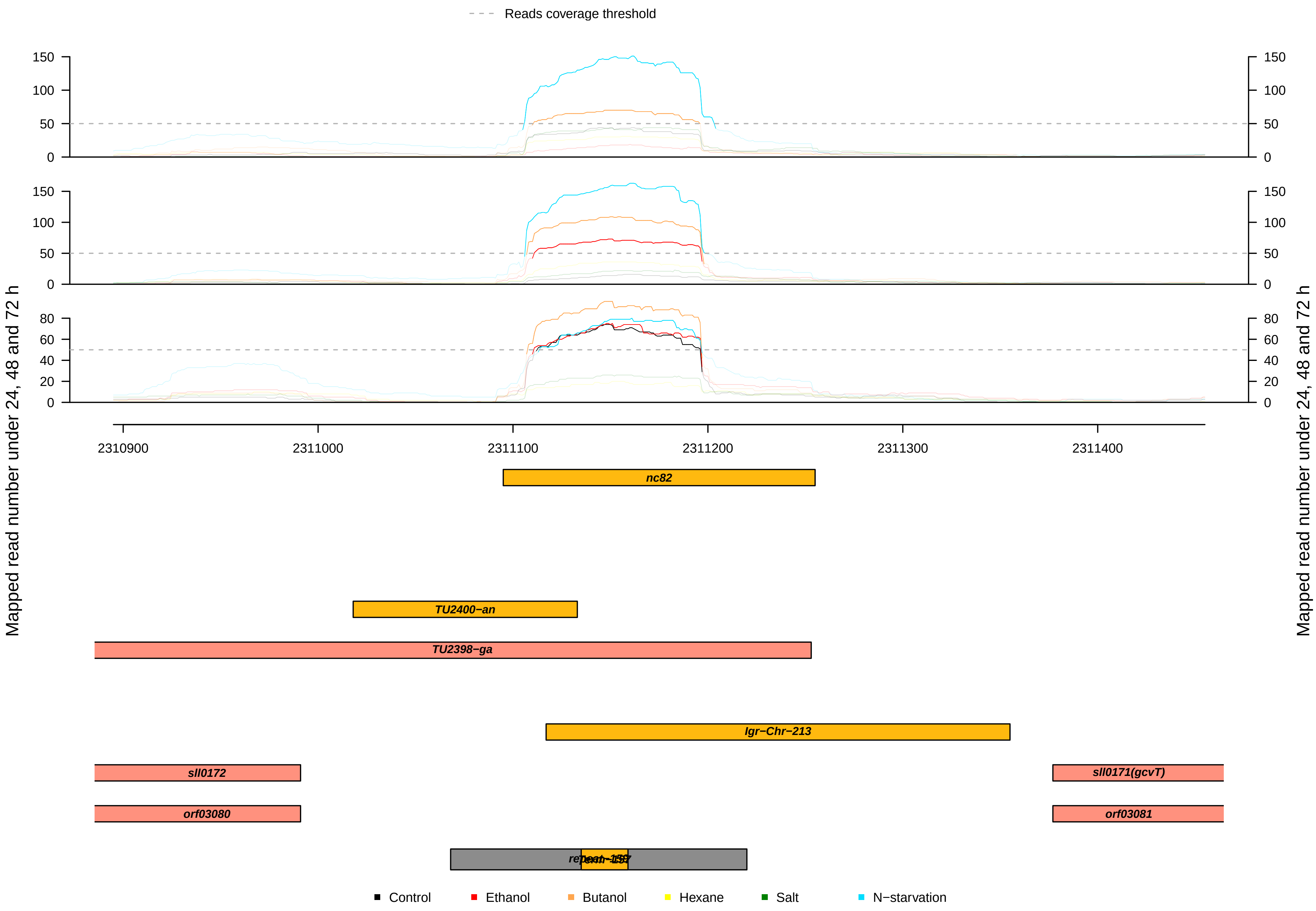

Reads coverage threshold
Mapped read number under 24, 48 and 72 h
150
100
50
0
80
60
40
20
2310900
2311000
2311100
2311200
2311300
2311400
nc82
TU2400−an
TU2398−ga
Igr−Chr−213
sll0172
sll0171(gcvT)
orf03080
orf03081
Control
Ethanol
Butanol
Hexane
Salt
N−starvation

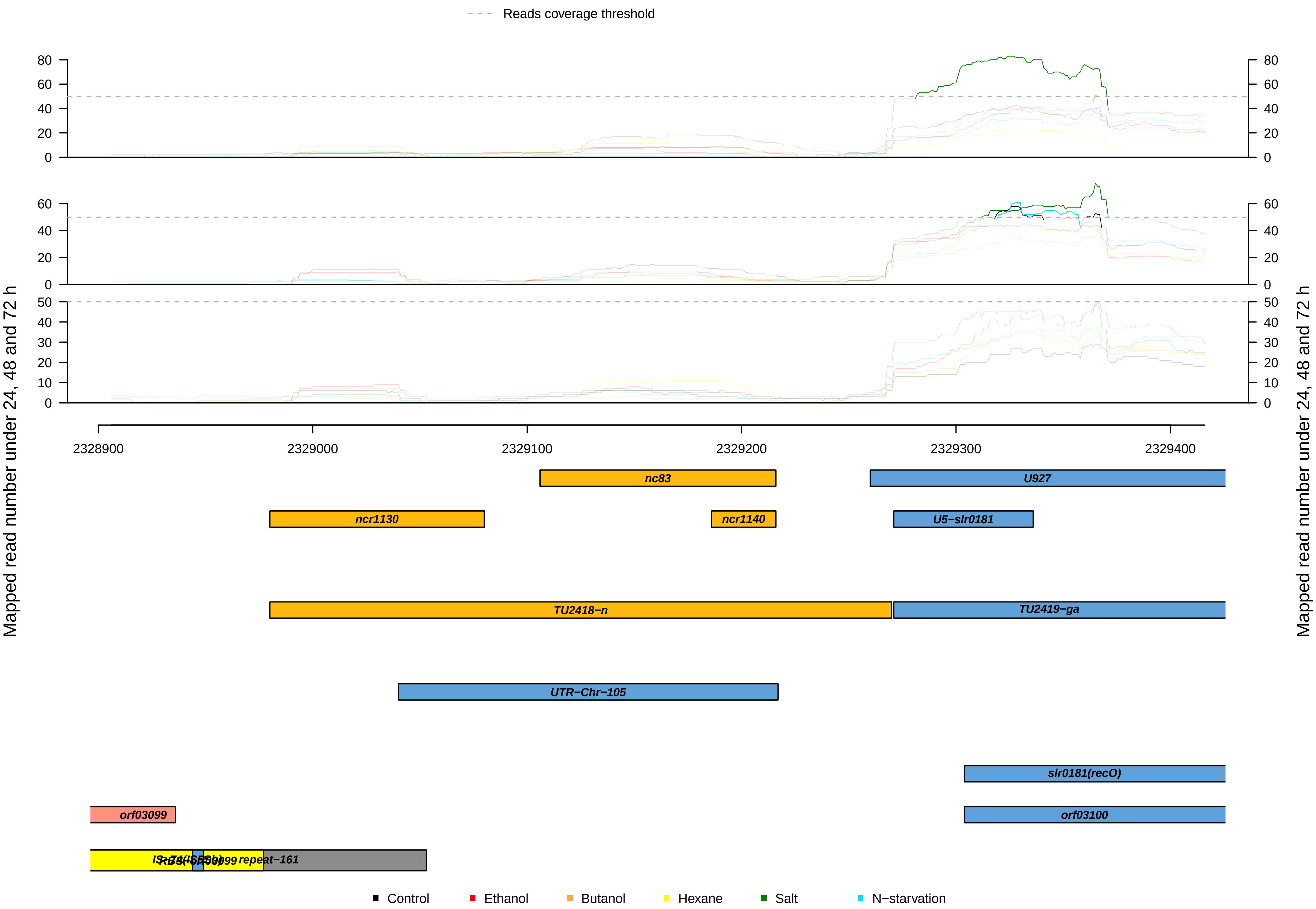

Reads coverage threshold
Mapped read number under 24, 48 and 72 h
2328900
2329000
2329100
2329200
2329300
2329400
nc83
U927
ncr1130
ncr1140
U5−slr0181
TU2418−n
TU2419−ga
UTR−Chr−105
slr0181(recO)
orf03099
orf03100
repeat−161
Control
Ethanol
Butanol
Hexane
Salt
N−starvation

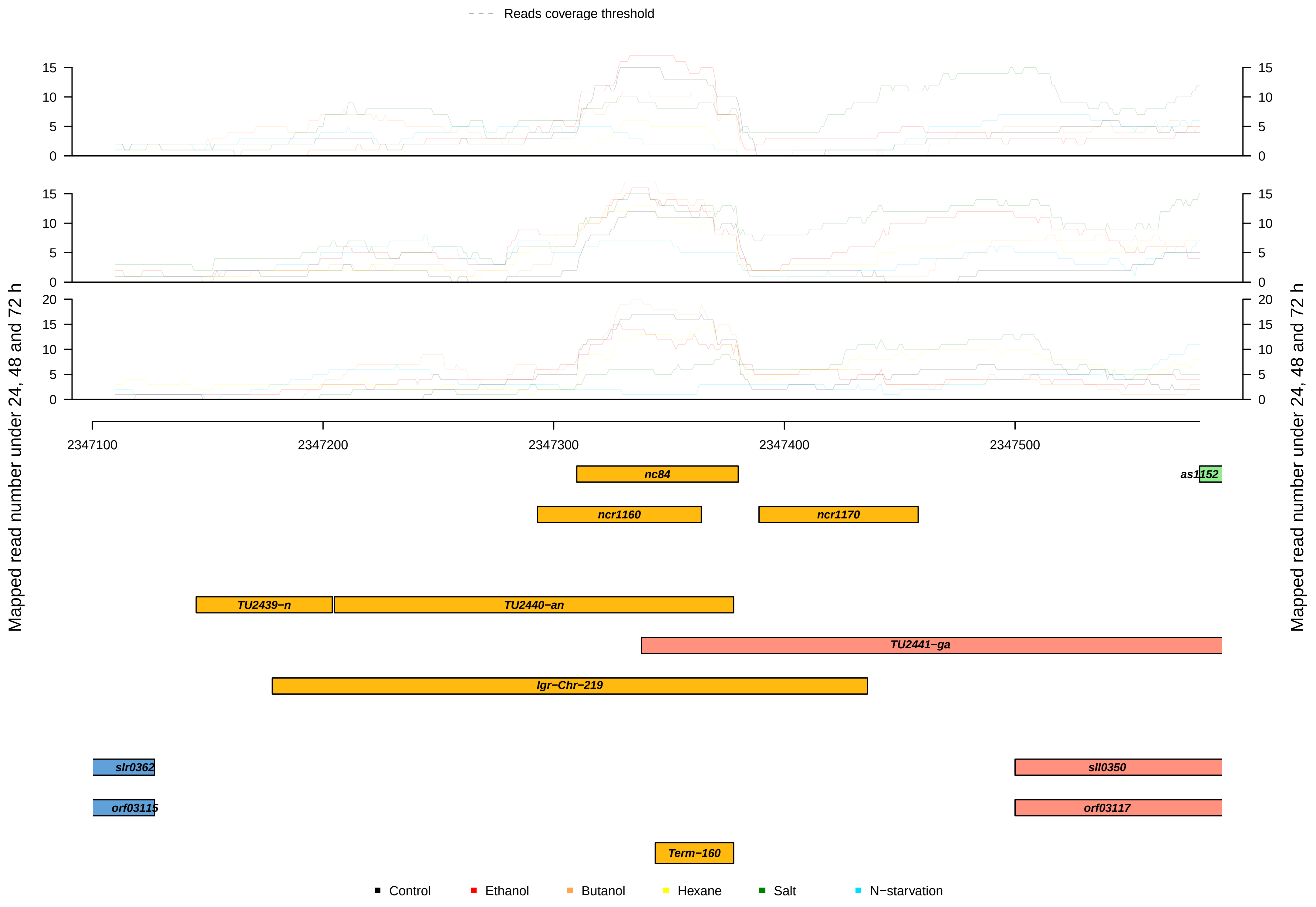

Reads coverage threshold
Mapped read number under 24, 48 and 72 h
2347100
2347200
2347300
2347400
2347500
nc84
as1152
ncr1160
ncr1170
TU2439−n
TU2440−an
TU2441−ga
Igr−Chr−219
slr0362
sll0350
orf03115
orf03117
Term−160
Control
Ethanol
Butanol
Hexane
Salt
N−starvation

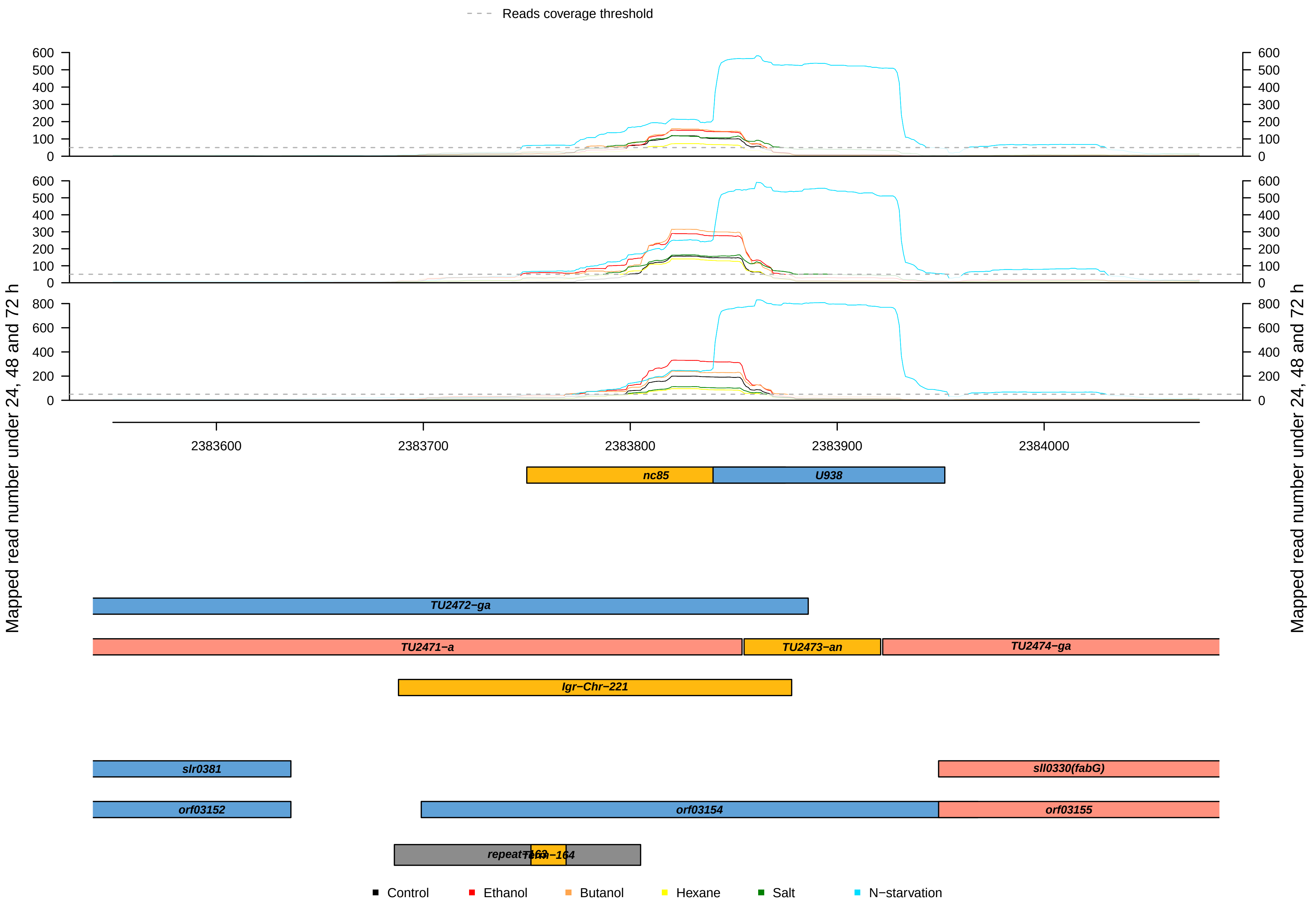

Reads coverage threshold
Mapped read number under 24, 48 and 72 h
600
500
400
300
200
100
0
800
2383600
2383700
2383800
2383900
2384000
nc85
U938
TU2472−ga
TU2471−a
TU2473−an
TU2474−ga
Igr−Chr−221
slr0381
sll0330(fabG)
orf03152
orf03154
orf03155
Control
Ethanol
Butanol
Hexane
Salt
N−starvation

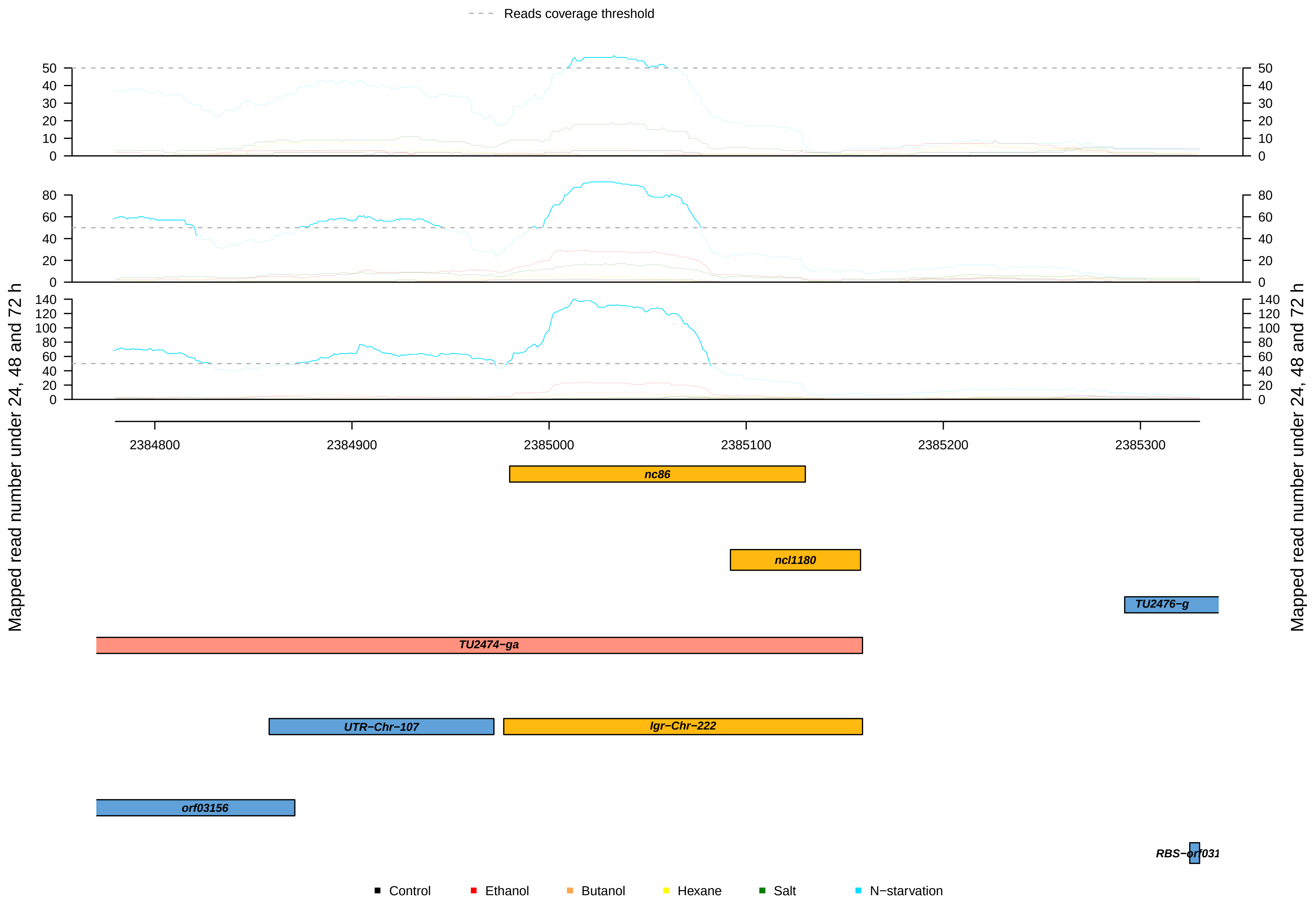

Reads coverage threshold
50
40
30
20
10
0
80
60
40
20
0
140
120
100
80
60
40
20
0
Mapped read number under 24, 48 and 72 h
2384800
2384900
2385000
2385100
2385200
2385300
nc86
ncl1180
TU2476−g
TU2474−ga
UTR−Chr−107
Igr−Chr−222
orf03156
RBS−orf031
Control
Ethanol
Butanol
Hexane
Salt
N−starvation

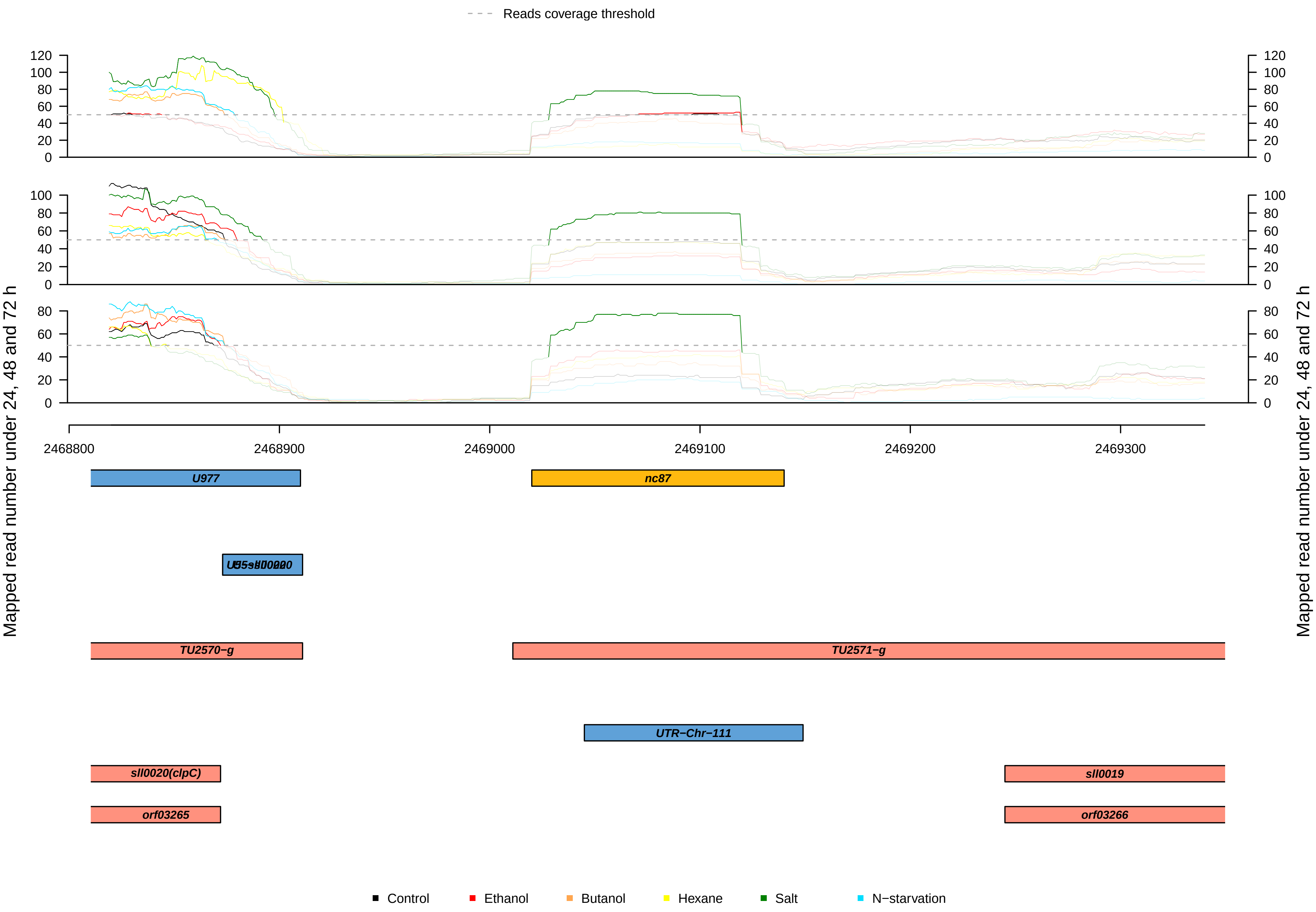

Reads coverage threshold
Mapped read number under 24, 48 and 72 h
2468800
2468900
2469000
2469100
2469200
2469300
U977
nc87
TU2570−g
TU2571−g
UTR−Chr−111
sll0020(clpC)
sll0019
orf03265
orf03266
Control
Ethanol
Butanol
Hexane
Salt
N−starvation

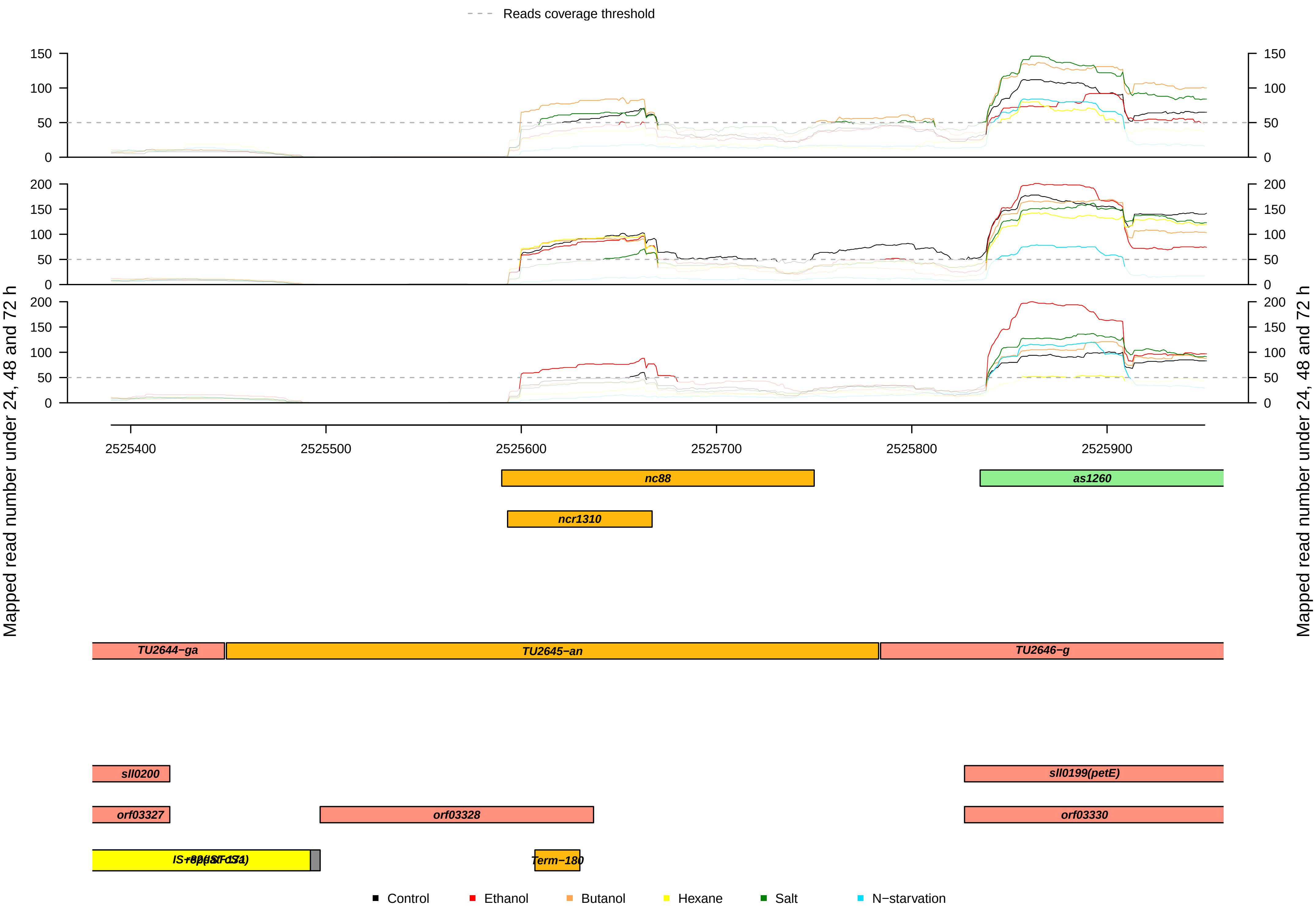

Reads coverage threshold
150
100
50
0
200
150
100
50
0
200
150
100
50
0
Mapped read number under 24, 48 and 72 h
2525400
2525500
2525600
2525700
2525800
2525900
nc88
as1260
ncr1310
TU2644−ga
TU2645−an
TU2646−g
sll0200
sll0199(petE)
orf03327
orf03328
orf03330
Term−180
Control
Ethanol
Butanol
Hexane
Salt
N−starvation

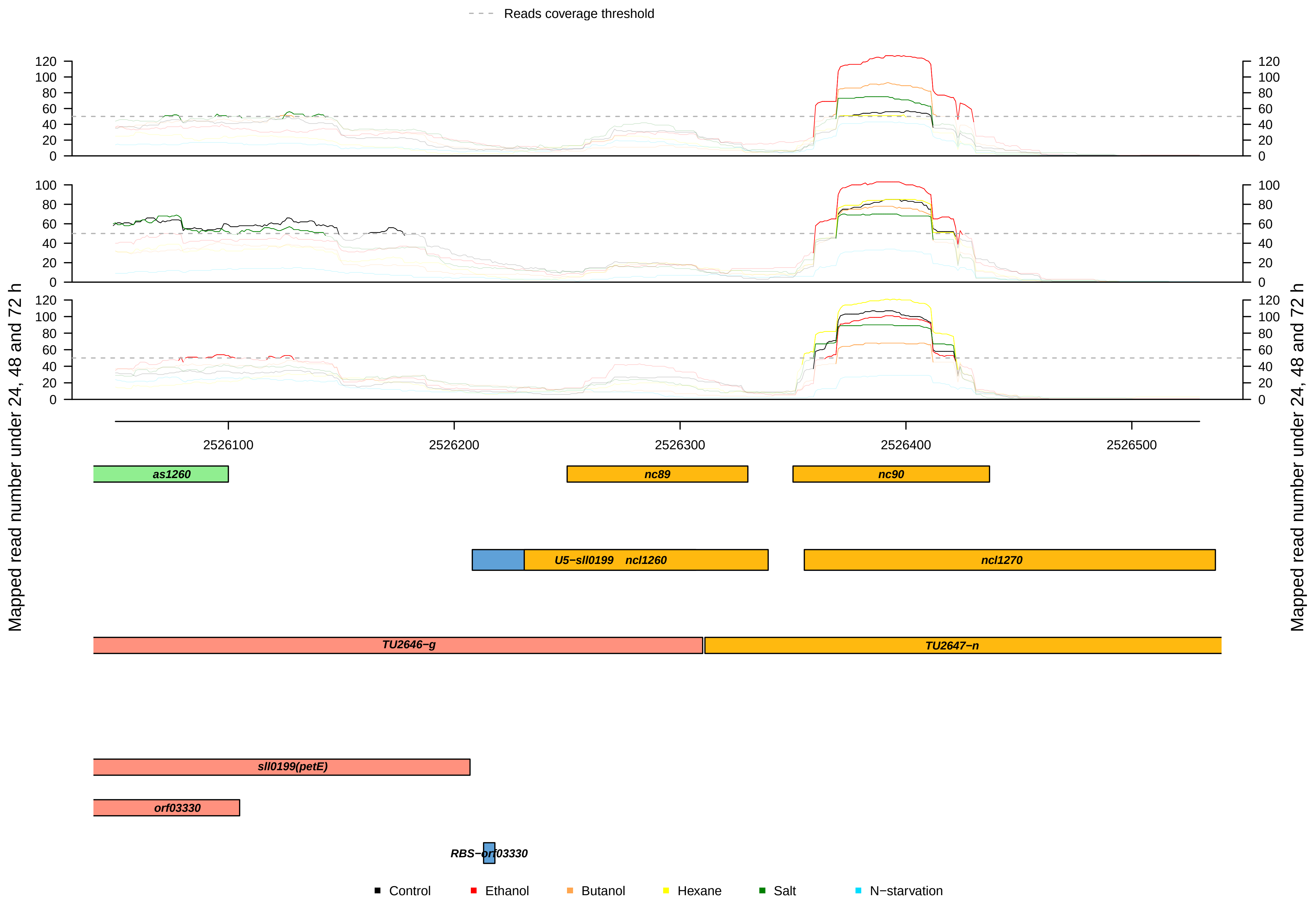

Reads coverage threshold
Mapped read number under 24, 48 and 72 h
0
20
40
60
80
100
120
2526100
2526200
2526300
2526400
2526500
as1260
nc89
nc90
U5−sll0199
ncl1260
ncl1270
TU2646−g
TU2647−n
sll0199(petE)
orf03330
RBS−orf03330
Control
Ethanol
Butanol
Hexane
Salt
N−starvation

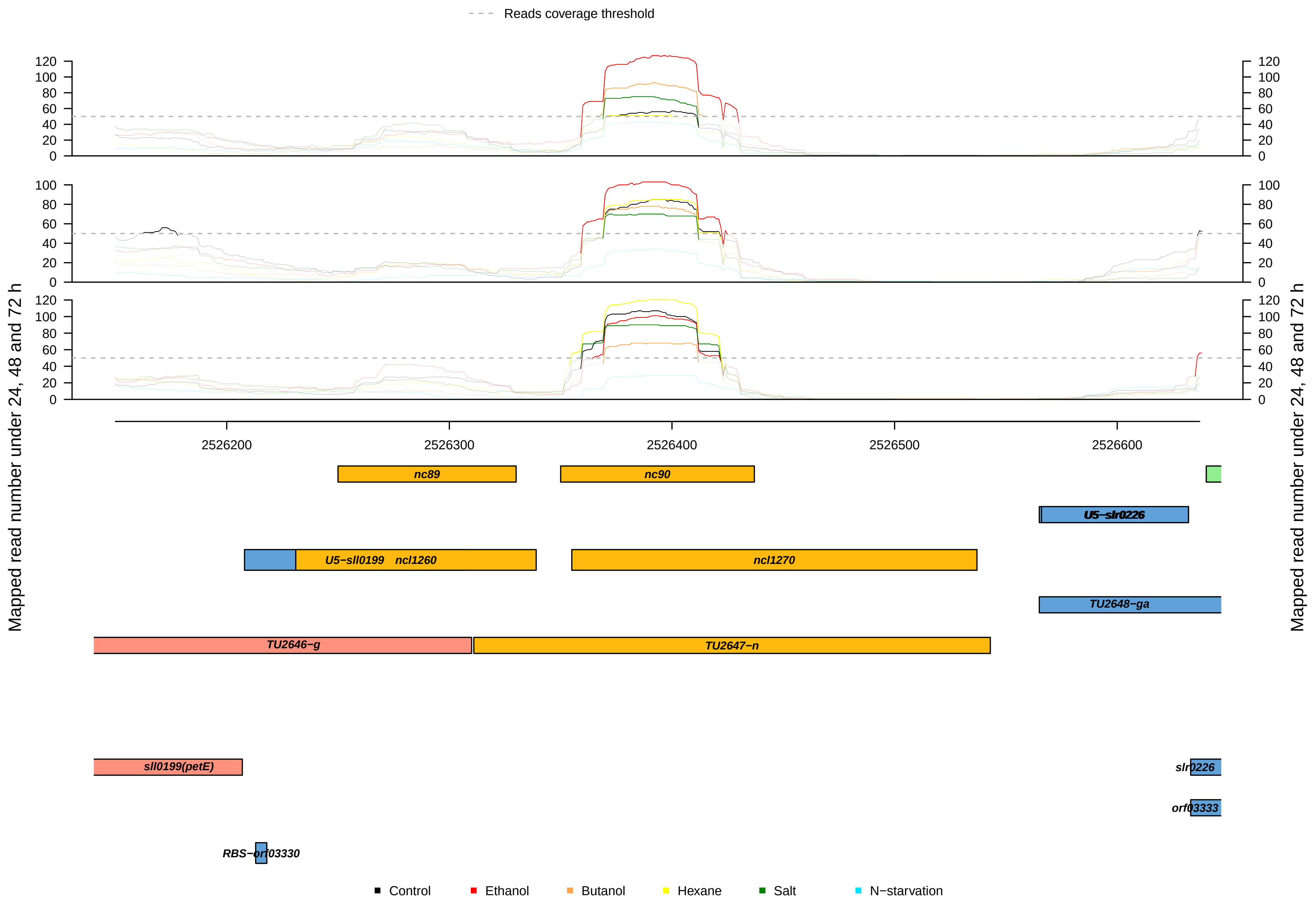

Reads coverage threshold
Mapped read number under 24, 48 and 72 h
2526200
2526300
2526400
2526500
2526600
nc89
nc90
U5−slr0226
U5−sll0199
ncl1260
ncl1270
TU2648−ga
TU2646−g
TU2647−n
sll0199(petE)
slr0226
orf03333
RBS−orf03330
Control
Ethanol
Butanol
Hexane
Salt
N−starvation

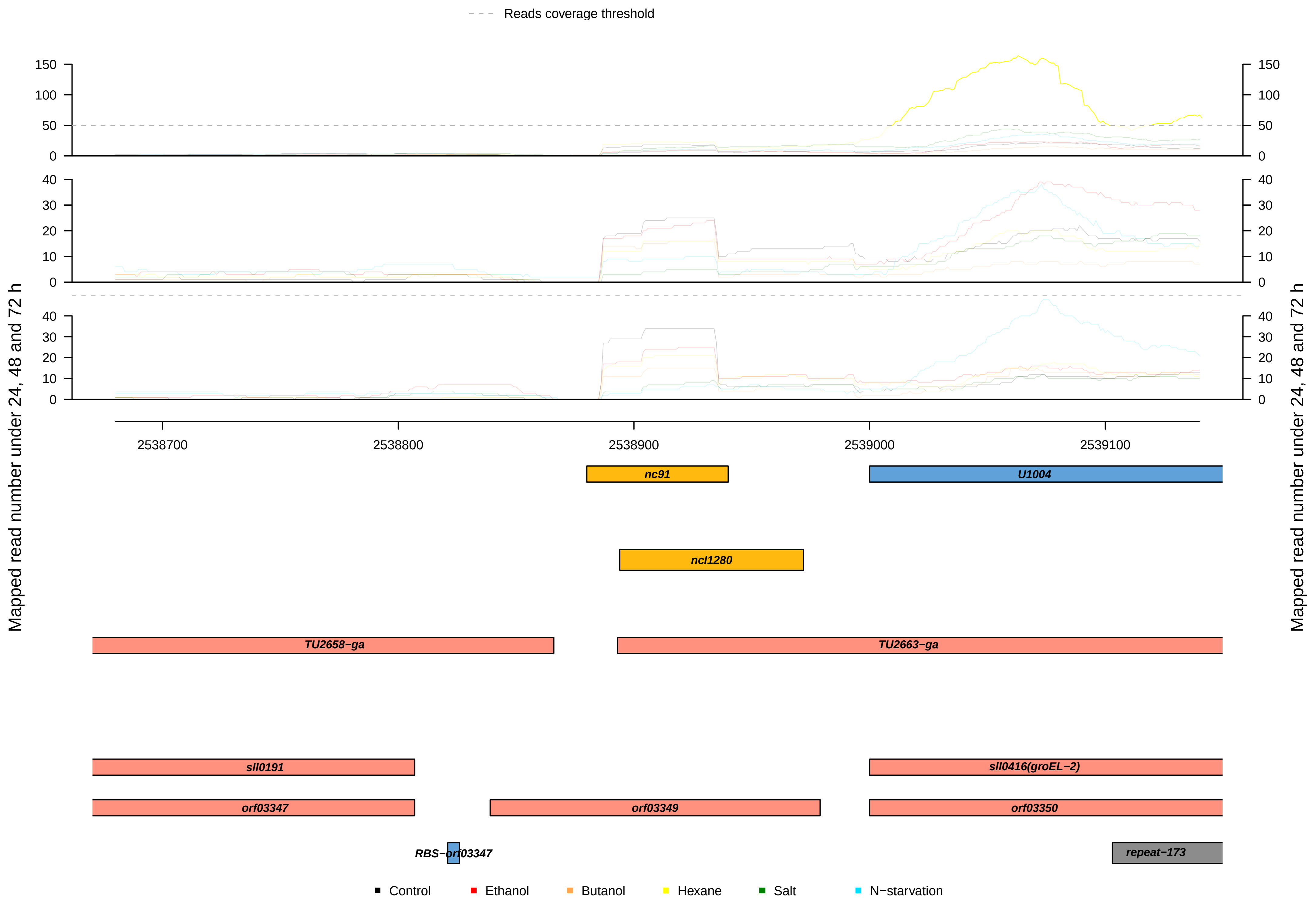

Reads coverage threshold
150
100
50
0
40
30
20
10
0
Mapped read number under 24, 48 and 72 h
2538700
2538800
2538900
2539000
2539100
nc91
U1004
ncl1280
TU2658−ga
TU2663−ga
sll0191
sll0416(groEL−2)
orf03347
orf03349
orf03350
RBS−orf03347
repeat−173
Control
Ethanol
Butanol
Hexane
Salt
N−starvation

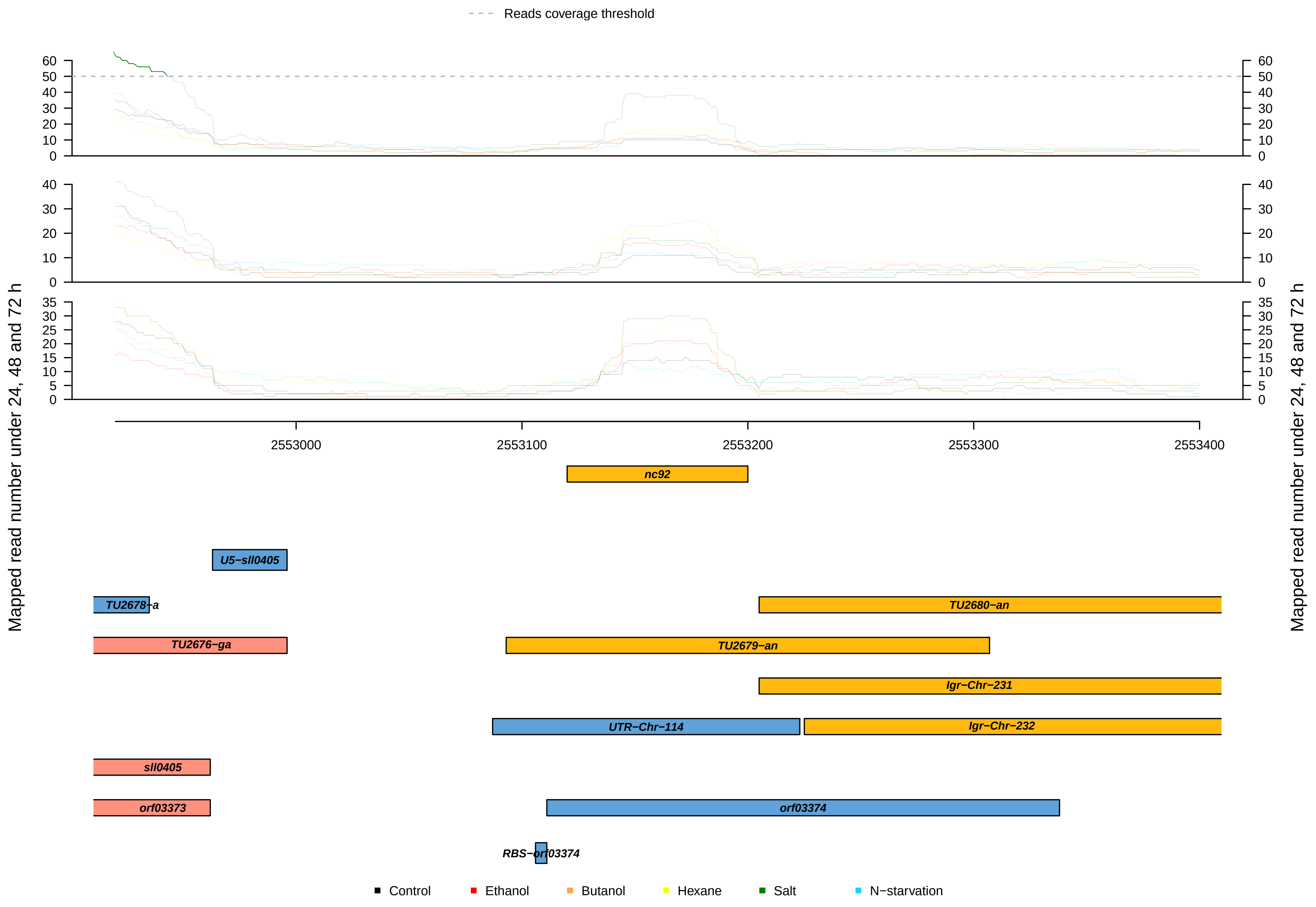

Reads coverage threshold
Mapped read number under 24, 48 and 72 h
2553000
2553100
2553200
2553300
2553400
nc92
U5−sll0405
TU2678−a
TU2680−an
TU2676−ga
TU2679−an
Igr−Chr−231
UTR−Chr−114
Igr−Chr−232
sll0405
orf03373
orf03374
RBS−orf03374
Control
Ethanol
Butanol
Hexane
Salt
N−starvation

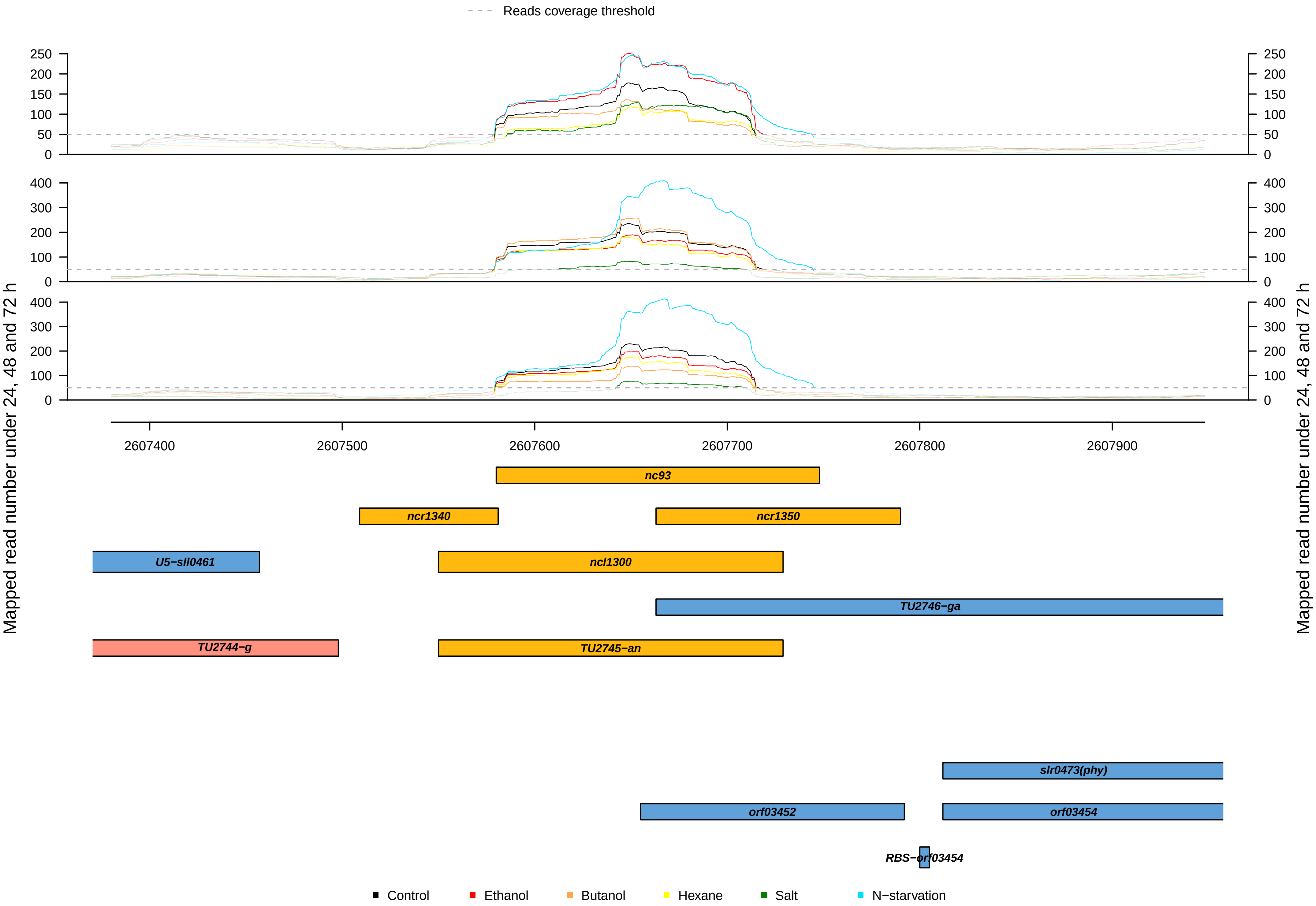

Reads coverage threshold
Mapped read number under 24, 48 and 72 h
2607400
2607500
2607600
2607700
2607800
2607900
nc93
ncr1340
ncr1350
U5−sll0461
ncl1300
TU2746−ga
TU2744−g
TU2745−an
slr0473(phy)
orf03452
orf03454
RBS−orf03454
Control
Ethanol
Butanol
Hexane
Salt
N−starvation

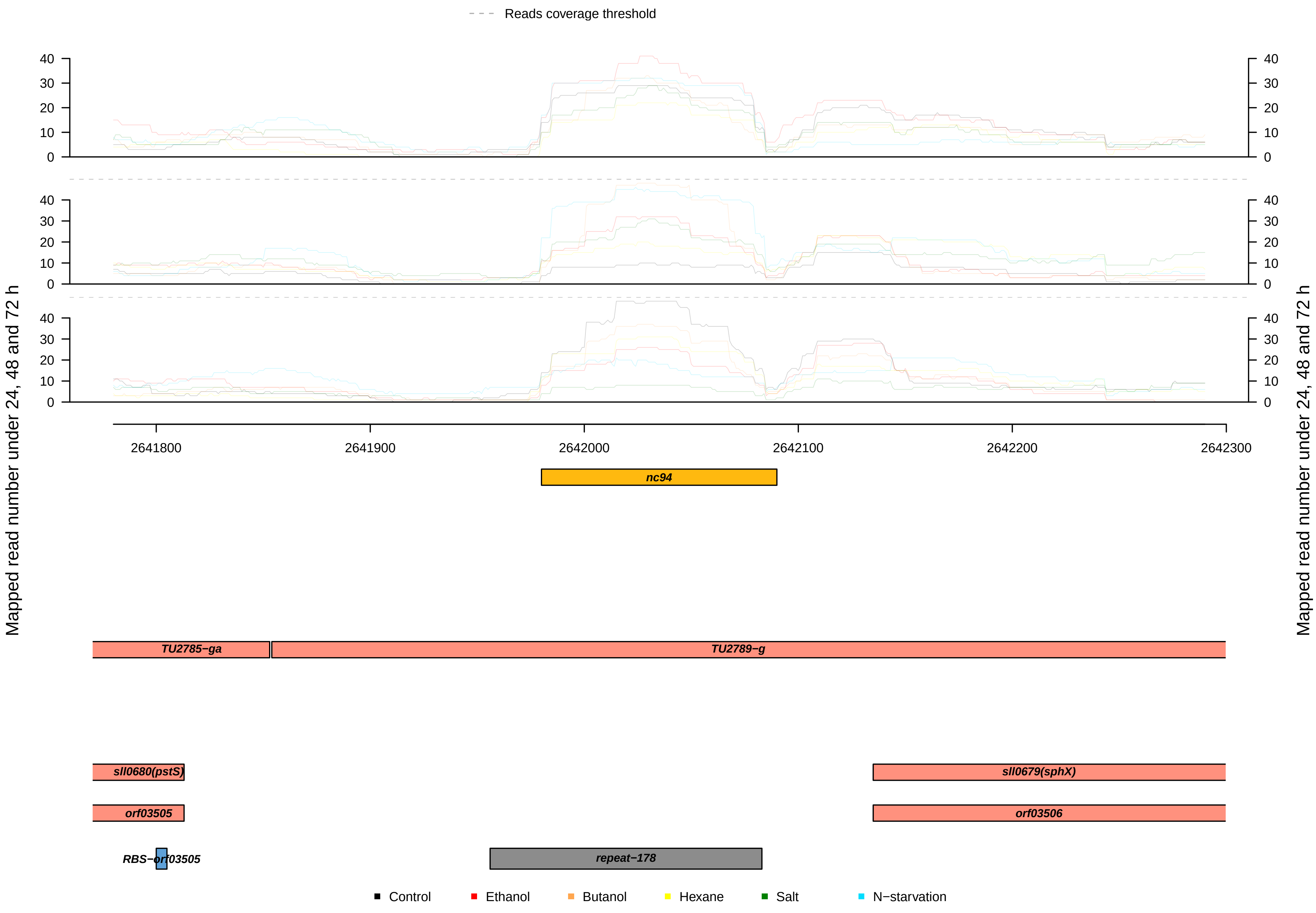

Reads coverage threshold
Mapped read number under 24, 48 and 72 h
2641800
2641900
2642000
2642100
2642200
2642300
nc94
TU2785−ga
TU2789−g
sll0680(pstS)
sll0679(sphX)
orf03505
orf03506
RBS−orf03505
repeat−178
Control
Ethanol
Butanol
Hexane
Salt
N−starvation

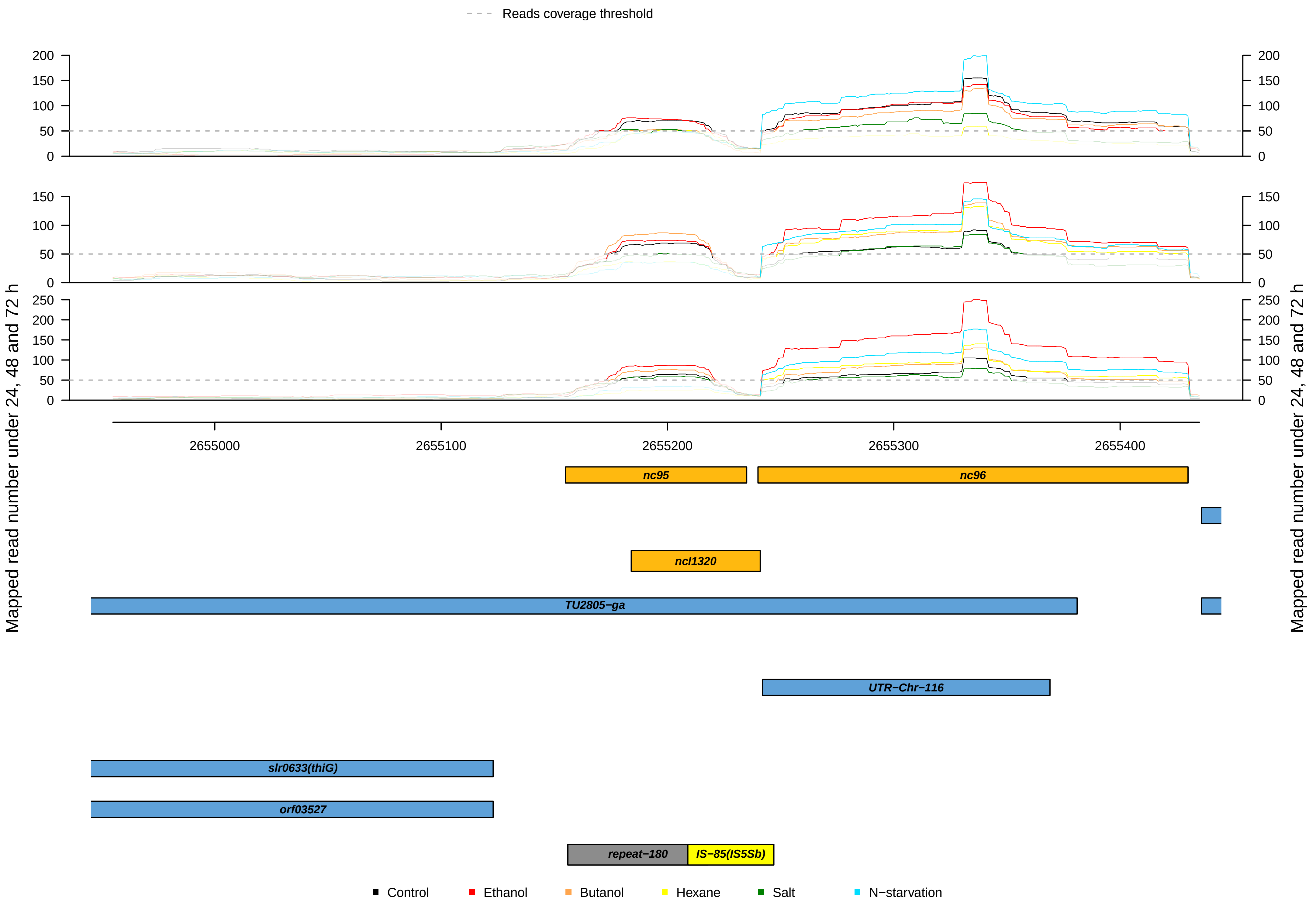

Reads coverage threshold
200
150
100
50
0
250
Mapped read number under 24, 48 and 72 h
2655000
2655100
2655200
2655300
2655400
nc95
nc96
ncl1320
TU2805−ga
UTR−Chr−116
slr0633(thiG)
orf03527
repeat−180
IS−85(IS5Sb)
Control
Ethanol
Butanol
Hexane
Salt
N−starvation

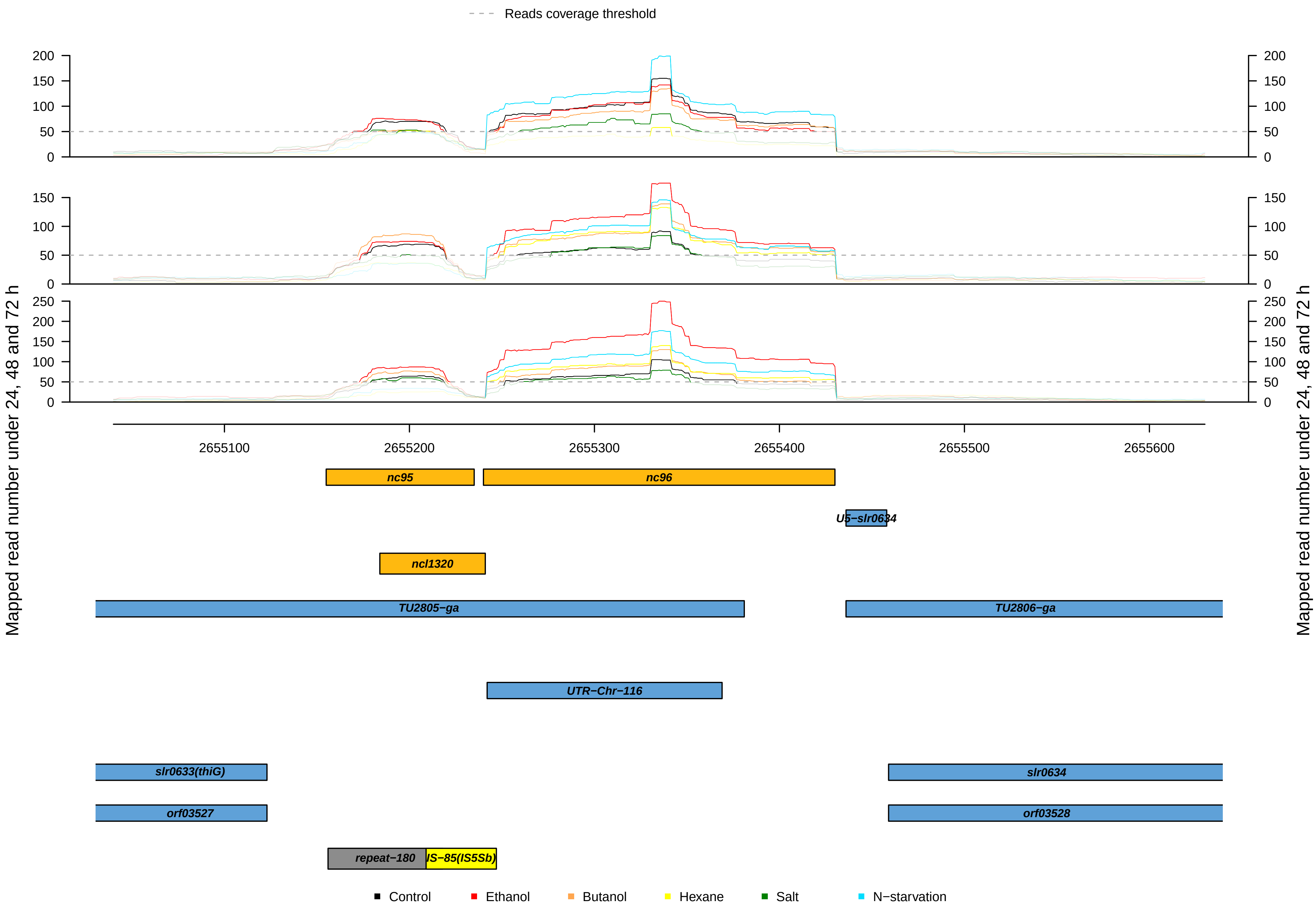

Reads coverage threshold
Mapped read number under 24, 48 and 72 h
200
150
100
50
0
250
2655100
2655200
2655300
2655400
2655500
2655600
nc95
nc96
U5−slr0634
ncl1320
TU2805−ga
TU2806−ga
UTR−Chr−116
slr0633(thiG)
slr0634
orf03527
orf03528
repeat−180
IS−85(IS5Sb)
Control
Ethanol
Butanol
Hexane
Salt
N−starvation

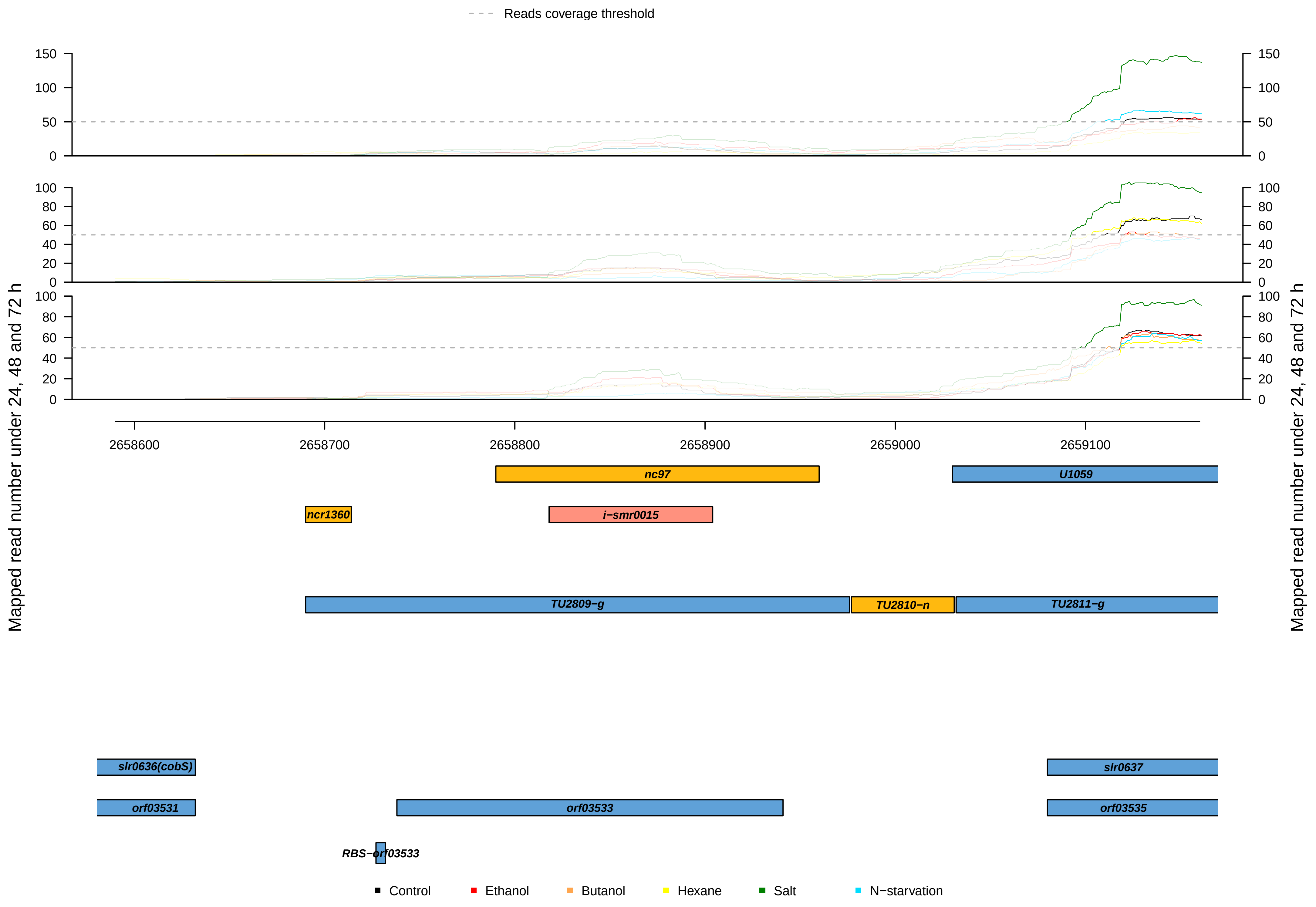

Reads coverage threshold
Mapped read number under 24, 48 and 72 h
150
100
50
0
100
80
60
40
20
0
2658600
2658700
2658800
2658900
2659000
2659100
nc97
U1059
ncr1360
i−smr0015
TU2809−g
TU2810−n
TU2811−g
slr0636(cobS)
slr0637
orf03531
orf03533
orf03535
RBS−orf03533
Control
Ethanol
Butanol
Hexane
Salt
N−starvation

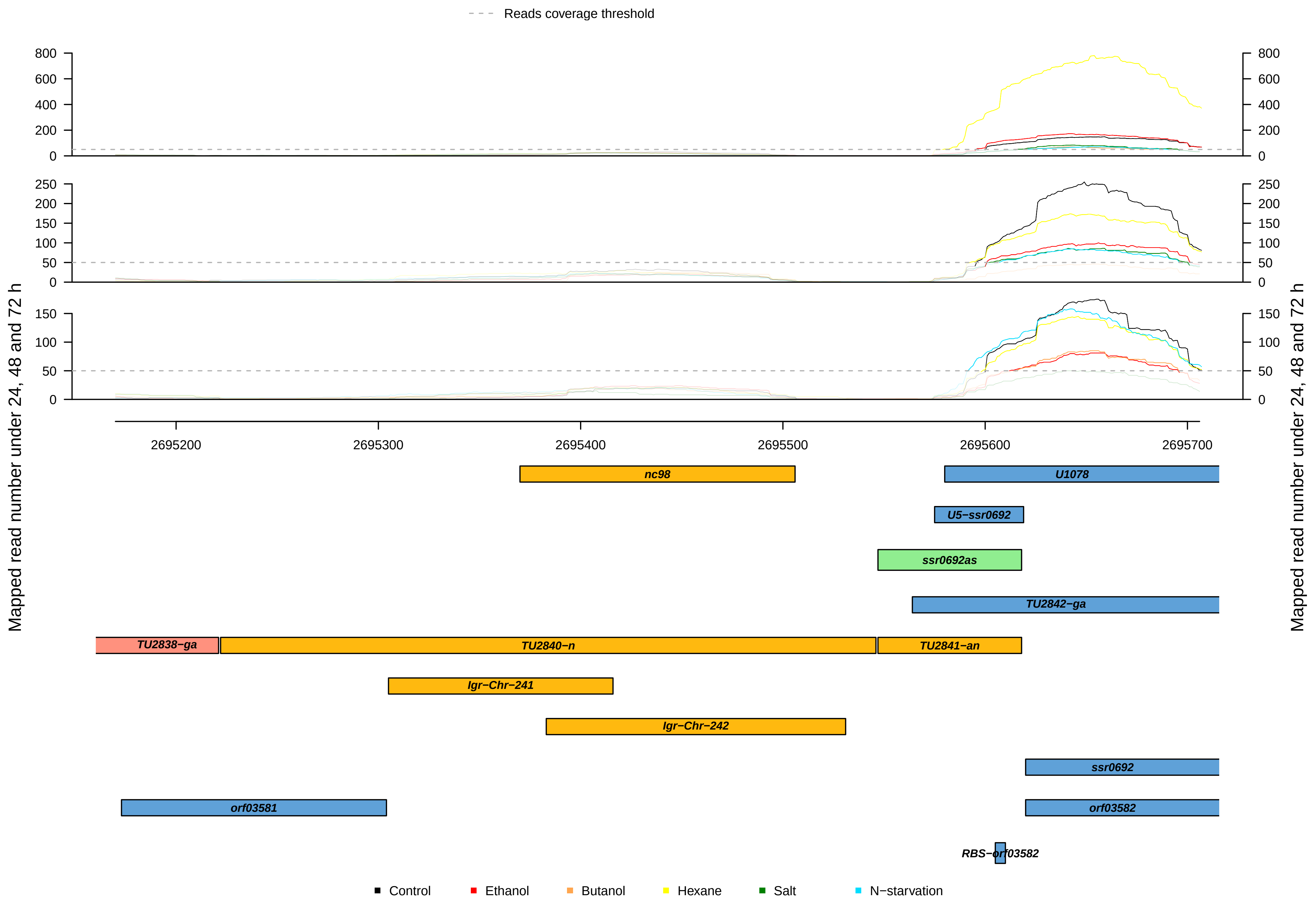

Reads coverage threshold
Mapped read number under 24, 48 and 72 h
800
600
400
200
0
250
150
100
50
2695200
2695300
2695400
2695500
2695600
2695700
nc98
U1078
U5−ssr0692
ssr0692as
TU2842−ga
TU2838−ga
TU2840−n
TU2841−an
Igr−Chr−241
Igr−Chr−242
ssr0692
orf03581
orf03582
RBS−orf03582
Control
Ethanol
Butanol
Hexane
Salt
N−starvation

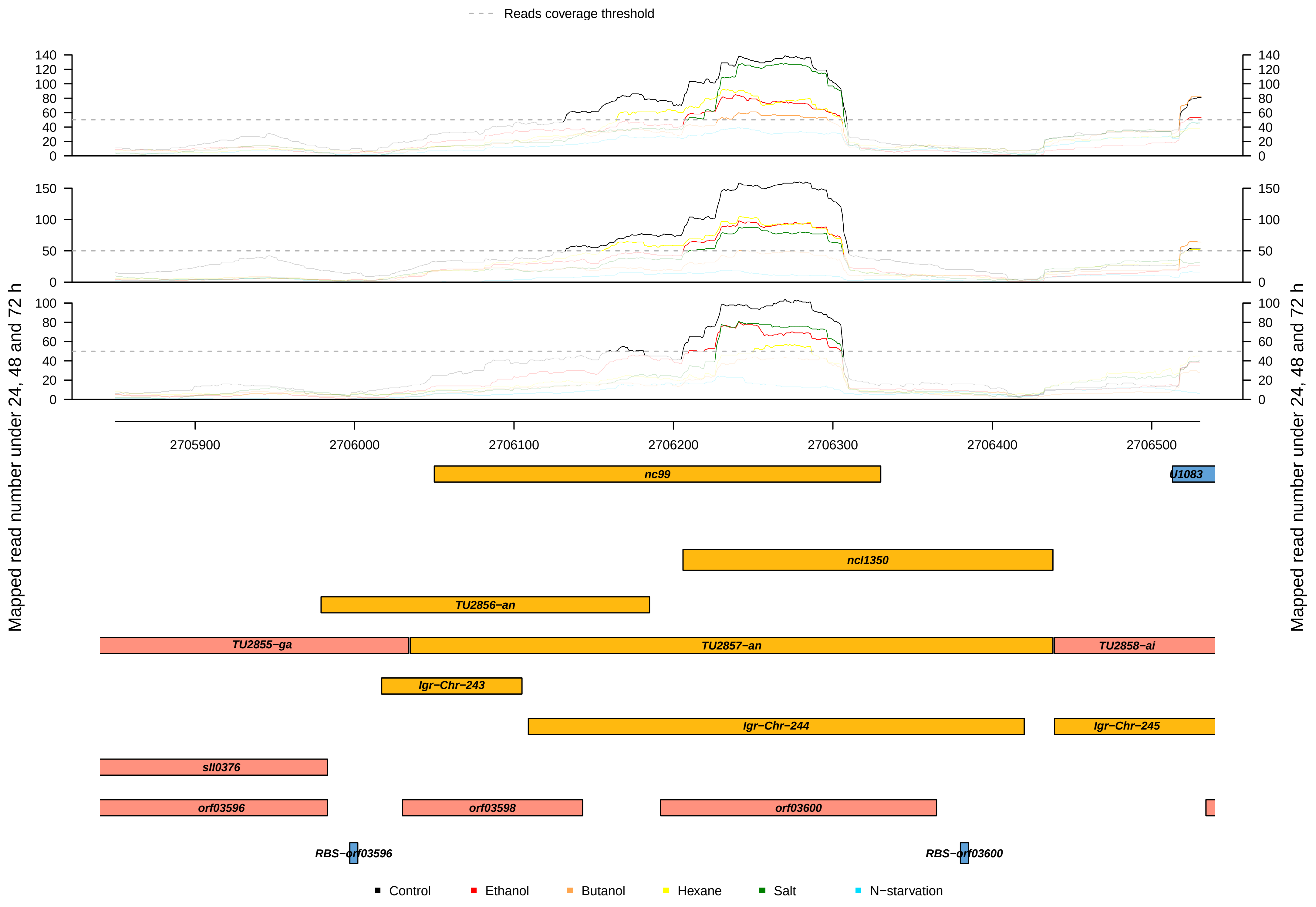

Reads coverage threshold
Mapped read number under 24, 48 and 72 h
2705900
2706000
2706100
2706200
2706300
2706400
2706500
nc99
U1083
ncl1350
TU2856−an
TU2855−ga
TU2857−an
TU2858−ai
Igr−Chr−243
Igr−Chr−244
Igr−Chr−245
sll0376
orf03596
orf03598
orf03600
RBS−orf03596
RBS−orf03600
Control
Ethanol
Butanol
Hexane
Salt
N−starvation

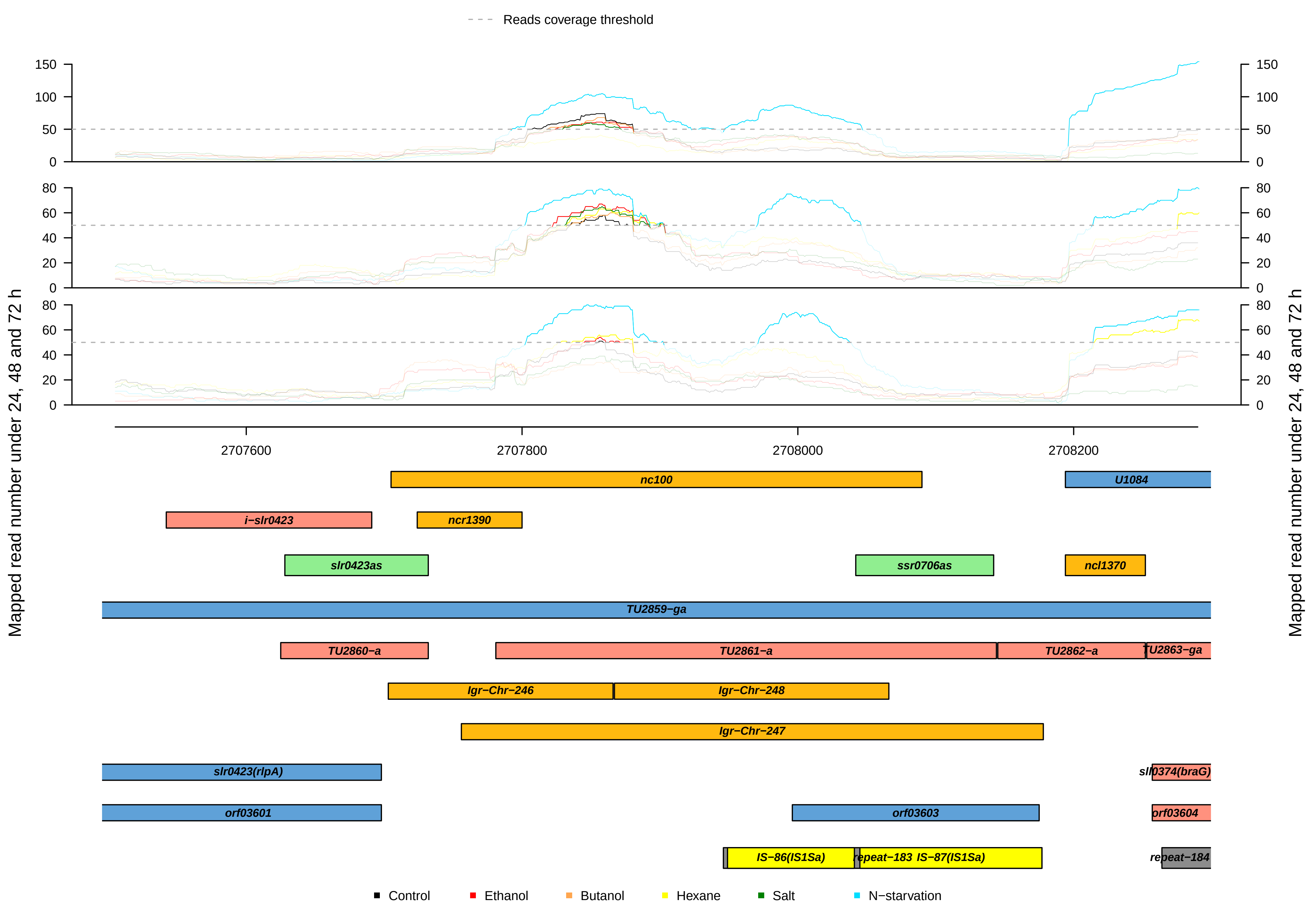

Reads coverage threshold
150
100
50
0
80
60
40
20
0
Mapped read number under 24, 48 and 72 h
2707600
2707800
2708000
2708200
nc100
U1084
i−slr0423
ncr1390
slr0423as
ssr0706as
ncl1370
TU2859−ga
TU2860−a
TU2861−a
TU2862−a
TU2863−ga
Igr−Chr−246
Igr−Chr−248
Igr−Chr−247
slr0423(rlpA)
sll0374(braG)
orf03601
orf03603
orf03604
IS−86(IS1Sa)
repeat−183
IS−87(IS1Sa)
repeat−184
Control
Ethanol
Butanol
Hexane
Salt
N−starvation

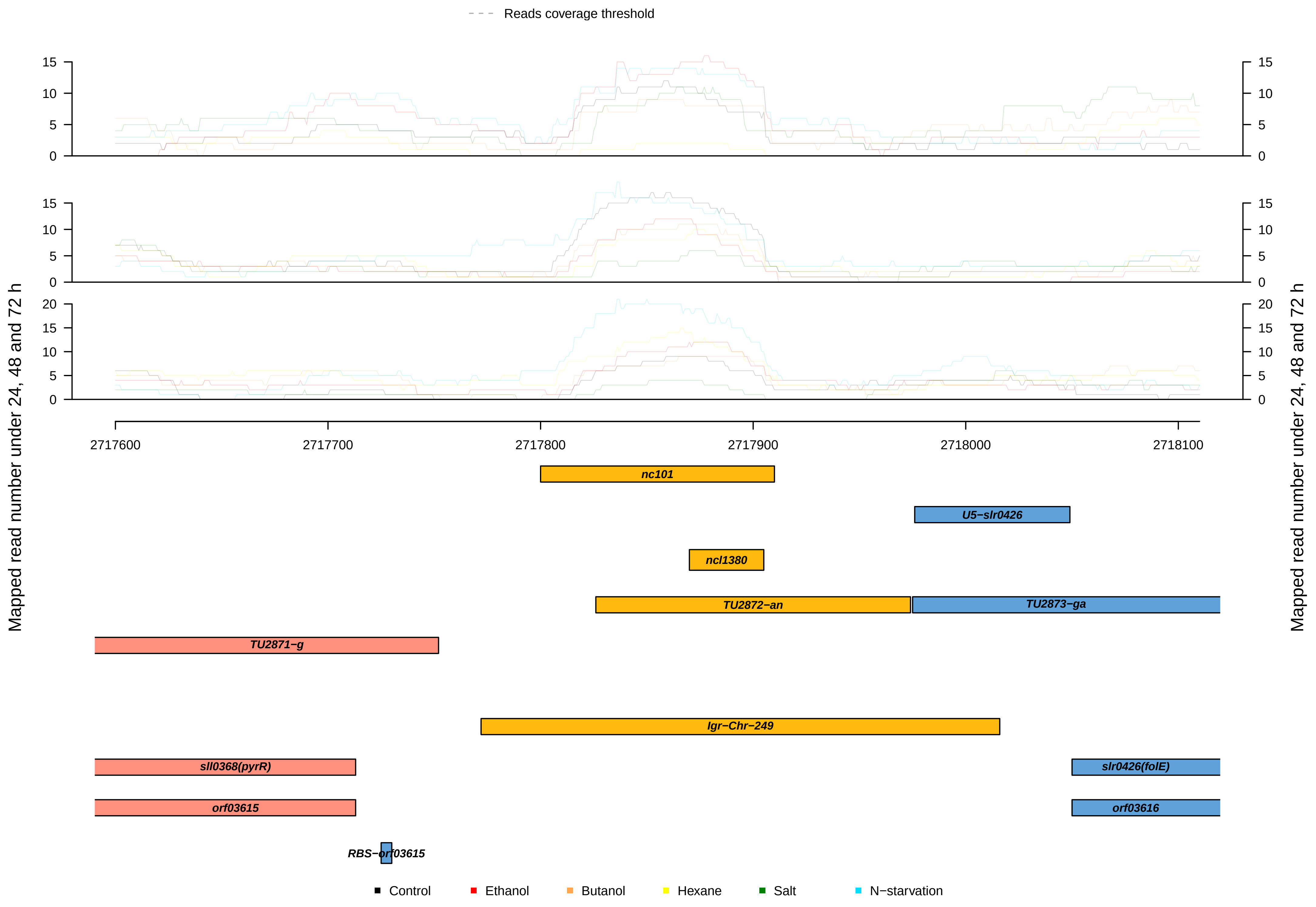

Reads coverage threshold
Mapped read number under 24, 48 and 72 h
2717600
2717700
2717800
2717900
2718000
2718100
nc101
U5−slr0426
ncl1380
TU2872−an
TU2873−ga
TU2871−g
Igr−Chr−249
sll0368(pyrR)
slr0426(folE)
orf03615
orf03616
RBS−orf03615
Control
Ethanol
Butanol
Hexane
Salt
N−starvation

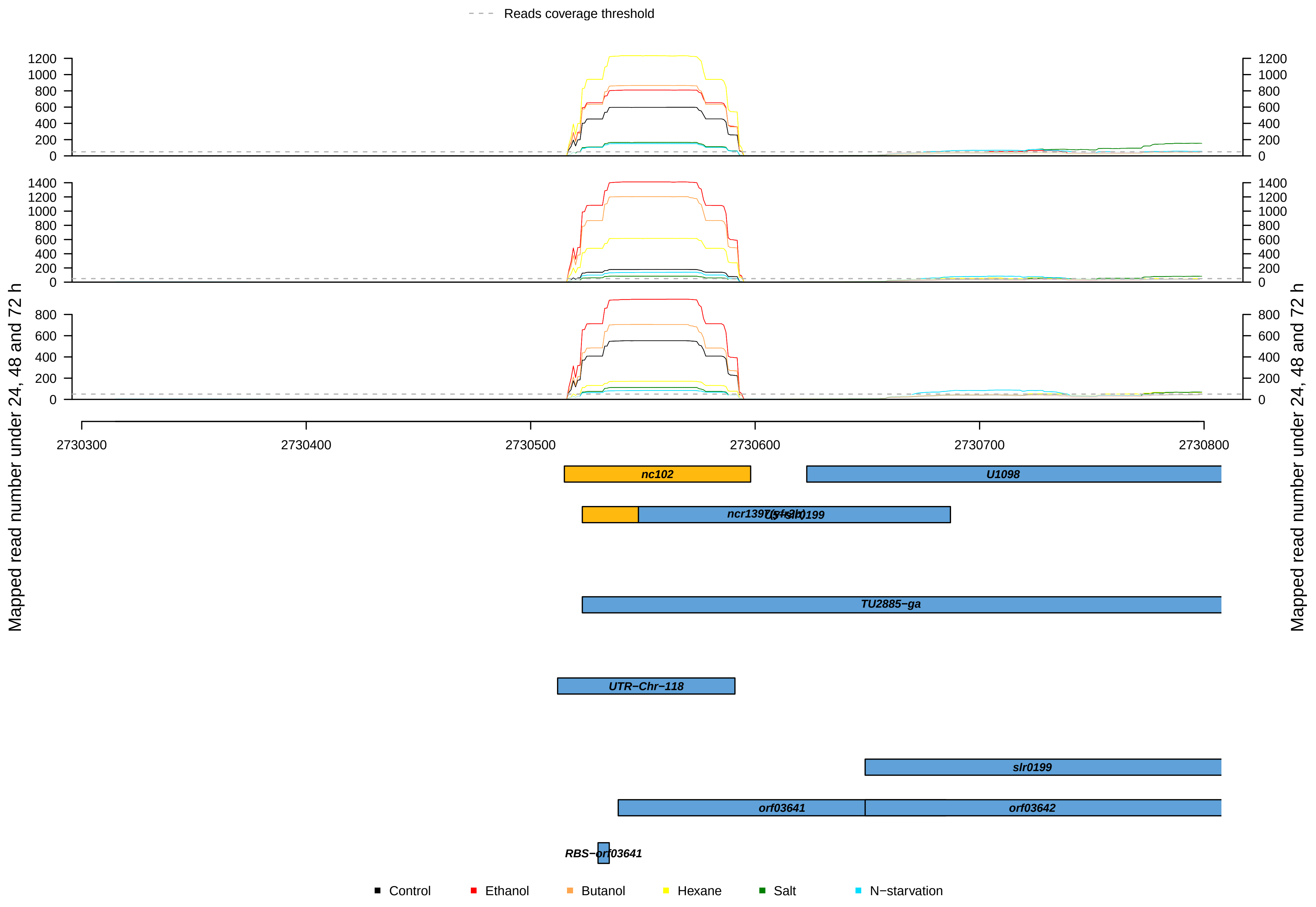

Reads coverage threshold
Mapped read number under 24, 48 and 72 h
2730300
2730400
2730500
2730600
2730700
2730800
nc102
U1098
ncr1397(sfs2h)
U3-slr0199
TU2885-ga
UTR-Chr-118
slr0199
orf03641
orf03642
RBS-orf03641
Control
Ethanol
Butanol
Hexane
Salt
N-starvation

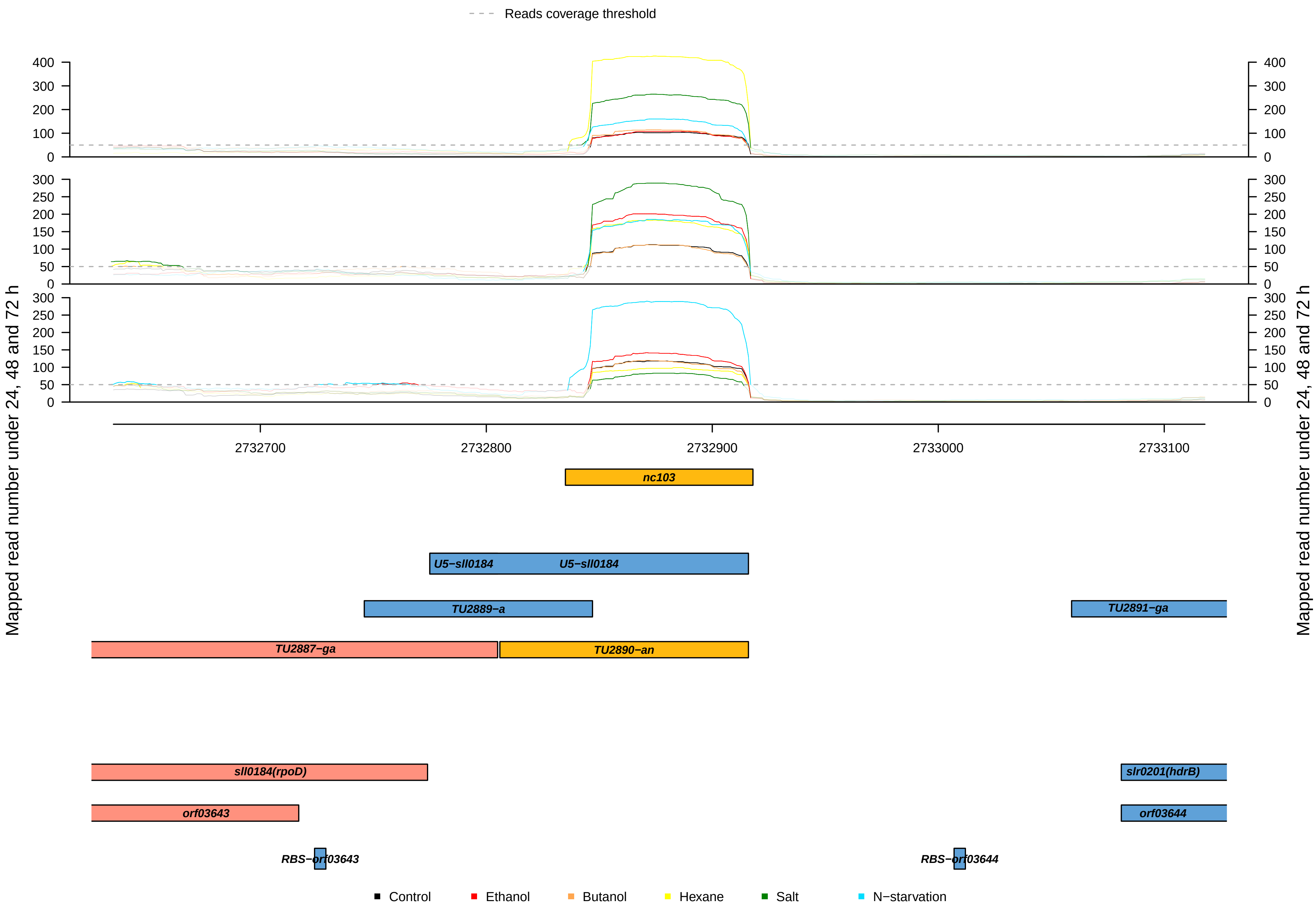

Reads coverage threshold
Mapped read number under 24, 48 and 72 h
400
300
200
100
0
250
150
50
2732700
2732800
2732900
2733000
2733100
nc103
U5−sll0184
U5−sll0184
TU2889−a
TU2891−ga
TU2887−ga
TU2890−an
sll0184(rpoD)
slr0201(hdrB)
orf03643
orf03644
RBS−orf03643
RBS−orf03644
Control
Ethanol
Butanol
Hexane
Salt
N−starvation

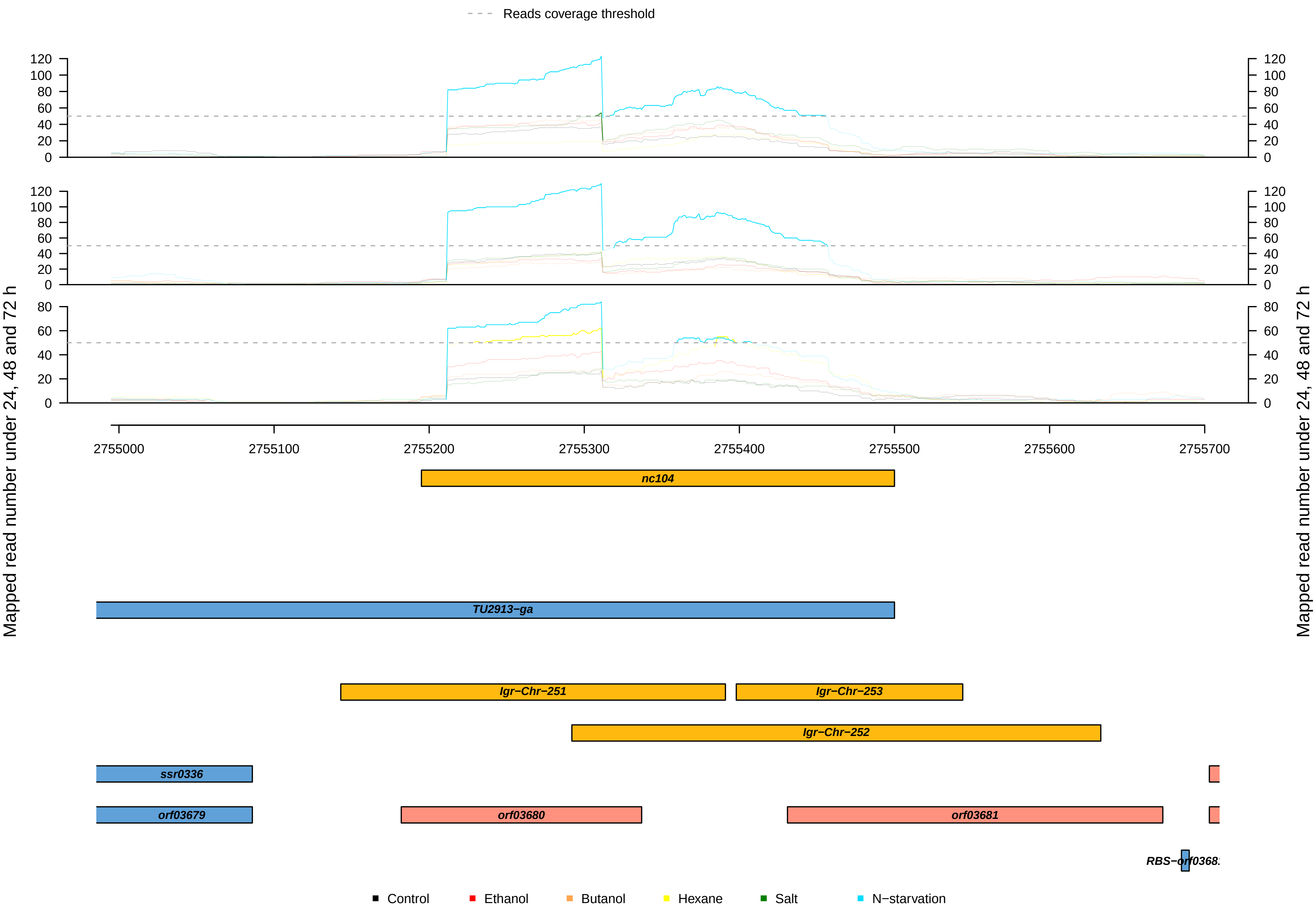

Reads coverage threshold
Mapped read number under 24, 48 and 72 h
2755000
2755100
2755200
2755300
2755400
2755500
2755600
2755700
nc104
TU2913-ga
Igr-Chr-251
Igr-Chr-253
Igr-Chr-252
ssr0336
orf03679
orf03680
orf03681
Control
Ethanol
Butanol
Hexane
Salt
N-starvation

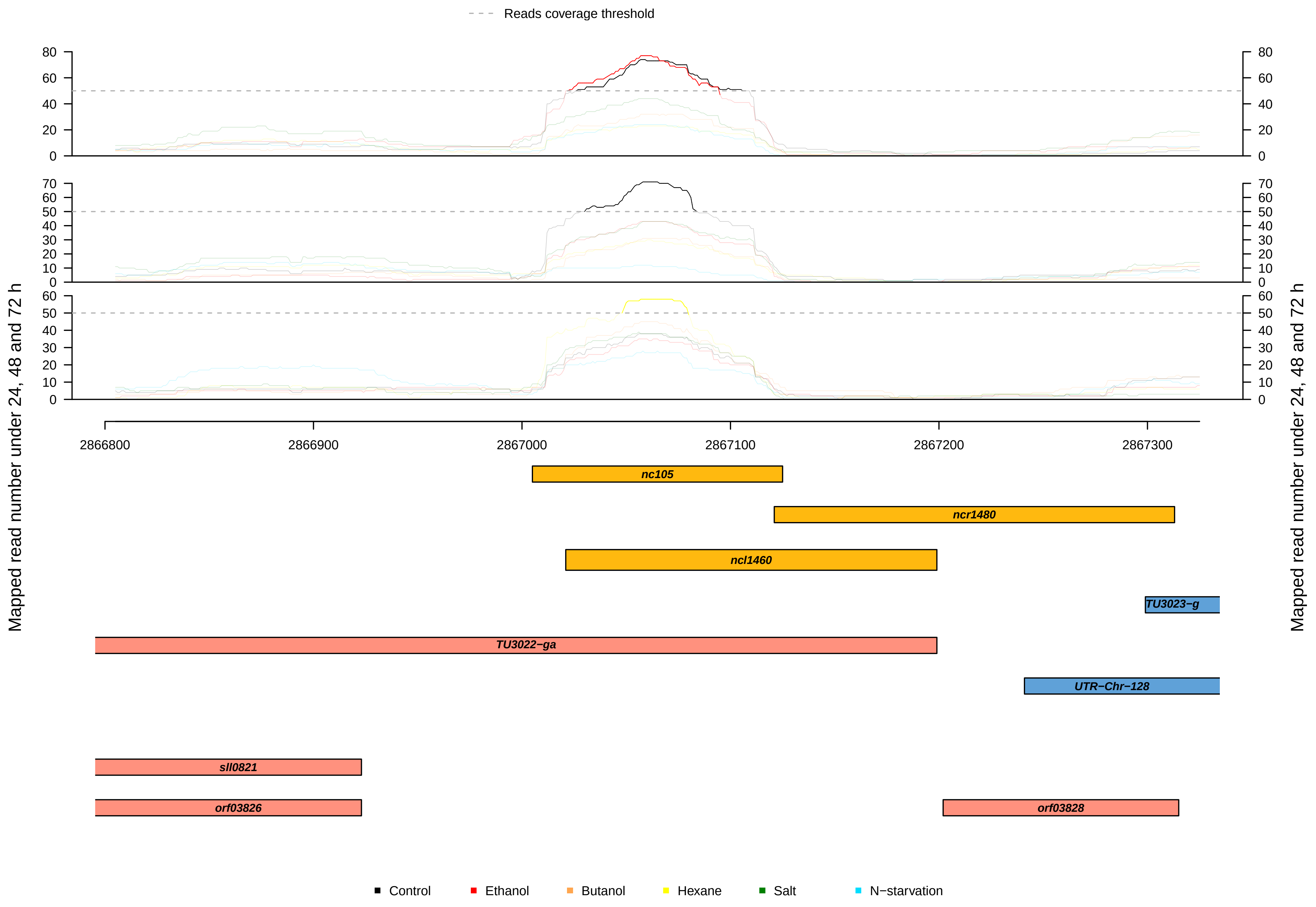

Reads coverage threshold
Mapped read number under 24, 48 and 72 h
2866800
2866900
2867000
2867100
2867200
2867300
nc105
ncr1480
ncl1460
TU3023−g
TU3022−ga
UTR−Chr−128
sll0821
orf03826
orf03828
Control
Ethanol
Butanol
Hexane
Salt
N−starvation

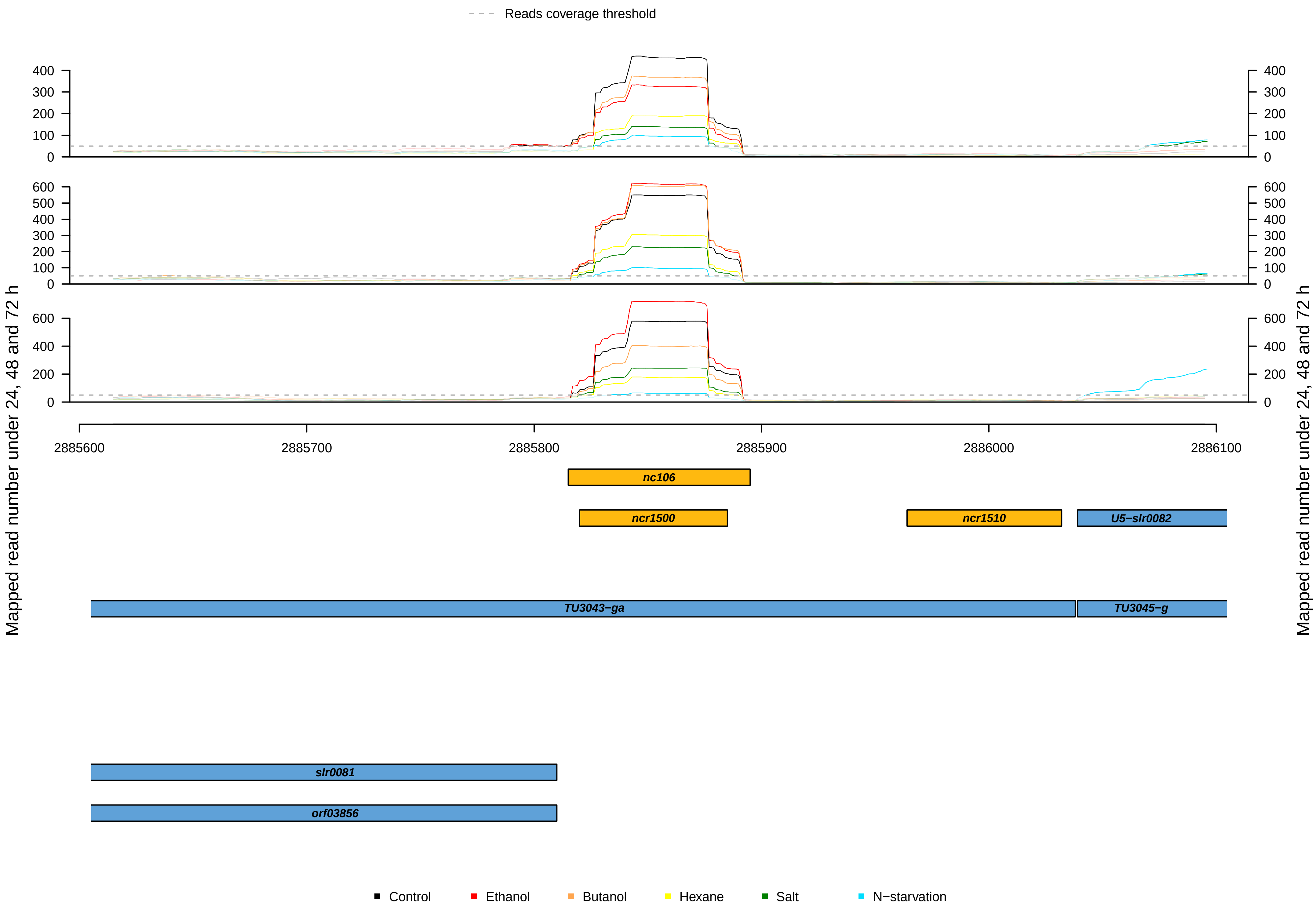

Reads coverage threshold
400
300
200
100
0
600
500
400
300
200
100
0
600
400
200
0
Mapped read number under 24, 48 and 72 h
2885600
2885700
2885800
2885900
2886000
2886100
nc106
ncr1500
ncr1510
U5−slr0082
TU3043−ga
TU3045−g
slr0081
orf03856
Control
Ethanol
Butanol
Hexane
Salt
N−starvation

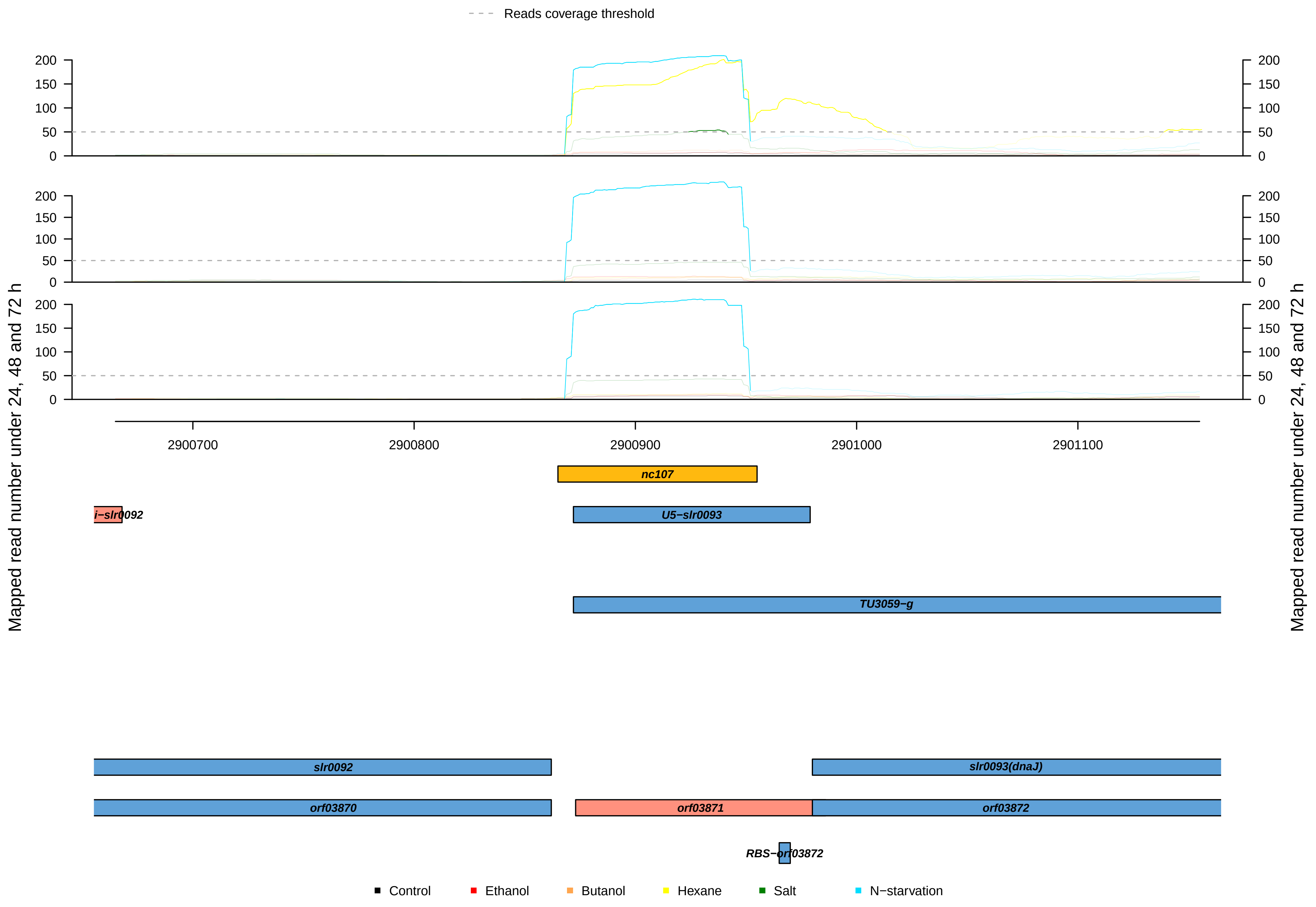

Reads coverage threshold
200
150
100
50
0
Mapped read number under 24, 48 and 72 h
2900700
2900800
2900900
2901000
2901100
nc107
i−slr0092
U5−slr0093
TU3059−g
slr0092
slr0093(dnaJ)
orf03870
orf03871
orf03872
RBS−orf03872
Control
Ethanol
Butanol
Hexane
Salt
N−starvation

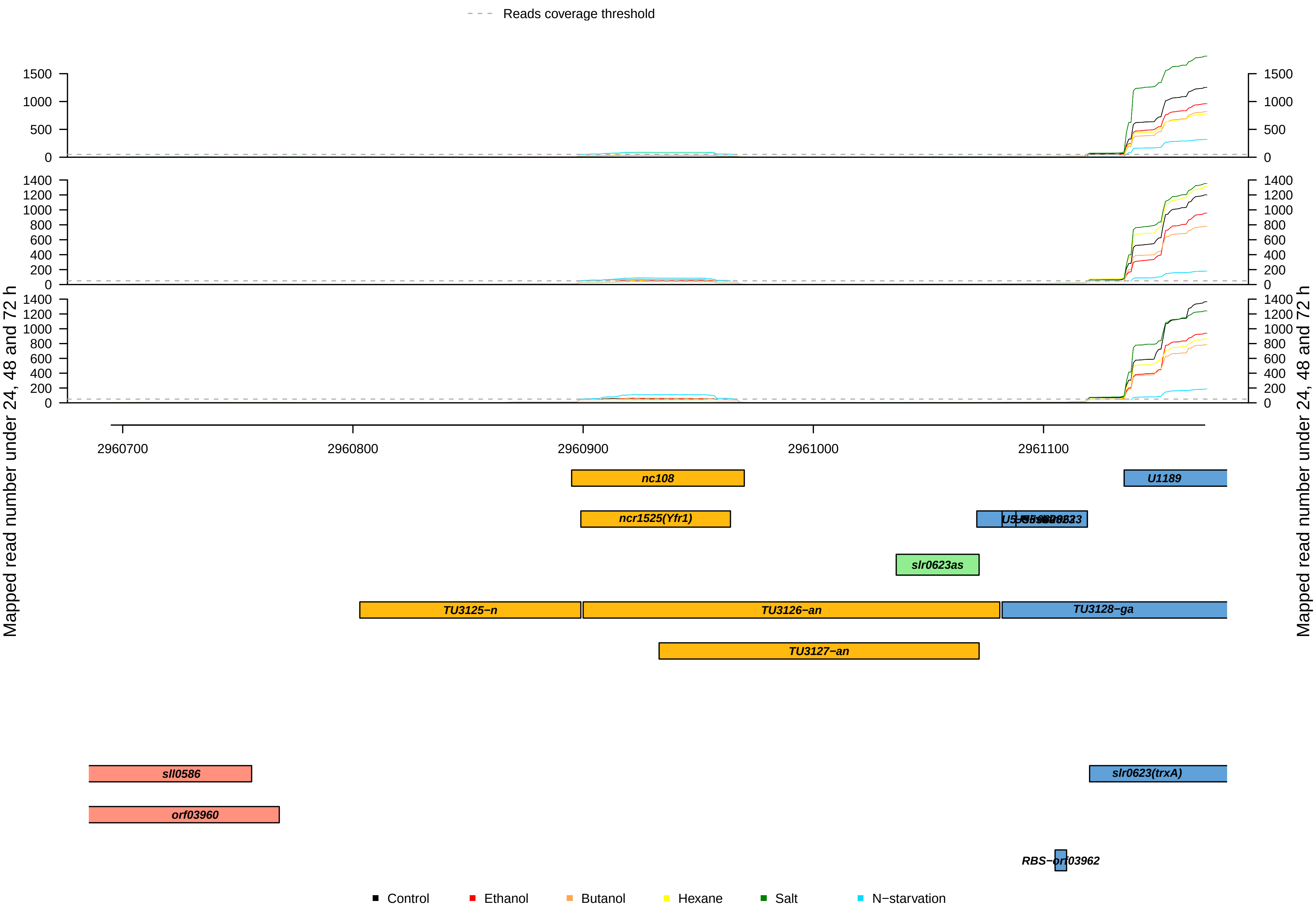
Reads coverage threshold
Mapped read number under 24, 48 and 72 h
1500
1000
500
0
1400
1200
1000
800
600
400
200
0
2960700
2960800
2960900
2961000
2961100
nc108
U1189
ncr1525(Yfr1)
slr0623as
TU3125−n
TU3126−an
TU3128−ga
TU3127−an
sll0586
slr0623(trxA)
orf03960
RBS−orf03962
Control
Ethanol
Butanol
Hexane
Salt
N−starvation

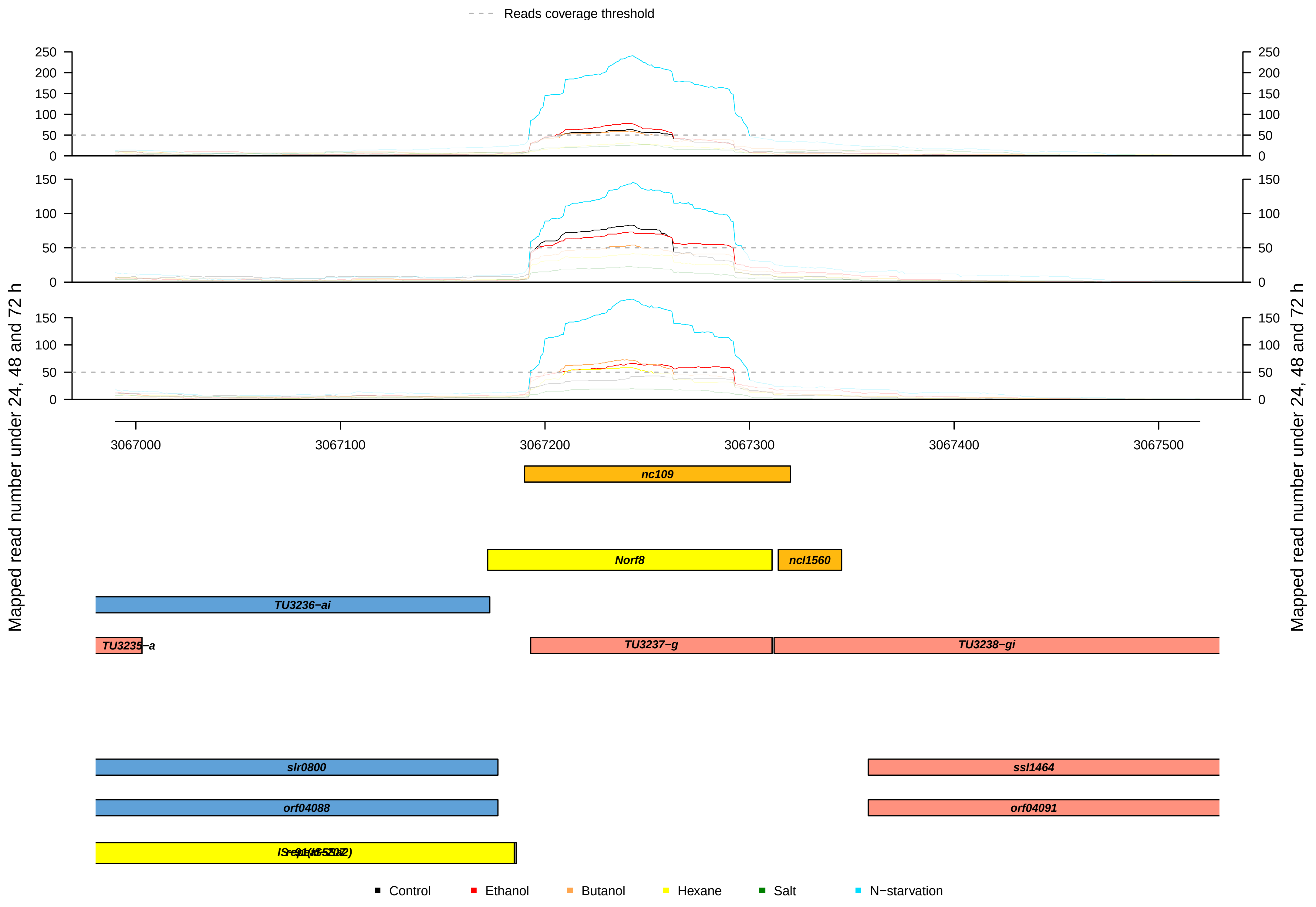

Reads coverage threshold
250
200
150
100
50
0
Mapped read number under 24, 48 and 72 h
3067000
3067100
3067200
3067300
3067400
3067500
nc109
Norf8
ncl1560
TU3236−ai
TU3235−a
TU3237−g
TU3238−gi
slr0800
ssl1464
orf04088
orf04091
Control
Ethanol
Butanol
Hexane
Salt
N−starvation

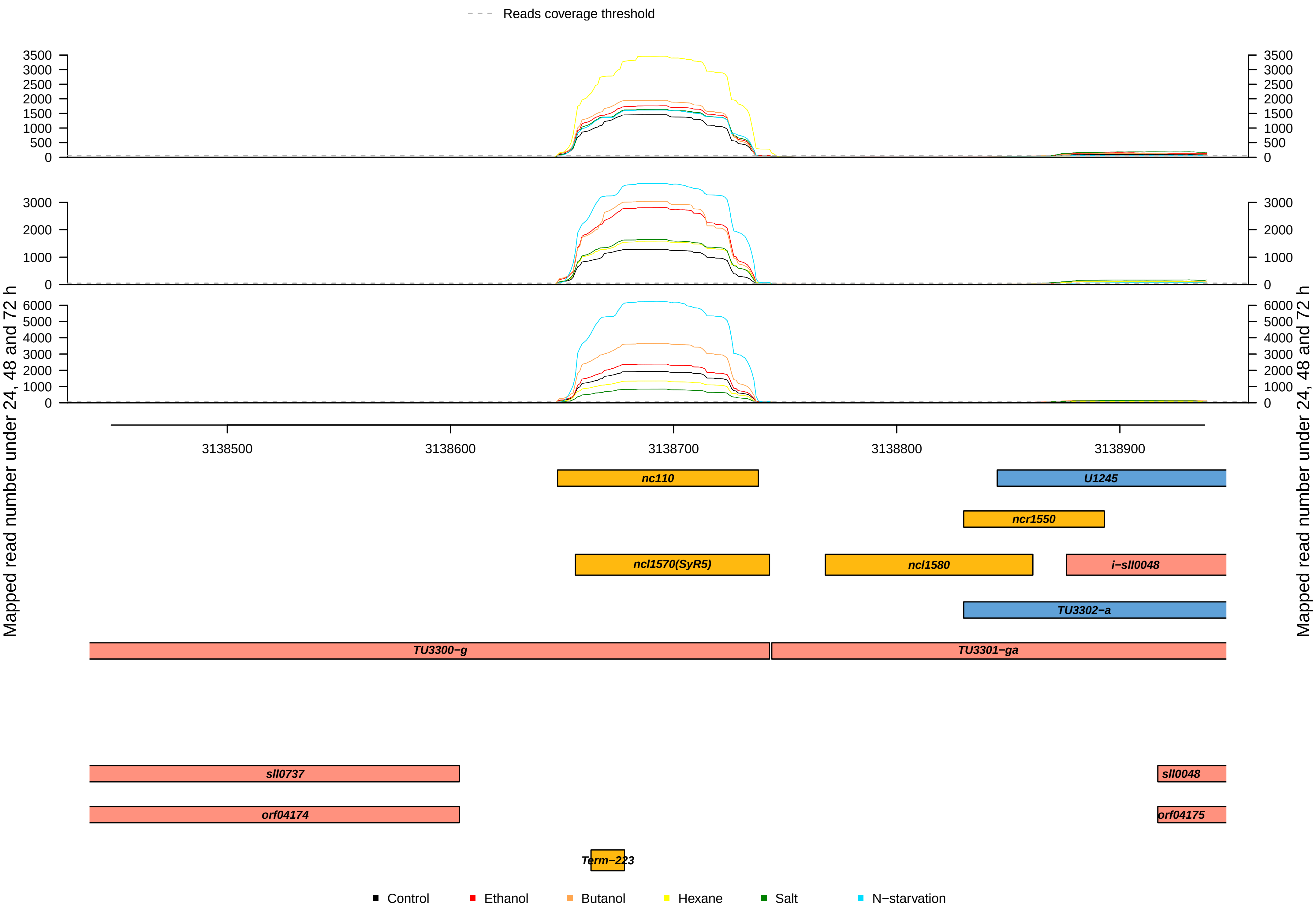

Reads coverage threshold
Mapped read number under 24, 48 and 72 h
3500
3000
2500
2000
1500
1000
500
0
6000
5000
4000
3138500
3138600
3138700
3138800
3138900
nc110
U1245
ncr1550
ncl1570(SyR5)
ncl1580
i−sll0048
TU3302−a
TU3300−g
TU3301−ga
sll0737
sll0048
orf04174
orf04175
Term−223
Control
Ethanol
Butanol
Hexane
Salt
N−starvation

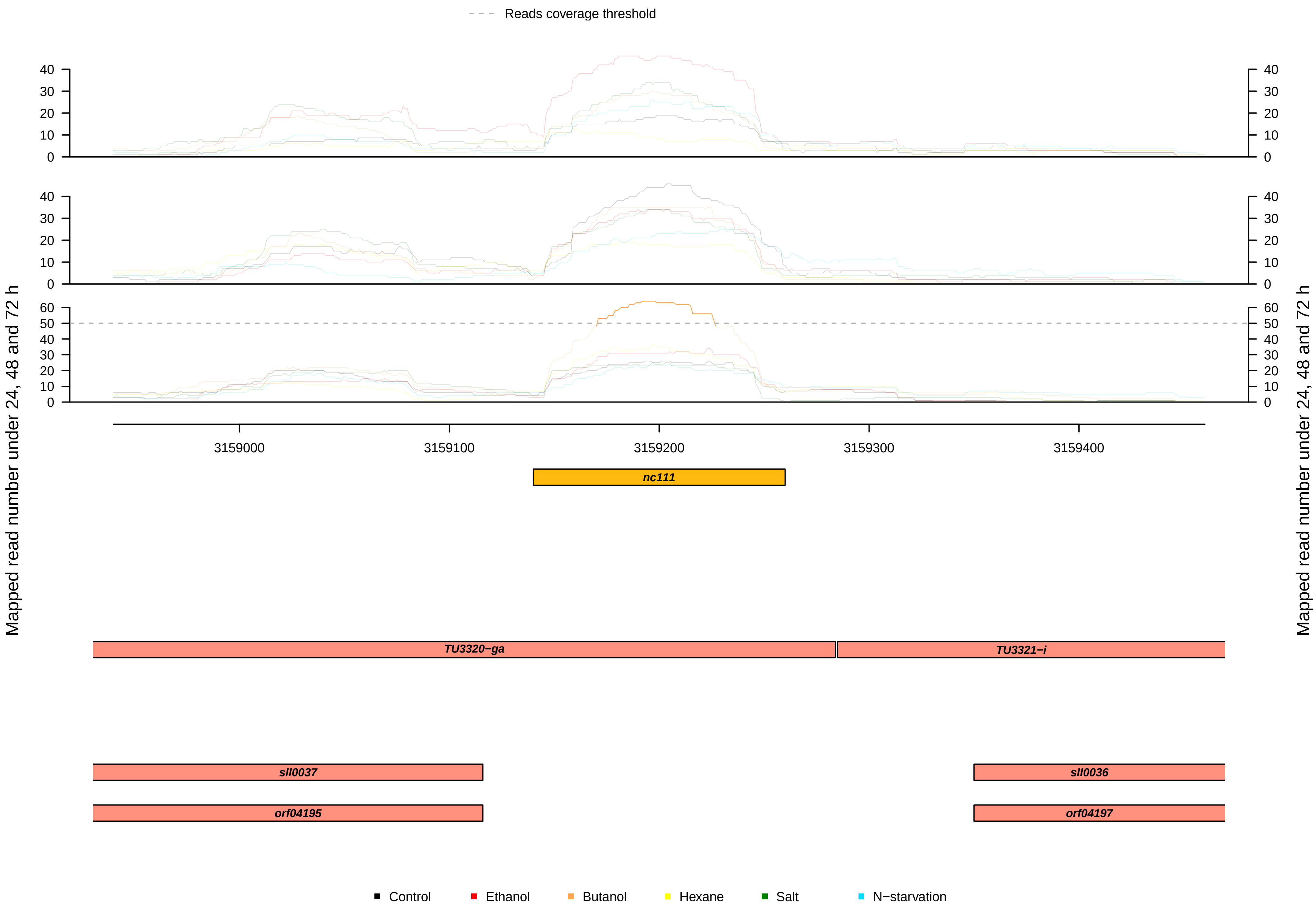

Reads coverage threshold
40
30
20
10
0
60
50
Mapped read number under 24, 48 and 72 h
3159000
3159100
3159200
3159300
3159400
nc111
TU3320−ga
TU3321−i
sll0037
sll0036
orf04195
orf04197
Control
Ethanol
Butanol
Hexane
Salt
N−starvation

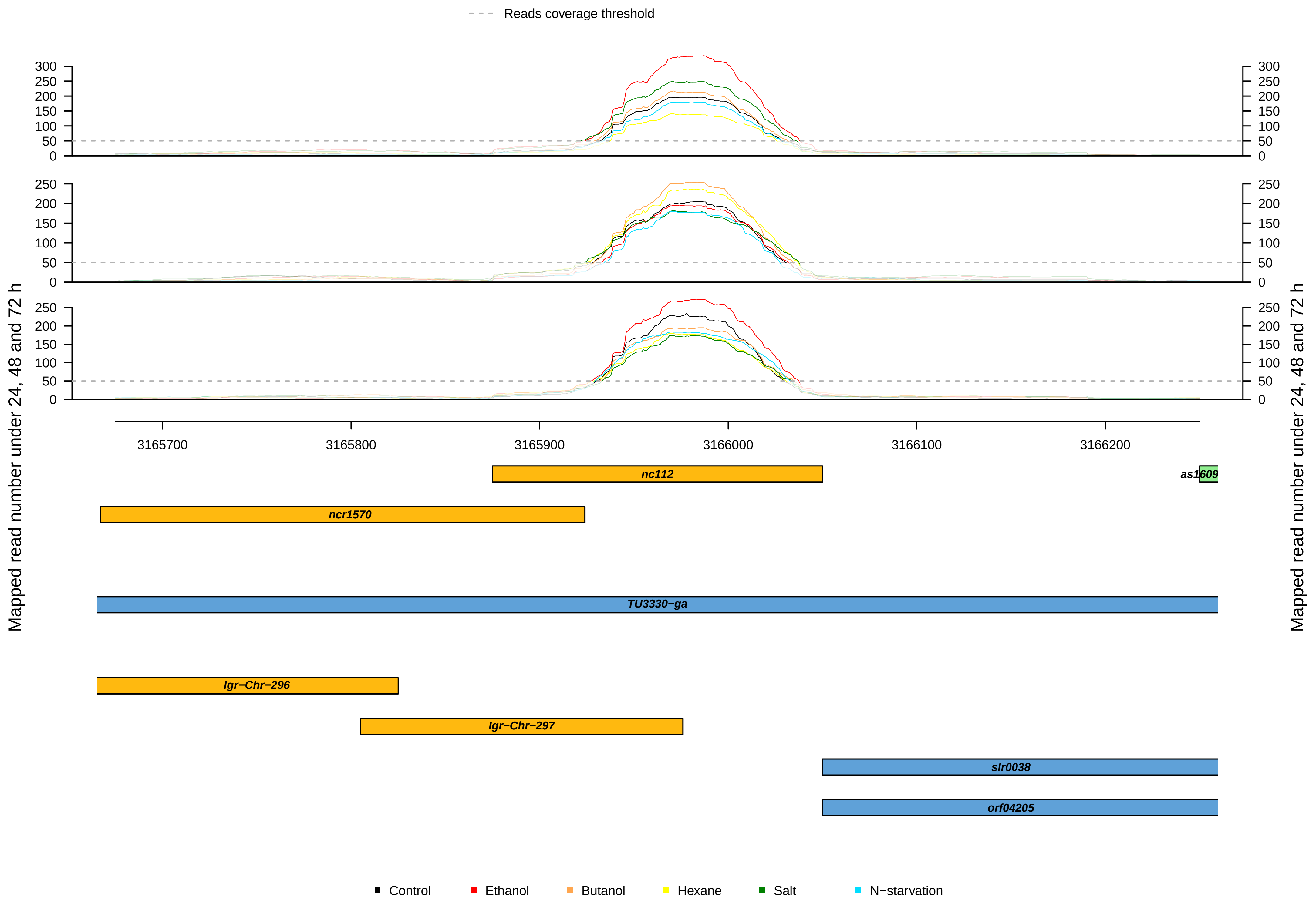
Reads coverage threshold
Mapped read number under 24, 48 and 72 h
300
250
200
150
100
50
0
3165700
3165800
3165900
3166000
3166100
3166200
nc112
as1609
ncr1570
TU3330−ga
Igr−Chr−296
Igr−Chr−297
slr0038
orf04205
Control
Ethanol
Butanol
Hexane
Salt
N−starvation

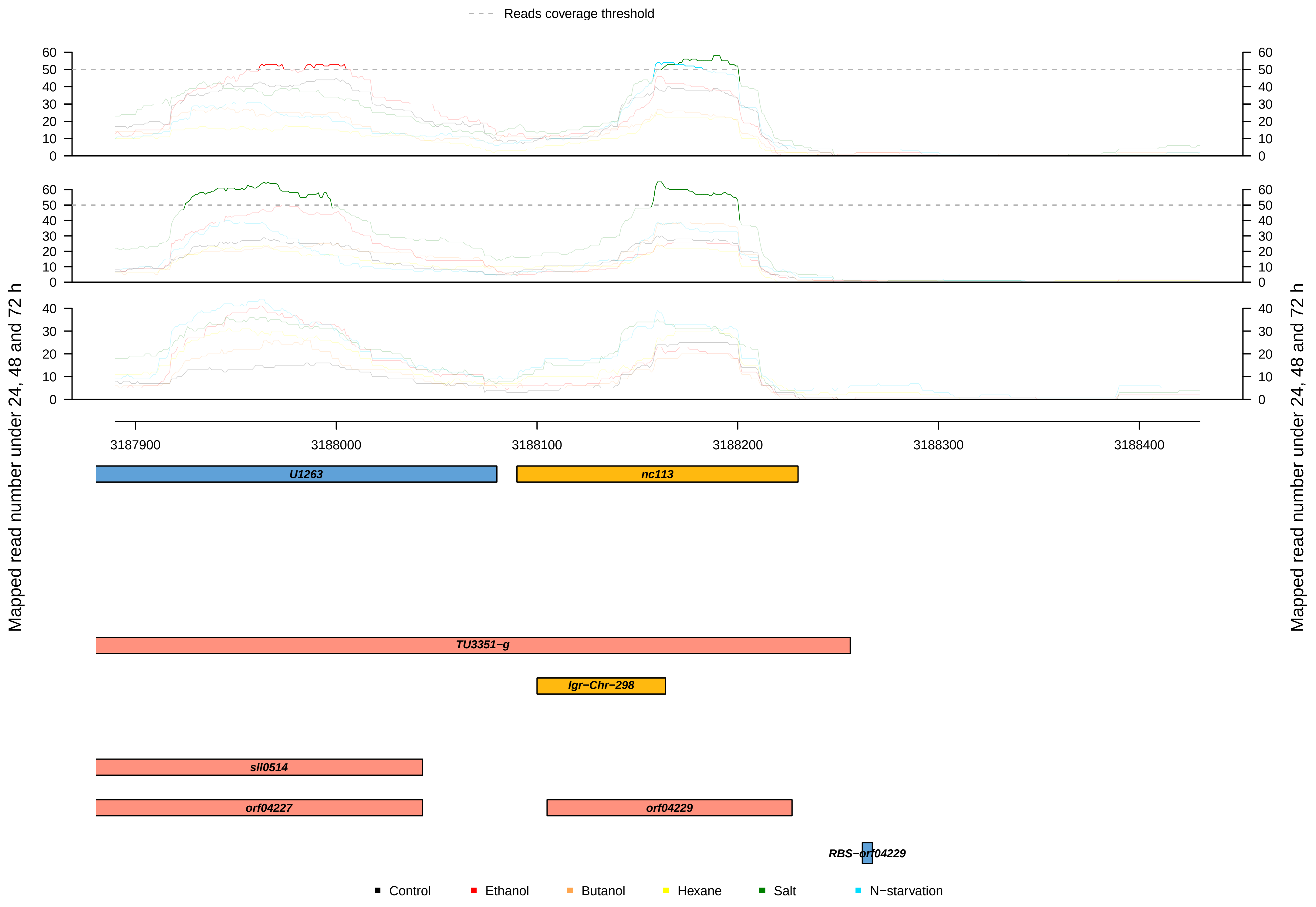

Reads coverage threshold
60
50
40
30
20
10
0
Mapped read number under 24, 48 and 72 h
3187900
3188000
3188100
3188200
3188300
3188400
U1263
nc113
TU3351−g
Igr−Chr−298
sll0514
orf04227
orf04229
RBS−orf04229
Control
Ethanol
Butanol
Hexane
Salt
N−starvation

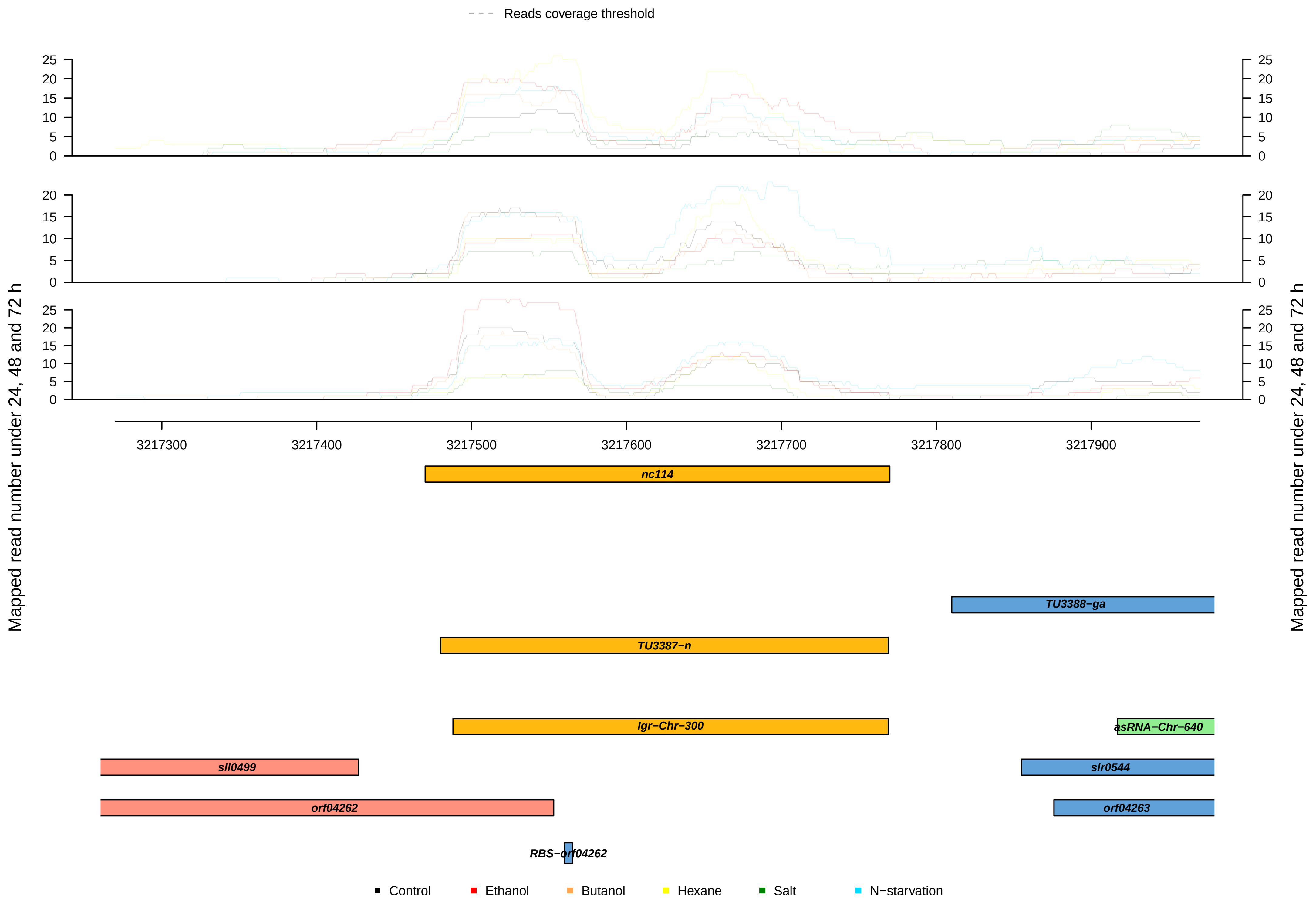

Reads coverage threshold
Mapped read number under 24, 48 and 72 h
3217300
3217400
3217500
3217600
3217700
3217800
3217900
nc114
TU3388−ga
TU3387−n
Igr−Chr−300
asRNA−Chr−640
sll0499
slr0544
orf04262
orf04263
RBS−orf04262
Control
Ethanol
Butanol
Hexane
Salt
N−starvation

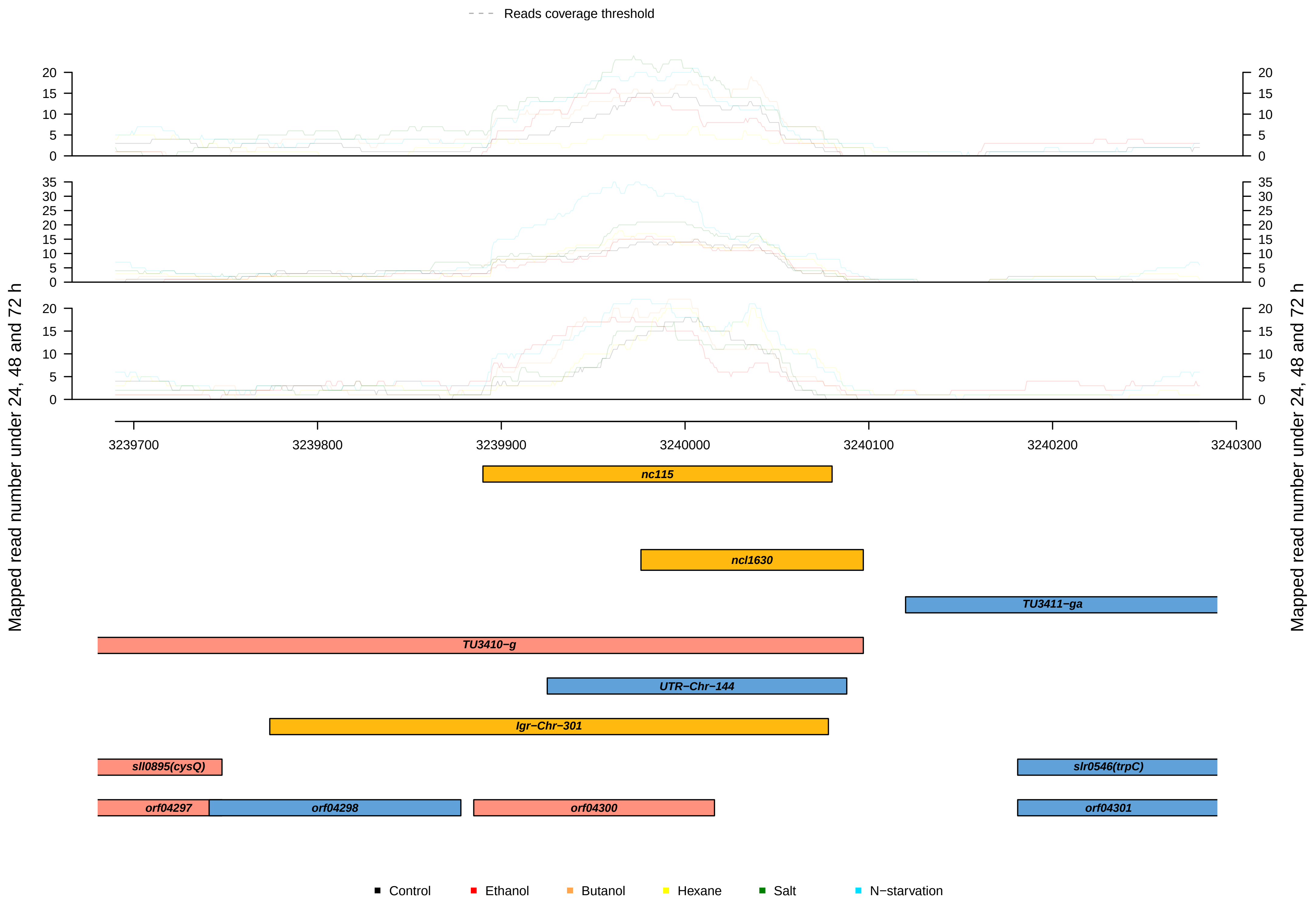

Reads coverage threshold
Mapped read number under 24, 48 and 72 h
20
15
10
5
0
35
30
25
20
15
10
5
0
20
15
10
5
0
3239700
3239800
3239900
3240000
3240100
3240200
3240300
nc115
ncl1630
TU3411−ga
TU3410−g
UTR−Chr−144
Igr−Chr−301
sll0895(cysQ)
slr0546(trpC)
orf04297
orf04298
orf04300
orf04301
Control
Ethanol
Butanol
Hexane
Salt
N−starvation

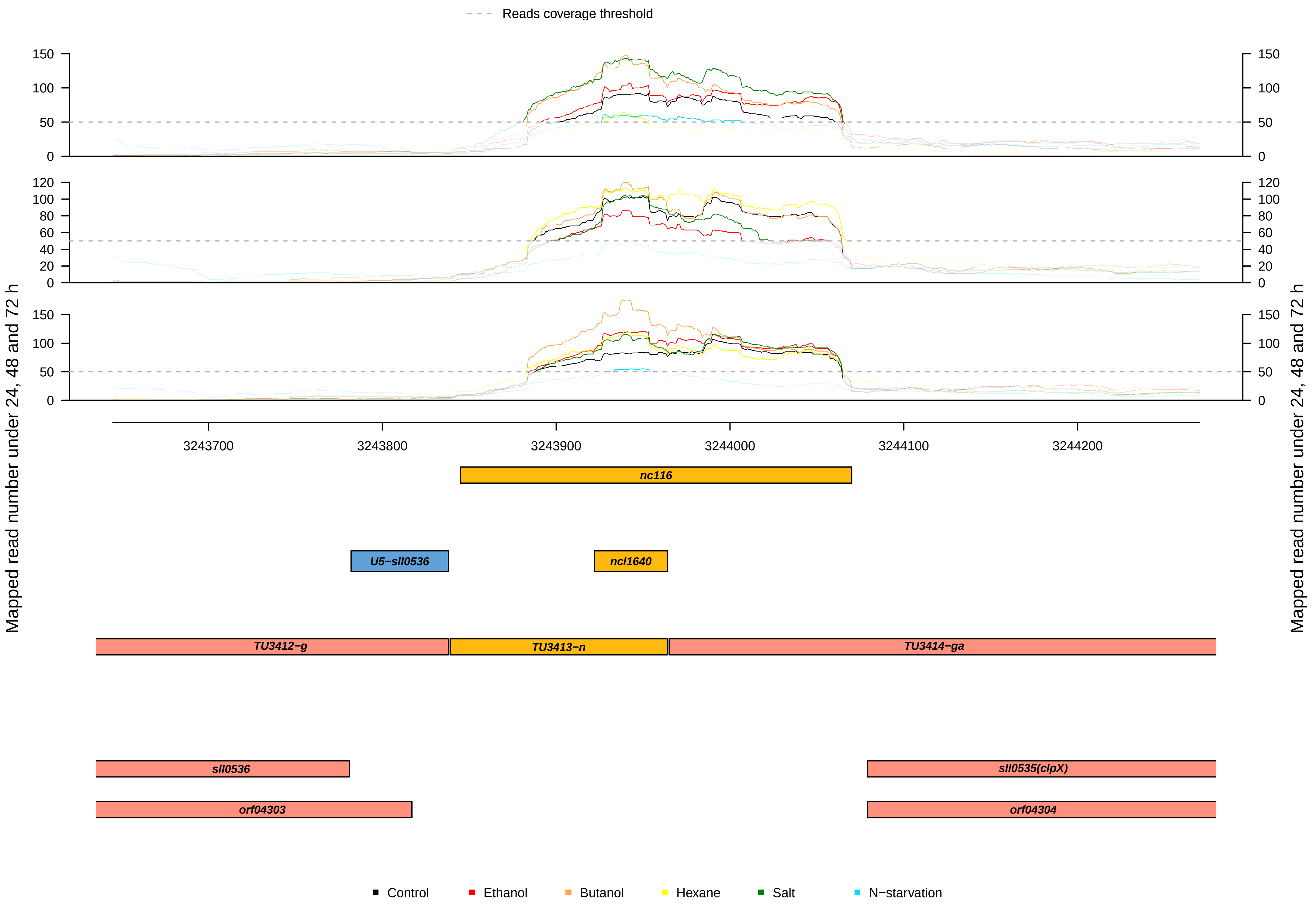

Reads coverage threshold
Mapped read number under 24, 48 and 72 h
3243700
3243800
3243900
3244000
3244100
3244200
nc116
U5−sll0536
ncl1640
TU3412−g
TU3413−n
TU3414−ga
sll0536
sll0535(clpX)
orf04303
orf04304
Control
Ethanol
Butanol
Hexane
Salt
N−starvation

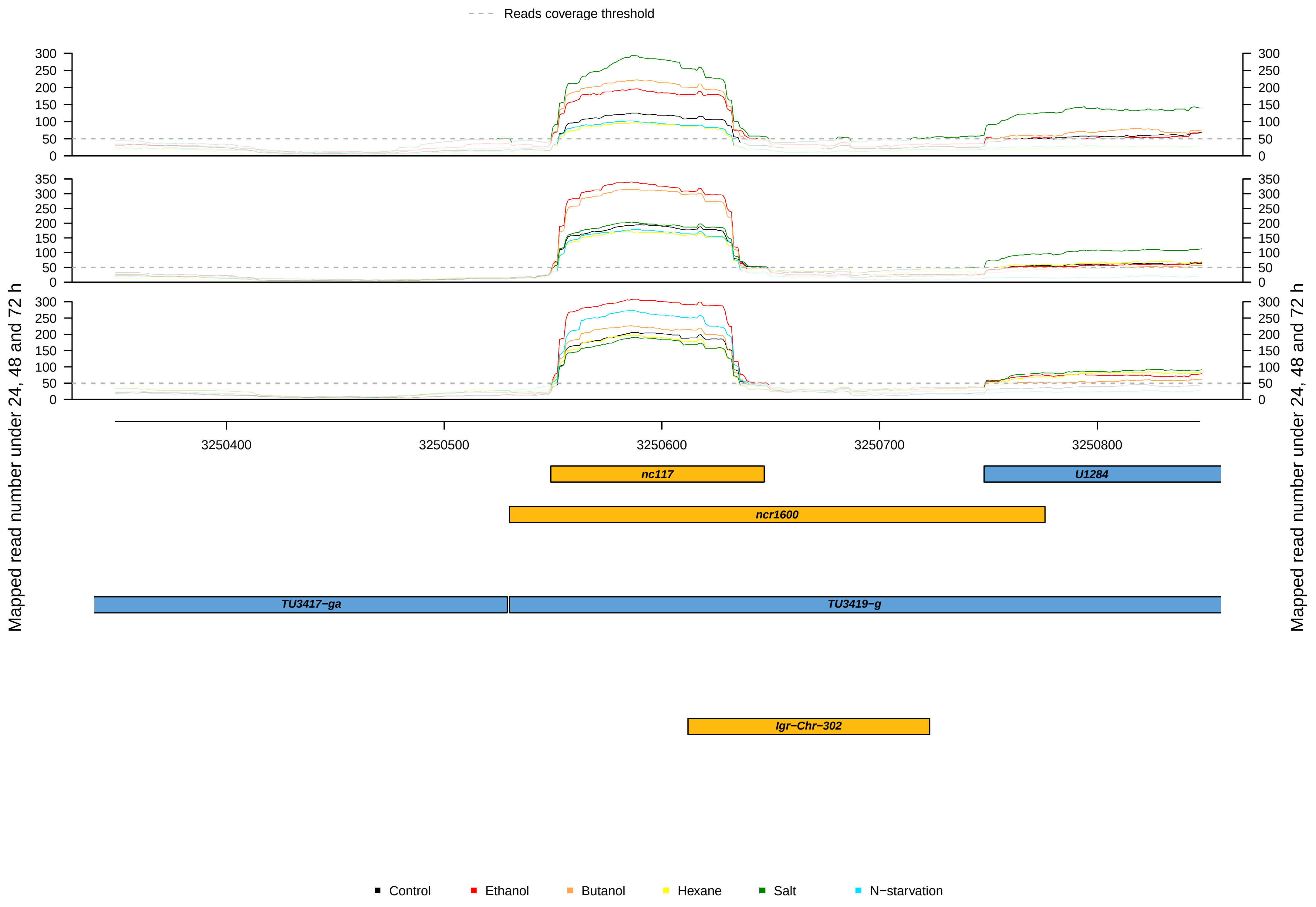

Reads coverage threshold
Mapped read number under 24, 48 and 72 h
3250400
3250500
3250600
3250700
3250800
nc117
U1284
ncr1600
TU3417−ga
TU3419−g
Igr−Chr−302
Control
Ethanol
Butanol
Hexane
Salt
N−starvation

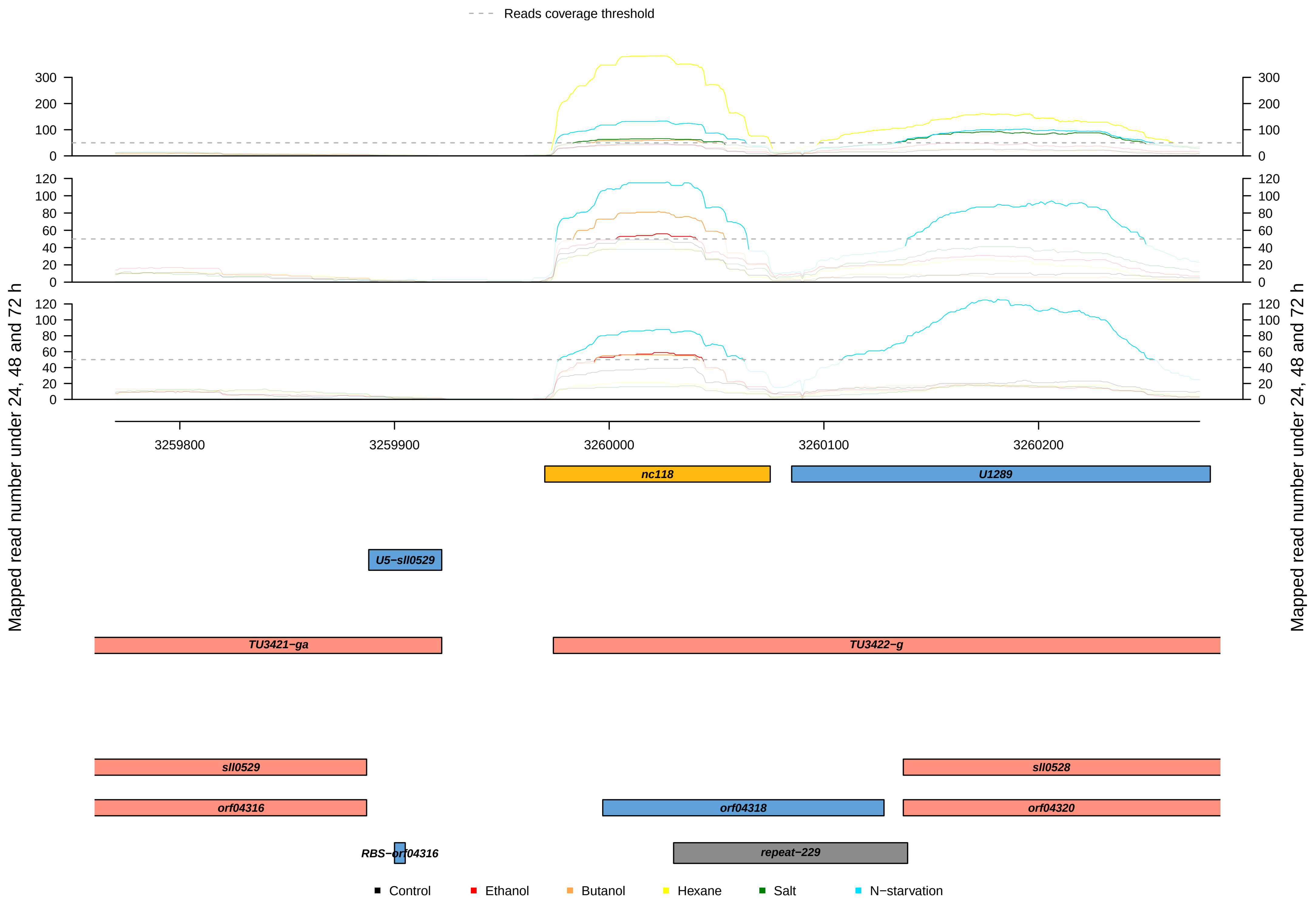

Reads coverage threshold
Mapped read number under 24, 48 and 72 h
3259800
3259900
3260000
3260100
3260200
nc118
U1289
U5−sll0529
TU3421−ga
TU3422−g
sll0529
sll0528
orf04316
orf04318
orf04320
RBS−orf04316
repeat−229
Control
Ethanol
Butanol
Hexane
Salt
N−starvation

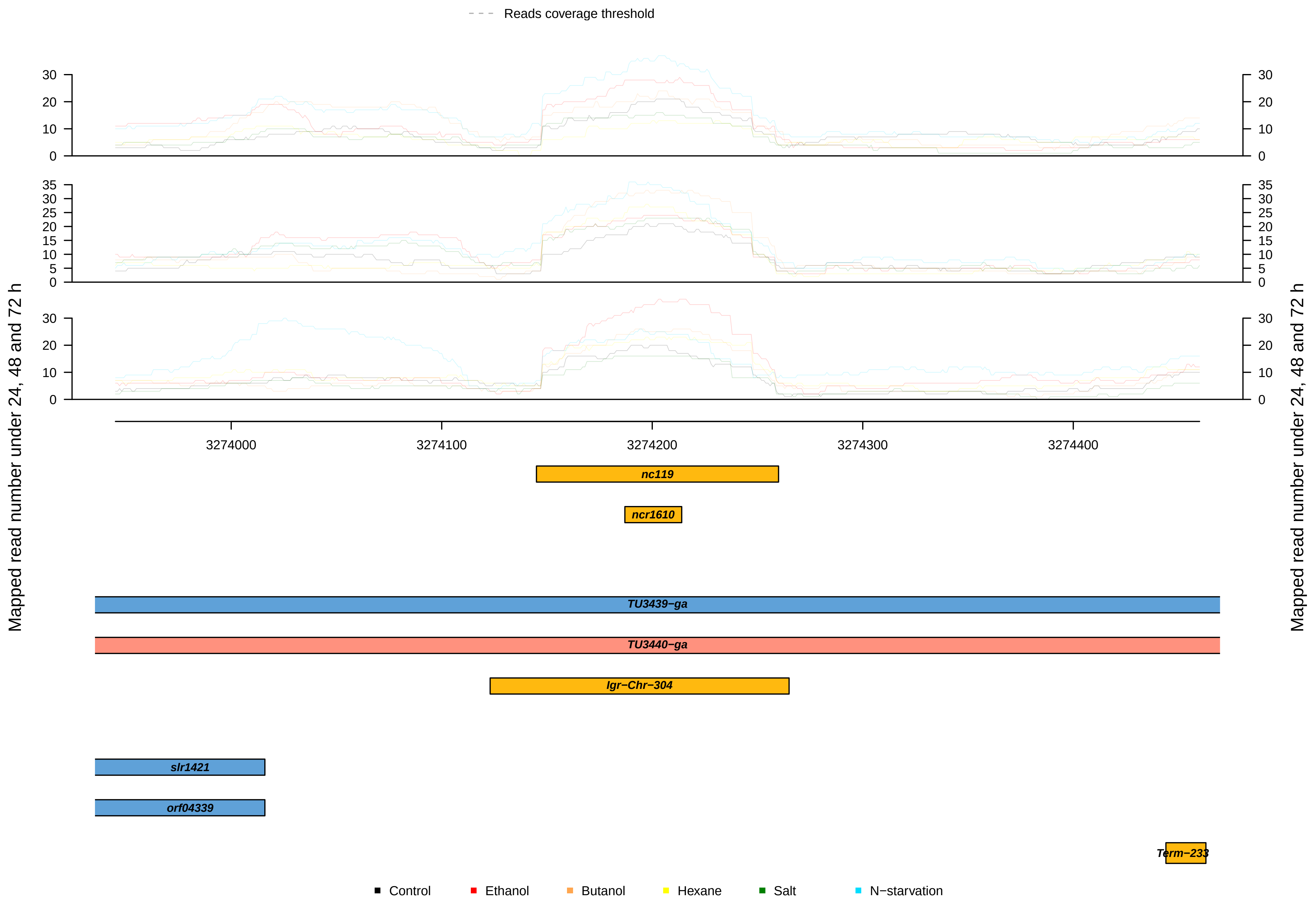

Reads coverage threshold
Mapped read number under 24, 48 and 72 h
30
20
10
0
35
30
25
20
15
10
5
0
3274000
3274100
3274200
3274300
3274400
nc119
ncr1610
TU3439−ga
TU3440−ga
Igr−Chr−304
slr1421
orf04339
Term−233
Control
Ethanol
Butanol
Hexane
Salt
N−starvation

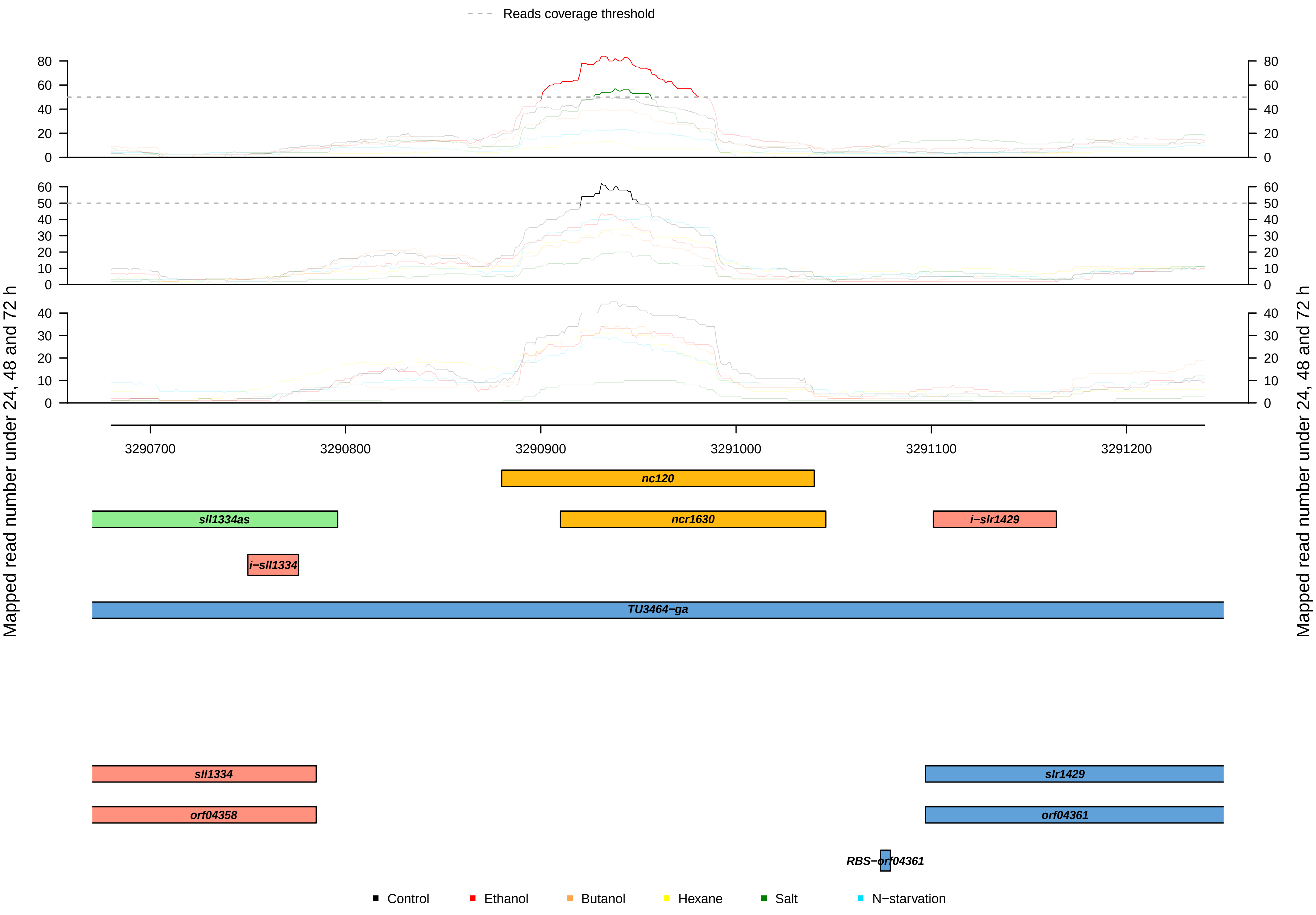

Reads coverage threshold
Mapped read number under 24, 48 and 72 h
3290700
3290800
3290900
3291000
3291100
3291200
nc120
sll1334as
ncr1630
i−slr1429
i−sll1334
TU3464−ga
sll1334
slr1429
orf04358
orf04361
RBS−orf04361
Control
Ethanol
Butanol
Hexane
Salt
N−starvation

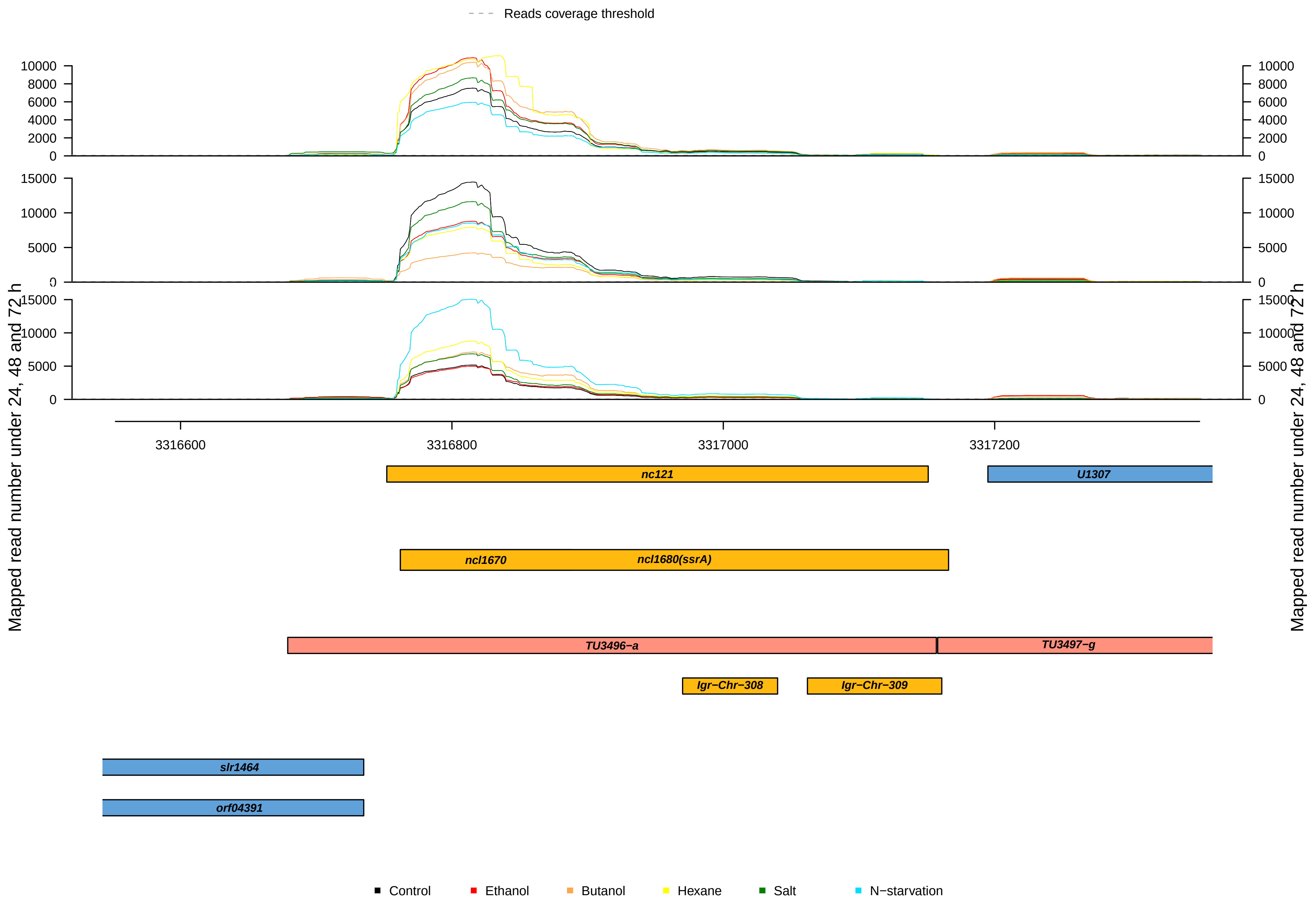

Reads coverage threshold
10000
8000
6000
4000
2000
0
15000
10000
5000
0
Mapped read number under 24, 48 and 72 h
3316600
3316800
3317000
3317200
nc121
U1307
ncl1670
ncl1680(ssrA)
TU3496−a
TU3497−g
Igr−Chr−308
Igr−Chr−309
slr1464
orf04391
Control
Ethanol
Butanol
Hexane
Salt
N−starvation

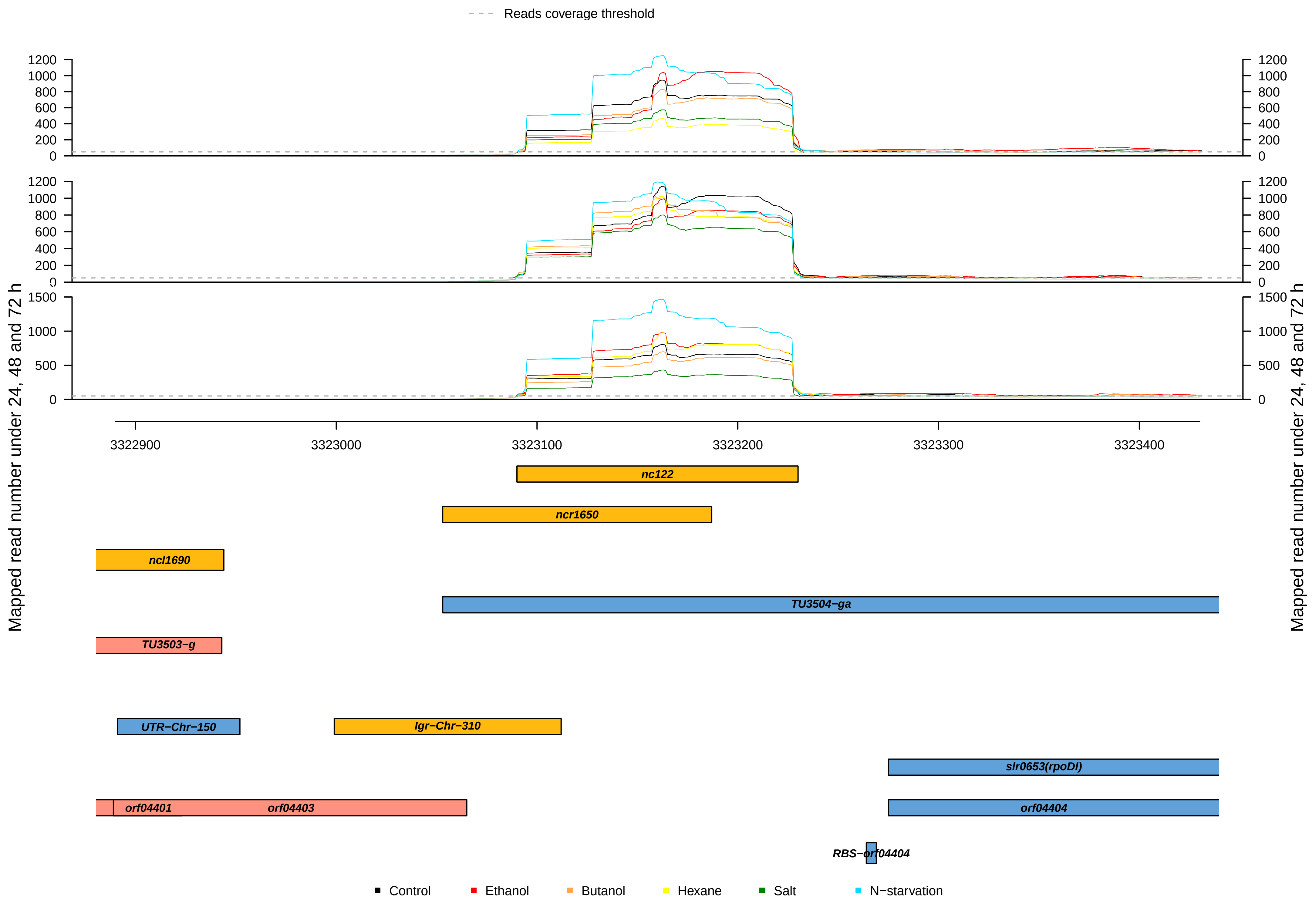

Reads coverage threshold
Mapped read number under 24, 48 and 72 h
1200
1000
800
600
400
200
0
1500
1000
500
0
3322900
3323000
3323100
3323200
3323300
3323400
nc122
ncr1650
ncl1690
TU3504−ga
TU3503−g
UTR−Chr−150
Igr−Chr−310
slr0653(rpoDI)
orf04401
orf04403
orf04404
RBS−orf04404
Control
Ethanol
Butanol
Hexane
Salt
N−starvation

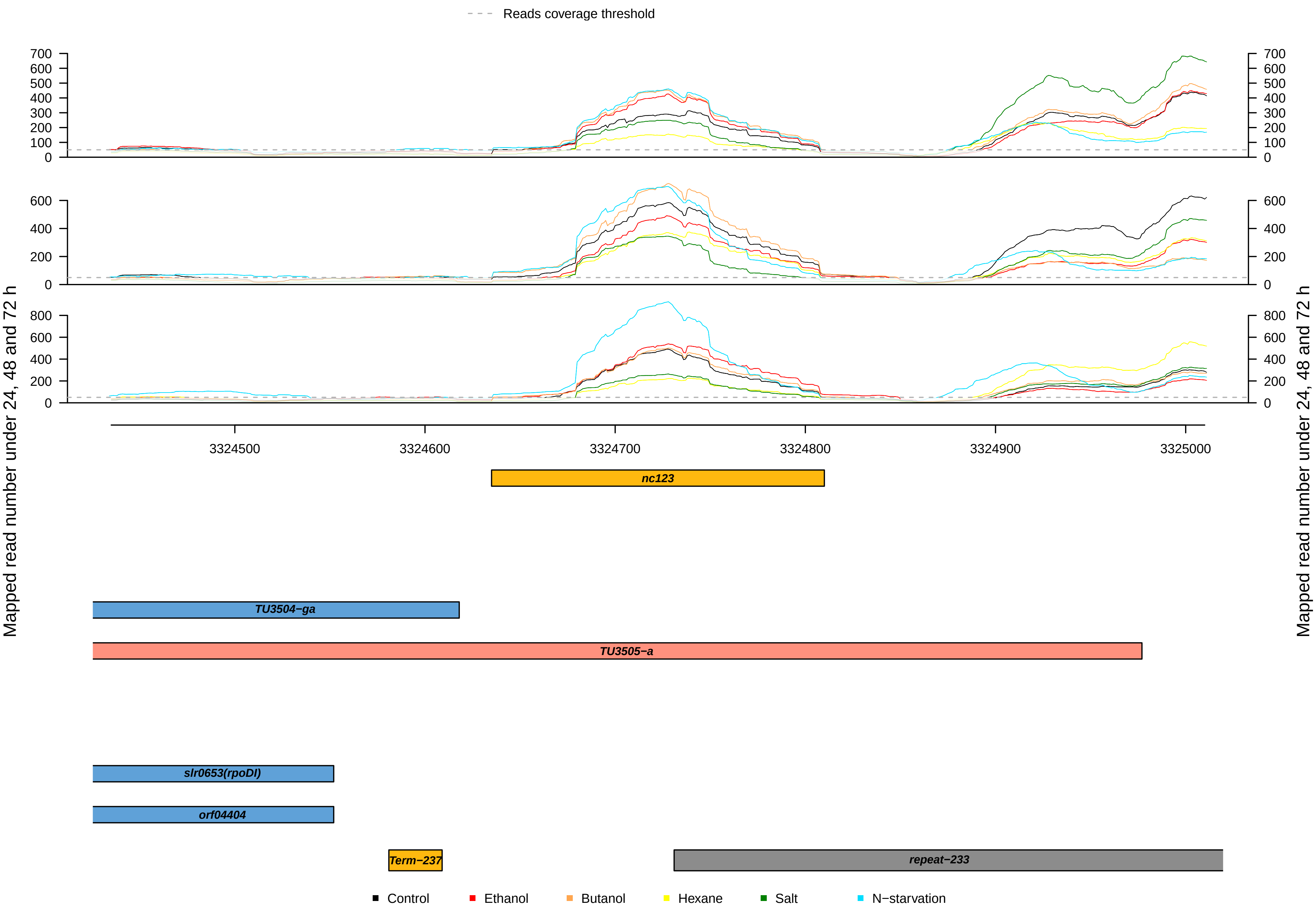

Reads coverage threshold
700
600
500
400
300
200
100
0
600
400
200
0
800
600
400
200
0
Mapped read number under 24, 48 and 72 h
3324500
3324600
3324700
3324800
3324900
3325000
nc123
TU3504−ga
TU3505−a
slr0653(rpoDI)
orf04404
Term−237
repeat−233
Control
Ethanol
Butanol
Hexane
Salt
N−starvation

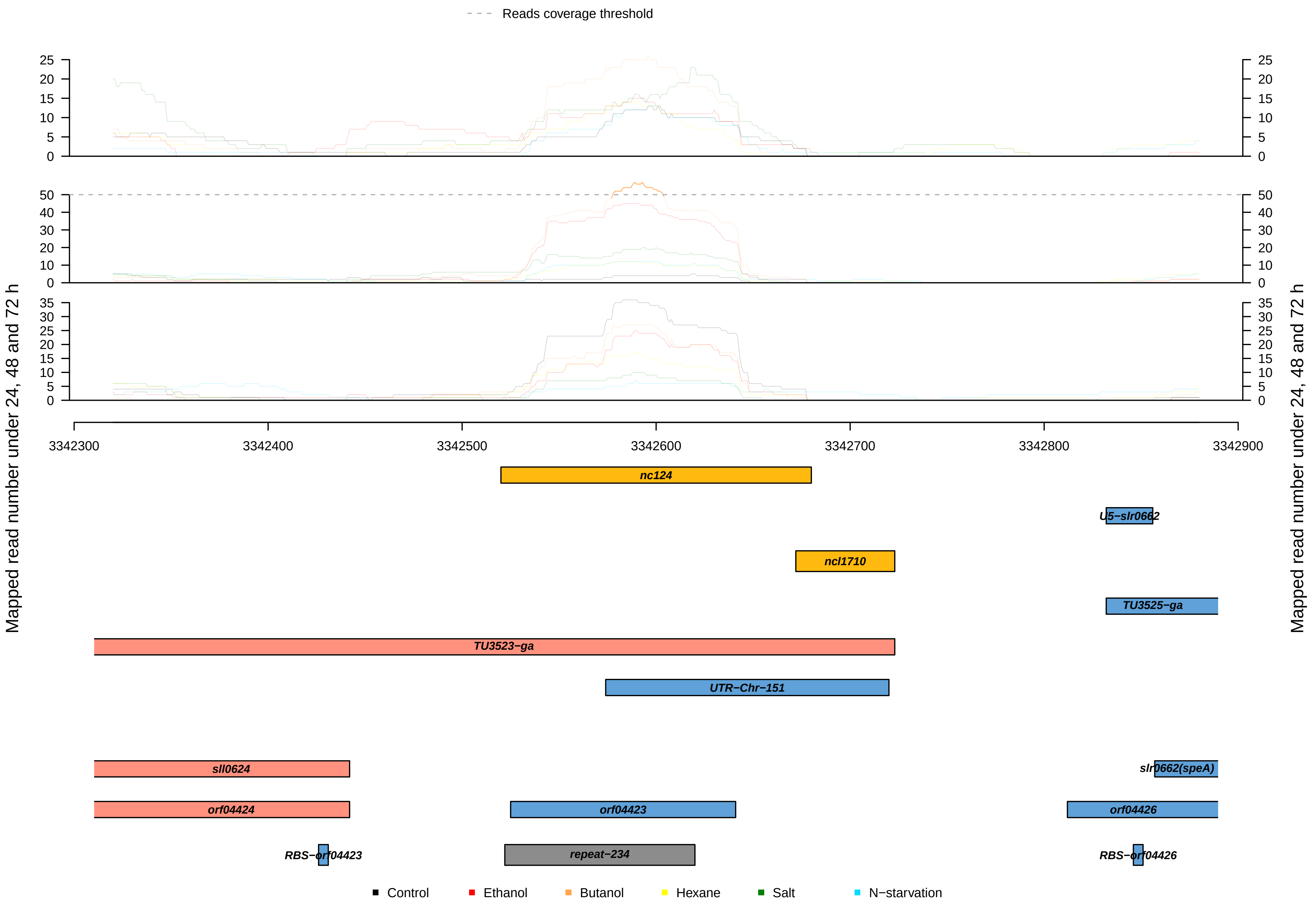

Reads coverage threshold
Mapped read number under 24, 48 and 72 h
3342300
3342400
3342500
3342600
3342700
3342800
3342900
nc124
U5−slr0662
ncl1710
TU3525−ga
TU3523−ga
UTR−Chr−151
sll0624
slr0662(speA)
orf04424
orf04423
orf04426
RBS−orf04423
repeat−234
RBS−orf04426
Control
Ethanol
Butanol
Hexane
Salt
N−starvation

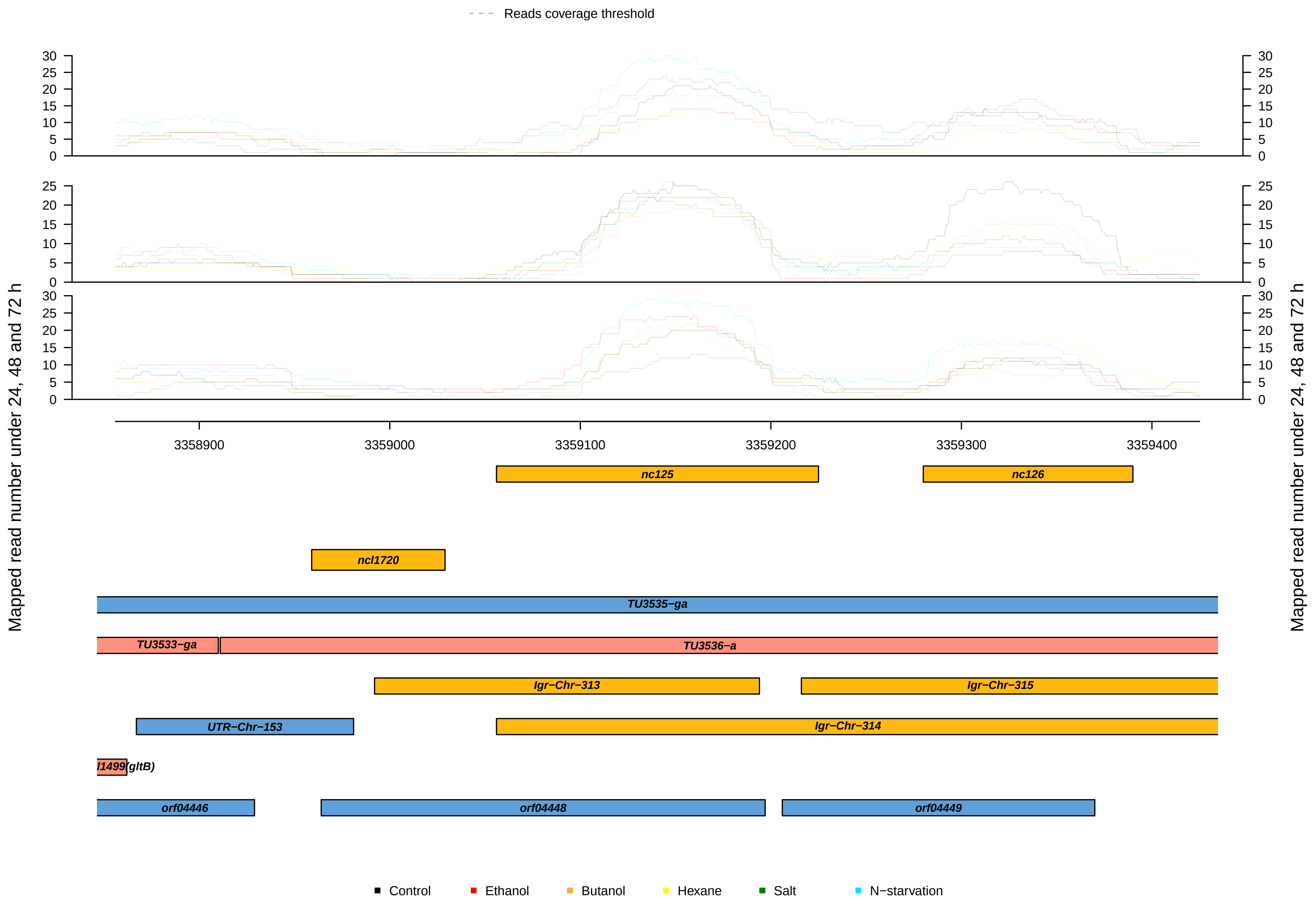

Reads coverage threshold
Mapped read number under 24, 48 and 72 h
3358900
3359000
3359100
3359200
3359300
3359400
nc125
nc126
ncl1720
TU3535−ga
TU3533−ga
TU3536−a
Igr−Chr−313
Igr−Chr−315
UTR−Chr−153
Igr−Chr−314
l1499(gltB)
orf04446
orf04448
orf04449
Control
Ethanol
Butanol
Hexane
Salt
N−starvation

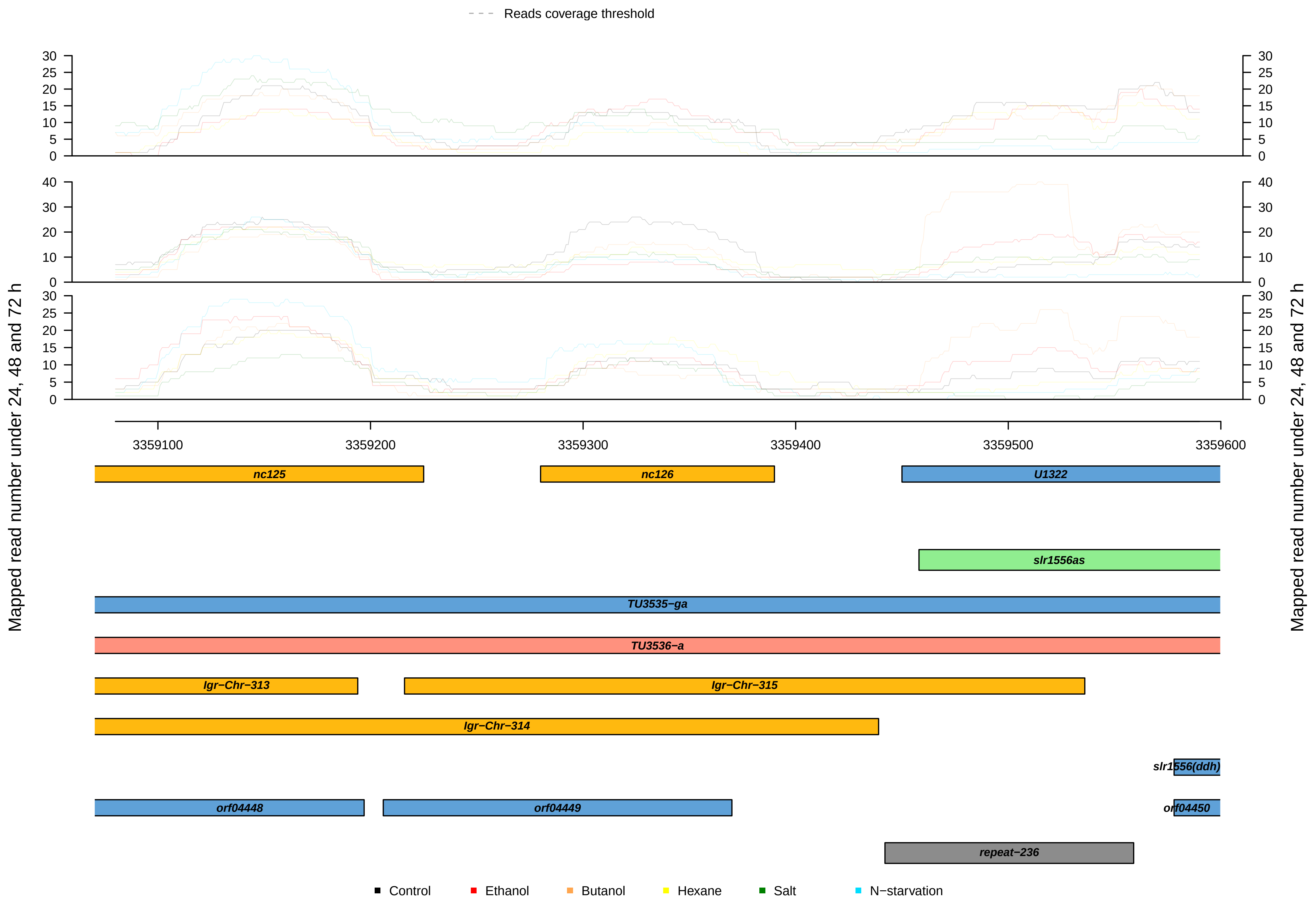

Reads coverage threshold
Mapped read number under 24, 48 and 72 h
3359100
3359200
3359300
3359400
3359500
3359600
nc125
nc126
U1322
slr1556as
TU3535−ga
TU3536−a
Igr−Chr−313
Igr−Chr−315
Igr−Chr−314
slr1556(ddh)
orf04448
orf04449
orf04450
repeat−236
Control
Ethanol
Butanol
Hexane
Salt
N−starvation

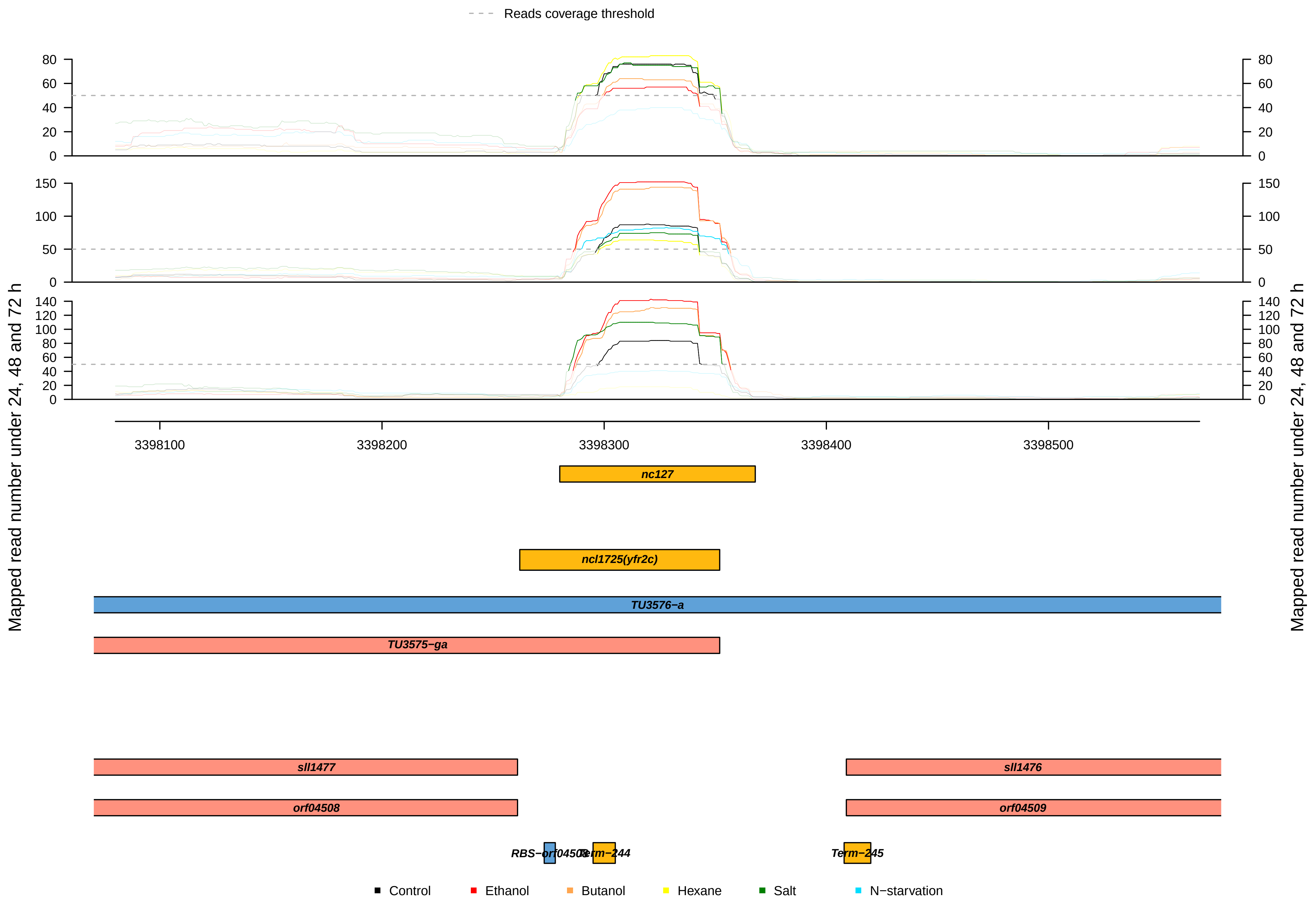
Reads coverage threshold
80
60
40
20
0
150
100
50
0
140
120
100
80
60
40
20
0
Mapped read number under 24, 48 and 72 h
3398100
3398200
3398300
3398400
3398500
nc127
ncl1725(yfr2c)
TU3576−a
TU3575−ga
sll1477
sll1476
orf04508
orf04509
RBS−orf04508
Term−244
Term−245
Control
Ethanol
Butanol
Hexane
Salt
N−starvation

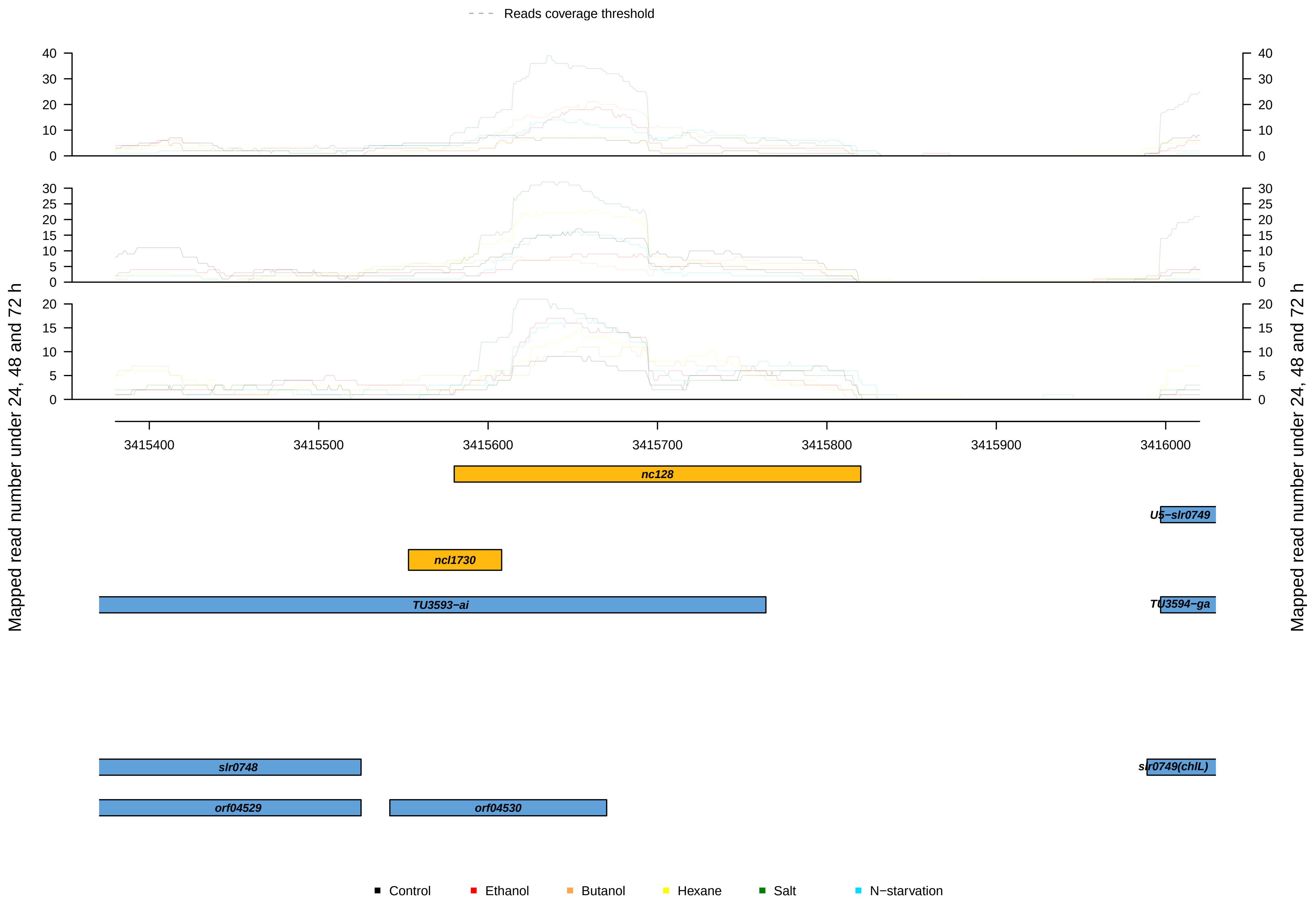

Reads coverage threshold
Mapped read number under 24, 48 and 72 h
3415400
3415500
3415600
3415700
3415800
3415900
3416000
nc128
U5−slr0749
ncl1730
TU3593−ai
TU3594−ga
slr0748
slr0749(chlL)
orf04529
orf04530
Control
Ethanol
Butanol
Hexane
Salt
N−starvation

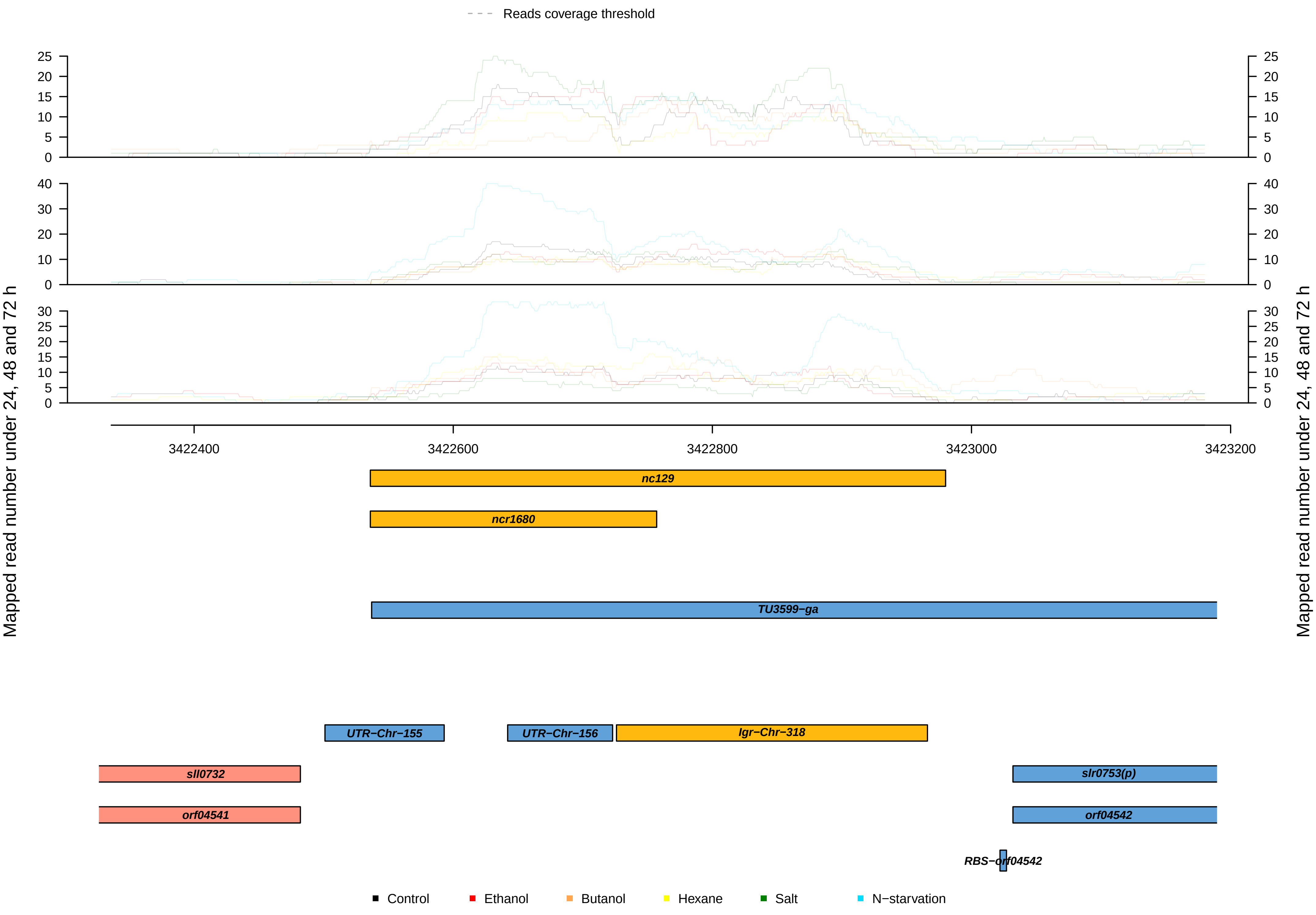

Reads coverage threshold
Mapped read number under 24, 48 and 72 h
3422400
3422600
3422800
3423000
3423200
nc129
ncr1680
TU3599−ga
UTR−Chr−155
UTR−Chr−156
Igr−Chr−318
sll0732
slr0753(p)
orf04541
orf04542
RBS−orf04542
Control
Ethanol
Butanol
Hexane
Salt
N−starvation

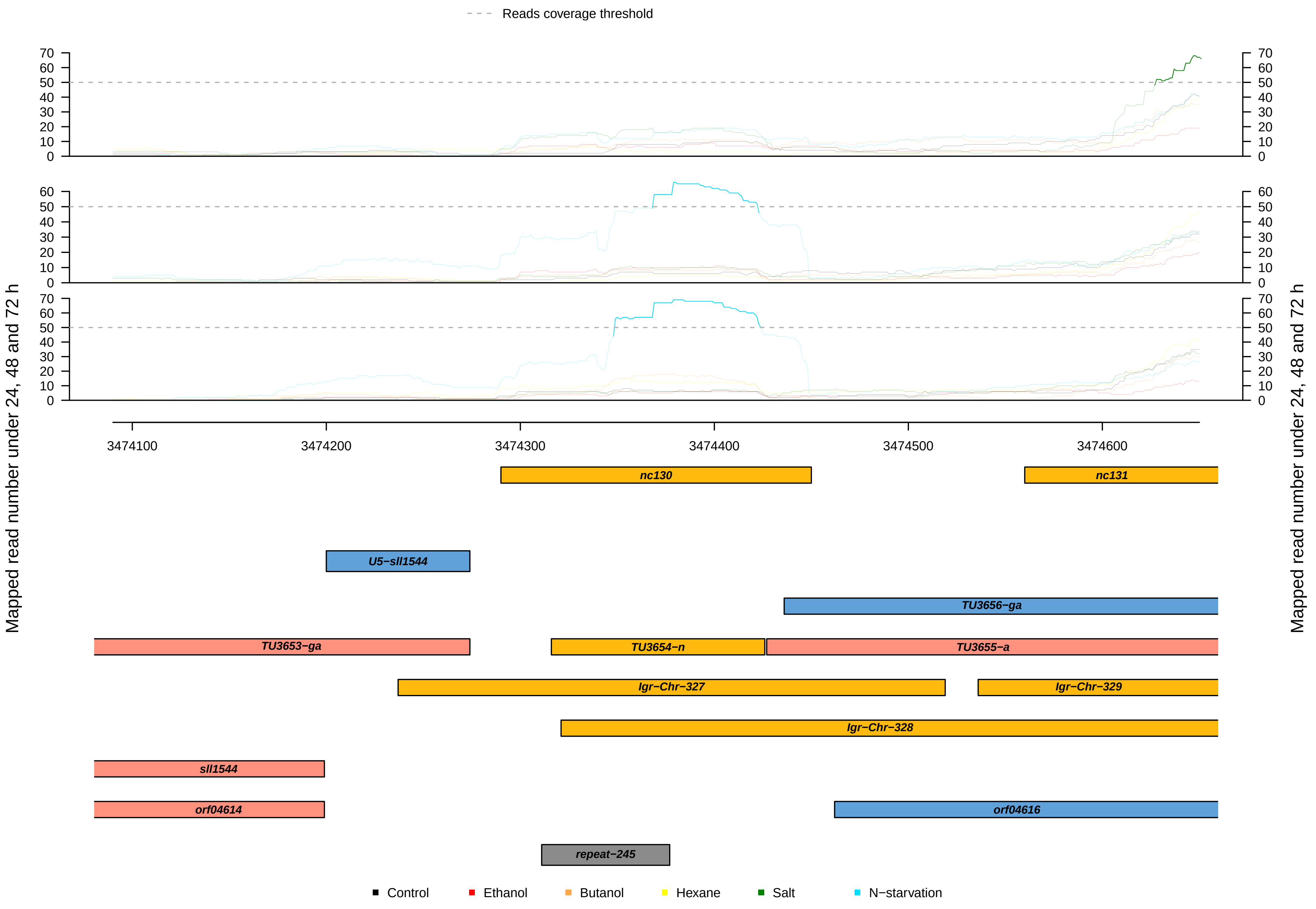

Reads coverage threshold
Mapped read number under 24, 48 and 72 h
70
60
50
40
30
20
10
0
3474100
3474200
3474300
3474400
3474500
3474600
nc130
nc131
U5−sll1544
TU3656−ga
TU3653−ga
TU3654−n
TU3655−a
Igr−Chr−327
Igr−Chr−329
Igr−Chr−328
sll1544
orf04614
orf04616
repeat−245
Control
Ethanol
Butanol
Hexane
Salt
N−starvation

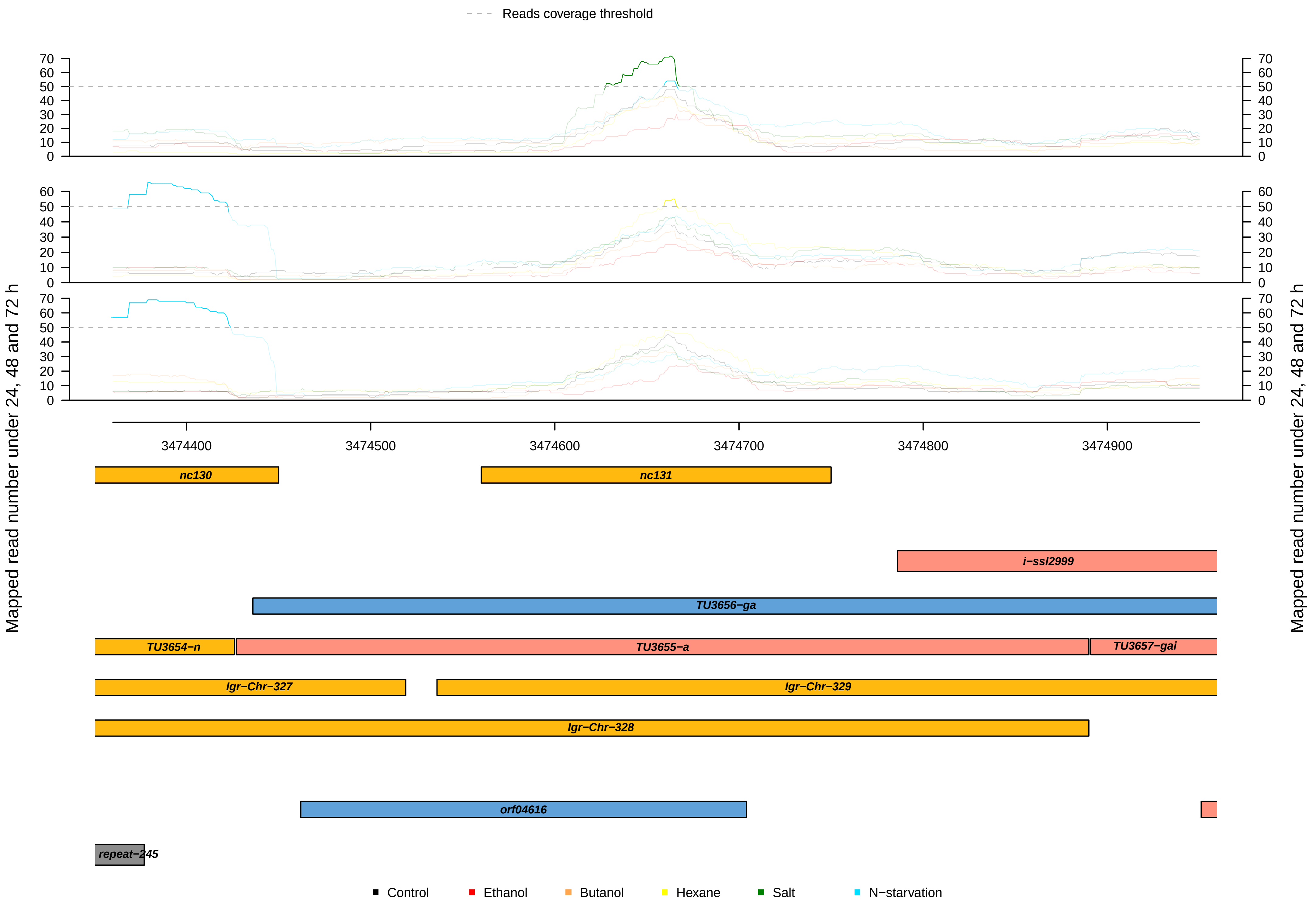

Reads coverage threshold
Mapped read number under 24, 48 and 72 h
3474400
3474500
3474600
3474700
3474800
3474900
nc130
nc131
i−ssl2999
TU3656−ga
TU3654−n
TU3655−a
TU3657−gai
Igr−Chr−327
Igr−Chr−329
Igr−Chr−328
orf04616
repeat−245
Control
Ethanol
Butanol
Hexane
Salt
N−starvation

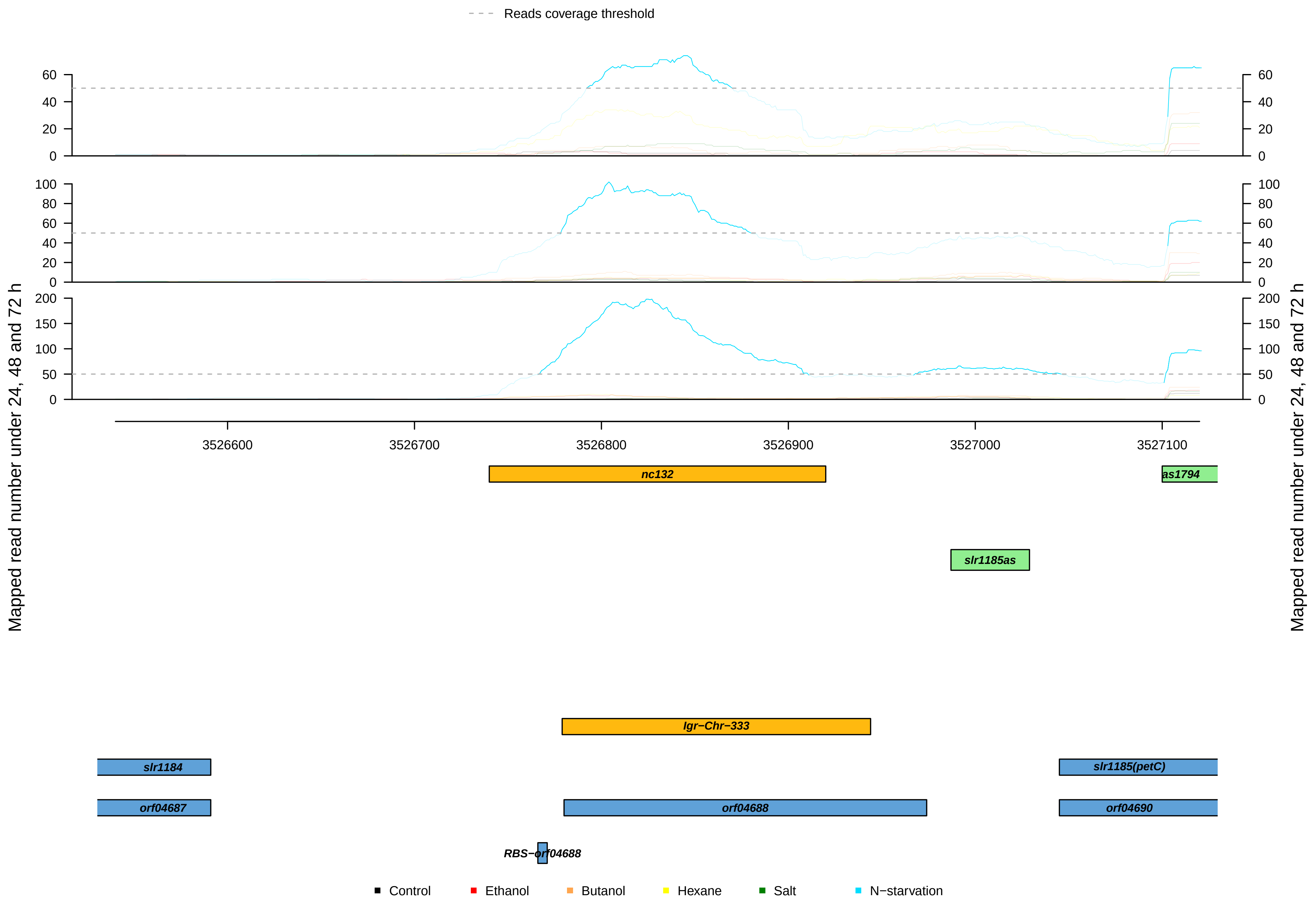

Reads coverage threshold
Mapped read number under 24, 48 and 72 h
60
40
20
0
100
80
60
40
20
0
200
150
100
50
0
3526600
3526700
3526800
3526900
3527000
3527100
nc132
as1794
slr1185as
Igr−Chr−333
slr1184
slr1185(petC)
orf04687
orf04688
orf04690
RBS−orf04688
Control
Ethanol
Butanol
Hexane
Salt
N−starvation

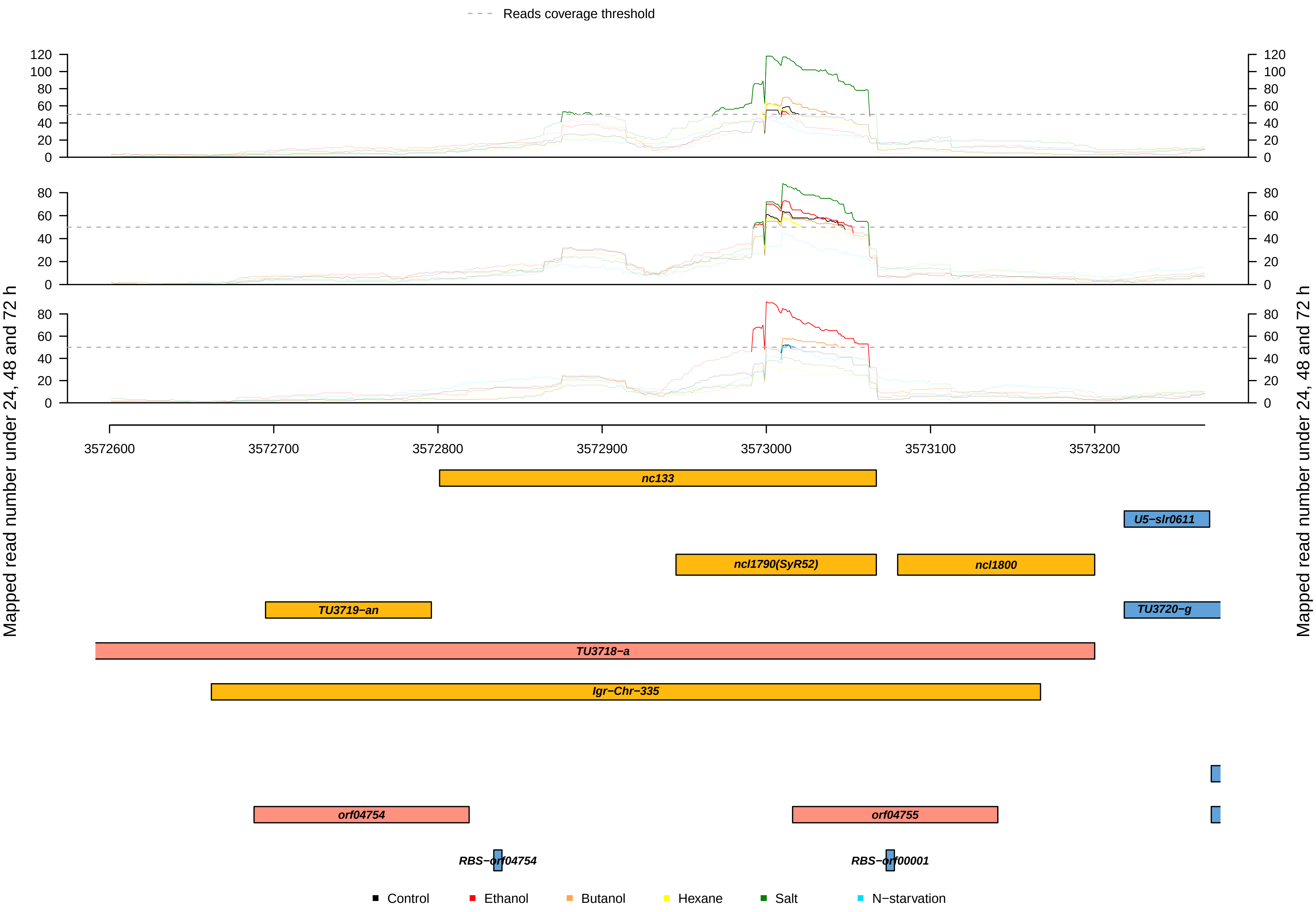

Reads coverage threshold
Mapped read number under 24, 48 and 72 h
3572600
3572700
3572800
3572900
3573000
3573100
3573200
nc133
U5−slr0611
ncl1790(SyR52)
ncl1800
TU3719−an
TU3720−g
TU3718−a
Igr−Chr−335
orf04754
orf04755
RBS−orf04754
RBS−orf00001
Control
Ethanol
Butanol
Hexane
Salt
N−starvation
