# Supplementary figures and images for: Systematic and functional identification of small non-coding RNAs associated with exogenous biofuel stress in cyanobacterium Synechocystis sp. PCC 6803
Source: Biotechnol Biofuels. 2017 Mar 7;10:57. doi: 10.1186/s13068-017-0743-y (PMC5341163; doi:10.1186/s13068-017-0743-y)

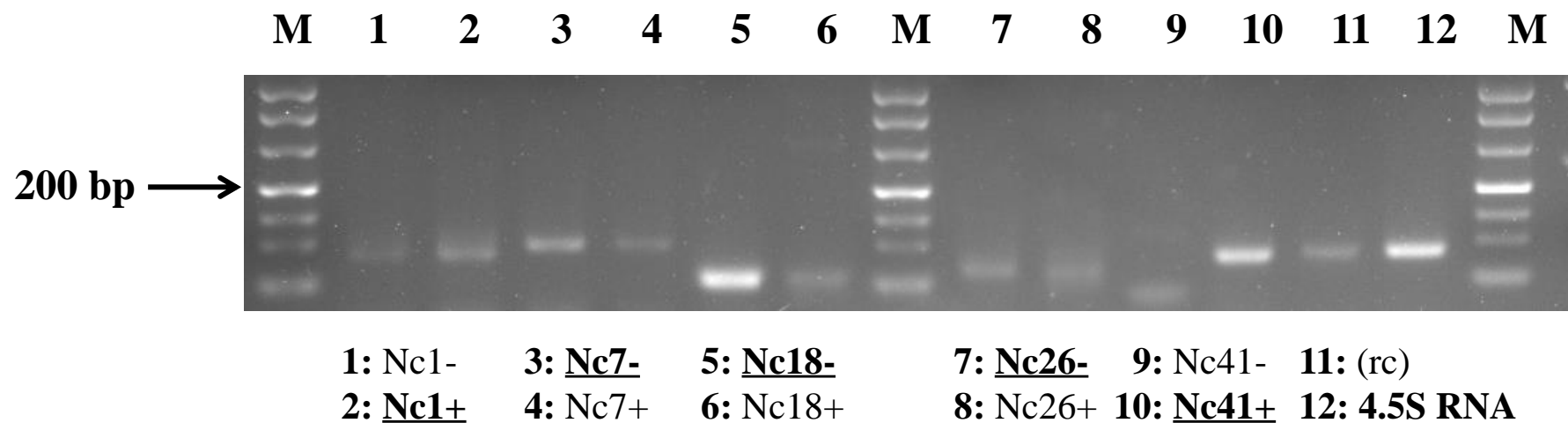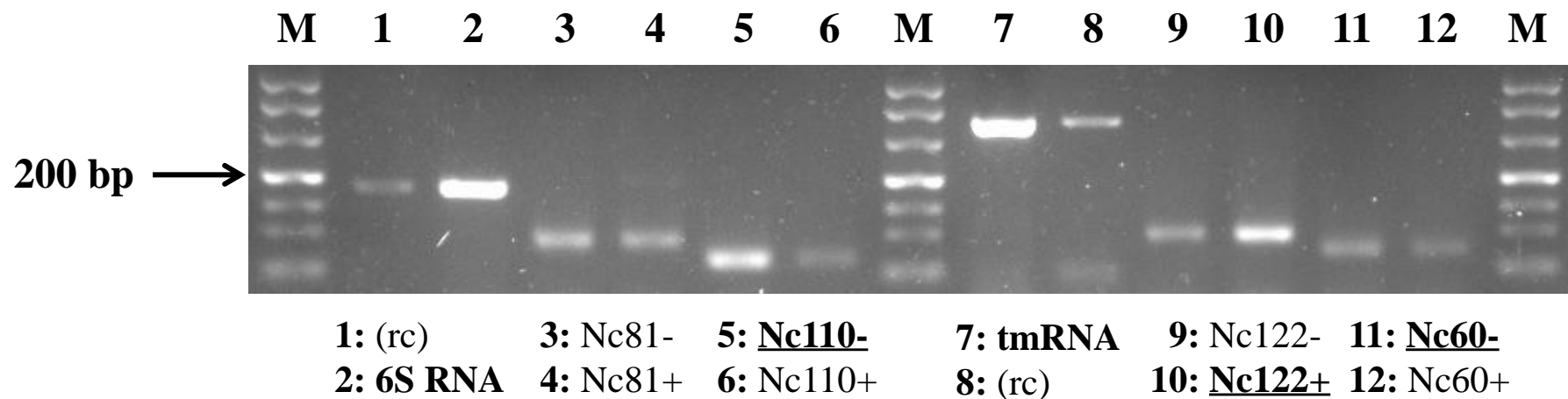

Supplement: Supplementary file 6 — Additional file 6: Figure S3. Experimental verification of the top abundant sRNAs and determination of transcriptional orientation by two-step RT-PCR. “+” denotes the orientation of the sRNA on the positive genome strand, “−” denotes the orientation of the sRNA on the negative genome strand. The bold and underlined names indicate the determined orientation of an sRNA, and “rc” denotes the reverse complement direction of the known sRNAs. [file 13068_2017_743_MOESM6_ESM.pdf]

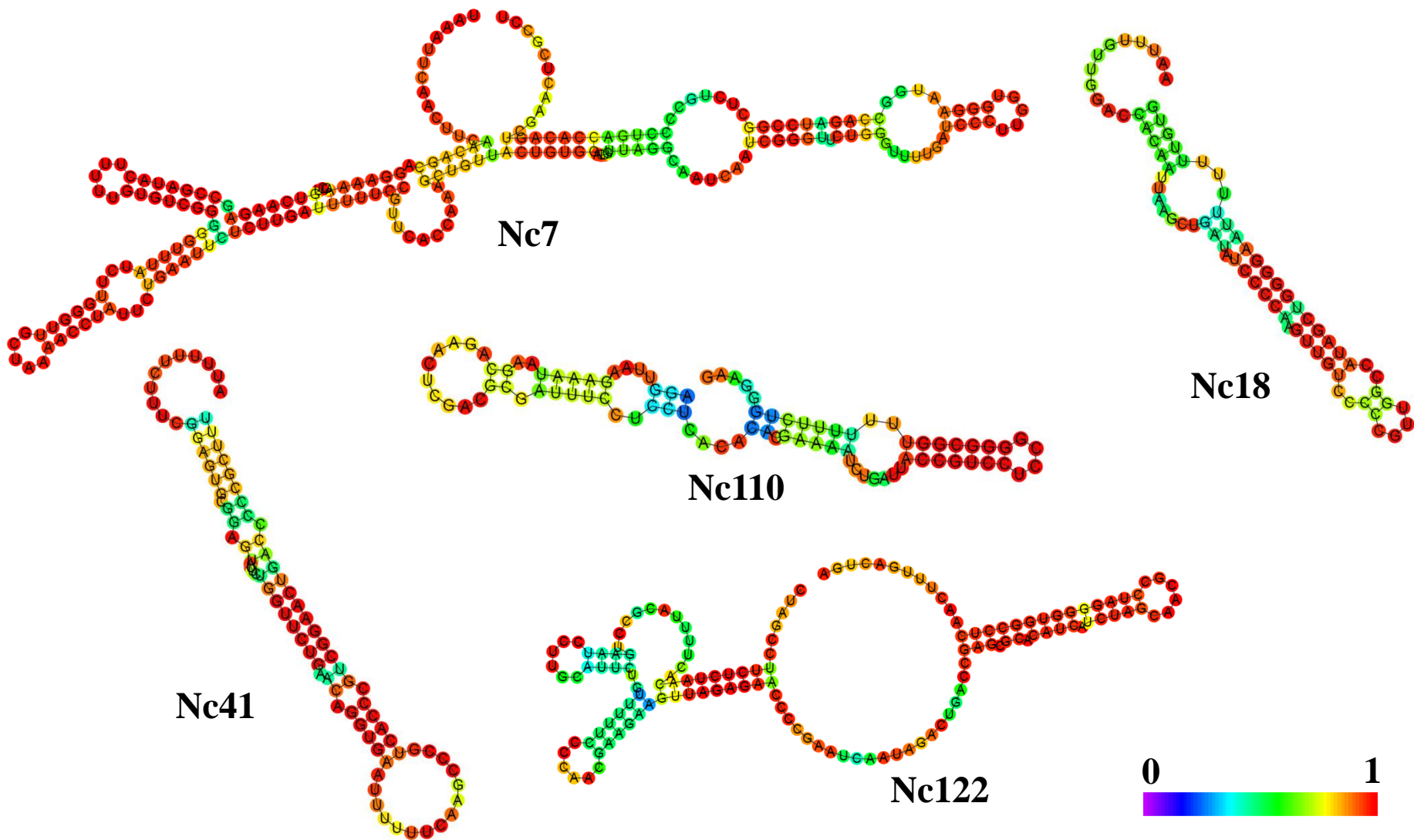

Supplement: Supplementary file 7 — Additional file 7: Figure S4. Secondary structures predicted for abundant sRNAs. Color bar represents base-pair probabilities within sRNAs. [file 13068_2017_743_MOESM7_ESM.pdf]

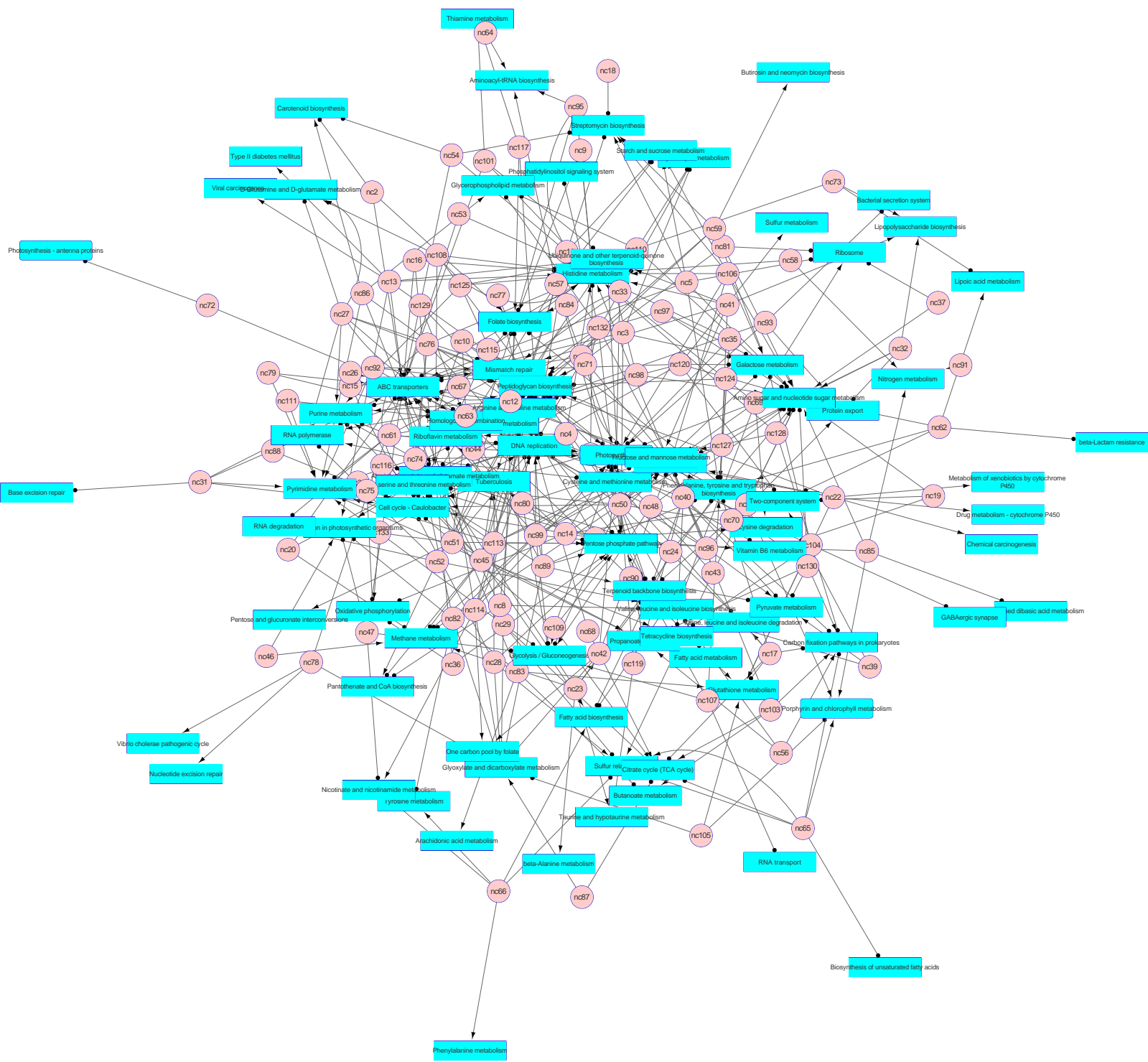

Supplement: Supplementary file 8 — Additional file 8: Figure S5. Visualization of trans-encoded sRNAs regulatory network in Synechocystis. Detailed description is the same as Fig. 5. [file 13068_2017_743_MOESM8_ESM.pdf]

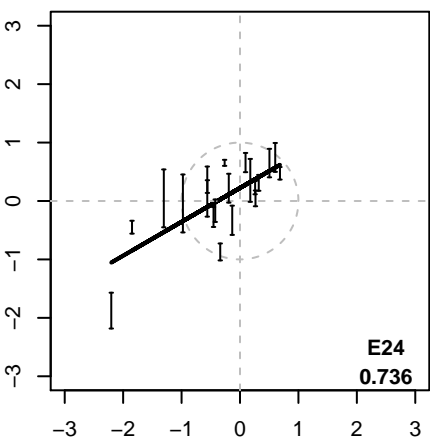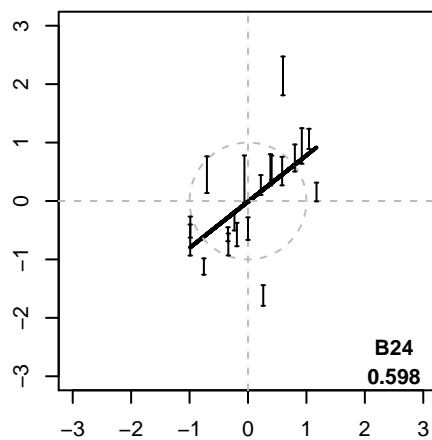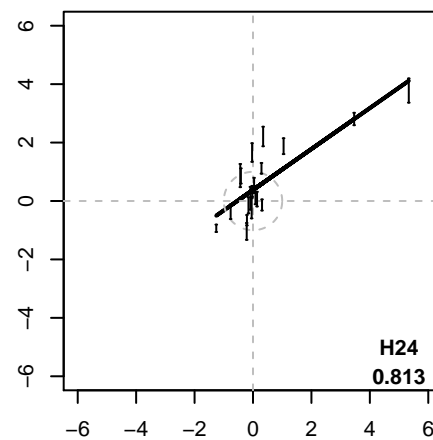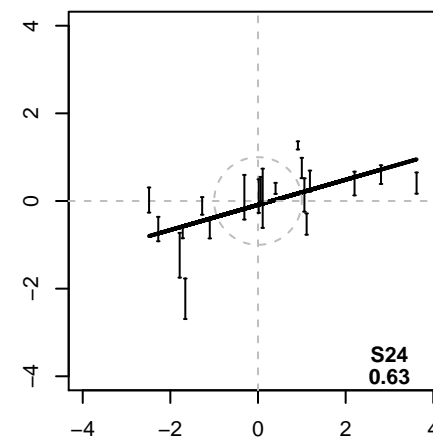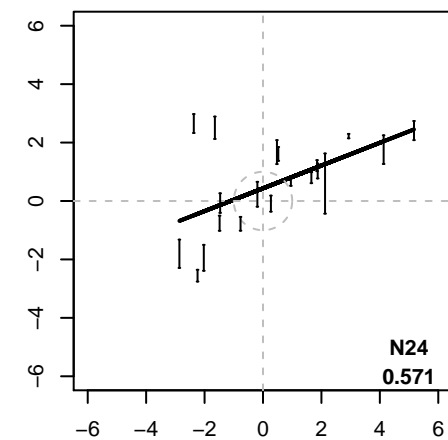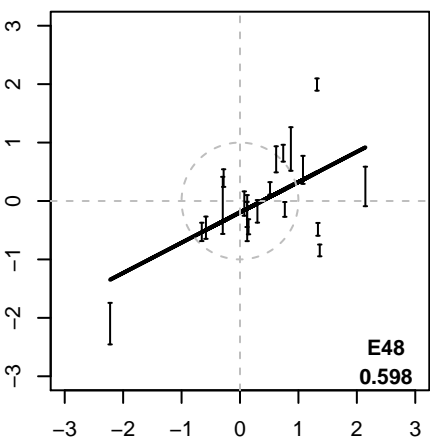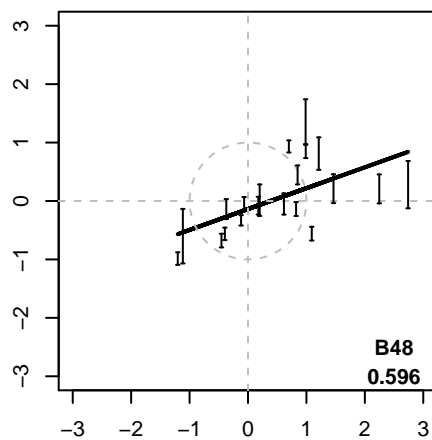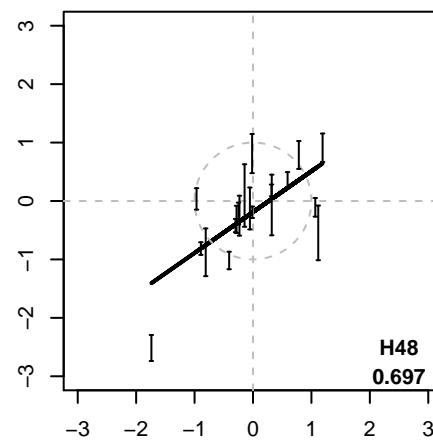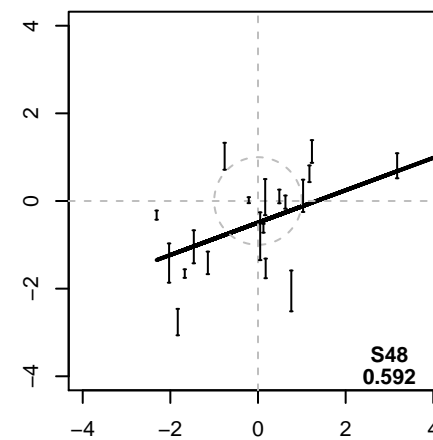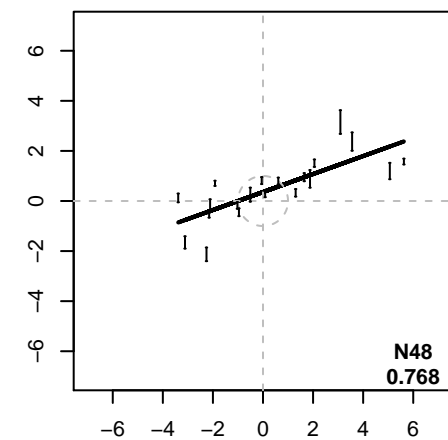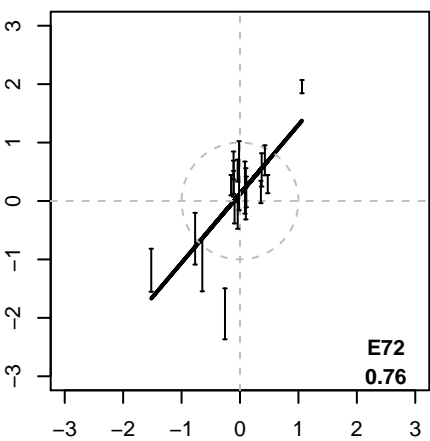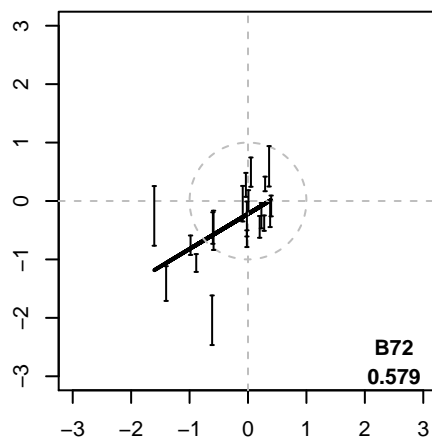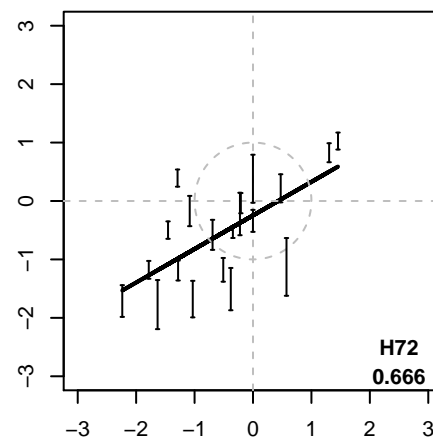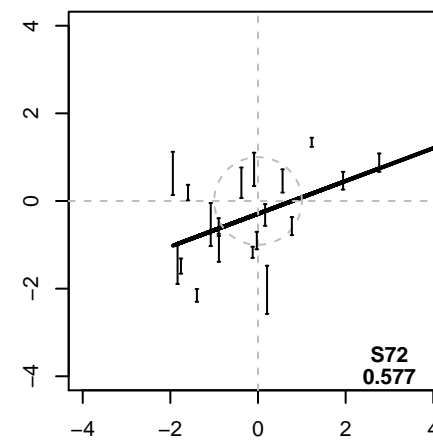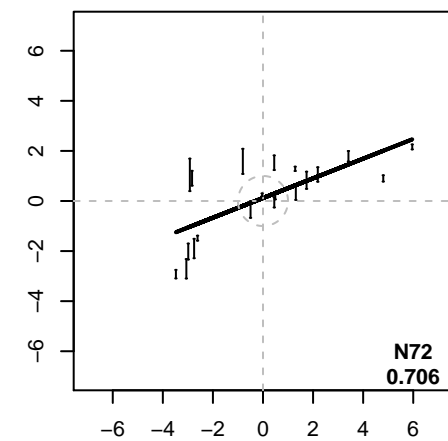

Supplement: Supplementary file 10 — Additional file 10: Figure S6. Correlation between qRT-PCR and sRNA-seq analyses for selected genes. For sRNA-seq (horizontal coordinate), values represent log2 fold change of sRNA under stress conditions compared to WT. For qRT-PCR (vertical coordinate), values represent the mean log2 fold changes in sRNA of three technical and three biological replicates under stress conditions compared to WT. The error bar represents the standard deviation of three replicates. Sample names and Pearson correlation coefficients are indicated at the right lower corner of each plot. [file 13068_2017_743_MOESM10_ESM.pdf]

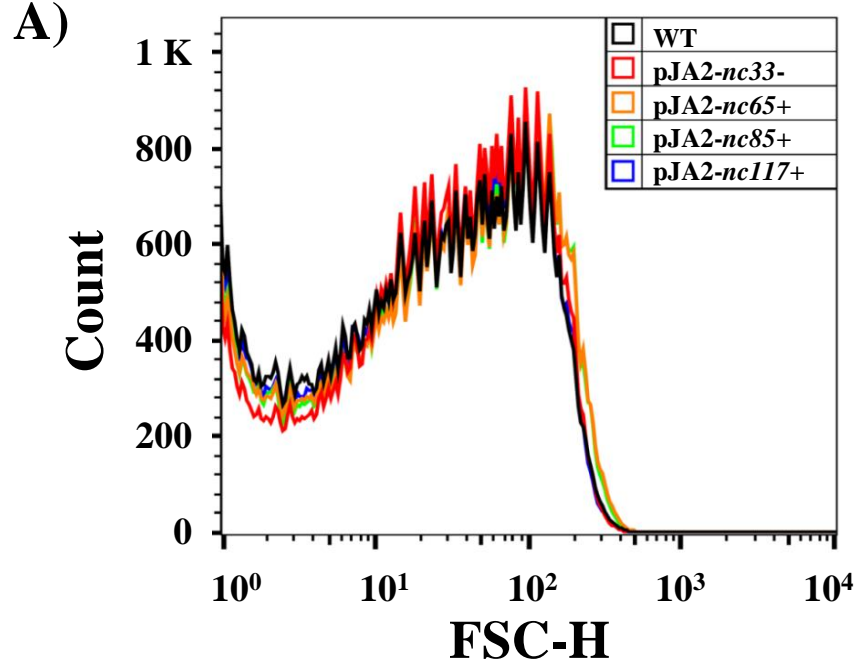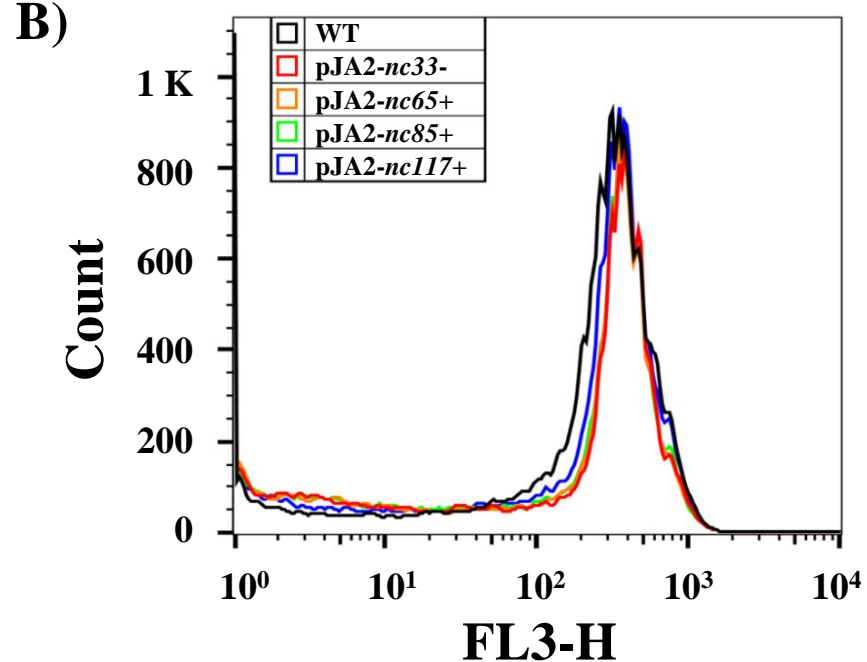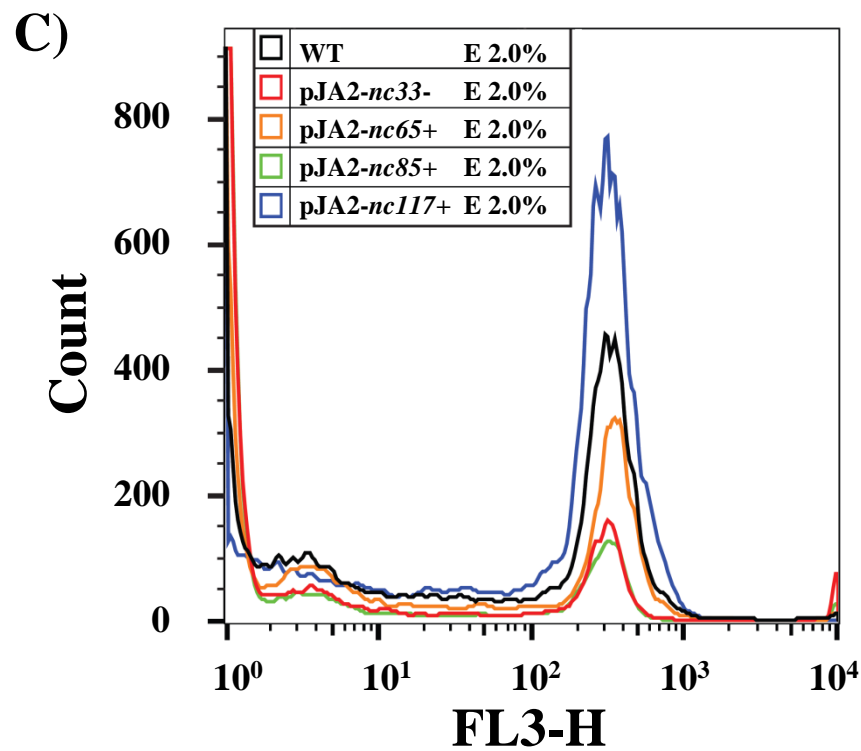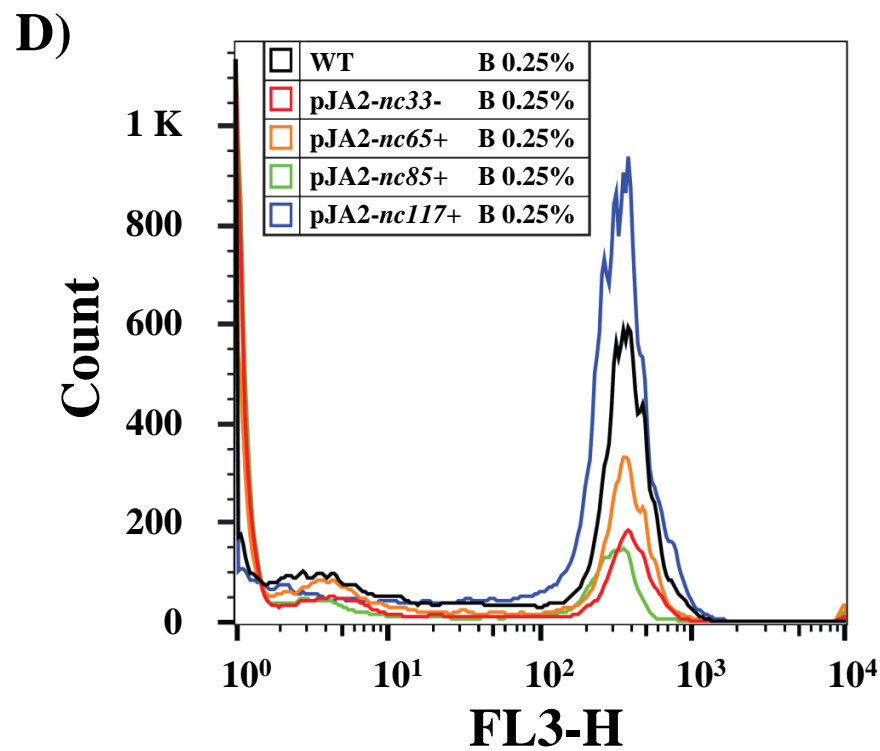

Supplement: Supplementary file 15 — Additional file 15: Figure S9. Flow-cytometric analysis of WT, pJA2-nc33−, pJA2-nc65+, pJA2-nc85+ and pJA2-nc117+ strains. Forward scatter (FSC) is related to cell size and the FL3 channel with the 670/LP filter, which is related to chlorophyll fluorescence. The y-axis was normalized according to pixel count. (A) FSC histogram of cells under BG11; (B) FL3 histogram of cells under BG11; (C) FL3 histogram of cells under BG11 with 2.0% (v/v) ethanol; (D) FL3 histogram of cells under BG11 with 0.25% (v/v) butanol. [file 13068_2017_743_MOESM15_ESM.pdf]
