# Supplementary material for: Systematic and functional identification of small non-coding RNAs associated with exogenous biofuel stress in cyanobacterium Synechocystis sp. PCC 6803
Source: Biotechnol Biofuels. 2017 Mar 7;10:57. doi: 10.1186/s13068-017-0743-y (PMC5341163; doi:10.1186/s13068-017-0743-y)
Supplement: Supplementary file 12 — Additional file 12: Figure S7. RT-PCR verification for biofuel responsive sRNA mutant construction. The upper portion is the validation for the target sRNAs, while the lower part is an internal control of 16S rRNA. [file 13068_2017_743_MOESM12_ESM.pdf]

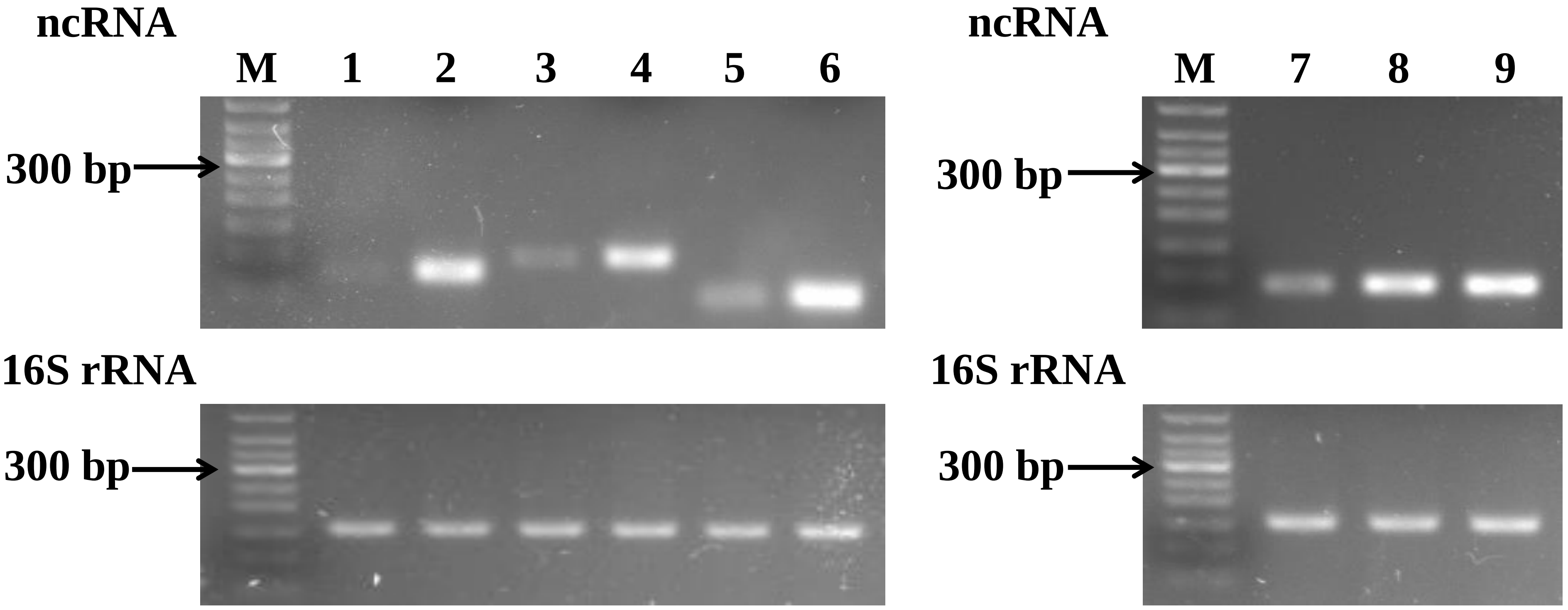

1: WT (Nc33)
2: pJA2-*nc33*- (Nc33)

3: WT (Nc65)
4: pJA2-*nc65*+ (Nc65)

5: WT (Nc85)
6: pJA2-*nc85*+ (Nc85)

7: WT (Nc117)
8: pJA2-*nc117*+ (Nc117)
9: pJA2-*nc117*- (Nc117)
