# Supplementary material for: Systematic and functional identification of small non-coding RNAs associated with exogenous biofuel stress in cyanobacterium Synechocystis sp. PCC 6803
Source: Biotechnol Biofuels. 2017 Mar 7;10:57. doi: 10.1186/s13068-017-0743-y (PMC5341163; doi:10.1186/s13068-017-0743-y)
Supplement: Supplementary file 13 — Additional file 13: Figure S8. Two-step RT-PCR validation for Nc117 overexpression and suppression strains. The upper portion is the target sRNA validation; the lower portion is 16S rRNA used as an internal control. [file 13068_2017_743_MOESM13_ESM.pdf]

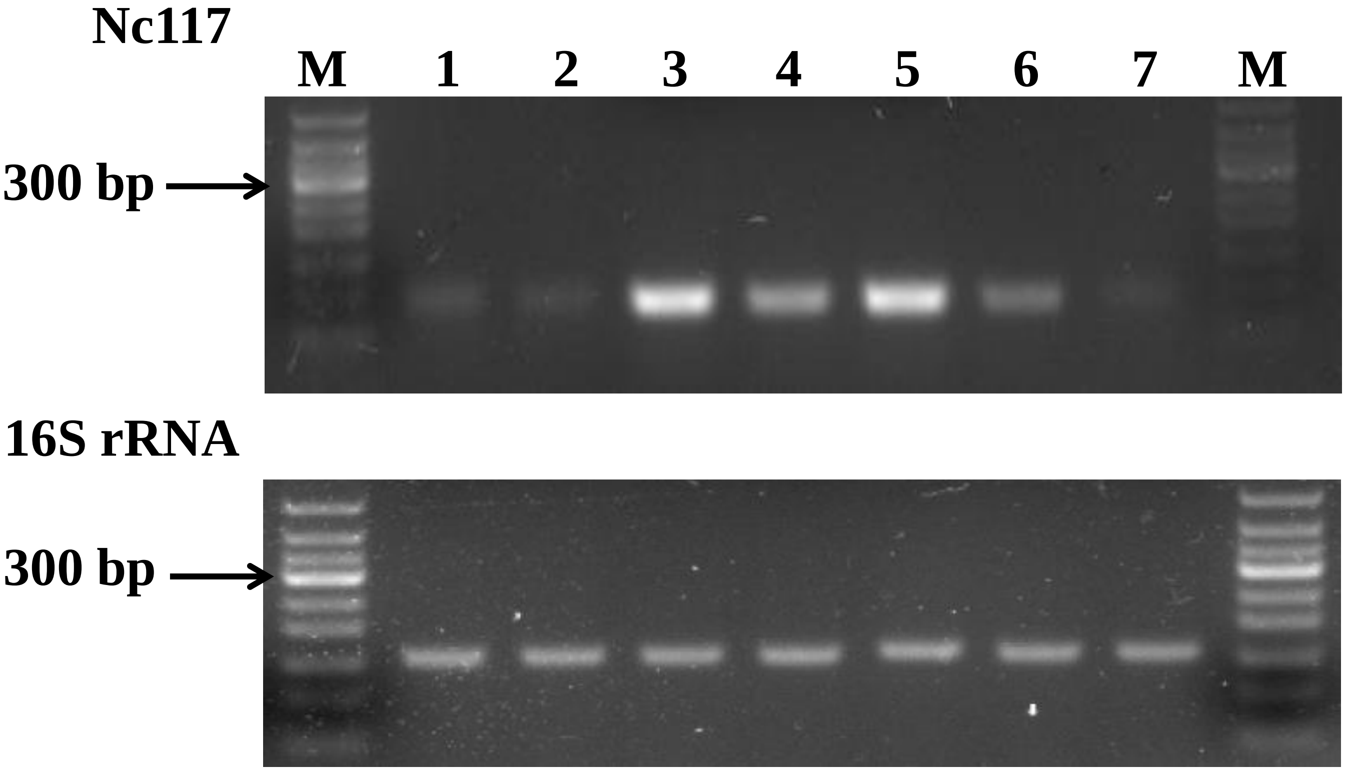

1: WT (Nc117-**)**
2: pJA2-*nc117*+ (Nc117-**)**
3: pJA2-*nc117*- (Nc117-**)**

4: WT (Nc117+)
5: pJA2-*nc117*+ (Nc117+)
6: pJA2-*nc117*- (Nc117+)

7: Background (Primer-free)
