# Supplementary material for: Systematic and functional identification of small non-coding RNAs associated with exogenous biofuel stress in cyanobacterium Synechocystis sp. PCC 6803
Source: Biotechnol Biofuels. 2017 Mar 7;10:57. doi: 10.1186/s13068-017-0743-y (PMC5341163; doi:10.1186/s13068-017-0743-y)
Supplement: Supplementary file 16 — Additional file 16: Figure S10. Genome-wide visualization of all sRNA mapping data in pSYSM of Synechocystis. Detailed description is the same as Additional file 2: Figure S1. [file 13068_2017_743_MOESM16_ESM.pdf]

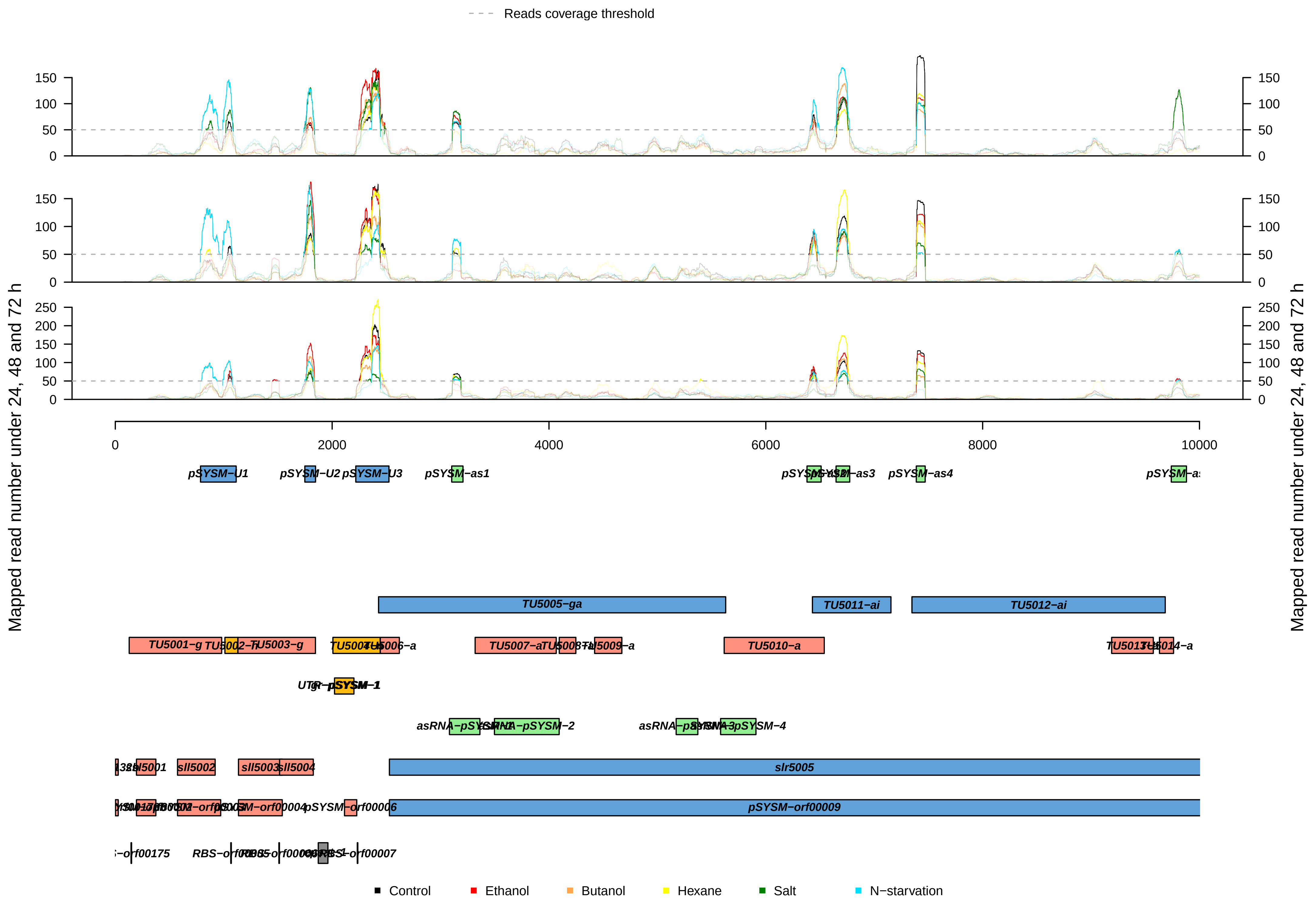

Reads coverage threshold
Mapped read number under 24, 48 and 72 h
pSYSM−U1
pSYSM−U2
pSYSM−U3
pSYSM−as1
pSYSM−as3
pSYSM−as4
TU5005−ga
TU5011−ai
TU5012−ai
TU5001−g
TU5003−g
TU5006−a
TU5007−a
TU5009−a
TU5010−a
TU5014−a
asRNA−pSYSM−2
asRNA−pSYSM−4
sll5001
sll5002
sll5003
sll5004
slr5005
pSYSM−orf00004
pSYSM−orf00006
pSYSM−orf00009
RBS−orf00175
RBS−orf00007
Control
Ethanol
Butanol
Hexane
Salt
N−starvation

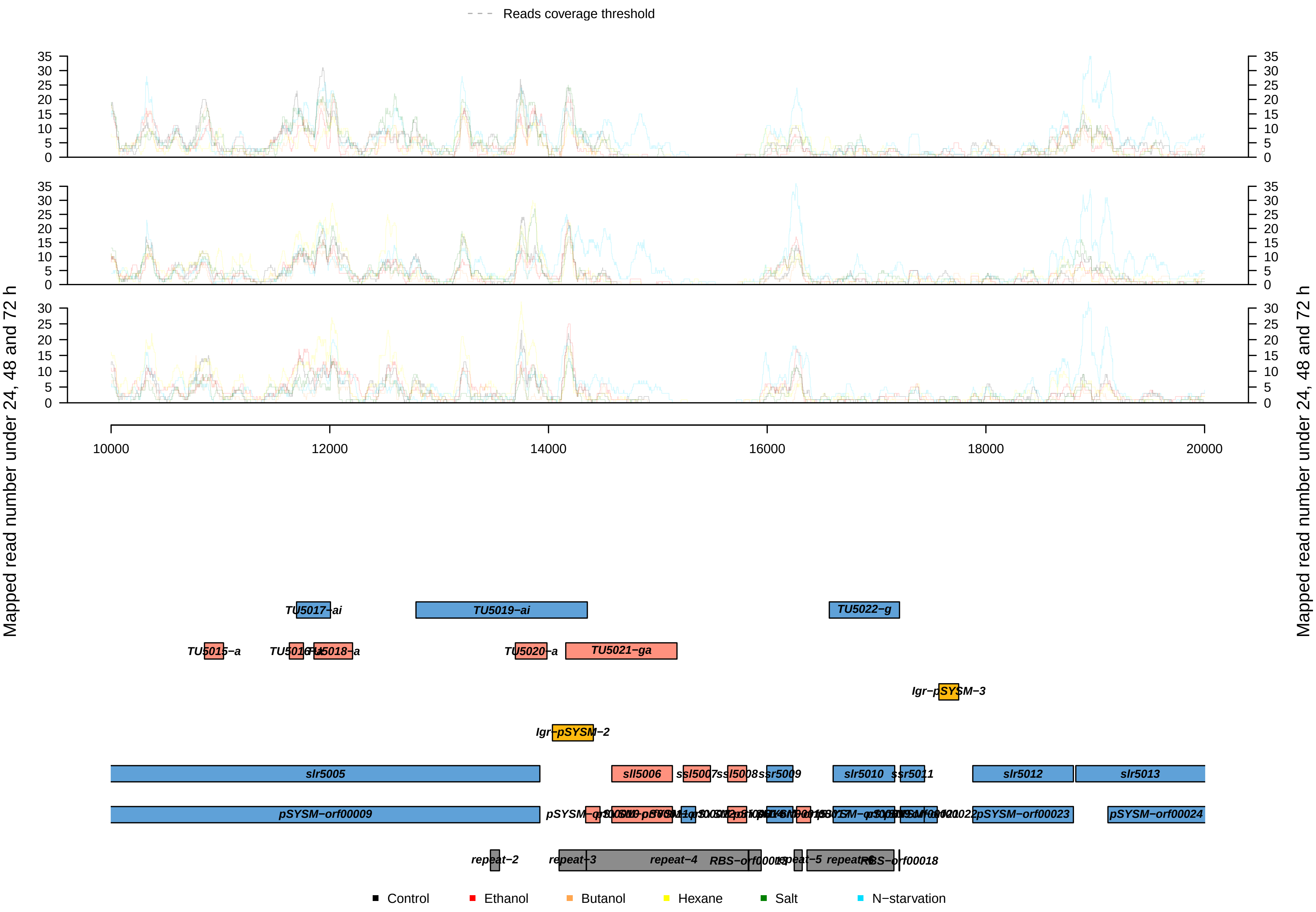

Reads coverage threshold
Mapped read number under 24, 48 and 72 h
10000
12000
14000
16000
18000
20000
TU5017−ai
TU5019−ai
TU5022−g
TU5015−a
TU5020−a
TU5021−ga
Igr−pSYSM−3
Igr−pSYSM−2
slr5005
sll5006
slr5010
slr5012
slr5013
pSYSM−orf00009
pSYSM−orf00023
pSYSM−orf00024
repeat−2
repeat−3
repeat−4
Control
Ethanol
Butanol
Hexane
Salt
N−starvation

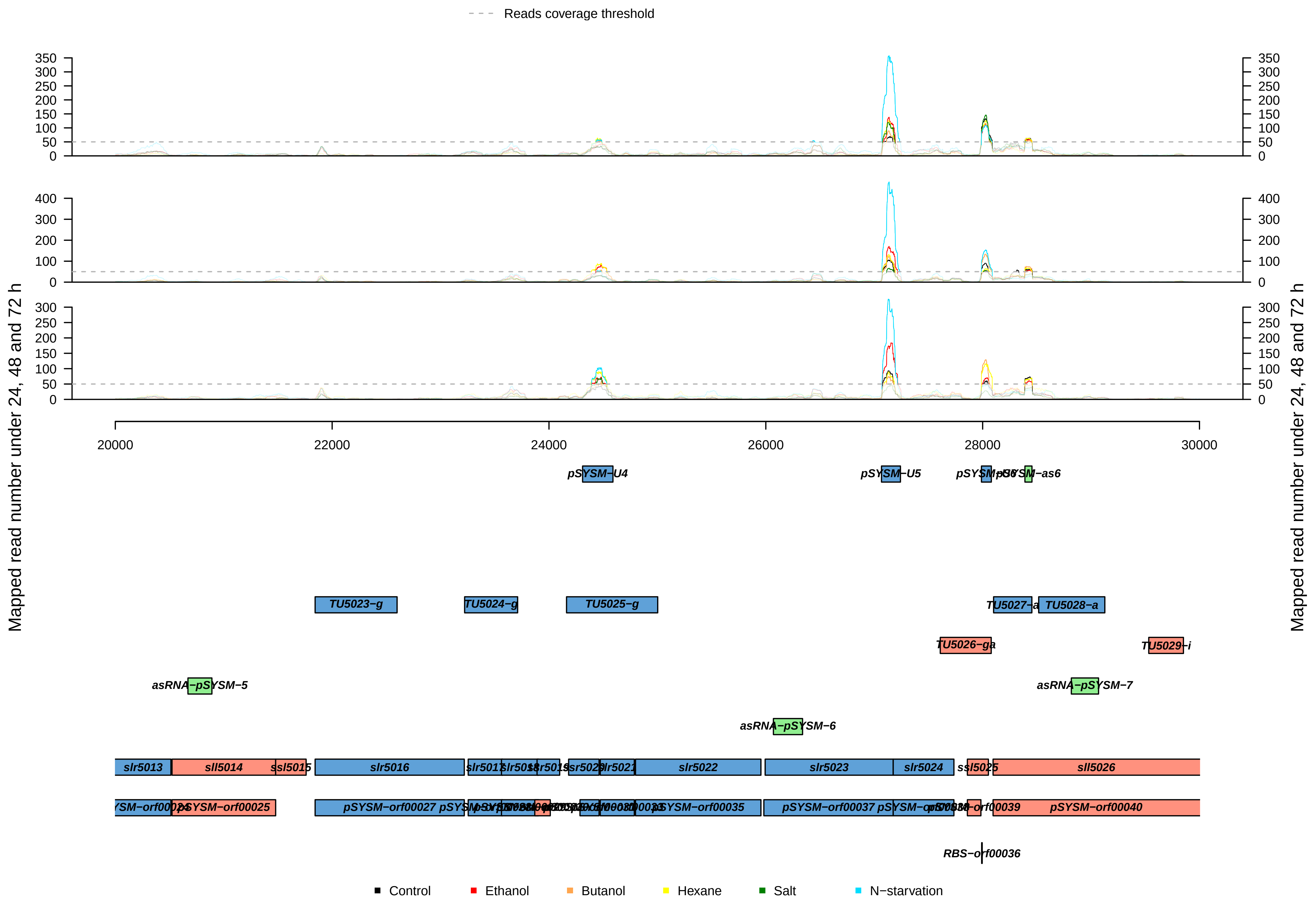
Reads coverage threshold
Mapped read number under 24, 48 and 72 h
pSYSM−U4
pSYSM−U5
pSYSM−as6
TU5023−g
TU5024−g
TU5025−g
TU5027−a
TU5028−a
TU5026−ga
TU5029−i
asRNA−pSYSM−5
asRNA−pSYSM−7
asRNA−pSYSM−6
slr5013
sll5014
ssl5015
slr5016
slr5022
slr5023
slr5024
ssl5025
sll5026
pSYSM−orf00025
pSYSM−orf00027
pSYSM−orf00035
pSYSM−orf00037
pSYSM−orf00039
pSYSM−orf00040
RBS−orf00036
Control
Ethanol
Butanol
Hexane
Salt
N−starvation

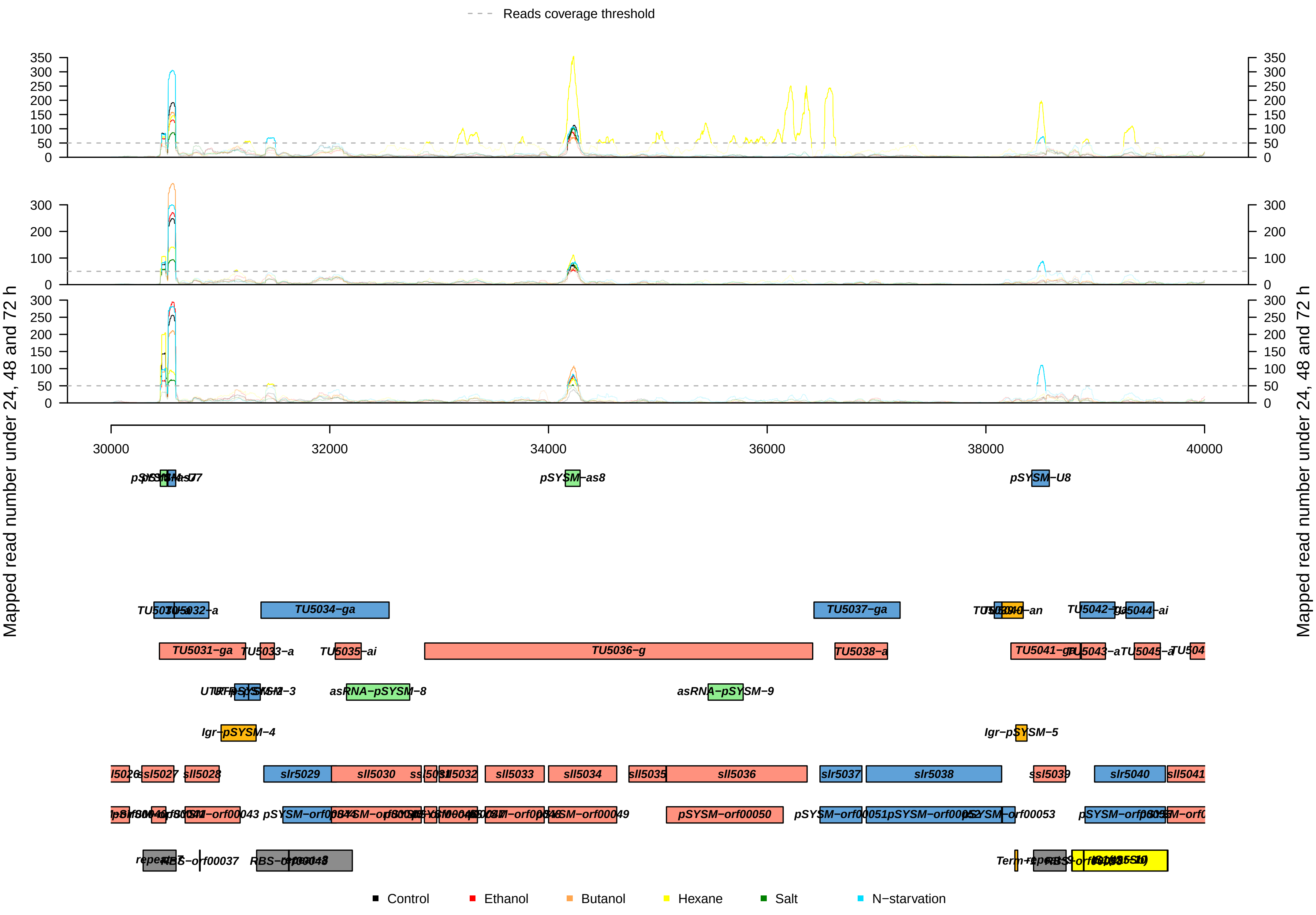

Reads coverage threshold
Mapped read number under 24, 48 and 72 h
30000
32000
34000
36000
38000
40000
pSYSM−as8
pSYSM−U8
TU5034−ga
TU5037−ga
TU5031−ga
TU5036−g
TU5038−a
asRNA−pSYSM−8
asRNA−pSYSM−9
Igr−pSYSM−4
Igr−pSYSM−5
ssl5027
sll5028
slr5029
sll5030
sll5032
sll5033
sll5034
sll5035
sll5036
slr5037
slr5038
ssl5039
slr5040
sll5041
pSYSM−orf00043
pSYSM−orf00049
pSYSM−orf00050
pSYSM−orf00053
Control
Ethanol
Butanol
Hexane
Salt
N−starvation

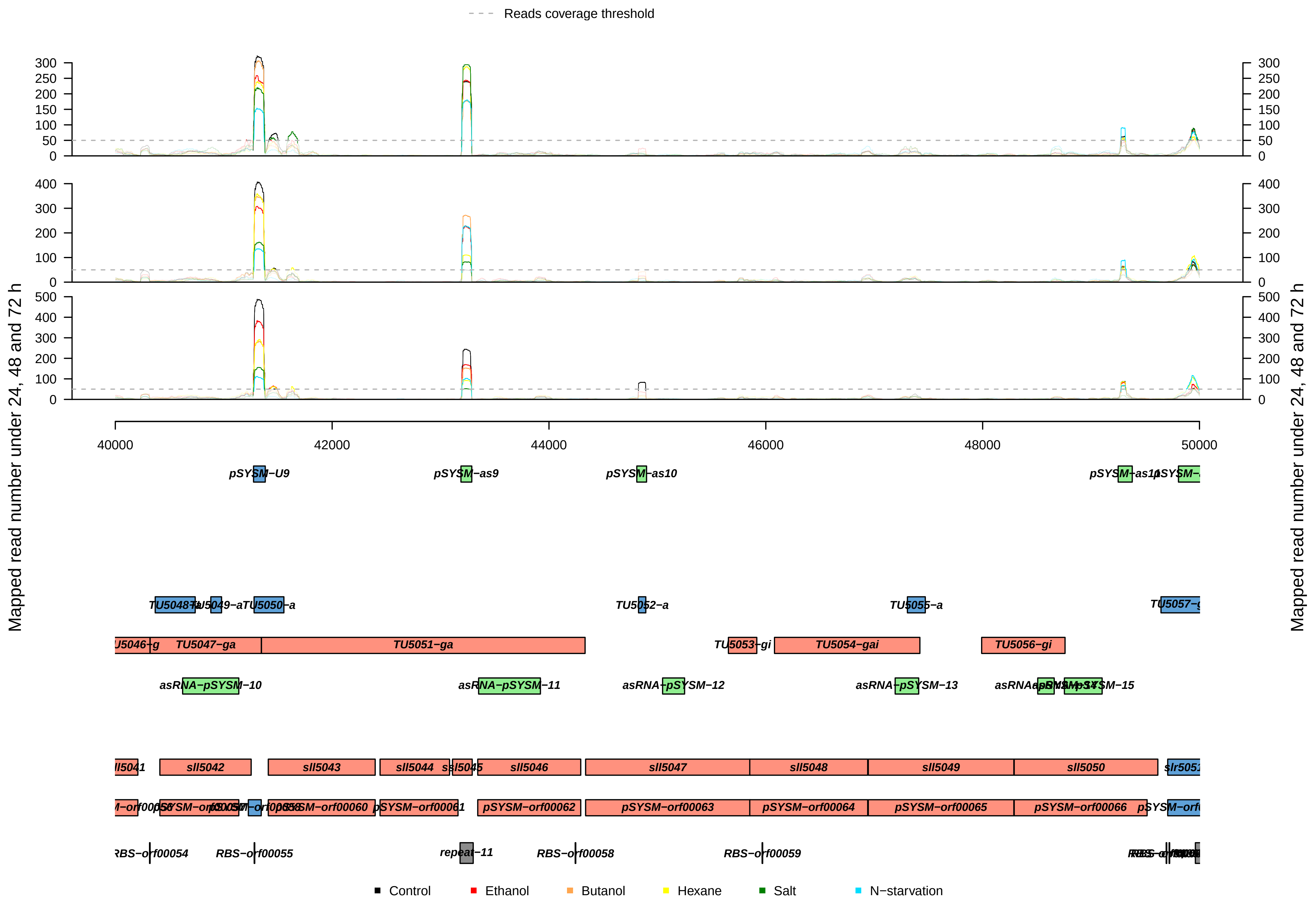
Reads coverage threshold
Mapped read number under 24, 48 and 72 h
40000
42000
44000
46000
48000
50000
pSYSM−U9
pSYSM−as9
pSYSM−as10
TU5050−a
TU5052−a
TU5047−ga
TU5051−ga
TU5054−gai
TU5056−gi
asRNA−pSYSM−10
asRNA−pSYSM−11
asRNA−pSYSM−12
asRNA−pSYSM−13
sll5042
sll5043
sll5044
sll5046
sll5047
sll5048
sll5049
sll5050
pSYSM−orf00060
pSYSM−orf00061
pSYSM−orf00062
pSYSM−orf00063
pSYSM−orf00064
pSYSM−orf00065
pSYSM−orf00066
RBS−orf00055
repeat−11
RBS−orf00058
RBS−orf00059
Control
Ethanol
Butanol
Hexane
Salt
N−starvation

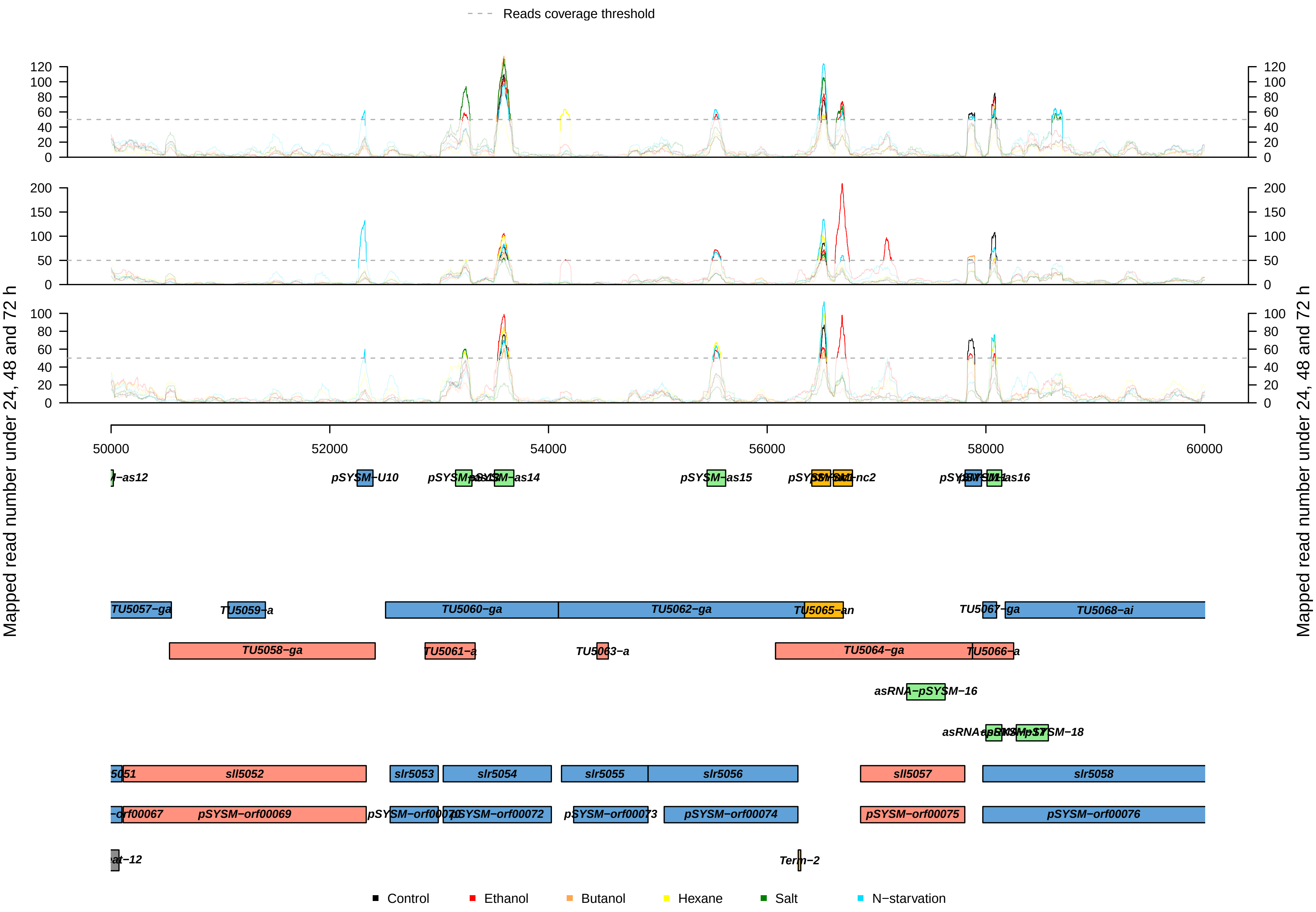

Reads coverage threshold
Mapped read number under 24, 48 and 72 h
50000
52000
54000
56000
58000
60000
pSYSM−U10
pSYSM−as14
pSYSM−as15
pSYSM−as16
TU5057−ga
TU5059−a
TU5060−ga
TU5062−ga
TU5065−an
TU5068−ai
TU5058−ga
TU5061−a
TU5063−a
TU5064−ga
TU5066−a
asRNA−pSYSM−16
pSYSM−18
sll5052
slr5053
slr5054
slr5055
slr5056
sll5057
slr5058
pSYSM−orf00069
pSYSM−orf00072
pSYSM−orf00073
pSYSM−orf00074
pSYSM−orf00075
pSYSM−orf00076
Term−2
Control
Ethanol
Butanol
Hexane
Salt
N−starvation

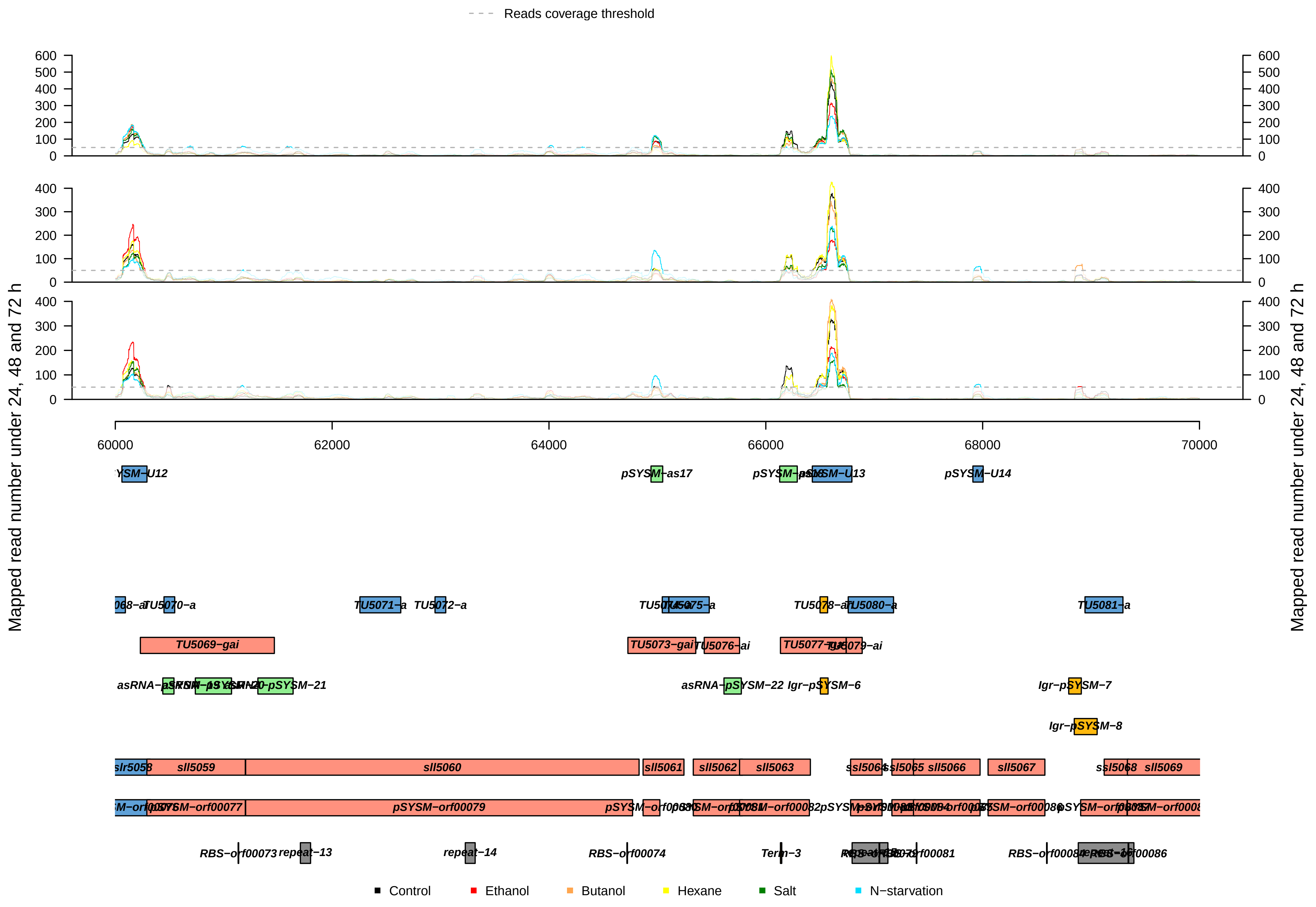

Reads coverage threshold
Mapped read number under 24, 48 and 72 h
60000
62000
64000
66000
68000
70000
pSYSM-as17
pSYSM-U14
TU5069-gai
TU5073-gai
asRNA-pSYSM-22
Igr-pSYSM-6
Igr-pSYSM-7
Igr-pSYSM-8
sll5059
sll5060
sll5061
sll5062
sll5063
sll5066
sll5067
sll5069
pSYSM-orf00077
pSYSM-orf00079
repeat-13
repeat-14
RBS-orf00073
RBS-orf00074
Term-3
Control
Ethanol
Butanol
Hexane
Salt
N-starvation

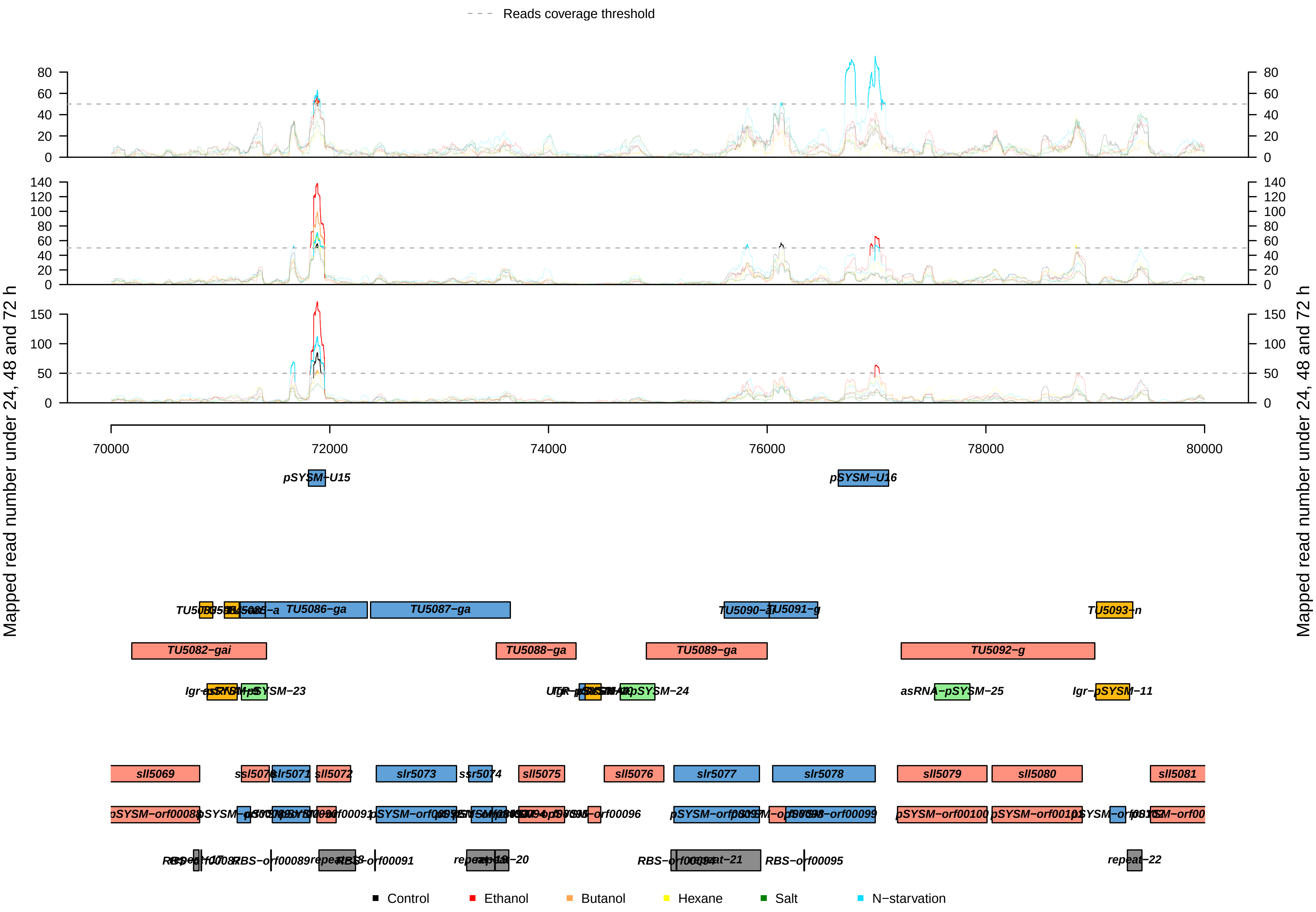

Reads coverage threshold
Mapped read number under 24, 48 and 72 h
80
60
40
20
0
140
120
100
150
50
70000
72000
74000
76000
78000
80000
pSYSM−U15
pSYSM−U16
TU5086−ga
TU5087−ga
TU5093−n
TU5082−gai
TU5088−ga
TU5089−ga
TU5092−g
pSYSM−24
asRNA−pSYSM−25
Igr−pSYSM−11
sll5069
slr5071
sll5072
slr5073
ssr5074
sll5075
sll5076
slr5077
slr5078
sll5079
sll5080
sll5081
pSYSM−orf00096
pSYSM−orf00100
pSYSM−orf00101
RBS−orf00089
RBS−orf00091
RBS−orf00095
repeat−22
Control
Ethanol
Butanol
Hexane
Salt
N−starvation

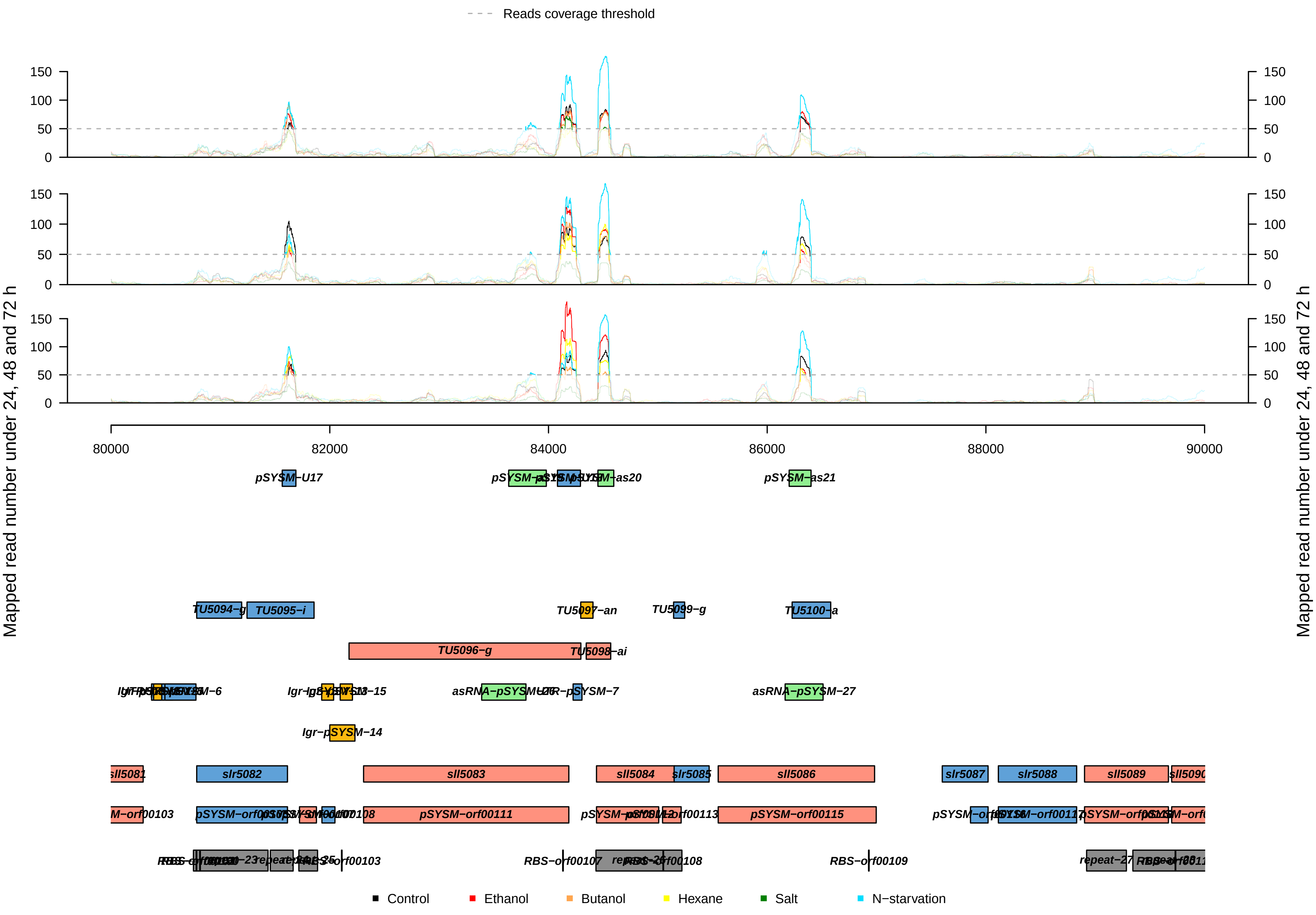
Reads coverage threshold
Mapped read number under 24, 48 and 72 h
150
100
50
0
80000
82000
84000
86000
88000
90000
pSYSM−U17
pSYSM−as21
TU5094−g
TU5095−i
TU5097−an
TU5099−g
TU5100−a
TU5096−g
TU5098−ai
asRNA−pSYSM−27
Igr−pSYSM−14
sll5081
slr5082
sll5083
sll5084
slr5085
sll5086
slr5087
slr5088
sll5089
pSYSM−orf00111
pSYSM−orf00115
RBS−orf00103
RBS−orf00107
RBS−orf00108
RBS−orf00109
Control
Ethanol
Butanol
Hexane
Salt
N−starvation

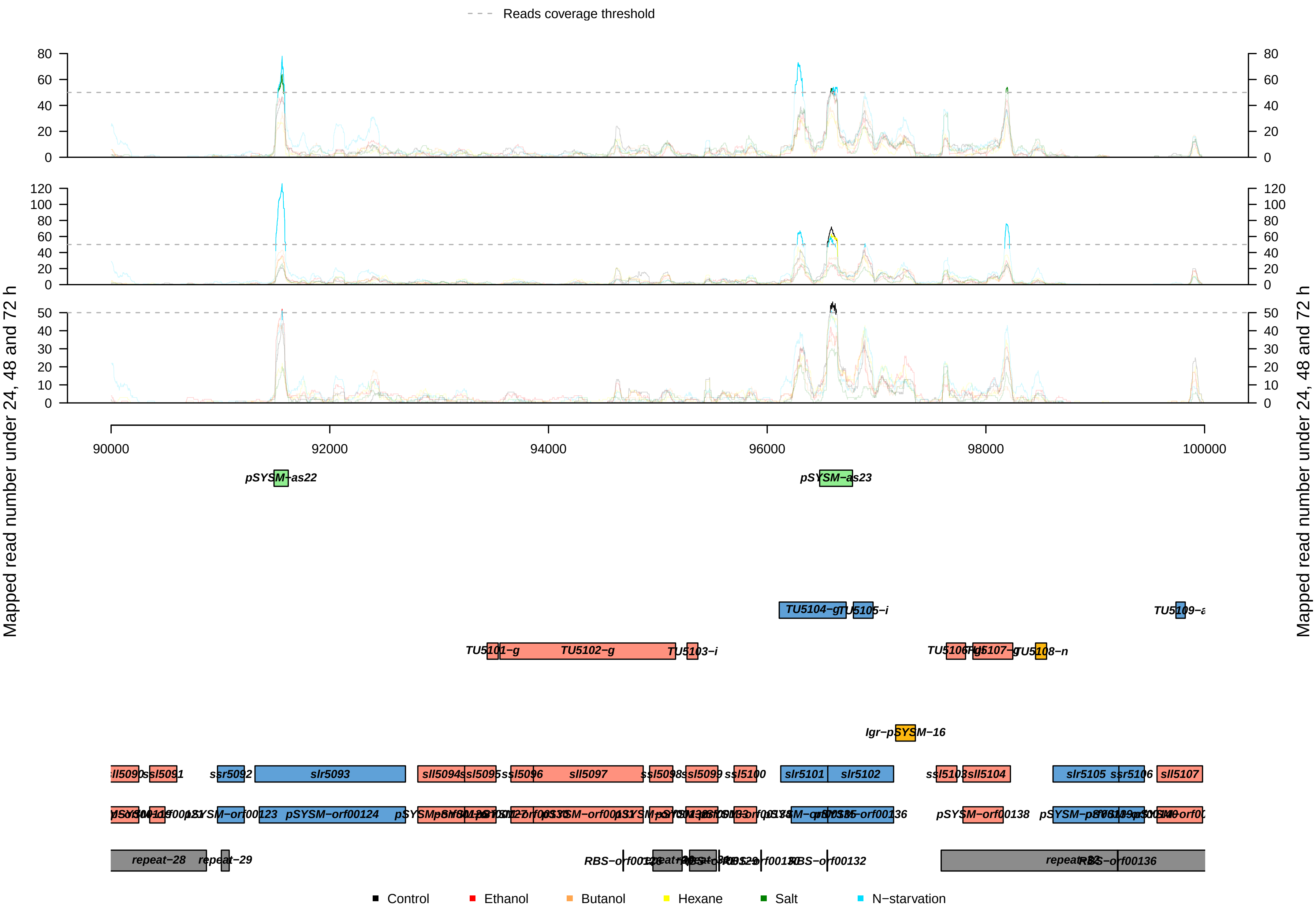

Reads coverage threshold
Mapped read number under 24, 48 and 72 h
90000
92000
94000
96000
98000
100000
pSYSM−as22
pSYSM−as23
TU5104−g
TU5102−g
TU5103−i
TU5108−n
Igr−pSYSM−16
ssl5091
ssr5092
slr5093
sll5097
ssl5099
ssl5100
slr5101
slr5102
sll5104
slr5105
ssr5106
sll5107
pSYSM−orf00124
pSYSM−orf00138
repeat−28
repeat−29
RBS−orf00132
Control
Ethanol
Butanol
Hexane
Salt
N−starvation

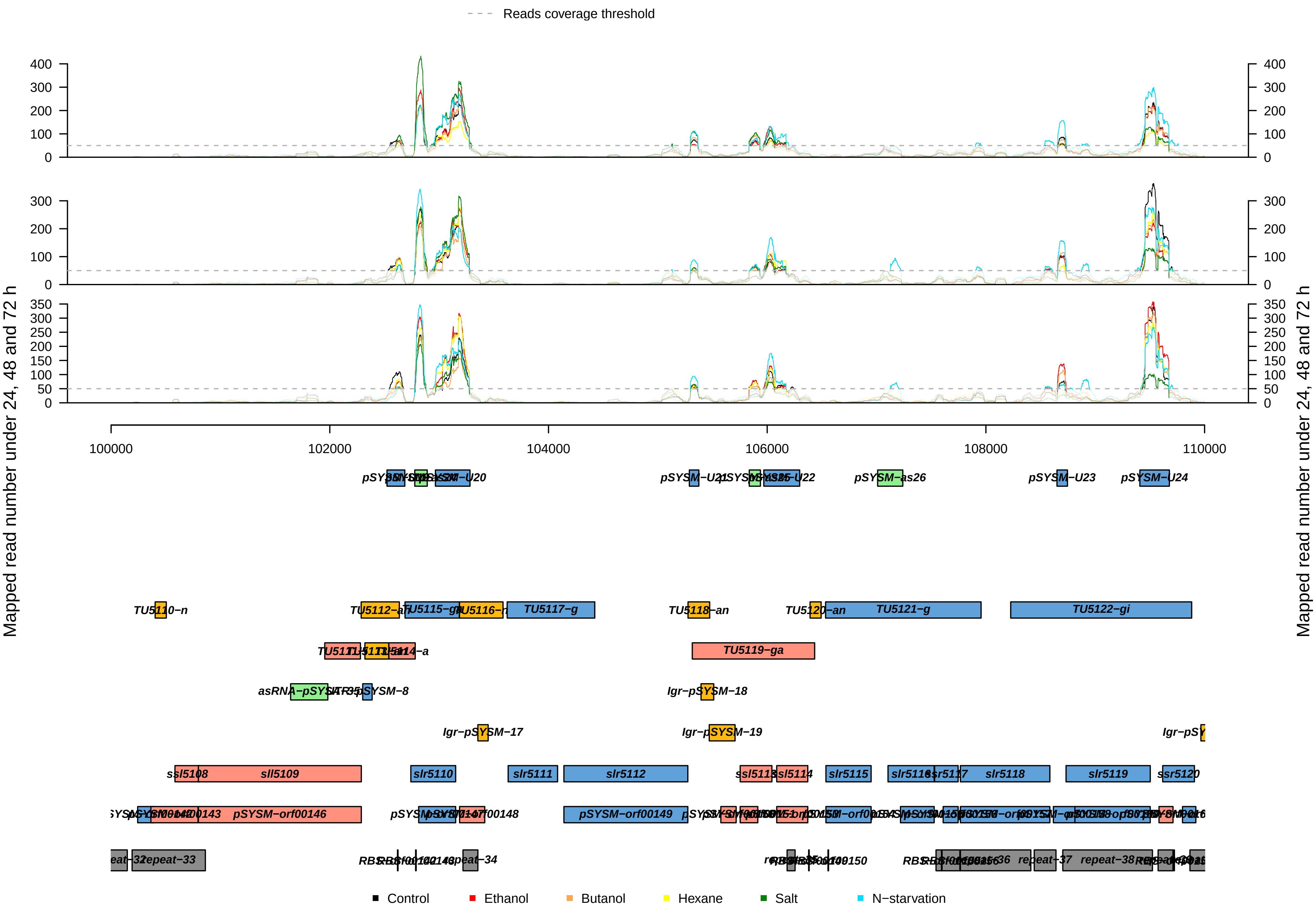

Reads coverage threshold
Mapped read number under 24, 48 and 72 h
100000
102000
104000
106000
108000
110000
pSYSM-U20
pSYSM-U22
pSYSM-as26
pSYSM-U23
pSYSM-U24
TU5110-n
TU5117-g
TU5121-g
TU5122-gi
TU5119-ga
Igr-pSYSM-17
Igr-pSYSM-18
Igr-pSYSM-19
ssl5108
sll5109
slr5110
slr5111
slr5112
slr5115
slr5118
slr5119
ssr5120
pSYSM-orf00146
pSYSM-orf00149
repeat-33
repeat-34
repeat-37
Control
Ethanol
Butanol
Hexane
Salt
N-starvation

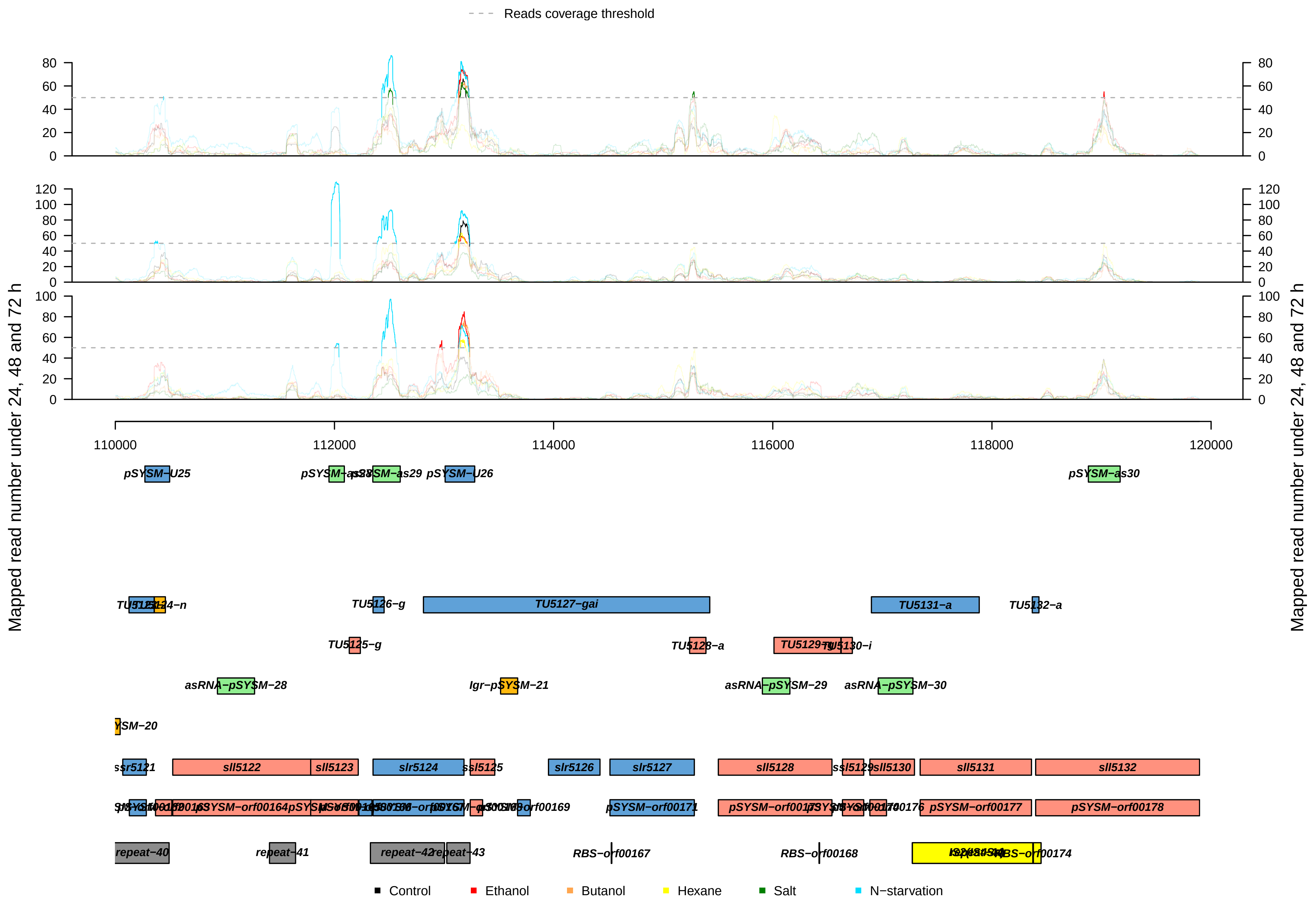

Reads coverage threshold
Mapped read number under 24, 48 and 72 h
110000
112000
114000
116000
118000
120000
pSYSM−U25
pSYSM−U26
pSYSM−as30
TU5126−g
TU5127−gai
TU5131−a
TU5132−a
TU5125−g
TU5128−a
asRNA−pSYSM−28
Igr−pSYSM−21
asRNA−pSYSM−29
asRNA−pSYSM−30
sll5122
sll5123
slr5124
ssl5125
slr5126
slr5127
sll5128
sll5130
sll5131
sll5132
pSYSM−orf00164
pSYSM−orf00169
pSYSM−orf00171
pSYSM−orf00177
pSYSM−orf00178
repeat−40
repeat−41
repeat−42
repeat−43
RBS−orf00167
RBS−orf00168
Control
Ethanol
Butanol
Hexane
Salt
N−starvation
