# Supplementary material for: Systematic and functional identification of small non-coding RNAs associated with exogenous biofuel stress in cyanobacterium Synechocystis sp. PCC 6803
Source: Biotechnol Biofuels. 2017 Mar 7;10:57. doi: 10.1186/s13068-017-0743-y (PMC5341163; doi:10.1186/s13068-017-0743-y)
Supplement: Supplementary file 17 — Additional file 17: Figure S11. Genome-wide visualization of all sRNA mapping data in pSYSA of Synechocystis. Detailed description is the same as Additional file 2: Figure S1. [file 13068_2017_743_MOESM17_ESM.pdf]

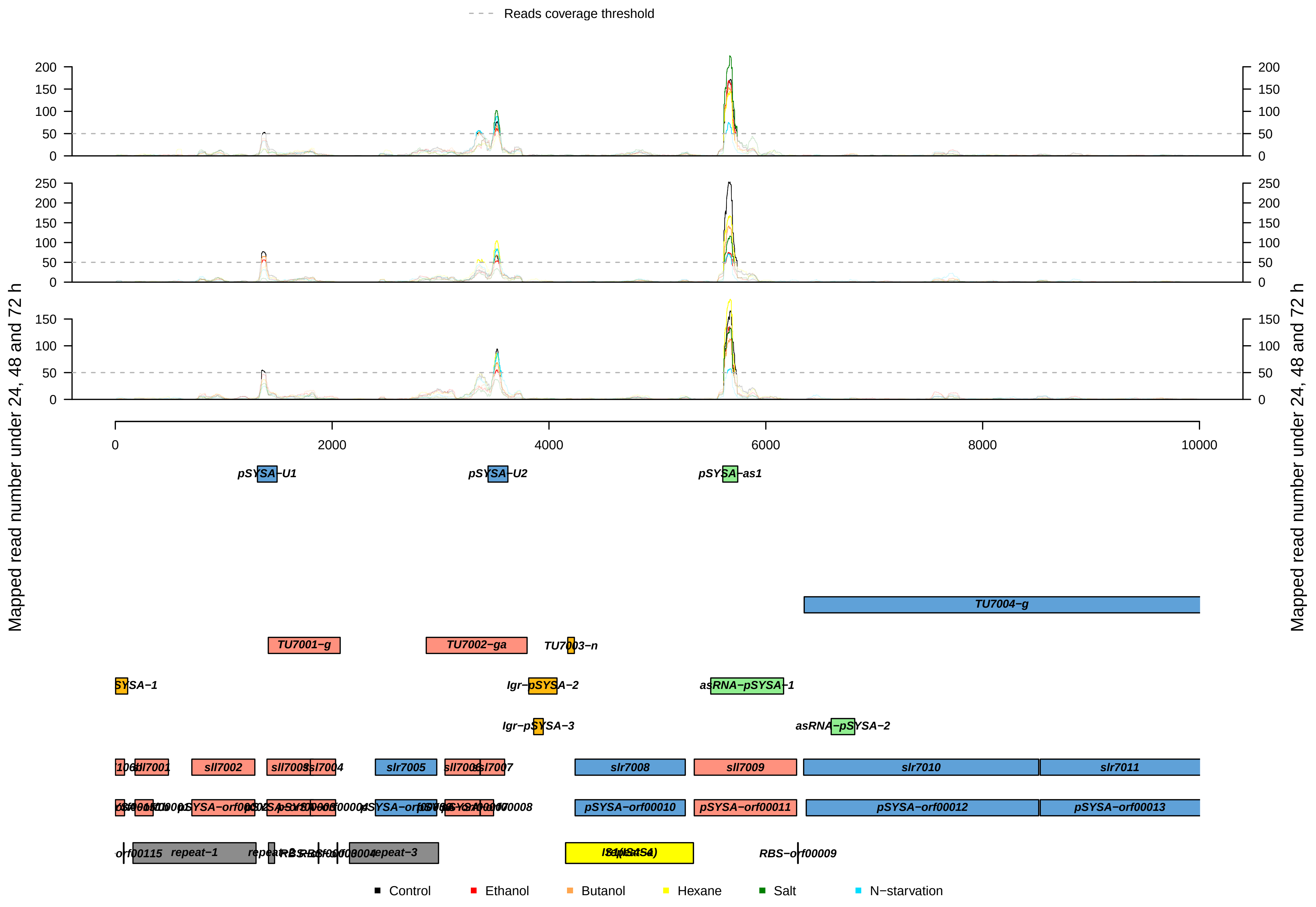

Reads coverage threshold
Mapped read number under 24, 48 and 72 h
0
2000
4000
6000
8000
10000
pSYSA−U1
pSYSA−U2
pSYSA−as1
TU7004−g
TU7001−g
TU7002−ga
TU7003−n
Igr−pSYSA−2
asRNA−pSYSA−1
Igr−pSYSA−3
asRNA−pSYSA−2
sll7001
sll7002
slr7005
slr7008
sll7009
slr7010
slr7011
pSYSA−orf00010
pSYSA−orf00011
pSYSA−orf00012
pSYSA−orf00013
repeat−1
repeat−3
RBS−orf00009
Control
Ethanol
Butanol
Hexane
Salt
N−starvation

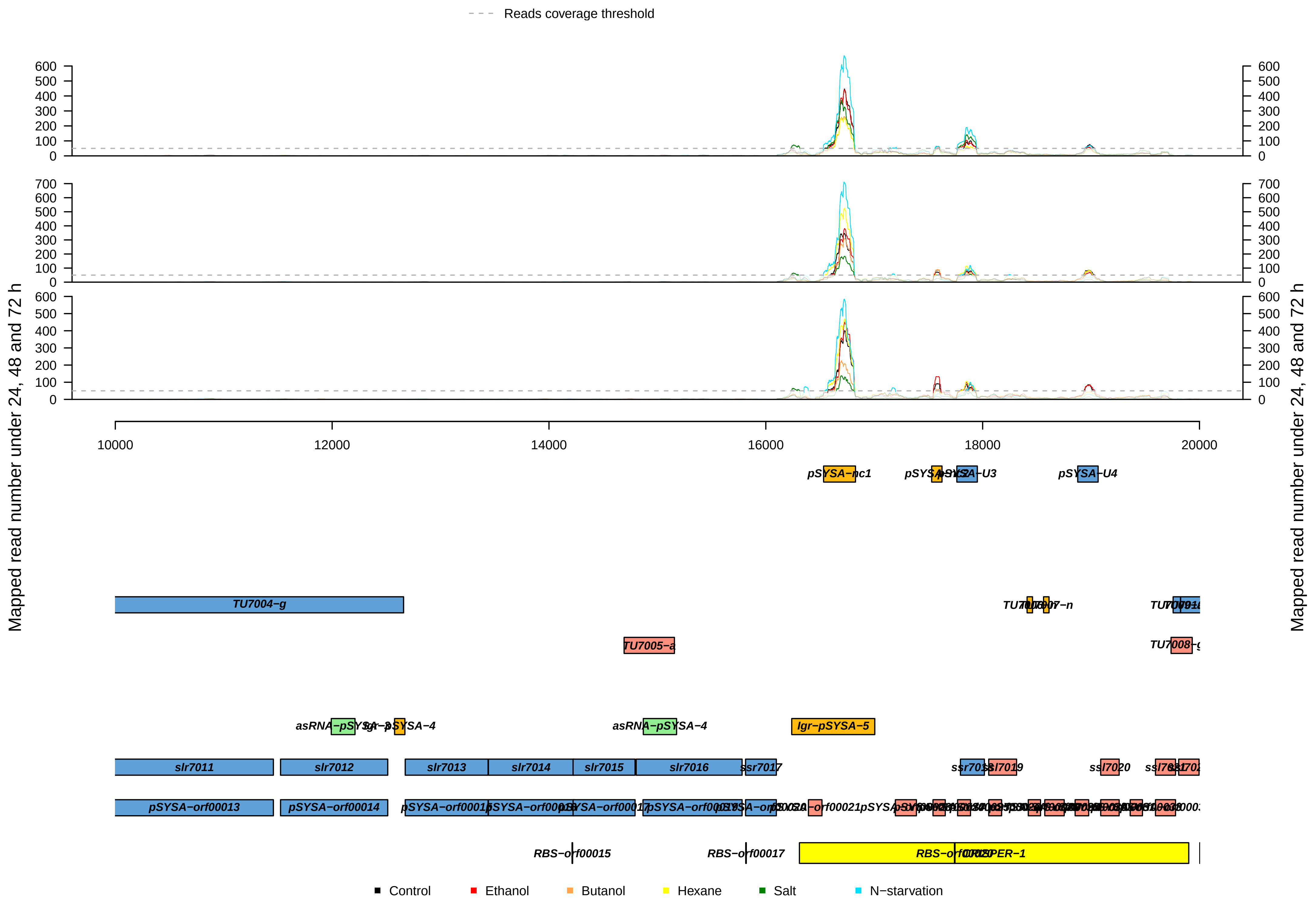

Reads coverage threshold
Mapped read number under 24, 48 and 72 h
0
100
200
300
400
500
600
700
10000
12000
14000
16000
18000
20000
pSYSA−nc1
pSYSA−U3
pSYSA−U4
TU7004−g
TU7005−a
asRNA−pSYSA−4
Igr−pSYSA−5
slr7011
slr7012
slr7013
slr7014
slr7015
slr7016
ssr7017
ssl7020
pSYSA−orf00013
pSYSA−orf00014
RBS−orf00015
RBS−orf00017
Control
Ethanol
Butanol
Hexane
Salt
N−starvation

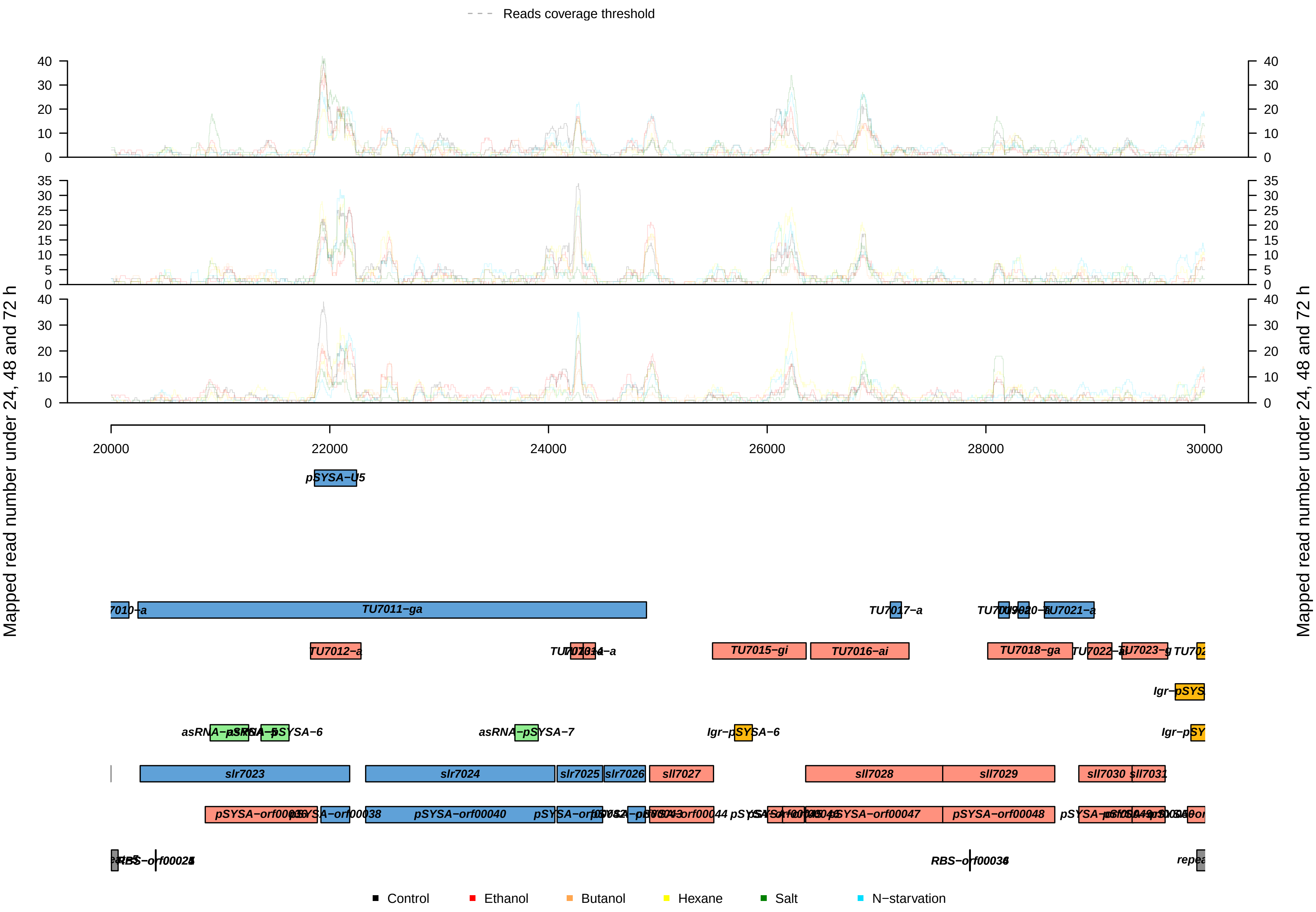

Reads coverage threshold
Mapped read number under 24, 48 and 72 h
20000
22000
24000
26000
28000
30000
pSYSA−U5
TU7011−ga
TU7017−a
TU7021−a
TU7012−a
TU7015−gi
TU7016−ai
TU7018−ga
asRNA−pSYSA−6
asRNA−pSYSA−7
Igr−pSYSA−6
slr7023
slr7024
slr7025
slr7026
sll7027
sll7028
sll7029
sll7030
sll7031
pSYSA−orf00040
pSYSA−orf00047
pSYSA−orf00048
Control
Ethanol
Butanol
Hexane
Salt
N−starvation

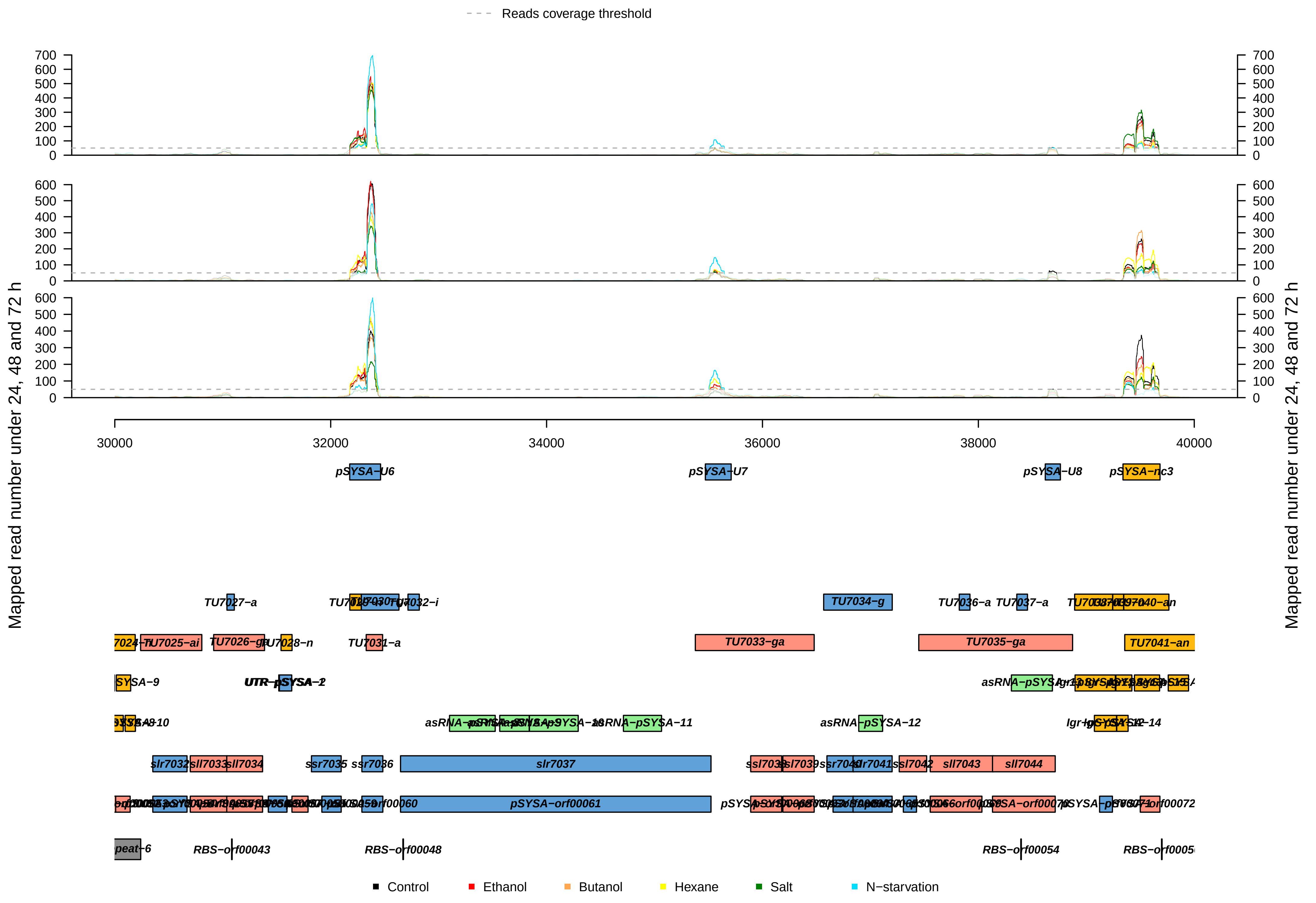

Reads coverage threshold
Mapped read number under 24, 48 and 72 h
30000
32000
34000
36000
38000
40000
pSYSA−U6
pSYSA−U7
pSYSA−U8
pSYSA−nc3
TU7027−a
TU7032−i
TU7034−g
TU7036−a
TU7037−a
TU7025−ai
TU7031−a
TU7033−ga
TU7035−ga
TU7041−an
asRNA−pSYSA−11
asRNA−pSYSA−12
slr7032
sll7033
sll7034
ssr7035
ssr7036
slr7037
sll7043
sll7044
pSYSA−orf00060
pSYSA−orf00061
RBS−orf00043
RBS−orf00048
RBS−orf00054
Control
Ethanol
Butanol
Hexane
Salt
N−starvation

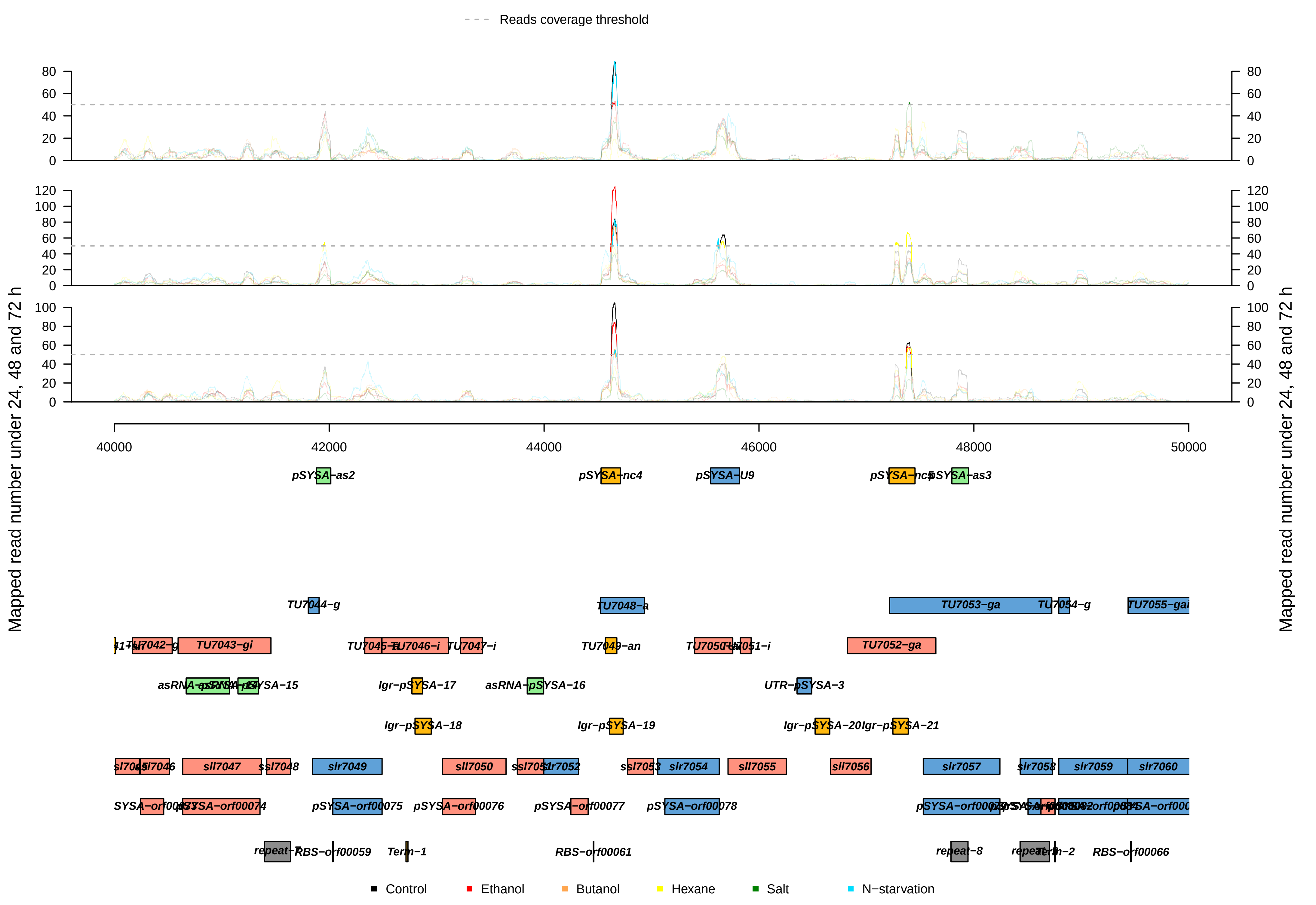

Reads coverage threshold
Mapped read number under 24, 48 and 72 h
40000
42000
44000
46000
48000
50000
pSYSA−as2
pSYSA−nc4
pSYSA−U9
pSYSA−nc5
pSYSA−as3
TU7044−g
TU7048−a
TU7053−ga
TU7054−g
TU7055−gai
TU7042−g
TU7043−gi
TU7046−i
TU7047−i
TU7049−an
TU7052−ga
asRNA−pSYSA−15
Igr−pSYSA−17
asRNA−pSYSA−16
UTR−pSYSA−3
Igr−pSYSA−18
Igr−pSYSA−19
Igr−pSYSA−20
Igr−pSYSA−21
ssl7046
sll7047
ssl7048
slr7049
sll7050
slr7052
slr7054
sll7055
sll7056
slr7057
slr7059
slr7060
pSYSA−orf00074
pSYSA−orf00075
pSYSA−orf00076
pSYSA−orf00077
pSYSA−orf00078
RBS−orf00059
Term−1
RBS−orf00061
repeat−8
Term−2
RBS−orf00066
Control
Ethanol
Butanol
Hexane
Salt
N−starvation

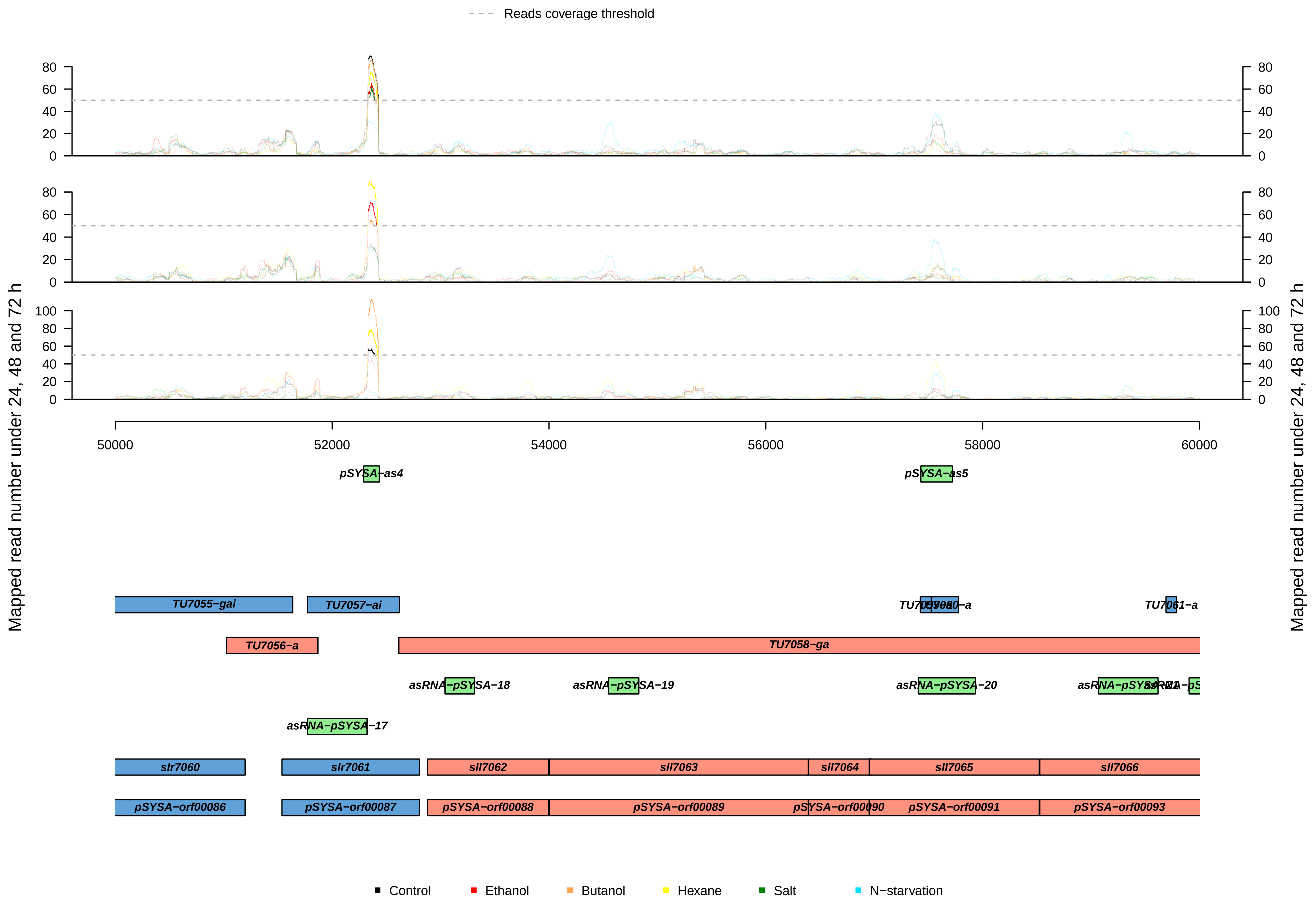

Reads coverage threshold
Mapped read number under 24, 48 and 72 h
50000
52000
54000
56000
58000
60000
pSYSA-as4
pSYSA-as5
TU7055-gai
TU7057-ai
TU7061-a
TU7056-a
TU7058-ga
asRNA-pSYSA-18
asRNA-pSYSA-19
asRNA-pSYSA-20
asRNA-pSYSA-17
slr7060
slr7061
sll7062
sll7063
sll7064
sll7065
sll7066
pSYSA-orf00086
pSYSA-orf00087
pSYSA-orf00088
pSYSA-orf00089
pSYSA-orf00090
pSYSA-orf00091
pSYSA-orf00093
Control
Ethanol
Butanol
Hexane
Salt
N-starvation

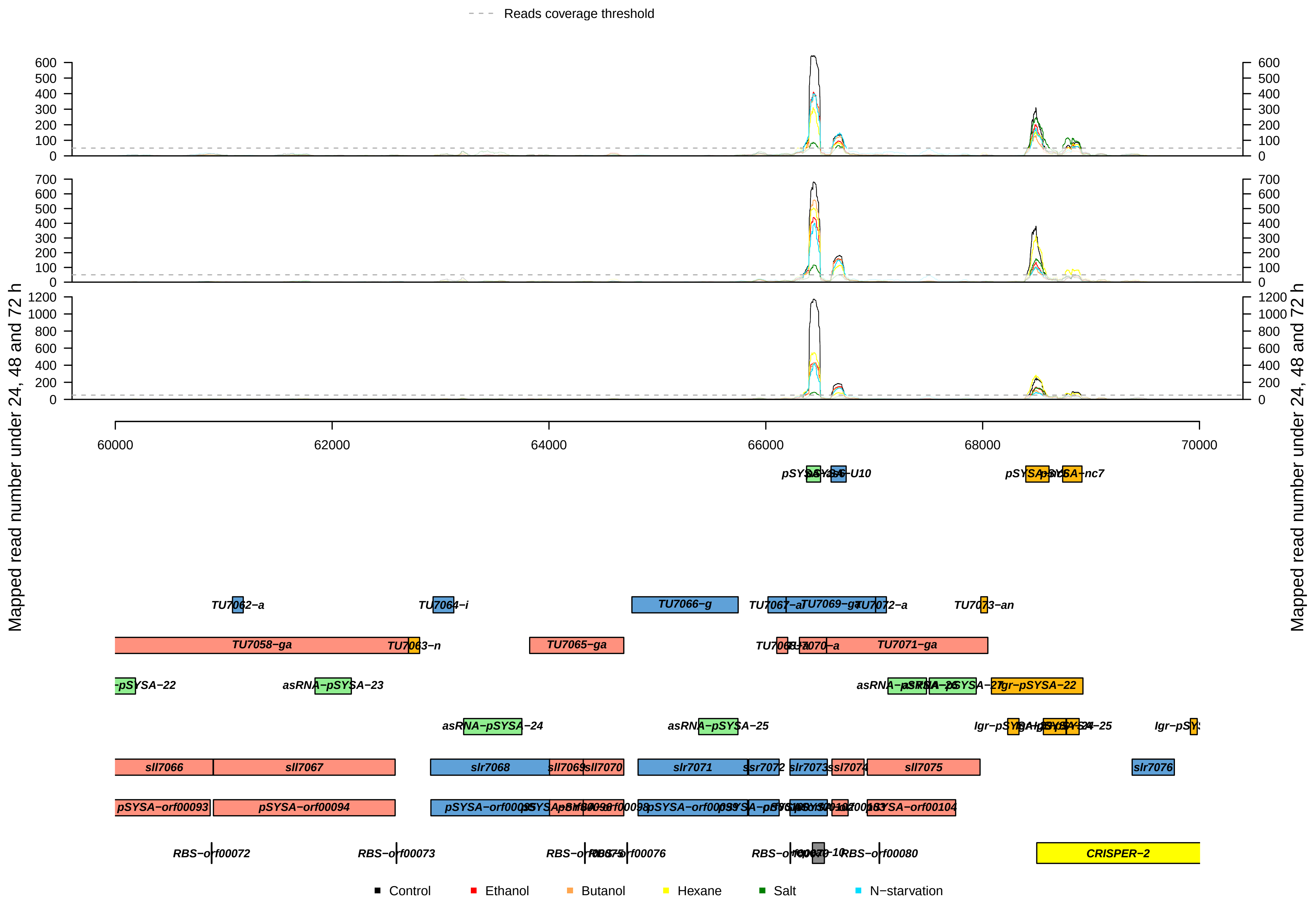

Reads coverage threshold
Mapped read number under 24, 48 and 72 h
60000
62000
64000
66000
68000
70000
TU7062−a
TU7064−i
TU7066−g
TU7073−an
TU7058−ga
TU7063−n
TU7065−ga
TU7071−ga
asRNA−pSYSA−23
asRNA−pSYSA−24
asRNA−pSYSA−25
Igr−pSYSA−22
sll7066
sll7067
slr7068
sll7069
sll7070
slr7071
ssr7072
slr7073
ssl7074
sll7075
slr7076
pSYSA−orf00093
pSYSA−orf00094
RBS−orf00072
RBS−orf00073
RBS−orf00080
CRISPER−2
Control
Ethanol
Butanol
Hexane
Salt
N−starvation

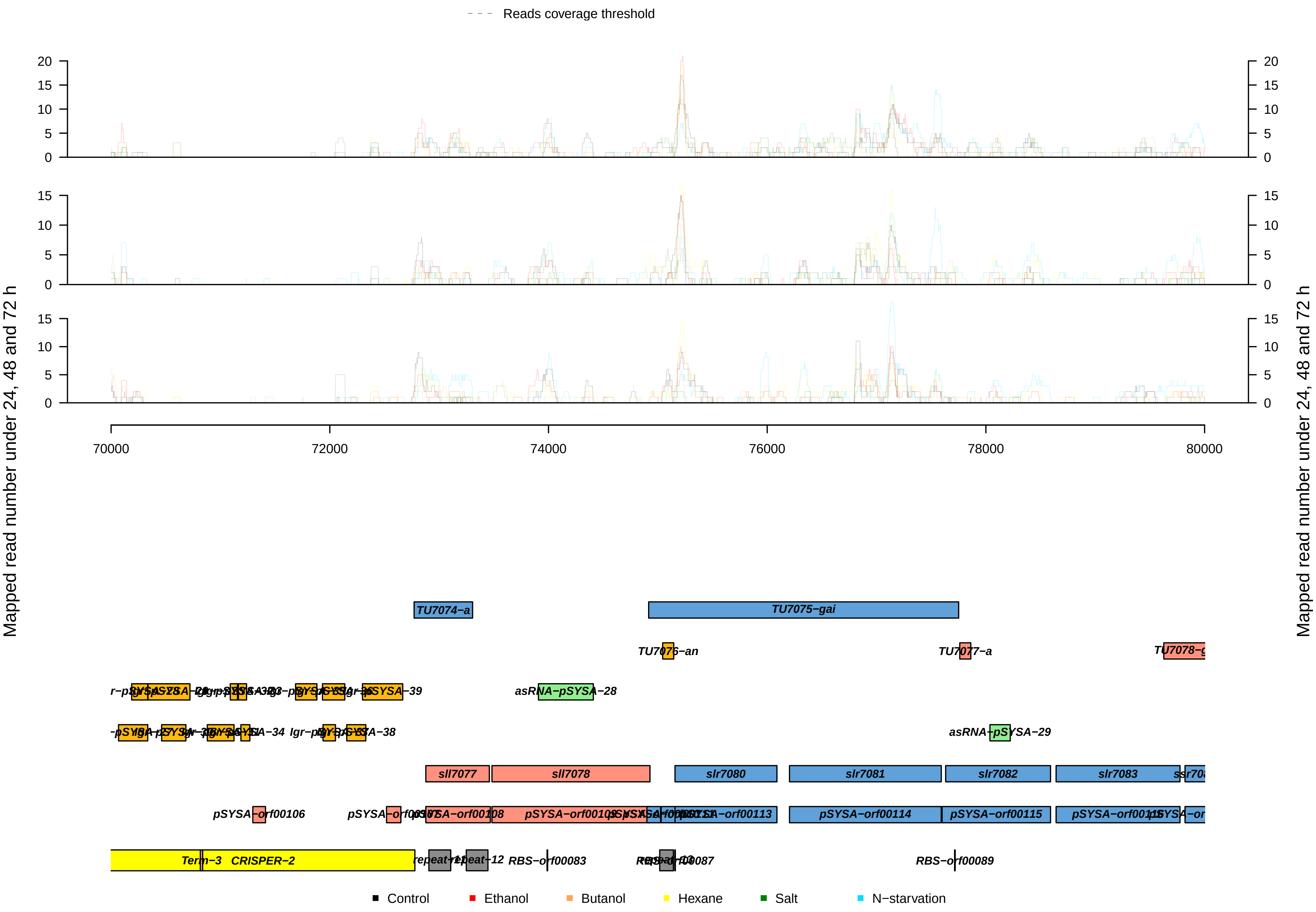

Reads coverage threshold
Mapped read number under 24, 48 and 72 h
70000
72000
74000
76000
78000
80000
TU7074−a
TU7075−gai
asRNA−pSYSA−28
asRNA−pSYSA−29
sll7077
sll7078
slr7080
slr7081
slr7082
slr7083
pSYSA−orf00106
pSYSA−orf00114
pSYSA−orf00115
CRISPER−2
RBS−orf00083
RBS−orf00089
Control
Ethanol
Butanol
Hexane
Salt
N−starvation

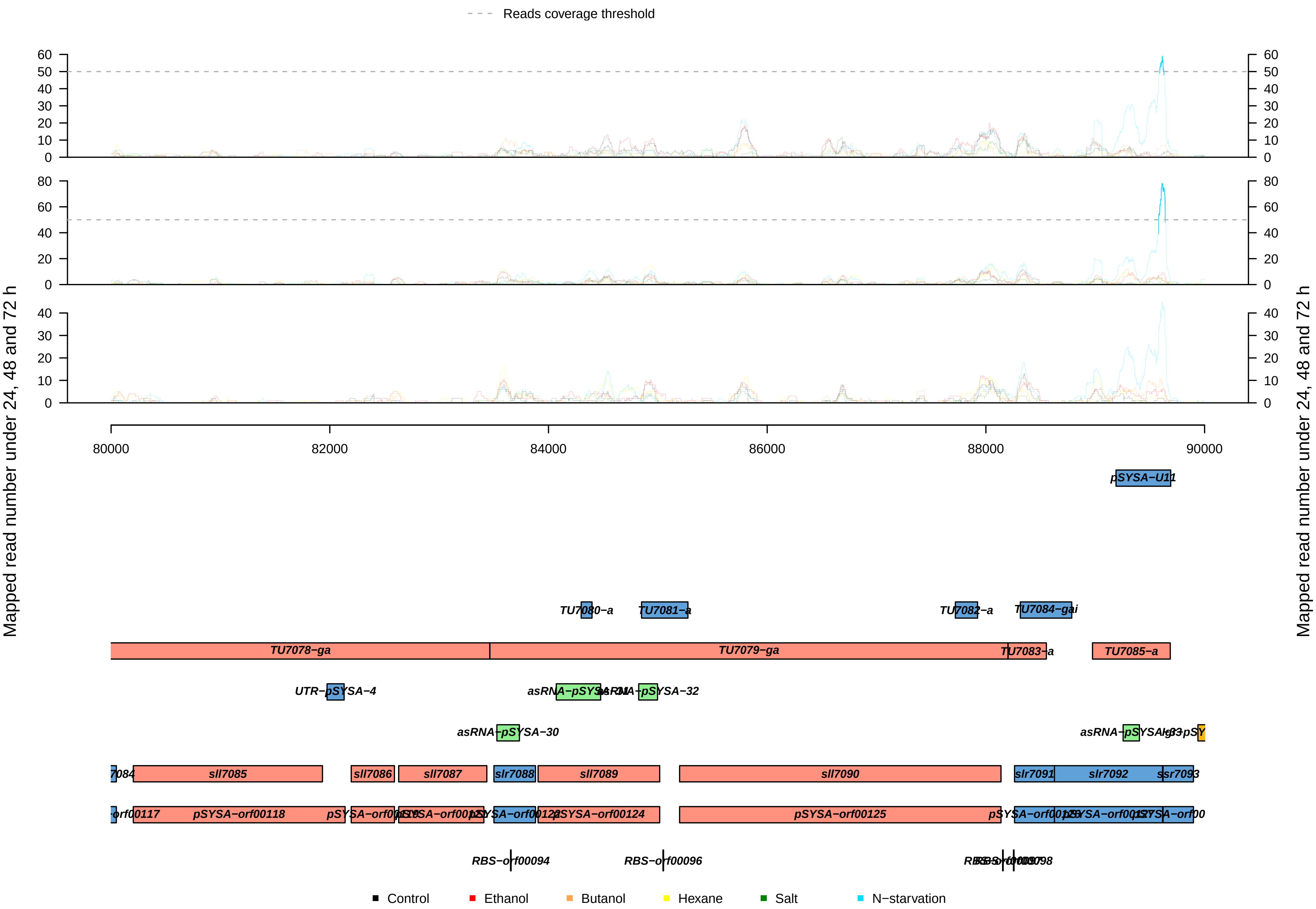
Reads coverage threshold
Mapped read number under 24, 48 and 72 h
80000
82000
84000
86000
88000
90000
pSYSA−U11
TU7080−a
TU7081−a
TU7082−a
TU7084−gai
TU7078−ga
TU7079−ga
TU7083−a
TU7085−a
UTR−pSYSA−4
asRNA−pSYSA−30
asRNA−pSYSA−32
sll7085
sll7086
sll7087
slr7088
sll7089
sll7090
slr7091
slr7092
ssr7093
pSYSA−orf00118
pSYSA−orf00124
pSYSA−orf00125
RBS−orf00094
RBS−orf00096
Control
Ethanol
Butanol
Hexane
Salt
N−starvation

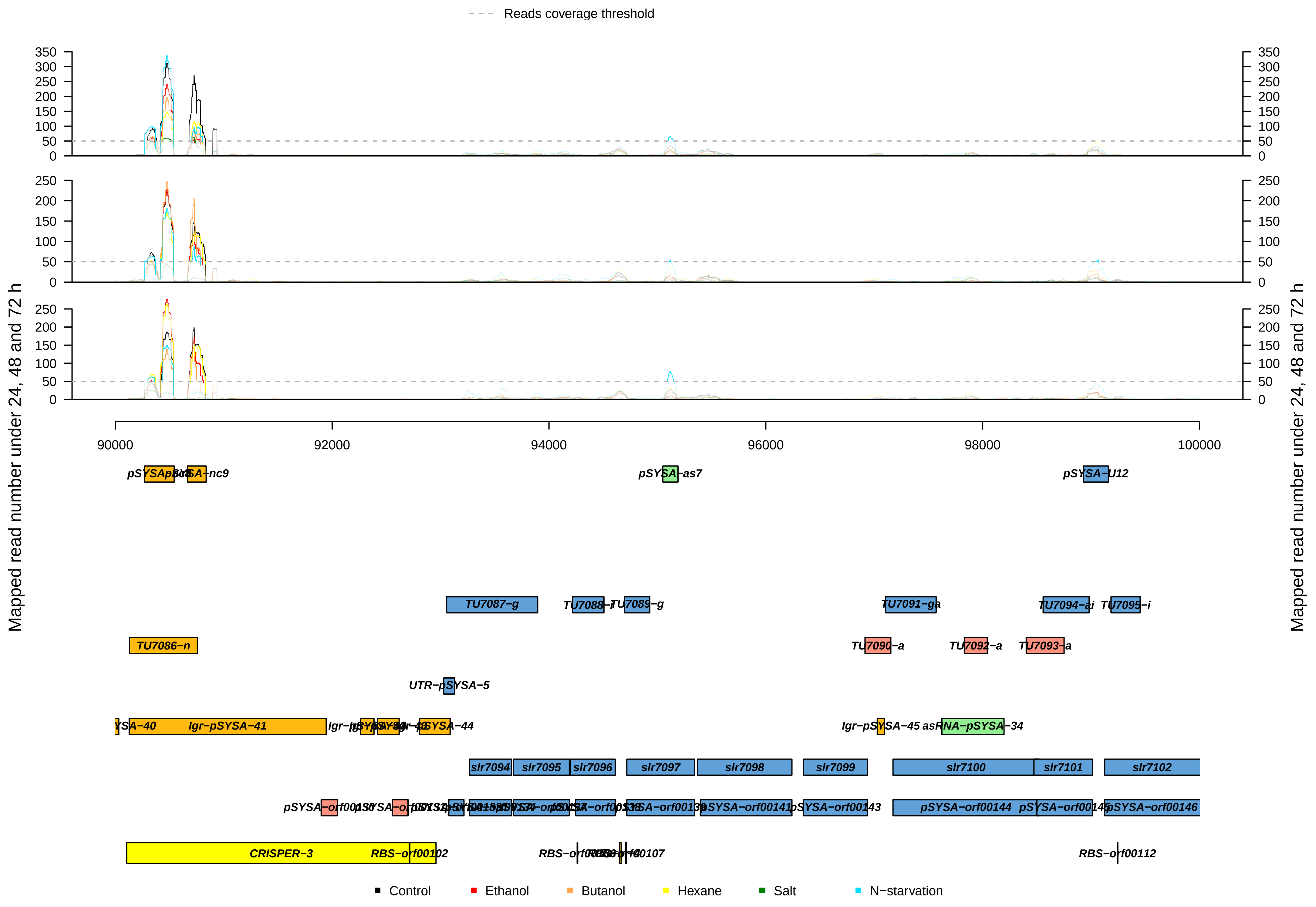

Reads coverage threshold
Mapped read number under 24, 48 and 72 h
90000
92000
94000
96000
98000
100000
pSYSA-as7
TU7087-g
TU7086-n
UTR-pSYSA-5
Igr-pSYSA-41
Igr-pSYSA-45
slr7094
slr7095
slr7096
slr7097
slr7098
slr7099
slr7100
slr7101
slr7102
pSYSA-orf00141
pSYSA-orf00143
pSYSA-orf00144
pSYSA-orf00146
CRISPER-3
RBS-orf00102
RBS-orf00107
RBS-orf00112
Control
Ethanol
Butanol
Hexane
Salt
N-starvation

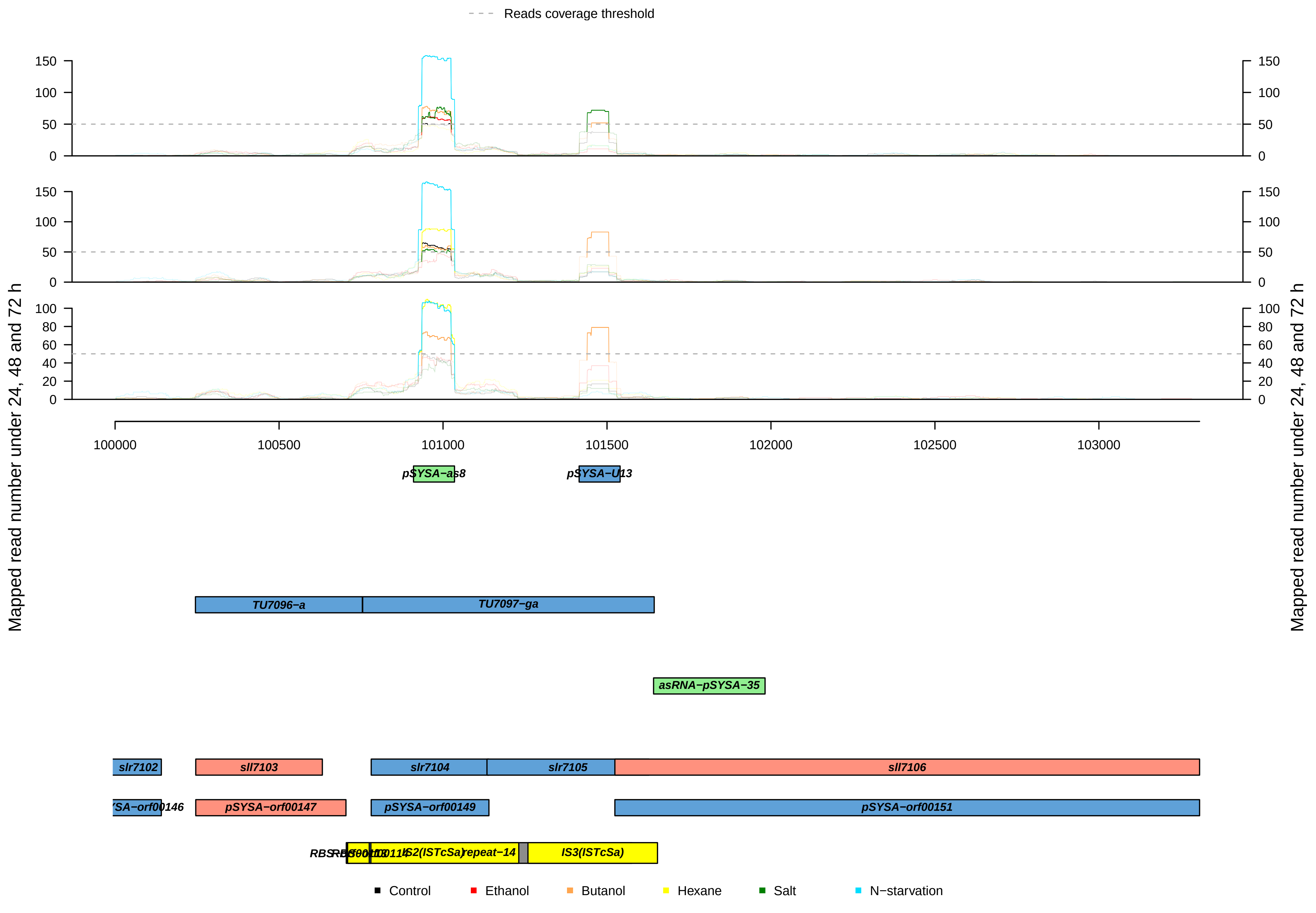

Reads coverage threshold
Mapped read number under 24, 48 and 72 h
150
100
50
0
150
100
50
0
100
80
60
40
20
0
100000
100500
101000
101500
102000
102500
103000
pSYSA−as8
pSYSA−U13
TU7096−a
TU7097−ga
asRNA−pSYSA−35
slr7102
sll7103
slr7104
slr7105
sll7106
pSYSA−orf00147
pSYSA−orf00149
pSYSA−orf00151
IS3(ISTcSa)
Control
Ethanol
Butanol
Hexane
Salt
N−starvation
