# Supplementary material for: Systematic and functional identification of small non-coding RNAs associated with exogenous biofuel stress in cyanobacterium Synechocystis sp. PCC 6803
Source: Biotechnol Biofuels. 2017 Mar 7;10:57. doi: 10.1186/s13068-017-0743-y (PMC5341163; doi:10.1186/s13068-017-0743-y)
Supplement: Supplementary file 18 — Additional file 18: Figure S12. Genome-wide visualization of all sRNA mapping data in pSYSG of Synechocystis. Detailed description is the same as Additional file 2: Figure S1. [file 13068_2017_743_MOESM18_ESM.pdf]

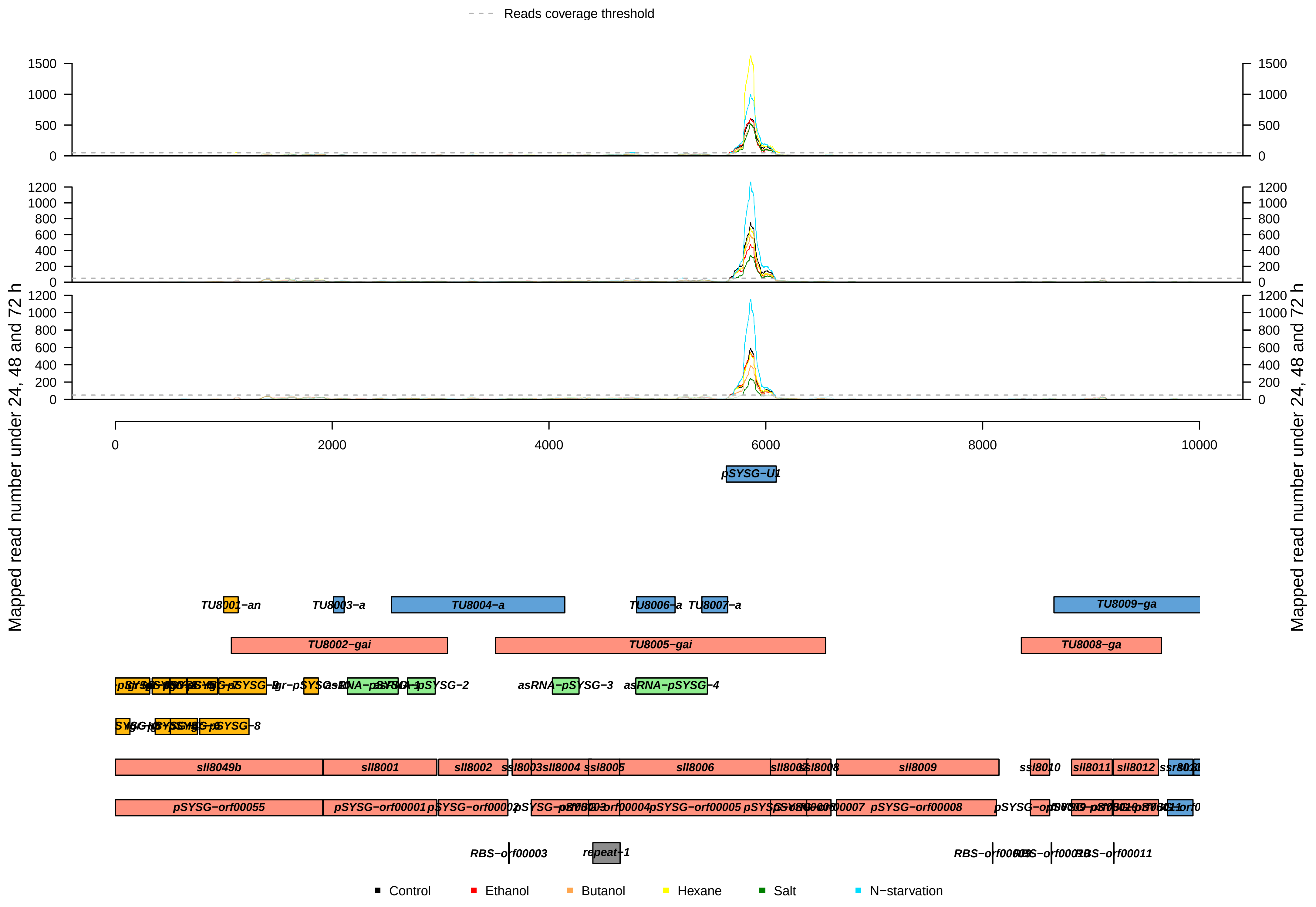

Reads coverage threshold
Mapped read number under 24, 48 and 72 h
0
2000
4000
6000
8000
10000
pSYSG−U1
TU8001−an
TU8003−a
TU8004−a
TU8006−a
TU8007−a
TU8009−ga
TU8002−gai
TU8005−gai
TU8008−ga
asRNA−pSYSG−3
asRNA−pSYSG−4
sll8049b
sll8001
sll8002
sll8006
sll8009
ssl8010
sll8011
sll8012
pSYSG−orf00055
pSYSG−orf00001
pSYSG−orf00008
RBS−orf00003
repeat−1
RBS−orf00011
Control
Ethanol
Butanol
Hexane
Salt
N−starvation

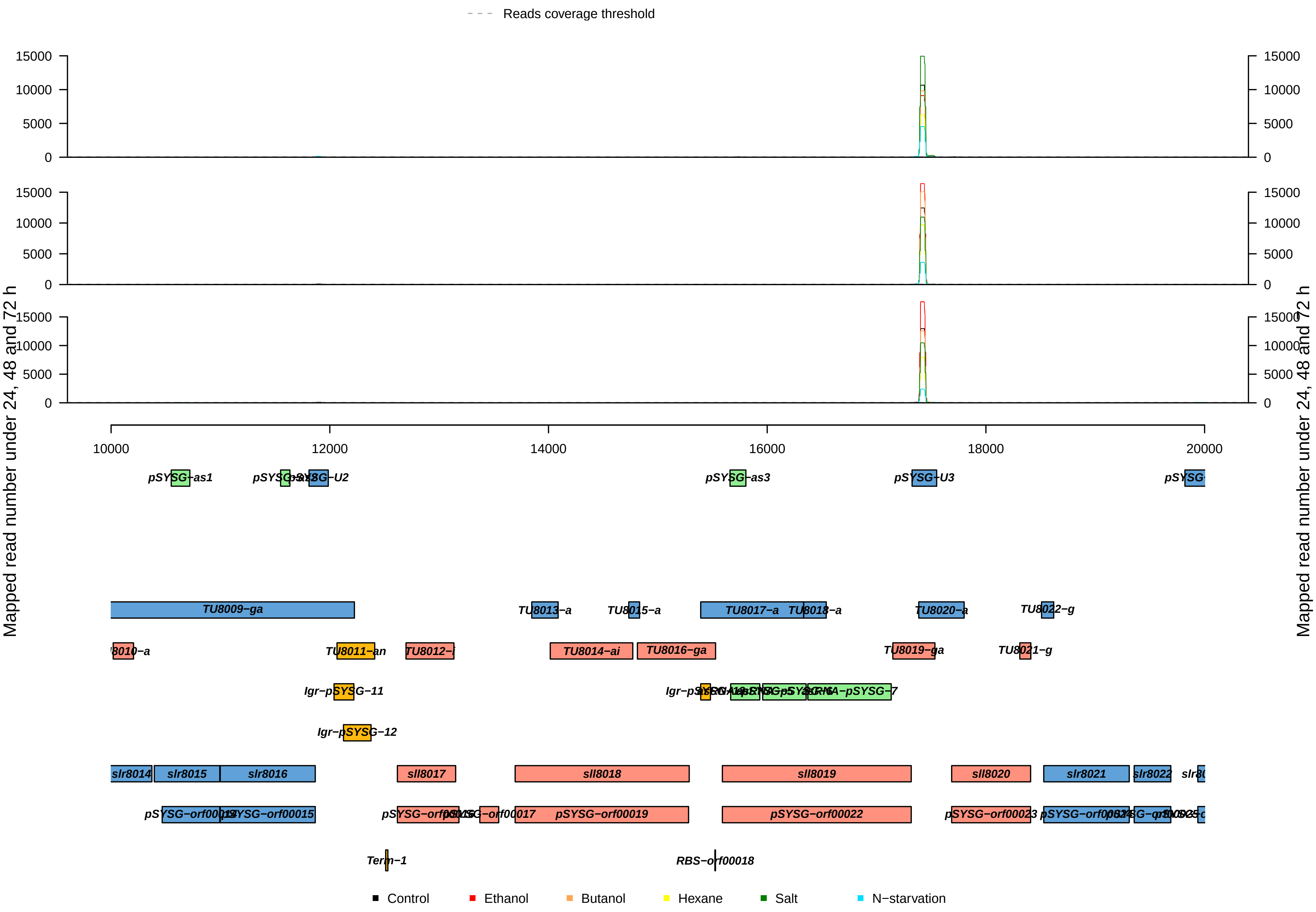

Reads coverage threshold
Mapped read number under 24, 48 and 72 h
15000
10000
5000
0
10000
12000
14000
16000
18000
20000
pSYSG−as1
pSYSG−U2
pSYSG−as3
pSYSG−U3
TU8009−ga
TU8013−a
TU8015−a
TU8017−a
TU8018−a
TU8020−a
TU8022−g
TU8011−an
TU8014−ai
TU8016−ga
TU8019−ga
TU8021−g
Igr−pSYSG−11
Igr−pSYSG−12
slr8014
slr8015
slr8016
sll8017
sll8018
sll8019
sll8020
slr8021
slr8022
pSYSG−orf00015
pSYSG−orf00017
pSYSG−orf00019
pSYSG−orf00022
pSYSG−orf00023
Term−1
RBS−orf00018
Control
Ethanol
Butanol
Hexane
Salt
N−starvation

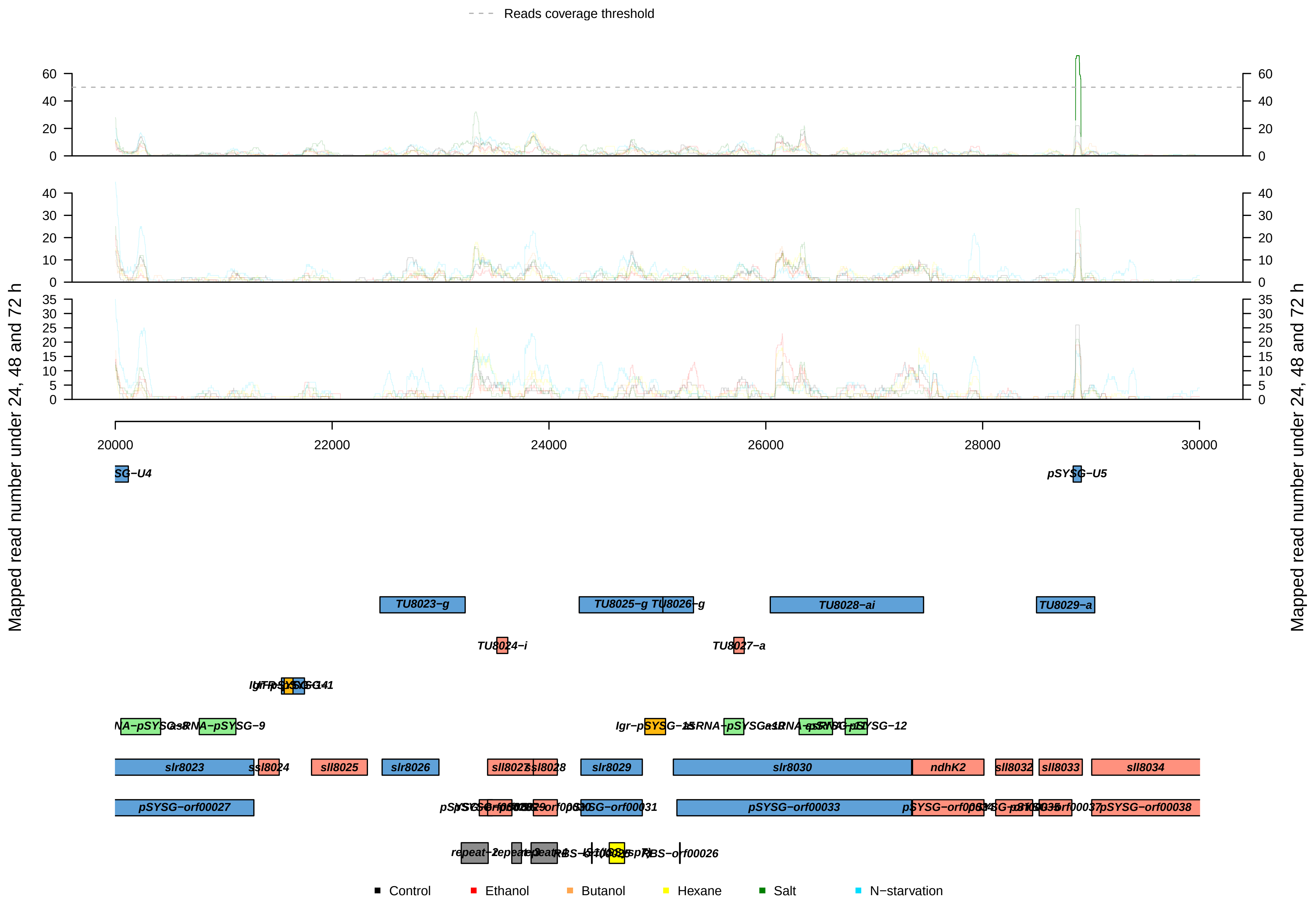

Reads coverage threshold
Mapped read number under 24, 48 and 72 h
20000
22000
24000
26000
28000
30000
pSYSG−U5
TU8023−g
TU8025−g
TU8026−g
TU8028−ai
TU8029−a
TU8024−i
TU8027−a
slr8023
ssl8024
sll8025
slr8026
sll8027
ssl8028
slr8029
slr8030
ndhK2
sll8032
sll8033
sll8034
pSYSG−orf00027
pSYSG−orf00031
pSYSG−orf00033
pSYSG−orf00038
RBS−orf00026
Control
Ethanol
Butanol
Hexane
Salt
N−starvation

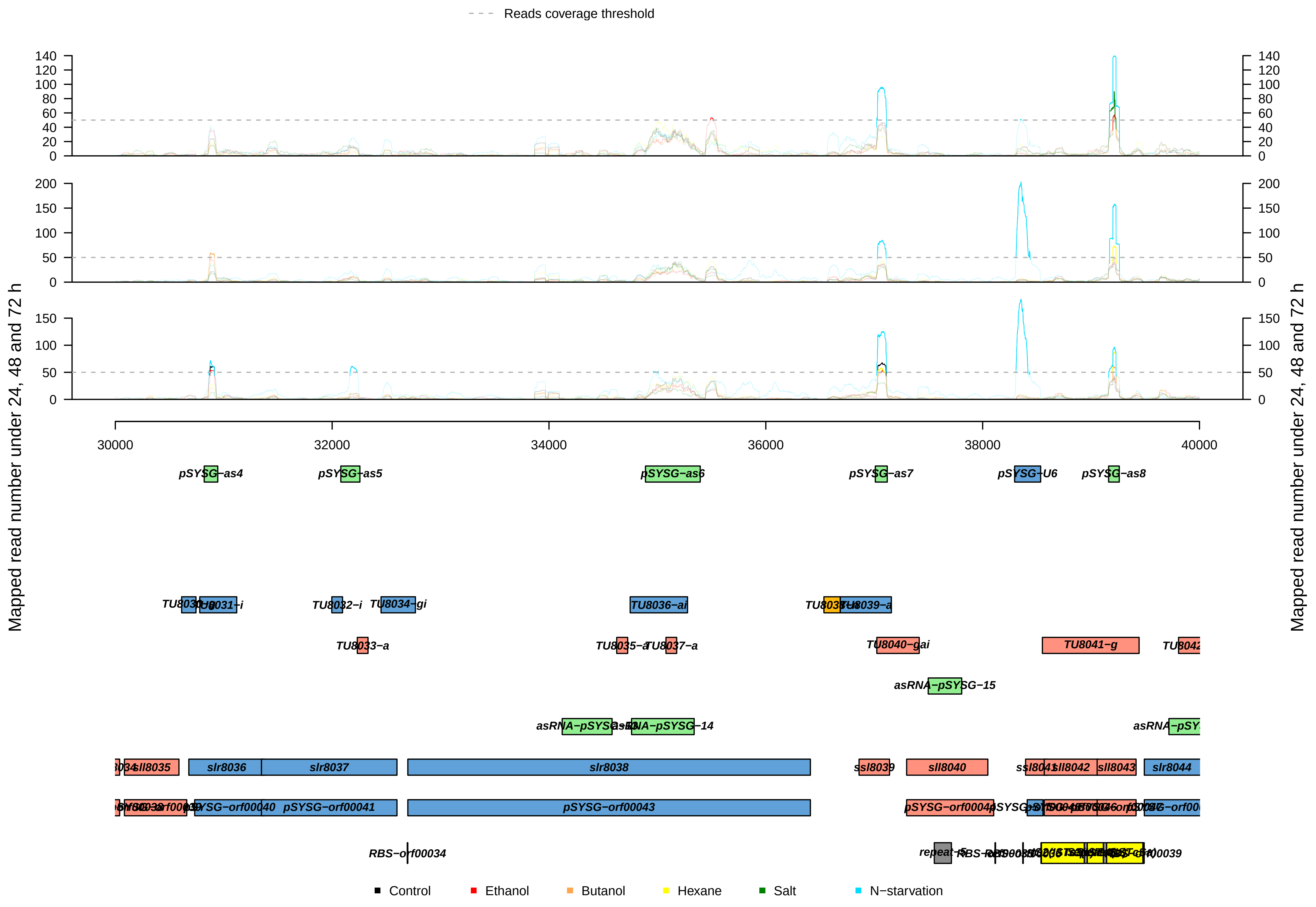

Reads coverage threshold
Mapped read number under 24, 48 and 72 h
30000
32000
34000
36000
38000
40000
pSYSG−as4
pSYSG−as5
pSYSG−as6
pSYSG−as7
pSYSG−U6
pSYSG−as8
TU8036−ai
TU8039−a
TU8033−a
TU8037−a
TU8040−gai
TU8041−g
asRNA−pSYSG−15
asRNA−pSYSG−14
sll8035
slr8036
slr8037
slr8038
ssl8039
sll8040
sll8042
sll8043
slr8044
pSYSG−orf00041
pSYSG−orf00043
RBS−orf00034
Control
Ethanol
Butanol
Hexane
Salt
N−starvation

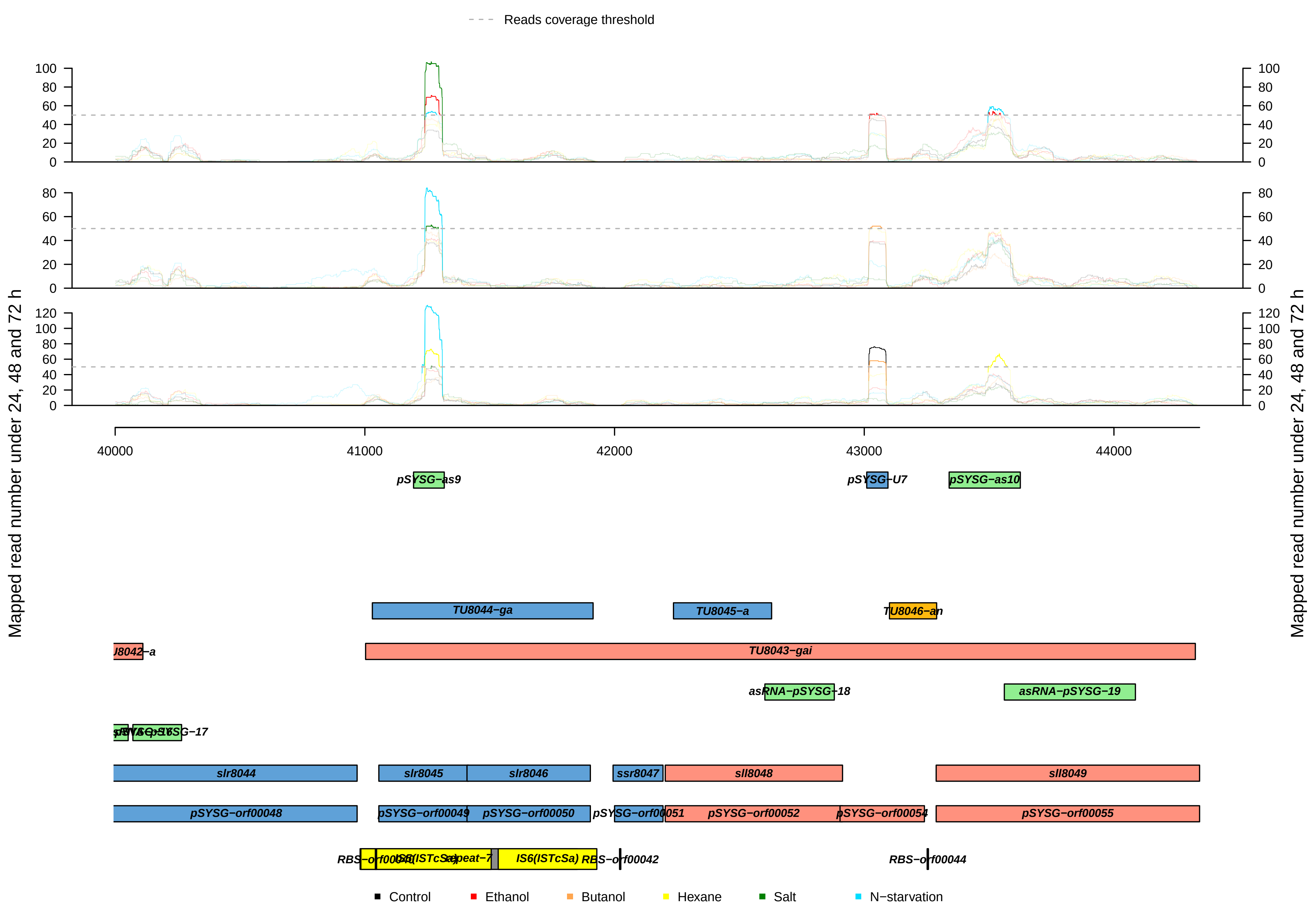

Reads coverage threshold
Mapped read number under 24, 48 and 72 h
40000
41000
42000
43000
44000
pSYSG−as9
pSYSG−U7
pSYSG−as10
TU8044−ga
TU8045−a
TU8046−an
TU8043−gai
asRNA−pSYSG−18
asRNA−pSYSG−19
slr8044
slr8045
slr8046
ssr8047
sll8048
sll8049
pSYSG−orf00048
pSYSG−orf00049
pSYSG−orf00050
pSYSG−orf00051
pSYSG−orf00052
pSYSG−orf00054
pSYSG−orf00055
IS6(ISTcSa)
RBS−orf00042
RBS−orf00044
Control
Ethanol
Butanol
Hexane
Salt
N−starvation
