# Supplementary material for: Systematic and functional identification of small non-coding RNAs associated with exogenous biofuel stress in cyanobacterium Synechocystis sp. PCC 6803
Source: Biotechnol Biofuels. 2017 Mar 7;10:57. doi: 10.1186/s13068-017-0743-y (PMC5341163; doi:10.1186/s13068-017-0743-y)
Supplement: Supplementary file 19 — Additional file 19: Figure S13. Genome-wide visualization of all sRNA mapping data in pSYSX of Synechocystis. Detailed description is the same as Additional file 2: Figure S1. [file 13068_2017_743_MOESM19_ESM.pdf]

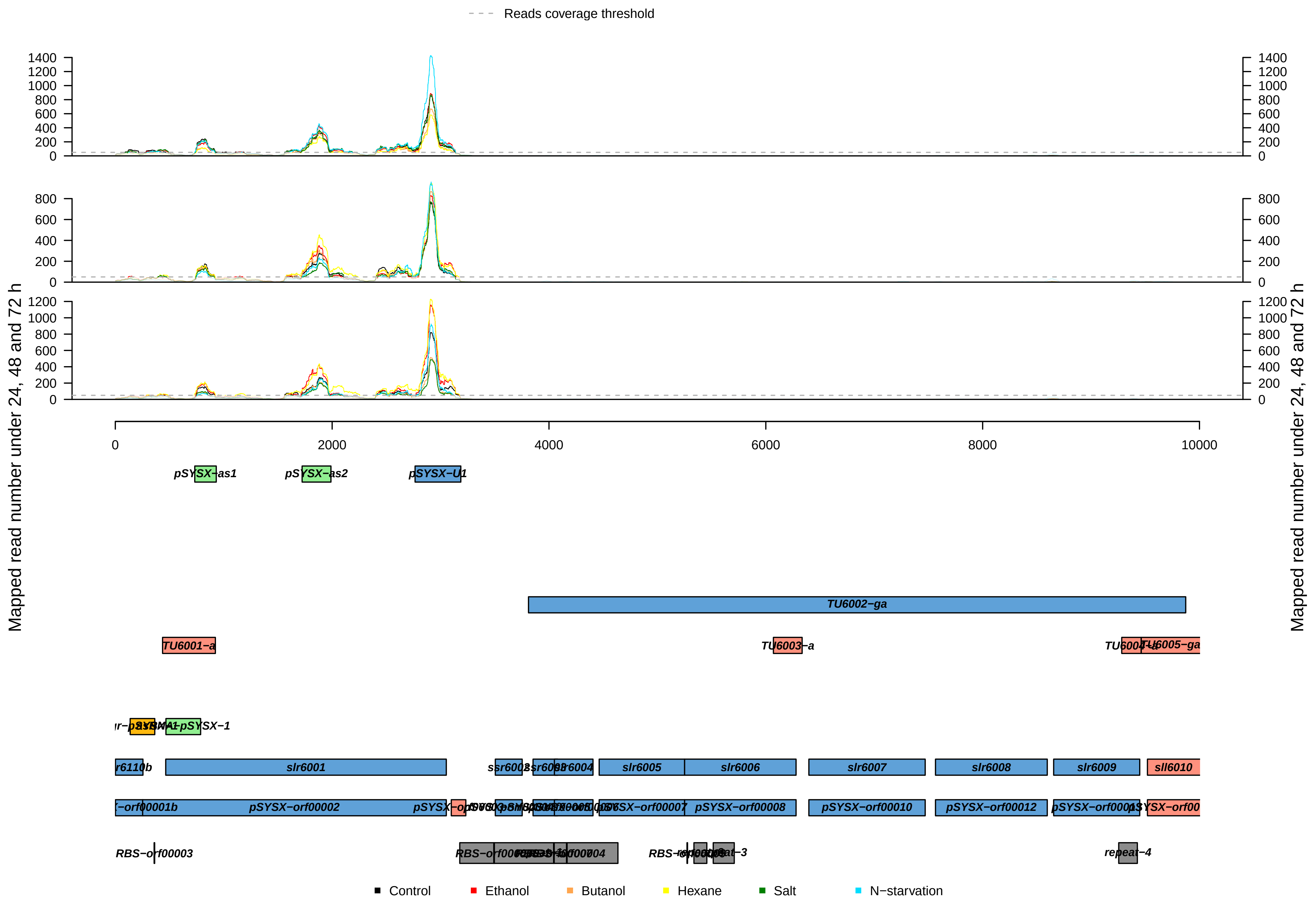

Reads coverage threshold
Mapped read number under 24, 48 and 72 h
0
2000
4000
6000
8000
10000
pSYSX−as1
pSYSX−as2
pSYSX−U1
TU6002−ga
TU6001−a
TU6003−a
TU6005−ga
slr6001
slr6005
slr6006
slr6007
slr6008
slr6009
sll6010
pSYSX−orf00002
pSYSX−orf00007
pSYSX−orf00008
pSYSX−orf00010
pSYSX−orf00012
RBS−orf00003
repeat−4
Control
Ethanol
Butanol
Hexane
Salt
N−starvation

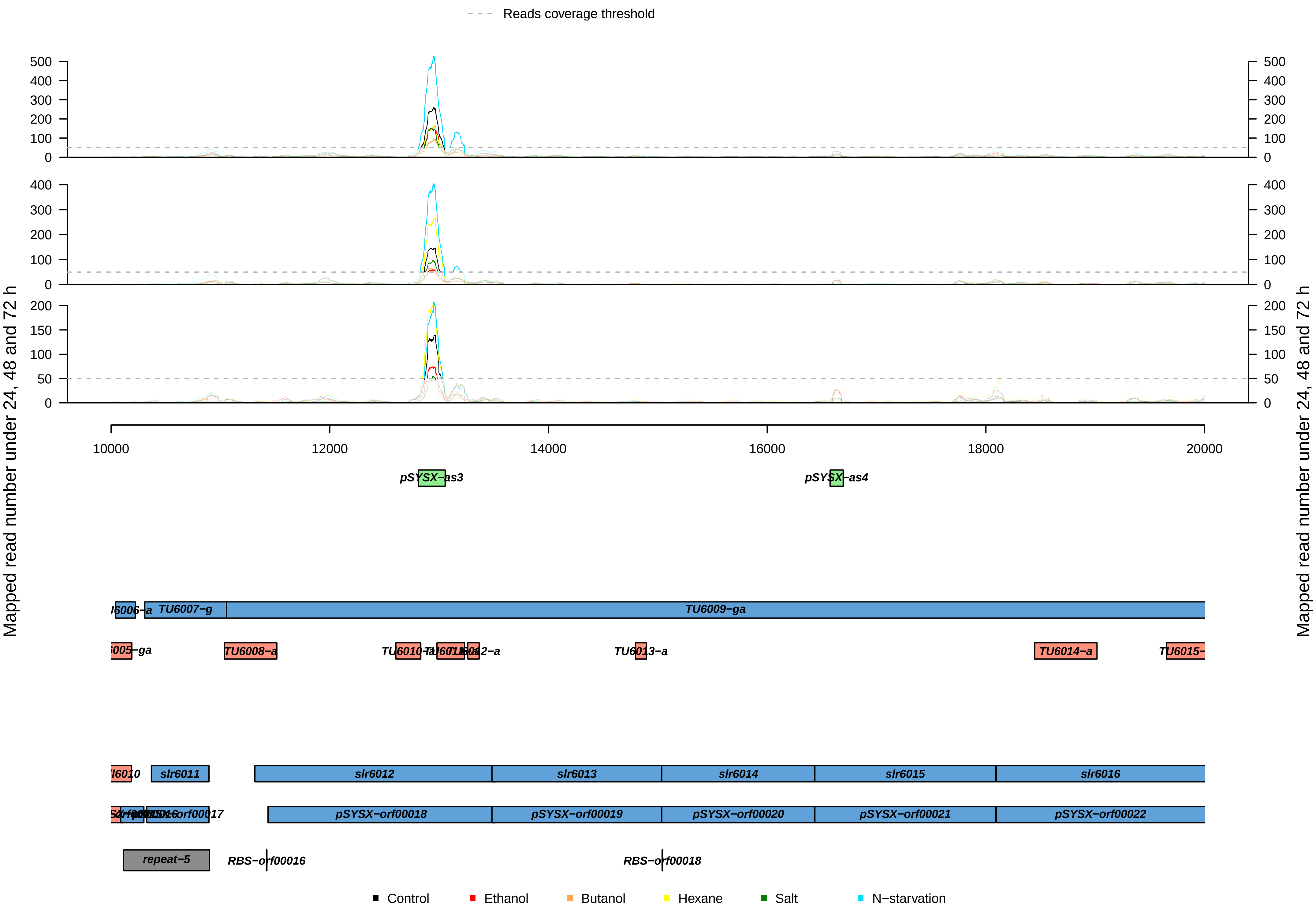

Reads coverage threshold
Mapped read number under 24, 48 and 72 h
500
400
300
200
100
0
10000
12000
14000
16000
18000
20000
pSYSX−as3
pSYSX−as4
TU6007−g
TU6009−ga
TU6008−a
TU6013−a
TU6014−a
slr6011
slr6012
slr6013
slr6014
slr6015
slr6016
pSYSX−orf00017
pSYSX−orf00018
pSYSX−orf00019
pSYSX−orf00020
pSYSX−orf00021
pSYSX−orf00022
repeat−5
RBS−orf00016
RBS−orf00018
Control
Ethanol
Butanol
Hexane
Salt
N−starvation

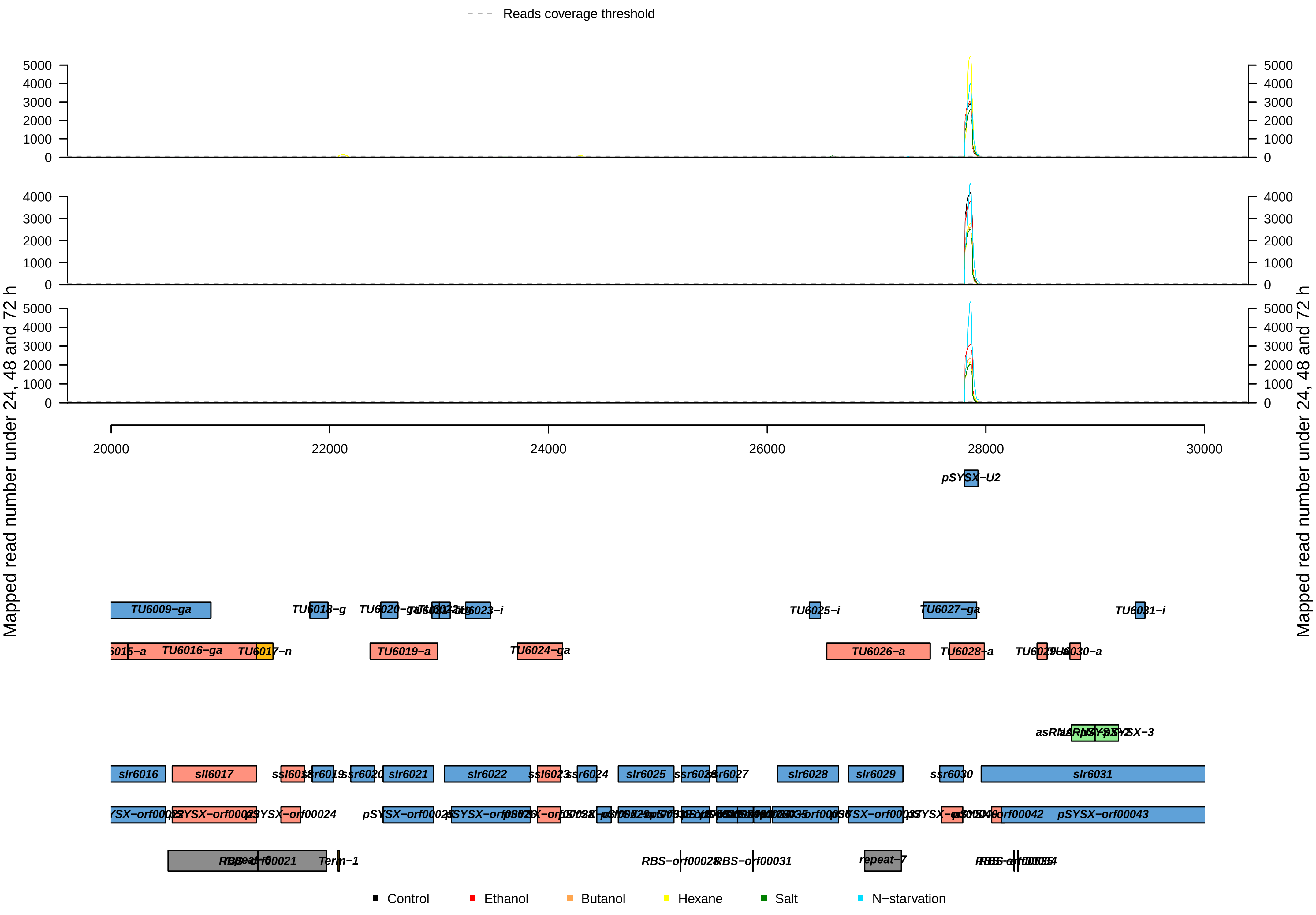

Reads coverage threshold
Mapped read number under 24, 48 and 72 h
0
1000
2000
3000
4000
5000
20000
22000
24000
26000
28000
30000
pSYSX−U2
TU6009−ga
TU6018−g
TU6023−i
TU6025−i
TU6027−ga
TU6031−i
TU6016−ga
TU6017−n
TU6019−a
TU6024−ga
TU6026−a
TU6028−a
TU6030−a
slr6016
sll6017
ssr6019
ssr6020
slr6021
slr6022
ssl6023
ssr6024
slr6025
ssr6027
slr6028
slr6029
ssr6030
slr6031
pSYSX−orf00024
pSYSX−orf00042
pSYSX−orf00043
RBS−orf00021
Term−1
RBS−orf00028
RBS−orf00031
repeat−7
Control
Ethanol
Butanol
Hexane
Salt
N−starvation

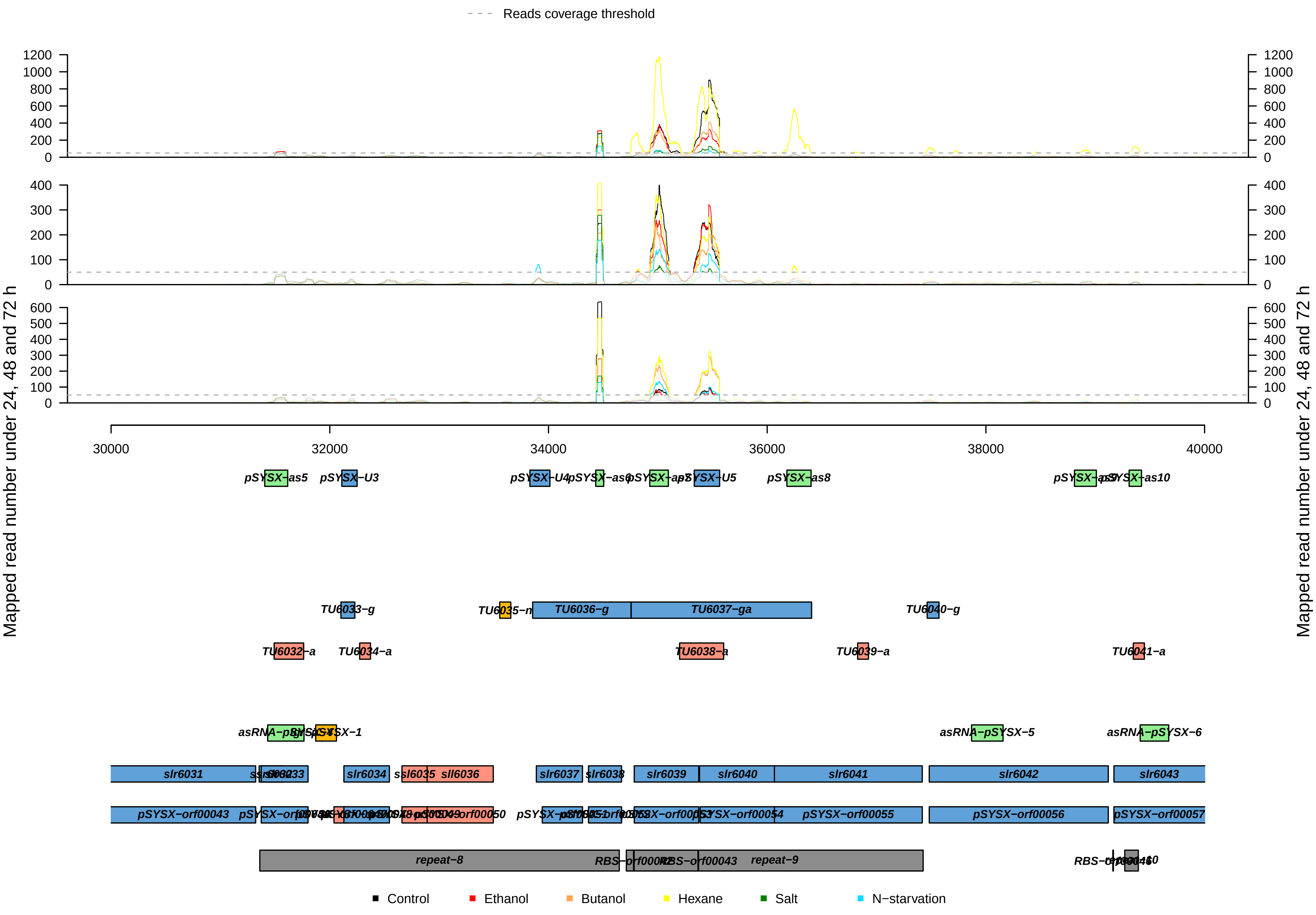

Reads coverage threshold
Mapped read number under 24, 48 and 72 h
30000
32000
34000
36000
38000
40000
pSYSX−as5
pSYSX−U3
pSYSX−U4
pSYSX−U5
pSYSX−as8
pSYSX−as10
TU6033−g
TU6036−g
TU6037−ga
TU6040−g
TU6032−a
TU6034−a
TU6038−a
TU6039−a
TU6041−a
asRNA−pSYSX−5
asRNA−pSYSX−6
slr6031
slr6034
ssl6035
sll6036
slr6037
slr6038
slr6039
slr6040
slr6041
slr6042
slr6043
pSYSX−orf00043
pSYSX−orf00050
pSYSX−orf00054
pSYSX−orf00055
pSYSX−orf00056
pSYSX−orf00057
repeat−8
repeat−9
Control
Ethanol
Butanol
Hexane
Salt
N−starvation

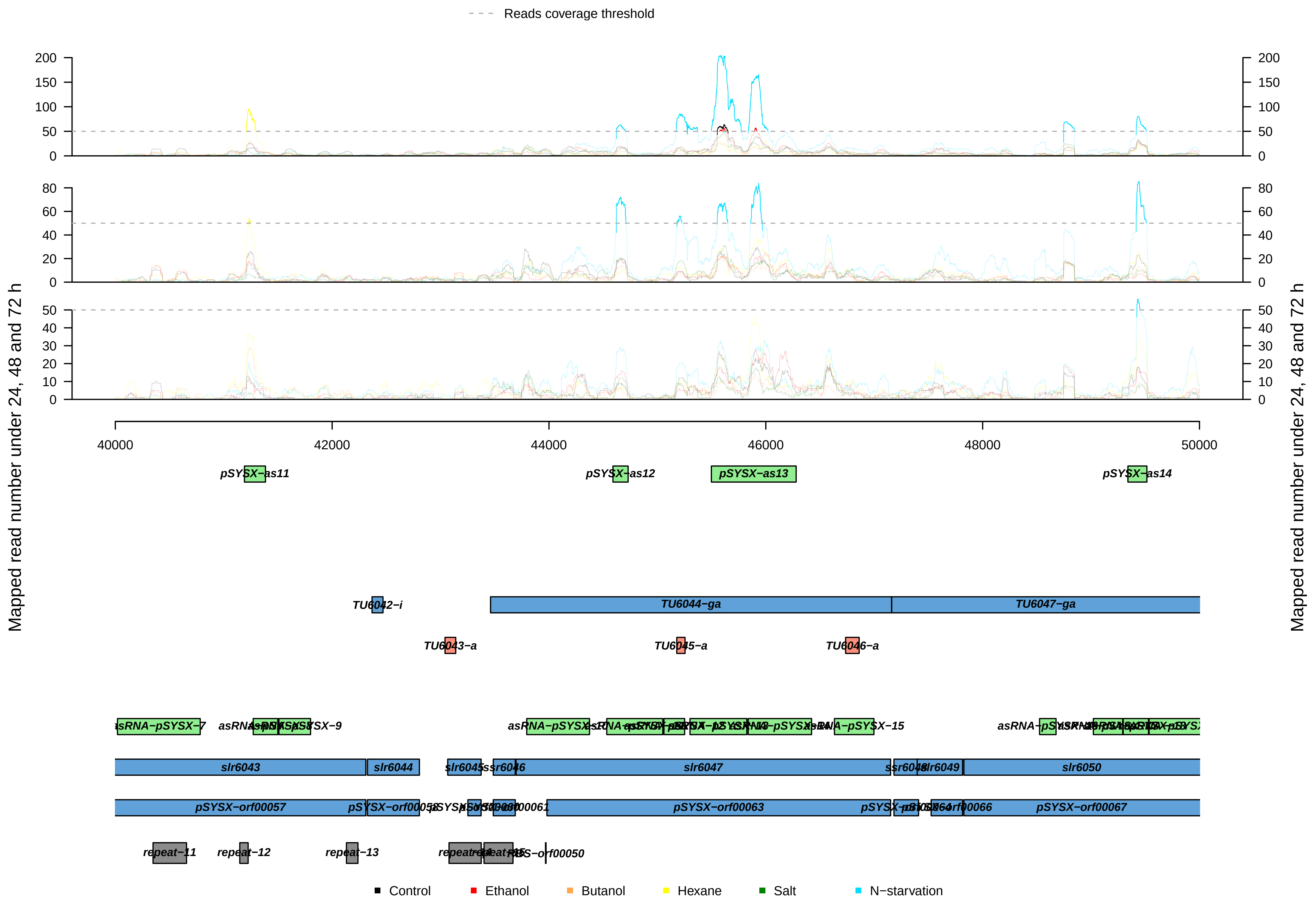

Reads coverage threshold
Mapped read number under 24, 48 and 72 h
pSYSX−as11
pSYSX−as12
pSYSX−as13
pSYSX−as14
TU6042−i
TU6044−ga
TU6047−ga
TU6043−a
TU6045−a
TU6046−a
asRNA−pSYSX−7
asRNA−pSYSX−15
slr6043
slr6044
slr6047
slr6050
pSYSX−orf00057
pSYSX−orf00063
pSYSX−orf00067
repeat−11
repeat−12
repeat−13
Control
Ethanol
Butanol
Hexane
Salt
N−starvation

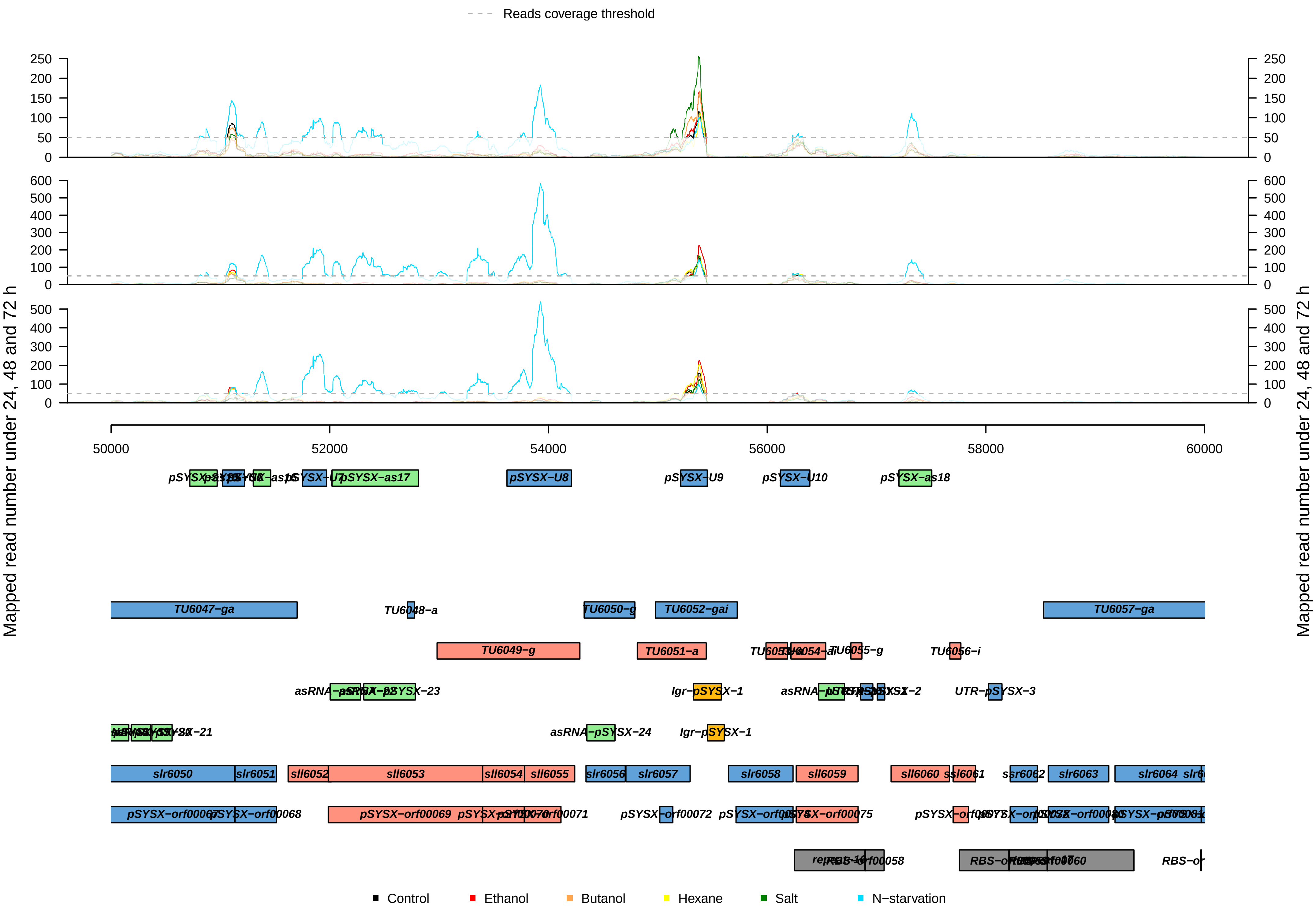

Reads coverage threshold
250
200
150
100
50
0
600
500
400
300
200
100
0
500
400
300
200
100
0
Mapped read number under 24, 48 and 72 h
50000
52000
54000
56000
58000
60000
pSYSX−U8
pSYSX−U9
pSYSX−U10
pSYSX−as18
pSYSX−as17
TU6047−ga
TU6048−a
TU6052−gai
TU6057−ga
TU6049−g
TU6051−a
TU6055−g
TU6056−i
Igr−pSYSX−1
UTR−pSYSX−3
asRNA−pSYSX−24
slr6050
slr6051
sll6052
sll6053
sll6054
sll6055
slr6056
slr6057
slr6058
sll6059
sll6060
ssl6061
ssr6062
slr6063
slr6064
pSYSX−orf00069
pSYSX−orf00072
Control
Ethanol
Butanol
Hexane
Salt
N−starvation

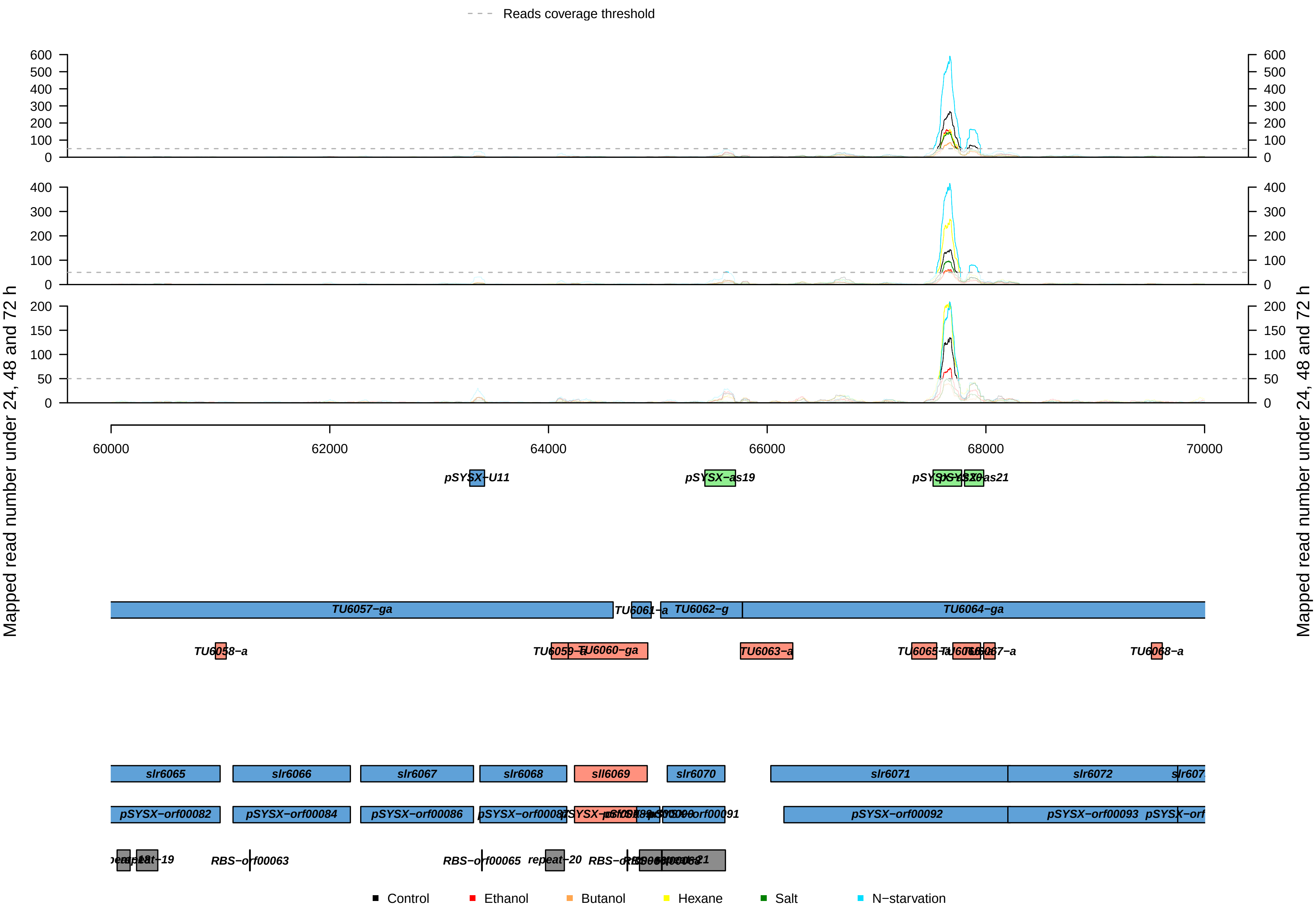

Reads coverage threshold
Mapped read number under 24, 48 and 72 h
600
500
400
300
200
100
0
400
300
200
100
0
200
150
100
50
0
60000
62000
64000
66000
68000
70000
pSYSX−U11
pSYSX−as19
TU6057−ga
TU6062−g
TU6064−ga
TU6058−a
TU6060−ga
TU6063−a
TU6068−a
slr6065
slr6066
slr6067
slr6068
sll6069
slr6070
slr6071
slr6072
pSYSX−orf00082
pSYSX−orf00084
pSYSX−orf00086
pSYSX−orf00092
pSYSX−orf00093
RBS−orf00063
RBS−orf00065
repeat−20
Control
Ethanol
Butanol
Hexane
Salt
N−starvation

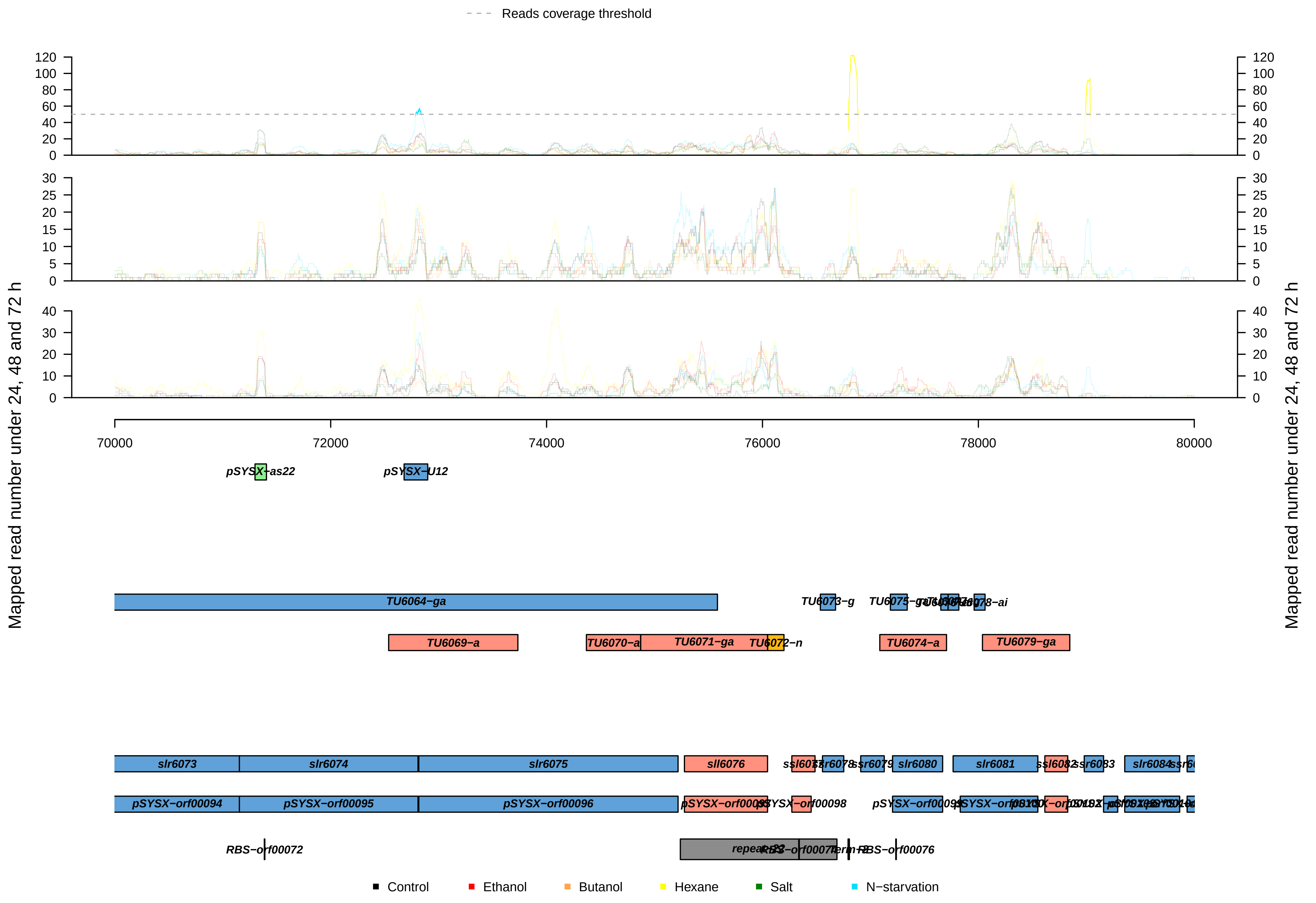

Reads coverage threshold
Mapped read number under 24, 48 and 72 h
70000
72000
74000
76000
78000
80000
pSYSX-as22
pSYSX-U12
TU6064-ga
TU6073-g
TU6069-a
TU6070-a
TU6071-ga
TU6072-n
TU6074-a
TU6079-ga
slr6073
slr6074
slr6075
sll6076
slr6080
slr6081
slr6084
pSYSX-orf00094
pSYSX-orf00095
pSYSX-orf00096
RBS-orf00072
RBS-orf00076
Control
Ethanol
Butanol
Hexane
Salt
N-starvation

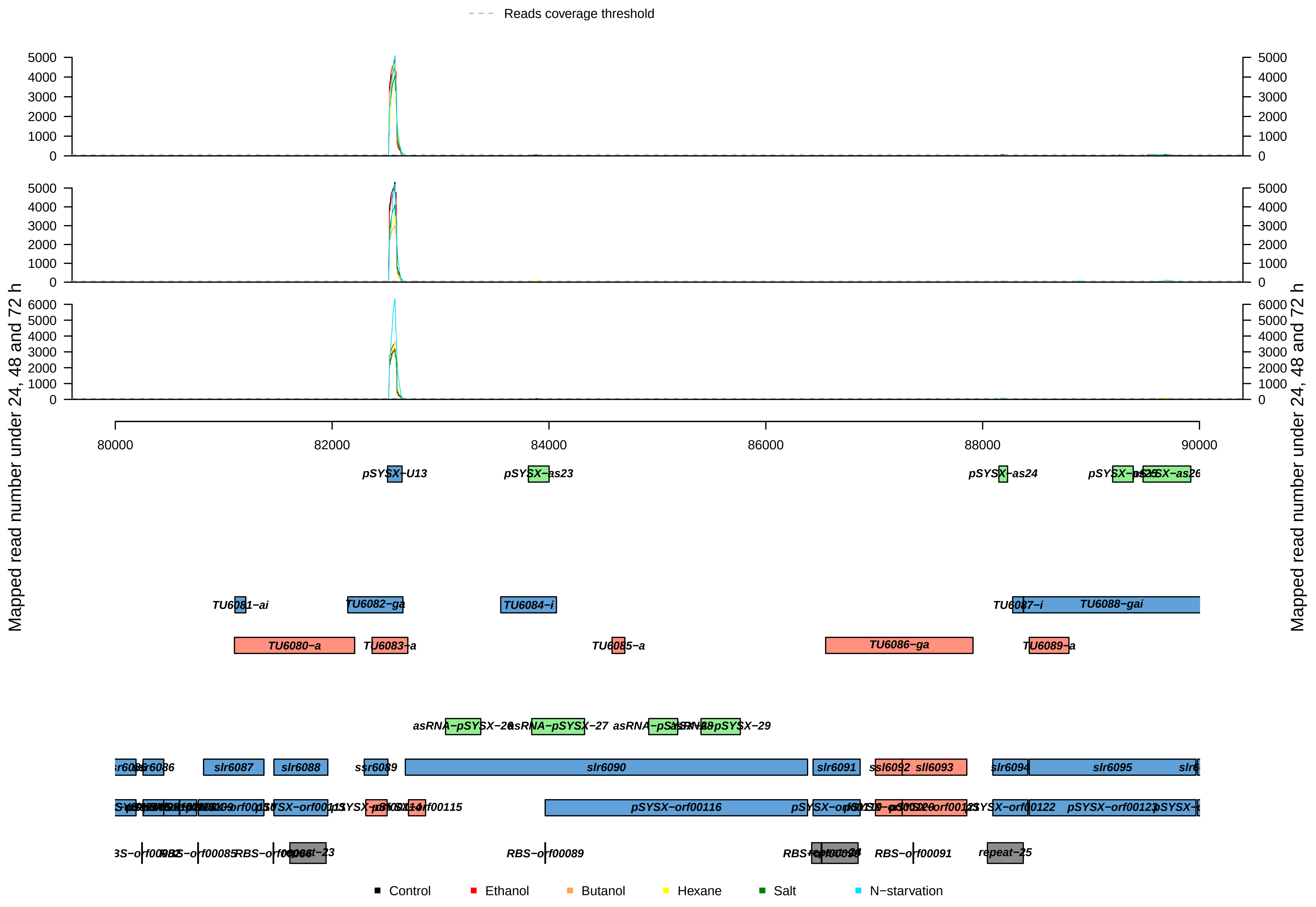
Reads coverage threshold
Mapped read number under 24, 48 and 72 h
80000
82000
84000
86000
88000
90000
pSYSX-U13
pSYSX-as23
pSYSX-as24
TU6081-ai
TU6082-ga
TU6084-i
TU6088-gai
TU6080-a
TU6083-a
TU6085-a
TU6086-ga
TU6089-a
asRNA-pSYSX-27
slr6087
slr6088
ssr6089
slr6090
slr6091
sll6093
slr6095
pSYSX-orf00116
pSYSX-orf00122
RBS-orf00089
RBS-orf00091
repeat-23
repeat-25
Control
Ethanol
Butanol
Hexane
Salt
N-starvation

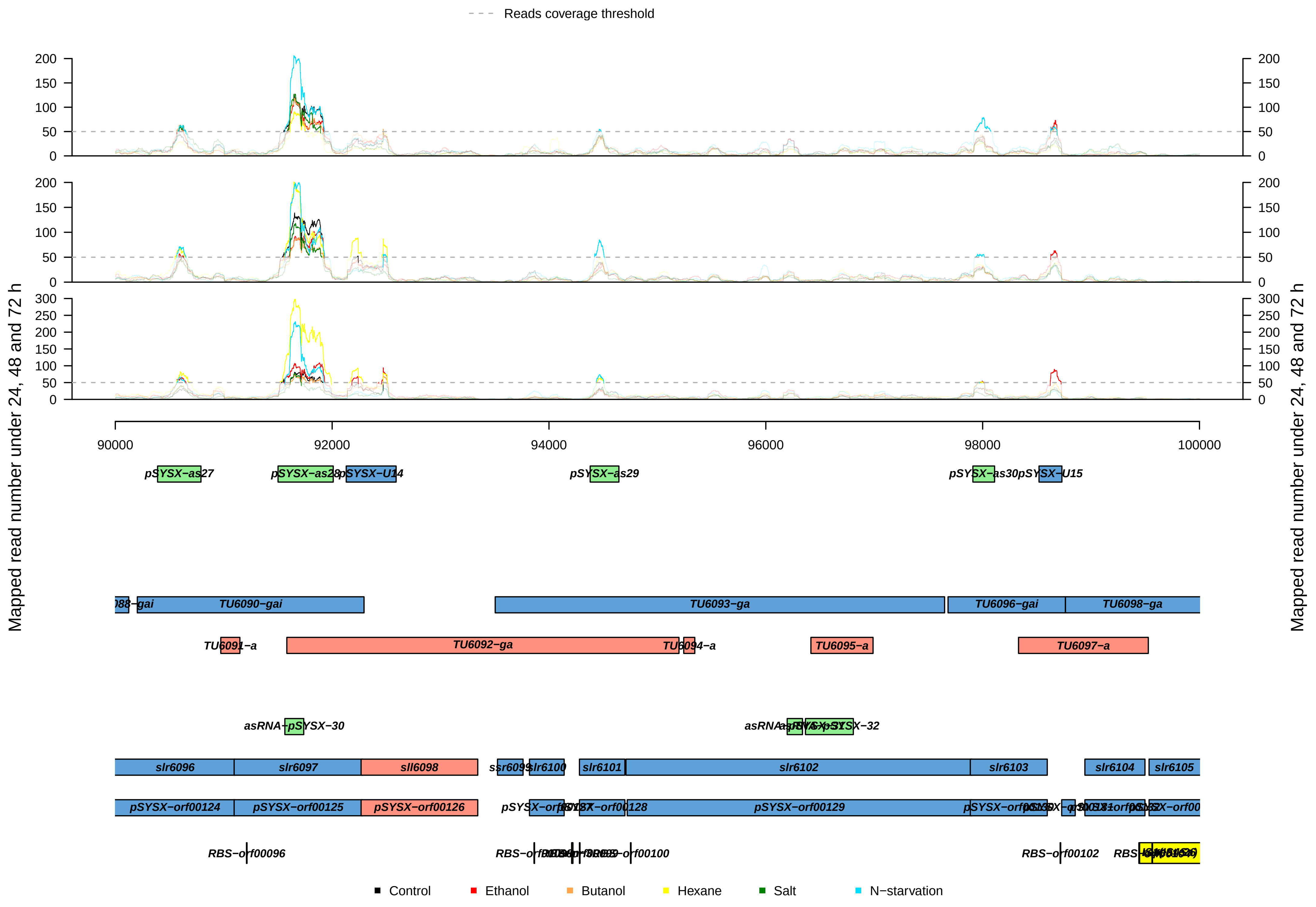
Reads coverage threshold
Mapped read number under 24, 48 and 72 h
90000
92000
94000
96000
98000
100000
pSYSX−as27
pSYSX−as28
pSYSX−U14
pSYSX−as29
pSYSX−as30
pSYSX−U15
TU6090−gai
TU6093−ga
TU6096−gai
TU6098−ga
TU6091−a
TU6092−ga
TU6094−a
TU6095−a
TU6097−a
asRNA−pSYSX−30
slr6096
slr6097
sll6098
slr6100
slr6101
slr6102
slr6103
slr6104
slr6105
pSYSX−orf00124
pSYSX−orf00125
pSYSX−orf00126
pSYSX−orf00129
RBS−orf00096
RBS−orf00102
Control
Ethanol
Butanol
Hexane
Salt
N−starvation

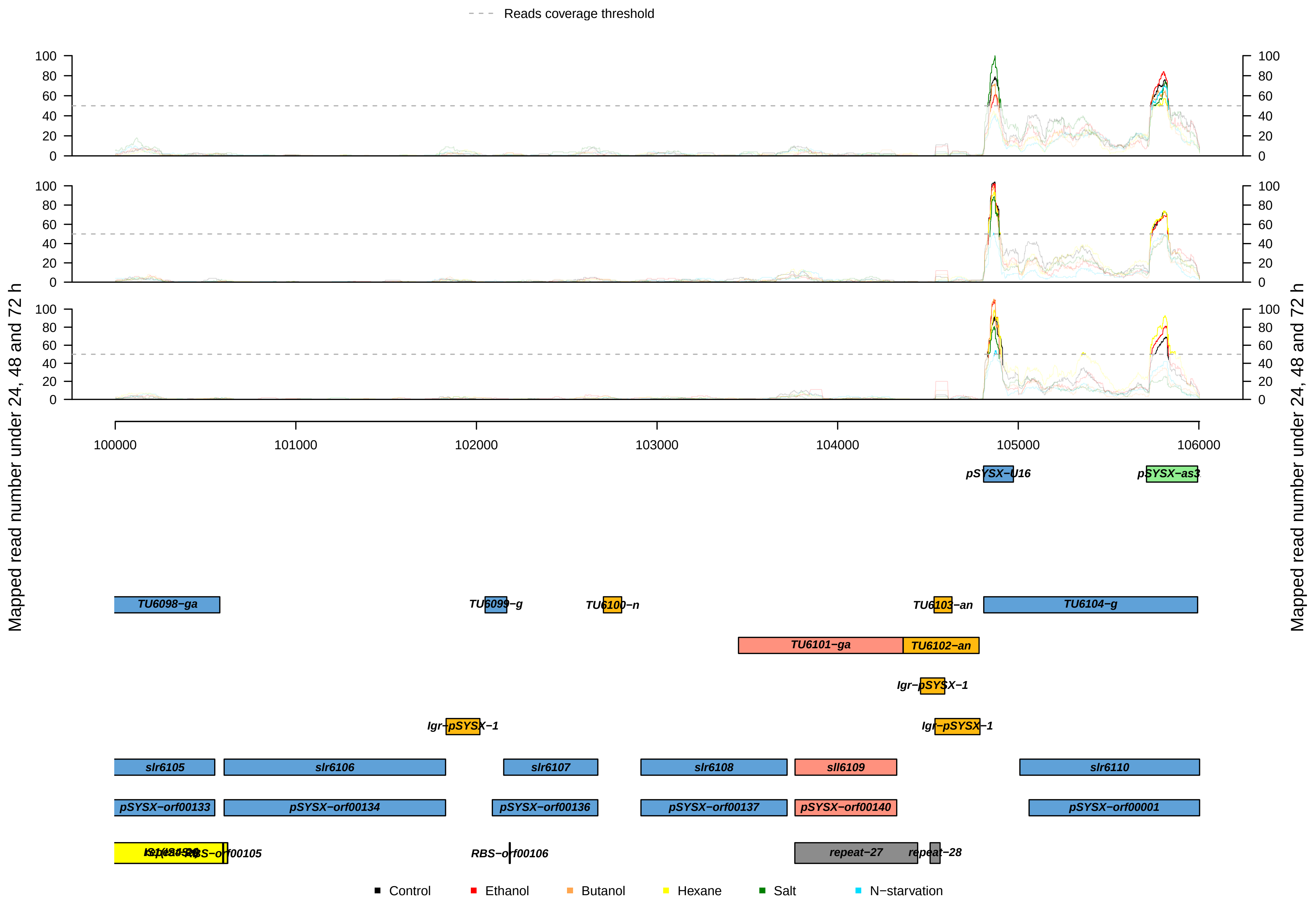

Reads coverage threshold
Mapped read number under 24, 48 and 72 h
100000
101000
102000
103000
104000
105000
106000
pSYSX−U16
pSYSX−as3
TU6098−ga
TU6099−g
TU6100−n
TU6103−an
TU6104−g
TU6101−ga
TU6102−an
Igr−pSYSX−1
Igr−pSYSX−1
Igr−pSYSX−1
slr6105
slr6106
slr6107
slr6108
sll6109
slr6110
pSYSX−orf00133
pSYSX−orf00134
pSYSX−orf00136
pSYSX−orf00137
pSYSX−orf00140
pSYSX−orf00001
RBS−orf00105
RBS−orf00106
repeat−27
repeat−28
Control
Ethanol
Butanol
Hexane
Salt
N−starvation
