# Supplementary material for: Systematic and functional identification of small non-coding RNAs associated with exogenous biofuel stress in cyanobacterium Synechocystis sp. PCC 6803
Source: Biotechnol Biofuels. 2017 Mar 7;10:57. doi: 10.1186/s13068-017-0743-y (PMC5341163; doi:10.1186/s13068-017-0743-y)
Supplement: Supplementary file 21 — Additional file 21: Figure S14. Schematic diagram of two-step RT-PCR for sRNA orienting. [file 13068_2017_743_MOESM21_ESM.pdf]

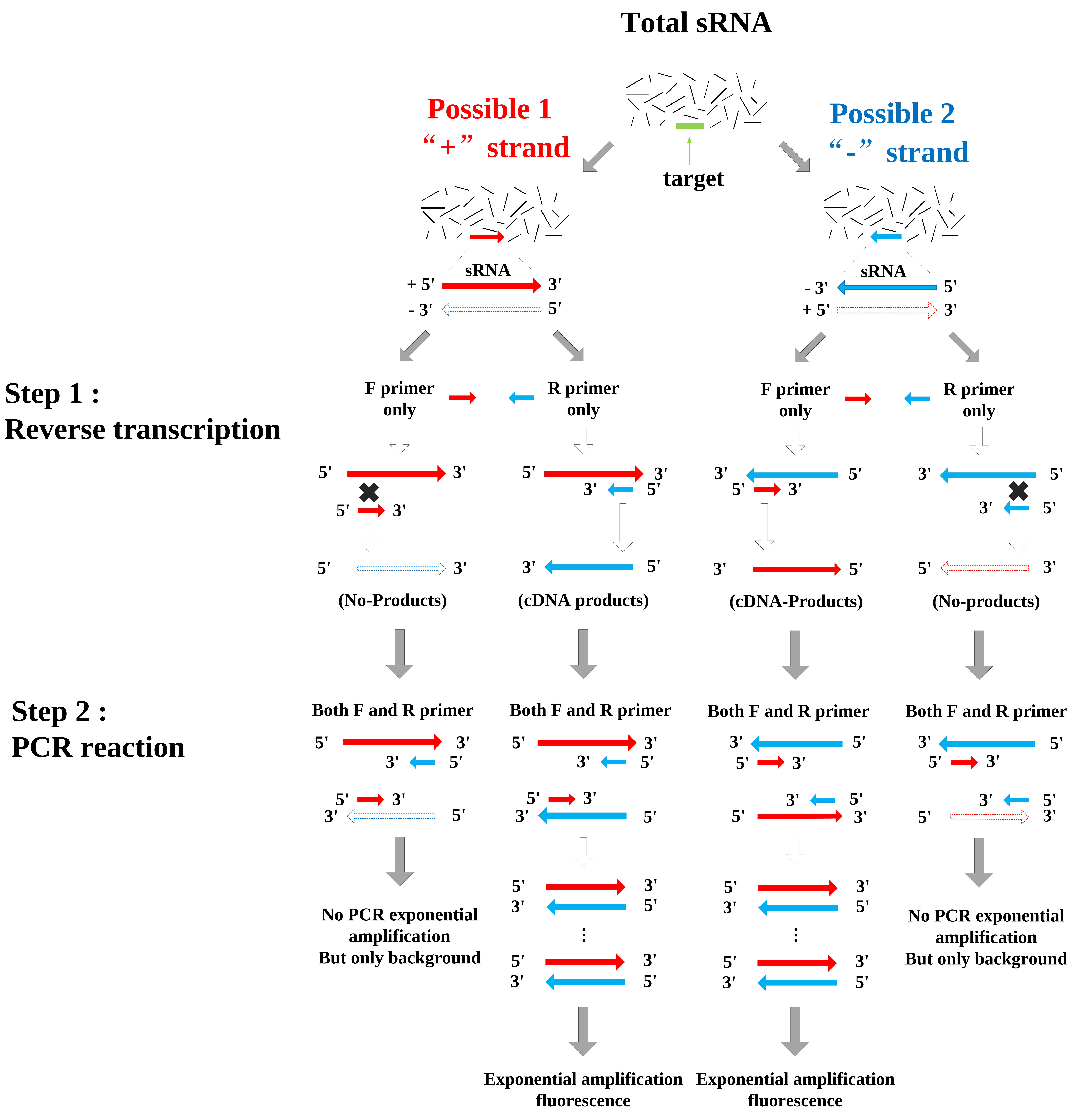
Total sRNA
Possible 1
"+" strand
target
Possible 2
"-" strand
sRNA
+ 5' 3'
- 3' 5'
sRNA
- 3' 5'
+ 5' 3'
Step 1 :
Reverse transcription
F primer only
R primer only
F primer only
R primer only
(No-Products)
(cDNA products)
(cDNA-Products)
(No-products)
Step 2 :
PCR reaction
Both F and R primer
Both F and R primer
Both F and R primer
Both F and R primer
No PCR exponential amplification
But only background
No PCR exponential amplification
But only background
Exponential amplification fluorescence
Exponential amplification fluorescence
