# Supplementary material for: Systematic and functional identification of small non-coding RNAs associated with exogenous biofuel stress in cyanobacterium Synechocystis sp. PCC 6803
Source: Biotechnol Biofuels. 2017 Mar 7;10:57. doi: 10.1186/s13068-017-0743-y (PMC5341163; doi:10.1186/s13068-017-0743-y)

Case1 :

sRNA located on positive strand

Case2 :

sRNA located on negative strand

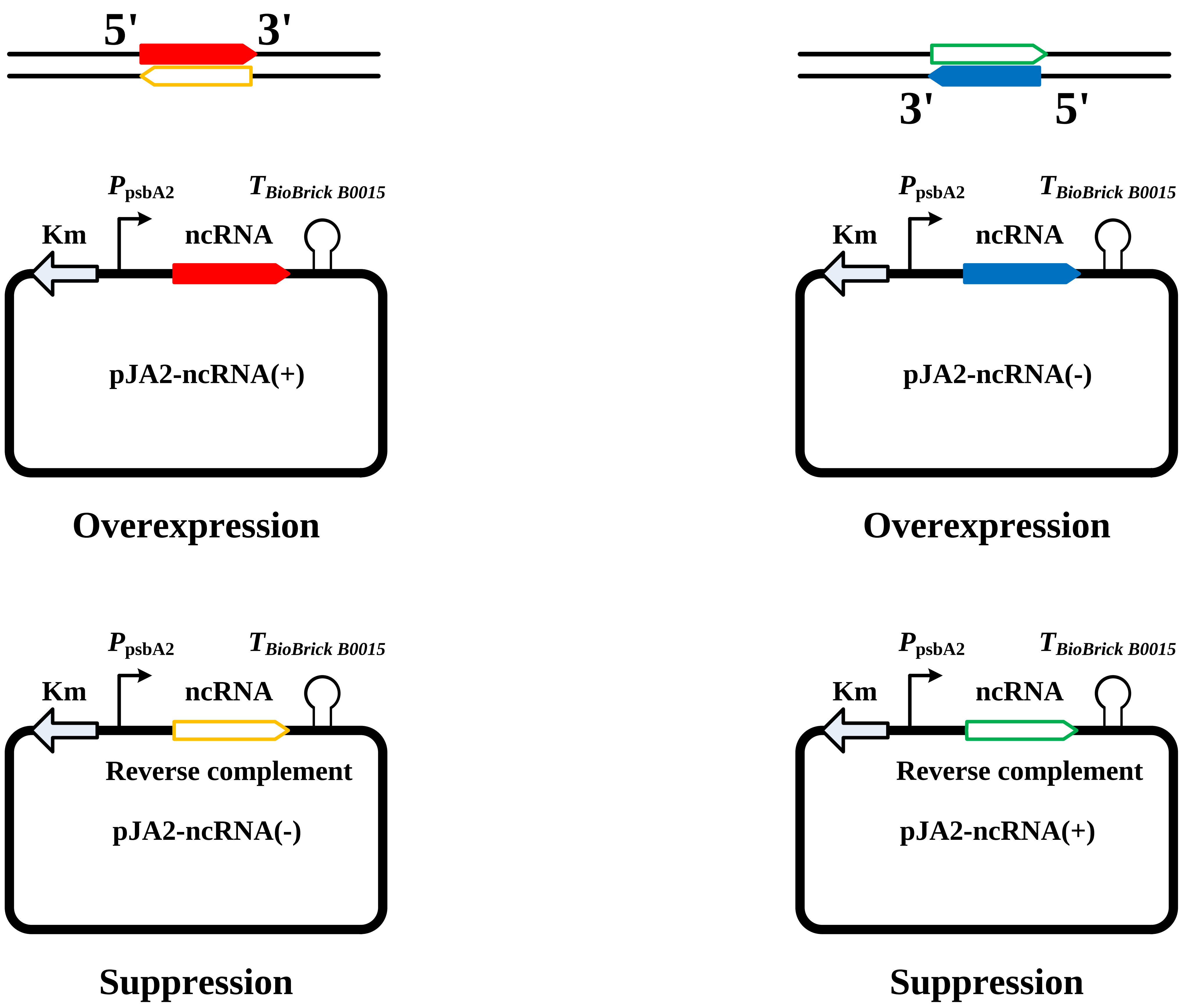

Supplement: Supplementary file 22 — Additional file 22: Figure S15. Schematic diagram for construction of sRNA overexpression and suppression strains. [file 13068_2017_743_MOESM22_ESM.pdf]
